# ABP-Finder: A Tool to Identify Antibacterial Peptides and the Gram-Staining Type of Targeted Bacteria

**DOI:** 10.3390/antibiotics11121708

**Published:** 2022-11-26

**Authors:** Yasser B. Ruiz-Blanco, Guillermin Agüero-Chapin, Sandra Romero-Molina, Agostinho Antunes, Lia-Raluca Olari, Barbara Spellerberg, Jan Münch, Elsa Sanchez-Garcia

**Affiliations:** 1 Computational Biochemistry, Center of Medical Biotechnology, University of Duisburg-Essen, 45141 Essen, Germany; 2 CIIMAR—Centro Interdisciplinar de Investigação Marinha e Ambiental, Universidade do Porto, Terminal de Cruzeiros do Porto de Leixões, Av. General Norton de Matos, s/n, 4450-208 Porto, Portugal; 3 Departamento de Biologia, Faculdade de Ciências, Universidade do Porto, Rua do Campo Alegre, 4169-007 Porto, Portugal; 4 Institute of Molecular Virology, University Hospital Ulm, 89081 Ulm, Germany; 5 Institute of Medical Microbiology and Hygiene, University Hospital Ulm, 89081 Ulm, Germany

**Keywords:** antibacterial peptide, machine learning, AMPs database, StarPep, Gram staining-based target, peptide library screening, human peptidome

## Abstract

Multi-drug resistance in bacteria is a major health problem worldwide. To overcome this issue, new approaches allowing for the identification and development of antibacterial agents are urgently needed. Peptides, due to their binding specificity and low expected side effects, are promising candidates for a new generation of antibiotics. For over two decades, a large diversity of antimicrobial peptides (AMPs) has been discovered and annotated in public databases. The AMP family encompasses nearly 20 biological functions, thus representing a potentially valuable resource for data mining analyses. Nonetheless, despite the availability of machine learning-based approaches focused on AMPs, these tools lack evidence of successful application for AMPs’ discovery, and many are not designed to predict a specific function for putative AMPs, such as antibacterial activity. Consequently, among the apparent variety of data mining methods to screen peptide sequences for antibacterial activity, only few tools can deal with such task consistently, although with limited precision and generally no information about the possible targets. Here, we addressed this gap by introducing a tool specifically designed to identify antibacterial peptides (ABPs) with an estimation of which type of bacteria is susceptible to the action of these peptides, according to their response to the Gram-staining assay. Our tool is freely available via a web server named ABP-Finder. This new method ranks within the top state-of-the-art ABP predictors, particularly in terms of precision. Importantly, we showed the successful application of ABP-Finder for the screening of a large peptide library from the human urine peptidome and the identification of an antibacterial peptide.

## 1. Introduction

Antibiotic resistance is a life-threatening health problem worldwide, and one of the main causes of death in developing countries [1,2]. The potential capability of peptides to overcome resistance [3] has motivated the development of new antibiotics from antimicrobial peptides (AMPs) to combat multi-drug resistant pathogens and the threats of Gram-negative infections [4,5].

AMPs are oligopeptides produced by a great variety of organisms, from prokaryotes to eukaryotes, including humans. Due to their various functions, AMPs are considered a part of the innate immune system of higher eukaryotes. The structural diversity of AMPs allows them to display a broad range of antimicrobial activity against pathogenic agents, including viruses, Gram-positive and Gram-negative bacteria, as well as fungi. Besides, the bacterial selectivity of AMPs over eukaryotic cells and their different action modes make peptides excellent antibiotic candidates [3,4,6]. A widespread mechanism of antibacterial peptides (ABPs) is the destabilization and destruction of bacterial membranes. However, these peptides can also interfere with intracellular processes such as nucleic acid and protein synthesis, enzymatic modulation, and protein degradation [7,8,9], which is an advantage over traditional antibiotics [3,10].

Most AMPs are naturally occurring peptides that represent promising candidates for optimization in advanced steps of the drug design process [11]. AMP-based drugs have been clinically approved to treat both topical and systemic infections. For instance, polymyxins and gramicidin S were formulated for the prevention of topical infections caused by *Pseudomonas aeruginosa* and *Acinetobacter baumannii*. Colistin, a polymyxin derivative, is currently used for the systemic treatment of lung infections, especially those caused by *Pseudomonas aeruginosa* [12]. Due to its problematic resistance profile, *Pseudomonas aeruginosa* is often difficult to treat by antibiotics [13]. However, it can be targeted by a variety of different AMPs [13,14,15] that may be further developed into innovative therapeutics.

The specificity of peptides toward certain targets is usually highlighted as an important benefit for therapeutic intervention. Nonetheless, a downside of this feature is the associated challenge for the drug design process, given that small structural modifications can significantly influence both the activity and pharmacokinetic properties of the peptides. Consequently, optimizing the precision of tools for the screening of large datasets of peptides is of utmost relevance to improve efficiency at the early steps of drug design processes.

For over a decade, growth in the publicly available data of AMPs has been witnessed, with the subsequent development of several machine learning (ML)-based predictors integrated with AMP databases such as DAMP [16], APD3 [17], CAMP [18], CAMP_R3_ [19], LAMP [20], DRAMP [21], ADAM [22], and DBAASP [23]. However, most of these prediction tools only discriminate between AMPs and non-AMPs. This is a highly ambiguous outcome given the broad scope of antimicrobial activity, which typically refers to more than 20 biological functions, such as the annotations in APD3 [17].

A group of predictors addressed this issue by applying a hierarchical classification scheme where first the peptides are classified as AMPs or not, and the positive cases are then sub-divided into a couple of classes based on selected AMP functions (e.g., antibacterial, antiviral, and antifungal peptides). Examples of such predictors, which include the antibacterial function are AntiBP2 [24], ClassAMP [25], MLAMP [26], *i*AMPpred [27], AMAP [28], AMP Scanner [29,30], and AMPDiscover [31]. However, of them, only AMP Scanner vr.1 predicts a type of bacterial target (*E. coli* or *S. aureus*) for the identified ABP [29].

In this context, we implemented a two-level predictor focused on antibacterial peptides (ABPs), named ABP-Finder, whose inner classifier estimates the Gram staining type of the putative targets. This tool leverages random forest (RF) classifiers trained with peptide data extracted from StarPep, the largest up to date public database of AMPs [32]. ABP-Finder categorizes ABPs and non-ABPs in the first classification level. Subsequently, the peptides identified as ABPs are sub-classified according to the Gram staining type of the potential targets i.e., exclusively Gram-positive, exclusively Gram-negative bacteria, or broad-spectrum peptides with expected activity against both types of bacteria. The ABPs used to develop this predictor show activity against at least one of nine representative bacterial targets (see Dataset section), among which are species with known multi-drug resistance such as *Acinetobacter baumannii*, *Enterococcus faecium*, *Klebsiella pneumonia*, *Pseudomonas aeruginosa*, and *Staphylococcus aureus.* With ABP-Finder, we weigh precision as the main performance feature of the prediction. In this way, we boost the efficiency of the screening step at the early stages of the drug design process aiming at the development of peptide-based antibiotics. Remarkably, we prove the efficacy of ABP-Finder for such screenings with the identification of a peptide from the human urine peptidome, displaying antimicrobial activity against *Pseudomonas aeruginosa*.

## 2. Materials and Methods

### 2.1. Data Collection and Pre-Processing

The models developed in this study were derived from the StarPep database [32,33]. This resource, as described by the authors, is a non-redundant compendium from 40 publicly available data sources, which encompasses annotations of more than 20 functions in approximately 45,000 AMPs, with nearly 8000 entries labelled as antibacterial peptides.

Before describing the construction of our training and test sets, we point out a shortcoming of several AMP-based predictors found in the literature [16,17,18,19,20,21,22], whose models do not obey the first principle dictated by the Organisation for Economic Co-operation and Development (OECD) to build reliable Quantitative Structure–Activity Relationship (QSAR)/ML-based models [34] (https://doi.org/10.1787/9789264085442-en (accessed on 16 November 2022)). This principle is stated as “a defined endpoint”. Commonly, AMPs are annotated as such regardless of the target, mechanism, source, the method used to study the activity, to name some characteristics. The lack of such detailed information makes the discrimination between AMPs and non-AMPs a largely ambiguous endpoint for data analysis. In consequence, several criteria must be introduced to better define the modelled data and thus bring reliability to the predicted outcome. Notably, the most recent AMP predictors [24,25,26,27,28,29,31] have designed their modeling approaches to break down the AMP annotation into three classes (typically antibacterial, antifungal, and antiviral peptides). This strategy is a suitable approach to fulfil the need for a defined endpoint.

Our work focused on the identification of ABPs. To this end, we extracted peptides from the StarPep database ranging between 5 and 50 residues, and whose composition contains only the 20 standard amino acids. To further refine the selection of ABPs, we only extracted those peptides annotated as active against at least one of the following targets: *Acinetobacter baumannii*, *Bacillus subtilis*, *Enterococcus faecium*, *Escherichia coli*, *Klebsiella pneumonia*, *Listeria monocytogenes*, *Pseudomonas aeruginosa*, *Streptococcus agalactiae*, and *Staphylococcus aureus*. In this way, we discarded entries that are annotated as ABPs without information of their targets, and those exclusively reported with activity against underrepresented targets in the entire database. The selected species cover a set of both Gram-positive and Gram-negative bacteria and are examples of relevant targets for therapeutic applications. The peptides labeled as non-ABP for our learning process are not annotated as antibacterial, against any target, in StarPep, but with a different function such as antifungal or anticancer, among others. This approach clearly carries the risk of mislabeling non-ABP in our dataset, due to insufficient annotation of the peptide in the original source. The pseudo-negative cases in the training data lead to a more stringent prediction of positive cases, and consequently lower false-positive rate and higher precision. The downside is the expected lower recall as the true positives can be also diminished. Nonetheless, the favourable precision is aligned with our stated goal of boosting the precision of the classifier instead of its recall or a combined metric such as accuracy or AUC.

Hence, we extracted a total of 22,707 peptides to design our training and testing schemes. This collection was partitioned into four datasets: training, development, validation, and test sets. The two first are intended for the learning process, while the others are meant for testing the models with hold-out data. The development (Dev) set was used to monitor the generalization of the models built during the optimization of the hyper-parameters in the learning algorithm. Usually, the terms development and validation set are applied indistinctively to a dataset used for the above-mentioned purpose. In this work, we made a distinction between these nomenclatures and reserved the term validation for a hold-out set, i.e., peptides that are not used in any step of the learning process. The difference between the validation and the strict test set is that we built the validation set in a way that its peptides share high similarity (≥90% identity) with at least one peptide in the training set (excluding identical matches). In turn, the test set was built in a way that its peptides share less than 90% identity among them, and with any peptide in the training data. Consequently, the test set comprises non-redundant peptides that are also not closely represented in our training. Challenging a peptide predictor in both scenarios, one that closely resembles the training conditions (without strict superposition), and another more distant setup, is important to assess the biasing effect on the generalization of the model due to the characteristics of the training data.

**Figure 1:**
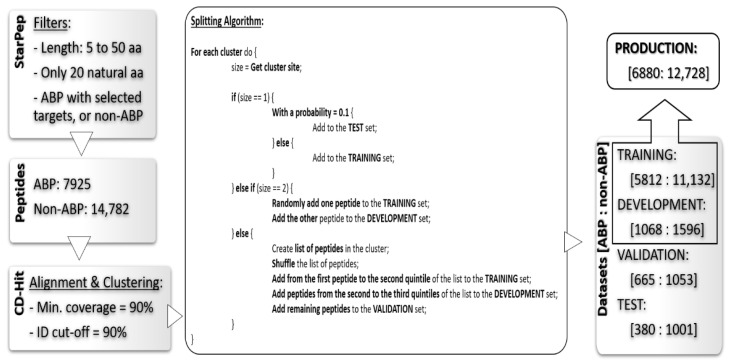
Workflow for the preparation of the datasets. The peptides extracted from StarPep were clustered with CD-Hit and subsequently distributed among the four sets used for training and testing the predictor. The final panel of the pipeline contains information about the number of peptides in every subset as well as their classification according to StarPep.

Finally, a production dataset was generated by combining the training and the development sets. The purpose of this set is to perform a final re-training of the model with an augmented dataset, while keeping the selection of descriptors and configuration of hyper-parameters as optimized with the training and development sets. Figure 1 depicts the workflow followed to obtain the four datasets.

Together with the peptide sequences and their classification as ABP or non-ABP, we also extracted, from StarPep, the information about the Gram staining type of their known targets. Accordingly, we further categorized the ABPs into three activity classes: exclusively against Gram-positive targets (Gram+), exclusively against Gram-negative targets (Gram-), and broad-spectrum peptides. The four datasets resulting from the previous splitting were also used to train and assess the secondary classifier based on the Gram staining type of the targets. For this purpose, the non-ABP peptides were removed from such datasets. Table 1 summarizes the number of peptides per type of Gram staining class in the four datasets.

### 2.2. Performance Measures

In this section, we summarize the formulations of the performance measures used to assess the different models described here. The measures are sensitivity (Sn), precision (Pr), accuracy (Acc), F1 score, and the Mathew Correlation Coefficient (MCC) [35]. All of them are formulated in terms of the elements of a binary confusion matrix: true positives (TP), true negatives (TN), false positives (FP), and false negatives (FN).

$$\mathrm{Sn}=\frac{\mathrm{TP}}{\mathrm{TP}+\mathrm{FN}}$$

$$\mathrm{Pr}=\frac{\mathrm{TP}}{\mathrm{TP}+\mathrm{FP}}$$

$$\mathrm{Acc}=\frac{\mathrm{TP}+\mathrm{TN}}{\mathrm{TP}+\mathrm{TN}+\mathrm{FP}+\mathrm{FN}}$$

F1=2Sn∗PrSn+Pr=TPTP+12FP+FN

$$\mathrm{MCC}=\frac{\mathrm{TP}*\mathrm{TN}-\mathrm{FP}*\mathrm{FN}}{\sqrt{\left(\mathrm{TP}+\mathrm{FP}\right)\left(\mathrm{TP}+\mathrm{FN}\right)\left(\mathrm{TN}+\mathrm{FP}\right)\left(\mathrm{TN}+\mathrm{FN}\right)}}$$

Besides, we define an ad-hoc measure named Fitness–Robustness Score (FRS) that is specifically used as a scoring function to tune the values of the hyper-parameters of the learning technique.

$$\mathrm{FRS}={\left(\frac{R_{T}+R_{\mathrm{CV}}+R_{D}}{3}\right)}^{2}-{\left(R_{T}-R_{\mathrm{CV}}\right)}^{2}-{\left(R_{T}-R_{D}\right)}^{2}$$

The FRS is a quality measure that provides a consolidated value for the performance of a particular model considering its goodness-of-fit, generalization, and robustness. The first term corresponds to the average performance in the following assessments: re-substitution (RT, fitting the training data), 10-fold cross-validation (RCV, within the training data), and generalization (RD, using the development set). The other two terms weigh the robustness of the model by measuring the deviations from the performance in training samples when the model is evaluated in hold-out data (cross-validation and development set). We formulated this ad-hoc measure as a function of another base quality measure, labelled as R, which should be evaluated in the different assessment schemes. For this study, we selected the MCC as the base measure to evaluate our fitness-robustness score. In the case of the multi-classifier trained to distinguish between the Gram staining classes, the average MCC value among the three classes was used as the base measure. The average was weighted according to the number of peptides in each class.

The FRS, when computed as a function of the MCC, has an optimum maximum value of one. We leveraged this score to identify optimum values for the hyper-parameters of the random forest [36] algorithm used to develop our models.

### 2.3. Machine Learning Approach and Software

The classifiers developed in this work were random forest (RF) [36] predictors, based on the implementation of this technique in the WEKA environment [37]. RF belongs to the family of ensemble methods [38] with base classifiers formed by decision trees. Recently, RF has been compared with deep learning approaches showing comparable performance for modeling AMP datasets [39]. There, the authors conclude that no definitive evidence was found to support using deep-learning approaches for this problem, knowing the increased algorithmic complexity and computational cost of these methods.

Within RF, all the trees provide a prediction for every instance entering the forest, and the unified outcome is obtained as the majority vote among all the predictions. The hyper-parameters optimized during the learning process were the number of trees, the maximum number of descriptors used to build a tree (these descriptors are taken at the beginning of the training process from the global pool of attributes), and the maximum depth of the trees. In addition, the minimum number of instances in the final leaves of the trees was fixed to 10 in the case of the main classifier (ABPnon-ABP), and to five for the multi-classifier (Gram+/Gram−/broad spectrum).

The peptide descriptors fed to the learning algorithm were computed with the ProtDCal-Suite [40] using the configuration files enclosed in the Supplementary Material. The ProtDCal module [41] is intended for the calculation of general-purpose and alignment-free descriptors of amino acid sequences and protein structures. These features are descriptive statistics (such as the variance, average, maximum, minimum, percentiles, etc.) of the distribution of amino acid properties (such as hydrophobicity, isoelectric point, molar weight, among others), in multiple groups of residues extracted from a given protein or peptide. The program possesses additional procedures that modify the intrinsic properties of a residue according to its vicinity in the sequence, thus adding connectivity information in the descriptors. The features derived from ProtDCal have been used by us and other authors to develop machine-learning-based predictors of posttranslational modifications [42,43], protein–protein interaction [44], enzyme-like amino acid sequences [45], residues critical for protein functions [46], and antibacterial peptides [47,48], although with smaller databases. The project files enclosed in the Supplementary Material contain the setup used to compute all the descriptors employed in this work.

### 2.4. Web Servers Available for ABPs Predictions

In this section, we briefly describe the most relevant state-of-the-art ABP predictors that are available via web server tools. ClassAMP was among the first methods that broke down the AMP family thus allowing the prediction of ABPs specifically [25]. This tool was trained with peptides from the CAMP database [18] and used RF and support vector machine (SVM) [49] algorithms to identify antibacterial, antifungal, and antiviral peptides.

MLAMP, a multi-label classifier of AMPs was developed using a variant of Chou’s pseudo amino acid composition (PseACC) features [50] to build an RF-based classifier that firstly distinguishes AMP from non-AMPs, and then subdivides the biological activity into antibacterial, anticancer, antifungal, antiviral, and anti-HIV [26].

Similarly, the *i*AMPpred predictor combines compositional, physicochemical, and structural features into Chou’s general PseACC as input variables for an SVM multi-classifier [27]. This work reunited peptides from the databases CAMPR3 [19], APD3 [17], and AntiBP2 [24]. The multi-classifier uses three categories in the outcome variable: antibacterial, antifungal, and antiviral peptides [27].

The Antimicrobial Activity Predictor (AMAP) [28], with a hierarchical multi-label classification scheme, was trained with AMPs annotated with 14 biological activities in the APD3 database and a designed subset of non-AMP. The models used amino acid composition features to feed SVM and XGboost tree [51] algorithms.

The introduction of the AMP-Scanner webserver represented a significant improvement with respect to other predictors. AMP-Scanner vr.1 consists of two RF classifiers, trained with peptides selected from multiple sources [18,52,53]. The first output of the classifier is the identification of ABPs. The second is a classifier trained to distinguish between peptides with Gram-positive or Gram-negative targets, using data of *S. aureus* and *E. coli* as reference targets. The authors refer that peptides predicted with scores within the range [0.4–0.6] for both classes should be considered as active against both types of targets (broad-spectrum peptides) [29]. On the other hand, AMP-Scanner vr.2 is based on a Deep Neural Networks (DNN) classifier fed with ABP data only, obtained from the updated version of the ADP3 database [19,30].

Very recently, AMPDiscover [31] was developed by mining AMP data from StarPep [33]. AMPDiscover encompasses several binary (active/non-active) predictors of functions such as antibacterial, antiviral, antifungal, and antiparasitic peptides. The authors analyzed the performance of RF to model the antibacterial peptides data, which agrees with our choice of this learning scheme for our models.

### 2.5. Experimental Determination of Antibacterial Activity

Two batches of chemically synthetized peptides from different providers (KE Biochem and the U-PEP facility at Ulm University) were used to assess antimicrobial effects. Antibacterial activity was evaluated by agar diffusion as previously described [54]. Bacteria were cultured in liquid broth at 37 °C overnight, pelleted by centrifugation, and washed in 10 mM sodium phosphate buffer. Following resuspension, optical density was determined at 600 nm and 2 × 10^7^ bacteria were seeded into a Petri dish in 1% agarose. After cooling at 4 °C for 30 min, 3–5 mm holes were placed into the 1% agarose. Peptides adjusted to the desired concentration in 10 µL of buffer were filled into the agar-holes. Following incubation at 37 °C in ambient air for 3 h, plates were overlaid with 1% agarose, tryptic soy solved in 10 mM phosphate buffer. Inhibition zones in cm were determined after 16-18 h incubation time at 37 °C in 5% CO_2_. LL37 at a concentration of 100 µg/mL served as positive control. Antimicrobial activity was tested on the following bacterial strains: *Bacillus subtilis*, *Streptococcus agalactiae* ATCC 12403, *Staphylococcus aureus* MRSA ATCC 43300, *Klebsiella pneumoniae* Extended Spectrum β-Lactamase (ESBL) ATCC 700603, *Pseudomonas aeruginosa* (ATCC 27853) and *Listeria monocytogenes* (ATCC BAA-679/EGD-e).

## 3. Results and Discussion

Below, we summarize the characteristics of the ML-based models developed in this work, as well as their performance relative to the available state-of-the-art ABP predictors. We also introduce a web server, ABP-Finder, which permits the free and user-friendly screening of large peptide libraries. Finally, we present the application of ABP-Finder for the screening of peptides obtained from the human urine peptide. Notably, ABP-Finder permitted to screen and propose a reduced set of eight ABP candidates out of an initial pool of 4696 peptides. From them, one active hit was experimentally validated with activity against *Pseudomonas aeruginosa*.

### 3.1. Modeling Antibacterial Peptide Data

*Feature selection:* The feature selection process comprises three steps. (*i*) First, the Information Gain (IG) [55,56] of all the descriptors was calculated with WEKA, retaining only those descriptors whose IG is >5% of the information content of the class variable. This procedure reduced an initial set of 11,298 descriptors to 2746, whose information contents are the most closely related to our end point variable. (*ii*) Secondly, the redundancy in this subset of features was removed, by clustering the descriptors using a quality-threshold-based [57] clustering algorithm, which employs the Spearman correlation coefficient [58] as the similarity measure to group the descriptors. A correlation cut-off of 0.9 was used to form the clusters. The outcome of these steps is thus a non-redundant and smaller dataset that contains only the central attributes of the formed clusters. This step rendered 1242 attributes. (*iii*) Given the still large set of features, a last selection step was used by employing the Wrapper Evaluator and the Classifier Subset Evaluators of WEKA coupled with a genetic search algorithm [59]. The Wrapper Evaluator used five-fold cross-validation on the training data to assess the models obtained from diverse subsets of descriptors. Such models were built with an RF whose number of trees was limited to 15. Next, the Classifier Subset Evaluator used the performance with the development set to identify the most suitable pool of descriptors to train the RF. For both evaluators, the F1 measure was used to score all the assessed subsets of attributes. The genetic search employed to explore the space of all possible combinations of attributes was configured with 20 chromosomes (subsets of attributes) per population, 500 generations, and probabilities of cross-over and mutation of 0.6 and 0.1 respectively. The optimal subset resulting from these selection steps comprised 281 descriptors. A project file type IDL (Individual Descriptor Labels) is enclosed in the Supplementary Material; this project file can be uploaded directly to ProtDCal-Suite to compute the selected 281 descriptors in new peptide datasets.

**Figure 2:**
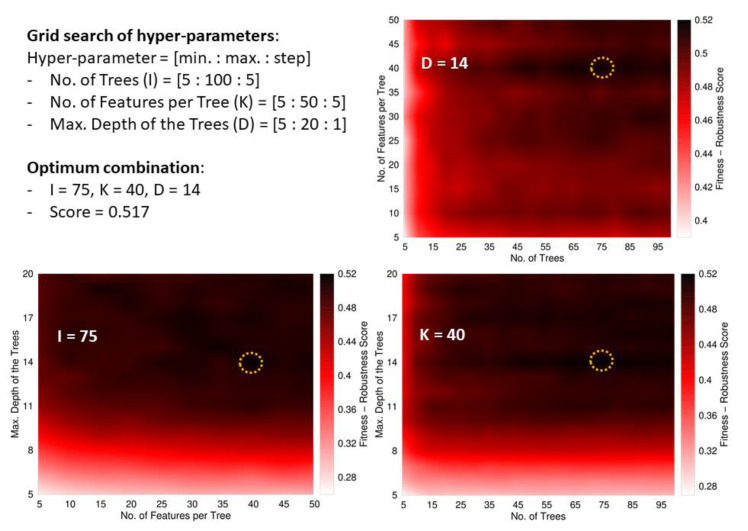
Tuning scheme of the RF’s hyperparameters. The top-left panel summarizes the boundaries and binning of the grid search with the three hyper-parameters. This panel also shows the optimum value found for the FRS function and the values of the hyper-parameters in the corresponding solution. The remaining panels show surfaces plotted as heat maps keeping one of the hyper-parameters fixed at its optimum value. The dark regions indicate the best solutions. The optimum regions are highlighted with a dashed circle. The plots highlight that the most critical parameter is the depth of the trees, while high-scored models can be obtained with almost any value of the other hyper-parameters; solutions with a depth below 10 are poorly scored.

*Tuning hyperparameters*: The hyperparameters of the RF were explored using a grid search according to ranges and binning schemes summarized in the top-left panel of Figure 2. The ad hoc FRS function was used to determine the optimum combination of hyperparameters’ values, which was obtained with 75 trees each one built from a pool of 40 descriptors and a maximum depth of 14 splits. Such combinations of values rendered the maximum FRS at 0.517.

### 3.2. Modeling Data of Gram-Staining Types

This model was trained with the same set of 281 descriptors obtained from the feature selection procedure to discriminate between ABPs and non-ABPs. The training, development, validation, and test sets used for this model were obtained from the splitting described in the Methods section, by removing the non-ABP present in these datasets. The ABPs were then subdivided according to the Gram-staining type of their known targets.

**Figure 3:**
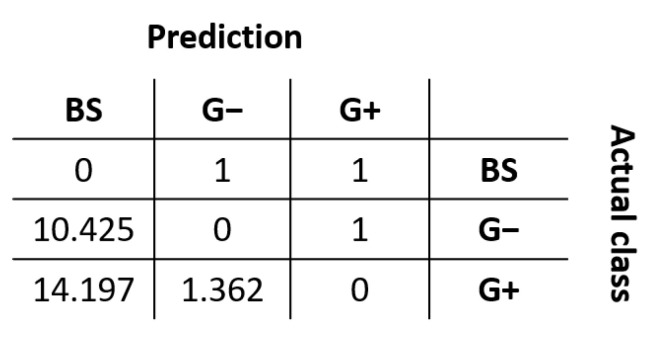
Cost matrix applied during the training process of the multi-classifier based on the Gram-staining types of the targets.

Due to the imbalance in the number of instances from each class, the cost-sensitive RF multi-classifier was trained by applying a cost matrix in the training process with distinct weights for the different types of misclassified cases. The cost matrix takes the form shown in Figure 3.

The multi-class classifier was built with a cost-sensitive learning scheme, which aims to balance the effective error between pairs of classes considering their different prevalence in the training data. The costs were defined as the inverse ratio of the imbalance between the two classes involved in the matrix element, i.e., given the imbalance between Gram+ and broad-spectrum (BS) peptides in the training data is [1: 14.197], then the cost of a Gram+ peptide classified as BS was fixed at 14.197 and the cost of a BS peptide classified as Gram+ remained at 1. This approach diminishes the trend towards BS predictions that originates due to the highest representation of this class in the training data.

The costs affect the training process by re-weighting the training samples in the calculation of the different misclassification errors during the training. No re-weighting is applied to the instances in the test datasets.

**Figure 4:**
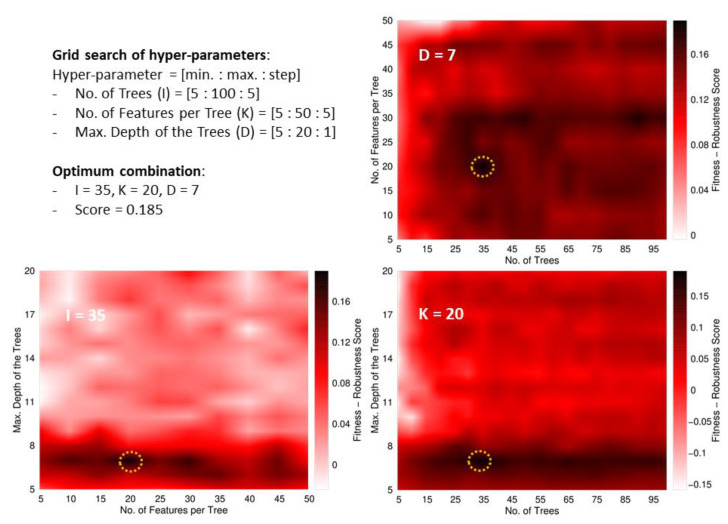
Tuning scheme of the RF’s hyper-parameters. The top-left panel summarizes the boundaries and binning of the grid search with the three hyper-parameters. This panel also shows the optimum value found for the FRS function and the hyper-parameters’ values of the corresponding solution. The remaining panels show surfaces plotted as heat maps keeping one of the hyper-parameters fixed at its optimum value. The dark regions indicate the best solutions. The optimum regions are highlighted with a dashed circle. As in the exploration for the model ABPs /non-ABPs, the plots show that the most critical parameter is the depth of the trees. Nonetheless, the opposite trend is observed because high-scored models are only obtained with low (< 8) depth values. The smaller size of the dataset for this model, as compared with the previous one, leads to the occurrence of overfitting when deep trees are trained.

*Tuning hyperparameters*: Analogous to the previous model, the hyper-parameters of the RF were explored using a grid search with the ranges and binning schemes summarized in the top-left panel of Figure 4. The FRS function rendered a maximum value for a solution with 35 trees, 20 descriptors per tree, and a maximum depth of 7 splits. Such combinations of values rendered the maximum FRS at 0.185. The lower value of the optimum FRS value, compared with the ABP/non-ABP model, indicates the larger difficulty of discriminating between the three classes of Gram-staining types. Such difficulty is a natural consequence of the overlap between the classes, given that the peptides in the broad-spectrum category should gather intrinsic features of the other two classes.

### 3.3. Applicability Domain

Following the regulatory principles for QSAR models established by the OECD, we discuss the applicability domain (AD) of our models. Both of our models were built using peptides with lengths between 5 and 50 residues and containing exclusively the 20 standard amino acids. Thus, these length and composition boundaries constitute soft limits of our applicability domain. A quantitative approach for the AD is provided via the range of the descriptors’ values in the training or production dataset. In the Supplementary Material, we provide the minimum and maximum values of the descriptors in these datasets. As part of the implementation of these predictors, we automatically evaluate whether any new peptide is found within these ranges or not. If any of the descriptor values of a new peptide falls outside the training ranges, this peptide is labelled as an outlier and the corresponding information is given in the outcome of the program.

### 3.4. Performance of ABP-Finder in the Context of the State-of-the-Art

*Predictors of antibacterial peptides*: We compare the performance of our models to five ML-based ABP predictors by employing the hold-out validation and test sets, respectively (Table 2 and Table 3). In addition, we employ an external test set originally used by Veltri et al. [30] to assess the performance of AMP-Scanner vr2 (Table 4). We present the performance of our models obtained with the training data only, and with the production dataset. Additionally, we show the performance of our tool considering only those instances that are within the AD of our models.

Table 2 and Table 3 show that our models achieved the best precision and global accuracy in the test and validation sets. Particularly, the precision was significantly higher with ABP-Finder with respect to the other methods. This is a key feature to be leveraged when filtering large peptide libraries because the main aim during the screenings for new hits is to avoid false-positive predictions.

We also challenged our models with an external test set designed by Veltri et al. [30] (Table 4) to further assess the robustness of our predictions. This dataset is qualitatively different from our test set since it is not derived from the StarPep database as all our data, and therefore it was not subjected to any of the curation procedures carried out by the StarPep’s developers.

These comparisons confirm that our RF-based models render the most precise predictions, although the sensitivity (and consequently the global accuracy) decays in this case compared with other ABP predictors. Nevertheless, we note the importance of a low false-positive rate in virtual screening analyses, which highlights the higher practical value of our predictors.

*Predictors of Gram-staining types*: Our antibacterial predictor was designed to provide an estimation of against which type of bacteria are the peptides active. Therefore, we tested how our multi-classifier performs for the Gram+, Gram−, and Broad-Spectrum classes compared to AMP-Scanner vr.1. Table 5 and Table 6 summarize the comparison with respect to precision and sensitivity of our models and AMP-Scanner vr.1 on the validation and test sets, respectively. The performance measures were computed for the three classes (Gram+, Gram−, and Broad Spectrum).

Our models largely outperformed AMP-Scanner vr.1, particularly in terms of precision when detecting the specific types of Gram-staining types (Gram+ and Gram−). Regarding the prediction of broad-spectrum peptides, both methodologies delivered the same precision. However, in this case we greatly surpassed the sensitivity of AMP-Scanner vr.1, thus making more accurate predictions overall. Notably, our multi-classifier showed the best performance for the three classes of Gram-staining types, thus providing a valuable complement to the identification of antibacterial peptides.

The comparison with the state-of-the-art tools showed that, together with ABP-Finder, the top-ranked methods in our tests were *i*AMPred, AMP-Scanner vr2, and AMPDiscover. These approaches were thus confirmed as suitable tools for ABP identification. Nonetheless, ABP-Finder outperformed these predictors, particularly in terms of precision. Importantly, as a distinctive feature, we complement our outcome with an estimation of the Gram-staining type of the putative targets, which can be further pinned down to specific bacterial species by considering that our models were trained with data from nine representative targets (see Dataset section). Furthermore, unlike previously published tools [24,25,26,27,28,29,30], we provide an estimation of our applicability domain, which delivers reliability to the predicted outcome.

### 3.5. ABP-Finder Web Server

Our emphasis in the application of regulatory principles to the development of ML-based predictors relies on our commitment to offer a freely accessible and well-maintained tool to reliably screen peptide libraries. To this end, we implemented our models in a user-friendly web server named ABP-Finder (https://protdcal.zmb.uni-due.de/ABP-Finder/ (accessed on 16 November 2022)). This tool allows screening seamlessly thousands of peptides with a single submission job. The ABP-Finder server delivers for each entry a prediction of the antibacterial function, as well as whether each specific peptide is or not within the AD of our models. ABP predictions are also accompanied by a Gram-staining-based estimation of the putative targets of the antibacterial peptides. Furthermore, the web server offers the functionality of screening regions within a long amino acid sequence to identify promising antibacterial fragments. This application of ABP-Finder’s models was recently leveraged by us for the identification of antibacterial motifs within β2-microglobulin [60].

### 3.6. Virtual Screening of the Human Urine Peptidome

**Figure 5:**
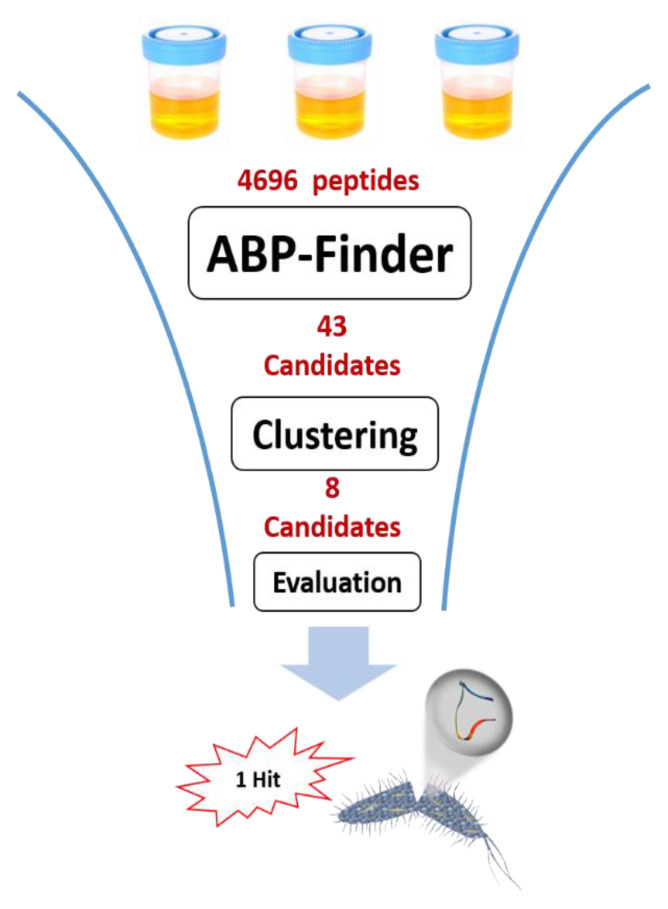
Schematic representation of the virtual screening process carried out on a library of peptides from the human urine peptidome.

In this section, we describe the successful application of ABP-Finder to screen a peptide library obtained from the human urine peptidome. The library contains 4696 endogenous peptide fragments, detected in the Core Facility Functional Peptidomics at the University Hospital in Ulm, Germany. The peptide library was screened for antibacterial activity following the workflow depicted in Figure 5.

ABP-Finder was used to score the original 4696 peptides of the library, obtaining 43 candidates with a probability score larger than 0.6, and within the applicability domain of the model. Subsequently, Blastp [61] was used to cross-align these peptides with known ABPs of our training samples. From there, we excluded two hits that showed 100% identity and coverage in the alignment with previously reported ABPs and therefore did not have value as newly identified peptides. Afterward, we clustered the peptide sequences using CD-Hit [62] with a cut-off of 90% of identity, and minimum coverage of the shortest sequence in the alignment of 90%. From this analysis, eleven clusters were obtained, from which we extracted the shortest sequence as representative of each cluster. Three polyproline peptides, containing none or only one residue other than proline were finally discarded because we considered them unsuitable as candidates for possible lead compounds due to synthetic unfeasibility and the highly homogenous character of their sequences. The final eight candidates (Table 7) were experimentally evaluated using an agar diffusion assay, leading to one active hit, Urine-3462, against *Pseudomonas aeruginosa.*

### 3.7. Experimental Evaluation of the Reduced Set of Peptides from the Human Urine Peptidome

**Figure 6:**
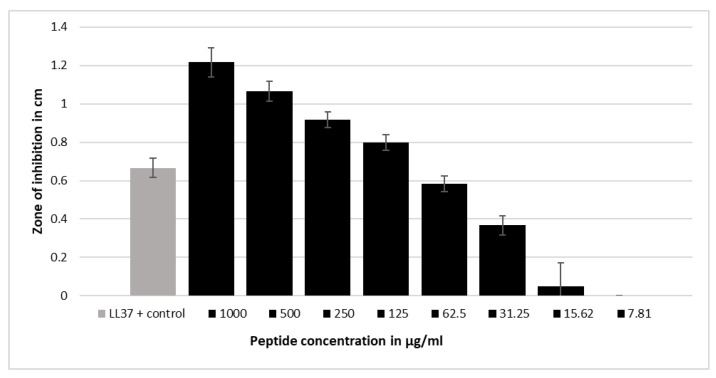
A radial diffusion assay indicated that the peptide Urine-3462 is active against the *Pseudomonas aeruginosa* strain ATCC 27853. Inhibition zones are quantified in cm. The mean values and standard deviations of six independent experiments are shown. LL37 at 100 µg/mL was used as positive control (see Table S3 for exact values).

To test the antimicrobial potential of the eight candidate peptides identified with ABP-Finder, a radial diffusion assay was carried out, allowing the sensitive detection of antibacterial activity. Activity was determined against various Gram-positive and Gram-negative bacteria species, including *Bacillus subtilis*, *Streptococcus agalactiae*, *Staphylococcus aureus* (MRSA), *Escherichia coli*, *Pseudomonas aeruginosa*, *Klebsiella pneumoniae* (ESBL). While the peptide Urine-3462 was active against *Pseudomonas aeruginosa*, no relevant antibacterial activity could be detected at concentrations of 100 µg/mL and 1 mg/mL of the other peptides. Urine-3462 exhibited a dose-dependent growth of inhibition of *Pseudomonas aeruginosa*, comparable to the inhibitory activity observed for the well described antimicrobial peptide LL37 [54,65], which served as a positive control (Figure 6).

## 4. Conclusions

Antibacterial peptides are promising candidates for a new generation of antibiotics designed to address the challenging problem of drug resistance in bacteria. With ABP-Finder we provide a tool that delivers top-ranked predictions as established by several comparisons with prominent examples of the state-of-the-art ABP predictors. Remarkably, ABP-Finder produces the most precise predictions in validation tests with known data. Furthermore, unlike other tools of the state-of-the-art that were used for comparison in this work, we present a successful application of the method in a real-life scenario dealing with the massive screening of unlabeled peptides from the human urine peptidome.

We implemented this RF-based predictor in the user-friendly and freely accessible web server ABP-Finder, which was also leveraged in the identification of the new ABP hit from a large library of peptides derived from the human peptidome.

In this way, the combination of in silico screening and experiments confirmed the applicability of ABP-Finder as a screening tool for the early steps of the design of peptide-based antibiotics. To the best of our knowledge, no other publicly available ABP predictor has delivered a similar study leading to the successful identification of an active hit from tens of thousands of unlabeled peptides. Further developments of our predictor will include its combination with target-specific models. This will allow improving the design of broad-spectrum candidates, as well as to orient the selection of targets in massive screenings of bioactive peptides.

## Figures and Tables

**Table 1:** Number of peptides per type of Gram staining class in the training, development, validation, and test datasets.

	Gram+	Gram−	Broad Spectrum
Training	351	478	4983
Development	52	105	911
Validation	37	82	546
Test	27	38	315

**Table 2:** Comparison with external predictors in the validation set. The values in bold denote the best performance for a given measure.

Webserver	Algorithm	Pr.	Sn.	Acc.
ClassAMP	SVM	0.46	0.33	0.59
MLAMP	RF	0.48	0.82	0.59
iAMPred	SVM	0.48	0.90	0.58
AMPScanner_v1 ^#^	RF	0.50	0.98	0.61
AMPScanner_v2 *	DNN	0.48	0.97	0.58
AMPDiscover	RF	0.50	**0.99**	0.61
				
ABP-Finder (Training)	RF	0.72	0.95	0.84
ABP-Finder (Training, AD)	RF	0.70	0.95	0.83
ABP-Finder (Production)	RF	**0.75**	0.95	**0.85**
ABP-Finder (Production, AD)	RF	**0.75**	0.95	**0.85**

AD: only instances within our applicability domain are considered as valid predictions. # AMPScanner_v1 only considers peptides ≥ 10 AA for the predictions. * The method was updated on 20.02.2020.

**Table 3:** Comparison with external predictors in the test set. The values in bold denote the best performance for a given measure.

Webserver	Algorithm	Pr.	Sn.	Acc.
ClassAMP	SVM	0.34	0.41	0.61
MLAMP	RF	0.38	0.77	0.59
*i*AMPred	SVM	0.36	0.81	0.56
AMPScanner vr.1 ^#^	RF	0.50	0.80	0.68
AMPScanner vr.2 *	DNN	0.37	0.84	0.57
AMPDiscover	RF	0.42	**0.94**	0.62
				
ABP-Finder (Training)	RF	0.77	0.68	0.86
ABP-Finder (Training, AD)	RF	0.78	0.67	0.86
ABP-Finder (Production)	RF	**0.80**	0.71	**0.87**
ABP-Finder (Production, AD)	RF	**0.80**	0.70	**0.87**

AD: only instances within our applicability domain are considered valid predictions. # AMPScanner_v1 only considers peptides ≥ 10 AA for the predictions. * The method was updated on 20.02.2020.

**Table 4:** Comparison with external predictors in the test set built by Veltri et al. [30]. Redundant instances with our training set were removed. The values in bold denote the best performance for a given measure.

Webserver	Algorithm	Pr.	Sn.	Acc.
ClassAMP	SVM	0.36	0.27	0.66
MLAMP	RF	0.51	0.65	0.72
*i*AMPred	SVM	0.74	**0.90**	0.88
AMPScanner vr.1 ^#^	RF	0.64	0.77	0.81
AMPScanner vr.2 *	DNN	0.82	0.89	**0.91**
AMPDiscover	RF	0.83	0.84	**0.91**
				
ABP-Finder (Training)	RF	0.83	0.43	0.81
ABP-Finder (Training, AD)	RF	0.83	0.51	0.86
ABP-Finder (Production)	RF	**0.84**	0.48	0.83
ABP-Finder (Production, AD)	RF	**0.84**	0.57	0.86

AD: only instances within our applicability domain are considered valid predictions. # AMPScanner_v1 only considers peptides ≥ 10 AA for the prediction. * Performance based on the model from the original training in Veltri et al. [30], where the cases in this test set are held out of the training process.

**Table 5:** Comparison of ABP-Finder with AMP-Scanner_v1 in the discrimination between Gram-staining classes within the validation set. The values in bold denote the best performance for a given measure.

Method	Gram+		Gram−		Broad Spectrum	
	Pr	Sn	Pr	Sn	Pr	Sn
AMPScanner vr.1 ^#^	0.04	0.19	0.16	0.42	0.81	0.27
						
ABP-Finder (Training *)	**0.63**	**0.73**	**0.91**	0.38	0.90	**0.97**
ABP-Finder (Production *)	0.62	0.70	0.85	**0.48**	**0.91**	0.96

AD: only instances within our applicability domain are considered valid predictions. # AMPScanner_v1 only considers peptides ≥ 10 AA for the predictions. * There are no instances outside the AD of the model.

**Table 6:** Comparison of ABP-Finder with AMP-Scanner_v1 in the discrimination between Gram-staining classes within the test set. The values in bold denote the best performance for a given measure.

Method	Gram+		Gram−		Broad Spectrum	
	Pr	Sn	Pr	Sn	Pr	Sn
AMPScanner vr.1 ^#^	0.08	**0.42**	0.13	**0.33**	**0.88**	0.23
						
ABP-Finder (Training)	**0.44**	0.41	**0.90**	0.24	0.87	**0.96**
ABP-Finder (Training, AD)	**0.44**	0.39	**0.90**	0.24	0.87	**0.96**
ABP-Finder (Production)	**0.44**	0.41	0.82	0.24	**0.88**	**0.96**
ABP-Finder (Production, AD)	**0.44**	0.39	0.82	0.24	**0.88**	**0.96**

# AMPScanner_v1 only considers peptides ≥ 10 AA for the predictions.

**Table 7:** The resulting eight ABP candidates from the virtual human urine peptidome screening and some of its global sequence descriptors. Global peptide descriptors were calculated using the Peptide Design and Analysis Under Galaxy (PDAUG) package [63].

Peptide	Sequence	Length	pI	Total Charge *^#^*	Global Hydrophobicity *	GRAVY Index ^&^
U2162	KKVLGAFSDGLAHLDNLKGT	20	10.42	1.09	0.08	-0.12
U687	DKTNVKAAWGKVGAHAGEYGAE	22	9.53	0.10	0.01	-0.73
U4507	WLKEGVLGLVHEF	13	7.70	-0.90	0.39	0.52
U3462	RVDPVNFKLLSHCLLVT	17	10.03	1.03	0.18	0.67
U2125	KAVGKVIPELNGKLTGM	17	10.99	1.99	0.15	0.12
U1930	IAGVGAEILNVAKGIRSF	18	11.40	0.99	0.35	0.92
U1982	IFVKTLTGKTI	11	13.0	1.99	0.32	0.86
U2273	KVVAGVANALAHK	13	13.0	2.09	0.24	0.67

***^#^*** Total Molecular Charge given at pH = 7. * Eisenberg scale. **^&^** GRAVY (Grand Average of Hydropathy) is calculated as the sum of hydropathy values of all the amino acids, divided by the number of residues in the sequence [64]. Positive GRAVY values indicate hydrophobic; negative values mean hydrophilic.

## Data Availability

The web server presented in this manuscript, which evaluates the described models for ABP prediction and Gram staining type classification, is freely accessible at: https://protdcal.zmb.uni-due.de/ABP-Finder/index.php (accessed on 16 November 2022). The StarPep database, which was the source for all the in-silico data used to train and validate our models is accessible at: http://mobiosd-hub.com/starpep (accessed on 4 February 2020). WEKA was the machine-learning framework used for feature selection, hyper-parameters optimization, model training and validation steps. This program can be downloaded and installed following the guidelines at: https://waikato.github.io/weka-wiki/downloading_weka/ (accessed on 16 November 2022). ProtDCal descriptors, used to encode the peptide sequences into numeric vectors, can be computed directly from the web server: https://protdcal.zmb.uni-due.de/pages/form.php (accessed on 16 November 2022), using the project files given as Supplementary Material of this manuscript. The project files gather all the configuration of parameters used to obtain the initial set of descriptors screened in this work. The training, development, validation, and test datasets, as well as the boundaries of the applicability domains for the training and production models are included as part of the Supplementary Material of this work. The assessment of the applicability domain is also a feature implemented in our web server (ABP-Finder), therefore it is automatically done and reported by the server for any evaluated peptide.

## Supplementary Materials

The following supporting information can be downloaded at: https://www.mdpi.com/article/10.3390/antibiotics11121708/s1, The Supporting Information is available free of charge and includes the project files containing the setup used to compute all the descriptors employed in this work, and the AD of the datasets.
