# Supplementary material for: ABP-Finder: A Tool to Identify Antibacterial Peptides and the Gram-Staining Type of Targeted Bacteria
Source: Antibiotics (Basel). 2022 Nov 26;11(12):1708. doi: 10.3390/antibiotics11121708 (PMC9774453; doi:10.3390/antibiotics11121708)
Supplement: Supplementary file 1 [file antibiotics-11-01708-s001.zip › antibiotics-2012608-supplementary.pdf]

Supporting Information

ABP-Finder: A tool to identify antibacterial peptides and the Gram-staining type of targeted bacteria

Yasser B. Ruiz-Blanco[1*], Guillermin Agüero-Chapin[2,3], Sandra Romero-Molina[1], Agostinho Antunes[2,3], Lia-Raluca Olari[4], Barbara Spellerberg[5], Jan Münch[4], and Elsa Sanchez-Garcia[1*]

[1] Computational Biochemistry, Center of Medical Biotechnology, University of Duisburg-Essen, Essen, Germany.
[2] CIIMAR – Centro Interdisciplinar de Investigação Marinha e Ambiental, Universidade do Porto, Terminal de Cruzeiros do Porto de Leixões, Av. General Norton de Matos, s/n, 4450-208, Portugal,
[3] Departamento de Biologia, Faculdade de Ciências, Universidade do Porto, Rua do Campo Alegre, 4169-007 Porto, Portugal
[4] Institute of Molecular Virology, University Hospital Ulm, Ulm, Germany
[5] Institute of Medical Microbiology and Hygiene, University Hospital Ulm, Ulm, Germany

* Correspondence: ybruizblanco@gmail.com (Y.B.R.-B.); elsa.sanchez-garcia@uni-due.de (E.S.-G.)

Table of contents

Table S1. Number of peptides per type of Gram staining class in the training, development, validation, and test datasets.

Section S1. ProtDCal's configuration for vicinity operator Autocorrelation of grade 1 (AC1).

Section S2. ProtDCal's configuration for vicinity operator Autocorrelation of grade 2 (AC2).

Section S3. ProtDCal's configuration for vicinity operator Electrotopological State (EC).

Section S4. ProtDCal's configuration for no vicinity operator.

Section S5. List (FASTA format) of all the peptides in the training dataset.

Section S6. List (FASTA format) of all the peptides in the development dataset.

Section S7. List (FASTA format) of all the peptides in the validation dataset.

Section S8. List (FASTA format) of all the peptides in the test dataset.

Table S2. Minimum (Min) and maximum (Max) boundaries of the applicability domains defined for the models derived from the training and the production datasets. The first model distinguishes antibacterial peptides from non-antibacterial peptides (ABP) and the second model categorizes antibacterial peptides as anti-Gram+, anti-Gram- or anti- both types of bacteria (Gram).

Table S3. Values resulting from the radial diffusion assay applied to the peptide Urine-3462 against the *Pseudomonas aeruginosa* strain ATCC 27853.

Table S1. Number of peptides per type of Gram staining class in the training, development, validation and test datasets.

	Gram+	*Gram-*	*Broad Spectrum*
Training	351	478	4983
Development	52	105	911
Validation	37	82	546
Test	27	38	315

Section S1. ProtDCal's configuration for vicinity operator Autocorrelation of grade 1 (AC1).

directory:
Datasets/Fasta_Protein_Format
indices:
Gs(U),Gw(U),W(U),Mw,HP,Z1,IP,Z2,ECI,ISA,Z3
groups:
AHR,PCR,ARM,NPR,BSR,NCR,ALR,PLR,RTR,UCR,UFR,PRT
invariants:
N1,N2,N3,Ar,P2,P3,K,CV,Q1,RA,DE,Q2,S,MN,Q3,V,MX,I50,SI,MI,TI
parameters(t_cont,s_cont,A%,HydGroup,n,bins,K,SubG):
4.0,8.0,5.0,9.4,3.0,5,1,3
options(decimals,harmonicMeanType,geometricMeanType,windexID,datasetType,outputOrd
er):
2,0,0,0,fasta,true

Section S2. ProtDCal's configuration for vicinity operator Autocorrelation of grade 2 (AC2).

directory:
Datasets/Fasta_Protein_Format
indices:
Gs(U),Gw(U),W(U),Mw,HP,Z1,IP,Z2,ECI,ISA,Z3
groups:
AHR,PCR,ARM,NPR,BSR,NCR,ALR,PLR,RTR,UCR,UFR,PRT
invariants:
N1,N2,N3,Ar,P2,P3,K,CV,Q1,RA,DE,Q2,S,MN,Q3,V,MX,I50,SI,MI,TI
parameters(t_cont,s_cont,A%,HydGroup,n,bins,K,SubG):
4.0,8.0,5.0,9.4,3.0,5,2,3
options(decimals,harmonicMeanType,geometricMeanType,windexID,datasetType,outputOrder):
2,0,0,0,fasta,true

Section S3. ProtDCal's configuration for vicinity operator Electrotopological State (EC).

directory:
Datasets/Fasta_Protein_Format
indices:
Gs(U),Gw(U),W(U),Mw,HP,Z1,IP,Z2,ECI,ISA,Z3
groups:
AHR,PCR,ARM,NPR,BSR,NCR,ALR,PLR,RTR,UCR,UFR,PRT
invariants:
N1,N2,N3,Ar,P2,P3,K,CV,Q1,RA,DE,Q2,S,MN,Q3,V,MX,I50,SI,MI,TI
parameters(t_cont,s_cont,A%,HydGroup,n,bins,K,SubG):
4.0,8.0,5.0,9.4,3.0,5,5,3
options(decimals,harmonicMeanType,geometricMeanType,windexID,datasetType,outputOrder):
2,0,0,4,fasta,true

Section S4. ProtDCal's configuration for no vicinity operator

directory:
Datasets/Fasta_Protein_Format
indices:
Gs(U),Gw(U),W(U),Mw,HP,Z1,IP,Z2,ECI,ISA,Z3
groups:
AHR,PCR,ARM,NPR,BSR,NCR,ALR,PLR,RTR,UCR,UFR,PRT
invariants:
N1,N2,N3,Ar,P2,P3,K,CV,Q1,RA,DE,Q2,S,MN,Q3,V,MX,I50,SI,MI,TI
parameters(t_cont,s_cont,A%,HydGroup,n,bins,K,SubG):
4.0,8.0,5.0,9.4,3.0,5,5,3
options(decimals,harmonicMeanType,geometricMeanType,windexID,datasetType,outputOrder):
2,0,0,-1,fasta,true

Section S5. List (FASTA format) of all the peptides in the training dataset.

>ID00000-ABP_both
GIGAVLKVLTTGLPALISWIKRKRQQ
>ID00001-ABP_both
GIGKFLHSAKKFGKAFVGEIMNS
>ID00002-ABP_both
ILPWKWPWWPWRR
>ID00003-ABP_both
KWCFRVCYRGICYRRCR
>ID00004-ABP_both
LLGDFFRKSKEKIGKEFKRIVQRIKDFLRNLVPRTES
>ID00005-ABP_both
FFHHIFRGIVHVGKTIHRLVTG
>ID00006-ABP_both
FLPLIGRVLSGIL
>ID00007-ABP_both
FWGALAKGALKLIPSLFSSFSKKD
>ID00008-ABP_both
GWGSFFKKAAHVGKHVGKAALTHYL
>ID00009-ABP_both
ACNFQSCWATCQAQHSIYFRRAFCDRSQCKCVFVRG
>ID00010-ABP_both
ALWKTMLKKLGTMALHAGKAALGAAADTISQGTQ
>ID00012-ABP_both
FLPVLAGIAAKVVPALFCKITKKC
>ID00013-ABP_both
GLLSVLGSVAKHVLPHVVPVIAEHL
>ID00014-ABP_both
GLNTLKKVFQGLHEAIKLINNHVQ
>ID00015-ABP_both
HVDKKVADKVLLLKQLRIMRLLTRL
>ID00018-ABP_both
KWKLFKKIEKVGQNIRDGIIKAGPAVAVVGQATQIAK
>ID00019-ABP_both
LCNERPSQTWSGNCGNTAHCDKQCQDWEKASHGACHKRENHWKCFCYFNC
>ID00020-ABP_both
RGGRLCYCRRRFCVCVGR
>ID00021-ABP_both
ACYCRIPACIAGERRYGTCIYQGRLWAFCC
>ID00022-ABP_both
ALWKNMLKGIGKLAGKAALGAVKKLVGAES
>ID00023-ABP_both
DHYNCVSSGGQCLYSACPIFTKIQGTCYRGKAKCCK
>ID00024-ABP_both
DKLIGSCVWGAVNYTSDCNGECKRRGYKGGHCGSFANVNCWCET
>ID00025-ABP_both
FKCRRWQWRMKKLGAPSITCVRRAF
>ID00026-ABP_both
FLPAIAGMAAKFLPKIFCAISKKC
>ID00027-ABP_both

FLPAIAGVAAKFLPKIFCAISKKC
>ID00029-ABP_both
FLPFIAGMAAKFLPKIFCAISKKC
>ID00031-ABP_both
FLPIIAGVAAKVFPKIFCAISKKC
>ID00032-ABP_both
FWGALIKGAAKLIPSVVGLFKKKQ
>ID00033-ABP_neg
GFSSIFRGVAKFASKGLGKDLARLGVNLVACKISKQC
>ID00034-ABP_both
GIFSKLGRKKIKNLLISGLKNVGKEVGMDVVRTGIDIAGCKIKGEC
>ID00035-ABP_both
GKVWDWIKSAAKKIWSSEPVSQLKGQVLNAAKNYVAEKIGATPT
>ID00036-ABP_both
GLFDVIKKVASVIGGL
>ID00037-ABP_both
GLFGVLAKVAAHVVPAIAEHF
>ID00038-ABP_both
GLLDSLKGFAATAGKGVLQSLLSTASCKLAKTC
>ID00039-ABP_both
GLLKRIKTLL
>ID00040-ABP_both
GLLNTFKDWAISIAKGAGKGVLTTLSCKLDKSC
>ID00041-ABP_both
GSKKPVPIIYCNRRTGKCQRM
>ID00042-ABP_both
KWKLFKKIGAVLKVL
>ID00045-ABP_both
ALWFTMLKKLGTMALHAGKAALGAAANTISQGTQ
>ID00046-ABP_both
ATCDLLSGIGVQHSACALHCVFRGNRGGYCTGKGICVCRN
>ID00047-ABP_both
CYCRIPACIAGERRYGTCIYQGRLWAFCC
>ID00048-ABP_both
DCYCRIPACIAGERRYGTCIYQGRLWAFCC
>ID00050-NO
DKLIGSCVWGAVNYTSNCNAECKRRGYKGGHCGSFANVNCWCET
>ID00051-ABP_both
FFGWLIKGAIHAGKAIHGLIHRRRH
>ID00055-ABP_both
FLPILASLAAKFGPKLFCLVTKKC
>ID00056-ABP_both
FLPLLAGLAANFLPKIFCKITRKC
>ID00058-ABP_both
FLPMLAGLAASMVPKLVCLITKKC
>ID00059-ABP_both
FLPVVAGLAAKVLPSIICAVTKKC
>ID00060-ABP_both
FLSLIPHAINAVSAIAKHN
>ID00061-ABP_both
FVQWFSKFLGRIL

>ID00062-ABP_both
GFGALFKFLAKKVAKTVAKQAAKQGAKYVVNKQME
>ID00063-ABP_both
GFGCPLDQMQCHRHCQTITGRSGGYCSGPLKLTCTCYR
>ID00064-ABP_both
GFLGPLLKLAAKGVAKVIPHLIPSRQQ
>ID00065-ABP_both
GIFSKLAGKKIKNLLISGLKNVGKEVGMDVVRTGIDIAGCKIKGEC
>ID00066-ABP_both
GIGKFLKKAKKFGKAFVKILKK
>ID00067-ABP_both
GIGSAILSAGKSALKGLAKGLAEHFAN
>ID00068-ABP_both
GILDTIKSIASKVWNSKTVQDLKRKGINWVANKLGVSPQAA
>ID00069-ABP_both
GIMDTLKNLAKTAGKGALQSLLNKASCKLSGQC
>ID00070-ABP_both
GIPCGESCVWIPCISAALGCSCKNKVCYRN
>ID00072-ABP_both
GIWGTLAKIGIKAVPRVISMLKKKKQ
>ID00073-ABP_both
GLFDIIKKIAESF
>ID00074-ABP_both
GLFSILRGAAKFASKGLGKDLTKLGVDLVACKISKQC
>ID00075-ABP_both
GLLDSIKGMAISAGKGALQNLLKVASCKLDKTC
>ID00076-ABP_both
GLLGPLLKIAAKVGSNLL
>ID00077-ABP_both
GLWSKIKTAGKSVAKAAAKAAVKAVTNAV
>ID00079-ABP_both
GVLSNVIGYLKKLGTGALNAVLKQ
>ID00080-ABP_both
IDWKKLLDAAKQIL
>ID00081-ABP_both
IIGPVLGLVGSALGGLLKKI
>ID00082-ABP_both
INWKGIAAMAKKLL
>ID00083-ABP_both
MPCSCKKYCDPWEVIDGSCGLFNSKYICCREK
>ID00084-ABP_both
RGLRRLGRKIAHGVKKYGPTVLRIIRIAG
>ID00085-ABP_both
RQRVEELSKFSKKGAAARRRK
>ID00086-ABP_both
RSVCRQIKICRRRGGCYYKCTNRPY
>ID00087-ABP_both
SCASRCKGHCRARRCGYYVSVLYRGRCYCKCLRC
>ID00089-ABP_both
SWLSKTAKKLENSAKKRISEGIAIAIQGGPR
>ID00090-ABP_both

VDKGSYLPRPTPPRPIYNRN
>ID00094-ABP_both
WLNALLHHGLNCAKGVLA
>ID00095-ABP_pos
YRGGYTGPIPRPPPIGRPPLRLVVCACYRLSVSDARNCCIKFGSCCHLVK
>ID00096-ABP_both
AGRGKQGGKVRAKAKTRSSRAGLQFPVGRVHRLLRKGNY
>ID00097-ABP_both
AKIPIKAIKTVGKAVGKGLRAINIASTANDVFNFLKPKKRKH
>ID00098-ABP_both
ALWKDILKNVGKAAGKAVLNTVTDMVNQ
>ID00099-ABP_both
ALWMTLLKKVLKAAAKALNAVLVGANA
>ID00100-ABP_both
ATCDILSFQSQWVTPNHAGCALHCVIKGYKGGQCKITVCHCRR
>ID00101-ABP_both
CFCKRPVCDSGETQIGYCRLGNTFYRLCCRQ
>ID00102-ABP_both
DFKDWMKTAGEWLKKKGPGILKAAMAAAT
>ID00103-ABP_both
DSHAKRHHGYKRKFHEKHHSHRGY
>ID00104-ABP_both
ELCEKASKTWSGNCGNTGHCDNQCKSWEGAAHGACHVRNGKHMCFCYFNC
>ID00105-ABP_both
FIGLLISAGKAIHDLIRRRH
>ID00106-ABP_both
FIHHIFRGIVHAGRSIGRFLTG
>ID00107-ABP_both
FKLGSFLKKAWKSKLAKKLRAKGKEMLKDYAKGLLEGGSEEVPGQ
>ID00109-ABP_both
FLPIIASVAAKVFSKIFCAISKKC
>ID00110-ABP_both
FLPIIASVAANVFSKIFCAISKKC
>ID00111-ABP_both
FLPIIGKLLSGLL
>ID00112-ABP_both
FLPIVGKLLSGLL
>ID00114-ABP_both
GCASRCKAKCAGRRCKGWASASFRGRCYCKCFRC
>ID00115-ABP_both
GFVDFLKKVAGTIANVVT
>ID00116-ABP_both
GIINTLQKYYCRVRGGRCAVLSCLPKEEQIGKCSTRGRKCCRRKK
>ID00117-ABP_pos
GILDSFKQFAKGVGKDLIKGAAQGVLSTMSCKLAKTC
>ID00118-ABP_both
GILDTLKNLAISAAKGAAQGLVNKASCKLSGQC
>ID00120-ABP_both
GILSLVKGVAKLAGKGLAKEGGKFGLELIACKIAKQC
>ID00122-ABP_both
GIMSIVKDVAKTAAKEAAKGALSTLSCKLAKTC

>ID00123-ABP_both
GINTLKKVIQGLHEVIKLVSNHE
>ID00124-ABP_both
GKPRPYSPRPTSHPRPIRV
>ID00125-ABP_both
GLFDIIKKVASVVGGL
>ID00126-ABP_both
GLFGKLIKKFGRKAISYAVKKARGKH
>ID00127-ABP_both
GLLDTIKGVAKTVAASMLDKLKCKISGC
>ID00128-ABP_both
GLLSGLKKVGKHVAKNVAVSLMDSLKCKISGDC
>ID00129-ABP_both
GLLSVLGSVAKHVLPHVVPVIAEKL
>ID00130-ABP_both
GLNALKKVFQGIHEAIKLINNHVQ
>ID00131-ABP_both
GLPVCGETCVGGTCNTPGCTCSWPVCTRN
>ID00132-ABP_both
GLVDVLGKVGGLIKKLLP
>ID00133-ABP_both
GLWSKIKAAGKEAAKAAAKAAGKAALNAVSEAV
>ID00134-ABP_both
GLWSTIKQKGKEAAIAAAKAAGQAALGAL
>ID00135-ABP_both
GNNRPVYIPQPRPPHPRI
>ID00136-ABP_both
GRFKRFRKKFKKLFKKLSPVIPLLHLG
>ID00137-ABP_both
GRPNPVNNKPTPHPRL
>ID00138-ABP_both
GVFLDALKKFAKGGMNAVLNPK
>ID00139-ABP_both
GVIPCGESCVFIPCISTLLGCSCKNKVCYRN
>ID00140-ABP_both
GWKDWAKKAGGWLKKKGPGMAKAALKAAMQ
>ID00141-ABP_both
GWKDWLNKGKEWLKKKGPGIMKAALKAATQ
>ID00142-ABP_both
IFGAILPLALGALKNLIK
>ID00143-ABP_both
INLKALAALAKKIL
>ID00144-NO
KTCENLSGTFKGPCIPDGNCNKHCRNNEHLLSGRCRDDFRCWCTNRC
>ID00145-ABP_both
KWCFRVCYRGICYRKCR
>ID00146-ABP_both
KWKIFKKIEKVGRNIRNGIIKAGPAVAVLGEAKAL
>ID00148-ABP_both
LLKELWTKIKGAGKAVLGKIKGLL
>ID00149-ABP_both

QKLCERPSGTWSGVCGNNNACKN
>ID00151-ABP_both
RRWCFRVCYKGFCYRKCR
>ID00153-ABP_both
SHQDCYEALHKCMASHSKPFSCSMKFHMCLQQQ
>ID00154-ABP_both
SLFSLIKAGAKFLGKNLLKQGAQYAACKVSKEC
>ID00155-ABP_both
SYSMEHFRWGKPV
>ID00156-ABP_both
VFQFLGKIIHHVGNFVHGFSHVF
>ID00157-ABP_both
VFQFLGRIIHHVGNFVHGFSHVF
>ID00160-ABP_both
ACYCRIPACLAGERRYGTCFYMGRVWAFCC
>ID00161-ABP_both
AIKLVQSPNGNFAASFVLDGTKWIFKSKYYDSSKGYWVGIYEVWDRK
>ID00162-ABP_both
ALWKNMLKGIGKLAGQAALGAVKTLVGA
>ID00163-ABP_both
ALWKNMLKGIGKLAGQAALGAVKTLVGAES
>ID00165-ABP_both
ALYKKFKKKLLKSLKRLG
>ID00166-ABP_both
ATCDLLSGTGINHSACAAHCLLRGNRGGYCNGKAVCVCRN
>ID00167-ABP_both
ATCDLLSGTGVKHSACAAHCLLRGNRGGYCNGRAICVCRN
>ID00168-ABP_both
ATCYCRTGRCATRESLSGVCEISGRLYRLCCR
>ID00169-NO
CIANRNGCQPDGSQGNCCSGYCHKEPGWVAGYCR
>ID00170-NO
CIKNGNGCQPDGSQGNCCSRYCHKEPGWVAGYCR
>ID00171-ABP_both
DFASCHTNGGICLPNRCPGHMIQIGICFRPRVKCCRSW
>ID00172-ABP_both
FCTMIPIPRCY
>ID00173-ABP_both
FIGTALGIASAIPAIVKLFK
>ID00175-ABP_neg
FLPAIVGAAGKFLPKIFCAISKKC
>ID00177-ABP_both
FLPILASLAAKLGPKLFCLVTKKC
>ID00178-ABP_both
FLPKMSTKLRVPYRRGTKDYH
>ID00179-ABP_both
FRGLAKLLKIGLKSFARVLKKVLPKAAKAGKALAKSMADENAIRQQNQ
>ID00180-ABP_both
GAWKNFWSSLRKGFYDGEAGRAIRR
>ID00181-NO
GDPTFCGETCRVIPVCTYSAALGCTCDDRSDGLCKRN

>ID00182-ABP_both
GFFALIPKIISSPLFKTLLSAVGSALSSSGGQE
>ID00183-ABP_neg
GFGCPFNQGACHRHCRSIRRRGGYCAGLFKQTCTCYR
>ID00184-ABP_both
GFGSLFKFLAKKVAKTVAKQAAKQGAKYVANKHME
>ID00185-ABP_both
GFKDLLKGAAKALVKTVLF
>ID00186-ABP_both
GFLGSLLKTGLKVGSNLL
>ID00187-ABP_both
GFLSILKKVLPKVMAHMK
>ID00188-ABP_both
GFMKYIGPLIPHAVKAISDLI
>ID00189-ABP_both
GFWKKVGSAAWGGVKAAAKGAAVGGLNALAKHIQ
>ID00193-ABP_both
GIGKFLHSAGKFGKAFVGEIMKS
>ID00194-ABP_both
GILDAIKAIAKAAG
>ID00198-ABP_both
GILSLFTGGIKALGKTLFKMAGKAGAEHLACKATNQC
>ID00199-ABP_both
GILSSIKGVAKGVAKNVAAQLLDTLKCKITGC
>ID00200-ABP_both
GILSTFKGLAKGVAKDLAGNLLDKFKCKITGC
>ID00201-ABP_both
GIMDSVKGLAKNLAGKLLDSLKCKITGC
>ID00202-ABP_both
GKFSVFGKILRSIAKVFKGVGKVRKQFKTASDLDKNQ
>ID00203-ABP_both
GLLDIVKKVVGAFGSL
>ID00204-ABP_both
GLLNGLALRLGKRALKKIIKRLCR
>ID00205-ABP_both
GLLQTIKEKLESLESLAKGIVSGIQA
>ID00206-ABP_both
GLVSSIGRALGGLLADVVKSKGQPA
>ID00207-ABP_both
GMATKAGTALGKVAKAVIGAAL
>ID00208-ABP_both
GPLSCGRNGGVCIPIRCPVPMRQIGTCFGRPVKCCRSW
>ID00209-ABP_both
GVGDLIRKAVSVIKNIV
>ID00210-NO
GVPICGETCTLGTCYTAGCSCSWPVCTRN
>ID00214-ABP_both
ILGTILGLLKSL
>ID00215-ABP_both
ILPLVGNLLNDLL
>ID00216-ABP_both

ILQKAVLDCLKAAGSSLSKAAITAIYNKIT
>ID00217-ABP_both
INLKAIAALAKKLL
>ID00218-ABP_both
KDRPKKPGLCPPRPQKPCVKECKNDDSCPGQQKCCNYGCKDECRDPIFVG
>ID00219-ABP_both
KGRGKQGGKVRAKAKTRSS
>ID00221-ABP_both
LLGRCKVKSNRFHGPCLTDTHCSTVCRGEGYKGGDCHGLRRRCMCLC
>ID00223-ABP_both
RGGGLCYCRRRFCVCVGR
>ID00224-ABP_both
RIIDLLWRVRRPQKPKFVTVWVR
>ID00226-ABP_both
RVCFAIPLPICH
>ID00227-ABP_both
RVCYAIPLPIC
>ID00229-ABP_both
SGISGPLSCGRNGGVCIPIRCPVPMRQIGTCFGRPVKCCRSW
>ID00231-ABP_both
SIPCGESCVFIPCTVTALLGCSCKSKVCYKN
>ID00232-ABP_both
SLLSLIRKLIT
>ID00234-ABP_both
TRSSRAGLQFPVGRVHRLLRK
>ID00235-ABP_both
VCSCRLVFCRRTELRVGNCLIGGVSFTYCCTRV
>ID00236-ABP_both
VNWKKILGKIIKVVK
>ID00237-ABP_both
VRNFVTCRINRGFCVPIRCPGHRRQIGTCLGPQIKCCR
>ID00238-ABP_both
VRNHVTCRINRGFCVPIRCPGRTRQIGTCFGPRIKCCRSW
>ID00239-ABP_both
VTCDLLSFEAKGFAANHSICAAHCLAIGRKGGSCQNGVCVCRN
>ID00240-ABP_both
VTCDLLSFEAKGFAANHSICAAHCLVIGRKGGACQNGVCVCRN
>ID00241-ABP_both
VTCDLLSLQIKGIAINDSACAAHCLAMRRKGGSCKQGVCVCRN
>ID00243-ABP_both
VTCFCRRRGCASRERLIGYCRFGNTIYGLCCRR
>ID00245-ABP_both
WNPFKELERAGQRVRDAIISAGPAVATVAQATALAK
>ID00248-ABP_both
YVSCLFRGARCRVYSGRSCCFGYYCRRDFPGSIFGTCSRRNF
>ID00249-ABP_both
ACGILHDNCVYVPAQNPCCRGLQCRYGKCLVQV
>ID00250-ABP_both
ACSAG
>ID00251-ABP_both
ALWKTMLKKLGTMALHAGKAAFGAAADTISQ

>ID00252-ABP_both
APGNKAECEREKGYCGFLKCSFPFVVSGKCSRFFFCCKNIW
>ID00253-ABP_pos
ATCDLLSAFGVGHAACAAHCIGHGYRGGYCNSKAVCTCRR
>ID00254-ABP_both
ATCDLLSGFGVGDSACAAHCIARGNRGGYCNSKKVCVCRN
>ID00255-ABP_both
ATCDLLSGTGINHSACAAHCLLRGNRGGYCNGKGVCVCRN
>ID00256-NO
CIKNGNGCQPNGSQGNCCSGYCHKQPGWVAGYCRRK
>ID00261-ABP_both
DYDWSLRGPPKCATYGQKCRTWSPPNCCWNLRCKAFRCRPR
>ID00262-ABP_both
ELPKLPDDKVLIRSRSNCPKGKVWNGFDCKSPFAFS
>ID00263-ABP_both
FFGWLIRGAIHAGKAIHGLIHRRRH
>ID00264-ABP_both
FFPIGVFCKIFKTC
>ID00267-ABP_both
FISAIASMLGKFL
>ID00269-ABP_both
FLGGLMKAFPALICAVTKKC
>ID00270-ABP_both
FLIGMTQGLICLITRKC
>ID00271-ABP_both
FLPAVLRVAAKIVPTVFCAISKKC
>ID00272-ABP_both
FLPAVLRVAAQVVPTVFCAISKKC
>ID00273-ABP_both
FLPFIARLAAKVFPSIICSVTKKC
>ID00274-ABP_both
FLPFLATLLSKVL
>ID00278-ABP_both
FLPLFASLIGKLL
>ID00279-ABP_both
FLPLIGKVLSGIL
>ID00280-ABP_both
FLPLLLAGLPKLLCLFFKKC
>ID00282-ABP_both
FLSLALAALPKFLCLVFKKC
>ID00284-ABP_neg
FVPYNPPRPGQSKPFPSFPGHGPFNPKIQWPYPLPNPGH
>ID00285-ABP_both
FWGHIWNAVKRVGANALHGAVTGALS
>ID00286-ABP_both
GFFALIPKIISSPLFKTLLSAVGSALSSSGEQE
>ID00288-ABP_both
GFFSTVKNLATNVAGTVIDTLKCKVTGGCRS
>ID00289-ABP_both
GFGCPGNQLKCNNHCKSISCRAGYCDAATLWLRCTCTDCNGKK
>ID00290-ABP_both

GFGCPWNRYQCHSHCRSIGRLGGYCAGSLRLTCTCYRS
>ID00291-ABP_both
GFISTVKNLATNVAGTVIDTIKCKVTGGC
>ID00292-ABP_both
GFLSTVKNLATNVAGTVIDTLKCKVTGGCRS
>ID00293-NO
GFPCGESCVFIPCISAAIGCSCKNKVCYRN
>ID00294-ABP_both
GFSSLFKAGAKYLLKSVGKAGAQQLACKAANNCA
>ID00295-ABP_both
GFVDLAKKVVGGIRNALGI
>ID00296-ABP_both
GGAGHVPEYFVGIGTPISFYG
>ID00297-ABP_both
GGLRSLGRKILRAWKKYGPIIVPIIRIG
>ID00300-ABP_both
GIGDPVTCLKSGAICHPVFCPRRYKQIGTCGLPGTKCCKKP
>ID00301-ABP_both
GIGGVLLSAGKAALKGLAKVLAEKYAN
>ID00302-ABP_both
GILDFAKTVVGGIRNALGI
>ID00303-ABP_both
GILDSFKGVAKGVAKDLAGKLLDKLKCKITGC
>ID00304-ABP_both
GILSSFKGVAKGVAKDLAGKLLETLKCKITGC
>ID00305-ABP_both
GINTLKKVIQGLHEVIKLVSNHA
>ID00306-NO
GIPCGESCVFIPCLTTVAGCSCKNKVCYRN
>ID00307-ABP_both
GIPCGESCVWIPCISSAIGCSCKSKVCYRN
>ID00311-ABP_both
GLFDIAKKVIGVIGSL
>ID00312-ABP_both
GLFDIIKKIAESI
>ID00313-ABP_both
GLFDIIKKVASVIGGL
>ID00315-ABP_both
GLFDIVKKIAGHIASSI
>ID00316-ABP_both
GLFDIVKKIAGHIVSSI
>ID00318-ABP_both
GLFDIVKKVVGAIGSL
>ID00319-ABP_both
GLFDIVKKVVGALGSL
>ID00320-ABP_both
GLFDVVKGVLKGVGKNVAGSLLEQLKCKLSGGC
>ID00321-ABP_both
GLFGVLAKVASHVVPAIAEHFQA
>ID00322-ABP_both
GLFKVLGSVAKHLLPHVAPIIAEKL

>ID00323-ABP_both
GLFKVLGSVAKHLLPHVAPVIAEK
>ID00324-ABP_both
GLFLDTLKGAAKDVAGKLEGLKCKITGCKLP
>ID00325-ABP_both
GLFSVLGAVAKHVLPHVVPVIAEK
>ID00326-ABP_both
GLFSVLGSVAKHLLPHVAPIIAEKL
>ID00327-ABP_both
GLFSVLGSVAKHLLPHVVPVIAEKL
>ID00328-ABP_both
GLFSVVTGVLKAVGKNVAKNVGGSLLEQLKCKISGGC
>ID00329-ABP_both
GLFTLIKGAAKLIGKTVAKEAGKTGLELMACKITNQC
>ID00330-ABP_both
GLIGSIGKALGGLLVDVLKPKLQAAS
>ID00332-ABP_both
GLKEIFKAGLGSLVKGIAAHVAS
>ID00333-ABP_both
GLLDFVTGVGKDIFAQLIKQI
>ID00336-ABP_both
GLWSKIKEAAKTAGLMAMGFVNDMV
>ID00337-ABP_both
GMASKAGAIAGKIAKVALKAL
>ID00338-ABP_both
GNNRPVYIPQPRPPHPRL
>ID00339-ABP_both
GRKSDCFRKSGFCAFLKCPSLTLISGKCSRFYLCCKRIR
>ID00340-ABP_neg
GRPNPVNTKPTPYPRL
>ID00341-ABP_both
GRRKRKWLRRIGKGVKIIGGAALDHL
>ID00342-NO
GSVLNCGETCLLGTCYTTGCTCNKYRVCTKD
>ID00343-NO
GTACGESCYVLPCFTVGCTCTSSQCFKN
>ID00345-NO
GVIPCGESCVFIPCISAAIGCSCKNKVCYRN
>ID00346-ABP_both
GVLDAFRKIATVVKNVV
>ID00347-ABP_both
GVWSTVLGGLKKFAKGGLEAIVNPK
>ID00349-ABP_both
GWLRKIGKKIERVGQHTRDATIQVLGIAQQAANVAATAR
>ID00350-ABP_both
HGVSGHGQHGVHG
>ID00351-ABP_both
ICIFCCGCCHRSKCGMCCKT
>ID00352-ABP_both
IGKEFKRIVQRIKDFLRNLVPRTES
>ID00353-ABP_both

ILGPVISTIGGVLGGLLKNL
>ID00354-ABP_both
INLLKIAKGIIKSL
>ID00355-ABP_both
INWKGIAAMKKLL
>ID00357-ABP_both
KIKWFKTMKSIAKFIAKEQMKKHLGGE
>ID00358-NO
KIPCGESCVWIPCLTSVFNCKCENKVCYHD
>ID00359-ABP_both
KNLRRIIRKGIHIIKKYG
>ID00360-ABP_both
KNLRRITRKIIHIIKKYG
>ID00361-ABP_both
KWKVFKKIEKMGRNIRNGIVKAGPAIAVLGEAKAL
>ID00363-ABP_both
KYYGNGVSCNKKGCSVDWGKAIGIIGNNSAANLATGGAAGWSK
>ID00364-ABP_both
LFCRKGTCHFGGCPAHLVKVGSCFGFRACCKWPWDV
>ID00365-ABP_both
LKLKSIVSWAKKVL
>ID00366-ABP_both
LNLKGIFKKVASLLT
>ID00367-ABP_both
LPVNEAQCRQVGGYCGLRICNFPSRFLGLCTRNHPCCSRVWV
>ID00368-ABP_both
LSCKRGTCHFGRCPSHLIKGSCSGG
>ID00371-ABP_both
NFLGTLINLAKKIM
>ID00372-ABP_both
QCRRLCYKQRCVTYCRGR
>ID00373-NO
QKLCERPSGTWSGVCGNNNACKNQCIN
>ID00374-ABP_pos
QQCGRQASGRLCGNRLCCSQWGYCGSTASYCGAGCQSQCRS
>ID00376-NO
QTCASRCPRPCNAGLCCSIYGYCGSGNAYCGAGNCRCQCRG
>ID00377-ABP_both
RGFRKHFNKLVKKVKHTISETAHVAKDTAVIAGSGAAVVAAT
>ID00379-ABP_both
RLCRIVVIRVCR
>ID00380-ABP_both
RPKHPIKHQGLPQEVLNENLLRF
>ID00381-ABP_both
RWCVYAYVRVRGVLVRYRRCW
>ID00383-ABP_both
SAPRGCWTKSYPPKPCK
>ID00384-ABP_both
SIGSALKKALPVAKKIGKIALPIAKAALP
>ID00385-ABP_both
SISCGESCAMISFCFTEVIGCSCKNKVCYLN

>ID00386-ABP_both
SWKSMAKKLKEYMEKLKQRA
>ID00387-ABP_both
TKYYGNGVYCNSKKCWVDWGTAQGCIDVVIGQLGGGIPGKGKC
>ID00388-ABP_both
TTKNYGNGVCNSVNWCQCGNVWASCNLATGCAAWLCKLA
>ID00389-ABP_both
VDKPDYRPRPWPRPN
>ID00392-ABP_pos
VNYGNGVSCSKTKCSVNWGQAFQERYTAGINSFVSGVASGAGSIGRRP
>ID00394-ABP_pos
YITCLFRGARCRVYSGRSCCFGYYCRRDFPGSIFGTCSRRNF
>ID00395-ABP_both
YKQCHKKGGHCFPKEKICLPPSSDFGKMDCRWRWKCCKKGSG
>ID00397-ABP_both
AAGMGFFGAR
>ID00398-ABP_both
ACYCRIPACFAGERRYGTCFYLGRVWAFCC
>ID00401-ABP_both
ALFSILRGLKKLGNMGQAFVNCKIYKKC
>ID00402-ABP_both
ALLHHGLNCAKGVLA
>ID00403-ABP_both
ALRLAIRKR
>ID00406-ABP_pos
ASIIKTTIKVSKAVCKTLTCICTGSCSNCK
>ID00408-ABP_both
AVPDVAFNAYG
>ID00409-ABP_both
AWLLAIRKR
>ID00410-ABP_pos
AYPGNGVHCGKYSCTVDKQTAIGNIGNNAA
>ID00411-ABP_both
DFGCGQGMIFMCQRRCMRLYPGSTGFCRGFRCMCDTHIPLRPPFMVG
>ID00412-ABP_both
DIQIPGIKKPTHRDIIIPNWNPNVRTQPWQRFGGNKS
>ID00413-ABP_both
DKLIGSCVWGATNYTSDCNAECKRRGYKGGHCGSFWNVNCWCEE
>ID00414-ABP_both
DYHHGVRVL
>ID00415-ABP_both
EADEPLWLYKGDNIERAPTTADHPILPSIIDDVKLDPNRRYA
>ID00416-ABP_both
EIRLPEPFRFPSPTVPKPIDIDPILPHPWSPRQTYPIIARRS
>ID00418-ABP_both
FIGPIISALASLFG
>ID00419-ABP_both
FLGALIKGAIHGGRFIHGMIQNHH
>ID00420-ABP_both
FLGLLFHGVHHVGKWIHGLIHGHH
>ID00421-ABP_both

FLGSIVGALASALPSLISKIRN
>ID00422-ABP_both
FLPAIAGILSQLF
>ID00423-ABP_both
FLPAIVGAAAKFLPKIFCAISKKC
>ID00424-ABP_pos
FLPFLAKILTGVL
>ID00425-ABP_both
FLPFLASLLSKVL
>ID00427-ABP_pos
FLPIASLLGKYL
>ID00429-ABP_both
FLPIIAGIAAKFLPKIFCTISKKC
>ID00430-ABP_both
FLPILAGLAAKIVPKLFCLATKKC
>ID00431-ABP_both
FLPILAGLAAKLVPKVFCSITKKC
>ID00432-ABP_both
FLPILAGLAANILPKVFCSITKKC
>ID00433-ABP_both
FLPILINLIHKGLL
>ID00434-ABP_both
FLPLLFGAISHLL
>ID00436-ABP_both
FLSIIAKVLGSLF
>ID00437-ABP_both
FLSLIPHAINAVSAIAKHF
>ID00440-ABP_both
GAIKDALKGAAKTVAVELLKKAQCKLEKTC
>ID00441-ABP_both
GCRFCCNCCPNMSGCGVCCRF
>ID00442-ABP_both
GFCRCLCRRGVCRCICTR
>ID00443-ABP_both
GFGCNGPWDEDDMQCHNHCKSIKGYKGGYCAKGGFVCKCY
>ID00445-ABP_both
GFGSLLGKALKIGTNLL
>ID00446-ABP_both
GFKGAFKNVMFGIAKSAGKSALNALACKIDKSC
>ID00447-ABP_both
GFLDIINKLGKTFAGHMLDKIKCTIGTCPPSP
>ID00448-ABP_both
GFLDSFKNAMIGVAKSVGKTALSTLACKIDKSC
>ID00451-ABP_neg
GFLSTVKNLATNVAGTVLDTIRCKVTGGCRP
>ID00452-ABP_both
GIFNVFKGALKTAGKHVAGSLLNQLKCKVSGEC
>ID00454-ABP_both
GIFPKIIGKGIKTGIVNGIKSLVKGVGMKVFKAGLSNIGNTGCNEDEC
>ID00455-ABP_both
GIFPKIIGKGIVNGIKSLAKGVGMKVFKAGLNNIGNTGCNNRDEC

>ID00460-ABP_both
GILDTLKQFAKGVGKDLVKGAAQGVLSTVSCKLAKTC
>ID00462-ABP_both
GIMSLFKGVLKTAGKHVAGSLVDQLKCKITGGC
>ID00463-NO
GIPCGESCVFIPCITSVAGCSCKSKVCYRN
>ID00466-ABP_both
GLADFLNKAVGKVVDFVKS
>ID00467-ABP_both
GLASTLGSFLGKFAKGGAQAFLQPK
>ID00468-ABP_both
GLFDVVKGVLKGAGKNVAGSLLEQLKCKLSGGC
>ID00469-ABP_both
GLFKVLGSVAKHLLPHVVPVIAEK
>ID00470-ABP_both
GLFLDTLKGLAGKLLQGLKCIKAGCKP
>ID00471-ABP_both
GLFPKFNKKKVKTGIFDIIKTVGKEAGMDVLRTGIDVIGCKIKGEC
>ID00473-ABP_both
GLGSLLGKAFKIGLKTVGKMMGGAPREQ
>ID00474-ABP_both
GLLDSFKNAMIGIAKSAGKTALNKIACKIDKTC
>ID00476-ABP_both
GLLGGLLGPLLGGGGGGGGGLL
>ID00477-ABP_both
GLLGSIFGAGKKIACALSGLC
>ID00479-ABP_both
GLLSKVLGVGKKVLCGVSGLC
>ID00480-ABP_both
GLPRKILCAIAKKKGKCKGPLKLVCKC
>ID00482-ABP_both
GLWEKVKEKANELVSGIVEGVK
>ID00483-ABP_both
GLWSKIKEAAKAAGKAALNAVTGLVNQGDQPS
>ID00485-ABP_both
GNNRPIYIPQPRPPHPRL
>ID00486-ABP_neg
GRGREFMSNLKEKLSGVKEKMKNS
>ID00487-ABP_both
GRLQAFLAKMKEIAAQTL
>ID00490-ABP_both
GVIDAAKKVVNVLKNLP
>ID00491-ABP_neg
GVLDILKNAAKNILAHAAEQI
>ID00492-ABP_both
GVVDILKGAAKDIAGHLASKVMNKL
>ID00493-ABP_neg
GVVDILKGAGKDLLAHLVGKISEKV
>ID00494-ABP_both
GWGSIFKHGRHAAKHIGHAAVNHYL
>ID00495-ABP_both

GWIRDFGKRIERVGQHTRDATIQTIAVAQQAANVAATLKG
>ID00496-ABP_both
GWKKWLRKGAKHLGQAAIK
>ID00497-ABP_both
GWLKKIGKKIERVGQHTRDATIQGLGIAQQAANVAATAR
>ID00498-ABP_both
HFLGTLVNLAKKIL
>ID00500-ABP_both
HSDAVFTDNYTRLRKQMAVKKYLNSILN
>ID00501-ABP_both
HSHACTSYWCGKFCGTASCTHYLCRVLHPGKMCACVHCSR
>ID00502-ABP_both
IGGIISFFKRLF
>ID00503-ABP_both
IIEKLVNTALGLLSGL
>ID00504-ABP_both
IIGHLIKTALGMLGL
>ID00506-ABP_both
ILPLLLGKVVCAITKKC
>ID00507-ABP_both
INLKAIAAMAKKLL
>ID00508-ABP_both
INWKKIAEIGKQVLSAL
>ID00509-ABP_both
INWKKIAEVGGKILSSL
>ID00510-ABP_both
INWLKLGKAIIDAL
>ID00511-ABP_pos
ITSISLCTPGCKTGALMGCNMKTATCHCSIHVSK
>ID00512-ABP_both
KNLRRIIRKIIHIIKKYG
>ID00514-ABP_both
KRLFKKLLFSLRKY
>ID00515-ABP_both
KTCMTKKEGWGRCLIDTTCAHSCRKYGYMGGKCQGITRRCYCLLNC
>ID00517-ABP_both
KWKLFKKIGIGKFLHSAKKF
>ID00518-ABP_both
KWKLFKKIKFLHSAKKF
>ID00519-ABP_both
KWKLFKKIPKFLHLAKKF
>ID00520-ABP_pos
KYYGNGVHCGKHSCTVDWGTAIGNIGNNAAANWATGGNAGWNK
>ID00522-ABP_both
LAHQKPFIRKSYKCLHKRCR
>ID00523-ABP_both
LFCKGGSCHFGGCPSHLIKVGSCFGFRSCCKWPWNA
>ID00524-ABP_both
LLGMIPLAISAISALSKL
>ID00527-ABP_both
LLPIVGNLLKSLL

>ID00528-ABP_both
LRQSQFVGSR
>ID00530-ABP_both
NLCERASLTWTGNCGNTGHCDTQCRNWESAKHGACHKRGNWKCFCYFNC
>ID00534-ABP_both
QGVRNFVTCRINRGFCVPIRCPGHRRQIGTCLGPRIKCCR
>ID00537-ABP_both
RCLPAGKTCVRGPMRVPCCGSCSQNKCT
>ID00539-ABP_both
RKSKEKIGKEFKRIVQRIKDFLRNLVPRTES
>ID00540-ABP_both
RRICRCRIGRCLGLEVYFGVCFLHGRLARRCCR
>ID00541-ABP_both
RTCRCRLGRCSRRESYSGSCNINGRIYSLCCR
>ID00543-ABP_both
RWCFRVCYRGICYRKCR
>ID00544-ABP_both
RWCVYAYVRIRGVLVRYRRCW
>ID00545-ABP_both
RWGKWFKKATHVGKHVGKAALTAYL
>ID00549-ABP_both
SIVPIRCRSNRDCRRFCGFRGGRCTYARQCLCGY
>ID00550-ABP_both
SMLSVLKNLGKVGLGFVACKINKQC
>ID00551-ABP_both
SMLSVLKNLGKVGLGLVACKINKQC
>ID00552-ABP_both
SMWSGMWRRKLKKLRNALKKKLKGE
>ID00553-ABP_both
SVAGRAQGM
>ID00557-ABP_both
VGALAVVVWLWLWLW
>ID00559-ABP_both
VGKTWIKVIRGIGKSKIKWQ
>ID00561-ABP_both
VTCDVLSFEAKGIAVNHSACALHCIALRKKGGSCQNGVCVCRN
>ID00562-ABP_pos
VTSWSLCTPGCTSPGGGSNCSFCC
>ID00563-ABP_both
YDLSKNCRLRGGICYIGKCPRRFFRSGSCSRGNVCCLRFG
>ID00565-ABP_both
YSRCQLQGFNCVVRSYGLPTIPCCRGLTCRSYFPGSTYGRCQRY
>ID00566-ABP_both
AACSDRAHGHICESFKSFCKDSGRNGVKLRANCKKTCGLC
>ID00567-ABP_both
ACQCPDAISGWTHTDYQCHGLENKMYRHVYAICMNGTQVYCRTEWGSSC
>ID00568-ABP_both
AFTCHCRRSCYSTEYSYGTCTVMGINHRFCCL
>ID00570-ABP_both
AIGSILGALAKGLPTLISWIKNR
>ID00571-ABP_both

AIMDTIKDTAKTVAVGLLNKLKCKITGC
>ID00573-ABP_both
AKKVFKRLEKLFSKIQNDK
>ID00575-ABP_both
ALLLAIRKR
>ID00576-ABP_both
ALSILRGLEKLAKMGIALTNCKATKKC
>ID00577-ABP_both
ALWKDILKNAGKAALNEINQIVQ
>ID00578-ABP_both
ALWKDVLKKIGTVALHAGKAALGAVADTISQ
>ID00579-ABP_both
ALWLAIRKR
>ID00580-ABP_both
ANTAFVSSAHNTQKIPAGAPFNRNLRAMLADLRQNAAFAG
>ID00583-NO
ARCENFADSYRQPPISSSQT
>ID00586-NO
ATITVVNRCSYTVWPGALPGGGVRLDPGQRWALNMPAGTAGAAV
>ID00587-ABP_pos
ATRSYGNGVYCNNSKCWVNWGEAKENIAGIVISGWASGLAGMGH
>ID00588-ABP_pos
ATYYGNGLYCNKEKCWVDWNQAKGEIGKIIVNGWVNHGPWAPRR
>ID00590-NO
AVRIGPCDQVCPRIVPERHECCRAHGRSGYAYCSGGGMYCN
>ID00591-ABP_pos
CANSCSYGPLTWSCDGNTK
>ID00592-ABP_both
CIAKGNGCQPSGVQGNCCSGHCHKEPGWVAGYCK
>ID00593-ABP_both
CKQSCSFGPFTFVCDGNTK
>ID00594-ABP_pos
CTFTLPGGGGVCTLTSECIC
>ID00598-ABP_both
DLHIPPPDNKINWPQLSGGGGGSPKTGYDININAQQK
>ID00599-ABP_both
DLWNSIKDMAAAAGRAALNAVTGMVNQ
>ID00601-ABP_both
EVERKHPLGGSRPGRCPTVPPGTFGHCACLCTGDASEPKGQKCCSN
>ID00603-ABP_both
FFHHIFRGIVHVGKTIHKLVTG
>ID00604-ABP_both
FFPIIAGMAAKLIPSLFCKITKKC
>ID00605-ABP_both
FFRLLFHGVHHGGGYLNAA
>ID00606-ABP_both
FFRLLFHGVHHVGKIKPRA
>ID00612-ABP_both
FKRIVQRIKDFLR
>ID00613-ABP_both
FLFSLIPSAISGLISAFK

>ID00614-ABP_both
FLGGLIKIVPAMICAVTKKC
>ID00615-ABP_both
FLGGLMKIIPAAFCAVTKKC
>ID00616-ABP_both
FLIGMTHGLICLISRKC
>ID00617-ABP_both
FLIIRRPIVLGLL
>ID00618-ABP_both
FLPAVLLVATHVLPTVFCAITRKC
>ID00619-ABP_both
FLPIIAGAAAKVVEKIFCAISKKC
>ID00621-ABP_both
FLPIIAGVAAKVLPKLFCAITKKC
>ID00622-ABP_pos
FLPIIGQLLSGLL
>ID00623-ABP_both
FLPIITNLLGKLL
>ID00624-ABP_both
FLPILGKLLSGIL
>ID00625-ABP_both
FLPIPRPILLGLL
>ID00626-ABP_both
FLPIVGKLLSGLSGLS
>ID00627-ABP_both
FLPIVGRLISGLL
>ID00628-ABP_both
FLPLAVSLAANFLPKLFCKITKKC
>ID00629-ABP_both
FLPLLASLFSRLF
>ID00630-ABP_both
FLPLLLAGLPLKLCFLFKKC
>ID00631-ABP_both
FLPLVTGLLSGLL
>ID00632-ABP_both
FLPLVTMLLGKLF
>ID00636-ABP_both
FLRFIGSVIHGIGHLVHHIGVAL
>ID00637-ABP_both
FLSLALAALPKLFCLIFKKC
>ID00638-ABP_both
FLSLIPHAINAVSTLVHHF
>ID00640-ABP_both
FLSLIPHIVSGVASIAKHF
>ID00641-ABP_both
FLSSIGKILGNLL
>ID00642-ABP_both
FMPIIGRLMSGSL
>ID00643-ABP_both
FRFGSFLKKVWKSKLAKKLRSKGKQLLKDYANKVLNGPEEEAAAPAE
>ID00646-NO

GADFQECMKEHSQKQHQHQG
>ID00648-ABP_both
GFFGKMKEYFKKFGASFKRRFANLKKRL
>ID00649-ABP_both
GFFSLIKGVAKIATKGLAKNLGKMGLDLVGCKISKEC
>ID00650-ABP_both
GFGCPLNQGACHRHCRSIRRRGGYCAGFFKQTCTCYRN
>ID00651-ABP_both
GFGCPNNYQCHRHCKSIPGRCGGYCGGWHRLRCTCYRC
>ID00652-ABP_both
GFGKAFHSVSNFAKKHKTA
>ID00653-ABP_both
GFGSFLGKALKAALKIGANVLGGAPEQ
>ID00654-ABP_both
GFGSFLGKALKAALKIGANVLGGAPQQ
>ID00655-ABP_both
GFGSLFKFLAKKVAKTVAKQAAKQGAKYIANKQTE
>ID00660-ABP_both
GFMDTAKNVAKNVAATLLDKLKCKITGGC
>ID00661-ABP_both
GFMNTAKNVAKNVAVTLLDNLKCKITGGC
>ID00662-ABP_both
GFRDVLKGAAKAFVKTVAGHIAN
>ID00663-ABP_both
GFSSIFRGVAKFASKGLGKDLAKLGVDLVACKISKQC
>ID00664-ABP_both
GFWGKLFKLGLHGIGLLHLHL
>ID00666-ABP_both
GIFDVVKGVLKGVGKNVAGSLLEQLKCKLSGGC
>ID00667-ABP_both
GIFGKILGVGKKTLCELSGMC
>ID00670-ABP_both
GIFSKISGKAIKNLFIKGAKNVGKEVGMDVVRTGIDVVGCKIKGEC
>ID00671-ABP_both
GIFSKISGKAIKNLFIKGAKNVGKRVGMDVVRTGMDVVGCKIKGEC
>ID00672-ABP_both
GIFTLIKGAAKLIGKTVAKEAGKTGLELMACKITNQC
>ID00673-ABP_both
GIGASILSAGKSALKGLAKGLAEHFAN
>ID00674-ABP_both
GIGGKILSGLKTALKGAAKELASTYLH
>ID00675-ABP_both
GIGTKILGGVKTALKGALKELASTYAN
>ID00676-ABP_both
GILGNIVGMGKKIVCGLSGLC
>ID00677-ABP_both
GILGNIVGMGKKVVCGLSGLC
>ID00679-ABP_both
GILSGVLGMGKKIVCGLRGLC
>ID00680-ABP_both
GILSGVLGMGKKIVCGLSGLC

>ID00683-NO
GIPCAESCVWIPCTVTALVGCSCSDKVCYN
>ID00684-ABP_both
GIRCPKSWKCKAFKQRVLKRLLAMLRQHAF
>ID00685-ABP_both
GIWDTIKSMGKVFAGKILQNL
>ID00686-ABP_both
GKGRWLERIGKAGGIIIGGALDHL
>ID00688-ABP_both
GKLSGISKVLRAIAKFFKGVGKARKQFKEASDLDKNQ
>ID00689-ABP_both
GLASFLGKALKAGLKIGSHLLGGAPQQ
>ID00690-ABP_both
GLFDIIKNIFSGL
>ID00691-ABP_both
GLFGKILGVGKKVLCGLSGMC
>ID00692-ABP_both
GLFGVLGSIAKHVLPHVVPVIAEKL
>ID00693-ABP_both
GLFLDTLKGAAKDVAGKLLEGLKCKIAGCKP
>ID00694-ABP_both
GLFSKFAGKGIKNLIFKGVKHIGKEVGMDVIRTGIDVAGCKIKGEC
>ID00695-ABP_both
GLFSKFNKKKIKSGLFKIIKTAGKEAGLEALRTGIDVIGCKIKGEC
>ID00696-ABP_both
GLFSKFNKKKIKSGLIKIIKTAGKEAGLEALRTGIDVIGCKIKGEC
>ID00697-ABP_both
GLFSKFSGKGIKNFLIKGVKHIGKEVGMDVIRTGIDVAGCKIKGEC
>ID00698-ABP_both
GLFTLIKGAAKLIGKTTAKEAGKTGKLEMACKITNQC
>ID00700-ABP_both
GLGGAKKNFIIAANKTAPQSVKKTFSCKLYNG
>ID00701-ABP_pos
GLGKAQCAALWLQCASGGTIGCGGGAVACQNYRQFCR
>ID00703-ABP_both
GLIDTIKNMALNAAKSAGVSVLNTLSCKLSKTC
>ID00704-ABP_both
GLKDKFKSMGEKLKQYIQTWKAKF
>ID00705-ABP_both
GLLDGILNANFNAAKSAGTSVLNALSCKLSKTC
>ID00706-ABP_both
GLLDTFKNLALNAAKSAGVSVLNSLSCKLSKTC
>ID00707-ABP_both
GLLDTFKNMAINAAHGAGVSVLNALSCKLKKTC
>ID00708-ABP_both
GLLDTIKNMALNAAKSAGVSVLNSLSCKDSKTC
>ID00709-ABP_both
GLLDTIKNMALNAAKSAGVSVLNSLSCKLSKTC
>ID00711-ABP_both
GLLDTLKGAAKNVVGSLASKVMEKL
>ID00712-ABP_both

GLLDTLKNMAINAAKGAGQSVLNTLSCKLSKTC
>ID00714-ABP_both
GLLLDTLKGAAKDIAGIALEKLKCKITGCKP
>ID00715-ABP_both
GLLRASSVWGRKYYVDLAGCAKA
>ID00716-ABP_both
GLLSGILGAGKNIVCGLSGLC
>ID00717-ABP_both
GLLSGILGAGKQKVCGLSGLC
>ID00718-ABP_both
GLLSRLRDFLSDRGRRLGEKIERIGQKIKDLSEFFQS
>ID00719-ABP_both
GLLSVLGSVVKHVIPHVVPVIAEHL
>ID00720-ABP_both
GLMDTIKGVAKTVAASWLDKLKCKITGC
>ID00721-ABP_both
GLMDTVKNAAKNLAGQLLDTIKCKMTGC
>ID00722-ABP_both
GLMDVFKGAAKNLLASALDKIRCKVTKC
>ID00723-ABP_both
GLMSLFKGVLKTAGKHIFKNVGGSLLDQAKCKITGEC
>ID00725-ABP_both
GLMSVLKGVLKTAGKHIFKNVGGSLLDQAKCKITGQC
>ID00726-ABP_both
GLPTCGETCTLGTCYVPDCSCSWPICMKN
>ID00727-ABP_both
GLPVCGETCFGGTCNTPGCSCTWPICTRD
>ID00728-ABP_both
GLPVCGETCVGGTCNTPGCSCSWPVCTRN
>ID00729-ABP_both
GLRKKFRKTRKRIQKLGRKIGKTGRKVWKAWREYGQIPYPCRI
>ID00730-ABP_both
GLRKRLRKFRNKIKEKLKKIGQKIQGFVPKLAPRTDY
>ID00731-ABP_both
GLVTSLIKGAGKLLGGLFGSVTGGQS
>ID00733-ABP_both
GLWSTIKNVGKEAAIAAGKAALGAL
>ID00735-ABP_both
GMASKAGSVLGKVAKVALKAAL
>ID00736-ABP_both
GNAACVIGCIGSCVISEGIGSLVGTAFTLG
>ID00737-ABP_both
GRFKRFRKKFKKLFKKLS
>ID00738-ABP_both
GRILSFIKGLAEHL
>ID00739-ABP_both
GRRRRSVQWCA
>ID00740-ABP_both
GRRRRSVQWCAVSQPEATKCFQWQRNMRKVRGPPVSCIKRDSPIQCIQA
>ID00741-ABP_both
GVFSFLKTGAKLLGSTLLKMAGKAGAEHLACKATNQC

>ID00742-ABP_both
GVFTLIKGATQLIGKTLGKELGKTGLEIMACKITKQC
>ID00743-ABP_both
GVFTLIKGATQLIGKTLGKELGKTGLELMACKITEQC
>ID00744-ABP_both
GVFTLIKGATQLIGKTLGKELGKTGLELMACKITNQC
>ID00747-ABP_both
GVIKSVLKGVAKTVALGML
>ID00748-ABP_both
GVLATVKNLLIGTGDGAAQSVLKTLSCKLSNDC
>ID00749-ABP_both
GVLATVKNLLNGTGDGAAQSVLKTLSCKLSNDC
>ID00750-ABP_both
GVLGTVKDLLIGAGKSAAQSTLKTLSCKISNDC
>ID00751-ABP_both
GVLGTVKNLLIGAGKSAAQSVLKTLSCKLSNDC
>ID00752-NO
GVPCGESCVFIPCITGVIGCSCSSNVCYLN
>ID00754-ABP_both
GVVDILKGAAKDLAGHLATKVMNKL
>ID00755-ABP_both
GVVTDLLKTAGKLLGNLVGSLSG
>ID00756-ABP_both
GWANTLKNVAGGLCKITGAA
>ID00759-ABP_both
GWKKWLRKGAKHLGQAAIKGLAS
>ID00762-ABP_both
HPLKQYWWRPSI
>ID00763-ABP_both
HRHQGPIFDTRPSPFNPNQPRPGPIY
>ID00764-ABP_both
HSSGYTRPLRKPSRPIFIRPIGCDVCYGIPSSTARLCCFRYGDCCHL
>ID00765-ABP_both
IDPFVAGVAAEMMQHVYCAASKKC
>ID00766-ABP_both
IDWLKLGKMVMDVL
>ID00767-ABP_both
IFGAIAGLLKNIF
>ID00768-ABP_both
IIGHLIKTALGFLGL
>ID00769-ABP_both
IIGPVLGMVGSALGGLLKKI
>ID00770-ABP_both
IIPFVAGVAAEMMEHVYCAASKKC
>ID00771-ABP_both
IIPLPLGYFAKKT
>ID00772-ABP_both
ILGKLLSTAAGLLSNL
>ID00773-ABP_both
ILGPVISKIGGVLGGLLKNL
>ID00774-ABP_both

ILGPVLGLVGNALGGLIKKI
>ID00775-ABP_both
ILGPVLGLVSDTLDDVLGIL
>ID00777-ABP_both
ILGPVLSMVGSALGGLIKKI
>ID00778-ABP_both
ILPFVAGVAAEMMEHVYCAASKKC
>ID00779-ABP_both
ILPFVAGVAAEMMKHVYCAASKKC
>ID00780-ABP_both
ILPFVAGVAAEMMQHVYCAASKKC
>ID00781-ABP_both
ILPFVAGVAAMEMEHVYCAASKKC
>ID00782-ABP_both
ILPILGNLLNGLL
>ID00783-ABP_both
ILPILGNLLNSLL
>ID00784-ABP_both
INMKASAAVAKKLL
>ID00785-ABP_both
INPFVAGVAAEMMQHVYCAASKKC
>ID00786-ABP_both
INWKKIASIGKEVLKAL
>ID00787-ABP_both
INWKKIFEKVKNLV
>ID00788-ABP_both
INWLKLGKKILGAL
>ID00789-ABP_both
INWLKLGKKMMSAL
>ID00790-ABP_both
IPPFIKKVLTTVF
>ID00791-ABP_both
ISRLAGLLRKGGEKIGEKLKKIGQKIKNFFQKLVPQPE
>ID00795-ABP_both
KFFRKLKKSVKKRAKEFFKKPRVIGVSIPF
>ID00796-ABP_both
KGIGSALKKGGKIIKGGLGALGAIGTGQQVYEHVQNRQ
>ID00797-NO
KIPCGESCVWIPCVTSIFNCKCENKVCYHD
>ID00798-ABP_both
KKLLKWLKKLL
>ID00803-ABP_both
KRFKKFFKKLKNSVKKRAKKFFKKPKVIGVTFPF
>ID00804-ABP_both
KRIVQRIKDFLR
>ID00808-NO
KTCENLADDY
>ID00809-ABP_both
KVPIGAIKKGGKIIKKGLGVIGAAGTAHEVYSHVKNRH
>ID00811-ABP_both
KVPIGAIKKGGKIIKKGLGVLGAAGTAHEVYNHVRNRQ

>ID00812-ABP_both
KVPVGAIKKGGKAIKTGLGVVGAAGTAHEVYSHIRNRH
>ID00813-ABP_both
KWKLFKKIPKFLHSAKKF
>ID00816-ABP_both
LFGFLIKLIPSLFGALSNIGRNRNQ
>ID00817-ABP_both
LLGDFFRKSKEKIGKEFKRIVQR
>ID00820-ABP_both
LRDLVCYCRTRGCKRRERMNGTCRKGHLMYTLCCR
>ID00822-ABP_both
LVRGCWTKSYPPKPCFVR
>ID00823-ABP_both
MLAKIKAMIKKFPNPYTLAAKLTTYEINWYKQQYGRYPWERPVA
>ID00824-ABP_both
MTPFWRGVSLRPIGASCRDDSECITRLCRKRRCSLSVAQE
>ID00825-ABP_both
NALSMPRNKCNRALMCFG
>ID00826-ABP_both
NALSSPRNKCDRASSCFG
>ID00828-ABP_both
NFLGTLVNLAKKIL
>ID00830-ABP_both
NPVSCVRNKGICVPIRCPGSMKQIGTCVGRAVKCCRKK
>ID00831-ABP_both
PDPGQPWQVKAGRPPCYSIPCRKHDECRVGSCSRCNNGLWGDRTCR
>ID00832-NO
QKLCERPSGTWSGVCGNNNACKNQCINLEKARHGSCNYVFPAHK
>ID00834-ABP_both
QLKVDLWGTRSGIQPEQHSSGKSDVRRWRSRY
>ID00835-ABP_both
QLPFVAGVACEMCQCVYCAASKKC
>ID00837-ABP_both
QWGRRCCGWGPGRRYCRRWC
>ID00839-ABP_both
RAGLQFPVGRLLRRLLRRLLR
>ID00840-ABP_both
RCVCRRGVCRCVCRRGVC
>ID00841-ABP_both
RIKRFWPVVIRTVVAGYNLYRAIKKK
>ID00842-ABP_both
RLGNFFRKVKEKIGGGLKKVGQKIKDFLGNLVPRTAS
>ID00845-ABP_both
RRWWRF
>ID00846-ABP_both
RSTEDIIKSISGGGFLNAMNA
>ID00849-ABP_both
SAVGRHGRRFGLRKHRKH
>ID00851-ABP_both
SFLDTLKNLAISAAKGAGQSVLSTLSCKLSETC
>ID00854-ABP_both

SFLSTFKELAINAAKNAGQSLLHTLSCKLDKTC
>ID00855-ABP_both
SIGAKILGGVKTFFKGALKELASTYLQ
>ID00856-ABP_both
SIGTAVKKAVPIAKKVGKVAIPIAKAVLSVVGQLVG
>ID00857-ABP_both
SIITMTKEAKLPQLWKQIACRLYNTC
>ID00858-ABP_both
SIITMTKEAKLPQSWKQIACRLYNTC
>ID00859-ABP_both
SIRDKIKTIAIDLAKSAGTGVLKTLICKLDKSC
>ID00865-ABP_both
SMISVLKNLGKVGLGFVACKVNKQC
>ID00867-ABP_both
SSGWVCTLTIECGTVICAC
>ID00868-ABP_both
SSLLEKGLDGAKKAVGGLGKLGKDAVEDLESVGKGAVHDVKDVLDSV
>ID00869-ABP_both
SVLGTVKDLLIGAGKSAAQSVLTALSCKLSNSC
>ID00870-ABP_both
SVLGTVKDLLIGAGKSAAQSVLTANSCKLSNSC
>ID00871-ABP_both
SVMGTVKDLLIGAGKSAAQSVLKALSCKLSKDC
>ID00872-ABP_both
SVMGTVKDLLIGAGKSAAQSVLKSLSCKISNDC
>ID00875-ABP_both
TESYFVFSVGM
>ID00876-ABP_both
TPVVNPPFLQQT
>ID00877-ABP_pos
TTHSGKYYGNGVYCTKNKCTVDWAKATTCIAGMSIGGFLGGAIPGKC
>ID00878-ABP_both
VFIDILDKVENAIHNAAQVGIGFAKPFEKLINPK
>ID00881-ABP_both
VNWKKILGKIIKVAK
>ID00882-ABP_both
VNWKKVLGKIIKVAK
>ID00886-ABP_both
WLGSALKIGAKLLPSVVGLFQKKKK
>ID00887-ABP_both
WLRRIGKGVKIIGGAALDHL
>ID00888-ABP_both
WRSLGRTLLRLSHALKPLARRSGW
>ID00891-ABP_both
YPSKPDNPGEDAPAEDMARYYSALRHYINLITRQRY
>ID00892-NO
YQCGQGG
>ID00893-ABP_both
YVPLPNVPQPGRRPFPTFPGQGPFNPKIKWPQGY
>ID00894-ABP_both
YVPPVQKPHPNGPKFPTFP

>ID00895-ABP_both
AALKGCWTKSIPPKPCFGKR
>ID00897-ABP_both
ACDTATCVTHRLAGLLSRSGGVVKNNFVPTNVGSKAF
>ID00898-ABP_both
ACHAHCQSVGRRGGYCGNFRMTCYCY
>ID00899-ABP_both
ADRGWIKTLTKDCPNVISSICAGTIITACKNCA
>ID00902-ABP_both
AGECVQGRCPSGMCCSQFGYCGRGPKYCGR
>ID00904-ABP_pos
AISYGNGVYCNKEKCWVNKAENKQAITGIVIGGWASSLAGMGH
>ID00906-NO
AKITFTNNHPRTIWP
>ID00907-ABP_neg
ALPKKLKYLNLFNDGFNYMGVV
>ID00908-ABP_both
AMVGT
>ID00909-ABP_both
AMVSS
>ID00910-ABP_both
AMWKDVLKKIGTVALHAGKAALGAVADTISQ
>ID00911-ABP_both
ANDPQCLYGNVAAKF
>ID00912-NO
ANFEIVNNCPYTVWAAASPGGGRRLDRGQT
>ID00914-ABP_both
ATCDLASGFGVGSSLCAAHCIARRYRGGYCNSKAVCVCRN
>ID00915-ABP_pos
ATCDLLSGFGVGDSACAAHCIARGNRGGYCNSQKVCVCRN
>ID00916-NO
ATFNFINNCPFTVWAAAVPG
>ID00920-ABP_both
AVDLAKIANKVLSSLF
>ID00921-ABP_both
AVNIPFKVHFRCKAAFC
>ID00922-ABP_both
AWKLFDDGV
>ID00923-ABP_both
CIGNGGRCNENVGPPYCCSGFCLRQPNQGYGVCRNR
>ID00925-ABP_pos
CRQSCSFGPLTFVCDGNTK
>ID00926-ABP_both
CVISAGWNHKIRCKLTGNC
>ID00927-ABP_both
DDFLCAGGCL
>ID00928-ABP_both
DHYICAKKGGTCNFSPCPLFNRIEGTCYSGKAKCCIR
>ID00929-ABP_both
DNGEAGRAAR
>ID00931-ABP_both

DTLIGSCVWGATNYTSDCNAECKRRGYKGGHCGSFLNVNCWCE
>ID00932-ABP_both
ECRRLCYKQRCVTYCRGR
>ID00933-ABP_both
EFTNVSCTTSKECWSVCQRLHNTSRGKCMNKKCRCYS
>ID00938-ABP_both
FALGAVTKLLPSLLCMITRKC
>ID00941-ABP_both
FFGSLLSLGSKLLPSVFKLFQRKKE
>ID00942-ABP_both
FFGSVLKLIPKIL
>ID00943-ABP_both
FFGTALKIAANVLPTAICKILKKC
>ID00944-ABP_both
FFPIIAGMAAKVICAITKKC
>ID00946-ABP_both
FFPIVGKLLFGLL
>ID00947-ABP_both
FFPIVGKLLFGLSGLL
>ID00948-ABP_both
FFPIVGKLLSGLL
>ID00949-ABP_both
FFPIVGKLLSGLSGLL
>ID00950-ABP_both
FFPLALLCKVFKKC
>ID00953-ABP_both
FFPMLAGVAARVVPKVICLITKKC
>ID00954-ABP_both
FFPTIAGLTKLFCAITKKC
>ID00955-ABP_both
FIGPVLKIAAGILPTAICKIFKKC
>ID00956-ABP_both
FIITGLVRGLTKLF
>ID00957-ABP_both
FILPLIASFLSKFL
>ID00958-ABP_both
FKCRRWQWRMKKLGA
>ID00959-ABP_both
FKTWKRPPFQTSCWGIIKE
>ID00960-ABP_both
FKVQNQHGQVVKIFHH
>ID00961-ABP_both
FLGAIAAALPHVINAVTNAL
>ID00963-ABP_both
FLGPIIKIATGILPTAICKFLKKC
>ID00964-ABP_both
FLGSLLGLVGKVVPTLFCKISKKC
>ID00965-ABP_both
FLGVVFKLASKVFPAVFGKV
>ID00966-ABP_both
FLPIAGKLLSGLSGLL

>ID00969-ABP_both
FLPILGNLLSGLL
>ID00971-ABP_both
FLPKLFAKITKKNMAHIR
>ID00972-ABP_both
FLPLIASLAANFVPKIFCKITKKC
>ID00974-ABP_both
FLPLLAGVVANFLPQIICKIARKC
>ID00975-ABP_both
FLPLLASLFSGLF
>ID00978-ABP_both
FLPLVRGAAKLIPSVVCAISKRC
>ID00980-ABP_both
FLPVLTGLTPSIVPKLVCLLTKKC
>ID00981-ABP_pos
FLSAITSLLGKLL
>ID00982-ABP_both
FLSLIPHAINAVSALANHG
>ID00983-ABP_both
FMPILSCSRFKRC
>ID00984-ABP_both
FQWQRNMRKVR
>ID00985-ABP_both
FVGAIAAALPHVISAIKNAL
>ID00986-ABP_both
GAARKSIRLHRLYTWKATIYTR
>ID00987-ABP_both
GAFGNFLKGVAKKAGLKILSIAQCKLFGTC
>ID00988-ABP_both
GAFGNFLKGVAKKAGLKILSIAQCKLSGTC
>ID00989-ABP_neg
GALRGCWTKSYPPKPCK
>ID00993-ABP_both
GFGMALKLLKKVL
>ID00996-ABP_both
GFGSLLGKALRLGANVL
>ID00997-ABP_both
GFMDTAKNVAKNMAGNLLDNLKCKITKAC
>ID00998-ABP_both
GFRDVLKGAAKAFVKTVAGHIANI
>ID00999-NO
GGTIFDCGETCFLGTCYTPGCSCGNYGFCYGTN
>ID01000-ABP_both
GGYYCPFFQDKCHRHCRSFGRKAGYCGGFLKKTCICV
>ID01001-ABP_both
GGYYCPFRQDKCHRHCRSFGRKAGYCGGFLKKTCICV
>ID01002-ABP_both
GICRCICGRGICRCICGR
>ID01003-ABP_both
GIFALIKTAAKFVGKNLLKQAGKAGLEHLACKANNQC
>ID01004-ABP_both

GIFGKILGVGKKVLCGLSGVC
>ID01005-ABP_both
GIFSKFAGKGIKNLLVKGVKNIGKEVGMDVIRTGIDIAGCKIKGEC
>ID01006-ABP_both
GIFSLIKGAAKLITKTVAKEAGKTGLELMACKVTNQC
>ID01007-ABP_both
GIFSLIKTAAKFVGKNLLKQAGKAGVEHLACKANNQC
>ID01009-ABP_both
GIFSTVFKAGKGIVCGLTGLC
>ID01010-ABP_both
GIGAAILSAGKSALKGLAKGLAEHF
>ID01012-ABP_both
GIGGKPVQTAFVDNDGIYD
>ID01013-ABP_both
GIGKFLHAAKKFAKAFVAEIMNS
>ID01014-ABP_both
GIGKFLKKAKKGIGAVLKVLTTGL
>ID01015-ABP_both
GIGSALAKAAKLVAGIV
>ID01016-ABP_both
GIGSLLAKAAKLGANLL
>ID01017-ABP_both
GIGTKILGGVKTALKGALKELASTYVN
>ID01018-ABP_both
GIHDILKYGKPS
>ID01019-ABP_both
GIKCRFCCGCCTPGICGVCCRF
>ID01020-ABP_both
GILDTFKNMALNAAKSAGVSVLNALSCKLSKTC
>ID01021-ABP_both
GILDTIKNAAKTVAVGLLEKIKCKMTGC
>ID01022-ABP_both
GILGTVFKAGKGIVCGLTGLC
>ID01024-ABP_both
GIMDSVKNVAKNIAGQLLDKLKCKITGC
>ID01025-ABP_both
GIMDTVKNAAKDLAGQLDKLKCRITGC
>ID01026-ABP_both
GIMDTVKNAAKDLAGQLLDKLKCRITGC
>ID01027-NO
GIPCAESCVWIPCTVTALIGCGCSNKVCYN
>ID01028-NO
GIPCAESCVYIPCTVTALLGCSCSNRVCYN
>ID01029-ABP_both
GIPCGESCVFIPCITAAIGCSCKSKVCYRN
>ID01033-ABP_neg
GKIPVKAIKKGGQIIGKALRGINIASTAHDIISQFKPKKKKNH
>ID01034-ABP_both
GKIPVKAIKQAGKVIGKGLRAINIAGTTHDVVSFFRPKKKKH
>ID01035-ABP_both
GKLNLFLSRLEILKLFVGAL

>ID01037-ABP_both
GKWGWIYITILFADVGGFKSSRHPEERRVQERRFKRITRGPD
>ID01040-ABP_both
GLFGKSSVWGRKYYVDLAGCAKA
>ID01042-ABP_both
GLFPKINKKKAKTGVFNIIKTVGKEAGMDLIRTGIDTIGCKIKGEC
>ID01043-ABP_both
GLFSKFAGKGIVNFLIEGVE
>ID01044-ABP_both
GLFSKFVGKGIKNFLIKGVKHIGKEVGMDVIRVGIDVAGCKIKGVC
>ID01045-ABP_both
GLFTLIKCAYQLIAPTVACN
>ID01047-ABP_both
GLFTLIKGAYKLDAPTVACN
>ID01048-ABP_both
GLGSFFKNAIKIAGKVGSTIGKVADAIGNKE
>ID01050-ABP_both
GLGSILGKILNVAGKVGKTIGKVADAVGNKE
>ID01051-ABP_both
GLGSLVGNALRIGAKLL
>ID01053-ABP_both
GLKDIFKAGLGSLVKGIAAHVAN
>ID01054-ABP_both
GLKEIFKAGLGSLVKGIAAHVAN
>ID01055-ABP_both
GLKKLLGKLLKKLGKLLLK
>ID01056-ABP_both
GLLGLLGSVVSHVVPAIVGHF
>ID01057-ABP_both
GLLGVLGSVAKHVLPHVVPVIAEHL
>ID01058-NO
GLLPCAESCVYIPCLTTVIGCSCKSKVCYKN
>ID01060-ABP_both
GLLSAVKGVLKGAGKNVAGSLMDKLKCKLFGGC
>ID01061-ABP_both
GLLSGILGAGKHIICGLSGLC
>ID01062-ABP_both
GLLSGILGAGKHIVCGLSGLC
>ID01063-ABP_both
GLLSGILGAGKHIVCGLTGCAKA
>ID01065-ABP_both
GLLSGVLGVGKKIVCGLSGLC
>ID01066-ABP_both
GLLSKFGRLARKLARVIPKV
>ID01067-ABP_both
GLLSSLSSVAKHVLPHVVPVIAEHL
>ID01068-ABP_both
GLLSVLGSVAQHVLPHVVPVIAEHL
>ID01069-ABP_both
GLMSLFRGVLKTAGKHIFKNVGGSLLDQAKCKITGEC
>ID01070-ABP_both

GLMSTLKDFGKTAAKEIAQSLLSTASCKLAKTC
>ID01071-NO
GLPICGETCVGGTCNTPGCSCSWPVCTRN
>ID01073-ABP_both
GLPVCGETCTLGTCYTQGCTCSWPICKRN
>ID01074-ABP_both
GLRSKIWLWVLLMIWQESNKFKKM
>ID01076-ABP_both
GLVRKGGEKFGEKLRKIGQKIKEFFQKLALEIEQ
>ID01077-ABP_both
GLVSSIGRALGGLLADVVKSKEQPA
>ID01078-ABP_both
GLWEKIKEKASELVSGIVEGVK
>ID01081-ABP_both
GLWQKIKSAAGDLASGIVEAIKS
>ID01082-ABP_both
GLWQKIKSAAGDLASGIVEGIKS
>ID01083-ABP_pos
GMSGYIQGIPDFLKGYLHGISAANKHKKGRL
>ID01084-ABP_both
GMWSKIKNAGKAAKAAAKAAGKAALGAVSEAM
>ID01086-ABP_both
GPVGLLSSPGSLPPVGGAP
>ID01087-ABP_both
GRCVCRKQLLCSYRERRIGDCKIRGVRFPFCCPR
>ID01088-ABP_both
GRKSDCFRKNGFCAFLKCPYLTLISGKCSRFHLCCKRIW
>ID01089-ABP_both
GSCSCSGTISPYGLRTCRATKTKPSHPTTKETHPQTLPT
>ID01091-NO
GTFPCGESCVFIPCLTSAIGCSCKSKVCYKN
>ID01093-NO
GVGYKVVVTTTAAADDDDVV
>ID01094-ABP_both
GVITDALKGAAKTVAAELLRKAHCKLTNSC
>ID01096-ABP_both
GVVTDLLKTAGKLLGNLFGSLSG
>ID01097-ABP_both
GWKKWFNRAKKVGKTVGGLAVDHYL
>ID01099-ABP_both
GWLKKIGKKIERVGQHTRDATIQVIGVAQQAANVAATAR
>ID01102-ABP_both
GYGGHGGHGGHGGHGGHGGHGHGGGGHG
>ID01103-ABP_both
GYHGGHGGHGGGYNGGGGHGGHGGGYNGGGHHGGGGHG
>ID01104-ABP_both
HKTDSFVGLM
>ID01105-ABP_both
HNKQEGRDHDKSKGHFHRVVIHHKGGKAH
>ID01106-ABP_both
IFGAIWNGIKSLF

>ID01108-ABP_both
IFNSIYHRKCVVKNRCETVSGHKTCKDLTCCRAVIFRHERPEVCRPST
>ID01110-ABP_both
IKIPAVVKDTLKKVAKGVLSAVAGALTQ
>ID01111-ABP_both
IKIPPIVKDTLKKVAKGVLSTIAGALST
>ID01112-ABP_both
IKLSPETKDNLKKVLKGAIKGAIAVAKMV
>ID01113-ABP_both
ILENLLARSTNEDREGSIFDTGPIRRPKPRPRPRPEG
>ID01114-ABP_both
ILGKILEGIKSLF
>ID01115-ABP_both
ILGKILKGIKKLF
>ID01116-ABP_both
ILGKIWKGIKSLF
>ID01117-ABP_both
IPAMEPAARVKRSPGYGGCSPRWACGGYG
>ID01118-ABP_both
IPCGESCVWIPCISGMFGCSCKDKVCYS
>ID01120-ABP_pos
ITSISLCTPGCKTGALMGCNMKTATCNCSIHVSK
>ID01121-ABP_both
IWSAIWSGIKGLL
>ID01122-ABP_both
KFCEKPSGTWSGVCGNSGACKDQCIRLEGAKHGSCNYKLPAHRCICYYEC
>ID01125-NO
KQTENLADTY
>ID01126-ABP_both
KRRGSVTTRYQFLMIHLLRPKKLFA
>ID01131-ABP_both
KTCENLANTYRGPCFTTGSCDDHCKNKEHLRSGRCRDDFRCWCTRNC
>ID01132-ABP_both
KTKLTEEEKNRLNFLKKISQRYQKFALPQYLKTVYQHQK
>ID01133-ABP_both
KWKLFKKIGIGAVLKVLTTG
>ID01134-ABP_both
KWKSFIKKLTSAAKKVVTTAKPLISS
>ID01135-ABP_both
KYYGNGVSCNKKGCSVDWGKAIGIIGNNSAANLATGGAAGWKS
>ID01136-ABP_pos
KYYGNGVSCNKNGCTVDWSKAIGIIGNNAAANLTTGGAAGWNKG
>ID01137-ABP_pos
KYYGNGVTCGKHSCSVNWGQAFSCSVSHLANFGHGKC
>ID01139-ABP_both
LGAWLAGKVAGTVATYAWNRYV
>ID01140-ABP_both
LIGPVLGLVGSALGGLLKKI
>ID01142-ABP_both
LKIPGFVKDTLKKVAKGIFSAVAGAMTPS
>ID01144-ABP_both

LTCEIDRSLCLLHCRLKGYLRAYCSQQKVCRCVQ
>ID01147-ABP_pos
MNFLKNGIAKWMTGAELQAYKKKYGCLPWEKISC
>ID01149-ABP_both
NCIQQCVSKGAQGGYCTNEKCTCY
>ID01150-ABP_both
NFLDTLINLAKKFI
>ID01151-ABP_both
NILNTIINLAKKIL
>ID01152-ABP_pos
NIPQLTPTP
>ID01154-ABP_both
NWRKILGKIAKVAAGLLGSMLAGYQV
>ID01155-ABP_both
NWRKILGQIASVGAGLLGSLLAGYE
>ID01157-ABP_both
QAFQTFKPDWNKIRYDAMKMQTSLGQMKKRFNL
>ID01158-ABP_both
QGVRSYLSCWGNRGICLLNRCPGRMRQIGTCLAPRVKCCR
>ID01161-ABP_both
QQCGRQAGNRRCANNLCCSQYGYCGRTNEYCCTSQGCQSQCRRCG
>ID01163-ABP_both
QWGRRCCGWGPGRRYCVRWC
>ID01166-ABP_both
RECKTESNTFPGICITKPPCRKACISEKFTDGHCSKILRRCLCTKPC
>ID01169-ABP_both
RFRPPIRRPPIRPPFRPPFRPPVRPPIRPPFRPPFRPPIGPFP
>ID01170-ABP_both
RGGRLCYCRGWICFCVGR
>ID01171-ABP_both
RIFSKIGGKAIKNLILKGIKNIGKEVGMDVIRTGIDVAGCKIKGEC
>ID01172-ABP_both
RIGVLLARLPKLFSLFKLMGKKV
>ID01173-ABP_both
RKCNFLCKLKEKLRTVITSHIDKVLRPQG
>ID01174-ABP_both
RLGNFFRKAKKKIGRGLKKIGQKIKDFLGNLVPRTES
>ID01175-ABP_both
RPRPNYRPRPIYRP
>ID01176-ABP_both
RRCICTTRTCRFPYRRLGTCIFQNRVYTFCC
>ID01177-ABP_both
RRSRRGRGGGRRGGSGGRGGRGGGRSGAGSSIAGVGSRGGGGGRHYA
>ID01178-ABP_both
RVKRFWPLVPVAINTVAAGINLYKAIRRK
>ID01179-ABP_both
RWKIFKKIEKMGRNIRDGIVKAGPAIEVLGSAKAI
>ID01180-ABP_both
RWKIFKKIEKVGRNVRDGIIKAGPAVAVVGQAATVVK
>ID01182-ABP_both
SFHVFPPWMCKSLKKC

>ID01183-ABP_both
SFLTTFKDLAIKAAKSAGQSVLSTLSCKLSNTC
>ID01184-ABP_both
SFPFFPPGICKRLKRC
>ID01186-ABP_both
SILPTIVSFLSKFL
>ID01187-ABP_both
SIRDKIKTIAIDLAKSAGMGILKTLICKLDKSC
>ID01188-ABP_both
SIRDKIKTIAIDLAKSAGTGVLKTLICKLNKSC
>ID01189-ABP_both
SIWEGIKNAGKGFLVSILDKVRCKVAGGCNP
>ID01190-NO
SNDIYFNFQR
>ID01191-ABP_both
SPRVSRRYGRPFGGRPFVGGQFGGRPGCVCIRSPCPCANYG
>ID01193-ABP_both
SRWPSPGRPRPFPGRPKPIFRPRPCNCYAPPCPCDRW
>ID01194-ABP_both
SSMKLSFRARAYGFRGPGPQL
>ID01195-ABP_both
SWLSKTAKKLENSAKKRISEGIAIAIKGGSR
>ID01196-ABP_both
SWLSKTYKKLENSAKKRISEGIAIAIQGGPR
>ID01198-ABP_pos
TKYYGNGVYCNSKKCWVDWGQASGCIGQTVVGGWLGGAIPGKC
>ID01199-ABP_both
TSRCIFYRRKKCS
>ID01200-ABP_neg
TYMPVEEGEYIVNISYADQPKKNSPFTAKKQPGPKVDLSGVKAYGPG
>ID01201-ABP_both
VAIALKAAHYHTHKE
>ID01202-ABP_both
VDKPPYLPRPPPPRRIYNNR
>ID01204-ABP_neg
VGECVRGRCPSGMCCSQFGYCGKGPKYCG
>ID01209-ABP_both
VKLIQIRIWIQYVTVLQMFSMKTKQ
>ID01212-ABP_both
VTCYCRRTRCGFRERLSGACGYRGRIYRLCCR
>ID01214-ABP_both
WWRELLKKLAFTAAGHLGSVLAAKQSGW
>ID01217-ABP_both
YSRCQLQGFNCVVRSYGLPTIPCCRGLTCRSYFPGSTYGRCQRF
>ID01220-ABP_both
AALKGCWTKSIPPKPCSGKR
>ID01221-ABP_both
AANFGPSVFTPEVHETWQKFLNVVVAALGKQYH
>ID01222-ABP_both
AAPRGGKGFFCKLFKDC
>ID01224-ABP_both

ACYCRIGACVSGERLTGACGLNGRIYRLCCR
>ID01225-ABP_both
ADNKNPLEECFRETNYEEFLEIAR
>ID01226-ABP_both
ADTLACRQSHQSCSFVACRAPSVDIGTCRGGKLKCCKWAPSS
>ID01227-ABP_both
AEVAPAPAAAAPAKAPKKKAAAKPKKAGPS
>ID01228-ABP_both
AFKLLGRIIHHVGNFVYGFSHVF
>ID01229-ABP_both
AILTTLANWARKFL
>ID01231-ABP_both
ALLGDFFRKSKEKIGKEFKRIVQRIKDFLRNLVPRTES
>ID01233-ABP_both
ALWMTLLKKVLKAAAKAALNAVLVGANA
>ID01234-ABP_pos
APAGLVAKFGRPIVKKYYKQIMQFIGEGSAINKIIPWIARMWRT
>ID01237-ABP_both
ARLKKCFNKVTGYCRKKCKVGERYEIGCLSGKLCCAN
>ID01238-ABP_both
ATAWDFGPHGLLPIRPIRIRPLCGKDKS
>ID01239-ABP_both
ATCDLFSFRSKWVTPNHAACAAHCLLRGNRGGRCKGTICHCRK
>ID01240-ABP_neg
ATCDLLSGFGVGDSACAAHCIARGNRGGYCNSKKVCVCPI
>ID01243-ABP_neg
ATCDLLSMWNVNHSACAAHCLLLGKSGGRCNDDAVCVCRK
>ID01245-ABP_both
ATNIPFKVHFRCKAAFC
>ID01249-ABP_both
AWLDKLKSLGKVVGKVALGVAQNYLNPQQ
>ID01250-ABP_both
CAWYNISCRLGNKGAYCTLTVECMPSCN
>ID01251-ABP_both
CLGVGSCNNFAGCGYAIVCFW
>ID01253-ABP_pos
CSTNTFSLSDYWGNKGNWCTATHECMSWCK
>ID01256-ABP_both
DAEFRHDSGYEVHHQKLVFFAEDVGSNKGAIIGLMVGGVV
>ID01257-ABP_both
DAEFRHDSGYEVHHQKLVFFAEDVGSNKGAIIGLMVGGVVIA
>ID01258-ABP_both
DEKGPKWKR
>ID01260-NO
DKLIGSCVWGAVNYTSNCNAECKRRGYKGGHCGSFLNVNCWCET
>ID01261-NO
DKLIGSCVWGAVNYTSNCRAECKRRGYKGGHCGSFANVNCWCET
>ID01263-ABP_both
DLRFLYPRGKLPVPTPPPFNPKPIYIDMGNRY
>ID01265-ABP_both
DSMGAVKLAKLLIDKMKCEVTKAC

>ID01267-ABP_both
DWTCWSCLVCAACSVELLNLVTAATGASTAS
>ID01269-ABP_both
EKKCPGRCTLKCGKHERPTLPYNCGKYICCVPVKVK
>ID01270-ABP_both
FAEPLPSEEEGESYSKEVPEMEKRYGGFM
>ID01271-ABP_neg
FALALKALKKALKKLKKALKKAL
>ID01272-ABP_both
FALLGDFFRKSKEKIGKEFKRIVQRIKDFLRNLVPRTES
>ID01273-ABP_both
FDIMGLIKKVAGAL
>ID01274-ABP_both
FDITKLNIKKLTKATCKVISKGASMCKVLFDKKKQE
>ID01275-ABP_both
FFFLSRIF
>ID01276-ABP_both
FFHLHFHY
>ID01277-ABP_both
FFPIVGKLLS
>ID01278-ABP_both
FFRHLFRGAKAIFRGARQGWRAHKVVSRYRNRDVPETDNNQEEP
>ID01279-ABP_both
FFSASCVPGADKGQFPNLCRLCAGTGENKCA
>ID01281-ABP_both
FIFHIIKGLFHAGKMIHGLVTRRRH
>ID01282-ABP_both
FIGKLISAASGLLSHL
>ID01283-ABP_both
FLFSLIPHAIGGLISAFK
>ID01284-ABP_both
FLGALAKIISGIF
>ID01286-ABP_both
FLGALWNVAKSVF
>ID01287-ABP_both
FLGGILNTITGLL
>ID01289-ABP_both
FLKPLFNAALKLLP
>ID01290-ABP_both
FLLFPLMCKIQGKC
>ID01291-ABP_both
FLPFVGNLLKGLL
>ID01292-ABP_both
FLPGLIAGIAKML
>ID01293-ABP_both
FLPGVLRLVTKVGPAVVCAITRNC
>ID01294-ABP_pos
FLPIALKALGSIFPKIL
>ID01295-ABP_both
FLPIVAGLAANFLPKIVCKITKKC
>ID01296-ABP_both

FLPIVTNLLSGLL
>ID01298-ABP_both
FLPLIGKILGTIL
>ID01299-ABP_both
FLPLLAGLAANFLPKIFCKITKKC
>ID01300-ABP_both
FLPMLAGLAANFLPKIVCKITKKC
>ID01302-ABP_both
FLPVIAGVAAKFLPKIFCAITKKC
>ID01303-ABP_both
FLPVILPVIGKLLNGIL
>ID01304-ABP_both
FLPVLAGVLSRA
>ID01305-ABP_both
FLSHIAGFLSNLF
>ID01306-ABP_both
FLSLIPSLVGGSISAFK
>ID01307-ABP_both
FLSLLPSIVSGAVSLAKKL
>ID01308-ABP_both
FLSTIWNGIKSLL
>ID01309-ABP_both
FLSTLLNVASKVVPTLFCKITKKC
>ID01310-ABP_both
FLSTLWNAAKSIF
>ID01311-ABP_both
FLWGLIPGAISAVTSLIKK
>ID01312-ABP_both
FMGGLIKAATKALPAAFCAITKKC
>ID01314-ABP_both
FQWQRNMRKVRGPPVS
>ID01315-ABP_both
FRPALIVRTKGTRL
>ID01317-ABP_both
FTCAISCDIKVNGKPCKGSGEKKCSGGWSCKFNVCVKV
>ID01318-ABP_pos
FTCDVLGFEIAGTKLNSAACGAHCLALGRRGGYCNSKSVCVCR
>ID01320-ABP_both
FVDLKKIANIINSIFGK
>ID01321-ABP_both
FVGPVLKIAAGILPTAICKIYKKC
>ID01322-ABP_both
FVLPLVMCKILRKC
>ID01326-ABP_both
GCSRWIIGIHGQICRD
>ID01327-ABP_both
GEFLKCGESCVQGECYTPGCSCDWPICKKN
>ID01328-ABP_both
GETFDKLKEKLKTFYQKLVEKAEDLKGDLKAKLS
>ID01329-ABP_both
GFCRCICTRGFCRCICTR

>ID01330-ABP_both
GFGCPLNQGACHNHCRSIGRRGGYCAGIIKQTCTCYRK
>ID01333-ABP_both
GFKDWIKGAAKKLIKTVASSIANQ
>ID01335-ABP_both
GFLDTFKNLALNAAKSAGVSVLNSLSCKLFKTC
>ID01336-ABP_both
GFMDTAKNAAKNVAVTLLDNLKCKITKAC
>ID01339-ABP_both
GFSPNLPGKGLRIS
>ID01340-ABP_both
GFWGKLWEGVKNAI
>ID01341-ABP_both
GGLRSLGRKILRAWKKYG
>ID01342-ABP_both
GGLRSLGRKILRAWKKYGPQATPATRQ
>ID01344-ABP_both
GIFSALAAGVKLLGNTLFKMAGKAGAEHLACKATNQC
>ID01345-ABP_both
GIFTKINKKKAKTGVFNIIKTIGKEAGMDVIRAGIDTISCKIKGEC
>ID01347-ABP_both
GIGGALLSAGKAALKGLAKVLV
>ID01348-ABP_both
GIGGALLSAGKSALKGLAKGLAEHL
>ID01349-ABP_both
GIGGALLSVGKLALKGLANVLADKFAN
>ID01350-ABP_both
GIGGKILGGLKTALKGAAKELASTYLH
>ID01351-ABP_both
GIGGKILGGLRTALKGAAKELAATYLH
>ID01352-ABP_both
GIGGVLLGAGKATLKGLAKVLAEKYAN
>ID01353-ABP_both
GIGGVLLSAGKAALKGLTKVLAEKYAN
>ID01356-ABP_both
GIGTKFLGGVKTALKGALKELAFTYVN
>ID01357-ABP_both
GIGTKFLGGVKTALKGALKELASTYVN
>ID01358-ABP_both
GIGTKILGGVKAALKGALKELASTYVN
>ID01359-ABP_both
GIKDWIKGAAKKLIKTVASHIANQ
>ID01360-ABP_both
GILDTFKGVAKGVAKDLAVHMLENLKCKMTGC
>ID01361-ABP_both
GILDTLKEFGKTAAKGIAQSLLSTASCKLAKTC
>ID01362-ABP_both
GILGKLWEGFKSIV
>ID01363-ABP_both
GILSGILGAGKSLVCGLSGLC
>ID01364-ABP_both

GILSGILGMGKKLVCGLSGLC
>ID01366-ABP_both
GIMDSVKNAAKNLAGQLLDTIKCKITAC
>ID01367-ABP_both
GIMDTLKNLAKTAGKGALQSLVKMASCKLSGQC
>ID01368-ABP_both
GIMDTVKNVAKNLAGQLLDKLKCKITAC
>ID01370-ABP_both
GIPCGESCVWIPCLTSAIGCSCKSKVCYRN
>ID01372-ABP_both
GKQYFPKVGGRLSGKAPLAAKTHRRLKP
>ID01373-ABP_both
GKWMSLLKHILK
>ID01374-ABP_pos
GLFDIVKKVVGTLAGL
>ID01378-ABP_both
GLFKTLIKGAGKMLGHVAKQFLGSQGQPES
>ID01380-ABP_both
GLFNVFKGALKTAGKHVAGSLLNQLKCKVSGGC
>ID01381-ABP_both
GLFRRLRDSIRRGQQKILEKARRIGERIKDIFRG
>ID01382-ABP_both
GLFSAFKKVGKNVLKNVAGSLMDNLKCKVSGEC
>ID01383-ABP_both
GLFSILKGVGKIALKGLAKNMGKMGLDLVSCKISKEC
>ID01384-ABP_both
GLFTLIKGAVKMIGKTVAKEAGKTGLELMACKVTNQC
>ID01385-ABP_both
GLGSLLGKAFKFGLKTVGKMMGGAPREE
>ID01388-ABP_both
GLHKVMREVLGYERNSYKKFFLR
>ID01389-ABP_both
GLIGSIGKALGGLLVDVLKPKL
>ID01392-ABP_both
GLLDAIKDTAQNLFANVLDKIKCKFTKC
>ID01393-ABP_both
GLLDLAKHVIGIASKL
>ID01395-ABP_both
GLLGAMFKVASKVLPHVVPAITEHF
>ID01397-ABP_both
GLLRKGGEKIGEKLKKIGQKIKNFFQKLVPQPE
>ID01399-ABP_both
GLLSGILGAGKHIVCGLSGPCQSLNRKSSDVEYHLAKC
>ID01400-ABP_both
GLLSGVLGVGKKVDCGLSGLC
>ID01401-ABP_both
GLLSGVLGVGKKVLCGLSGLC
>ID01402-ABP_both
GLMDTIKGVAKNVAASLLEKLKCKVTGC
>ID01403-ABP_both
GLMDTVKNAAKNLAGQLLDRLKCKITGC

>ID01404-NO
GLPICGETCVGGSCNTPGCSCSWPVCTRN
>ID01406-ABP_both
GLPVCGETCAGGTCNTPGCSCSWPICTRN
>ID01409-ABP_both
GLPVCGETCFGGTCNTPGCTCDPWPVCTRN
>ID01412-ABP_both
GLRKRLRKFRNKIKEKLKKI
>ID01414-ABP_both
GLVTSLIKGAGKLLGGLFGSVTG
>ID01415-ABP_both
GLWDSIKNFGKTIALNVMDKIKCKIGGGCPP
>ID01420-ABP_both
GLWSKIKEAAKTAGKAAMGFVNEMV
>ID01421-ABP_both
GMWSKILGHLIR
>ID01422-ABP_both
GRFRRLRKKTRKRLKKIGKVLKWIPPIVGSIPLGCG
>ID01423-ABP_both
GRLKKLGKKIEGAGKRVFKAAEKALPVVAGVKALG
>ID01424-ABP_both
GSGVIPTISHECHMNSFQFVFTCCS
>ID01430-ABP_both
GSQLVYREWVGHSNVIKGPP
>ID01431-ABP_both
GSQLVYREWVGHSNVIKP
>ID01433-NO
GTCKAECPTWEGICINKAPCVKCCKAQPEKFTDGHCSKILRRCLCTKPC
>ID01435-ABP_both
GTRCGETCFVLPCWSAKFGCYCQKGFCYRN
>ID01437-NO
GVCDMADLA
>ID01438-ABP_both
GVLDILKGAGKDLLAHALSKISEKV
>ID01439-ABP_both
GVLDILTGAGKDLLAHALSKLSEKV
>ID01443-ABP_both
GWFKKAWRKVKNAGRRVLKGVGIHYGVGLI
>ID01445-ABP_pos
GWINEEKIQKKIDEP
>ID01447-ABP_both
GWKIGKKLEHHGQNIRDGLISAGPAVFAVGQAATIYAAAK
>ID01448-ABP_both
GWLKKIGKKIERVGQHTRDATIQGLGIAQQAANVAATARG
>ID01453-ABP_both
GWMSKIASGIGTFLSGMQQ
>ID01454-ABP_neg
HAEHKVKIGVEQKYGQFPQGTEVTYTCSGNYFLM
>ID01456-ABP_both
IASKFLCTPGCAKTGSFNSYCC
>ID01458-ABP_both

IFKAIWSGIKSLF
>ID01459-ABP_both
IIGAIAAALPHVINAIKNTF
>ID01461-ABP_both
IIIQYEGHKH
>ID01463-ABP_both
ILGAIIPLVSGLLSHL
>ID01464-ABP_both
ILGKIAEGIKSLF
>ID01465-ABP_both
ILGKIWKIKKLF
>ID01466-ABP_both
ILGPILGLVSNALGGLL
>ID01467-ABP_both
ILGPVLGLVGNALGGLIKKL
>ID01468-ABP_both
ILSAIWSGIKSLF
>ID01469-ABP_both
INLKAIAALAKKLLG
>ID01470-ABP_both
IPCGESCVWIPCITAIAGCSCKNKVCYT
>ID01471-ABP_both
IPWKLPATFRPVERPFSKPFCRKD
>ID01472-ABP_both
IRNSLTCRFNFGICLPKRCPGRMRQIGTCF
>ID01474-ABP_pos
KCKWWNISCDLGNNGHVCTLSHECQVSCN
>ID01475-ABP_both
KFHEKHHSHRGY
>ID01480-NO
KICRRRSAGFKGPCMSNKNCAQVCQQEGWGGGNCDGPFRRCKCIRQC
>ID01482-ABP_both
KNIGNSVSCLRNKGVCMPGKCAPKMKQIGTCGMPQVKCCKRK
>ID01483-ABP_both
KPFKKLEKVGRNIRDGIIKAGPAVAVIGQATSIARPTGK
>ID01484-ABP_both
KPWRFRRAIRRVRWRKVAPYIPFVVKTVGKK
>ID01485-ABP_both
KRFKKFFKKLKNSVKKRAKKFFKKPRVIGVSIPF
>ID01486-ABP_both
KRGPNCVGNFLGGLFAGAAAGVPLGPAGIVGGANLGMVGGALTCL
>ID01490-ABP_both
KSKEKIGKEFKRIVQRIKDFLRNLVPR
>ID01491-ABP_both
KSLRPRCWIKIKFRCKSLKF
>ID01492-ABP_both
KTCENLADTFRGPCFATSNC
>ID01494-ABP_both
KVFLGLK
>ID01495-ABP_both
KVNVNAIKKGGKAIGKGFKVISAASTAHDVYEHIKNRRH

>ID01497-ABP_both
KWKLFKKAVLKVLTT
>ID01499-ABP_pos
KYYGNGVHCGKKTCYVDWGQATASIGKIIVNGWTQHGPWAHR
>ID01500-ABP_pos
KYYGNGVHCTKSGCSVNWGEAASAGIHRLANGGNGFW
>ID01502-ABP_pos
KYYGNGVTCGKHSCSVDWGKATTCIINNGAMAWATGGHQGTHKC
>ID01504-ABP_both
LDVKKIICVACKIRPNPACKKICPK
>ID01505-ABP_both
LFCKRGTCHFGRCPSHLIKVGSCFGFRSCCKWPWDA
>ID01506-ABP_both
LFGFLIPLLPHIIGAIPQVIGAIR
>ID01507-ABP_both
LGGLIKIVPAMICAVTKKC
>ID01510-ABP_both
LLSLALAALPKLFCLIFKKC
>ID01511-ABP_both
LNWGAILKHIIK
>ID01514-ABP_both
LVQRGRFGRFLKKVRRFIPKVIIAAQIGSRFG
>ID01515-ABP_both
LVQRGRFGRFLRKIRRFRPKVTITIQGSARFG
>ID01518-ABP_pos
MGAIAKLVAKFGWPIVKKYYKQIMQFIGEGWAINKIIEWIKKHI
>ID01519-ABP_pos
MKTILRFVAGYDIASHKKKTGGYPWERGKA
>ID01520-ABP_both
MQFITDLIKKAVDFFKGLFGNK
>ID01524-ABP_neg
MRTGNAD
>ID01530-ABP_both
NRWWQGVVPTVSYECRMNSWQHVFTCC
>ID01532-ABP_both
PAQPFRFPKHPQGPQTRPPI
>ID01536-ABP_both
PIRNWWIRIWEWLNGIRKRLRQRSPFYVRGHLNVTSTPQP
>ID01537-ABP_both
PKRKSATKGDEPA
>ID01539-NO
PRGSPRTEYEAARR
>ID01543-ABP_neg
QLINSPVTCMSYGGSCQRSCNGGFRLGGHCGHPKIRCCRRK
>ID01546-ABP_both
QRPYTQPLIYYPPPPTPPRIYRA
>ID01547-ABP_both
QRVRNPQSCRWNMGVCIPFLCRVGMRQIGTCFGPRVPCCRR
>ID01549-ABP_both
RCVCRRGVCRCVCTRGFC
>ID01551-ABP_both

RCVCTRGFCRCICLLGIC
>ID01552-ABP_both
RCVCTRGFCRCVCRRGVC
>ID01553-ABP_both
RCVCTRGFCRCVCTRGFC
>ID01554-NO
RDWERREFERRQNELRREQEQRREELL
>ID01555-NO
RECQSQSHRYKGACVHDTNCASVCQTEGFSGGKCVGFRGRCFCTKAC
>ID01557-ABP_both
RFIYMKGFGKPRFGKR
>ID01558-ABP_both
RFRPPIRRPPIRPPFNPPFRPPVRPPFRPPFRPPFRPPIGPFP
>ID01563-ABP_both
RKKWFW
>ID01564-ABP_both
RLARIVVIRVAR
>ID01566-ABP_both
RLGDILQKAREKIEGGLKKLVQKIKDFFGKFAPRTES
>ID01567-ABP_both
RMCKTPSGKFKGYCVNNTNCKNVCRTEGFPTGSCDFHVAGRKCYCYKPCP
>ID01568-ABP_neg
RPDKPRPYLPRPRPPRPVR
>ID01570-ABP_both
RPRCWIKIKFRCKSLKF
>ID01571-ABP_both
RQKDKRPYSERKNQYTGPQFLYPPERIPP
>ID01573-ABP_both
RRTCHCRSRCLRRESNSGSCNINGRIFSLCCR
>ID01574-ABP_both
RRWVRRVRRWVRRVVRVVRRWVRR
>ID01575-ABP_both
RSNKGFNFMVDMIQALSK
>ID01577-NO
RTCQSQSHKFKGACFSDTNCDSVCRTENFPRGQCNQHHVERKCYCERDC
>ID01578-NO
RVCMKGSAGFKGLCMRDQNCAQVCLQEGWGGGNCDGVMRQCKCIRQC
>ID01579-ABP_both
RVIEVVQGACRAIRHIPRRIRQGLERIL
>ID01581-ABP_both
RVRRFWPLVPVAINTVAAGINLYKAIRRK
>ID01582-ABP_neg
RWKLFKKIEKVGRNVRDGLIKAGPAIAVIGQAKSL
>ID01583-ABP_both
RWKVFKKIEKMGRNIRDGIVKAGPAIEVLGSAKALGK
>ID01584-ABP_both
RWKVFKKIEKVGRNVRDGIIKAGPAIGVLGQAKALG
>ID01585-ABP_both
SALVGCWTKSWPPKPCFGR
>ID01586-ABP_both
SALVGCWTKSWPPKPCFGRG

>ID01588-ABP_pos
SCNCVCGVCCSCSP
>ID01591-ABP_both
SIGAKILGGVKTFFKGALKELAFTYLQ
>ID01592-ABP_both
SILDKIKNVALGVARGAGTGILKALLCKLDKSC
>ID01593-ABP_both
SILPTIVSFLTKFL
>ID01594-ABP_both
SILSGNFGVGKKIVCGLSGLC
>ID01600-ABP_both
SLGNFFRKARKKIGEEFKRIVQRIKDFLQHLIPRTEA
>ID01602-ABP_both
SLLDTFKNLAVNAAKSAGVSVLNALSCKISRTC
>ID01603-ABP_both
SLRGFLKGVGTALAGVGKVVADQFDKLLQAGQ
>ID01604-ABP_pos
SLSRFLSFLKIVYPPAF
>ID01605-ABP_both
SLWENFKNAGKQFILNILDKIRCRVAGGCRT
>ID01607-ABP_both
SPIHACRYQRGVCIPGPCRWPYYRVGSCGSGLKSCCVRNRWA
>ID01612-ABP_pos
TITLSTCAILSKPLGNNGYLCTVTKECMPSCN
>ID01614-ABP_both
TSYGNGVHCNKSKCWIDVSELETYKAGTVSNPKDILWSLKE
>ID01616-ABP_pos
TTPATTSSWTCITAGVTVSASLCPTTKCTSRC
>ID01617-ABP_both
TWLKKRRWKKAKPP
>ID01619-ABP_both
VIGSILGALASGLPTLISWIKNR
>ID01621-NO
VKSTGRADDDLAVKTKYLPP
>ID01623-NO
VRPYLVAF
>ID01628-ABP_both
VVKCSYRLGSPDSQCN
>ID01632-ABP_neg
YQEPVLGPVRGPFPI
>ID01633-ABP_neg
YQEPVLGPVRGPFPIIV
>ID01637-ABP_both
AAFRGCWTKNYSPKPCL
>ID01638-ABP_both
ACQFWSCNSSCISRGYRQGYCWGIQYKYCQCQ
>ID01641-ABP_neg
ADDKNPLEEFRETNYEVFLEIAKNGLKATSNPKRVVIVGAGMAGLSAAY
>ID01646-ABP_both
AGYLLGKINLKALAALAKKIL
>ID01648-ABP_both

AKRHHGYKRKFH
>ID01649-ABP_both
ALKAALLAILKIVRVIKK
>ID01651-ABP_pos
ANKCIIDCMKVKTTCGDECKGAGFKTGGCALPPDIMKCCHNC
>ID01652-ABP_both
APPPGLSAGV
>ID01653-ABP_both
APPPGYAMESDSFS
>ID01658-ABP_pos
ATYYGNGLYCNKQKCWVDWNKASREIGKIIVNGWVQHGPWAPR
>ID01660-ABP_both
AVLDFIKAAGKGLVTNIMEKVG
>ID01661-ABP_both
AVLDILKDVGKGLLSHFMEKV
>ID01663-ABP_both
CLAGRLDKQCTCRRSQPSRRSGHEVGRPSPHCGPSRQCGCHMD
>ID01665-NO
CPAIQRCCQQLRNIQPPCRCCQ
>ID01667-ABP_both
CSCRTSSCRFGERLSGACRLNGRIYRLCC
>ID01668-ABP_neg
CYSAAKYPGFQEFINRKYKSSRF
>ID01673-NO
DKLIGSCVWGAVNYTRNCNAECKRRGYKGGHCGSFANVNCWCET
>ID01674-NO
DKLIGSCVWGAVNYTSNCNAECKRRGYKGGHCGSFANINCWCET
>ID01675-NO
DKLIGSCVWGAVNYTSNCNAECKRRGYKGGHCGSFANVNCWCER
>ID01676-NO
DKLIGSCVWGAVNYTSNCNAECKRRGYKGGHCGSFANVNCWCQT
>ID01677-NO
DKLIGSCVWGAVNYTSNCNAECKRRGYKGGHCGSFINVNCWCET
>ID01678-NO
DKLIGSCVWGAVNYTSNCNAECKRRGYKGGHCGSFLNINCWCET
>ID01679-NO
DKLIGSCVWGAVNYTSRCNAECKRRGYKGGHCGSFANVNCWCET
>ID01683-ABP_both
DQYKCLQHGGFCLRSSCPSNTKLQGTCKPDKPNCCKS
>ID01686-ABP_both
DTHISEKIIDCNDIG
>ID01687-ABP_both
DTLACRQSHGSCSFVACRAPSVDIGTCRGGKLKCCKWAPSS
>ID01688-ABP_neg
DVQCGEGHFCHDQTCCRASQGGACCPYSQGVCCADQRHCCPVGF
>ID01690-ABP_neg
EFELDRICGYGTARCRKKCRSQEYRIGRCPNTYACCLRKWDESLLNRTKP
>ID01692-ABP_both
EGVRNFVTCRINRGFCVPIRCPGHRRQIGTCLGPQIKCCR
>ID01694-ABP_both
EGVRNHVTCRINRGFCVPIRCPGRTRQIGTCFGPRIKCCRSW

>ID01699-ABP_both
EPILGIITSLLKSL
>ID01703-NO
EQPGGDKVNLGYFTN
>ID01705-NO
ESGINLQGDATLANN
>ID01706-ABP_both
EVVRNPQSCRWNMGVCIPISCPGNMRQIGTCFGPRVPCCR
>ID01707-ABP_both
FASLLGKALKALAKQ
>ID01708-ABP_both
FCKSLPLPLSVK
>ID01710-NO
FFDEKCNKLKGTCKNNCGKNEELIALCQKSLKCCRTIQPCGSIID
>ID01712-ABP_pos
FFHHIFRGIVHVGKTIHKLVTGG
>ID01713-ABP_both
FFHHIFRGIVHVGRTIHKLVTGG
>ID01714-ABP_both
FFHHIFRGIVHVGRTIHRLVTGG
>ID01715-ABP_both
FFPIVGKLLSGLF
>ID01716-ABP_both
FIHHIIGGLFSAGKAIHRLIRRRRR
>ID01719-ABP_both
FLAGLIGGLAKML
>ID01720-ABP_both
FLGGLIKIVPAMICAVTKK
>ID01721-ABP_both
FLGSLIGAAIPAIKQLLGLKK
>ID01722-ABP_both
FLNALKNFAKTAGKRLKSLLN
>ID01723-ABP_both
FLPAALAGIGGILGKLF
>ID01724-ABP_both
FLPAVIRVAANVLPTVFCAISKKC
>ID01726-ABP_both
FLPGLLAGLL
>ID01728-ABP_both
FLPLVGKILSGLI
>ID01729-ABP_both
FLPMLAGLAANFLPKLFCKITKKC
>ID01732-ABP_both
FLSLIPHIATGIAALAKHL
>ID01733-ABP_both
FLSTALKVAANVVPTLFCKITKKC
>ID01734-ABP_both
FLSTLLNVASNVVPTLICKITKKC
>ID01735-ABP_both
FPPPGESAVDMSFFYALSNP
>ID01736-ABP_neg

FQWQRNIRKVR
>ID01739-ABP_both
GDACGETCFTGICFTAGCSCNPWPTCTRN
>ID01741-ABP_both
GEYCGESCYLIPCFTPGCYCVSRQCVNKN
>ID01744-ABP_both
GFGCPFNENECHAHCLSIGRKFGFCAGPLRATCTCGKQ
>ID01745-ABP_both
GFGCPGDQYECNRHCRSIGCRAGYCDAVTLWLRCTCTGCSGKK
>ID01746-ABP_both
GFGCPLNQGACHNHCRSIRRRGGYCSGIIKQTCTCY
>ID01747-ABP_both
GFGCPNDYPCHRHCKSIPGRAGGYCGGAHRLRCTCYR
>ID01749-ABP_both
GFKDWIKGAAKKLIKTVAANIANQ
>ID01750-ABP_both
GFLGPLLKLGLKGVAKVLPHLIPSRQQ
>ID01751-ABP_both
GFMDTAKNVAKNMAVTLLDNLKCKITKAC
>ID01753-ABP_both
GFWGKLWEGVKSAI
>ID01754-ABP_both
GFWGSLWEGVKSVV
>ID01756-ABP_both
GGKCTVDWGGQGGGRRLPSPLFCCYKPTRICYLNQETCETETCP
>ID01758-ABP_both
GGLKKLGKKLEGAGKRVFKASEKALPVVVGIKAIGK
>ID01759-ABP_both
GGLKKLGKKLEGAGKRVFNAAEKALPVVAGAKAL
>ID01764-ABP_both
GICRCLCRRGVCRCICVL
>ID01765-ABP_both
GIFGKILGAGKKVLCGLSGLC
>ID01766-ABP_both
GIFGKILGVGKKVLCGLSGMC
>ID01767-ABP_both
GIFGKILGVGKKVLCGLSGWC
>ID01768-ABP_both
GIGGALLSAGKSALKGLAKGLAEHF
>ID01770-ABP_both
GIGGALLSVGKSALKGLTKGLAEHF
>ID01771-ABP_both
GIGKFLHSAGKFGKAFIGEIMKS
>ID01772-ABP_both
GIGRKFLGGVKTTFRCGVKDFASKHLY
>ID01776-NO
GIPCGESCVFIPCISSVIGCSCSSKVCYRN
>ID01778-NO
GIPCGESCVWIPCLTSAVGCSCKSKVCYRN
>ID01779-NO
GIPCGESCVYIPCLTSAIGCSCKSKVCYRN

>ID01781-ABP_neg
GIRNTVCFMQRGHCRLFMCRSGERKGDICSDPWNRCCVSSSIKNR
>ID01782-ABP_both
GKREKCLRRNGFCAFLKCPTLSVISGTCSRFQVCC
>ID01788-ABP_both
GLLCYCRKGHCKRGERVRGTCGIRFLYCCPRR
>ID01789-ABP_both
GLLDALSGILGL
>ID01790-ABP_both
GLLDFAKHVIGIASKL
>ID01791-ABP_both
GLLDTFKNMALNAAKSAGVSVLNALSCKLSKTC
>ID01794-ABP_both
GLLGTLGNLLNGLGL
>ID01795-ABP_both
GLLSGILGAGKHIVCGLSGLK
>ID01796-ABP_both
GLLSGILGAGKHIVCGLSGLR
>ID01797-ABP_both
GLLSVLKGVLKTTGKHIFKNVGGSLLDQAKCKISGQC
>ID01798-ABP_both
GLMDTVKNVAKNLAGHMLDKLKCKITGC
>ID01799-ABP_both
GLMSVTKGVLKTAGKHIFKNVGGSLLDQAKCKISGQC
>ID01800-ABP_both
GLPVCGETCFGGTCNTPGCACDPWPVCTRD
>ID01809-ABP_both
GMASKAGSVLGKITKIALGAL
>ID01812-ABP_both
GRKSDCFRKSGFCAFLKCPSLTLISGKCSRFYLCCKRIW
>ID01815-ABP_both
GSKKPVPIIYCNRRGKCQRM
>ID01817-NO
GTLPCGESCVWIPCISAVVGCSCKSKVCYKN
>ID01818-ABP_both
GTLPCGESCVWIPCISSVVGCSCKSKVCYKD
>ID01819-NO
GTPCGESCVWIPCISSAVGCSCKNKVCYKN
>ID01820-ABP_neg
GTPGFQTPDARVISRFGFN
>ID01821-ABP_both
GVCRCLCRRGVCRCLCRR
>ID01822-ABP_both
GVIDAAKKVVNVLKNLF
>ID01825-ABP_neg
GVLDIFKDAAKQILAHAAEKQI
>ID01826-ABP_both
GVLDILKGAAKDLAGHVATKVINKI
>ID01827-NO
GVPICGETCVGGTCNTPGCSCSWPVCTRN
>ID01828-NO

GVPVCGETCFGGTCNTPGCSCDPWPVCSRN
>ID01829-ABP_both
GWASKIGQTLGKMAKVGLQELIQPK
>ID01830-ABP_both
GWFKKAWRKVKHAGRRVLDTAKGVGRHYLNNWLNRYRG
>ID01833-ABP_neg
GWLKKIGKKIERVGQHTRDATIQTIGVAQQAANVAATLK
>ID01836-ABP_pos
IAPIIVAGLGYLVKDAWDHSDQIISGFKKGWNGGRRK
>ID01838-ABP_both
IDWLKLGKMVIDAL
>ID01839-ABP_both
IFGAIWNGIKS
>ID01840-ABP_neg
IFHHIFKGIVHVGKTIHRLVTG
>ID01841-ABP_both
IIGLVSKGTCVLVKTVCKKVLKQG
>ID01842-ABP_both
IIKVPLKKFKSMREVMRDHGIKAPVVDPATKY
>ID01846-ABP_both
ILKKWPWWPWRRK
>ID01847-ABP_pos
ILPILSLIGGLLGK
>ID01852-ABP_both
INWKALLDAAKKVL
>ID01854-ABP_both
ITPATPFTPAIITEITAAVIA
>ID01855-ABP_both
ITSVSWCTPGCTSEGGGSGCSHCC
>ID01859-ABP_both
KCWNLRGSCREKCIKNEKLYIFCTSGKLCCLKPKFQPNMLQR
>ID01862-ABP_both
KGLKKLLKLLKKLLKL
>ID01863-NO
KICEALSGNFKGLCLSSRDCGNVCRREGFTDGSCIGFRLQCFCTKPCA
>ID01866-ABP_both
KIKIPWGKVKDFLVGGMKAV
>ID01868-ABP_both
KKKKPLFGLFFGLF
>ID01870-ABP_both
KKLFKKILKYL
>ID01872-ABP_neg
KKWKKFIKKIGIGAVLTTPGAKK
>ID01873-ABP_both
KLAKLAKKLAKLAK
>ID01875-ABP_pos
KNYGNGVYCNKHKCSVDWATFSANIANNSVAMAGLTGGNAGNK
>ID01876-ABP_both
KPYCSCKWRCGIGEEEKGICHKFPIVTYVCCRRP
>ID01877-ABP_both
KQEGRDHDKSKGHFHMIVIHHKGGQAHHG

>ID01880-ABP_both
KRFKKFFKKVKKSVKKRLKKIFKKPMVIGVTIPF
>ID01881-ABP_both
KRGFGKKLRKRLKKFRNSIKKRLKNFNVVIPIPLPG
>ID01891-ABP_both
KVCRQRSAGFKGPCVSDKNCAQVCLQEGWGGGNCDGPFRRCKCIRQC
>ID01892-ABP_both
KVHGSLARAGK
>ID01897-ABP_both
KWKKFIKKIGIGAVLKVLTTGLPALKLTKK
>ID01898-ABP_both
KWKLFKKGAVLKVLT
>ID01899-ABP_both
KWKLFKKGIGAVLKV
>ID01900-ABP_both
KWKLFKKIGIGAVLKVLT
>ID01901-ABP_both
KWKLFKKLKVLTTGL
>ID01902-ABP_both
KWKSFIKKLTTAVKKVLTTGLPALIS
>ID01903-ABP_both
KWKSFLKTFKSAKKTVLHTALKAISS
>ID01908-ABP_both
LIKIVPAMICAVTKKC
>ID01909-ABP_both
LKLLKKL
>ID01910-ABP_both
LKLLKKLLKLLKKLGGGK
>ID01912-ABP_both
LKLLKKLLKLLKKLGK
>ID01913-ABP_pos
LLGDFFRKSKEKIGKEFKRIVQRIKDFLR
>ID01921-ABP_both
LRDLVCYCRKRGCKRRERMNGTCRKGHLMYTLCCR
>ID01922-ABP_both
LRDLVCYCRSRGCKGRERMNGTCRKGHLLYTLCCR
>ID01926-ABP_both
LTCDILGSTPACAAHCIARGYRGGWCDGQSVCNCRR
>ID01930-ABP_both
MAADIISTIGDLVKLIINTVKKFQK
>ID01935-ABP_both
MHDFWVLWVLLEYIYNSACSVLSATSSVSSRVLNRSLQVKVVKITN
>ID01951-ABP_pos
NLCERASLTWTGNCGNTGHCDTQCRNWESAKHGACHKRGNWKCFCYFDC
>ID01954-ABP_both
PDPAKTAPKKGSKKAVTKA
>ID01955-NO
PGLGFY
>ID01956-NO
PGMGIYLPM
>ID01959-NO

QGIGVGDNDGKRGKR
>ID01962-NO
QKIQEIDLQTYLQPQ
>ID01968-ABP_both
QPFIPRPIDTCRLRNGICFPGICRRPYYWIGTCNNGIGSCCARGWRS
>ID01969-ABP_both
QPFSLERW
>ID01970-NO
QSERFEQQMQGQDFSHDERFLSQAA
>ID01971-ABP_both
QSHLSLCRWCCNCCRSNKGC
>ID01972-ABP_both
QVVRNPQSCRWNMGVCIPISCPGNMRQIGTCFGPRVPCCRRW
>ID01974-ABP_both
RCICTTRTCRFPYRRLGTCLFQNRVYTFCC
>ID01975-ABP_both
RCRFCCRCCPRMRGCGICCRF
>ID01976-ABP_pos
RCTCTTIISSSSTF
>ID01981-ABP_both
RGLRRLGRKIAHGVKKYGPTVLRIIRIA
>ID01984-NO
RILSILRHQNLLKELQDLALQGAK
>ID01985-ABP_both
RKFHEKHHSHRGYR
>ID01987-ABP_both
RQIKIWFQNRRMKWKK
>ID01993-ABP_both
RRTCRCRFGRCFRRESYSGSCNINGRIFSLCCR
>ID01994-ABP_pos
RRTCRCRFGRCFRRESYSGSCNINGRISSLCCR
>ID01995-ABP_both
RRWCFRVCYRGRFCYRKCR
>ID01997-NO
RTCENLADKYRGPCFSGCDTHCTTKENAVSGRCRDDFRCWCTKRC
>ID01998-ABP_both
RTCMIKKEGWGKCLIDTTCAHSCKNRGYIGGNCKGMTRTCYCLVNC
>ID02005-ABP_both
RYHMQCGYRGTFCTPGKCPYGNAYLGLCRPKYSCCRWL
>ID02006-ABP_both
SALVGCGTKSYPPKPCFGR
>ID02008-ABP_both
SALVGCWTKSYPPNPCFGRG
>ID02009-ABP_pos
SCTTCVCTCSCCTT
>ID02013-NO
SIPCGESCVWIPCTITALAGCKCKSKVCYN
>ID02014-ABP_both
SLGSFLKGVGTTLASVGKVVSDQFGKLLQAGQ
>ID02015-ABP_both
SLWETIKNAGKGFIQNILDKIR

>ID02016-ABP_both
SNMIEGVFAKGFKKASHLFKGIG
>ID02017-ABP_both
SPPNQPSIMTFDYAKTNK
>ID02022-ABP_pos
SVSCLRNKGVCMPGKCAPKMKQIGTCGMPQVKCCKRK
>ID02025-NO
TLRGDERILSILRHQNLLKE
>ID02026-NO
TLRGDERILSILRHQNLLKELQDLALQGAK
>ID02027-ABP_both
TNYGNGVGVPDAIMAGIIKLIFIFNIRQGYNFGKKAT
>ID02028-ABP_both
TPFKISIHL
>ID02029-ABP_both
TPFKLSLHL
>ID02033-ABP_both
VAKCTEESGGKYFVFCCYKPTRICYMNEQKCESTCIGK
>ID02039-ABP_both
VKVGINGFGRIGRLVTRAAFHGKKVEVVAIND
>ID02046-ABP_pos
WKSESVCTPGCVTGVLQTCFLQTITCNCHISK
>ID02049-ABP_neg
WYVKKCLNDVGICKKKCKPEEMHVKNGWAMCGKGRDCCVPAD
>ID02051-NO
YKRGGGGWGGGGGWKGGGGGGGGWKGGGGGGKGGGG
>ID02052-ABP_both
YSKSLPLSVLNP
>ID02055-ABP_both
AALRGCWTKSIPPKPCSGKR
>ID02057-ABP_both
ACIKNGGRCVASGGPPYCCSNYCLQIAGQSYGVCKKH
>ID02059-NO
ADPTFGFTPLGLSEKANLQIMKAYD
>ID02065-ABP_both
AKAWGIPPHVIPQIVPVRIRPLCGNV
>ID02070-ABP_both
APPGARPPPGPPPPGPPPPGP
>ID02071-ABP_both
AREASKSLIGTASCTCRRAWICRWGERHSGKCIDQKGSTYRLCCRR
>ID02072-ABP_pos
ASFPWSCPSLSGVCRKVCLPTELFFGPLGCGKGFLCGVSHFL
>ID02073-ABP_both
ATCDLLSGFGVGDSACAAHCIARRNRGGYCNAKKVCVCRN
>ID02080-ABP_pos
CLGIGSCNDFAGCGYAIVCFW
>ID02082-ABP_both
CLRIGMRGRELMGGIGKTM
>ID02083-ABP_both
CVHWMTNTARTACIAP
>ID02087-ABP_both

DDDDDD
>ID02095-ABP_both
DLVCYCRARGCKGRERMNGTCRKGHLLYMLCCR
>ID02097-ABP_both
DRCTKRYGRCKRDCLESEKQIDICSLPRKICCTEKLYEEDDMF
>ID02098-ABP_both
DSHAKRHHGYKRKFHEKHHSHRGYR
>ID02100-NO
DSHEKRHHGYRRKFHEKHHSHREFPFYGDYGSNYLYDN
>ID02101-ABP_pos
DSIRDVSPTFNKIRRWFDGLFK
>ID02105-ABP_both
EGVRNHVTCRIYGGFCVPIRCPGRTRQIGTCFGRPVKCCRRW
>ID02109-ABP_both
ENREVPPGFTALIKTLRKCKII
>ID02110-NO
EQCREEEDDR
>ID02111-ABP_both
ERPPGFSPFR
>ID02112-ABP_both
ERVRNPQSCRWNMGVCIPFLCRVGMRQIGTCFGPRVPCCRR
>ID02113-ABP_both
FAEPLPSEEEGESYSKEPPEMEKRYGGFM
>ID02114-ABP_both
FFHHIFRGIVHVGKTIHRLVTGG
>ID02115-ABP_both
FFLLFLQGAAGNSVLCRIRGGRCHVGSCHFPERHIGRCSGFQACCIRTWG
>ID02116-ABP_both
FFPIVGKRLYGLL
>ID02117-ABP_both
FFPVIGRILNGIL
>ID02118-ABP_both
FFSMIPKIATGIASLVKNL
>ID02121-ABP_both
FIHHIIGGLFSVGKHIHSLIHGH
>ID02122-ABP_both
FIPGLRRLFATVVPTVVCAINKLPPG
>ID02123-ABP_both
FKCRRWQWRMKKLG
>ID02124-ABP_both
FLGAIAGVAAKFLPKVFCFITKKC
>ID02125-ABP_both
FLGGLLASLLGKI
>ID02126-ABP_both
FLGLIFHGLVHAGKLIHGLIHRNRG
>ID02127-ABP_both
FLPAVIRVAANVLPTAFCAISKKC
>ID02129-ABP_both
FLPFLIPALTSLISSL
>ID02130-ABP_pos
FLPFLKSILGKIL

>ID02131-ABP_both
FLPIIASVAAKLIPSIVCRITKKC
>ID02132-ABP_both
FLPLFLPKIICVITKKC
>ID02134-ABP_both
FLPLIAGVAANFLPKIFCLISKKC
>ID02135-ABP_both
FLPLIAGVAASILPKIFCFITKKC
>ID02137-ABP_both
FLPLIASVAANLVPKIFCKITKKC
>ID02138-ABP_both
FLPLIGKILGTILGK
>ID02139-ABP_both
FLPMLAGLAANFLPELFCKITKKC
>ID02140-ABP_both
FLPMLAGLAANLLPKLFCKITKKC
>ID02141-ABP_both
FLPVIASVAAKVLPKVFCFITKKC
>ID02142-ABP_both
FLPVILPVIGKLLNGILGK
>ID02143-ABP_both
FLSLIPHAINAVGVHAKHF
>ID02146-ABP_both
FLSLIPKIAGGIASLVKNL
>ID02148-ABP_pos
FNRGGYNFGKSVRHVVDAIGSVAGILKSIR
>ID02150-ABP_both
FQWQRNPRKVR
>ID02151-ABP_both
FRWWHR
>ID02155-ABP_both
FVDLKKIANIINSIF
>ID02158-ABP_both
FWGKLLKLGMHGIGLLHQHLG
>ID02161-ABP_both
GFGCPFDQGACHRHCQSIGRRGGYCAGFIKQTCTCYHN
>ID02163-ABP_both
GFGCPFNQYECHAHCSGVPGYKGGYCKGLFKQTCNCY
>ID02164-ABP_both
GFGCPGDAYQCSEHCRALGGGRTGGYCAGPWYLGHPTCTCSF
>ID02165-ABP_both
GFGCPNDYSCSNHCRDSIGCRGGYCKYQLICTCYGCKKRRSIQE
>ID02167-ABP_both
GFGCPRDQYKCNSHCQSIGCRAGYCDAVTLWLRCTCTDCNGKK
>ID02170-ABP_both
GFLDKLKKGASDFANALVNSIKGT
>ID02172-ABP_both
GFMDTAKNVAKNVAVTLIDNLKCKITKAC
>ID02173-ABP_both
GFMDTAKNVAKNVAVTLLDKLKCKITGGC
>ID02176-ABP_both

GFWDSVKEGLKNAAVTILNKIKCKISECPPA
>ID02177-ABP_both
GFWSSALEGLKKFAKGGLEALTNPK
>ID02178-ABP_both
GFWTTAAEGLKKFAKAGLASILNPK
>ID02180-ABP_both
GGSVPCGESCVFIPCITSLAGCSCKNKVCYYD
>ID02184-ABP_both
GIFSKFAGKGIKDLIIKGVKGIAKEAGMDVIRTGIDIAGCKIKGEC
>ID02185-ABP_both
GIFSKISGKAIKNLFIKGAENVGKHVGIDVVRTGIDVVGCKIKGEC
>ID02186-ABP_both
GIFSKISGKAIKNLFIKGAKNVGKEVGIDVVRTGMDVVGCKIKGEC
>ID02189-ABP_both
GIFTLFKGAAKLLGKTLAKEAGKTGLELMACKVTNQC
>ID02190-ABP_neg
GIGGALLSAAKVGLKGLAKGLAEHFAN
>ID02201-ABP_neg
GIWSSIKNLASKAWNSDIGQSLRNKAAGAINKFVADKIGVTPSQAAS
>ID02205-ABP_neg
GLFDIVKKLVSDF
>ID02206-ABP_both
GLFDIVKKVVGTIAGL
>ID02207-ABP_neg
GLFDKLKSLVSDF
>ID02209-ABP_both
GLFSKFAGKGIKDLIFKGVKHIGKEVGMDVIRVGIDVAGCKIKGVC
>ID02210-ABP_both
GLFSKFAGKGIKNFLIKGVKHIGKEVGMDVIRVGIDVAGCKIKGVC
>ID02211-ABP_both
GLFSKFAGKGIKNFLNKGVKHIGKE
>ID02212-ABP_both
GLFSKFAGKGIKNMIIKGIKGIGKEVGMDVIRTGIDVAGCKIKGEC
>ID02214-ABP_both
GLFTKFAGKGIKDLIFKGVKHIGKEVGMDVIRVGIDVAGCKIKGVC
>ID02215-ABP_both
GLISGILGVGKKLVCGLSGLC
>ID02217-ABP_both
GLISGLLGVGKMLVCGLSGLC
>ID02220-ABP_both
GLLDTFKNLAINAAESAGVSVLNSLSCKLSKTC
>ID02231-ABP_both
GLVSSIGKALGGLLVDVVKSKGQPA
>ID02239-ABP_both
GLWSTIKNVGKEAAIAAGKAVLGSLGEQ
>ID02241-ABP_both
GMASKAGSVLGKLAKVAIGAL
>ID02243-ABP_pos
GNGVVLTLTHECNLATWTKKLKCC
>ID02245-ABP_both
GSAQPYKQLHKVVNWDPYG

>ID02249-ABP_both
GVLDTFKDVAIGVAKGAGTGVLKALLCKLDKSC
>ID02251-ABP_both
GVWTTILGGLKKFAKGGLEALTNPK
>ID02252-ABP_both
GWGSIFKHIFKAGKFIHGAIQAHNDG
>ID02253-ABP_both
GWKSVFRKAKKVGKTVGGLALDHYLG
>ID02254-ABP_both
GWLKKIGKKIERVGQHTRGLGIAQIAANVAATAR
>ID02256-ABP_both
IDWKKVDWKKVSKKTCKVMLKACKFLG
>ID02257-ABP_pos
IGGALGNALNGLGTWANMMNGGGFVNQWQVYANKGKINQYRPY
>ID02262-ABP_both
IIGPVLGLVGKPLESLLE
>ID02263-ABP_both
ILGPVIKTIGGVIGGLLKNL
>ID02265-ABP_both
ILPILAPLIGGLL
>ID02266-ABP_both
ILRWPWWPWRRK
>ID02268-ABP_both
ISDYSIAMDKIRQQDFVNWLLAQKGKKSDWKHNITQ
>ID02270-ABP_pos
ITSFIGCTPGCGKTGSFNSFCC
>ID02271-ABP_both
ITSKSLCTPGCKTGILMTCPLKTATCGCHFG
>ID02274-ABP_both
KAYSMPRCKGGFRAVMCWL
>ID02275-ABP_both
KAYSTPRCKGLFRALMCWL
>ID02278-ABP_both
KFFRKLKKSVKKRAK
>ID02280-ABP_both
KKKSFIKLLTSAKVSVLTTAKPLISS
>ID02281-ABP_both
KKVVFKVKFK
>ID02282-ABP_neg
KKWWKFIKKAVNSGTTGLQTLAS
>ID02287-ABP_both
KRFGRLAKSFLRMRILLPRRKILLAS
>ID02291-ABP_both
KRRHWFPLSFQEFLEQLRRFRDQLPFP
>ID02293-NO
KSCCPSTTARNIYNTCRLTGASRSVCASLSGCKIISGSTCDSGWNH
>ID02294-ABP_pos
KSWSLCTPGCARTGSFNSYCC
>ID02295-ABP_pos
KSYGNGVQCNKKKCWVDWGSAISTIGNNSAANWATGGAAGWKS
>ID02297-ABP_both

KWFRVYRGIYRRR
>ID02300-ABP_both
KWKLFKKISKFLHLAKKF
>ID02301-ABP_both
KWKSFIKKLTSAAKKVLTTGLPALIS
>ID02302-ABP_both
KWKSFIKKLTSVLKKVVTTAKPLISS
>ID02303-ABP_neg
KWKSFIKNLTKGGSKILTTGLPALIS
>ID02304-ABP_both
KWKSFLKTFKSAKKTVLHTLLKAISS
>ID02305-ABP_both
KWKSFLKTFKSAVKTVLHTALKAISS
>ID02306-ABP_both
KWKSFLKTFKSLKKTVLHTLLKAISS
>ID02309-ABP_both
LKKLLKKLKK
>ID02311-ABP_both
LKKLLKKLKKLLKK
>ID02313-ABP_both
LLGMIPLAISAISSLSKL
>ID02314-ABP_both
LLGMIPVAISAISALSKL
>ID02315-ABP_pos
LLGNFFRKSKQKIGKQFKRIVQRIKNFFRNLVPRTQS
>ID02323-ABP_both
LYENKPRRPYIL
>ID02324-ABP_pos
LYKLVKVVLNM
>ID02352-ABP_both
NEPVSCIRNGGICQYRCIGLRHKIGTCGSPFKCCK
>ID02354-ABP_both
NNEAQCEQAGGICSKDHCFHLHTRAFGHCQRGVPCCRTVYD
>ID02361-ABP_both
PAQPFRIKKRQGPFERP
>ID02362-ABP_both
PEEMNKYLTALRHYINLVTRQRY
>ID02364-NO
PNPKVFFDMTIGGQSAGRIVMEEYA
>ID02365-NO
PVSRQQCSQRIQGERFNQCRSQMQDGQLQSCCQELQNVEEQCQC
>ID02376-ABP_both
QVFTLIKGATQLIRKTLGEQ
>ID02378-ABP_both
RAGLKFPVGRVHRLLR
>ID02379-ABP_both
RFGRFLRKIRRFRPKVTITIQGSARFG
>ID02380-ABP_both
RFRPPIRRPPIRPPFYPPFRPPIRPPIFPPIRPPFRPPLRFP
>ID02382-ABP_both
RICRIIFLRVCR

>ID02383-ABP_both
RIWVIWRR
>ID02387-ABP_both
RLSRIVVIRVSR
>ID02392-ABP_both
RPWAGNGSVHRYTVLSPRLKTQ
>ID02396-NO
RTCESKSHRFKGPCVSTHNCANVCHNEGFGGGKCRGFRRRCYCTRHC
>ID02397-ABP_both
RTKRRIKLIKNGVKKVKDILKNNNIIILPGSNEK
>ID02399-NO
RVCRRRSAGFKGVCMSDHNCAQVCLQEGYGGGNCDGIMRQCKCIRQC
>ID02400-ABP_both
RWRWRWRW
>ID02401-ABP_both
RYCLSQSHRFKGLCMSSSNCANVCQTENFPGGECKADGATRKCFCKKIC
>ID02403-NO
SAIACGESCVYIPCFIPGCSCRNRVCYLN
>ID02404-ABP_both
SCTCRRAWICRWGERHSGKCIDQKGSTYRLCCRR
>ID02405-ABP_both
SDEKASPDKHHRFSLSRYAKLANRLANPKLLETFLSKWIGDRGNRSVK
>ID02406-ABP_both
SGYLPGKEYVYKYKGKVF
>ID02411-ABP_both
SLLGTVKDLLIGAGKSAAQSVLKGLSGKLSKDC
>ID02413-ABP_both
SNMIEGVFAKGFKKASH
>ID02414-ABP_pos
STIVCVSLRICNWSLRFCPSFKVRCPM
>ID02415-ABP_both
STNCFCYPCCSCSS
>ID02419-ABP_pos
TPGGIDFISGGPHVAQDVLNAIKNFFK
>ID02423-ABP_neg
VGRFRRLRKKTRKRLKKIGKVLKWIPPIVGSIPLGCG
>ID02426-ABP_both
VRNSQSCRRNKGICVPIRCPGSMRQIGTCLGAQVKCCRRK
>ID02428-ABP_pos
VTSKSLCTPGCITGVLMCLTQNSCVSCNSCIRC
>ID02429-NO
VYINKLTPPCGTMYYACEAV
>ID02430-ABP_neg
WKLLSKAQEKFGKNKSR
>ID02431-ABP_neg
WKVFKSFIKKASSFAQSVLD
>ID02441-ABP_pos
YSLQMGATAIKQVKKLFKKKGG
>ID02442-NO
YTSLIHSLIEESQNQQEKNEQELLELDKWASLWNWF
>ID02443-ABP_both

AALRGALRAVARVGKAILPHVAIANPYVRTPYVHNNP
>ID02444-NO
ADDRCERMCQRYHDRREKKQCMKGCRYG
>ID02452-ABP_both
AKRGGFWRKVGRKLGKGIRKIGKTIKSQLGKFRPRLQYRYQF
>ID02458-ABP_both
ALWKTLLKKVLKA
>ID02461-ABP_both
ALYLAIRRR
>ID02462-ABP_both
APAHRSSTFPKWVTKTERGRQPLRS
>ID02463-ABP_neg
APRKNVRW
>ID02464-ABP_pos
APVPFSCTRGCLTHLV
>ID02471-ABP_neg
ATCDLLSFRSKWVTPNHAGCAAHCLLRGNRGGHCKGTICHCRK
>ID02472-NO
ATFTIRNNCPYTIWAAAVPGGGRRLNSGGTWTINVAPGTA
>ID02473-NO
ATITFTNKCTRTVWPGG
>ID02489-NO
CPFVC
>ID02493-NO
DFLEENITALLEEAQIQQEKNMYELQKLNSWDVFG
>ID02494-ABP_both
DHYLCVKNEGICLYSSCPSYTKIEGTCYGGKAKCCK
>ID02498-ABP_neg
DPVTCLKSGAICHPVFCPRRYKQIGTCGLPGTKCCKKPP
>ID02502-ABP_both
ELDRICGYGTARCRKKCRSQEYRIGRCPNTYACCLRK
>ID02503-NO
ERKVDFLEENITALLEEAQIQQEKNMYELQKLNSW
>ID02505-NO
EWERKVDFLEENITALLEEAQIQQEKNMYELQKLN
>ID02506-ABP_pos
EYHLMNGANGYLTRVNGKTVYRVTKDPVSAVFGVISNCWGSAGAGFGPQH
>ID02507-NO
FAKKFAKKFKKFAKKFAKFAFAF
>ID02508-ABP_both
FAVWGCADYRGYCRAACFAFEYSLGPKGCTEGYVCCVPNTF
>ID02509-ABP_pos
FDIVKKVVGALGSL
>ID02510-ABP_pos
FDIVKKVVGTIAGL
>ID02512-ABP_both
FEDLPNFGHIQVKVFNHGEHIHH
>ID02513-ABP_both
FELDRICGYGTARCRKKCRSQEYRIGRCPNTYACCLRKWDESLLNRTKP
>ID02514-ABP_both
FFGHLYRGITSVVKHVHGLLSG

>ID02515-ABP_both
FFGKVLKLIRKIF
>ID02516-ABP_both
FFGRLKSVWSAVKHGWKAAKSR
>ID02518-ABP_both
FFPLLFGALSSMMPKLF
>ID02521-ABP_both
FIHHIIGGLFSVGKHIHGLIHGH
>ID02522-ABP_both
FIHHIIGWISHGVRAIHRAIHG
>ID02523-ABP_both
FKFGSFIKRMWRSKLAKKLRAKGKELLRDYANRVLSPEEEAAAPAPVPA
>ID02524-ABP_both
FKRLKKLFKKIWNWK
>ID02525-NO
FLEENITALLEEAQIQQEKNMYELQKLNSWDVFGN
>ID02526-ABP_both
FLFRVASKVFPALIGKFKKK
>ID02527-ABP_both
FLFSLIPKAIGGLISAFK
>ID02528-NO
FLGFLHHLF
>ID02529-NO
FLGFLKNLF
>ID02530-ABP_both
FLGGLFKLVPSVICAVTKKC
>ID02531-ABP_both
FLGPIIKMATGILPTAICKGLKKC
>ID02532-ABP_both
FLGWLFKWAKK
>ID02533-ABP_both
FLGWLFKWAWK
>ID02534-NO
FLHFLHHLF
>ID02535-ABP_both
FLHHIVGLIHHGLSLFGDRAD
>ID02537-ABP_both
FLPIIGKLLSG
>ID02538-ABP_both
FLPKLFAGIISKNF
>ID02540-ABP_both
FLSGILKLAFKIPSVLCAVLKNC
>ID02543-ABP_both
FLSTLLKVAFKVVPTLFCPITKKC
>ID02544-ABP_both
FLWWLFKWAWK
>ID02547-ABP_both
FRSGILKLASKIPSVLCAVLKNC
>ID02548-ABP_both
FSCDVLSFQSKWVSPNHSACAVRCLAQRRKGGKCKNGDCVCR
>ID02550-ABP_both

FWGAVWKILSKVLPHIPGTVKWLQEKV
>ID02551-ABP_both
FWPVVIRTVVAGYNLYRAIKKK
>ID02552-NO
GAPICGESCFTGKCYTVQCSCSWPVCTRN
>ID02553-ABP_neg
GCWSTVLGGLKKFAKGGLEAIVNPK
>ID02557-ABP_both
GFFDRIKALTKNVTLELLNTITCKLPVTPP
>ID02566-ABP_both
GFGCPFNQGACHRHCRSIGRRGGYCAGLFKQTCTCYSR
>ID02567-ABP_both
GFGCPLNQGACHNHCRSIRRRGGYCSGIIKQTCTCYRN
>ID02568-ABP_both
GFIFHIIKGLFHAGKMIHGLV
>ID02569-ABP_both
GFKRIVQRIKDFLRNLV
>ID02570-ABP_both
GFLGILFHGVHHGRKKALQMNSERRS
>ID02579-ABP_both
GIFSLFKAGAKFFGKHLLKQAGKAGAEHLACKATNQC
>ID02580-ABP_both
GIFSLIKGAAKVVAKGLGKEVGKFGLDLMACKVTNQC
>ID02581-NO
GIGAILKVLATGLPTLISWIKNKRKQ
>ID02582-ABP_both
GIGSILGVIAKGLPTLISWIKNR
>ID02584-ABP_both
GILSKLGKALKKAAKHAAKA
>ID02586-ABP_neg
GIMDTLKNLAKTAGKGALQSLLNHASCKLSGQC
>ID02587-ABP_both
GIPCAESCVWIPPCTITALMGCSCKNNVCYNN
>ID02589-ABP_both
GIPCGESCVFIPCTVTALLGCSCKDKVCYKN
>ID02596-ABP_both
GIWDTLKNVGKAVLGKVLENV
>ID02599-ABP_both
GKREKCLRRNGFCAFLKCPTLSVISGTCSRFQVCCKTLLG
>ID02600-ABP_both
GLFAVIKKVASVIKKL
>ID02601-ABP_both
GLFDVIAKVASVIKKL
>ID02602-ABP_both
GLFGILGSVAKHVLPHVIPVVAEHL
>ID02603-ABP_both
GLFGKSSVHGRKYYVDLAGCAKA
>ID02605-ABP_both
GLFSVLGSVAKHVVPRVVPVIAEHLG
>ID02606-ABP_pos
GLFVGLAKVAAHNNPAIAEHFQA

>ID02609-ABP_both
GLLDSVKEGLKKVAGQLLDTLKCKISGCTPA
>ID02610-ABP_both
GLLRRLRDFLKKIGEKFKKIGY
>ID02611-ABP_both
GLLSGTSVRGST
>ID02616-NO
GLPVCGETCFGGTCNTPGCSCSSWPICTRN
>ID02619-NO
GLPVCGETCTLGKCYTAGCSCSWPVCYRN
>ID02620-NO
GLPVCGETCVGGTCNTPGCTCSWPVCTRD
>ID02621-ABP_both
GLRKALRKFRNKIKEALKKI
>ID02622-ABP_both
GLRKRLRKARNKIKEKLKKI
>ID02624-ABP_both
GLRRLFADQLVGRRNI
>ID02637-ABP_both
GRADYNFGYGLGRGTRKFFNGIGRWVRKTF
>ID02638-ABP_both
GRKSDCFRKNGFCAFLKCPYLTLISGLCSFHLC
>ID02640-ABP_both
GRLRNLIEKAGQNIRGKIQGIGRRIKDILKNLQPRPQV
>ID02643-ABP_neg
GSPEFGWLKKIGKKIERVGQHTRDATIQTIGVAQQAANVAATLKG
>ID02648-NO
GTLPCESCVWIPCISSVVGCSCKSKVCYKN
>ID02651-NO
GTPCGESCVYIPCISGVIGCSCTDKVCYLN
>ID02652-ABP_both
GVGKFLHSAKKFGQALVSEIMKS
>ID02654-ABP_both
GVPCAESCVWIPCTVTALLGCSCKDKVCYLN
>ID02656-ABP_both
GWASKIGQALGKVAKVGLQQFIQPK
>ID02657-ABP_both
GWASKIGTQLGKMAKVGLKEFVQS
>ID02659-ABP_both
GWLKKIGKKIERVGQHTRDATIQTIGVAQQAANVAATLKG
>ID02662-ABP_neg
GWRLLLKKAEVKTVGKLALKHYL
>ID02666-ABP_both
HVINLEESFQEPEYENHLA
>ID02668-ABP_both
IGKKFKRIVQRIKDFLRNL
>ID02669-ABP_pos
IIGAIAAALPHVINAIKNTFG
>ID02671-ABP_both
ILAWKWAWWAWRR
>ID02672-ABP_both

ILGAILPLVSGLLSNKL
>ID02673-ABP_pos
ILGIITSLLKSLGKK
>ID02674-ABP_both
INLKAIAALARNY
>ID02675-ABP_both
INLKAIAALVKKLL
>ID02679-ABP_both
INWKKMAATALKMI
>ID02684-ABP_both
IWDAIFHGAKHFLHRLVNPGGKDAVKDVQQKQ
>ID02685-ABP_both
KAYSMPRCKYLFRAVLCWL
>ID02687-ABP_both
KILRGVCKKIMRTFLRRISKDILTGKK
>ID02689-ABP_both
KKLALALAKKWLALAKKLALALAKK
>ID02691-ABP_both
KKLALHALKKWLHALKKLAHLALKK
>ID02692-ABP_both
KKLALLALKKWLLALKKLALLALKK
>ID02693-ABP_both
KKLALLALKKWLPALKKLALLALKK
>ID02694-ABP_neg
KKWWKAKKFANSGPNALQTLAQ
>ID02695-ABP_neg
KKWWKAQKAVNSGPNALQTLAQ
>ID02698-ABP_both
KLLKQWPIGKLLKKLLKKLLK
>ID02699-ABP_both
KRFKKFFKKLKNSVKKRVKKFFRKPRVIGVTFPF
>ID02700-ABP_both
KRFRIRVRVIRK
>ID02705-NO
KSCCPNTTGRNIYNTCRFGGGSREVCASLSGCKIISASTCPSYPDK
>ID02709-ABP_both
KTKQQFLIKAQTQLFKVFGYTL
>ID02710-ABP_pos
KVAKQEKKKKKTGRAKRR
>ID02711-NO
KVCYRAIPCGESCVWIPCISAAIGCSCKN
>ID02713-ABP_both
KVVKQWPIGKVVKKVVKKVVK
>ID02714-ABP_neg
KWKSFLKTFKSLKKTVLHTALKAISS
>ID02716-ABP_both
KWKVFKKIEKMGRNIRNGIVKAGPAIAVLGEAKAILS
>ID02719-ABP_both
KYYCRVRGGRCAVLSCLPKEEQIGKCSTRGRKCCRRKK
>ID02725-ABP_both
LCAAHCLAILRR

>ID02726-ABP_both
LCAAHCLAIRRR
>ID02728-ABP_pos
LFCKRGTCHFGRCPSHLIKV
>ID02729-ABP_neg
LFKLLGKIIHHVGNFVHGFSHVF
>ID02730-ABP_both
LKLLKKLLKKLLKLL
>ID02731-ABP_both
LLGDFFRKAREKIGEEFKRIVQRIKDFLRNLVPRTES
>ID02732-ABP_neg
LLGDFFRKSKEK
>ID02733-ABP_both
LLIILRRRIRKQAHAHSK
>ID02735-ABP_both
LRDLVCYCRARGCKGRERMNGTCRKGHLLYMLCCR
>ID02736-ABP_both
LRLKSIVSYAKKVL
>ID02738-ABP_both
LRVRRTLQCSCRRVCRNTCSCIRLSRSTYAS
>ID02739-ABP_neg
LSPNLLKSLL
>ID02747-ABP_both
MASRAAGLAARLARLALRAL
>ID02748-ABP_both
MDSFQKIEKIGEGTYGVVYKAKDKVSGRLVALKKIRLENESEGVPSTA
>ID02797-ABP_pos
MTCGQVQGNLAQCIGFLQKGG
>ID02798-ABP_both
MVALLKSLERRRLMITISTMLQFGLFLIALIGLVIKLIELSNKK
>ID02806-ABP_both
NGVQPKY
>ID02808-ABP_both
NKGCAICSIGAACLVDGPIPDFEIAGATGLFGLWG
>ID02812-ABP_both
NQGRHFCGGALIHARFVMTAASCFQ
>ID02814-NO
NSMERVEELRKKLQD
>ID02819-ABP_both
PFWRIRIRR
>ID02823-ABP_both
PWNIFKEIERAVARTRDAVISAGPAVRTVAAATSVAS
>ID02826-NO
QEWERKVDFLEENITALLEEAQIQQEKNMYELQKL
>ID02827-ABP_both
QKKIRVRLSA
>ID02829-ABP_neg
QLPICGETCVLGGCYTPNCRCQYPICVR
>ID02830-ABP_pos
QNNICKTTSKHFKGLCFADSKCRKVCIQEDKFEDGHCSKLQRKCLCTKNC
>ID02849-ABP_both

RKGWFKAMKSIAKFIAKEKLKEHL
>ID02856-ABP_both
RRWCFRVCYKGFCRYKCR
>ID02858-NO
RTCESQSHKFKGPCASDHNCASVCQTERFSGGRCRGFRRRCFCTTHC
>ID02859-NO
RTCESQSHRFHGTCVRESNCASVCQTEGFIGGNCRAFRRRCFCTRNC
>ID02862-ABP_both
RVVRQWPIGRVVRRVVRRVVR
>ID02871-ABP_both
SIRDKGKTIAIDLAKSAGTGVLKTLMCKLDKSC
>ID02887-ABP_pos
STPVLASVAVSMELLPTASVLYSDVAGCFKYSAKHHC
>ID02888-NO
TFPKCAPTRPPGPKPCDINNFKSKFWHIWRA
>ID02889-ABP_pos
TGRAKRRMQYNRR
>ID02892-ABP_both
TLISWIKNKRKQRPRVSRRRRRRGGRRRR
>ID02893-ABP_pos
TNWKKIGKCYAGTLGSAVLGFGAMGPVGYWAGAGVGYASFC
>ID02895-ABP_both
TVVTNA
>ID02896-ABP_both
VAPIAKYLATALAKWALKQGFAKLKS
>ID02897-ABP_both
VCGETCVGGTCNTPGCTCSWPVCTRNGLP
>ID02900-ABP_both
VFHAYSARGNYYGNCPANWPSCRNNYKSAGGK
>ID02904-ABP_both
VKLKVYPLKVKLYP
>ID02906-ABP_both
VQLRIRVAVIRA
>ID02907-ABP_both
VQRWLIVWRIRK
>ID02908-ABP_both
VRLIVAVRIWRR
>ID02909-ABP_both
VRLRIRVAVIRK
>ID02910-ABP_both
VRLRIRVAVRRA
>ID02917-NO
WERKVDFLEENITALLEEAQIQQEKNMYELQKLNS
>ID02919-ABP_both
WLNALLKKGLNCAKGVLA
>ID02920-ABP_both
WPVVIRTVVAGYNLYRAIKKK
>ID02924-ABP_both
YCRVRGGRCAVLSCLPKEEQIGKCSTRGRKCCRRKK
>ID02931-ABP_pos
YYGNGVTCGKHSCSVDWGKATTCIINNGAMAWATG

>ID02936-ABP_both
AATKPKKAGAEAAPKKPAKKQTKKKPAKKAGGKKKPKRAGAKKAKK
>ID02939-NO
AFGCPFDQGTCHSHCRSIRRRGRRCASFAKRTCTCYQK
>ID02941-ABP_both
AGFRKRFNKLVKKVKHTIKETANVSKDVAIVAGSGVAVGAAMG
>ID02942-ABP_both
AHCLAIGRR
>ID02943-ABP_both
AIFIFIRWLLKLGHHGRAPP
>ID02944-NO
AIRDTNKAVQSVQSSIGNLIVAIKSVQDYVNKEIV
>ID02946-NO
AKQARSDIEKLKEAIRDTNKAVQSVQSSIGNLIVA
>ID02947-ABP_both
AKRLKKLAKKIWKWK
>ID02948-ABP_both
AKRLKKLFKKIWNWK
>ID02949-NO
ALDPIDISIELNKAKSDLEESKEWIRRSNQKLDSI
>ID02950-ABP_both
ALLLAIRRR
>ID02951-NO
ALLSTNKAVVSLSNGVSVLTSKVLDLKNYIDKQ
>ID02952-ABP_both
ALRLAIRRR
>ID02960-ABP_both
ALWLAIRRR
>ID02961-ABP_both
ANVGFVIQLQARLRGFLVRQKFA
>ID02963-ABP_both
APKGVQGPNG
>ID02966-NO
ARSDIEKLKEAIRDTNKAVQSVQSSIGNLIVAIKS
>ID02970-ABP_both
ATCDLLSPFKVGHAACAAHCIARGKRGGWCDKRAVCNCRK
>ID02971-ABP_pos
ATQSHQ
>ID02972-ABP_pos
ATYYGNGVYCNKQKCWVDWSRARSEIIDRGVKAYVNGFTKVLG
>ID02973-ABP_both
ATYYGNGVYCNKQKCWVDWSRARSEIIDRGVKAYVNGFTKVLGGIGGR
>ID02974-ABP_both
AVAGEKLWLLPHLLKMLLTPTP
>ID02975-NO
AVALVEAKQARSDIEKLKEAIRDTNKAVQSVQSSI
>ID02977-NO
AVSKVLHLEGEVNKIALLSTNKAVVSLSNGVSV
>ID02979-ABP_both
AWLLAIRRR
>ID02982-ABP_both

CVLIGQRCDNDRGPRCCSGQGNCVPLPFLGGVCAV
>ID02986-NO
DISIELNKAKSDLEESKEWIRRSNQKLDSIGNWHQ
>ID02987-NO
DLFQVIKEKLKELTGGVIEGIQGV
>ID02988-NO
DLGPPISLERLDVGTNLGNAIAKLEAKELLESSD
>ID02989-ABP_both
DLIWKLLSKAQEKFGKNKSR
>ID02991-NO
DPIDISIELNKAKSDLEESKEWIRRSNQKLDSIGN
>ID02996-NO
EAKQARSDIEKLKEAIRDTNKAVQSVQSSIGNLIV
>ID02997-NO
EDGLHPRLCSC
>ID02999-ABP_pos
EKYTEAPEYI
>ID03001-NO
ELNKAKSDLEESKEWIRRSNQKLDSIGNWHQSSTT
>ID03002-ABP_both
EPEPSYVGDCGSNGGSCVSSYCPYGNRLNYFCPLGRTCCRHAYV
>ID03003-NO
EQCGRQAGGKLCPNNLCCSQWGWCGSTDEYCSPDHNCQSNCKD
>ID03004-NO
EQCGRQAGGKLCPNNLCCSQYGWCGSSDDYCSPSKNCQSNCKGGG
>ID03005-ABP_both
EQEENVVKIQAFWKGYKQRKEYM
>ID03008-ABP_both
EVASFDKSKLK
>ID03009-NO
EVNKIALLSTNKAVVSLSNGVSVLTSKVLDLKN
>ID03013-ABP_both
FFHHIFRAIVHVAKTIHRLVTG
>ID03014-ABP_both
FFHHIFRPIVHVGKTIHRLVTG
>ID03015-ABP_both
FFKKFWGGVKAIFKGARKGWK
>ID03019-ABP_both
FFPFLLGALGSLLPKIF
>ID03021-ABP_both
FFSALLSGIKSLF
>ID03026-ABP_both
FIGALLRPALKLLAGK
>ID03027-NO
FIGMIPGLIGGLISAFK
>ID03029-ABP_both
FIPLVSGLFSRLL
>ID03030-ABP_both
FIQYLAPLIPHAVKAISDLI
>ID03032-ABP_both
FITGLIGGLMKAL

>ID03033-ABP_both
FITGLISGLMKAL
>ID03036-ABP_both
FKLFKKIPKFLHLAKKF
>ID03037-NO
FKLRAKIKVRLRAKIKL
>ID03038-ABP_neg
FKRIVQRIKDFLRNLV
>ID03039-ABP_both
FKRLKKLAKKIWNWK
>ID03041-ABP_both
FLFSLIPSVIAGLVSAIRN
>ID03042-ABP_both
FLGALFKVASKVLPSVKCAITKKC
>ID03043-ABP_both
FLGLLGSLL
>ID03045-ABP_both
FLPAVLKVAAHILPTAICAISRRC
>ID03048-ABP_both
FLPGLIKAAVGIGSTIFCKISRKC
>ID03050-ABP_both
FLPGLIKVAVGVGSTILCKITKKC
>ID03051-ABP_both
FLPILGKLLSGFL
>ID03055-ABP_both
FLPKTLRKFFCRIRGGRCAVLNCLGKEEQIGRCSNSGRKCCRKKK
>ID03056-ABP_both
FLPLFLPKIICEITKKC
>ID03057-ABP_both
FLPLKKLRFGLL
>ID03059-ABP_both
FLPLLFGALSTLLPKIF
>ID03060-ABP_both
FLPLLLSALPSFLCLVFKKC
>ID03061-ABP_both
FLPLLVGAISSILPKIF
>ID03065-ABP_both
FLPVLGKVIKLVGGLL
>ID03066-ABP_pos
FLSAITSILGKFF
>ID03077-ABP_both
FLSLIPHIVSGVAALAKHLG
>ID03080-ABP_both
FLSTIWNGIKGLL
>ID03082-ABP_both
FPFLLSLIPSAISAIKRL
>ID03083-ABP_both
FPFSLIPHAIGGLISAIK
>ID03086-NO
FRWCFRVCYKGRCRYKCR
>ID03087-ABP_both

FSLFFPYAALKWLRKLLKK
>ID03088-ABP_both
FSVTWRWWKWWKG
>ID03089-ABP_both
FTCNSYACKAHCILQGHKSGSCARINLCKCQR
>ID03091-ABP_both
FVPWFSKFLGRIL
>ID03092-ABP_both
FWGALAKGALKLIPSLVSSFTKKD
>ID03093-ABP_both
FWGFLGKLAMKAVPSLIGGNKSSSK
>ID03094-ABP_both
GAFGDLLKGVAKEAGLKLLNMAQCKLSGNC
>ID03097-ABP_pos
GDINGEFTTSPACVYSVMVVSKASSAKCAAGASAVSGAILSAIRC
>ID03099-ABP_both
GEKLKKIGQKIKNFFQKL
>ID03100-NO
GELDELVYLLDGPGYDPIHS
>ID03101-NO
GELGRLVYLLDGPGYDPIHCD
>ID03102-NO
GEVNKIALLSTNKAVVSLSNGVSVLTSKVLDLK
>ID03104-ABP_both
GFGCPEDEYECHNHCKNSVGCRGGYCDAGTLRQRCTCYGCNQKGRSIQE
>ID03105-ABP_both
GFGCPKSALSCSQQCRENNTHSGGYCNGPFNIVCSCY
>ID03109-ABP_both
GFGSVLGKALKFGANLL
>ID03114-ABP_both
GFKKLLKGAAKALVKTVLF
>ID03115-ABP_both
GFMDTAKNVAKNVAKNVAVTLLDKLRCKVTGGC
>ID03117-ABP_both
GFMSKVANFAKKFAKGGVNAIMNQK
>ID03120-ABP_both
GFRKRFNKLVKKVKHTIKETANVSKDVAIVAGSGVAVGAAMG
>ID03127-ABP_both
GIFSLFKAGAKFFGKNLLKEAGKAGAEHLACKAANQC
>ID03128-ABP_both
GIFSLIKTAAKFVGKNLLKQAGKAGLEHLACKANNQC
>ID03130-NO
GIGAILKVLSTGLPALISWIKRKRQE
>ID03138-ABP_both
GIKEFAHSLGKFGKAFVGGILNQ
>ID03142-ABP_both
GILDTLKNLAKTAGKGILKSLVNTASCKLSGQC
>ID03144-NO
GILGKIWEGVKSLI
>ID03150-NO
GIPCAESCVYIPCTITALLGCSCKNKVCYN

>ID03154-ABP_both
GKCNVLCQLKQKLRSIGSGSHIGSVVLPRG
>ID03155-ABP_both
GKFSGFAKILKSIAKFFKGVGKVRKQFKQASDLDKNQ
>ID03156-ABP_both
GKGLEVIKWKLKHVIQL
>ID03157-ABP_neg
GKVKVGVNGFGRIGRLVTRAAFNSGKVDIVA
>ID03159-ABP_pos
GLEESPGHPGQPGPPGPPGAPGP
>ID03160-ABP_both
GLFAVIKKVASVIGGL
>ID03162-ABP_both
GLFKVIKKVASVIGGL
>ID03164-ABP_both
GLFRALLRLLRSLWRLLLRA
>ID03165-ABP_both
GLFSKFAGKGIKDLIFKGVKHIGKEVGMDVIRTGIDVAGCKIKGEC
>ID03166-ABP_both
GLFSKFAGKGIKNFLIKGVKHIGKEVGMDVIRTGIDVAGCKIKGEC
>ID03169-ABP_both
GLFTKFAGKGIKDLIFKGVKHIGKEVGMDVIRTGIDVAGCKIKGEC
>ID03171-ABP_both
GLKDWWNKHKDKIIDVVKEMGKAGLQAA
>ID03172-ABP_both
GLKEVAHSAKKFAKGFISGLTGS
>ID03173-ABP_both
GLLDTFKNLALNAAESAGVSVLNSLSCKLSKTC
>ID03174-ABP_both
GLLKPLLKIAAKVGSNLL
>ID03175-ABP_both
GLLLDTVKGAAKNVAGILLNKLKCKMTGDC
>ID03178-ABP_both
GLLSNVAGLLKQFAKGGVNAVLNPK
>ID03179-ABP_both
GLLSVFKGVLKTAGKNVAKNVAGSLLDQLKCKISGGC
>ID03186-NO
GLPCGETCFTGKCYTPGCSCSYPICKKIN
>ID03187-ABP_both
GLPICGETCFKTKCYTKGCSCSYPVCKRN
>ID03189-NO
GLPTCGETCFGGTCNTPGCTCDPWPICTRD
>ID03192-ABP_both
GLRDKIKNVAIDVAKGAGTGVLKALLCQLDKSC
>ID03193-ABP_both
GLRKRLRKFRNKIKEKLKKIG
>ID03194-ABP_both
GLRKRLRKFRNKIKEKLKKIGQKIQGLLPKLAPRTDY
>ID03195-ABP_both
GLVGTLLGHIGKAILG
>ID03196-ABP_both

GLVGTLLGHIGKAILS
>ID03200-ABP_both
GLWDSIKIAGKKLFVNVLDKIRCKVAGGC
>ID03201-ABP_both
GLWDTIKQAGKKFFLNVLDKIRCKVAGGCRT
>ID03206-ABP_both
GLWNSIKIAGKKLFVNVLDKIRCKVAGGC
>ID03207-ABP_both
GLWNSIKIAGKKLFVNVLDKIRCKVAGGCKTSPDVEYHK
>ID03210-ABP_both
GLWNTIKEAGKKFALNLLDKIRCGIAGGCKG
>ID03215-ABP_both
GLWSTIKNVAAAAGKAALGAL
>ID03216-ABP_both
GMATKAGTAFGKAAKAIIGAAL
>ID03220-NO
GPPISLERLDVGTNLGNAIAKLEAKELLESSDQI
>ID03221-ABP_both
GRFKRFRKKFKKLFKKLSPVIPLLHL
>ID03222-ABP_both
GRFRRLGRKFKKLFKKYGP
>ID03223-ABP_both
GRFRRLRKKTRKRLKKIGKV
>ID03225-NO
GRKKRRQRRR
>ID03227-ABP_both
GRRKRQMEARFEPQNRNYRKRELDLEKLFANMPDY
>ID03228-ABP_both
GRRRRSVQWCAVSQPEATKCFQWQRNMRKVRGPPVSCIKRDSPIQCI
>ID03229-ABP_both
GSAIRCGESCLLGKCYTPGCTCDRPICKKN
>ID03231-ABP_both
GSKKPVPIIYCNRRAATGKCQRM
>ID03233-ABP_both
GSKKPVPIIYCNRRKCQRM
>ID03234-ABP_both
GSKKPVPIIYCNRRTKCQRM
>ID03235-ABP_both
GSVIGCGETCLRGRCYTPGCTCDHGICKKN
>ID03236-ABP_both
GTCSFSSALCVVHCRVRGYPDGYCSRKGICTCRR
>ID03237-NO
GTLPCGESCVWIPCISAAVGCSCKSKVCYKN
>ID03242-ABP_both
GVFGLLAKAALKGASKLIPHLLPSRQQ
>ID03246-ABP_both
GVIAAAKKVVNVLKNLF
>ID03247-ABP_both
GVLDTLKNVAIGVAKGAGTGVLKALLCQLDKSC
>ID03248-ABP_both
GVLSAFKNALPGIMKIIV

>ID03250-ABP_both
GVWDWIKKTAGKIWNSEPVKALKSQALNAAKNFVAEKIGATPS
>ID03251-ABP_both
GVWDWLKKTAKNVWNSDIVKQLKGKAINAAKNYVAEKIGATPS
>ID03254-ABP_both
GWASSIGSILGKFAKGGAQAFLQPK
>ID03255-ABP_both
GWGDTFGKVLKNFAKVAGVKAAK
>ID03256-ABP_both
GWGDTFLKTMAKIAKVGPKLLHS
>ID03259-ABP_both
GWLKKIGKKIERVGQHTRDASIQAIGIAQQAANVAATARG
>ID03260-ABP_both
GWLKKIGKKIERVGQHTRDATIQVLGVAQQAANVAATARG
>ID03261-ABP_both
GWLPTFGKILRKAMQLGPKLIQPI
>ID03262-ABP_both
GWLRDFGKRIERVGQHTRDATIQAIGVAQQAANVAATVRG
>ID03265-ABP_both
GYFCPYNGYCDHHCRKKLRWRGGYCGGRWKLTCICVRG
>ID03267-ABP_both
HCLAIGRR
>ID03268-ABP_both
HHHHRFGKIGHELHKGVKKVEKVTHDVNKVTSGVKKVASSIEKAKNV
>ID03269-ABP_both
HHHLFGHVGHEVERSLHKVGHKLEHACHEVHKTAKKVQK
>ID03270-NO
HRIDLGPPISLERLDVGTNLGNAIAKLEAKELLE
>ID03272-NO
IALLSTNKAVVSLSNGVSVLTSKVLDLKNYIDK
>ID03274-NO
IDISIELNKAKSDLEESKEWIRRSNQKLDSIGNWH
>ID03275-NO
IDLGPPISLERLDVGTNLGNAIAKLEAKELLESS
>ID03276-NO
IELNKAKSDLEESKEWIRRSNQKLDSIGNWHQSST
>ID03277-ABP_both
IGGYCSWLRL
>ID03278-ABP_both
IGKEFKRIVERIKRFLRELVRPLR
>ID03279-ABP_both
IGKKFKRIVKRIKKFLRKL
>ID03280-ABP_both
IGKKFKRIVKRIKKWLRKL
>ID03284-ABP_both
IGKLFKRIVKRILKFLRKL
>ID03285-ABP_both
IGKLFKRIVQRIKKFLRNL
>ID03286-ABP_both
IGKLFKRIVQRILKFLRNL
>ID03288-ABP_both

IKRFWPVVIRTVVAGYNLYRAIKKK
>ID03289-ABP_both
IKWKKLLRAAKRIL
>ID03293-ABP_both
ILKLFKRIVKRILKFLRKL
>ID03295-ABP_both
ILPIIGKILSTIFGK
>ID03296-ABP_both
ILPILSLIGGLL
>ID03297-ABP_both
ILPIRSLIKKLL
>ID03299-NO
IMFFEMQACWSHSGVCRDKSERNCKPMAWTYCENRNQKCCEY
>ID03300-ABP_both
INWKKIKSIIKAAMN
>ID03302-ABP_both
IRRIIRKIIHIIKK
>ID03303-NO
ISIELNKAKSDLEESKEWIRRSNQKLDSIGNWHQS
>ID03304-ABP_both
ISQSDAILSAIWSGIKSLF
>ID03307-ABP_both
ITIPPIVKDTLKKFFKGGIAGVMGKSQ
>ID03308-ABP_both
ITIPPIVKDTLKKFFKGGIAGVMGQ
>ID03309-ABP_both
ITIPPIVKDTLKKFIKGAISGVM
>ID03311-ABP_both
ITIPPIVKNTLKKFIKGAVSALMS
>ID03314-ABP_both
KEEQIGKSSTRGRKSSRRKK
>ID03316-ABP_neg
KFKSFIKKLTSKFLHSAKKF
>ID03317-ABP_pos
KGLGKLIGIDWLLGQAKDAVKQYKKDYKRWH
>ID03319-ABP_both
KIAHGVKKYGPTVLRIIRIAG
>ID03320-NO
KIALLSTNKAVVSLSNGVSVLTSKVLDLKNYID
>ID03322-ABP_both
KIFGAIWPLALGALKNLIK
>ID03324-ABP_both
KIKEKLKKIGQKIQG
>ID03326-ABP_both
KKCGFFCKLKNKLKSTGSRSNIAAGTHGGTFRV
>ID03327-ABP_both
KKCKFFCKVKKKIKSIGFQIPIVSIPFK
>ID03328-ABP_both
KKKKKKAAFAAWAAFAA
>ID03330-ABP_neg
KKLKLALAKLAPLWKALALKLKKA

>ID03331-ABP_neg
KKLKLALAKPALLWKALALKLKKA
>ID03334-ABP_both
KKWWKF
>ID03335-ABP_both
KLAGLAKKLAGLAKKLAGLAK
>ID03338-ABP_both
KLGFENFLVKALKTVMHVPTSPLL
>ID03339-NO
KLKEAIRDTNKAVQSVQSSIGNLIVAIKSVQDYVN
>ID03340-ABP_both
KLKLLLLL
>ID03342-ABP_both
KLKNFAKGVAQSLLNKASCKLSGQC
>ID03343-ABP_neg
KLKSLLKTLSKAKKKLLKTALKALSK
>ID03344-ABP_both
KLSPSLGPVSKGKLLAGQR
>ID03346-NO
KQARSDIEKLKEAIRDTNKAVQSVQSSIGNLIVAI
>ID03347-ABP_both
KQIMTQFFNFARSPAVKD
>ID03349-ABP_both
KRFWPVVIRTVVAGYNLYRAIKKK
>ID03350-ABP_both
KRLRKFRNKIKEKLKKIG
>ID03351-ABP_both
KRLRRVWRRWR
>ID03353-NO
KSCCRNTLARNCYNACRFTGGSQPTCGILCDCIHVTTTTCPSSHPS
>ID03355-ABP_both
KSSTRGRKSSRRKK
>ID03357-ABP_pos
KTVNYGNGLYCNQKKCWVNWSETATTIVNNSIMNGLTGGNAGWHSGGRA
>ID03360-NO
KVLHLEGEVNKIALLSTNKAVVSLSNGVSVLTS
>ID03361-ABP_both
KVTKSVKSIPVKI
>ID03362-ABP_both
KWFKKIPKFLHLAKKF
>ID03363-ABP_both
KWFKKIPKFLHLLKKF
>ID03364-ABP_neg
KWKFKKIGIGAVLKVLTTGLPALKLTLK
>ID03365-NO
KWKKALRALARHLK
>ID03366-ABP_both
KWKKFIKSLTKSAAKTVVKTAKKPLIV
>ID03367-ABP_both
KWKLFKKIEKVGQGIGAVLKVLTTGL
>ID03368-NO

KWKLFKKIEKVGQNIRDGIIKAGPAVAVVGQATQIAKG
>ID03369-ABP_both
KWKLFKKIEKVGQRVDAVISAGPAVATVAQAATALAK
>ID03370-ABP_neg
KWKLFKKIGIGAVLKVLTTGLPALKLTLK
>ID03371-ABP_both
KWKLFKKILKFLHLAKKF
>ID03375-ABP_both
KWKLFKKIPKFLHL
>ID03376-ABP_both
KWKLFKKIPKFLHLA
>ID03379-ABP_both
KWKLFKKIPLAKKF
>ID03380-ABP_both
KWKLFKKIPLHLAKKF
>ID03384-ABP_neg
KWKSFIKKLLSKFLHSAKKF
>ID03385-ABP_neg
KWKSFIKKLTDKFLHSAKKF
>ID03386-ABP_both
KWKSFIKKLTKKFLHSAKKF
>ID03387-ABP_neg
KWKSFIKKLTSKALHSAKKF
>ID03390-ABP_neg
KWKSFIKKLTSKFLHSAKKF
>ID03392-ABP_neg
KWKSFIKKLTSKFLHSANKF
>ID03393-ABP_neg
KWKSFIKKLTSKFLHSKKKF
>ID03394-ABP_neg
KWKSFIKKSTSKFLHSAKKF
>ID03395-ABP_neg
KWKSFINKLTSKFLHSAKKF
>ID03396-ABP_both
KWKSFLKTFKSAAKTVLHTALKAISS
>ID03397-ABP_neg
KWKSFLKTFKSAKKTVLHTALKLISS
>ID03399-ABP_neg
KWKSFLKTFKSLKKTVLHTALKLISS
>ID03400-ABP_neg
KWKSFLKTFSKAKKKALKTLLKAISK
>ID03401-ABP_neg
KWKSFLKTFSKAKKKKLKTLLKAISK
>ID03404-ABP_both
KWLRRVWRWWR
>ID03411-NO
LDPIDISIELNKAKSDLEESKEWIRRSNQKLDSIG
>ID03414-NO
LERLDVGTNLGNAIAKLEAKELLESSDQILRSMK
>ID03415-ABP_both
LFAKINGLKVGPLKIQIV

>ID03416-NO
LGPPISLERLDVGTNLGNAIAKLEAKELLESSDQ
>ID03417-ABP_both
LGRKIAHGVKKYGPTVLRII
>ID03418-NO
LHRIDLGPPISLERLDVGTNLGNAIAKLEAKELL
>ID03420-NO
LKEAIRDTNKAVQSVQSSIGNLIVAIKSVQDYVNK
>ID03423-ABP_both
LKLSPKTKDTLKKVLKGAIKGAIAIASMA
>ID03424-ABP_both
LLAAHLLAIGRR
>ID03427-ABP_both
LLRHVVKILEKYL
>ID03429-ABP_both
LNLKALLAVAKKIL
>ID03430-NO
LNNSVALDPIDISIELNKAKSDLEESKEWIRRSNQ
>ID03431-ABP_both
LPFFLLSLIPSAISAIKKI
>ID03433-ABP_both
LQDAALGWGRRCPRCPPCPRCSWCPRCPTCPGCNCNPK
>ID03435-ABP_pos
LQDAALGWSRRCPRCPPCPNCRRCPRCPTCPSCNCNPK
>ID03436-ABP_pos
LQDAAVGWGRRCPQCPRCPSCPSCPRCPRCPRCKCNPK
>ID03438-ABP_both
LRAAHRLAIGRR
>ID03439-ABP_both
LRGLLCYCRKGHCKRGERVRGTCGIRFLYCCPRR
>ID03440-ABP_both
LRKFRNKIKEKLKKIGQKI
>ID03441-ABP_both
LRKFRNKIKEKLKKIGQKIQG
>ID03442-ABP_both
LRPAILVRIK
>ID03443-ABP_both
LRRIIRKIIHIIKK
>ID03444-ABP_neg
LTCNIDRSFCLAHCLLRGYKRGFCTVKKICVCRH
>ID03447-ABP_pos
LVPLFLSKLICFITKKC
>ID03461-NO
MEHFRWG
>ID03468-ABP_both
MITISTMLQFGLFLIALIGLVIKLIELSNKK
>ID03539-ABP_both
NILSSIVNGINRALSFFG
>ID03543-NO
NNSVALDPIDISIELNKAKSDLEESKEWIRRSNQK
>ID03544-ABP_both

NPQSCRWNMGVCIPISCPGNMRQIGTCS
>ID03545-NO
NQGRHFCGGALIHARFVMTAASSFQ
>ID03546-NO
NQGRHFSGGALIHARFVMTAASCFQ
>ID03547-NO
NQGRHFSGGALIHARFVMTAASSFQ
>ID03548-NO
NSVALDPIDISIELNKAKSDLEESKEWIRRSNQKL
>ID03549-ABP_both
NVLSSVANGINRALSFFG
>ID03553-NO
PIDISIELNKAKSDLEESKEWIRRSNQKLDSIGNW
>ID03554-NO
PISLERLDVGTNLGNAIAKLEAKELLESSDQILR
>ID03555-ABP_both
PKLLETFLSKWIG
>ID03556-ABP_both
PPCRGIFCRRVGSSSAIARPGKTLSTFITV
>ID03557-NO
PPISLERLDVGTNLGNAIAKLEAKELLESSDQIL
>ID03558-ABP_both
PVVIRTVVAGYNLYRAIKKK
>ID03562-ABP_both
QFTNVSCTTSKECWSVCQRLHNTSRGKCMNKKCRCYS
>ID03566-ABP_both
QIINNPITCMTNGAICWGPCPTAFRQIGNCGHFKVRCCKIR
>ID03567-ABP_pos
QINWGSVVGHCIGGAIIGGAFSGGAAAGVGCLVGSGKAIINGL
>ID03568-NO
QKLCQRPSGTWSGVCGNNNACKNQCIRLEKARHGSC
>ID03572-NO
QQCGRQAGNRRCPNNLCCSQFGYCGRTNEYCCTGFGCQSNCRRCG
>ID03575-ABP_both
QWGGG
>ID03580-ABP_both
RAGLQWPIGRLLRRLLRRLLR
>ID03583-NO
RDCKSDSHKFHGACFSDTNCANVCQTEGFTRGKCDGIHCHCIKDC
>ID03584-NO
RDCRSQSKTFVGLCVSDTNCASVCLTEHFPGGKCDGYRRCFCTKDC
>ID03586-NO
RECRSESKKFVGLCVSDTNCASVCLTERFPGGKCDGYRRCFCTKDC
>ID03588-ABP_both
RFGRFLRKIRRFRPKVTITIQGSARF
>ID03590-ABP_both
RFWPVVIRTVVAGYNLYRAIKKK
>ID03592-ABP_both
RGLRRLGRKIAHGVKKYG
>ID03594-ABP_both
RGRKSSRRKK

>ID03598-ABP_both
RILTMTKRVKMPQLYKQIVCRLFKTC
>ID03599-ABP_both
RISFKKGKGSWIKNGLIKGIKGLGKEISLDVIRTGIDIAGCKIKGEC
>ID03600-ABP_both
RKFRNKIKEKLKKIG
>ID03602-NO
RKSYKALHKRAR
>ID03603-NO
RLGDGCTR
>ID03604-ABP_both
RLGTALPALLKTLLAGLNG
>ID03605-ABP_both
RLKLLLLLRLK
>ID03606-ABP_both
RLKLLLLRLK
>ID03607-ABP_both
RLKLLLRLK
>ID03610-ABP_neg
RQRDPQQQYEQCQERCQRHETEPRHMQTCQQRCERRYEKEKRKQQ
>ID03612-ABP_both
RRLFRRILRWL
>ID03613-ABP_both
RRLFRRILRYL
>ID03616-ABP_both
RRWFWR
>ID03617-ABP_both
RRWKIVVIRWRR
>ID03618-ABP_both
RRWRIVVIRVRR
>ID03619-ABP_both
RRWWFR
>ID03620-ABP_both
RRYYRF
>ID03622-NO
RTCASQSQRFKGKCVSDTNCENVCHNEGFPGGDCRGFRRRCFCTRNC
>ID03623-NO
RTCESPSNKFQGVCLNSQSCAKACPSEGFSGGRCSSLRCYCSKAC
>ID03624-NO
RTCESQSHKFKGPCFSDSNCATVCRTENFPRGQCNQHHVERKCYCERSC
>ID03625-NO
RTCESQSHRFKGTCVSASNCANVCHNEGFVGGNCRGFRRRCFCTRHC
>ID03626-NO
RTCETSSNLFNGPCLSSSNCANVCHNEGFSDGDCRGFRRRCLCTRPC
>ID03628-NO
RVCMGKSQHHSFPCISDRLCSNECVKEDGGWTAGYCHLRYCRCQKAC
>ID03630-NO
RVCRRRSAGFKGLCMSDHNCAQVCLQEGWGGGNCDGVIRQCKCIRQC
>ID03631-ABP_both
RVCSAIPLPICH
>ID03632-ABP_both

RWKPFKKELKVGRNIRDGIIKAGPAVAVIGQATSIARPTGK
>ID03633-ABP_both
RWRWRWRWRW
>ID03634-ABP_both
RYPAVGYT
>ID03635-ABP_neg
SDEKASPDRHHRFSLSRYAKLANRLSKWIGNRGNRLANPKLLETFKSV
>ID03636-NO
SDIEKLKEAIRDTNKAVQSVQSSIGNLIVAIKSVQ
>ID03637-ABP_both
SDYLNNNPLFPRYDIGNVELSTAYRSFANQKAPGRLNQNWALTADYTYR
>ID03638-ABP_both
SFKKFWGGVKAIFKGARKGWK
>ID03640-ABP_both
SGTPEKERESGRLLGVVKRYIVCVRNPCP
>ID03641-ABP_neg
SGTSEKERESERLLGVVNPLIKCFRSPCP
>ID03642-ABP_neg
SGTSEKERESGRLLGVVKRLIVGFRSPFR
>ID03644-ABP_both
SILSTLKDVGISAIKSAGSGVLSTLLCKLNKNC
>ID03646-NO
SKVLHLEGEVNKIALLSTNKAVVSLSNGVSVLT
>ID03648-NO
SLERLDVGTNLGNAIAKLEAKELLESSDQILRSM
>ID03649-ABP_both
SLFGTFAKMALKGASKLIPHLLPSRQQ
>ID03653-ABP_both
SLLGTVKDLLIGAGKSAAQSVLKGLSCKLSKDC
>ID03656-NO
SRSELIVHQRLF
>ID03657-NO
SRSELIVHQRMK
>ID03659-ABP_both
SSLLEKGLDGAKKAVGGLGKLGKDAVEDLESVGKGAVHDVKDVLDSVL
>ID03660-NO
SVALDPIDISIELNKAKSDLEESKEWIRRSNQKLD
>ID03664-NO
SWLRDIWDWVCEVLSDFK
>ID03666-ABP_both
SYVGDCGSNGGSCVSSYCPYGNRLNYFCPLGRTCCRRSY
>ID03667-NO
TAAVALVEAKQARSDIEKLKEAIRDTNKAVQSVQS
>ID03670-ABP_both
TKPTLLGLPLGAGPAAGPGKR
>ID03671-NO
TLNNSVALDPIDISIELNKAKSDLEESKEWIRRSN
>ID03672-NO
TPCGESCVYIPCISGVIGCSCTDKVCYLN
>ID03673-ABP_both
TSRCYIGYRRKWCS

>ID03674-NO
TTCCPSIVARSNFNVCRLPGTPEALCATYTGCIIIPGATCPGDYAN
>ID03678-ABP_pos
TVVTQA
>ID03679-ABP_pos
TVYTNA
>ID03680-ABP_pos
TVYTQA
>ID03682-ABP_both
TWLPAVIKIQAHWRGYRQRKIYL
>ID03683-NO
VALDPIDISIELNKAKSDLEESKEWIRRSNQKLDS
>ID03684-ABP_both
VARGWKRKCPLFGKGG
>ID03685-ABP_both
VDKPPYLPRPRPPRRIYNR
>ID03687-ABP_both
VFQFLGKIIKKVGNFVKGFSKVF
>ID03690-ABP_both
VIRTVVAGYNLYRAIKKK
>ID03691-ABP_neg
VIVKAIATLSKKLL
>ID03692-ABP_neg
VKLEILGSKGGAKI
>ID03693-ABP_both
VKLFPVKLFP
>ID03695-ABP_neg
VNHSACAAHCILRGKTGGRCNSNAVCVCR
>ID03699-ABP_both
VRLEFKLQQTSCRKRDWKKP
>ID03700-ABP_both
VRLRIRVRVIRK
>ID03702-ABP_both
VSKIKKYLKYKDRI
>ID03703-ABP_pos
WAIVLL
>ID03706-ABP_neg
WFRKQLKW
>ID03708-ABP_both
WKKIPKFLHLLKKF
>ID03710-ABP_both
WLLVNG
>ID03712-ABP_pos
WNDTGKDADGAEY
>ID03718-ABP_both
YCSYTMEA
>ID03719-ABP_both
YGQSTHAVIYAQGYTYSSDWR
>ID03723-ABP_both
AAAAGSVWGAVNYTSDCNGECKRRGYKGGYCGSFANVNCWCET
>ID03725-ABP_both

AAGGVKKPKKAAAAKKSPKKPKKPAAA
>ID03726-ABP_both
AAKIILNPKFRCKAAFC
>ID03728-NO
AAPCFCSGKPGRGDLWILRGTCPGGYGYTSNCYKWPNICCYPH
>ID03730-ABP_both
AASKAAKTLAKLLSSLLKL
>ID03731-ABP_both
AASKALRTASRLARSLLT
>ID03732-ABP_both
ACDFQQCWVTCQRQYSINFISARCNGDSCVCTFRT
>ID03734-ABP_both
ADSGEGDFLAEGGGVR
>ID03737-ABP_neg
AFRKQLKW
>ID03740-NO
AHKCICYFP
>ID03744-ABP_both
AKAVKPKTAKPKTAKPKTAKA
>ID03746-ABP_both
AKKPVAKKAAGGVKKPK
>ID03748-ABP_both
AKKVFKRLEKLFSKIQNWK
>ID03749-ABP_both
AKKVFKRLEKLFSKIWNDK
>ID03750-ABP_both
AKKVFKRLEKLFSKIWNWK
>ID03753-ABP_both
AKRLKKLAKKIWKWL
>ID03756-NO
ALGGLLADVVKSKEQPA
>ID03757-ABP_both
ALRSAVRTVARVGRAVLPHVAI
>ID03758-ABP_both
ALWDTLLKKVLKAAAKAALDAVLVGANA
>ID03761-ABP_both
ALWKTLLKKVLKAAAKAALKAVLVGANA
>ID03762-ABP_both
ALWKTMLKKAAHVGKHVGKAALGAAARRRK
>ID03766-ABP_both
ANLDAIIKIQAWARMWAARRQYL
>ID03768-ABP_pos
APAKCTPYCYPTRDGVFCGVRCDFQHHHHHH
>ID03769-ABP_neg
APKKQLKW
>ID03771-ABP_neg
APRKNLKW
>ID03772-ABP_neg
APRKNVKW
>ID03773-ABP_neg
APRKNVRF

>ID03774-ABP_neg
APRKQLKW
>ID03775-ABP_neg
APRRQLKW
>ID03777-ABP_neg
ARDGYIVDEKGCKFACFIN
>ID03778-NO
ARHGSCNYVFPAH
>ID03779-NO
ARHGSCNYVFPAHK
>ID03780-NO
ARHGSCNYVFPAHKC
>ID03781-NO
ARHGSCNYVFPAHKCI
>ID03782-NO
ARHGSCNYVFPAHKCIC
>ID03783-NO
ARHGSCNYVFPAHKCICY
>ID03784-NO
ARHGSCNYVFPAHKCICYF
>ID03785-ABP_both
ARLAKKALRRLAKKD
>ID03786-ABP_pos
ARSYGNGVYCNNKKCWVNRGEATQSIIGGMISGWASGKAGM
>ID03787-ABP_both
ARTKQTARKSTGGKAPRKQLAT
>ID03788-ABP_both
ASAAGAVRAGDDETLLKPVLNSLDNLVSGL
>ID03789-ABP_both
ASAAGAVRAGDDETLLNPVLNSLDNLVSGL
>ID03790-ABP_both
ASAAGAVREDDDETLLNPVLNSLDNLVSGL
>ID03791-NO
ASHGACHKRENHWKCFCYF
>ID03792-ABP_both
ASHLGHHALDHLLK
>ID03794-ABP_both
ASITHVKNRGKYIYMHLKFRKTNVLI
>ID03795-ABP_pos
ASIVKTTIKASKKLCRGFTLTCGCHFTGKK
>ID03797-ABP_both
ATCDLFSFQSKWVTPNHAACAAHCTARGNRGGRCKKAVCHCRK
>ID03798-ABP_both
ATCDLLSISTPWGSVNHAACAAHCLALNRGFRGGYCSSKAVCTCRK
>ID03801-ABP_pos
ATYYGNGLYCNKQKCWVDWNKASREIGKIIVNGNVQHGPWAPR
>ID03805-ABP_both
AVGRHGRRFGLRKHRKH
>ID03807-ABP_both
AVKPKTAKPKTAKPKTA
>ID03811-ABP_both

CADLRGKTFCRLFKSYCDKKGIRGRLMRDKCSYSCGCRG
>ID03812-ABP_neg
CAKAKAKAGSGAKAKAKAC
>ID03813-ABP_both
CARLNCVPKGTSGNTETCPCYASLHSCRKYG
>ID03814-ABP_both
CETPSKHFNGLCIRSSNCASVCHGEHFTDGRCQGVRRRCMCLKPC
>ID03815-ABP_neg
CFKFKFKFGSGFKFKFKFC
>ID03817-NO
CICYFP
>ID03818-NO
CIRLEKARHGSCNYV
>ID03819-NO
CKNQCIRLEKARHGS
>ID03820-ABP_both
CLAIGRR
>ID03821-ABP_both
CLSGRYKGPCAVWDNETCRRVCKEEGRSSGHCSPSLKCWCEGC
>ID03823-NO
CNYVFPAHKC
>ID03824-NO
CNYVFPAHKCICY
>ID03828-ABP_both
CSYTMEA
>ID03831-ABP_neg
CVKVQVKVGSGVKVQVKVC
>ID03832-ABP_neg
CVKVRVKVGSGVKVRVKVC
>ID03833-ABP_neg
CVKVSVKVGSGVKVSVKVC
>ID03834-ABP_pos
CVWGGDCTDFLGCGTAWICV
>ID03835-ABP_neg
CWKWKWKWGSGWKWKWKWC
>ID03837-NO
CYIQNCPLG
>ID03838-ABP_both
DAQEKRQPWIPFV
>ID03839-ABP_both
DAQEKRQPWLPFV
>ID03842-ABP_both
DEDLDE
>ID03851-ABP_both
DNTDSVVKIQSWFRMATARKSYL
>ID03853-ABP_both
DPVTCLKSGAICHPVFCPRRYKQIGTCGLPGTKCCKKPN
>ID03854-NO
DRVYIHPF
>ID03857-ABP_both
DVIKKVASVIGGL

>ID03858-NO
DVPKSDQFVGLM
>ID03859-NO
DWLKAFYDKVAEKLKEAF
>ID03860-ABP_both
DWTCWSCLVCAACS
>ID03861-NO
DYMGWMDF
>ID03864-ABP_both
ECKFTVKPYLKRFQVYYKGRMWCP
>ID03866-ABP_both
EKAAAKSAAAKTLARR
>ID03867-NO
EKARHGSCNYVF
>ID03868-NO
EKARHGSCNYVFP
>ID03869-NO
EKARHGSCNYVFPA
>ID03870-NO
EKARHGSCNYVFPAH
>ID03871-NO
EKARHGSCNYVFPAHK
>ID03872-NO
EKARHGSCNYVFPAHKC
>ID03873-NO
EKARHGSCNYVFPAHKCI
>ID03874-NO
EKARHGSCNYVFPAHKCIC
>ID03875-NO
EKARHGSCNYVFPAHKCICY
>ID03876-ABP_both
EKTLARTAAKTALKK
>ID03878-ABP_both
ELAKKALKALKKALKSAR
>ID03879-ABP_both
ELAKKALRALKKALKSAK
>ID03883-ABP_both
ENMFNIKSSVESDSFWG
>ID03884-ABP_both
ERSAAKSAARSLARR
>ID03887-ABP_both
ESKAAKAAKKAAKAKASE
>ID03888-ABP_both
ESLAKALSKEALKALK
>ID03889-ABP_both
ESLKARSLKKSLKLKKLL
>ID03890-ABP_both
ESSLKKKALSKLSKLLKKG
>ID03892-ABP_both
ETELAKKALKALKLKKLA
>ID03893-ABP_both

ETFAKKALKALEKLLKKG
>ID03894-ABP_both
FASLLGKALKALLAKLAKQ
>ID03895-ABP_both
FASLLGKLAKKLAKKALK
>ID03897-NO
FCTCNVKGFNAKNKRGIIYP
>ID03899-ABP_both
FFGHLFRGIINVGKHIHGLLSG
>ID03900-ABP_both
FFGPLIKIATGVLPNLICKALGKC
>ID03902-NO
FFHHIFRGIVHVGKSIHKLVTG
>ID03906-ABP_both
FFWHHIGHALDAAKRVHGMLSG
>ID03908-ABP_both
FGLIPSLIGGLVSAFK
>ID03910-ABP_pos
FIGAVAGLLSKIF
>ID03912-ABP_both
FIKWKFRWWKWRK
>ID03916-ABP_both
FKCRRWQWRMKKLGAPSITCVRRAFA
>ID03917-NO
FKDLKKIANIINSIFKK
>ID03919-ABP_neg
FKIKPGKVLDKFGKIVGKVLKQLKKVS
>ID03920-ABP_neg
FKIKPGKVLDKFGKIVGKVLKQLKKVSAVAKV
>ID03921-ABP_both
FKKFWKWFRRF
>ID03924-ABP_both
FLAKAVAKAAAKALAKAL
>ID03925-ABP_pos
FLFSLIPSAISGLISAF
>ID03927-ABP_both
FLGALFKALSKLL
>ID03929-ABP_neg
FLKLLKKLAAKLF
>ID03931-ABP_both
FLPFLLSALPKVFCFFSKKC
>ID03933-ABP_both
FLPIVAKLLSGLL
>ID03934-NO
FLPLIAGLLGKLF
>ID03935-ABP_both
FLPLILPSIVTALSSFLKQG
>ID03936-ABP_both
FLPLLAGLAANFLPKIFCKITRK
>ID03937-ABP_both
FLPPSPWKETFRTT

>ID03939-ABP_both
FLPVIAGVLSKLF
>ID03940-ABP_both
FLSLIPKIAGGIASLVKDL
>ID03942-NO
FPAHKC
>ID03944-NO
FPAHKCICYFPC
>ID03945-ABP_both
FPISTLLKWWKG
>ID03947-ABP_both
FPVTFRFFKFFKG
>ID03948-ABP_both
FPVTWGWWKWWKG
>ID03949-ABP_both
FPVTWPTKWLKG
>ID03950-ABP_both
FPVTWPTKWRKG
>ID03952-ABP_both
FPVTWPTKWWKS
>ID03953-ABP_both
FPVTWRWWTWWKG
>ID03954-ABP_both
FSEAIKKIIDFLGEGLFDIIKKIAESF
>ID03955-ABP_both
FSGGNCRGFRRRCFCTK
>ID03956-ABP_both
FSIARLLKWWKG
>ID03957-ABP_neg
FSISPGKVLDKFGKIVGKVLKQLKKVSAVAKV
>ID03958-ABP_both
FSLLPSLIGGLVSAIK
>ID03963-NO
FVKLKKIANIINSIFKK
>ID03965-NO
FVKLKKILNIINSIFKK
>ID03966-ABP_pos
FVLPLLGILPKELCIVLKKNC
>ID03967-ABP_pos
FVNVVPTFGKKKGPNANS
>ID03968-ABP_both
FVPWFSKFLPRIL
>ID03970-ABP_both
FWGALAKGALKLIGPGSLFSSFSKKD
>ID03972-NO
FWSFLVKAASKILPSLIGGGDDNKSSS
>ID03974-ABP_pos
GACRAIRHIPRRIR
>ID03978-ABP_pos
GALWGAPAGGVGALPGAFVGAHVGAIAGGFACMGGMIGNKFN
>ID03979-ABP_both

GASPALWGCDSFLGYCRIACFAHEASVGQKDCAEGMICCLPNVF
>ID03980-ABP_pos
GDCGGTCTWTKDCSICPSWSCWSWSC
>ID03981-NO
GDGTGPGPGP
>ID03982-ABP_both
GESLASKAAKAAER
>ID03984-ABP_both
GFFTLIKAANKLINKTVNKEAGKGGLEIMA
>ID03989-ABP_both
GFGSKPLDSFGLNFF
>ID03991-ABP_both
GFKEVLKADLGSLVKGIAAHVAN
>ID03993-ABP_both
GFLDIIKDTGKEFAVKILNNLKCKLAGGCPP
>ID03995-ABP_both
GFLDVIKHVGKAALGVVTHLINQ
>ID03996-ABP_both
GFLDVVKHIGKAALGAVTHLINQ
>ID03998-ABP_both
GFMDTAKNVFKNVAVTLLDKLKCKIAGGC
>ID03999-ABP_both
GFMGDTLKGIARNAALALMNAAQCKLSGKC
>ID04006-ABP_both
GGLRSLGRKILRAWKKYGPIIVPIIRI
>ID04007-ABP_both
GGYKNFYGSALRKGFYEGEAGRAIRR
>ID04010-ABP_both
GIGKALKKAKKGIGAVLKVLTTGL
>ID04011-NO
GIGKFLHSAGKFGKAFVGEIMK
>ID04012-ABP_both
GIGKFLHSAKKFGKAFVAEIMNS
>ID04013-NO
GIGKFLHSAKKFGKAFVGEIMNSK
>ID04014-ABP_both
GIGKHVGKALKGLKGLLKGLGEC
>ID04015-ABP_both
GIGSMLLGLAKNVGMSLLNKAQCKISGKC
>ID04017-ABP_both
GIIKKIIKKIIKKI
>ID04018-ABP_both
GIIKKIIKKIIKKIIKKI
>ID04019-ABP_pos
GIIKVIKSLIEQFTGK
>ID04021-ABP_both
GIINTLQKYYARVRGGRAAVLSALPKEEQIGKASTRGRKAARRKK
>ID04022-ABP_both
GIINTLQKYYFRVRGGRFAVLSFLPKEEQIGKFSTRGRKFFRRKK
>ID04024-ABP_both
GIINTLQKYYWRVRGGRWAVLSWLPKEEQIGKWSTRGRKWWRRKK

>ID04025-ABP_both
GIINTLQKYYYRVRGGRYAVLSYLPKEEQIGKYSTRGRKYYRRKK
>ID04026-NO
GIKEFKRIVQRIKDFLRNLV
>ID04027-NO
GIKEWKRIVQRIKDFLRNLV
>ID04029-ABP_both
GILDAIKAFANALG
>ID04030-ABP_both
GILDKLKEFGISAARGVAQSLLNTASCKLAKTC
>ID04031-ABP_both
GILDPIKAFAKAAG
>ID04036-ABP_both
GIMNTVKDVATGVATHLLNMVKCKITGC
>ID04037-ABP_both
GIMRVFKGVLKTAGKSVAKNVAGSFLDRLKCKISGGC
>ID04038-ABP_both
GIMSSLMKKLAAHIAK
>ID04039-NO
GIPCAESCVWIPCTVTAIVGCSCSDKVCYN
>ID04041-NO
GIVEQCCASVCSLYQLENYCN
>ID04042-ABP_both
GIWKTIKSMGKVFAGKILQNL
>ID04043-ABP_both
GKFLHSAGKFGKAFLGEVMIG
>ID04044-ABP_both
GKIIKLKASLKLL
>ID04045-ABP_both
GKKLFVNVLDKIRCKVAGGC
>ID04046-ABP_both
GKLTKDKLKRGAKKALNVASKVAPIVAAGASIAR
>ID04047-ABP_neg
GKVLDKFGKIVGKVLKQLKKVSAVAKV
>ID04048-ABP_both
GLAANFLPKIFCKITRKC
>ID04049-ABP_both
GLADYWRTAFRANFANLGPGIRCKSARC
>ID04052-NO
GLASLLGKALKAGLKIGTHFLGGAPQQ
>ID04054-ABP_both
GLFDIIKKIAESFKFSEAIKKIIDFLG
>ID04056-NO
GLFDIIKKVASVVGLASP
>ID04060-ABP_both
GLFDVIAKVASVIGGL
>ID04061-NO
GLFDVIKKVASVIGLASQ
>ID04067-NO
GLGSVLGKILKMGANLLGGAPKGA
>ID04068-ABP_both

GLKGLLGKALKGIGKHIGKAQGC
>ID04069-ABP_neg
GLLDTKNTAKNLAVGLLDKIKCKMTGC
>ID04070-ABP_both
GLLNVIKDTAQNLFAAALDKLKCKVTKCN
>ID04071-ABP_both
GLLNVIKDTAQNLFAAALEKLKCKVTKCN
>ID04076-NO
GLPLCGETCVGGTCNTPGCSCGWPVCVRN
>ID04079-NO
GLPVCGETCKGGTCNTPGCTCSWPVCTRN
>ID04080-NO
GLPVCGETCVGGTCNTPGCKCSWPVCTRN
>ID04081-NO
GLPVCGETCVGGTCNTPGCTCSWPVCTRK
>ID04086-NO
GLWSNIKTAGKEAAKAALKAAGKAALGAVTDAV
>ID04089-ABP_pos
GMKCKFCCNCCNLNGCGVCCRF
>ID04093-ABP_both
GPIRRPKPRPRPRPE
>ID04095-NO
GRFKRFRKKFKKLFK
>ID04097-ABP_both
GRKKRRQRRRPPQ
>ID04101-ABP_both
GRWKRWRKKWKKLWKKLS
>ID04102-NO
GSCGAPISKYDFQVLAKRPPPCRRPRLENTEDVTHTTRP
>ID04104-NO
GSCNYVFPAHKCI
>ID04105-NO
GSCNYVFPAHKCIC
>ID04106-NO
GSCNYVFPAHKCICY
>ID04107-NO
GSCNYVFPAHKCICYF
>ID04110-ABP_both
GTFIKQQRKQKQQRHHTSGTRKRMAK
>ID04111-ABP_both
GTKRGKLCRISRLAL
>ID04113-ABP_pos
GTPLALLGGAATGVIGYISNQTCPTTACTRAC
>ID04116-NO
GTWSGVCGNNNACKN
>ID04117-NO
GVCGNNNACKNQCIR
>ID04119-ABP_both
GVFDIIKGAGKQLIAHAMEKIAEKVGLNKDGN
>ID04122-ABP_both
GVIDIIKGAGKDLIAHAIGKLAEKV

>ID04125-ABP_both
GVLSVIKNALPGIMRF
>ID04127-ABP_both
GWGSFFKKAAHVGKHVGKAALGAAARRRK
>ID04128-NO
GWGSFFKKAAHVGKHVGKAALHTYL
>ID04130-ABP_both
GWLDVAKKIGKAAFNVAKNFL
>ID04131-ABP_both
GWLLLEYIPVIAAL
>ID04132-ABP_both
GWWRRTVDKVRNAGRKVAGFASKACGALGH
>ID04135-ABP_both
GYYCPFRQDKCHRHCRSFGRKAGYCGNFLKRTCICVKK
>ID04136-ABP_both
HFLGKLVNLAKKIL
>ID04137-NO
HFRWGKPV
>ID04138-NO
HGSCNYVFPAHK
>ID04140-NO
HGSCNYVFPAHKCI
>ID04141-NO
HGSCNYVFPAHKCIC
>ID04143-NO
HGSCNYVFPAHKCICYF
>ID04146-NO
HKCICY
>ID04147-NO
HKCICYFPC
>ID04148-ABP_both
HLGHHALDHLLK
>ID04149-ABP_both
HNSSKQWSHWLWHNGIRI
>ID04151-ABP_both
HQFRFRFRVRRK
>ID04152-NO
HSQGTFTSDYSKYLDSRRAQDFVQWLMNT
>ID04153-NO
HVDKKVADKVLLLKQLRIMRL
>ID04156-NO
ICYFPC
>ID04157-ABP_both
IDWKKIFEKVKDLV
>ID04158-ABP_both
IDWKKIFEKVKNLV
>ID04160-ABP_pos
IGCGGGAVACQNYRQFCR
>ID04161-ABP_both
IGCWTKSIPPRPCFVK
>ID04162-ABP_both

IKELLPHLSGIIDSVANAIK
>ID04163-ABP_both
ILELAGNAARDNKKTRIIPRHLQL
>ID04165-ABP_both
ILGEIWKGIKDIL
>ID04172-ABP_both
ILKKFMLHRGTKVYKMRTLSKRSH
>ID04173-NO
ILKKWPWWPWR
>ID04174-NO
ILKKWPWWPWRR
>ID04176-ABP_both
ILKWKWPWWPWRR
>ID04178-ABP_both
ILPLKWPWWPWRR
>ID04179-ABP_both
ILPWKLPWWPWRR
>ID04180-ABP_both
ILPWKWKWWPWRR
>ID04181-ABP_both
ILPWKWPLWPWRR
>ID04182-ABP_both
ILPWKWPWLPWRR
>ID04183-ABP_both
ILPWKWPWWPARR
>ID04184-ABP_both
ILPWKWPWWPLRR
>ID04185-ABP_both
INFLKLGKKILGAL
>ID04188-ABP_both
INSLKLGKKILGAL
>ID04189-ABP_both
INWKKGKEVLKAL
>ID04190-ABP_both
INWKKIASIGKEVL
>ID04191-ABP_both
INWKKIASIGKEVLK
>ID04192-ABP_both
INWKKIASIGKEVLKA
>ID04193-ABP_both
INWKKIASIGKEVLKAI
>ID04194-ABP_both
INWKKIFEKVKDLV
>ID04195-ABP_both
INWKKIFEKVSNLV
>ID04200-ABP_both
INWLKAKKVAGMIL
>ID04202-ABP_both
INWLKLGKKLLSAL
>ID04203-ABP_both
INWLKLGKKMMSAI

>ID04645-ABP_both
RERSKGSKYLYVG
>ID04647-ABP_both
RGFRKHFNKLVKKVKHTISETAHVAKDTAVIAG
>ID04648-ABP_both
RGGRLYRRRFVVGR
>ID04649-ABP_both
RGLRALGRKIAHGVKAYG
>ID04650-ABP_both
RGLRRLGRKIAHGVKKY
>ID04651-ABP_both
RGLRRLGRKIAHGVKKYGATVLRIIRIA
>ID04652-ABP_both
RGLRRLGRKIAHGVKKYGPTVKRIKRKA
>ID04653-NO
RHGSCNYVF
>ID04662-ABP_both
RIIRKIIHII
>ID04663-ABP_both
RIIRKIIHIIK
>ID04664-ABP_both
RIKRFWPVVIR
>ID04665-ABP_both
RIKRFWPVVIRT
>ID04666-ABP_both
RIKRFWPVVIRTVV
>ID04668-ABP_both
RIKRFWPVVIRTVVAGY
>ID04669-ABP_both
RIKRFWPVVIRTVVAGYN
>ID04671-ABP_both
RIKRFWPVVIRTVVAGYNLY
>ID04672-ABP_both
RIKRFWPVVIRTVVAGYNLYR
>ID04676-ABP_both
RIWFQNRRMRWRR
>ID04677-NO
RKKRRQRRR
>ID04688-ABP_both
RLFRHAFKAVLRL
>ID04689-ABP_both
RLRLRIGRR
>ID04690-ABP_both
RLYRRRFVVGR
>ID04692-ABP_both
RMKLNAKKLSFC
>ID04693-NO
RMKQIEDKIEEIESKQKKIENEIARIKKLLQLTVWGIKQLQARIL
>ID04694-ABP_both
RNGCIVDPRCPYQQCRRPLYCRRR
>ID04696-NO

RPPGFTPFR
>ID04697-ABP_both
RQIKIWFQNRRLKWKK
>ID04698-ABP_both
RQIRIWFQNRRMRWRR
>ID04701-ABP_both
RRAAVVLIVIRR
>ID04702-ABP_both
RRIIRKIIHII
>ID04706-NO
RRQRRTSKLMKRGGKLAKLAKKLAKLAK
>ID04707-ABP_both
RRRAAFAAWAAFAARRR
>ID04711-ABP_both
RRRRRRR
>ID04712-ABP_both
RRRRRRRRR
>ID04713-ABP_both
RRRRRRRRRR
>ID04714-ABP_neg
RRVVRRVRRVVRRVVRVVRRVVRR
>ID04715-ABP_both
RRWQWRMKK
>ID04716-ABP_both
RRWWKWWWR
>ID04717-NO
RSELIVHQR
>ID04718-NO
RTCESQSHRFKGLCFSKSNCGSVCHTEGFNGGHCRGFRRRCFCTRHC
>ID04720-NO
RVCMGKSAGFKGLCMRDQNCAQVCLQEGWGGGNCDGVMRQCKCIRQCW
>ID04721-ABP_pos
RVIEVVQGACRAIRHIPRRIR
>ID04722-ABP_pos
RVIEVVQGAYRAIRHIPRRIRQGLERIL
>ID04723-ABP_both
RVIRVVQGACRAIRHIPRRIR
>ID04724-ABP_both
RVIRVVQGACRAIRHIPRRIRQGLRRIL
>ID04725-ABP_both
RVIRVVQRACRAIRHIVRRIRQGLRRIL
>ID04727-ABP_neg
RVVRVVRRVVRR
>ID04728-ABP_neg
RVVRVVRRVVRRVRRVVRRVVRVVRRVVRRVRRVVRRVVRVVRRVVRR
>ID04729-ABP_neg
RVVRVVRRWVRR
>ID04731-ABP_both
RWFKIQLQIRRWKNKK
>ID04733-ABP_both
RWFRIQLQIRRWRNRR

>ID04734-NO
RWGRWLRKIRRWRPK
>ID04735-ABP_both
RWIRVVQRWCRAIRHIWRRIRQGLRRWLRVV
>ID04737-ABP_both
RWKRWWRRKK
>ID04738-ABP_both
RWRWRWF
>ID04745-ABP_both
SDKPDVKEVESFDKSKLKKVETQEKNPLPTKETIEQEKKG
>ID04746-NO
SDWSLWECCSTGSLFACC
>ID04747-ABP_both
SEEEKRQPWLPFG
>ID04748-NO
SEEPPISLDLTFHLLREVLEMARAEQLAQQAHSNRKLMEII
>ID04749-NO
SGECNMYGRCPPGYCCSKFGYCGGVRAYCG
>ID04750-ABP_pos
SGIPCTIGAAVAASIAVCPTTKCSKRCGKRKK
>ID04751-NO
SGIVQQQNNLLRAIEAQQHLLQLTVWGIKQLQARIL
>ID04752-NO
SGPNGQCGPGWGGCRGGLCCSQYGYCGSGPKYCAH
>ID04754-ABP_both
SGRGKTGGKARAKAKTRSSRAGLQFPVGRVHRLLRKGNYAQRVGAGAPVY
>ID04755-NO
SGRGSCRSQCMRRHEDEPWRVQECVSQCRRRRGGGD
>ID04759-ABP_both
SILSTLKDVGISAIKNAGSGVLKTLLCKLNKNCEK
>ID04761-NO
SKWQHQQDSCRKQLQGVNLTPCEKHIMEKIQGRGDDDDDDDDD
>ID04762-ABP_both
SLSRYAKLANRLANPKLLETFLSKWIG
>ID04765-NO
SNASVWECCSTGSWVPFTCC
>ID04766-ABP_pos
SNDSLWYGVGQFMGKQANCITNHPVKHMIIPGYCSKILG
>ID04769-ABP_both
SPKKTKPVKPKKVA
>ID04770-NO
SRRSCHRNKGVCALTRCPRNMRQIGTCFGPPVKCCR
>ID04772-NO
SSSKEENRIIPGGI
>ID04775-ABP_both
SVSNIPESIGF
>ID04776-NO
SWLRDIWDWACEVLSDFK
>ID04777-NO
SWLRDIWDWECEVLSDFK
>ID04778-NO

SWLRDIWDWGCEVLSDFK
>ID04779-NO
SWLRDIWDWICELLSDFK
>ID04780-NO
SWLRDIWDWICEVLSDFK
>ID04781-NO
SWLRDIWDWKCEVLSDFK
>ID04782-NO
SWLRDIWDWLCELLSDFK
>ID04783-NO
SWLRDIWDWLCEVLSDFK
>ID04785-NO
SWLRDLWDWICELLSDFK
>ID04788-NO
SWLRDLWDWLCEVLSDFK
>ID04791-NO
TDVILMCFSIDSPDSLENI
>ID04796-NO
THRPPMWSPVWPGGGKLLLKLLKKLLKLLKKK
>ID04802-ABP_both
TSKYR
>ID04803-NO
TTWEAWDRAIAEYAARIEALIRAAQEQQEKLEAALREL
>ID04804-NO
TTWEAWDRAIAEYAARIEALIRAAQEQQEKNEAALREL
>ID04805-NO
TTWEAWDRAIAEYAARIEALIRALQEQQEKNEAALREL
>ID04807-ABP_neg
TWLKKRRWKKAK
>ID04808-ABP_both
TWWRWW
>ID04811-ABP_both
VAGRAQGM
>ID04814-NO
VDCGANPFKVACFNSCLLGPSTVFQCADFCACRLPAG
>ID04816-NO
VDCSPGIWQLDCTHL
>ID04817-NO
VDIHVWDGV
>ID04821-ABP_neg
VGIGGGGGGGGGGSCGGQGGGCGGCSNGCSGGNGGSGGSGSH
>ID04822-ABP_neg
VGKVLKQLKKVSAVAKVAMKKGAALLK
>ID04823-ABP_both
VGPGGECGGRFGGCAGGQCCSRFGFCGSGPKYCAH
>ID04824-ABP_both
VGRKHSILNCIPYLKKKKIMRL
>ID04825-ABP_pos
VGTIIKIIKAIIDIFAK
>ID04826-ABP_both
VGVGGGFGR

>ID04828-ABP_both
VNWKKILAKIIKVVK
>ID04829-ABP_both
VNWKKILKKIIKVVK
>ID04830-ABP_both
VNWKKILPKIIKVVK
>ID04834-ABP_both
VRLWIRVAVIRA
>ID04835-ABP_both
VRRFAWWWAFLRR
>ID04836-ABP_both
VRRFGWWWGFLRR
>ID04837-ABP_both
VRRFPFFFPFLRR
>ID04839-ABP_both
VRWRIRVAVIRA
>ID04840-ABP_neg
VSAVAKVAMKKGAALLKKMGVKISPLK
>ID04842-ABP_both
VSFPWSCAALSGVCRQGACLPSELYFGPLGCGKGSLCCVSYFL
>ID04843-ABP_both
VSSKYLSKALVKAGR
>ID04844-ABP_both
VSSKYLSKVKVKAGK
>ID04847-ABP_both
VTQPLAPVHNPISV
>ID04849-ABP_pos
VVQGACRAIRHIPRRIR
>ID04850-ABP_both
VWLSALKFIGKHLAKHQLSKL
>ID04851-ABP_both
WGHKLRSSWNKVKHAVKKGAGYASGACRVLGH
>ID04853-ABP_both
WKKIRVRLSA
>ID04854-ABP_both
WKLFDDGV
>ID04855-ABP_both
WKSDVRRWRSRY
>ID04856-ABP_both
WKSYVRRW
>ID04857-ABP_both
WKSYVRRWR
>ID04858-ABP_both
WKSYVRRWRS
>ID04859-ABP_both
WKSYVRRWRSR
>ID04861-ABP_both
WLKKLLKKLLK
>ID04862-ABP_both
WLKKW
>ID04863-ABP_both

WLLKW
>ID04866-ABP_both
WLSKTYKKLENSAKKRISEGVAIAILGGLR
>ID04867-NO
WMEWDREINNYTSLIHSLIEESQNQQEKNEQELL
>ID04868-ABP_both
WNPFKELEKVGQRVRDAVISAGP
>ID04869-NO
WQEWEQKITALLEQAQIQQEKNEYELQKLDKWASLWEWF
>ID04870-ABP_both
WRWFIH
>ID04871-ABP_neg
WRWRVRVWR
>ID04872-NO
WWPWRRK
>ID04873-ABP_both
WWSYVRRWRSR
>ID04876-NO
YAFGYPS
>ID04878-NO
YCERSSGTWSGVCGNSGKCSNQCQRLEGAAHGSCNYVFPAHKCICYYPC
>ID04882-ABP_neg
YGLVLKYCPKIGYCSNTCSKTQIWATSHGCKMYCCLPASWKWK
>ID04886-NO
YMFHLMD
>ID04889-ABP_both
AAAAAAAAAAGIGKFLHSAKKFGKAFVGEIMNS
>ID04890-ABP_neg
AAAKAALNAVLVGANA
>ID04891-NO
AAAMSQVTN
>ID04892-NO
AAGKFLHSAKKFGKAFVGDIMNS
>ID04894-NO
AAGTTCVTTGWGLTRYTNAN
>ID04895-NO
AAHGACHVRNGKHMCFCYF
>ID04896-ABP_pos
AARRAARRAARR
>ID04897-NO
AASYACLHAACA
>ID04898-NO
AASYKCLHKRCR
>ID04900-NO
AAWWAGIKQEF
>ID04901-NO
AAYFLLKLAGRW
>ID04902-NO
ACAWAGIKQEF
>ID04906-NO
ACVGDGQRCASWSGPYCCDGYYCSCRSMPYCRCRNNS

>ID04907-NO
ACWAAGIKQEF
>ID04908-NO
ACWGAGIKQEF
>ID04909-NO
ACWWAAIKQEF
>ID04910-NO
ACWWAG
>ID04911-NO
ACWWAGAKQEF
>ID04912-NO
ACWWAGIAQEF
>ID04913-NO
ACWWAGIKAEF
>ID04914-NO
ACWWAGIKQAF
>ID04916-NO
ACWWAGIKQEF
>ID04917-NO
ACWWAGIRQEF
>ID04926-ABP_both
ADVRNPLEEFRETDYEVL
>ID04927-NO
AEAASQVTNTATIM
>ID04928-NO
AEAMAQVTN
>ID04929-NO
AEAMSQ
>ID04931-NO
AEAMSQVANTATIM
>ID04932-NO
AEAMSQVT
>ID04933-NO
AEAMSQVTN
>ID04935-NO
AEASQVTNTATIM
>ID04936-ABP_both
AEILFGDVRPPWMPPPIFPEMP
>ID04941-ABP_both
AFFARLLASVRAAVKAFAKKPRLIGLSTLL
>ID04942-ABP_both
AFGMALKLLKKVL
>ID04943-NO
AGCKNFFWKTFTSC
>ID04944-NO
AGDES
>ID04946-NO
AGERIVDIIA
>ID04947-NO
AGERIVDIIATDIQ
>ID04949-ABP_both

AGGLDDLLEPVLNSADNLVHGL
>ID04952-ABP_both
AGLLDILGL
>ID04955-NO
AIAIFKRIAKINFKALMGEAVQT
>ID04956-NO
AIAKFAKKALKSMLALMGEAVQT
>ID04957-NO
AIANFERLMKKLIWALMGEAVQT
>ID04959-ABP_both
AIHDILKYGKPS
>ID04960-ABP_both
AINPKSVQSLL
>ID04963-ABP_both
AIPWIWIWWLLRKG
>ID04965-ABP_both
AIPWSIWWRLLFKG
>ID04981-ABP_both
AKAKAYPAKAKAYP
>ID04982-NO
AKGFAANHS
>ID04983-NO
AKKFAKKFAKKFAKKFAKKFAKKF
>ID04984-ABP_both
AKKKGKCKGPLKLVAKC
>ID04989-ABP_both
AKKVFKRLEKLFSKI
>ID04992-ABP_both
AKKVFKRLGIGAVLKVLTTG
>ID04993-ABP_both
AKKVFKRLGIGAVLKVLTWG
>ID04995-ABP_both
AKKVFKRLGIGAVLWVLTWG
>ID04996-ABP_both
AKKVFKRLRKLFKKI
>ID04997-ABP_both
AKLKAYPLKAKLYP
>ID05002-NO
ALLDKLKSLGKVVGKVAIGVAQHYLNPQ
>ID05003-NO
ALLDKLKSLGKVVGKVAIGVAQHYLNPQQ
>ID05006-ABP_both
ALPLIGRVLSGIL
>ID05007-NO
ALRWPWWPWRRK
>ID05009-ABP_neg
ALWDTLLKKVLKAAAKAALNAVLVGANA
>ID05010-ABP_neg
ALWKTLLKKV
>ID05011-ABP_both
ALWKTLLKKVLKAAA

>ID05012-ABP_both
ALWKTLLKKVLKAAAK
>ID05016-ABP_neg
ALWMTLLKKVLK
>ID05020-ABP_neg
ALWMTLLKKVLKAAAKAALN
>ID05022-NO
ALYKKLFKKLLKR
>ID05023-NO
ALYKKWKNKLLKS
>ID05026-NO
ANLIATKKNGRKLCL
>ID05027-NO
APKEWMAWAREIAAYAKLIAALI
>ID05029-NO
APKEWMEWDREINNYTSLIHSLIKQGI
>ID05030-NO
APLEPEYPGDNATPEQMAQYAAELRRYINMLTRPRY
>ID05031-NO
APLEPVYPGDNATPEQMAQYAADLRRYINMLTRPRY
>ID05032-NO
APPGFTPFR
>ID05034-ABP_both
APRKNVRWCAISLPEWSKCYQWQRRMRKLGAPSITCIRRTS
>ID05036-NO
ARATCYCRTGRCATRESLSGVCEISGRLYRLCCR
>ID05037-NO
ARILMRIRQMMT
>ID05039-NO
ARLDVASEFRKKWNKWALSR
>ID05048-NO
ARYKKFKKKLLKS
>ID05049-NO
ARYRKFKNKILKS
>ID05050-NO
ARYRKFRNKILRS
>ID05051-NO
ASCDKCQLKG
>ID05055-NO
ASRAAGLAARLARLALR
>ID05056-NO
ASTTTNYT
>ID05057-ABP_both
ASVVKTTIKASKKLCKGATLTCGCNITGKK
>ID05058-ABP_both
ASVVNKLTGGVAGLLK
>ID05061-ABP_pos
ATCDLASFSSQWVTPNDSLCAAHCIARRYRGGYCNGKRVCVCR
>ID05062-ABP_pos
ATCDLASFSSQWVTPNDSLCAAHCLVKGYRGGYCKNKICHCRDKF
>ID05064-ABP_pos

ATCDLASIFNVNHALCAAHCIARRYRGGYCNSKAVCVCRN
>ID05065-NO
ATCDLLSGFGVNDSACAAHCILRGNRGGYCNGKKVCVCRN
>ID05066-NO
ATCDLLSGMGVNHSACAAHCVLRGNRGGYCNSKAVCVCR
>ID05071-NO
ATKKNGRKLCLDLQAAL
>ID05072-ABP_pos
ATPTITTSSATCGGIIVAASAAQCPTLACSSRCGKRKK
>ID05076-ABP_pos
AVNIPFKVHFRCKSIFC
>ID05078-NO
AVPLIYNRPGIYAPKRPKGK
>ID05079-ABP_both
AVPLIYNRPGIYVTKRPKGK
>ID05081-NO
AVSEHQLLHDKGKSIQDLRRRFFLHHLIAEIHTAEI
>ID05082-ABP_both
AVVKVPLKKFKSIRE
>ID05083-ABP_both
AVVKVPLKKFKSIRETMKEKGLLEDF
>ID05084-NO
AVVVWLWLWLW
>ID05086-ABP_both
AWASFFKKAAHVAKHVAKAALTHYL
>ID05087-ABP_both
AWASFFKKAAHVGKHVGKAALTHYL
>ID05090-NO
AWLDKLKSIGKVVGKVAIGVAKNLLNPQ
>ID05092-NO
AWLDKLKSLGKVVGKVGLGVVQNYLNPRQ
>ID05096-NO
CATCEQIADSQHRSH
>ID05097-NO
CATCEQIADSQHRSHR
>ID05100-NO
CATCEQIADSQHRSHRQMV
>ID05102-ABP_pos
CEWYNISCQLGNKGQWCTLTKECQRSCK
>ID05103-NO
CGESCVFIPCITTVLGCSCSIKVCYKNGSIP
>ID05115-NO
CGGGGGGGGGKWKAFKKAFKKFAKILACG
>ID05116-NO
CGNLSTCMLGTYTQDFNKFHTFPQTAIGVGAP
>ID05117-NO
CHTNGGYCVRAICPPSARRPGSCFPEKNPCCKYM
>ID05118-NO
CIRLEKARH
>ID05123-NO
CLRWPWWPWRRK

>ID05125-NO
CNDFRSKTC
>ID05126-NO
CREKA
>ID05128-NO
CSCSSLMDKECVYFCHLDIIW
>ID05130-NO
CTVAGWGRVSMRRGT
>ID05131-NO
CVDIHVWDGVC
>ID05132-NO
CVHWQTNPARTSCIGP
>ID05133-NO
CVISAGWDHKVRCKLTGNC
>ID05134-ABP_neg
CVKVKVKVGSGVKVKVKVC
>ID05135-ABP_both
CVVSSGWKWNYKIRCKLTGNC
>ID05136-ABP_both
CVYAYVRVRGVLVRYRRCW
>ID05137-ABP_both
CWFWKWWRRRRR
>ID05138-ABP_neg
CYCRRRFCVC
>ID05141-ABP_both
DAACAAHCLWR
>ID05143-NO
DADSSIEKQVALLKALYGHGQISHKRHKTDSFVGLM
>ID05145-ABP_both
DANVENGEDAEDLTDKFIGLMG
>ID05147-NO
DASISQVNEKINQSLAFIRKSDELLHNVNAGKSTT
>ID05148-ABP_both
DDALRRLLRRLLRRL
>ID05150-ABP_both
DDDDDDD
>ID05151-NO
DDPPLSIDLTFHLLRTLLELARTQSQRERAEQNRIIFDSV
>ID05152-ABP_both
DEDDD
>ID05153-NO
DEFDASISQVNEKINQSLAFIRKSDELLHNVNAGK
>ID05154-NO
DEPKPDQFVGLM
>ID05157-NO
DGPKKKKKKSPSKSSG
>ID05161-NO
DIIKKVASVVG
>ID05166-ABP_neg
DKLIGSCVWGAVNYTSDCNGECLLRGYKGGYCSGFANVNCWCET
>ID05168-NO

DLPKINRKGPRPPGFSPFR
>ID05169-NO
DLWETLKKGGRWILAIPRRIKQGLELTL
>ID05170-NO
DLWETLRRGCRWILAIPRRIR
>ID05171-NO
DLWETLRRGCRWILAIPRRIRQGLELTL
>ID05172-NO
DLWETLRRGGRWILAIPRRIR
>ID05173-NO
DLWETLRRGGRWILAIPRRIRQGLELCL
>ID05174-NO
DLWETLRRIIRWILAIPRRIR
>ID05176-NO
DLWETLRRIIRWILAIPRRIRQGLELTL
>ID05177-NO
DMHDFFVGLM
>ID05179-NO
DPLVFPSDEFDASISQVNEKINQSLAFIRKSDELL
>ID05180-NO
DPPDPDRFYGMM
>ID05182-NO
DPVTCLKSGAICHPVFCPRRYKQIGTCGLPGTKCCK
>ID05183-NO
DPWDWV
>ID05186-NO
DRVYIHPFHL
>ID05187-NO
DSHAKRHHGYKIKFHEKHHSLRGY
>ID05193-NO
DSWMEEVIKLCGRELVRAQIAICGMSTWS
>ID05195-NO
DTLACRQSHGSCSFVACRAPSVDIGTCRGGKLKCCK
>ID05197-NO
DTNFPICIFCCKCCNNSQCGICCKT
>ID05198-NO
DTVVELSEWGVPCATCIL
>ID05199-NO
DVLAGLSSSCCKWGCSKSEISSLC
>ID05200-ABP_both
DVNDLKNLCAKTHNLLPMCAMF
>ID05201-ABP_both
DVNDLKNLCAKTHNLLPMCAMFGKK
>ID05202-NO
DWHLGQGVSIEWRKK
>ID05203-NO
DWLRIIWDWVCSVVSDFK
>ID05204-NO
EAQTRCQVAGWGSQSRSGGR
>ID05205-NO
ECRSTSYAGAVVNDL

>ID05206-ABP_both
ECWMDGHCRLLCKDGEDSIIRCRNRKRCC
>ID05207-ABP_neg
ECYCRRRFCVCVGR
>ID05208-NO
EEHEKYHSNW
>ID05209-NO
EEYTKKIEEYTKKIEEYTKKIEEYTKKIWASLWNWF
>ID05210-NO
EFDASISQVNEKINQSLAFIRKSDELLHNVNAGKS
>ID05211-NO
EFVFLM
>ID05212-NO
EGVNDNEEGFFSA
>ID05213-ABP_both
ELLKAVRLIK
>ID05215-ABP_both
ELLVDLL
>ID05217-NO
ELVNQIIEQLIKKEKVYLAW
>ID05218-ABP_both
EMLKKKKEVKMERKT
>ID05219-NO
EMRLSKFFRDFILQRKK
>ID05220-ABP_both
ENAEEDIVLMENLFCSYIVGSADSFWT
>ID05222-NO
EPRTPWDWV
>ID05223-NO
EQIWNNMTWMEWDREINNYTSLIHSLIEESQNQQEK
>ID05230-NO
ESMNKELKKI
>ID05231-NO
ETWETWWTEYWQATWIPEWE
>ID05232-NO
EVCEKASKTWSGNCGNTGHC
>ID05235-NO
EWDREINNYTSLIHSLIEESQNQQEKNEQELLELDK
>ID05236-NO
EWDREINNYTSLIHSLIEESQNQQEKNEQELLELDKWASLW
>ID05237-ABP_neg
EWESFLETFESAKETVLHTALEAISS
>ID05239-NO
EWRKKRYS
>ID05241-NO
EWVQKYVSDLELSAWKKILK
>ID05242-ABP_neg
FAGLAANFLPTIICKISYKC
>ID05243-NO
FAKKFAKKFAKKFAKKFAKKFAKK
>ID05244-ABP_neg

FAKKLAKKLKKLAKKLAK
>ID05245-ABP_neg
FAKKLAKLAKKLAKLAL
>ID05246-ABP_neg
FAKLLAKALKKLL
>ID05247-ABP_neg
FAKLLAKLAKKLL
>ID05249-ABP_both
FAKWAFKWLKK
>ID05250-ABP_neg
FALALKALKKLLKKLKKLAKKAL
>ID05251-ABP_neg
FALKALKKLKKALKKAL
>ID05252-ABP_neg
FALLGDFFRKSKEKIGKEFKRIVKRIKDFFRNLVPRTES
>ID05255-ABP_both
FAPLIGRVLSGIL
>ID05256-NO
FAVGLRAIKRALKKLRRGVRKVAKDL
>ID05262-NO
FDLGGLIKGVVDLF
>ID05263-ABP_both
FDLLGLVKKVASAL
>ID05264-ABP_both
FDLLGLVKSVVSAL
>ID05266-ABP_both
FDWDSVLKGVEGFVRGYF
>ID05268-ABP_neg
FFDEKCNKLKGTCKNNCGKNEELIALCQKSLKCCRTIQPSGSIID
>ID05269-ABP_both
FFGHLFKLATKIIPSFFRRKNQ
>ID05270-ABP_both
FFGHLFKLATKIIPSLFQRKKE
>ID05273-ABP_both
FFHHIFRAIVHVGKTIHRLVTG
>ID05274-ABP_both
FFHHIFRAIVHVPKTIHRLVTG
>ID05276-ABP_both
FFHHIFRGIVHVGKTIHKLVTGT
>ID05278-ABP_both
FFHHIFRGIVHVPKTIHRLVTG
>ID05284-NO
FFKKWPWWPWRRK
>ID05287-NO
FFPLIAGLAANFLPQILCKIARKC
>ID05289-ABP_both
FFPLIPGVRCKILRTC
>ID05290-ABP_both
FFPSIAGLAAKFLPKIFCSITKRC
>ID05292-ABP_both
FFRHIKSFWKGAKAIFRGARQG

>ID05294-ABP_pos
FFRRFFRR
>ID05295-ABP_pos
FFRRFFRRFFRR
>ID05297-ABP_both
FFSMIPKIAGGIASLVKNLG
>ID05298-ABP_both
FFSMIPKIATGIASLVKDL
>ID05302-NO
FGIPYNPQSQ
>ID05303-ABP_both
FGPVIGLLSGILKSLL
>ID05304-ABP_both
FGVLAKVAAHVVPAIAEHF
>ID05305-NO
FHFFHHFFHFFHHF
>ID05306-ABP_pos
FHPSLWVLIPQYIQLIRKILKS
>ID05307-ABP_both
FIFHIIKGLFHAGKMI
>ID05308-ABP_both
FIGALLGPLLNLLK
>ID05309-ABP_both
FIGALLRPALKLLA
>ID05312-NO
FIHFRIGCQHSRIGI
>ID05314-ABP_both
FIKELLPHLSGIIDSVANAIKG
>ID05315-ABP_both
FIKHFIHRFGGGFKKFWKWFRRF
>ID05317-ABP_both
FIKHFIHRFSATRWRWRWF
>ID05318-NO
FITVH
>ID05319-NO
FKAFKA
>ID05320-NO
FKAFKAFKAFKA
>ID05321-NO
FKAFKAFKAFKAFKAFKA
>ID05322-NO
FKAFKAFKAFKAFKAFKAFKA
>ID05323-NO
FKAFKAFKAFKAFKAFKAFKAFKA
>ID05325-NO
FKAFKAFKAFKAFKAFKAFKAFKAFKAFKA
>ID05328-NO
FKCKKVVISLRRY
>ID05330-ABP_both
FKCRRWQWRMKK
>ID05331-ABP_both

FKCRRWQWRWKKLGAKPVPIIYCNRRTGKCQRM
>ID05332-NO
FKCRRWQWRWKKLGAPSITCVRRAF
>ID05333-ABP_both
FKCWRWQWRWKKLGAKVFKRLEKLFSKI
>ID05334-NO
FKGDSGGPLLCNNVAHGIVSY
>ID05336-ABP_both
FKRLEKLF
>ID05338-ABP_both
FKRLEKLFSKIQNDK
>ID05339-ABP_both
FKRLEKLFSKIWNWK
>ID05342-ABP_both
FKVKFKVKVK
>ID05343-NO
FKVLGSVAKHLLPHVAPVIAEK
>ID05344-ABP_both
FLALIGRVLSGIL
>ID05345-ABP_both
FLFSLIPHAISGLISAFK
>ID05346-ABP_both
FLFSLIPNAISGLLSAFK
>ID05349-ABP_both
FLFSLIPSAISGLISAFKGRRKRDLN
>ID05351-NO
FLGALFKALSHLL
>ID05352-ABP_both
FLGALLGPLMNLLQ
>ID05357-ABP_both
FLGGLIKPWWPWRR
>ID05358-ABP_both
FLGGLIKRPPAMICAVRKKC
>ID05359-ABP_both
FLGGLIKRVPAMICAVRKKC
>ID05360-ABP_both
FLGGLIKWKWPWWPWRR
>ID05362-ABP_both
FLGGLIKWWPWRR
>ID05363-ABP_both
FLGMIPKLIKKLIKAFK
>ID05364-ABP_both
FLGMLLHGVGHAIHGLIHGKQNVE
>ID05366-NO
FLIKQLIKLLTWLFSNCKTLLSKVY
>ID05368-NO
FLIRQLIRLLTWLFPNCRTLLSRVY
>ID05369-NO
FLIRQLIRLLTWLFSNCRTLL
>ID05370-NO
FLIRQLIRLLTWLFSNCRTLLSEVY

>ID05372-NO
FLIRQLIRQLLTWQPILQYILQ
>ID05374-ABP_both
FLKGCWTKWYSLKPKCPF
>ID05376-ABP_neg
FLKLLKKLAAKFLPTIICKISYKC
>ID05379-ABP_both
FLPAIGRVLSGIL
>ID05380-ABP_both
FLPAIIGMAAKVLPAFLCKITKKC
>ID05382-ABP_both
FLPAVLRVAAKVGPAVFCAITQKC
>ID05384-ABP_both
FLPFLAGLFGKIF
>ID05385-ABP_both
FLPFVGNLLNGLL
>ID05388-NO
FLPLIAGLAANFLPKLFCKITKKC
>ID05391-ABP_both
FLPLIGRALSGIL
>ID05392-ABP_both
FLPLIGRVASGIL
>ID05397-ABP_both
FLPLLAGLAAKWF
>ID05400-ABP_both
FLPLLAGLAANFLPTIICKIARKC
>ID05401-ABP_both
FLPLLAGLCKITRKCAANFLPKIF
>ID05404-ABP_both
FLPWFSKFLGRIL
>ID05405-ABP_both
FLSLIPKLVKKIIKAFK
>ID05407-NO
FNAPFDVGIKLSGAQYQQHGRAL
>ID05408-NO
FPLPCAYKGTYC
>ID05409-NO
FPPWE
>ID05410-NO
FPPWF
>ID05412-NO
FPPWM
>ID05413-NO
FPPWVL
>ID05414-NO
FPSDEFDASISQVNEKINQSLAFIRKSDELLHNVN
>ID05415-ABP_both
FPVKLKFPKVKL
>ID05417-NO
FQFVNPSDIVFGS
>ID05419-ABP_both

FRIRVRV
>ID05420-ABP_both
FRKLFRVYSNFLRGKLKL
>ID05422-NO
FRLKFH
>ID05424-NO
FSPQMLQDIIEAATAIL
>ID05425-NO
FSPQMLQDIIEKKTKIL
>ID05428-NO
FVFLM
>ID05429-NO
FVGAALKVLANVLPPVISWIKQ
>ID05430-NO
FVHPM
>ID05431-NO
FVNQHLCGSHLVEALYLVCGERGFFYTPKA
>ID05432-NO
FVNQHLCGSHLVEALYLVCGERGFFYTPKT
>ID05433-NO
FVPIFTYGELQRMQEKERNKGQ
>ID05435-ABP_both
FVQWFSKFLGKIL
>ID05437-ABP_both
FVQWFSRFLGRIL
>ID05440-ABP_both
FWGALAKGALKLIGVGSLFSSFSKKD
>ID05445-ABP_both
FWQKMSFA
>ID05447-ABP_neg
FWRRFWRR
>ID05448-NO
FYDPLVFPSDEFDASISQVNEKINQSLAFIRKSDE
>ID05449-NO
GACRAIRRIPRRIR
>ID05453-ABP_both
GAHDILKYGKPS
>ID05454-ABP_both
GAPKGCWTKSYPPQPCFGKK
>ID05455-NO
GASLSFKILKTVLEALGNV
>ID05456-NO
GASLSFKILKTVLEALGNVKRK
>ID05457-ABP_neg
GATPEDLNQKLS
>ID05459-ABP_both
GCASRCKAKCAGRRCKGWASASFRRRCYCKCFRC
>ID05460-NO
GCKKWFKKAAHVGKNVGKVALNAYL
>ID05461-NO
GCKKYRRFRWKFKGKFWFWG

>ID05463-NO
GDFFRKSKEKIGKEFKRIVQRIKDFLRN
>ID05465-ABP_both
GEGFLGMLLHGVGHAIHGLIHGK
>ID05466-NO
GEILCNLCTGLINTLENLLTTKGAD
>ID05467-ABP_both
GEKLKKIGKKIKNFFQKL
>ID05468-ABP_both
GEKLKKIGQKIKKFFQKL
>ID05470-ABP_both
GFADLFGKAVDFIKS
>ID05471-ABP_both
GFCWNVCVYRNGVRVCHRRCN
>ID05472-ABP_both
GFFALIAKIISSPLFKTL
>ID05473-ABP_both
GFFALIAQIISSPLFQTL
>ID05474-NO
GFFALIPGIE
>ID05475-ABP_both
GFFALIPKIIS
>ID05480-ABP_both
GFFCPYNGYCDRCRKKLRRRGGYCGGRWKLTCICIMN
>ID05487-ABP_both
GFGCPFNARRCHRHCRSIRRRAGYCAGRLRLTCTCVR
>ID05489-NO
GFGCPWNAYECDRHCVSKGYTGGNCRGKIRQTCHCY
>ID05492-NO
GFGMALRLLRRVL
>ID05496-ABP_both
GFGTILKALAKIAGKVVKKLATKPGATYMLKENLK
>ID05497-ABP_both
GFKDLLKGAAKALKKTVLF
>ID05498-ABP_both
GFKDLLKGAAKALVKAVLF
>ID05499-ABP_both
GFKDLLKGAAKALVKTVKF
>ID05502-ABP_neg
GFKLKGKAKISCLPNGQWSNFPPKCIRECAMVSS
>ID05503-ABP_both
GFKMALKLLKKVL
>ID05504-NO
GFLDIIEKIAKSW
>ID05505-NO
GFLDTLKNMALNAAKGAGGSVLKALFCKLFKTC
>ID05507-ABP_both
GFLSILKKVLAKVMAHMK
>ID05508-ABP_both
GFLSILKKVLGKVMAHMK
>ID05511-ABP_both

GFLSILKKVLP
>ID05518-ABP_both
GFMKYIGPLIPHAVKAIKKLI
>ID05522-ABP_both
GFMKYIKPLIPHAVKAISKLI
>ID05524-ABP_both
GFRKFHKFWA
>ID05527-ABP_both
GFVALLKKLPLILKHLP
>ID05530-ABP_neg
GGGGGGHLVA
>ID05531-NO
GGGGSGGGGSGGGGS
>ID05532-NO
GGKFIHSAKKFGKAFVGEIMNS
>ID05533-NO
GGKFLHSAKKFGKAFVGEIMNS
>ID05538-ABP_both
GIADILKYGKPS
>ID05539-ABP_both
GIAEFLNYIKSKA
>ID05540-ABP_both
GIAKFGKAAAHFGKKWVGELMNS
>ID05541-NO
GIAKFLHSAKKFGKAFVAEIMNS
>ID05542-NO
GICICICGRGICYCICGR
>ID05543-NO
GICICICGYGICRCICGR
>ID05544-NO
GICKCICGKGICKCICGR
>ID05545-NO
GICKCICGRRICRCICGR
>ID05546-NO
GICRCICGKGICRCYCGR
>ID05548-NO
GICRCICGKRICRCICGR
>ID05549-NO
GICRCICGRGICRCYCGR
>ID05550-NO
GICRCICGRGYCRCICGR
>ID05551-NO
GICRCICGRKICRCICGR
>ID05553-NO
GICRCICGRRICRCICGK
>ID05554-NO
GICRCICGRYICRCICGR
>ID05556-NO
GICYCICGKGICRCICGR
>ID05558-ABP_both
GIFSKFAGKGLKNLFMKGAKTIGKEVGMDVLRTGIDIAGCKIKGEC

>ID05559-ABP_both
GIFSKFAGRGLKNLFMKGAKKIGKEVGMDVLRTGIDIAGCKIRGEC
>ID05561-ABP_both
GIFSKLAGKKIKNLLISGLKG
>ID05562-ABP_neg
GIGAVLIVLTTGLPALISWIKRKRQQ
>ID05563-ABP_both
GIGAVLKVLALISWIKRKR
>ID05564-NO
GIGAVLKVLSTGLPALISWIKRKRQQ
>ID05565-NO
GIGAVLKVLTTGLPALISWIGGGGGG
>ID05567-NO
GIGAVLKVLTTGLPALISWIKRKRQQC
>ID05573-ABP_both
GIGKFIHAAKKFGKLFIGEIMNS
>ID05575-NO
GIGKFIHSAKKFGKAFVGEIMNSK
>ID05576-ABP_both
GIGKFIHSAKKFGKLFVGEIMNS
>ID05577-ABP_both
GIGKFLHKVGSFIKSWKGEIMNS
>ID05578-ABP_both
GIGKFLHKVKSFGKSWIGEIMNS
>ID05580-ABP_both
GIGKFLHSAKKFGKAWVGEIMNS
>ID05582-ABP_both
GIGKFLHTLKTFGKKWVGEIMNS
>ID05585-NO
GIGKFLREAGKFGKAFVGEIMKP
>ID05586-ABP_both
GIGKLFLHAAKKFAKAFVAEKMNS
>ID05590-ABP_both
GIHAILKYGKPS
>ID05591-ABP_both
GIHDALKYGKPS
>ID05592-ABP_both
GIHDIAKYGKPS
>ID05593-ABP_both
GIHDILAYGKPS
>ID05594-ABP_both
GIHDILKAGKPS
>ID05595-ABP_both
GIHDILKYAKPS
>ID05596-ABP_both
GIHDILKYGAPS
>ID05597-ABP_both
GIHDILKYGKAS
>ID05599-ABP_both
GIHRILKYGKPS
>ID05601-NO

GIINTLQKYYCRVRGAICHPVFCPRRYKQIGTCGLPGTKCCKKP
>ID05602-ABP_both
GIINTLQKYYCRVRGGRCAVLSCLPKEEQICKCSTRGRKCCRRKK
>ID05603-NO
GIKEFKREFQRIKDFLRNLV
>ID05604-NO
GILDAITGLL
>ID05607-ABP_both
GILDTLKQFAKGVGKDLVKGAAQGVLSTVSCKLALTC
>ID05609-ABP_neg
GILKTIKSIASKVANTVQKLKRKAKNAV
>ID05611-ABP_both
GILMDTFKGAAKNVAGFLLDKLKCKISGGC
>ID05612-ABP_neg
GILNTIKSIASKLKRKAK
>ID05615-ABP_both
GIMDTIKGAAKDVAAQLLDKLKCKITKC
>ID05616-ABP_both
GIMDTIKNAAKDVVQSLLNKASCKLAKTC
>ID05620-NO
GIRKWFKKAAHVGKKVGKVALNAYL
>ID05622-NO
GIVEQCCTSICSLYQLENYCN
>ID05623-ABP_both
GIWKSLFTKLLKG
>ID05624-NO
GIWSDLAEIIKKF
>ID05625-NO
GKARGWFY
>ID05627-ABP_both
GKFMSLLKHILK
>ID05629-NO
GKGDRRTRRGKIWRGTYGKYRPRKKK
>ID05632-ABP_both
GKKLLKKLKKLLKKG
>ID05635-ABP_both
GKKLLKKWKKLWKKW
>ID05638-ABP_both
GKLIKKFGRKAISYAVKKARGKH
>ID05639-NO
GKLKKKWKAAKKFLKKCS
>ID05640-ABP_both
GKMKEYFKKFGASFKRRFANLKKRL
>ID05641-ABP_neg
GKPRPYLPRPTSHPRPIRV
>ID05642-NO
GKPRPYSPIPTSPRPIRY
>ID05643-NO
GKSSGVPPEVFTRFVSSFLPWIRTTMR
>ID05644-NO
GKWKAFKKAAKKFAKKCS

>ID05645-NO
GKWKAFKKAFKKFAKILAG
>ID05646-ABP_both
GKWKKILGHLIR
>ID05650-NO
GKWKLFKKAFKKFLKILAG
>ID05651-ABP_both
GKWKSLLKHILK
>ID05652-ABP_both
GKWLSLLKHILK
>ID05653-ABP_both
GKWMKLLKHILK
>ID05654-ABP_both
GKWMSFLKHILK
>ID05655-ABP_both
GKWMSLLKHIWK
>ID05656-ABP_both
GKWMSLLKHWLK
>ID05657-ABP_both
GKWMSLLKKILK
>ID05658-ABP_both
GKWMSLWKHILK
>ID05659-ABP_both
GKWMTLLKHILK
>ID05660-ABP_both
GKWSKILGHLIR
>ID05661-ABP_both
GKWSKILGKLIR
>ID05662-NO
GLAGAISSVLDKLKQSQLIKNYAKKLGYPR
>ID05663-ABP_both
GLARIVVIRVAR
>ID05664-ABP_both
GLFAVIKKVAAVIKKL
>ID05665-ABP_both
GLFAVIKKVAAVIRRL
>ID05666-ABP_both
GLFAVIKKVAKVIKKL
>ID05668-ABP_both
GLFDAIGNLLGGLGL
>ID05674-ABP_both
GLFDVIKKVAAVIGGL
>ID05677-ABP_both
GLFGKLIKKFLRKAISYAVKKARGKH
>ID05678-ABP_both
GLFGKLIKKKGRKAISYAVKKARGKH
>ID05681-ABP_both
GLFGVLAKVAAKVVPAIAKHF
>ID05682-ABP_both
GLFKVIKKVAKVIKKL
>ID05684-NO

GLFTLIKGAAKLIGRTVAKEAGKTGLELMACKITNQC
>ID05685-ABP_both
GLGDILGLLGL
>ID05686-ABP_both
GLGKFLHSAKRFGKAFVGEAMNS
>ID05688-NO
GLIQTIKEKLKELAGGLVTGIQS
>ID05691-ABP_both
GLKDWWNKHKDKIVEVVKEMGKAGLNAA
>ID05698-ABP_both
GLLALISWIKRKRQQ
>ID05699-NO
GLLDIVTGLLGNLIVDVLKPKTPAS
>ID05700-NO
GLLDMVTGLLGNL
>ID05704-NO
GLLDTLKNMAINAAKDAGVSVLNTLSCKLSKTC
>ID05705-NO
GLLDTLKNMAINAAKGAGVSVLNALSCKLSKTC
>ID05707-ABP_both
GLLDVVGNLLGGLGL
>ID05708-ABP_both
GLLGKILGVEKKVLCGLSGMC
>ID05709-ABP_both
GLLGKLLKIAAKVGKKLL
>ID05710-ABP_both
GLLGKLLKIAAKVGSNLL
>ID05711-ABP_both
GLLGPLLKIAAKVGKKLL
>ID05712-ABP_both
GLLGPLLKIAAKVGKNLL
>ID05713-ABP_both
GLLGPLLKIAAKVGSKLL
>ID05716-NO
GLLQTIKEKLKEFAGGVVTGVQS
>ID05717-NO
GLLQTIKEKLKELATGLVIGVQS
>ID05719-ABP_both
GLLSALRKMIPHILSHIKK
>ID05720-ABP_both
GLLSGILGAGKKIVCGLSGLC
>ID05721-ABP_both
GLLSGILGAGKRIVCGLSGLC
>ID05723-NO
GLLSGILNTAGGLLGNLIGSLSNGES
>ID05728-ABP_both
GLMSVLGHAVGNVLGGLFKPKS
>ID05729-ABP_both
GLPALILWIKRKRQQ
>ID05730-ABP_both
GLPALISLIKRKRQQ

>ID05731-ABP_both
GLPALISSIKRKRQQ
>ID05732-ABP_both
GLPALISWIKRKRQQ
>ID05737-ABP_both
GLPCGESCVFIPCITTVVGCSCKNKVCYNN
>ID05738-NO
GLPKCGETCVGGTCNTPGCTCSWPVCTRN
>ID05740-ABP_both
GLPRKILAAIAKKK
>ID05742-ABP_both
GLPRKILAAIAKKKGKCKGPLKLVAKC
>ID05744-NO
GLPVCGDTCVGGTCNTPGCTCSWPVCTRN
>ID05745-NO
GLPVCGEKCVGGTCNTPGCTCSWPVCTRN
>ID05746-NO
GLPVCGETCVGGTCKTPGCTCSWPVCTRN
>ID05747-NO
GLPVCGETCVGGTCNKPGCTCSWPVCTRN
>ID05749-NO
GLPVCGETCVGGTCNTPGCTCSKPVCTRN
>ID05750-NO
GLPVCGETCVGGTCNTPGCTCSWPKCTRN
>ID05751-NO
GLPVCGETCVGGTCNTPGCTCSWPVCTKN
>ID05752-ABP_both
GLPWILLRWLFFRG
>ID05753-NO
GLRRLLGRLLRRLGRLLLR
>ID05754-NO
GLRSRIWLWVLLMIWQESNRFKRM
>ID05756-NO
GLSKGCFGLKLDRIGSMSGLGC
>ID05758-ABP_both
GLVTGLLKTAGKLLGDLFGSLSG
>ID05759-NO
GLWEKIDKFASII
>ID05761-NO
GLWQLIKDKLKDAATGFVTGIQS
>ID05762-ABP_both
GLWTTIKEGLKKFSLGVLDKIRCKIAGGC
>ID05763-NO
GLYKRLFKKLLKS
>ID05764-ABP_both
GMASKAGAIAGKIAKVALKA
>ID05766-ABP_both
GMASLLAKVLPHVVKLIK
>ID05767-ABP_both
GMATAAGTTLGKLAKFVIGAV
>ID05769-ABP_both

GMWKKILGKLIR
>ID05770-ABP_both
GMWSKILGHLIK
>ID05774-ABP_both
GMWSKLLGHLLR
>ID05775-NO
GNGVQCLAMGWGLLGRNRGI
>ID05776-NO
GNHWAVGHLM
>ID05777-NO
GNLWATGHFM
>ID05780-NO
GPIPWQR
>ID05781-NO
GPIPWQRRI
>ID05787-NO
GRFKRFRKPFKKLFKKIS
>ID05788-NO
GRFKRIRKKLKKLFKKIS
>ID05789-ABP_both
GRIKRIRKKIKKLIKKLS
>ID05790-ABP_both
GRKKRRQRRRGGWMWVTNLRTD
>ID05795-NO
GRLRKKWKAFKKFLKILAC
>ID05796-NO
GRRCCGWGPGRRYCRRWC
>ID05799-ABP_both
GRSPRRRTPSPRRRRSQSPRRRRS
>ID05800-NO
GRVPYPRGGLLKLLKKLLKKLLKL
>ID05804-ABP_both
GSKKPVPIIYCNRRSGKCQRM
>ID05806-ABP_both
GSKRWRKFEKRVKKIFEETKEALPVVQGVVAVATAVGRR
>ID05807-ABP_both
GSKRWRKFEKRVKKVFEHTKEALPVIQGVATVVGAVGRR
>ID05810-ABP_both
GSPALISWIKRKRQQ
>ID05811-NO
GSSFLSPEHQRVQQRKESKKPPAKLQPR
>ID05812-NO
GSSKSPSKDKDKDPGDC
>ID05813-NO
GSSKSPSKKKKKKPGD
>ID05815-ABP_both
GSVIKCGESCLLGKCYTPGCTCSRPICKKN
>ID05817-NO
GSYDPRRQICVGDRRERKAA
>ID05819-ABP_both
GVAKFGKAAAHFGKGWIKEMLNS

>ID05820-NO
GVARAVARRGVARAVARR
>ID05821-NO
GVARCVARRGVARCVARR
>ID05822-NO
GVARCVCRRGVCRCVARR
>ID05823-NO
GVCRAVARRGVARAVCRR
>ID05825-NO
GVCRCICGRGVCRCICGR
>ID05829-ABP_both
GVFDIIKGAGKQLIAHAMGKIAEKVGLNKDGN
>ID05832-NO
GVLGTVKNLLIGAGKSAAQSVLKTLSCKLFNDC
>ID05834-ABP_both
GVRNSQSCRRNKGICVPIRCPGSMRQIGTCLGAQVKCCRRK
>ID05835-ABP_both
GVVDILKGAAKDIAGHLASKVM
>ID05836-NO
GVVDTLKNLLMGLL
>ID05837-ABP_both
GVVKVSRLKGESLRARL
>ID05839-NO
GWASKIAQTLGKMAKVGLQELIQPK
>ID05840-NO
GWFDIIKKIASEL
>ID05841-ABP_both
GWFDVVKHIAKRF
>ID05842-NO
GWFYRHHY
>ID05843-ABP_both
GWGSFFKKAAAVGKAVGKAALTAYL
>ID05847-NO
GWGSFFKKAAHVGKHVGKA
>ID05849-NO
GWKDWFRKAKKVGKTVGGLALNHYL
>ID05851-NO
GWLKKIESIIDAF
>ID05852-ABP_both
GWLRKLGAVLKVLTT
>ID05853-NO
GWLRRIGRRIERVGQHKLKKALRALARHWK
>ID05854-ABP_neg
GWMSKIASGIGTFLSGIQQ
>ID05855-NO
GWRKWIKKATHVGKHIGKAALDAYI
>ID05856-NO
GWTLNSAGYLLGKINLKALAALAKKIL
>ID05863-ABP_both
GYFPGRPPFPRPFPRPPSRPFPRPPFPGPFPRPYPWR
>ID05864-NO

GYIEAEVI
>ID05865-NO
GYSAGERIVD
>ID05866-NO
HADGSFSDEMNTILDNLAARDFINWLIQTKITD
>ID05867-NO
HAEGTFTSDVSSYLEGQAAKEFIAWLVKGR
>ID05870-NO
HAKFWW
>ID05871-NO
HAQYNQR
>ID05872-NO
HCAFWW
>ID05873-NO
HCKAWW
>ID05874-NO
HCKFAW
>ID05875-NO
HCKFWA
>ID05876-NO
HCKFWF
>ID05877-NO
HCKFWI
>ID05878-NO
HCKFWW
>ID05880-ABP_both
HFKGTLVNLAKKIL
>ID05882-ABP_both
HFLKTLVNLAKKIL
>ID05885-NO
HGQVDCSPGIWQLDCTH
>ID05887-NO
HHQYNQR
>ID05889-NO
HISHISMCRWCCNCCKAKGCGFCCKF
>ID05890-NO
HKLSQFRATIRFGLVVSKAVGNAVTRHRVSRQLRHFHVVELRADVQAALD
>ID05893-NO
HPAFDRK
>ID05894-NO
HPAYDDK
>ID05895-NO
HPAYNAK
>ID05896-NO
HPAYNPK
>ID05897-NO
HPAYNPR
>ID05898-NO
HPAYNQR
>ID05899-NO
HPDYNAT

>ID05900-NO
HPDYNPD
>ID05901-NO
HPDYNPK
>ID05902-NO
HPDYNQR
>ID05903-NO
HPEYNQR
>ID05904-NO
HPLYNQR
>ID05905-NO
HPNYNQR
>ID05906-NO
HPQANQR
>ID05908-NO
HPQKNTY
>ID05909-NO
HPQYAQR
>ID05910-NO
HPQYNAR
>ID05911-NO
HPQYNPK
>ID05912-NO
HPQYNPQ
>ID05913-NO
HPQYNPR
>ID05914-NO
HPQYNQ
>ID05915-NO
HPQYNQA
>ID05916-NO
HPQYNQRTIQNDIMLLQLSR
>ID05918-NO
HRALMRIRQMMT
>ID05919-NO
HRIAMRIRQMMT
>ID05922-NO
HRILARIRQMMT
>ID05923-NO
HRILMRARQMMT
>ID05924-NO
HRILMRIAQMMT
>ID05926-NO
HRILMRIRAMMT
>ID05927-NO
HRILMRIRQAMT
>ID05928-NO
HRILMRIRQMAT
>ID05931-NO
HSDGIFTDSYSRYRKQMAVKKYLAAVL
>ID05932-NO

HSDGIFTDSYSRYRKQMAVKKYLAAVLGKRYKQRVKNK
>ID05933-NO
HSDGTFTSELSRLRDSARLQRLLQGLV
>ID05935-NO
HTDNGSNF
>ID05938-ABP_both
IAKVALKAL
>ID05939-NO
IARWPWWPWRRK
>ID05941-NO
ICERRSKTWTGFCGNTRGCDSQCKRWERASHGACHAQFPGFACFCYFNC
>ID05943-NO
IEAYFERIGY
>ID05944-NO
IEDIAVNSKYQGQGLGKLLIPRT
>ID05945-NO
IEDISVAKSEQGKKLGYYLV
>ID05947-ABP_both
IFGLLLHGAIHVGKLIHGLVRRH
>ID05949-ABP_neg
IFWRRIVIVKKF
>ID05950-NO
IGEDVYTPGISGDSLR
>ID05952-NO
IGKFLHSAKKFAKAFAFVAEIMNS
>ID05953-NO
IHAEIKNSLKIDNLDVNRCIEAL
>ID05954-NO
IIGGH
>ID05955-NO
IIGGRESRPHSRPYMAYLQI
>ID05956-NO
IIGGV
>ID05958-ABP_both
IIGPVLGLVGKALGGLL
>ID05959-NO
IINFYDPLVFPSDEFDASISQVNEKINQSLAFIRK
>ID05962-ABP_both
IISTIGKLVKWIIKTV
>ID05963-ABP_both
IKFEPPLPPKKAHKKFWEDDGIYYPPNHNFP
>ID05964-ABP_both
IKIKIKIK
>ID05965-NO
IKISGKWKAQKRFLKMSGC
>ID05968-ABP_both
IKKILSKIKKLLK
>ID05969-NO
IKKWPWWPWRRK
>ID05970-NO
IKQEF

>ID05973-NO
ILAWPWWPWRRK
>ID05974-ABP_both
ILGKIWEGIKS
>ID05975-ABP_both
ILGKLLKTAAGLLSNL
>ID05976-ABP_both
ILGKLLKTAAKLLSNL
>ID05977-ABP_both
ILGKLLSTAAGLLKNL
>ID05978-ABP_both
ILGKLLSTAAGLLSKL
>ID05979-ABP_both
ILGKLLSTAAKLLSKL
>ID05981-NO
ILGKLLSTAWGLLSKL
>ID05983-NO
ILGKLLSTAWKLLSNL
>ID05987-NO
ILGPVLGLVSRTLRRVLGIL
>ID05988-NO
ILKKFPFFPFKKK
>ID05989-NO
ILKKFPFFPFRRK
>ID05991-NO
ILKKFPWFPWRRK
>ID05992-NO
ILKKIPIIPIRRK
>ID05994-NO
ILKKPWWPWRRK
>ID05995-NO
ILKKWAWWPWRRK
>ID05996-NO
ILKKWPW
>ID05997-NO
ILKKWPWPWRRK
>ID05998-NO
ILKKWPWWAWRRK
>ID05999-NO
ILKKWPWWPRRK
>ID06002-NO
ILKKWPWWPWKRR
>ID06003-NO
ILKKWPWWPWKWKK
>ID06004-NO
ILKKWPWWPWPPFFRRK
>ID06005-NO
ILKKWPWWPWPPRRK
>ID06008-NO
ILKKWPWWPWRRIMILKKAGS
>ID06010-NO

ILKKWPWWPWRRKMILKKAGS
>ID06012-NO
ILKKWPWWPWRRWWK
>ID06013-NO
ILKKWPWWPWRWWRR
>ID06015-NO
ILKKWPWWRWRR
>ID06017-NO
ILKKWPWWWRK
>ID06019-NO
ILKKWVWWPWRRK
>ID06020-NO
ILKKWVWWVWRRK
>ID06021-NO
ILKKWWWPWRK
>ID06023-NO
ILKKYPWYPWRRK
>ID06024-NO
ILKKYPYYPYRRK
>ID06025-NO
ILKPWKWPWWPWRR
>ID06026-NO
ILKPWKWPWWPWRRKK
>ID06027-NO
ILKWPWWPWRK
>ID06031-ABP_both
ILPKKKPWWPWRR
>ID06033-ABP_both
ILPLKLPLLPLRR
>ID06034-ABP_both
ILPLKLPLLPWRR
>ID06035-ABP_both
ILPLKLPWLPLRR
>ID06039-ABP_both
ILPWKAPAAPARR
>ID06040-ABP_both
ILPWKKPKLPWRR
>ID06041-NO
ILPWKKWPWWRWRR
>ID06043-NO
ILPWKWFFPPWPWRR
>ID06044-ABP_both
ILPWKWKFFPWRR
>ID06045-ABP_both
ILPWKWPAAPARR
>ID06046-ABP_neg
ILPWKWPFFPWRR
>ID06047-ABP_both
ILPWKWPKLPWRR
>ID06048-ABP_both
ILPWKWPLLPWRR

>ID06049-NO
ILPWKWPPWPPWPWRR
>ID06050-ABP_both
ILPWKWPWAPARR
>ID06051-ABP_both
ILPWKWPWLPKRR
>ID06053-ABP_both
ILPWKWPWLPWKK
>ID06055-NO
ILPWKWPWRR
>ID06056-ABP_both
ILPWKWPWWKWRR
>ID06057-NO
ILPWKWPWWPWKKWK
>ID06058-NO
ILPWKWPWWPWPP
>ID06060-NO
ILPWKWPWWPWRRWR
>ID06061-NO
ILPWKWPWWPWWKKPWRR
>ID06062-NO
ILPWKWPWWPWWPWRR
>ID06063-NO
ILPWKWPWYVRR
>ID06064-ABP_both
ILPWKWPYLPWRR
>ID06065-ABP_both
ILPWKWRWWKWRR
>ID06066-ABP_both
ILPYKYPYYPYRR
>ID06067-NO
ILRAPWWPWRRK
>ID06068-NO
ILRRWPWWPWRK
>ID06069-NO
ILRRWPWWPWRRK
>ID06071-NO
ILRRWVWWVWRRK
>ID06072-NO
ILRWAWWPWRRK
>ID06073-ABP_both
ILRWKWRWWRWRR
>ID06074-NO
ILRWPAWPWRRK
>ID06075-NO
ILRWPWAPWRRK
>ID06076-NO
ILRWPWRRWPWRRK
>ID06077-NO
ILRWPWWAWRRK
>ID06078-NO

ILRWPWWPARRK
>ID06082-NO
ILRWPWWPWRRKDMILKKAGS
>ID06083-NO
ILRWPWWPWRRKILMRWPWWPWRRKMAA
>ID06084-NO
ILRWPWWPWRRKIMILKKAGS
>ID06088-NO
ILRWVWWVWRRK
>ID06089-NO
ILRWWVWWVWWRRK
>ID06090-NO
ILRYVYYVYRRK
>ID06092-NO
ILWPWWPWRRK
>ID06094-NO
INFYDPLVFPSDEFDASISQVNEKINQSLAFIRKS
>ID06095-NO
INLKAIAALVKKVL
>ID06096-ABP_both
INLKGLIKKVASLLT
>ID06098-ABP_pos
INTWNTTATSTSIIISETFGNKGKVCTYTVECVNNCRG
>ID06099-NO
INWDILIDTIKDKL
>ID06100-NO
INWKAIIEAAKQAL
>ID06102-ABP_both
IPHAVKAISDLI
>ID06103-NO
IQAQPDQSESELVNQIIEQL
>ID06104-NO
IQRVAQKLKKALRALARHWKRAL
>ID06105-NO
IQRVAQKLKKALRALARHWKRALAGPGVTIGIAHAKSQLW
>ID06107-NO
IRALQRAVRHPRAIRRIYRGWKKAIRAGPGVTIGIAHAKSQLW
>ID06108-ABP_both
IRIKIRIK
>ID06109-NO
IRILQQLLFIHFRIG
>ID06111-ABP_both
IRVKIRVKIRVK
>ID06114-NO
ISSEVHIPLGDARLV
>ID06115-ABP_both
ISSPLFKTLLSAVGSALSSSGGQE
>ID06119-NO
ITFEDLLDYYGP
>ID06120-NO
ITLNNSVALDPIDISIELNKAKSDLEESKEWIRR

>ID06121-NO
ITLNNSVALDPIDISIELNKAKSDLEESKEWIRRS
>ID06122-ABP_pos
ITSFSLCTPGCAKTGSFNSYCC
>ID06123-NO
ITTYWGLHTGERDWHL
>ID06125-NO
IVGGR
>ID06126-NO
IVLSLDVPIGLLQILLEQARARAAREQATTNARILARVGHC
>ID06128-NO
IVPWKWTLWPWRR
>ID06129-ABP_neg
IVRVAVALRRIR
>ID06130-ABP_neg
IVWKIKRWWVGR
>ID06133-NO
IWNNMTWMEWDREINNYTSLIHSLIEESQNQQEKNE
>ID06134-ABP_both
IWRIFR
>ID06137-NO
IYEPEIA
>ID06138-NO
IYWNVSGW
>ID06139-ABP_both
KAAAKWAAKAAK
>ID06140-ABP_both
KAACAAHCLWR
>ID06141-ABP_both
KAACAAKCLWR
>ID06142-NO
KAAKKAAKKAAKKAAKKAAKKAAKKAAK
>ID06144-NO
KAIQTAQGVVAVAPGAKIIGDRINQGVKEIKKFLKWK
>ID06146-NO
KAVAAKKSPKKAKKPAT
>ID06148-ABP_both
KCFQWQRNMRKVR
>ID06149-NO
KCLHKRCR
>ID06152-NO
KCQLKGEAMHGQVDC
>ID06153-ABP_pos
KCSWWNASCHLGNNGKICTVSHECAAGCNL
>ID06157-NO
KFAFKFAFKFAFKFAFKFAFKFAFKFAF
>ID06159-NO
KFAKKAAKKFAKKAAK
>ID06160-NO
KFAKKFAK
>ID06161-NO

KFAKKFAKKAAKKAAK
>ID06162-NO
KFAKKFAKKFAK
>ID06164-NO
KFAKKFAKKFAKKFAK
>ID06165-NO
KFAKKFAKKFAKKFAKKFAK
>ID06167-NO
KFAKKFAKKFAKKFAKKFAKKFAKKFAK
>ID06168-NO
KFAKKFAKKFAKKFAKKFAKKFAKKFAKKFAK
>ID06169-NO
KFAKKFAKKFAKKFAKKFAKKFAKKFAKKFAKKFAKKFAKKFAKKFAK
>ID06173-NO
KFDKSKLKKTETQEKNPL
>ID06175-ABP_neg
KFFKFFKFF
>ID06176-NO
KFFKKFFKKFFKKFFKKFFKKFFKKFFK
>ID06177-NO
KFKHYFFWKYK
>ID06178-NO
KFKKFKKFK
>ID06179-NO
KFKKFKKFKKFK
>ID06180-NO
KFKKFKKFKKFKKFK
>ID06181-NO
KFKKFKKFKKFKKFKKFK
>ID06182-NO
KFKKFKKFKKFKKFKKFKKFK
>ID06183-NO
KFKKFKKFKKFKKFKKFKKFKKFK
>ID06185-NO
KFKKFKKFKKFKKFKKFKKFKKFKKFKKFK
>ID06186-NO
KFKKFKKFKKFKKFKKFKKFKKFKKFKKFKKFKKFK
>ID06187-NO
KFKKFKKFKKFKKFKKFKKFKKFKKFKKFKKFKKFKKFKKFKKFKKFKKFK
>ID06191-ABP_both
KFLGTLVNLAKKIL
>ID06192-NO
KFRRQRPRLSHKGPMPF
>ID06193-NO
KGEAMHGQVDCSPGIWQLDC
>ID06194-ABP_both
KGGGKWGGKGGK
>ID06196-NO
KGKKGKKGKKGKKGKKGKKGK
>ID06197-NO
KGKKGKKGKKGKKGKKGKKGKKGKKGKKGKKGK

>ID06198-NO
KGKKGKKGKKGKKGKKGKKGKKGKKGKKGKKGKKGKKGKKGKKGK
>ID06200-NO
KGLSGPGGWWGFV
>ID06201-NO
KGYFYFLFKFK
>ID06202-NO
KIAGYGLKGLAVIIKICIKGLNLIFEIIK
>ID06205-NO
KILEPFRKQNPDIVIYQYMD
>ID06206-ABP_pos
KILRGVAKKIMRTFLRRISKDILTGKK
>ID06207-ABP_both
KILRGVSKKIMRTFLRRILTGKK
>ID06208-ABP_both
KILRGVSKKIMRTFLRRISKDILTGKK
>ID06212-ABP_both
KIWWWWRKR
>ID06213-NO
KKAAHVGKHVGKAALTHYL
>ID06214-NO
KKAKKKAKKKAKKKAKKKAKKKAKKKAK
>ID06215-ABP_both
KKALLAHALHLLALLALHLAHALKKA
>ID06217-ABP_both
KKFPWWWPFKK
>ID06221-NO
KKIEKAIKHIPKKIKAGPGVTIGIAHAKSQLW
>ID06222-NO
KKIEKAIKHIPKKIKLKKALRALARHWK
>ID06223-ABP_both
KKKFPWWWPFKKK
>ID06225-ABP_neg
KKKKKKAAFAAWAAFDA
>ID06228-ABP_neg
KKKKKKAAFALWLAFAA
>ID06229-ABP_neg
KKKKKKAAFALWLAFLA
>ID06230-ABP_neg
KKKKKKAAFLAWALFAA
>ID06231-ABP_neg
KKKKKKALFAAWAAFLA
>ID06233-ABP_neg
KKKKKKALFALWLAFLA
>ID06234-ABP_both
KKKKKKKKKKGIGKFLHSAKKFGKAFVGEIMNS
>ID06236-ABP_both
KKKKKKKKKKKKKKKKKKKKGIGKFLHSAKKFGKAFVGEIMNS
>ID06237-ABP_neg
KKKKKKLLFAAWAAFAA
>ID06241-ABP_both

KKLGAPSITCVRRAFA
>ID06242-ABP_both
KKLKKLKKKWKKLKKKLK
>ID06243-ABP_both
KKLKKLLKKWKKLLKKLK
>ID06244-ABP_both
KKLLKKLKKLLK
>ID06246-ABP_both
KKLLKLLLKLLK
>ID06247-ABP_both
KKLLKWKLKLKK
>ID06248-ABP_both
KKLLPIVANLLKSLL
>ID06249-NO
KKSFFKKLTSVASSVLS
>ID06251-ABP_both
KKVADKVLLLKQLRIMRL
>ID06252-ABP_both
KKVADKVLLLKQLRIMRLLTRL
>ID06253-ABP_both
KKVVFKFKFK
>ID06254-ABP_both
KKVVFKVKFKK
>ID06255-ABP_both
KKVVFWVKFK
>ID06256-ABP_both
KKWLKLKLKLKK
>ID06258-NO
KKWWRRALQALKNGLPALIS
>ID06259-NO
KKWWRRALQALKNGPALSNV
>ID06261-NO
KKWWRRVLKGLSSGPALSNV
>ID06262-NO
KKWWRRVLSGLKTAGPAIQSVLNK
>ID06263-NO
KKWWRRVLSGLKTGPALSNV
>ID06265-NO
KLAGRW
>ID06267-NO
KLAKLAKKLAKLAKGKRKKKGKLGKKRDP
>ID06268-NO
KLAKLAKKLAKLAKKHLKKHLKKHLK
>ID06269-ABP_both
KLALKLALKAWKAALKLA
>ID06270-NO
KLCQRP
>ID06271-NO
KLCQRPSGT
>ID06272-NO
KLFKKIGIGAVLKVLKVLTTGLPALKLTLK

>ID06273-ABP_both
KLFKRHLKWKII
>ID06274-NO
KLIRKLIRWLRRKIRALQRAVAGPGVTIGIAHAKSQLW
>ID06278-NO
KLKKALRALARHWKAGPGVTIGIAHAKSQLW
>ID06279-NO
KLKKALRALARHWKGWLRRIGRRIERVGQH
>ID06280-NO
KLKKALRWLARHAK
>ID06283-NO
KLKLKLKLK
>ID06286-ABP_both
KLKLLKLLKLLKLLK
>ID06289-ABP_both
KLLKKLLKLWKKLLKKLK
>ID06290-ABP_both
KLLKKPLKLKL
>ID06291-NO
KLLKLLLKLLLKLLK
>ID06292-ABP_both
KLLKLLLKLWKKLLKLLK
>ID06293-ABP_both
KLLKLLLKLWLKLLKLLL
>ID06294-ABP_both
KLLLKLKLKLLK
>ID06295-ABP_both
KLLLKLKLKLWK
>ID06296-NO
KLLLKLLKKLLKLLKKK
>ID06298-ABP_both
KLLLKLLLKLLK
>ID06299-ABP_both
KLLLKWKLKLLK
>ID06300-ABP_both
KLLLKWLLKLLK
>ID06301-ABP_both
KLLLLLKLLLWK
>ID06302-ABP_both
KLLLLWKLLLLK
>ID06303-NO
KLWKLFKKIGIGAVLKVLKVLTTGLPALKLTLK
>ID06304-ABP_neg
KLWWMIRRW
>ID06305-NO
KLYKKWKKKLLKLK
>ID06306-NO
KLYKKWKNKLKRSLKRLG
>ID06307-NO
KLYRKFKNKLLKLK
>ID06310-NO

KNLRRAIRKIIHIIKKYG
>ID06311-NO
KNLRRDIRKIIHIIKKYG
>ID06312-NO
KNLRREIRKIIHIIKKYG
>ID06314-NO
KNLRRIARKIIHIIKKYG
>ID06315-NO
KNLRRIDRKIIHIIKKYG
>ID06318-NO
KNLRRIIRKAIHIIKKYG
>ID06319-NO
KNLRRIIRKDIHIIKKYG
>ID06320-NO
KNLRRIIRKEIHIIKKYG
>ID06323-NO
KNLRRIIRKIAHIIKKYG
>ID06324-NO
KNLRRIIRKIDHIIKKYG
>ID06325-NO
KNLRRIIRKIEHIIKKYG
>ID06335-ABP_both
KNWGAILKHIIK
>ID06337-ABP_both
KNWKAILKHIIK
>ID06338-NO
KNWKGIAGMAKKLLGENWELM
>ID06339-NO
KNWKGIAGMAKKLLGENWKLM
>ID06340-NO
KNWKGIAGMAKKLLGKNW
>ID06341-NO
KNWKGIAGMAKKLLGKNWELMEK
>ID06345-NO
KNWKKIAGMAKKLLKKNWKLM
>ID06346-NO
KNWKKILGKIIKVVK
>ID06349-NO
KPFIRKSYKCLHKRCR
>ID06350-NO
KPFVFLM
>ID06352-NO
KPKQIKPPLPSV
>ID06353-ABP_both
KPLLKKLLKKL
>ID06354-NO
KPPWRL
>ID06355-NO
KPQDVCYVAGWGRMAPMGKY
>ID06356-NO
KPWERE

>ID06357-NO
KPWERL
>ID06358-ABP_neg
KQFRIRVRV
>ID06359-NO
KQLIRFLKRLDRNGGGKLLLKLLKKLLKLLKKK
>ID06360-ABP_both
KQLIRFLKRLDRNLWGLA
>ID06365-NO
KRFKKFFKKPK
>ID06366-ABP_both
KRFKKFFMKLKKSVKKRVMKFFKKPMVIGVTFPF
>ID06368-ABP_both
KRFWQLVPLAIKIYRAWKRR
>ID06369-ABP_pos
KRGFGCNGPWNEDDLRCHNHCKSIKGYKGGYCAKGGFVCKCY
>ID06371-NO
KRKWPWWPWRLI
>ID06374-NO
KRLFKELLKSLRKY
>ID06375-NO
KRLFRRLLFSMKKY
>ID06376-NO
KRLFRRWQWRMKKY
>ID06380-NO
KRRWPWWPWKKLI
>ID06381-NO
KRRWVWWVWRLI
>ID06382-ABP_neg
KRWRWIVRNIRR
>ID06384-ABP_both
KRWWRKWWR
>ID06385-ABP_both
KRWWWWRFR
>ID06387-NO
KSCCPSTSARNVYNSCRFAGGSREACAKLSTCKHFDGSCQPPYDH
>ID06389-NO
KSCCPSTTARNIYNSCRFTGASRDKCCKISGCKIVDGKCKPPFIH
>ID06391-NO
KSCCPSTTARNVYNSCRFAGGSRDTCAKLSGCKIVDGNCKPPYVH
>ID06393-NO
KSCCPTTTARNIYNACRFAHGTRERCSKLSGCKIVDGKCKPPYIH
>ID06399-NO
KSLEQIWNNMTWMEWDREINNYTSLIHSLIEESQNQ
>ID06402-NO
KTCENLADKYRGPCFSGCDTHCTTKENAVSGRCRDDFRCWCTKRC
>ID06403-NO
KTCENLADTFRGPCFTDGSCDDHCKNKEHLIKGRCRDDFRCWCTRNC
>ID06406-ABP_both
KVFKRLEKLFS
>ID06407-ABP_both

KVFKRLEKLFSKI
>ID06408-ABP_both
KVFKRLEKLFSKIQNDK
>ID06410-NO
KVIEVVQGACKAIKHIPKKIKQGLEKIL
>ID06411-NO
KVKVKVKVPPTKVKVKVK
>ID06413-NO
KVLTTGLPALISWIKRKRQQ
>ID06415-ABP_both
KVVVKWVVKVVK
>ID06416-ABP_both
KWAKKWKWFAKAAWKWYKK
>ID06417-ABP_both
KWARLWRWFRITRWLWYIK
>ID06418-ABP_both
KWASLWNWFNITNWLWYIK
>ID06419-NO
KWCFRVCYRGICYRRC
>ID06421-ABP_neg
KWKEFLKEFKEAKKEVLHEALKAISE
>ID06423-NO
KWKFKKIGIGAVLKVLKVLTTGLPALKLTLK
>ID06426-NO
KWKKFIKELQKVLAPGGLLSNIVTSL
>ID06428-ABP_both
KWKKFKKIGAVLKKL
>ID06431-ABP_both
KWKKLLKKLLKLL
>ID06432-ABP_both
KWKKLLKKLLKLLKKLL
>ID06434-ABP_both
KWKKLLKKLLKLLKKLLKKLK
>ID06435-ABP_both
KWKKLLKKLLKLLKKLLKKLKKLLK
>ID06438-ABP_both
KWKKLLKKLLPLLKKLLKKLK
>ID06441-ABP_both
KWKKLLKKPLKLKL
>ID06442-ABP_both
KWKKLLKKPLKLLKKLL
>ID06444-ABP_both
KWKKLLKKPLLKK
>ID06445-ABP_both
KWKKLLKKPLLKKLLKKL
>ID06449-ABP_both
KWKKPLLKKLLKKL
>ID06450-NO
KWKLFIKKLTPAVKKVLLTGLPALIS
>ID06457-ABP_both
KWKLFKKIGKVLKKL

>ID06459-ABP_both
KWKLFKKILLKFLHLAKLF
>ID06461-NO
KWKLFKKKGTGAVLTVLTTGLPALIS
>ID06462-NO
KWKLFKKLPLIGRVL
>ID06468-ABP_both
KWKSFAKTFKSAKKTVAHTALKAISS
>ID06469-ABP_neg
KWKSFAKTFKSAKKTVLHTAAKAISS
>ID06470-NO
KWKSFIKK
>ID06471-ABP_neg
KWKSFIKKLPKAAKKVVTTAKKPLIV
>ID06472-ABP_neg
KWKSFIKKLPSAAKKVTTAAKPLTK
>ID06473-ABP_neg
KWKSFIKKLPSAAKKVVTTAKPLALIS
>ID06475-NO
KWKSFIKNLEKVLKKGPILANLVSIV
>ID06476-NO
KWKSFIKNLEKVLKPGGLLSNIVTSL
>ID06477-NO
KWKSFIKNLTKVLKKVVTTALPALIS
>ID06478-ABP_neg
KWKSFKKKLTSKFLHSAKKF
>ID06479-ABP_both
KWKSFLKKLTSAAKKVLTTALKPISS
>ID06480-ABP_both
KWKSFLKTFKAAVKTVLHTALKAISS
>ID06481-ABP_both
KWKSFLKTFKGAVKTVLHTALKAISS
>ID06482-ABP_both
KWKSFLKTFKKAVKTVLHTALKAISS
>ID06485-ABP_both
KWKSFLKTFKSAKKKVLHKALKAISK
>ID06492-ABP_both
KWKSFLKTFKSPARTVLHTALKPISS
>ID06496-ABP_both
KWKSFLKTLKSPVKTVFYTALKPISS
>ID06497-ABP_both
KWKSFLRTFKSPVRTVFHTALKPISS
>ID06498-ABP_both
KWKSFLRTLKSPAKTVFHTALKAISS
>ID06501-ABP_neg
KWKWWKWKK
>ID06502-NO
KWKWWWWWKWK
>ID06503-NO
KWLDAFYKDVAKELEKAF
>ID06505-ABP_both

KWLLLLKLLLLK
>ID06507-NO
KWVREYINSLEMSKKGLAG
>ID06511-NO
KYKETDL
>ID06512-NO
KYKETDLLILFKDDYFAKKNEERK
>ID06513-ABP_neg
KYKKALKKLAKLL
>ID06514-ABP_both
KYPKYP
>ID06517-NO
KYYGNGVTCGLHDCRVDRGKATCGIINNGGMWGDIG
>ID06519-NO
LAHQKPFI
>ID06520-NO
LAKLAVKAIKGAIAGAKSAMG
>ID06521-ABP_pos
LAREYKKIVEKLKRWLRQVLRTLR
>ID06523-ABP_both
LCAIAKKKGKCKGPLKLVCKC
>ID06524-NO
LCDCPNGPWVWVPAFCQAVG
>ID06526-NO
LCQRPSGTW
>ID06529-ABP_neg
LCYCRRRFCVC
>ID06531-ABP_both
LCYCRRRFCVCVGR
>ID06532-ABP_both
LCYTRPRFTVCV
>ID06535-NO
LEDFFVMSDYRGFGIGSEIL
>ID06537-NO
LEQIWNNMTWMEWDREINNYTSLIHSLIEESQNQQE
>ID06542-ABP_both
LFGVLAKVAAHVVPAIAEHF
>ID06544-ABP_pos
LFKEILEKIKAKL
>ID06545-ABP_both
LFRLIKSLIKRLVSAFK
>ID06546-NO
LGDARLVITTYWGLHT
>ID06547-ABP_both
LGGIVSAVKKIVDFLG
>ID06548-NO
LGQGVSIE
>ID06550-NO
LGRVDIHVWDGVYI
>ID06551-NO
LGRVDIHVWDGVYIRGR

>ID06552-NO
LIHSLIEESQNQQEKNEQELLELDKWASLWNWFNIT
>ID06553-ABP_both
LIQRGRFGRFLGRIRRFRPRINFDIRARGSIRLG
>ID06554-NO
LIRELGIRIRRPIHRIARCAG
>ID06555-NO
LIRELGIRIRRPIHRIARCAGQVV
>ID06556-NO
LIRELGIRIRRPIHRIARCAGQVVEIVR
>ID06557-NO
LIRELGQRIRRPIHRIARCAG
>ID06558-NO
LIRELGQRIRRPIHRIARCAGQVV
>ID06559-NO
LIRELGQRIRRPIHRIARCAGQVVEIVR
>ID06560-NO
LIRELGQRIRRPIHRIARCAGQVVRIVR
>ID06561-NO
LIRELGQRIRRPIHRIARCAGRVV
>ID06563-NO
LIRELGQRIRRPIHRIARCAI
>ID06571-NO
LIRELRQRIRRPIHRIARCARQVV
>ID06572-ABP_both
LIRGLFKSFWQVF
>ID06576-ABP_both
LKAKSIVSWAKKVL
>ID06577-ABP_both
LKDVEEAQQKIINIIRRL
>ID06579-ABP_both
LKKLKGRVSRSFLFFVKLRPAKRTLKKRIL
>ID06582-NO
LKKLLKKLLKKLLKKLLKKLLKKL
>ID06583-NO
LKKLLKKLLKKLLKKLLKKLLKKLLKKL
>ID06585-ABP_both
LKKWLK
>ID06586-ABP_both
LKKWLKK
>ID06588-ABP_both
LKLASIVSWAKKVL
>ID06591-NO
LKLKLKLKLK
>ID06594-NO
LKLKLKLKLKLKLKLKLK
>ID06597-NO
LKLKLKLKLKLKLKLKLKLKLKLK
>ID06599-NO
LKLKLKLKLKLKLKLKLKLKLKLKLKLKLKLKLKLKLKLKLKLKLKLK
>ID06601-ABP_both

LKLKLYPLKLKLYP
>ID06603-ABP_both
LKLKSIVSAAKKVL
>ID06604-ABP_both
LKLKSIVSWAKAVL
>ID06605-ABP_both
LKWGAILKHIIK
>ID06607-NO
LLASDEEIQDVSGTWYLKA
>ID06608-NO
LLEYS
>ID06609-NO
LLEYSI
>ID06611-NO
LLFGKIISRLLGN
>ID06612-ABP_neg
LLGDFFRKSKEKIGKEFKRIVQRIK
>ID06614-NO
LLGGLLQSLL
>ID06615-ABP_both
LLIILKKKWKKQAKAKSK
>ID06616-ABP_both
LLIILRRRWRKQAKAKSK
>ID06617-ABP_both
LLIILRRRWRKQARARSK
>ID06618-ABP_both
LLIILRRRWRRQARARSR
>ID06619-ABP_pos
LLKKLLKKLLKK
>ID06620-ABP_pos
LLKKLLKKLLKKLLKK
>ID06625-ABP_both
LLQWLSKLLGRWL
>ID06627-ABP_pos
LLRRLLRR
>ID06628-ABP_pos
LLRRLLRRLLRR
>ID06629-ABP_pos
LLRRLLRRLLRRLLRR
>ID06630-NO
LLSEVYQILQPILQELSATLQRIREVLR
>ID06631-NO
LLSKVYQILQPILQKLSATLQKIKEVLK
>ID06632-NO
LLSRVYQILQPILQRLCATLQRIREVLR
>ID06633-NO
LLSRVYQILQPILQRLSATLQAIREVL
>ID06636-NO
LLTRCNSFLWTLLRILQRILF
>ID06638-NO
LMRIRQMMT

>ID06641-ABP_both
LNWGAALKHAAK
>ID06642-ABP_both
LNWGAFLKHFFK
>ID06643-ABP_both
LNWGAGLKHGGK
>ID06644-ABP_both
LNWGAIKKHIIK
>ID06647-ABP_both
LNWGAILKKIIK
>ID06648-ABP_both
LNWGAKLKHIIK
>ID06649-ABP_both
LNWGALLKHLLK
>ID06650-ABP_both
LNWGAVLKHVVK
>ID06651-ABP_both
LNWGAWLKHWWK
>ID06653-ABP_both
LNWGKILKHIIK
>ID06655-ABP_both
LPKVMAHMK
>ID06656-NO
LPLPAPSFHRTT
>ID06657-NO
LPPWIG
>ID06658-NO
LQDSGLEVNIVTDSQYALGI
>ID06659-NO
LQEVDAGNFIPPPRWLLL
>ID06663-ABP_both
LRFILWWKR
>ID06665-ABP_neg
LRIRWIFKR
>ID06675-NO
LRLRLRLRLRLRLR
>ID06676-NO
LRLRLRLRLRLRLRLRLR
>ID06677-NO
LRLRLRLRLRLRLRLRLRLRLR
>ID06678-NO
LRRGGRWILAIPRAIL
>ID06679-NO
LRRGGRWILAIPREIL
>ID06682-NO
LRWPWWPW
>ID06684-NO
LSELDDRADALQAGASQFETSAAKLKRKYWWKN
>ID06685-NO
LSHSFKGTCLSDTNCANVCHSERFSGGKCRGFRRRCFCTTHC
>ID06686-ABP_both

LSPAAMASLA
>ID06687-ABP_both
LSPAVMASLA
>ID06689-NO
LSSGRPDGFIHVQGHLQEVD
>ID06693-NO
LVRRRSELMGRRNPVCPG
>ID06695-NO
LWELLRRGGRWILAIPRRIRQGLELTL
>ID06696-NO
LWETLGRVGRWVLAIPRRIRQGLELAL
>ID06698-NO
LWETLRRGCRWILAIPRRIRQGLELTL
>ID06700-NO
LWETLRRGGRWILAIPREIL
>ID06706-NO
LWETLRRGGRWILAIPRRIRRQIELTL
>ID06707-NO
LWETLRRIIRWILAIPRRIR
>ID06708-ABP_both
LWKTLLKKVLKAAA
>ID06710-NO
LWRLLRRGGRWILAIPRRIR
>ID06712-NO
LWRTLRRGGRWILAIPRRIRQGLELTL
>ID06713-NO
MAARAAGLAARLAALALR
>ID06714-NO
MAARAAGLAARLAALALRA
>ID06733-ABP_pos
MAIVGTIIKIIKAIIDIFAK
>ID06784-NO
MASRAAGLAARLARLALR
>ID06786-NO
MASRAAGLARRLARLARR
>ID06787-NO
MASRAAGLARRLARLARRA
>ID06793-NO
MCICKNGKPLPGFIGKICRKICMMQQTH
>ID06794-NO
MDFIIDIIKKIVGLFTGK
>ID06804-NO
MEHFPGP
>ID06844-ABP_pos
MGIIAGIIKVIKSLIEQFTGK
>ID06851-NO
MHGQVDCSPGIWQLD
>ID06869-ABP_pos
MKFKFNPTGTIVKKLTQYEIAWFKNKHGYYPWEIPRC
>ID06921-NO
MKSNRQARHILGLDHKISNQRKIVTEGDKSSVVNNPTGRKRPAEK

>ID06931-ABP_both
MKTLVLLSALFLLAFQVQADPIQNTDEETNTEVQPQEEDQA
>ID06981-NO
MPKEKVFLKIEKMGRNIRN
>ID07050-NO
MSDFVNAISEAVKAGLSADWVTMGTSIADALAKGADFILGFFN
>ID07051-NO
MSGIVEAISNAVKSGLDHDWVNMGTSIADVVAKGADFIAGFFS
>ID07076-ABP_neg
MTPLWRVMGNKPFGAYCQDHVECSTGICKGGHCITSQPIKS
>ID07081-NO
MTWEAWDRAIAEYAARIEALIRAAQEQQEKNEAALREL
>ID07082-NO
MTWMAWDRAIANYAALIHALIEAAQNQQEKNEAALLEL
>ID07083-NO
MTWMEWDREINNYTSLIHSLIEESQNQQEKNEQELLEL
>ID07099-NO
MVSRAAGLAARLARLALRA
>ID07104-NO
MWKEFHNVLSSGQLLADKRWARWYNRW
>ID07105-NO
MWKWFHNVLSSGQLLADKWWAWWYNWW
>ID07107-NO
MWKWFHNVLSWWWLLADKRPARDYNRK
>ID07110-NO
NDFRSKT
>ID07111-NO
NEEEKVKWQPDVP
>ID07113-NO
NEPSINFYKRRGA
>ID07116-NO
NKPFVFLM
>ID07119-ABP_both
NLCASLRARHTIPQCKKFGRR
>ID07120-ABP_both
NLVSALIEGRKYLKNVLKKLNRLKEKNKAKNSKENN
>ID07121-ABP_both
NLWAGILKHIIK
>ID07122-NO
NMTWMEWDREINNYTSLIHSLIEESQNQQEKNEQEL
>ID07123-NO
NNMTWMEWDREINNYTSLIHSLIEESQNQQEKNEQE
>ID07124-ABP_both
NPAGCRFCCGCCPNMIGCGVCCRF
>ID07125-NO
NQGRHFCAAALIHARFVMTAASCFQ
>ID07126-NO
NQGRHFCAAALIHARFVMTAASSFQ
>ID07127-NO
NQGRHFCAGALIHARFVMTAASCFQ
>ID07128-NO

NQGRHFCAGALIHARFVMTAASSFQ
>ID07129-NO
NQGRHFCGAALIHARFVMTAASCFQ
>ID07131-NO
NQGRHFCGGALIHARFAMTAASCFQ
>ID07132-NO
NQGRHFCGGALIHARFIMTAASCFQ
>ID07133-NO
NQGRHFCGGALIHARFLMTAASCFQ
>ID07134-NO
NQGRHFCGGALIHARFVMTAAHSFQ
>ID07151-ABP_both
NRLARHFRDIAGRVNQRL
>ID07159-NO
NVGFYEKCGY
>ID07160-NO
NVGFYKKFDY
>ID07161-NO
NVKFYEKCGF
>ID07164-ABP_both
NWRKLYRRK
>ID07166-NO
NYQWVPYQGRVPYPRGGLLKLLKKLLKKLLKL
>ID07167-NO
NYTSLIHSLIEESQNQQEKNEQELLELDKWASLWNW
>ID07168-NO
PAETGQET
>ID07169-NO
PAWRKAFRWAWRMLKKAA
>ID07170-NO
PDAVYLHRIDLGPPISLERLDVGTNLGNAIAKLED
>ID07171-NO
PDIVIYQYMDDLYVGSDLEI
>ID07173-NO
PEVFLM
>ID07174-ABP_neg
PFFWRIRIRR
>ID07175-NO
PFVFLE
>ID07176-NO
PFVFLM
>ID07177-NO
PFVFLR
>ID07178-NO
PFVYLI
>ID07181-NO
PGIWQLDCTHLEGKI
>ID07182-NO
PHGTQCLAMGWGRVGAHPPP
>ID07183-NO
PICKRNGLPVCGETCTLGTCYTQGCTCSW

>ID07184-NO
PICTRNGLPVCGETCFGGTCNTPGCTCTW
>ID07186-ABP_both
PKKKRKV
>ID07187-ABP_both
PKLKTFLSKWIG
>ID07188-ABP_both
PKLLKFLKWIG
>ID07189-ABP_both
PKLLKFLSKWIG
>ID07190-ABP_both
PKLLKKFLKKWIG
>ID07191-ABP_both
PKLLKTFLKWIG
>ID07192-ABP_both
PKLLKTFLSKWIG
>ID07193-ABP_both
PKLLKTFLSKWKKIG
>ID07194-ABP_both
PKVMAHMK
>ID07196-ABP_neg
PLYKKIIKKLLES
>ID07197-NO
PMPVSQECFETLRGHERILSILRHQNLLKELQDLALQGAKERAHQQ
>ID07199-NO
PPDVHTPPHALWRLHLSLRVCLVRMWIH
>ID07200-ABP_both
PPHKKKLAVYPVFLFYLFLSWFSLIV
>ID07201-ABP_both
PPIQNPSMAPPTQNPYGQPMTPPTQNPYGQPMAPP
>ID07202-NO
PRLSHKGPMPF
>ID07203-ABP_both
PRPLPFPRPGPRPI
>ID07206-NO
PSDEFDASISQVNEKINQSLAFIRKSDELLHNVNA
>ID07211-NO
PVPMR
>ID07212-NO
PWWPWRRK
>ID07215-ABP_both
QADFQKVVAGVANALAHRYH
>ID07216-NO
QADPKTFYGLM
>ID07217-NO
QADPNAFYGLM
>ID07218-NO
QADPNKFYGLM
>ID07219-NO
QARATCYCRTGRCATRESLSGVCEISGRLYRLCCR
>ID07223-NO

QDKPFWDPPIYPV
>ID07224-NO
QDKPFWPPPIYIM
>ID07227-NO
QDKPFWSPPIYPV
>ID07228-NO
QEKPYWPPPIYPM
>ID07229-NO
QETAYFLLKLAGR
>ID07231-NO
QEYRGWMDF
>ID07232-NO
QEYTGWMDF
>ID07234-NO
QGRLGTQWAVGHLM
>ID07237-NO
QHWSYGLRPG
>ID07238-NO
QIGTCFGRPVL
>ID07239-NO
QIHLSLCGLCCNCCHNIGCGFCCKF
>ID07241-NO
QKEGLHYTCSSHFPYSQYQF
>ID07249-NO
QKLCQR
>ID07264-NO
QKLCQRPSGTWSGVCGNNNACKNQCIRLEKARHGSCNYVPAHKCICYFPC
>ID07271-NO
QKLCQRSGTWSGVCGNNNACKNQCIRLEKARHGSCNYVFPAHKCICYFPC
>ID07272-NO
QKYYCRVRGGRCAVLSCLPKEEQIGKCSTRGRKCCR
>ID07274-NO
QLLIRMIYKNI
>ID07275-NO
QLLIRMIYKNILFYLVPGPGHGAEPERRNIKYL
>ID07276-ABP_both
QLQGKQVSGEVVQKVLQELIQSVAKP
>ID07277-NO
QLSAMGLYQSLGF
>ID07278-NO
QLWATGHFM
>ID07279-NO
QLYSALANKCCHVGCTKRSLARFC
>ID07282-NO
QNPNRFIGLM
>ID07284-NO
QPHPNEFVGLM
>ID07286-NO
QPNPDEFFGLM
>ID07287-NO
QPNPDEFVGLM

>ID07288-NO
QPNPNEFFGLM
>ID07290-NO
QPTRRPRPGTGPGRRPRPRPRP
>ID07291-NO
QPWLPFR
>ID07292-NO
QQDYGTGWFDF
>ID07294-NO
QQDYTGAHMDF
>ID07295-NO
QQDYTGSHMDF
>ID07296-NO
QQDYTGWFDF
>ID07297-NO
QQDYTGWMDF
>ID07298-NO
QQLLFIHFRIGCQHS
>ID07301-NO
QRAVGWLRRIGRRIERVGQHLRALAGPGVTIGIAHAKSQLW
>ID07302-NO
QRAVKKIEKAIKHIPKKIKIRALAGPGVTIGIAHAKSQLW
>ID07303-NO
QRAVRRIYRAIRHIPRRIRIRALAGPGVTIGIAHAKSQLW
>ID07305-ABP_both
QSHLSMCRYCCNCCRNNKGCGFCCKF
>ID07306-NO
QSPAGQSRCGGFLVREDFVL
>ID07307-NO
QTPQWATGHFM
>ID07308-ABP_both
QTRRLTYKPRTVTYTRGR
>ID07313-NO
RAAPYGVRLCGREFIRAVIFTCGGSRW
>ID07315-ABP_both
RAGLQFPVGKLLKKLLKRLKR
>ID07316-NO
RAGLQFPVGRLLRRLLR
>ID07318-NO
RAIRRAIRGAPRAIL
>ID07319-NO
RAIRRAIRGAPRAILRAIL
>ID07320-ABP_both
RAVAVIIRLRRV
>ID07321-ABP_both
RAYCRRRFCVAR
>ID07323-NO
RCICGLGIC
>ID07325-NO
RCICGLRFC
>ID07326-NO

RCICGLRIC
>ID07327-NO
RCICGLRVC
>ID07328-NO
RCICGRGFC
>ID07329-NO
RCICGRGIC
>ID07331-NO
RCICGRRFC
>ID07332-NO
RCICGRRIC
>ID07333-NO
RCICGRRVC
>ID07334-NO
RCICRLGFC
>ID07335-NO
RCICRLGIC
>ID07336-NO
RCICRLGVC
>ID07337-NO
RCICRLRFC
>ID07338-NO
RCICRLRIC
>ID07339-NO
RCICRLRVC
>ID07340-NO
RCICRRGFC
>ID07341-NO
RCICRRGIC
>ID07342-NO
RCICRRGVC
>ID07343-NO
RCICRRRFC
>ID07344-NO
RCICRRRIC
>ID07345-NO
RCICRRRVC
>ID07346-NO
RCICTLGFC
>ID07347-NO
RCICTLGIC
>ID07348-NO
RCICTLGVC
>ID07349-NO
RCICTLRFC
>ID07350-NO
RCICTLRIC
>ID07351-NO
RCICTLRVC
>ID07352-ABP_pos
RCICTRGFCRCLCRRGVC

>ID07353-NO
RCICTRGIC
>ID07354-NO
RCICTRGVC
>ID07355-NO
RCICTRRFC
>ID07356-NO
RCICTRRIC
>ID07357-NO
RCICTRRVC
>ID07358-NO
RCICVLGFC
>ID07359-NO
RCICVLGVC
>ID07360-NO
RCICVLRFC
>ID07361-NO
RCICVLRVC
>ID07362-NO
RCICVRGIC
>ID07363-NO
RCICVRGVC
>ID07364-NO
RCICVRRIC
>ID07365-NO
RCICVRRVC
>ID07366-NO
RCLCGLGIC
>ID07367-NO
RCLCGLGVC
>ID07369-NO
RCLCGLRVC
>ID07370-NO
RCLCGRGFC
>ID07371-NO
RCLCGRGIC
>ID07372-NO
RCLCGRGVC
>ID07373-NO
RCLCGRRFC
>ID07374-NO
RCLCGRRIC
>ID07375-NO
RCLCGRRVC
>ID07376-NO
RCLCRLGIC
>ID07377-NO
RCLCRLRIC
>ID07378-NO
RCLCRRGFC
>ID07379-NO

RCLCRRRFC
>ID07380-NO
RCLCRRRVC
>ID07381-NO
RCLCTLGIC
>ID07382-NO
RCLCTLGVC
>ID07383-NO
RCLCTLRIC
>ID07385-NO
RCLCTRGFC
>ID07386-NO
RCLCTRGIC
>ID07387-NO
RCLCTRGVC
>ID07388-NO
RCLCTRRFC
>ID07389-NO
RCLCTRRIC
>ID07390-NO
RCLCVLGIC
>ID07392-NO
RCLCVLRIC
>ID07394-NO
RCLCVRGIC
>ID07395-NO
RCLCVRGVC
>ID07396-NO
RCLCVRRIC
>ID07398-ABP_both
RCYARRRFAVCR
>ID07400-ABP_both
RCYCRRRFCVCVGR
>ID07401-ABP_both
RDAKELVELFFEEIRRAL
>ID07403-NO
RERDHELRHRRHHHQ
>ID07405-NO
RFARRFARRFARRFARRFARRFARRFAR
>ID07406-NO
RFARRFARRFARRFARRFARRFARRFARRFAR
>ID07407-NO
RFEKSKIK
>ID07408-NO
RFGRFLRKIRRFRPK
>ID07409-NO
RFGRFLRKIRRFRPKVTITIQ
>ID07410-NO
RFPFHRCGAGPKLTKDLE
>ID07412-ABP_neg
RFWKVRVKYIRF

>ID07413-ABP_both
RGARIVVIRVAR
>ID07415-NO
RGDLLRHVVKILSKYL
>ID07417-ABP_neg
RGGRLAYCRRRFCVAVGR
>ID07420-ABP_neg
RGGRLCYCRRRFCVCE
>ID07421-ABP_neg
RGGRLCYCRRRFCVCI
>ID07422-ABP_both
RGGRLCYCRRRFCVCR
>ID07423-ABP_neg
RGGRLCYCRRRFCVCT
>ID07425-ABP_both
RGGRLCYTRPRFTVCVGR
>ID07426-ABP_both
RGGRLTYTRPRFTVTVGR
>ID07429-NO
RHARHD
>ID07430-NO
RHFCAAALIHARFVMTAASS
>ID07431-NO
RHFCAGALIHARFVMTAASS
>ID07433-NO
RHFCGGALIHARFAMTAASS
>ID07434-NO
RHFCGGALIHARFIMTAASS
>ID07435-NO
RHFCGGALIHARFLMTAASS
>ID07437-NO
RHFCGGALIHARFVMTAAHS
>ID07438-NO
RHFCGGALIHARFVMTAAKC
>ID07439-NO
RHFCGGALIHARFVMTAAKS
>ID07440-NO
RHFCGGALIHARFVMTAARC
>ID07446-NO
RHFSAGALIHARFVMTAASC
>ID07447-NO
RHFSGAALIHARFVMTAASC
>ID07449-NO
RHFSGGALIHARFIMTAASC
>ID07453-NO
RHFSGGALIHARFVMTAARC
>ID07456-NO
RHFSGGALIHARYVMTAASC
>ID07458-NO
RHPQYNQR
>ID07460-NO

RHYSGGALIHARFVMTAASC
>ID07461-NO
RIAGYGLRGLAVIIRCIIRGLNLIFEIIR
>ID07463-NO
RIAGYGLRGLAVIPRRICIRGLNLIFEIIR
>ID07464-ABP_both
RICRIVVIRCIR
>ID07466-NO
RIIEFILNLGRICIRIIVALGRLGYGAIR
>ID07467-ABP_both
RIKRWWWWR
>ID07468-ABP_neg
RIKWIVRFR
>ID07469-ABP_both
RILRGVSRRIMRRILTGRR
>ID07470-NO
RINNIPWSEAMM
>ID07471-ABP_both
RIRFPWPWRWPWWRRVRG
>ID07472-NO
RIRRPIALIWRGGRRLTEWL
>ID07473-NO
RIRRPIHRIARCAGQVVEIVR
>ID07474-NO
RIRRPIHRIARCAGQVVRIVR
>ID07475-NO
RIRRPIHRIARCAGRVVEIVR
>ID07477-NO
RIRRPIHRIIRCIGQVVRIVR
>ID07481-ABP_both
RIWVIRWR
>ID07482-ABP_both
RLARIVKIRVAR
>ID07483-ABP_neg
RLARIVPIRVAR
>ID07484-ABP_both
RLARIVVIRVAG
>ID07487-ABP_both
RLARRVVIRVAR
>ID07489-NO
RLCIPYIIPC
>ID07490-ABP_both
RLCPRVRIRVCR
>ID07491-ABP_both
RLCRIVPVIRVCR
>ID07492-ABP_both
RLCRIVWVIRVCR
>ID07493-NO
RLCRVVIRVCR
>ID07494-ABP_both
RLEKLFSKIQNDK

>ID07496-NO
RLFVWWVFRR
>ID07497-NO
RLGGGWVWWVWRR
>ID07498-ABP_both
RLLLRIGRR
>ID07500-NO
RLLTWLFSNRRTLLSRVYQILQEIL
>ID07501-NO
RLLTWLRRTLLSRVYQILQEIL
>ID07503-ABP_both
RLPWRWPRRPWRR
>ID07504-ABP_both
RLRKAVRLIK
>ID07506-ABP_both
RLRRIVVIRVRR
>ID07507-ABP_both
RLSRIVVIRVCR
>ID07508-NO
RLVERIRQLTASLRQLIPQLIQYVRSLL
>ID07509-NO
RLVERIRQLTASRQLIPQLIQYV
>ID07511-NO
RLVVWVVWRR
>ID07514-NO
RLWVWWVWRR
>ID07517-ABP_both
RLYLRIGRR
>ID07519-ABP_both
RLYRRLYR
>ID07520-ABP_both
RLYRRLYRRLYRRLYR
>ID07521-ABP_both
RLYRRLYRRLYRRLYRRLYRRLYRRLYRRLYR
>ID07522-NO
RMIYKNILFYLVPGPGHGAEPERRNIKYL
>ID07527-NO
RPGGQIAIAIGESIRKKASNELKKATKSLWS
>ID07528-NO
RPGLTLCTVAGWG
>ID07530-NO
RPPGFSPF
>ID07531-NO
RPPGFSPFRIY
>ID07532-NO
RPPGFTPF
>ID07533-NO
RPPGFTPFRIY
>ID07535-NO
RQIIVFMRKKNFVTKILKKQR
>ID07537-NO

RQLLSGIVQQQNNLLRAIEAQQHLLQK
>ID07542-ABP_both
RRCARMYPGSTGYCQGFRCMCDTHIPIRRPPFIMG
>ID07544-ABP_both
RRFPWWWPFRR
>ID07545-ABP_neg
RRGKDSGGPKMGRKDSKGCWRGRPGSGSRPGFGSGIAGASGVNHVGTLPA
>ID07546-NO
RRGLLEVIRCVILLLDRL
>ID07547-NO
RRGLLEVIRCVILLLDRLRHY
>ID07548-NO
RRGLLEVIRTVILALDRL
>ID07549-NO
RRGLLEVIRTVILALDRLRHY
>ID07550-NO
RRGLLEVIRTVILLLDRLRHY
>ID07551-NO
RRGLLEVIRTVILLLRRL
>ID07553-NO
RRGLLEVIRTVILPRRLLDRL
>ID07554-NO
RRGLLRVIRTVILALDIL
>ID07555-NO
RRGLLRVIRTVILLLDRL
>ID07557-ABP_both
RRGWALRLVLAY
>ID07558-ABP_both
RRGWVLALVLRYGRR
>ID07559-ABP_both
RRGWVLALYLRYGRR
>ID07561-ABP_both
RRIPRPILLPWRPPRPIPRPQPQPIPRWL
>ID07562-NO
RRIRHIPRAIRVVQGAC
>ID07563-ABP_both
RRIRPRPPRLPRPRPR
>ID07564-NO
RRIWKPKWRLPKR
>ID07565-NO
RRIYRAIRHIPRRIR
>ID07566-NO
RRIYRAIRHIPRRIRGWLRRIGRRIERVGQH
>ID07568-NO
RRKKA
>ID07569-NO
RRKKAA
>ID07570-NO
RRKKAAV
>ID07571-NO
RRKKAAVA

>ID07572-NO
RRKKAAVAL
>ID07573-NO
RRKKAAVALL
>ID07575-NO
RRKKAAVALLPA
>ID07578-NO
RRKKAAVALLPAVLL
>ID07580-NO
RRKKAAVALLPAVLLAL
>ID07581-NO
RRKKAAVALLPAVLLALL
>ID07582-NO
RRKKAAVALLPAVLLALLA
>ID07583-NO
RRKKAAVALLPAVLLALLAP
>ID07584-NO
RRKKALLAP
>ID07585-NO
RRKKALLPAVLLALLAP
>ID07586-NO
RRKKAP
>ID07587-NO
RRKKAVALLPAVLLALLAP
>ID07588-NO
RRKKLAALPLVLAAPLAVLA
>ID07589-NO
RRKKLAP
>ID07590-NO
RRKKLLALLAP
>ID07591-NO
RRKKLLAP
>ID07592-NO
RRKKLLPAVLLALLAP
>ID07593-NO
RRKKLPAVLLALLAP
>ID07594-NO
RRKKP
>ID07595-NO
RRKKPAVLLALLAP
>ID07597-NO
RRKKRRRRRR
>ID07598-NO
RRKKVALLPAVLLALLAP
>ID07600-ABP_both
RRLCRIVVIRVCRR
>ID07602-NO
RRLDRGGVWNLNVNPGTTGARVWARTK
>ID07604-ABP_both
RRLMAAKAESRK
>ID07607-ABP_both

RRPWRWPRWPWRR
>ID07608-ABP_both
RRPWRWPWWPWRR
>ID07610-NO
RRRFPWWWPFLRRR
>ID07611-ABP_both
RRRLCPIVIRVCRR
>ID07612-ABP_both
RRRRRRRR
>ID07613-NO
RRRRRRRRHPAEPGSTVTTQNTASQTMS
>ID07614-ABP_both
RRRRRRRRRRGIGKFLHSAKKFGKAFVGEIMNS
>ID07615-ABP_both
RRRRRRRRRRR
>ID07616-ABP_both
RRRTPSPRRRRSQSPRRRR
>ID07617-ABP_both
RRSKVRICSRGKNCVSFNDEFIRDHSDGNRFA
>ID07618-ABP_both
RRSKVRICSRGKNCVSRPGGGSFNDEFIRDHSDGNRFA
>ID07620-NO
RRVVRRVRRVVRRVVRVVRRVVRRVRRVVRRVVRVVRRVVRR
>ID07621-NO
RRWCFIVCRRGACYRRCR
>ID07623-NO
RRWCFRVCYRGFCRYFCR
>ID07626-NO
RRWCRRVCYAGFCYRKCR
>ID07628-ABP_both
RRWPLKPKKWPLI
>ID07629-ABP_both
RRWPLKPWKKPLI
>ID07630-ABP_both
RRWPLLPWKWPLI
>ID07631-ABP_both
RRWPWWPWKWPLI
>ID07632-ABP_both
RRWVIWRR
>ID07634-ABP_neg
RRWVVWRIVQRR
>ID07635-ABP_both
RRYHWRIYI
>ID07636-NO
RSGPPGLQGRLQRLLQASGNHAAGILTM
>ID07637-NO
RSGRGECRRQCLRRHEGQPWETQECMRRCRRRGG
>ID07639-NO
RSMRLSFRARGYGFRGPGLQL
>ID07644-NO
RTCESQSHKFKGTCLSDTNCANVCHSERFSGGKCRGFRRRCFCTTHC

>ID07646-NO
RVIEVVQGACRAIEHIPRRIEQGLERIL
>ID07647-NO
RVIEVVQGACRAIEHIPRRIRQGLERIL
>ID07648-NO
RVIEVVQGACRAIRHIPRRIEQGLERIL
>ID07649-NO
RVIEVVQGACRAIRHIPRRIRQGLRRIL
>ID07652-NO
RVIEVVQGACRAIRRIPRRIR
>ID07664-NO
RVRRNRNVNPVALPRAQEGL
>ID07667-NO
RVWCRRRCYRGFCRYFCR
>ID07668-NO
RVWCRYRCYRGFCRRFCR
>ID07669-ABP_both
RWCVYAYVRVAGVLVRYRRCW
>ID07670-ABP_both
RWKIFKKIEKVGRNVRDGIIKAGPAVAVVGQAATVVKG
>ID07672-ABP_both
RWRRKWWWW
>ID07673-NO
RWRSFFKKAAHRGKHVGKRARTHYL
>ID07674-ABP_neg
RWRWWRWRR
>ID07675-ABP_pos
RWSVYAYVRVRGVLVRYRRSW
>ID07676-ABP_both
RWWKIWVIRWWR
>ID07678-NO
RWWWPWRRK
>ID07681-ABP_both
SADLVKKIWDNPAL
>ID07682-NO
SADVAGAVIDGASLSFKILKTVLEALGNVKRK
>ID07683-NO
SAIHPSSILKLEVICIGVLQ
>ID07687-NO
SDRDTVVELSEWGVPCAT
>ID07690-NO
SFFKKAAHVGKHVGKAALTHYL
>ID07693-ABP_both
SFLSTFKKLAIDAAKNAGQSVLHTLTCKLDNQC
>ID07694-NO
SFLTSFKDMAIKVAKDAGVNILNTISCKIFKTC
>ID07696-NO
SGIDQEQNNLTRLIEAQIHELQLTQWKIKQLLARILK
>ID07697-NO
SGIVQQLNNQLRAEEANQHLEQLSVWGSKQNQARRLK
>ID07698-ABP_both

SIGRRGGYCAGIIKQTCTCYR
>ID07703-NO
SKDGKKKKKKSKTK
>ID07710-NO
SLGSFMKGVGKGLATVGKIVADQFGKLLEA
>ID07712-ABP_pos
SLGVTLGAAGVYTATQTIATQIWKCGAVLTTSAECSRTGKSC
>ID07714-NO
SLIHSLIEESQNQQEKNEQELLELDKWASLWNWFNI
>ID07716-ABP_both
SLLSLLRKLIT
>ID07717-NO
SLRRSSCFGGRIDRIGAQSGLGCNSFRY
>ID07718-NO
SLRRSSCFGGRMDRIGAQSGLGCNSFRY
>ID07719-ABP_both
SLSRFLRFLKIVYRRAF
>ID07720-ABP_both
SLSRYAKLANRLA
>ID07726-NO
SNQGGSPLPRSV
>ID07728-NO
SPAIFQSSMTKILEPFRKQN
>ID07729-NO
SPKSHFELPHYPGLLAHQKPFIRKSYKCLHKRCR
>ID07730-ABP_both
SPRRRTPSPRRRRSQSPRRRR
>ID07731-NO
SPSNETPKKKKKRFSFKKSG
>ID07735-NO
SSLLEKGLDGAKKAVGGLGKLGK
>ID07737-NO
SSLLEKGLDGAKKAVGGLGKLGKDAVEDL
>ID07739-ABP_both
SSRRPCRGRSCGPRLRGGYTLIGRPVKNQNRPKYMWV
>ID07740-NO
SSVITSLGAIVSCYGKT
>ID07741-NO
STSYAGAVVNDL
>ID07742-NO
STTVKAASWWA
>ID07743-ABP_both
STVLTSKYR
>ID07744-ABP_both
SVDMVMKGLKIWPL
>ID07745-ABP_pos
SVIGCWTKSIPPRPRCFVK
>ID07746-NO
SVLSTITDMAKAAGRAALNAITGLVNQGEQ
>ID07747-NO
SVQARWEAAFDLDLY

>ID07748-NO
SVSVGMKPSPRP
>ID07750-NO
SWLRDIWDWICEVL
>ID07751-NO
SWLRDVWDWVCTILTDFK
>ID07752-NO
SWLRDVWDWVCTVLSDFK
>ID07754-NO
SWVQEYVYDLEL
>ID07756-NO
SYKCLHKRCR
>ID07759-NO
TAAFLLKLAGRW
>ID07760-NO
TAAHCWGSNINVTLGAHNIQ
>ID07762-NO
TAYALLKLAGRW
>ID07763-NO
TAYFALKLAGRW
>ID07764-NO
TAYFLAKLAGRW
>ID07765-NO
TAYFLL
>ID07766-NO
TAYFLLALAGRW
>ID07767-NO
TAYFLLILAGRW
>ID07768-NO
TAYFLLKAAGRW
>ID07769-NO
TAYFLLKLAARW
>ID07771-NO
TAYFLLKLAGRA
>ID07774-ABP_both
TDHQMAQSACIGVSQDNAYASAIPRDCHGG
>ID07778-NO
TEVSEALGGAGLTGGFYEPL
>ID07780-NO
TGEKVWDRGNVTLLCDCP
>ID07781-NO
TGRAKRR
>ID07783-ABP_both
TKLTEEEKNRLNFLKKISQRYQKFALPQYLK
>ID07784-ABP_both
TLLKKVLKAAAK
>ID07785-ABP_both
TLLKKVLKAAAKAALNAVLVGANA
>ID07786-NO
TLNNSVALDPIDISIELNKAKSDLEESKEWIRRS
>ID07788-NO

TLPCLWPWWPWSI
>ID07798-NO
TSLIHSLIEESQNQQEKNEQELLELDKWASLWNWFN
>ID07800-NO
TTWEAWDRAIAEYAARIEALIRAAQELQEKNEAALREL
>ID07801-NO
TTWEAWDRAIAEYAARIEALIRAAQEQQEKLEAVLREL
>ID07806-NO
TTWEAWDRAIAEYAARIEALIRALQEQQEKNEAILREL
>ID07809-NO
TTWEEWDREINEYTSRIESLIRESQEQQEKNEQELREL
>ID07810-ABP_both
TVVRRRGRSPRRRTPSPRRRR
>ID07811-ABP_both
TVVRRRGRSPRRRTPSPRRRRSQSPRRRR
>ID07812-ABP_both
TVVRRRGRSPRRRTPSPRRRRSQSPRRRRSQSRESQC
>ID07813-NO
TWAGVEAIIRILQQL
>ID07814-NO
TWLRAIWDWVCTALTDFK
>ID07816-NO
TYGDTWAGVEAIIRI
>ID07817-NO
TYICEVEDQKEE
>ID07818-ABP_neg
VAKKLAKLAKKLAKLALAL
>ID07822-ABP_neg
VCYCRRRFCVCVGR
>ID07824-NO
VDIHVWDGVVDIHVWDGV
>ID07825-NO
VDIHVWEGV
>ID07828-ABP_neg
VDKGSYLPRPTSHPRPIRV
>ID07829-NO
VDKPPYLPRPRPPRAIYNA
>ID07830-NO
VEAIIRILQQLLFIH
>ID07831-NO
VEDVVVSDECRGKQLGKLLL
>ID07832-NO
VEIHVWEGV
>ID07833-ABP_both
VEVQVRDKGKGIYGLSPLRQPAP
>ID07834-NO
VFIDILDKMENAIHKAAQAGIG
>ID07840-ABP_neg
VGIGTPISFYGGGAGHVPEYF
>ID07841-NO
VHIPLGDA

>ID07842-NO
VHVASGY
>ID07843-ABP_both
VHVPPICSHRECRK
>ID07844-ABP_both
VIPFVASVAAEMMQHVYCAASKRC
>ID07845-NO
VITIELSNIKENKCNGTDAKVKLIKQELDKYKNAV
>ID07846-ABP_both
VKAKVYPAKVKAYP
>ID07847-ABP_both
VKGSWSKKFEVIA
>ID07850-ABP_both
VKKLVYPLKVKLYP
>ID07851-ABP_both
VKLFVYPLKVKLYP
>ID07852-ABP_both
VKLGVYPLKVKLYP
>ID07853-ABP_both
VKLKVFPLKVKLFP
>ID07854-ABP_both
VKLKYPKVKLYP
>ID07855-ABP_both
VKLLVYPLKVKLYP
>ID07857-ABP_both
VKLYPVKLYP
>ID07861-NO
VKSGHYKGPCYHDENCNGVCRDEGYKSGHCSRWGGACWCDT
>ID07863-ABP_both
VKYPKLYP
>ID07864-ABP_both
VLLVTLTRLHQRGVIYRKWRHFSGRKYR
>ID07865-ABP_both
VLNENLLR
>ID07868-ABP_both
VLSAADKGNVKAAWGKVGGHAAE
>ID07869-ABP_pos
VLSIVACSSGCGSGKTAASCVATCGNKCFTNVGSLC
>ID07872-ABP_both
VNFLLHKIYGNIRYS
>ID07873-ABP_both
VNRKKILGKSIKVVK
>ID07874-ABP_both
VNSKKISGKSIKVSK
>ID07877-NO
VNWKKILGKIKKVVK
>ID07878-ABP_both
VNWKKILGKSIKVSK
>ID07879-NO
VNWKKLLGKLLKVVK
>ID07880-ABP_both

VNWKKSLGKSIKVVK
>ID07881-NO
VNWKKVLGKVVKVVK
>ID07883-NO
VNWRRILGRIIRVVR
>ID07887-NO
VPIYEKKYGQVPMCDAGEQCAVRKGARIGKLCDCPRGTSCNSFLLKCL
>ID07888-NO
VPKCCKPV
>ID07892-NO
VPPIGWF
>ID07893-ABP_neg
VQWRAIRVRVIR
>ID07894-ABP_neg
VQWRIRVRVIKK
>ID07895-ABP_neg
VQWRRIRVWVIR
>ID07896-ABP_pos
VRDICKKEAERQDLSSCENYITQRRGY
>ID07897-NO
VRDQAEHLKT
>ID07899-ABP_neg
VRLRIRVA
>ID07900-ABP_both
VRLRIRVAVIRA
>ID07901-ABP_neg
VRLRIRWWVLRK
>ID07903-ABP_both
VRRFAWWWPFLRR
>ID07904-ABP_both
VRRFPAAAPFLRR
>ID07905-ABP_both
VRRFPWWWAFLRR
>ID07906-ABP_both
VRRFPYYYPFLRR
>ID07907-NO
VRRRRRPR
>ID07909-ABP_both
VRRYPWWWPYLRR
>ID07911-ABP_both
VSCDFEEANEDAVCQEHCLPKGYTYGICVSHTCSCIYIVELIKWYTNTYT
>ID07913-NO
VSIEWRKK
>ID07915-NO
VSRRRRRRGGRRRR
>ID07921-NO
VTNTATIM
>ID07924-ABP_both
VVKCSYRLGSPDSRCN
>ID07925-ABP_both
VVKFSYRKGSPAPQKN

>ID07926-NO
VVQGACRAIRRIPRRIRGLERIL
>ID07927-NO
VVQGICRAIRHIPRRIR
>ID07928-NO
VVQGICRAIRHIPRRIRGLERIL
>ID07930-NO
VVQRACRAIRRIPRRIR
>ID07933-ABP_both
VVYALKRNGRTLYGF
>ID07935-ABP_pos
VWTVWGTIAG
>ID07938-NO
WAGIKQ
>ID07939-ABP_both
WALRLYLVY
>ID07940-NO
WASLKIDNLDV
>ID07941-NO
WASLWNWF
>ID07942-NO
WCFAVCRRGRCRYKCRR
>ID07943-NO
WCFAVCYRGRCRRKCRR
>ID07945-ABP_both
WCPPMIPLCSRF
>ID07946-NO
WDREINNYTSLIHSLIEESQNQQEKNEQELLELDKW
>ID07947-NO
WDRGNVTLLCDCPNGPWVWV
>ID07948-NO
WEAKLAKALAKALAKHLAKALAKALKACEA
>ID07949-NO
WEEWDKKIEEYTKKIEELIKKSEEQQKKNEEELKK
>ID07950-NO
WETLPRRIRGGRLWILAI
>ID07954-ABP_pos
WGNVLL
>ID07955-ABP_neg
WGRKKRRQRRRPPQ
>ID07958-NO
WILAIPRRIRGGRLWETL
>ID07959-NO
WKGPAKLLWK
>ID07960-ABP_both
WKKVFKRLEKLFSKIWNWK
>ID07962-ABP_both
WKWKWK
>ID07964-ABP_pos
WLIING
>ID07965-ABP_pos

WLLING
>ID07966-ABP_pos
WLLINK
>ID07967-ABP_pos
WLLVNK
>ID07968-ABP_pos
WLVING
>ID07969-NO
WMEWDREIEEYTKKIEEYTKKIEEYTKKIEEYTKKI
>ID07970-NO
WMEWDREIEEYTKKIEEYTKKIEEYTKKIWASLWNWF
>ID07973-NO
WNALKIDNLDV
>ID07976-NO
WNSAKIDNLDV
>ID07977-NO
WNSLKADNLDV
>ID07979-NO
WNSLKIANLDV
>ID07980-NO
WNSLKIDALDV
>ID07981-NO
WNSLKIDNADV
>ID07982-NO
WNSLKIDNLDA
>ID07984-NO
WPWWPWRRK
>ID07985-NO
WQCLTLTHRG
>ID07986-NO
WQCLTLTHRGFVLLTITVLR
>ID07989-ABP_both
WRRRRRRR
>ID07990-NO
WRWWKPKWRWPKW
>ID07991-NO
WRWWKVAWRWVKW
>ID07992-NO
WRWWKVVWRWVKW
>ID07994-ABP_pos
WTTIVKVSKAVCKTGTCICTTSCSNCK
>ID07995-ABP_both
WVLVLRLGY
>ID07996-NO
WVNQLAVLGLPAVDAAVA
>ID07997-NO
WVRLWWRRVW
>ID08001-ABP_both
WWWLRKIW
>ID08030-NO
YADAIFTNSYRKVLGQLSARKLLQDIMSRQQGESNQERGARARL

>ID08031-NO
YAEGTFISDYSIAMDKIHQQDFVNWLLAQKGKKNDWKHNITQ
>ID08032-NO
YAELR
>ID08033-NO
YAERLCTCSIKAEV
>ID08035-NO
YALLIRMIYKNI
>ID08038-ABP_pos
YDTGIQGWTCGSRGLCRKHCYAQEHTVGYHGCPRRYRCCALRF
>ID08039-NO
YFLLKL
>ID08040-ABP_both
YGAAKKAAKAAKKAAKAA
>ID08041-ABP_both
YGAKAKAAKAAKAKAAKA
>ID08043-NO
YGGFLRRIRPKLK
>ID08045-NO
YGGFMRF
>ID08046-NO
YGGIKKEIEAIKKEQEAIKKKIEAIEKEIEA
>ID08047-NO
YGRHSHHKEHFKRKC
>ID08048-NO
YHELRDLLLIVTRIVELLGRE
>ID08049-NO
YHKLKLLLIVTKIVELLGKK
>ID08050-NO
YHRLLRDLLIVTRIVELL
>ID08051-NO
YHRLLRDLLIVTRIVELLGRR
>ID08052-NO
YHRLRDLALIVTRIVELL
>ID08053-NO
YHRLRDLLLIVCRIVELL
>ID08054-NO
YHRLRDLLLIVCRIVELLGRR
>ID08055-NO
YHRLRDLLLIVRRIVCLL
>ID08056-NO
YHRLRDLLLIVRRIVCLLGRR
>ID08057-NO
YHRLRDLLLIVRRIVELL
>ID08058-NO
YHRLRDLLLIVRRIVELLGRR
>ID08063-NO
YHRLRDLLLIVTRIVRLL
>ID08065-NO
YHRLRDLLRIVTRIVELL
>ID08066-NO

YHRLRDLLRIVTRIVELLGRR
>ID08067-NO
YHRLRRLLLIVTRIVELL
>ID08071-NO
YKIIQQWFHWRRV
>ID08072-NO
YKQCHKKGGHCFPKEKICIPPSSDFGKMDCRWRWKCCKKGSG
>ID08074-NO
YPAKPEAPGEDASPEELSRYYASLRHYLNLVTRQRY
>ID08075-ABP_neg
YPCKLNLKLGKVPFHF
>ID08076-NO
YPCYDEY
>ID08077-NO
YPCYDPA
>ID08078-NO
YPIKPEAPREDASPEELNRYYASLRHYLNLVTRQRY
>ID08081-ABP_both
YPLKVKYPKLKV
>ID08082-ABP_pos
YPVEPF
>ID08085-ABP_both
YPVKLYPVKL
>ID08088-NO
YQALIRMIYKNI
>ID08089-NO
YQLAIRMIYKNI
>ID08090-NO
YQLLARMIYKNI
>ID08091-NO
YQLLIAMIYKNI
>ID08092-NO
YQLLIRAIYKNI
>ID08093-NO
YQLLIRMAYKNI
>ID08094-NO
YQLLIRMI
>ID08095-NO
YQLLIRMIAKNI
>ID08096-NO
YQLLIRMIY
>ID08097-NO
YQLLIRMIYANI
>ID08098-NO
YQLLIRMIYKAI
>ID08101-NO
YQLLIRPIYKNI
>ID08107-NO
YRQSMNQGSRSNGCRFGTCTFQKLAHQIYQLTDKDKDGMAPRNKISPQGY
>ID08109-NO
YRWYGYTPQNVIGGGKLLLKLLKKLLKLLKKK

>ID08112-NO
YTSLIHSLIEEAQNQQEKNEQELLELDKWASLWNWF
>ID08113-NO
YTSLIHSLIEEDQNQQEKNEQELLELDKWASLWNWF
>ID08114-NO
YTSLIHSLIEEEQNQQEKNEQELLELDKWASLWNWF
>ID08115-NO
YTSLIHSLIEEFQNQQEKNEQELLELDKWASLWNWF
>ID08116-NO
YTSLIHSLIEEGQNQQEKNEQELLELDKWASLWNWF
>ID08117-NO
YTSLIHSLIEEHQNQQEKNEQELLELDKWASLWNWF
>ID08118-NO
YTSLIHSLIEEIQNQQEKNEQELLELDKWASLWNWF
>ID08119-NO
YTSLIHSLIEELQNQQEKNEQELLELDKWASLWNWF
>ID08120-NO
YTSLIHSLIEEMQNQQEKNEQELLELDKWASLWNWF
>ID08121-NO
YTSLIHSLIEENQNQQEKNEQELLELDKWASLWNWF
>ID08122-NO
YTSLIHSLIEEPQNQQEKNEQELLELDKWASLWNWF
>ID08123-NO
YTSLIHSLIEEQQNQQEKNEQELLELDKWASLWNWF
>ID08124-NO
YTSLIHSLIEERQNQQEKNEQELLELDKWASLWNWF
>ID08125-NO
YTSLIHSLIEESQNLQEKNEQELLELDKWASLWNWF
>ID08146-NO
YTSLIHSLIQQSQNQQQKNQQQLLQLDKWASLWNWF
>ID08148-NO
YTSVITIELSNIKENKCNGTDAKVKLIKQELDKYK
>ID08149-NO
YVRSLLTRCNSFLWTLLRILQRILF
>ID08150-NO
YVSGKARGWFYRHHY
>ID08151-NO
YVSGKARGWFYRHHYESPHPRISSEVHIPLGDARLV
>ID08153-NO
AAAEETRRMLHRAFDTLA
>ID08155-NO
AAAPAATLEEHMTACQGV
>ID08156-NO
AAAQWDFGNTMC
>ID08157-NO
AAATQAANGGPATPA
>ID08158-NO
AACEVAKNLNESLIDLQELGKYEQYIKW
>ID08159-NO
AACYSSDCRVKCVAMGFSSGKCINSKCKCYK
>ID08161-NO

AAGAVVNDL
>ID08162-NO
AAGHATLREHLRDIK
>ID08163-ABP_both
AAGKGLVSNLLEK
>ID08164-NO
AAGLAMLFLGILSAAGSTMGARA
>ID08166-ABP_both
AAGYPFGIKV
>ID08167-NO
AAHLIDALYAEFLGGRVLTT
>ID08168-NO
AAHLIDALYAEFLGGRVLTTPVVHRALFYASAVLRQPFLAGVPSA
>ID08169-ABP_both
AAIYPFGIKIRCKAAFC
>ID08170-NO
AAKKWAKAKWAKAKKWAKAA
>ID08171-NO
AALEAKICHQIEYYFGDF
>ID08172-NO
AALKGCWTKSIPPKPCFRKR
>ID08173-NO
AALVVAQLLRIPQAIMDM
>ID08174-NO
AALYKKKIIKKLLES
>ID08175-NO
AANGGPATPAPPPLG
>ID08176-NO
AANTHRYLALAYKKFKKKLLKSLKRLG
>ID08178-NO
AAPTGDPKPKKNKKP
>ID08180-NO
AAQRRGRVGRNPNQVGD
>ID08181-NO
AATLEEMMTACQGVGGPGHKARVLA
>ID08182-NO
AAVALLPAVLLALLAP
>ID08183-NO
AAVPIVNLKDELLFPSWEALFSGSE
>ID08184-NO
AAWKWAWAKKWAKAKKWAKAA
>ID08186-NO
ACDCRGDCFCGGGGIVRRADRAAVP
>ID08187-NO
ACFPWGKEYCGGK
>ID08188-NO
ACFPWGNQWCGGK
>ID08189-NO
ACFPWGNTWCGGK
>ID08190-NO
ACFWKYCV

>ID08192-NO
ACNTATCVTHRLAGLLSRSGGMVKSNFVPTNVGSKAF
>ID08196-NO
ACSKKWEYCIVPILGFVYCCPGLICGPFVCV
>ID08199-NO
ACYCRIPACIAGERRAGTCAYQGRAWAACC
>ID08210-NO
ACYCRRRFCVCVGR
>ID08211-NO
ADDDCLPRGSKCLGENKQCCKGTTCMFYANRCVGV
>ID08214-ABP_pos
ADDKNPLEECFRETDYEEFLEIARNGLKATSNPKRVV
>ID08215-NO
ADGAHRSLLGRMKGA
>ID08216-NO
ADGAHSSLLGRMKGA
>ID08217-NO
ADGALLGRMKGA
>ID08218-NO
ADGALLGRMKPA
>ID08220-NO
ADGHRSSLLGRMKGA
>ID08222-NO
ADGPRSSLLGRMKGA
>ID08223-NO
ADGPYRVEHFRWGNPPKD
>ID08224-NO
ADGSLLGRMKGA
>ID08225-NO
ADGSLLGRMKGAAG
>ID08226-NO
ADGSLLGRMKPA
>ID08228-NO
ADGTLLGRMKLA
>ID08230-NO
ADGYQRSLLGRMKGA
>ID08232-NO
ADLEVVAATFVLVA
>ID08233-NO
ADLEVVAATHVLVA
>ID08234-NO
ADLEVVAATYAAAA
>ID08235-NO
ADLEVVAATYKKKK
>ID08236-NO
ADLEVVAATYV
>ID08237-NO
ADLEVVAATYVDDA
>ID08238-NO
ADLEVVAATYVDDD
>ID08239-NO

ADLEVVAATYVDVA
>ID08241-NO
ADLEVVAATYVKKK
>ID08246-NO
ADLKVVAATYVLVA
>ID08247-NO
ADLPFEF
>ID08249-NO
ADRFKQVDGFYARDL
>ID08253-NO
ADVILMCFSIDSPDSLENI
>ID08254-NO
AEAIPMSIPPEVKFNKPFVF
>ID08255-NO
AECMVDETVCYIHNHNNC
>ID08256-NO
AECVVSCSMSYTKA
>ID08258-NO
AEEENTGGLPGVQLSPCTSPPVGLIPCSAPWS
>ID08259-NO
AEGIGALFLGFLGAAGSTMGARSMTLTVQARQL
>ID08260-NO
AEKDCIAPGAPCFGTDKPCCNPRAWCSSYANKCL
>ID08262-NO
AELYVREHLREQSRK
>ID08263-NO
AENTDANFYVCPPPT
>ID08264-NO
AEPVAML
>ID08265-NO
AESTTPCSGSYLKA
>ID08266-NO
AETVESCLAKPHTEN
>ID08267-NO
AFCNLRMCQLSCRSLGLLGKCIGDKCECVKH
>ID08268-NO
AFCNLRRCELSCRSLGLLGKCIGEECKCVPY
>ID08269-NO
AFFYRWWIR
>ID08270-NO
AFHRYGTTVNCIVEE
>ID08271-NO
AFKAFWKFVKFVK
>ID08272-NO
AFKQSPPAIPALGTN
>ID08273-NO
AFLGWIGAIVSTALPQWR
>ID08275-ABP_both
AFQWQRNMRKVR
>ID08276-NO
AFRKVKRWG

>ID08278-NO
AFWRWIRFK
>ID08279-NO
AGAGTGATAIGMVTQYHQVL
>ID08280-NO
AGALMFAWLLLGLQGIFN
>ID08281-NO
AGAVVNDL
>ID08282-NO
AGDDQGLDKCVPNSKEK
>ID08283-NO
AGDNATVAAGHATLREHLRDIKAENTDAN
>ID08285-NO
AGEGLSSPFWSLAAPQRF
>ID08287-NO
AGHATLREHLRDIKAENTDAN
>ID08288-NO
AGHNKVGSLQYLALA
>ID08289-NO
AGKRKSG
>ID08290-NO
AGPAIALVGDAR
>ID08291-NO
AGPDTPSQFRKKWNKWALSR
>ID08294-NO
AGVKDGKLDF
>ID08295-NO
AGVLW
>ID08296-NO
AGVSGHGQHGVHG
>ID08297-ABP_both
AGYLLGKINLKPLAALAKKIL
>ID08299-NO
AHWGVLAGIKYFSMVGNW
>ID08300-NO
AICKRIPNKKPGKKT
>ID08301-NO
AIFQSSMTKILEPFR
>ID08302-ABP_both
AIGHCLGATL
>ID08306-NO
AINNALNKVCSTGRRQRSICKQLLKKLRQQ
>ID08309-ABP_both
AIRRWRIRK
>ID08311-NO
AITLIFI
>ID08312-NO
AIWHKTRRL
>ID08313-ABP_both
AKCLRWQWAMRKVGG
>ID08314-ABP_both

AKCLRWQWAMRKYGG
>ID08315-ABP_both
AKCLRWQWEMRKVGG
>ID08316-ABP_both
AKCLRWQWEMRKYGG
>ID08317-ABP_both
AKCLRWQWRMRKVGG
>ID08318-ABP_both
AKCLRWQWRMRKYGG
>ID08319-ABP_both
AKCRRWQWRMKKLGA
>ID08322-NO
AKDLEVVTSTYVLVEA
>ID08323-NO
AKFDKFYGLM
>ID08324-NO
AKFEVNNPQVQRQAFNELIRVVHQLLPESSLRKRKRSRC
>ID08325-NO
AKKAAKKAKKAAKKAKKAAKK
>ID08326-NO
AKKAAKKAKKAAKKAKKWAKK
>ID08328-ABP_both
AKKFGKAFVGEIM
>ID08329-NO
AKKIKWLVW
>ID08330-ABP_both
AKKIRVRLSA
>ID08332-NO
AKKQRFRHRNRKG
>ID08333-ABP_both
AKKVFKRLEKLFSKIFNFK
>ID08338-ABP_both
AKKVFKRLEKLFSKIYNYK
>ID08342-NO
AKLEVVAATYVKKK
>ID08343-NO
AKLIALTLLGMGLALFRNHQS
>ID08344-NO
AKLKVVAATYVKKK
>ID08345-NO
AKLKVVAATYVLKK
>ID08348-NO
AKRFFGYKRKFF
>ID08349-NO
AKRHHGYHRKFH
>ID08350-NO
AKRHHGYKR
>ID08352-NO
AKRHHKYHRKFH
>ID08353-NO
AKRHHKYKRKFH

>ID08355-NO
AKRYYGYKRKFY
>ID08356-NO
AKTIRLESEVTAIKNALKKTNEAVSTLGNGVRVLATAVRELKDFVSKN
>ID08358-NO
AKYVRNNLETTAFHR
>ID08359-NO
ALAALITPKKIKPPL
>ID08360-NO
ALAGTIIAGASLTFQVLDKVLEELGKVSRK
>ID08361-NO
ALCNCNRIIIPHMCWKKCGKK
>ID08363-NO
ALDKAEESNSKADKVNVKLT
>ID08364-NO
ALDKAEESNSKLDKVNVKLT
>ID08368-NO
ALGPAAAAAEMMTACQGV
>ID08369-NO
ALGPAATLEEMMAAAQGV
>ID08370-NO
ALGPAATLEEMMTACAAA
>ID08371-NO
ALGPAATLEEMMTACQGV
>ID08372-NO
ALGVATSAQITAAVALVEAKQARSDIEKLKEAIRD
>ID08373-NO
ALHALGYQLAFVLDSPSAY
>ID08375-NO
ALLHHGLNCAKGVLAALLHHGLNCAKGVLA
>ID08376-NO
ALLPRMKG
>ID08377-NO
ALLTRILG
>ID08379-NO
ALNCYWPLNDYGFYTTTGIGYQPYRVVVLSFEL
>ID08381-NO
ALNSVAYERSVMQDYE
>ID08382-NO
ALNTLVKQLSSNFGAISSVLNDILSRLDKVEAEVQIDRL
>ID08383-NO
ALPCAKKSCDSWCRRLDYPGGECVTKWKCSCNWMQIDK
>ID08384-NO
ALPQWRIYSYAGDNIVTA
>ID08386-NO
ALQELLGEYIQWLKDGGPSSGRPPPS
>ID08387-NO
ALQELLGQWLKDGGPSSGRPPPS
>ID08388-NO
ALRGPKMMRDSGCFGRRLDRIGSLSGLGCNVLRRY
>ID08391-ABP_both

ALWKDLLKNVGKAAGKAVLNKVTDMVNQ
>ID08393-ABP_both
ALWKSLLKNVGKAAGKAALNAVTDMVNQ
>ID08394-ABP_pos
ALWKTLLKKVLKAYSPWTNF
>ID08398-ABP_both
AMPWRPATGLLPIKPTHIKPLCGDD
>ID08400-NO
ANDRSNATLLDGPSGALLLRLVQLAGAPEPAEPAQPGVY
>ID08401-ABP_both
ANFLPKIFCKITRKC
>ID08403-NO
ANGSRIPTGERVWDRGNVTL
>ID08404-NO
ANIVFDVESATTGTYSTFLTSF
>ID08405-ABP_both
ANLGNYTSQCYSSQCYSSKCYSDSCYSSNCYTGRHMCGYTHGYSC
>ID08406-NO
ANPDCKTILKALGPAATLEEMMTAC
>ID08407-NO
ANRETKLGKAGYVTNRGRQK
>ID08410-NO
ANVVATYPAHS
>ID08418-ABP_both
APDRPRKFCGILG
>ID08420-NO
APEDIGFCLEGGCLVALG
>ID08421-NO
APERASSVYTRSTGEQEI
>ID08422-ABP_both
APFCLGYLSPKLKDMEPKPRG
>ID08423-NO
APFNWDLPEPRSRASKIRVHPRGNLWATGHFM
>ID08425-ABP_both
APKAMRLLRRLLRLQKKGI
>ID08426-NO
APLEPMYPGDYATHEQRAQYETQLRRYINTLTRPRY
>ID08428-NO
APLGWDLPESRSRASKIRVHPRGNLWATGHFM
>ID08430-NO
APLSWDLPEPRSRASKIRVHSRGNLWATGHFM
>ID08432-NO
APPGFTPF
>ID08433-NO
APPGFTPFRIA
>ID08434-NO
APPGFTPFRID
>ID08435-NO
APPGFTPFRIY
>ID08437-NO
APPGFTPFRSK

>ID08438-NO
APPGFTPFRSKAV
>ID08439-NO
APPGRSDVYPPPLGSEHNGQVAEDAVSRPKDDSVPEVRAA
>ID08440-NO
APSDPRLRQF
>ID08441-NO
APTSPGTPGVAAATQ
>ID08442-NO
APVPGLSPFR
>ID08444-NO
APVSTGAGGGTVLAKMYPRGSHWAVGHLM
>ID08445-NO
APVTAGRGGALAKMYTRGNHWAVGHLM
>ID08446-NO
APWLVPSQITTCCGYNPGTMCPSCMCTNTC
>ID08449-NO
AQDIISTIGDLVKWIIDTVNKFTKK
>ID08450-NO
AQEEAEAEERRLQEQEELENYIEHVLLRRP
>ID08451-NO
AQITAAVALVEAKQARSDIEKLKEAIRDTNKAVQS
>ID08452-NO
AQLLESVLQEERILALHAQARERLT
>ID08454-NO
ARALAHGVRVLEDGVNYA
>ID08455-NO
AREPSNATQLDGPARALLLRLVQLAGTRESVDSAKPRVY
>ID08457-NO
ARKWKYWRF
>ID08460-NO
ARLHRPSEFARLGLVIAKRFAARAVTRNTLKRVIRELDYVVLKRSARAEV
>ID08461-NO
ARLKGGFLRVLFTVGKKLVPRAVDRNRIKRLMRELTDHQVLERFRAIRH
>ID08462-NO
ARLLKRKQFVKVGITVSKKFGKAHQRNRFKRIVRELQVVILSEELLQRI
>ID08463-NO
ARLLKRKQFVKVGVTVSKKFGKAHQRNRFKRIVRELQVVVLSADLLKHI
>ID08464-NO
ARMLGDVMAVSTCVP
>ID08465-NO
ARRAKRRARRAARRARRKARR
>ID08466-NO
ARRAKRRARRAKRRARRKKRR
>ID08468-NO
ARRYNATHIKVGQPQ
>ID08469-NO
ARWMWRRWR
>ID08470-NO
ASANASVERIKTTSS
>ID08471-NO

ASCDLFSFSSQWVTPNDSVCAAHCLVKGYKGGSCKNKICHCRDKF
>ID08472-NO
ASCQIGDIWGAGDAACSASCLKDGTYHGGYCNEESVCVCTY
>ID08473-NO
ASCRTPKDCADPCRKETGCPYGKCMNRKCKCNRC
>ID08474-NO
ASCYLLDGYAAGRDDCRAHCIAPRNRRLYCASYQVCVCRY
>ID08475-NO
ASDRSNATQLDGPAGALLLRLVQLAGAPEPFEPAQPDAY
>ID08476-ABP_both
ASENGKCNLLCLVKKKLRAVGNVIKTVVGKIA
>ID08478-NO
ASKQASKQASKQASKQASKQASRSLKNHLL
>ID08479-NO
ASLRVRIKK
>ID08481-NO
ASSRTPS
>ID08483-NO
ASVTVGRRVSARMLG
>ID08484-NO
ASYYY
>ID08485-NO
ATAAETRRMLHRAFDTLA
>ID08486-NO
ATAEATRRMLHRAFDTLA
>ID08487-NO
ATAEEARRMLHRAFDTLA
>ID08488-NO
ATAEETARMLHRAFDTLA
>ID08489-NO
ATAEETRAMLHRAFDTLA
>ID08490-NO
ATAEETRRALHRAFDTLA
>ID08498-NO
ATAGWTFGAGAALQIPFAMQMAY
>ID08499-NO
ATAPTTRNLLTTPKF
>ID08503-NO
ATCDLFSFESKWFTPNHAACAAHCILLGNRGGHCVGTVCHCRK
>ID08504-NO
ATCDLFSFQSKWVTPNHAACAAHCLLRGNRGGQCKGTICHCRK
>ID08508-NO
ATCDLLSFLNVKDAACAAHCLAKGYRGGYCDGRKVCNCRR
>ID08515-NO
ATCDLLSIFNVNHAACAAHCLAIGRRGGYCNSKQVCVCR
>ID08516-NO
ATCENLANTYRGPCFGGCDFHCKTKEHLLSGRCRDDFRC
>ID08517-NO
ATCYCRRGRCATRESLSGVCEISGRLYRLCCR
>ID08524-NO
ATGQETAYFLLKLAGKA

>ID08526-NO
ATHIKVGQPQYYQAN
>ID08528-NO
ATHQEAIEKVTGALKINNLR
>ID08529-NO
ATHTICDRNHTW
>ID08530-NO
ATKALTEVIPLTEEAEC
>ID08531-NO
ATKVKAKQRGKEKVSSGRPGQHN
>ID08532-NO
ATLEEMM
>ID08533-ABP_both
ATLHLVLRLRGG
>ID08534-NO
ATMYYKDVTVSQVWF
>ID08535-NO
ATSAQITAAVALVEAKQARSDIEKLKEAIRDTNKA
>ID08537-NO
ATSSANSKA
>ID08539-NO
ATVSIC
>ID08540-NO
ATWVCGPCT
>ID08542-NO
AVASVPRARGKYWWG
>ID08548-NO
AVGIGALFLGFLGAAGSTMGARSAMTLTVQRQL
>ID08550-NO
AVGIGAMFLGFLGAAGSTMGAAS
>ID08552-NO
AVKRVGRRLKKLARKIARLGVAF
>ID08554-ABP_both
AVPLIYKRPGVYYVTKPKGK
>ID08555-NO
AVPRVDDEPRAQLGALLARYIQQARKAPSGRMSVIKNLQSLDPSHRISD
>ID08557-ABP_both
AVPYPQR
>ID08560-NO
AVSPTTLRTEVVKTFRRDKPFPHRMDCVTT
>ID08562-NO
AVVNDL
>ID08564-NO
AVYLHRIDLGPPISLERLDVGTNLQNAIAKLEDAK
>ID08565-NO
AWDFGSIGGVFTSVGKLVHQVFGTAYGVL
>ID08566-NO
AWDFGSLGGVFTSIGKALHQVFGAIYGAA
>ID08567-NO
AWDFGSVGGLFTSLGKAVHQVFGSVYTIM
>ID08568-NO

AWDFGSVGGVLNSLGKMVHQIFGSAYTAL
>ID08569-NO
AWKKWAKAWKWAKAKWWAKAA
>ID08571-NO
AWLVHTQWFLDLPLPWLPGADTQGSNWI
>ID08572-ABP_both
AWRFKNIRK
>ID08573-ABP_both
AWRWKAFRNCWRVRSSSL
>ID08574-NO
AWVPAHKGIGGNEQ
>ID08576-NO
AYGFRGPGPQL
>ID08577-NO
AYQPLLSNTLAELYV
>ID08579-NO
AYRWWTRFK
>ID08580-NO
CACIADHSH
>ID08581-NO
CAGKRKSG
>ID08582-NO
CAHNLTHAC
>ID08583-NO
CANLLLQYGSFCTQLNRALSGIA
>ID08584-ABP_both
CAQWQRNMRKVR
>ID08585-NO
CATCIADHRSH
>ID08586-NO
CATCIADHRSHRQMV
>ID08587-NO
CATCQIADSHRSH
>ID08588-NO
CATCQIADSHRSHRQMV
>ID08590-NO
CCFLRIQNDSIIRLGDLQPLSQRVSTDWQ
>ID08591-NO
CCGCCYIAACARNNSNCSSHLECSTC
>ID08592-NO
CCNCSSKWCRDHSRCC
>ID08593-NO
CCRLACGLGCHPCC
>ID08594-NO
CDATSAQVC
>ID08598-NO
CDQRTTRLC
>ID08600-NO
CDSCERCWYVWLILLRVRLRLLVSL
>ID08601-NO
CDVIALL

>ID08602-NO
CDVIALLACHLNT
>ID08605-NO
CDVIALLCHLNT
>ID08606-NO
CDVIALLCHLNTPSF
>ID08607-NO
CDVIALLCHLNTPSFNTTHYRESWY
>ID08608-NO
CDVIALLECHLNT
>ID08610-NO
CEELRVRLASHLRKLRKRLLRDADDLQKRLAVY
>ID08611-NO
CEEQAQQIRLQAEAFQARLKSWFEPLVEDM
>ID08612-NO
CEGLPNIDC
>ID08613-NO
CEHLSHAAC
>ID08614-NO
CEKLHTASC
>ID08615-NO
CERLNLPQC
>ID08617-NO
CEVLSDFK
>ID08618-NO
CEWTESMMC
>ID08619-NO
CEYQAGHNKVGSLQY
>ID08622-ABP_both
CFAWQRAMRKVR
>ID08624-NO
CFIRNCPKG
>ID08625-NO
CFLNITNSHVSILQEAPPLENAV
>ID08626-NO
CFLNITNSHVSILQEAPPLENRV
>ID08628-NO
CFNTHTANC
>ID08629-NO
CFPTTSRGC
>ID08630-NO
CFPWGC
>ID08631-ABP_both
CFQAQRNMRKVR
>ID08632-ABP_both
CFQWARNMRKVR
>ID08633-ABP_both
CFQWKRAMRKVR
>ID08634-ABP_both
CFQWKRNMRKVR
>ID08635-ABP_both

CFQWQANMRKVR
>ID08637-ABP_both
CFQWQRNARKVR
>ID08638-ABP_both
CFQWQRNMAKVR
>ID08639-ABP_both
CFQWQRNMRAVR
>ID08643-NO
CFQWQRNMRKVRGPPVSCIKRDSPIQ
>ID08644-NO
CFRTLEHLC
>ID08645-NO
CFSIDSPDSLENI
>ID08646-NO
CFSSARLSC
>ID08647-NO
CGAHHAADAHHAADAHHAADGC
>ID08648-NO
CGAHHAADAHHAADGC
>ID08649-NO
CGECGGGHIVGRFCMVVRFLRLVFI
>ID08651-NO
CGESCFLGTCYTKGCSCGEWKLCYGTNGGTIFD
>ID08652-NO
CGESCVFIPCITSLAGCSCKNKVCYYDGGSVP
>ID08656-NO
CGETCFGGTCNTPGCSCTWPICTRDGLPV
>ID08657-NO
CGETCLLGTCYTTGCTCNKYRVCTKDGSVLN
>ID08658-NO
CGETCTLGTCYTAGCSCSWPVCTRNGVPI
>ID08662-NO
CGETCVGGTCNTPGCTCSWPVCTRNGLNPV
>ID08663-NO
CGFSNFRSC
>ID08666-NO
CGKLLEQKNFFLKTR
>ID08667-NO
CGKRKLC
>ID08668-NO
CGKRKSC
>ID08669-NO
CGKRRC
>ID08670-NO
CGLKTNPAC
>ID08671-NO
CGMLNWNRC
>ID08672-NO
CGNLSTCMLGTYTQDLNKFHTFPQTSIGVEAP
>ID08675-NO
CGRCLQRACCKYCRLKCRLILFVIF

>ID08676-NO
CGRRAGGSC
>ID08677-NO
CGSVFLVGQLFTFSPRHH
>ID08678-NO
CGTATCETQRLANFLAPSSNKLGAIFSPTKMGSNTY
>ID08679-NO
CGTCVRDCWPETGSVRFPFH
>ID08680-NO
CGYGLC
>ID08681-NO
CGYKGC
>ID08682-NO
CGYKLC
>ID08683-NO
CGYKRC
>ID08684-ABP_both
CGYRHGRLNCGRG
>ID08685-NO
CHADQLPMC
>ID08686-NO
CHANLTHAC
>ID08687-NO
CHARNSTQC
>ID08689-NO
CHGSTKWAC
>ID08690-NO
CHGVYALHC
>ID08691-NO
CHHALTHAC
>ID08692-NO
CHHNATHAC
>ID08693-NO
CHHNLAHAC
>ID08695-NO
CHHNLTHAC
>ID08696-NO
CHKGHTWNC
>ID08697-NO
CHLLLPRPC
>ID08698-NO
CHNLESGTC
>ID08699-NO
CHNLKRPTC
>ID08700-NO
CHPGSSSRC
>ID08701-NO
CHPSKALQC
>ID08702-NO
CHPSTHRYC
>ID08703-NO

CHPTGLHQC
>ID08704-NO
CHQLMQNLC
>ID08705-NO
CHRCFHFRRHPVAVF
>ID08706-NO
CHSLRNAFC
>ID08707-NO
CHSPLTSSC
>ID08708-NO
CHTKIFPNC
>ID08709-NO
CHTLPHTKC
>ID08710-NO
CHTNASPHC
>ID08711-NO
CHVLDPHLC
>ID08712-NO
CHWMFSPWC
>ID08713-NO
CICCCMCVLYAVLWWLSVLVSLCELLIRSEFELERSMNRRY
>ID08714-NO
CIGAVLKVLTTGLPALISWIKRKRQQ
>ID08715-NO
CIGAVLKVLTTGLPALISWIKRKRQQC
>ID08716-NO
CIHAPHTQC
>ID08717-NO
CIIRNCPRG
>ID08719-NO
CINASHAHC
>ID08720-NO
CIPMMTEFC
>ID08721-NO
CIRLQAEAFQARLKSWFEPLV
>ID08722-NO
CIVEEVDARSVYPYD
>ID08723-NO
CKAAGKPCSRIAYNCCTGSCRSGKC
>ID08724-NO
CKEYGRQMC
>ID08725-NO
CKFCYGSAQCPTFLFIVRLLRFVWV
>ID08726-NO
CKFPLNAAC
>ID08727-NO
CKGKGAKCSRLMYDCCTGSCRSGKC
>ID08728-NO
CKGKGAPCRKTMYDCCSGSCGRRGKC
>ID08729-NO
CKGTGKSCSRIAYNCCTGSCRSGKC

>ID08730-NO
CKHHNWWTC
>ID08731-NO
CKHQCMSTRGDQARKICESVCM
>ID08732-NO
CKIALPYHMRCRVLGRC
>ID08733-NO
CKKIGAVLKVLTTGLPALISWIKRKRQQKKC
>ID08734-NO
CKLKGQSCRKTSYDCCSGSCGRSGKC
>ID08735-NO
CKPDAISVC
>ID08737-NO
CKPTHYNSC
>ID08738-NO
CKPWPMYSC
>ID08739-NO
CKQADEPCDVFSLDCCTGICLGVCMW
>ID08740-NO
CKQAGESCDIFSQNCCVGTCAFICIE
>ID08741-NO
CKSCDTRCTCLRRRLRVGVGLPCMGC
>ID08742-NO
CKSFTTTRC
>ID08744-NO
CKSPGSSCSPTSYNCCRSCNPYTKRCY
>ID08745-NO
CKSTFSPNC
>ID08746-NO
CKSTSNVYC
>ID08747-NO
CKTGHMRIC
>ID08748-NO
CKTRTSTNTDHLWIAKSKARLSET
>ID08749-NO
CKWEEAKVKFHCQRTQSQPGSW
>ID08751-NO
CLALRHSNC
>ID08752-NO
CLCCDKVRTCRRLLGLVMVLSVVRC
>ID08753-NO
CLFNHPKYC
>ID08755-NO
CLHKPWSRC
>ID08759-ABP_both
CLLKKLLKKC
>ID08760-NO
CLLLGTEVSEALGGAGLT
>ID08761-NO
CLLLGTEVSEALGGAGLTG
>ID08762-NO

CLLLGTEVSEALGGAGLTGG
>ID08763-NO
CLLNKQNAC
>ID08764-NO
CLMGSSHSC
>ID08766-NO
CLPTPHHVC
>ID08767-NO
CLQDMRQFC
>ID08768-NO
CLQHDALNC
>ID08769-NO
CLQHNEREC
>ID08770-NO
CLRKLRKRLLRC
>ID08771-NO
CLRVRLASHLRKLRKRLLRDADDL
>ID08772-NO
CLSIHSSVC
>ID08773-NO
CLTDRHRTC
>ID08774-NO
CLTSPTSTC
>ID08775-NO
CLVRNLAWC
>ID08776-NO
CLWPTLKGC
>ID08777-NO
CMQSAAAHC
>ID08780-NO
CNANKPKMC
>ID08781-NO
CNCTNSSSSYSGTKMACPSNRG
>ID08782-ABP_both
CNGKRVCVCR
>ID08783-NO
CNGVIHNQC
>ID08784-NO
CNHTRNMAC
>ID08785-NO
CNIAPASIVSRNIVYTRAQPNQDIA
>ID08786-NO
CNKPFSLPC
>ID08787-NO
CNKQFSAAC
>ID08788-NO
CNLLRQQTC
>ID08789-NO
CNLMGNPHC
>ID08790-NO
CNPMHSRTC

>ID08791-NO
CNPMKPLMC
>ID08792-NO
CNPYNTSMC
>ID08793-NO
CNQSTLLTC
>ID08794-NO
CNSHSPVHC
>ID08795-NO
CNSMWVRNC
>ID08796-NO
CNSPKGKPC
>ID08797-NO
CNSSSPTAC
>ID08798-NO
CNSTKNLTFAMRSSGDYGEV
>ID08799-NO
CNSVPPYQC
>ID08801-NO
CNTSSTPHC
>ID08802-NO
CPAASHPRC
>ID08803-NO
CPDHPFLRC
>ID08804-NO
CPFVKTQLC
>ID08806-NO
CPGHIHRTC
>ID08807-NO
CPGNWWSTC
>ID08808-NO
CPGPEGAGC
>ID08809-NO
CPHDPNHPC
>ID08810-NO
CPHTQFWQC
>ID08811-NO
CPKHVLKVC
>ID08812-NO
CPKLHPGGC
>ID08813-NO
CPLASTRTC
>ID08814-NO
CPLGKLPWC
>ID08815-NO
CPMSQNPTC
>ID08816-NO
CPPGKSSMC
>ID08817-NO
CPPPQGQTC
>ID08818-NO

CPPPTGATVVQFEQP
>ID08819-NO
CPRSGTAYC
>ID08820-NO
CPSHLDAFC
>ID08821-NO
CPSHYTQAC
>ID08822-NO
CPSLHTREC
>ID08823-NO
CPSNVNNIC
>ID08824-NO
CPSRLSSQC
>ID08825-NO
CPSSPFNHC
>ID08826-NO
CPSTGYSYC
>ID08828-NO
CPTTKSNVC
>ID08829-NO
CPWSTQYAC
>ID08830-NO
CQAMHNRFC
>ID08831-NO
CQATTARNC
>ID08833-NO
CQGPVKPLC
>ID08834-NO
CQGSPYRHC
>ID08835-NO
CQLPAERWC
>ID08836-NO
CQLSLAPYC
>ID08838-NO
CQPHTLPTC
>ID08839-NO
CQPSDPHLC
>ID08841-NO
CQRTTEANC
>ID08842-NO
CQSASPRLC
>ID08843-NO
CQSIRGPMC
>ID08844-NO
CQSLPTHNC
>ID08845-NO
CQSQNHNTC
>ID08846-NO
CQSQTRNHC
>ID08847-NO
CQTTNWNTC

>ID08848-NO
CQVLNGNHC
>ID08849-NO
CQWPGQSGC
>ID08850-ABP_both
CQWQFISPSRAGCIGP
>ID08852-NO
CRANGSRIPTGERVWDRGNV
>ID08853-NO
CRCCELKSLCPTLMRVVRLLGLVLL
>ID08855-NO
CRFPNITNSHVPILQERPPLENRVLTGWGL
>ID08856-NO
CRHNNLHHC
>ID08857-NO
CRIPNQKCFQHLDDCCSRKCNRFNKCV
>ID08858-ABP_neg
CRLGRGKCRRTCIESEKIAGWCKLNFFCCRERI
>ID08859-NO
CRNTDTALC
>ID08860-ABP_neg
CRRWQWRMKKLGA
>ID08861-ABP_both
CRRWQWRMKKLGAPSITCV
>ID08863-NO
CRSLTDNQC
>ID08864-NO
CRSQKSCWIIKGHCRKNCKPGEQVKKPCKNGDYCCIPSNTDS
>ID08865-NO
CRTLPPILC
>ID08866-NO
CRVTQTHTC
>ID08867-ABP_both
CRWRWKCCKK
>ID08869-NO
CSAAERLNC
>ID08871-NO
CSAPFTKSC
>ID08872-NO
CSASTESLC
>ID08873-NO
CSCKNKVCYRNGIPCGESCVWIPCISAALG
>ID08874-NO
CSCKNKVCYRNGVIPCGESCVFIPCISTLLG
>ID08875-NO
CSCKSKVCYKNSIPCGESCVFIPCTVTALLG
>ID08877-NO
CSCRANGSRIPTGERVWDRG
>ID08878-NO
CSCSSLMDKECVYFCHLDIIWVNTPEHVVPYGLGSPRS
>ID08879-NO

CSCSSWLDKECVYFCHLDIIW
>ID08880-NO
CSDSWLPRC
>ID08881-NO
CSGSETKVC
>ID08882-NO
CSGVINTTC
>ID08883-NO
CSHLPPNRC
>ID08884-NO
CSICSNNPTCWAICKRIPNKKPGKK
>ID08885-NO
CSIELSDIPLSVDFNTMID
>ID08886-NO
CSLAPANTC
>ID08887-NO
CSLHSHKGC
>ID08888-NO
CSLRTYAAC
>ID08889-NO
CSLSPLGRC
>ID08890-NO
CSLSSPRIC
>ID08891-NO
CSLTNTLLC
>ID08892-NO
CSLTPHRSC
>ID08893-NO
CSNLSTCVLGKLSQELHKLQTYPRTNTGSGTP
>ID08895-NO
CSPGPHRVC
>ID08896-NO
CSPLLRTVC
>ID08897-NO
CSPLQIPYC
>ID08898-NO
CSPQLAPFC
>ID08899-NO
CSPYAKHNC
>ID08901-NO
CSQWLQGS
>ID08902-NO
CSRCLELALCLDPAN
>ID08903-NO
CSRNHILTC
>ID08904-NO
CSSQNGRIC
>ID08905-NO
CSSRTMHHC
>ID08906-NO
CSTNPPTQC

>ID08907-NO
CSTPELTFC
>ID08908-NO
CSTRAENQC
>ID08909-NO
CSTTNATWC
>ID08910-NO
CTARYTQHC
>ID08911-NO
CTCFTYKDKECVYYCHLDIIW
>ID08912-NO
CTCIADHRH
>ID08913-NO
CTCSWPVCTRNGLPVCGETCVGGTCNTPG
>ID08914-NO
CTDNALKAC
>ID08916-NO
CTEEHIVATELVIQEMYIKINVKNSP
>ID08917-NO
CTEMEKEGKISKIGP
>ID08918-NO
CTESTINTC
>ID08919-NO
CTFHSPRFC
>ID08921-NO
CTGSNLPIC
>ID08922-NO
CTGYHNNTC
>ID08923-NO
CTKYWARNC
>ID08924-NO
CTLCRVVDCCLYAIVFVMVRVVVLCVNKIRIRAREIYES
>ID08925-ABP_both
CTLISWIKNKRKQRPRVSRRRRRRGGRRRR
>ID08926-NO
CTLNFPISPIETVPVKLKPG
>ID08927-NO
CTLTTKLYC
>ID08928-NO
CTMCRYQQNCFTRRLIVGGMLLVFV
>ID08929-NO
CTMLLFHRC
>ID08930-NO
CTNPATPFC
>ID08931-NO
CTNPESLTC
>ID08932-NO
CTNRHAAGC
>ID08933-NO
CTNSTLQSC
>ID08934-NO

CTPDKKSFC
>ID08935-NO
CTPGRSATC
>ID08936-NO
CTPLSTLQC
>ID08937-NO
CTPSLPWLC
>ID08938-NO
CTPTMHNHC
>ID08939-NO
CTPTVTRSC
>ID08940-NO
CTQCCAPSTCLNYRIFVGLLRFVVI
>ID08941-NO
CTQKNIAAC
>ID08942-NO
CTQSGLLSC
>ID08943-NO
CTRPNNNTRKSIRIQRGPGRAFVTIGKIGNMRQAHC
>ID08944-NO
CTSAAVHMC
>ID08945-NO
CTSDLPSSWGYMNCNCTNSSSS
>ID08946-NO
CTSMAYHHC
>ID08947-NO
CTSTHTKTC
>ID08948-NO
CTTHISQTC
>ID08950-NO
CTVGPTRSC
>ID08951-NO
CTYPYPKFC
>ID08952-NO
CVCVKTTSLVRPRHI
>ID08953-ABP_both
CVDIGFSPTGKRPPFCPYPG
>ID08954-NO
CVHWQTNPARTSRIGP
>ID08955-NO
CVISAGWNHKIR
>ID08956-NO
CVMSKHQHC
>ID08957-NO
CVNSPPTMC
>ID08959-NO
CVRLASHLRKLRKRLLRDADDL
>ID08960-NO
CVRLASHLRKLRKRLLRDADDLQKRLAVY
>ID08961-NO
CVRTPTHHC

>ID08962-NO
CVSGQESIC
>ID08964-NO
CVTCKSTVLCDKMQHPCRRGPRCISC
>ID08966-NO
CWVRLGRYLLRRLKTPFT
>ID08967-NO
CWVRLGRYLLRRLKTPFTRL
>ID08968-NO
CYATTLGAC
>ID08969-NO
CYCFRRFCVC
>ID08971-NO
CYCRKGHCKRGERVRGTCGIRFLYCCPRRGLL
>ID08972-NO
CYETKTHSC
>ID08973-NO
CYHCQ
>ID08974-NO
CYHPSFNTC
>ID08975-NO
CYKMDNHTC
>ID08976-NO
CYRTQFTQC
>ID08977-NO
CYSCPCERRCHKIARGLLILRSVLF
>ID08978-NO
CYSLLPRVC
>ID08979-NO
CYSRPLVSFRYEDQG
>ID08981-ABP_both
DAAVEPELYHWGKVWLPN
>ID08982-NO
DADSSIEKQVALLKALYGHGQLSHKRHKTDSFVGLM
>ID08984-NO
DAEEDDSLANSSDLLKELLETGDNRERSHHQDGSDNEEEVS
>ID08985-NO
DAENLMDSFQEIVKEVGQLAETQHFECTMHQPRSPLQDLKGALESLIEE
>ID08986-NO
DAGHGQISHKRHKTDSFVGLM
>ID08987-NO
DAILHTPGCVPCVREGNA
>ID08988-NO
DARDAMDRIFARRYN
>ID08990-NO
DAVYLHRIDLGPPISLERLDVGTNLQNAIAKLEDA
>ID08991-NO
DCFSESAIRKAILGH
>ID08992-NO
DCGHLHDPCPNDRPGHRTCCIGLQCRYGKCLVRV
>ID08993-NO

DCIGKDARDAMDRIF
>ID08994-NO
DCLGWFKSCDPKNDKCCKNYTCSRRDRWCKYDL
>ID08995-NO
DCNTKACWALCQREHGIYFRRAVCEGSRCKCILVNGR
>ID08996-NO
DCPNGPWVWVPAFCQAVGWG
>ID08997-NO
DCWVRLGRYLLRRLKTPF
>ID09001-NO
DDCGKLFSGCDTNADCCEGYVCRLWCKLDW
>ID09002-NO
DDCIKPYGFCSLPILKNGLCCSGACVGVCADL
>ID09003-NO
DDDEVVAATYVA
>ID09004-NO
DDDEVVAATYVLVA
>ID09007-NO
DDHETDMELKPANAA
>ID09012-NO
DDSVVAAMSYSFA
>ID09013-NO
DDSVVAAMSYSHA
>ID09014-NO
DDSVVAAMSYSYA
>ID09015-NO
DDSVVCAAMSYSFA
>ID09016-NO
DDSVVCAAMSYSHA
>ID09019-NO
DDSVVSAAMSYSHA
>ID09023-NO
DEDTAGLPGRQLPPCTSLLVGLMPCAAARS
>ID09024-NO
DEGPYRMEHFRWGSPPKD
>ID09028-NO
DEMEEC
>ID09032-ABP_both
DEMKLDGFNMHLE
>ID09034-NO
DEVVCC
>ID09045-NO
DFDMLRCMLGRVYRPCWQV
>ID09047-NO
DFRRLPGAFWQLRQP
>ID09048-NO
DFWEVQLGIPHPAGLKKKKS
>ID09049-NO
DGFIHVQGHLQEVDAGNF
>ID09050-NO
DGIVGKASSYAAL

>ID09052-NO
DGSWSTVSSGADTED
>ID09053-NO
DGSYKMKHFRWSSPPAG
>ID09054-NO
DGSYKMNHFRWSGPPAS
>ID09056-NO
DGSYRMGHFRWGSPTAI
>ID09057-NO
DHILEPSIPWKSKLLTLVQL
>ID09058-NO
DHYACVSSGGQCLYSACPIFTKIQGTCYRGKAKCCK
>ID09060-NO
DHYNCVSAGGQCLYSACPIFTKIQGTCYRGKAKCCK
>ID09061-NO
DHYNCVSSGGACLYSACPIFTKIQGTCYRGKAKCCK
>ID09062-NO
DHYNCVSSGGQCAYSACPIFTKIQGTCYRGKAKCCK
>ID09070-NO
DHYNCVSSGGQCLYSACPIFTRIQGTCYRGRARCCR
>ID09071-NO
DIEKLKEAIRDTNKAVQSVQSSIGNLIVAIKSVQD
>ID09072-NO
DILRG
>ID09073-NO
DIQKLVGKLNWASQIYPGIK
>ID09074-NO
DISGINASVVNIQKEIDRLNEVAKNLNESLIDLQELGKYE
>ID09077-NO
DITLNNSVALDPIDISIELNKAKSDLEESKEWIRRSNQKLDSIGN
>ID09078-NO
DIWDWICEVLSDFK
>ID09079-NO
DKEGTLDFLECGSPHSAVPRWVFSLSCVPRCLGQENGGV
>ID09080-NO
DKEGTLDFLECGSPHSAVPRY
>ID09081-NO
DKFSTNEEFSKIAVVASKKVGKAVVRNRSKRILRALQKYIFLEKNLKWGL
>ID09083-NO
DLADQLIHLYYFDCF
>ID09085-NO
DLEAVPFVNRTTPFTIRGPL
>ID09087-NO
DLFQVIKEKLKELTGGVIEGIQ
>ID09090-NO
DLHTHAQ
>ID09091-ABP_neg
DLIWKLLSKAQEKFGKNKSRSFQLFGSPPGQR
>ID09092-NO
DLKGKYVQIP
>ID09095-NO

DLPECCSATELELDSGKQTS
>ID09096-NO
DLPNYNWNSFGLRF
>ID09097-ABP_both
DLQCAETCVHSPCIGPCYCKHGLICYRN
>ID09098-NO
DLSLDFEKLNVTLLDLTYEMNRIQDAIKKLNESYINLKE
>ID09099-NO
DLSNQINSINKSLKSAEDWIADSNFFANQARTAK
>ID09100-NO
DLVKWIIDTVNKFTKK
>ID09102-NO
DLYVGSDLEIGQHRTKIEEL
>ID09103-NO
DMELKPANAATRTSR
>ID09104-NO
DMSSDLERDHRPHVSMPQNAN
>ID09105-NO
DNATVAAGHATLREH
>ID09106-NO
DNFLTGLGHRS
>ID09108-NO
DNSEMLAPPPRPEEFTSAQQLRQYLAALNEYYSIMGRPRF
>ID09109-NO
DPCYEVCLQQHGNVKECEEACKHPVE
>ID09113-NO
DPKPKKNKKPKNPTP
>ID09114-NO
DPTARLQLEARLQHLVAEILEREQSLALHA
>ID09117-NO
DQAEHLKTAVQMAVFIHNYKA
>ID09118-NO
DQYLESVKKIHKRLDV
>ID09119-NO
DRCSQQCQHHRDPDRKQQCMRECRRH
>ID09120-NO
DREINNYTSLIHSLIEESQNQQEKNEQELLELDKWA
>ID09121-NO
DRLRQKVAIQRLGLAVSRKVGNAVVRNRIKRRLRETDVLVMGAYL
>ID09122-NO
DRLYSFGL
>ID09123-NO
DRMPCRNFFWKTFSSCK
>ID09127-NO
DSGCFGRRLDRIGSLSGLGCNVLRRY
>ID09128-NO
DSGLLDYTEVQRRNQ
>ID09129-NO
DSGPYKMEHFRWGSPPKD
>ID09130-NO
DSLKNKSEFDKLGLSVSKKVGNAVKRNLIKRRLRSCQALVFLEKHFLEML

>ID09133-NO
DSWMDEVIKLCGRELVRAQIAICGMSTWS
>ID09134-NO
DSYYY
>ID09135-NO
DTHTTGGVAGRDTLRFTGFFSFGPKQK
>ID09138-NO
DTRACDVIALLCHLNT
>ID09139-NO
DTRACDVIALLECHLNT
>ID09140-NO
DTRACDVIPLL
>ID09141-NO
DTRAPLAI
>ID09142-ABP_both
DTTPCGESCVWIPCVSSIVGCSCQNKVCYQN
>ID09143-NO
DVAARLRAGFGAVGAGTAEETRRMLHRAFDTLA
>ID09144-NO
DVAHEILNEAYRKVLDQLSARKYLQSMVARGMGENLAAAAVDDRAPLT
>ID09145-NO
DVAHEILNEAYRKVLDQLSARKYLQSVVARGAGENLGGSAVDDPAPLT
>ID09146-NO
DVAHGILNEAYRKVLDQLSAGKHLQSLVARGVGGSLGGGAGDDAEPLS
>ID09147-NO
DVAHGILNKAYRKVLDQPSARRSPADAHGQGLGWDPGGSADDDSEPLS
>ID09148-NO
DVDLGDISGINASVVNIQKEIDRLNEVAKNLNESLIDLQELGKYEQYI
>ID09149-NO
DVILMCFSIDSPDSLENI
>ID09151-NO
DVKQLTEAVQKITTESI
>ID09152-NO
DVLAGLSSSCCEWGCSKSQISSLC
>ID09153-NO
DVMAVSTCVPVAADN
>ID09155-NO
DVREEEQLGERATGLNLNI
>ID09157-NO
DVSFSLSGGGTASYEK
>ID09158-NO
DVSTPPTVLPDNFPRYPVGKFFQYDTWKQSTQRL
>ID09159-NO
DVSTSQAVLPDDFPRYPVGKFFKFDTWRQSAGRL
>ID09162-NO
DVTFSMLGANGATYYQFF
>ID09163-NO
DWEYHAHPKPNSFWT
>ID09164-NO
DWVAVKQSYF
>ID09165-NO

DWVRWI
>ID09167-NO
DYPKLTFTTS
>ID09168-NO
DYQAKLAAYQKEL
>ID09169-NO
DYTEVQRRNQLHDLR
>ID09171-ABP_neg
EAKPEAKPGNNRPVYIPQPRPPHPRI
>ID09174-NO
EALMWEGF
>ID09175-NO
EALYNSEDLYEETSDSDD
>ID09176-NO
EARKLNPNAIASVTV
>ID09178-ABP_both
EATKCFAWQRNMRKVRGPPVSCIKR
>ID09179-ABP_both
EATKCFQWKRNMRKVRGPPVSCIKR
>ID09180-ABP_both
EATKCFQWQRAMRKVRGPPVSCIKR
>ID09182-NO
EAVRHFPRIWLHSLG
>ID09184-NO
ECCEDGWCCTAAP
>ID09185-NO
ECCHRQLLCCLRFV
>ID09186-NO
ECCNPACGRHYSC
>ID09187-NO
ECGKFMWKCKNSNDCCKDLVCSSRWKWCVLASPF
>ID09188-NO
ECKGFGKSCVPGKNECCSGYACNSRDKWCKVLL
>ID09189-NO
ECLGFGKGCNPSNDQCCKSSNLVCSRKHRWCKYEI
>ID09192-NO
ECRYLFGGCSSTSDCCKHLSCRSDWKYCAWDGTFS
>ID09193-NO
ECRYWLGGCSAGQTCCKHLVCSRRHGWCVWDGTFS
>ID09194-NO
ECVPENGHCRDWYDECCEGFYCSCRQPPKCICRNNN
>ID09196-NO
EDCSTPSSGSFLDR
>ID09200-NO
EDVSAGEDCGPLPEGGPEPRSDGAKPGPRE
>ID09206-NO
EEAAKKLEEAAKKLEEAAKKLEEAAKKLWASLWNWF
>ID09207-NO
EEEEAAAKWKLFKKIPKFLHLAKKF
>ID09208-NO
EEEEEEEEEAEAGEEAVPEEEGPTVVLNPHPSL

>ID09209-NO
EEEGSANRRPEDQELESLSAIEAELEKVAHQLQALRR
>ID09210-ABP_pos
EEKDPVMQYWTCGYRGLCRRFCYAQEYIIGHHGCPRRYRCCAMRF
>ID09212-NO
EELAKKAEELAKKAEELAKKAEELAKKAWASLWNWF
>ID09215-NO
EEMKKSDEEIKKYIEEIKKVEEESKYDEE
>ID09216-NO
EEPHKAASAEGKK
>ID09217-NO
EERKKLGEEIKKEAEEAKKQIEETKKNDEE
>ID09218-NO
EESQNQQEKNEQELLELDKWASLWNWFNITNWLWLI
>ID09219-NO
EEYAYSHQLSRADIT
>ID09220-NO
EFKRCWKGQGACQTYCTRQETYMHLCPDASLCCLSYALKPPPV
>ID09221-NO
EFVLATGDFVYMSPF
>ID09222-NO
EFWSLAAPQRF
>ID09223-NO
EGCCSNPACRTNHPEVCD
>ID09225-NO
EGRERDHELRHRRHHHQSPK
>ID09226-NO
EICTEMEKEGKISKIGPENP
>ID09227-NO
EIGDEENSAKFPI
>ID09229-NO
EINCTRPNNNTRKSIHIGPGRAFYTTGEIIGDIRQAHCNIS
>ID09230-NO
EINCTRPNNNTRKSIRIQRGPGRAFVTIGKIGNMRQAHCNIS
>ID09232-NO
EIWATLFKKATRQCRRGRIW
>ID09234-ABP_both
EKCLRWQWRMRKYGG
>ID09236-NO
EKKPPRPPQWAVGHFMM
>ID09240-NO
ELATNEIDYPQEEGALNQQDKKDGSYKMSHFRWSSPPAS
>ID09241-NO
ELATNEVNHPQEDSALIQQKKKDGSYKMKHFRWSSPPAG
>ID09243-NO
ELEGEQPDGLEQVLEPDTEKADGPYRVEHFRWGNPPKD
>ID09244-NO
ELEGERPLGLEQVLESDAEKDDGPYRVEHFRWSNPPKD
>ID09245-NO
ELELAENREILK
>ID09248-NO

ELKNEAVRHFPRIWL
>ID09250-NO
ELLFDGTNPSTEEMGDDFRSGLCPFDTSPV
>ID09254-NO
ELQREESPTGPPGSIRT
>ID09255-NO
ELQREESPTGPPGSIRTWFQRIPLGWFH
>ID09256-NO
ELQREESPTGPPGSIRTWFQRIPLGWFHCTYQK
>ID09259-NO
ELRLVCMGQL
>ID09261-NO
ELSEWGVPCVTCILDRRPAS
>ID09262-NO
ELSNIKENKCNGTDAKVKLIKQELDKYKNAVTELQ
>ID09264-NO
EMLKKKEVKMERKT
>ID09265-NO
EMRISRIILDFLFLRKK
>ID09267-ABP_neg
ENFFKEKERKGQRIRDAIISRRPRVETLAQAQKIIKGGD
>ID09268-NO
ENGLHNRSLNPR
>ID09269-NO
ENKCNGTDAKVKLIKQELDKYKNAVTELQLLMQST
>ID09272-NO
EPCTVGHRRYFTFGG
>ID09273-NO
EPDEICRARMTHKEFNYKSNVCNGCGDQVAACEAECFRNDVYTACHEAQK
>ID09275-NO
EPEPSYFNDCGSNGGSCTRGYCSYSNRLPYTCSLGRTCCRLAYV
>ID09276-NO
EPEPSYILDCRTNGGRCVTGYCSNTLPYSCGGGAICCRHAYG
>ID09277-NO
EPYNEWTLELLEELK
>ID09278-NO
EQCGRQAGGATCPNNLCCSQYGY
>ID09281-NO
EQSRKPPNPTPPPPG
>ID09282-NO
EQVDKLVSAGIRKVLFLDGI
>ID09283-NO
EQVLKAVTNVLSPVFPGGET
>ID09284-NO
ERGMT
>ID09285-NO
ERLRGSCRVRRFLATFRRGYGKAVARNRARRLSKELVDLVLLLCVL
>ID09287-NO
ERLRLRRDFLRLGIVVKRKFGKATRRNKLKRWVREIDIVVVREKL
>ID09290-NO
ERTEESWGRRFWRRGEAC

>ID09291-NO
ESAIRKAILGHIVSP
>ID09292-NO
ESGGLMSQQCCHVGCSRRSIAKLYC
>ID09293-NO
ESGRIKKEEFAEIMKICSTIEELRRQK
>ID09295-NO
ESQNQQEKNEQELLELDKWASLWNWFNITNWLWLIK
>ID09296-NO
ESTHPRISSEVHIPL
>ID09297-NO
ESVKITCARPYQNTRQRTPIGLGQSLYTTRSRSIIGQAHCNIS
>ID09298-NO
ESVVINCTRPNNNTRRRLSIGPGRAFYARRNIIGDIRQAHCNIS
>ID09299-NO
ETFSDLWKLL
>ID09300-NO
ETGSVRFPFHRCGTGPRLTK
>ID09301-NO
ETPDCFWKYCV
>ID09302-NO
ETVPVKLKPGMDGPKVKQWP
>ID09303-NO
ETWRTEAPSATGQASSLLGGRLLGQ
>ID09304-NO
EVDEMLRSEYGGSFR
>ID09305-NO
EVEPEGFHYVSELSDGTYETAEGK
>ID09306-ABP_both
EVEPSDTIENVKAKIQ
>ID09308-NO
EVIDKINAKGVCRST
>ID09309-NO
EVKKQR
>ID09310-NO
EVRRRRRRPPCEDVNGQCQPRGNPCLRLRGACPRGSRCCMPTVAAH
>ID09311-NO
EVSEALGGAGLTGGFYEP
>ID09312-NO
EVSHPKVG
>ID09313-NO
EVVLFLLNVF
>ID09315-NO
EWDREINNYTSLIHSLIEESQNQQEKNEQE
>ID09318-NO
EWRKKRYSTQW
>ID09319-NO
EWTLELLEELKNEAV
>ID09320-NO
EWYNQTKDLQQKFYEIIMDIEQNNVQGKKGIQQLQ
>ID09326-NO

FAAGRK
>ID09327-ABP_both
FACRRWQWRMKKLGA
>ID09328-NO
FADIDTVIHADANAA
>ID09329-NO
FAEALPSDEEGESYSKEVPEME
>ID09330-NO
FAFAKIIAKIAKKII
>ID09331-NO
FAFGKGIGKIGKKGL
>ID09332-NO
FAFGKGIGKVGKKLL
>ID09333-NO
FAIAIKAIKKAIKKIKKAIKKAI
>ID09334-NO
FAIKWEYVLLLFLL
>ID09335-NO
FAKAIAKIAFGKGIGKVGKKLL
>ID09336-NO
FAKALAKLAKKLL
>ID09337-NO
FAKALKALLKALKAL
>ID09338-NO
FAKFLAKFLKKAL
>ID09340-NO
FAKIIAKIAKIAKKIL
>ID09341-NO
FAKIIAKIAKKI
>ID09342-NO
FAKKALKALKKL
>ID09343-NO
FAKKLAKALL
>ID09344-NO
FAKKLAKKAKLAKKL
>ID09345-NO
FAKKLAKKLAKAAL
>ID09346-NO
FAKKLAKKLAKAL
>ID09348-NO
FAKKLAKKLAKLL
>ID09349-NO
FAKKLAKKLKKLAKKLAKKWKL
>ID09350-NO
FAKKLAKKLKKLAKKLAKLAKKL
>ID09353-NO
FAKKLAKKLKKLAKKLIGAVLKV
>ID09354-NO
FAKKLAKKLKKLAKLALAK
>ID09356-NO
FAKKLAKKLL

>ID09357-NO
FAKKLAKLAKKALAL
>ID09358-NO
FAKKLAKLAKKL
>ID09359-NO
FAKKLAKLAKKLAKAL
>ID09361-NO
FAKKLAKLAKKLLAL
>ID09362-NO
FAKKLAKLALKLAKL
>ID09364-NO
FAKKLKKLAKLAKKL
>ID09365-NO
FAKKLLAKALKL
>ID09366-NO
FAKLA
>ID09369-NO
FAKLF
>ID09370-NO
FAKLFAKAFKKAL
>ID09372-NO
FAKLLAKAFKKAL
>ID09373-NO
FAKLLAKALKKAL
>ID09374-NO
FAKLLAKALKKFAL
>ID09376-NO
FAKLLAKALKKL
>ID09377-NO
FAKLLAKALKLKL
>ID09378-NO
FAKLLAKFLKKAL
>ID09379-NO
FAKLLAKKLL
>ID09381-NO
FAKLLAKLAKAKA
>ID09382-NO
FAKLLAKLAKAKG
>ID09384-NO
FAKLLAKLAKK
>ID09385-NO
FAKLLAKLAKKAA
>ID09386-NO
FAKLLAKLAKKAL
>ID09387-NO
FAKLLAKLAKKEL
>ID09388-NO
FAKLLAKLAKKFAL
>ID09389-NO
FAKLLAKLAKKGL
>ID09395-NO

FAKLLALALKLKL
>ID09397-NO
FAKLLKLAAKKLL
>ID09398-NO
FAKLWAKLAFGKGIGKVGKKLL
>ID09399-NO
FAKLWAKLAKKL
>ID09401-NO
FALAAKALKKLAKKLKKLAKKAL
>ID09402-NO
FALAKALKKAL
>ID09403-NO
FALAKKALKKAKKAL
>ID09404-NO
FALAKLAKKAKAKLKKALKAL
>ID09405-NO
FALALKA
>ID09406-NO
FALALKAKKL
>ID09408-NO
FALALKALKKA
>ID09409-NO
FALALKALKKAL
>ID09412-NO
FALALKALKKLKKALKKAL
>ID09413-NO
FALALKKALKALKKAL
>ID09414-NO
FALALKLAKKAL
>ID09416-NO
FALALKLKKL
>ID09417-NO
FALKALKK
>ID09418-NO
FALKALKKAL
>ID09419-NO
FALLKALKKAL
>ID09421-NO
FALLKL
>ID09423-NO
FAVAVKAVAVKAVAVKAVKKAVKKVKKAVKKAVKKKK
>ID09424-NO
FAVNPGLLEAAAGCRQIL
>ID09425-NO
FAVNPGLLETSEAAAQIL
>ID09426-NO
FAVNPGLLETSEGCRAAA
>ID09427-NO
FAVNPGLLETSEGCRQIL
>ID09428-NO
FCHNSISCMMGGDSTCNNVCVRQGNPNGGRCLPRDGCPGYDICACYPNN

>ID09429-NO
FCLEGGCLVALGCTICTD
>ID09430-NO
FCQAVGWGDPITHWSHGQNQ
>ID09434-NO
FDEMEECASHLPYA
>ID09435-NO
FDEMEECSQHLPYA
>ID09436-NO
FDEMEESSSHLPYI
>ID09437-NO
FDSQQGWFEGWFNRS
>ID09438-NO
FDVVKHIASAV
>ID09439-NO
FEDRAPVPFEEVIDK
>ID09440-NO
FELVDWLETNLGKILKSKSA
>ID09443-NO
FESKILNASKELDKEKKVNTALSFNSHQDFAKAYQNGKI
>ID09445-ABP_both
FFCFKGTPCG
>ID09447-ABP_both
FFFHIIKGLFHAGRMIHGLV
>ID09448-ABP_both
FFFHIIKGLFHAGRMIHGLVNRRRHRH
>ID09449-ABP_both
FFGSIIGALAKGLPSLISLIKK
>ID09450-ABP_both
FFGSMIGALAKGLPSLISLIKK
>ID09452-NO
FFIFPNYTIVSDFGRPNAA
>ID09453-NO
FFIILLTIDRYLAVV
>ID09454-ABP_both
FFIYVWRRR
>ID09456-ABP_both
FFLLSLIPSAISAIKKI
>ID09457-ABP_both
FFSTSCRSGC
>ID09460-NO
FGFLPIYRRPAS
>ID09461-NO
FGGASCCLYCRCHIDHPNPKGFCDLKGKY
>ID09462-NO
FGGFTGARKSARKLANQ
>ID09463-NO
FGKGIGKVGKKLL
>ID09464-NO
FHCQRTQSQPGSWFRAISSWKQ
>ID09465-NO

FHFEVFNFVPCSICSNNPTCWAICKRIPNKKPGKK
>ID09467-NO
FHNHGAA
>ID09468-NO
FHNHGAP
>ID09469-NO
FHNHGAT
>ID09471-NO
FHNHGKQ
>ID09472-NO
FHNHGST
>ID09474-NO
FHPHE
>ID09476-NO
FHQNWPS
>ID09477-NO
FHRCGAGPKLTKDLEAVP
>ID09478-NO
FHRKKGRGKHK
>ID09480-NO
FIGAIANLLSKIF
>ID09484-ABP_both
FIHHIIGALGHLF
>ID09486-NO
FILAFLGWIGAIVSTALP
>ID09487-ABP_neg
FIRWRFRWWRWRR
>ID09489-NO
FKAEYQSPSVGQSKGYFLFRPRN
>ID09491-ABP_both
FKARRWQWRM
>ID09492-ABP_both
FKARRWQWRMKKLGA
>ID09493-ABP_both
FKCARWQWRMKKLGA
>ID09494-ABP_both
FKCFRWQWRMKKLGA
>ID09496-ABP_both
FKCRAWQWRMKKLGA
>ID09497-ABP_both
FKCRRWAWRMKKLGA
>ID09498-ABP_both
FKCRRWQWAMKKLGA
>ID09499-ABP_both
FKCRRWQWR
>ID09500-ABP_both
FKCRRWQWRAKKLGA
>ID09502-ABP_both
FKCRRWQWRMK
>ID09507-ABP_both
FKCRRWQWRMKKLGAPSITCVRRAFALECITIRA

>ID09509-ABP_both
FKCWRWQWRWKKLGA
>ID09510-ABP_both
FKFGSFIKRMWRSKLAKKLRAKGKELLRDYANRVLSPEEEAAAPAPYPA
>ID09511-NO
FKIVHVKVR
>ID09512-NO
FKIVWWRRR
>ID09513-ABP_both
FKKALHLFKPIKKFLKWK
>ID09516-NO
FKKIRVRL
>ID09517-ABP_neg
FKKVIVIRRWFI
>ID09518-NO
FKLAFKLAKKAFL
>ID09519-NO
FKLFKKILKVL
>ID09520-NO
FKLPIQKETWETWWTEYWE
>ID09521-NO
FKLPLGINITNFRAILTAFS
>ID09523-NO
FKPSSPPSITLW
>ID09525-NO
FKRKRWWGI
>ID09526-NO
FKRLAKIKVLRLAKIKR
>ID09527-NO
FKRSWVQIV
>ID09530-NO
FKVKAKVKAKVKAKVKAKKKK
>ID09531-NO
FLAAARIAKRVAKKARKLAKRAARKRK
>ID09533-NO
FLDSKAELEKARKILSEVGRWY
>ID09535-NO
FLFAFRIFKRVFKKFRKLFKRAF
>ID09537-ABP_both
FLFKLIPKAIKGLLKAFK
>ID09538-NO
FLFQPQRF
>ID09539-ABP_both
FLGAIAQALTSLLGKL
>ID09540-ABP_both
FLGALGNALSRVL
>ID09541-ABP_both
FLGFVGQALNALLGKL
>ID09544-NO
FLGWLFKWASKVL
>ID09546-ABP_both

FLKGIIDTVSNWL
>ID09547-NO
FLKVPAQNAISTTFPYT
>ID09549-ABP_pos
FLPFFASLFSGLF
>ID09550-ABP_both
FLPGLECVSGLKIVPTVFCAITRIC
>ID09551-ABP_both
FLPGLECVW
>ID09553-ABP_pos
FLPILINLIHGKLL
>ID09555-NO
FLPIVTGLLSSLL
>ID09556-ABP_neg
FLPKKFRWWKYRK
>ID09557-ABP_both
FLPLIGKCCCCCCILGTI
>ID09560-NO
FLPLLILGSLLMTPPVIQAIHDAQR
>ID09561-NO
FLPLVLGALSGILPKILGK
>ID09562-NO
FLPPCAYKGTC
>ID09563-NO
FLPVIAGAANFLPKLFCAISKKC
>ID09564-NO
FLQDSKAELEKARKILSEVG
>ID09565-ABP_both
FLQHIIGALGHLF
>ID09566-ABP_both
FLQHIIGALSHFF
>ID09567-ABP_both
FLQHIIGALTHIF
>ID09568-NO
FLREDLAF
>ID09569-NO
FLRQNLAF
>ID09570-ABP_both
FLRSLLRGAKAIYRGARAGWRG
>ID09571-ABP_pos
FLSGLIGGLAKMLGK
>ID09576-NO
FLSLIPHIVSGVASLAIHFG
>ID09581-NO
FLYTAFAMQELGCNQNQFFC
>ID09582-NO
FNAPFDVGIKLSGVQYQQHSQAL
>ID09583-NO
FNKAISQIQESLTTTSTALGKLQDVVNQNAQALNTLVKQL
>ID09585-NO
FNMTKAVEMVNIAGNWSCTS

>ID09587-NO
FNVALDQVFESIENSQALVDQSNRILSSAE
>ID09588-NO
FNVALDQVFESIENSQALVDQSNRILSSAEKGN
>ID09590-NO
FNWRCCLIPACRRNHKKFC
>ID09592-NO
FPESSQDLISSLLEK
>ID09596-NO
FPRPRICNLACRAGIGHKYPFCHCR
>ID09603-ABP_both
FPWSCLSLSGVCRKVCLPTELFFGPLGCGKGSLCCVSHFL
>ID09606-NO
FQKPFTGEEVEDFQDDDEIPTII
>ID09609-ABP_both
FQWQRNMRKV
>ID09612-NO
FRFKIKFRLKFRFKARFKFRAKFRA
>ID09613-NO
FRKKYRVRR
>ID09614-NO
FRKSKEKIGKEFKRIVQRIKDFLRNLV
>ID09615-NO
FRPKVTITIQGSARF
>ID09616-NO
FRRRYRVYR
>ID09617-ABP_both
FRRWWKWFK
>ID09618-NO
FRVDEEFQSPFASQSRGYFLFRPRN
>ID09619-NO
FRVKREKDFKRVGLSVSKKLGNAVTRNQIKRRIRHLVDFVVMEKNL
>ID09621-NO
FSEFMRQYLVLSMQSSQ
>ID09622-NO
FSGSVNQACSGFGWK
>ID09623-NO
FSSDAISTTFTTNLT
>ID09624-NO
FSVPLDEDFRKYTAFTIPSI
>ID09625-NO
FTAGIETSFSCSQNGGFCISPKCLPGSKQIGTCILPGSKCCRKK
>ID09627-ABP_both
FTCDVLSAEGGFRGVSIKLNHAACAAHCLYLKKRGGYCNDKAVCVCRK
>ID09628-ABP_both
FTCDVLSAEGSFRGVSVKLNHSACATHCLFLKKRGGYCNNKAICVCRN
>ID09629-ABP_both
FTCDVLSVEAKGVKLNHAACGIHCLFRRRTGGYCNKKRVCICR
>ID09630-NO
FTFGGGYVYFEEYAY
>ID09631-NO

FTGCMC
>ID09634-NO
FTLSLDVPTNIMNILFNIDKAKNLRAKAAANAQLMAQI
>ID09636-NO
FVIWKYIRV
>ID09637-NO
FVPIFTYGELQRMQEKERSKGQ
>ID09642-NO
FVYRGWRRK
>ID09644-NO
FWFRIRKLK
>ID09645-NO
FWFTLIKTQAKQPARYRRFC
>ID09646-ABP_both
FWGFLGKLAMKAVPSLIGGNKK
>ID09647-ABP_both
FWGLKGLKGPGKFSKKL
>ID09648-ABP_both
FWGLKGLKKFSKKL
>ID09649-NO
FWNWLSAWIKKTYEEIKKTYEEIKKTYEEIERDWEMV
>ID09650-ABP_neg
FWQRNIRIRR
>ID09651-ABP_neg
FWQRNIRKWR
>ID09652-ABP_neg
FWRIRKWR
>ID09653-NO
FWRRDSRATAADFTKKDYTATLGRPFFLFRPRN
>ID09660-NO
FYHSKRRLIFSKRKP
>ID09662-NO
GAETFYVDGAANRETKLGKA
>ID09663-NO
GAFLKCGESCVYLPCLTTVVGCSCQNSVCYRD
>ID09664-NO
GAHHAADGC
>ID09665-NO
GAIVSTALPQWRIYSYAG
>ID09666-NO
GAKALTEVIPLTEEAEC
>ID09667-NO
GAKKGAKKGKKGAKKGAKGAGAKGAGAFKKKK
>ID09668-NO
GAKTETLVIPETELEAC
>ID09670-NO
GAPASVLGSRPFDYGLKWQS
>ID09672-NO
GAPLGGAARALAHGVRVL
>ID09673-NO
GASLSSPAESSGSPQRRGLSAPSSRQIPAPQGAVLVQREKDLPNYNW

>ID09674-NO
GATVVQFEQPRRCPT
>ID09675-NO
GAVPCGETCVYLPCITPDIGCSCQNKVCYRD
>ID09676-NO
GAVVNDL
>ID09677-NO
GAWYKGRARPVSAVA
>ID09681-NO
GCCGSYPNAACHPCSCKDRPSYCGQ
>ID09682-NO
GCCSDKRCAWRC
>ID09683-NO
GCCSDPPCRNKHPDLC
>ID09686-NO
GCCSDPRCNYDHPEIC
>ID09687-NO
GCCSDPRCRYRC
>ID09688-NO
GCCSHPACAGNNQHIC
>ID09691-NO
GCCSHPVCSAMSPIC
>ID09692-NO
GCCSLPPCAANNPDYC
>ID09693-NO
GCCSLPPCALSNPDYC
>ID09694-NO
GCCSNPVCHLEHSNLC
>ID09695-NO
GCCSTPPCAVLYC
>ID09698-NO
GCKGFGDSCTPGKNECCPNYACSSKHKWCKVYL
>ID09700-ABP_both
GCKKFRRLKWKYKGKFWFWCG
>ID09701-NO
GCKLTFWKCKNKKECCGWNACALGICMPR
>ID09702-NO
GCLGEGEKCADWSGPSCCDGFYCSCRSMPYCRCRNNS
>ID09703-NO
GCMKEYCAGQCRGKVSQDYCLKHCKCIPR
>ID09704-NO
GCMPEYCAGQCRGKVSQDYCLKNCRCIR
>ID09706-NO
GCPWDPWC
>ID09707-NO
GCPWEPWC
>ID09708-NO
GCPWQPWC
>ID09709-NO
GCTQWINNIHGRICVRN
>ID09710-NO

GCVLYPWC
>ID09711-NO
GCVPCVREGNASRCWVAV
>ID09712-NO
GDCLPHLKLCKENKDCCSKKCKRRGTNIEKRCR
>ID09713-NO
GDCLPHLKRCKADNDCCGKKCKRRGTNAEKRCR
>ID09714-NO
GDCPWKPWC
>ID09715-ABP_both
GDFFRKSKEKIGKEFKRIVQRIKDFLRNLVPRTES
>ID09717-NO
GDISGINASVVNIQKEIDRLNEVAKNLNESLIDLQELGKY
>ID09719-NO
GEDLA
>ID09720-NO
GEDLAF
>ID09721-NO
GEEELAEKAPEFARELAN
>ID09722-NO
GEEELQENQELIREKSN
>ID09724-NO
GEETLKKWEEQTKKLEEKFKKIEENIKKNEEQVKKGEEQLKK
>ID09726-NO
GELGRIPSDTYDLAVGALHCPFYLVSGLVYLDG
>ID09727-NO
GELGRLVYLLDGPGYDPI
>ID09728-NO
GELGRLVYLLDGPGYDPIHCDVVTRGGSHLFNF
>ID09730-NO
GELGRPVYVLGDPGYYAT
>ID09731-NO
GELGRPVYVLGDPGYYATHCIYATTNDALIFSV
>ID09732-NO
GENNELRLTRDAI
>ID09734-NO
GEWYNQTKDLQQKFYEIIMDIEQNNVQGKKGIQQL
>ID09736-NO
GFADLFGKAVDFIKSRV
>ID09737-NO
GFADLFGKVANLIKS
>ID09738-NO
GFCRCLCRRGVCRCLCTK
>ID09741-NO
GFFALIPKIISSPLFKTLLSAVGSAL
>ID09742-NO
GFFFP
>ID09743-ABP_both
GFFLNALKNFAKTAGKRLKSLLNHASCKLSGQC
>ID09745-NO
GFGCPFNQYQCHSHCLSIGRRGGYCGGSFKTTCTCYN

>ID09746-ABP_both
GFGCPLNERECHSHCQSIGRKFGYCGGTLRLTCICGKE
>ID09750-ABP_both
GFLEKLKTGAKDFASAFVNSIKGT
>ID09751-ABP_both
GFLNTAMNTVTNLAGTLMDKAKCKIRGC
>ID09755-NO
GFMDTAKNAAKNVAVTLLDKLKCKITGGC
>ID09756-NO
GFMDTAKNAARNVAVTLLDKLKCKITGGC
>ID09757-NO
GFMDTAKNVAKNMAGNLLDNLKCKIIKPC
>ID09760-ABP_both
GFMSTASNVLTNVAGTVMDKLKCKFTGAC
>ID09762-NO
GFTPFRIY
>ID09763-NO
GFTWKKKRR
>ID09764-ABP_both
GFWSSVWDGAKNVGTAIIKNAKVCVYAVCVSHK
>ID09776-NO
GGASCCLYCRCH
>ID09777-NO
GGCLPHNRFCNALSGPRCCSGLKCKELSIWDSRCL
>ID09778-NO
GGFLIAYQPLLSNTL
>ID09779-NO
GGGEDEVGEEDEEAAEAEAEAEEAERARQNALL
>ID09780-NO
GGKMLYNKVTKQLSYCTDPL
>ID09782-NO
GGSFRFSSDAISTTF
>ID09783-NO
GGSLYSFGL
>ID09784-ABP_both
GGVCPKILKKCRRDSDCPGACICRGNGYCGSGSD
>ID09785-NO
GGWTQWWWTAFY
>ID09786-NO
GGYCSGIIKQTCTCYRN
>ID09788-NO
GHACYRNCWREGNDEETCKERC
>ID09789-NO
GHKARVLAEAMSQVTNPATIM
>ID09790-NO
GHRRYFTFGGGYVYF
>ID09791-NO
GHRYSQFMGIFEDRA
>ID09792-NO
GIADILKGLLG
>ID09793-NO

GICRCICGRGICRCICGRIGGRVPGVGVPGVGHHHHHH
>ID09794-NO
GICYVLTCNSLCFPKLGRCSYNTCYCY
>ID09796-NO
GIFDKLAKEISIW
>ID09797-NO
GIFSKKAGKGFKKKSPKAPTPKATKMASECSEPGQALQEKKKR
>ID09798-ABP_both
GIFSVLNEVCKKNDYKPEICAHFSQNKP
>ID09799-NO
GIGAILKVLATGLPTLISWI
>ID09800-NO
GIGALSAKGALKGLAKGLAQHFAN
>ID09804-NO
GIGAVLKVLTTGLPALISWIRKKRQQ
>ID09808-NO
GIGGALLSAGKAALKGLAKVLAEKYAN
>ID09809-NO
GIGGVLLCAGKAALKGLAKVLAEKYAN
>ID09821-ABP_pos
GIGLFLHSAGLFGLAFVGEIMKS
>ID09824-NO
GIGVTQNVLYENQKQIANQF
>ID09825-NO
GIIKKI
>ID09826-NO
GIIKKIIIKKI
>ID09827-NO
GIIKKIIIKKIIIKKI
>ID09828-NO
GIIKKIIIKKIIIKKIIIKKI
>ID09830-NO
GIKKFLHIIWKFIKAFVGEIMNS
>ID09831-NO
GIKYFSMVGNWAKVLVVL
>ID09832-ABP_both
GILDTLKQFAKGVGKWLVKGAAQ
>ID09833-ABP_both
GILSSFKGVAKGVAKNVAAQLLDTLKCKITGC
>ID09835-ABP_both
GIMDTVKNAAKNLAGQLLDKLKCSITAC
>ID09836-NO
GINASVVNIQKEIDRLNEVAKNL
>ID09837-NO
GINASVVNIQKEIDRLNEVAKNLNESLIDL
>ID09838-NO
GINASVVNIQKEIDRLNEVAKNLNESLIDLQELGK
>ID09839-NO
GINASVVNIQKEIDRLNEVAKNLNESLIDLQELGKYE
>ID09847-NO
GIRIIPVIIPGYKKWARLIKRGLSRLGG

>ID09848-NO
GIVDQCCTSICSLYQLENYCN
>ID09849-NO
GIVLIGLKLIPLLANVLR
>ID09850-NO
GIWKKWIKKWLKKLLKKLWKKG
>ID09856-NO
GKINISLCLTGGKMLYNKVT
>ID09858-NO
GKKLALALALALALALALALKKA
>ID09859-ABP_both
GKKYRRFRWKFKGKWFWFG
>ID09864-NO
GKPFYPPPIYPEDM
>ID09865-ABP_both
GKPICGETCFKGKCYTPGCTCSYPICKKD
>ID09866-ABP_both
GKPICGETCFKGKCYTPGCTCSYPICKKN
>ID09873-NO
GKRGDSFRKREFFRTNGERYPEDAAAWTEFQ
>ID09874-NO
GKTKCTASNKNRGIIKT
>ID09875-NO
GKVIDTLTCGFADLMGYI
>ID09876-NO
GKVWDWIKSTAKKLWNSEPVKELKNTALNAAKNLVAEKIGATPSE
>ID09877-ABP_both
GKYGFYTHVFRLKKWIQKVIDQFGE
>ID09880-NO
GLCPFDTSPVVKGKYNTTLLNGSAFYLVCP
>ID09882-ABP_both
GLFDIWAWWRWRR
>ID09883-ABP_both
GLFDIWKKLRWRR
>ID09884-ABP_both
GLFDIWKKWRWRR
>ID09885-ABP_both
GLFDIWKWWRWRR
>ID09890-ABP_both
GLFGRLRDSLQRGGQKILEKAERIWCKIKDIFRG
>ID09893-NO
GLFKALLKLLKSLWKLLLKA
>ID09894-ABP_pos
GLFKVLGSIAKHLLPHVVPVVAEK
>ID09895-ABP_both
GLFRRLRDSIRRGQQKILEKARRIGERIKDIFR
>ID09896-NO
GLFSKFAGKGIKNFLIKGVKHIGKEVGLDVIRTGIDVAGCKIKGEC
>ID09899-NO
GLFTLIKGAAKLIGKTVVKEAGKTGLELMACKITNQC
>ID09900-NO

GLGSVFGRLARIGRVIPKV
>ID09901-NO
GLIGSIGKALGGLLVDVLKPKLQA
>ID09903-ABP_both
GLKDMIKNLAKEAAVKLAGAVINKFSPQPQ
>ID09905-ABP_pos
GLKLRFEFSKIKGEFLKTPEVRFRDIKLKDNRISVQR
>ID09906-NO
GLLCYCGKGHCKRGERVRGTCGIRFLYCCPRR
>ID09910-ABP_both
GLLDFLKAAGKGLVSNLLEK
>ID09911-NO
GLLDGLLGGLGL
>ID09912-NO
GLLDGLLGTLGL
>ID09913-NO
GLLDILKGAAKDLIATGLNALRCKLTKC
>ID09914-NO
GLLDSVKEGLKKAAGQLLDTLKCEISGCTPA
>ID09915-NO
GLLDVVGNVLHSLGL
>ID09916-NO
GLLEALAELLEGLRKRLRKFRNKIKEK
>ID09917-NO
GLLGFVGSLLGGLGI
>ID09918-NO
GLLGMVGSLLGGLGL
>ID09919-ABP_both
GLLKFIKKLL
>ID09922-ABP_both
GLLKRIKTL
>ID09925-ABP_both
GLLPLLSLLGKLL
>ID09927-NO
GLLSGILGAGKHIVCGLGGLC
>ID09928-ABP_both
GLLSGILGAGKKIVF
>ID09929-NO
GLLSGILGAGKNIVCGLSGLLKLESEII
>ID09930-ABP_both
GLLSKVLGVGKKVLCGVSGLVC
>ID09935-ABP_both
GLMDIFKVAVNKLLAAGMNKPRCKAAHC
>ID09938-NO
GLPICGESCVGGTCNTPGCTCTWPVCTRN
>ID09940-ABP_both
GLPTCGETCFKGKCYTPGCSCSYPICKKN
>ID09941-NO
GLPTCGETCTLGKCNTPKCTCNWPICYKD
>ID09943-NO
GLPVCGETCFTGSCYTPGCSCNWPVCNRN

>ID09945-NO
GLQLLGFILAFLGWIGAI
>ID09946-ABP_both
GLRKRLRKFRNKIKEK
>ID09948-NO
GLTGGFYEPLVRRCSELMGR
>ID09949-ABP_both
GLTRLFSVIK
>ID09951-NO
GLVRDNMAKLRERLK
>ID09955-NO
GLWRFWFGDFLT
>ID09958-NO
GMASKAGAIAGKIAKVALKALA
>ID09959-NO
GMASKAGSIVGKIKIALGAL
>ID09960-ABP_both
GMASTAGSVLGKLAKAVAIGAL
>ID09961-NO
GMCTEKFY
>ID09962-NO
GMLKWKNDFFHFLQVWLLISCQNYFVK
>ID09963-NO
GMVTQYHQVLATHQEAIEKV
>ID09966-NO
GNHILSLVQNAPYGLYFIHFSW
>ID09967-NO
GNITLGEWYNQTKDLQQKFYEIIMDIEQNNVQG
>ID09968-ABP_both
GNNKPVYIPRPRPPHPRLV
>ID09969-ABP_both
GNNRPVYIPQPRPPHPR
>ID09970-ABP_both
GNNRPVYIPRPRPPHPRL
>ID09972-ABP_both
GNPLKLFLPSTWVHFFKFLR
>ID09973-NO
GNQGRGNPVRSPLGFGSYTM
>ID09974-NO
GNTPNVSGANPVDCTMYSNK
>ID09976-NO
GPAVFPAENGVQNTESTQE
>ID09980-NO
GPPCCLYGSCRPFPGCYNALCCRK
>ID09981-NO
GPPISLERLDVGTNLGNAIAKLEDAKELLESSDQI
>ID09982-NO
GPPPHHRDYHGP
>ID09983-NO
GPQREPYNEWTLELL
>ID09984-NO

GPRSGPGPWQGGRRKFRRQRPRLSHKGPMPF
>ID09985-NO
GPSFCKADEKPCEYHADCCNCCLSGICAPSTNWILPGCSTSSFFKI
>ID09986-NO
GPSQPTYPGDDAPVEDLIRFYNDLQQYLNVVTRHRY
>ID09987-NO
GPWVWVPAFCQAVGWGDPIT
>ID09988-NO
GQHRTKIEELRQHLLRWGLT
>ID09991-NO
GQLFTFSPRHHWTTQDCN
>ID09992-NO
GQPEEGAPCQVVLQGA
>ID09993-NO
GQPSHDPVPPTT
>ID09994-NO
GQVWEATATVNAIRGSVTPAVSQFNARTAD
>ID09995-NO
GRCCHPACGKYYSC
>ID09996-NO
GRCTKSIPPICFPD
>ID09998-NO
GRDYRTSLTIVQKLKKMVD
>ID10002-NO
GRKKRRQRRRC
>ID10003-ABP_both
GRKKRRQRRRPWQ
>ID10004-NO
GRKRKKRT
>ID10007-NO
GRLQSLQTYVTQQLIRAAEIRASANLAATKMSEC
>ID10008-NO
GRMKG
>ID10009-NO
GRPDGFIHVQGHLQEVDA
>ID10012-NO
GRRRRSVQWCAVSQPEATKCFQW
>ID10014-NO
GRRSTHWRI
>ID10015-NO
GRRVSARMLGDVMAV
>ID10018-NO
GRTTLSTRGPPRGGPG
>ID10021-NO
GSAKVAFSAIRSTNH
>ID10022-NO
GSASCTIAALGSSDRDTV
>ID10023-NO
GSATLCSALYVGDLCGSV
>ID10024-NO
GSCDGFRVCYMH

>ID10026-NO
GSEGPLKPGARIFSFDGKDVLRHPT
>ID10027-NO
GSHTEHTTYAADRFK
>ID10028-NO
GSHWAVGHLM
>ID10029-NO
GSIPCGESCVFIPCISAIIGCSCSNKVCYKN
>ID10030-NO
GSIRTWFQRIPLGWFHCTYQKGKQHCRLRIRQKVEE
>ID10037-NO
GSLLGRMKGA
>ID10043-ABP_both
GSNTGFNFKTLDKE
>ID10044-NO
GSPRTEYEACRVRCQVAEHGVERQRRCQQVCEKRLREREGRRE
>ID10046-NO
GSRNGPGPWQGGRRKFRRQRPRLSHKGPMPF
>ID10047-NO
GSRVQIRCRFRNSTR
>ID10048-NO
GSSFLSPEHQKAQQRKESKKPPAKLQPR
>ID10049-NO
GSSFLSPEHQLAQQRKESKKPPAKLQPR
>ID10052-NO
GSVIKKRRKRMAKKKHRKLLKKTRIQRRRAGK
>ID10053-NO
GSWFRAISSWKQRNRWEWRPDF
>ID10055-NO
GSYYY
>ID10056-NO
GTAALTEVIPLTEEAEC
>ID10057-ABP_both
GTAWRWHYRARS
>ID10059-NO
GTGNI
>ID10060-NO
GTKAATEVIPLTC
>ID10061-NO
GTKAATEVIPLTEEAEC
>ID10062-NO
GTKALAEVIPLTEEAEC
>ID10063-NO
GTKALTAVIPLTEEAEC
>ID10064-NO
GTKALTEAIPLTEEAEC
>ID10065-NO
GTKALTEVAPLTEEAEC
>ID10070-NO
GTKALTEVIPLTC
>ID10075-NO

GTKWATEWAPLTAEAEC
>ID10076-NO
GTKWLTEVWPLC
>ID10078-NO
GTKWLTEWIPLTAEAEC
>ID10079-NO
GTKWLTEWIPLTAEC
>ID10082-NO
GTSCGETCVLLPCLSSVLGCTCQNKRCYKD
>ID10083-NO
GTTVNCIVEEVDARS
>ID10088-NO
GVATSAQITAAVALVEAKQARSDIEKLKEAIRDTN
>ID10091-NO
GVFVFNGTSWFITQRNFFS
>ID10092-NO
GVGSPYVSRLLGICL
>ID10095-ABP_both
GVLDILKGAAKDLAGH
>ID10096-ABP_both
GVLDILKGAAKDLAGHVATKVI
>ID10097-NO
GVLLLILCLPTLVDCIRNCI
>ID10100-NO
GVTQNVLYENQKQIANQFNKAISQIQESLTTTSTALGKLQ
>ID10101-ABP_both
GVVDILKGAAKDIAGHLASKVMN
>ID10102-ABP_both
GVVKVSLRKGESLRARL
>ID10103-NO
GVYYDPSKDLIAEIQKQGQG
>ID10105-NO
GWAGWLLSPRGSRPSWGP
>ID10106-NO
GWFEGWFNRSPWFTT
>ID10107-NO
GWFGKAFRSVSNFYKKHTYIHAGLSAATLLG
>ID10110-NO
GWHTTDLKYNPSRVE
>ID10111-NO
GWIGAIVSTALPQWRIYS
>ID10112-ABP_both
GWLDVAKKIGKAAFNVAKNFI
>ID10113-NO
GWLDVAKKIGKAAFNVAKNFLSPACERLFNKAVNFAAKGIKKAVDLWG
>ID10118-NO
GWMDF
>ID10119-ABP_both
GWRLIKKILRVFKGL
>ID10120-NO
GWRPSSREDSLEAGLPLQV

>ID10121-NO
GWTLNSAGYLLGPHAIDNHRSFSDKHGLT
>ID10122-NO
GWTLNSAGYLLGPHALDSHRSFQDKHGLA
>ID10123-NO
GWTLNSAGYLLGPHAVGNHRSFSDKNGLTS
>ID10124-NO
GWVKPAKLDG
>ID10125-NO
GWWKNWRWW
>ID10127-NO
GWWYKGRARAVSAVA
>ID10130-NO
GYCAEKGIRCDDIHCCTGLKCKCNASGYNCVCRKK
>ID10131-NO
GYFVQDDGEWKFTGSSYYY
>ID10133-NO
GYGCPNDYSCSNYCSSIGRNGGYCGGFLWQTCKCNEKK
>ID10135-NO
GYPEEKKEEEGSANRRPEDQELESLSAIEAELEKVAHQLQALRR
>ID10138-NO
GYRARPKFKAGKR
>ID10140-NO
HADAIFTSSYRRILGQLYARKLLHEIMNRQQGERNQEQRSRFN
>ID10141-NO
HADGSFSDEMNTILDNLATRDFINWLIQTKITD
>ID10142-NO
HADGSFSDEMNTVLDSLATRDFINWLLQTKITD
>ID10144-NO
HADGTYTSDVSTYLQDQAAKDFVSWLKSGRA
>ID10145-NO
HADGVFTSDFSKLLGQLSAKKYLESLM
>ID10146-NO
HADGVFTSDFSKLLGQLSAKKYLESLMGKRVSSNISEDPVPV
>ID10147-NO
HADGVFTSDYSRLLGQISAKKYLESLI
>ID10148-NO
HADGVFTSDYSRLLGQISAKKYLESLIGKRISSSISEDPVPI
>ID10150-NO
HADGVFTSDYSRLLGQLSAKKYLESLI
>ID10152-NO
HALQDTEENPRSFPASQTEAHEDPDEMNED
>ID10155-NO
HAPQDTEENARSFPASQTEPLEDPDQINED
>ID10156-NO
HARIKPTFRRLKWKYKGKFW
>ID10158-NO
HAWNYIF
>ID10159-NO
HCIYATTNDALIFSV
>ID10160-NO

HCKFWC
>ID10161-NO
HCKFWD
>ID10162-NO
HCKFWE
>ID10163-NO
HCKFWG
>ID10166-NO
HCKFWL
>ID10167-NO
HCKFWM
>ID10168-NO
HCKFWN
>ID10169-NO
HCKFWP
>ID10170-NO
HCKFWQ
>ID10171-NO
HCKFWR
>ID10172-NO
HCKFWS
>ID10173-NO
HCKFWT
>ID10174-NO
HCKFWV
>ID10175-NO
HCKFWY
>ID10176-NO
HCSLAYGDASTLVVF
>ID10177-NO
HCTYQKGKQHCRLRIRQKVEE
>ID10178-NO
HDEFERHAEGTFTSDVSSYLEGQAAKEFIAWLVKGRG
>ID10179-NO
HDWTKNITDKIDQIIHDFVDKTLPD
>ID10180-NO
HEDHVGHI
>ID10181-NO
HEFVPLEVYTRHEIK
>ID10184-NO
HFFATSTGDVVYISPFYNGTNRNASYFG
>ID10185-NO
HFLLKLVNLAKKIL
>ID10186-NO
HFLLTKVNLAKKIL
>ID10187-NO
HFLLTLKNLAKKIL
>ID10188-NO
HFLLTLVKLAKKIL
>ID10189-NO
HFLLTLVNKAKKIL

>ID10190-NO
HFLLTLVNLAKKIK
>ID10192-NO
HFLLTLVNLAKKKL
>ID10194-NO
HFLLTLVNLKKKIL
>ID10197-NO
HFWNRPL
>ID10199-NO
HGLASTLTRWAHYNALIRAF
>ID10200-NO
HGNITLGEWYNQTKDLQQKF
>ID10201-NO
HHKRRR
>ID10202-NO
HHLRDRKVFARAAVSISKTKYKLAVERNLIRRQVKALNDVLVKQTIF
>ID10203-NO
HHPHG
>ID10204-NO
HHPHGHHPHG
>ID10205-NO
HHPHGHHPHGHHPHG
>ID10206-NO
HHPHGHHPHGHHPHGHHPHG
>ID10207-NO
HIEHLIA
>ID10208-NO
HISHISMCRWCCNCCKAKGCGPCCKF
>ID10209-NO
HKEDTLAFSEWGSPHAAVPR
>ID10210-NO
HKILGYTVIAMPEVEGEEIQ
>ID10211-NO
HKLSNSEQFRRFGLVVSKAVGNAVTRHRVSRQLRHFHVVLRADVQAAL
>ID10212-NO
HKRKWWRFR
>ID10215-NO
HLEHLLF
>ID10216-NO
HLPWKWPWWPWRR
>ID10217-ABP_both
HLRRINKLLTRIGLYRHAFG
>ID10218-NO
HLVAVIGSYR
>ID10219-NO
HMNASDMEIKSYINMIESVEESSNYDF
>ID10220-NO
HPAGLKKKKSVTVLDVGDAY
>ID10222-NO
HPTWPQKSVWHGSDPNGRRLTESY
>ID10223-NO

HQWRIRVAVRRH
>ID10225-NO
HRIKKNDEFQRIGLSVSKKIGNAVVRNRIKRMIRQIDDFVILKKSL
>ID10226-NO
HRIKRSDEFSRVLSVSKKIGNAVTRNRVKRLIRTISDYVIVKGSL
>ID10227-NO
HRILMAIRQMMT
>ID10229-NO
HRMANMMMNWSPTAALV
>ID10230-NO
HRSLLGRMKGA
>ID10231-ABP_both
HSDGIFTDSYSRYRKQMAVKKYLAAVLGRRYRQRFRNK
>ID10232-NO
HSDGMFTSELSRLQDSARLQRLLQGLV
>ID10233-NO
HSDGTFTSELSRLQDSARLQRLLQGLV
>ID10235-NO
HSHRDFQPVLHLVALNSPLSGGMRG
>ID10236-NO
HSLIEESQNQQEKNEQELLELDKWASLWNWFNITNW
>ID10237-NO
HSLRERKVFTRVAISIAKTKYKLAVQRNLIKRQIRSLEDILVKQKLF
>ID10238-ABP_neg
HSPGGA
>ID10240-NO
HSQGTFTSDYSKYLDSRRAQDFVQWLMNTKRNRNNIA
>ID10241-NO
HTGERDWHLGQGVSI
>ID10243-NO
HTTYAADRFKQVDGF
>ID10244-NO
HVDAIFTTNYRKLLSQLYARKVIQDIMNKQGERIQEQRARLS
>ID10245-NO
HVMLALATVLSIAGAGTGATAI
>ID10246-NO
HVNDMLGRVAIAWCE
>ID10247-NO
HVRRWWRII
>ID10248-NO
HVTTTFAPPPPR
>ID10251-NO
HWSHGQNQWPLSCPQYVYGS
>ID10252-NO
HYRIKPTFRRLKWKYKGKFW
>ID10253-NO
IAEIQKQGQGQWTYQIYQEP
>ID10255-NO
IAVVFKENIAPYKFK
>ID10256-NO
IAWCELQNHELTLWN

>ID10257-NO
ICCNPACGPKYSC
>ID10261-NO
IDLNITMLEDHEFVP
>ID10262-NO
IDRLITGRLQSLQTYVTQQLIRAAEIRASANLAATK
>ID10263-NO
IDRLNEVAKNLNESLIDLQELGKYEQYIKWPW
>ID10264-NO
IDWSKIFEKVKNLV
>ID10265-NO
IEAQQHLLQLTVWGIKQLQARIL
>ID10266-NO
IEEINKKVEEIQKKIEELNKKAEELNKKLEELQKK
>ID10267-NO
IEESQNQQEKNEQELLELDKWASLWNWFNITNWLWL
>ID10268-NO
IEFARLQFTYNHIQR
>ID10269-NO
IEFFA
>ID10270-NO
IEFFT
>ID10275-NO
IELSNIKENKCNGTDAKVKLIKQELDKYKNAVTEL
>ID10277-NO
IEQLIKKEKVYLAWV
>ID10278-NO
IEVTFVNRRGDGAELWYLSA
>ID10279-NO
IFECVFSCDIKKEGKPCKPKGEKKCTGGWRCKIKLCLKI
>ID10284-NO
IFWDCWAPEEPACQDFLGAMIH
>ID10285-NO
IGDLVKWIIDTVNKFTKK
>ID10286-NO
IGIKLLKSKLKAL
>ID10287-NO
IGITQQRRARNGASR
>ID10288-ABP_neg
IGKEFKRIVQRIK
>ID10290-NO
IGNIPQYLKGLLGGILGIGL
>ID10291-NO
IGRFWRRWL
>ID10293-NO
IGVRPGKLDL
>ID10294-NO
IHPWKWPWWPWRR
>ID10297-NO
IIIVIIVILLSLIAVGL
>ID10298-NO

IIKKFLHSIWKFGKAFVGEIMNI
>ID10299-NO
IIKQTCTCYRK
>ID10300-NO
IIKWKRIMI
>ID10301-NO
IIKWWRRWR
>ID10302-NO
IILWWYRRK
>ID10304-NO
IIRILQQLLFIHFRI
>ID10305-NO
IIRLIKWWR
>ID10307-NO
IISYEL
>ID10308-ABP_both
IIYCNRRTGKCQRM
>ID10309-NO
IIYKWRWYW
>ID10310-NO
IKCNCKRHVIKPHICRKICGKN
>ID10311-NO
IKENKCNGTDAKVKLIKQELDKYKNAVTELQLLMQ
>ID10313-ABP_both
IKHQGLPQE
>ID10314-NO
IKKEIEAIKKEQEAIKKKIEAIEKEIEAQQHLLQLTVWGIKQLQARIL
>ID10315-NO
IKKEIEAIKKEQEAIKKKIEAIEKLLQLTVWGIKQLQARIL
>ID10316-NO
IKKEKVYLAWVPAHK
>ID10317-NO
IKKTYEEIKKTYEEIKKTYEEIKKTYEEIERDWEMV
>ID10318-NO
IKKTYEEIKKTYEEIKKTYEEIKKTYEEIKKTYEE
>ID10319-NO
IKPEAPREDASPEELNRYYASLRHYLNLVTRQRY
>ID10320-ABP_both
IKQLLHFFQRF
>ID10323-NO
IKTFIKRWR
>ID10325-NO
IKWEYVLLLFLL
>ID10327-ABP_both
ILAWKWPWWPWRR
>ID10329-NO
ILGKFCDEIKRIV
>ID10331-ABP_both
ILGKIIKVVK
>ID10336-ABP_both
ILGLVISTIGNVLGGLLKNL

>ID10340-ABP_both
ILGPVLGLDGNALGGLIKKI
>ID10341-ABP_both
ILGPVLGLVGDTLGDLL
>ID10343-ABP_both
ILGPVLGLVSNALGGLL
>ID10345-ABP_both
ILGPVLGLVSNTLDDVLGIL
>ID10348-ABP_both
ILKWKWPWWKWRR
>ID10350-NO
ILMAFSIDSPDSLEN
>ID10351-NO
ILMCFS
>ID10352-NO
ILMCFSI
>ID10354-NO
ILMCFSIDS
>ID10355-NO
ILMCFSIDSP
>ID10357-NO
ILMCFSIDSPDS
>ID10359-NO
ILMCFSIDSPDSLE
>ID10361-NO
ILMCFSIDSPDSLENI
>ID10362-NO
ILMCFSINSPNSLQN
>ID10364-NO
ILPFKFPFPFRR
>ID10368-NO
ILPWHWPWWPWRR
>ID10370-NO
ILPWKFPWWPWRR
>ID10371-NO
ILPWKGPWWPWRR
>ID10374-ABP_both
ILPWKWAWWPWRR
>ID10375-NO
ILPWKWGWWPWRR
>ID10377-NO
ILPWKWPGWPWRR
>ID10378-NO
ILPWKWPHWPWRR
>ID10379-ABP_both
ILPWKWPKWPWRR
>ID10381-NO
ILPWKWPWGPWRR
>ID10382-NO
ILPWKWPWHPWRR
>ID10396-NO

ILPWWWPWWPWRR
>ID10397-NO
ILQRGSGTAAVDFTKKDHTATWGRPFFLFRPRN
>ID10398-ABP_both
ILRRVRVRAVAI
>ID10399-ABP_both
ILSLRWRWKWWKK
>ID10400-NO
IMDMIAGAHWGVLAGIKY
>ID10401-NO
INAKGVCRSTAKYVR
>ID10403-NO
INASVVNIQKEIDRLNEVAKNLNESLIDLQELGK
>ID10409-ABP_both
INLKILARLAKKIL
>ID10410-NO
INNYTSLIGSLIEESQNQQEKNEQELLE
>ID10411-NO
INNYTSLIHSLIEESQNQQEKNEQELLE
>ID10415-NO
INVKQIAARLLPPLYSLV
>ID10416-ABP_both
INWKKIASIGKEV
>ID10419-NO
INWLKLGKKVSAIL
>ID10422-NO
IPESSELTLQELLGEERR
>ID10423-NO
IPIYEKKYGQVPMCDAGEQCAVRKGARIGKLCDCPRGTSCNSFLLKCL
>ID10424-NO
IPLRGAFINGRWDSQCHRFSNGAIACA
>ID10425-NO
IPQSLDSWWTSL
>ID10426-NO
IPYCGQTGAECYSWCIKQDLSKDWCCDFVKDIRMNPPADKCP
>ID10427-NO
IPYIL
>ID10428-NO
IQAQPDKSESELVSQ
>ID10429-ABP_both
IQCGESCVWIPCISSAWGCSCKNKICSS
>ID10431-NO
IQKEIDRLNEVAKNLNESLI
>ID10435-NO
IRCSGSRDCYSPCMKQTGCPNAKCINKSCKCYGC
>ID10436-NO
IRDECCSNPACRVNNPHVC
>ID10438-NO
IRKAHCNISRADWND
>ID10440-NO
IRKVLFLDGIDKAQDEHEKY

>ID10441-NO
IRLPATSTRIGLTVAKKNVRRAHERNRIKRLTRELDFVVLSEAL
>ID10442-ABP_both
IRMRIRVLL
>ID10444-NO
IRRWKRVWW
>ID10445-ABP_neg
IRWRIRVWVRRIC
>ID10446-NO
ISCDLLSGLGWGHSICAGHCLAISWRYRGGYCNDQGVCVCRT
>ID10447-NO
ISDRDYMGWMDF
>ID10448-NO
ISGINASVVNIQEEIKKLNEEAKKLNESLIDLQEL
>ID10451-NO
ISGINASVVNIQKEIDRLNEVAKNLNESLIDLQELGKYEQYIKWPW
>ID10452-ABP_both
ISGPVLGLVGNALGGLIKKI
>ID10456-NO
ISLKSKIEIQRILVTFSKGFRGSVKRNRIRRLFKELEDIIFIESLM
>ID10457-NO
ISLMKRPPGFSPFR
>ID10458-NO
ISTTFTTNLTEYPLS
>ID10459-NO
ISYEL
>ID10460-NO
ISYEY
>ID10461-NO
ITAAVALVEAKQARSDIEKLKEAIRDTNKAVQSVQ
>ID10463-NO
ITCPQVTQSLAPCVPYLISG
>ID10465-NO
ITGCIC
>ID10467-NO
ITLGEWYNQTKDLQQKFYEIIMDIEQNNVQGKKGI
>ID10470-NO
IVDRCCNKGNGKRGCSRWCRDHSRCC
>ID10471-NO
IVHVTAKDKGTGKENTIRIQEGSGLSKEDIDRMIKDAEAH
>ID10472-NO
IVIYQYMDDLYVGSD
>ID10473-NO
IVKNLQNLDPSHRISDRDYMGWMDF
>ID10474-NO
IVRRADRAAVP
>ID10475-NO
IVRVGIFRL
>ID10476-NO
IVSYPDDAGEHAHKMG
>ID10477-NO

IWCEFEEATETAICQEHCLPKGYSYGICVSNTCSCI
>ID10478-NO
IWGIGCNP
>ID10480-NO
IWKYGWRYK
>ID10481-NO
IWLHSLGQHIYETYG
>ID10483-NO
IWVIWRR
>ID10484-ABP_both
IWWKWRRWV
>ID10488-NO
IYFWWWRIR
>ID10489-NO
IYRRRRKLR
>ID10492-NO
KAAKKAAKAAKKAAKAAKKAA
>ID10493-NO
KAAKKAAKAAKKAAKWAKKAA
>ID10495-NO
KAAKKAWKAAKKAWKAAKKAA
>ID10497-NO
KAAKKAWKWAKKAAKWAKKAA
>ID10499-NO
KAAKKWAKAAKKWAKAWKKAA
>ID10502-NO
KAKLAKKALAKLL
>ID10503-NO
KAKLF
>ID10506-ABP_both
KALAKALAKLWKALAKAA
>ID10507-ABP_both
KALKKLLAKWLAAAKALL
>ID10508-NO
KAPSGRMSIVKNLQNLDPSHRISDRDYMGWMDF
>ID10509-NO
KAPSGRMSVIKNLQSLDPSHRISDRDYMGWMDF
>ID10510-NO
KAPSGRMSVLKNLQGLDPSHRISDRDYMGWMDF
>ID10512-NO
KAQIRAMECNIL
>ID10513-NO
KAQIRAMECNILGRKKRRQRRR
>ID10514-NO
KAYSTPRCKYLFRAVLCWL
>ID10517-NO
KCCYSL
>ID10518-ABP_both
KCFQWQRNMRKVRGPPVSCI
>ID10520-NO
KCNRHCRSIGCRAGYCDFWTFYRRCT

>ID10522-ABP_both
KCNTATCVTQRLADFLVRSSNTIGTVYAPTNVGAAAY
>ID10523-NO
KCPTPAIEPPTGTFGFFPGV
>ID10524-ABP_both
KCRQWQSKIRRTNPIFCIRR
>ID10525-ABP_pos
KCRRRKVHGPMIRIRKK
>ID10527-ABP_both
KCRRWQWRMKKLGAPSITCV
>ID10529-ABP_both
KDEPQRRSARLSAKPAPPKPEPKPKKAPAKK
>ID10532-NO
KDSTKWRKLVDFRELNKRTQ
>ID10533-NO
KDVTVSQVWFGHRYS
>ID10535-NO
KECMTDGTVCYIHNHNDCCGSCLCSNGPIARPWEMMVGNCMCGPKA
>ID10538-NO
KEGSNICLTRTDRGWYC
>ID10539-NO
KEIDRLNEVAKNLNESLIDLQELGKYEQYIKWPWYVW
>ID10540-NO
KELKKIIGQVRDQAEHLKTA
>ID10541-NO
KENIAPYKFKATMYY
>ID10542-NO
KENKCNGTDAKVKLIKQELDKYKNAVTELQLLMQS
>ID10543-NO
KETPIRNEWGCRGETPIRNEWGCR
>ID10544-NO
KETWFETWFTEWSQPKKKRKV
>ID10546-NO
KFDSLVECIWDWIDRLWS
>ID10547-ABP_both
KFFKKLKNSVKKRAKKFFKKPRVIGVSIPF
>ID10551-ABP_neg
KFHWWKWRK
>ID10552-NO
KFKKLAKKF
>ID10553-NO
KFKKLAKKW
>ID10554-ABP_both
KFKWWRMLI
>ID10555-ABP_both
KFLHLAKKFPKWKLFKKI
>ID10556-NO
KFPKFRRGIPFLFV
>ID10557-NO
KFQGEFTNIGQSYIVSASHMSTSLNTGK
>ID10558-ABP_both

KFRRFVRFI
>ID10559-NO
KFSDQIDKGQDALKDKLGDL
>ID10561-ABP_both
KFVRLKIYCRDKNKGRGISF
>ID10563-NO
KGACDYPEWQWLCAA
>ID10564-NO
KGACELLGWEWAWLCAA
>ID10565-NO
KGATYITYVNFLNELRVKTKPEGNSHGIPSLRK
>ID10568-NO
KGIYQTSNFRVVPSGDVVRF
>ID10569-NO
KGLKKDSDFRRVGISVSKKVGKAITRNRVRRLIKEKIKDIVFIKNL
>ID10571-NO
KGLSGPAVPWWVV
>ID10572-NO
KGLSGPCGWWVWSRGSGK
>ID10573-NO
KGLSGPESRWWVV
>ID10574-NO
KGLSGPFCSWWWV
>ID10575-NO
KGLSGPFFWWLFV
>ID10576-NO
KGLSGPQGSWWVV
>ID10577-NO
KGLSGPRYVWWLV
>ID10578-NO
KGLSGPSEWWVWV
>ID10579-NO
KGLSGPSIRWWLV
>ID10580-NO
KGLSGPSWWLFCV
>ID10581-NO
KGLSGPTAWWVVV
>ID10582-NO
KGLSGPTVQWWVV
>ID10583-NO
KGLSGPWCRWWLV
>ID10584-NO
KGSPAIFQSSMTKIL
>ID10586-NO
KGVSGHGQHGVHG
>ID10587-NO
KGWIIWKIV
>ID10588-NO
KHKRRR
>ID10591-NO
KIAKVALAKLGIGAVLKVLTTGL

>ID10594-ABP_both
KIKWILKYWKWS
>ID10595-NO
KILEPFRKQNPDIVI
>ID10599-NO
KIPLELWTRYNMTINQTIWN
>ID10600-NO
KIPYIL
>ID10601-NO
KISKIGPENPYNTPVFAIKK
>ID10602-ABP_both
KITLKLAIKAWKLALKAA
>ID10603-NO
KIWVRWK
>ID10604-ABP_both
KKAAAAAAAAAAAAWAAAAAAKKKK
>ID10605-ABP_both
KKAAAIAAAAAIAAWAAIAAAKKKK
>ID10607-ABP_both
KKAAAMAAAAAMAAWAAMAAAKKKK
>ID10608-ABP_both
KKAAASAAAAASAAWAASAAAKKKK
>ID10609-ABP_both
KKAAAVAAAAAVAAWAAVAAAKKKK
>ID10610-ABP_both
KKAAAWAAAAAWAAWAAWAAAKKKK
>ID10611-ABP_both
KKAAAYAAAAAYAAWAAYAAAKKKK
>ID10612-NO
KKALLKHALHKLALLAKHLAHKLKKA
>ID10613-NO
KKCCYHCQ
>ID10617-NO
KKFACPECPKRFMRSDHLSKHIKTHQNKK
>ID10619-NO
KKGACEARHREWAWLCAA
>ID10621-NO
KKIKPPLPSVTKLTE
>ID10623-NO
KKKFPWWWPFKKKCKKKFPWWWPFKKKC
>ID10625-NO
KKKKAVAATYVLV
>ID10626-NO
KKKKEVAATYVLV
>ID10627-NO
KKKKFVAATYVLV
>ID10628-NO
KKKKFVKKVAKKVKKVAKKVAKVAVAV
>ID10634-ABP_both
KKKKKKK
>ID10635-ABP_both

KKKKKKKKK
>ID10636-ABP_both
KKKKKKKKKK
>ID10637-NO
KKKKKKKKKKAAFAAWAAFAA
>ID10638-NO
KKKKKVAATYVLV
>ID10639-NO
KKKKLLAAFLFFF
>ID10641-NO
KKKKLLAPFLFFF
>ID10642-NO
KKKKLLAPFLFFV
>ID10644-NO
KKKKLLLAFLFFF
>ID10645-NO
KKKKLLLAFLFFV
>ID10648-NO
KKKKLLLATLFFF
>ID10649-NO
KKKKLLLPFLFFF
>ID10650-NO
KKKKLLLPFLFFV
>ID10651-NO
KKKKLLLPFLFLF
>ID10652-NO
KKKKLLLPFLFLV
>ID10655-NO
KKKKLLLPFLVLF
>ID10658-NO
KKKKLLLPFYFLF
>ID10659-NO
KKKKLLLPFYVFF
>ID10660-NO
KKKKLLLPTLFFF
>ID10661-NO
KKKKLLLPTLFFV
>ID10662-NO
KKKKLLLPTLFLF
>ID10665-NO
KKKKLVAAFYVLV
>ID10666-NO
KKKKLVAATYVFV
>ID10667-NO
KKKKLVAATYVLF
>ID10669-NO
KKKKLVAPFLFFF
>ID10670-NO
KKKKLVAPTYVLV
>ID10671-NO
KKKKLVLAFLFFF

>ID10672-NO
KKKKLVLATYVLV
>ID10673-NO
KKKKLVLPFLFFF
>ID10674-NO
KKKKLVLPFLFFV
>ID10675-NO
KKKKLVLPFLFVF
>ID10678-NO
KKKKLVLPTLFFF
>ID10679-NO
KKKKPVAATYVLV
>ID10680-NO
KKKKTVAATYVLV
>ID10681-NO
KKKKVAAATYVLV
>ID10682-NO
KKKKVEAATYVLV
>ID10683-NO
KKKKVFAATYVLV
>ID10684-NO
KKKKVGAATYVLV
>ID10685-NO
KKKKVKAATYVLV
>ID10686-NO
KKKKVLAAFYVLV
>ID10688-NO
KKKKVLAATYFLV
>ID10690-NO
KKKKVLAATYVLV
>ID10691-NO
KKKKVLAPFLFFF
>ID10692-NO
KKKKVLAPTYVLV
>ID10693-NO
KKKKVLLAFLFFF
>ID10694-NO
KKKKVLLATYVLV
>ID10695-NO
KKKKVLLPFLFFF
>ID10696-NO
KKKKVLLPFLFFV
>ID10697-NO
KKKKVLLPFLFVF
>ID10700-NO
KKKKVPAATYVLV
>ID10701-NO
KKKKVTAATYVLV
>ID10702-NO
KKKKVVAAAYVLV
>ID10703-NO

KKKKVVAAEYVLV
>ID10704-NO
KKKKVVAAFLVLV
>ID10705-NO
KKKKVVAAFYFLV
>ID10706-NO
KKKKVVAAFYVFV
>ID10707-NO
KKKKVVAAFYVLF
>ID10709-NO
KKKKVVAAGYVLV
>ID10710-NO
KKKKVVAAKYVLV
>ID10711-NO
KKKKVVAALYVLV
>ID10712-NO
KKKKVVAAPYVLV
>ID10713-NO
KKKKVVAATAVLV
>ID10714-NO
KKKKVVAATEVLV
>ID10715-NO
KKKKVVAATFVLV
>ID10716-NO
KKKKVVAATGVLV
>ID10717-NO
KKKKVVAATKVLV
>ID10718-NO
KKKKVVAATLFLV
>ID10719-NO
KKKKVVAATLVFV
>ID10720-NO
KKKKVVAATLVLF
>ID10721-NO
KKKKVVAATPVLV
>ID10722-NO
KKKKVVAATTVLV
>ID10723-NO
KKKKVVAATYALV
>ID10724-NO
KKKKVVAATYELV
>ID10726-NO
KKKKVVAATYFFV
>ID10727-NO
KKKKVVAATYFLV
>ID10730-NO
KKKKVVAATYKKKA
>ID10731-NO
KKKKVVAATYKKKK
>ID10732-NO
KKKKVVAATYKKVA

>ID10737-NO
KKKKVVAATYVAV
>ID10738-NO
KKKKVVAATYVEV
>ID10739-NO
KKKKVVAATYVFF
>ID10740-NO
KKKKVVAATYVFV
>ID10745-NO
KKKKVVAATYVLA
>ID10761-NO
KKKKVVAETYVLV
>ID10762-NO
KKKKVVAFTYVLV
>ID10763-NO
KKKKVVAGTYVLV
>ID10764-NO
KKKKVVAKTYVLV
>ID10766-NO
KKKKVVAPFYVLV
>ID10767-NO
KKKKVVAPTLVLV
>ID10769-NO
KKKKVVAPTYVFV
>ID10770-NO
KKKKVVAPTYVLF
>ID10772-NO
KKKKVVATTYVLV
>ID10773-NO
KKKKVVEATYVLV
>ID10774-NO
KKKKVVFATYVLV
>ID10775-NO
KKKKVVGATYVLV
>ID10776-NO
KKKKVVKATYVLV
>ID10778-NO
KKKKVVLATLVLV
>ID10779-NO
KKKKVVLATYVLF
>ID10781-NO
KKKKVVLPFLFFF
>ID10782-NO
KKKKVVLPTYVLV
>ID10783-NO
KKKKVVTATYVLV
>ID10784-NO
KKKKVVTSTYVLVEA
>ID10785-NO
KKKKYRNIRRPG
>ID10786-NO

KKKLFINTW
>ID10787-NO
KKKLWRKFR
>ID10788-NO
KKKMVLGVFALLSLISGSLKK
>ID10789-ABP_neg
KKLAKALKLLALLWLKLAKALKKA
>ID10790-ABP_neg
KKLAKALKLLAPLWLKLAKALKKA
>ID10794-ABP_neg
KKLAKLALLKWLLALKLLALKALKK
>ID10795-ABP_neg
KKLALALKKLALLWKKLALALKKA
>ID10796-ABP_neg
KKLALALKKLAPLWKKLALALKKA
>ID10801-ABP_both
KKLALKALLLWLKALLKLAKLALKK
>ID10802-NO
KKLFKKLLKYL
>ID10804-ABP_both
KKLLFKLKFK
>ID10806-NO
KKNEEIIKKLEETKKNEEKNKKSEEILKKLEELRKK
>ID10808-NO
KKRKRRFLGFLLGVGSA
>ID10809-NO
KKRRFKRRY
>ID10810-NO
KKRRGNRVR
>ID10811-ABP_both
KKRWVWIRY
>ID10812-NO
KKVRFTITW
>ID10813-NO
KKVRRVIWW
>ID10814-ABP_both
KKVVPKVKFK
>ID10815-ABP_both
KKVVVKVKFK
>ID10816-NO
KKWGGGLVK
>ID10817-ABP_neg
KKWKIVVIKWKK
>ID10819-NO
KKWKVWRFG
>ID10829-ABP_both
KLALKAAAKAWKAAAKAA
>ID10832-NO
KLALKLALKALKAAKLA
>ID10834-NO
KLFKFLRKHLL

>ID10836-ABP_both
KLIPIASKTCPAGKNLCYKI
>ID10837-NO
KLIRIWWWW
>ID10842-NO
KLKLK
>ID10843-NO
KLKLKLK
>ID10844-NO
KLKLKLKLKLKLK
>ID10845-NO
KLKLKLKLKLKLKLK
>ID10846-ABP_both
KLLAKAALKWLLKALKAA
>ID10848-ABP_both
KLLKFIKKLL
>ID10850-NO
KLLLK
>ID10851-NO
KLLLKLLLK
>ID10856-ABP_both
KLRKLFRKLLKLIRKLLR
>ID10861-NO
KLWYWKKVV
>ID10863-NO
KMRLKWKRR
>ID10864-NO
KMVQGSGCFGRKMDRISSSSGLGCKVL
>ID10865-NO
KMVQGSGCFGRKMDRISSSSGLGCKVLR
>ID10866-NO
KMVQGSGCFGRKMDRISSSSGLGCKVLRRH
>ID10868-NO
KNGRKLCLDLQAALY
>ID10870-NO
KNKKPKNPTPPRPAG
>ID10873-NO
KNPTPPRPAGDNATV
>ID10875-NO
KPKCGLCRYRCCSGGCSSGKCVNGACDCS
>ID10877-NO
KPLWRL
>ID10878-NO
KPNNDFHFEVFNFVPCSICSNNPTCWAICKRI
>ID10885-NO
KPPSKPNNDFHFEVFN
>ID10891-NO
KPPSKPNNDFHFEVFNFVP
>ID10893-NO
KPPSKPNNDFHFEVFNFVPCSICSNNPTCWAICKRI
>ID10898-ABP_both

KPRRPVRPI
>ID10900-NO
KPTFRRLKWKYK
>ID10901-ABP_both
KPVPIIYCNRRTGKCQRM
>ID10903-NO
KQLLPIVNKQSCSISNI
>ID10904-NO
KQLSSNFGAISSVLNDILSRLDKVEAEVQIDRLITG
>ID10905-NO
KQLSYCTDPLQIPLINYTFG
>ID10906-NO
KQNAANILRLKESIAATNEAVHEV
>ID10907-NO
KQRNRWEWRPDFKSKKVKISLPC
>ID10908-NO
KQRQNKPPSKPNNDFHF
>ID10910-NO
KQRQNKPPSKPNNDFHFEVFN
>ID10911-NO
KQRQNKPPSKPNNDFHFEVFNFVPCSICSNNPTCWAICKRI
>ID10912-NO
KQRQNKPPSKPNNDFHFEVFNFVPCSICSNNPTCWAICKRIPNKKPGKK
>ID10915-NO
KQRQQLFDSQQGWFEGWFNRSPWFT
>ID10918-ABP_both
KRFKKFFRKIKKGFRKIFKKTKIFIGGTIPI
>ID10919-NO
KRFKQDGGWSHWSPWSS
>ID10920-ABP_both
KRIMKLKMR
>ID10921-NO
KRIRRWKWW
>ID10923-NO
KRIRYKRWH
>ID10926-ABP_both
KRKKRFKWW
>ID10928-NO
KRKRAVKRVGRRLKKKLARKIARLGVAF
>ID10929-NO
KRKRAVKRVGRRLKKLARKIARLGVAKLAGLRAVKLF
>ID10930-NO
KRKRFAKKFLRFLRKVIRFLKRFIRRF
>ID10931-NO
KRKRQQGIGAVLKVLTTGLPALISWI
>ID10933-NO
KRLLTARQFSRLGLVIGKKNVKLAVQRNRLKRLIRELDIVVLHQQF
>ID10934-NO
KRLLTPRHFKRLGLVIGKKSVKLAVQRNRLKRLMRDLDIVILHQHF
>ID10935-ABP_both
KRNGFRKFMRRLKKFFAGGGSSIAHIKLH

>ID10936-NO
KRPPGFSPFR
>ID10937-NO
KRQWWRVFK
>ID10942-NO
KRRKKRRVR
>ID10944-ABP_both
KRRKLAFRLFRLFLKLVLKK
>ID10945-ABP_both
KRRKLIKILKLIAKLIRKKR
>ID10947-NO
KRRKQRKYR
>ID10951-ABP_both
KRRLFLFRLFRLFLRLFLKK
>ID10952-ABP_both
KRRLIARILRLAARALVKKR
>ID10955-ABP_both
KRRLILRILRLAIRALVKKR
>ID10957-NO
KRRVWRMWR
>ID10958-ABP_both
KRRWRIWLV
>ID10959-NO
KRSKLLLGIGILVLLIIVILGVPLIIFTIKKKKKK
>ID10960-NO
KRVKVRWVT
>ID10961-NO
KRVKWTWRK
>ID10962-NO
KRWKHIRRI
>ID10963-NO
KRWLKWWRV
>ID10964-NO
KRWNETITGPSGCANNTCYN
>ID10966-NO
KRWTFWSRR
>ID10967-NO
KRWWQWRWF
>ID10968-NO
KSKKVKISLPCNSTKNLTFA
>ID10970-NO
KSLLTEVETPIRGSLLTEVETPIR
>ID10971-NO
KSNVVRGWVFGSTMNNKSQS
>ID10974-NO
KSWRKLFIW
>ID10977-ABP_neg
KTKKKLLKKT
>ID10978-NO
KTKLFKKFAKKLAKKLKKLAKKL
>ID10979-ABP_both

KTLTGKTITLE
>ID10980-NO
KTNVTLSKKRKRRFLGF
>ID10981-NO
KTTSSIEFARLQFTY
>ID10982-NO
KVDISSQISSMNQSLQQSKDYIKEAQRLLDTVNPSL
>ID10985-NO
KVGSLQYLALAALIT
>ID10986-NO
KVINPEPIVEPFMSKPFALF
>ID10987-NO
KVPAQNAISTTFPYT
>ID10989-NO
KVRWKWWGW
>ID10990-NO
KVRWWYNIK
>ID10991-NO
KVVRKKRLR
>ID10992-NO
KVYLAWVPAHKGIGG
>ID10997-ABP_both
KWCRCVCRRGICRCRCRG
>ID10998-ABP_both
KWCRCVCRRGICRCRCRK
>ID11000-NO
KWEYVLLLFLL
>ID11001-NO
KWFWKFVKFVK
>ID11002-ABP_both
KWIKWIKWI
>ID11003-ABP_both
KWIRWVRWI
>ID11004-ABP_both
KWKARKNFIKGSSLGWLIQLFRKR
>ID11005-ABP_both
KWKFFKKIERVGQNIRDGIIKAGPAVAVVGQATNIAKG
>ID11006-NO
KWKIFKKIEHMGQNIRDGLIKAGPAVQVVGQAATIYK
>ID11007-ABP_both
KWKIFRRWW
>ID11008-ABP_both
KWKIRVRLSA
>ID11009-NO
KWKKFKKGIGKLFV
>ID11011-NO
KWKKLAKKW
>ID11013-NO
KWKLAKKALALL
>ID11014-NO
KWKLF

>ID11016-NO
KWKLFKKALKKLKKALKKAL
>ID11017-ABP_both
KWKLFKKI
>ID11026-NO
KWKLFKKIGIGKVVKKVLKKVLK
>ID11029-NO
KWKLFKKKTKLFKKFAKKLAKKL
>ID11030-ABP_neg
KWKSFIKKKTSKFLHSAKKF
>ID11034-NO
KWKWLQGRR
>ID11035-ABP_both
KWKWWWRKI
>ID11036-NO
KWLCRIWSWISDVLDDFE
>ID11038-NO
KWLTEWIPLTAEC
>ID11039-NO
KWPKFKKGIPWLFV
>ID11040-ABP_neg
KWPWWKWRR
>ID11043-NO
KWRRWYYWR
>ID11044-NO
KWVWFRWRK
>ID11045-NO
KWWIWKRRR
>ID11047-ABP_neg
KWWKWKK
>ID11059-NO
KYKLKHIVWASREL
>ID11060-NO
KYLRSVIFY
>ID11061-NO
KYTAFTIPSINNETPGIRYQ
>ID11065-ABP_both
KYYSRVRGGRSAVLSSLPK
>ID11066-NO
LAAIPMSIPPEVKFNKPFVF
>ID11067-ABP_both
LAAKLTKAATKLTAALTKLAAALT
>ID11068-NO
LAEGPPVKECAVTCRYDKDADINVVTQARN
>ID11069-NO
LAHKSRLYERHM
>ID11070-NO
LALALKHALHKALLLAKHLAHKLALA
>ID11074-NO
LCYCFRRRCVCV
>ID11075-NO

LCYCHHHFCVCV
>ID11076-NO
LCYCRFRRCVCV
>ID11077-NO
LCYCRRFRCVCV
>ID11078-NO
LCYCRRRFCFCV
>ID11079-NO
LCYCRRRFCGCV
>ID11080-NO
LCYCRRRFCTCV
>ID11083-NO
LCYCRRRRCVCV
>ID11084-NO
LCYTFRGRFVCV
>ID11085-NO
LCYTFRPRFVCV
>ID11087-NO
LCYTRGRFTVCVR
>ID11088-NO
LDCWVRLGRYLLRRLKTP
>ID11089-NO
LDCWVRLGRYLLRRLKTPFTRL
>ID11090-NO
LDFAKHVIGIASKL
>ID11094-NO
LDPAFR
>ID11096-NO
LDPSHRISDRDYMGWMDF
>ID11099-NO
LEAAPMSIPPEVKFNKPFVF
>ID11100-NO
LEAIAMSIPPEVKFNKPFVF
>ID11101-NO
LEAIPASIPPEVKFNKPFVF
>ID11102-NO
LEAIPCSIPPCFAFNKPFVF
>ID11103-NO
LEAIPCSIPPCFLFGKPFVF
>ID11105-NO
LEAIPCSIPPCVFFGKPFVF
>ID11106-NO
LEAIPCSIPPCVFFNKPFVF
>ID11108-NO
LEAIPCSIPPCVLFNKPFVF
>ID11109-NO
LEAIPMCIPPECAFNKPFVF
>ID11110-NO
LEAIPMSAPPEVKFNKPFVF
>ID11111-NO
LEAIPMSIAPEVKFNKPFVF

>ID11112-NO
LEAIPMSIPAEVKFNKPFVF
>ID11116-NO
LEAIPMSIPPEVAFAKPFVF
>ID11117-NO
LEAIPMSIPPEVAFNKPFVF
>ID11127-NO
LEEWYKKTEELQKKFEEIIKKIEENNKKVEEGLKK
>ID11128-NO
LEGQEEEEDNRDSSMKLSF
>ID11130-NO
LESEVTAIKNALKKTNEAVSTLGNGVRVLATAVRE
>ID11131-NO
LEVYTRHEIKDSGLL
>ID11133-NO
LFFLLTVPFWAHYA
>ID11137-ABP_both
LGALFRVASKVFPAVISMVK
>ID11140-NO
LGCNQNQFFCKIPLELWTRY
>ID11141-NO
LGEWYNQTKDLQQKFYEIIMDIEQNNVQGKKGIQQ
>ID11142-NO
LGEWYNQTKELQQKFYEIIMNIEQNNVQVKKGLQQ
>ID11146-NO
LGNWAREIWATL
>ID11148-NO
LGQGVSIEWRKKRYS
>ID11149-NO
LGQSAASAHHAYIVLAIENSFMTASKKK
>ID11150-NO
LGRMKG
>ID11151-NO
LGRRWRYRR
>ID11152-NO
LGRTLAAIKLTQHYFGLLTAFGSNFGTIG
>ID11153-NO
LGRVAIAWCELQNHE
>ID11154-NO
LGSSDRDTVVELSEWGVPCV
>ID11155-NO
LGTEVSEALGGAGLTGGF
>ID11156-NO
LGTYTQDFNKFHTFPQTAIGVGAP
>ID11158-NO
LHDLRFADIDTVIHA
>ID11159-NO
LHPHE
>ID11162-NO
LIAHNQVRQV
>ID11165-NO

LIIFVPPFF
>ID11166-NO
LIILVPAFF
>ID11167-NO
LIILVPPAY
>ID11169-NO
LIILVPPFY
>ID11170-NO
LIILVPPWF
>ID11172-NO
LITPKKIKPPLPSVT
>ID11175-NO
LKARTVTFGVVTSVI
>ID11176-NO
LKCNKLVPLFYKTCPAGKNL
>ID11178-ABP_neg
LKKISQ
>ID11179-ABP_both
LKKISQRYQKFALPQYLKTVYQHQKAMKPWIQPKTKVIPYVRYL
>ID11180-NO
LKKLAKLALAF
>ID11185-NO
LKLKLKLK
>ID11186-NO
LKLKNIVSWAKKVL
>ID11187-NO
LKLKSIVSFAKKVL
>ID11190-NO
LKLKSIVSWALLVL
>ID11193-NO
LKLLLLKLLKLKLWK
>ID11200-ABP_both
LKRRWKWWI
>ID11201-NO
LKTVRLIKFLY
>ID11204-NO
LLDCWVRLGRYLLRRLK
>ID11207-NO
LLDCWVRLGRYLLRRLKTPFT
>ID11209-NO
LLETSEG
>ID11210-NO
LLEYVF
>ID11212-NO
LLEYVW
>ID11213-NO
LLEYVY
>ID11214-NO
LLFLKVPAQNAISTTFPYT
>ID11220-ABP_neg
LLGDFFRKSKEKIGKEFKR

>ID11225-NO
LLGFILAFLGWIGAIVST
>ID11226-NO
LLGGILGIGLGVLLLILCLP
>ID11227-NO
LLGRM
>ID11228-NO
LLGRMKG
>ID11229-NO
LLGTEVSEALGGAGLTGG
>ID11230-ABP_both
LLKALKKLLKKLL
>ID11235-NO
LLLKLLKLLLKLLLKLLK
>ID11236-ABP_both
LLPPWLRPRNG
>ID11237-NO
LLPRRGPRLGVRATRKTS
>ID11238-NO
LLQLTVWGIKQLQARIL
>ID11240-NO
LLRIPQAIMDMIAGAHWG
>ID11241-NO
LLRVKR
>ID11242-NO
LLSLINDMPITNDQKKL
>ID11244-NO
LMAAKAESRK
>ID11245-NO
LNFKALAALAKKIL
>ID11247-NO
LNKAKSDLEESKEWIRRSNQKLDSIGNWHQSSTT
>ID11251-NO
LNNCLLLGTEVSEALGGA
>ID11252-NO
LNNCLLLGTEVSEALGGAGLTGG
>ID11255-NO
LNWGAACALKHACAACAK
>ID11257-NO
LPEKDSWTVNDIQKLVGKLN
>ID11263-ABP_both
LPLNTIPRPPYFPGKLPPRGGHLFPPTCVCVRSPCPCDQNWG
>ID11264-ABP_both
LPLSINPWRPPFPGRPLPGGPLVLPGCVCVRAPCYCSPSRQKDFPGFEHY
>ID11267-NO
LPPNPTN
>ID11270-NO
LPRLLHTDSRMATIDFPKKDPTTSLGRPFFLFRPRN
>ID11272-NO
LPRPGP
>ID11273-NO

LPRRLHLEPAFLPYSVKAHE
>ID11274-NO
LPRRLHLEPAFLPYSVKAHEC
>ID11277-NO
LPTTQLRRHIDLLVGSAT
>ID11278-NO
LQFTYNHIQRHVNDM
>ID11279-NO
LQITLW
>ID11282-NO
LQLEARLQHLVAEILER
>ID11283-ABP_both
LQLLKQLLKLLKQF
>ID11284-NO
LQNHELTLWNEARKL
>ID11286-NO
LQYLALAALITPKKI
>ID11289-NO
LRCRNEKKRHRAVRLIFTI
>ID11290-NO
LRDIKAENTDANFYV
>ID11297-ABP_both
LRFLKKILKKLF
>ID11298-ABP_both
LRFLRRILRRLL
>ID11300-ABP_both
LRIIKKILKKLI
>ID11301-ABP_both
LRIIRRILRRLI
>ID11302-ABP_both
LRILRRLLRRLF
>ID11303-ABP_both
LRKAKKIAKKLF
>ID11304-ABP_both
LRKLKKILKKLF
>ID11306-NO
LRLKHWQDFQRFGITVSQKVSKKATVRNRLKRQIRAIKDVVIFLREL
>ID11308-NO
LRLKKRRWKYRVP
>ID11309-ABP_both
LRLKKYKVPQL
>ID11312-NO
LRLLTPAQFKRLGLTVAKRYVKRANQRNRIKRVIRDIDIVVLNKLI
>ID11314-NO
LRLLTPIQFKRLGLTVAKKHLKRAHERNRIKRLVRELDFVFFAQIL
>ID11315-NO
LRLLTPKHFNRIGLTIAKKNVKRAHERNRIKRLARELDFVVLTEVL
>ID11316-NO
LRLLTPSHFTRIGLTVAKKHVKRAHERNRIKRLTRELDFVVLTEAL
>ID11317-NO
LRLLTPSHFTRIGLTVAKKNVKRAHERNRIKRLTRELDFVVLSEAL

>ID11320-ABP_both
LRPAILVRTK
>ID11321-ABP_both
LRPAILVRVKGKGL
>ID11325-ABP_both
LRRWIRIRW
>ID11326-NO
LRSEYGGSFRFSSDA
>ID11327-NO
LRSFGCRFGTCTVQKL
>ID11328-NO
LRSRTKIIRIRH
>ID11330-ABP_both
LRWTPTPSYPRYPTRSRGSRWSR
>ID11331-ABP_both
LRWWWIKRI
>ID11332-NO
LSCPQYVYGSVSVTCVWGSV
>ID11333-NO
LSEMERRRLRKRA
>ID11334-NO
LSGAQYQQHGRAL
>ID11335-ABP_both
LSIRRLLAAGVLKQTKGVGA
>ID11336-NO
LSLATFAKIFMTRSNWSLKRFNRL
>ID11337-NO
LSNIKENKCNGTDAKVKLIKQELDKYKNAVTELQL
>ID11338-NO
LSNIKKIKCNGTDAKVK
>ID11339-NO
LSNTLAELYVREHLR
>ID11343-NO
LSWKWWRRV
>ID11344-NO
LTAEHYAAQATS
>ID11345-NO
LTCDVIGSTQLCAAHCIAKGYRGGWCDGKSVCNCRR
>ID11346-NO
LTEDRWNKPQKTKGH
>ID11347-NO
LTEEKIKALVEICTEMEKEG
>ID11348-NO
LTFEHYWAQLTS
>ID11349-NO
LTLWNEARKLNPNAI
>ID11350-NO
LTSDTYFLPVPA
>ID11351-NO
LTTTSTALGKLQDVVNQNAQALNTLVKQLSSNFG
>ID11355-NO

LVKCPTPAIEPPTGTFGF
>ID11356-NO
LVKCRGTSDCGRPCQQQTGCPNSKCINRMCKCYGC
>ID11357-NO
LVKPRTSRTGPGAWQGGRRKFRRQRPRLSHKGPMPF
>ID11360-NO
LVLRICTDLFTFIKWTIKQRKS
>ID11361-ABP_both
LVPFIGRTLGGLLARF
>ID11362-NO
LVQPRGSRNGPGPWQGGRRKFRRQRPRLSHKGPMPF
>ID11363-NO
LVRDSMAKLRERLNQRQKLFESTQ
>ID11364-NO
LVRWVWGKR
>ID11365-NO
LVSFRYEDQGPLVEG
>ID11366-NO
LVTLEHQVLVIGLKVEAMEK
>ID11367-NO
LVTLVFV
>ID11370-ABP_both
LVVAVTDGEADAAVEGLHDNTDFIHYGSHGKYPDNRPHGYPLD
>ID11373-NO
LWVGGGRNA
>ID11375-NO
LYGNEGCGWAGWLLSPRG
>ID11377-NO
LYYFDCFSESAIRKA
>ID11401-NO
MAGRSGDSDEELLKTVRLIKFLYQSNPPPS
>ID11402-NO
MAILGDTAWDFGSLGGVFTSIGKALHQVFGAIY
>ID11419-NO
MANAGLQLLGFILA
>ID11420-NO
MANAGLQLLGFILAFL
>ID11422-NO
MANAGLQLLGFILAFLGWIG
>ID11423-NO
MANAGLQLLGFILAFLGWIGAI
>ID11425-NO
MANLGYWLLALFVTMWTDVGLCKKRPKP
>ID11427-NO
MAQDIISTIGD
>ID11428-NO
MAQDIISTIGDLVKWIIDTVNKFPKK
>ID11429-NO
MARHRNWPLVMV
>ID11431-NO
MASAGMQILGVVLTLLGW

>ID11436-NO
MASTGLELLGMTLAVLGW
>ID11441-NO
MAVGLVLCDWWLGEYLLEA
>ID11442-NO
MCMPCFTTDPNMAKKCRDCCGGNGKCFGPQCLCNR
>ID11446-NO
MDGPKVKQWPLTEEKIKALV
>ID11448-NO
MDRIFARRYNATHIK
>ID11451-NO
MDVNPCLLFLKVPAQ
>ID11452-NO
MDVNPFLLFLKVPAQ
>ID11453-NO
MDVNPHLLFLKVPAQ
>ID11454-NO
MDVNPT
>ID11455-NO
MDVNPTFLFLKVPAQ
>ID11457-NO
MDVNPTLL
>ID11459-NO
MDVNPTLLFIDVPAQ
>ID11460-NO
MDVNPTLLFL
>ID11461-NO
MDVNPTLLFLK
>ID11464-NO
MDVNPTLLFLKVPA
>ID11468-NO
MDVNPTLLFLKVPAQNAIST
>ID11469-NO
MDVNPTLLFLKVPAQNAISTTFPYT
>ID11471-NO
MDVNPYFLFLKVPAQ
>ID11474-NO
MDYQVSSPIYDINYYTSEPCQKINVK
>ID11489-NO
MEQAPEDQGPQREPY
>ID11712-NO
MGFTRKWQF
>ID11715-NO
MGIIAGIIKFIKGLIEKFTGK
>ID11725-NO
MGYIPLVGAPLGGAARAL
>ID11726-NO
MHKRRR
>ID11731-NO
MIRIAMKALNCFKVSGLKCWSFNSPRGQESPCPG
>ID11732-NO

MIRIRSPTKKKLNRNSISDWKSNTSGRFFY
>ID11773-NO
MKLLLLTLAALLLVSQLTPGDAQKCWNLHGKCRHRCSRKESVY
>ID11801-NO
MKRRRCNWCGKLFYLEEKSKEAYCCKECRKKAKKVKK
>ID11819-NO
MKVWIRWRI
>ID11824-NO
MLCVLQGLRE
>ID11826-NO
MLCVLQGLREGG
>ID11828-ABP_both
MLLKKLLKKM
>ID11829-NO
MLMACYSAGQLGCLVFCNEAEYSYGKCIGRGRCCCYDL
>ID11831-NO
MLQDWLSSLGDLLKSLLDTVNKFTKK
>ID11835-NO
MLRKRRKRL
>ID11844-NO
MMNWSPTAALVVAQLLRI
>ID11856-NO
MNINPTLLFLKVPAQ
>ID11858-NO
MNINPYPLFIDVPIQ
>ID11872-NO
MPEVEGEEIQPQMELRRNGR
>ID11873-NO
MPITNDQKKLMSNNVQI
>ID11876-NO
MPRRRRIRRRQK
>ID11877-NO
MPRVRSLFQEQEEPEPGMEEAGEMEQKQLQ
>ID11878-NO
MPRVRSLFQRQKRTEPGLEEVGEIEQKQLQ
>ID11879-NO
MPRVRSLVQVRDAEPGADAEPGADAEPGADDAEEVEQKQLQ
>ID11880-NO
MPRVRSVVQARDAEPEADAEPVADEADEVEQKQLQ
>ID11881-NO
MPRYRLFRRIDRVGK
>ID11882-NO
MPRYRLFRRIDRVGKQIKQGILRAGPAIALVGDARAVG
>ID11900-NO
MRFGSLALVAYDSAIKHSWPRPSSVRRLRM
>ID11901-NO
MRGIRGADFQAFQQARAVGLAGTFR
>ID11917-NO
MRKRRR
>ID11931-NO
MRSSGDYGEVTGAWIEFGCH

>ID11949-NO
MSEALWTAWTQW
>ID11955-NO
MSTNPKPQRKTKRNTNRR
>ID11957-NO
MSTNPKPQRKTKRNTNRRPQDVKFPGGGQIVGGV
>ID11958-NO
MSTNPKPQRKTKRNTNRRPQDVKFPGGGQIVGGVY
>ID11962-NO
MTGRLISWWWSL
>ID11968-NO
MTKWQEVDEMLRSEY
>ID11969-NO
MTLTG
>ID11970-NO
MTLTVQARQLLSQIVQQQNNLLRAIEAQ
>ID11978-NO
MVQGSGCFGRKMDRISSSSGLGCK
>ID11980-NO
MVQGSGCFGRKMDRISSSSGLGCKVLR
>ID11981-NO
MVQGSGCFGRKMDRISSSSGLGCKVLRR
>ID11986-ABP_both
MWGRILGTVAKYGPKAVSWAWQHKWELINMGDLAFRYIQRIWG
>ID11990-NO
MWWWFLWRR
>ID11994-NO
MYNCSLQNGFTMKVDDLIVH
>ID11995-NO
NADIIKSLIRKTIINASKNTASLSILQHLYVLRS
>ID11997-NO
NAPVSIPQ
>ID11998-NO
NASDMEIKKVNKKIEEYIKKIEEVEKKLEEVNKK
>ID11999-NO
NCCAPEDIGFCLEGGCLV
>ID12002-NO
NCLLLGTEVSEALGGAGLTGG
>ID12003-NO
NCVKMLCTHTGTGQAITVTP
>ID12004-ABP_both
NDAACAAHCLFRGRSGG
>ID12006-NO
NDPCEEVCLQHTGNVKACEEACQ
>ID12008-NO
NEEGFFSARGHRPLDGGGKKKKKK
>ID12009-NO
NEELGKKYEETKKKQEEFYKKIEEIEKKNEEVKKKLEELQKK
>ID12010-NO
NELRLTRDAIEPCTV
>ID12013-NO

NFYDPLVFPSDEFDASISQVNEKINQSLASIRKSDELLHNVNAGK
>ID12014-ABP_both
NFYEIFRNRNRGGLIKDAATPWLPCEILRPC
>ID12016-NO
NGIGVTQNVLYENQKQIANQFNKAISQIQESLTTTSTA
>ID12017-ABP_both
NGKRVCVCR
>ID12019-NO
NGPGPWQGGRRKFRRQRPRLSHKGPMPF
>ID12021-ABP_both
NGVQPKYKWWKWWKKWW
>ID12022-ABP_both
NGVQPKYRWWRWWRRWW
>ID12023-NO
NHFTLKCPKTALTEPPTLAY
>ID12024-NO
NHIQRHVNDMLGRVA
>ID12025-NO
NHTTWMEWDREINNYTSLIHSLIEESQNQQEKNEQELLELDKW
>ID12026-NO
NIAGNWSCTSDLPSSWGYMN
>ID12030-NO
NILRLKESITATIEAVHEVTDGLS
>ID12032-NO
NITLGEWYNQTKDLQQKFYEIIMDIEQNNVQGK
>ID12036-NO
NKKIRVRL
>ID12038-NO
NKRVWFIYR
>ID12039-NO
NKSLLTEVETPIRNEWGCRCNDSSD
>ID12041-NO
NLGKQWAVGHFM
>ID12043-ABP_both
NLLNDALGTVNGLLGRS
>ID12045-NO
NLQGLDPSHRISDRDYMGWMDF
>ID12046-ABP_both
NLVSDIIGSKKHMEKLISIIKKCR
>ID12048-NO
NMAKLRERLKQRQQL
>ID12049-NO
NMTINQTIWNHGNITLGEWY
>ID12050-NO
NNAKKTNVTLSKKRKRR
>ID12051-NO
NNCLLLGTEVSEALGGAG
>ID12052-NO
NNCLLLGTEVSEALGGAGL
>ID12055-NO
NNETPGIRYQYNVLPQGWKG

>ID12056-NO
NNLETTAFHRDDHET
>ID12058-NO
NNTRKSIRIQRGPGRAFVTIGKIG
>ID12059-NO
NNWWYWWDTLVN
>ID12062-NO
NPAFLFQPQRF
>ID12063-NO
NPGLLETSEGCRQ
>ID12064-NO
NPGLLETSEGCRQILGQLQPSLQT
>ID12065-NO
NPMYNAVSNADLMDFKNLLDHLEEKMPLED
>ID12066-NO
NPNAIASVTVGRRVS
>ID12067-NO
NPPDHSAPLGATRPSAPPLPHVVDLPQLGP
>ID12068-NO
NPQSCRWNMGVCIPISCPGNMRQIGTCFGPRVPCCR
>ID12069-NO
NPRLYE
>ID12070-NO
NPTCWAICKRIPNKK
>ID12071-NO
NPVLVKDATGSTQFGPVQALGAQYSMWKLK
>ID12073-NO
NQNAQALNTLVKQLSSNFGAISSVLNDILSRLDKVEAEVQIDRLIT
>ID12077-NO
NQVGD
>ID12078-NO
NREILKEPVHGVYYDPSKDL
>ID12079-ABP_both
NRFTARFRRTPWRLCLQFRQ
>ID12080-NO
NRLKKNEDFQRVGLSVSKKIGNAVMRNRIKRLIRQLKDYIITKKSL
>ID12082-NO
NRLRRREDFARAGFVVSKAVGVAVVRNKVKRRLRHLPLVVVLARDL
>ID12083-NO
NRLRRREDFARIGIVVSKKVSKLAVTRNRFKRQLRALKQIVVLGDDL
>ID12084-NO
NRMRRSADFERVGLIIAKSVGSAVERHRVARRLRHLHDHVVILEQQL
>ID12085-NO
NRMRRSSEFDHVGLIIAKTVGSAVERHRVARRLRHLGDQVVILAQQL
>ID12086-NO
NRMTRSTEFDRVGLVVGKAVGTAVQRHRVARRLRHLEDRLVILAQEL
>ID12088-NO
NRRPQDVKFPGGGQIVGGV
>ID12089-NO
NRRPQDVKFPGGGQIVGGVY
>ID12092-NO

NSHAIYP
>ID12094-ABP_both
NSQIRPLPDKGLDLSIRDASIKIRGKWKARKNFIK
>ID12096-NO
NTTHYRVIRLTIG
>ID12098-NO
NVQGKTGIQQLQKWEDWVRW
>ID12099-NO
NVRFDLSGATSSSYKTFIKN
>ID12100-ABP_both
NVWKKILGKIIKVAK
>ID12102-NO
NWCKRGRKQCKTHPH
>ID12104-NO
NWIRWRKWR
>ID12106-NO
NYAVVSHT
>ID12107-NO
NYRLVNAIFSKIFKKKFIKF
>ID12108-NO
NYTEGIAVVFKENIA
>ID12110-NO
PAARKAARWAWRMLKKGA
>ID12111-NO
PAARKAFRWAWRMLKKAA
>ID12112-NO
PAATLEEHMTA
>ID12113-NO
PACQDFLGAMIHLKAKTNISIR
>ID12114-NO
PAFCQAVGWGDPITHWSHGQ
>ID12115-NO
PAIAQRATATLGTVGSNTSGTTEIEACILL
>ID12116-NO
PAICQRAEIEACILL
>ID12117-NO
PAICQRATATLGTVGSNTSGTTAIEACILL
>ID12118-NO
PAICQRATATLGTVGSNTSGTTEIAACILL
>ID12121-NO
PAIEPPTGTFGFFPGVPP
>ID12123-NO
PALNCYWPLNDYGFYTTSGI
>ID12124-NO
PANAATRTSRGWHTT
>ID12125-NO
PAQPFRFPKH
>ID12126-NO
PAQPFRIKK
>ID12127-NO
PATPAPPPLGAAPTG

>ID12128-NO
PAWAKAFRAAARMKLKAA
>ID12129-NO
PAWFKARRWAWRMKKLAA
>ID12130-NO
PAWFKARRWAWRMLKKAA
>ID12131-NO
PAWRKAARWAWRMLKKAA
>ID12136-NO
PAWRKAFRWAWRMKKLAA
>ID12137-NO
PAWRKARRWARRMKKLAA
>ID12139-NO
PCKNFFWKTFSSCK
>ID12140-NO
PDAPRTCYHKPILAALSRIVVTDR
>ID12143-NO
PDKDFIVNPSDLVLDNKAALRDYLRQINEYFAIIGRPRF
>ID12144-NO
PDKSESELVSQIIEQ
>ID12145-NO
PDKWTVQPIVLPEKDSWTVN
>ID12146-NO
PDVDLGDISGINASVVNIQKEIDRLNEVAKNLNESLIDLQ
>ID12148-NO
PEDQFNVALDQVFESIENSQALVDQSNRILSSAEKGNTG
>ID12149-NO
PEDQGPQREPYNEWT
>ID12150-NO
PEGRTWAQPGYPWPLYGN
>ID12152-NO
PFCNAFTGC
>ID12153-NO
PFDYGLKWQSCSCRANGSRI
>ID12154-NO
PFFHLIG
>ID12155-NO
PFLWMGYELHPDKWTVQPIV
>ID12156-NO
PFRKQNPDIVIYQYM
>ID12160-NO
PGAGSQEERMQGQMEGQDFSHEERFLSMVRE
>ID12161-NO
PGDPDLSDGSWSTVSSGA
>ID12162-NO
PGGETARKDKFLHMVLPRRL
>ID12164-NO
PHAQLLRVGCVLGTCQVQNLSHRLWQLVRPAGRRDSAPVDPSSPHSY
>ID12165-NO
PHAQLLRVGCVLGTCQVQNLSHRLWQLVRPSGRRDSAPVDPSSPHSY
>ID12167-NO

PIPKARRPEGRTWAQPGY
>ID12170-NO
PKDGPSPGGTLMDLSERQEVSSVRSLSST
>ID12172-NO
PKLLKTFLSKWK
>ID12175-NO
PKSSWSDHEASSGV
>ID12178-NO
PLETLMAKAIDAGFIR
>ID12182-NO
PLLQATLGGGS
>ID12185-NO
PLNNCLLLGTEVSEALGGAGLTGG
>ID12186-NO
PLSPPLRNTHPQAMQWNSTTF
>ID12187-NO
PLSSIFSRIGDP
>ID12188-NO
PLVEGQLGENNELRL
>ID12189-NO
PNDITLNNSVALDPIDISIELNKAKSDLEESKEWI
>ID12190-NO
PNGPWVWVPAFCQAVGWGDP
>ID12191-NO
PNITLNNSVALDPIDISIELNKAKSDLEESKEWI
>ID12192-NO
PNQTCMWNTSQIQDPEIPKC
>ID12194-NO
PPARSSPPMPPNLPPLRRRIILLRFLFH
>ID12195-NO
PPATHTIADRNHTPFSDV
>ID12196-NO
PPCPSCPSCPWCPMCPRCPSCKCNPK
>ID12197-NO
PPCYERMPRRLIRPPPLLSVLLILRLCH
>ID12198-NO
PPDEGYPPMRMQVPPLGGRIILVRVLLHLNKIRIRAREIYES
>ID12199-NO
PPDGCNRPQQSRKPPPRCLICLMVGLIHLNKIRIRAREIYES
>ID12200-NO
PPDQTTYPSAECPPPPLVSILLIGLWLH
>ID12201-NO
PPDTSLLPPVGLHLVVRLFLLRLSVH
>ID12202-NO
PPEPSDLPPGNPVPPPLNYGDPTKLRSPP
>ID12203-NO
PPESNHPPWKGPYPPPTSNVNPLILLSHLNKIRIRAREIYES
>ID12204-NO
PPFLSYLPAETDFPPPEARETPEPFSNPP
>ID12205-NO
PPGAPPAPFRTHTPPPRMVIVLIRVWCH

>ID12206-NO
PPGAPPQPDSVCELHLLCVLRLLVIRIH
>ID12207-NO
PPGETPTPVSDLWPPPSYQGRPSRQGHPP
>ID12209-NO
PPGKQHTPTSFTHPPPADILLPLSAMIHLNKIRIRAREIYES
>ID12210-NO
PPGSLGWPPNTTEPPPYLRLRLIFLFIHLNKIRIRAREIYES
>ID12211-NO
PPGTGEEPEQAGLPPPELLVEPDYMWSPP
>ID12212-NO
PPINNCMPLGTEVSEALGGA
>ID12213-NO
PPIPIPDPPQRNRPPPRWFISLMVIRIH
>ID12215-NO
PPLASMSPLPATDPPPLEGAHPELGVPPP
>ID12217-NO
PPLFEDTPMVNSIPPLRVRLFLLRLVFH
>ID12219-NO
PPLPSVTKLTEDRWN
>ID12220-NO
PPLRYSPPGQRVIPPPADERFLRFLVFHLNKIRIRAREIYES
>ID12221-NO
PPLVVPVEESEIIFWDCWAPEE
>ID12222-NO
PPLYGRDPTTRRMPHLLLRCCLLRLVVH
>ID12224-NO
PPPEE
>ID12225-NO
PPPLGAAPTGDPKPK
>ID12226-NO
PPPLPYGPNRNGEPHLRVLLRLLCIRLH
>ID12227-NO
PPPPGASANASVERI
>ID12228-NO
PPPTHQSPNTTDMPPPATLSGPTGINRPP
>ID12229-NO
PPRGAHRPNSTVVLHLVIRLCLLRFVVH
>ID12230-NO
PPRGTETPQRCRRLHLVEMLCLVRVVFH
>ID12232-ABP_both
PPRLPRPRPRPLPFPRPG
>ID12233-NO
PPRTTPIPHLDVSLHLLILRILRVRVH
>ID12234-NO
PPSAVCNYEVAEDPSYVREH
>ID12235-NO
PPSHRTTPASRSLPPLRVFVVLSFSGVHLNKIRIRAREIYES
>ID12236-NO
PPSHSFRPESLERLHLLRRVLLLMRIVH
>ID12237-NO

PPTCYVPPHSPATPPPSVRISLLRLVFTLIRSEFELERSMNRRY
>ID12238-NO
PPTSPLLPTVNLRPPPIIIVFLLRVWFH
>ID12239-NO
PPVCETGPLHPPSPPPLVRISLILVFVHLNKIRIRAREIYES
>ID12240-NO
PPVYTKDVDISSQISSMNQSLQQSKDYIKEAQKILDTVNPSL
>ID12242-NO
PQGPQTRPPI
>ID12243-NO
PQMELRRNGRQCGMSEKEEE
>ID12247-NO
PRAWEYWLRLME
>ID12248-NO
PRFWEAWLRLME
>ID12249-NO
PRFWEYALRLME
>ID12250-NO
PRFWEYWLALME
>ID12252-NO
PRFWEYWLRLAE
>ID12253-NO
PRFWEYWLRLME
>ID12254-NO
PRHHWTTQDCNCSIYPGH
>ID12255-NO
PRPAGDNATVAAGHA
>ID12257-NO
PRPGPIYY
>ID12259-NO
PRPISYLKGSSGGPL
>ID12260-ABP_both
PRPLPFPRPG
>ID12261-ABP_both
PRPPRLPRPRPRPLPFPRPG
>ID12262-ABP_both
PRPRPLPFPRPG
>ID12263-ABP_both
PRPRPRPLPFPRPG
>ID12264-ABP_both
PRRRK
>ID12265-ABP_neg
PRRRTPSPRRRRSQSPRRRRS
>ID12266-ABP_both
PRVIETKVHGREVTGLARNVSEENVDRLAKRWIK
>ID12267-NO
PSGSNIISNLFKED
>ID12268-NO
PSLFSWGFGS
>ID12269-NO
PSQPTYPGDDAPLEDLMAFAIDLSFYLGVV

>ID12270-NO
PSRVEAFHRYGTTVN
>ID12271-NO
PSSKRFQPFQQFGRDVSDFT
>ID12272-NO
PSVCTMTKWQEVDEM
>ID12273-NO
PTGERVWDRGNVTLLCDCPN
>ID12277-NO
PTTCANDP
>ID12278-NO
PTTFMLKYDENGTITDAVDC
>ID12279-NO
PVDCTMYSNKMYNCSLQNGF
>ID12280-NO
PVKVYPNGAEDESAEAFPLEF
>ID12281-NO
PVKVYPNGAEDESAQAFPLEF
>ID12282-NO
PVKVYPNVAENESAEAFPLEF
>ID12283-NO
PVLQPALSLSCGPEPLLLSC
>ID12285-NO
PVSRQQCSQRIQGERFNQC
>ID12288-ABP_both
PWWPWRR
>ID12294-NO
PYVGSGLYRR
>ID12295-ABP_neg
PYVRYL
>ID12296-NO
QAGLAVRPGKSAAQLVGE
>ID12297-NO
QARQLLSQIVQQQNNLLRAIEAQQHLLQ
>ID12298-NO
QATVGDVNTDRPGLLDLK
>ID12302-NO
QDDGSEATGLLLGEAEKVGGLLGTLAEELNGYSRKKGGFSFRF
>ID12303-NO
QDEGSEATGFLPAAGEKTSGPLGNLAEELNGYSRKKGGFSFRF
>ID12305-NO
QDSGSEATGFLPTDSEKASGPLGTLAEELSSYSRRKGGFSFRF
>ID12306-NO
QDYCAQEGQQEVQRKDLSDLERYLRQSRQR
>ID12308-NO
QEDAELQPRALDIYSAVEDASHEKELIEALQEVLKKLKS
>ID12309-NO
QEFFGLNNCSSSNRLD
>ID12311-NO
QEGAPPQQSARRDRMPCRNFFWKTFSSCK
>ID12312-NO

QERPPLQQPPHRDKKPCKNFFWKTFSSCK
>ID12313-NO
QETFSDLWKLLP
>ID12314-NO
QFEQPRRCPTRPEGQ
>ID12315-NO
QFMGIFEDRAPVPFE
>ID12317-NO
QGCCNGPKGCSSKWCRDHARCC
>ID12318-NO
QGGGPQWAVGHFM
>ID12320-ABP_both
QGGQANQ
>ID12322-NO
QGILR
>ID12323-NO
QGILRAGPAIALVGDARAVG
>ID12324-NO
QGKRPWIL
>ID12325-NO
QGMIGTLTSKRIKQ
>ID12327-NO
QGPWLEEEEEAYGWMDF
>ID12328-NO
QGPWVEEEEAAYGWMDF
>ID12332-NO
QHGTAPECFWKYCI
>ID12335-NO
QHSRIGITQQRRARN
>ID12336-NO
QHWSHGWYPG
>ID12338-NO
QIPLINYTFGPNQTCMWNTS
>ID12339-NO
QIPWFHR
>ID12340-NO
QIQDPEIPKCGWWNQMAYYN
>ID12341-NO
QITAAVALVEAKQARSDIEKLKEAIRDTNKAVQSV
>ID12342-NO
QIVGGVYLLPRRGPRLGV
>ID12343-NO
QIWNNMTWMEWDREINNYTSLIHSLIEESQNQ
>ID12345-ABP_both
QKALNEINQF
>ID12346-NO
QKAVLDCLKAAGSSLSKAAITAI
>ID12347-ABP_both
QKKARVRLSA
>ID12348-NO
QKKFRVRLSA

>ID12350-ABP_both
QKKIRARLSA
>ID12352-NO
QKKIRIRLSA
>ID12353-ABP_both
QKKIRVALSA
>ID12354-ABP_both
QKKIRVRASA
>ID12355-NO
QKKIRVRFSA
>ID12358-ABP_both
QKKIRVRLAA
>ID12361-NO
QKKIRWRLSA
>ID12363-NO
QKQGQGQWTYQIYQEP
>ID12364-NO
QKQIANQFNKAISQIQESLTTTSTALGKLQDVVNQNAQALNTLVKQ
>ID12365-ABP_both
QKRIRVRLSA
>ID12366-NO
QKWEDWVRWIGN
>ID12367-NO
QKWYRQRRN
>ID12368-NO
QLESLTDRELLLLIARKTCGSVE
>ID12369-NO
QLGENNELRLTRDAI
>ID12370-NO
QLGLQDPPHMVADLSKKQGPWVEEEEAAYGWMDF
>ID12371-NO
QLGPQGPPHLVADPSKKQGPWLEEEEEAYGWMDF
>ID12372-NO
QLGPQGPQHFIADLSKKQRPPMEEEEEAYGWMDF
>ID12373-NO
QLIHLYYFDCFSESA
>ID12374-NO
QLLVDLI
>ID12375-NO
QLQKWEDWVRWI
>ID12376-NO
QLQKWEDWVRWIGNIPQYLKG
>ID12377-NO
QMMLTLLWAFWY
>ID12378-NO
QMRRKVELFTYMRFD
>ID12381-NO
QNQSANQFQKEISQINEVLTTTNTSLGKLQDDVNQNNQSLNTLQKE
>ID12382-NO
QPELAPEDPEDSALLEDPVGTVAPQ
>ID12384-NO

QPGYPWPLYGNEGCGWAG
>ID12386-NO
QPRGRRQPIPKARRPEGR
>ID12387-NO
QPSKDAFIGLM
>ID12388-NO
QPSVQIQVYQGEREIAAHNK
>ID12389-NO
QPWIPFV
>ID12390-NO
QPWLPFG
>ID12391-NO
QPWLPFV
>ID12392-NO
QPYVALFEKCCLIGCTKRSLANYC
>ID12395-NO
QQETAAAETETRTHT
>ID12396-NO
QQRKRKGIGAVLKVLTTGLPALISWI
>ID12397-NO
QQRLGNQWAVGHLM
>ID12398-NO
QQRRARNGASRS
>ID12400-NO
QRGRWLRRG
>ID12401-ABP_both
QRKIRVRLSA
>ID12402-NO
QRKTKRNTNRRPQDVKFP
>ID12405-NO
QRLGHQWAVGHLM
>ID12406-NO
QRLGNQWAVGHLM
>ID12408-NO
QRPPMEEEEEAYGWMDF
>ID12409-NO
QRQIRSISGWILSTYLGRPAEPVPLQ
>ID12410-NO
QRQQLFDSQQGWFEG
>ID12411-ABP_both
QRRIRVRLSA
>ID12412-NO
QRRNQLHDLRFADID
>ID12413-NO
QRRWWGRFK
>ID12415-NO
QSFGNQWARGHFM
>ID12416-NO
QSGALLSEQCCHIGCTRRSIAKLC
>ID12418-ABP_both
QSKKCCGRCSSRMCTKREKEEHTEDCRGSFCCLTHRKKK

>ID12421-NO
QTCVSCVNFGNGFCGDNCGNSWACSGC
>ID12424-NO
QTPCSSAKKVRCNVHCRGYTKLGSCYDDNCSCVDKPAAMKASFAA
>ID12425-NO
QTVYLIFRR
>ID12426-NO
QTWLPFV
>ID12427-NO
QVDGFYARDLTTKAR
>ID12428-NO
QVDPDLADQLIHLYY
>ID12429-NO
QVPQWAVGHFM
>ID12430-ABP_both
QVRWWGRYWRRKWATCR
>ID12435-NO
QWQRNMRKVR
>ID12436-NO
QWTYQIYQEPFKNLKTGKYA
>ID12437-NO
QWWWKYRWK
>ID12438-NO
QYDRVAELDQLLHY
>ID12440-NO
QYMDDLYVGSDLEIG
>ID12441-NO
RADITTVSTFIDLNI
>ID12442-NO
RAFVTIGK
>ID12443-NO
RAGLQFPVGRLLR
>ID12447-NO
RAVWRHSVATPSHSV
>ID12449-NO
RCGTGPRLTKDLEAVPFVNR
>ID12450-NO
RDCCTPPKKCKDRQCKPQRCCA
>ID12452-NO
RDPCCSNPVCTVHNPQIC
>ID12453-NO
RDPCCYHPTCNMSNPQIC
>ID12455-NO
RDVSDFTDSVRDPKTSEILD
>ID12458-NO
RECKTESNTFPGICITKPPCRKACISEKFTDGHCSLLRRCLCTKPC
>ID12459-NO
REHLREQSRKPPNPT
>ID12461-ABP_neg
RENTQQHITARRAIRHPQYN
>ID12462-NO

RERLKQRQQLFDSQQ
>ID12463-NO
RESKLIAMADMIRRRI
>ID12465-NO
RFGRFLRKIRR
>ID12466-NO
RFGRFLRKIRRF
>ID12469-ABP_both
RFKRVARVIW
>ID12470-NO
RFKWFIRRF
>ID12471-NO
RFKWWRRYW
>ID12472-NO
RFKYWRWWQ
>ID12473-NO
RFLRKIRRFRPK
>ID12474-NO
RFRIRCRWNVGSDTSLIDTC
>ID12475-NO
RFTWWWRKF
>ID12476-NO
RFWKKIRRH
>ID12477-ABP_both
RGGLCYCRGRFCVCVGR
>ID12479-NO
RGGRLCYCRRRFCVCA
>ID12484-NO
RGGSVVIVGRIILSGRK
>ID12485-NO
RGILIHNTIFGEQVF
>ID12486-NO
RGILRNWYNPFAGLRQSLEQ
>ID12487-NO
RGIYR
>ID12491-NO
RGNVTLLCDCPNGPWVWVPA
>ID12493-ABP_neg
RGRKVVRRKK
>ID12494-ABP_neg
RGRKYYRRKK
>ID12499-NO
RHEIKDSGLLDYTEV
>ID12500-NO
RHHYESTHPRISSEV
>ID12501-NO
RHIDLLVGSATLCSALYV
>ID12502-NO
RHKRRR
>ID12503-NO
RHLCW

>ID12504-NO
RHRCCAWGPGRKYCKRWC
>ID12505-NO
RHWCW
>ID12508-NO
RIGCQHSRIGITQQR
>ID12509-NO
RIGRKLRIR
>ID12510-NO
RIGRNPSQVGD
>ID12511-NO
RIILGQCIKREAEAAIEQIFRTKYNDSH
>ID12513-ABP_both
RIPCQYEDATEDTICQQHCLPKGYSYGICVSYRCSCV
>ID12515-NO
RIQRGPGRAFVTIGK
>ID12516-ABP_both
RIRFPWPWRWPWWPPFRG
>ID12517-NO
RIRKGWKWG
>ID12518-ABP_both
RIRKKLR
>ID12519-NO
RIRRKRKGR
>ID12520-NO
RIRRLWNIV
>ID12521-NO
RIRRVLVYV
>ID12523-ABP_both
RIRWILRYWRWS
>ID12524-NO
RIRYIQRVW
>ID12525-ABP_neg
RIVIVRIRRLFV
>ID12526-ABP_neg
RIVQRIKDFLRNLVPRTES
>ID12528-ABP_neg
RIVRVRIARLQV
>ID12529-ABP_neg
RIVWVRIRRLQV
>ID12530-ABP_neg
RIVWVRIRRWFV
>ID12532-NO
RIWILSWRW
>ID12533-NO
RIWMFKIFR
>ID12534-NO
RKAFRWAWRMLKKAAPSITCVR
>ID12535-NO
RKAILGHIVSPRCEY
>ID12537-ABP_both

RKCLRWQWAMRKYGG
>ID12545-ABP_both
RKFRWWVIR
>ID12546-ABP_both
RKKKAKIIKKII
>ID12550-ABP_both
RKKKIKIIKKII
>ID12551-ABP_both
RKKKIKIIKRLI
>ID12554-ABP_both
RKKKLKIIKRLI
>ID12555-ABP_both
RKKKLKVVKRLV
>ID12556-ABP_both
RKKKVKLLKRLV
>ID12561-ABP_both
RKKRLKLLKRLV
>ID12562-ABP_both
RKKRLKVVKRLV
>ID12563-ABP_both
RKKRLRVVRRLV
>ID12564-NO
RKKRRQRRRGKKKKKKKKKKKKKKKGRKKRRQRRR
>ID12565-NO
RKKRRQRRRGKKKKKKKKKKKKKKKKKKKKGRKKRRQRRR
>ID12566-ABP_both
RKKRVKLLKRLV
>ID12569-ABP_both
RKRLKWWIY
>ID12570-NO
RKRLYRWIK
>ID12571-NO
RKRRKHWRY
>ID12573-NO
RKRWFVYIW
>ID12574-ABP_neg
RKSKEKIGKEFKRIVQRIK
>ID12577-ABP_neg
RKTWFW
>ID12578-NO
RKVWWRVFY
>ID12579-ABP_neg
RKWHFW
>ID12580-ABP_both
RKWIWRWFL
>ID12581-ABP_both
RKWKIKWYW
>ID12582-ABP_both
RKWLFW
>ID12583-NO
RKWWWKWRR

>ID12584-NO
RKYRRRYRR
>ID12585-NO
RKYVWWKSI
>ID12586-NO
RLASWSVKQANLTTSLLGDLLDDVT
>ID12587-NO
RLCIPVIFPC
>ID12588-NO
RLCYTRGRFTVCV
>ID12590-NO
RLFTWEG
>ID12591-ABP_both
RLGRLVSLHTLG
>ID12592-NO
RLGVRATRKTSERSQPRG
>ID12594-NO
RLIRWWRKR
>ID12595-ABP_both
RLKRWWKFL
>ID12596-ABP_both
RLLGNCLTVVMAAKLGTAFSPEIQCAWQK
>ID12599-ABP_both
RLLRRLLRRLLRRLLRRLLR
>ID12600-ABP_both
RLMKCYKPNSRGFQLCE
>ID12601-NO
RLNNRARIILRA
>ID12602-ABP_both
RLPRPRPRPLPFPRPG
>ID12603-NO
RLRLWKSKR
>ID12605-NO
RLTGLLATSGFAFVNAAHANGAVCLSDLLGFLAHSRALAG
>ID12606-NO
RLVSKRRRK
>ID12607-ABP_both
RLWRIVVIRVKR
>ID12608-ABP_both
RLWWKIWLK
>ID12609-ABP_both
RLWWWWRRK
>ID12619-NO
RNKSNLHTEARFRIRCRWNV
>ID12620-NO
RNPNQVGD
>ID12621-NO
RNPSQVGD
>ID12622-NO
RNPVCPGYAWLSSGRPDGFI
>ID12623-NO

RNRTHWWRK
>ID12624-NO
RNTREVFAQVKQMYKTPTLKYFG
>ID12626-NO
RPAPYGVKLCGREFIRAVIFTCGGSRW
>ID12630-NO
RPETVETTVTTAQETKRGRI
>ID12632-NO
RPGPPGLQGRLQRLLQANGNHAAGILTM
>ID12633-ABP_neg
RPGTLRLCTVAGWGRVSMRRGT
>ID12635-NO
RPKHPIKHQ
>ID12636-ABP_both
RPKKVQGRKAEKDNGDGTTAANASGKKKSSNVFK
>ID12637-NO
RPLSWLPK
>ID12638-NO
RPPGCNPFR
>ID12640-NO
RPPGFSPFRIAPAS
>ID12641-NO
RPPGFSPFRVAPAS
>ID12644-ABP_both
RPPQFTRAQWFAIQHISLNPPRSTIAMRAINNYRWRSKNQNTFLR
>ID12646-NO
RQGPFERP
>ID12647-NO
RQHLLRWGLTTPDKKHQKEP
>ID12648-NO
RQIKINFQNRRMKNKKGELDELVYLLDGPGYDPIHS
>ID12649-NO
RQIKIWFPNRRMKWKK
>ID12651-NO
RQLVLRTR
>ID12652-NO
RQMRAWGQDYQHGGMGYSC
>ID12653-ABP_both
RQRRVVIWW
>ID12658-NO
RRFFWKKGI
>ID12659-ABP_both
RRFKFIRWW
>ID12660-ABP_neg
RRFRRTVGLSKFFRKARKKLGKGLQKIKNVLRKYLPRPQYAYA
>ID12661-NO
RRGCTERLRRMARRNAWDLYAEHFY
>ID12664-NO
RRGRIGRNPSQVGD
>ID12665-NO
RRGRTGRGRRGIYR

>ID12666-NO
RRGRVGRNPNQVGD
>ID12667-NO
RRGWRLVLALVY
>ID12669-NO
RRGWVLDLVLYYGRR
>ID12670-NO
RRGWVLRLALAY
>ID12671-NO
RRHGDEDMFYMHVRG
>ID12672-NO
RRHLCW
>ID12673-NO
RRHWCW
>ID12674-ABP_neg
RRIPFWPPNWPGPWLPPWSPPDFRIPRILRKR
>ID12677-ABP_both
RRIRPRP
>ID12678-ABP_both
RRIRPRPPRLPRPRP
>ID12679-ABP_both
RRIRPRPPRLPRPRPRP
>ID12680-ABP_both
RRIRPRPPRLPRPRPRPLPFPRPG
>ID12681-NO
RRKKAAVAALPAVLLALLAP
>ID12682-NO
RRKKAAVAELPAVLLALLAP
>ID12683-NO
RRKKAAVAKLPAVLLALLAP
>ID12684-NO
RRKKAAVALAPAVLLALLAP
>ID12685-NO
RRKKAAVALEPAVLLALLAP
>ID12687-NO
RRKKAAVALLKAVLLAALAP
>ID12689-NO
RRKKAAVALLKAVLLALKAP
>ID12699-NO
RRKKAVLLALLAP
>ID12700-NO
RRKKIFYFFR
>ID12703-NO
RRKRRR
>ID12704-NO
RRKVFKLRR
>ID12705-NO
RRKYWKKYR
>ID12707-NO
RRLMAAKAES
>ID12708-NO

RRLQVGGGTLKFFLT
>ID12709-NO
RRLRPRRPRLPRPRPRPRPRP
>ID12710-NO
RRMKWKK
>ID12711-NO
RRQRRTSKLMKR
>ID12712-NO
RRQRSICKQLLKKLRQQLSDALQNNDD
>ID12715-ABP_both
RRRIKIRWY
>ID12716-NO
RRRITRKRI
>ID12719-NO
RRRPRPPYLPR
>ID12720-ABP_both
RRRPRPPYLPRPRPPPFFPPRLPPRI
>ID12721-NO
RRRPRPPYLPRPRPPPFFPPRLPPRIPPFPPRFPPRFP
>ID12722-NO
RRRQRRKKR
>ID12723-ABP_both
RRRQRRKKRGGGDTRLNTVWMW
>ID12724-NO
RRRQRRKKRGKKKKKKKKKKKKKKKKKKKKGRRRQRRKKR
>ID12725-NO
RRRRHLCW
>ID12727-NO
RRRRMRKKI
>ID12728-NO
RRRRR
>ID12729-ABP_both
RRRRRFRRVIRRIRLPKYLTINTE
>ID12730-NO
RRRRRNWMWC
>ID12732-NO
RRRRRRRRGEDIIRNIARHAAQVGASMDR
>ID12735-NO
RRRRRRRRGNLWAAQRYGRELRRMSDEFVDSFKK
>ID12736-ABP_both
RRRRRRRRRRRR
>ID12742-NO
RRRRRWCMNW
>ID12743-ABP_both
RRRRRYRYWRRGLTIQGRPKSLPLNTGD
>ID12744-NO
RRRRWIRKK
>ID12746-ABP_both
RRRRWWW
>ID12747-NO
RRRSRNLGKVIDTLTCGF

>ID12748-ABP_neg
RRRWIVVIWRRR
>ID12749-NO
RRRWKIYKW
>ID12750-ABP_both
RRRWWKLMM
>ID12758-ABP_neg
RRRWWWRRWWRR
>ID12761-ABP_pos
RRSVQWCA
>ID12762-NO
RRSWWYKRR
>ID12770-ABP_neg
RRWIRVAVILRV
>ID12771-ABP_neg
RRWKIVVIRWKK
>ID12773-ABP_both
RRWQWRMKKL
>ID12774-ABP_both
RRWQWRMKKLGA
>ID12775-ABP_neg
RRWRRWWRRWRR
>ID12776-ABP_both
RRWRVIVKW
>ID12777-NO
RRWVIRIYK
>ID12779-NO
RRWWCRC
>ID12780-NO
RRWWCRD
>ID12781-NO
RRWWCRE
>ID12782-NO
RRWWCRF
>ID12783-NO
RRWWCRG
>ID12785-NO
RRWWCRI
>ID12786-NO
RRWWCRL
>ID12788-NO
RRWWCRN
>ID12789-NO
RRWWCRP
>ID12790-NO
RRWWCRQ
>ID12791-NO
RRWWCRR
>ID12792-NO
RRWWCRS
>ID12793-NO

RRWWCRT
>ID12794-NO
RRWWCRV
>ID12795-NO
RRWWCRW
>ID12796-NO
RRWWCRY
>ID12800-ABP_both
RRWWRWVVW
>ID12801-NO
RRWWWWRFN
>ID12802-ABP_both
RRWYRWAWRMR
>ID12803-NO
RSAVGRHGRRFGLRKHRKH
>ID12804-NO
RSCCPCYWGGCPWGQNCYPEGCSGPKV
>ID12805-NO
RSCIDTIPKSRCTAFQCKHSMKYRLSFCRKTCGTC
>ID12806-NO
RSFWWMWFK
>ID12807-ABP_both
RSGGYCNGKRVCVCR
>ID12808-NO
RSISGWILSTYLGRP
>ID12809-ABP_both
RSITRPVLVRRRWRVRPVF
>ID12810-ABP_both
RSLCLLHCRLK
>ID12811-NO
RSLLGRMKGA
>ID12812-NO
RSLQDTEEKSRSFSASQADPLSDPDQMNED
>ID12813-NO
RSLQNTEEKSSSFPAPQTDPLGDPDQINED
>ID12814-ABP_both
RSMRLSFRARGYGFR
>ID12815-ABP_both
RSPPQCQYTNCAAVLCPAVYCANAYTPPCGCCDICPPQKYGGGYRPR
>ID12817-NO
RSSCFGGRIDRIGAQSGLGCNSFR
>ID12818-NO
RSSCFGGRIDRIGAQSGLGCNSFRY
>ID12822-NO
RTCQRASQTWKGVCFRNEKCRNNCLREKARTGNCKYVFRACICYFPC
>ID12823-NO
RTGRGRRGIYR
>ID12824-ABP_both
RTKKWIVWI
>ID12826-NO
RTSKKR

>ID12827-NO
RVAAAAITWVPKPNVEVWPVDPPPPVNFNKTAEQEYGDKEVKLPHW
>ID12829-NO
RVDLGDCIGKDARDA
>ID12830-NO
RVGRNPNQVGD
>ID12831-NO
RVIKKNFEFQKYGISVGKKIGNAVIRNKVKRQIRMIGDIIILSKLL
>ID12833-NO
RVKKIKLFI
>ID12836-NO
RVRFKVYRV
>ID12837-ABP_neg
RVRWYRIFY
>ID12838-NO
RVSEEWMDGFIRMCGREYARELIKICGASVGRLAL
>ID12839-NO
RVSEEWMDQVIQVCGRGYARAWIEVCGASVGRLAL
>ID12840-ABP_neg
RVSSFLPWIRTTMRSFKLLDQMETPL
>ID12841-NO
RVTRTWQRW
>ID12842-NO
RVVNLNWRW
>ID12843-NO
RVVNWKGGL
>ID12845-NO
RVYIHPF
>ID12850-NO
RWLSKIWKV
>ID12851-NO
RWMVWRHWFHRLRLPYNPGKNKQNQQWP
>ID12852-NO
RWNKPQKTKGHRGSH
>ID12854-ABP_both
RWRRLLKKLHHLLH
>ID12857-NO
RWRRNWMTK
>ID12858-ABP_both
RWRRWKWWL
>ID12861-NO
RWRTRRNIV
>ID12862-NO
RWRVLKRRK
>ID12863-ABP_both
RWRWR
>ID12871-ABP_both
RWRWRWK
>ID12873-ABP_both
RWRWWWRVY
>ID12874-NO

RWVRIWKKK
>ID12875-NO
RWVRTRRRR
>ID12876-NO
RWWCC
>ID12877-NO
RWWFIRKWW
>ID12878-ABP_both
RWWIRIRWH
>ID12879-ABP_neg
RWWKWRR
>ID12880-ABP_both
RWWRKIWKW
>ID12881-NO
RWWRWRHVI
>ID12883-ABP_both
RWWRWWRRWWRR
>ID12884-NO
RWWVIWWWK
>ID12885-NO
RWYCG
>ID12888-NO
RYKRVVYRH
>ID12889-NO
RYKSRRVRR
>ID12890-NO
RYLGYL
>ID12891-NO
RYRRFRVRW
>ID12892-NO
RYRRWVRKR
>ID12893-NO
RYSTQVDPDLADQLI
>ID12894-NO
RYVKIRWVR
>ID12895-ABP_both
SADQTGMNKAALSPIRFISKSV
>ID12897-ABP_both
SALHLVLRLRGG
>ID12899-NO
SALVGCWTKSYPP
>ID12903-NO
SAYQVRNSSGLYHVTNDC
>ID12904-ABP_both
SCASRCKSRCRARRCRYYVSVRYGGFCYCRC
>ID12905-NO
SCLTVPASAYQVRNSSGL
>ID12908-NO
SCNNSCQSHSDCASHCICTFRGCGAVN
>ID12909-NO
SCNTATCVTHRLADLLSRSGGVLKDNFVPTDVGSEAF

>ID12912-NO
SCSCKRNFLCC
>ID12913-NO
SCSGRDSRCPPVCCMGLMCSRGKCVSIYGE
>ID12914-ABP_pos
SCTTCECCCSCS
>ID12919-NO
SCVSQSTGQIQCKVFDSLLNLSSTLQAT
>ID12920-ABP_pos
SCVWIPCISAALGCSCKNKVCYRNGIPCGE
>ID12921-NO
SDAAVDTSSEITTKDLKEKKEVVEEAEN
>ID12922-NO
SDDPKESEGDLHCVC
>ID12925-NO
SDHSESIQKKFQLMKEHVNKIGVDS
>ID12928-NO
SDLGAVISLLLWGRQLFA
>ID12930-NO
SDLGAVISWGRQLFA
>ID12931-NO
SDVISLLLWGRQLFA
>ID12932-NO
SDWDPLFTHK
>ID12933-NO
SEALAVDGAGKPGAEEAQDPEGKGEQEHSQQKEEEEEMAVVPQGLFRG
>ID12934-NO
SEDVVSSSMSYTFT
>ID12935-NO
SEEPPISLDLTFHLLREVLEMARAEQMAQQAHSNRKMMEIF
>ID12936-NO
SELAGTIIDGASLTFEVLDKVLGELGKVSRK
>ID12937-NO
SEPHLPFPVLPH
>ID12938-NO
SESILIVHQQQSRSSGS
>ID12939-NO
SFAIKWEYVLLLFLL
>ID12940-ABP_both
SFGAKNAVKNGLQKLRNQCQANNYQGPFCDIFKKNP
>ID12942-NO
SFKEELDKYFKNHTSPDVDLGDISGINASVVNIQKEIDRLNE
>ID12943-NO
SFLDTVKNMALNAAKSAGVSVLKTLSCKLSKEC
>ID12944-ABP_both
SFLNFFKGAAKNLLAAGLDKLKCKISGTQC
>ID12945-NO
SFLSKFKDIALDVAKNAGKGVLTTLACKIDGSC
>ID12946-NO
SFLSKFKDIALDVPRMRARVY
>ID12947-NO

SFLTSFKDMAIKVAKDAGVNILNTISCKISKTC
>ID12948-NO
SFMPEIQKNTIPTQMK
>ID12950-NO
SFYSVLFLWGTCGGFSHSWY
>ID12951-NO
SGCANNTCYNVSVIVPDYQC
>ID12952-NO
SGELEQEEERLSKEWEDS
>ID12955-NO
SGIVQQQNNLLRAIEAQQHLLQLTVWGIKQCCGR
>ID12956-NO
SGIVQQQNNLLRAIEAQQHLLQLTVWGIKQCCGRISGIVQQQNNLLRA
>ID12958-ABP_both
SGKLCCRRKK
>ID12959-ABP_both
SGKLWWRRKK
>ID12960-ABP_both
SGKLYYRRKK
>ID12961-ABP_both
SGKRWWRRKK
>ID12964-NO
SGSAKVAFSAIRSTNH
>ID12966-NO
SGTKMACPSNRGILRNWYNP
>ID12967-NO
SGTLSTFFRLFNRSFTQA
>ID12968-NO
SHQLSRADITTVSTF
>ID12970-ABP_both
SICCSFPDPWGGLCCEDHCSYIGKPGGQCSDKGVCTCN
>ID12973-NO
SIGRRGGYCAG
>ID12974-ABP_both
SIKILKIYFIQGKRHWSF
>ID12976-NO
SISCSRGLVCLLPRLTNESGNDRFDS
>ID12977-NO
SISNALNKLEESNRNLDKVNVKLT
>ID12978-NO
SIVYEAADAILHTPGCVP
>ID12979-NO
SIWRDWVDLICEFLSDWK
>ID12981-ABP_both
SKCRQWQWKIRRTNP
>ID12983-ABP_both
SKCYQWQWRMRKLGA
>ID12984-NO
SKFKVLRKIIIKEYKGELMLSIQKQR
>ID12986-NO
SKIDVWSLVPVGSASCTIAA

>ID12988-NO
SKLLKSTNFQRLGLSISRKNIKHAYRRNKIKRLIRELDFVVIVNIL
>ID12991-NO
SLEQIWNNMTWMQWDK
>ID12994-NO
SLGQHIYETYGDTWA
>ID12995-NO
SLHWPVSHPPPP
>ID12997-NO
SLIGGLVSAFK
>ID12998-NO
SLIGRL
>ID12999-NO
SLKIPNLD
>ID13001-NO
SLLGRMK
>ID13002-NO
SLLGRMKG
>ID13004-NO
SLLSSPQ
>ID13005-NO
SLQPGAPNVNNKDQPWQVSPHISRDDSGNTRTDINVQRHGENNDFEA
>ID13006-NO
SLQSQLGPCLHDQRH
>ID13007-NO
SLTFEVLDKVLGELGKVSRK
>ID13008-NO
SLTFQVLDKVLEELGKVSRK
>ID13009-NO
SLTLN
>ID13010-ABP_both
SLWETIKNAGKGFILMILDKIRCKVAGGCKT
>ID13011-ABP_both
SMATPHVAGAAALILSKHPTWTNAQVRDRLESTATYLGNSFYYGK
>ID13013-NO
SMIENLEYM
>ID13014-NO
SMRISSRPGACYSRP
>ID13015-NO
SMTDFYHSKRRLIFS
>ID13016-NO
SNFASITTPRPH
>ID13019-NO
SNNTIAIPTNFSISITTEVM
>ID13021-NO
SPAFLFQPQRF
>ID13023-ABP_both
SPGGA
>ID13024-NO
SPKMVQGSGCFGRKMDRISSSSGLGCKV
>ID13027-NO

SPLGFGSYTMTKIRDSLHLV
>ID13029-NO
SPQYWTGPA
>ID13030-NO
SPRGSRPSWGPTDPRRRS
>ID13031-NO
SPTGPPGSIRTWFQRIPLGWFHCTYQKGKQHCRLRIRQKVEE
>ID13032-NO
SPYHTQP
>ID13033-NO
SPYPMALFPLHN
>ID13035-ABP_both
SPYRCGSPDSRGSENTRCLIKK
>ID13037-NO
SQDSAFRIQERLRNSKMAHSSSCFGQKIDRIGAVSRLGCDGLRLF
>ID13040-NO
SQGVVESMNKELKKIIGQVRDQAEHLKTA
>ID13041-NO
SQGVVESMNKELKKIIGQVRDQAEHLKTAY
>ID13044-NO
SQVGD
>ID13045-NO
SQVWFGHRYSQFMGI
>ID13046-NO
SRAHQHSMEIRTPDINPAWYAGRGIRPVGRF
>ID13047-NO
SRAHQHSMETRTPDINPAWYTGRGIRPVGRF
>ID13048-NO
SRPGACYSRPLVSFR
>ID13052-NO
SRVLKRKQFLRMGITVSKKFGKAHERNSFKRVVRELQIVVLLQDFINQI
>ID13054-NO
SRWRDVARNFMRRYQSRVIQGLVA
>ID13055-ABP_both
SRWRWKSSKK
>ID13059-NO
SSCNMGWDTPACCVWFPYWV
>ID13060-NO
SSFDPVKFPEDQFNVALDQVFESIENSQALVDQSNRILSSAEK
>ID13062-NO
SSGLYHVTNDCPNSSIVY
>ID13063-NO
SSLPLRK
>ID13066-NO
SSPETLISDLLMKESTENAPRTRLEDPSMW
>ID13068-NO
SSPETLISDLLMRESTENVPRTRLEDPAMW
>ID13069-NO
SSPLAHYLNAPT
>ID13070-NO
SSRRCVRLHESCLGQQVPCCDPCATCYCRFFNAFCYCRKLGTAMNPCSRT

>ID13071-ABP_pos
SSSLLNTAWRKFG
>ID13072-NO
SSSTSTQVQILSNAL
>ID13073-NO
SSTSTVTPAHST
>ID13074-ABP_both
STAHLVLRLRGG
>ID13076-NO
STCVPVAADNVIVQN
>ID13077-NO
STFTHPR
>ID13078-NO
STIGDLVKWIIDTVNKFTKK
>ID13079-ABP_neg
STLALVLRLRGG
>ID13080-ABP_both
STLHAVLRLRGG
>ID13081-ABP_both
STLHLALRLRGG
>ID13082-ABP_neg
STLHLVARLRGG
>ID13083-ABP_both
STLHLVLALRGG
>ID13084-ABP_neg
STLHLVLRARGG
>ID13089-NO
STLPETTVVRRRGRSPRRRT
>ID13090-NO
STLPLPP
>ID13091-NO
STPIPAP
>ID13092-NO
STQRRGRTGRGRRGIYR
>ID13093-NO
STRTPEDSNSLGT
>ID13097-NO
SVERIKTTSSIEFAR
>ID13100-NO
SVPHFSDEDKDPE
>ID13102-NO
SVPHFSEEEKEPE
>ID13104-NO
SVSNAATRVARTGRSRWRDVARNFMR
>ID13107-NO
SVSVGTKPRPRP
>ID13108-NO
SVSWGMKPSPRQ
>ID13109-NO
SVTKLTEDRWNKPQK
>ID13111-NO

SVVPSKATWGFA
>ID13112-NO
SWETWEREIENYTKQIYKILEESQEQQDRNEKDLLE
>ID13114-NO
SWFASTGGRDSKIDVWSLVP
>ID13115-NO
SWGPTDPRRRSRNLGKVI
>ID13116-NO
SWLDDIWDWICEVLSDFE
>ID13117-NO
SWLDRIWRWICKVLSRFE
>ID13118-NO
SWLRDIWD
>ID13119-NO
SWLRDIWDWI
>ID13120-NO
SWLRDIWDWICE
>ID13122-NO
SWLRDIWDWICEVLSD
>ID13130-NO
SWLRDVWDWICTVLTDFK
>ID13131-NO
SWLRRIWRWICKVLSRFK
>ID13132-NO
SWLWEVWDWVLHVLSDFK
>ID13133-NO
SWPLN
>ID13134-NO
SWRLDIWDWICESVLDFK
>ID13135-NO
SWRLIDWDWICEVLSDFK
>ID13136-NO
SWSTLTANQNPSPPWSKLTY
>ID13142-NO
SYSMEHFRWGKPI
>ID13143-NO
SYSMEHFRWGKPIGHKRRPIKVYASSLEGGDSSEGTFPLQA
>ID13144-NO
SYSMEHFRWGKPVGKKRRPVKVYPNGAEDESAEAFPLEF
>ID13145-NO
SYSMEHFRWGKPVGKKRRPVKVYPNGAEDESAQAFPLEF
>ID13146-NO
SYSMEHFRWGKPVGKKRRPVKVYPNVAENESAEAFPLEF
>ID13147-NO
SYSMEHFRWGKPVGR
>ID13148-NO
SYSMEHFRWGKPVGRKRRPIKVYTNGVEEESAESLPAEM
>ID13150-NO
SYSMEHFRWGKPVGRKRRPVKVYTNGVEEESSEAFPSEM
>ID13151-NO
TAAGDARANAVAKAGLHDLNIETDTERNH

>ID13153-NO
TAALRLGIKLTQHYFGLLTAFGSNFGTIG
>ID13154-NO
TAFHRDDHETDMELK
>ID13155-NO
TAGIKLTVPIEKFPVTTQTFWG
>ID13156-NO
TARLQLEARLQHLVAEILEREQSLA
>ID13157-NO
TARLQLEARLQHLVAEILEREQSLALHALGYQLAFV
>ID13158-NO
TAVILMCFSIDSPDSLENI
>ID13159-NO
TCGETCFGGTCNTPGCTCDPWPICTDRGLP
>ID13160-NO
TCGFADLMGYIPLVGAPL
>ID13161-NO
TCHIRRKPKFRKFKLYHEGKFWCP
>ID13162-NO
TCILDRRPASCGTCVRDCWP
>ID13163-NO
TCRYLFGGCKTTADCCKHLACRSDGKYCAWDGTF
>ID13164-ABP_pos
TCVGGTCNTPGCTCSWPVCTRNGLPVCGE
>ID13166-NO
TDAILMCFSIDSPDSLENI
>ID13167-NO
TDAVDCSQNPLAELKCSVKSF
>ID13168-NO
TDIQTKELQKQITKIQNFRV
>ID13169-NO
TDVALMCFSIDSPDSLENI
>ID13170-NO
TDVIAMCFSIDSPDSLENI
>ID13171-NO
TDVILACFSIDSPDSLENI
>ID13172-NO
TDVILMAFSIDSPDSLENI
>ID13176-NO
TDVILMCFSI
>ID13180-NO
TDVILMCFSIDSP
>ID13184-NO
TDVILMCFSIDSPDSL
>ID13190-NO
TEYWQATWIPEWE
>ID13192-NO
TFFRLFNRGGGWGSFFKKAAHVGKL
>ID13195-NO
TFRAFLSSRLQDLYSIVRRADRAAV
>ID13196-NO

TFRRLKWK
>ID13197-NO
TFWWHPNYYVDW
>ID13198-NO
TGALKINNLRLVTLEHQVLV
>ID13199-NO
TGAWIEFGCHRNKSNLHTEA
>ID13200-NO
TGDFVYMSPFYGYRE
>ID13201-NO
TGPEFPGRPTRP
>ID13203-NO
TGPQTTCQAAMCEAGCKGLGKSMESCQGDTCKCKA
>ID13205-NO
TGTFGFFPGVPPINNCMPLG
>ID13206-NO
TGVAWRIT
>ID13207-NO
TGVGRGYRI
>ID13208-NO
THLEGKVILVAVHVASGYIE
>ID13210-NO
TIFSVFKNWGKVGGGFGVCKFNKQC
>ID13212-NO
TIRLESEVTAIKNALKKTNEAVSTLGNGVRVLATAVRELKDFV
>ID13214-NO
TISYDL
>ID13215-NO
TISYEL
>ID13216-NO
TISYKY
>ID13217-NO
TITYDY
>ID13218-NO
TKALTEVIPLTEEAELELAE
>ID13221-ABP_both
TKCFQWQWNMRKVRG
>ID13222-NO
TKDLQQKFYEIIMDIEQNNVQGKKGIQQLQKWEDW
>ID13223-NO
TKFPSVYAWERKKISNCVAD
>ID13224-NO
TKGPGRVIYATGQ
>ID13225-NO
TKIRDSLHLVKCPTPAIEPP
>ID13226-NO
TKKIELKRFVDAFVKKSYENYILERELKKLIKAINEELPTK
>ID13227-NO
TKKNSFKRKSIMLPKSLLSVVKSATLETKPESKYS
>ID13228-ABP_both
TKKTKLTEEEKNRL

>ID13231-NO
TKRNTNRRPQDVKFPGGGQIVGGVYL
>ID13232-NO
TLEKTLEATLEATLEA
>ID13236-ABP_both
TLIWEFYHQILDEYNKENKG
>ID13237-NO
TLKNRYYEPRDSYFQQYMLKGEYQYWFDLD
>ID13238-NO
TLKPIFKLPLGINITNFR
>ID13240-NO
TLLVYSGW
>ID13242-NO
TLPGP
>ID13244-NO
TLPRPGP
>ID13247-NO
TLREHLRDIKAENTD
>ID13248-NO
TLTVQARQLLSGIVQQQNNLLRAIEAQQHLLQLTVWGIKQLQARIL
>ID13249-NO
TLVDCIRNCIHKILGYTVIA
>ID13250-NO
TLWPWAWRHNWQ
>ID13251-NO
TMKVDDLIVHFNMTKAVEMV
>ID13253-ABP_both
TMSLRFWRWKVR
>ID13254-NO
TMTPPPTSVRGT
>ID13255-NO
TNDCPNSSVVYEAADAIL
>ID13256-NO
TNEIVEEQYTPQSLATLESVFQELGKLTGPNNQ
>ID13257-NO
TNEIVEEQYTPQSLATLESVFQELGKLTGPSNQ
>ID13258-NO
TNRRPQDVKFPGGGQIVG
>ID13259-NO
TNWYGSGW
>ID13261-NO
TPDINPAWYAGRGIRPVGRF
>ID13262-NO
TPDINPAWYASRGIRPVGRF
>ID13264-NO
TPDKKHQKEPPFLWMGYELH
>ID13265-ABP_both
TPFKISIH
>ID13267-NO
TPGSPEFDHYNCVSSGGQCLYSACPIFTKIQGTCYRGKAKCCK
>ID13268-NO

TPHQKTCHPLKEAHANAVCKEYCGSVGYLLGECGKEGICVCEKRQLNE
>ID13269-NO
TPKFKLPIQKETWETWWTEY
>ID13270-NO
TPLPTIRGDTGT
>ID13271-NO
TPNDITLNNSVALDPIDISIELNKAKSDLEESKEW
>ID13274-NO
TQPTDEEMLFIYSHFKQATVGDVNTDRPGLLDLK
>ID13275-NO
TQTVYEWCDDATQLLAAYILL
>ID13276-NO
TQTVYEWCGDATQLLAAYILL
>ID13279-NO
TRDAIEPCTVGHRRY
>ID13280-NO
TRHRHVPRFLPLRHV
>ID13281-NO
TRNLLTTPKFTVAWD
>ID13283-NO
TRQARRNRRRWRERQR
>ID13284-NO
TRQARRNRRRWRERQRAAAAC
>ID13285-NO
TRQCRRGRIWKRWNETITGP
>ID13286-ABP_both
TRRKFWKKVLNGALKIAPFLLG
>ID13287-NO
TRSAWLDSGVTGSGLEGDHLSDTSTTSLELDSR
>ID13288-NO
TRSAWLTSYVAGTGLEEDYLSDISATSLELNSR
>ID13289-NO
TRSAWPGTTGSGLLEDPQPHTSPTSTSLEPSSR
>ID13290-NO
TRSAWPSTAASGLLEDPLPHTSRTSLEPSLR
>ID13291-ABP_both
TRSRWRRFIRGAGRFARRYGWRIALGLVG
>ID13292-ABP_neg
TRSSRAGLQWPVGRVHRLLRK
>ID13293-NO
TRSSRAGLQWPVGRVHRLLRKGGC
>ID13294-NO
TRTSRGWHTTDLKYN
>ID13296-ABP_both
TRWLWLLRGGLKAAGWGIRAHLNRNQ
>ID13298-NO
TSDVAAATNADLRTALARADHQKTLFWL
>ID13299-NO
TSLKNQKEFELGIKVSRKLNKKAVVRNKIKRRIRHSNAIIILQYEL
>ID13300-NO
TSPDVDLGDISGINASVVNIQKEIDRLNEVAKNLNESLIDLQELGKYE

>ID13301-NO
TSQNIRS
>ID13303-NO
TSSMRGVYYPDEIFRSDTLYL
>ID13305-NO
TSVITIELSNIKKIKCN
>ID13306-ABP_both
TSVPYPRPFPRPPIGPRPLPFPGGGRPFQS
>ID13307-NO
TSYHRSA
>ID13309-NO
TTKARATAPTTRNLL
>ID13310-NO
TTMPLW
>ID13311-NO
TTNLTEYPLSRVDLG
>ID13312-NO
TTPFTIRGPLGNQGRGNPVR
>ID13313-NO
TTPKFTVAWDWVPKR
>ID13314-NO
TTSTRR
>ID13315-NO
TTTPAKRKKTKK
>ID13316-NO
TTVKVHASDERLGPMPCRPKEIVSSAGPVM
>ID13319-NO
TTYSRFP
>ID13320-NO
TVAWDWVPKRPSVCT
>ID13321-NO
TVCNLRRCQLSCRSLGLLGKCIGVKCECVKH
>ID13323-NO
TVSSGADTEDVVC
>ID13324-NO
TVSTFIDLNITMLED
>ID13325-NO
TVSYD
>ID13327-NO
TVTYDC
>ID13328-NO
TVVKKRVKK
>ID13330-NO
TWFNPFGYYSWA
>ID13331-NO
TWFWPYPYPHLP
>ID13332-NO
TWLATRGLLRSPGRYVYFSPSASTWPVGIWTTGELVLGCDAAL
>ID13334-NO
TWWTGTYPWYPR
>ID13336-NO

VAADNVIVQNSMRIS
>ID13337-NO
VAAKWKDDVIKLCGRELVRAQIAICGMSTWS
>ID13338-NO
VAGLRQSLEQYQVVKQPDYL
>ID13339-ABP_pos
VAGTWY
>ID13340-ABP_neg
VAHGIVSYGKSSGVPPEVFTRVSSFL
>ID13341-ABP_both
VAIYGRDDRSDVCRQVQHNWLVCDTY
>ID13342-NO
VAKALAKALLKALKAL
>ID13346-NO
VAKKLAKLAKKLAKLAL
>ID13352-NO
VALALKALKKL
>ID13354-NO
VALDPIDISIELNKAKSDLEESKEWIRR
>ID13366-NO
VATRDGKLPTTQLRRHID
>ID13370-NO
VCCGYKLCHPC
>ID13377-ABP_both
VCRTGRSRWRDVCRNFMRRYQSR
>ID13379-NO
VCYFMVFLQYGRKKRRQRR
>ID13380-NO
VDARSVYPYDEFVLA
>ID13381-NO
VDFFA
>ID13382-NO
VDGIPVAWDADARAPA
>ID13383-NO
VDGIPVEWDADARAPA
>ID13387-NO
VDPDVRAYCKHQCLSTRGDQARKICESVCLRQD
>ID13389-NO
VDWKKIGQHILSVL
>ID13391-NO
VEELMKLLEELLKKLEELFKKLEEWFKKWFELSKKFT
>ID13392-NO
VEENMKKLEERLKKREELFKKQEEWFKKWFERSKKFT
>ID13393-NO
VEGQLGENNELRLTRDAIE
>ID13396-NO
VESMNKELPKIIGQVRDQAEHLKTAY
>ID13397-NO
VETTVTTAQETKRGRIQTKK
>ID13398-NO
VEWNEMTWMEWEREIENYTKLIYKILEESQEQ

>ID13400-NO
VFASLPGIIFTRSQKEGL
>ID13402-NO
VFHRIRRIK
>ID13403-ABP_both
VFLGNIVSMGKKI
>ID13405-ABP_neg
VFWRRIRVWVIR
>ID13410-NO
VGCVLGTCQVQNLSHRLWQLVRPAGRRDSAPVDPSSPHSY
>ID13411-NO
VGCVLGTCQVQNLSHRLWQLVRPSGRRDSAPVDPSSPHSY
>ID13415-NO
VGERCCKNGKRGCGRWCRDHSRCC
>ID13416-NO
VGFFT
>ID13417-ABP_both
VGHNADLQIKLSIRRLLAAGVLKQTKGVGA
>ID13419-NO
VGLLLYCKARSTPVTLS
>ID13420-NO
VGNWAKVLVVLLLFAGVD
>ID13421-ABP_both
VGPGAINAGTYLVSRELFER
>ID13423-NO
VGQPQYYQANGGFLI
>ID13425-NO
VGVCCGYKLCHPC
>ID13427-NO
VIKNLQSLDPSHRISDRDYMGWMDF
>ID13430-NO
VILSLDVPIGLLRILLEQARYKAARNQAATNAQILAHV
>ID13431-ABP_both
VIPFVASVAAEMMQHIFCAASRKC
>ID13433-ABP_both
VIRRRRILAAVV
>ID13434-NO
VIVQNSMRISSRPGA
>ID13435-NO
VKFPGGGQIVGGVYLLPR
>ID13438-NO
VKKRKIVIY
>ID13439-NO
VKKRRHGDEDMFYMH
>ID13440-NO
VKLFPFFNQY
>ID13441-NO
VKLFPWFNQY
>ID13442-NO
VKLFPWWNQY
>ID13444-NO

VKQCRWCGFDFDGPDGLPHYPIGKCILANE
>ID13447-NO
VKREKDFQRVGISVGKKIGNAVTRNAVKRKIRHLKDFVVLQQNL
>ID13450-NO
VKVIKKFVR
>ID13452-NO
VLDLIYSLHKQINRGLKKIVL
>ID13453-ABP_both
VLHTGYRKFLHRSKRFFHLR
>ID13455-NO
VLPFPAIPLSRRRACVAAPRPRSRQRAS
>ID13456-ABP_pos
VLPLISMALGKL
>ID13457-ABP_neg
VLSAADKNNVKGIFTKIAGHAEEYGAETLERMFTTYPPTKTY
>ID13458-NO
VLSHNNESSYSDTSSCTSQ
>ID13462-NO
VMQSLYVKPPLILVTKLAQQN
>ID13463-NO
VMRKLRRRW
>ID13464-NO
VNKKIEEIDKKIEELNKKLEELEKKLEEVNKK
>ID13470-ABP_both
VNWKKVLAKIIKVAK
>ID13471-ABP_both
VNWKKVLKKIIKVAK
>ID13483-NO
VPAHKGIGGNEQVDKLVSAG
>ID13485-ABP_both
VPIIYCNRRTGKCQRM
>ID13486-NO
VPLPAGGGTVLTKMYPRGNHWAVGHLM
>ID13490-NO
VPPGFTPFRQ
>ID13491-NO
VPPGFTPFRQS
>ID13492-NO
VPPGFTPFRVD
>ID13493-NO
VPPKGVSM
>ID13496-NO
VPTGYKP
>ID13497-NO
VPTHLATDVELKEIQGMMDASEGTNYTCCK
>ID13499-NO
VPVGSASCTIAALGSSDR
>ID13500-ABP_neg
VQFRIRVRIVIRK
>ID13506-NO
VQQQNNLLRAIEAQQHLLQLTVWGIKQL

>ID13507-ABP_neg
VQVTSTAV
>ID13508-NO
VRDNMAKLRERLKQRQQLFDSQQGWFEGWFNRSPWFT
>ID13509-ABP_both
VRIHISGSSLGWLIQLFRKRIESLLQKS
>ID13510-NO
VRIRKRRWR
>ID13511-NO
VRLASHLRKLRKRLLRDADDLIRLQAEAFQARLKSWFEPLV
>ID13514-NO
VRLGSISVIGIVRGKK
>ID13515-ABP_neg
VRLIRAVRAWRV
>ID13516-ABP_both
VRLIVRIWRR
>ID13517-ABP_both
VRLIWAVRIWRR
>ID13518-NO
VRLRIRVWVIRA
>ID13520-NO
VRQLCKLLRGTKALTEVIPL
>ID13521-NO
VRRCSELMGRRNPVCPGYAW
>ID13527-NO
VRVLEDGVNYATGNLPGC
>ID13528-ABP_neg
VRWARVARILRV
>ID13530-NO
VSAFK
>ID13531-NO
VSCDFEEANEDAVCQEHCLPKGYTYGICVSHTCSCI
>ID13532-NO
VSCEDCPEHCSTQKAQAKCDNDKCVCEPI
>ID13533-NO
VSCTGSKDCYAPCRKQTGCPNAKCINKSCKCYGC
>ID13534-NO
VSFAIKWEYVL
>ID13535-NO
VSFAIKWEYVLL
>ID13539-NO
VSPRCEYQAGHNKVG
>ID13540-NO
VSQIIEQLIKKEKVY
>ID13542-NO
VSRRYLASLHKKALPTSVTFELLFDGTNPS
>ID13543-NO
VSTALPQWRIYSYAGDNI
>ID13544-NO
VSVIVPDYQCYLDRVDTWLQ
>ID13545-NO

VSVTCVWGSVSWFASTGGRD
>ID13547-ABP_both
VTCDILSVEAKGVKL
>ID13548-NO
VTCDLLGPTGWGDALCAAHCISKGYRGGYCNAQKVCVCR
>ID13549-ABP_both
VTCDLLSAEAKGVKVNHAACAAHCLLKRKRGGYCNKRRICVCRN
>ID13557-ABP_both
VTCDVLSFEAKGIAVNH
>ID13558-NO
VTDSQYALGIIQAQPDQSES
>ID13560-NO
VTLSLDVPTNIMNILFNIAKAKNLRAKAAANAHLMAQI
>ID13561-ABP_both
VTPPWARIYYGCAKA
>ID13562-NO
VTTAQETKRGRIQTKKEVSI
>ID13563-NO
VTVLDVGDAYFSVPLDEDFR
>ID13565-NO
VVDRGWGNGAGLFGKGSID
>ID13566-NO
VVKCPYRLGSPDSRCN
>ID13567-NO
VVKCSNCLGSPDSRCN
>ID13568-NO
VVKCSSRLGSPDSRCN
>ID13569-NO
VVKCSYGLGSPDSRCN
>ID13571-ABP_both
VVKCSYRQGSPDSR
>ID13572-ABP_both
VVLKIVRRF
>ID13573-NO
VVNDL
>ID13574-NO
VVNIQKEIDRLNEVAKNLNESLIDLQELGK
>ID13575-NO
VVNIQKEIDRLNEVAKNLNESLIDLQELGKYEQYIKWPW
>ID13576-ABP_both
VVTCGSLCKAHCTFRKCGYFMSVLYGHRCYCRCLLC
>ID13577-NO
VVTLTDTTNQKTELQAIYLA
>ID13578-NO
VVTTRLFMSLVASVRNAFQSGYISFDEIIKTE
>ID13581-NO
VVYPW
>ID13582-NO
VVYPWTQRF
>ID13583-NO
VWGIKQLQARILAVERYLKDQQLLGIWG

>ID13584-NO
VWHWWRWRW
>ID13585-NO
VWIRRIKRR
>ID13586-NO
VWLKVYWFK
>ID13587-NO
VWRKWYRVK
>ID13588-NO
VWRRKKYWR
>ID13589-NO
VWRRYWWYR
>ID13590-NO
VWVRWKIWY
>ID13591-NO
VWWKWWRWW
>ID13595-NO
VYDPLQPELDSFKEELDKYFKNHTSPDVDLGDISGINASVV
>ID13597-NO
VYPYDEFVLATGDFV
>ID13598-ABP_both
VYQHQKAMKPWIQPKTKVIPYVRYL
>ID13599-NO
VYRRRRVKG
>ID13600-NO
VYSFLYVLVIVRKLLSMKKRIERL
>ID13603-NO
WADWVRWI
>ID13605-NO
WALAL
>ID13607-NO
WASRELERFAVNPGLLETSEGCRQIL
>ID13608-NO
WCKQSGEMCNLLDQNCCDGYCIVLVCT
>ID13609-NO
WCNWHNIDPWIQLMNRTQADLAEGPPVKEC
>ID13611-NO
WDLAWMFRLPVG
>ID13612-NO
WDWICEVLSDFK
>ID13614-NO
WEAWVRWI
>ID13615-NO
WEDAVRWI
>ID13616-NO
WEDWARWI
>ID13617-NO
WEDWVAWI
>ID13618-NO
WEDWVGWI
>ID13619-NO

WEDWVR
>ID13620-NO
WEDWVRAI
>ID13621-NO
WEDWVRWA
>ID13622-NO
WEDWVRWI
>ID13624-NO
WEEWDKKIEEYTKKIEELIKKSEEQQKN
>ID13625-NO
WEEWDRKIEEYTKKIKKLIEESQEQQEKNEKELK
>ID13627-NO
WEIHHINKFAQAYSSYSRVIGGTVFVA
>ID13629-NO
WFFKSKVYW
>ID13630-NO
WFIRVWRYR
>ID13631-ABP_both
WFKMRWWGR
>ID13632-NO
WFQRIPLGWFHCTYQKGKQHCRLRIRQKVEE
>ID13633-NO
WFTRWKWRW
>ID13634-NO
WFWRRKMIR
>ID13636-ABP_both
WGRRWRIRIPRLPRPWPPRPKWPRSATINTDQ
>ID13637-NO
WHSQRLSPVPPA
>ID13638-NO
WHSRGSTWLYRETANLNAMLTITTARSKYPY
>ID13639-NO
WHWWIRWWR
>ID13643-NO
WIRLWVKWR
>ID13644-ABP_neg
WIVVIWRRKRRRC
>ID13645-NO
WIVWIRKRI
>ID13646-ABP_both
WKIVFWWRR
>ID13647-NO
WKIWKIRWF
>ID13648-NO
WKKIRRFVSQVIM
>ID13649-ABP_both
WKKWWKRRW
>ID13651-NO
WKLFKKILKKL
>ID13658-NO
WKRRWSRSR

>ID13659-NO
WKRWLIIGR
>ID13660-ABP_both
WKRWWKKWR
>ID13661-ABP_both
WKWLKKWIK
>ID13662-ABP_both
WKWRVRVTI
>ID13663-NO
WKWVTRMYI
>ID13664-NO
WKYKYRIRL
>ID13668-NO
WLGCARVKEACGPWEWPCCSGLKCDGSECHPQ
>ID13679-ABP_both
WLNALLHHGLNC
>ID13681-ABP_both
WLNALLHHGLNCAK
>ID13683-ABP_both
WLNALLHHGLNCAKGV
>ID13684-NO
WLNALLHHGLNCAKGVLAWLNALLHHGLNCAKGVLA
>ID13685-NO
WLRKVWNWR
>ID13686-NO
WLVFFVIAYFAR
>ID13687-NO
WLVFFVIFYFFR
>ID13688-NO
WLVFFVIFYFFRRRKK
>ID13689-NO
WLVFFVRRKK
>ID13690-ABP_both
WLWKAIWKLLK
>ID13693-NO
WMEWDREIEALAKAAEALAKAAEALAKAAWASLWNWF
>ID13694-NO
WMEWDREIEEAAKKLEEAAKKLEEAAKKLEEAAKKL
>ID13695-NO
WMEWDREIEEAAKKLEEAAKKLEEAAKKLWASLWNWF
>ID13696-NO
WMEWDREIEELAKKAEELAKKAEELAKKAEELAKKA
>ID13697-NO
WMEWDREIEELAKKAEELAKKAEELAKKAWASLWNWF
>ID13698-NO
WMEWDREINNYTSLIHSLIEEAQNQQEKNEQELL
>ID13699-NO
WMEWDREINNYTSLIHSLIEELQNQQEKNEQELL
>ID13701-NO
WMEWDREINNYTSLIHSLIEESQNQQEK
>ID13703-NO

WMEWDREINNYTSLIHSLIEESQNQQEKNEQELLELDKWASLWNWF
>ID13707-NO
WMEWEREIDNYTSEIYTLIEESQNQQEKNEQELL
>ID13709-NO
WNHGNITLGEWYNQTKDLQQKFYEIIMDIEQNNVQ
>ID13710-NO
WNPFKELERAGQRIRDSIISAAP
>ID13713-NO
WPTNPTTVPVPS
>ID13714-ABP_both
WQQPSCSSICDYSCGKSACISYSGRCGCCASCRRGPIY
>ID13715-ABP_neg
WQRRMKLGAPSIT
>ID13717-NO
WQVMIVWQVDRMRIR
>ID13718-NO
WQVVMRYRR
>ID13719-NO
WRINIFKRI
>ID13720-NO
WRIRTTAEFRRLGVVASKRNVRKAVWRNRVRRVVKELDIVVLYECI
>ID13722-ABP_both
WRRWWRRWWRWWRRWWRR
>ID13723-NO
WRVKGKRSK
>ID13724-ABP_neg
WRVWVRVKR
>ID13725-NO
WRWGLRWWQ
>ID13727-ABP_both
WRWWKIWKR
>ID13728-NO
WRWYCRCK
>ID13729-NO
WRWYVTRRK
>ID13731-NO
WSFFSNI
>ID13732-NO
WSKMDQLA
>ID13733-NO
WSKMDQLAKELTAE
>ID13734-NO
WSRMDQLAKELTAE
>ID13735-NO
WSRVPGHSDTGWKVWHRW
>ID13737-NO
WTDMFTAWWSTP
>ID13738-NO
WVAVTPTVATRDGKLPTT
>ID13739-NO
WVEYMYSWIPTA

>ID13741-NO
WVPKRPSVCTMTKWQ
>ID13743-NO
WVRRYQMRR
>ID13745-NO
WVRWI
>ID13746-NO
WVTTSNQW
>ID13748-NO
WWFSIRLWR
>ID13749-NO
WWFWVIRKY
>ID13750-NO
WWKRYIVKK
>ID13751-NO
WWKWIRKIV
>ID13752-NO
WWLRGRRWL
>ID13753-NO
WWQWIVWRK
>ID13754-NO
WWRRKWWRR
>ID13755-NO
WWRRLRWLV
>ID13756-NO
WWWRSFRKR
>ID13759-NO
WYCRCK
>ID13761-NO
WYKHVASPRYHTVGRAAGLLMGL
>ID13762-NO
WYKHVASPRYHTVGRAAGLLMGLRRSPYLW
>ID13763-NO
WYKHVASPRYHTVGRASGLLMGL
>ID13764-NO
WYKHVASPRYHTVGRASGLLMGLRRSPYLW
>ID13766-NO
WYKPAAGHSSYSVGRAAGLLSGLRRSPYA
>ID13768-NO
WYKPAAGSHHYSVGRAAGLLSSFHRFPST
>ID13769-NO
WYKPTAGQGYYSVGRAAGLLSGFHRSPYA
>ID13770-NO
WYLTLTLGYGRR
>ID13772-NO
WYQLEKEPIVGAETFYVDGA
>ID13775-NO
WYWRRRRLK
>ID13845-NO
YADAIFTNSYRKVLGQLSARKLLQDIMNRQQGERNQEQGAKVRL
>ID13847-NO

YADHTGLVRDNMAKLRERLKQRQQLFDSQQGWFEGWFNRSPWFTT
>ID13848-NO
YAEGTFISDYSIAMDKIRQQDFVNWLLAQKGKKSDWIHNITQ
>ID13851-NO
YAFDVVG
>ID13852-NO
YAFEVVG
>ID13853-NO
YAFQVVG
>ID13854-NO
YAGAVANDL
>ID13855-NO
YAGAVVNDL
>ID13856-NO
YAGAVVNDLL
>ID13857-NO
YAGFL
>ID13858-ABP_both
YAGFLR
>ID13860-ABP_pos
YAPWTNA
>ID13861-ABP_pos
YAPWTNF
>ID13862-NO
YARAAARQARA
>ID13863-NO
YARAARRAARR
>ID13864-NO
YARDLTTKARATAPT
>ID13865-NO
YASPKCFRYPNGVLACT
>ID13866-NO
YAVVNDL
>ID13867-NO
YCKKCCYHCQ
>ID13868-ABP_both
YCNGKRVCVC
>ID13870-ABP_both
YCNRRTGKCQRM
>ID13871-NO
YCQKWMWTCDEERKCCEGLVCRLWCKRIINM
>ID13872-NO
YCQKWMWTCDSARKCCEGLVCRLWCKKII
>ID13875-NO
YDHIQDHVNTMFSRLATSWCLLQNKERALWAEAA
>ID13877-ABP_both
YDPEAASAPGSGNPCHEASAAQKENAGEDP
>ID13878-NO
YDPLVFPSDEFDASISQVNEKINQSLAFIRKSDELLHNV
>ID13881-NO
YEIIMDIEQNNVQGKTGIQQ

>ID13885-NO
YEPLVRRRSELMGRRNPV
>ID13888-NO
YFRWWKRWI
>ID13891-NO
YGGFLRKYPK
>ID13894-NO
YGGFLRRIRPKLKWDNQKRYGGFLRRQFKVVT
>ID13895-NO
YGGFLRRQFKVVT
>ID13896-NO
YGGFLRRQFKVVTRSQEDPNAYSGELFDA
>ID13898-NO
YGGFLRRQFKVVTRSQENPNTYSEDLDV
>ID13899-NO
YGGFMKPYTQQSHKPLITLLKHVTLKNEQ
>ID13903-NO
YGGFMTSEKSQTPLVTLFKNAIIKNAHKKGQ
>ID13904-NO
YGGFMTSEKSQTPLVTLFKNAIIKNAYKKGE
>ID13905-NO
YGGFMTSEKSQTPLVTLFKNAIIKNVHKKGQ
>ID13908-NO
YGRKKRRQRRRGSGIEPHDWTKNITDKIDQIIHDFVDK
>ID13909-NO
YGRKKRRQRRRREADFFWSLCTADMS
>ID13910-NO
YGWMDF
>ID13911-NO
YGYREGSHTEHTTYA
>ID13912-NO
YIQFHLNQQPRPKVKKIKIFL
>ID13913-NO
YIQQARKAPSGRMSIVKNLQNLDPSHRISDRDYMGWMDF
>ID13914-NO
YIQQARKAPSGRMSVIKNLQSLDPSHRISDRDYMGWMDF
>ID13915-NO
YIQQVRKAPSGRMSVLKNLQGLDPSHRISDRDYMGWMDF
>ID13917-ABP_both
YKARRWAWRMK
>ID13919-ABP_both
YKAWRWAWRWK
>ID13920-NO
YKFACPECPKRFMRSDHLSKHITLHELLGEERR
>ID13921-NO
YKFACPECPKRFMRSDHLTLHILLHENKK
>ID13923-NO
YKGGNKKGLSKGCFGLKLDRIGSMSGLGC
>ID13924-NO
YKGYQPIDVVRDLPSGFNTL
>ID13926-ABP_both

YKLLKLLLPKLKGLLFKL
>ID13927-NO
YKPRRKRAAIHVMLALATVLSI
>ID13928-NO
YKQCHKKGGHCFPKEKICIPPSSDLGKMDCRWKWKCCKKGSG
>ID13930-NO
YKRCHIKGGHCFPKEKLICIPPSSDIGKMDCPWKRKCCKKRS
>ID13931-NO
YKRCHKKEGHCFPKTVICLPPSSDFGKMDCRWKWKCCKKGSVN
>ID13932-NO
YKRCHKKEGHCFPKTVICLPPSSDFGKMDCRWKWKCCKKGSVNNA
>ID13934-NO
YKRCHKKGGHCFPKTVICLPPSSDFGKMDCRWKWKCCKKGSVNNA
>ID13935-NO
YKRGGGGWGGGGGWKGGGGGGGGWKGGGGGGKGG
>ID13937-NO
YKVNEYQGPVAPSGGFFLFRPRN
>ID13938-NO
YKYRYL
>ID13940-NO
YLDRVDTWLQGKINISLCLT
>ID13944-NO
YLQELLGE
>ID13945-NO
YLRGVNRSLHGIWPGKICKGVPTHLATDVE
>ID13946-NO
YLTTLK
>ID13947-NO
YMGWMDF
>ID13949-NO
YMSPFYGYREGSHTE
>ID13952-NO
YNTPVFAIKKKDSTKWRKLV
>ID13953-NO
YNVLPQGWKGSPAIFQSSMT
>ID13955-NO
YNWNSFGLRF
>ID13956-NO
YNWNSFGLRY
>ID13957-NO
YNYKYRYLRHGKLRPFERDI
>ID13958-NO
YPAKPQAPGEHASPDELNRYYTSLRHYLNLVTRQRF
>ID13959-NO
YPAPIKVLLPNS
>ID13961-NO
YPFPG
>ID13964-NO
YPLPHPMWSMLP
>ID13967-NO
YPYYPGEARGAP

>ID13968-NO
YQDVNCTDVSTAIHADQLTP
>ID13969-NO
YQFHLVLHEALRAQALSRQLILGRELAQELVAELAT
>ID13970-NO
YQSTCSAVSKGYLSALR
>ID13971-NO
YQVVKQPDYLLVPEEVMEYK
>ID13972-ABP_neg
YQWQRMRKLGAPSIT
>ID13973-ABP_neg
YQWQRRMKLGAPSIT
>ID13974-ABP_both
YQWQRRMRKL
>ID13979-NO
YRFVRRWIV
>ID13980-NO
YRIKKNADFQRLGISVSKKLGNAVLRNKIKRAIREILDIIVIQNSL
>ID13981-NO
YRLLKTDDFSRIGLVVGKKTAKRANERNYMKRVIRDLDFVVARAEL
>ID13983-ABP_both
YRMWRWAWRWR
>ID13984-ABP_both
YRMWRWRWRWR
>ID13987-NO
YRVKSDKDFQRVGLSVGKRLGNAVVRNAIKRKLRHLGDFVVMKKNL
>ID13988-NO
YRVYWWWWR
>ID13989-NO
YRWRGPTAAFLSLV
>ID13992-ABP_pos
YSPWTNF
>ID13993-ABP_both
YSWPRMPRIPRLPRYPRYPRYPRWPRHPTIYA
>ID13994-ABP_both
YSWPRMPRIPRLPRYPRYPRYPRWPRWPRQPTIYA
>ID13997-NO
YSYEL
>ID14002-NO
YVREHGVPIHADKYFEQVLK
>ID14004-NO
YVTQQLIRAAEIRASANLAATKMSECVLGQSKRVDFCGKG
>ID14005-NO
YWRRKWRRK
>ID14007-NO
YWWLWRKKR
>ID14009-NO
YYGNGLYCNKEKCWVDWNQAKGEIGKIIVNGWV
>ID14010-ABP_both
YYHFWHRGVTKRSLSPHRPRHSRLQR
>ID14011-NO

YYQANGGFLIAYQPL
>ID14016-ABP_both
AAAAAAAAAK
>ID14017-ABP_both
AAAAAAAIKMLMDLVNERIMALNKKAKK
>ID14020-ABP_both
AAAAGSCVWGAVNYTSDCAAECLLRGYKGGHCGSFANVNCWCET
>ID14021-ABP_both
AAAAGSCVWGAVNYTSDCAAECLLRGYKGGHCGSFANVNCWCRT
>ID14024-ABP_both
AAAAGSCVWGAVNYTSDCNGECLLRGYKGGHCGSFANVNCWCET
>ID14027-NO
AAAAPGAAGGAQLPLGNRERKAGCKNFFWKTFSSC
>ID14028-ABP_both
AAAARRRR
>ID14029-NO
AAADPNFLRF
>ID14030-NO
AAAEECEEYCEEEEKTCCGEEDGEPVCAEFCL
>ID14031-NO
AAAGDNFMRF
>ID14034-NO
AAAISCVGSKECLPKCKAQGCKSGKCMNKKCKCYC
>ID14035-NO
AAAISCVGSPECPPKCRAQGCKNGKCMNRKCKCYYC
>ID14036-NO
AAAMWFGPRL
>ID14038-NO
AAAQHLCGSHLVDALYLVCGEKGFFYTP
>ID14040-NO
AAASDNFMRF
>ID14041-NO
AAATNPARYCCLSGCTQQDLLTLCPY
>ID14045-NO
AACKCDDEGPDIRTAPLTGTVDLGSCNAGWEKCASYYTIIADCCRKKK
>ID14046-NO
AACLGMFESCDPNNDKCCPNRECNRKHKWCKYKLW
>ID14049-NO
AADDKPSDWIALAIKQCCANPPCKHVNCR
>ID14050-NO
AADGAPFIRF
>ID14051-NO
AADGAPLIRF
>ID14053-NO
AADGKPSDWISLVTPECCSNPPCNLQNCR
>ID14055-NO
AADHDVGSELPPEGVLGALLRVKRLETPAPQVPARRLLPP
>ID14056-NO
AADISQWAGPL
>ID14057-NO
AADYKRSDWFAWVIHSCCSNPPCAHVNCRRRR

>ID14058-NO
AADYKRSDWIARVIDSCCSNPPCAHVHCHRRR
>ID14059-NO
AAEFPDFYDSEEQMGPHQEAEDEKDRADQRVLTEEEKKELENLAAMDLEL
>ID14061-NO
AAERSPSLRLRF
>ID14067-NO
AAFRGCWTKNYSPKPCLGKR
>ID14070-NO
AAGLFQFPRV
>ID14071-NO
AAGLQNYDFG
>ID14072-NO
AAGQDFMRF
>ID14073-NO
AAGQDNFMRF
>ID14074-ABP_neg
AAGRLWVLWRR
>ID14075-ABP_neg
AAGRWWRWWRRWWRR
>ID14077-ABP_neg
AAGWGLRRLLKYGKRS
>ID14078-ABP_both
AAHCIALRKGYK
>ID14079-ABP_both
AAHCIQLGKR
>ID14080-ABP_both
AAHCIVLHHN
>ID14081-ABP_both
AAHCLAIGRK
>ID14082-ABP_both
AAHCLAKRKK
>ID14083-ABP_both
AAHCLAMRRK
>ID14084-ABP_both
AAHCLIGRK
>ID14085-ABP_both
AAHCLLKRKR
>ID14088-ABP_both
AAHHIARPIVHVGKTIHRLVTG
>ID14089-ABP_both
AAKCLVQRRR
>ID14092-NO
AAKFKAPALMELTVRDECCSDPRCAVKHQDLCG
>ID14093-NO
AAKFKAPALMELTVREGCCSDPRCSGKHQDLCS
>ID14094-NO
AAKFKAPALMERNVWEKCCLDPRCSGKHQNKCG
>ID14095-NO
AAKFKAPALMKRTDSEECCLDSRCAGQHQDLCS
>ID14096-NO

AAKFKAPALMKRTVRDACCSDPRCSGKHQDLCG
>ID14097-NO
AAKFKAPALMKRTVREACCSDPRCSGQHQEKCG
>ID14098-NO
AAKFKAPALMKRTVSQECCLDTRCAGKNLDECG
>ID14099-ABP_both
AAKHAAHRA
>ID14102-NO
AAKVKYSKTPEECCPNPPCFATHSEICG
>ID14103-NO
AAKVKYSKTPEECCPNPPCFATHSEICGGRR
>ID14104-NO
AAKVKYSKTPEECCPNPPCFATHSNICG
>ID14105-NO
AAKVKYSKTPEECCPNPPCFATHSNICGGRR
>ID14106-NO
AAKVKYSKTPEECCPNPPCFATNSDICG
>ID14108-NO
AAKVKYSLTPAECCPNPPCFAQHSDLCGA
>ID14109-NO
AAKVKYSLTPAECCPNPPCFAQHSDLCGARR
>ID14110-NO
AAKVKYSLTPAECCPNPPCFAQHSNLCGA
>ID14119-NO
AAKVKYSNTREECCPNPPCFATNSDICGGRR
>ID14129-NO
AALKGCWTNSIPPKPCSGKR
>ID14130-NO
AALPPGFTPFR
>ID14131-NO
AALSDSYDLRGKQQRFADFNSKFVAVR
>ID14132-NO
AALSEAYDVRGKKERYADFNSKFVAVR
>ID14133-ABP_both
AALSELHCDKLHVDPENFKLL
>ID14135-NO
AAMDRY
>ID14136-NO
AAMRSFNMGF
>ID14138-NO
AANAKLFAVMQSCCSTPPCALRHMDMC
>ID14139-NO
AANAKLFAVMQSCCSTPPCALRHMDMCG
>ID14142-NO
AANAKLFAVRQSCCSTPPCALLYMEMCG
>ID14144-NO
AANAKLFDVGQSCCSAPLCALLYMVICG
>ID14145-NO
AANAKLFDVRQSCCSAPLCALLYRVMCG
>ID14147-NO
AANAKLSERLDPCCRDPPCASTHIDRCG

>ID14148-NO
AANAKLSERLDPCCRDPPCASTHTDICTRRR
>ID14149-NO
AANAKLSERLDPCCREPPCASTHIDRCG
>ID14151-ABP_both
AANCITLGKA
>ID14152-ABP_both
AANCLSLGKA
>ID14153-NO
AANDKAPVQIVLTVQECCADSACSLTNPLIC
>ID14154-NO
AANDKAPVQIVLTVQECCADSACSLTNPLICGRR
>ID14155-NO
AANDKASAWIARTVRQSCCAAPSCFMLC
>ID14157-NO
AANDKASDLMALRDGCCSDPACAVNHPDICGGGR
>ID14160-NO
AANDKASDLMALRGGCCSRPPCILEHPEICGRRR
>ID14162-NO
AANDKASDLMALTVRGCCVYPPCAVNHPDICRGGR
>ID14163-NO
AANDKASNLMALRDECCPNPPCKASNPDLCDWRS
>ID14164-NO
AANDKASVQIALTVQECCADAACSLTNPLIC
>ID14168-NO
AANDKTSAWIAWAGSQSCCATPSCAKLY
>ID14175-NO
AANNKATDLMALRYHECCKHPPCRNTRPDLCGGRR
>ID14176-NO
AANNKATDLMALTVRGCCADPSCSILMPYFCI
>ID14179-NO
AANNKATDLMALTVRGCCDDPSCSIHMPFFCF
>ID14180-NO
AANNKATDLMALTVRGCCGNPSCSIHIPYVCN
>ID14181-NO
AANNKATDLMARTVRGFCSDPSCRFRNPELCDWRR
>ID14182-NO
AANNKATDLMARTVRRFCSDPPCRISNPESCGWEP
>ID14183-NO
AANQHLCGSHLVEALYLVCGERGFFYSPKA
>ID14184-NO
AANQHLCGSHLVEALYLVCGERGFFYSPKT
>ID14190-NO
AAPFLECQGRQGTCHFFAN
>ID14192-NO
AAPRFF
>ID14195-NO
AAQILRVAQGPSAFVAGPH
>ID14198-ABP_both
AARCLSQRRK
>ID14202-NO

AARPPLGCKAAFC
>ID14203-NO
AARPPLRCKAAFC
>ID14204-NO
AARPPTGIRCKAAFC
>ID14206-ABP_pos
AASDISLLDAQSAPLR
>ID14207-NO
AASDKASELMALAVRGCCSHPACAGNNPDICG
>ID14208-NO
AASDKASELMALAVRGCCSHPACAGNNPHICGRRR
>ID14209-NO
AASDKASELMALAVRGCCSHPACAGSNAHICGRRR
>ID14213-NO
AASPNIFGQWM
>ID14214-NO
AASPYSFGL
>ID14216-NO
AATEECEEYCEDEEKTCCGLEDGEPVCATTCL
>ID14217-NO
AATENM
>ID14218-ABP_both
AATGTGKTAAFALPVLERLI
>ID14220-NO
AAVACRICMRNFSTRQARRNHRRRHRR
>ID14222-NO
AAVALLPAVLLALLAKKNNLKDCGLF
>ID14224-NO
AAVALLPAVLLALLAPEILLPNNYNAYESYKYPGMFIALSK
>ID14225-NO
AAVALLPAVLLALLAPRKKRRQRRRPPQ
>ID14227-NO
AAVALLPAVLLALLAPRRRRRR
>ID14228-NO
AAVALLPAVLLALLAPSGASGLDKRDYV
>ID14229-NO
AAVALLPAVLLALLAPVQRKRQKLMP
>ID14230-NO
AAVALLPAVLLALLAVTDQLGEDFFAVDLEAFLQEFGLLPEKE
>ID14233-ABP_both
AAVLNA
>ID14234-ABP_both
AAVLNALGKEEQIGRASNSGRKCARKKK
>ID14235-NO
AAVRLRPVGSLFFLNRPHE
>ID14244-ABP_both
ACAVRCLAQRRK
>ID14245-NO
ACCDPDWCDAGCYDGCC
>ID14246-NO
ACCPYEPSCCI

>ID14251-NO
ACCSNPACRVNNPHVC
>ID14254-NO
ACCSYPPCNVNYPEICGGR
>ID14257-NO
ACDFHSCWATCQAQHGICFRRAYCDGPSCQCVFLNQG
>ID14258-NO
ACDFNSCWATCKAQNGIYFRRAFCDGPTCLCVFLNAG
>ID14259-NO
ACDFQSCWFTCQRQYSIYFIRAYCDGSTCMCVHN
>ID14260-NO
ACDFQSCWVACQRQYNIYFRKAYCEKSRCVCVHN
>ID14261-NO
ACDFQSCWVLCQRQYNIYFRKAYCEKSRCICVYNYGG
>ID14262-NO
ACDFQSCWVLCQRQYNIYFRKAYCEKSRCVCVYNYGG
>ID14264-NO
ACDFQSCWVLCQRQYNIYFRRAYCEHSKCVCVYNYGG
>ID14267-NO
ACDFQSCWVTCQRQHSIYFIRAFCDGSRCMCVYNNGG
>ID14268-NO
ACEFQSCWVTCQRKYNIYFRKAYCEKSKCICVYNYGG
>ID14275-NO
ACGPGCSGSCRQKGDRIKCINGSCHCYP
>ID14276-NO
ACGQFWWKCGEGKPPCCANFACKIGLYLCIWSP
>ID14277-NO
ACGSCRKKCKGPGKCINGRCKCY
>ID14280-NO
ACKGLFVTCTPGKDECCPNHVCSSKHKWCKYKI
>ID14281-NO
ACKGVFDACTPGKNECCPNRVCSDKHKWCKWKL
>ID14282-NO
ACKPKNNLCAITEMAECCSGFCLIYRCS
>ID14283-NO
ACLDIGNSCRED
>ID14284-NO
ACLGFGEKCNPSNDKCCKSSSLVCSQKHKWCKYGW
>ID14288-NO
ACNFQSCWAICKAHYGIYFRRAYCDGPNCQCVHLIQG
>ID14289-NO
ACNFQSCWAICKEHYGIYFRRAYCDGPNCQCVHLIQG
>ID14291-NO
ACNRNACWASCQRQHGIYFRRAFCEGSRCRCVRVNGR
>ID14292-NO
ACNTATCMTHRLAGWLSRSGSMVRSNLLPTKMGFKIFNGPRRNSWF
>ID14293-NO
ACNTATCMTHRLAGWLSRSGSMVRSNLLPTKMGFKIFSGPRKNFWF
>ID14296-NO
ACNTATCVTHRLADFLSRSGGMAKNNFVPTNVGSAF
>ID14297-NO

ACNTATCVTHRLADFLSRSGGVGKNNFVPTNVGSKAF
>ID14299-ABP_both
ACPIFTKIQGTYRGKAKCK
>ID14300-ABP_both
ACPIFTKIQGTYRGKAKRIGRRIC
>ID14301-ABP_both
ACPIFTKIQGTYRGRAKCR
>ID14303-NO
ACQGYMRKCGRDKPPCCKKLECSKTWRWCVWNPWE
>ID14304-NO
ACRKKWEYCIVPIIGFIYCCPGLICGPFVCV
>ID14314-NO
ACSRAGENCYKSGRCCDGLYCKAYVVTCYKP
>ID14315-NO
ACSRGSRCPPQCCMGLRCGRGNPQKCIGAHEDV
>ID14317-NO
ACTLECEGKLPSAKAWGTCKELLQLTKLDGVQDGEKYQDNNDSHYIA
>ID14318-NO
ACVELGEICATGFFLDEECCTGSCHVFCVL
>ID14319-NO
ACVGENKQCADWAGPHCCDGYYCTCRYFPKCICRNNN
>ID14320-NO
ACVGENQQCADWAGPHCCDGYYCTCRYFPKCICRNNN
>ID14321-NO
ACVPVYEECGTPKKRCCEERPCK
>ID14322-NO
ACVPVYKECWYPQKPCCEDRVCQCSFGMTN
>ID14323-NO
ACVTHEDCTLLCYDTIGTCVDGKCKCM
>ID14326-NO
ACYPPCFGSSVCYGGRCFFIGF
>ID14329-ABP_both
ADADDDDDK
>ID14330-NO
ADAFFLGPRY
>ID14332-NO
ADCNGACSPFEVPPCRSRDCRCVPIGLFVGFCIHPTG
>ID14333-NO
ADCRQKPCL
>ID14335-NO
ADCVGDGQRCADWAGPYCCSGYYCSCRSMPYCRCRSDS
>ID14337-NO
ADDGAPLIRF
>ID14338-NO
ADDRNPLEQCFRETDYEEFLEIARNNLKATSNPKHVVIVGAGMAGLSAAY
>ID14339-NO
ADECCSNPACRVNNPHVC
>ID14345-NO
ADGAPRPGAPLA
>ID14347-NO
ADKNFLRF

>ID14348-NO
ADKPDLGEINSFDKAKLKKTETQEKNTLPTKETIEQEKQAK
>ID14349-NO
ADLGFTHSFV
>ID14350-ABP_both
ADMDFTGIAESIIKKIKETNAKPPA
>ID14351-NO
ADNKCENSLRREIACGQCRDKVKTDGYFYECCTSDSTFKKCQDLLH
>ID14353-NO
ADNRRPIWVM
>ID14357-NO
ADSRKPDD
>ID14358-NO
ADSRKPDDRYDMSGNDALGDVKLATYEDNPWETFK
>ID14359-NO
ADSRKPI
>ID14360-NO
ADSRKPIWI
>ID14362-NO
ADVFDRGGPYLQRGVADLVPTATLLDTYSP
>ID14363-NO
ADVGHVFLRF
>ID14364-NO
ADVPGNYPLDKNGNRYY
>ID14365-NO
ADVPGNYPLDSYGNCYPCTILGDNQYCI
>ID14366-NO
ADVPGNYPLNSYGASYYCTI
>ID14368-NO
ADWNKFQGSW
>ID14372-NO
ADYLQLTRA
>ID14373-NO
ADYLRLARA
>ID14374-NO
AEAAGLLPFPRV
>ID14375-NO
AEAASQVTN
>ID14377-NO
AEAMSQVAN
>ID14378-NO
AEAVPPGFTPFRKP
>ID14379-NO
AECCSNPACRVNNPHVC
>ID14383-NO
AEEPPISLDLTFHLLREVLEMARAEQIAQQAHSNRKLMDII
>ID14384-NO
AEEQNQPPPV
>ID14385-NO
AEFGS
>ID14386-NO

AEGEFGHWCDQHWLQYWYEGDPAK
>ID14387-NO
AEGEFGWWGDSHWLQYEGDPAK
>ID14388-NO
AEGEFIHNRYNRFFYWYGDPAK
>ID14389-NO
AEGEFLMWGGSHWLEYWYEGDPAK
>ID14390-NO
AEGEFMYWGDSHWLQYWYEGDPAK
>ID14391-NO
AEGEFPRWGDSHWLQYWYEGDPAK
>ID14393-NO
AEGLSSPLIRF
>ID14402-NO
AELPQGLWVRPRL
>ID14404-ABP_both
AELRCMCIKTTSGIH
>ID14405-NO
AELRCMCIKTTSGIHPKNIQSLEVIGKGTHCNQVEVIATLKDGRKICLDP
>ID14406-NO
AELTSCFPVDHECDGGASNCNCCGDDVYCA
>ID14420-NO
AFCCPSGWSAYDQ
>ID14421-NO
AFCKYNGEQCTSDGQCCNGRCRTAFMGKICMG
>ID14422-NO
AFCNLKMCQLSCRSLGLLGKCIGDKCECVKH
>ID14423-NO
AFCNLLMCQLSCRSLGLLGKCIGDKCECVKH
>ID14424-NO
AFCNLMRCQLSCRSLGLLGKCIGDKCECVKH
>ID14425-NO
AFCNLRKCQLSCRSLGLLGKCIGDKCECVKH
>ID14427-NO
AFCNLRMCQLSCRKSLGCLLGKCIGDKCKCYGC
>ID14431-NO
AFCRFNGQQCTSDGQCCNGRCINAFQGRICIG
>ID14434-NO
AFDHYGFTGGL
>ID14436-NO
AFDRFDNSGVFSFGS
>ID14438-NO
AFDRIEGSGFGLD
>ID14439-NO
AFDRMDNSDFFGA
>ID14440-NO
AFDSLAGQGFTGFE
>ID14441-NO
AFDSLAGSGFDNGFN
>ID14442-NO
AFDSLAGSGFGAFN

>ID14443-NO
AFDSLAGSGFSGFD
>ID14447-NO
AFFTTVKNLVTNVAGTVIDKMKCKLTGEC
>ID14449-NO
AFGLLTYPRI
>ID14450-NO
AFGTILKALAKIAAKAVKKLATKPGATYMLKQNLE
>ID14452-NO
AFGTILKALAKIAGKVVKKLATKPGATYMLKENLQ
>ID14453-ABP_both
AFGVLAKVAAHVVPAIAEHF
>ID14454-NO
AFHAMR
>ID14455-ABP_both
AFHHIFRGIVHVGKTIHRLVTG
>ID14456-NO
AFHSWG
>ID14461-NO
AFKAWAVAR
>ID14470-NO
AFPPPNVPGPRFPPPNFPGPRFPPPNFPGPRFPPPNFPGPRFPPPNFPGP
>ID14472-NO
AFPRS
>ID14476-NO
AFSSWG
>ID14480-ABP_both
AFTRCRRSYSTEYSYGTCTV
>ID14481-ABP_both
AFTRRRSYSTEYSYGTTV
>ID14484-NO
AFVKGSAQRVAHGY
>ID14489-NO
AGAGEPLAFSPDMLSLRF
>ID14490-NO
AGAKFIRF
>ID14491-NO
AGAKFIRFG
>ID14492-NO
AGANDLCQECEDIVHLLTKMTKEDAFQDTIRKFLEQECDILPLKLLVPRC
>ID14493-NO
AGAPEPAEPAQPGVY
>ID14497-NO
AGCCPTIMYKTGACRTNRC
>ID14499-ABP_pos
AGCICSGSVAVANSHNAGPAYCVGYCGNNGAVTRNANANLARTA
>ID14500-NO
AGCINGLCG
>ID14502-NO
AGCKNFYWKGFTSC
>ID14503-NO

AGCRLKSCA
>ID14505-NO
AGCRVESC
>ID14507-ABP_both
AGDKKIKIGINGFGRIGRL
>ID14509-NO
AGDRSNVTQLDGPAGALLLRLMQLAGAPEPQPAAPGGY
>ID14512-NO
AGEGLSSPFWSLAAPQR
>ID14518-ABP_neg
AGFAKLLAKLAKKLL
>ID14519-NO
AGFKNLNREQ
>ID14521-NO
AGFQHHPSFYRF
>ID14523-NO
AGGADNFMRF
>ID14524-NO
AGGAYSFGL
>ID14525-NO
AGGDSLYEPGKALASACQVAVEACAAWFPGPE
>ID14526-NO
AGGKSTCCPCAMCKYTAGCPWGQCAHHCGCS
>ID14527-NO
AGGLLDFGLSRGASGAEAAKARLGLKLANDPYGP
>ID14529-NO
AGGRQYGFGL
>ID14531-NO
AGGSGGVGGEYDDYGHLRF
>ID14533-NO
AGGTSGLYAFPRV
>ID14535-NO
AGIRRPPGFSPLRIA
>ID14537-ABP_both
AGKKTIRQYLKNKIKKKGRKWVIAW
>ID14538-ABP_both
AGKKTIRQYLKNKIKKKWRKAVIAW
>ID14539-ABP_both
AGKMIHGLV
>ID14541-NO
AGLAPY
>ID14543-NO
AGLFAQPRL
>ID14545-NO
AGLLVYPRL
>ID14550-NO
AGLTDADLKTEKGFLSGLLNVAGSVCCKVDTSCC
>ID14555-NO
AGLVAFPRV
>ID14556-NO
AGNAKMSALMALTIRGCCSHPVCSAMSPICG

>ID14558-NO
AGNDKATDLMALTVRGCCSHPACAGNNPHICS
>ID14559-NO
AGNSGANSGMWFGPRL
>ID14563-NO
AGPAPSRLYSFGL
>ID14568-NO
AGPRFIRF
>ID14569-NO
AGPSATTGVWFGPRL
>ID14570-ABP_both
AGPVSKLVSGIGL
>ID14571-NO
AGPYAFGL
>ID14572-NO
AGPYSFGL
>ID14573-NO
AGQDGFMRF
>ID14575-NO
AGQGFMRF
>ID14576-NO
AGQTLTASGD
>ID14577-ABP_both
AGRGKQGGKVRAKAKTRSS
>ID14579-ABP_both
AGRLTGNKGGLGGT
>ID14582-NO
AGSDGRLYSFGL
>ID14584-NO
AGSDPNFLRFG
>ID14585-NO
AGSEAGGNLQRTNFLRF
>ID14588-NO
AGTADCFWKYCV
>ID14589-NO
AGTKPQGKPASNLVECVFSLFKKCN
>ID14590-NO
AGTKPQGKPASSISKCVFSFFKKC
>ID14591-NO
AGTRMSWEV
>ID14593-ABP_both
AGTSLVKFFSSKMNK
>ID14595-NO
AGVSGYDNIYQVLAPRF
>ID14597-ABP_both
AGWLIERYLVPNWHAFTSWLTAGKDAFSKGK
>ID14599-NO
AGWNKFQGSW
>ID14600-ABP_both
AGWRDIVRGIRKVAAPVLST
>ID14601-NO

AGWSSLKGAW
>ID14602-NO
AGWSSMRGAW
>ID14605-NO
AGYGQISH
>ID14606-NO
AGYLLGKINLKALAALAKKILTYADFIASGRTGRRNAI
>ID14610-ABP_both
AGYLLPKINLKPLAKLPKKIL
>ID14611-ABP_both
AGYNLYRAIKKK
>ID14612-NO
AGYSRMIRFPRPPGFTPFRFAPEII
>ID14614-NO
AHALCLTERQIKIWFQNRRMKWKKEN
>ID14615-NO
AHALCPPERQIKIWFQNRRMKWKKEN
>ID14621-NO
AHEPVK
>ID14625-NO
AHKNFLRF
>ID14630-NO
AHRFAAEDFGALDTA
>ID14634-NO
AHVQTVGK
>ID14641-NO
AICTGADRPCAACCPCCPGTSCKAESNGVSYCRKDEP
>ID14646-ABP_pos
AIFIFIRWLLKLGHHGR
>ID14647-NO
AIFILAS
>ID14649-NO
AIGFDGLNDPDIVAR
>ID14652-NO
AIGNILKTLGNLAQKILGKQPKMLKLWKWNWKSSDVEYHLAKC
>ID14653-ABP_both
AIGSILGALAKGLPTLKSWIKNR
>ID14654-ABP_both
AIGSILGALAKGLPTLRSWIKNR
>ID14655-ABP_both
AIGSILGRLAKGLPTLISWIKNR
>ID14656-ABP_both
AIGSILGRLAKGLPTLKSWIKNR
>ID14657-NO
AIGWGDPITHWSHGQNRW
>ID14660-ABP_both
AIHKLAHKLLKKLLKAVKKLAK
>ID14662-ABP_both
AIHKLAHKLLKKTLRAVKKLAN
>ID14663-ABP_both
AIHKLAHKLLPKTLRAVKKLAN

>ID14666-ABP_both
AIHNLAHKLLKKLTRAVKKLAN
>ID14672-NO
AIHSELMWVS
>ID14673-NO
AIIRILQQLLFIHFRIGRRRRRRRR
>ID14675-NO
AIIYRDLIS
>ID14676-NO
AIKLTCCSELTCAGNYPNIC
>ID14679-NO
AILPYFAGCL
>ID14680-NO
AILRG
>ID14684-NO
AINPFLDSMG
>ID14685-NO
AINPFTDSI
>ID14688-NO
AIPFNGGMYG
>ID14689-NO
AIPNFVHKFV
>ID14690-NO
AIPNNQLGFPFK
>ID14692-NO
AIPSYSHNFV
>ID14694-ABP_both
AIQPKTKVIPYVRYL
>ID14695-ABP_both
AIQRCC
>ID14696-NO
AIRAPQLRLRF
>ID14697-ABP_both
AIRCLAKRRK
>ID14698-ABP_both
AIRCLAQRRK
>ID14705-NO
AITAKL
>ID14707-ABP_both
AIVVGGVMLGIIAGKNSGVDEAFFVLKQHHVEYGSDHRFEAD
>ID14714-ABP_both
AKAGGIPPHVIPQIVPVRIRPLCGNV
>ID14715-ABP_both
AKAWGIGGHVIGQIVGVRIRGLCGNV
>ID14720-NO
AKDEH
>ID14725-NO
AKDNFLRF
>ID14726-NO
AKDQG
>ID14727-NO

AKEKEVTFQ
>ID14728-NO
AKEKEVTFQSGGPT
>ID14733-NO
AKGKGAKASR
>ID14736-ABP_both
AKHQGLPQE
>ID14741-ABP_both
AKKAQKSGAQTIVKIFAKGM
>ID14746-NO
AKKELCTCQQPKHLKYIEKGLQKAKDYAT
>ID14747-ABP_both
AKKFFKKPRVIGVSIPF
>ID14748-ABP_both
AKKFGKAFVKIL
>ID14753-ABP_both
AKKPFVQRVKNAASKAYNKLKGLAMQSQYG
>ID14755-NO
AKKRAGNGCFGLKLDRIGSMSGLGC
>ID14769-NO
AKKRRQRRR
>ID14770-ABP_neg
AKKVFKRLGIGKFLHSAKKF
>ID14772-NO
AKKWFW
>ID14774-NO
AKLEK
>ID14776-ABP_both
AKLWALKAKLWALKAKLWALK
>ID14777-ABP_both
AKLWLKA
>ID14778-ABP_both
AKLWLKAAKLWLKA
>ID14779-ABP_both
AKLWLKAAKLWLKAAKLWLKA
>ID14780-ABP_both
AKLWLKAAKLWLKAAKLWLKAAKLWLKA
>ID14786-NO
AKPDT
>ID14787-NO
AKPEG
>ID14789-NO
AKQHHGYKQKFH
>ID14793-NO
AKRHHGLNCAKFH
>ID14794-NO
AKRHHGLNCAKGVLA
>ID14797-NO
AKRHHGYKRKF
>ID14799-NO
AKRHHGYKRKFHAKRHHGYKRKFH

>ID14800-NO
AKRHHGYKRKFHAKRHHGYKRKFHAKRHHGYKRKFH
>ID14801-NO
AKRLLGYKRKFL
>ID14803-NO
AKRRH
>ID14804-ABP_both
AKRRLLKKMK
>ID14813-NO
AKSCQ
>ID14818-NO
AKSYNFGL
>ID14819-NO
AKTGILDSDGPTVAGNSLSGT
>ID14824-NO
AKTVQ
>ID14826-NO
AKVKDEPQRRSARLSAKPAPPKPEPKPKKAPAKK
>ID14832-NO
AKWMDDVIKACGRELVRAQIAICGKSTLG
>ID14834-NO
AKYCY
>ID14835-NO
AKYSY
>ID14840-NO
ALAFQ
>ID14841-NO
ALAGDHFFRF
>ID14847-NO
ALARQPLTGSPPNERAFFCSSLRR
>ID14848-ABP_both
ALASLLKTLSKAAKKALKTLLKALSA
>ID14853-NO
ALCCYGYRFCCPNFR
>ID14855-NO
ALDDLEGVGFGGML
>ID14857-NO
ALDGLDGSGFGFD
>ID14858-NO
ALDIYSAVDDASHEKELPR
>ID14861-NO
ALDSLGGFQVHGW
>ID14867-NO
ALFKIMKKIAESL
>ID14868-NO
ALFKIMNKIADSL
>ID14869-ABP_both
ALFKTMLKKLGTMAL
>ID14870-ABP_both
ALFKVASKVLPSV
>ID14871-ABP_both

ALFSLASKVVPSVFSMVTKK
>ID14872-ABP_both
ALGALFKVASKVL
>ID14873-NO
ALGAPAAGDCVSASPQALLSILNAAQAEVQKLIDCSRFTSEANS
>ID14875-NO
ALGERKYHFQ
>ID14876-ABP_both
ALGKLASKVFPAVYCTISRK
>ID14880-NO
ALGTLLKGVGSAVATVGKMVADQFGKLLQA
>ID14910-NO
ALGVPLKRK
>ID14912-ABP_both
ALHKTMLKKLGTMAL
>ID14914-NO
ALIILRRRIRKQAHAHSK
>ID14917-NO
ALKAWSVAR
>ID14918-NO
ALKCQGWVDYCNGNVECCNECVMY
>ID14922-ABP_both
ALKSLLATLSKAKKKKLKTLLAALSK
>ID14924-ABP_both
ALKSLLKTLSAAKKKKLATLLKALSK
>ID14926-ABP_both
ALKSLLKTLSKAKAAKLKTLLKALSK
>ID14927-NO
ALKYPFRCKAAFC
>ID14928-ABP_both
ALKYRLFKKLKKF
>ID14931-NO
ALLCGGTHARCNRDNDCCGSLCCFGTCISAFVPC
>ID14932-NO
ALLGFHGMR
>ID14934-ABP_both
ALLKLAPRLLAGIF
>ID14935-ABP_both
ALLKRIKTLL
>ID14936-ABP_both
ALLKTMLKKLGTMAL
>ID14942-NO
ALLVTAGLVLA
>ID14944-ABP_both
ALNALKKVSQGIHEAIKLIANHVQ
>ID14946-NO
ALNEINQFY
>ID14947-NO
ALNEINQFYQK
>ID14949-NO
ALNSLDGAGFGFE

>ID14951-NO
ALNSLDGQGFGFE
>ID14952-NO
ALNSVAFERSAMQNYE
>ID14954-NO
ALPCPYGCPLRCCHMTDGVCLRNKQGC
>ID14955-NO
ALPFSSWG
>ID14956-NO
ALPHA
>ID14958-NO
ALPMH
>ID14959-NO
ALPMHIR
>ID14961-ABP_pos
ALPQYLKTVYQHQKAMKPWIQPKTKVIPYVRYL
>ID14962-NO
ALPVCGETCVGGTCNTPGCTCSWPVCTRN
>ID14964-NO
ALQGAKERAHQQ
>ID14966-NO
ALRAAAVAGSPQQLLPLGQRERKAGCKNFFWKTFSSC
>ID14967-NO
ALRDRPM
>ID14968-NO
ALRGCKTKSIPPKRCPGKK
>ID14975-NO
ALSGDAFLRF
>ID14981-NO
ALTPPSLLSHGHFNRASDLGFSDLYDVQDWSSE
>ID14982-NO
ALTTLYAFGL
>ID14984-NO
ALVGLMR
>ID14985-NO
ALVLIAFAQVLQQCP
>ID14988-ABP_both
ALVYAGIKKTAFLKVQKCDG
>ID14989-ABP_both
ALWHHLLHHLLHSAHHLG
>ID14990-ABP_both
ALWKDILKNLLKAALNEINQIVQ
>ID14993-ABP_both
ALWKEVLKNAGKAALNEINNLVQ
>ID14995-ABP_both
ALWKKLLKKLLKSAKKLG
>ID14996-ABP_both
ALWKNMLKGI
>ID14997-ABP_both
ALWKNMLKGIGK
>ID14999-ABP_both

ALWKNMLKGIGKLAGK
>ID15000-ABP_both
ALWKNMLKGIGKLAGKAALG
>ID15001-ABP_both
ALWKSLLKNVGKA
>ID15002-ABP_neg
ALWKTLLKKVLK
>ID15003-ABP_neg
ALWKTLLKKVLKAA
>ID15004-NO
ALWKTLLKKVLKAPKKKRKV
>ID15005-ABP_both
ALWKTMLKKAAHVGKHVGKAALTHYLN
>ID15006-ABP_both
ALWKTMLKKLGT
>ID15008-ABP_both
ALWKTMLKKLGTMA
>ID15010-ABP_both
ALWKTMLKKLGTMALHAGK
>ID15013-NO
ALWMRWYSPTTRRYG
>ID15015-ABP_neg
ALWMTLKKKVLKAKAKALNAVLVGANA
>ID15017-ABP_both
ALWRRLLRRLLRSARRLG
>ID15018-NO
ALWTTMLKKLGKMALHAGKAALGAAADTI
>ID15029-ABP_neg
ALYKKIIKKLLESAKKLG
>ID15034-ABP_both
AMAINNWVRVPPCDQVCSRSNPEKDECCRAHGHAFHAHCNGGMNCYRR
>ID15035-NO
AMAPKFF
>ID15037-NO
AMDIIIIVGG
>ID15038-ABP_pos
AMDPTKYYGNGVYCNSKKCWVDFGQASGCIGQTVVGGWLGGAIPGKC
>ID15039-ABP_pos
AMDPTKYYGNGVYCNSKKCWVDWGQASGCIGQTVVGGWLGGAIPGKC
>ID15043-NO
AMDSPLIRF
>ID15046-NO
AMGMLRM
>ID15047-NO
AMKPW
>ID15048-NO
AMKPWIQPK
>ID15051-NO
AMMGGFQGMR
>ID15052-NO
AMMRFG

>ID15053-NO
AMPKPW
>ID15056-ABP_both
AMRLTYNKGCLYGT
>ID15057-ABP_both
AMRLTYNKPCLGGT
>ID15058-ABP_both
AMRLTYNKPCLYGT
>ID15059-NO
AMRLTYNKPCLYGTKRTKEM
>ID15061-NO
AMRLTYNRPCIYATKRTKEM
>ID15062-NO
AMRMTYNRPCLYATKRTKEM
>ID15064-NO
AMRNALVRFG
>ID15065-NO
AMRNSLVRF
>ID15066-NO
AMRPPFRCKAAFC
>ID15068-NO
AMRTTYNRPCLFASKRTKEM
>ID15075-NO
AMVRF
>ID15079-NO
ANCVNYFEITFPEVCEANWCAAECLKAYKNGKGTCWQKFCQCVYDC
>ID15080-NO
ANEDEDAASLFAFGL
>ID15085-NO
ANFPTVSLNTVGITRQIPQDFMNAV
>ID15086-NO
ANIKLSVQMKLFKRHLKWKIIVKLNDGRELSLDA
>ID15090-NO
ANKRPVWIMAHMVNAVAQIDEFVNLGANSIETD
>ID15091-NO
ANKRPVWIMGHMVNAVYQIDEFVNLGANSIDTDVS
>ID15093-NO
ANLMRF
>ID15094-NO
ANMEAGTMSHFPSLPQRF
>ID15098-NO
ANPICPKIPNPTIRISGRNDLCVDVK
>ID15099-NO
ANQYAFGL
>ID15100-NO
ANQYTFGL
>ID15101-ABP_both
ANRLLEAYKMLLKFLGNLR
>ID15102-NO
ANRSLRLRF
>ID15104-NO

ANRYGFGL
>ID15107-NO
ANSKVAFSAVRSTN
>ID15108-NO
ANSLRACGPALMDMLRVACPNGFNSMFA
>ID15110-NO
ANSNPALAPRE
>ID15111-NO
ANSPNIFGQWM
>ID15112-NO
ANSSIL
>ID15114-NO
ANTPCGPYTHDCPVKR
>ID15117-NO
APAAEPAPSVKSQNFGAPGGAYPW
>ID15118-NO
APAAGFFGMR
>ID15120-NO
APAEHRFSFGL
>ID15123-NO
APAGFLGMR
>ID15128-NO
APALRLRF
>ID15129-NO
APAMGFQGVR
>ID15130-NO
APANSVWS
>ID15132-NO
APAPSGGGSAPLAKIYPRGSHWAVGHLM
>ID15133-NO
APASGFFGMR
>ID15134-NO
APCCSHLDASPFQRP
>ID15136-NO
APCGLLACI
>ID15137-NO
APCTYPGQQCKSDDECCHGTCKTAFIGRICMR
>ID15138-NO
APEASPFIRF
>ID15140-NO
APEESPKRAPSGFLGVR
>ID15150-NO
APFCIGYLSPKLKDMEPKPRG
>ID15151-NO
APFIFPGPKV
>ID15152-ABP_both
APFLKKVIQKILDSGNKKN
>ID15153-NO
APFPRIL
>ID15154-NO
APFRMGICTTN

>ID15155-NO
APFRMWYMYHKLKDMEPKPMA
>ID15156-NO
APFRNSEMMTARGF
>ID15157-NO
APGAGVY
>ID15158-NO
APGCCNNPACVKHRC
>ID15161-NO
APGDRIYVHPF
>ID15162-NO
APGKIPVKAIKKAGAAIGKGLRAINIASTAHDVYSFFKPKHKKKH
>ID15164-NO
APGLFELPSRSV
>ID15167-NO
APGPYAFGL
>ID15168-NO
APGQDFMRF
>ID15170-NO
APGYSHSFV
>ID15171-ABP_both
APKAMHSAKKFGKAFVGEIMLQKKGI
>ID15173-ABP_both
APKAMRLLRRLLRRLLR
>ID15177-NO
APKPKFIRFG
>ID15178-NO
APKQMVFGF
>ID15180-NO
APKQMVRF
>ID15182-ABP_both
APKVNVNALKKGGHVIKKGLGVIGAAGTAHEVYNHVRNRNQG
>ID15184-NO
APLAFVGLR
>ID15185-NO
APLDRSALVRF
>ID15186-NO
APLEGFEDMSGFLRTIDGIQKPRF
>ID15187-NO
APLEPAYPGDNATPEQMAQYAAELRKYINMVTRPRY
>ID15188-NO
APLEPVYPGDDATPEQMAQYAAELRRYINMLTRPRY
>ID15189-NO
APLEPVYPGDDATPQQMAQYAAEMRRYINMLTRPRY
>ID15190-NO
APLEPVYPGDNATPEQMAQYAAELRRYINMLTRPRY
>ID15192-NO
APLMGFQGVR
>ID15193-NO
APLQPGGSPALTKIYP
>ID15195-NO

APLSGFYGVR
>ID15197-NO
APLVDIGEQYRKYLEEKQNNP
>ID15199-NO
APMGFQGMR
>ID15200-NO
APMGFYGT
>ID15201-NO
APMGFYGTR
>ID15204-NO
APMSFVGMR
>ID15206-NO
APNQPSDNMIRF
>ID15208-NO
APNRVLMRFG
>ID15210-NO
APNVKDSKASGSCCDNPSCAVNNRHCGRRR
>ID15211-NO
APNVKDSKASGSCCDNPSCAVNNSHC
>ID15214-NO
APPAALVTNGHNLGLLDIYDIQDRPTDI
>ID15215-NO
APPEPVYPGDDATPEQMAEYVADLRRYINMLTRPRY
>ID15216-NO
APPGWSPFR
>ID15217-NO
APPQILKSQSLIPFPRV
>ID15218-NO
APPQPSDNFIRF
>ID15219-NO
APPSDFMRF
>ID15221-NO
APQAGFYGVR
>ID15222-NO
APQGNFLRF
>ID15226-NO
APQPYAFGL
>ID15227-NO
APQRNFLRF
>ID15229-ABP_both
APRIKKIVQKKLAGD
>ID15236-ABP_neg
APRKNVRWCTISQPEWFKCRRWQWRMKKLGAPSITCVRRAFALECIRA
>ID15239-ABP_both
APRKNVRWCTISQPEWLECIRA
>ID15241-NO
APRLRFY
>ID15242-NO
APRLRFYS
>ID15243-NO
APRLRFYSL

>ID15244-ABP_both
APRMEIGKRREKLGRNVFKAAKKALPVIAGYKALG
>ID15245-NO
APRNFLRF
>ID15246-NO
APRPG
>ID15247-NO
APRPGPWLWSNADSV
>ID15248-NO
APRPYSFGL
>ID15250-NO
APSAK
>ID15252-NO
APSAPAGLEEKLR
>ID15253-NO
APSDFMRF
>ID15254-NO
APSEPHHPGDQATQDQLAQYYSDLYQYITFVTRPRF
>ID15255-NO
APSFCGTAMLGASRYCYSGP
>ID15256-NO
APSGAQRLYGFGL
>ID15257-NO
APSGFFGMR
>ID15258-NO
APSGFLDMR
>ID15259-NO
APSGFLGLR
>ID15260-NO
APSGFLGMR
>ID15262-NO
APSGFLGVR
>ID15263-NO
APSGFMGMR
>ID15264-NO
APSGFNGMR
>ID15265-NO
APSGFQ
>ID15266-NO
APSGFQGMR
>ID15267-NO
APSGQDFMRF
>ID15268-NO
APSKNFLRF
>ID15270-NO
APSLRLRF
>ID15271-NO
APSMGFMGMR
>ID15272-NO
APSMGFQGMR
>ID15273-NO

APSQDFMRF
>ID15274-NO
APSQRLRW
>ID15275-NO
APSQTYH
>ID15279-NO
APSSGFFGTR
>ID15280-NO
APSSMGFMGMR
>ID15281-NO
APSSNSFMGMR
>ID15282-NO
APSWRPQGRF
>ID15283-NO
APTAFYGVR
>ID15284-NO
APTDMYSFGL
>ID15286-NO
APTGHQEMQ
>ID15288-NO
APTQRLRW
>ID15292-NO
APVMGFQGMR
>ID15293-NO
APVNSFLGVR
>ID15296-NO
APVPP
>ID15298-NO
APWHLSSQYSRT
>ID15303-NO
APWLVPSTITTCCGYNPGTMCPPCRCDNTC
>ID15306-NO
APWTVVTATTNCCGITGPGCLPCRCTQTC
>ID15309-NO
APYGFGI
>ID15310-NO
APYGFTGMR
>ID15324-NO
AQEPVKGPVSTKPGSCPIILIRCAMLNPPNRCLKDTDCPGIKKCCEGSCG
>ID15329-NO
AQGIVLQLAL
>ID15330-NO
AQHQYSFGL
>ID15331-NO
AQIKIWFQNRRMKWKK
>ID15339-NO
AQKCGEQGRGAKCPNCLCCGRYGFCGSTPDYCGVGCQSQCRGCR
>ID15340-ABP_neg
AQKIISTIGKLPKWIIKTVNKFTKK
>ID15341-ABP_both
AQKIISTIGKLVKWIIKTVNKFTKK

>ID15345-NO
AQNVIAPNTLSNSIRMLGSQSPLIQAYG
>ID15346-NO
AQPSFAF
>ID15348-NO
AQQHHGYKQQFH
>ID15350-NO
AQQICKAPSQTFPGLCFMDSSCRKYCIKEKFTGGHCSKLQRKCLCTKPC
>ID15351-NO
AQQLLFIHFRIGRRRRRRRR
>ID15353-NO
AQRHHGYKRQFH
>ID15354-NO
AQRSPSLRLRF
>ID15355-NO
AQSFLRL
>ID15356-NO
AQSFVRL
>ID15358-NO
AQSTAFQKPLLM
>ID15361-NO
AQTQSL
>ID15362-NO
AQTQSLVYP
>ID15364-ABP_both
AQWFAIQHISLNPPRSTIAMRAINNYRWR
>ID15365-ABP_both
AQWFAIQHISLNPPRSTIAMRAINNYRWRSKNQNTFLR
>ID15374-NO
ARAYDFGL
>ID15375-NO
ARCEQCPSYCCQSDSPPECDGCE
>ID15376-NO
ARCRG
>ID15377-NO
ARCRVDPCV
>ID15379-NO
ARDAYIAKPHNCVYECFDAFSSYCNGVCTKNGAKSGYCQILGTYGNGCWC
>ID15381-NO
ARDAYIANDRNCVYTCALNPYCDSECKKNGADSGYCQWFGRFGNACWCKN
>ID15382-NO
ARDECCSNPACRVNNPHVC
>ID15384-NO
ARDLPGQ
>ID15385-NO
AREFV
>ID15389-NO
ARFLHPFQYYTLYRYLTRFLHRYPIYYIRY
>ID15393-ABP_both
ARGLTYNKPCLYGT
>ID15394-NO

ARGMT
>ID15395-NO
ARGPQLRLRF
>ID15396-NO
ARGQT
>ID15399-NO
ARGYDFGL
>ID15400-NO
ARHEI
>ID15409-NO
ARHPHPHLSF
>ID15422-NO
ARIQESTNDILKPITCNTNADCAKFCKGPIHNCVYHTCQCVPGNPHCC
>ID15430-ABP_pos
ARKKHRCRVYNNGLPTGLYRWC
>ID15431-ABP_both
ARKKWFW
>ID15440-NO
ARMGFHGMR
>ID15441-NO
ARMMSFDAEAPQYLQHLLQNLKPRF
>ID15443-ABP_both
ARNFGKEFTPV
>ID15444-ABP_both
ARNFGKEFTPVLQADFQKVVAGVANALAHRYH
>ID15446-NO
ARNHFIRL
>ID15447-NO
ARNMT
>ID15448-NO
ARNQT
>ID15450-NO
ARPAK
>ID15451-NO
ARPAKAAATQKKVERKAPDA
>ID15452-NO
ARPDKPRPYLPRPRPPRPVR
>ID15453-NO
ARPDNFIRF
>ID15454-NO
ARPGYLAFPRM
>ID15456-NO
ARPPGFSPFRIV
>ID15457-NO
ARPSR
>ID15458-NO
ARPSSNFIRL
>ID15459-NO
ARPVKEP
>ID15460-NO
ARPVKEPR

>ID15461-NO
ARPVKEPRGLSAASPPLAETGAPRRF
>ID15462-NO
ARPVKEPRSLSAASAPLAETSTPLRL
>ID15464-NO
ARPYAFGL
>ID15465-NO
ARPYSFGL
>ID15468-ABP_both
ARRARKWARRALKLA
>ID15495-NO
ARSDNFVRL
>ID15497-NO
ARSSIQSLLNLPQ
>ID15501-NO
ARSYNFGL
>ID15502-NO
ARTDNFVRL
>ID15503-NO
ARTINAQQAELDSALLAAAGFGNTTADVFDRG
>ID15504-NO
ARTQPGNIAPVGDFTLYPNAPRQGHIVA
>ID15506-NO
ARVSFWRYSSFAPTY
>ID15511-ABP_both
ARWWWKIKS
>ID15521-NO
ASALIAQAILRDCCSNPPCAHNNPDC
>ID15528-NO
ASCNGVCSPFEMPPCGTSACRCIPVGLVIGYCRNPSG
>ID15529-NO
ASDAAHQGCCSNPVCHVEHPELC
>ID15530-NO
ASDDRVLGWNKAHGLW
>ID15532-NO
ASDRSNATLLDGPSGALLLRLVQLAAAPEPAEPAQPGVY
>ID15534-NO
ASDVTLNSFAEDVTVGECCDCVDLTTVY
>ID15535-NO
ASDWNRLSGMW
>ID15536-NO
ASEDALFGTMRF
>ID15538-ABP_both
ASEELLLRFQATSSGPILREEFE
>ID15544-NO
ASGDPNFLRF
>ID15545-NO
ASGENQAELKLATHNVYML
>ID15546-NO
ASGFFGMR
>ID15547-NO

ASGGMRNALVRF
>ID15548-NO
ASGGQDFMRF
>ID15549-NO
ASGLVAFPRV
>ID15551-NO
ASGQDFMRF
>ID15552-NO
ASGRDIAMAIGTLSGQFVAGGIGAAAGGVAGGAIYDYASTHKPNPAMSPS
>ID15553-NO
ASGSSDFMRF
>ID15558-ABP_both
ASHLPSDFTPAVHASLDKFLANVSTVLTSKYR
>ID15570-NO
ASKQANLMRF
>ID15574-NO
ASLFNAPRV
>ID15579-NO
ASMRGFQGMR
>ID15580-NO
ASMRSFNMGF
>ID15581-NO
ASMWERVKSIIKSSLAAASNI
>ID15589-NO
ASPKCFKYYVLACT
>ID15591-NO
ASPSAPFIRF
>ID15592-NO
ASPSAPLIRF
>ID15593-NO
ASPSFIRF
>ID15594-ABP_both
ASPTVFRSSVFLSLFVVAKK
>ID15596-NO
ASPYAFGL
>ID15597-NO
ASPYGFAFA
>ID15604-NO
ASSAPFIRF
>ID15605-NO
ASSAPLIRF
>ID15606-NO
ASSAPLIRFGR
>ID15607-NO
ASSGLISMPRV
>ID15608-NO
ASSLHTI
>ID15609-NO
ASSPRVDHRLV
>ID15612-NO
ASSSYPLIHWRPWAR

>ID15614-NO
ASVPPGFTPFRVAPEIV
>ID15615-NO
ASVRT
>ID15617-NO
ASWASSVRF
>ID15618-NO
ASWGEFQ
>ID15619-NO
ASWSACSVSCGGGARQRTR
>ID15621-NO
ASYDTKF
>ID15622-NO
ASYDYIRF
>ID15623-NO
ATAEETRRMLHRAFDA
>ID15625-NO
ATALGIPPRGFLPIVNKFKDIILC
>ID15626-NO
ATALGLSSRGLLPIGFMFKDTIRCRKY
>ID15627-NO
ATAVDFGPHGLLPIRPIRIRPLCG
>ID15629-NO
ATAWRMPPNGIPPIVAVRIRPLCGTV
>ID15630-NO
ATCAGQDQPCKETCDCCGERGECVCGGPCICRQGYFWIAWYKLANCKK
>ID15631-NO
ATCAGQDQTCKVTCDCCGERGECVCGGPCICRQGNFLIAWYKLASCKK
>ID15632-ABP_pos
ATCDLASIFNVNHTLCAAHCIARRYRGGYCNSKAVCVCR
>ID15633-ABP_pos
ATCDLASIFNWNHALCAAHCIARRYRGGYCNSKAVCVCR
>ID15634-ABP_pos
ATCDLASIFNWNHTLCAAHCIARRYRGGYCNSKAVCVCR
>ID15635-ABP_pos
ATCDLASIWNVNHALCAAHCIARRYRGGYCNSKAVCVCR
>ID15643-ABP_pos
ATCDLASKWNWNHTLCAAHCIARRYRGGYCNSKAVCVCR
>ID15644-ABP_both
ATCDLLSATGFSGTACAAHCLLIGHRGGYCNTKSVCVCRD
>ID15645-NO
ATCDLLSFDTKWGSLNHSACAAHCIALRKGYKGGRCYKQVCRCRK
>ID15649-NO
ATCDLLSLTSKWFTPNHAGCAAHCIFLGNRGGRCVGTVCHCRK
>ID15650-NO
ATCEDCPEHCATQNARAKCDNDKCVCEPK
>ID15663-NO
ATGQYAFGL
>ID15664-ABP_both
ATHCLFLKKR
>ID15665-NO

ATITFTNKCTRTVWPGTLTGDQKPQLSKTGFELASGVSTRGAAPPATLIE
>ID15666-ABP_both
ATKCFQWQRNMRKVR
>ID15689-NO
ATLDGVS
>ID15690-NO
ATNNKATDLMALTVRGCCDDPSCRAENPFLCSWMG
>ID15694-NO
ATPFGGSSYEGH
>ID15695-NO
ATPFIECSGARGTCHYFAN
>ID15696-NO
ATRATNGGYTTYTTNGT
>ID15706-NO
ATSNRPCKPKGRKCFPHQKDCCNKTCTRSKCP
>ID15707-NO
ATTGCSCPQCIIFDPICASSYKNGRRGFSSGCHMRCYNRCHGTDYFQISK
>ID15718-NO
ATYNGKCYKKDNICKYKAQSGKTAICKCYVKKCPRDGAKCEFDSYKGKCY
>ID15719-ABP_both
ATYRTGRATRESLSGVEISGRLYRLR
>ID15723-NO
AVALPRI
>ID15726-NO
AVCNLARCQLSCASLGLLGKCIGDKCECVKH
>ID15727-NO
AVCNLARCQLSCRSLGLLGKCIGDKCECVKH
>ID15728-NO
AVCNLKRCPLSCRSLGLLGKCIGDKCECVKH
>ID15731-NO
AVCNLKRCQLSCRSLGLLGKCMNGKCECVKH
>ID15733-NO
AVCVYRTCDKDCKRRGYRSGKCINNACKCYPY
>ID15736-NO
AVDFSSCARMDVPGLSKVAQGLCISSCKFQNCGTGHCEKRGGRPTCVCDR
>ID15738-NO
AVDLAKIANIANKVLSSLF
>ID15740-NO
AVDPRMFYLLLRGGA
>ID15741-NO
AVDQDLGPEVPPENVLGALL
>ID15742-NO
AVDQDLGPEVPPENVLGALLRV
>ID15743-NO
AVDQDLGPEVPPENVLGALLRVKRLENSSPQAPARRLLPP
>ID15746-NO
AVEKMFTFRCGLSGNC
>ID15747-NO
AVERPRQDGQVHEPPGRERKAGCKNFFWKTFTSC
>ID15750-NO
AVESTVATL

>ID15754-NO
AVFTDNYSRF
>ID15764-NO
AVHYSGGQPLGSKRPNDMLSQRYHFGL
>ID15765-NO
AVIPPLRCKAVFC
>ID15770-NO
AVKLPFRCKAVFC
>ID15771-NO
AVKLSSDGNYPFDLSKEDGAQPYFMTPRLRFYPI
>ID15773-NO
AVKQPAVVYP
>ID15774-NO
AVKSSSYEKYPFDLSKEDGAQPYFMTPRLRFYPI
>ID15782-NO
AVLHLAINEFQRL
>ID15786-ABP_both
AVLSALPKEEQIGKASTRGRKAARRKK
>ID15787-ABP_both
AVLSWLPKEEQIGKWSTRGRKWWRRKK
>ID15788-NO
AVMGLAA
>ID15790-NO
AVNACSSLF
>ID15791-ABP_both
AVNIPFKVHFR
>ID15793-ABP_both
AVNIPFKVHLRCFAAFC
>ID15795-NO
AVNPFLDSIG
>ID15796-NO
AVNPIR
>ID15799-NO
AVPAENALNNPF
>ID15803-NO
AVPGVLRF
>ID15816-NO
AVPLIYNPPGIYATKDQKENNLLEII
>ID15818-NO
AVPRPRI
>ID15819-NO
AVPYP
>ID15820-NO
AVPYPQ
>ID15821-NO
AVPYPQP
>ID15832-NO
AVQRTDGESRAHLGALLARYIQQARKAPSGRMSIIKNLQNLDPSHRISD
>ID15837-NO
AVRPPWRCKAAFC
>ID15845-NO

AVTCNTVVSSLAPCVPFFAGSAAQPTAACCNGVRSLNSAARTTPDRRTAC
>ID15849-ABP_both
AVVDILKGAAKDIAGHLASKVMNKL
>ID15850-NO
AVVPPLRCKAVFC
>ID15853-ABP_neg
AVWMTRSCVIWKR
>ID15856-ABP_both
AWCARVCYRGICYRRCR
>ID15857-ABP_both
AWCFRVCARGICYRRCR
>ID15858-ABP_both
AWCFRVCYRGACYRRCR
>ID15859-ABP_both
AWCFRVCYRGICYRRCR
>ID15860-NO
AWERFHGSW
>ID15862-ABP_both
AWKEKIRKKLKNEIKKKWRKAVIAW
>ID15863-ABP_both
AWKEKIRQYLKNEIKKKWRKAVIAW
>ID15865-ABP_both
AWKETIRQYLKNKIKKKWRKAVIAW
>ID15867-ABP_both
AWKKTIRQYLKNEIKKKWRKAVIAW
>ID15870-NO
AWKSMNVAW
>ID15871-ABP_both
AWLDKLKSLGKVVG
>ID15872-ABP_neg
AWLKKRRWKKAKPP
>ID15873-NO
AWLVTCP
>ID15878-NO
AWPENPSRRPF
>ID15881-NO
AWQDLGSAW
>ID15882-NO
AWQDLNAGW
>ID15884-NO
AWQDLNTGW
>ID15886-NO
AWQDLQGAW
>ID15887-NO
AWQDLQGGW
>ID15888-NO
AWQSLQSSW
>ID15891-NO
AWRRKLKALAPAKKAKV
>ID15892-ABP_pos
AWRRWIRWL

>ID15895-NO
AWSALHGAW
>ID15897-NO
AWSNLGQAW
>ID15898-NO
AWSNLQGAW
>ID15923-NO
AYCCHPACGKNFDC
>ID15924-NO
AYCCHPACGPNYSCGTSCS
>ID15925-NO
AYCCHPVCGKNFDC
>ID15926-NO
AYDPDPYKRYSAEHTFFLL
>ID15927-NO
AYEES
>ID15929-NO
AYFDEKKSVPGVLRF
>ID15931-NO
AYFSPWG
>ID15932-NO
AYFTPE
>ID15934-NO
AYFYP
>ID15935-NO
AYFYPE
>ID15936-NO
AYFYPEL
>ID15937-NO
AYGFRDPGPQL
>ID15942-NO
AYIASKGL
>ID15945-NO
AYKRQ
>ID15954-NO
AYMYTNGGPGM
>ID15955-NO
AYNGPLA
>ID15956-NO
AYNQSFLRF
>ID15957-NO
AYNRSFLRF
>ID15959-NO
AYPQQ
>ID15962-NO
AYRIKPTFRRLKWKYKGKFW
>ID15963-ABP_both
AYRIPAIAGERRYGTIYQGRLWAF
>ID15964-NO
AYRKPPFNGSIF
>ID15965-NO

AYRKPPFNGSLF
>ID15974-NO
AYSMEHFRWGKPV
>ID15975-NO
AYSMEHFRWGKPVGRKRRPIKVYPNGVEEESAESFPMEL
>ID15976-NO
AYSMEHFRWGKPVGRKRRPIKVYPNGVEEESAESYPMEL
>ID15977-NO
AYSNLNYLRF
>ID15979-NO
AYSYVSEYKRLPVYSFGL
>ID15980-NO
AYTGV
>ID15981-NO
AYTKCSRQWRTCMTTH
>ID15982-NO
AYTQQ
>ID15983-NO
AYTTEQCRALNGTCRFYACFPKNVVIGKCDWLG
>ID15984-NO
AYTYVSEY
>ID15985-NO
AYTYVSEYKRLPVYNFGI
>ID15987-ABP_both
AYVLDEPKPIKDLEKSLQHNLVYCRRLVLEYFLKSIFEYH
>ID15998-NO
CAAHYRVGPWC
>ID16001-NO
CADPNSVRAHC
>ID16003-NO
CAEFQSKCKKDSECCGTLECSPTWKWCVYPSPF
>ID16005-NO
CAFIM
>ID16006-NO
CAGAGAKCSRLMYDCCTGSCRSGKC
>ID16008-NO
CAGKGAKCSRLMYDCCTGSCRSGKC
>ID16010-NO
CAGRRSAYC
>ID16011-ABP_both
CAIEHMRTPLKPFSFGPPYNL
>ID16013-NO
CAKGYRSC
>ID16014-NO
CAKIDPELC
>ID16015-NO
CAKKRNWCGKNEDCCCPMKCIYAWYNQQGSCQTTITGLFKKC
>ID16016-NO
CAKKRNWCGKTEDCCCPMKCVYAWYNEQGSCQSTISALWKKC
>ID16017-NO
CALIIQKNEC

>ID16018-NO
CALRDRPMC
>ID16021-NO
CAMVSMED
>ID16023-NO
CANIFRKDGLLNNCRSNCFYNTE
>ID16026-NO
CAPFLHPCTFFFPNCCNSYCVQFICL
>ID16028-NO
CAQLLQVSC
>ID16029-NO
CAQSSELCDALDSDCCSGVCMVFFCL
>ID16032-NO
CASLSCR
>ID16033-NO
CASNNGRVVL
>ID16066-NO
CATYGKPCGIQNDCCNICDPARRTCT
>ID16067-NO
CAVCNGRCGF
>ID16068-NO
CAVLQCN
>ID16072-NO
CCAPSACRLGCRPCCR
>ID16081-NO
CCCNPACGPNYGCGTSCSRPSEPRR
>ID16083-NO
CCDDSECDYSCWPCCIFS
>ID16086-NO
CCDDSECSYSCWPCCY
>ID16087-NO
CCDEEECSSACWPCCW
>ID16088-NO
CCDLPCNAGCVPCC
>ID16089-NO
CCDPDWCDAGCYDGCC
>ID16090-NO
CCDRPCGIGCVPCCLP
>ID16094-NO
CCDWEWCDELCSCCW
>ID16099-NO
CCDWPCTIGCVPCCLP
>ID16100-NO
CCEADCPMCQDMLCC
>ID16101-NO
CCEAPFCNAGCTCCNP
>ID16102-NO
CCEEPFCNAGCACCNP
>ID16103-NO
CCELPCHGCVPCCWP
>ID16104-NO

CCEPQWCDGACDCCS
>ID16105-NO
CCFLSGCFECW
>ID16107-NO
CCFWPDCRGCYCCL
>ID16109-NO
CCFWPMCRGCDCCYL
>ID16135-NO
CCGIPNAACHPCVCTGKC
>ID16143-NO
CCGPTACLAGCKPCCY
>ID16144-NO
CCGPTACMAGCRPCC
>ID16146-NO
CCGVPNAACHPCVCKNTC
>ID16148-NO
CCGVPNAACPPCVCNKTCG
>ID16155-NO
CCHAPYCTPPHLGCPCC
>ID16156-NO
CCHPACAERYNC
>ID16158-NO
CCHPACGKHFNC
>ID16163-NO
CCHPACGQNTSC
>ID16165-NO
CCHPACGRKYNC
>ID16166-NO
CCHPACGRKYNCGR
>ID16167-NO
CCHPSCGRKYNC
>ID16169-NO
CCHSSWCKHLC
>ID16171-NO
CCHWNWCDHLCSCCGS
>ID16172-NO
CCHWPYCAPPPLGCRCC
>ID16175-NO
CCIGPKCMEECYCCV
>ID16176-NO
CCIKFHPCCHN
>ID16177-NO
CCILCWKCTYNCCRAW
>ID16178-NO
CCISPACHEECYCC
>ID16180-NO
CCISPACNDSCYCCQ
>ID16181-NO
CCITFESCCEFDL
>ID16186-NO
CCKQSCTTCMPCCW

>ID16187-NO
CCKVLCESCTPCC
>ID16188-NO
CCKVQCESCTPCC
>ID16190-NO
CCKYGWTCLLGCSPCGC
>ID16191-NO
CCKYGWTCVLGCSPCGC
>ID16193-NO
CCLWPACWGCVCCY
>ID16195-NO
CCLWPECGGCVCCYL
>ID16197-NO
CCMTCFGCTPCC
>ID16198-NO
CCNAGFCRFGCTPCCY
>ID16215-NO
CCNCSSKWCRDHSRCCGR
>ID16217-NO
CCNKGKNKGSH
>ID16220-NO
CCNWPCSFGCIPCCY
>ID16221-NO
CCPARMCMAACSCCD
>ID16222-NO
CCPESPPCCHYF
>ID16227-NO
CCPGWELCCEWDDGW
>ID16228-NO
CCPGWELCCEWDDWW
>ID16232-NO
CCPKEWCNRDCSCCT
>ID16234-NO
CCPNKPCCFI
>ID16235-NO
CCPNLFYCCPD
>ID16236-NO
CCPPVACNMGCKPCC
>ID16238-NO
CCPRKIWCCMIP
>ID16241-NO
CCQAACSPWLCLPCC
>ID16242-NO
CCQAACSPWPCLPCC
>ID16245-NO
CCQIVPQCCEWN
>ID16251-NO
CCQVMPQCCEWN
>ID16254-NO
CCRCACCANGGYTCCCA
>ID16258-NO

CCRLLCLSCNPCC
>ID16259-NO
CCRLRRCKTHCHCCVYRP
>ID16264-NO
CCRRGYMGCIPCCF
>ID16268-NO
CCSDSDCNANHPDMCS
>ID16270-NO
CCSICDTTCVYPTCCDDLISD
>ID16272-NO
CCSLRRCEPHCHCCVQRH
>ID16273-NO
CCSNPACRVNNPHVC
>ID16276-NO
CCSNPVCHLEHSNLCTNGG
>ID16278-NO
CCSQDCLVCIPCCPN
>ID16280-NO
CCSQDCRVCIPCCPH
>ID16281-NO
CCSQDCRVCIPCCPN
>ID16283-NO
CCSQDCSVCIPCCP
>ID16288-NO
CCSQDCWVCIPCCPN
>ID16290-NO
CCSRDCLVCIPCCPYGSD
>ID16291-NO
CCSRDCWVCIPCCPNGS
>ID16292-NO
CCSRDCWVCIPCCPNGSA
>ID16293-NO
CCSRHCWVCIPCCPN
>ID16294-NO
CCSRHCWVCIPCCPNGS
>ID16297-NO
CCSRNCAVCIPCCPNWPA
>ID16299-NO
CCSRYCWKCIPCCPY
>ID16302-NO
CCSVSICQSPPVCECCA
>ID16304-NO
CCSWDVCDHPSCTCCG
>ID16305-NO
CCSWDVCDRPSCACC
>ID16307-NO
CCTACRMPPCKCCA
>ID16308-NO
CCTALCSRYHCLPCC
>ID16309-NO
CCTGSCRSGKC

>ID16310-NO
CCTQSCTTCFPCC
>ID16311-NO
CCTVCTTGCVVCC
>ID16313-NO
CCVVCNAGCSGNCCS
>ID16314-NO
CCWNRACTRLVPCSK
>ID16316-NO
CDAPNAPCEKFDNDCCDACMLREKQQPICAV
>ID16317-NO
CDCRGDCFC
>ID16318-NO
CDDSWKC
>ID16319-NO
CDFIF
>ID16320-NO
CDFIM
>ID16324-NO
CDLEGMFCMYDFECCLSECCMGICAFGCT
>ID16325-NO
CDPSRGKNC
>ID16327-NO
CDSDSDITWDQLWDLMK
>ID16328-NO
CDTAVVEGL
>ID16329-NO
CDTRL
>ID16331-NO
CDYVLIC
>ID16334-NO
CEDGGVPSASCRANTEDYRYCNACYLQEVIGK
>ID16335-NO
CEDLICYCRTRGCKRRERLNGTCRKGHLMYMLWCC
>ID16337-NO
CEGVNGRRLR
>ID16338-NO
CEKRGDNLC
>ID16339-NO
CEKRGDSLC
>ID16340-NO
CEKRGDSVC
>ID16341-NO
CELDENNTPMC
>ID16342-NO
CELSLISKC
>ID16344-NO
CEQCNGRCGQ
>ID16345-NO
CERACRNLCREGC
>ID16346-NO

CESYGKPCGIYNDCCNACDPAKKTCT
>ID16347-NO
CETCNGRCVG
>ID16349-NO
CETWRTETTGATGQASSLLSGRLLEQKAASCHNSYIVLCIENSFMTSFSK
>ID16350-NO
CEVCNGRCAL
>ID16351-NO
CEYQLDVE
>ID16353-NO
CFCTKPC
>ID16354-NO
CFDGNHIWC
>ID16357-NO
CFESWVACESPKRCCSHVCLFVCA
>ID16359-NO
CFFDRIKALTKNVTLELLNTITCKLPVTPP
>ID16360-ABP_both
CFFFFKKKKKC
>ID16361-NO
CFFRDCSNMDWYR
>ID16364-ABP_both
CFGGSLGIQYEDKINVGGGLSGDHC
>ID16365-NO
CFIRNCPP
>ID16367-NO
CFITNCPPG
>ID16373-NO
CFPPGTFCSRYLPCCSGRCCSGWCTRRCSPRY
>ID16375-NO
CFPPGVYCTRHLPCCRGRCCSGWCRPRCFPRY
>ID16376-ABP_both
CFQWLRNMRKVR
>ID16377-ABP_both
CFQWQRLMRKVR
>ID16379-ABP_both
CFQWQRRMRKVR
>ID16381-ABP_both
CFQWRRNMRKVR
>ID16383-NO
CFWPNRC
>ID16384-NO
CGAYDLRRRERQSRLRRRERQSR
>ID16385-NO
CGEACGGQCALPC
>ID16386-NO
CGECNGRCVE
>ID16387-NO
CGEGHPC
>ID16422-NO
CGETCVGGTCNTPGCTCSWPVCGHFRWGV

>ID16441-NO
CGETMRC
>ID16442-NO
CGGERGKSC
>ID16444-NO
CGGLF
>ID16445-ABP_pos
CGGLLLFLLKKRKKRKY
>ID16448-NO
CGGTGDSCNEPAGELCCRRLKCVNSRCCPTTDGC
>ID16450-ABP_both
CGGWHRLRC
>ID16455-ABP_both
CGGYCGGWHRLRCTSYRCG
>ID16457-ABP_both
CGGYSGGWHRLRSTSYRCG
>ID16460-NO
CGGYSTYCEVDSECCSDNCVRSYCTLF
>ID16464-NO
CGKRK
>ID16465-NO
CGKRKKKGKLGKKRDP
>ID16466-NO
CGKRKKKGKLGKKRPRSR
>ID16467-NO
CGLIINKNEC
>ID16468-NO
CGLIIQKNEC
>ID16469-NO
CGLIIQRNEC
>ID16470-NO
CGLILQKNEC
>ID16471-NO
CGLMCQGACFDVC
>ID16472-NO
CGLSDSC
>ID16473-NO
CGLSGLGVA
>ID16474-ABP_both
CGNFLKRTCICVKK
>ID16475-NO
CGNKRTRGC
>ID16476-NO
CGNSNPKSC
>ID16477-NO
CGPCFTTDHQMEQKCAECCGGIGKCYGPQCLCNR
>ID16478-NO
CGPCFTTDPYTESKCATCCGGRGKCVGPQCLCNRI
>ID16479-NO
CGQKRTRGC
>ID16480-NO

CGRCNGRCLL
>ID16481-NO
CGRECPRLCQSSC
>ID16482-NO
CGRGDMPSC
>ID16483-NO
CGRGDNLAC
>ID16484-NO
CGRGDNLPC
>ID16485-NO
CGRGDSPDC
>ID16487-NO
CGRPRESGKKRKRKRLKP
>ID16488-NO
CGSKRAWCKEKKDCCCGYNCVYAWYNQQSSCERKWKYLFTGEC
>ID16489-NO
CGSLF
>ID16491-NO
CGSPGWVRC
>ID16492-NO
CGTKRKC
>ID16493-NO
CGTRCVRCQNGPEASCEQPL
>ID16494-NO
CGTRVDHC
>ID16497-NO
CGVGSSC
>ID16498-ABP_both
CGYKYGCMVKVDR
>ID16499-NO
CGYRHGKANCGKG
>ID16500-NO
CGYRHGRANCGRG
>ID16501-NO
CGYSFTDYVLIWVKQSC
>ID16503-NO
CHAEDCHAVGCHAI
>ID16505-NO
CHMDCSKMICCSGICCFYCGLPSCDDT
>ID16506-NO
CHVLWSTRC
>ID16507-NO
CHWSYGLRPG
>ID16509-NO
CIAGGQPCEFHRGYMCCSEHCIIFVCA
>ID16512-NO
CIDGGEICDIFFPNCCSGWCIILVCA
>ID16519-NO
CIEGVLGGC
>ID16522-NO
CIEQFDPCEMIRHTCCVGVCFLMACI

>ID16524-NO
CIGGGDPCEFHRGYTCCSEHCIIWVCA
>ID16525-NO
CIGGGDPCEFHRPYTCCSGYCIVFVCA
>ID16527-NO
CIISRDLISH
>ID16530-NO
CIPDHHGCGLLHHSRYCCNGTCFFVCIP
>ID16532-NO
CIPEGTYCQFNADCCLSQCCWGSCGNPCRFP
>ID16533-NO
CIPFLHPCTFFFPDCCNSICAQFICL
>ID16534-NO
CIPHFDPCDPIRHTCCFGLCLLIACI
>ID16536-NO
CIPQFDPCDMVRHTCCKGLCVLIACSKTA
>ID16539-NO
CIRCNGRCSV
>ID16540-NO
CIRSAVSC
>ID16545-NO
CISLDRSC
>ID16546-NO
CITPGTRCKVPSQCCRGPCKNGRCTPSPSEW
>ID16549-NO
CIVGTPCHVCRSQSKSCNGWLGKQRYCGYC
>ID16551-ABP_both
CKAAFC
>ID16553-NO
CKAAKNK
>ID16554-NO
CKAENELCNIFIQNCCDGTCLLICIQNPQ
>ID16555-NO
CKAESEACNIITQNCCDGKCLFFCIQIPE
>ID16556-NO
CKALSQAC
>ID16559-ABP_both
CKDDQNPHISCDKF
>ID16562-NO
CKFAWATYTSC
>ID16563-NO
CKFFWATYTSC
>ID16564-NO
CKGAGAACSRLMYDCCTGSCRSGKC
>ID16565-NO
CKGAGAKCSRLLYDCCTGSCRSGKC
>ID16566-NO
CKGAGAKCSRLMYDCCTGSCRSGKC
>ID16567-NO
CKGAGAKCSRLMYDCCTGSCRSGKCG
>ID16568-NO

CKGAGAKCSRLPYDCCTGSCRSGAC
>ID16569-NO
CKGAKAR
>ID16571-NO
CKGGRAKDC
>ID16572-NO
CKGKGAACSRLMYDCCTGSCRSGKC
>ID16573-NO
CKGKGACSRLMYKCC
>ID16574-NO
CKGKGAGCDYSHECCSRQCTGRIFQTCN
>ID16576-NO
CKGKGAKASRLMYDAC
>ID16578-NO
CKGKGAKCRKTSYDCCTGSCRSGKC
>ID16579-NO
CKGKGAKCSALMYDCCTGSCRSGKC
>ID16582-NO
CKGKGAKCSR
>ID16589-NO
CKGKGAKCSRLMYDCC
>ID16593-NO
CKGKGAKCSRLMYDCCTGSCRSGKCTRNG
>ID16594-NO
CKGKGAKCSRLMYDCCTGSCRSGKCTRNGLPG
>ID16597-NO
CKGKGAPCTRLMYDCCHGSCSSSKGRC
>ID16599-NO
CKGKGASCHRTSYDCCTGSCNRGKC
>ID16600-NO
CKGKGASCRKTMYDCCRGSCRSGRC
>ID16603-NO
CKGKGASCRRTSYDCCTGSCRSGKCG
>ID16608-NO
CKGKGASCSRTMYNCCTGSCNRGKC
>ID16609-NO
CKGKGQSCSKLMYDCCTGSCSRRGKC
>ID16611-NO
CKGPGAKCLKTMYDCCKYSCSRGRC
>ID16612-NO
CKGPGASCIRIAYNCCKYSCRNGKCS
>ID16614-NO
CKGRGKRCREKQRPSDKPRR
>ID16619-NO
CKIALPYT
>ID16620-NO
CKIALPYTMRCRVLGKC
>ID16621-ABP_both
CKILSKTIKCRIPCGRRKEY
>ID16623-NO
CKITRCPMIPCYISSPDECLWMDWVTEKNINGHQAKFFAC

>ID16624-NO
CKITRCPMIPCYISSPDECLWMDWVTEKNINGHQAKFFACIKRSDGSC
>ID16627-ABP_pos
CKKKC
>ID16628-NO
CKKKKKKKKK
>ID16629-ABP_pos
CKKKRC
>ID16630-ABP_both
CKKKWWWWKKC
>ID16631-ABP_both
CKKLLKLCKKLLKLAG
>ID16636-NO
CKLKGQSCSRLMYDCCSGSCGRSGKC
>ID16641-NO
CKPPGSPCRVSSYNCCSSCKSYNKKCG
>ID16643-NO
CKQADEPCDVFSLECCTGICLGFCTW
>ID16644-NO
CKQADESCNVFSLDCCTGLCLGFCVS
>ID16646-ABP_neg
CKRFRIRVRV
>ID16649-ABP_neg
CKRWWKWIRW
>ID16650-NO
CKSCNGRCLA
>ID16651-ABP_both
CKSIFC
>ID16653-NO
CKSKGAKCDKLMYDCCSGSCSGTVGRC
>ID16654-NO
CKSKGAKCDRLMYDCCSGSCSGTVGRC
>ID16655-NO
CKSKGAKCSKLAYDCCSGSCSGTVGRC
>ID16656-NO
CKSKGAKCSKLLYDCCSGSCSGTVGRC
>ID16657-NO
CKSKGAKCSKLMYDCCSGSCSGAVGRC
>ID16658-NO
CKSKGAKCSKLMYDCCSGSCSGTVGRC
>ID16665-NO
CKSKGSKAARSGKC
>ID16667-NO
CKSPGTPCSRGMRDCCTSCLLYSNKCRRY
>ID16670-NO
CKSTGASCRRTPYDCCTGSCRSGRC
>ID16671-NO
CKSTGASCRRTSYDCCTGSCDRGRC
>ID16678-NO
CKTRVSCGV
>ID16679-NO

CKTYSKYCPADSPCCTPQCVRSYCTLF
>ID16680-NO
CKVCNGRCCG
>ID16684-NO
CKYKAQ
>ID16687-NO
CKYKGAKCSRLLYDCCSGSCSGTVGRC
>ID16689-NO
CKYKWR
>ID16691-NO
CKYLLRWR
>ID16692-NO
CKYRWR
>ID16693-NO
CLAEHETCNIFTQNCCEGVCIFICVQAPE
>ID16694-NO
CLAGSAPCEFHKRSTCCSGHCIIWWCA
>ID16695-NO
CLAGSAPCEFHRGYTCCSGHCLIWVCA
>ID16697-NO
CLAGSARCEFHKPSTCCSGHCIFWWCA
>ID16703-NO
CLASGETCWRDTSCCSFSCTNNVCF
>ID16706-NO
CLDAGEMCDLFNSKCCSGWCIILVCA
>ID16709-NO
CLDAGEVCDIFFPTCCGYCILLFCA
>ID16715-NO
CLDGGEICGILFPSCCSGWCIVLVCA
>ID16717-NO
CLDGGRPKC
>ID16718-NO
CLEFGELCNFFFPTCCGYCVLLVCL
>ID16719-NO
CLEKGVLCDPSAGNCCSGECVLVCL
>ID16723-NO
CLESGSLCFAGYGHSSCCSGACLDYGGLGVGACR
>ID16724-NO
CLFSGEYCWLDTSCCSKSCTNNVCF
>ID16726-NO
CLGFGEACLILYSDCCGYCVGAICL
>ID16727-NO
CLGFGEACLMLYSDCCSYCVALVCL
>ID16729-NO
CLGFGEVCNFFFPNCCSYCVALVCL
>ID16730-NO
CLGGGEVCDIFFPQCCGYCILLFCT
>ID16733-NO
CLGSGELCVRDTSCCSMSCTNNICF
>ID16734-NO
CLGSGEQCVRDTSCCSMSCTNNICF

>ID16735-NO
CLGSGETCWLDSSCCSFSCTNNVCF
>ID16736-NO
CLGSRELCVRDTSCCSMSCTNNICF
>ID16739-NO
CLGSREQSVRDTSCCSMSCTNNICF
>ID16741-NO
CLIQDCPEG
>ID16744-NO
CLITNCPRL
>ID16751-ABP_both
CLLKKKKKLLC
>ID16752-ABP_both
CLLKKLLKKLLKKC
>ID16755-ABP_both
CLMARPNYRCKIFKQC
>ID16758-ABP_both
CLNLKALLAVAKKILC
>ID16760-NO
CLQFGSTCFLGDDDICCSGECFYSGGTFGICS
>ID16761-NO
CLQLFSTC
>ID16763-NO
CLRDGQSCGYDSDCCRYSCCWGYCDLTCLIN
>ID16766-NO
CLRRERQSRLRRERQSR
>ID16768-NO
CLSCNGRCPS
>ID16769-NO
CLSDGK
>ID16770-NO
CLSDGKPVS
>ID16771-NO
CLSDGKRKC
>ID16773-NO
CLSGGEVCDFLFPKCCNYCILLFCS
>ID16774-NO
CLSGSLSC
>ID16775-NO
CLSPGSSCSPTSYNCCRSCNPYSRKC
>ID16778-NO
CLSRYLDQC
>ID16780-NO
CLSYYPSYC
>ID16781-NO
CLTTGETCWLASSCCSFSCTNNICF
>ID16783-NO
CLTTGEYCWLASSCCGYSCTNNVCF
>ID16784-NO
CLTTGEYCWLASSCCSYSCTDNVCF
>ID16786-NO

CLVVHEAAC
>ID16788-ABP_both
CMCIKTTSGIHPKNI
>ID16789-NO
CMEAGSYCGSTTRICCGYCAYSASKNVCDYPSN
>ID16790-NO
CMEMGVKC
>ID16791-NO
CMENSA
>ID16793-NO
CMGYDIHCTDRLPCCFGLECVKTSGYWWYKKTYCRRKS
>ID16794-NO
CMHPEGGCRFSYECCSKICYTPSFTCYPS
>ID16795-NO
CMHWQTGPARTSCIGP
>ID16796-NO
CNAAESSKNC
>ID16797-NO
CNAGDSSKNC
>ID16798-NO
CNAGESSKNC
>ID16799-NO
CNAGESSRNC
>ID16800-NO
CNAGESTKNC
>ID16803-NO
CNDPGGSCTRHYHCCQLYCNKQESVCLENEPAF
>ID16804-NO
CNDRGGGCSQHPHCCGGTCNKLIGVCL
>ID16805-NO
CNEAQEHCTQNPDCCSESCNKFVGRCLSD
>ID16807-ABP_both
CNGPFNIVCSCY
>ID16809-NO
CNGRCVSGCAGRC
>ID16811-NO
CNKTDGDEGVTC
>ID16812-NO
CNNRGGGCSQHPHCCSGTCNKIFGVCL
>ID16816-ABP_neg
CNPLNGADRRTDSFPRFTVI
>ID16818-NO
CNRRTKAGC
>ID16819-NO
CNSKGTPCTNADECCGGKCAYNVWNCIGGGCSKTCGY
>ID16820-NO
CNTATCATQRLANFLVRTSNNLGAILSPTNVGSNTY
>ID16821-NO
CNTVTCATQRLANFLIHSSNNFGAIFSPPSVGS
>ID16826-ABP_both
CPAIQRCC

>ID16828-NO
CPEKFRPMC
>ID16830-NO
CPFWIC
>ID16832-NO
CPHNLTKLC
>ID16833-NO
CPHSKPCLC
>ID16834-NO
CPIDERPMC
>ID16835-NO
CPIEDRPMC
>ID16836-NO
CPIRPMEDC
>ID16837-NO
CPLCNGRCAL
>ID16839-NO
CPNGRC
>ID16840-NO
CPNTGELCDVVEQNCCYTYCFIVVCL
>ID16842-NO
CPRECESIC
>ID16843-NO
CPRGCLAVCVSQC
>ID16844-NO
CPRGSRC
>ID16845-NO
CPSDLKDAC
>ID16846-NO
CPTCNGRCVR
>ID16848-NO
CPWEPWC
>ID16852-NO
CQGRGASCRKTMYNCCSGSCRSGRC
>ID16854-NO
CQGRGASCRRTSYDCCTGSCRSGRC
>ID16858-NO
CQLAAVC
>ID16859-NO
CQLTGTHGAGC
>ID16860-NO
CQNHHAKHGKVC
>ID16863-NO
CQRANFVCDAFHHAAVCCEGVCVLVCAW
>ID16864-NO
CQSCNGRCVR
>ID16866-NO
CRAEGTYCENDSQCCLNECCWGGCGHPCRHP
>ID16870-NO
CRCCNGRCSP
>ID16871-NO

CRCEQTCGTCVPCC
>ID16873-NO
CREAGRKAC
>ID16874-NO
CREKAKKLFKKILKKL
>ID16876-NO
CRESLKNC
>ID16877-NO
CRFRFKCCKK
>ID16878-NO
CRFRWKCCKK
>ID16879-NO
CRGDAGINC
>ID16880-NO
CRGDCF
>ID16881-NO
CRGDCGGKWCFRVCYRGICYRRCR
>ID16882-NO
CRGDGWC
>ID16883-NO
CRGDHAANC
>ID16884-NO
CRGDHAGDC
>ID16885-NO
CRGDHGVEC
>ID16886-NO
CRGDK
>ID16887-NO
CRGDKGENC
>ID16888-NO
CRGDKGPDC
>ID16889-NO
CRGDKGPEC
>ID16890-NO
CRGDKHADC
>ID16891-NO
CRGDKTTNC
>ID16893-NO
CRGDRGPDC
>ID16894-NO
CRGDSAC
>ID16896-NO
CRGRRST
>ID16897-NO
CRGSGAGRC
>ID16914-ABP_pos
CRKKKC
>ID16916-NO
CRKKRRRESRKKRRRES
>ID16917-NO
CRKKRRRESRRARRSPRHL

>ID16919-NO
CRLEGSSCRRSYQCCHKSCCIRECKFPCRWD
>ID16921-NO
CRLGAESCDVISQNCCQGTCVFFCLP
>ID16923-NO
CRLTGGKGVGC
>ID16924-NO
CRMTRNKPC
>ID16925-NO
CRNCNGRCEG
>ID16926-NO
CRPGGMICGFPKPGPYCCSGWCFVVCL
>ID16927-NO
CRPPGMVCGFPKPGPYCCSGWCFAVCLPV
>ID16930-NO
CRPSGSPCGVTSICCGRCYRGKCT
>ID16931-NO
CRQIKIWFPNRRMKWKKC
>ID16933-NO
CRRHWGFEFC
>ID16934-ABP_both
CRRLCYKQRCVTYC
>ID16935-ABP_pos
CRRLCYKQRCVTYCRGR
>ID16936-NO
CRRRRRRRRR
>ID16937-NO
CRRTNWQGAGC
>ID16939-ABP_both
CRRWQWRC
>ID16942-ABP_both
CRRWWRFC
>ID16947-NO
CRSRKG
>ID16948-NO
CRSSGSPCGVTGICCGRCYRGKCT
>ID16953-NO
CRTCNGRCQV
>ID16954-NO
CRTEGMSCEENQQCCWRSCCRGECEAPCRFGP
>ID16956-NO
CRTTRGTKC
>ID16957-NO
CRTWNAPCSFTSQCCFGKCAHHRCIAW
>ID16959-NO
CRVENKCPHTVCCDRSRCSCKLIRTRPLMYHVCVC
>ID16962-NO
CRVSRQNKC
>ID16964-NO
CRWLQHSCLQ
>ID16965-NO

CRWRFKCCKK
>ID16967-NO
CRWRWKCG
>ID16968-NO
CRWRWKCGCKK
>ID16972-NO
CSCADMTDKECLYFCHQDVIW
>ID16973-NO
CSCCNGRCGD
>ID16975-NO
CSCNDINDKECMYFCHQDVIWDEP
>ID16978-NO
CSCSSLMDKECVYFCHLDIIWVNTPEHIVP
>ID16979-NO
CSCTDMSDLECMNFCHKDVIWINRN
>ID16980-NO
CSDEGASCEKKSDCCFLSCCWSVCDRPCRLVP
>ID16981-NO
CSDGQHYWC
>ID16983-NO
CSDSWHYWC
>ID16984-NO
CSDWQHPWC
>ID16985-NO
CSDYNHHWC
>ID16988-NO
CSFTDYVLIWVKC
>ID16990-NO
CSGGKVLDC
>ID16991-ABP_both
CSGIIKQTCTCYRK
>ID16992-NO
CSGLF
>ID16993-NO
CSGRGDSLC
>ID16994-NO
CSIPPEVKFNPFVYLI
>ID16995-NO
CSKCNGRCGH
>ID16996-NO
CSMSAKKKC
>ID16997-NO
CSNFGSDCIPATHDCCSGECFGFEDMGLCT
>ID16998-NO
CSNIDARAC
>ID17000-NO
CSNLSTCVLGTYSKDLNNFHTFSGIGFGAETP
>ID17002-NO
CSNLSTCVLSAYWKDLNNYHRYSGMGFGPETP
>ID17003-NO
CSNRDARRC

>ID17006-NO
CSPGGEVCTRHSPCCTGFLCNHIGGMCHP
>ID17007-NO
CSPGGEVCTSKSPCCTGFLCSHIGGMCHH
>ID17009-NO
CSPPGSYCFGPAACCSNFCSTMSDVCQENWSG
>ID17010-NO
CSPQSQPMC
>ID17011-NO
CSRLMYDCCTGSC
>ID17012-NO
CSRPRRSEC
>ID17013-NO
CSRPRRSVC
>ID17014-NO
CSRPRRSWC
>ID17015-NO
CSRRARRSPRESGKKRKRKR
>ID17016-NO
CSRRARRSPRHLGSG
>ID17019-NO
CSSLF
>ID17020-NO
CSSTMRC
>ID17021-NO
CSTCLDKP
>ID17022-NO
CSVANSC
>ID17024-NO
CSWPVCTRNGLPVCGETCVGGTCNTPGC
>ID17025-NO
CTAMRNTDC
>ID17026-NO
CTAPGGACYAAYTCCSNACNLNTKKCVLS
>ID17029-NO
CTCKDMTDKECLYFCHQDIIW
>ID17030-NO
CTCRVLDQELSTKALFR
>ID17035-NO
CTDFPRSFC
>ID17036-NO
CTDPGGACGNPGHCCSKFCITTSSTCN
>ID17041-NO
CTDYVLIWC
>ID17043-NO
CTDYVRC
>ID17044-NO
CTECNGRCQ
>ID17048-NO
CTGGATCCGGRTGRCAVARYTTRTANCC
>ID17049-NO

CTGRGDALC
>ID17051-NO
CTHAYEACDATTNCCYMTCNLPTRKCRGPLF
>ID17052-NO
CTHPGGACGFYYHCCSNYCITISSTCN
>ID17053-NO
CTHPGGACGGHHHCCSLFCNTAANACN
>ID17054-NO
CTHPGGACGGHHHCCSLSCNTAANACN
>ID17056-NO
CTHPGGACGGHHHCCSQSCNTAANSCN
>ID17057-NO
CTHPGGACGGHHHCCSQSCNTAATTCN
>ID17059-NO
CTPAGDACDATTECCILFCNLATKECQVPAFP
>ID17060-NO
CTPAGDACDATTECCILFCNLATKKCQVPTFP
>ID17061-NO
CTPAGDACDATTKCCIPFCNLATKKCQVPTFP
>ID17064-NO
CTPAGKACDATATCCVLFCNLVTNKCQVPRFP
>ID17070-NO
CTPGGEACDATTNCCFLTCNLATNKCRSPNFP
>ID17071-NO
CTPPDGACGFHYHCCSKFCITVSSTCN
>ID17072-NO
CTPPEGGCLSSYECCSKICWRPRCYPS
>ID17073-NO
CTPPGGACGVYYHCCSNYCITISSTCR
>ID17074-NO
CTPPGGSCGGHAHCCSKSCNIMASTCND
>ID17075-NO
CTPPGGYCYHPDPCCSQVCNFPRKHCL
>ID17077-NO
CTPPSGYCYHPYYCCSRACNLTRKRCL
>ID17079-NO
CTPRHGVCFYSYFCCSKACNPSSKRCL
>ID17080-NO
CTPRNGVCFYSYFCCSRACNPSTKRCL
>ID17081-NO
CTPRNGYCYYRYFCCSRACNLTIKRCL
>ID17082-NO
CTPSGGACGFYYHCCSNYCITISSTCR
>ID17083-NO
CTPSGGACYVASTCCSNACNLNSNKCV
>ID17084-NO
CTPSGGACYYDYFCCSMTCNFNSKSCV
>ID17086-NO
CTPSPPFSHC
>ID17088-NO
CTQDRQHPC

>ID17089-NO
CTQSGELCDVIDPDCCNNFCIIFFCI
>ID17091-NO
CTQTNGACYHRDTCCSKSCNLTINRCLAS
>ID17092-ABP_both
CTRPNYNKRKRIHIG
>ID17093-NO
CTSAVLQCN
>ID17094-NO
CTSEGYSCSSDSNCCKNVCCWNVCESHCGHH
>ID17096-NO
CTSPDGACNTPPQCCSKYCISISTTCN
>ID17099-NO
CTTGPCCRQCKLKPAGTTCWKTSLTSHYCTGKSCDCPLYPG
>ID17101-NO
CTTHWGFTLC
>ID17109-NO
CVDPGEFCGPGFGDCCTGFCLLVCI
>ID17111-NO
CVEDGDFCGPGYEECCSGFCLYVCI
>ID17114-NO
CVFSLFKKCN
>ID17115-NO
CVGIW
>ID17121-ABP_both
CVHGGTNTARTACIAG
>ID17122-ABP_both
CVHGMTNTARTACIAP
>ID17133-NO
CVISAAWFHKIRCKLTGNC
>ID17134-NO
CVISPGWDHKVKSNPRDLVCRRPPMGYLH
>ID17135-ABP_neg
CVKCKCKCGSGVKVKVKVC
>ID17136-ABP_both
CVKGGKKYKRQGKGHRMRRYRNNH
>ID17137-NO
CVKRGLKLRHVRPRVTRDV
>ID17138-NO
CVKRGLKLRHVRPRVTRMDV
>ID17139-NO
CVKYLDPCDMLRHTCCFGLCVLIACI
>ID17140-NO
CVLCNGRCWS
>ID17141-NO
CVLNGRMEC
>ID17145-NO
CVMVRDGDC
>ID17146-NO
CVNHPAFAC
>ID17147-NO

CVPCNGRCHE
>ID17148-NO
CVPELGHEC
>ID17149-NO
CVPHEGPCNWLTQNCCSGYNCIIFFCL
>ID17154-NO
CVPYEGPCNWLTQNCCDELCVFFCL
>ID17156-NO
CVQWSLLRGYQPC
>ID17157-NO
CVRIRPC
>ID17158-ABP_both
CVRPGRVC
>ID17159-ABP_both
CVRVRPGRVRVC
>ID17160-ABP_both
CVRVRVRPGRVRVRVC
>ID17162-ABP_both
CVRVRVRVRPGRVRVRVRVC
>ID17163-ABP_both
CVRVRVRVRVRPGRVRVRVRVRVC
>ID17164-NO
CVSGPRC
>ID17166-NO
CVSNPRWKC
>ID17168-NO
CVTCNGRCRV
>ID17169-NO
CVTPALR
>ID17170-NO
CVVSADWNYKIRCKLTGNC
>ID17171-NO
CVWCNGRCGL
>ID17173-NO
CWGCNGRCRM
>ID17174-NO
CWGTGLC
>ID17175-NO
CWKKK
>ID17176-ABP_both
CWKKKC
>ID17177-NO
CWKKKKKKKK
>ID17179-NO
CWKKKKKKKKKKKKKKKKKK
>ID17180-ABP_both
CWKKKKKKWWC
>ID17181-NO
CWLCNGRCGR
>ID17182-NO
CWLPVY

>ID17183-NO
CWRKFYC
>ID17185-ABP_both
CWTASIPPRPC
>ID17186-ABP_both
CWTKSIPPKPC
>ID17188-ABP_both
CWTKSIPPKPG
>ID17190-ABP_both
CWTVSIPPRPC
>ID17193-ABP_both
CWWKKKC
>ID17194-ABP_both
CWWKKKKC
>ID17195-ABP_both
CWWKKKKKWWC
>ID17196-ABP_both
CWWKKKKKWWWC
>ID17198-NO
CWWRLEGC
>ID17200-ABP_both
CWWWKKKKKC
>ID17202-ABP_both
CWWWWKKKKKKC
>ID17215-NO
CYDGGTGCDSGNQCCSGWCIFVCL
>ID17217-NO
CYDGGTSCDSGIQCCSGWCIFVCL
>ID17219-NO
CYDSGTSCNTGNQCCSGWCIFVCL
>ID17221-NO
CYDSWHYWC
>ID17224-NO
CYFQNCPKG
>ID17225-NO
CYFQNCPR
>ID17226-NO
CYFRNCPIG
>ID17228-NO
CYGFGEACLVLYTDCCGYCVLAVCL
>ID17232-NO
CYIQNCPIG
>ID17234-NO
CYIQNCPRGG
>ID17236-NO
CYISNCPIG
>ID17238-NO
CYLGVSNC
>ID17239-NO
CYLVNVDC
>ID17241-NO

CYPADPC
>ID17242-NO
CYSYFLAC
>ID17243-NO
CYTADPC
>ID17245-NO
CYVELHC
>ID17248-NO
DAADFADPYSFLYRLIQAEARKMSGCSN
>ID17250-NO
DAADVKPVARHNDGPGRDPAPCCQHPIETCC
>ID17251-NO
DAADVKPVARQNEGPGRDPAPCCQHPIETCC
>ID17259-NO
DAATATRGRSAASRPTQRPRAPARSASRPRRPVE
>ID17260-NO
DACEQAAIQCVESACESLCTEGEDRTGCYMYIYSNCPPYV
>ID17262-NO
DADEGDTLANSSDLLKELLGTGDNRAKDSHQQESTNNDEDSTS
>ID17263-NO
DADEGDTLANSSDLLKELLGTGDNRAREGRHQESTDNDDNMS
>ID17264-NO
DADIAVWAPPVNAQN
>ID17266-NO
DAECEICKFVIQQVEAFIESNHSQAEIQKELNKLCSSVPSITQTCLSIAR
>ID17267-NO
DAEDGDALANSSDLLKELLGTGDDRDRENHHQEGGDSDEGVS
>ID17268-NO
DAEDLARYYSALRHYINLITRQRY
>ID17269-NO
DAEEDDGLGNSSNLLKELLGAGDQREGSLHQEGSDAEDVS
>ID17270-NO
DAEEPSAFMTRL
>ID17271-NO
DAEEVRESAETLHEISVY
>ID17273-NO
DAEFRHDSGYQVHHQKLVFFAEDVGSNK
>ID17277-NO
DAEVVSTESDVIVTCEPCMNPACGPNYGKC
>ID17278-NO
DAFSPPEASLTGGQSLS
>ID17279-NO
DAGDYGDPYSFLYRLIQAEARKMSGCSN
>ID17280-NO
DAGYGQISH
>ID17281-ABP_both
DAHGLLKRIKTLL
>ID17283-ABP_both
DAHRAGLQFPVGRVHRLLRK
>ID17284-NO
DAHVLVPRTP

>ID17290-NO
DAIWNLLRQAQEKFGKDKSPK
>ID17294-NO
DALSPPAAGLGADHSFT
>ID17297-NO
DAPGHPGKHYLQVNVPSDVRTIGVAGGGVQQCFRVTPGAWNDTRALVSNG
>ID17299-ABP_both
DAPRIKKIVQKKLAG
>ID17303-NO
DAQSAPLRVY
>ID17306-NO
DARTAPLRLRF
>ID17307-NO
DARTPALRLRF
>ID17309-NO
DASFHSWG
>ID17310-NO
DASFSSWG
>ID17312-NO
DASSSNENNSRPPFAPRL
>ID17313-ABP_neg
DATPHAALFFTVKDHTAGDN
>ID17316-NO
DAYPSGA
>ID17317-NO
DAYPSGAW
>ID17318-NO
DAYSAFSF
>ID17321-NO
DCANIFR
>ID17322-NO
DCANIFRQDGLLNNCR
>ID17324-NO
DCCDPKEPCCFI
>ID17326-NO
DCCEQGWCDGGCDCCQ
>ID17327-NO
DCCGVKLEMCHPCLCDNSCKKSGK
>ID17331-NO
DCCPAKLLCCNP
>ID17332-NO
DCCPAKMFCCQW
>ID17333-NO
DCCPANLLCCNP
>ID17336-NO
DCCPSKLLCCNP
>ID17338-NO
DCCRRNFLCC
>ID17341-NO
DCCSMSACVPPPACECC
>ID17342-NO

DCCVMPWCDGACDCCVSS
>ID17347-NO
DCGEQGQGCYTRPCCPGLHCAAGATGGGSCQP
>ID17348-NO
DCGGQGEGCYTQPCCPGLRCRGGGTGGGACQL
>ID17349-NO
DCGGQGEGCYTQPCCPGLRCRGGGTGGGSCQP
>ID17352-NO
DCGPWCWGQNKCCPDESCRSLHESCT
>ID17353-NO
DCGRCGLGQICDAGACRPSTMM
>ID17354-NO
DCGRCPLGQYCDAEAGMCKPTLIM
>ID17355-NO
DCGTIWHYCGTDQSECCEGWKCSRQLCKYVIDW
>ID17358-NO
DCIPGGENCDVFRPYRCCSGYCILLLCA
>ID17359-NO
DCKHPVGPYTDSCFTDCVSGKYGYNYESAFCSRDETGTCKICCCELINE
>ID17360-NO
DCKHQNDSCAEEGEECCSDLRCMTSGAGAICVT
>ID17365-NO
DCLGQWASCEPKNSKCCPNYACTWKYPWCRYRA
>ID17366-NO
DCLGWFKGCDPDNDKCCEGYKCNRRDKWCKYKLW
>ID17368-NO
DCLKFGWKCNPRNDKCCSGLKCGSNHNWCKLHI
>ID17369-NO
DCLPCGHDVCC
>ID17370-NO
DCLPDWFHYEGHCYRVFDEPKKWADAEKFC
>ID17372-NO
DCLPGWSSHEGHCYKVFNQEMYWADAEKFC
>ID17375-NO
DCLSGRYRGSCAVWHRKKCVDICQREGRTSGHCSPSLKCWCEGC
>ID17376-NO
DCPPWCPTSHCNAGTC
>ID17378-NO
DCPSDWSPYEGHCYKHFIKWMNNEDAERFC
>ID17380-NO
DCPTRCPTTCANGWECCKGYPCVNKACSGCTHGK
>ID17386-NO
DCQDSGVVCGFPKPEPHCCSGWCLFVCA
>ID17387-NO
DCQEKWDFCPAPFFGSRYCCFGLFCTLFFCA
>ID17388-NO
DCQEKWDYCPVPFLGSRYCCDGFICPSFFCA
>ID17390-NO
DCQGEWEFCIVPVLGFVYCCPWLICGPFVCVDI
>ID17393-NO
DCRALGEYCGLPYVHNSRCCSQLCGFICVPESP

>ID17395-NO
DCRGYDAPCSSGAPCCDWWTCSARTNRCF
>ID17396-NO
DCRWRWKCCKK
>ID17409-NO
DCTRMFGACRRDSDCCPHLGCKPTSKYCAWDGTI
>ID17410-NO
DCTSWFGRCTVNSECCSNSCDQTYCELYAFPS
>ID17413-NO
DCVAGGHFCGFPKIGGPCCSGWCFFVCA
>ID17414-NO
DCVGENGRCRDWYNDCCDGFYCSCRQPPYCICRNNN
>ID17416-NO
DCVKAGTACGFPKPEPACCSSWCIFVCT
>ID17417-NO
DCVRFWGKCSQTSDCCPHLACKSKWPRNICVWDGSV
>ID17418-NO
DCWPQYWFCGLQRGCCPGTTCFFLCF
>ID17420-ABP_both
DDALKHLLKHLLKHL
>ID17421-ABP_both
DDALKKLLKKLLKKL
>ID17422-ABP_both
DDALRHLLRHLLRHL
>ID17423-NO
DDCAGWMESCSSKPCCAGRKCFSEWYCKLVVDQN
>ID17425-NO
DDCCPDPACRQNHPEICPS
>ID17426-NO
DDCCPDPACRQNHPELCST
>ID17427-NO
DDCCPNPPCKASNPDLCDWRS
>ID17428-NO
DDCEPPGNFCGMIKIGPPCCSGWCFFACA
>ID17430-NO
DDCGTLFSGCDTSKDCCEGYVCHLWCKYK
>ID17433-NO
DDCLGMFSSCDPDNDKCCEGRKCNRKDKWCKYVL
>ID17434-NO
DDCLGMFSSCDPKNDKCCPNRVCRSRDQWCKYKLW
>ID17436-NO
DDCRPEAAYCEYNEQCCIDKCCQASCSDACRTP
>ID17437-NO
DDCTTYCYGVHCCPPAFKCAASGCVRNN
>ID17438-NO
DDCTTYCYGVHCCPPAFKCAASPSCKQT
>ID17439-NO
DDDCEPPGNFCGMIKIGPPCCSGWCFFACA
>ID17443-NO
DDDDDNDKIPDDRDN
>ID17450-NO

DDDDKRAGSPSGGPFCALARQPLTGSPPNERAFFCSSRDV
>ID17451-NO
DDDDLEGFSE
>ID17460-ABP_both
DDDKTEEEDDKENETTKVVE
>ID17462-NO
DDDQVEVQQEVKRGFLSTVKNLATNVAGTVIDTLKCKVTGGCRT
>ID17464-NO
DDECEPPGDFCGFFKIGPPCCSGWCFLWCA
>ID17479-NO
DDEPRSYFV
>ID17481-NO
DDETTFPCNSGRCACLPEDSHSYTCQSP
>ID17483-NO
DDEVEIIVL
>ID17487-ABP_both
DDGDKGGLKGKNDFFQEF
>ID17489-NO
DDGIEMTEEEVKRGILDLVTHVAKNLAAQLLDKLKCKMTGC
>ID17490-ABP_pos
DDGIFIAFIAINIILTVIFY
>ID17491-NO
DDGLALICHAC
>ID17492-NO
DDGPYKMEHFRWGSPGKD
>ID17495-NO
DDGSYRMEHFRWGTPRKG
>ID17497-NO
DDGVEITEEEVKRGLMDTVKNVAKNLAGHMLDKLKCKITGSC
>ID17501-NO
DDKRRQI
>ID17504-NO
DDLHPFNENNMYYGCKGLSNSNKFED
>ID17505-NO
DDMSVISELICTSPLDFIN
>ID17506-NO
DDMTMKPTPPPQYPLNLQGGGGGGSGDGFGFAVQGHQKVWTSDNGRHEIG
>ID17507-NO
DDNFLRF
>ID17509-NO
DDPDENEANEGEVKWKK
>ID17510-ABP_both
DDPDKGGLKWKNDFFQEF
>ID17511-ABP_both
DDPDKGMLKGKNDFFQEF
>ID17512-ABP_both
DDPDKGMLKWKNDFFQEF
>ID17516-NO
DDRCERMCQHCHDRREKKQCMKGCR
>ID17517-ABP_pos
DDRFFFYWLMCIWCTFVMSH

>ID17518-ABP_pos
DDRGAAMFAVLFNAFSSLLE
>ID17521-NO
DDSSNVETFDTEEEATTDSTLRV
>ID17522-NO
DDSSPGFFLKITKNVPRL
>ID17523-NO
DDTGHDFEDTGEAM
>ID17524-NO
DDTNKFLRLS
>ID17529-NO
DDWSQFQGSW
>ID17534-NO
DEAIRCTGTKDCYIPCRYITGCFNSRCINKSCKCYGCT
>ID17535-NO
DECANAAAQCSITLCNLYCGPLIEICELTVMQNCEPPFS
>ID17538-NO
DECCEPQWCDGACDCCS
>ID17541-NO
DECCSDPRCAVKHQDLC
>ID17542-NO
DECCSNPACRLNNPHACRRR
>ID17544-NO
DECCSNPACRLNNPHVCRRR
>ID17545-NO
DECCSNPACRVNNPHVCRRR
>ID17546-NO
DECCSNPPCAQAHPEVC
>ID17548-NO
DECCSNPSCAQTHPEVC
>ID17549-NO
DECCSRPPCRVNNPHVCRRR
>ID17550-NO
DECFSPGTFCGFKPGLCCSARCFSLFCISLEF
>ID17551-NO
DECFSPGTFCGIKPGLCCSARCLSFFCISLEF
>ID17552-NO
DECFSPGTFCGIKPGLCCSAWCYSFFCLTLTF
>ID17560-NO
DECSAPGAFCLIRPGLCCSEFCFFACF
>ID17562-NO
DECYPPGTFCGIKPGLCCSAICLSFVCISFDF
>ID17563-NO
DECYPPGTFCGIKPGLCCSERCFPFVCLSLEF
>ID17570-NO
DEEDLLDLKCSSLHAAAPR
>ID17573-NO
DEEKRENEDEENQEDDEQSEMRRGLRSKIWLWVLLMIWQESNKFKKM
>ID17574-NO
DEERSFHPARPSRSLRSNFIRF
>ID17577-NO

DEGCLPDDSSRT
>ID17578-NO
DEGGTQYTPRL
>ID17579-NO
DEGHDPVHESPVDTA
>ID17580-NO
DEGHKMLYF
>ID17582-NO
DEGPKSDCKP
>ID17583-NO
DEGPYKMEHFRWGSPAKD
>ID17585-NO
DEIDRAGMGFA
>ID17586-NO
DEIDRSGFGFA
>ID17588-ABP_both
DEKIYLIKVADVDQR
>ID17589-ABP_both
DEKIYLKKVADKDQK
>ID17593-NO
DELQDKIHPFAQTQSLVYPFPGPIPNS
>ID17595-NO
DENGCCWNPSCPRPRCT
>ID17598-NO
DEPEYAEAIREYQLKYGKI
>ID17601-NO
DERCTIIIHPGSPCDPSDCVQYCYAEYNGVGKCIASKPGRSANCMCTYNC
>ID17604-NO
DETPGFFIKLSKSVPRI
>ID17607-NO
DEVKIVL
>ID17608-NO
DEVKIVLD
>ID17618-NO
DFAFNPRL
>ID17619-NO
DFAFSPRL
>ID17621-NO
DFDDYGHMRF
>ID17622-NO
DFDEIDR
>ID17624-NO
DFDEIDRSG
>ID17625-NO
DFDEIDRSGFA
>ID17626-NO
DFDEIDRSGFG
>ID17627-NO
DFDEIDRSGFGF
>ID17628-NO
DFDEIDRSGFGFA

>ID17629-NO
DFDEIDRSGFGFV
>ID17630-NO
DFDEIDRSSFA
>ID17631-NO
DFDEIDRSSFG
>ID17632-NO
DFDEIDRSSFGF
>ID17633-NO
DFDEIDRSSFGFA
>ID17634-NO
DFDEIDRSSFGFH
>ID17635-NO
DFDEIDRSSFGFN
>ID17636-NO
DFDEIDRSSFGFV
>ID17638-NO
DFDGAMPGVLRFG
>ID17642-NO
DFDTLRCMLGRVYQRCWQV
>ID17648-ABP_both
DFGCARGMIFVCMRRCARMYPGSTGYCQGFRCMCDTHIPIRRPPFIMG
>ID17649-NO
DFHINQ
>ID17651-NO
DFKCPSEWYAYDQHCYRIIN
>ID17652-NO
DFKLFAVTIKYR
>ID17653-NO
DFKLFAVYIKYR
>ID17657-NO
DFPLSKEYESCVRPRKCKPPLKCNKAQICVDPNKGW
>ID17659-NO
DFRGVVDSCCRKSCSFSTLRAYCDS
>ID17660-NO
DFSFNPRL
>ID17662-NO
DFYKRFVPNCNYKFSLANCFGKERYMNWRSPDAVYHLAK
>ID17664-NO
DGAETPGAAASLWFGPRV
>ID17671-NO
DGCCSDPACSVNHPDICGG
>ID17674-NO
DGCPPHPVPGMHPCMCTNTC
>ID17675-NO
DGCSNAGAFCGIHPGLCCSEICIVWCT
>ID17678-NO
DGCSSGGTFCGIHPGLCCSEFCFLWCITFID
>ID17680-NO
DGCSSGGTFCGIRPGLCCSEFCFLWCITFID
>ID17682-NO

DGCYNAGTFCGIRPGLCCSEFCFLWCITFVDS
>ID17684-NO
DGDMSGEGKGMWFGPRL
>ID17685-NO
DGECGGFWWKCGRGKPPCCKGYACSKTWGWCAVEAP
>ID17687-NO
DGGDVMSGGEGGEMTAMADAIKYLQGLDKVYGQAARPRF
>ID17688-NO
DGGEPAAPLWFGPRV
>ID17689-NO
DGGRNFLRF
>ID17690-NO
DGGWSHWSPWSSC
>ID17693-NO
DGIWKLLSKAQEKFGKNKSR
>ID17694-NO
DGKEDLIWKLLSKAQEKFGKNKSR
>ID17696-NO
DGNRNFLRF
>ID17698-NO
DGPIYLK
>ID17699-NO
DGPYSFGL
>ID17700-NO
DGRCCHPACAKHFNC
>ID17703-NO
DGRELCLDPKENWVQRVVEKFLK
>ID17704-ABP_both
DGRGKQGGKVRAKAKTRSS
>ID17705-NO
DGRKICLDPDAPRIKKIVQKKL
>ID17707-NO
DGRNFLRF
>ID17708-NO
DGRTPALRLRF
>ID17709-NO
DGSSNLRGAW
>ID17711-NO
DGSYKMKHFRWSGPPAS
>ID17714-NO
DGVKLCDVPSGTWSGHCGSSSKCSQQCKDREHFAYGGACHYQFPSVKCFC
>ID17767-NO
DGVLNLYPFPRV
>ID17770-NO
DGYRPLQF
>ID17771-NO
DGYTPRL
>ID17774-NO
DHHDGHLGGHQTGHQGGQQGGHLGGQQGGHLGGHQGGQPGGHLGGHQGGI
>ID17775-NO
DHHHDHGHDDHEHEELTLEKIKEKIKDYADKTPVDQLTERVQAGRDYLLG

>ID17777-NO
DHLPHVYSPRL
>ID17779-NO
DHMSHDVYSPRL
>ID17780-ABP_both
DHNNEIVKIQSLLRANKARDDYK
>ID17781-NO
DHPFL
>ID17782-NO
DHPFLF
>ID17783-NO
DHVPFLRF
>ID17784-NO
DHYICVRSGGQCLYSACPIYTKIQGTCYHGKAKCC
>ID17791-NO
DICDIAIAQCSLTLCQDCENTPICELAVKGSCPPPWS
>ID17792-NO
DICLSTPDVKSKTCPPG
>ID17793-NO
DICNAYF
>ID17795-NO
DIDFSTCARMDVPILKKAAQGLCITSCSMQNCGTGSCKKRSGRPTCVCYR
>ID17796-NO
DIDITGCSACKYAAGQVCTIGCSAAGGFICGLLGITIPVAGLSCLGFVEI
>ID17800-NO
DIFKLVIDHISMKARKK
>ID17801-NO
DIGDLLEGKD
>ID17802-ABP_both
DIGKYCGYAHALN
>ID17803-NO
DIGTKILGGVKTALKGALKELASTYVN
>ID17804-NO
DIGYY
>ID17805-NO
DIIIIFPPFG
>ID17806-NO
DIIIIVGG
>ID17807-NO
DIKQME
>ID17809-NO
DINFPICRFCCQCCNKPSCGICCEE
>ID17810-NO
DINGGGATLPQALYQTSGVL
>ID17813-NO
DIPEVVVSLAWDESLAPKHPGSRKNMDCYCRIPACIAGERRYGTCIYQGR
>ID17815-NO
DIRHRINNSIWRDIFLKRK
>ID17824-ABP_both
DKERPICSNTFRGRKC
>ID17826-ABP_both

DKGRYLEAPTRPRPERNRK
>ID17830-NO
DKIHP
>ID17832-NO
DKIYPSFQPQPLIYP
>ID17856-NO
DKLMYD
>ID17857-ABP_both
DKPKKKPPPPAGPPPPPPPPPGPPPPGP
>ID17860-NO
DKPTAFVSVYLKTAL
>ID17864-NO
DKRCSIIIDLSPCYPIECRLSCITERNGDGECVVSKVGSTPNCLCTYDC
>ID17865-NO
DKRLPYFFKHLFSNRTK
>ID17868-NO
DKTRYFKWPPRCGEEKNMRLSGGLKE
>ID17869-NO
DKVGINY
>ID17870-NO
DKVGINYW
>ID17871-ABP_both
DKVKDYISSLCNKAS
>ID17877-NO
DKYLFGL
>ID17880-NO
DLCEQSALQCNEQGCHNFCSPEDKPGCLGMVWNPELCP
>ID17881-NO
DLDSVLDPSIF
>ID17882-NO
DLDVNVFNR
>ID17885-NO
DLELQKIAEKFSGTRRG
>ID17891-NO
DLFQFGKMIECANKGSRPSLDYMNYGCYCGK
>ID17893-NO
DLGWKLLSKAQEKFGKNKSR
>ID17895-NO
DLIGKLLSKAQEKFGKNKSR
>ID17896-NO
DLIWGLLSKAQEKFGKNKSR
>ID17898-NO
DLIWKLLSAAQEKFGKNKSR
>ID17900-NO
DLIWKLLSGAQEKFGKNKSR
>ID17901-NO
DLIWKLLSKAQEAFGKNKSR
>ID17906-NO
DLIWKLLSKAQEKFGK
>ID17908-NO
DLIWKLLSKAQEKFGKNK

>ID17912-NO
DLIWKLLSKAQEKFGKNKSRKGLKKMRWQWRRCKF
>ID17913-NO
DLIWKLLSKAQEKFGKNKSRKRSKNKGFKEQAKSLLKWILD
>ID17915-ABP_both
DLIWKLLVKAQEKFGRGKPSKRVKKMRRQWQACKSSHHHHHH
>ID17917-ABP_both
DLKGTFAALSELHCDKLHVDPENFKL
>ID17920-NO
DLLASLTHEQKQLIMSQLLPELLSELSNAEDHLHPMRDRDYAGWMDF
>ID17924-NO
DLLTTIKRVKESMKRRT
>ID17927-NO
DLPKVDTALK
>ID17928-NO
DLPMHPM
>ID17932-NO
DLQGK
>ID17934-NO
DLQRRCVIALVHKMRCRVTGRC
>ID17936-ABP_both
DLRGACAAAHAL
>ID17940-NO
DLRPDNAKA
>ID17948-NO
DLTDY
>ID17950-NO
DLVCPDNPDNCIQQCVSKGAQGGYCTNEKCTCYEKIPSATKRVRIVA
>ID17951-ABP_both
DLVELAKLEADKMSK
>ID17952-ABP_both
DLVELAMLEADRMSR
>ID17955-NO
DLWIRETLTSPKSLTG
>ID17956-NO
DLWSWGQMIQETGLLPSYTTY
>ID17960-NO
DMCNGYF
>ID17963-NO
DMIPAQK
>ID17965-NO
DMPKQLLAPWYY
>ID17966-ABP_both
DMQSLFKQYFQKMTK
>ID17967-NO
DMSPPWH
>ID17970-NO
DMYEIKGFKSAHGRPRVCPPGEQCPIWV
>ID17971-NO
DMYEIKQYKTAHGRPPICAPGEQCPIWV
>ID17976-NO

DNFLRF
>ID17978-NO
DNGYLLDKYTGCKVWCVINNESCNSECKIRRGNYGYCYFWKLACYCEGAP
>ID17983-NO
DNPSVGQFYGLM
>ID17984-ABP_both
DNRMVNHFVQEFKRKHKK
>ID17985-NO
DNSCTPKPSCFF
>ID17986-NO
DNSQWGGFA
>ID17987-NO
DNTVRSKPLNCMNYFWKSSTAC
>ID17988-ABP_both
DNVYSRPPQRFGQNVIS
>ID17989-NO
DNWWPKPPHQGPRPPRPRPKP
>ID17990-NO
DNYIKMSDKCCNVGCTRRELASRC
>ID17993-NO
DPAEDLARYYSALRHYINLITRQRY
>ID17994-NO
DPAFIFYHSTLFFNS
>ID17995-NO
DPAFNSWG
>ID17998-NO
DPATNPGPHFPR
>ID17999-NO
DPCCGYRMCVPC
>ID18001-NO
DPCCSNPACNVNNPQIC
>ID18004-NO
DPCCSYPACGANHPEICG
>ID18006-NO
DPERPPGFTPFRVY
>ID18008-NO
DPFFKVPVNKLAAVSNFGYDLYRVRSSMSPTTN
>ID18009-NO
DPGFNHAFV
>ID18010-NO
DPGFSSWG
>ID18011-NO
DPHHDFMRF
>ID18014-NO
DPLNEERRANRYGFGL
>ID18015-NO
DPLTNIM
>ID18016-NO
DPMTCEQAMASCEHTMCGYCQGPLYMTCIGITTDPECGLP
>ID18018-NO
DPPFAPRM

>ID18019-NO
DPPFSPRL
>ID18020-NO
DPQQRIVTDETVLRF
>ID18021-NO
DPSFIRF
>ID18026-NO
DPSPEPFNPNYNRFRQKIPRI
>ID18027-NO
DPSQDFMRF
>ID18028-NO
DPVDTPNPTRRKPGK
>ID18031-NO
DPYAFGL
>ID18032-NO
DPYAFGLGKRPADL
>ID18033-NO
DPYAFGLGKRPADLYEFGL
>ID18034-NO
DQAAALPYYLYE
>ID18035-NO
DQCTYCGIYCCPPKFCTSSGCRSP
>ID18037-NO
DQDEGNFRRFPTNAVSMSADENSPFDLSNEDGAVYQRDL
>ID18039-NO
DQFDDYGHMRF
>ID18040-NO
DQGFNSWG
>ID18041-NO
DQGTACTGEHAHSFCLNGGTCRHIQQLGE
>ID18042-NO
DQLFPFPRV
>ID18045-NO
DQNRNFLRF
>ID18046-NO
DQQAPPPMFPPRL
>ID18048-NO
DQSCPWCGFTCCLPNYCQGLTCTVI
>ID18051-NO
DQVFPMNPPK
>ID18053-NO
DQWQKLHGGW
>ID18062-ABP_neg
DQYRCLQNGGFCLRSSCPSHTKLQGTCKPDKPNCCRS
>ID18079-NO
DRDDRDDRDDRDDRDDR
>ID18083-NO
DRDRDRDRDR
>ID18084-NO
DRDSCVDKSRCAKYGYYQECQDCCKNAGHNGGTCMFFKCKCA
>ID18090-NO

DRGEMRDILEWGSPHAAAPR
>ID18091-NO
DRGETLDILEWGSPHAAAPR
>ID18095-NO
DRKYKMHHFRWEGPPKD
>ID18097-NO
DRLMYD
>ID18099-NO
DRLYAFGL
>ID18100-NO
DRNFLRF
>ID18102-NO
DRPYSFGL
>ID18103-NO
DRRDPRGIIGIGKKLFG
>ID18105-NO
DRRRRGSRPSGAERRRRRAAAA
>ID18107-NO
DRSDNFIRF
>ID18108-NO
DRSTREPIYMSTI
>ID18110-ABP_both
DRVFKFACRRNKAAR
>ID18111-NO
DRVGA
>ID18114-NO
DRVYI
>ID18115-NO
DRVYIHPFH
>ID18116-NO
DRVYIHPFHLLVYS
>ID18117-NO
DRVYVHPF
>ID18118-NO
DRVYVHPFHL
>ID18119-NO
DRVYVHPFNL
>ID18120-NO
DRVYVIHPF
>ID18122-NO
DRWRPALP
>ID18125-NO
DSAAMHTEYDVIATDNCIPCSHPACGINRGKC
>ID18126-NO
DSACHLGI
>ID18127-NO
DSACVFGA
>ID18128-NO
DSACVVGI
>ID18129-NO
DSACVYGF

>ID18133-NO
DSCNLKCFRYRLNDKY
>ID18137-NO
DSDVSLFNGDLLPNGRCS
>ID18141-NO
DSGEASGDLEE
>ID18143-NO
DSHAKRHHGYKR
>ID18144-NO
DSHAKRHHGYKRKF
>ID18146-NO
DSHEERRQGRHGHHEYGRKFHEKH
>ID18149-NO
DSLKSYWYLQKFSWR
>ID18151-NO
DSNAES
>ID18152-NO
DSPKNLMRF
>ID18153-NO
DSRIRMGFDFSKLFGK
>ID18154-NO
DSSGIVGRPFFLFRPRN
>ID18155-NO
DSSLRLP
>ID18156-NO
DSSNLPPNQKQIVD
>ID18157-NO
DSSPVSTEQLAPTA
>ID18159-NO
DSVCASYF
>ID18160-NO
DSVDPRASYF
>ID18161-NO
DSVGENHQRPPFAPRL
>ID18162-NO
DSVSASYF
>ID18164-NO
DTAVTGLASPLSTGKILDQKAYSCANRLIVLCIENSFMTDARK
>ID18166-NO
DTCRALRCN
>ID18173-ABP_both
DTFDYKKFGYRYDSLELEGR
>ID18174-NO
DTGFAFSPRL
>ID18176-NO
DTHFPICIFCCGCCKTPKCGLCCKT
>ID18177-NO
DTHFPICIFCCGCCRKAICGMCCKT
>ID18178-NO
DTHFPIYIFCCGCCHRSKCGMCCKT
>ID18179-NO

DTLRLRI
>ID18181-NO
DTNFPICLFCCKCCKNSSCGLCCIT
>ID18182-NO
DTSTPALRLRF
>ID18183-NO
DTTFCRCRVSCNILEKYSGKCELSGRTARICC
>ID18184-NO
DTWAGVEAIIRILQQLLFIHFR
>ID18186-NO
DVAHGILDKAYRKVLDQLSARRYLQTLMAKGLGGTPGGGADDDSEPLS
>ID18187-NO
DVAHGILNKAYRKVLDQLSARKYLQTLMAKSVGGNLDGGAEDDSEPLS
>ID18216-NO
DVDCVGWSSYCGPWNNPPCCSWYTCDYYCKL
>ID18217-NO
DVDFNSESTRRKNKQKEIVDLHNSLKKTV
>ID18218-NO
DVDHVFLRF
>ID18220-NO
DVENLHLPLPLLQSW
>ID18224-NO
DVGTQGLSPLRQPAP
>ID18229-ABP_both
DVIVVGAGIMGSSAAYQLAKRGQKTLL
>ID18232-NO
DVKDGGADRGAHSDRGGMWFGPRI
>ID18239-NO
DVPGVLRF
>ID18241-NO
DVPSERYLG
>ID18245-NO
DVRAPALRLRF
>ID18252-NO
DVSASLAVLPDNFPRYPVGKFFQYDTWRQSTQRL
>ID18253-NO
DVSASTTVLPDDFTAYPVGKFFQSDTWKQSTQRL
>ID18254-NO
DVSDGSAERRPYTRMGSGGLKLHCQVHPANCPGGLMVT
>ID18257-ABP_both
DVSIEDKVISLKGDK
>ID18258-NO
DVSTPPTVLPDDSPRYPVVKLFQYNAWKQSTQRL
>ID18259-NO
DVSTPPTVLPDNFPRYPVGKFFQYDTWKQSAQRL
>ID18261-NO
DVVSPPVCGN
>ID18266-NO
DWCDPCPW
>ID18271-NO
DWHLGQGV

>ID18272-NO
DWNGCCVKKAGCCPW
>ID18275-NO
DWRISETIRNLIFPRRK
>ID18318-NO
DYCCHRGPCMVWC
>ID18321-NO
DYDCEPPGNFCGMIKIGPPCCSGWCFFACA
>ID18322-NO
DYDFVRF
>ID18323-NO
DYDFVRFG
>ID18325-NO
DYGCPVFQIECQQHCSATFGWQGRCGGSRRSECICRN
>ID18326-NO
DYGLYP
>ID18329-NO
DYKFHVCDPSFDEKDCDFECKEFGHPGGYCRPDRVQPRIRMCYCTDR
>ID18331-NO
DYLGWMDF
>ID18332-NO
DYLRSC
>ID18333-ABP_both
DYPWFN
>ID18334-NO
DYRPLQF
>ID18335-ABP_both
DYSIRTRLHQESSRNVF
>ID18337-NO
DYTIRTRLHQESSRKVL
>ID18338-NO
DYVGN
>ID18349-NO
EAAGLISFPRV
>ID18351-NO
EAAGLLAFPRV
>ID18352-NO
EAAGLLPFPRL
>ID18354-NO
EACEMAGCL
>ID18362-NO
EACYPPGTFCGIKPGLCCSELCLPAVCVG
>ID18365-NO
EADQDLSPSISIV
>ID18367-NO
EAEDLQVGQVELGGGPGAGSLQPLALEGSLQ
>ID18369-NO
EAEEPLGTMRF
>ID18371-NO
EAEIRIKQQLLFIHFRIGRRRRRRRR
>ID18379-NO

EAFPGQST
>ID18381-NO
EAHISKARRPYIL
>ID18382-NO
EAIERILKQLLFIHFRIGRRRRRRRR
>ID18383-NO
EAIIRILQELLFKHFRIGRRRRRRRR
>ID18384-NO
EAIIRILQQLEFIHKRIGRRRRRRRR
>ID18385-NO
EAIIRILQQLLFIEFRIKRRRRRRRR
>ID18387-NO
EAIVSKARRPYIL
>ID18393-NO
EAPAEDLARYYSALRHYINLITRQRY
>ID18398-NO
EASAFGDIIGELKGKGLGGRMRF
>ID18399-NO
EASGSITCECCFNQCRIFELAQYCRLPDHFFSRIS
>ID18401-NO
EATKCFQWQREMRKKRGPPVSCIKR
>ID18403-NO
EAYAFGL
>ID18404-NO
EAYAGFL
>ID18406-NO
ECAAKNKRCADWAGPWCCEGLYCSCRSYPGCMCRPNS
>ID18407-NO
ECAHSGGACNSHDQCCNAFCDTATRTCV
>ID18409-NO
ECCDDPPCRQNNMEHCPAS
>ID18412-NO
ECCELEWCDGACDCCD
>ID18413-NO
ECCEPSWCDAGCTDGCC
>ID18414-NO
ECCEWEWCDGACDCCN
>ID18417-NO
ECCHPACGQNTSC
>ID18419-NO
ECCNPACGNHTSC
>ID18420-NO
ECCNPACGQHTSC
>ID18422-NO
ECCNPACGRHYSCKG
>ID18424-NO
ECCPQSPPCCHYYYYGSW
>ID18429-NO
ECCTHPACHVSHPELC
>ID18433-NO
ECDNPACGNHTSK

>ID18434-NO
ECDNPACGQHTSK
>ID18439-NO
ECENPACGRHTSK
>ID18441-NO
ECGGLMTRCDGKTTFCCSGMNCSPTWKWCVYAP
>ID18442-NO
ECGQAWC
>ID18443-NO
ECGTLFSGCSTHADCCEGFICKLWCRYERTW
>ID18444-NO
ECIEGSEPCEVFRPYTCCSGHCIIFVCA
>ID18448-ABP_both
ECKFCP
>ID18449-ABP_both
ECKFTVKPYIKRFQLYYKGRMWCP
>ID18451-ABP_both
ECKFTVKPYLKRFQVYCP
>ID18453-NO
ECKKLFGGCTTSSECCAHLGCKQKWPFYCAWDWSF
>ID18455-NO
ECKYLWGTCEKDEHCCEHLGCNKKHGWCGWDGTF
>ID18460-NO
ECLEADYYCVLPFVGNGMCCSGICVFVCIAQKY
>ID18467-NO
ECLEIFKACNPSNDQCCKSSKLVCSRKTRWCKYQI
>ID18469-NO
ECLWMDWVTEKNINGHQAKFFACI
>ID18470-ABP_both
ECMWCP
>ID18471-NO
ECPPRCPTSHCNAGTC
>ID18485-NO
ECREKGQGCTNTALCCPGLECEGQSQGGLCVDN
>ID18492-NO
ECREQSQGCTNTSPPCCSGLRCSGQSQGGVCISN
>ID18494-NO
ECRGYNAPCSAGAPCCSWWTCSTQTSRCF
>ID18497-NO
ECRKMFGGCSVDSDCCAHLGCKPTLKYCAWDGTF
>ID18498-NO
ECRQPGEFCFPVVAKCCGGTCLVICI
>ID18499-ABP_neg
ECRRLWYKGRWVTYCRGR
>ID18500-NO
ECRRRGQGCTQSTPCCDGLRCDGQRQGGMCVDS
>ID18501-NO
ECRWYLGGCKEDSECCEHLQCHSYWEWCLWDGSF
>ID18503-NO
ECRYLFGGCKTTADCCKHLGCKFRDKYCAWDFTFS
>ID18504-NO

ECSESGEWCGLDPALCCGSSCFFTCN
>ID18510-NO
ECSSPDESCTYHYNCCQLYCNKEENVCLENSPEV
>ID18511-NO
ECTAPSGYCDYPEECCEVECGRHYCDWWY
>ID18513-NO
ECTHSGGACNSHDQCCNAFCDTATRACV
>ID18514-NO
ECTHSGGACNSHDQCCNAFCDTATRTCV
>ID18517-NO
ECTHSGGACNSHTQCCDDFCSTATSTCI
>ID18518-NO
ECTKLLGGCTKDSECCPHLGCRKKWPYHCGWDGTF
>ID18522-NO
ECTPPEGACNHPSHCCEDFCDRGRNRCM
>ID18525-NO
ECTPPGGACNIHPHCCEEFCDMANNRCLEM
>ID18526-NO
ECTPPGGACYYHSQCCGDFCQRYINSCL
>ID18527-NO
ECTRPGGACYYDSHCCRHVCHEVFNTCM
>ID18531-NO
ECTRSGGACNSHTQCCDDFCSTATSTCI
>ID18534-NO
ECTRSGGACNSHTQCCNAFCDTATRTCV
>ID18535-NO
ECTRSGGACYSHNQCCDDFCSTATSTCI
>ID18536-NO
ECTRSGGACYSHNQCCDDFCSTATSTCV
>ID18539-NO
ECVGENGHCRSWYNDCCDGYYCSCMQPPNCICRNNN
>ID18540-ABP_both
ECVYYKGRMWCP
>ID18541-NO
ECWSQAADCSDGHCCAGRSFSKNCRPYGGD
>ID18542-ABP_both
ECYLKRFQVYYKGRMWCP
>ID18545-NO
EDCEAGGRFCGFPKIGEPCCSGWCFFVCA
>ID18546-NO
EDCIAVGQLCVFWNIGRPCCSGLCVFACTVKLP
>ID18547-NO
EDCIPKWKGCVNRHGDCCEGLECWKRRRSFEVCVPKTPKT
>ID18548-NO
EDCPGEGEQCDVEFNPCCPPLTCIPGDPYGICYII
>ID18549-NO
EDCTSRFCS
>ID18557-NO
EDEEMLLPAAAPDYADPMQSYWWYPSYAGYADLDWNDYRRAE
>ID18558-NO
EDEKDQRAADWMGPDPLDYADMDEDSIYYEN

>ID18559-NO
EDENNPFYLR
>ID18562-NO
EDGGSFMCLWCGEVHG
>ID18563-NO
EDGGSWIESWPEWWLG
>ID18564-NO
EDGYLLNRDTGCKVSCGTCRYCND
>ID18575-NO
EDMNQKLFDLRGKFKRPPLRRVRMSADAML
>ID18576-NO
EDNCIAEDYGKCTWGGTKCCRGRPCRCSMIGTNCECTPRLIMEGLSFA
>ID18578-NO
EDPFLRF
>ID18583-NO
EDSGDGWPQQPFVPRL
>ID18585-NO
EDVDHVFLRF
>ID18586-NO
EDVEHVFLRF
>ID18587-NO
EDVVCC
>ID18590-NO
EDYKGWMDF
>ID18594-NO
EECCPNPPCFATHSEICG
>ID18595-NO
EECCPNPPCFATNSDICG
>ID18602-NO
EEDFMRF
>ID18605-NO
EEDYDERPYMQPF
>ID18607-NO
EEEAA
>ID18608-NO
EEEAAGRKRKKRT
>ID18609-NO
EEEAAKKK
>ID18610-ABP_neg
EEEEAAAGFAKLLAKLAKKLL
>ID18611-ABP_neg
EEEEAAAGKWKLFKKLPKFHLHLAKKF
>ID18613-ABP_neg
EEEEAAAGRWWRWWRRWWRR
>ID18614-ABP_neg
EEEEAAAGWGLRRLLKYGKRS
>ID18616-ABP_both
EEEEEEEEEEKKRLKKIFKKPMVIGVTIPF
>ID18617-ABP_both
EEEEWWW
>ID18618-ABP_both

EEEEWWWW
>ID18619-NO
EEEKREGENEKEQEDDNQSEEKRGLVSDLLSTVTGLLGNLGGGGLKKI
>ID18626-NO
EEIIRKLQQLLFIHFRIGRRRRRRRR
>ID18629-NO
EELEAKDVIESKALATLDEER
>ID18646-NO
EEVFDDTDVGDELTNALESVLTDLKD
>ID18654-NO
EFDWWNLG
>ID18661-NO
EFFPL
>ID18662-NO
EFILPAVGRTCCDLTWCDGNCRCCTP
>ID18668-ABP_both
EFKRIVQRIKDFLRNLV
>ID18669-NO
EFLFRPRN
>ID18670-NO
EFLRI
>ID18671-NO
EFNADDLTLRF
>ID18673-NO
EFRGSIVIQGTKEGKSRPSLDIDYKQRVYDKNGMTGDAYGGLNIRPGQPS
>ID18674-NO
EFVAPFPEVFGK
>ID18675-NO
EFVDPNLRF
>ID18677-NO
EGANSNEAKGMWFGPRL
>ID18679-NO
EGCAFEGESCNVQFYPCCPGLGLTCIPGNPDGTCYYL
>ID18680-NO
EGCCSDPRCSGKHQDLCS
>ID18682-NO
EGDFTPRL
>ID18683-NO
EGECGGFWWKCGSGKPACCPKYVCSPKWGLCNFPMP
>ID18684-NO
EGEPR
>ID18686-NO
EGGPKP
>ID18690-NO
EGIIVIVVG
>ID18691-ABP_both
EGLEPG
>ID18694-NO
EGLHYTCSSHFPYSQY
>ID18695-NO
EGLPGPQGPKGFPGLPGLTG

>ID18696-NO
EGLRW
>ID18697-NO
EGNCTPWLGGCTSPEECCPGNCETYCRAW
>ID18699-NO
EGPKLVAS
>ID18700-NO
EGRERDHELRHRRHHHQSPKSHFELPHYPGLLAHQKPFIRKSYKCLHKRC
>ID18702-NO
EGRMYSFGL
>ID18703-NO
EGSGSGETSGMWFGPRL
>ID18710-ABP_both
EHMDRILAQLL
>ID18714-NO
EHSHGWYPG
>ID18715-NO
EHSYGLSPG
>ID18716-NO
EHWSDYFKPG
>ID18718-NO
EHWSGLRPG
>ID18719-NO
EHWSHGLNPG
>ID18720-NO
EHWSHGLSPG
>ID18721-NO
EHWSHGWLPG
>ID18722-NO
EHWSHGWYP
>ID18724-NO
EHWSLCHAPG
>ID18725-NO
EHWSYGLQPG
>ID18727-NO
EHWSYGLSPG
>ID18728-NO
EHWSYGMNPG
>ID18730-NO
EHWSYLLRP
>ID18731-NO
EHYSLEWKPG
>ID18733-NO
EIAKLM
>ID18735-NO
EICCNPACGPKYSC
>ID18738-NO
EICVDGLCV
>ID18741-NO
EIDRSGFGF
>ID18744-NO

EIDRTAFDNFF
>ID18745-ABP_both
EIEKFDKSKLK
>ID18746-ABP_both
EIENGVKWVSFKGAK
>ID18751-NO
EIILPALRVQSFIACTMGWC
>ID18753-NO
EILDEI
>ID18757-NO
EINFTPNW
>ID18759-NO
EIPGVLRF
>ID18760-NO
EIPSCESSASPDQSDSSVPPEE
>ID18763-NO
EIRQTHNIFFNFFKRR
>ID18765-NO
EITFTPNW
>ID18766-NO
EITVEPVRHPKKDPSEAE
>ID18767-NO
EIVNIIDSISDVAKQICCEITVQCCVLDEE
>ID18770-ABP_both
EKALEKLIAIQKAIKGMLAGWFTGVGARRKR
>ID18771-ABP_both
EKALEKLIAIQKAIKGMLNGWFTGVGFRRKR
>ID18780-ABP_both
EKCLRWQNEMRKVGG
>ID18791-NO
EKDERF
>ID18795-NO
EKERERQ
>ID18796-NO
EKGKEILGKI
>ID18804-ABP_both
EKHWLWLW
>ID18813-NO
EKLKPNMRRAFGLLTYPRI
>ID18817-NO
EKMIG
>ID18822-NO
EKRQRLCCGFPKSCRSRQCKPHRCCGR
>ID18823-NO
EKSLVPSVITTCCGYDPGTMCPPCRCTNSC
>ID18824-NO
EKSSRPEFYKVILGAHEEYIRG
>ID18829-NO
EKYEGKISKTMSGLDCQAWDS
>ID18838-NO
ELALELALEALEAALELA

>ID18846-ABP_both
ELCCDRIGCYTARCRKKRSCCL
>ID18847-ABP_both
ELCDRICGYGTARCRKKCR
>ID18872-ABP_both
ELDRICGYGTARCRKKR
>ID18873-ABP_both
ELDRICGYGTARRKKCR
>ID18875-ABP_both
ELDRIGYGTARRKKR
>ID18876-ABP_both
ELDRISSGYGTARCRKKRSQEYRIGRSPNTYASCLRK
>ID18882-NO
ELEELNVPGE
>ID18888-NO
ELGRF
>ID18891-NO
ELHVNKARRPYIL
>ID18892-NO
ELHVNKARRVYIL
>ID18894-NO
ELIKESR
>ID18901-NO
ELLGGRF
>ID18902-NO
ELNFQHAFV
>ID18905-NO
ELNFSPNWGN
>ID18906-NO
ELNFSTGW
>ID18907-NO
ELNFSWGT
>ID18908-NO
ELNFTPNW
>ID18909-NO
ELNFTPNWGT
>ID18917-NO
ELPVM
>ID18923-ABP_both
ELRCMCIKTTSGIHP
>ID18925-NO
ELSAERPLNEQIAEAE
>ID18928-NO
ELSLEFDYPDTNSEEELDNGELLEGPVKKGRKYKMHHFRWEGPPKD
>ID18932-NO
ELSLELDYPEIDLDEDIEDNEVESALTKKNGNYRMHHFRWGSPPKD
>ID18936-NO
ELTFSPDWGK
>ID18937-NO
ELTFSSGWGN
>ID18938-NO

ELTFTPAW
>ID18939-NO
ELTFTPGW
>ID18941-NO
ELTFTPNW
>ID18942-NO
ELTFTPNWGS
>ID18943-NO
ELTFTPNWGT
>ID18958-NO
ELVHNKARPYIL
>ID18960-ABP_both
ELVRKNVDHLSTPDVLELV
>ID18961-NO
ELVVRDPYFV
>ID18962-NO
ELYAFPRV
>ID18963-NO
ELYENKPRRPIL
>ID18964-NO
ELYVSRL
>ID18965-NO
EMEEC
>ID18968-ABP_both
EMIAAAIRAEKSRGGSSRQS
>ID18969-ABP_both
EMIAAAIRAEKSRGGSSRQSIQKYIKSHYK
>ID18970-NO
EMIFGAPMWALGHLM
>ID18971-NO
EMILPALRQWQCCTMAWCDSGCLCCE
>ID18972-NO
EMPFPK
>ID18973-NO
EMPFPKYPVEP
>ID18974-NO
EMPGVLRF
>ID18975-NO
EMPGVLRFG
>ID18977-NO
EMRKPDGALFNLFRRR
>ID18978-NO
EMRKSNNNFFHFLRRI
>ID18979-NO
EMRLPKILRDFIFPRKK
>ID18983-NO
ENAEEDIVLMENLFCSYIVGRLIPSGLNIILE
>ID18985-NO
ENCCPRG
>ID18986-NO
ENCGRQAG

>ID18987-NO
ENDHRMPNNLNRPNNLSKGGAKCGAAIAGGLFGIPKGPLAWAAGLANVYS
>ID18989-NO
ENEKKAVPGVLRF
>ID18991-NO
ENFAGGCATGYLRTADGRCKPTF
>ID18992-ABP_both
ENFFKRIRRAGKRIRDAIISAAPAVETLAQAQKIIKGGD
>ID18993-NO
ENFHLRP
>ID18994-NO
ENFNGGCLAGYMRTADGRCKPTF
>ID18995-NO
ENFSGGCVAGYMRTPDGRCKPTF
>ID18999-NO
ENLHLP
>ID19000-NO
ENLHLPLP
>ID19001-NO
ENLHLPLPLL
>ID19003-NO
ENLLRF
>ID19004-NO
ENLLRFFVAPFPEVFG
>ID19011-NO
EPAEDLARYYSALRHYINLITRQRY
>ID19017-ABP_neg
EPCSPKNNYHDLFYRT
>ID19023-NO
EPDNWSLDFPRR
>ID19025-NO
EPEPMARPTRPKVFESPEELRQYLDLVKEYYSLSGKARY
>ID19026-NO
EPFDDYGHMRFG
>ID19036-NO
EPIPYGFLP
>ID19038-NO
EPLWSGRF
>ID19039-NO
EPNSIWS
>ID19040-NO
EPNSMWS
>ID19041-NO
EPPGGSKVILF
>ID19043-NO
EPQYSFGL
>ID19045-NO
EPRLRFHDV
>ID19046-NO
EPSKDAFIGLM
>ID19048-NO

EPTWNNLKGMW
>ID19049-NO
EPVLGPVRGPFP
>ID19050-NO
EPVPPGFTPFRLT
>ID19051-NO
EPYAFGL
>ID19052-NO
EPYEFGL
>ID19053-NO
EPYGFGI
>ID19054-NO
EPYLRF
>ID19059-NO
EQDFMRF
>ID19064-NO
EQERDSDDEDQGEVTEQVVKRSVIGCWTKSIPPRPCFVKG
>ID19068-NO
EQFEDYGHMRF
>ID19070-ABP_both
EQHSSGKSDV
>ID19073-NO
EQLEQWAAILGAGWN
>ID19074-NO
EQLIKKDVKHKDKFAKKSECVRAAAAAFTGGDKS
>ID19076-NO
EQLIPFPRV
>ID19077-ABP_both
EQLKKCWNNYVQGHCRKICRVNEVPEALCENGRYCCLNIK
>ID19078-NO
EQLSFTSIGILQLLTIGTRSCWFFYCRY
>ID19079-ABP_pos
EQLTK
>ID19081-NO
EQPGLW
>ID19087-NO
EQWGREEGEE
>ID19092-NO
ERAMT
>ID19093-NO
ERAYSFGL
>ID19098-NO
ERDMHRFSFGL
>ID19104-NO
ERERERERERERER
>ID19107-NO
ERGQT
>ID19108-NO
ERGSRGQRCGPGEVFNQCGSACPRVCGRPPAQACTLQCVSGCFCRRGYIR
>ID19110-NO
ERIGIW

>ID19111-NO
ERILDLRKTKKSCKNGEVLGCVSGHGPPGCSENECGMGPRPKACFFDCHY
>ID19112-NO
ERILSILRHQNLLKE
>ID19113-NO
ERKIKVYL
>ID19114-NO
ERKKRRRE
>ID19117-NO
ERNNT
>ID19118-NO
ERNQT
>ID19121-NO
ERPRMEEEEEAYGWMDF
>ID19122-NO
ERPVG
>ID19123-NO
ERPYSFGL
>ID19124-NO
ERPYSGL
>ID19127-NO
ERVCCGYPMSCKSRACKPSYCC
>ID19134-NO
ERYPI
>ID19135-NO
ESAGLIPFPRV
>ID19139-NO
ESDDYGHMRF
>ID19140-NO
ESDRNGRCCHPACARKYNCGR
>ID19143-NO
ESEEGGSNATKKPYIL
>ID19146-NO
ESFSDWWKLLAE
>ID19147-NO
ESGGSGEANGMWFGPRL
>ID19150-ABP_both
ESGNEPLWLYQGDNIPKAPSTAEHPFLPSIIDDVKFNPDRRYAR
>ID19151-NO
ESGWNNLKGLW
>ID19152-NO
ESIIAF
>ID19153-NO
ESIINF
>ID19154-NO
ESIVRPPPVEAKVEETPE
>ID19158-NO
ESLARPCAPGAPAEARL
>ID19160-NO
ESLRW
>ID19161-NO

ESQFSRDFLNF
>ID19163-NO
ESRISDILLDFLFQRKK
>ID19165-NO
ESRLPKIRFDFIFPRKK
>ID19166-NO
ESRNPPLNGSMF
>ID19167-NO
ESRVSRIILDFLFQRKK
>ID19168-NO
ESRYRQCYFNPISCF
>ID19170-NO
ESSNKRENQKQIV
>ID19176-NO
ESVLHQPQRF
>ID19177-NO
ESVPTFTPRL
>ID19184-NO
ETESTPDYLKNIQQQLEEYTKNFNTQVQNAFDSDKIKSEVNNFIESLGKI
>ID19185-NO
ETFADWWKLLAE
>ID19186-NO
ETFQYSHGWTN
>ID19187-NO
ETFQYSRGWTN
>ID19188-NO
ETFSDWWKLLAE
>ID19190-NO
ETHFGASTRFGW
>ID19194-NO
ETNFLRF
>ID19195-NO
ETNGQ
>ID19197-NO
ETSDDYGHLRF
>ID19198-NO
ETSFTPRL
>ID19200-NO
EVAQMHVWRAVNHDRNHGTGSGRHGRFLIRNRYRYGGGHLSDA
>ID19205-NO
EVEEPEAPAPPAK
>ID19206-NO
EVEGPQVGALELAGGPGAGGLEGPPQ
>ID19208-NO
EVHHQKLVFF
>ID19212-NO
EVLNENLLRF
>ID19215-NO
EVMAGNLYPG
>ID19216-NO
EVMAGNYLPG

>ID19217-NO
EVNFPTSW
>ID19218-NO
EVNFSPGWGT
>ID19220-NO
EVNFSPSW
>ID19221-NO
EVNFSPSWGN
>ID19222-NO
EVNFSPTW
>ID19223-NO
EVNFSPYW
>ID19228-NO
EVNFTPSW
>ID19230-NO
EVPNFQADNVPEAGGRV
>ID19233-NO
EVRFRQCYFNPISCF
>ID19234-NO
EVRPFPEVYERIA
>ID19235-NO
EVRYRQCYFNPISCF
>ID19238-NO
EVVTEECEEYCKEQNKTCCGLTNGRPRCVGVCF
>ID19240-NO
EWCGTNSDCGEGECCTGGSFNRH
>ID19250-ABP_both
EWFKARRWGWRMKKLQA
>ID19252-NO
EWFNGRF
>ID19255-ABP_both
EWKLPDLIINHITLTRRNCFKYRCG
>ID19256-ABP_both
EWKRWVQRWKDWLRNLV
>ID19257-NO
EWLGGRF
>ID19258-NO
EWLKGRF
>ID19259-NO
EWLRGRF
>ID19262-ABP_both
EWRPHGSIGGSGLRPGRPQTLPPQRPRRPDFNGPRHRF
>ID19263-ABP_both
EWRPHGSNGGSSLRPGRPQTLPPQRPIQPDFNGPRQRF
>ID19279-NO
EYDDMYTEKRPKVYAFGL
>ID19286-NO
FAATSAE
>ID19288-NO
FAEFLPSDEEGESYSKEVPEME
>ID19292-NO

FAESLPSDEEGENYSKEVPEIE
>ID19302-ABP_neg
FAKLLKKLL
>ID19303-NO
FAKRYVELVIVA
>ID19307-NO
FALGAVTKRLPSLFCLITRRC
>ID19308-NO
FALPC
>ID19309-NO
FALPQY
>ID19311-NO
FANTIRLLINKVREWKNKQSS
>ID19313-NO
FAPRSPQLRLRF
>ID19314-NO
FAPWDTASFMLG
>ID19315-NO
FAQTQS
>ID19317-ABP_both
FASGIAGMAGKLF
>ID19321-NO
FAVIFTCTPPGSHCTGHSDCCSDFCSTMSDVCQ
>ID19328-NO
FCCPFIRYCCW
>ID19329-NO
FCCPVIRYCCW
>ID19332-NO
FCCVFPWCTGCYCC
>ID19334-NO
FCFFTITNMSGGCLV
>ID19336-NO
FCFWKTCT
>ID19337-NO
FCGQACSSVKCPKKCFCHPEEKVCYREMRTKERD
>ID19339-NO
FCHNSISCALGGDSTCNNVCVRQGNPHGGRCLPRDGCPGYDICACYPNN
>ID19340-NO
FCNINNVCNFASRNDYSYW
>ID19345-NO
FCSDPSCRFGNPELCDWRR
>ID19347-NO
FCVLRP
>ID19348-NO
FCYWKVCT
>ID19351-NO
FCYWRTPENT
>ID19354-NO
FDAFTTGFGH
>ID19355-NO
FDAFTTGFGHN

>ID19364-NO
FDAYTTGFGHS
>ID19365-NO
FDDARL
>ID19366-NO
FDDFLRF
>ID19367-NO
FDDLMLLLCCRQGPVCFIPLNEWPCSRM
>ID19368-NO
FDDYGHMRF
>ID19369-NO
FDEIDRAGMG
>ID19370-NO
FDEIDRAGMGFA
>ID19373-NO
FDEIDRSGFGFA
>ID19374-NO
FDEIDRSGFGFV
>ID19375-NO
FDEIDRSSFA
>ID19376-NO
FDEIDRSSFGF
>ID19378-NO
FDFGFAGLDTYDAIHRALEQPARGTSNSGSGYNMLMKMQRH
>ID19379-NO
FDFHTV
>ID19380-NO
FDGRNAAADAKVINTVARIAWDICCSEPDCNHKCV
>ID19386-NO
FDHSSKWTRTSP
>ID19393-NO
FDKPVSPL
>ID19394-NO
FDNPFGCPIDEGKCFDHCNNKAYDGGYCGGSYRATCICHRK
>ID19395-NO
FDPFFWKYSPRD
>ID19397-NO
FDRDFMHF
>ID19399-NO
FDRYDDETAYGYGFDNHIF
>ID19400-NO
FDRYEEENPYGYNFGAHIF
>ID19401-NO
FDSLTGLGINSQ
>ID19404-ABP_both
FDVMGIIKKIASAL
>ID19407-NO
FECSISCEIEKKGESCKPKKCKGGWKCKFNMCVKV
>ID19408-NO
FEDFVLGFI
>ID19410-NO

FEDYVPLSCF
>ID19416-NO
FEPSLRLRF
>ID19417-NO
FEQKIGAWWCA
>ID19419-ABP_both
FFAAIFRPIVHVGKTIHRLVTG
>ID19422-NO
FFCPFGCALVDCGPNRPCRDTGFMSCDC
>ID19426-NO
FFDDKCDKLRGTCKNSCEKNEELTSFCQKSLKCCRTIQTCGNTTD
>ID19431-NO
FFFAAGRKRKKRT
>ID19432-ABP_both
FFFDTLKNLAGKVIGALT
>ID19436-ABP_pos
FFFFFF
>ID19437-ABP_pos
FFFFFFF
>ID19440-ABP_both
FFFFRRRR
>ID19443-ABP_both
FFFHIVKGLFHAGRMIHGLV
>ID19444-ABP_both
FFFHIVKGLFHAGRMIHGLVNRRRHRHGMEELDLDQRAFEREKAFA
>ID19447-ABP_both
FFFLRRIF
>ID19448-ABP_both
FFFLSRIFGK
>ID19452-ABP_both
FFGHIWHGARTLFRDVFA
>ID19453-ABP_both
FFGLHNLVPSMLCVVRKKC
>ID19454-NO
FFGRCVSP
>ID19455-ABP_both
FFGSLLKLLPKLL
>ID19457-ABP_both
FFGSTIGALANFLPSLISKIRN
>ID19458-ABP_both
FFGTKGIFSKVEPIFCKISHSC
>ID19459-NO
FFGVRCVSP
>ID19461-NO
FFGWLIKGAIHAGKAIHGLIH
>ID19462-ABP_both
FFGWLIKGAIHAGKAIHPLIHRRRH
>ID19468-ABP_both
FFHHIARGIVHVGKTIHRLVTG
>ID19469-ABP_both
FFHHIFNGLVGVGKTIHRLI

>ID19474-ABP_both
FFHHIFRGIVHVGKKIHRLVKG
>ID19481-ABP_both
FFHHIKRGIKHVGKTIHRLVTG
>ID19482-ABP_both
FFHHIKRGIVHVGKTIHRLVTG
>ID19486-ABP_pos
FFIFFF
>ID19490-ABP_both
FFKLIPKLVKGLISAFK
>ID19495-NO
FFLPLLGAAAQVLPSLICKIFKKC
>ID19498-NO
FFPALISCVVLKDC
>ID19500-ABP_neg
FFPFVAKLLKGLF
>ID19503-ABP_both
FFPGIIKVASAILPTAICAITKRC
>ID19504-ABP_neg
FFPGVKKLLKGLF
>ID19505-ABP_neg
FFPIFGKLLRGLF
>ID19506-ABP_neg
FFPIFKKLLNGLF
>ID19508-NO
FFPIVAGVAGQVLKKIFCTISKKC
>ID19509-ABP_neg
FFPIVAKLLRGLF
>ID19513-ABP_neg
FFPIVGKLLKGLL
>ID19514-ABP_neg
FFPIVGKLLRKLF
>ID19518-ABP_neg
FFPIVKILLKGLF
>ID19520-ABP_neg
FFPIVKKILKGLF
>ID19521-ABP_neg
FFPIVKKILNGLF
>ID19523-ABP_neg
FFPIVKKLLKLLF
>ID19524-ABP_neg
FFPIVKKLLNGLF
>ID19525-ABP_neg
FFPIVKKLLRALF
>ID19526-ABP_neg
FFPIVKKLLRGLF
>ID19527-ABP_neg
FFPIVKKLLSGLF
>ID19528-ABP_neg
FFPIVKKMLSGLF
>ID19529-ABP_neg

FFPIVKRLLKGLF
>ID19530-NO
FFPLLFGALSSLLPKLF
>ID19531-NO
FFPLLFGALSSMMPKLFGK
>ID19532-ABP_neg
FFPLVKKLLKGLF
>ID19534-ABP_neg
FFPLVKKLLSGLF
>ID19535-ABP_both
FFPLVLGALGSILPKVFGK
>ID19536-ABP_both
FFPMLADLVSKIF
>ID19538-NO
FFPNVASVPGQVLKKIFCAISKKC
>ID19540-ABP_neg
FFPWVKKLLGGLF
>ID19546-NO
FFRVLAKLGKLA
>ID19547-NO
FFSGILKLVFKIPSVLCAVLKNC
>ID19554-ABP_both
FFSNK
>ID19556-NO
FFVAP
>ID19557-NO
FFVAPFEVFGK
>ID19558-NO
FFVAPFPEVFGK
>ID19559-NO
FFVAPFPFEVFGK
>ID19560-NO
FFVAPFPGVFGK
>ID19561-NO
FFVAPFPQVFGF
>ID19568-ABP_both
FFYNVIKIYGNMAGRISK
>ID19570-NO
FGASTRGA
>ID19571-NO
FGCVMASCR
>ID19576-NO
FGGIDDINQIGQSD
>ID19582-NO
FGLIDVKCFASSECWTACKKVTGSGQGKCQNNQCRCY
>ID19583-NO
FGMIPSII
>ID19585-NO
FGRCVSP
>ID19589-NO
FGSFIPCAHKGEPCTICCRPLRCHEEKTPTCV

>ID19591-NO
FGSFSPYYFYQQPK
>ID19592-ABP_both
FGTGVKRKAQCDLKSK
>ID19595-ABP_both
FHAGAM
>ID19596-ABP_both
FHEAIAPVVHAAVKALVGFLG
>ID19599-ABP_both
FHFHLHF
>ID19600-ABP_both
FHFHLHFASASAFIKHFIHRF
>ID19602-ABP_both
FHFHLHFPSGSPFIKHFIHRF
>ID19604-ABP_both
FHFHLHFPYPFIKHFIHRF
>ID19605-ABP_both
FHFHLHFSATFIKHFIHRF
>ID19607-NO
FHGGSWYRFPWGY
>ID19611-ABP_pos
FHLGHLK
>ID19614-NO
FHWWQTSPAHFS
>ID19618-ABP_both
FIFHIIKGLFH
>ID19620-ABP_both
FIFHIIKGLFHAG
>ID19622-ABP_both
FIFHIIKGLFHAGKMIHGLVTRRRHGVEELQDLDQRAFEREKAFA
>ID19624-NO
FIGDLLSSLLGHI
>ID19625-ABP_both
FIGGLRRLFATVVGTVVCAINKLGGG
>ID19629-NO
FIGPIIKIASSLLPTAICKIFKKC
>ID19630-NO
FIGPKKNIINSLFGR
>ID19631-NO
FIGPMIKIATNLLPTVICKIFKKC
>ID19634-ABP_pos
FIGRAIGDFVYFGAKGLRESGKLLNYYYKHKH
>ID19638-NO
FIIFRIAASHKK
>ID19640-NO
FIKGLKRLCAVIVPSVICAVDKLPPG
>ID19641-ABP_both
FIKHFIHRF
>ID19642-ABP_both
FIKHFIHRFGGGIKQLLHFFQRF
>ID19643-ABP_both

FIKHFIHRFSATLQLLKQLLKLLKQF
>ID19644-ABP_both
FIKHFIHRFSATRWRRLLKKLHHLLH
>ID19647-ABP_both
FIKHFIHRFSGGRWRWRWF
>ID19649-ABP_both
FILGKLWKGVKSIF
>ID19653-ABP_both
FINKAGKLQSQLRTTVVAAAAFLDAFQKVA
>ID19654-NO
FINTIKLLIEKYREWKNKQSS
>ID19657-ABP_both
FIPGLRRLFATVVPTVVGAINKLPPG
>ID19659-NO
FIPINNRLSDVLQSLEEERLRDSLLQDFFDRVAGRDGSAV
>ID19661-ABP_neg
FIPIVKKLLSGLF
>ID19663-NO
FIPLIGSVLSQLL
>ID19664-NO
FIPVTLLALHKIKEKLN
>ID19669-NO
FIRIGC
>ID19671-NO
FIRKFLKKWLL
>ID19672-NO
FIRKFLQKLHL
>ID19674-ABP_both
FIRSLFFF
>ID19676-NO
FISAIASFLGKFLGK
>ID19678-NO
FISDQSRRKDLSDRPLPE
>ID19683-ABP_both
FISQIISTARI
>ID19685-NO
FITKALGISYGRKKRR
>ID19697-ABP_both
FKAFRWAWRMKKLAAPS
>ID19698-ABP_both
FKAFRWAWRWKKLAAPS
>ID19700-ABP_both
FKALLWQWRM
>ID19701-ABP_both
FKALRALRLEDLRIPTSYIK
>ID19702-NO
FKAPYNIHWHCKPGLLC
>ID19710-ABP_both
FKCRRAQARMKKLGA
>ID19712-ABP_both
FKCRRFQFRMKKLGA

>ID19716-ABP_both
FKCRRWQWRMKKLGAPS
>ID19722-NO
FKCRRWQWRMKKLGKGLKKMRWQWRRCKF
>ID19723-ABP_neg
FKCRRWQWRMKKLGKWKLLSKAQEKFGKNKSR
>ID19725-ABP_both
FKCWRWQWRWKKLGAPS
>ID19728-ABP_both
FKFGSFIKRMWRSKLAKKLRAKGKELLRDYA
>ID19734-NO
FKGLSHFDEN
>ID19735-NO
FKGLSHFGEN
>ID19736-NO
FKGRYYP
>ID19743-ABP_both
FKKFFKKLKNSVKKR
>ID19745-NO
FKKFRKF
>ID19746-ABP_both
FKKFWKWFRRFGGGFIKHFIHRF
>ID19748-ABP_both
FKKKFKCFCKFFKKFF
>ID19751-ABP_both
FKKLKKLFSKLFSFK
>ID19752-ABP_both
FKKLKKLFSKLWNWK
>ID19759-NO
FKMWKRPPFQTSCSGGIKE
>ID19766-ABP_both
FKQFHFKDFNRAFGFMTRVALQAEKL
>ID19767-NO
FKQQQQQQQQQ
>ID19768-ABP_both
FKRFIGSVKHGIGHLVHHIGVAL
>ID19770-ABP_both
FKRIVQRIKDFLRGIGKFLHSAKKF
>ID19772-ABP_both
FKRLKKLISWIKRKRQQ
>ID19774-ABP_both
FKRWVQRWKRFLR
>ID19775-NO
FKSLTKYSPGRQN
>ID19778-ABP_both
FKTWKNRPILSSCSGIIKG
>ID19782-NO
FKVTLSCTPPGTYCVGPSTCCSDVCSMSNVCQ
>ID19783-ABP_both
FKVTWKTKWWKG
>ID19784-ABP_both

FKVVISKPGLSVRVGTALVT
>ID19786-ABP_both
FKWRRWQWRMKKLWA
>ID19792-NO
FLAKKLAKHLAKKQAES
>ID19793-NO
FLAKKVAKKLVSHVAQKQLE
>ID19795-NO
FLAKKVAKTVAKQAAKQGAKYIANKQTE
>ID19796-NO
FLAKKVAKTVAKQAAKQGAKYVANKHME
>ID19799-NO
FLAKNVAKKLVSHVAKKQLE
>ID19800-ABP_neg
FLALILRKIVTAL
>ID19801-ABP_neg
FLALLKKLL
>ID19807-NO
FLEGVISTIKDFAGKVCCSVSVNFCCPTA
>ID19809-ABP_both
FLFGLASKVFPAVYCKVTRK
>ID19812-ABP_both
FLFKLIPKAIKKLISKFK
>ID19813-ABP_both
FLFKLIPKVIKGLVKAIRK
>ID19814-NO
FLFNVIPHAINATASLIKK
>ID19815-NO
FLFPIASMLGKVL
>ID19816-NO
FLFPKANIIKSLFGK
>ID19818-NO
FLFPLAKASFLGKVL
>ID19819-NO
FLFPLIASFLGKVL
>ID19820-NO
FLFPLITSFLSKFL
>ID19824-ABP_both
FLGALAKALSKLL
>ID19825-NO
FLGALFGAAAKIVPSLICKISGKC
>ID19826-NO
FLGALFHALSHLL
>ID19827-ABP_both
FLGALFKALSKLV
>ID19829-ABP_both
FLGALFKAVSKLL
>ID19830-ABP_both
FLGALFKFASK
>ID19832-ABP_both
FLGALFKLASK

>ID19833-ABP_both
FLGALFKVA
>ID19834-ABP_both
FLGALFKVASK
>ID19835-ABP_both
FLGALFKVASKV
>ID19837-ABP_both
FLGALFKVASW
>ID19838-ABP_both
FLGALFKVAWK
>ID19839-ABP_both
FLGALFKVVSKLL
>ID19840-ABP_both
FLGALFKVWSK
>ID19841-ABP_both
FLGALFKWASK
>ID19842-ABP_both
FLGALFWVASK
>ID19844-NO
FLGALLGAAANIVPSLICKISRKC
>ID19845-NO
FLGALLGVGAKILPSLICKISGKC
>ID19846-NO
FLGALLSAGATILPSLICKIFKKC
>ID19849-ABP_both
FLGALWEVAKSVF
>ID19850-ABP_both
FLGALWKVAKSVF
>ID19851-ABP_both
FLGALWKVASK
>ID19852-ABP_both
FLGALWNVAKKVF
>ID19854-ABP_both
FLGAVFKALSKLL
>ID19855-ABP_both
FLGAVLKVAGKLVPAAI
>ID19856-ABP_both
FLGAVLKVAGKLVPAAICKISKKC
>ID19857-ABP_both
FLGAVLKVCKISKKCAGKLVPAAI
>ID19858-ABP_both
FLGAVVKALSKLL
>ID19859-ABP_both
FLGAWFKVASK
>ID19862-ABP_both
FLGELWNVWKSVF
>ID19863-ABP_both
FLGFLFKVASK
>ID19864-NO
FLGGILKVASKIPSVFCAVLKTC
>ID19867-ABP_both

FLGGLIKIVPAMICAVTKKCHHHHHH
>ID19868-NO
FLGGLLASLLGKIGKK
>ID19870-NO
FLGGLLSGIFKHLGKK
>ID19871-NO
FLGGLLSSIFGHLGK
>ID19873-NO
FLGGLLSSVLGPLGKNKSIETLKMELEII
>ID19877-ABP_both
FLGGVFKLASKVFPAVFGKV
>ID19878-NO
FLGKKFKKYFLQLLK
>ID19879-NO
FLGKKVLKAVGKQAAKKQME
>ID19880-ABP_both
FLGKLFKVASK
>ID19881-ABP_both
FLGKVFKGASKVFGAVFGKV
>ID19884-ABP_both
FLGKVFKGVSKVFPAVFGKV
>ID19885-ABP_both
FLGKVFKKASKVFPAVFGKV
>ID19886-ABP_both
FLGKVFKKASKVFPAVVGKV
>ID19887-ABP_both
FLGKVFKKASKVVPAVFGKV
>ID19888-ABP_both
FLGKVFKKAVKVFPAVFGKV
>ID19891-ABP_both
FLGKVFKLASKVFKAVFGKV
>ID19892-ABP_both
FLGKVFKLASKVFPAVFGKV
>ID19893-ABP_both
FLGKVFKLASKVFPAVFKKV
>ID19894-ABP_both
FLGKVFKLASKVFPKVFGKV
>ID19897-ABP_both
FLGLIGSLL
>ID19898-ABP_both
FLGLLFKVASK
>ID19899-ABP_both
FLGLLGGLL
>ID19900-NO
FLGPIIKIATGILPTAICKILKKMLKLWKWKSSDVEYHLAKCTSDVL
>ID19901-NO
FLGPIIKIATGILPTAICKILKNVETLEMEII
>ID19902-NO
FLGRVLPPTRATASTHRSRL
>ID19906-ABP_both
FLGSVFKLASKVFPAVFGKV

>ID19907-ABP_both
FLGSVFKSASKVFPAVFGKV
>ID19908-ABP_both
FLGTINLSLCEEERDADEEERRDEPDESNVEVKKRFFFLSRIF
>ID19910-NO
FLGVIKIATGILPTAFCKFLKKC
>ID19911-ABP_both
FLGVLFKVLSKLL
>ID19912-ABP_both
FLGVVFKGASKVFPAVFGKV
>ID19913-ABP_both
FLGVVFKGASKVFPAVVGKV
>ID19914-ABP_both
FLGVVFKKASKVFGAVFGKV
>ID19915-ABP_both
FLGVVFKKASKVFKAVFGKV
>ID19916-ABP_both
FLGVVFKKASKVFPAVFGKV
>ID19917-ABP_both
FLGVVFKKASKVFPAVFKKV
>ID19918-ABP_both
FLGVVFKKASKVFPKVFGKV
>ID19919-ABP_both
FLGVVFKLASKVFGAVFGKV
>ID19920-ABP_both
FLGVVFKLASKVFGAVFKKV
>ID19921-ABP_both
FLGVVFKLASKVFGKVFGKV
>ID19930-ABP_both
FLGWLFKVASK
>ID19931-ABP_both
FLHHIVGLIHHGKLDMYRSNN
>ID19935-NO
FLIFIRVICIVIAKLKANLMCKT
>ID19938-ABP_both
FLKALFKALKKLL
>ID19940-ABP_both
FLKALFKVALKVL
>ID19942-ABP_neg
FLKALKKLL
>ID19944-NO
FLKDHRISTFKNWPF
>ID19946-NO
FLKFLKKFFKKLKY
>ID19948-ABP_both
FLKGIKGMLGKLL
>ID19949-ABP_both
FLKGIVGKLGKLF
>ID19950-ABP_both
FLKGIVGMLGKLF
>ID19951-ABP_both

FLKGIVGMLGKLL
>ID19953-ABP_both
FLKKAKKFGKAF
>ID19954-ABP_both
FLKKAKKFGKAFVKILK
>ID19955-ABP_both
FLKKVFKLASKVFPAVFGKV
>ID19956-ABP_neg
FLKLAKKLL
>ID19957-ABP_both
FLKLIPRKIVTAL
>ID19958-ABP_neg
FLKLLAKLL
>ID19960-ABP_neg
FLKLLKKAL
>ID19961-ABP_neg
FLKLLKKLA
>ID19962-ABP_neg
FLKLLKKLAAKFL
>ID19963-ABP_both
FLKLLKKLL
>ID19964-ABP_both
FLKVVFKKASKVFPAVFGKV
>ID19965-ABP_both
FLKVVFKLASKVFGAVFGKV
>ID19969-NO
FLNPFRWVINKYR
>ID19970-NO
FLNPFRWVINKYREWKNKKDS
>ID19972-NO
FLPAAFRLAAKIVPTVFCAISKKC
>ID19973-NO
FLPAAICLVIKTC
>ID19974-NO
FLPAALAGIGGILGKLFGK
>ID19975-NO
FLPAFYVWLLRLCQQCFVQFPKNVET
>ID19976-NO
FLPALAGIAGLLGKIF
>ID19978-NO
FLPAVIHVAANVLPTVFCAISKKC
>ID19979-NO
FLPAVIRAAANVLPTVFCAISKKC
>ID19980-NO
FLPAVIRVAADVLPTVFCAISKKC
>ID19988-NO
FLPFFASLLGKL
>ID19996-ABP_both
FLPFVGRIASKVVPSVIGKV
>ID20000-NO
FLPGPLRVAAKVGKAVFCEIFQKMLKLELEIIRCGISF

>ID20001-NO
FLPHVFAELSDRKGFVQGNGAVEALHDHFYPDWMDF
>ID20004-NO
FLPIAPMLGKYLGK
>ID20006-NO
FLPIASLLGKYLGK
>ID20009-NO
FLPIIAKLVSGLL
>ID20011-NO
FLPIIASLLGKLL
>ID20019-NO
FLPILGKILGGLL
>ID20024-NO
FLPIVASLAASFLPKIICKITKKC
>ID20025-ABP_both
FLPIVENCSLVCWENNQKC
>ID20028-NO
FLPIVGKLLSLF
>ID20029-ABP_both
FLPIVGLLKSLLK
>ID20032-ABP_neg
FLPIVKKLLKQLF
>ID20033-ABP_neg
FLPIVKKLLRGLF
>ID20034-ABP_neg
FLPIVKKLLRKLF
>ID20035-ABP_neg
FLPIVKKLLRQMF
>ID20036-ABP_neg
FLPIVKKVLKGLF
>ID20040-ABP_both
FLPIVTNLLSGLLGK
>ID20043-ABP_both
FLPKTLRKFFARIRGGRAAVLNA
>ID20044-ABP_both
FLPKTLRKFFARIRGGRAAVLNALGKEEQIGRASNSGRKAARKKK
>ID20045-ABP_both
FLPKTLRKFFARIRGGRAAVLNALGKEEQIGRASNSGRKCARKKK
>ID20046-ABP_pos
FLPLAGRVLSGAL
>ID20047-ABP_both
FLPLAMALGKLL
>ID20049-NO
FLPLFASLAANLLPKIICKIAKKC
>ID20053-NO
FLPLIGGILAKLL
>ID20054-NO
FLPLIGGILSQLL
>ID20058-ABP_both
FLPLIGRVLSGILGWKRKRFG
>ID20059-NO

FLPLIGSLLSKVLEK
>ID20067-ABP_both
FLPLLASLIGGML
>ID20068-NO
FLPLLASLVGGLL
>ID20072-ABP_pos
FLPLLGRVLSGLL
>ID20073-ABP_both
FLPLLLAGLPKFLCLVFKKC
>ID20074-ABP_both
FLPLLLAGLPSFLCLVFKKC
>ID20080-NO
FLPMIAKLLGGLL
>ID20082-NO
FLPMLAGLAANFLPTIVCKITKKC
>ID20083-NO
FLPMLAGLAANSLPKIFCKIIRKC
>ID20084-NO
FLPMLAKLLSGFL
>ID20085-NO
FLPMLAKLLSGFLGK
>ID20087-NO
FLPVAGVAAEMQHVFCAISKKC
>ID20088-NO
FLPVCLRGKISCALSPLC
>ID20091-ABP_both
FLPVLVKVFRYSKKTAAGCF
>ID20093-NO
FLPYPYY
>ID20094-NO
FLQPQRF
>ID20098-ABP_both
FLRFIGSVKHGKGHLVHHIGVAL
>ID20101-ABP_both
FLRRIRV
>ID20102-ABP_both
FLRRIRVTPVVNPPFLQQT
>ID20103-ABP_both
FLRRIRVTPWVNPPFLQQT
>ID20106-NO
FLSAIASMLGKFLGK
>ID20110-ABP_both
FLSFPTTKTYFPH
>ID20111-ABP_both
FLSFPTTKTYFPHF
>ID20112-ABP_both
FLSFPTTKTYFPHFDLSHGSAQVKGHGAKVAAAL
>ID20116-NO
FLSGILKLASKIPSVLCAVLKIVET
>ID20117-NO
FLSGILKLASKIPSVLCAVLKKLLKLKLEII

>ID20119-NO
FLSGILKLASKIPSVLVQF
>ID20120-ABP_both
FLSGIVGMLGKLFGK
>ID20121-ABP_both
FLSHIAGFLSNLFGK
>ID20122-ABP_both
FLSLIPAAISAVSALANHF
>ID20123-NO
FLSLIPGAISAIASLFK
>ID20126-ABP_both
FLSLIPHIVSGVASLAIHF
>ID20128-ABP_both
FLSLIPKAIKAVGVKAKKF
>ID20129-ABP_both
FLSLIPKIAGGIAALAKHL
>ID20131-ABP_both
FLSMIPHIAGGIASLVKNL
>ID20132-ABP_both
FLSMIPHIVSGVAALAKHL
>ID20137-NO
FLSSRLQDLYSIVRRADRAA
>ID20140-NO
FLTFPGMTFGKLLGK
>ID20145-NO
FLVGILPRMRGFITPFLKKVR
>ID20146-NO
FLVMFLSG
>ID20147-NO
FLVPP
>ID20151-NO
FLWSLIPSAISAVTSLIKK
>ID20152-NO
FMEIET
>ID20153-NO
FMGLSHFDEN
>ID20155-ABP_both
FMKVLAVFGSVVTSAPKASK
>ID20158-NO
FMPILPCSRFKRC
>ID20164-NO
FNADDLTLRF
>ID20165-NO
FNDYRL
>ID20167-NO
FNEFV
>ID20169-NO
FNFNPFGLRF
>ID20171-NO
FNKLKQGSSKRTCAKCFRKIMPSVHELDERRRGANRWAAGFRKCVSSICR
>ID20176-NO

FNVPLYEFSY
>ID20178-NO
FPAFTTGFGHS
>ID20180-NO
FPCEGKKCL
>ID20186-NO
FPELSQDLISNLLEK
>ID20189-NO
FPFEVFGK
>ID20193-ABP_both
FPFFNQYVKL
>ID20197-ABP_both
FPFLKLSLKIPKSAIKSAIKRL
>ID20199-NO
FPIGRRDFDMLRCMLGRVYRPCWQV
>ID20203-NO
FPLIASLAGNVVPNIFCKITKRC
>ID20204-NO
FPLPSCVYTRTC
>ID20205-ABP_both
FPLTCPTKWWKG
>ID20206-ABP_both
FPLTWPTKWWKG
>ID20208-NO
FPMKKSLLLIFFLGTINLSFCEEERNAEEEKRDGDDEMDVEVQKR
>ID20210-NO
FPNKPDSPGEDAPAEDLARYLSAVRHYINLITRQRY
>ID20211-NO
FPNQHLCGSHLVEALYLVCGEKGFYYIPRM
>ID20213-NO
FPPKPDNPGDNASPEQMARYKAAVRHYINLITRQRY
>ID20215-NO
FPQSFLPRG
>ID20216-NO
FPQYLQY
>ID20217-NO
FPRPGGGGNGDFEEIPEEYL
>ID20218-NO
FPRPRICNLACRAGIGYKYPFCHCR
>ID20221-NO
FPSSLIIPPLPN
>ID20223-ABP_both
FPTTKTYFPH
>ID20225-ABP_both
FPVGRVHRLLRK
>ID20226-ABP_both
FPVTWKWWKWWKG
>ID20227-ABP_both
FPVTWPTKWWEG
>ID20228-ABP_both
FPVTWRTKWWKG

>ID20229-ABP_neg
FPVTWRWWRWWRG
>ID20234-ABP_both
FPWFNQYVKL
>ID20237-ABP_both
FPWWNQYVKL
>ID20246-NO
FQHPSFI
>ID20248-NO
FQKPKR
>ID20249-NO
FQKVVA
>ID20250-NO
FQKVVAG
>ID20251-NO
FQKVVAK
>ID20255-NO
FQPQPLIYP
>ID20256-NO
FQPYDHPAEVSY
>ID20257-NO
FQSLF
>ID20262-ABP_both
FQWQRNMRKVRGSRRRRG
>ID20264-NO
FQYSRGWTN
>ID20266-NO
FRADHPFL
>ID20267-NO
FRADPFL
>ID20268-NO
FRAHPFL
>ID20273-ABP_both
FRCRRRFCRF
>ID20276-NO
FREGD
>ID20277-ABP_both
FRFKFRFK
>ID20278-ABP_both
FRFKRFRKKGKKLFKKVSPPILLLHL
>ID20281-ABP_both
FRFRCRRRFCRFRF
>ID20285-NO
FRGLMHY
>ID20288-NO
FRHEA
>ID20290-ABP_both
FRIRVRVAKKFGKAFVGEIM
>ID20291-ABP_both
FRIRVRVFKRIVQRIKDFLR
>ID20293-ABP_both

FRIRVRVKWKLFKKI
>ID20294-ABP_both
FRKKWFW
>ID20298-NO
FRLDEEFQGPIASQVRRQFLFRPRN
>ID20299-NO
FRLHF
>ID20300-ABP_both
FRLMLRLLRW
>ID20301-NO
FRLPFQFFGANEDFNSGLT
>ID20303-NO
FRPNRAQDYNTN
>ID20306-NO
FRQFYQL
>ID20307-ABP_both
FRRFFKWFRRFFKFF
>ID20308-ABP_both
FRRFFKWFRRPFKFF
>ID20310-ABP_both
FRRFFKWPRRPFKFF
>ID20311-ABP_both
FRRFRKWFRRFLKLF
>ID20315-ABP_both
FRRPFKWPRRFFKFF
>ID20320-NO
FRVGVADV
>ID20321-NO
FRVPLRIRPCVVAPRLVMVRHTFGRIARWVAGPLETR
>ID20329-NO
FSCDHSACAVRCLAQRRKGGKCKNGDCVCR
>ID20330-NO
FSDARL
>ID20331-ABP_neg
FSDKIAK
>ID20332-NO
FSDKIAKYIPIQ
>ID20333-NO
FSDMRL
>ID20334-NO
FSDSQLCRNNHGHCRRLCFHMESWAGSCMNGRLRCCRFSTKQPFSNPKHS
>ID20335-NO
FSDTRL
>ID20336-ABP_both
FSEAIKKIIDFLG
>ID20337-NO
FSEEEKEPE
>ID20338-NO
FSEFLKQYLGMSPR
>ID20340-NO
FSFKRLKGFAKKLWNSKLARKIRTKGLKYVKNFAKDMLSEGEEAPPAAEP

>ID20342-ABP_both
FSFLSRIF
>ID20343-NO
FSGASPYGL
>ID20344-ABP_both
FSGGDCRGLRRRCFCTR
>ID20345-NO
FSGLMSEGSSLEA
>ID20346-NO
FSGTYNFGL
>ID20347-NO
FSHDRNFLRF
>ID20348-NO
FSKYERQKDKRPYSERKNQYTGPQFLYPPERIPPQKVIKWNEEGLPIYEI
>ID20350-ABP_both
FSLIPSAIGGLISA
>ID20352-NO
FSLLDAR
>ID20353-NO
FSLLLTQQPRPVL
>ID20358-NO
FSPRL
>ID20361-NO
FSQAQGKVDMPLPRQ
>ID20363-NO
FSQAQGKVDMPLPRQRTSS
>ID20364-NO
FSQAQGKVDMPLPRQRTSSRSSERWAPKS
>ID20365-NO
FSRYARMRDSRPWSDRKNNYSGPQFTYPPEKAPPEKLIKWNNEGSPIFEM
>ID20366-ABP_pos
FSSLSLCSLGCTGVKNPSFNSYCC
>ID20372-NO
FTCCRRGTCSQHC
>ID20373-NO
FTDIRL
>ID20374-NO
FTDVDFIK
>ID20375-NO
FTESQS
>ID20377-NO
FTIAEPYIHPCMKGFCSFKSECANKCIFMGHHKGGDCIGGLDGIYCCCLA
>ID20378-NO
FTLKKSLLVLFFLGTISLSLCQEERAADEEDNGEVEE
>ID20379-NO
FTMKKSLFLILFLGAIPLSMC
>ID20381-NO
FTMKKSLLFIFFLGTISLSLC
>ID20382-NO
FTMKKSLLLIFFLGTINLSLCEEERNAEEEKRDGDDEMDVEVQKR
>ID20384-NO

FTMKKSLLLIFFLGTISLSLCEQER
>ID20387-NO
FTMKKSPLLLFFLGTISLSLC
>ID20389-NO
FTPNWGT
>ID20390-NO
FTPSVSFSQNGGVVEAAAQRGYIYKKYPKGAKVPNKVKMLVNIRGKQTMR
>ID20391-ABP_both
FTPVLQADFQKVVAGVANALAHRYH
>ID20392-ABP_both
FTQGVGNPVSCARNKGICVPSRCPGNNRQIGTCLGPPVKCCRRK
>ID20394-NO
FTRKFLKFLHL
>ID20395-NO
FTSKNYLRF
>ID20396-NO
FTSVKMPRDEHWPYN
>ID20397-NO
FTTGFGHS
>ID20399-NO
FTTVCRQPRGHEAIVCGSGK
>ID20404-NO
FTYKNFFWLPEL
>ID20407-NO
FVAPFPEV
>ID20408-NO
FVAPFPEVF
>ID20411-NO
FVAPFPEVFGKEKVNE
>ID20413-NO
FVAPFPEVFGKEKVNELSKDIGSE
>ID20417-NO
FVDVRL
>ID20418-NO
FVEPIP
>ID20423-NO
FVGGSRY
>ID20424-ABP_both
FVGGVASKVVPSVYCKVSKK
>ID20426-NO
FVGMR
>ID20427-ABP_both
FVGSLASKVVPSVFGAIKTK
>ID20429-NO
FVKQHLCGPHLVEALYLVCGERGFFYTPKS
>ID20430-NO
FVKQHLCGSHLVEALYLVCGERGFFYTPMS
>ID20431-NO
FVLGVVTKLLPSLFCMITKKC
>ID20435-NO
FVNKHLCGSHLVDALYLVCGDRGFFYTPMA

>ID20436-NO
FVNPEAGS
>ID20437-NO
FVNPQAGS
>ID20438-NO
FVNQHLCGPHLVEALYLVCGERGFFYAPKT
>ID20439-NO
FVNQHLCGSHLVEALYILVCGERGFFYTPMS
>ID20442-NO
FVNQHLCGSHLVEALYLVCGNDGFFYRPKA
>ID20443-NO
FVNSRY
>ID20444-NO
FVNTIRLLINKAREWNNKQSS
>ID20445-NO
FVPAILCSILKTC
>ID20446-NO
FVPFFTQSDIQKMQEKERNKGQ
>ID20447-NO
FVPGFICTVLKRC
>ID20449-NO
FVPIFTHSELQKIREKERNKIRNKGQ
>ID20450-NO
FVPIFTHSELQRIREKERNKGQ
>ID20455-NO
FVPIFTYGEVQRMQEKERYKGQ
>ID20460-NO
FVPIFTYSELQRMQERERNRGH
>ID20461-NO
FVPIFTYSELRRTQEREQNKRL
>ID20464-ABP_both
FVPWFSKFLKRIL
>ID20467-NO
FVQHRPRDCESINGVCRHKDTVNCREIFLADCYNDEQKCCRK
>ID20470-NO
FVSRHLCGSNLVETLYSVCQDDGFFYIPKD
>ID20471-ABP_both
FVTNSKRLAEGIEKGVGNSILIKVN
>ID20472-NO
FVTRGCPRRLVARLIRVMVPRR
>ID20475-NO
FVVAPFPEVF
>ID20476-ABP_both
FVVKKKKKVF
>ID20484-ABP_both
FWFHWK
>ID20485-ABP_both
FWGALAKGALKLIPSLVSSFT
>ID20492-NO
FWKTFTSC
>ID20497-NO

FWPFM
>ID20535-ABP_both
FWRRIRV
>ID20536-ABP_both
FWRRIRVTPVVNPPFLQQT
>ID20547-NO
FWTERAGWAY
>ID20564-NO
FYANRY
>ID20565-NO
FYAPFSPRL
>ID20566-NO
FYCPGVGCR
>ID20567-NO
FYCVIERLGVCLY
>ID20599-NO
FYEIIMDIEQNNVQGKKGLQQLQKWEDWVNGWIGNI
>ID20601-NO
FYNPRDLVHSGFRPRLCSVSGVEGYPPCVESHSDRKMKNLLDDLFGL
>ID20602-NO
FYPEL
>ID20603-ABP_both
FYPFG
>ID20607-NO
FYSQRY
>ID20608-NO
FYTGG
>ID20609-ABP_both
FYTHVFRLKKWMQKVIDRFGG
>ID20613-ABP_both
GAAAAGKVVGGKGGK
>ID20614-NO
GAACLCKSDGPNTRGNSMSGTIWVFGCPSGWNNCEGRAIIGYCCKQ
>ID20615-NO
GAAELPCSADWW
>ID20616-ABP_both
GAALAGLAKILCLWAKEFTGAFKKLNKKFAMKKK
>ID20618-NO
GAAPKFF
>ID20620-NO
GACLGFGKSCNPSNDQCCKSSSLACSTKHKWCKYEL
>ID20621-NO
GACRWFLGGCKSTSDCCEHLSCKMGLDYCAWDGTF
>ID20623-NO
GADFYSWG
>ID20630-NO
GAFTDLLKGVAKQAGIKILGIAQCKLAKTC
>ID20635-NO
GAHKNYLRF
>ID20637-NO
GAILCNLCKDTVKLVENLLTVDGAQAVRQYIDNLCGKASGFLGTLCEKIL

>ID20638-ABP_both
GAKALTKAATAFTKFYKTIW
>ID20639-NO
GAKERAHQ
>ID20647-NO
GAKPCGGFF
>ID20648-ABP_both
GAKYAKIIYNYLKKIANALW
>ID20649-ABP_both
GAKYAKYIYNFYKYIAKYIW
>ID20651-ABP_both
GALFGLASKVFPAVFGAFKK
>ID20653-ABP_both
GALFKVASKVLPS
>ID20654-NO
GALFLAFLAAALSLMGLWSQPKKKRRV
>ID20655-NO
GALFLGFLGAAGSTMGAWSQPKKKRKV
>ID20660-ABP_both
GALKRIKTLL
>ID20664-NO
GAMPGVLRF
>ID20665-ABP_both
GANAAKKFANLIKKIFNYIW
>ID20666-ABP_both
GANAAKKFATIAKKFINYLW
>ID20667-ABP_both
GANAAKKLATFAKKIFTAYW
>ID20668-ABP_both
GANALKKYFTILKKFFKLAW
>ID20669-NO
GANDFMRF
>ID20670-ABP_both
GANLAKKFYTYINKFINYAW
>ID20672-NO
GANPCALYY
>ID20674-NO
GAPGLPGP
>ID20675-NO
GAPGPAGPGGIPGERG
>ID20680-NO
GAPRFI
>ID20681-NO
GAPRFL
>ID20682-NO
GAPRFV
>ID20683-NO
GAPSITCVRRAF
>ID20686-NO
GAPVCGETCVGGTCNTPGCTCSWPVCTRN
>ID20687-NO

GAQFSSWG
>ID20688-NO
GARECESGGPGMRKLCTQIN
>ID20689-NO
GARGCNTGYTAYGCNCC
>ID20697-NO
GASGLIAFPRL
>ID20698-NO
GASGLIAFPRV
>ID20699-NO
GASGLIPFPRL
>ID20700-NO
GASGLIPVMRN
>ID20701-NO
GASGLISFPRV
>ID20705-ABP_pos
GATAIKQVKKLWKKKGG
>ID20706-NO
GATIKKCVVDVKLSKPCTFQECQPLCLQKYNGNGLCPGDDNNICACVYNC
>ID20707-ABP_both
GATYAKKIIKTITKIATTAW
>ID20708-ABP_both
GAVDILKGAAKDIAGHLASKVMNKL
>ID20709-ABP_both
GAVFGVASKVVPSVFSAIKK
>ID20710-NO
GAVGPSG
>ID20713-NO
GAVPAAQFSPRL
>ID20714-NO
GAVPAAQWFSPRL
>ID20718-ABP_both
GAVSGLLTNL
>ID20719-ABP_both
GAVSGLLTNLGL
>ID20722-NO
GCASDPRCRYRAR
>ID20725-NO
GCCADPRCRYRCR
>ID20726-NO
GCCADPSCSILMPYFCI
>ID20727-NO
GCCAHLPCALMYTACSW
>ID20731-NO
GCCAMLTCCV
>ID20737-NO
GCCARAACAGIHQELCGGRR
>ID20738-NO
GCCARLTCCV
>ID20741-NO
GCCCNPACGPNYGCGTSCS

>ID20743-NO
GCCCNPACGPNYGCGTSCSRTL
>ID20744-NO
GCCDDPPCRARYPFLCI
>ID20746-NO
GCCDDPSCRAENPFLCSWM
>ID20747-NO
GCCDDPSCSIHMPFFCF
>ID20749-NO
GCCDYDWCDEFCYCCE
>ID20753-NO
GCCFHPVCYINLLEMCRQR
>ID20754-NO
GCCGAFACRFGCTPCC
>ID20760-NO
GCCGKYPNAACHPCGCTVGRPPYCDRPSGG
>ID20762-NO
GCCGNPSCSIHIPYVCN
>ID20765-NO
GCCGSFACRFGCVPCCV
>ID20770-NO
GCCHLLACRMGCSPCCW
>ID20772-NO
GCCHPSTCHMRKGCSRCCS
>ID20773-NO
GCCHPSTCHVRKGCSRCCP
>ID20776-NO
GCCIEPLCYQYDCDCCRYL
>ID20777-NO
GCCIVPWCTGCYCCH
>ID20781-NO
GCCPFPACTHTIICRCC
>ID20783-NO
GCCPIGPCMQSVCSPCCP
>ID20785-NO
GCCPPQWCGPDCTSPCC
>ID20786-NO
GCCPPSLCDPGCDEGCCPVVTPAC
>ID20787-NO
GCCPRSFLCC
>ID20790-NO
GCCRWPCPSICGMARCCSS
>ID20809-NO
GCCSDPKCRYRCR
>ID20812-NO
GCCSDPPCIANNPDLC
>ID20824-NO
GCCSDPRCAYRCR
>ID20830-NO
GCCSDPRCKHQC
>ID20848-NO

GCCSDPRCNYDHPEICGGAAGG
>ID20860-NO
GCCSDVRCRYRCR
>ID20866-NO
GCCSEPRCRYRCR
>ID20868-NO
GCCSFPACRKYRPEMCG
>ID20871-NO
GCCSHPACAGNNPDIC
>ID20874-NO
GCCSHPACAGSNAHIC
>ID20879-NO
GCCSHPACNVDHPEIC
>ID20880-NO
GCCSHPACNVNNPHIC
>ID20884-NO
GCCSHPACSGKYQPYCRPS
>ID20886-NO
GCCSHPACSGNNPEFCRQ
>ID20888-NO
GCCSHPACSGNNREYCRES
>ID20904-NO
GCCSHPPCAMNNPDYC
>ID20905-NO
GCCSHPPCAQNNQDYC
>ID20906-NO
GCCSHPPCFLNNPDYC
>ID20914-NO
GCCSLPPCAASNPDYC
>ID20922-NO
GCCSNPACMVNNPQIC
>ID20927-NO
GCCSNPPCYANNQAYCN
>ID20932-NO
GCCSNPVCFATHSNLC
>ID20941-NO
GCCSNPVCHLEHSNLCGAGGAAG
>ID20944-NO
GCCSNPVCHLEHSNMC
>ID20947-NO
GCCSNPVCHVEHSYMC
>ID20948-NO
GCCSNPVCWQNNAEYCRES
>ID20950-NO
GCCSPWNCIQLRACPCCPN
>ID20957-NO
GCCSRPPCIANNPDIC
>ID20970-NO
GCCSYPPCFATNPDCA
>ID20972-NO
GCCSYPPCFATNPDCAGAGA

>ID20973-NO
GCCSYPPCFATNPDCAGG
>ID20975-NO
GCCSYPPCFATNPDCGAGAAG
>ID20976-NO
GCCSYPPCFATNPDCGGAA
>ID20977-NO
GCCSYPPCFATNPDCGGAAAGG
>ID20978-NO
GCCSYPPCFATNPDCGGAAG
>ID20981-NO
GCCSYPPCFATNSDC
>ID20982-NO
GCCSYPPCFATNSDYC
>ID20983-NO
GCCSYPPCFATNSGYC
>ID20984-NO
GCCSYPPCNVSYPEICG
>ID20985-NO
GCCTPPRKCKDRACKPARCCGP
>ID20986-NO
GCCTYPPCAVLSPLCD
>ID20987-NO
GCCVKQGCWNVETCTCCPADPYIPKPFPTRST
>ID20988-NO
GCCVYPPCAVNHPDIC
>ID20989-NO
GCCVYPPCAVNHPDICRG
>ID20991-NO
GCDPKWTICNNDAECCFPYSCENSNCQ
>ID20994-NO
GCFEDWSRCSPSTSRGTGVLWRDCDSYCKVCFKADRGECFDSPSLNCPQR
>ID20997-NO
GCGGVCAYGESCPSSCNTCYSAQCTAQ
>ID20999-NO
GCGYLGEPCCISPKRAYCHGDLECNNVAMCVN
>ID21000-NO
GCGYLGEPCCVAPKRAYCHGDLECNNIAMCVN
>ID21003-NO
GCIAKNKECAWFSGEWCCGALSCKYSIKRNLKICV
>ID21004-NO
GCIEDKKYCGILPFANSGVCCSYLCIFVCVPKAP
>ID21005-NO
GCIEIGGDCDGYQEKSYCQCCRNNGFCS
>ID21006-NO
GCIGRNESQKKDNVYKFKE
>ID21012-ABP_both
GCKKYRRFRWKFKGKFWFW
>ID21015-NO
GCLDPGYFCGTPFLGAYCCGGICLIVCIET
>ID21016-NO

GCLEFWWKCNPNDDKCCRPKLKCSKLFKLCNFSSG
>ID21017-NO
GCLEVDYFCGIPFANNGLCCSGNCVFVCTPQ
>ID21019-NO
GCLGDKCDYNNGCCSGYVCSRTWKWCVLAGP
>ID21022-NO
GCLPDEYFCGFSMIGALLCCSGWCLGICMT
>ID21023-NO
GCNGRC
>ID21027-NO
GCPADCPNTCDSSNECSPNFP
>ID21029-NO
GCPAECPDTCSSSGSCAPDFI
>ID21036-NO
GCPYNPKC
>ID21037-NO
GCQKFFWTCHPGQPPCCSGLACTWPTEICIDG
>ID21038-ABP_both
GCRALCYKQRCVTYCRGA
>ID21039-NO
GCREGGEFCGTLYEERCCSGWCFFVCV
>ID21040-ABP_both
GCRFTVKPYIKRIQLHYKGKMWCG
>ID21042-ABP_both
GCRRLCWKQRCVTYCRGR
>ID21043-ABP_both
GCRRLCWRQRCVTWCRGR
>ID21045-ABP_both
GCRRLCYKQRCVTYCRGPPR
>ID21049-ABP_both
GCRRWKKFRWRYRGKFWFWCG
>ID21050-NO
GCRRYKKFKWRYRGRFWFWCG
>ID21055-NO
GCSKPIKGIHGICCRD
>ID21057-NO
GCSRWIIGIHDKFVEIKKNYWNLNWKSGILFS
>ID21058-NO
GCSRWIIGINGQVCRD
>ID21059-NO
GCSRWIIGINGRICRD
>ID21060-NO
GCSRWIISINGRICRD
>ID21061-NO
GCSVSSVGALCTHV
>ID21068-NO
GCTPEYCSMWCKVKVSQNYCVKNCKCPGR
>ID21070-NO
GCTPPGGACGGHAHCCSQSCNILASTCNA
>ID21071-NO
GCTPRNGACGYHSHCCSNFCHTWANVCL

>ID21072-NO
GCTPRNGFCRYHSDCCSNFCHTWAIMCL
>ID21077-NO
GCTRWIIGIKLCR
>ID21079-NO
GCVANCQANQTGIDCIKYCGIGI
>ID21081-NO
GCVGENQQCADWAGPHCCSGYYCTCRYFPKCICRKDS
>ID21082-NO
GCVGHRKSCEHDKKNGCCYFMTCNCWHPMGQ
>ID21083-NO
GCVLLPWC
>ID21084-NO
GCWICWGPNACCRGSVCHDYCPS
>ID21085-NO
GCWLCLGPNACCRGSVCHDYCPR
>ID21087-ABP_both
GCWTKSIPPKPCF
>ID21099-NO
GDACSLLNGDDCGPGELCCTPSGDHQGTCETSCW
>ID21102-NO
GDAQTIQEVFEMFALDSDGCCWHPACGRHYC
>ID21104-NO
GDCSCEGQICKCGYRVSPGKSGCACTCRNAK
>ID21108-NO
GDEECNEHCEDRNKECCGRTNGHPRCANVCF
>ID21109-NO
GDEECNEYCDDRNKECCGRTNGHPRCANVCF
>ID21110-NO
GDEEVAKFIEREREAGRLDLSKFP
>ID21111-NO
GDEEVSKFIEREREAGRLDLSKFP
>ID21112-NO
GDEEYSKFIELARENIAKGCKVNCYP
>ID21114-NO
GDEEYSKMAAEREREAGRLDLSKFP
>ID21115-NO
GDFAFNPRL
>ID21116-NO
GDFAFSPRL
>ID21117-NO
GDFENFFLKQSKSVPRI
>ID21119-NO
GDGRLYAFGL
>ID21120-NO
GDGVDITRIR
>ID21125-NO
GDLAFAF
>ID21130-NO
GDNFMRF
>ID21131-NO

GDNKPPKKGPPNGCFGHKIDRIGSHSGLGCNKVDDNKG
>ID21133-NO
GDPFLRF
>ID21138-NO
GDRNFLRF
>ID21139-NO
GDSEVPGMWFGPRL
>ID21140-NO
GDSVCASYF
>ID21143-NO
GDTRRQIL
>ID21144-NO
GDVIDTDRDIDR
>ID21146-NO
GDVNWVDVGKTVATNGAGVIGGAFGAGLCGPVCAGAFAVGSSAAVAALYD
>ID21147-NO
GDVPGVLRF
>ID21151-ABP_neg
GDVPPGIRNTICRMQQGICRLFFCHSGTGQQHRQRCG
>ID21152-NO
GDVWLFLTSTSHFAR
>ID21153-NO
GDVYADAAPDLFDFLDSSVTTARTINA
>ID21164-NO
GECCHPACGQNTSC
>ID21165-NO
GECCTDCAQTAAANYC
>ID21169-NO
GECLGWSNYCTSHSICCSGECILSYCDIW
>ID21174-NO
GEEEHSKYQECLREIRVNKVQQEC
>ID21177-NO
GEEEVAKFIELARENIAKGCKVNCYP
>ID21179-NO
GEEEVAKMAAELARENIAKGCKVNCYP
>ID21180-NO
GEEEVAKMAAEREREAGRLDLSKFP
>ID21183-NO
GEEEYAKMAAELARENIAKGCKVNCYP
>ID21186-ABP_both
GEFKKIVQKIKDFLKNLV
>ID21187-ABP_both
GEFKRIVQRIKDFLRNLV
>ID21189-ABP_both
GEFRRIVQRIRDFLRNLV
>ID21191-NO
GEGGP
>ID21192-NO
GEGIIAEYMNSESFPHEGSLSNFFLKASKAVPRL
>ID21193-NO
GEGKMFWRCYFNAVSCF

>ID21195-ABP_both
GEILCNLCTGLINTLENLLTTK
>ID21196-ABP_both
GEILCNLCTGLINTLENLLTTKGADKVK
>ID21197-NO
GEILCNLCTGLINTLENLLTTKGADKVKDYISSLCNKASGFIATLCTKVL
>ID21198-ABP_both
GEILCNLCTGLINTLENLLTTKRKRQQ
>ID21199-NO
GEKESRPLSSYPGSV
>ID21200-ABP_both
GEKVPKGKKGKADAGKEGNNPAENGDAKTDQAQKAEGAGDAK
>ID21201-NO
GENFAIKNLKTIPRI
>ID21203-NO
GEPEVAKWAEGLREKAASN
>ID21205-NO
GEPPVAKMAAPLARPNIAKGCKVNCYP
>ID21209-NO
GEQIAQLIAGYIDIILKKKKSK
>ID21213-NO
GETDPNTQLLNDLGNNMAWGAALGAPGGLGSAALGAAGGALQTVGQGLID
>ID21234-ABP_both
GFALAGLARILCLWFREFSGFFRRLNRRFAMRRR
>ID21235-ABP_both
GFARIVQRIKDFLRNLV
>ID21237-NO
GFASFLGKALKAALKIGANMLGGAPQQ
>ID21239-NO
GFASLPILKNG
>ID21241-NO
GFCAEAGIKCNDIHCCGNLKCKAVGSNRV
>ID21246-NO
GFCAQKGIKCHDIHCCTNLKCVREGSNRVCRKA
>ID21388-ABP_both
GFCIPIRCPGHTRQIGTCFGPRVKSCRKW
>ID21749-ABP_neg
GFCWYVCYRNGVRVCYRRCN
>ID21765-ABP_both
GFFALIPKI
>ID21766-ABP_both
GFFALIPKIISSPLF
>ID21767-ABP_both
GFFALIPKIISSPLFKTLLSA
>ID21770-ABP_both
GFFCPYNGYCDRHCRKKLRRRGGYCGGRWKLTCICIMN
>ID21772-ABP_both
GFFDRIKALTKNVTLELLNTITCKLGVTGG
>ID21781-NO
GFFPLIKGPAKLIAKTVAKKPAKTGLEFML
>ID21782-NO

GFFTLIKGAAKLIGKTVAKEAGKTGLEIMACKITNQC
>ID21783-NO
GFFWIFKTAAKFVAKNLFNQPAKAGLDHLACKVKNEC
>ID21785-NO
GFGALFKFL
>ID21786-NO
GFGALFKFLAKKVAKTVAKQVAKKQME
>ID21787-ABP_pos
GFGCNGPWNEDDLRCHNHCKSIKGYKGGYCAKGGFVCKCY
>ID21790-ABP_pos
GFGCNGPWSEKDMHCHNHCKSIKGYKGGYCAKGGFICKCY
>ID21791-NO
GFGCPFDERACHAHCQSVGRRGGYCGNFRMTCYCYKN
>ID21792-ABP_pos
GFGCPFNANECHAHCLSIGRKFGFCAGPLRATCTCGKQ
>ID21793-ABP_pos
GFGCPFNKNECHAHCLSIGRKFGFCAGPLRATCTCGKQ
>ID21795-NO
GFGCPFNQGACHRHCQSIGRKGGYCSGLFKQTCTCYRH
>ID21796-ABP_pos
GFGCPFNQGQCHKHCQSIRRRGGYCDGFLKTRCVCYR
>ID21802-NO
GFGCYRSCWKAGHDEETCKAECS
>ID21803-NO
GFGCYRSCWKAGHDEETCKEECS
>ID21806-NO
GFGDEMSMPGVLRF
>ID21807-ABP_both
GFGDSVKEGLKNAAVTILNKIKCKISECGGA
>ID21808-ABP_both
GFGDSVKEGLKNAAVTILNKIKCKISECPPA
>ID21811-ABP_both
GFGLGGLARILCLGNRQWSNFFKKLNRKCAMVKK
>ID21813-NO
GFGMLFKFL
>ID21814-NO
GFGMLFKFLAKKVAKKLVSHVAQKQLE
>ID21815-NO
GFGSFLGKALKAALKIGANALGGAPQQ
>ID21817-ABP_both
GFGSFLGSLFKTGLKIIPKLLPSIQQ
>ID21818-NO
GFGSLFKFL
>ID21819-NO
GFGSLFKFLAKKVAK
>ID21820-NO
GFGSLFKFLGKKLAKTVAKQAAKKQME
>ID21821-NO
GFGSLFKFLGKKLLKTVAKQAAKKQME
>ID21822-NO
GFGSLFKFLGKKVLK

>ID21824-NO
GFGSLLGKALKAGLKLGANLLGGAPQQ
>ID21830-NO
GFHDHGPCDPPSHK
>ID21832-ABP_both
GFIATLCTKVLDFGIDKLIQLIEDK
>ID21834-ABP_both
GFIFH
>ID21835-ABP_both
GFIFHIIKG
>ID21837-ABP_both
GFIGKLASKVVPSVYCKVTG
>ID21842-NO
GFISGILGAGKKIVWGLSGLC
>ID21846-ABP_both
GFKLGRKLVKVFKWII
>ID21847-NO
GFKNVALSTARGF
>ID21849-NO
GFKNVQLSTARGF
>ID21851-ABP_both
GFKRIVQKIRDFLRNLV
>ID21854-ABP_both
GFKRLVQRLKDFLRNLV
>ID21858-NO
GFLDIVKGVGLVALGAVSKS
>ID21859-NO
GFLDIVLHVGLAAGKAALNAVNEAVNQ
>ID21860-NO
GFLDKVIKVCGRDLVRIKIDICGKILLGDMTTG
>ID21861-NO
GFLDKVKEFGKTAAKSVAQGLLNAASCKLAKTC
>ID21863-NO
GFLDTLKNMAINAAKGAGVSVLNALSCKLDKSC
>ID21865-NO
GFLDTVKEFGKAAAKNIAQSLLSTASCKLAKTC
>ID21866-NO
GFLDVIKDTAQNLFATVLDKIKCKVTKC
>ID21867-NO
GFLDVLKGVGKAALGAVTHHINNLVNQQ
>ID21868-NO
GFLDVVHVGKAVGKAALNAVNDLVN
>ID21869-ABP_both
GFLEKLKKGAKDFASALVNSIKGT
>ID21877-ABP_both
GFLLKLKLGAKLFASAFVNSIK
>ID21878-NO
GFLNGLLGAGEKKGRGINGVG
>ID21880-NO
GFLRGCWTKSFPPKPCLFKP
>ID21882-NO

GFLRGCWTKSFPPKPCLKR
>ID21883-NO
GFLRGVLGGGKKIVCGLSGLC
>ID21884-NO
GFLSGILGAGKHIVCGLSGLC
>ID21885-NO
GFLSGILGAGKHIVCGLSGVC
>ID21900-NO
GFMDTARERCKEPVMQRIAICRYLTQKKN
>ID21902-NO
GFNEIVQDIEDFLQNLV
>ID21908-NO
GFPGTPGLPGF
>ID21911-NO
GFQNNAEGPV
>ID21913-NO
GFRDGSADRISHGF
>ID21915-NO
GFRGDAASRVAHGF
>ID21917-ABP_both
GFRKIVQKIRDFLRNLV
>ID21919-NO
GFRMNSSNRVAHGF
>ID21922-ABP_both
GFRTAHVDLVCPDNPD
>ID21923-NO
GFSKLYF
>ID21925-ABP_both
GFSSIFRGVAKFASKGLGKDLAKLGVDLVA
>ID21929-NO
GFWDNIKNFGKTFALNAIEKLKCKITGGCPP
>ID21931-NO
GFWDTIKQAGKKFFLNVLDKIRCKVAGGCRT
>ID21934-ABP_both
GFWGKLLEGVKKAI
>ID21935-ABP_both
GFWGKLWEGVKNAIKKK
>ID21936-ABP_both
GFWGKLWKPVKKAI
>ID21938-ABP_both
GFWSSVWDGAKNVGTAI
>ID21944-NO
GFYGG
>ID21946-NO
GGAGEPLAFSPDMLSLRF
>ID21948-NO
GGALFRF
>ID21949-NO
GGAPATSANAAGAAAIVGALAGIPGGPLGVVVGAVSAGLTTGIGSTVGSG
>ID21950-NO
GGARAFLTEM

>ID21951-NO
GGARAFVENS
>ID21952-NO
GGARAFYDE
>ID21953-ABP_both
GGARSLGRKALRAWKKYGPAIVPIIRI
>ID21955-ABP_both
GGARSLGRKILRAWKKYGPIIVPIIRI
>ID21956-NO
GGARVFQGFEDE
>ID21959-NO
GGCCSHPACAANNQDYC
>ID21960-NO
GGCCSHPACAVNHPELC
>ID21962-NO
GGCCSHPACFASNPDYC
>ID21964-NO
GGCCSHPACQNNPDYC
>ID21966-NO
GGCCSHPVCYFNNPQMCR
>ID21967-NO
GGCCSHPVCYVNYPQICR
>ID21968-NO
GGCCSRPPCILEHPEIC
>ID21971-NO
GGCCSYPPCAVSNPQHC
>ID21972-NO
GGCCSYPPCIANNPLC
>ID21973-NO
GGCIKWNHSCQTTTLKCCGKCVVCYCHTPWGTNCRCDRTRLFCTED
>ID21975-NO
GGCLQILPTLSECFGR
>ID21976-NO
GGCRGDMFGC
>ID21978-NO
GGCVGESQQCADWSGPYCCKGYYCTCRYFPKCICVNDN
>ID21981-NO
GGDDQFDDYGHMRF
>ID21982-ABP_both
GGEAVQKLVREVKDG
>ID21985-NO
GGESSNEAKGMWFGPRL
>ID21987-NO
GGETGNDAKAMWFGPRL
>ID21989-NO
GGETSGEGKGMWFGPRL
>ID21990-NO
GGETSGETKGMWFGPRL
>ID21993-NO
GGFGQQSQFG
>ID21996-NO

GGGARKKAAKAARKKAAKAARKKAAKAARKKAAKA
>ID22000-NO
GGGGEGEQFDDYGHMRF
>ID22004-NO
GGGGGSGETSGMWFGPRL
>ID22011-NO
GGGGSSGLFAFPRV
>ID22012-NO
GGGGTSGLFAFPRV
>ID22021-NO
GGGRAFNHNANLFRFD
>ID22023-NO
GGGRAFYDE
>ID22025-NO
GGGRSFRLSSLGE
>ID22026-NO
GGGSSGETNGMWFGPRL
>ID22027-NO
GGGTRAGMKY
>ID22029-NO
GGGWG
>ID22032-NO
GGHDGDPVLTGTLFY
>ID22033-ABP_both
GGHGGHGGHGGHGGHGGH
>ID22034-ABP_both
GGHGLLKRIKTLL
>ID22037-ABP_both
GGHRAGLQFPVGRVHRLLRK
>ID22038-NO
GGHTRQ
>ID22045-NO
GGKMFMRF
>ID22047-NO
GGKNDNFIRF
>ID22049-NO
GGKPDLRPCYPPCHYIPRPKPR
>ID22050-NO
GGKQDNFIRF
>ID22052-NO
GGKSGSNFIRF
>ID22053-NO
GGKVCSAYF
>ID22054-NO
GGKYMRF
>ID22056-ABP_pos
GGLFYVKHKEQQRKRF
>ID22059-NO
GGLGRCIYNCMNSGGGLSFIQCKTMCY
>ID22062-ABP_both
GGLKKLGKKLEGAGKRVFNAAEKALP

>ID22067-ABP_both
GGLRKLGRKILRAWKKYGPIIVPIIRI
>ID22071-NO
GGNAKSYPQQIPYSFGL
>ID22072-NO
GGNDFMRF
>ID22077-NO
GGPQGPLRF
>ID22079-NO
GGPYAFGL
>ID22082-NO
GGQGTVLDKMYPRGNHWAVGHLM
>ID22085-ABP_both
GGRAAVLNALGKEEQIGRASNSGRKCARKKK
>ID22088-NO
GGRNFLRF
>ID22089-NO
GGRPTY
>ID22090-NO
GGRSNDNFIRF
>ID22091-NO
GGRSPSLRLRF
>ID22103-NO
GGSLDALRSGHQVPMLRA
>ID22104-NO
GGSLFRF
>ID22105-NO
GGSSGLIAFPRL
>ID22106-NO
GGSSGLISMPRV
>ID22107-NO
GGSSGLISVPRV
>ID22109-NO
GGSTSRSGCFGHKMDRIGTISGMGC
>ID22117-NO
GGVCPAILKKCRRDSDCPGACICRGNGYCGSGSD
>ID22118-NO
GGVCPKILAACRRDSDCPGACICRGNGYCGSGSD
>ID22123-NO
GGVIPN
>ID22125-NO
GGVKGEEKRVCPPDYVRCIRQDDPQCYSDNDCGDQEICCFWQCGFKCVLP
>ID22126-NO
GGVKRFKKFFRKLKKSV
>ID22127-NO
GGVWNLNVNPGTTGARVWARTK
>ID22130-NO
GGWSHWSPWSS
>ID22135-NO
GHACYRNCWREGNDEETCKERCG
>ID22137-NO

GHADNFMRF
>ID22141-NO
GHDFDQDDVNSSGEKDESLVRI
>ID22142-NO
GHDFEVFLRF
>ID22146-NO
GHGGASNYVRL
>ID22147-ABP_both
GHGHGHGHGH
>ID22148-NO
GHGKHKNK
>ID22149-NO
GHGQISHKRHKTDSFVGLM
>ID22155-NO
GHKIATFQER
>ID22156-NO
GHKIATFQQR
>ID22159-NO
GHLGRPYIGGGGGFNRGGGFHRGGGFHRGGGFHSGGGFHRGGGFHSGGSF
>ID22160-NO
GHLIPLRQPSH
>ID22164-NO
GHRATSDLASTGEESQD
>ID22165-ABP_both
GHRGALKDWVQAAGGAVAAFDFTTKG
>ID22166-NO
GHRNFLRF
>ID22178-NO
GICCGVSFCYPC
>ID22184-NO
GICKDLWCQ
>ID22193-NO
GIFAILKIATKLIGKTLAKAAGKAGTGLLACKAAKEC
>ID22195-NO
GIFDSIKEGFKNAAVTLLDKIKCKISACPPA
>ID22196-NO
GIFDSIKEGFKNAAVTLLNKIK
>ID22197-NO
GIFDSIKEGFKNAAVTLLNKIKCKISDCPPA
>ID22200-NO
GIFGKILGAGKEVLCGLSGLC
>ID22201-NO
GIFGKILGGGKKVLCGLSGM
>ID22211-NO
GIFKKFVKILYKVQKL
>ID22212-NO
GIFLDKLKNFGKDVAGILLKKASCALSGQC
>ID22213-NO
GIFLDTLKNMAGKMLESLKCKLFGCKP
>ID22214-NO
GIFLNALKNFAKTAGKGVLQSVLNTASCKLSKQC

>ID22215-NO
GIFLPGSVILRALSRQ
>ID22216-NO
GIFPIFAKLLGKVIKVASSLISKGRTK
>ID22219-ABP_neg
GIFSKLAGKKLKNLLISG
>ID22220-NO
GIFSKVLGAGKKVLCGVSGLC
>ID22221-NO
GIFSLFKTAAKFVGKNLLKEAGKAGLEHLACKVKNEC
>ID22223-NO
GIFSSRKCKTPSKTFKGICTRDSNCDTSCRYEGYPAGDCKGIRRRCMCSK
>ID22227-NO
GIFTLIKGAAKLIGKTVAKEAGKTGLELMACKITNQR
>ID22230-NO
GIGAKILGGVKTALKGALKELAFTYVN
>ID22231-ABP_neg
GIGAVLAVLTTGLPALISWIKRKRQQ
>ID22241-NO
GIGAVLTTGLPALISWIKRKRQQ
>ID22245-ABP_both
GIGAVVKVLTTGVPAVISWIKRKRQQ
>ID22250-ABP_both
GIGGALLNVGKVALKGLAKGLAEHFAN
>ID22251-ABP_both
GIGGALLSAGKAALKGLAKGLAEHFAN
>ID22255-NO
GIGGALLSAGKSALKGVAIGLAEHFAN
>ID22257-NO
GIGGALLSVGKSALKDLAKGLAEHF
>ID22260-NO
GIGGKILAGVKTALKGAAKELAATYLH
>ID22261-NO
GIGGKILFGLKTALKGAAKELAATYLH
>ID22262-NO
GIGGKILGGFKTALKGAAKELAATYLH
>ID22263-NO
GIGGKILGGLKTALKGAAKELAATYLH
>ID22265-NO
GIGGKILGGLKTALKGAAKELAFTYLH
>ID22266-NO
GIGGKILGGLKTALKGAAKELASTYQH
>ID22267-NO
GIGGKILGGLKTVLKDAAKELAATYLH
>ID22282-ABP_both
GIGKFIHSAKRFGRAWVGEIMNS
>ID22283-ABP_both
GIGKFIHSVKKWGKTFIGEIMNS
>ID22284-ABP_both
GIGKFLHAALLFAKAFVAEIMNS
>ID22301-ABP_both

GIGKFLKKAKKF
>ID22306-ABP_both
GIGRFLHSARRFGRAFVGEIMNS
>ID22307-ABP_both
GIGRIGKVHAANLIKIPKG
>ID22308-NO
GIGRKFLGGVKTTFRCGDKDFASKHLY
>ID22313-NO
GIGTKFLGGLKTAVKGALKELASTYVN
>ID22314-NO
GIGTKFLGGLKTAVKGALKELASTYVS
>ID22316-NO
GIGTKIIGGFKTAVKGALKELAFTYVN
>ID22317-NO
GIGTKIIGGLKTAVKGALKELAFTYVN
>ID22324-NO
GIGTRIIGGLKTAVKGALKELASTYVN
>ID22328-ABP_both
GIHDILKYGKRS
>ID22330-ABP_both
GIHHILKYGKPS
>ID22331-ABP_both
GIHKILKYGKPS
>ID22336-ABP_both
GIINTLQKYYARVRGGRYAVLSVLPKEEQIGKVSTRGRKKVRRKK
>ID22349-NO
GIISGLLGAGKKIVCGLSGLC
>ID22351-ABP_both
GIKCCFCCGCCNSGVCELCCRF
>ID22354-ABP_both
GIKDLLKGAAKALVKTVLK
>ID22355-NO
GIKDYLKKLLQKAINKIKSLRKKQDA
>ID22357-ABP_both
GIKHILFMAKTKLPRATCTAEIKENCDRKK
>ID22358-ABP_both
GIKIAKKAITIAKKIAKIYW
>ID22359-ABP_both
GIKKILKYGKKS
>ID22360-ABP_neg
GIKKWVKGVAKGVAKDLAKKIL
>ID22363-NO
GILDCFKNMALNAAKSAGVSVLNALSCKLSKTC
>ID22364-NO
GILDSFKDVAKGVATHLLNMAKCKMTGC
>ID22369-NO
GILDTIKEVGISAVQGAAQSFLSTLSCKIAKTC
>ID22370-ABP_both
GILDTLKQFAKGVGKDLVKGAAQG
>ID22371-ABP_both
GILDTLKQFAKGVGKDLVKGAAQGV

>ID22373-ABP_both
GILDTLKQFAKGVGKDLVKGAAQGVLS
>ID22374-ABP_both
GILDTLKQFAKGVGKDLVKGAAQGVLST
>ID22379-ABP_both
GILDTVKGVAKDVAAHLLNMVKCKITGC
>ID22380-NO
GILDVFKQLGKTAAKSVAQSLLNTASCKLAKTC
>ID22384-ABP_both
GILGKLWKGVKSIF
>ID22385-NO
GILKGILGMGKNLVCGLSGLC
>ID22386-NO
GILKKLFTKVF
>ID22387-ABP_both
GILKSLLKKLKKIIAK
>ID22388-NO
GILLDKLKNFAKGVAQSLLNKASCALSGQC
>ID22389-NO
GILLPALRKFCCDSNWCHISDCECCY
>ID22395-NO
GILRG
>ID22397-NO
GILSGLGRCGEENSMWI
>ID22401-ABP_both
GILSKLLKKLKKIIAK
>ID22404-ABP_neg
GILSSFKGVAKGVAKDLAGKLLETLK
>ID22406-ABP_both
GILSSLLKKLKKWIAK
>ID22407-ABP_both
GILSSLLKKWKKIIAK
>ID22408-ABP_both
GILSSLWKKLKKIIAK
>ID22409-ABP_both
GILSSLWKKLKKWIAK
>ID22410-ABP_both
GILSSLWKKPKKIIAK
>ID22411-ABP_both
GILSSWLKKLKKIIAK
>ID22414-ABP_both
GIMDTVKNAAKNLAGQLLDKLK
>ID22416-ABP_both
GIMISLMKKLAAHIAK
>ID22417-ABP_both
GIMSSLMKKLAAHIKK
>ID22420-ABP_both
GIMSSLMKKLAKIIAK
>ID22421-ABP_both
GIMSSLMKKLAKIIKK
>ID22423-ABP_both

GIMSSLMKKLKKHIAK
>ID22425-ABP_both
GIMSSLMKKLKKIIKK
>ID22426-NO
GINAEWPLRAGLK
>ID22486-NO
GIPGERGPVGPSG
>ID22495-NO
GIRGNCCMFHTCPIDYSRFNCP
>ID22499-ABP_both
GIRRILKYGKRS
>ID22500-ABP_both
GIRRILRYGRPS
>ID22504-ABP_both
GISLANKMSLAKWEK
>ID22506-NO
GISRVRICREKGGHCDADCHLEERHLGGCRAAYLTFCC
>ID22508-NO
GITSWLPF
>ID22509-NO
GIVDECCLRPCSVDVLLSYC
>ID22511-NO
GIVDQCCTGTCTRHQLQSYCN
>ID22513-NO
GIVDQCCTSICTLYQLENYCN
>ID22514-NO
GIVEECCHKPCTIFDLQNYCN
>ID22515-NO
GIVEECCKGVCSMYQLENYCN
>ID22516-NO
GIVEQCCAGVCSLYQLENYCN
>ID22521-NO
GIVEQCCHHPCNIFDLQNYCN
>ID22522-NO
GIVEQCCHKPCNIFDLQNYCN
>ID22524-NO
GIVEQCCHNTCSLVNLEGYCN
>ID22525-NO
GIVEQCCHNTCSLYQLENYCN
>ID22527-NO
GIVEQCCHSPCSLYDLENYCN
>ID22528-NO
GIVEQCCHSTCSLFQLENYCN
>ID22530-NO
GIVEQCCNSICSLYQLETYCN
>ID22537-NO
GIVEQCCTSICSLYQLENYCNFVNQHLCGSHLVEALYLVCGERGFFYTPK
>ID22543-NO
GIWCDPPCPKGETCRGGECSDEFNSDV
>ID22544-ABP_both
GIWDTIKSMGKVFAGAILQNL

>ID22549-ABP_both
GIWKTIKSMGKVFAGAIKQNL
>ID22550-ABP_both
GIWKTIKSMGKVFAGAILQNL
>ID22553-ABP_both
GIWSSLLKKLKKIIAK
>ID22554-ABP_both
GIWTCRKKRA
>ID22558-NO
GKAEF
>ID22562-ABP_both
GKAPKLKIFDTKNLK
>ID22564-NO
GKATSSISKCVFSFFKKC
>ID22565-NO
GKCEWQGGQLNCIAT
>ID22566-NO
GKCGDINAGCSSDCDCCGSSVTCDCAIRKKF
>ID22567-NO
GKCGDINAPCKAECDCCGYTTCDCYWGN
>ID22568-NO
GKCGEINGSCDECYGGSVTCDCY
>ID22569-NO
GKCVLVTL
>ID22573-ABP_both
GKEFKRIVGRIYRLCCR
>ID22574-ABP_both
GKEFKRIVKWPWWPWRR
>ID22576-ABP_both
GKEFKRIVWLSKTAKKL
>ID22578-NO
GKEIIVKAQR
>ID22579-ABP_both
GKFHQIKKMFLSVGVKVTSLKRIQFGDF
>ID22580-ABP_both
GKFLKKAKKFGK
>ID22581-ABP_both
GKFLKKAKKFGKAFVKI
>ID22603-NO
GKHRHERGHHRDRRER
>ID22605-ABP_neg
GKIALPIAKAALPVAAGLVG
>ID22606-ABP_both
GKIIKVVK
>ID22607-ABP_both
GKILKYLLYLLRKYANLIIR
>ID22608-NO
GKINLKALAALAKKIL
>ID22613-NO
GKKIATYQER
>ID22615-NO

GKKKKRKREKL
>ID22616-NO
GKKKRKLSNRESAKRSR
>ID22617-ABP_both
GKKLAKTIAKEVAKQGAKFA
>ID22627-NO
GKKTNLFSALIKKKKTA
>ID22628-NO
GKKVLQ
>ID22629-NO
GKKYRRFRWKFRGRFWFWG
>ID22631-ABP_both
GKKYRRFWKFKGKWFWFWG
>ID22633-ABP_both
GKLFKKILKIL
>ID22642-ABP_both
GKLWGLKGKLWGLKGKLWGLK
>ID22643-ABP_both
GKLWLKG
>ID22644-ABP_both
GKLWLKGGKLWLKG
>ID22645-ABP_both
GKLWLKGGKLWLKGGKLWLKG
>ID22648-NO
GKMVKVVSWY
>ID22651-NO
GKNPTLQCMGNRGFCRPSCKKGEQAYFYCRTYQICCLQSHVRISLTGVED
>ID22655-NO
GKPASNLVECVFSLFKKCN
>ID22656-NO
GKPCHEEGQLCDPFLQNCCLGWNCVFVCI
>ID22658-ABP_both
GKPIGNAILGLRPKISRWL
>ID22661-ABP_neg
GKPKPYSPRPTSHPRPIRV
>ID22662-ABP_both
GKPRGNARLGLRPKRSRWL
>ID22663-NO
GKPRHYPYSPRPTSHPRPIR
>ID22665-ABP_neg
GKPRPYPPRPPPHPRPIRV
>ID22666-ABP_neg
GKPRPYSPKPTSHPRPIRV
>ID22690-NO
GKPYAFGL
>ID22691-ABP_both
GKPYGNAYLGLRPKYSRWL
>ID22693-NO
GKRARNTEAARRSRARKL
>ID22700-NO
GKRSQEEPESYEWGTVQIYDKRR

>ID22701-NO
GKRVAKRKLIEQNRERRR
>ID22703-NO
GKSDNFIRF
>ID22704-ABP_both
GKSDVRRWRSRY
>ID22705-ABP_both
GKSNFLKSYVSKHPK
>ID22706-NO
GKTDNFIRF
>ID22710-ABP_both
GKVKIGINGFGRIGRLVA
>ID22718-ABP_both
GKWMHLLKHILK
>ID22719-ABP_both
GKWMSALKHILK
>ID22720-ABP_both
GKWMSKLKHILK
>ID22721-ABP_both
GKWMSLLKQILK
>ID22722-ABP_both
GKWMSWLKHILK
>ID22724-ABP_both
GKWQSLLKHILK
>ID22726-ABP_both
GKWWSLLKHILK
>ID22736-ABP_both
GKYGFYTHVFRLKKWMQKVIDRFGG
>ID22741-NO
GKYTWYGYSLRANWMR
>ID22746-ABP_both
GLAKGIKEAAKALDKAL
>ID22748-ABP_both
GLARALTRLLRQLTRQLTRA
>ID22749-ABP_both
GLARGLREASKALDR
>ID22750-NO
GLAVCGETCVGGTCNTPGCTCSWPVCTRN
>ID22751-NO
GLCAVNEFVALAAIPGGAATFAVCQMPNLDEIVSNAAYV
>ID22752-NO
GLCCPMRWSSSEG
>ID22755-NO
GLDAYSFTGAL
>ID22761-NO
GLDIQK
>ID22762-NO
GLDLGLGRGFSGSQAAKHLMGLAAANFAGGP
>ID22763-NO
GLDLGLNRGFSGSQAAKHLMGLAAANYAGGP
>ID22766-NO

GLDRYGFVGGL
>ID22767-NO
GLDSLSGASFGIE
>ID22771-NO
GLEEELETVELVEELQQA
>ID22776-NO
GLFAVIHHVASVIGGL
>ID22777-NO
GLFAVIHKVASVIGGL
>ID22780-ABP_both
GLFAVIKKVASVI
>ID22781-ABP_both
GLFAVIKKVASVIG
>ID22793-ABP_both
GLFDIVKKVVGAL
>ID22798-ABP_both
GLFDKWAWWRWRR
>ID22800-ABP_both
GLFDVIK
>ID22801-ABP_both
GLFDVIKASVIGGL
>ID22802-ABP_both
GLFDVIKKLLKKIGGL
>ID22803-ABP_both
GLFDVIKKLLKKIKGL
>ID22804-ABP_both
GLFDVIKKV
>ID22807-ABP_both
GLFDVIKKVASKIKGL
>ID22808-ABP_both
GLFDVIKKVASV
>ID22813-ABP_both
GLFDVVIGGL
>ID22814-NO
GLFEALLELLESLWELLLEA
>ID22820-NO
GLFGLAKGSVAKPHVVPVISQLVG
>ID22826-ABP_both
GLFKKLRRKIKKGFKKIFKRL
>ID22827-ABP_both
GLFKKLRRKIKKGFKKIFKRLPPIGVGVSIPLAGKR
>ID22830-NO
GLFKTLIKGAGKMLGHVAKEFLGSQGEPES
>ID22834-ABP_both
GLFLDTLKKFAKAGMEAVTNPK
>ID22855-NO
GLFSFLPKVIGVIGPLIHPPS
>ID22856-NO
GLFSIFKTAAKFVGKNLLKQAGKAGLEHLACKAKNEC
>ID22858-NO
GLFSILKIGAEVIGKNLLKQAGKAGMEYAACKAANQC

>ID22859-NO
GLFSILKIGAKVIGKNLLKQAGKAGMEYAACKATNQC
>ID22862-NO
GLFSKFAGEGIKNLLFKGVQAHRQGSWHGCDQNWDRRCRL
>ID22878-NO
GLFSMFAGKGIKNFLIEGVKHIGKEVGMDVIRSGIDVAGCKIKGEC
>ID22879-NO
GLFSTLFHADCCENPACRHTQGC
>ID22883-NO
GLFTFIKCAYKLRAPAVAC
>ID22884-NO
GLFTIKCAYKLIAPTVACN
>ID22892-NO
GLFTLIKGAAKLIGKTVAKEAGRLGLNLWLVKLPTNVKT
>ID22897-NO
GLGDKFGESIVNANTVLDDLNSRMPQSRHDIQQL
>ID22899-ABP_both
GLGKDLAKLGVDLVACKISKQC
>ID22900-NO
GLGKGCFGLKLDRIGSMSGLGC
>ID22904-NO
GLGNNAFLGVR
>ID22905-NO
GLGNNAFVGVR
>ID22908-NO
GLGPRPLRF
>ID22909-NO
GLGRVIGRLIKQIIWRR
>ID22910-NO
GLGSLLKKAGKKLKQPKSKRKV
>ID22914-NO
GLGSVLGKILKMGANLLGGAPKQ
>ID22916-NO
GLHMLRL
>ID22921-NO
GLIDVRCYDSSQCE
>ID22925-NO
GLIKPGTLWCGMGNNAETYDQLGPFADVDSCK
>ID22929-NO
GLIRPSKRCIGGGDPCEFHRPYTCCSGYCIVFVCA
>ID22931-NO
GLISGILGAGKKVLCGLSGLC
>ID22933-ABP_both
GLISNLGI
>ID22935-ABP_both
GLKALKKVFKGIHKAIKLINKHVK
>ID22936-ABP_both
GLKALKKVFKGIHKAIKLINKHVQ
>ID22938-ABP_both
GLKALKKVFQGIHEAIKLINNHVQ
>ID22939-ABP_both

GLKALKKVFQGIHKAIKLINNHVQ
>ID22941-ABP_both
GLKEVLHSTKKFAKGFITGLTGQ
>ID22942-ABP_both
GLKKDALQSIVKKAQLAAMG
>ID22944-NO
GLKKKFKELAGNATKKS
>ID22945-NO
GLKVCGRYPGICDGIR
>ID22947-NO
GLLAFPRV
>ID22948-ABP_both
GLLARIKTLL
>ID22950-NO
GLLDILKKVGKVA
>ID22956-NO
GLLDTFKNLALNAAKSAGVGTEFIIL
>ID22959-NO
GLLDTLGGILGL
>ID22965-NO
GLLDTVKNLATNLAGQLLDRLKCKVTGC
>ID22966-NO
GLLDVLKNVAIGVAKGAGVGVLNALSCKLDKSC
>ID22967-NO
GLLEALAELLEGRKKRRQRRRPPQ
>ID22970-ABP_both
GLLFKELQKLIRYQIFIGK
>ID22975-NO
GLLGLLGSVVSHVLPAITQH
>ID22979-ABP_both
GLLKAIKTLL
>ID22980-ABP_both
GLLKFIKTLL
>ID22981-ABP_both
GLLKFIKWLL
>ID22982-NO
GLLKGILGAGKHIVCGFSGLC
>ID22983-ABP_both
GLLKIIKTLL
>ID22987-ABP_both
GLLKKIKKLL
>ID22988-ABP_both
GLLKKIKTLL
>ID22990-ABP_both
GLLKKWLKKWKEFKRIVGY
>ID22991-ABP_both
GLLKLIKTLL
>ID22992-ABP_both
GLLKNIKTLL
>ID22993-ABP_both
GLLKR

>ID22994-ABP_both
GLLKRAKTLL
>ID22996-ABP_both
GLLKRI
>ID22997-ABP_both
GLLKRIATLL
>ID22998-ABP_both
GLLKRIK
>ID22999-ABP_both
GLLKRIKALL
>ID23000-ABP_both
GLLKRIKFLL
>ID23001-ABP_both
GLLKRIKILL
>ID23003-ABP_both
GLLKRIKKLLKKIKKLL
>ID23005-ABP_both
GLLKRIKNLL
>ID23006-ABP_neg
GLLKRIKSLL
>ID23007-ABP_both
GLLKRIKT
>ID23008-ABP_both
GLLKRIKTAL
>ID23012-ABP_both
GLLKRIKVLL
>ID23013-ABP_both
GLLKRIKWLL
>ID23021-ABP_both
GLLKSLCKKFVKGHLGELIEELTTSDD
>ID23023-ABP_both
GLLKVIKTLL
>ID23024-ABP_both
GLLKVIKVLL
>ID23025-ABP_both
GLLKWIKKLL
>ID23026-ABP_both
GLLKWIKTLL
>ID23027-NO
GLLLVVP
>ID23028-NO
GLLMASSVRGRT
>ID23034-NO
GLLRGILGAGKHIVCGLSGLC
>ID23035-NO
GLLRGVLGVGKKIVCGLSGLC
>ID23040-ABP_both
GLLRKWGKKWKEFLRRVWK
>ID23041-NO
GLLRPPRCGEAYSMWT
>ID23084-NO

GLLSGKSVKGSI
>ID23087-NO
GLLSGTSGKKGSI
>ID23088-NO
GLLSGVKGRGKKIVCGLSDKC
>ID23105-ABP_neg
GLLSRIKTLL
>ID23107-ABP_both
GLLSSLGLNL
>ID23108-NO
GLLSTFKQVGISALQGAAQGLLNTLSCKIAKTC
>ID23109-NO
GLLSVLGSVA
>ID23110-NO
GLLSVLGSVAKH
>ID23115-NO
GLLSVLGSVAKHVLPH
>ID23119-NO
GLLTGVKGRGKKIVCGLSGLC
>ID23120-ABP_neg
GLLTRIKTLL
>ID23123-ABP_both
GLLWKWGWKWKEFLRIVGY
>ID23127-ABP_neg
GLMKRIKTML
>ID23128-NO
GLMNTLKGAATNAAVTLLDKIQCKLTGTC
>ID23130-NO
GLMSILGKVAGNVLGGLFKPKENVQKM
>ID23131-ABP_both
GLMSLVKDIAKLAAKQGAKQ
>ID23132-NO
GLMSSIGKALGGLIVNVLKPKTPAS
>ID23133-ABP_both
GLNAFKKVFQGIHEAIKLINNHVQ
>ID23135-ABP_both
GLNALKKFFQGIHEAIKLINNHVQ
>ID23136-ABP_neg
GLNALKKVAQGIHEAIKLINNHVQ
>ID23137-ABP_both
GLNALKKVFKGIHEAIKLINNHVQ
>ID23144-ABP_both
GLNALKKVFQGIHKAIKKINNHVQ
>ID23145-ABP_both
GLNALKKVFQGIHKAIKLINNHVQ
>ID23146-ABP_both
GLNALKKVFQKIHEAIKKINNHVQ
>ID23151-ABP_both
GLNALKKVSQGIHEAIKLINNHVQ
>ID23155-NO
GLNNKCAYFRGQCRRKCPQRDIFFGFCRNHDQCCLSSLHTRH

>ID23156-NO
GLNRPSKRCLAGSAPCEFHKRSTCCSGHCIIWWCA
>ID23160-ABP_both
GLPALISWIKRKR
>ID23161-ABP_both
GLPALISWIKRKRGG
>ID23162-ABP_both
GLPALISWIKRKRL
>ID23166-ABP_both
GLPGPLGPAGPK
>ID23167-NO
GLPGSRGERGLPG
>ID23251-NO
GLPVCGETCVGGTCNTPGCTCSWDKCTRN
>ID23255-NO
GLPVCGETCVGGTCNTPGCTCSWPVCTAN
>ID23278-ABP_both
GLQFPVGRVHRLLRK
>ID23279-NO
GLQMLRL
>ID23282-ABP_both
GLRILLLKV
>ID23286-ABP_both
GLRKRKRKFRNKKKEKLKKI
>ID23288-ABP_neg
GLRKRLRKFRNKDKEKLKKIGQKIQGLLPKLAPRTDY
>ID23289-ABP_neg
GLRKRLRKFRNKFKEKLKKIGQKIQGLLPKLAPRTDY
>ID23290-ABP_neg
GLRKRLRKFRNKHKEKLKKIGQKIQGLLPKLAPRTDY
>ID23291-ABP_neg
GLRKRLRKFRNKIKECLKKIGQKIQGLLPKLAPRTDY
>ID23292-ABP_neg
GLRKRLRKFRNKIKEDLKKIGQKIQGLLPKLAPRTDY
>ID23293-ABP_neg
GLRKRLRKFRNKIKEKDKKIGQKIQGLLPKLAPRTDY
>ID23294-ABP_neg
GLRKRLRKFRNKIKEKKKKIGQKIQGLLPKLAPRTDY
>ID23295-ABP_neg
GLRKRLRKFRNKIKEKLKKEGQKIQGLLPKLAPRTDY
>ID23307-ABP_both
GLRKRLRKPRNKPKEKLKKI
>ID23310-NO
GLRQKVQSLV
>ID23313-NO
GLRSKIKEAAKTAGKMALGFVNDMAGEQ
>ID23314-NO
GLSAASPPLAETGAPRRF
>ID23315-NO
GLSDGEWQ
>ID23316-NO

GLSMLRL
>ID23317-NO
GLSRNYLRF
>ID23319-NO
GLSRPSKRCIAGGQPCEFHRGYMCCSEHCIIFVCA
>ID23320-NO
GLSRPSKRCIGGGDPCEFHRGYTCCSEHCIIWVCA
>ID23321-NO
GLSRSCFGVKLDRIGSMSGLGC
>ID23322-ABP_both
GLTFLKKILNFAKKIYTAIW
>ID23324-NO
GLTFLSPADMQKIAERQSQNKLRHGNMN
>ID23326-NO
GLTRPSKRCLAGSAPCEFHRGYTCCSGHCLIWVCA
>ID23334-ABP_both
GLVPNLLNNLGL
>ID23337-ABP_both
GLVSGLLNSVTGLLGNLAGGGL
>ID23338-ABP_both
GLVSNLGI
>ID23339-ABP_both
GLVTGLLNTAGGLLGDLFGSLSG
>ID23341-NO
GLWDSVKEGFKNAAVTLLNKIKCKISACPPA
>ID23345-NO
GLWDTIKQAGKNIFLNVLDKIKCKVAGGCKP
>ID23348-NO
GLWESIKNLGKKFALNIMEKLKCKFGGGCLP
>ID23349-ABP_both
GLWKGKLGLWKGKLGLWKGKL
>ID23350-NO
GLWKTLKDSAKSAVTNVAVTMLDKLRCKLTGGC
>ID23352-ABP_neg
GLWNTKLEAGLLFAMGKLDLKRCLKAGGC
>ID23353-NO
GLWQGP
>ID23354-ABP_both
GLWQIFSSKEEGKDNSQQKSKGDQAKEL
>ID23355-NO
GLWRALWRALRSLWKLKRKV
>ID23356-NO
GLWRALWRALWRSLWKKKRKV
>ID23357-NO
GLWRALWRALWRSLWKLKRKV
>ID23361-NO
GLWRALWRGLRSLWKLKRKV
>ID23362-NO
GLWRALWRLLRSLWRLLWKA
>ID23367-NO
GLWSKIKDAGKAVLKAAGKAALGAVTDAV

>ID23369-NO
GLWSKIKETGKEAAKAAGKAALDKIAEAV
>ID23370-ABP_both
GLWSKIKEVGKEAAKAAAKAAGK
>ID23374-ABP_both
GLWSTIKQKGKE
>ID23377-NO
GLWWRLWWRLRSWFRLWFRA
>ID23382-NO
GLYCCQPKPNGQMMCNRWCEINSRCCGRR
>ID23386-ABP_both
GMAKLLAKVLPHVVKLIK
>ID23387-ABP_neg
GMASKAGAIAGKIAKVALKALGGC
>ID23389-ABP_neg
GMASKAGAIAGKIAKVAWKALGGA
>ID23392-NO
GMASKAGQVLGKLAKVAIGAA
>ID23393-ABP_both
GMASKAGSVAGKIAKFALGAL
>ID23396-ABP_both
GMASKLAKVLPHVVKLIK
>ID23399-NO
GMASTAGSVLGKLAKTAIGIL
>ID23402-NO
GMATKAGTIVGKIAKVALNAL
>ID23404-NO
GMDDFAFSPGL
>ID23405-NO
GMDHFAFTGGL
>ID23406-NO
GMDRFSFSGHL
>ID23407-NO
GMDSFTFAPGL
>ID23409-NO
GMDSLAFSGGL
>ID23412-NO
GMKDYFKKLLQKVIKKIKSFRKKQD
>ID23413-NO
GMKEKCVTMGGYCRKQCRVQDALSGYCRNENPCCV
>ID23417-ABP_neg
GMLKRIKTLL
>ID23419-NO
GMLKWKNDFFQEF
>ID23422-NO
GMMGPSII
>ID23425-NO
GMMYRS
>ID23426-NO
GMPGVLRF
>ID23430-NO

GMRLTYNRPCIYATKKTKEM
>ID23434-NO
GMSAFSF
>ID23436-NO
GMWDECCDDPPCRQNNMEHCPAS
>ID23437-NO
GMWGDCDDWLAACTTPSQCCTEVCDGFCRLWE
>ID23438-NO
GMWGECKDGLTTCLAPSECCSEDCEGSCTMW
>ID23439-NO
GMWGKCKDGLTTCLAPSECCSGNCEQNCKMW
>ID23440-NO
GMWGTVFKGIKTVAKHLLPHVFSSQQS
>ID23441-ABP_both
GMWPKILGHLIR
>ID23443-ABP_both
GMWSKIKETAMAAAKEAAKAAGKTISDMIKQ
>ID23444-ABP_both
GMWSKIKNAGKAAAKAAAKAAGKAALDAVSEAI
>ID23445-ABP_both
GMWSKILGHL
>ID23447-ABP_both
GMWSKILGPLIR
>ID23448-ABP_both
GMWSKILPHLIR
>ID23449-ABP_both
GMWSKIPGHLIR
>ID23451-NO
GMYGGW
>ID23452-NO
GMYGGY
>ID23454-NO
GNCLELGEYCDGSKDDCQCCRDNAYCGCDIF
>ID23455-ABP_both
GNDYLHKLTQRKSVK
>ID23457-NO
GNFFRF
>ID23458-ABP_both
GNFLRNGGSYYIVPAFRG
>ID23461-NO
GNGRAHA
>ID23463-NO
GNIIPDRPMHPT
>ID23464-NO
GNLHSLF
>ID23467-NO
GNNFLRF
>ID23470-ABP_both
GNNRPVYIAQPRPPHPAL
>ID23471-ABP_both
GNNRPVYIAQPRPPHPRL

>ID23472-ABP_both
GNNRPVYIPKPRPPHPRI
>ID23475-ABP_neg
GNNRPVYIPQPKPPHPRL
>ID23476-ABP_neg
GNNRPVYIPQPRPHPRL
>ID23477-NO
GNNRPVYIPQPRPPHP
>ID23479-ABP_neg
GNNRPVYIPQPRPPHPL
>ID23481-ABP_both
GNNRPVYIPQPRPPHPRPIRV
>ID23502-NO
GNSFLRF
>ID23504-NO
GNSYRSFFNPNEYTVVE
>ID23506-NO
GNTKKAVPGFYGTR
>ID23508-NO
GNWCCSARVCC
>ID23509-NO
GNWNKFQGSW
>ID23510-NO
GNWNN
>ID23514-NO
GNYYGYCRVHLNTAY
>ID23516-NO
GPAESSGESAHPLE
>ID23520-NO
GPCASDHNC
>ID23521-NO
GPCFSGC
>ID23522-NO
GPCSR
>ID23524-NO
GPDSAFLRL
>ID23525-NO
GPDSSFLRL
>ID23526-NO
GPDSTFLRL
>ID23532-NO
GPESAFLRL
>ID23533-NO
GPETAFFRL
>ID23534-NO
GPETAFLRL
>ID23544-NO
GPFHFYQFLFPPV
>ID23545-NO
GPFLRF
>ID23546-NO

GPFPIIV
>ID23547-NO
GPFPILV
>ID23561-ABP_both
GPHSANHVRGLARVGNANPGLYLAKYAFIPVILAAKPLSPTPTKT
>ID23562-ABP_both
GPHSCNHQRGLCRVGNCNPGYYLAKYCFSPVILCCKPLSPTPTKT
>ID23563-NO
GPIGPP
>ID23564-NO
GPIGSVGAP
>ID23565-NO
GPIQISYNYNYGPCGRYCGILGVSPGDNLDCGNQR
>ID23566-ABP_both
GPIRRPEPRPRPE
>ID23567-ABP_both
GPIRRPKPRPRPE
>ID23570-NO
GPLGLLGFLGPLGLS
>ID23572-NO
GPLPLR
>ID23573-NO
GPLQLSWNYNYGAAGK
>ID23575-NO
GPLTILHINDVHAAFEQFNT
>ID23585-NO
GPPDPNKFIGLM
>ID23586-NO
GPPGAP
>ID23588-NO
GPPGTDGAP
>ID23590-NO
GPPPGLW
>ID23591-NO
GPPRFI
>ID23596-NO
GPPYDFGM
>ID23597-NO
GPPYQPLVPR
>ID23598-NO
GPPYSFGL
>ID23601-NO
GPRNFLRF
>ID23602-NO
GPRPPGFSPFRGKFHSQS
>ID23603-ABP_both
GPRSANHKRGLARVGNANPGKYLAKYSFRPVILSSKPLSPTPTKT
>ID23605-NO
GPRTYSFGL
>ID23607-NO
GPSAFLRL

>ID23609-NO
GPSATTGVWFGPRL
>ID23614-NO
GPSGFLGMR
>ID23615-NO
GPSGFYGVR
>ID23617-NO
GPSGNLHIRPAS
>ID23618-NO
GPSMGFHGMR
>ID23619-NO
GPSMR
>ID23621-NO
GPSQDFMRF
>ID23622-NO
GPSRVGG
>ID23624-NO
GPSSGFFGMR
>ID23626-NO
GPSSSAFFGMR
>ID23629-NO
GPTGRISMMGNRVQNIDPTHRINDRDYMGWMDF
>ID23630-NO
GPTSECFWKYCV
>ID23631-NO
GPTVGLFAFPRV
>ID23633-NO
GPVGPA
>ID23634-NO
GPVQPTYPGDDAPVEDLVNDLQQYLNVVTRHRY
>ID23635-NO
GPVRGPF
>ID23636-NO
GPVRGPFP
>ID23637-NO
GPVRGPFPII
>ID23638-NO
GPVRGPFPIIV
>ID23639-ABP_both
GPVSAVLTELRCTCLRVTLRVNPKTIGKLQVFPAGPQCSKVEVVASLKNG
>ID23640-NO
GPWAPCSASCGGGSQSRS
>ID23641-NO
GPWEDCSVSCGGGEQLRSR
>ID23643-NO
GPWERCTAQCGGGIQARRR
>ID23644-NO
GPWGDCSRTCGGGVQFSSR
>ID23645-NO
GPWGPCSGSCGPGRRLRRR
>ID23650-NO

GPYSFGL
>ID23653-NO
GQAKGRVYWRCYFNAVTCF
>ID23654-NO
GQAPRFF
>ID23655-NO
GQDFMRF
>ID23657-NO
GQERNFLRF
>ID23660-NO
GQMRQPI
>ID23662-NO
GQPGYGNAFL
>ID23667-NO
GQSSQRVFWRCYFNAVSCF
>ID23668-NO
GQTFVRF
>ID23680-NO
GRADNFLRL
>ID23681-NO
GRAENFLRL
>ID23683-ABP_both
GRAKRFRKKAKKLFKKLSPVIPLLHL
>ID23689-NO
GRCCEGPNGCSSRWCKDHARCC
>ID23704-NO
GRCCHPACGNNTSC
>ID23705-NO
GRCCHPACGPNYSC
>ID23706-NO
GRCCHPACGQNFSC
>ID23713-NO
GRCDHPACGNNTSK
>ID23714-NO
GRCDHPACGQNTSK
>ID23716-NO
GRCEHPACGQNTSK
>ID23717-NO
GRCLLMQCR
>ID23718-NO
GRCRDDFRC
>ID23719-NO
GRCRDDFRCWCTKRC
>ID23721-NO
GRCRGFRRRCFCTTHC
>ID23722-NO
GRCVDGGCT
>ID23724-NO
GRDAYIADSENCTYT
>ID23725-ABP_pos
GRDPYKLRPV

>ID23727-NO
GRDYRTCLTIVQKLKKMVDKPTQRSVSNAATRVCRTGRSRWRDVCRNFMR
>ID23730-NO
GRDYSFGL
>ID23735-ABP_both
GRFKRFRKKLKRLWHKVGPFVGPILHY
>ID23745-ABP_both
GRFRRLRKKTRK
>ID23746-ABP_both
GRFRRLRKKTRKRLKKIGKVLKAI
>ID23747-ABP_both
GRFRRLRKKTRKRLKKIGKVLKKI
>ID23750-NO
GRGASSNYVRL
>ID23751-NO
GRGDSPK
>ID23753-NO
GRGGPSNYVRL
>ID23758-NO
GRGRCCHPACGPNYSC
>ID23763-NO
GRKGKHKRKKLP
>ID23764-NO
GRKKRRQARAPPQC
>ID23765-NO
GRKKRRQPPQC
>ID23767-NO
GRKKRRQRPPQC
>ID23768-NO
GRKKRRQRRPPQC
>ID23769-ABP_both
GRKKRRQRRRGALWKSLLKNVGKA
>ID23774-NO
GRKKRRQRRRPPQTYADFIASGRTGRRNAI
>ID23778-NO
GRKLKKKKNEKEDKRPRT
>ID23779-ABP_both
GRKLWRKWLKRWLP
>ID23796-NO
GRLVLEITADEVKALGEALANAKI
>ID23801-NO
GRNDLNFIRY
>ID23803-NO
GRNFLRF
>ID23806-ABP_both
GRPMGHQAIETGLNIFRGLFKGKKKNKKTK
>ID23807-NO
GRPNFLRF
>ID23810-ABP_both
GRPNPVNNKPTSHPRPIRV
>ID23813-ABP_neg

GRPNPVPNPRPPHPRL
>ID23816-ABP_both
GRPRPFPGRPKPIFRPR
>ID23817-NO
GRPSNNFVRF
>ID23818-NO
GRQGCYEHLWRLIAWCAIFL
>ID23821-NO
GRQLRIAGKRLRGRSK
>ID23822-NO
GRQLRIAGRRLRGRSR
>ID23823-NO
GRQLRIAGRRLRRRSR
>ID23825-NO
GRQLRRAGRRLRRRSR
>ID23826-NO
GRQPYSFGL
>ID23829-NO
GRRERNKMAAAKCRNRRR
>ID23830-ABP_both
GRRGPRRANQNGTRRRRRRT
>ID23831-NO
GRRHHCRSKAKRSRHH
>ID23832-NO
GRRIAGPYIALE
>ID23833-NO
GRRINRLILPRN
>ID23836-NO
GRRND
>ID23839-NO
GRRPMKLNKTP
>ID23840-ABP_both
GRRPRPRPRP
>ID23841-ABP_both
GRRPRPRPRPFFF
>ID23842-ABP_both
GRRPRPRPRPFFFF
>ID23843-ABP_both
GRRPRPRPRPFFFFF
>ID23844-ABP_both
GRRPRPRPRPWWW
>ID23845-ABP_both
GRRPRPRPRPWWWW
>ID23848-NO
GRRRRATAKYRTAH
>ID23850-NO
GRRRRRERNK
>ID23851-NO
GRRRRRRRRRPPQ
>ID23852-ABP_neg
GRRRRS

>ID23853-ABP_both
GRRRRSVQW
>ID23856-NO
GRRTRSRRLRRS
>ID23860-ABP_both
GRRYKKFKWRFRGRFWFWG
>ID23861-ABP_both
GRRYKKFRWKFKGRWFWFG
>ID23866-NO
GRSQMQI
>ID23872-NO
GRVMP
>ID23875-NO
GRWYKWA
>ID23881-NO
GSAESPAALGEASAAHPLE
>ID23888-NO
GSASGLISMPRV
>ID23889-NO
GSASHHRRQLGLQGPQHFIADLSKKERPRMEEEEEAYGWMDF
>ID23891-NO
GSCVPVDQPCSLNTQPCCDDATCTQERNENGHTVYYCRA
>ID23894-NO
GSDIDDPRFFSGAF
>ID23896-NO
GSFAFLV
>ID23899-NO
GSFTPRI
>ID23901-NO
GSGEDLSYGDAYEVDEDDHPLFVPRL
>ID23903-NO
GSGFSSWG
>ID23905-ABP_both
GSGILILIKRK
>ID23906-NO
GSGKKGGKKHCQKY
>ID23908-NO
GSGNLMRF
>ID23910-NO
GSGQYAYGLGKAGQYSFGL
>ID23912-ABP_neg
GSGSRQSPSYGR
>ID23913-NO
GSGVSNGGTEMIQLSHIRERQRYWAQDNLRRRFLEK
>ID23918-ABP_both
GSHLVEKLYLVKERK
>ID23943-ABP_neg
GSKKPVPIIYCQRRTGKCQRM
>ID23945-NO
GSLACQNIVVCVKKQCNALC
>ID23946-ABP_both

GSLHGFMYKYLKNMVLNLF
>ID23947-ABP_both
GSLHGFMYKYLKNMVLTLF
>ID23948-ABP_both
GSLHGFMYKYLKTLVLRLY
>ID23951-NO
GSLSTFFRLFNRSFTQALGK
>ID23952-NO
GSLTGLISMPRT
>ID23953-NO
GSNDFMRF
>ID23958-NO
GSPGFSHSFV
>ID23960-NO
GSPHFI
>ID23961-NO
GSPQCPGGFNCPRCDCGAGY
>ID23962-NO
GSPRFF
>ID23965-NO
GSPWGLQHHPPRT
>ID23966-NO
GSPYFV
>ID23970-NO
GSRHPSLIIPRQ
>ID23975-NO
GSSDTDLDLQGHVDLGLDDLDKLRLIFPPGLIEEA
>ID23976-NO
GSSFLSPEHQKVQQRKESKKPAAKLKPR
>ID23979-NO
GSSFLSPSQKPQVRQGKGKPPRVG
>ID23980-NO
GSSFLSPSQRPQGKDKKPPRV
>ID23981-NO
GSSGGLITFGRT
>ID23983-NO
GSSGIIPFPRV
>ID23986-NO
GSSGLIPFGRT
>ID23987-NO
GSSGLIPFPRV
>ID23989-NO
GSSGLIPMGRV
>ID23990-NO
GSSGLIPMPRV
>ID23992-NO
GSSGLISFPRT
>ID23993-NO
GSSGLISMPRV
>ID23996-NO
GSSGMIPFPRV

>ID23997-NO
GSSGMISFPRT
>ID24000-NO
GSSTGRYAVLPNRPVIDPTHRINDRDYMGWMDF
>ID24004-NO
GSTGLIPFGRT
>ID24010-NO
GSVAKHLLPHVAPIIAEKL
>ID24011-NO
GSVAVFPAENGVQNTESTQE
>ID24012-NO
GSVCCKVDTSCCSN
>ID24016-NO
GSVLPVL
>ID24018-NO
GSVSHRR
>ID24019-ABP_both
GSWIKRLNSWLRK
>ID24029-NO
GSWPAARGVQ
>ID24037-NO
GSYRMMRL
>ID24042-NO
GTCNTPGCTCSWPVCTRNGLPVCGETCVG
>ID24048-NO
GTEKC
>ID24059-NO
GTKMIFVGIKKKEERADLIAYLKKA
>ID24062-NO
GTLLRF
>ID24064-NO
GTNGKPPDPKKESQDYLGWMDF
>ID24066-NO
GTPGPQGIAGQRGVV
>ID24071-NO
GTRCARCARTCRTACAT
>ID24074-NO
GTRTMERSVRTSSQYASGGPMPN
>ID24075-NO
GTSGLISFPRT
>ID24079-NO
GTTCKCGSTLGIYWFAVTSCPPGRGYTTHCGYF
>ID24080-NO
GTTCYCGKTIGIYWFGTKTCPSNRGYTGSCGYFLGICCYPVD
>ID24082-ABP_neg
GTVRRFPWWWPFLRR
>ID24098-NO
GTYCIELGERCPNPREGDWCCHKCVPEGKRFYCRDQ
>ID24099-NO
GTYLYPFSYYRLWRYFTRFLHKQPYYYVHI
>ID24101-ABP_both

GVADILKGAAKDIAGHLASKVMNKL
>ID24102-ABP_both
GVARIRDLLIIESRI
>ID24114-NO
GVDFGLGRGYSGSQAAKHLMGLAAANYAIGP
>ID24115-NO
GVDITVIRPNH
>ID24116-NO
GVDSSFLRL
>ID24117-NO
GVDSSFVRL
>ID24118-NO
GVEINVKCSGSPQCLKPCKDAGMRFGKCMNRKCHCTPK
>ID24120-NO
GVFDECCRKSCSISELQTYCG
>ID24121-ABP_both
GVFDIIKDAGKQLVAHAMGKIAEKV
>ID24122-ABP_both
GVFDIIKDAGKQLVAHATGKIAEKV
>ID24124-NO
GVFLDTLKGLAGKMLESLKCKIAGCKP
>ID24126-ABP_both
GVFRRLRKVTRKVLKKIGKVLKWI
>ID24127-ABP_both
GVFRVLRKVTRVVLKVIGKVLKWI
>ID24131-NO
GVGAGY
>ID24135-NO
GVHGV
>ID24137-NO
GVIELTKMIVQEMGKNALTSYSL
>ID24138-NO
GVIINVKCKISRQCLEPCKKAGMRFGKCMNGKCHCTPK
>ID24157-NO
GVIPKKIWETVCPTVEPWAKKCSGDIATYIKRECGKL
>ID24158-NO
GVKDYFKKLLQKVINKIKSFRKKQEA
>ID24161-NO
GVLGTVKDLLIGAGKSAAQSVLKILSCKLSNDC
>ID24162-NO
GVLGTVKDLLIGAGKSAAQSVLKTLSCKLFNDC
>ID24164-NO
GVLGTVKDLLIGAGKSAARVC
>ID24167-NO
GVLGTVKNLLIGAGKSAAQSVLKGLACKLSNNC
>ID24170-ABP_neg
GVLKRIKTLV
>ID24174-ABP_both
GVLSVIKNALPGIMRFIA
>ID24175-NO
GVMDFQIGLQ

>ID24176-NO
GVMGTVKDLLIGAGKSAAQSVLKTLTCKLSNNC
>ID24178-NO
GVNASSSLF
>ID24179-NO
GVNGEEGVPG
>ID24181-NO
GVNPCGGWF
>ID24182-NO
GVNWSNLRGAW
>ID24191-NO
GVPCLCDSDGPRPRGNTLSGILWFYPSGCPSGWHNCKAHGPNIGWCCKK
>ID24192-NO
GVPCRCDSDGPSVHGNTLSGTVWVGSCASGWHKCNDEYNIAYECCKE
>ID24193-NO
GVPCSCRGKSGTYWSAGKCPGEHYTTYCNNLIG
>ID24196-NO
GVPINVSCTGSKDCYAPCRKQTGCPNAKCINKSCKCYGC
>ID24197-NO
GVPINVSCTGSPQCIKPCKDAGMRFGKCMNRKCHCTPK
>ID24199-NO
GVPKVKETMVPK
>ID24202-NO
GVQGPM
>ID24208-NO
GVSCLCDSDGPSVRGNTLSGTLWLYPSGCPSGWHNCKAHGPTIGWCCKQ
>ID24209-NO
GVSLPEW
>ID24210-ABP_both
GVSSPIVAVKFKGAVASLIK
>ID24211-ABP_both
GVSVAGAKKVKVLFVFPFLF
>ID24212-NO
GVTDSSTSNLDMPHW
>ID24214-ABP_both
GVVAILKGAAKDIAGHLASKVMNKL
>ID24215-ABP_both
GVVDALKGAAKDIAGHLASKVMNKL
>ID24216-ABP_both
GVVDIAKGAAKDIAGHLASKVMNKL
>ID24217-ABP_both
GVVDILAGAAKDIAGHLASKVMNKL
>ID24218-ABP_both
GVVDILKAAAKDIAGHLASKVMNKL
>ID24219-ABP_both
GVVDILKGAAADIAGHLASKVMNKL
>ID24239-ABP_both
GVVGKLASKVVPSVFGSFTK
>ID24240-ABP_neg
GVVKRIKTVV
>ID24241-ABP_both

GVVPVVSVVGKVV
>ID24242-ABP_both
GVVVRIGRVIVRGVRR
>ID24243-ABP_both
GVVVRIGRVVVRGVRR
>ID24244-ABP_both
GVVVRVARVVVRWVRR
>ID24245-ABP_both
GVVVRVGRVVVRGVRR
>ID24246-ABP_both
GVVVRVGRVVVRWV
>ID24247-ABP_both
GVVVRVGRVVVRWVRR
>ID24248-ABP_both
GVVVRVGRVVVRWVRRRR
>ID24252-ABP_both
GVVVRWGRVWVRGVRR
>ID24255-NO
GVWDECCKDPQCRQNHMQHCPAR
>ID24256-ABP_both
GVWGIAKIAGKVLGNILPHVFSSNQS
>ID24263-NO
GVYPH
>ID24264-NO
GVYPHK
>ID24269-NO
GWASKIGQTLGKMAKVGLHELIQPK
>ID24287-ABP_both
GWGSFFKKAAHVG
>ID24292-NO
GWINEEKIQKKIDEKIGNNILGGMAKAVVHKLAKGEFQCVANIDTMGNCE
>ID24293-NO
GWINEEKIQKKIDERMGNTVLGGMAKAIVHKMAKNEFQCMANMDMLGNCE
>ID24296-ABP_neg
GWKDLLKGAAKALVKTVF
>ID24303-ABP_both
GWKRKRFG
>ID24309-ABP_both
GWLDVAKKIGKAAFNVAKNFLLFNKAVNFAAKGIKKAVDLWG
>ID24315-NO
GWLKMFKKIIGKFGKF
>ID24316-ABP_both
GWLQLALHLLQLGLHLLQLALQLRRR
>ID24317-ABP_both
GWLQLAQHLLQLGQHLLQLAQQLRRR
>ID24324-NO
GWNQLQGVW
>ID24326-NO
GWPQAPAMDGAGKTGAEEAQPPEGKGAREHSRQEEEEETAGAPQGLFRG
>ID24327-NO
GWQDLNGGW

>ID24328-NO
GWQDLNSAW
>ID24330-NO
GWQDMSSAW
>ID24331-ABP_both
GWQLLALHLQLLGLHLLQLALLQRRR
>ID24332-NO
GWRDLNGGW
>ID24337-NO
GWSMLRL
>ID24338-NO
GWTLNPAGYLLGKINLKALAALAKKIL
>ID24339-NO
GWTLNPPGYLLGKINLKALAALAKKIL
>ID24340-NO
GWTLNSAGYLLGKFLPLILRKIVTAL
>ID24344-NO
GWTLNSAGYLLGPHAIDNHRSFHDKHGLA
>ID24345-NO
GWTLNSAGYLLGPHAIDNHRSFHDKYGLA
>ID24347-NO
GWTLNSAGYLLGPHAIDNHRSFNEKHGIA
>ID24348-NO
GWTLNSAGYLLGPHAIDSHRSLGDKRGVA
>ID24349-NO
GWTLNSAGYLLGPHAVDNHRSFNDKHGFT
>ID24350-NO
GWTLNSAGYLLGPHAVDNHRSLNDKHGLA
>ID24351-NO
GWTLNSAGYLLGPHGIDGHRTLSDKHGLA
>ID24354-NO
GWTLNSKINLKALAALAKKIL
>ID24356-NO
GWVAVVGACGTVCLASGGVGTEFAAASYFL
>ID24361-NO
GWWEELLHETILSKFKITKALELPIQL
>ID24399-NO
GYALPHA
>ID24401-NO
GYCAEKGIKCHNIHCCSGLTCKCKGSSCVCRK
>ID24402-NO
GYCSWYRGWAPPDKSIINATDP
>ID24405-NO
GYEDEDEDRPFYALGLGKRPRTYSFGL
>ID24408-NO
GYFCESCRKIIQKLEDMVGPQPNEDTVTQAASQVCDKLKILRGLCKKIMR
>ID24409-NO
GYFLFRPRN
>ID24410-NO
GYGCPFNQYECHNHCKGVPGYKGGYCDGFLKMTCRCY
>ID24411-ABP_both

GYGCPFNQYQCHSHCKGIRGYKGGYCKGAFKQTCKCY
>ID24413-NO
GYGDRNFLRF
>ID24414-ABP_both
GYGGVSIPEWVCTTFAICSEK
>ID24416-NO
GYGGY
>ID24417-NO
GYGGYGGY
>ID24418-NO
GYGNCRHFKQKPRRD
>ID24419-NO
GYGRKKRRGRRRTHRLPRRRRRR
>ID24420-NO
GYGRKKRRQRRRG
>ID24421-NO
GYIRF
>ID24423-ABP_both
GYKYINNIIKYINKFFKYIW
>ID24424-ABP_both
GYLEQLLR
>ID24426-ABP_both
GYNYAKKLANLAKKFANALW
>ID24427-NO
GYPICGESCVGGICNIPGCSCSWPVCTTN
>ID24429-NO
GYPSRNYLRF
>ID24430-NO
GYPYQHRLVY
>ID24431-NO
GYQVDCVSYNYDNVNENLAQQFVDTQG
>ID24432-NO
GYRKPPFNGS
>ID24433-NO
GYRKPPFNGSIF
>ID24436-NO
GYRTCNTYF
>ID24437-NO
GYSDRNYLRF
>ID24438-NO
GYSKNYLRF
>ID24439-NO
GYSNKDFVRF
>ID24440-NO
GYSNKNFVRF
>ID24441-NO
GYSTCSYYF
>ID24448-NO
HAAESEKERAGQGVLTEEEEKELENLAAMDLELQ
>ID24450-NO
HADDLLNKAYRNLLGQLSARKYLHTLMAKHLGAVSSSLEDDSEPLS

>ID24451-NO
HADGIFSKAYRKLLGQLSARNYLHSLMAKRVGGASSGLGDEAEPLS
>ID24453-NO
HADGIFTTVYSHLLAKLAVKRYLHSLI
>ID24454-NO
HADGLLDRALRDILVQLSARKYLHSLTAVRVGEEEEDEEDSEPLS
>ID24456-NO
HADGMFNKAYRKALGQLSARKYLHTLMAKRVGGGSMIEDDNEPLS
>ID24457-NO
HADGRYTSDISSYLEGQAAKEFIAWLVNGR
>ID24458-NO
HADGRYTSDISSYLEGQAAKEFIAWLVNGRG
>ID24461-NO
HADGSFSDEMNTVLDNLATRDFINWLLHTKITDSL
>ID24462-NO
HADGSFSDEMNTVLDSLATRDFINWLLQTKI
>ID24464-NO
HADGSFTNDINKVLDIIAAQEFLDWVINTQETE
>ID24465-NO
HADGSFTSDFNKALDIKAAQEFLDWIINTPVKE
>ID24467-NO
HADGTFTSDINKILDDMAAKEFLKWLINTKVTQ
>ID24468-NO
HADGTFTSDMSSYLEEKAAKEFVDWLIKGRPK
>ID24469-NO
HADGTFTSDVSSYLKDQAIKDFVDRLKAGQVRRE
>ID24470-NO
HADGTFTSDVSSYLNDQAIKDFVAKLKSGKV
>ID24471-NO
HADGTFTSDYNQLLDDIATQEFLKWLINQKVTQ
>ID24472-NO
HADGTYTSDVSAYLQDQAAKDFITWLKSGQPKQE
>ID24473-NO
HADGTYTSDVSSYLQDQAAKDFVSWLKAGRG
>ID24474-NO
HADGTYTSDVSSYLQDQAAKKFVTWLKQGQDRRE
>ID24481-NO
HAEDVNALLDRTMAKTFIEWLEKQNSNDQTD
>ID24483-NO
HAEGTFTNDMTNYLEEKAAKEFVGWLINGRP
>ID24486-NO
HAEGTFTSDVTQHLDEKAAKEFIDWLINGGPTKEIIS
>ID24488-NO
HAEGTYTNDVTEYLEEKAAKEFIEWLIKGKP
>ID24491-NO
HAEGTYTSDITSYLEGQAAKEFIAWLVNGR
>ID24493-NO
HAEIN
>ID24494-NO
HAEYERHADGRYTSDISSYLEGQAAKEFIAWLVNGRG
>ID24499-NO

HANENIF
>ID24504-NO
HCCPIDLQCCPP
>ID24506-NO
HCTYHRHCCDLYCNKTTNVCLET
>ID24513-NO
HDRNFLRF
>ID24517-NO
HDYMRF
>ID24524-NO
HEEYQAHVQTV
>ID24526-NO
HERDPTHIKWGD
>ID24528-NO
HEVVAG
>ID24529-NO
HFAAWGGWSLVH
>ID24532-NO
HFHHALPPAR
>ID24555-NO
HGCCKGPEGCSSRECRPQHCC
>ID24560-NO
HGGYKPTDK
>ID24563-NO
HGKYFVS
>ID24565-NO
HGLGHGHEQQHGLGHGHKFKLDDDLEHQGGHVLD
>ID24570-NO
HGRFILPWWYAFSPS
>ID24572-NO
HGSTTLRDITV
>ID24578-NO
HGSYAFGL
>ID24587-NO
HHEWTHHWPPP
>ID24590-NO
HHGYKRKFHEKH
>ID24593-NO
HHHHHHTKRRITPKDVIDVRSVTTEINT
>ID24595-NO
HHQELCTKGDDALVTELECIRLRISPETNAAFDNAVQQLNCLNRACAYRK
>ID24599-NO
HHTRFVS
>ID24601-NO
HICHDYLEGDHCDPKDCNLDCRDKWKGTGTCEPPTGTPLTRTCYCTYDC
>ID24604-NO
HIDGIFTDSYSRYRKQMAVKKYLAAVLGKRYKQRVKNK
>ID24606-NO
HIGSLLR
>ID24607-NO
HIGSLYR

>ID24608-ABP_both
HIIKGL
>ID24611-NO
HIKWGD
>ID24612-NO
HILASLYKPR
>ID24614-ABP_both
HIQKEDVPSERYLGYLEQLLRLK
>ID24616-ABP_both
HIQKEDVPSERYLGYLEQLLRLKKYK
>ID24617-NO
HIQLSPFSQSWR
>ID24622-NO
HISPSYDVEIDAGNMRNLLDI
>ID24627-ABP_both
HKCAKIKWRGVHVKYCA
>ID24629-NO
HKDAFIGLM
>ID24630-NO
HKEMPFPKYPVEPF
>ID24631-NO
HKHGHGHGKHKNKGK
>ID24649-ABP_both
HKHWLWLW
>ID24650-NO
HKINSFVGLM
>ID24652-NO
HKKHKK
>ID24653-NO
HKKHKKHKK
>ID24654-NO
HKKHKKHKKHKK
>ID24655-NO
HKKHKKHKKHKKHKKHKK
>ID24656-NO
HKKHKKHKKHKKHKKHKKHKKHKK
>ID24659-NO
HKLDSFIGLM
>ID24661-NO
HKLINTEGHHS
>ID24662-NO
HKNKGKKN
>ID24669-ABP_both
HKVVFKVKFH
>ID24676-NO
HLGSLYR
>ID24677-ABP_both
HLHFPHLHFP
>ID24679-NO
HLIPFPRV
>ID24682-NO

HLLQFNKMIKFETGKNAIPFYAF
>ID24683-NO
HLLQFNKMIKFETRKNAIPFYAF
>ID24684-NO
HLLQFNKVIKFETRKNAIPFYAFYGCYCGWGGRG
>ID24686-ABP_both
HLNKRVQRELIGWLDWLK
>ID24687-NO
HLPLP
>ID24688-NO
HLPLPL
>ID24689-NO
HLPLPLL
>ID24690-NO
HLPPPV
>ID24691-NO
HLQIQPWYPQIS
>ID24692-NO
HLRGRF
>ID24699-NO
HNAYWHWPPSMT
>ID24701-NO
HNDGPGRDPAPCCQHPIETCC
>ID24706-NO
HNPASFIGLM
>ID24707-NO
HNPHLPDLF
>ID24709-NO
HNRTPENFPCKNL
>ID24713-NO
HPFAQ
>ID24714-NO
HPFAQTQ
>ID24720-NO
HPHPHLSF
>ID24721-NO
HPIKH
>ID24723-NO
HPKPNSFW
>ID24725-NO
HPLQDTEEKPRSFSTSQTDLLDDPDQMNED
>ID24728-NO
HPLSKHPYWSQP
>ID24729-NO
HPPCCLYGKCRRYPGCSSASCCQ
>ID24738-NO
HQAAGW
>ID24740-NO
HQHKPPPLTNNW
>ID24741-NO
HQIYP

>ID24744-NO
HQRSTGF
>ID24746-NO
HQSVNKE
>ID24747-NO
HQTLSGIDQEQNNLTRLIEAQIHELQK
>ID24750-ABP_pos
HRCRVYNNGLPTGLYRWC
>ID24753-NO
HRHIRRQSLIML
>ID24756-ABP_both
HRHRHRHRHR
>ID24758-ABP_both
HRKKHHGKRNSNRAHQGKHETYGHKTPY
>ID24759-ABP_both
HRKKWFW
>ID24768-ABP_both
HRRSVAHQEEASLHVKTDELPSPDTVREQL
>ID24769-ABP_both
HRRSVAHQQQASLHVKTNQLPSPNTVRQQL
>ID24773-ABP_both
HRWWRWWRH
>ID24776-NO
HSACAANCLSMGKAGGRCENGVCLCR
>ID24777-NO
HSCVCRRICAARQVRKGRCSRRRRICCLY
>ID24779-NO
HSDAIFTQQYSKLLAKLALQKYLASILGSRTSPPP
>ID24780-NO
HSDALFTDTYTRLRKQMAMKKYLNSVLN
>ID24781-NO
HSDATFTAEYSKLLAKLALQKYLESILGSSTSPRPPSS
>ID24782-NO
HSDAVFTDNYSRFRKQMAAKKYLNS
>ID24783-NO
HSDAVFTDNYSRFRKQMAVKKYLNSVLT
>ID24784-NO
HSDAVFTDNYSRIRKQMAVKKYINSLLA
>ID24785-NO
HSDAVFTDNYSRYRKQMAAKKYLNSVLA
>ID24786-NO
HSDGIFTDSYSRYRKQMAVKKYLAAVLGKRYKQ
>ID24788-NO
HSDGIFTDSYSRYRKQMAVKKYLAAVLGKRYRQRYRNK
>ID24790-NO
HSDGLFTSEYSKMRGNAQVQKFIQNLM
>ID24791-NO
HSDGSFTNDMNVMLDRMSAKNFLEWLKQQGRG
>ID24792-NO
HSDGTFTSDLSKQMEEEAVRLFIEWLKNGGPSSGAPPPS
>ID24795-NO

HSEFERHAEGTYTSDITSYLEGQAAKEFIAWLVNGRG
>ID24796-NO
HSEGTFSNDYSKYLEDRKAQEFVRWLMNN
>ID24797-NO
HSEGTFSNDYSKYLETQRAQDFVQWLMNS
>ID24798-NO
HSEGTFSNDYSKYLETRRAQDFVEWLMNS
>ID24801-NO
HSEGTFSNDYSKYQEERMAQDFVQWLMNS
>ID24802-NO
HSEGTFSSDYSKYLDSRRAKDFVQWLMST
>ID24803-NO
HSEGTFTSDYSKYLDNRRAKDFVQWLMNT
>ID24804-NO
HSEGTFTSDYSKYLENKQAKDFVRWLMNA
>ID24805-NO
HSFLHPWDLFDY
>ID24806-NO
HSGIQSEPKAIP
>ID24807-ABP_both
HSIIGRKLVVHKKAK
>ID24814-NO
HSQGSFTSDYSKHLDVKQAKDFVTWLLNT
>ID24815-NO
HSQGTFTNDYSKYLDTRRAQDFVQWLMST
>ID24816-NO
HSQGTFTNDYSKYLDTRRAQDFVQWLMSTKRSGGIT
>ID24817-NO
HSQGTFTSDYSKFLDTRRAQDFLDWLKNT
>ID24819-NO
HSQGTFTSDYSKHLDSRYAQEFVQWLMNT
>ID24820-NO
HSQGTFTSDYSKYLDSRRAQDFVQWLMNS
>ID24821-NO
HSQGTFTSDYSKYLDSRRAQDFVQWLMNSKRSGGIS
>ID24823-NO
HSQGTFTSDYSKYLDSRRAQQFLKWLLNV
>ID24824-NO
HSQGTFTSDYSKYLDSRRAQQFLKWLLNVKRNRNNIA
>ID24829-ABP_both
HSSGYTRPLRKPSRPIFIRPIG
>ID24832-NO
HSSIQSQPQAFT
>ID24833-NO
HTAGFIPRL
>ID24834-ABP_both
HTASDAAAAAALTAANAAAAAAASMA
>ID24835-ABP_both
HTCHFRRRPKVRKFKLYHEGKFWCPG
>ID24837-NO
HTCLVALCA

>ID24838-NO
HTEESGEKHNAFSN
>ID24839-NO
HTFEPGV
>ID24841-ABP_both
HTGRSGPATGHSGHSSTHGS
>ID24843-NO
HTMYYHHYQHHL
>ID24846-NO
HVDGSFTSDVNKVLDSLAAKEYLLWVMTSKTSG
>ID24848-NO
HVFLRF
>ID24850-NO
HVGGDECNINEHRSLVAIFVFTEFF
>ID24856-NO
HVRHFYGLM
>ID24857-NO
HVTCFYVKFGCKHTECITTIVFCWQTASDISSV
>ID24858-NO
HVVNFTPRL
>ID24859-NO
HWAWFK
>ID24863-ABP_pos
HWRLLLLKKH
>ID24864-NO
HWSHDWKPG
>ID24867-NO
HWTTQR
>ID24890-NO
HYRIKPTFRRLKWKYKGKFA
>ID24894-ABP_both
IAAAGLPQE
>ID24895-ABP_both
IAAAQRITSGAADIAINWAGGLHHAKK
>ID24896-ABP_both
IAAERRYATIYQARLWAF
>ID24898-ABP_both
IAAQGLPQE
>ID24902-NO
IADECCSNPACRVNNPHVC
>ID24909-NO
IAGAHKCKPCYGYKTNSCCNSPN
>ID24910-ABP_both
IAGERRYGTIYQGRLWAF
>ID24912-ABP_both
IAHQGLPQE
>ID24914-NO
IAIPP
>ID24920-NO
IAMAISSGACCAYPPCFEAYPERCL
>ID24926-NO

IARALFEKKV
>ID24928-ABP_neg
IARRALCASLRARHTIPQCKKFGRR
>ID24929-ABP_neg
IARRALKASLRARHTIPQCKKFGRR
>ID24930-ABP_neg
IARRALKKAKRAAHKIPAAKKFARR
>ID24931-ABP_neg
IARRALKKAKRAAHKIPAAKKFGRR
>ID24933-ABP_neg
IARRALKKAKRAAHKIPQCKKFGRR
>ID24934-ABP_neg
IARRALKKAKRAAHTIPQCKKFGRR
>ID24937-ABP_neg
IARRALKKSLRARHTIPQCKKFGRR
>ID24940-ABP_both
IASAVPVCVKGKISKSYISV
>ID24941-NO
IASGEPTSTPT
>ID24943-NO
IASGEPTSTPTTEA
>ID24944-ABP_both
IASKVANTVQKLKRKAKNAV
>ID24946-NO
IASVRWA
>ID24948-NO
IATSPYYACNCP
>ID24949-NO
IATSPYYACNCPNNCKHKKGSGC
>ID24952-NO
IAWVKAFIRKLRKGPLG
>ID24954-NO
IAYKP
>ID24955-NO
IAYKPAG
>ID24963-NO
ICCYPNEWCCD
>ID24971-ABP_both
ICLKKWPWWPWRRCK
>ID24972-NO
ICLLAHCA
>ID24973-NO
ICLLVQARVRPRVC
>ID24974-NO
ICLLVQARVRPRVCLKHYLVNFLTIVKT
>ID24975-NO
ICRDL
>ID24976-NO
ICTMIPISRCY
>ID24986-ABP_both
IDGLKAIWKKVADLLKNT

>ID24991-NO
IDKQLE
>ID24992-NO
IDLMQAR
>ID24993-NO
IDLSRFYGHF
>ID24995-NO
IDLSRFYGHFNT
>ID24997-NO
IDPNG
>ID24999-NO
IDPPRYCNHIICYEDSECSQWCTAGCNSITSKCDT
>ID25003-NO
IDSVKTFCCSTFNLGICCSKK
>ID25013-NO
IEAIRCGGSRDCYRPCQKRTGCPNAKCINKTCKCYGCS
>ID25024-NO
IEFEQ
>ID25048-NO
IEKPP
>ID25050-NO
IELLQAR
>ID25052-NO
IELPLG
>ID25055-NO
IEPQG
>ID25061-NO
IEWEG
>ID25067-NO
IFGAIAGFIKN
>ID25070-ABP_both
IFGAIWSGIKSLF
>ID25071-ABP_both
IFGLLLHGAIHVGKLIHGLVRRHGEEQLDDLEQLDKRALDYNPGRPGFD
>ID25078-NO
IFLLWQR
>ID25080-NO
IFNKAYRKVLGQLSARKYLHSLM
>ID25084-ABP_neg
IFPIVKKLLSGLF
>ID25087-NO
IFSGSRE
>ID25091-NO
IFVPAF
>ID25094-NO
IGCRH
>ID25097-NO
IGGSI
>ID25102-ABP_both
IGKFLKKAKKFGKAFVK
>ID25103-NO

IGKGG
>ID25104-ABP_neg
IGKLPKWIIKTVNKFTKK
>ID25106-ABP_both
IGLRGLGRKIALIHKKYG
>ID25107-NO
IGLRLPNML
>ID25109-NO
IGNTLI
>ID25112-NO
IGSENSEKTTMP
>ID25113-NO
IGSLSVVNSVDVLRERVLLELARRKAMENQQQLGENQYVFKSV
>ID25114-NO
IGSTAPTCTYNECRGCRYKCRAEQVPVEGNDPINSAYHYRCVCHR
>ID25115-NO
IGTISLSLCEQERDADEDEGETLEE
>ID25119-NO
IGYNQ
>ID25120-NO
IHAQQKEP
>ID25122-NO
IHEGEEGEAEEE
>ID25123-NO
IHETT
>ID25125-ABP_both
IHFKWRRWKFHI
>ID25126-NO
IHFPSAS
>ID25127-NO
IHFRIGRRRRRRRR
>ID25128-ABP_both
IHGLV
>ID25129-ABP_both
IHHQGLPQE
>ID25131-ABP_both
IHKFWRCRRRFCRWFKHI
>ID25132-ABP_both
IHKFWRGGRWFKHI
>ID25141-ABP_neg
IHRDQQHESFLDARPEPGLTE
>ID25144-NO
IHWRPWAR
>ID25148-NO
IIALPLGYFSK
>ID25149-NO
IIAMK
>ID25150-NO
IICAPEGGPCVAGIGCCAGLRCSGAKLGLAGSCQ
>ID25165-NO
IIFTPKL

>ID25176-ABP_both
IIIIRRRR
>ID25177-NO
IIIIVGG
>ID25179-NO
IIKNLQNLDPSHRISDRDYMGWMDF
>ID25182-NO
IILDALEELD
>ID25184-NO
IINWCCLIFYQCC
>ID25186-NO
IINWCCLTFYQCC
>ID25187-NO
IINWCCLVFYQCC
>ID25189-ABP_both
IIPLPLGYFAKKKKKKDPVPLDQ
>ID25195-ABP_both
IIRKIIRK
>ID25198-ABP_both
IISTIGDLVKWIIDTV
>ID25199-ABP_both
IISTIGKLPKWIIKTVNKFTKK
>ID25200-ABP_both
IISTIGKLVKWIIKTVNKFTKK
>ID25203-NO
IIVTQTMK
>ID25205-NO
IIYRDLISH
>ID25209-ABP_both
IKAQGLPQE
>ID25210-NO
IKARASP
>ID25213-NO
IKCTLSKDCYSPCKKETGCPRAKCINRNCKCYGCS
>ID25215-NO
IKDAAKLIGKTVAKEAGKTGLELMACKITNQC
>ID25216-NO
IKELQ
>ID25219-ABP_both
IKFEPPLPPKKAH
>ID25221-ABP_both
IKGLFHAGKMIHGLV
>ID25223-ABP_both
IKHAGLPQE
>ID25224-ABP_both
IKHQGLPQA
>ID25225-ABP_both
IKHQGLPQED
>ID25226-ABP_both
IKHQGLPQEE
>ID25228-ABP_both

IKHQGRPQE
>ID25230-ABP_both
IKHRGLPQE
>ID25236-NO
IKIPSFFRNILKKVGKKAVSLIAGALKQS
>ID25238-NO
IKIWFQNRRMKWKK
>ID25263-ABP_both
IKKIIKKI
>ID25264-ABP_both
IKKIIKKIIK
>ID25265-ABP_both
IKKIIKKIIKKI
>ID25266-ABP_both
IKKILSKIKKLL
>ID25268-ABP_both
IKKILSKIKKLWK
>ID25270-ABP_both
IKKILSKIKKWWK
>ID25271-ABP_both
IKKIVSKIKKLL
>ID25272-ABP_both
IKKIVSKIKKLLK
>ID25275-ABP_both
IKKIWSKIKKLLK
>ID25276-ABP_both
IKKIWSKIKKLWK
>ID25277-ABP_both
IKKIWSKIKKWLK
>ID25278-ABP_both
IKKIWSKIKKWWK
>ID25286-ABP_both
IKKVASV
>ID25287-ABP_both
IKKVASVIGGL
>ID25292-ABP_both
IKLRNVLKYLFRIDVIKEDIL
>ID25293-ABP_both
IKLSKETKDNLKKVLKGAIKGAIAVAKMV
>ID25294-ABP_both
IKLSKETKKNLKKVLKGAIKGAIAVAKMV
>ID25301-ABP_both
IKNAKVCVYAVCVSHK
>ID25302-NO
IKNGYPRDS
>ID25304-NO
IKNTAASNKAPSLVAIAVRGCCYNPSCWPKTYCS
>ID25310-NO
IKPLDY
>ID25311-NO
IKPLNY

>ID25312-NO
IKPVQ
>ID25315-ABP_both
IKQGLPQE
>ID25316-ABP_both
IKQLLHFFQRFGGGFIKHFIHRF
>ID25319-ABP_both
IKQVKKLFKK
>ID25328-ABP_both
IKSIASKVANTVQKLKRKAKNAVA
>ID25333-ABP_both
IKWAGKLFKLFK
>ID25334-ABP_both
IKWAGKWWKLFK
>ID25335-NO
IKWGD
>ID25336-ABP_both
IKWKAILDAVKKVI
>ID25337-ABP_both
IKWKRWWWR
>ID25342-ABP_both
IKYLLVKLQGASQKTITLMLRRN
>ID25343-ABP_both
IKYLLVKLQGASQKTITLMLRRNNLYVMGYS
>ID25347-NO
ILAWKWAWWAWPP
>ID25349-ABP_neg
ILAWKWPWWAWRR
>ID25356-ABP_both
ILENLLARSTNEDREGSIF
>ID25360-ABP_both
ILGAAWNGAKSLF
>ID25362-ABP_both
ILGAVWNGVKSLF
>ID25365-ABP_both
ILGKAWEGAKSLF
>ID25366-ABP_both
ILGKIWEGIESLF
>ID25367-ABP_both
ILGKIWKGIKNIL
>ID25368-ABP_both
ILGKIWKPIKKLF
>ID25371-ABP_both
ILGKLWEGLKSLF
>ID25373-ABP_both
ILGKVWEGVKSLF
>ID25374-NO
ILGPALGLVGNALGGLLKNL
>ID25378-NO
ILGPVIGTIGNVLGGLIKKI
>ID25379-NO

ILGPVIGTTGNVLGGLLKNL
>ID25381-NO
ILGPVISKIGVVLGGLLKNL
>ID25383-NO
ILGPVLDLVGRALRGLLKKI
>ID25384-NO
ILGPVLGLAGNALGGLIKKI
>ID25385-NO
ILGPVLGLDSNALEGLIKKI
>ID25388-NO
ILGPVLGLVDSALGGLLKYL
>ID25391-NO
ILGPVLGLVGNALGGYLKIL
>ID25392-NO
ILGPVLGLVGNDLEVYLKIL
>ID25395-ABP_both
ILGPVLGLVGSALGGLI
>ID25396-NO
ILGPVLGLVGSALGGLIKRL
>ID25397-NO
ILGPVLGLVGSALGGLLKNL
>ID25401-NO
ILGPVLGLVSNALDDVLGIL
>ID25402-NO
ILGPVLGLVSNALGGLIKRI
>ID25404-NO
ILGPVLSMVGSALGGFFKKI
>ID25405-NO
ILGPVVGLVGNALGGLLKNL
>ID25407-ABP_both
ILGTIL
>ID25410-ABP_both
ILGTILGPLKGL
>ID25411-ABP_both
ILGTILPLLKGL
>ID25415-NO
ILIIVGG
>ID25417-ABP_both
ILIKRK
>ID25425-ABP_neg
ILKKLLEGVKSI
>ID25426-ABP_neg
ILKKLLKGVKSI
>ID25427-ABP_neg
ILKKLLKKVKKI
>ID25429-ABP_neg
ILKKLWEGVKSI
>ID25433-ABP_both
ILKWKWKWWPWRR
>ID25434-NO
ILLPVLGLVGNALGGLLKNL

>ID25440-NO
ILNRCCNDDN
>ID25446-ABP_pos
ILPFIQSLFPFAER
>ID25449-NO
ILPIFSWIGHLFGK
>ID25453-NO
ILPILGKILSTILGK
>ID25458-NO
ILPLIASLIGGLLGK
>ID25461-NO
ILPLVGNLLNDLLGK
>ID25467-ABP_both
ILPSKLCRLLGNC
>ID25478-NO
ILPWGWPWWPWPP
>ID25484-ABP_both
ILPWKKPKKPWRR
>ID25507-ABP_both
ILPWKWPKKPWRR
>ID25558-ABP_both
ILPWKWPWWPWKK
>ID25619-ABP_both
ILQDIWNGIKNLF
>ID25620-NO
ILQQLLFIHFRIGRRRRRRRR
>ID25622-NO
ILQRYLDHPWCCAVKYSCCVPGIIPI
>ID25624-ABP_both
ILRAWKKYGPIIVPIIRI
>ID25629-NO
ILSAIWSGIKS
>ID25630-NO
ILSGAPCIPW
>ID25632-ABP_both
ILSLRWWRKWWKK
>ID25638-ABP_pos
ILVRWIRWRIQW
>ID25642-NO
ILWWLLAWWRWPH
>ID25646-NO
IMDILIIVGG
>ID25647-NO
IMGPVLGLVSNALGGLLKNL
>ID25652-NO
IMVAEAR
>ID25656-NO
IMYPGWL
>ID25658-NO
INCDFLL
>ID25659-NO

INDPF
>ID25663-NO
INGKVT
>ID25664-NO
INGSLDKRLLPDVET
>ID25665-NO
INGSLDKRVQDCYHG
>ID25669-NO
INKYREWKNKKN
>ID25670-NO
INLEACLGRTLMD
>ID25671-NO
INLEACLKRGRT
>ID25672-NO
INLKAIAALAKKL
>ID25682-NO
INNQFLP
>ID25683-NO
INNQFLPYPYYAKPAAVR
>ID25692-NO
INVKCSLPQQCIKPCKDAGMRFGKCMNKKCRCYS
>ID25697-ABP_both
INWKKWWQVFYTVV
>ID25699-ABP_both
INWLKLGKKIISAL
>ID25707-NO
IPAIN
>ID25708-NO
IPALK
>ID25709-NO
IPAQK
>ID25710-ABP_pos
IPAVFK
>ID25723-NO
IPEPYVWD
>ID25726-NO
IPGYSHSFV
>ID25729-NO
IPICAWKVCPPTPW
>ID25732-NO
IPIYE
>ID25737-NO
IPMIK
>ID25738-NO
IPMLK
>ID25741-NO
IPNPIGSE
>ID25743-NO
IPPGVPYWT
>ID25744-NO
IPPLTQ

>ID25745-NO
IPPLTQTPV
>ID25746-NO
IPPQFMRF
>ID25748-NO
IPPVPP
>ID25751-NO
IPQEVLP
>ID25755-NO
IPSRWKDQFWKRWHY
>ID25757-NO
IPVCIWKVCPPIPW
>ID25758-NO
IPVLCPVCTSLVGKLIDLVLGGAVDKVTDYLETLCAKADGLVETLCTKIV
>ID25760-ABP_both
IPWGKVKDFLVGGMKAV
>ID25761-NO
IPWKLPATLRPVENPFSKPLCRNY
>ID25769-NO
IQHST
>ID25771-NO
IQKEDVPSER
>ID25777-ABP_pos
IQRWWKVWLKVI
>ID25779-NO
IQWCA
>ID25780-NO
IQYCA
>ID25784-NO
IRAECCSNPACRVNNPHVC
>ID25792-NO
IRCQGSNQCYGHCREKTGCMNGKCINRVCKC
>ID25793-NO
IRCQGSNQCYGHCREKTGCPNGKCID
>ID25794-ABP_both
IRCRRRFCRI
>ID25795-NO
IRCTGSKECYSPCYKATGCPNAKC
>ID25796-NO
IRCTGSKECYSPSY
>ID25798-NO
IRDACCSNPACRVNNPHVC
>ID25800-NO
IRDECCANPACRVNNPHVC
>ID25825-NO
IREFV
>ID25826-NO
IRFVT
>ID25827-ABP_both
IRGGRAAVLNALGKEEQIGRAS
>ID25832-ABP_both

IRHQGLPQE
>ID25834-ABP_both
IRIKIR
>ID25835-ABP_both
IRIKIRIKIRIK
>ID25836-NO
IRILQQLLFIHFRIGRRRRRRRR
>ID25838-ABP_both
IRIRCRRRFCRIRI
>ID25839-ABP_both
IRIRIRIR
>ID25860-ABP_both
IRPIPFIPRGGKT
>ID25862-ABP_neg
IRPRPPRLPRPRPRPLPYPRP
>ID25863-ABP_both
IRPVPFFPPVHAKKVFPLH
>ID25864-NO
IRPVQ
>ID25869-NO
IRRGQ
>ID25873-ABP_both
IRRQGLPE
>ID25879-ABP_both
IRTVVAGYNLYRAIKKK
>ID25882-ABP_both
IRVKIRVK
>ID25888-NO
IRWCT
>ID25893-NO
IRYCT
>ID25895-NO
IRYLPRG
>ID25898-ABP_neg
ISAAEFGKINGPIKK
>ID25899-NO
ISCAVDAC
>ID25914-NO
ISGTSMSCPHVAGRAYVLDTSLRVYLLDTGLR
>ID25915-NO
ISHIYVWK
>ID25916-NO
ISIDPPCRFCYHRDGSGNCVYDAYGCGAV
>ID25919-NO
ISINQDLKAITDML
>ID25920-NO
ISINQDLKAITDMLLTEQIQARQRCLAALRQRLLDL
>ID25921-NO
ISINQDLKAITDMLLTEQIQARRRCLAALRQRLLDL
>ID25924-NO
ISINQDLKAITDMLLTEQIRERQRYLADL

>ID25925-NO
ISINQDLKAITDMLLTEQIRERQRYLADLRQRLLEK
>ID25927-ABP_both
ISKRILTGKK
>ID25930-NO
ISLLGAR
>ID25931-NO
ISLLQAR
>ID25932-ABP_both
ISLNPPRSTIAMRAINNYRWRSKNQNTFLR
>ID25941-ABP_both
ISRLAGLVRKGGEKFGEKLRKIGQKIKEFFQKLALEIEQ
>ID25943-NO
ISSEVHIPL
>ID25944-NO
ISSSK
>ID25954-NO
ITCCTRGTCAQHC
>ID25959-NO
ITDMAA
>ID25963-NO
ITFSKIYRSCKSDSDCGNQKCARGRCV
>ID25964-NO
ITFSKIYRSCKSNSVCGNQKCVRGRCV
>ID25971-NO
ITGQGNRIF
>ID26006-NO
ITMQGIQGQKIRMIMF
>ID26012-NO
ITRINKK
>ID26013-NO
ITRTQ
>ID26018-NO
ITTNPY
>ID26019-NO
ITTPWDEMRSFL
>ID26027-NO
IVADYQR
>ID26029-NO
IVAVLFLTACQFNAADDSRVRRNAEH
>ID26032-NO
IVCNFEGHCVTSDDCINVCKSGEDPFLCVRSGPHKGMCCCLKTNGSVLE
>ID26043-NO
IVGRPR
>ID26044-NO
IVGRPRH
>ID26046-NO
IVGRPRHQG
>ID26056-NO
IVPFLLGMVPKLICLITKKC
>ID26057-NO

IVPNSVEQKH
>ID26060-NO
IVQQCTSGICSLYQENYCN
>ID26062-ABP_both
IVRPPIRCKAAFC
>ID26063-NO
IVRRACCSDRRCRWRCG
>ID26084-NO
IWFQNRRMKWKK
>ID26085-ABP_both
IWGLIAHGVGHVGRLIHGLIRG
>ID26087-NO
IWHHT
>ID26094-ABP_both
IWKRWWWKR
>ID26095-ABP_pos
IWLRLKVVLKRK
>ID26099-ABP_both
IWLTALKFLGKNLGKLAKQQLAKL
>ID26107-ABP_both
IWQWIVGGLGFLAGDAWSHSDQISSGIKKRKKKGYGY
>ID26113-ABP_both
IWSFLIKAATKLLGVGSLFGGGKKDS
>ID26114-ABP_both
IWSFLIKAATKLLPSLFGG
>ID26138-NO
IYCPGQECE
>ID26139-NO
IYEGY
>ID26140-NO
IYFIADKMGIQLAPAWYQDIVNWVSAGGTLTTGFAIIVGVTVPAWIAEAA
>ID26147-NO
IYLPLFASRL
>ID26148-NO
IYNAIWPCKHCNKCKPGLLCKK
>ID26156-NO
IYRDLISH
>ID26158-NO
IYSFDGRDIMTDPSWPQKVIWHGSSPHGVRLVDNYCEAWRTA
>ID26159-NO
IYSNN
>ID26160-NO
IYWIADQFGIHLATGTARKLLDAVASGASLGTAFAAILGVTLPAWALAAA
>ID26163-ABP_both
IYYPPNHNFP
>ID26167-ABP_both
KACFRVCYRGICYRRCR
>ID26169-NO
KAEDLARYYSALRHYINLITRQRY
>ID26171-ABP_pos
KAFAKLAARKA

>ID26172-NO
KAFDITYVRLKF
>ID26173-NO
KAFITLALGQEGCCPSGPCHFAACNPPCCT
>ID26175-ABP_both
KAGLQFPVGRIARFLK
>ID26179-ABP_both
KAIAKSILKWIKSIAKAI
>ID26183-ABP_both
KAKAKAVSRSARAGLQFPVGRIHRHLK
>ID26184-NO
KAKCAEIDQDCKTSCDCCKGACTCY
>ID26186-NO
KAKFKAH
>ID26187-NO
KAKFKAM
>ID26188-NO
KAKFKAP
>ID26189-NO
KAKFKAQ
>ID26190-NO
KAKFKAR
>ID26191-NO
KAKFKAT
>ID26192-ABP_both
KAKKFGKAFVKI
>ID26193-ABP_pos
KAKKHRCRVYNNGLPTGLYRWC
>ID26197-NO
KAKTCTVLY
>ID26199-NO
KALAALLKKLAKLLAALK
>ID26200-NO
KALKLKLALALLAKLKLA
>ID26201-NO
KALPMHIRLAF
>ID26206-ABP_both
KAMKPW
>ID26207-ABP_both
KAMKPWIQPKTKVIP
>ID26208-ABP_both
KAMKPWIQPKTKVIPYVRYL
>ID26212-NO
KAMSWYA
>ID26213-NO
KAPAEDLARYYSALRHYINLITRQRY
>ID26216-NO
KAPSGRVSMIKNLQSLDPSHRISDRDYMGWMDF
>ID26217-NO
KAPVA
>ID26228-ABP_both

KARQWQWKIRRTWPIFAIRR
>ID26230-ABP_both
KARRAVRWI
>ID26233-ABP_both
KARRWVRAI
>ID26235-NO
KASNALKDMCKKMNYKAPFCAGLKPQ
>ID26236-NO
KASSSAPKGWTHHGSRFTFHRGSM
>ID26237-NO
KAVAAKKSPKKAKKPATPKKAAKSPKKVKKPAAAAKKAAKSPKKATKAAK
>ID26243-NO
KAVPYPQ
>ID26251-NO
KCAAAGEACVIPIIGNVFCCKGYCLFVCIS
>ID26253-NO
KCCKWRWRCK
>ID26254-NO
KCCMRPICMCPCCIGAG
>ID26259-NO
KCCSNPACNRYNKLC
>ID26260-NO
KCCSNPACNRYNPAICD
>ID26261-NO
KCCTMSVCQPPPVCTCCA
>ID26263-NO
KCFMWQEMLNKAGVPKLRCARK
>ID26264-NO
KCFQWQRNMRKVRGPPVSCIKR
>ID26266-NO
KCGCRWRWKCGCKK
>ID26267-NO
KCGHKHQCAVHN
>ID26268-NO
KCHFDEN
>ID26269-ABP_both
KCKKCLLKLLKKLLKLKL
>ID26270-NO
KCLAEAADCSPWSGDSCCKPYLCSCIFFYPCSCRPKGW
>ID26288-NO
KCNTATCATQRLANFLVHSNNNLGPVLSPTNVGSNTY
>ID26291-NO
KCNTATCATQRLTNFLVRSSHNLGAALPPTKVGSNTY
>ID26292-NO
KCPGSSFCDSKCKLRCSKAGLADRCLKYCGICCEECKCVPSGTYGNKHEC
>ID26294-NO
KCPSRRPKR
>ID26299-ABP_both
KCRRYCYRQRCVTYCRGR
>ID26300-NO
KCRWRWKCCKK

>ID26301-NO
KCSGKWAIHACWGGN
>ID26302-NO
KCTSPK
>ID26309-NO
KCYNN
>ID26313-NO
KDCRWRWKCCKK
>ID26317-NO
KDDYPVDTAK
>ID26320-NO
KDERF
>ID26321-NO
KDFCHLPPKPGPCRAAI
>ID26324-NO
KDGYIIEHRGCKYSCFFGTNSWCNTECTLKKGSSGYCAWPACWCYGLPDN
>ID26325-NO
KDGYLMEYGGCKMSCLMKKGTGCAEECT
>ID26326-NO
KDGYLMGADGCKLCVLTAPYDYCACE
>ID26327-NO
KDGYLPGNEGCKY
>ID26329-NO
KDGYLVGNDGCKYSCFTRPGTYCANECSRVKGKDGYCYAWMACYCYSMPN
>ID26331-NO
KDGYPVDNANCKYE
>ID26336-NO
KDKMKAGWER
>ID26338-NO
KDLHTVVSAILQA
>ID26345-NO
KDQDK
>ID26352-NO
KDYRL
>ID26355-NO
KECCTHPACHVSHPELCG
>ID26370-NO
KEGYIVNYHTGCKYTCAKLGDNDYCLRECK
>ID26371-NO
KEGYLFGSRG
>ID26372-NO
KEGYLLGSRG
>ID26380-NO
KEGYPKNSEGCKITCLFNDPYCKGLCINLSTQADY
>ID26390-NO
KEPAYQRFL
>ID26391-ABP_both
KESRAKKFQRQHMDSDSSPSSSSTYSNQMMRRRNMTQGRSKPVNTFVH
>ID26392-NO
KESVRLCGLEYIRTVIYICASSRW
>ID26404-NO

KETWWETWWTEWSQPGRKKRRQRRRPPQ
>ID26405-NO
KEVKRGCVATCPKPKKNEIIQCCAKDKCNK
>ID26409-ABP_both
KFASKGLGKDLAKLGVDLVACKISKQC
>ID26410-NO
KFAWPQ
>ID26411-NO
KFAYPQ
>ID26414-ABP_both
KFDPLEGAPMARGIVLEKVG
>ID26418-ABP_both
KFFKKLKKAVKKGFKKFAKV
>ID26420-ABP_both
KFFRKLAKSVKKAAKEFFKKPRVIGVSIPF
>ID26423-ABP_both
KFGKAFVKILKK
>ID26425-NO
KFGKVLKFLAKTLAKHLAKKQAQS
>ID26426-ABP_both
KFGYRYDSLELEGRSISRIDELIQQR
>ID26429-NO
KFHSGIQSEPKAIP
>ID26430-NO
KFHTFPQTAIGVGAP
>ID26447-ABP_both
KFKLKSLKIIEYKQ
>ID26470-ABP_both
KFWKLLKKALRLWAKVL
>ID26471-ABP_both
KFWKLLKKALRLWKKVL
>ID26472-ABP_both
KFWSLLKKALRLWANVL
>ID26478-ABP_both
KGCALVKVRGLTLKVCK
>ID26479-NO
KGCCSNPACAGNNPHVCRQ
>ID26480-NO
KGCGTRQCW
>ID26481-NO
KGCLPRNRFCNALSGPRCCSGLRCKELSIWASKCL
>ID26482-NO
KGEAMHGQVDCSPGI
>ID26490-NO
KGHHGKHG
>ID26494-ABP_both
KGIRGYKGGYCKGAFKQTCKCY
>ID26496-ABP_both
KGIVGMLGKLF
>ID26500-NO
KGKKIFIMK

>ID26506-ABP_both
KGLFHAGKMIH
>ID26519-NO
KGQPLCCPFGGCHELCHCCGS
>ID26521-ABP_both
KGRGKQGGKVRAKAK
>ID26522-ABP_both
KGRGKQGGKVRAKAKTR
>ID26523-NO
KGRGKRRRCKQRPSDCPRR
>ID26524-NO
KGRGKRRRECQRPSCKPRR
>ID26525-NO
KGRGKRRREKQRPCDKPRR
>ID26527-NO
KGRGKRRREKQRPSDKPR
>ID26529-NO
KGRTPIKFGKADCDRPPKHSQNGMGK
>ID26531-NO
KGSKKAVTKAQKKDGKKRKRSRKESYSVYVYKVLKQ
>ID26534-NO
KGVSLSYR
>ID26538-NO
KGYGGVSL
>ID26539-NO
KGYGGVSLPEW
>ID26543-NO
KHEYLRF
>ID26546-ABP_both
KHGATVKTALGKILK
>ID26547-NO
KHGHGHGK
>ID26551-NO
KHIQKEDVPSER
>ID26553-ABP_both
KHKHKHKHKH
>ID26554-NO
KHKNYLRF
>ID26558-NO
KHLPLP
>ID26559-ABP_both
KHLWLW
>ID26560-NO
KHMHWHPPALN
>ID26564-ABP_both
KHQGLPQE
>ID26569-ABP_both
KHVGKAALTHYL
>ID26572-NO
KHWWKHDWWRWRR
>ID26576-NO

KIAEKFSGNRR
>ID26577-ABP_both
KIAGKAGKIAKIAGKAGKIA
>ID26578-ABP_both
KIAGKIAAIAGKIAKIAGAIAKIAGK
>ID26579-ABP_both
KIAGKIAAIAGKIAKIAGAIAKIAGKIA
>ID26580-ABP_both
KIAGKIAAIAGKIAKIAGKIAK
>ID26581-ABP_both
KIAGKIAAIAGKIAKIAGKIAKIA
>ID26582-ABP_both
KIAGKIAKIAGKIA
>ID26583-ABP_both
KIAGKIAKIAGKIAK
>ID26584-ABP_both
KIAGKIAKIAGKIAKIA
>ID26585-ABP_both
KIAGKIAKIAGKIAKIAGK
>ID26586-ABP_both
KIAGKIAKIAGKIAKIAGKIAK
>ID26588-ABP_both
KIAGKIAKIAGKIAKIAGKIAKIAGK
>ID26591-ABP_both
KIAGKIAKIAKKIAKIA
>ID26598-ABP_both
KIAKGALKALKIAKGALKAL
>ID26599-ABP_both
KIAKGALKALKIAKVALKAL
>ID26600-ABP_both
KIAKKIAKIAKKIA
>ID26603-ABP_both
KIAKVALKALKIAKVALKAL
>ID26604-ABP_both
KIALKALKALKALGKALKAL
>ID26610-NO
KIEEIESKQKKIENEIARIKKLLQLTVWGIKQLQARIL
>ID26612-NO
KIFEPLR
>ID26613-NO
KIFEPLRDKN
>ID26615-NO
KIFEPLREDNL
>ID26616-NO
KIFEPLVA
>ID26617-ABP_neg
KIFKKIEKVGRNVRDGIIKAGPAVAVVEQA
>ID26620-NO
KIGAK
>ID26621-NO
KIGAKIK

>ID26622-NO
KIGAKIKIGAK
>ID26623-NO
KIGAKIKIGAKIK
>ID26626-ABP_both
KIGAKIKWGAKIKIGAKI
>ID26629-ABP_both
KIGKAIKWGKAIKIGKAI
>ID26630-ABP_both
KIGKALGKALKALGKALGKA
>ID26633-NO
KIHPFAQAQ
>ID26634-NO
KIHPFAQTQSLVYP
>ID26635-ABP_pos
KIIKVVK
>ID26638-ABP_both
KIKEKLKKIGQKI
>ID26640-ABP_both
KIKGAIKWKGAIKIKGAI
>ID26641-ABP_both
KIKKGFKKIFKRLPPIGVGVSIPLAGKR
>ID26643-NO
KIKKIENEIARIKKLLQLTVWGIKQLQARIL
>ID26645-NO
KIKSCYYLPCFVTS
>ID26648-NO
KIKWLKAMKSIAKFIAKKQMKKHLGGE
>ID26651-ABP_both
KILGKLWKGVKSIF
>ID26652-ABP_both
KILGVSKKIMRRISKDILTGKK
>ID26653-ABP_both
KILGVSKRILTGKK
>ID26654-ABP_both
KILILIKRK
>ID26655-NO
KILKFLFKKVF
>ID26658-ABP_both
KILRGVSKKIMRRILTGKK
>ID26659-ABP_pos
KILRGVSKKIMRRISKDILTGKK
>ID26660-ABP_both
KILRGVSKKIMRTFLRR
>ID26661-ABP_both
KILRGVSKRILTGKK
>ID26683-ABP_both
KISKKIMRTFLRR
>ID26684-ABP_both
KISKKIMRTFLRRISKDILTGKK
>ID26688-NO

KITRCPMIPCYISSPDE
>ID26690-ABP_both
KIVKVITVKSISPASLVPVF
>ID26698-ABP_both
KKAAACAAAAACAAWAACAAAKKKK
>ID26701-ABP_both
KKAAAHAAAAAHAAWAAHAAAKKKK
>ID26716-ABP_both
KKAAHVGKHVGKAAL
>ID26718-NO
KKAAQIRSQVMTHLRVI
>ID26720-ABP_both
KKAGKIAKKAGKIA
>ID26723-ABP_both
KKAKRRLLKK
>ID26724-ABP_both
KKALLAHALAHLALLALHLALHLKKA
>ID26725-ABP_both
KKALLAHALHHLALLAHHLAHALKKA
>ID26728-ABP_both
KKALLHLALALLALHAHALALHLKKA
>ID26737-ABP_both
KKCKGYRCRPVGFSSPISRRINDSENIYLPFGV
>ID26739-NO
KKCLTKYSAGLQTSQTCPAGQKICFKKW
>ID26740-NO
KKCRERGGQCHSGVCSWNEKFIGFCSFARPCC
>ID26741-ABP_both
KKCRNKKMEVSR
>ID26746-NO
KKDGYLVNKYTGCKVNCYKLGENKFCNRE
>ID26747-NO
KKDGYPIQENGCKY
>ID26750-ABP_both
KKDQLGKNEEGAPQEGILEDMPVDPDNEAYEMPSEEGYQDYEPEA
>ID26752-NO
KKEKDIMKKTI
>ID26755-NO
KKFFRAWWARRFLK
>ID26757-ABP_both
KKFGGGKTERARKAMAVE
>ID26759-NO
KKFKKFKVLKIL
>ID26760-ABP_both
KKFLHSAKKWLKAFVKLFKNW
>ID26762-ABP_both
KKFWEDDG
>ID26765-ABP_both
KKGKKGKKG
>ID26766-ABP_pos
KKHKWLWK

>ID26767-ABP_pos
KKHRCRVYNNGLPTGLYRWC
>ID26770-NO
KKICTRKPRFMSAWAQ
>ID26773-ABP_both
KKIGAIKKWGAIKKIGAI
>ID26777-ABP_both
KKIMRTFLRR
>ID26779-ABP_both
KKIMRTFLRRISKKILTGKK
>ID26780-ABP_neg
KKIRPRPPRLPRPRPRPLPYPRP
>ID26782-ABP_both
KKISQRYQKFALPQYLKTVYQHQK
>ID26785-ABP_neg
KKKAAFAAWAAFAAKKK
>ID26786-ABP_neg
KKKALFALWLAFLAKKK
>ID26787-NO
KKKCIAKDYGRCKWGGTPCCRGRGCICSIMGTNCECKPRLIMEGLGLA
>ID26790-NO
KKKEERADLIAYLKKA
>ID26795-ABP_both
KKKIIIIIKKK
>ID26798-ABP_both
KKKKGIGKFLHSAKKFGKAFVGEIMNS
>ID26805-NO
KKKKKKGGFLGFWRGENGRKTRSAYERMCILKGK
>ID26807-ABP_both
KKKKKKKK
>ID26809-ABP_both
KKKKKKKKKKKK
>ID26811-ABP_both
KKKKKKKKKKKKKK
>ID26831-ABP_both
KKKKWWWW
>ID26834-ABP_both
KKKLFKKL
>ID26839-ABP_both
KKKLLCCLLLLKKLKK
>ID26844-ABP_both
KKKLLLLLKKK
>ID26845-ABP_both
KKKPRPPYLPRPRPP
>ID26851-ABP_pos
KKKVVFKVKFK
>ID26852-ABP_pos
KKKVVFKVKFKK
>ID26853-ABP_pos
KKKVVFKVKFKKK
>ID26862-ABP_both

KKLAGLAKKWAGLAKKLAGLA
>ID26864-ABP_both
KKLFKKALKFL
>ID26866-ABP_both
KKLFKKIKKFL
>ID26867-NO
KKLFKKILKAL
>ID26868-NO
KKLFKKILKDL
>ID26869-NO
KKLFKKILKEL
>ID26913-ABP_both
KKLGEPSCNKLK
>ID26914-ABP_both
KKLIKKILKIL
>ID26918-ABP_both
KKLIKVWAKGFKKAKKLFKGIG
>ID26919-ABP_both
KKLKCLLCLLLLKKLKK
>ID26929-ABP_both
KKLKLCCLLLLKKLKK
>ID26930-ABP_both
KKLKLCLCLLLLKKLKK
>ID26937-ABP_both
KKLLKCLKCL
>ID26941-ABP_both
KKLLKLKLKLKK
>ID26944-ABP_both
KKLLLLCLLKCKKLKLK
>ID26945-ABP_both
KKLLLLLKK
>ID26946-ABP_both
KKLWLW
>ID26951-NO
KKNGYAVDSSGKVAE
>ID26956-NO
KKPGKKTTTKPTKK
>ID26957-NO
KKPGKKTTTKPTKKPTIKTTKK
>ID26958-ABP_both
KKPKKPWKPKK
>ID26961-ABP_both
KKPKLPWLPLK
>ID26963-NO
KKPPFNGSIF
>ID26964-ABP_both
KKPSKKPKPQAMTFPKVTVEYFPASFSTAALTVPED
>ID26965-NO
KKPTIKTTKK
>ID26967-ABP_both
KKPWWKPWWPKWKK

>ID26969-NO
KKPYILKKPYIL
>ID26976-NO
KKRKAPKKKRKFA
>ID26977-ABP_both
KKRLKKIFKKPMVIGVTIPF
>ID26982-ABP_both
KKRWWWWWR
>ID26984-ABP_both
KKSAGWVIPIGTLVK
>ID26991-NO
KKTTTKPTKK
>ID26993-ABP_both
KKTWWKTWWTKWKK
>ID26994-ABP_both
KKTWWKTWWTKWSQP
>ID26995-ABP_both
KKTWWKTWWTKWSQPKK
>ID27003-ABP_both
KKVIIEYVKIEA
>ID27004-ABP_both
KKVLFKLKFK
>ID27005-ABP_both
KKVLKAAA
>ID27029-ABP_both
KKWKKWKKWKK
>ID27030-NO
KKWKMRRGAGRRRRRRRR
>ID27031-NO
KKWKMRRNQFWIKIQR
>ID27033-ABP_both
KKWMQKVIDRFGG
>ID27037-ABP_both
KKWQWRMKKLG
>ID27039-ABP_both
KKWRKLLKKLKKLL
>ID27040-ABP_both
KKWRKLLKWLAKK
>ID27043-ABP_both
KKWRWWLKALAKK
>ID27044-ABP_both
KKWRWWLKALAKKLL
>ID27050-ABP_both
KKWWWKWKKW
>ID27058-NO
KKYAFEVVG
>ID27062-NO
KKYMVPQL
>ID27063-NO
KKYNVPQ
>ID27064-NO

KKYNVPQL
>ID27065-NO
KKYRRFRWKFKGKFWFW
>ID27067-ABP_both
KKYRYHLKPFSKK
>ID27068-NO
KLAAALLKKWKKLAAALL
>ID27069-ABP_both
KLAGKLAKWAGKLAKLAGKLA
>ID27071-ABP_both
KLAGLKAKWAGLKAKLAGLKA
>ID27072-ABP_both
KLAKGLAKWAKGLAKLAKGLA
>ID27075-NO
KLALKALKAALKLA
>ID27076-NO
KLALKLALKALKAA
>ID27077-NO
KLALKLALKALKAALK
>ID27079-NO
KLALKLALKALQAALQLA
>ID27080-ABP_both
KLALKLALKAWKLALKAA
>ID27081-NO
KLALKLALKWAKLALKAA
>ID27082-NO
KLALQLALQALQAALQLA
>ID27083-ABP_pos
KLAMSMSFNFVFYFMVNFDD
>ID27096-ABP_both
KLCKLLCLKLLKKKK
>ID27099-ABP_both
KLFAVIKKVAAVIGGL
>ID27107-ABP_pos
KLGHHGRAPP
>ID27112-NO
KLGVALKRK
>ID27113-NO
KLGVM
>ID27114-NO
KLGVPLARK
>ID27115-NO
KLGVPLKAK
>ID27116-NO
KLGVPLKRA
>ID27118-ABP_both
KLIFILSKTIPAGKNLFYKI
>ID27119-ABP_both
KLIFILSKTIPAIKNLFYKI
>ID27120-NO
KLIKGRTPIKFGK

>ID27122-NO
KLIKGRTPIKFGKADCDRPPKHSQNGK
>ID27123-NO
KLIKGRTPIKFGKADCDRPPKHSQNGM
>ID27125-NO
KLIKGRTPIKFGKARCRRPPKHSGK
>ID27128-ABP_both
KLILILSKTIPAIKNLFYKI
>ID27130-ABP_both
KLIPILSKTIPAIKNLFYKI
>ID27133-ABP_both
KLKAGLAKWKAGLAKLKAGLA
>ID27136-ABP_both
KLKFPKLKFP
>ID27137-NO
KLKFV
>ID27138-ABP_both
KLKHVGLHVGKLK
>ID27148-ABP_both
KLKKKLKKLLKK
>ID27149-ABP_both
KLKKLCCLLLLKKLKK
>ID27150-ABP_both
KLKKLCCLLLLKKLKKL
>ID27154-ABP_both
KLKKLKKLCCLKL
>ID27155-ABP_both
KLKKLLAGQKDGLLGQIAAMSDLYTKK
>ID27159-ABP_both
KLKKLWKKLLK
>ID27161-ABP_both
KLKLCKKLKLLKLKLCLK
>ID27165-ABP_both
KLKLLLKLK
>ID27166-ABP_both
KLKLLLLKLK
>ID27167-ABP_both
KLKLVALHKQVL
>ID27169-ABP_both
KLKRKAKNAVA
>ID27174-NO
KLLAKAAKKWLLLALKAA
>ID27184-NO
KLLKLLKKLLKLLK
>ID27189-ABP_both
KLLKRIKKLL
>ID27191-ABP_both
KLLKVIKKLL
>ID27192-ABP_both
KLLKWWKKLL
>ID27193-ABP_both

KLLLKCLKLLLKKKCLK
>ID27196-ABP_both
KLLLKLKLKLLKGWKRKRFG
>ID27199-ABP_both
KLLLLLK
>ID27201-ABP_both
KLLLLLLCKKKKC
>ID27202-ABP_neg
KLLLPKLKGLLFKL
>ID27203-ABP_both
KLLNLLPGLLAGIF
>ID27208-ABP_both
KLLSHSL
>ID27209-ABP_both
KLLSHSLL
>ID27210-ABP_both
KLLSKAQEKFGKNKSRSFQL
>ID27211-ABP_both
KLNRLKEKNKAKNSKENN
>ID27214-NO
KLPAGTLF
>ID27215-NO
KLPCRSNTFLNIFRRKKPG
>ID27216-ABP_both
KLPKLPWKPKK
>ID27220-ABP_both
KLPLLPWLPLK
>ID27221-NO
KLPTGYQFGL
>ID27222-NO
KLPVM
>ID27223-NO
KLPVT
>ID27225-NO
KLPYSFGL
>ID27229-ABP_both
KLRRLIRLL
>ID27230-ABP_pos
KLRRWIRWL
>ID27231-ABP_both
KLRRWVRWI
>ID27232-ABP_both
KLRRWVRWL
>ID27234-NO
KLSYDDKVFENVEFTPRL
>ID27235-NO
KLTFIQSTAAGDLYYNTNTHKYVYQQTQNAFGAAANTIVNGWMGGAAGGF
>ID27238-NO
KLTRAQRRAAARKNKRNTRGC
>ID27248-ABP_both
KLVKRFKKFFRKLKKS

>ID27249-ABP_both
KLVKTQLKVLK
>ID27250-ABP_both
KLVKTWLKVLK
>ID27251-ABP_both
KLVKWIIKTVNKFTKK
>ID27260-NO
KLWCAMS
>ID27267-ABP_both
KLWKLWKKWLK
>ID27268-ABP_both
KLWKLWLKWLK
>ID27271-ABP_both
KLWLLWLKWLL
>ID27272-NO
KLWMRWYSATTRRYG
>ID27273-NO
KLWMRWYSPTTRRYG
>ID27274-NO
KLWMRWYSPWTRRYG
>ID27276-NO
KLWSAWPSLWSSLWKP
>ID27287-NO
KMDCRWRWKCCKK
>ID27288-NO
KMDCRWRWKCKK
>ID27290-NO
KMDCRWRWKKK
>ID27292-NO
KMDCRWRWKSSKK
>ID27293-NO
KMDRWRWKKK
>ID27299-NO
KMGPKVW
>ID27302-NO
KMHGEKFLGMR
>ID27303-NO
KMIFVGIKKK
>ID27304-NO
KMIFVGIKKKEERA
>ID27306-NO
KMKELEGKLKWKT
>ID27310-NO
KMTRAQRRAAARRNRWTARGC
>ID27312-NO
KMYDFGL
>ID27315-NO
KNAWKHSSCHHRHQI
>ID27316-NO
KNCFFNEDFLWCVYATER
>ID27318-NO

KNDFCQLLYVWLVMFCQQCFVQFPKNVET
>ID27320-NO
KNECLWTDMLSNFGYPGYQSKHYACIRQKG
>ID27321-NO
KNEFIRF
>ID27325-NO
KNFFWKTFTSC
>ID27353-ABP_both
KNKRKRRRRRRGGRRRR
>ID27360-NO
KNNKFEFIRF
>ID27361-ABP_both
KNNRPVYIPKPRPPHPRL
>ID27364-NO
KNPYIL
>ID27380-NO
KPCCSIHDNSCCGI
>ID27388-NO
KPHLRGRF
>ID27390-ABP_both
KPKDMTSSQWFKTQHVQPSPQASNSAMSIINKYTERSKDLNTFLH
>ID27392-ABP_both
KPKGMTSSQWFKIQHMQPSPQACNSAMKNINKHTKRCKDLNTFLH
>ID27396-NO
KPKPHQFFGLM
>ID27397-NO
KPKTEKK
>ID27401-NO
KPMRLRW
>ID27403-NO
KPNFIRF
>ID27405-NO
KPNPERFYAPM
>ID27406-NO
KPNPERFYGLM
>ID27408-NO
KPNQDFMRF
>ID27409-NO
KPPAWVP
>ID27410-NO
KPPETV
>ID27411-NO
KPPFNGSIF
>ID27412-NO
KPPGLW
>ID27413-NO
KPPPWVPV
>ID27414-ABP_both
KPPQFTWAQWFETQHINMTSQQSTNAMQVINNYQRRSKNQNTFLL
>ID27415-NO
KPPSLWS

>ID27416-NO
KPPSSSEFIGLM
>ID27417-NO
KPPSSSEFVGLM
>ID27419-NO
KPQRLRF
>ID27420-NO
KPQRLRW
>ID27424-NO
KPRPDQFYGLM
>ID27427-NO
KPRPHQFIGLM
>ID27430-NO
KPRSKNPPKKPK
>ID27431-NO
KPSFVRF
>ID27432-NO
KPSPDRFYGLM
>ID27435-NO
KPVAL
>ID27441-ABP_both
KPYLKRFQVYYKGRMWC
>ID27443-NO
KQATCSIPYEYSNGKFKRTLYYSNGVYANS
>ID27448-NO
KQCSKP
>ID27454-NO
KQIKMKLREMVLFT
>ID27457-ABP_both
KQKLAKLKAKLQKLKQKLAKL
>ID27458-ABP_both
KQKWLWLW
>ID27466-ABP_both
KQRWLWLW
>ID27467-ABP_neg
KQSWQRMRKLALKR
>ID27478-ABP_pos
KRAKHRCRVYNNGLPTGLYRWC
>ID27479-NO
KRARNTEAARRSRARKLQRMKQGC
>ID27480-NO
KRASCQNPLFSNFFVCGLSE
>ID27484-NO
KRCFSNVEGYCRKKCRLVEISEMGCLHGKYCCVNELENKKHKKHSVVEET
>ID27485-NO
KRCHLTIDKATACSLSDCRLSCYSGYNGVGKCFDDPKVAGPSNCGCIYNC
>ID27486-ABP_both
KRCKNKMEGDDVAVSGRGARKAAKK
>ID27489-NO
KRCSSSLCA
>ID27495-ABP_both

KREDFLDQIIRDFRNFIYQKYRRLRDEFRKLRDILSG
>ID27499-ABP_both
KRFHSVGSLIQRLQQMIRDLSEATRHGIRKITRPKLLLAS
>ID27501-ABP_both
KRFIKWYKAWNKKWRKY
>ID27502-ABP_both
KRFIKWYNAWNEKRRVY
>ID27503-ABP_both
KRFKKFFKKLKKFVKKGFKKFAKV
>ID27504-ABP_both
KRFKKFFKKLKNSVKKRAKKFFKK
>ID27505-ABP_both
KRFKKFFKKVKKSV
>ID27506-ABP_both
KRFKKFFRKLKKSVKKRAKEFFKKPRVIGVSIPF
>ID27507-ABP_both
KRFKKFFRKLKKSVKKRKKEFKKKPRVIGVSIPF
>ID27509-ABP_both
KRFKKWYKAWRKKWRKY
>ID27512-ABP_both
KRFQNFFRELEKKFREFFRVYRITIGATIRF
>ID27518-NO
KRGFLDVIKHVGKAALSVVSHLINEGEH
>ID27519-NO
KRGFLDVIKHVGKAVGKAALNAVNDMVNKPEQQS
>ID27520-NO
KRGFLDVITHVGKAVGKAALNAVNEMVNQGEQ
>ID27532-NO
KRHHGYKRKFHEKHHSHR
>ID27539-NO
KRIHPRLTRSIR
>ID27540-NO
KRIIQRILSRNS
>ID27544-NO
KRIPNKKPGKKT
>ID27545-NO
KRIPNKKPGKKTTTKPTKK
>ID27546-NO
KRIPNKKPGKKTTTKPTKKPTIK
>ID27548-NO
KRIPNKKPGKKTTTKPTKKPTIKTTKKDLK
>ID27549-NO
KRIPNKKPGKKTTTKPTKKPTIKTTKKDLKPQTTKPK
>ID27550-NO
KRIPNKKPKK
>ID27552-ABP_both
KRIRKRIKKWLR
>ID27554-ABP_both
KRIVKLIKKWLR
>ID27555-ABP_both
KRIVKLILKWLR

>ID27557-ABP_both
KRIVKRIKKWLR
>ID27564-ABP_both
KRIVQRIKKWLR
>ID27568-ABP_pos
KRKAHRCRVYNNGLPTGLYRWC
>ID27571-ABP_both
KRKGKRK
>ID27572-ABP_both
KRKGSGKRK
>ID27573-ABP_both
KRKIFLRTKILV
>ID27575-ABP_both
KRKIL
>ID27576-ABP_both
KRKILI
>ID27578-ABP_both
KRKILIL
>ID27579-ABP_both
KRKILILI
>ID27580-ABP_both
KRKILILIGSG
>ID27581-ABP_both
KRKILILIK
>ID27582-ABP_both
KRKILILIKR
>ID27583-ABP_both
KRKILILIKRK
>ID27584-ABP_both
KRKILILIKRKR
>ID27586-ABP_both
KRKILILILIKRK
>ID27587-ABP_both
KRKILILILIL
>ID27589-ABP_pos
KRKKARCRVYNNGLPTGLYRWC
>ID27591-ABP_pos
KRKKHACRVYNNGLPTGLYRWC
>ID27592-ABP_pos
KRKKHRARVYNNGLPTGLYRWC
>ID27593-ABP_pos
KRKKHRCAVYNNGLPTGLYRWC
>ID27594-ABP_pos
KRKKHRCRAYNNGLPTGLYRWC
>ID27595-ABP_pos
KRKKHRCRVANNGLPTGLYRWC
>ID27596-ABP_pos
KRKKHRCRVFNNGLPTGLYRWC
>ID27597-ABP_pos
KRKKHRCRVSNNGLPTGLYRWC
>ID27623-ABP_both

KRKKRK
>ID27625-ABP_both
KRKKWFW
>ID27626-ABP_both
KRKLIKRK
>ID27627-ABP_both
KRKLILIKRK
>ID27628-ABP_both
KRKLILKRILIKRK
>ID27634-ABP_both
KRKRKILILIKRK
>ID27636-ABP_both
KRKSGSGSKRK
>ID27656-NO
KRPAAIKKAGQAKKKK
>ID27669-NO
KRRIRRERNKMAAAKSRNRRRELTDTGC
>ID27670-ABP_both
KRRKWHSVVLI
>ID27673-NO
KRRQRRR
>ID27676-ABP_both
KRRRRR
>ID27677-ABP_both
KRRRRRR
>ID27682-ABP_neg
KRSKRKRRIHQRVRIS
>ID27688-NO
KRVIQY
>ID27689-NO
KRVSRNKSEKKRR
>ID27697-ABP_both
KRWRWRW
>ID27709-NO
KSAYMRF
>ID27710-NO
KSAYMRFG
>ID27713-NO
KSCCKNTTGRNIYNTCRFAGGSRERCAKLSGCKIISAST
>ID27718-NO
KSCCPNTTGRNIYNTCRLGGGSRERCASLSGCKIISAST
>ID27727-NO
KSDLLGALLSRNSPSSYGLPSRDMSTAY
>ID27731-ABP_neg
KSGGGGWGSGGGGGGGGWWWSGWGVDR
>ID27733-NO
KSHAHAQKRIRRRLIILL
>ID27734-NO
KSICKTIPSNKPKKK
>ID27743-ABP_both
KSKWLWLW

>ID27746-NO
KSLSRHDHIHHH
>ID27747-ABP_both
KSLWLW
>ID27749-NO
KSMNPTGRRCPDPNGVEKKSMCYSSCKTQGFMGGSCQGHKGNYMCECYEG
>ID27756-ABP_both
KSRQWQWKIRRTWPIFSIRR
>ID27758-NO
KSSDVKYYLAKCTTDVQ
>ID27763-NO
KSVRGKGKGQKRKRKKSRYK
>ID27770-NO
KTCKLFRGECPVDPCEPEKCDECCKATFGKQICGKCEQESTELHCHCRR
>ID27771-ABP_both
KTCMTKKEGWGRCLIDTTCAHSCRKQGYKGGNCKGMRRTCYCLLDC
>ID27773-NO
KTCQRRWDFCPGSLVGVITCCGGLICFLFFCV
>ID27783-NO
KTIEAHPPYYAS
>ID27785-NO
KTIPSNKPKKK
>ID27788-NO
KTKTCTVLY
>ID27791-ABP_both
KTLLKKVLKAAA
>ID27792-ABP_both
KTLLSAVGS
>ID27798-ABP_both
KTRNWFSEHFKKVKEKLKDTFA
>ID27801-ABP_both
KTSPDVE
>ID27802-ABP_both
KTSPDVEYHK
>ID27811-NO
KTVLLRKLLKLLVRKI
>ID27812-ABP_both
KTVPVVLKASIKVSSAGFGF
>ID27815-ABP_pos
KTVYQHQKAMKPWIQPKTKVIPYVRYL
>ID27817-NO
KTYYGTNGVHCTKKSLWGKVRLKNVIPGTLCRKQSLPIKQDLKILLGWAT
>ID27821-NO
KVAGPK
>ID27822-ABP_both
KVALGVAQNYLNPQQ
>ID27823-ABP_both
KVANTVQKLKRKAKNAV
>ID27825-ABP_both
KVASVIGGL
>ID27826-ABP_neg

KVDKGSYLPRP
>ID27827-NO
KVEWICEDCSNIFR
>ID27830-ABP_both
KVFIATLVVSSFLLAKPPRV
>ID27831-ABP_both
KVGKGSYPCSFVKVVAKVSV
>ID27833-NO
KVGTKCCAKP
>ID27838-ABP_both
KVISMI
>ID27840-NO
KVKFSAWG
>ID27844-ABP_both
KVKYFKRWLR
>ID27846-NO
KVLAGM
>ID27848-NO
KVLGSVAKHLLPHVAPIIAEKL
>ID27849-NO
KVLILA
>ID27850-ABP_both
KVLKAAA
>ID27855-NO
KVLPLP
>ID27856-NO
KVLPVP
>ID27857-NO
KVLPVPE
>ID27858-NO
KVLPVPQ
>ID27859-NO
KVLPVQ
>ID27864-ABP_both
KVLSKVHTLLKAVLAL
>ID27866-ABP_both
KVNVNAIKKGGKAIGKGFKVISAASTAH
>ID27868-NO
KVPAYCKLPPDSGPCKGHFPAFYYDPVSSYCQKFI
>ID27879-NO
KVREEY
>ID27880-NO
KVREGGY
>ID27881-NO
KVREGT
>ID27889-ABP_pos
KVVFKVKFK
>ID27891-NO
KVVHLRPRSSFSSEDEYQIYLRNVSKYIQLYGRPRF
>ID27904-NO
KVYCCLGVRDDWCCAGQIQI

>ID27905-ABP_both
KVYLPRYKVYSTAAGRYQLL
>ID27908-ABP_both
KVYVVKIAVPCFPKSARSVS
>ID27909-NO
KWAAGRSAW
>ID27910-ABP_both
KWAFRVAYRGIAYRRAR
>ID27911-ABP_both
KWAFRVCYRGICYRRAR
>ID27912-ABP_both
KWAFRVCYRGICYRRSR
>ID27915-ABP_pos
KWARWIRWL
>ID27918-ABP_both
KWCARVCARGACYRRCR
>ID27919-ABP_both
KWCARVCYRGICYRRCR
>ID27920-ABP_both
KWCFAVCYRGICYRRCR
>ID27922-ABP_both
KWCFRRCYAGICYRRCR
>ID27923-ABP_both
KWCFRVAYRGIAYRRCR
>ID27924-ABP_both
KWCFRVAYRGISYRRCR
>ID27939-ABP_both
KWCRKWQWRGVKFIKCV
>ID27941-NO
KWCVIWSKEGCLF
>ID27942-ABP_pos
KWCVYAYVKVKGVLVKYKKCW
>ID27943-NO
KWDSFIKKLTSKFLHSAKKF
>ID27944-NO
KWFETWFTEWPKKRK
>ID27945-ABP_both
KWFFFH
>ID27946-ABP_both
KWFFHH
>ID27947-ABP_both
KWFFKH
>ID27948-ABP_both
KWFFLH
>ID27949-ABP_both
KWFFSH
>ID27950-ABP_both
KWFFWH
>ID27951-ABP_both
KWFHWK
>ID27952-ABP_both

KWFKKL
>ID27953-ABP_both
KWFKRLEKLFSKI
>ID27955-ABP_both
KWFLWH
>ID27956-NO
KWFNEKSIQNKIDEKIGKNFLGGMAKAVVHKLAKNEFMCVANVDMTKSCD
>ID27957-ABP_both
KWFWWK
>ID27959-ABP_both
KWICRRLWYRGRWITYCRWR
>ID27964-NO
KWKAFIKKLTKKFLKSAKKF
>ID27965-ABP_both
KWKAFKKIEKMIRNIRNKIVK
>ID27967-ABP_both
KWKIFKKIEKAGRNIRDGIIKAGPAVSVVGEAATIYKTG
>ID27969-ABP_both
KWKIFKKIEKVGRNIRNGIIKAVAVLGEAKAL
>ID27976-ABP_both
KWKKKKKKPKFL
>ID27978-ABP_both
KWKLFKGIRAVLKVL
>ID27982-ABP_both
KWKLFKKIFKCWRWQWRWKKLGA
>ID27984-ABP_both
KWKLFKKIFKRIVQRIKDFLRN
>ID27995-ABP_both
KWKLFKKIGIGKFL
>ID28000-ABP_both
KWKLFKKIGIGKFLKKK
>ID28007-ABP_both
KWKLFKKIRGGRLCYCRRRFCVCV
>ID28009-ABP_neg
KWKLFKKLPKFHLHLAKKF
>ID28010-NO
KWKLFKKVLKVL
>ID28012-ABP_both
KWKLFLGILAVLKVL
>ID28015-ABP_both
KWKLKKHIGIGKHFLSAKKF
>ID28018-NO
KWKLLLLLLKWK
>ID28019-ABP_both
KWKLWKKIEKWGQGIGAVLKWLTTWL
>ID28026-NO
KWKSFIKKLTSKFLHSAKKL
>ID28027-NO
KWKSFIKKLTSKFLHSAKKM
>ID28057-ABP_both
KWKVFKKAEKMIRNIRNKIVK

>ID28059-ABP_both
KWKVFKKIEKMARNIRNKIVK
>ID28060-ABP_both
KWKVFKKIEKMGRNIRNGIVK
>ID28062-ABP_neg
KWKVFKKIEKMGRNIRNGIVKAGPKWKVFKKIEK
>ID28066-ABP_both
KWKVLKKKIMKMLRNRINGLVKAGPALKVKLQALAL
>ID28075-ABP_both
KWLKKWLKWLKK
>ID28084-ABP_both
KWLRRPWRRWR
>ID28086-ABP_pos
KWLRWIRWL
>ID28087-ABP_both
KWLRWVRRRWW
>ID28097-ABP_pos
KWRAWIRWL
>ID28100-ABP_pos
KWRLWIRWL
>ID28101-ABP_both
KWRMCVDYTGFNKVCSKVFYFLFRID
>ID28102-ABP_both
KWRQWQSKWRRTNPWFWIRR
>ID28104-ABP_pos
KWRRAIRWL
>ID28105-ABP_both
KWRRAVRAI
>ID28106-NO
KWRRKLKKLR
>ID28107-NO
KWRRKLKKLRPKKKRKV
>ID28108-ABP_pos
KWRRLIRWL
>ID28110-ABP_pos
KWRRW
>ID28111-ABP_pos
KWRRWARWL
>ID28112-ABP_pos
KWRRWIAWL
>ID28113-ABP_pos
KWRRWILWL
>ID28114-ABP_pos
KWRRWIR
>ID28115-ABP_pos
KWRRWIRAL
>ID28116-ABP_pos
KWRRWIRLL
>ID28117-ABP_pos
KWRRWIRW
>ID28119-ABP_both

KWRRWIRWL
>ID28120-ABP_both
KWRRWIRWW
>ID28122-ABP_pos
KWRRWLRWL
>ID28124-ABP_both
KWRRWVRWL
>ID28139-ABP_neg
KWWCFRVCYRGICYRRCRWK
>ID28140-ABP_both
KWWFWK
>ID28141-ABP_both
KWWKIWRWR
>ID28144-ABP_both
KWWKKWWKWWKKWWKK
>ID28146-ABP_both
KWWKWWKKWWKK
>ID28151-ABP_both
KWWRWRRWW
>ID28153-ABP_both
KWWVFRVVYPRIVYRRVRWK
>ID28154-ABP_neg
KWWVFRVVYRGIVYRRVRWK
>ID28206-ABP_both
KYGPALVIAVKKSCSLTFRA
>ID28207-NO
KYHSGPSDKSKVIS
>ID28208-NO
KYHSGPSDKSKVISGKCEWQGGQLNCIAT
>ID28218-NO
KYLNFAKWLKGANLAKYANA
>ID28219-NO
KYNPCANYL
>ID28220-NO
KYNPCASYL
>ID28221-NO
KYNPCLGFL
>ID28222-NO
KYNPCSNYL
>ID28225-NO
KYPVEPFTESQSLTLTDVENLHL
>ID28226-NO
KYPVQPFTESQSLTL
>ID28241-ABP_pos
KYYGNGVHCTKSGCSVNWGEAFSAGVHRLA
>ID28254-NO
KYYPCFGYF
>ID28264-NO
LACVTNEGPKWANTYCAAVCHMSGRGAGSCNAKDECVCSMT
>ID28265-NO
LADDMPATMADQEVYRPEPEQIDSRNKYFSPRL

>ID28266-NO
LADDMPATPADQEMYRPDPEQIDSRTKYFSPRL
>ID28267-NO
LADDTPATPADQEMYRPDPEQIDSRTKYFSPRL
>ID28272-NO
LAGYTGIASGTAKKVVDAIDKGAAAFVIISIISTVISAGALGAVSASADF
>ID28274-ABP_both
LAHRYH
>ID28275-NO
LAIILRRRIRKQAHAHSK
>ID28276-NO
LAIPVNKP
>ID28278-NO
LAKRADICQPGKTSQRACET
>ID28280-NO
LALERRDGWLRLFGLKPRRKH
>ID28284-NO
LALPP
>ID28285-NO
LALRDECCASPPCRLNNPYVCH
>ID28286-ABP_both
LALRTAGWLRLLGFRDKKKN
>ID28293-NO
LANVST
>ID28296-NO
LAQLLFIHFRIGRRRRRRRR
>ID28308-NO
LASLKSDTVTPLR
>ID28311-ABP_pos
LATLLFTMCLNFCGVLAGDD
>ID28316-NO
LAVRGCCSHPACAGNNPHICGRRR
>ID28317-NO
LAVRGGCCSHPVCNLNNPQMCRGRR
>ID28319-NO
LAYFYP
>ID28320-NO
LAYFYPEL
>ID28321-NO
LCCVTEDWCCEWW
>ID28330-NO
LCEKFKVQRLVELNCVD
>ID28332-NO
LCHNSISCALGGDNVCNNVCVRQGNDNGGRCLPRDGCPGYDICACYPRS
>ID28334-ABP_both
LCKKKLKCLKKLLLLLK
>ID28335-NO
LCKRESETWSGRCVNDYQCRDHCINNDRGNDGYCAGGYPWYRSCFCFFSC
>ID28337-NO
LCLDQKPEMEPFRKDAQQALEPSRQRRWLHRRCLSGRGFCRAICSIFEEP
>ID28338-NO

LCNGS
>ID28342-NO
LCPDYTEPCSHAHECCSWNCYNGHCTG
>ID28343-NO
LCPKHPLGC
>ID28344-NO
LCPLDVLQLSSELLDIDGNEVEASRILSDITAFGGIRCPLTVVQSRGIGT
>ID28346-NO
LCSREGEFCYKLRKCCAGFYCKAFVLHCYRN
>ID28349-NO
LDAQSAPLR
>ID28352-NO
LDCLSELCS
>ID28354-ABP_both
LDDLKGTFAALSELHCDKLHVDPENFKLLGNVLVVVLARNFGKEFTPVL
>ID28355-ABP_both
LDDLPGALSELSDLHAHKLRVDPVNFKLLSHSL
>ID28356-NO
LDEPNMDTISKSREYKCKIDLDCSNHIACRHCSYRNCKCDHGTCKCMP
>ID28362-NO
LDGYPLSKNN
>ID28363-NO
LDHFAPM
>ID28364-NO
LDIQK
>ID28365-NO
LDITPFLSLTLP
>ID28366-NO
LDKKECTANGEFCGISVFGSYLCCSGRCVFVCI
>ID28367-NO
LDKKECTTNGEFCGISVFASFLCCSGLCVFVCI
>ID28368-NO
LDKKTTS
>ID28369-NO
LDKRCIPHFDPCDPIRHTCCFGLCLLIACI
>ID28376-NO
LDLTPGSHVDSYVEA
>ID28379-NO
LDPCCREPPCASTHIDRC
>ID28380-NO
LDPCCREPPCASTHTDICT
>ID28388-NO
LDSLTGLGFGSQ
>ID28390-NO
LDTYSPELFCTIRNFYDADRPDRGAAA
>ID28435-NO
LEDPYKSDSNSRYIEVVVVNDNSMFRKYNR
>ID28439-NO
LEFNQ
>ID28440-NO
LEGEDDPDRSMKLSF

>ID28441-NO
LEGEDDPDRSMKLSFRA
>ID28443-ABP_both
LEGRSISRIDELIQQRQEKD
>ID28444-NO
LEGSFLGGSEAGERLLQQGLAQVEA
>ID28445-NO
LEIAKNGLSTTSNPKR
>ID28446-NO
LEIVPK
>ID28460-NO
LEKTG
>ID28470-NO
LELNQ
>ID28472-NO
LENLHLPLP
>ID28473-NO
LENPSPQAPA
>ID28475-NO
LENSSPQAPA
>ID28476-NO
LENSSPQAPARRLLPP
>ID28478-NO
LEPAYQRFL
>ID28489-NO
LETPAPQVPA
>ID28490-NO
LETPAPQVPARRLLPP
>ID28494-NO
LEYSI
>ID28496-NO
LFAQLGP
>ID28497-NO
LFDKPVSPL
>ID28504-ABP_pos
LFFFFF
>ID28509-ABP_both
LFHAGKMIH
>ID28510-ABP_pos
LFIFFF
>ID28511-NO
LFKCYKPDSRGFRVCE
>ID28519-NO
LFRHVVKIFEKYLGK
>ID28543-NO
LFSLVLAG
>ID28544-NO
LFVVTLVG
>ID28548-NO
LFYLVPGPGHG
>ID28552-ABP_both

LGDFFRKSKEKIGKEFKRIVQRIKDFLRNLAKRHHGYKRKFH
>ID28556-NO
LGFPTTKTYFPHF
>ID28558-ABP_both
LGGAATGVIGYISNQTCPTTACTRAC
>ID28561-ABP_both
LGGDNYGTFSGSNGNNFQHGSN
>ID28563-NO
LGISYGRKKRRQRRRPPQ
>ID28566-ABP_both
LGKEEQIGRASNSGRKAARKKK
>ID28578-NO
LGPAPTPEMREKLCGHHFVRALVRVCGGPRWSTEA
>ID28579-NO
LGPDIVSPPVCGNELLEVGEECDCGTPENCQNE
>ID28583-NO
LGQTPTK
>ID28586-NO
LGTDVRQ
>ID28587-NO
LGTNPLKSSGIE
>ID28588-NO
LGTQY
>ID28589-NO
LGTQYTDAPSFSDIPNPIGSENSEK
>ID28594-ABP_both
LGVVGSLASKVVPAVISKVK
>ID28596-NO
LGWGRRCPQCPRCPSCPSCPRCPRCPRCKCNPK
>ID28599-ABP_both
LGYLEQLLRL
>ID28600-ABP_both
LHAHKLRVDPVNFKLLSHSLLVTL
>ID28606-ABP_both
LHCIALRKK
>ID28608-NO
LHCPALVTYNTDTFESMPNPEGRYTFGASCV
>ID28611-NO
LHEPG
>ID28612-NO
LHGPYP
>ID28615-ABP_both
LHLIALRKK
>ID28616-ABP_neg
LHLLHHL
>ID28618-NO
LHLPAP
>ID28621-NO
LHLPLP
>ID28623-NO
LHLPLPLL

>ID28624-NO
LHLPLR
>ID28626-NO
LHLPYP
>ID28627-NO
LHLWLP
>ID28628-NO
LHLYLP
>ID28631-NO
LHREN
>ID28632-ABP_both
LHRIALRKK
>ID28633-ABP_both
LHRLVKLVAALLRGYASKVDTH
>ID28634-NO
LHSMKEG
>ID28636-ABP_neg
LHWLHHL
>ID28638-NO
LIAKTALPQTN
>ID28641-NO
LICFICFSPTAH
>ID28642-NO
LICFNDFSPTARTLEYCQIGITTYNPS
>ID28644-NO
LICYNDHGYTGKTTETCENGETTCYEKSR
>ID28646-NO
LIDHLGAPRWAVDTILGAIAVGNLASWVLALVPGPGWAVKAGLATAAAIV
>ID28656-NO
LIIFAIAASHKK
>ID28657-NO
LIIFAILISHKK
>ID28658-NO
LIIFRIAASHKK
>ID28659-NO
LIIFRILISH
>ID28660-NO
LIIFRILISHHH
>ID28661-NO
LIIFRILISHK
>ID28663-NO
LIIFRILISHR
>ID28666-ABP_both
LIKHILHRL
>ID28667-ABP_both
LIKHILHRLGGGFHFHLHF
>ID28668-ABP_neg
LIKKALAALAKLNI
>ID28671-ABP_both
LIKVVNHVQYNVTLHWHGIR
>ID28676-ABP_both

LILIKRK
>ID28678-ABP_both
LILKRKRKRKRILI
>ID28679-NO
LILSSGELLRHPRG
>ID28681-NO
LINSILGLPK
>ID28682-NO
LINSLLGIPKVMNDA
>ID28687-ABP_both
LIPFPFP
>ID28693-ABP_neg
LIRCSRTCLQYKTSRFMRW
>ID28694-NO
LIRLWSHLIHIWFQNRRLKWKKK
>ID28702-NO
LIWKL
>ID28711-ABP_both
LKAAAAAAKLAAKAAKAALKAAAAAAKL
>ID28714-NO
LKAYDFGL
>ID28719-ABP_both
LKCKCKLLKLLKLKKLK
>ID28720-ABP_both
LKCKKCLLLLLKKLKK
>ID28723-ABP_both
LKCLLLLKKKCKKKL
>ID28726-NO
LKCNKLIPLAYKTCPAGKDLCYKMYMVSDK
>ID28729-NO
LKCNKLVPLF
>ID28730-NO
LKCNKLVPLFYKTCP
>ID28731-NO
LKCNKLVPLFYKTCPAGKDLCYKMYMVATP
>ID28733-NO
LKCNKLVPLFYKTCPAGKNLCYKMFMVSNK
>ID28740-NO
LKCYSSRTETMTCPEGKDKCEKYAV
>ID28743-NO
LKDAQ
>ID28745-NO
LKDTQ
>ID28750-NO
LKELQ
>ID28751-ABP_both
LKFLKFG
>ID28753-ABP_both
LKGASKLIPHLLPSRQQ
>ID28754-ABP_both
LKGCWTKSIPPKPCF

>ID28755-ABP_both
LKGCWTKSIPPKPCFG
>ID28756-ABP_both
LKGCWTKSIPPKPCFGF
>ID28760-ABP_both
LKHGLLKRIKTLL
>ID28773-ABP_neg
LKKISQYYYQKFAWPQYL
>ID28781-ABP_both
LKKKKLCKCLLKLKLL
>ID28782-ABP_both
LKKKKLKLLLKLKCLC
>ID28783-ABP_both
LKKKKLLKCKLLLKCLKL
>ID28787-ABP_both
LKKKLKCKCLLLKKLL
>ID28788-ABP_both
LKKKLKCLCKLLKKLL
>ID28793-ABP_both
LKKLLCKLKLKCLKKKLL
>ID28795-ABP_both
LKKLLKKL
>ID28800-ABP_both
LKKLLKLCKKLCKLAG
>ID28803-ABP_both
LKKLLKLLKLLKL
>ID28804-ABP_pos
LKKLWKRLARLLKRFIRQLRRPVR
>ID28805-ABP_pos
LKKLWKRLARLWKRIIRQLKKPVR
>ID28806-ABP_pos
LKKLWKRVFRIWKRIFRYLKRPVR
>ID28807-ABP_pos
LKKLYKRLFKILKRILRYLRKPVR
>ID28808-ABP_pos
LKKLYKRLFKLWKRLYRYLKKPVR
>ID28809-ABP_pos
LKKLYKRVAKIWKRWIRYLKKPVR
>ID28815-ABP_pos
LKKVWKRVARLIKRWFRYLRRPVR
>ID28816-ABP_pos
LKKVWKRVFRILKRFLRYLKRPVR
>ID28817-ABP_pos
LKKVYKRLARLLKRYIRYLRRPVR
>ID28818-ABP_pos
LKKVYKRVARLIKRLFRYLKRPVR
>ID28819-NO
LKKYKVPQ
>ID28823-ABP_both
LKLCKKLCL
>ID28824-ABP_both

LKLCKKLLLCKLKKKLLK
>ID28828-ABP_both
LKLKAIAALAKKKW
>ID28834-ABP_both
LKLKKKCKCLLLKKL
>ID28835-ABP_both
LKLKKKCKCLLLKKLL
>ID28837-ABP_both
LKLKKKCLCKLLKKLL
>ID28838-ABP_both
LKLKKLCKCLLKKKLL
>ID28845-ABP_both
LKLLKKLKLKCKCLKLKL
>ID28848-ABP_both
LKLLLLKLLL
>ID28853-NO
LKNVGKEVGMDVVRTGIDIAGCKIKGEC
>ID28855-NO
LKPDM
>ID28857-NO
LKPMN
>ID28858-NO
LKPNM
>ID28859-NO
LKPTPEGN
>ID28863-ABP_both
LKRIKTLL
>ID28870-ABP_pos
LKRLWKRLFKILKRYYRYLRRPVR
>ID28871-ABP_both
LKRLYKRLAKLIKRLYRYLKKPVR
>ID28872-ABP_both
LKRLYKRVFRLLKRYYRQLRRPVR
>ID28875-ABP_both
LKRVWKRVFKLLKRYWRQLKKPVR
>ID28876-NO
LKSQQ
>ID28878-NO
LKTLATALTKLAKTLTTL
>ID28879-NO
LKTLTETLKELTKTLTEL
>ID28881-NO
LKVGVKQY
>ID28885-NO
LKVPDLPLPE
>ID28886-ABP_both
LKVVFKVLFK
>ID28888-ABP_both
LKWLKKL
>ID28889-ABP_both
LKWLKWG

>ID28892-ABP_both
LKWRKMFRW
>ID28905-NO
LLACLFGNGRCSSNRDCCELTPVCKRGSCVSSGPGLVGGILGGIL
>ID28906-NO
LLADTTHHRPWT
>ID28907-NO
LLAILRRRIRKQAHAHSK
>ID28908-ABP_both
LLAIVGNLLKSLL
>ID28914-ABP_both
LLCIALRKK
>ID28915-NO
LLCISCCVSITECCQLMSGCAVEIKS
>ID28916-ABP_both
LLCKALRKI
>ID28917-ABP_both
LLCKKCL
>ID28918-ABP_both
LLCKKLKLKKLCLKKL
>ID28920-ABP_both
LLCLKKKLKCKL
>ID28921-ABP_both
LLCLKKKLLKLCKK
>ID28923-ABP_both
LLDKLKRTLLSIEAVLI
>ID28926-NO
LLDYVTGACCAGLNFVCC
>ID28934-NO
LLETLLKPFQCRICMRNFSTRQARRNHRRRHRR
>ID28956-NO
LLGIPKVMNDA
>ID28958-NO
LLGKINLKALAALAKKIL
>ID28959-NO
LLGLVTGACCAVLKFSFCC
>ID28960-ABP_both
LLGMIKVAITAISALSKL
>ID28968-ABP_both
LLGPVLGLVSNVLGGLL
>ID28973-NO
LLIALRRRIRKQAHAHSK
>ID28974-NO
LLIIARRRIRKQAHAHSK
>ID28975-NO
LLIILARRIRKQAHAHSK
>ID28976-NO
LLIILRARIRKQAHAHSK
>ID28981-NO
LLIILRRRIRAQAHAHSK
>ID28982-NO

LLIILRRRIRKAAHAHSK
>ID28983-NO
LLIILRRRIRKQAAAHSK
>ID28984-NO
LLIILRRRIRKQAHAASK
>ID28985-NO
LLIILRRRIRKQAHAHAK
>ID28988-ABP_both
LLIRAAKKFIKK
>ID28992-ABP_both
LLKKALRLWKKVL
>ID28996-ABP_both
LLKKKKCLKLLCLKL
>ID28998-ABP_both
LLKKLKCLCKLKKKLL
>ID28999-ABP_both
LLKKLLKKLLKKC
>ID29001-ABP_both
LLKKLLKLCKKCLLLKK
>ID29003-NO
LLKKRKVVRLIKFLLK
>ID29004-ABP_both
LLKKVLKAAA
>ID29007-NO
LLKLLKLKKLKF
>ID29008-ABP_neg
LLKLLLPKLKGLLFKL
>ID29010-NO
LLKTTALLKTTALLKTTA
>ID29013-NO
LLLAHLL
>ID29018-ABP_both
LLLIALRKK
>ID29019-ABP_both
LLLIVLRKK
>ID29020-ABP_both
LLLKCKKLKLKKCLKLK
>ID29023-ABP_both
LLLKKLLKKCLKCKK
>ID29024-ABP_both
LLLKKLLKLKLCLKCKK
>ID29025-ABP_both
LLLKKLLLLCKKKKKCKL
>ID29028-ABP_pos
LLLLKILLLKKLKA
>ID29032-ABP_both
LLLLRRRR
>ID29034-NO
LLMLDNDLPP
>ID29036-ABP_neg
LLMRKLIKGYGYLFGKGKRKKR

>ID29038-NO
LLNPPHQIYP
>ID29040-ABP_both
LLNSGVKLGTKLLSGLLN
>ID29045-ABP_both
LLPIVANLLKSLL
>ID29047-NO
LLPIVDNLLYGLL
>ID29048-ABP_both
LLPIVGALLKSLL
>ID29052-ABP_both
LLPIVGNLLKSLLGAKRKRAG
>ID29053-ABP_both
LLPIVGNLLKSLLGWKRKAFG
>ID29056-ABP_both
LLPIVINLLKSLL
>ID29057-ABP_both
LLPIVVNLLKSLL
>ID29058-ABP_neg
LLPKLKGLLFKL
>ID29060-NO
LLPPY
>ID29068-NO
LLQSW
>ID29071-NO
LLRHVVKILEKYLGK
>ID29072-ABP_both
LLRIALRKK
>ID29076-ABP_both
LLRRVAGLLKQFAK
>ID29077-ABP_both
LLRRVARLLKRFAK
>ID29078-ABP_both
LLSHSL
>ID29079-NO
LLSKAQEKFGKNKSR
>ID29080-ABP_both
LLSNVAGLLKQFAK
>ID29085-NO
LLTSL
>ID29086-NO
LLTSLRGPAESSGESAHPLE
>ID29087-NO
LLTSLRGSAESPAALGEASAAHPLE
>ID29088-ABP_both
LLWIALRKK
>ID29093-NO
LLYQEP
>ID29094-NO
LLYQEPVLGPVRGPFPIIV
>ID29095-NO

LLYQQPV
>ID29099-ABP_both
LMEIHHKASQDKPKK
>ID29100-ABP_both
LMEIHHRASQDTPKE
>ID29103-NO
LMPVLCSRFKKC
>ID29104-NO
LMPVLCSSFKRC
>ID29112-ABP_both
LNALKKVFQGIHEAIKKI
>ID29113-ABP_both
LNALKKVFQGIHEAIKLI
>ID29115-ABP_both
LNALKKVFQKIHEAIKLI
>ID29120-ABP_both
LNENLLRF
>ID29121-NO
LNENLLRFFVAPFPEVFG
>ID29131-NO
LNHDIVAPMVFGSGNYF
>ID29135-NO
LNKKGDDCLAVKKNCGFPKLGGPCCSGLCFFVCA
>ID29137-NO
LNKRCIDGGEICDIFFPNCCSGWCIILVCA
>ID29138-NO
LNKRCLDGGEICGILFPSCCSGWCIVLVCA
>ID29139-NO
LNKRCLEFGEVCNFFFPTCCGYCVLLVCL
>ID29140-NO
LNKRCQEFGEVCNFFFPDCCGYCVLLLCI
>ID29142-NO
LNKRSCLPLDWFCGFNIIGAFLCCSGYCLVVCM
>ID29143-ABP_both
LNLKKILGKIGVMLSHLN
>ID29144-NO
LNNSRAP
>ID29148-NO
LNPFRWMINKYREWKNKKN
>ID29156-NO
LNQRDCLSKNAFCAWPILGPLCCSGWCLYVCM
>ID29157-NO
LNQSDCLPRDTFCALPQLGLLCCSGRCLLFCV
>ID29159-NO
LNSAGYLLGKALAALAKKIL
>ID29160-NO
LNSAGYLLGKINLKALAALAKKIL
>ID29161-NO
LNSAGYLLGKLKALAALAK
>ID29163-NO
LNSPAY

>ID29165-NO
LNTRDDDCEPPGNFCGMIKIGPPCCSGWCFFACA
>ID29166-NO
LNVPGE
>ID29168-NO
LNVPPSWFLSQR
>ID29171-NO
LNWGTPDWDVRNCVGVLWCP
>ID29174-NO
LPAFCQAIGWGDPITHWS
>ID29175-NO
LPAMPNS
>ID29176-ABP_both
LPAVFKLASKVFPAVYCKAS
>ID29177-ABP_both
LPAVFKLASKVVPSVFGLVK
>ID29178-NO
LPCCDVGWCSRRCICDVYRP
>ID29180-NO
LPCCYIGWCSRRCNCGGYRP
>ID29183-NO
LPEQDFMRF
>ID29186-NO
LPFEH
>ID29189-NO
LPGLTGSKGVRGISGLPGFSG
>ID29190-NO
LPGLTSSGDSDESLPFLNTICCWSGACCG
>ID29191-NO
LPGMMG
>ID29193-ABP_both
LPGSSKKFSVYKDQK
>ID29195-NO
LPHNHGG
>ID29197-NO
LPHPVLHMGPLR
>ID29199-NO
LPHYPRL
>ID29201-NO
LPIYQFGL
>ID29206-NO
LPLRF
>ID29207-NO
LPLRFHGRFRLRTHKKL
>ID29210-NO
LPMYNFGL
>ID29212-NO
LPNFCMDTSGRAGPLCMGSE
>ID29213-NO
LPNRYNFGL
>ID29215-NO

LPPCCSLNKKHCPAPACKYKPCCKS
>ID29216-NO
LPPCCSLNLRLCPAPACKYKPCCKS
>ID29217-NO
LPPCCTPPKKHCPAPACKYKPCCKS
>ID29219-NO
LPPCCTPPLRLCPAPACKYKPCCKS
>ID29223-NO
LPPGFTPWR
>ID29224-NO
LPPGPLPRP
>ID29228-NO
LPPPVH
>ID29229-NO
LPQNILP
>ID29230-NO
LPQNIPPL
>ID29231-NO
LPQNIPPLTQTPVVVPPFLQPEVMGVSK
>ID29232-NO
LPQRDMFLCRIGSCHFGRCPIHLVRVGSCFGFRSCCKSPWDV
>ID29234-NO
LPQYLKTVYQHQKA
>ID29235-NO
LPRDTSRCVGYHGYCIRSKVCPKPFAAFGTCSWRQKTCCVDTTSDFHTCQ
>ID29236-NO
LPRFSTMPFIYCNINEVCHY
>ID29238-NO
LPSAHPLE
>ID29240-NO
LPSDATLVLDQTGKELDAR
>ID29241-NO
LPSIGHYY
>ID29247-NO
LPVFSTLPFAYCNIHQVCH
>ID29248-ABP_both
LPVLFKLASKVFPAVFSSLK
>ID29249-ABP_both
LPVLFSSAIAKVGIKLGAKV
>ID29251-ABP_both
LPVVFKIASKVVPSVISKIT
>ID29252-ABP_both
LPVVFRVASKVFPALISKLT
>ID29253-NO
LPVYNF
>ID29254-NO
LPVYNFGI
>ID29255-NO
LPVYNFGL
>ID29256-NO
LPWKWPWWPWPP

>ID29261-NO
LPYPVNCKTECECVMCGLGIICKQCYYQQ
>ID29262-NO
LPYPY
>ID29263-NO
LPYPYY
>ID29265-NO
LQALLFIHFRIGRRRRRRRR
>ID29270-NO
LQCNKLVPIASKTCPPGKNLCYKMFMVSD
>ID29271-NO
LQDAEDSSRFDADDTLAGEARELSTP
>ID29273-NO
LQDLALQGAKERAHQQ
>ID29274-NO
LQEDG
>ID29275-NO
LQEQEELENYIEHVLLHRP
>ID29277-ABP_both
LQFPVGRVHRLLRK
>ID29282-ABP_both
LQKYYARVRGGRAAVLS
>ID29283-ABP_both
LQKYYARVRGGRAAVLSALPKEEQIGKASTRGRKAARRKK
>ID29284-ABP_both
LQKYYCRVRGGRCAVLS
>ID29285-ABP_both
LQKYYWRVRGGRWAVLS
>ID29286-ABP_both
LQKYYWRVRGGRWAVLSWLPKEEQIGKWSTRGRKWWRRKK
>ID29290-ABP_both
LQLLKQLLKLLKQFSGGFIKHFIHRF
>ID29294-NO
LQNPTPE
>ID29296-NO
LQPEVMG
>ID29297-NO
LQPEVMGVSK
>ID29299-NO
LQPHQ
>ID29300-NO
LQPSS
>ID29302-NO
LQQALFIHFRIGRRRRRRRR
>ID29304-NO
LQQLAFIHFRIGRRRRRRRR
>ID29305-NO
LQQLLAIHFRIGRRRRRRRR
>ID29313-NO
LQQLLFRRRRRRRR
>ID29315-NO

LQQQP
>ID29316-NO
LQQQQ
>ID29318-ABP_neg
LQSTKRFIKWYNAWNEKRR
>ID29320-ABP_both
LQTKLKKLLGLESVF
>ID29327-ABP_both
LRALLRALLRAL
>ID29331-NO
LRCGG
>ID29332-ABP_both
LRCIALRKK
>ID29335-NO
LRCNG
>ID29338-NO
LRDLGCYCRKRGCTRRERINGTCRKGHLMYTLCCL
>ID29339-NO
LRDLKCFCRRKSCNWGEGIMGICKKRYGSPILCCR
>ID29340-NO
LRDLVCYCRARGCKGRERMNGTCSKGHLLYMLCC
>ID29342-NO
LRDLVCYCRKRGCKRREHINGTCRKGHLLYMLCCR
>ID29343-NO
LRDLVCYCRKRGCKRREHMNGTCRKGHLLYMLCCR
>ID29353-NO
LRECCGRVGPMCPKCMCPPRRC
>ID29356-NO
LRGCVPSGEICYFMDHIGCCSGKCTFVCM
>ID29357-NO
LRGEPIRF
>ID29364-NO
LRIPVA
>ID29369-ABP_both
LRKFFARIRGGR
>ID29370-ABP_both
LRKFFARIRGGRAAVLNALGKEEQIGRASNSGRKCARKKK
>ID29372-NO
LRKKQ
>ID29373-ABP_both
LRKKWFW
>ID29375-ABP_both
LRKLRKRLLLRKLRKRLL
>ID29376-ABP_both
LRKLRKRLVRLASHLRKLRKRLL
>ID29377-ABP_pos
LRKLWKRLAKIIKRLYRYLRRPVR
>ID29378-ABP_pos
LRKLWKRVVKIWKRYLRQLRRPVR
>ID29379-NO
LRKNLRWLF

>ID29381-ABP_both
LRKRLRKFRNKIKEKLKKIGQKIQGFVPKL
>ID29387-ABP_neg
LRLLRRL
>ID29392-NO
LRMTCAFGVAQRSADCALSS
>ID29396-NO
LRNQLDIGDLQ
>ID29399-ABP_both
LRPHPPRPQPIYVPRNNG
>ID29401-NO
LRPVAA
>ID29403-NO
LRQRQ
>ID29404-NO
LRQSQ
>ID29405-NO
LRRFSTMPFMFCNINNVCNF
>ID29409-ABP_pos
LRRLWKRLVKILKRWFRYLRRPVR
>ID29410-ABP_pos
LRRLWKRLVKLWKRFFRYLKKPVR
>ID29411-ABP_pos
LRRLWKRLVRIIKRIYRQLKRPVR
>ID29412-ABP_pos
LRRLWKRVFKIIKRLFRQLKKPVR
>ID29413-ABP_pos
LRRLYKRVFRLLKRWWRYLKRPVR
>ID29414-ABP_pos
LRRLYKRVVKLWKRLFRQLRRPVR
>ID29415-NO
LRRQQ
>ID29416-ABP_pos
LRRVWKRVARIIKRLYRYLKRPVR
>ID29417-ABP_pos
LRRVYKRLARLIKRYLRQLKKPVR
>ID29418-ABP_pos
LRRVYKRLFRLWKRIIRQLRRPVR
>ID29425-NO
LRSRGELVAKFLAGEQSPEDYVAE
>ID29427-NO
LRTNVVH
>ID29428-NO
LRVAPEEHPVLL
>ID29429-ABP_both
LRVRLASHLRKLRKRLL
>ID29430-ABP_both
LRVRLASHLRKLRKRLLR
>ID29434-NO
LRWCIPSGDLCFRSDHIGCCSGKCAFVCL
>ID29435-NO

LRWCIPSGDLCFRSDHIQCCSGKCAFVCL
>ID29437-ABP_both
LRWLRRL
>ID29438-ABP_both
LRWLRWG
>ID29460-ABP_both
LSAVGKIASKVVPSVIGAFK
>ID29461-ABP_both
LSAVGSALSSSGGQE
>ID29462-NO
LSCCADPACKHTPGC
>ID29465-NO
LSDSPNGPWVWVPAFSQAVG
>ID29469-ABP_both
LSFPTTKTYFPHFDLSHGSAQVKGHGAKVAAA
>ID29471-ABP_both
LSFPTTKTYFPHFDLSHGSAQVKGHGAKVAAALTKAVEHLDDLPGALSEL
>ID29474-ABP_both
LSFVGRVASKVVPSLISMIK
>ID29476-NO
LSITNDLRAIADSYLYDQHKLRERQEENLRRRFLEL
>ID29477-NO
LSKDIGSESTEDQAMED
>ID29478-NO
LSKRDCLPDYTICAFNMGLCCSDKCMLVCLP
>ID29495-NO
LSMFTRP
>ID29496-NO
LSMGSASLSP
>ID29505-NO
LSPLLSPATA
>ID29506-NO
LSPQSY
>ID29510-NO
LSQSKVLPVPQK
>ID29511-NO
LSRGQ
>ID29513-NO
LSSKFNFGL
>ID29514-NO
LSSSEESTRINKKIEKFQSEEQQQYEDELQDKIHPFAQTQSLVYPFPGPI
>ID29515-NO
LSSTCILVLVKDILVLVVKEILVLVVKDKPI
>ID29516-NO
LSSTCILVLVKSTY
>ID29517-NO
LSSVNSFPVVTP
>ID29518-NO
LSTDIVAPPV
>ID29522-NO
LSTFFRLFNRSFTQALGK

>ID29528-NO
LSYCG
>ID29531-ABP_neg
LTAEDKKLIQQAWEKAASHQEEFGAEALTRMFTTYPQTKTY
>ID29536-NO
LTCLTKMVECIQLPLDVEDSSDTLCC
>ID29537-NO
LTCTNPTCSAQCRGRGYRRGSCTIGRCFCSYV
>ID29542-NO
LTCYKGYHDTVVCKP
>ID29543-NO
LTCYKGYRDTV
>ID29544-NO
LTDLENLHLP
>ID29545-NO
LTDVEN
>ID29546-NO
LTEQIRERQRYLADLRQRLLEK
>ID29549-NO
LTGTCLQYQSRCGNTR
>ID29550-NO
LTIKGCCSDPSCNVNNPDYCG
>ID29551-NO
LTIKGCCSYPPCFATNPDCGRRR
>ID29553-ABP_neg
LTKKTKKQKRNLVGTT
>ID29558-NO
LTLRWVGLMS
>ID29559-NO
LTLTDVE
>ID29560-NO
LTLTDVEN
>ID29561-NO
LTMPSDLQPVLW
>ID29564-NO
LTQTPVVPP
>ID29565-NO
LTQTPVVVPPF
>ID29568-NO
LTRNYEAWVPTP
>ID29573-NO
LTSLRGPAESSGESAHPLE
>ID29574-NO
LTSNKDEEQRELLKAISNLLD
>ID29575-NO
LTVEPWL
>ID29576-NO
LTVLPW
>ID29580-NO
LTVQPWP
>ID29583-NO

LTVSLWT
>ID29584-NO
LTVSPLWD
>ID29586-NO
LTVSPWY
>ID29589-NO
LTWNKFQGSW
>ID29593-NO
LTYQTWP
>ID29594-NO
LVATGMAAGVAKTIVNAVSAGMDIATALSLFSGAFTAAGGIMALIKKYAQ
>ID29596-NO
LVAYGIAQGTAEKVVSLINAGLTVGSIISILGGVTVGLSGVFTAVKAAIA
>ID29597-NO
LVCLPPSCE
>ID29606-NO
LVGVRLL
>ID29611-ABP_both
LVIRTVIAGYNLYRAIKKK
>ID29613-ABP_both
LVKKLLKLAMGFG
>ID29614-ABP_both
LVKLVAGIKKFLKWK
>ID29616-ABP_both
LVKRFKKFFRKLKKS
>ID29617-ABP_both
LVKRFKKFFRKLKKSV
>ID29618-ABP_both
LVKRFKKFFRKLKKSVK
>ID29619-ABP_both
LVKRFKKFFRKLKKSVKK
>ID29624-NO
LVNDLVTPVFDNL
>ID29626-NO
LVNQHLCGSHLVEALYLVCGDRGFFYYPKV
>ID29630-NO
LVPFRPRL
>ID29631-NO
LVPLPKIKNSTFT
>ID29632-NO
LVPRGSRAGSPSGGPFCALARQPLTGARLMSGLFFALHET
>ID29634-NO
LVQGS
>ID29636-ABP_both
LVQRFHAYLHKFREAFMNVGAAAAVEGTKAA
>ID29638-ABP_both
LVQRGRFGRFLRKIR
>ID29639-ABP_both
LVQRGRFGRFLRKIRRFR
>ID29640-NO
LVQVQETKEGTTRLLNSCEYKGRLSKAGAGPAPDHQAEASTVTP

>ID29646-NO
LVRRWYL
>ID29649-ABP_both
LVRVRRGFGCPFDER
>ID29651-NO
LVSLPQIAHADCCSDPACKQTPGC
>ID29654-ABP_both
LVTLASHLPSDFTPAVHASLDKFLANVSTVLTSKYR
>ID29656-NO
LVVRGGCCSHPVCYFNNPQMCRGRR
>ID29657-NO
LVVYPWT
>ID29658-NO
LVVYPWTQR
>ID29667-NO
LVYPFP
>ID29668-NO
LVYPFPGP
>ID29670-NO
LVYPFPGPIH
>ID29672-NO
LVYPFPGPIPN
>ID29673-NO
LVYPFPGPIPNSLP
>ID29674-NO
LVYPFPGPIPNSLPQNIPP
>ID29678-NO
LWAEMTG
>ID29679-NO
LWDENEKW
>ID29681-NO
LWENLRF
>ID29683-ABP_both
LWFHWK
>ID29684-ABP_both
LWGTRSGIQP
>ID29685-ABP_pos
LWGVWRRVIDWLR
>ID29690-ABP_pos
LWLLWLRWL
>ID29691-ABP_pos
LWLWWLRWL
>ID29699-NO
LWVPGMV
>ID29725-NO
LYAAIIAKLLES
>ID29727-NO
LYAKIIKKLLES
>ID29729-NO
LYDFGL
>ID29732-NO

LYFAPRL
>ID29738-NO
LYKNG
>ID29740-NO
LYLKQADFDDPRMFTSSF
>ID29745-NO
LYPLHTYTPLSLPLF
>ID29749-NO
LYQKS
>ID29750-NO
LYRNG
>ID29756-NO
LYTHFSTRL
>ID29920-NO
MAIPPK
>ID29921-NO
MAIPPKK
>ID29938-NO
MAIYRDLIS
>ID29948-NO
MAKEFGIPAAVAGTVINVVEAGGWVTTIVSILTAVGSGGLSLLAAAGRES
>ID29966-NO
MAKFASIITLLFAALVVFAAFEAPTMVEA
>ID29982-NO
MAKHAM
>ID30028-ABP_neg
MAKLLRLDKKRNKFLCFV
>ID30048-NO
MAKSATIVTLFFAALVFFAALEAPMVVEA
>ID30143-NO
MANLGCWMLVLFVATWSDLGLCKKRPKP
>ID30158-NO
MAPRGFSCLLLLTSEIDLPVKRRA
>ID30207-NO
MARAKE
>ID30226-NO
MARSGL
>ID30228-NO
MARSICFFAVAILALMLFAAYETEA
>ID30230-NO
MARSLCFMAFAILAMMLFVAYEVQA
>ID30252-ABP_both
MARVLLRLIRL
>ID30254-NO
MARVSLLLIVLSIALVAPSQGFLKDLLFGEAKTALLEDGTTEILDHVCNF
>ID30352-NO
MCESASSK
>ID30358-ABP_both
MCIKTTSGIHPKNIQ
>ID30359-NO
MCIPCFTTNPNMAAKCNACCGSRRGSCRGPQCIC

>ID30364-NO
MCMPCFTTDHNMAKKCRDCCGGNGKCFGPQCLCNR
>ID30368-NO
MCMPCFTTRPDMAQQCRDCCGGNGKCFGYQCLCNR
>ID30380-NO
MDANAFRMSFG
>ID30382-NO
MDAQTRRRERRAEKQAQWKAANGC
>ID30388-NO
MDELYPMEPEEEANGGEILA
>ID30389-NO
MDELYPVEPEEEANGGEILA
>ID30394-NO
MDFLI
>ID30401-NO
MDGFAFAPGL
>ID30453-NO
MDNNFMRF
>ID30457-NO
MDPNAFRMSF
>ID30460-NO
MDQFSFGPGL
>ID30465-NO
MDSFAFAPGL
>ID30466-NO
MDSFMFGSRL
>ID30468-NO
MDSNFIRF
>ID30469-NO
MDSNFMRF
>ID30471-NO
MDSNKDERAYAQWVIIILHNVGSSPFKIANLGLSWGKLYADGNKDKEVYP
>ID30502-NO
MEGAQEAQGD
>ID30504-NO
MEHFKF
>ID30505-NO
MEHFRW
>ID30527-NO
MEKKSLAGLCFLFLVLFVAQEIVVTEA
>ID30545-ABP_both
MELRGCNNSGCDSVCRALGFNHGRCVSADTCRCYN
>ID30559-NO
MENRWQVM
>ID30563-NO
MEPECNLNCTD
>ID30601-NO
MEWYPEAAANAERYTQIVWYK
>ID30603-NO
MFCRMRSCD
>ID30757-NO

MFSPILSLEIILALATLQSVFAQPVICTTVGSAAEGS
>ID30928-NO
MFTLKKSLLLIVLLGIISLSLCEQERAADEDETNAEEERRDEKGPKWKR
>ID31701-ABP_pos
MFTMKKSMLLIVLLGIISLSLCEQERNADEDEQSEM
>ID31791-ABP_both
MGGLKKLGKKLEGAGKRVFNAAEKALPIAQQAANVAATARG
>ID31815-NO
MGLGLHLLVLAAALQGAKKKRKV
>ID31816-NO
MGLGLHLLVLAAALQGAWSQPKKKRKV
>ID31817-NO
MGLILPALRQQSCCDIQWCYDNCDCCI
>ID31822-NO
MGMRLPNIIFL
>ID31853-NO
MGWMDFG
>ID31854-NO
MGYILPALSQQTCCVRPWCDGACDCCVDP
>ID31866-NO
MHKRPTTPSRKM
>ID31869-ABP_both
MHMNVQKGKWDKDPK
>ID31870-ABP_both
MHMNVQNGKWDSDPS
>ID31875-NO
MHYGN
>ID31876-NO
MIAYRDLIS
>ID31877-NO
MICYSHKTPQPSATIGCEEKTCYKKSVRKL
>ID31885-NO
MIFLFGGCCRMSSCQPPPVCNCCAKQDLNPDER
>ID31886-NO
MIFPGAGGPEL
>ID31887-NO
MIFPGGPQL
>ID31891-ABP_both
MIHGLVTRR
>ID31893-NO
MIHTNHWWAQD
>ID31895-NO
MIIARDLIS
>ID31902-NO
MIIFRDLISH
>ID31905-NO
MIIFRIAAYHKK
>ID31906-NO
MIIFRILISHKK
>ID31908-NO
MIIRRDLISE

>ID31909-NO
MIISRDLISH
>ID31911-NO
MIIYADLIS
>ID31912-NO
MIIYARRAEE
>ID31913-NO
MIIYRAEISH
>ID31914-NO
MIIYRALIS
>ID31915-NO
MIIYRALISHKK
>ID31916-NO
MIIYRD
>ID31917-NO
MIIYRDAIS
>ID31918-NO
MIIYRDKKSH
>ID31919-NO
MIIYRDL
>ID31920-NO
MIIYRDLAS
>ID31921-NO
MIIYRDLI
>ID31922-NO
MIIYRDLIA
>ID31924-NO
MIIYRDLISH
>ID31926-NO
MIIYRIAASHKK
>ID31941-NO
MILPSLADLHRYTMYD
>ID31956-NO
MIPAY
>ID31970-NO
MISMLRCTFFFVSVILITSYFVTPTMSIKCNRKRHVIKPHICRKICGKNG
>ID31983-NO
MKAEH
>ID32019-NO
MKDLMSLVIAPIFVGLVLEMISRVLDEEDDSRK
>ID32236-NO
MKLMLSALRQQTCCEPSTCDGGCYHCC
>ID32304-NO
MKPWIQPK
>ID32328-NO
MKRSRGPSPRR
>ID32351-ABP_both
MKSIAKFIAKTVAKQGAKQG
>ID32373-ABP_both
MKTFLRLYRSLINKVLH
>ID32377-NO

MKTFSVAVAVAIVLAFICTQESSALPVTGVEELVELVSSDDPVADHQELP
>ID32379-NO
MKTFSVAVAVAVVLAFICTQESSALPVTGIEELVEPVSSDNNDNHQGLPV
>ID32492-NO
MKVFFLFAVLFCLVRRNSVHISHQEARGP
>ID32552-NO
MKYLWVFIVFSIAVLSHACSA
>ID32572-NO
MLGQTPT
>ID32573-NO
MLGQTPTK
>ID32584-ABP_both
MLKKFRGMF
>ID32598-NO
MLLCS
>ID32606-NO
MLLPATMSDKPDMAEIEKFDKSKLKKTETQEKNPLPSKETIEQEKQAGES
>ID32622-NO
MLQNSAVLLLLVISASA
>ID32630-ABP_both
MLSLIFLHRLKSMRKRLDRKLRLWHRKNYP
>ID32640-ABP_both
MLTAEEKAAVTAF
>ID32641-ABP_both
MLTAEEKAAVTAFWGKVKVDEVGGEALGRL
>ID32664-NO
MMEPMVK
>ID32668-NO
MMHKSGCFGRRLDRIGSLSGLGCNVLRKY
>ID32677-NO
MMKQQFFLFLAVIVMISSVIEAGRGKEM
>ID32688-NO
MMRDSGCFGRRLDRIGSLSGLGCNVLRRY
>ID32691-NO
MMSQLAH
>ID32697-NO
MMVPI
>ID32716-NO
MNFQQRLQSLWTLARPFCPPLLATASQMQMVVLPCLGFTLLLWSQVSG
>ID32723-NO
MNGKCKCYNN
>ID32778-NO
MNLNGLPASTNVIDLRGKDMGTYIDANGACWAPDTPSIIMYPGGSGPSYS
>ID32799-NO
MNPPK
>ID32853-NO
MNVKHWPWMK
>ID32855-NO
MNVRGCCSHPVCRFNYPKYCGGRR
>ID32861-NO
MNYLVFFSLALLVMTGRTVTREKRKDMMDL

>ID32869-NO
MPCPKILKQCKSDEDCCRGWKCFGFSIKDKMCISR
>ID32870-NO
MPDQDPDRNSMLLNENSMLTEPIEPLNM
>ID32880-NO
MPFDL
>ID32881-NO
MPFDLRRGSSDTDLDLQGHVDLGLDDLDKLRLIFPPGLIEEA
>ID32883-NO
MPFEF
>ID32886-NO
MPFPKYPVQPF
>ID32890-NO
MPHSFANLPLRF
>ID32906-ABP_both
MPKRKAEGDAKGDKAKV
>ID32909-NO
MPKTRRRPRRSQRKRPPTPWP
>ID32910-NO
MPKTRRRPRRSQRKRPPTPWPYGRKKRRQRRR
>ID32915-NO
MPPGMSPFR
>ID32916-NO
MPPKPDNPSPDASPELSKYMLAVRNYINLITRQRY
>ID32925-ABP_both
MPRRRRSSSRPVRRRRRPRVSRRRRRRGGRRR
>ID32937-NO
MPTWAWWLFLVLLLALWAPARG
>ID32938-ABP_both
MPVGIVIAPKKSPFTAKKPGPVLSGVKAGPG
>ID32957-NO
MQFITDLIKKAVDFFKGLFDNK
>ID32963-NO
MQGKISSEQHPMFDPIEGCCTQSCTTCFPCCLI
>ID32967-NO
MQIFVKTLTGKTITLEVEPSDTIENVKAKIQDKEGIPPDQQRLIFAGKQL
>ID33092-ABP_both
MRILSIIRWTRMKKSSA
>ID33093-NO
MRILYLLFSVLFLVLQVSPGLSLPQRDMFLCRIGSCHFGRCPIHLVRVGS
>ID33102-NO
MRKDQ
>ID33105-NO
MRKGVVTGLFVALVVMCLYLPQPCEAQYEALTAAILTKLSKMWHSDTLNF
>ID33117-NO
MRKWFHNVLSSGQLLADKWPAWDYNWK
>ID33200-NO
MRWRD
>ID33220-NO
MSDINATRLPIWGIGCNPCVGDDVTTLLTRGEALC
>ID33249-ABP_both

MSGSHHHHHHGSSGENLYFQSLEVASFDKSKLK
>ID33270-NO
MSKHLQSMRIGNLYLPVGRQEA
>ID33335-NO
MSRPHK
>ID33342-NO
MSRTMS
>ID33382-NO
MSWLNFLKYIAKYGKKAVSAAWKYKGKVLEWLNVGPTLEWVWQKLKKIAG
>ID33392-NO
MTCGQVQGNLAQCIGFLQKGGVVPPSCCTGVKNILNSSRTTADRRAVCSC
>ID33407-NO
MTEQKPKPSCHNVMVGNYVPTASDRAANRTLGFGLVTNIINGGLDC
>ID33434-NO
MTKCRG
>ID33445-NO
MTKHCTPPEVGCLFAYECCSKICWRPRCYPS
>ID33482-NO
MTKSAG
>ID33502-NO
MTMGCTHPGGACAGHHHCCSQSCNTAANSCN
>ID33518-NO
MTPSSLSTLPWP
>ID33528-NO
MTQMIS
>ID33535-NO
MTRNLQ
>ID33536-NO
MTRQIG
>ID33572-NO
MTVRECCSQPPCRWKHPELCS
>ID33573-NO
MTVREECCSDPRCSVGHQDMCR
>ID33602-NO
MVGSAPGVL
>ID33622-NO
MVKSKIGSWILVLFVAMWSDVGLCKKRPKP
>ID33668-NO
MVRRFLVTLRIRRACGPPRVRVFVVHIPRLTGEWAAP
>ID33690-NO
MVTVLFRRLRIRRACGPPRVRV
>ID33691-ABP_both
MVVFSVPKFKSTVAKLLSSA
>ID33698-NO
MVWSKGPLFL
>ID33726-NO
MYDCCT
>ID33727-NO
MYDESTGYSSALK
>ID33729-NO
MYECG

>ID33755-NO
MYPGIA
>ID33771-NO
NAAAKAFGLITPTVRKGCCSNPACMLKNPNLC
>ID33772-NO
NAAANDKASDVIPLALQGCCSNPVCHVDHPELCL
>ID33774-NO
NAAKYNPCASYL
>ID33775-NO
NAAVAGAVIEGATLTFEVLQ
>ID33776-NO
NACCIVRQCC
>ID33777-NO
NACESAICG
>ID33779-NO
NAECCYYPPCYEAYPEICL
>ID33780-NO
NAEDHHEHQ
>ID33783-NO
NAELINSLLGVPRVMSDA
>ID33786-NO
NAEPNFLRF
>ID33787-NO
NAERLGDSFQEMDKEVDQLAEPQHLECTVHWPRSPLRDLRGVLESLIEEE
>ID33788-NO
NAETDLDDPLRNIKLSSESALTYLY
>ID33789-NO
NAGAPQHLCGSHLVDALYLVCGPTGFFYNP
>ID33790-NO
NAGPFTPTVNREQLSTS
>ID33793-NO
NAKNVIDSFQEIAKEVDQPVEPKCCGCIVHQSHSPLRDLKAALESLIE
>ID33794-NO
NAKTRRHERRRKLAIERGC
>ID33798-NO
NAPPEPVPPPRAAPAPTHV
>ID33800-NO
NAQRP
>ID33802-NO
NASKYNPCSNYL
>ID33810-NO
NCCIYENWCCEWI
>ID33825-NO
NCGEQGEGCATRPCCAGLSCVGSRPGGLCQYD
>ID33827-NO
NCIPKNHFCGLLHHSRNCCTPTCLIVCF
>ID33830-NO
NCNKYGNACFGAH
>ID33831-NO
NCPAGCRSQGCCM
>ID33832-NO

NCPYCVVYCCPPAYCEASGCRPP
>ID33841-NO
NDDCELCVNVACTGCL
>ID33842-NO
NDDPPISIDLTFHLLRNMIEMARIENEREQAGLNRKYLDEV
>ID33845-NO
NDFSRDIMSF
>ID33848-NO
NDNADYPLRLNLDYLPVDNPAFHSQENTDDFLEE
>ID33849-NO
NDPCEEVCIQHTGDVKACEEACQ
>ID33851-NO
NDPEVPGMWFGPRL
>ID33852-NO
NDPFLRF
>ID33853-NO
NDPFLRFGKKSDPFLRF
>ID33854-NO
NDPLWTSFNENALLEENFE
>ID33855-NO
NDPYLRF
>ID33856-NO
NDQCLVIEIL
>ID33860-NO
NDVKDGAASGAHSDRLGLWFGPRL
>ID33862-NO
NDWSKFGQSW
>ID33863-NO
NECCDNPPCKSSNPDLCDWRS
>ID33867-NO
NEKWLWLE
>ID33868-ABP_both
NENLLRFF
>ID33869-NO
NENLLRFFVAPFPE
>ID33875-NO
NEVCPPGECQQYCCDLRKCKCINLSFYGLTCNCDS
>ID33876-NO
NEVCPPGRCEPYCCDPRKCKCLSIDFYGLVCNCDS
>ID33877-NO
NEVPFEF
>ID33881-NO
NFDEIDR
>ID33882-NO
NFDEIDRA
>ID33883-NO
NFDEIDRAG
>ID33884-NO
NFDEIDRAGF
>ID33885-NO
NFDEIDRAGFGFL

>ID33886-NO
NFDEIDRAGFGFN
>ID33889-NO
NFDEIDRS
>ID33890-NO
NFDEIDRSA
>ID33891-NO
NFDEIDRSDFA
>ID33894-NO
NFDEIDRSGFA
>ID33895-NO
NFDEIDRSGFDG
>ID33896-NO
NFDEIDRSGFDGFV
>ID33904-NO
NFDEIDRSGFNS
>ID33905-NO
NFDEIDRSGFNSFI
>ID33917-NO
NFDEIDRTGFEGFY
>ID33918-NO
NFDEIDRTGFGFH
>ID33919-NO
NFDEIDRVGF
>ID33920-NO
NFDEIDRVGFGSFI
>ID33921-NO
NFDEIDRYSTFG
>ID33922-NO
NFDELDRSGFGFH
>ID33926-NO
NFEHSCNGYMRPHPRGLCGEDLHVIISNLCSSLGGNRRFLAKYMV
>ID33927-NO
NFGAPGGASPI
>ID33928-NO
NFGAPGGAYPW
>ID33930-NO
NFGYDLYRVRSSTSPTTN
>ID33931-NO
NFKFNPWG
>ID33932-NO
NFKVEGACSKPCRKYCIDKGARNGKCINGRCHCYY
>ID33935-NO
NFLRF
>ID33936-NO
NFLTGLGHRSDHYNCVRSGGQCLYSACPIYTKIQGTCYHGKAKCCK
>ID33942-NO
NGAPQPFVRFG
>ID33943-NO
NGCCRAGDCCS
>ID33944-NO

NGCCRAGDCCSRFEIKENDF
>ID33945-NO
NGCCRNPACESHRC
>ID33947-NO
NGERAPGSKKAPSGFLGTR
>ID33949-NO
NGFFF
>ID33952-NO
NGFSHHAPLMRY
>ID33958-NO
NGIWY
>ID33962-NO
NGKKACLNPASPMVQKIIEKIL
>ID33964-NO
NGKQVCLDPEAPFLKKVIQKILDS
>ID33965-ABP_both
NGKTWKKNRKACQVK
>ID33966-NO
NGNYRMHHFRWGSPPKD
>ID33968-NO
NGRAHA
>ID33969-NO
NGRCCHPACAKYFSC
>ID33971-NO
NGRCCHPACARKYNC
>ID33972-NO
NGRCCHPACGAKYFKCG
>ID33973-NO
NGRCCHPACGGKYVKC
>ID33975-NO
NGRCCHPACGKHFSC
>ID33979-NO
NGREACLDPEAPMVQKIVQKMLKG
>ID33980-NO
NGRKACLNPASPIVKKIIEKMLNS
>ID33982-NO
NGRSL
>ID33984-NO
NGSAGNGGLWFGPRL
>ID33985-NO
NGSYPFGL
>ID33987-NO
NGVCCGYKLCHPCAG
>ID33991-ABP_both
NGVQPKYKWWKWWKKWWW
>ID33992-ABP_both
NGVQPKYRWWRWWRRWWW
>ID33995-NO
NGWNN
>ID33999-NO
NHLIGFDDPRLFSSSY

>ID34000-NO
NHRNRMMDHVH
>ID34001-NO
NHRSCYRNKGVCAPARCPRNMRQIGTCHGPPVKCCRKK
>ID34007-NO
NIASLARKYELP
>ID34008-NO
NIASLMRDYDQSRENRVPFP
>ID34009-NO
NIATMARLQSAPSTHRDP
>ID34010-NO
NIDEIDRTAFDNFF
>ID34011-NO
NIDTDI
>ID34012-NO
NIDTDL
>ID34020-NO
NIFNAISAAGLGNQIKVSTAIDTGVLGTSYPPSK
>ID34021-NO
NIFYCP
>ID34023-NO
NIGTLARDFQLPQN
>ID34025-NO
NIIDVPCRPCYYRDSSGNCVYDQLGCGA
>ID34026-ABP_both
NIILWG
>ID34027-NO
NIIPA
>ID34035-NO
NILNTIINLAKKILGK
>ID34037-NO
NILRG
>ID34038-NO
NILSGIANGINRVLSWFG
>ID34044-NO
NIPPLTQTPVVVPPFIQ
>ID34045-NO
NIPPLTQTPVVVPPFIQPEVMGVSK
>ID34046-NO
NIQIICCKHTPACCT
>ID34048-ABP_both
NIQPPCRCC
>ID34049-NO
NIQSLKNAQGQGGGSSSG
>ID34051-NO
NIQSLLRTGMLPSIAPK
>ID34052-NO
NIQTLVRDWNLPRQQSMAADNE
>ID34055-NO
NISRCTHPFMACGKQS
>ID34057-NO

NITVTLKKFPL
>ID34058-NO
NIVDVPCRDDYYRDSDGNCVCCKFGGA
>ID34059-NO
NIVDVPCRDDYYRDSSGNGVYDQLGGCGAA
>ID34060-NO
NIWNR
>ID34069-ABP_both
NKKAGLFVVQFPKKY
>ID34080-NO
NKLLYPSVI
>ID34082-NO
NKPILVFY
>ID34087-NO
NKRILIRIMTRP
>ID34090-NO
NKSVIKGNPASNLAQCVFSFFKKC
>ID34095-NO
NLAPFL
>ID34096-NO
NLAQFGFMIRCANGGSRSALDYADYGC
>ID34098-NO
NLASARASGYMLN
>ID34306-NO
NLDEIDRSDFSRFV
>ID34307-NO
NLDEIDRSNFGTFA
>ID34308-NO
NLDEIDRVGWSGFV
>ID34309-NO
NLDTDI
>ID34310-NO
NLDTDL
>ID34312-NO
NLFQFAEMIVKMTGKEAVH
>ID34313-NO
NLFQFARMINGKLGAFSV
>ID34314-NO
NLGALKSSPVHGVQQ
>ID34316-NO
NLGSLARAGLLRTPSTDYL
>ID34317-NO
NLHLP
>ID34323-NO
NLIQFANMIGCANHGSR
>ID34324-NO
NLIQFSNMIQLDRCCETHDNEAEKKGCYPKLTLY
>ID34325-ABP_both
NLLGFLQGAKDILKECEADNYQGWLCESYKPQ
>ID34326-ABP_both
NLLKSALKTVNKLLAAS

>ID34329-NO
NLLQFENMIRNVAGRSGIWWYSD
>ID34330-NO
NLLQFGFMIRCANKRRRPVWPYEESGC
>ID34331-NO
NLLQFGYMIRCANGRSRPVW
>ID34333-NO
NLLQFNKMIKIMTKKNAIPFYSS
>ID34335-NO
NLLQFRKMIKKMTGKEVVWYAFYGCYCGGGGK
>ID34336-NO
NLLQIGIMKRCANKRRRPVFHYRDYGCYC
>ID34338-NO
NLLRF
>ID34339-NO
NLLSLLQHAIETANNAYRSPR
>ID34341-NO
NLMQFELLIMKVAGRSGIVWYSD
>ID34342-NO
NLMQFETLIMKVAGRSGVWYYGS
>ID34343-NO
NLNNLGSFQVHGW
>ID34344-NO
NLPFSPRL
>ID34345-NO
NLPIVERPVCKDSTRIRITDNMFCAGYKPDEGKRGDACEGDSGGPFVMKS
>ID34352-NO
NLQYLKNMIKCTNTRHWLSFTNYGCYCG
>ID34354-NO
NLVQFELLIMKVAKRSGLLSYSA
>ID34355-NO
NLVQLGKMIFQETGKNPATSYGL
>ID34356-ABP_both
NLVSALIEGRKYLKNVLK
>ID34359-NO
NLWNTIKETGKKFAVGLLDKIKCGITGTCKT
>ID34360-NO
NLYQFGGMIGCANKGTRSWLSYVNYGCYCG
>ID34361-NO
NLYQFKEMIRYTIP
>ID34362-NO
NLYQFKNMIECAGTRTWIAYVKYGAYTYAYT
>ID34363-NO
NLYQFKNMIHCTVPSRPWWHFADYGCYCGR
>ID34364-NO
NLYQFKNMIKCTNTRMWWSFTNAGCYDG
>ID34365-NO
NLYQFKNMIQCTTKRSVLEFMEYGCYC
>ID34366-NO
NLYQFKNMIQCTVPNR
>ID34368-NO

NLYVASW
>ID34371-NO
NMAINPSK
>ID34378-NO
NMQSLARDNSLPHFAGAAAQES
>ID34379-NO
NMSPQLD
>ID34384-NO
NNAEVVNHILKNFGALDRLGDV
>ID34385-NO
NNAEVVNHILKNFGALDRLGDVG
>ID34390-NO
NNCKHKKGSGC
>ID34392-ABP_both
NNEAQCEQAGGDCSKDHCFHLHTRAFGHCQRGVPCCRDVYD
>ID34393-ABP_both
NNEAQCEQAGGICSKDHCFHLHTRAFGHCQRGDPCCRTDYD
>ID34397-NO
NNFYSYNAFITAAKSFPGFGTTGDTAVRGPIQISYNYNYGPCGR
>ID34398-NO
NNGNN
>ID34440-ABP_both
NNLRHIVAWCKNRNYSLAVCARFKPQ
>ID34444-NO
NNNAAGRKRKKRT
>ID34445-ABP_both
NNNPRPPYLPRPRPP
>ID34446-NO
NNNWNNN
>ID34448-NO
NNNWTKFQGSW
>ID34449-NO
NNPNVFYPWG
>ID34450-NO
NNPPCCSGYVCEGVYCAVDV
>ID34451-NO
NNQESYFV
>ID34452-NO
NNRACFRTSKGNPAECPYLG
>ID34454-NO
NNRSPQLRLRF
>ID34457-NO
NNVMLQW
>ID34458-NO
NNWGN
>ID34459-NO
NNWNG
>ID34460-NO
NNWNN
>ID34461-NO
NNWNRMQGMW

>ID34462-NO
NNWSKFQGSW
>ID34467-NO
NPANPLNLKKHHGVFCDVCKALVEGGEKVGDDDLDAWLDVNIGTLCWTML
>ID34469-NO
NPASCCSCADVDPGRASRKTPKGEDQVFIKEKDRC
>ID34470-NO
NPASGFFGMR
>ID34471-NO
NPAVVRP
>ID34476-NO
NPELYQMNHFRWGQPPTHF
>ID34477-NO
NPENDTPFGTMRF
>ID34478-NO
NPFHAWG
>ID34479-NO
NPFHSWG
>ID34487-NO
NPLGTMRF
>ID34488-NO
NPLGTMRFG
>ID34489-NO
NPLIPAIYIGATVGPSVWAYLVALVGAAAVTAANIRRASSDNHSCAGNRG
>ID34491-NO
NPLQDTEEKSRSFKASQSEPLDESRQLNEV
>ID34500-NO
NPPHQIYP
>ID34501-NO
NPQQDFMRF
>ID34502-NO
NPRWEFRGKFVGVR
>ID34504-NO
NPRWFWD
>ID34505-NO
NPSDFLRF
>ID34509-NO
NPTKYNFGL
>ID34510-NO
NPVYGSVSNADLMDFKNLLDHLEDKMPLED
>ID34512-NO
NPYAFGL
>ID34514-NO
NPYPGLW
>ID34515-NO
NPYSFGL
>ID34518-NO
NQFLP
>ID34522-ABP_both
NQHGQVVKIFHH
>ID34523-NO

NQKAISF
>ID34524-NO
NQKIITF
>ID34526-NO
NQKTISF
>ID34527-NO
NQKTMSF
>ID34530-NO
NQPGVNFL
>ID34531-NO
NQPNFLRF
>ID34532-NO
NQPTRAL
>ID34546-NO
NRARRNRRRVR
>ID34547-NO
NRCRGVSCT
>ID34551-NO
NRLKCRAQATHSAAPCIRGY
>ID34553-NO
NRLSRCIPSGDLCFPSDHIQCCNAECAFVCL
>ID34554-NO
NRLSRCIPSGDLCFPSDHIQCCNAKCAFACL
>ID34555-NO
NRLSRCIPSGDLCFPSDHIQCCNAKCAFVCL
>ID34559-NO
NRLSWCIPSGDLCFPSDHIRCCSAKCAFVCL
>ID34560-NO
NRLSWCIPTGDLCFPSDHIQCCSGKCTFVCM
>ID34562-NO
NRNFLRF
>ID34571-NO
NRPYIHPFQL
>ID34576-NO
NRRMKWKK
>ID34581-NO
NRSNLRF
>ID34585-NO
NRVYVHPFTL
>ID34591-NO
NRWYCNSAAGGVGGAAVCGLAGYVGEAKENIAGEVRKGWGMAGGFTHNKA
>ID34593-NO
NSASLISSWVDNTNFCCCSHDCATICDDCF
>ID34598-NO
NSEDGSPYPGPGQQPNCCKWPIVTCCNR
>ID34599-NO
NSEIINSLLGLPKVLNDA
>ID34601-NO
NSELINAILGSPTLMGEV
>ID34602-NO
NSELINSILGLPK

>ID34603-NO
NSELINSILGLPKVM
>ID34606-NO
NSELINSLLGIPK
>ID34607-NO
NSELINSLLGIPKVM
>ID34608-NO
NSELINSLLGIPKVMNDA
>ID34612-NO
NSELINSLLGLPRFMKVV
>ID34613-NO
NSELINSLLGLSRLMNEA
>ID34614-NO
NSELINSLLSLPKKLNDA
>ID34615-NO
NSELINSLLSLPKNMNNA
>ID34616-NO
NSFPLMLMHHHP
>ID34620-NO
NSGGGEGSGMWFGPRL
>ID34621-NO
NSGMINSILGIPRVMTEA
>ID34622-NO
NSGTMQSASRAT
>ID34624-NO
NSIINDVKNELFPEDIN
>ID34628-NO
NSKYVSKQKFYSWG
>ID34629-NO
NSPNIFGQWM
>ID34632-NO
NSSSCDTSVVRSTWACILQP
>ID34633-NO
NSSSGSAHQKREDVAAGEDRGLLPEGGPEPRGDGAGPGPRE
>ID34641-NO
NSVRGSR
>ID34648-NO
NTCEHLADTYRGVCFTNASCDDHCKNKAHLISGTCHDWKCFCTQNC
>ID34650-NO
NTEEGCLPPLSLCTMADDECCHDCILFLCLVSP
>ID34655-NO
NTFYCCELCCYPACAGCN
>ID34658-NO
NTGDKFYGLM
>ID34659-NO
NTGIAAERDLPKRYFDALAGQSL
>ID34660-NO
NTGMATLCR
>ID34663-NO
NTLFRF
>ID34664-NO

NTLPPFSPPSPP
>ID34667-NO
NTRGSWSNKRLSPR
>ID34670-ABP_both
NTVKETIKYLKSLFS
>ID34671-NO
NTVVLGKKQRFHSWG
>ID34672-NO
NVAAVARYNSQHGHIQRAGAE
>ID34674-NO
NVASLARTYTLPQNA
>ID34675-NO
NVCDGDACPDGVCRSGCTCDFNVAQRKDTCFYPQ
>ID34676-NO
NVDFNSESTRRKKKQKEIVDLHNSL
>ID34677-NO
NVDFNSESTRRKKKQKEIVDLHNSLRRRVSPTA
>ID34681-NO
NVGSHGFLRF
>ID34682-NO
NVGSVAREHGLPY
>ID34683-NO
NVGTLARDFALPP
>ID34684-NO
NVGTLARDFQLPIPN
>ID34686-NO
NVKCSGSPQCLKPCKDAGMRFGKCMNRKCHCTPK
>ID34687-ABP_both
NVLKKLNRLKEKNKAKNSKENN
>ID34690-ABP_both
NVLLWG
>ID34691-ABP_both
NVLLWK
>ID34692-ABP_both
NVLLWR
>ID34697-NO
NVPGEIVE
>ID34698-NO
NVPGEIVESL
>ID34699-NO
NVPIYQEPRF
>ID34700-NO
NVQLSTARGF
>ID34707-NO
NVVRQ
>ID34709-NO
NVWEKCCLDPRCSGKHQNKC
>ID34711-NO
NVWNK
>ID34712-NO
NVYSFGL

>ID34714-NO
NWGPLV
>ID34715-NO
NWGQFHGGW
>ID34717-NO
NWNKFQGSW
>ID34718-NO
NWNNLRGAW
>ID34720-NO
NWTPQAMLYLKGAQ
>ID34733-NO
NYAKSHFRSTALGRRTNGSVGSS
>ID34735-NO
NYCVAKRCRPGGRQCCSGKPCACVGKVCKCPRDNS
>ID34736-NO
NYDKNFLRF
>ID34737-ABP_both
NYISFFRKCKNSQST
>ID34739-NO
NYLHDTVGRLWGPSRAGLLDGLDWMVGDVQSR
>ID34744-NO
NYTTYKSHFQDR
>ID34746-NO
NYVMSHFHWNTFGQRMNGTPGGS
>ID34748-NO
NYYGWMDF
>ID34753-NO
PAASESGFRRDP
>ID34754-ABP_neg
PAAVRSPAQILQ
>ID34755-NO
PADLYEFGL
>ID34756-NO
PADPNFLRF
>ID34759-NO
PAETPNSLDLTFHLLREMIEIAKHENQQMQADSNRRIMDTI
>ID34760-NO
PAFYSQRY
>ID34761-NO
PAGPR
>ID34762-NO
PAGPVG
>ID34763-NO
PAHIAW
>ID34766-NO
PALEDPALVGPALI
>ID34768-NO
PALLAGDDDAEADEATELQQ
>ID34771-NO
PANLPWGSSNV
>ID34773-NO

PAPDSSFIRDP
>ID34774-NO
PAPDSSFLRDP
>ID34775-NO
PAPESGFIRDP
>ID34777-NO
PAPESSFIRDP
>ID34778-NO
PAPETNYLRDP
>ID34780-NO
PARAARRAARR
>ID34783-NO
PASDSGFLRDP
>ID34785-ABP_both
PAVFKIASKVVPSVYCKVSR
>ID34786-NO
PAVVLP
>ID34787-NO
PAVVRP
>ID34789-NO
PAWRHAFHWAWHMLHKAA
>ID34790-NO
PAWRKAFRWAWHMLHHAA
>ID34792-NO
PCCPLIPGCC
>ID34793-NO
PCCSGWCFFACA
>ID34797-NO
PCHIAW
>ID34799-NO
PCKELY
>ID34800-NO
PCKKSGRKCFPHQKDCCGRACIITICP
>ID34801-NO
PCKKTGRKCFPHQKDCCGRACIITICP
>ID34803-ABP_neg
PCNPDHDYRPFGNFRIAFTT
>ID34805-ABP_both
PCRGRSCGPRLRGGYTLIGRPVKNQNRPKYMWV
>ID34806-NO
PCRKTM
>ID34807-ABP_both
PDDFATKYSQQKYTK
>ID34809-NO
PDHIAW
>ID34812-NO
PDNFMRF
>ID34813-ABP_neg
PDRAIDTYRTSPVADQRYNA
>ID34814-NO
PDRFGFGL

>ID34815-NO
PDVDHVFLRF
>ID34816-NO
PDVGGFMVEDQRTHKSHNYMM
>ID34817-NO
PDYLQLARA
>ID34818-NO
PEAAELMMEVDP
>ID34822-NO
PECCSDPRCNSTHPELCG
>ID34827-NO
PEKFRPM
>ID34831-NO
PETSLLGGTEAGERLLQQGLAQVEA
>ID34834-ABP_neg
PEWSKCYQWQRRMRKLGAPSITCVRRTSA
>ID34835-NO
PFAQTQSLVYP
>ID34836-NO
PFCNAFAGC
>ID34837-NO
PFCNSYGCYNS
>ID34838-NO
PFDRISNSAFSDF
>ID34840-NO
PFFDPQIP
>ID34841-ABP_both
PFFPPRLPPRIPPGFPPRFPPRFP
>ID34844-NO
PFKLSKH
>ID34846-NO
PFNAFTG
>ID34847-NO
PFNFGL
>ID34851-NO
PFRGQGGWTLNSVGYNAGLGALRKLFE
>ID34852-NO
PFTESQS
>ID34867-NO
PGCCNNPACGANRCG
>ID34869-NO
PGCCNNPACVKHRCG
>ID34870-NO
PGDIPPGIRNTVCLMQQGHCRLFMCRSGERKGDICSDPWNRCCVPYSVKD
>ID34874-ABP_both
PGKYGFYTHVFRLKKWIQKVI
>ID34878-NO
PGPIHN
>ID34879-NO
PGPIP
>ID34881-NO

PGPLGLTGP
>ID34883-NO
PGQDFMRF
>ID34889-NO
PGTAVFK
>ID34890-NO
PGTCEICAYAACTGC
>ID34891-NO
PGVIPWN
>ID34892-NO
PGVNFLRF
>ID34898-NO
PHENACETYLKRR
>ID34902-NO
PHGGSAFVF
>ID34904-NO
PHQIYP
>ID34905-NO
PHSCNK
>ID34906-NO
PHSPTSL
>ID34907-ABP_both
PHVALKPGKLKFIIPSPKRPVKLLSG
>ID34908-ABP_both
PHVALKPGKLKFIIPSPKRPVKLLSGGNTLHLVSTTKT
>ID34912-NO
PICEVSRCW
>ID34914-NO
PIDERPM
>ID34916-NO
PIEDRPM
>ID34918-NO
PIHYIF
>ID34921-NO
PIKVFPTDAEEESSESFPIEL
>ID34922-NO
PIKVYASSLEGGDSSEGTFPLQA
>ID34924-NO
PIKVYPNGVEEESAESYPMEL
>ID34925-NO
PIKVYPNSFEDESVENMGPEL
>ID34927-NO
PIKVYTNGVEEESTETLPAEM
>ID34929-ABP_both
PIPFPPY
>ID34930-ABP_both
PIPRPLPFPRPGPRPIPRPLPFPRPGPRPIPRP
>ID34934-NO
PIRRRKKLRRLK
>ID34935-NO
PITHWSHGQNRWPLSCPQ

>ID34936-NO
PITNF
>ID34937-NO
PITYLDAILAAVRLLNQRISGPCILRLREAQPRPGWVGTLQRRREVSFLV
>ID34940-NO
PKAIP
>ID34941-ABP_both
PKAMRLLRRLLRLQKKG
>ID34943-NO
PKDLREN
>ID34946-NO
PKHKEMPFPPKYPVEPFT
>ID34948-NO
PKIDAMREKVLA
>ID34949-ABP_both
PKIISSPLFKTLLSAVGSALSSSGGQE
>ID34952-ABP_both
PKILNKILGKILRLAAAFK
>ID34954-NO
PKKKRKVALWKTLLKKVLKA
>ID34956-ABP_both
PKLKFLSKWIG
>ID34962-NO
PKQCSKPCKEL
>ID34965-NO
PKRKSATKGDEPARRSARLSARPVPKPAAKPKKAAAPKKAVKGKKAAENG
>ID34968-NO
PKSNFLRF
>ID34973-NO
PKVSPRWPPIPP
>ID34974-ABP_both
PKVTITIQGSARF
>ID34975-ABP_both
PKVVGLSIVVVKAKVSSALG
>ID34979-NO
PKYMDT
>ID34988-NO
PLASRPM
>ID34989-NO
PLCVNCWPQVCGDFPWGAVA
>ID34991-NO
PLDSVYGTHGMSGFA
>ID34992-NO
PLFDKRQRCCNGRRGCSSRWCRDHSRCCGRR
>ID34994-ABP_both
PLFKTLLSAVGSALSSSGGQE
>ID34995-NO
PLGDARLV
>ID34996-NO
PLGFLSQDHS
>ID34998-NO

PLGFLSQDHSVN
>ID34999-NO
PLIYP
>ID35003-ABP_both
PLPFPRPGPRPIPRP
>ID35004-ABP_both
PLPFPRPGPRPIPRPLPFPRPGPRPIPRP
>ID35009-NO
PLRIAQH
>ID35010-ABP_neg
PLSCRRKGGICILIRCPGPMRQIGTCFGRPVKCCR
>ID35011-ABP_neg
PLSCRRKIGICVLIRCSGNMRQIGTCLGALVKCCR
>ID35017-NO
PLWCA
>ID35019-NO
PMAHLEF
>ID35020-NO
PMLKE
>ID35022-NO
PMMMGFHGMR
>ID35023-NO
PMMRQRPM
>ID35025-NO
PMSGDDDDNDAMELLQ
>ID35027-NO
PMSMLRL
>ID35028-NO
PNDMLSQRYHFGL
>ID35029-ABP_neg
PNDPDSPCVYRMPNARGCSI
>ID35031-NO
PNFMRY
>ID35034-NO
PNNKPFQ
>ID35035-NO
PNPNNST
>ID35036-NO
PNRIKYGD
>ID35037-NO
PNSHP
>ID35038-NO
PNTRVRPDVSF
>ID35039-NO
PNVDPYSYLPSV
>ID35040-NO
PNWTKIGKCAGSIAWAIGSGLFGGAKLIKIKKYIAELGGLQKAAKLLVGA
>ID35041-NO
PPCPSCLSCPWCPRCLRCPMCKCNPK
>ID35042-NO
PPEIN

>ID35045-ABP_both
PPGASPRKKPRKQ
>ID35047-NO
PPHNRIQRRLNM
>ID35048-NO
PPHQIYP
>ID35050-ABP_both
PPIVGSIPLGCG
>ID35052-NO
PPKKSAQCLRYKKPE
>ID35055-NO
PPLTQTPV
>ID35059-NO
PPPPPGPPPNP
>ID35061-ABP_both
PPPPPPPP
>ID35063-ABP_both
PPPPPPPPPP
>ID35064-ABP_both
PPPPPPPPPPP
>ID35067-ABP_both
PPPPPPPPPPPPPP
>ID35068-NO
PPPRAAPAPTHV
>ID35069-NO
PPPVHL
>ID35075-NO
PPRLRKRRQLNM
>ID35079-NO
PPVTTRSKFTF
>ID35080-NO
PPYAFEVVG
>ID35081-ABP_both
PPYNLNSHTQEYSKPEDTFD
>ID35083-NO
PQAFP
>ID35085-NO
PQCCSHPACNVDHPEICD
>ID35086-NO
PQEVLP
>ID35088-NO
PQLGLPFPRV
>ID35089-NO
PQNILP
>ID35090-NO
PQNRLQIRRHSK
>ID35092-NO
PQPIFYHTTSPRL
>ID35093-NO
PQPIP
>ID35095-NO

PQPLIYP
>ID35096-ABP_both
PQPRPPHPRL
>ID35103-NO
PQRHVNY
>ID35104-NO
PQRRSARLSA
>ID35106-NO
PQTLALP
>ID35110-NO
PRCCSNPACGAGHPEICA
>ID35111-NO
PRCESQLCP
>ID35112-NO
PRCPPCPRCSWCPRCPTCPRCNCNPK
>ID35113-ABP_both
PRCRRRFCRP
>ID35114-NO
PRDYAFGL
>ID35115-ABP_both
PRFRWRIRI
>ID35123-ABP_both
PRIPPGFPPRFPPRFP
>ID35125-NO
PRKEKLCTTS
>ID35127-ABP_both
PRLKVYLPRYKVYSTAAGRYQLLSRYW
>ID35128-ABP_both
PRLKVYLPRYKVYSTAAGRYQLLSRYWDAYR
>ID35129-ABP_both
PRLPPRIPPGFPPRFPPRFP
>ID35134-NO
PRNICSRRDPTCWTTY
>ID35136-ABP_both
PRPFPGRPKPIFRPR
>ID35137-NO
PRPGAPLAGSWPGTS
>ID35138-ABP_both
PRPGPRP
>ID35140-ABP_both
PRPLPRP
>ID35141-ABP_both
PRPPHPPRPPHPPRPPHPPRPPHPRL
>ID35142-ABP_both
PRPPHPPRPPHPPRPPHPRL
>ID35143-ABP_both
PRPPHPPRPPHPRL
>ID35147-ABP_both
PRPPRLPRPRPRPLPFPRPGPRPIPRPLPFP
>ID35150-ABP_both
PRPRPRP

>ID35151-ABP_both
PRPWPRP
>ID35152-NO
PRQFV
>ID35157-NO
PRRRRSSSRP
>ID35163-NO
PRVYSFGL
>ID35172-NO
PSCAYMCIT
>ID35174-NO
PSDNFLRF
>ID35176-NO
PSFQP
>ID35177-NO
PSFQPQPLIYP
>ID35178-NO
PSGFLGMR
>ID35179-NO
PSGQYY
>ID35186-NO
PSKIKWGD
>ID35187-NO
PSKRLLHNNLRR
>ID35188-ABP_both
PSLLYKAKAVFCKPSAVAVF
>ID35189-NO
PSLRLRF
>ID35193-NO
PSPPGFSPFR
>ID35196-NO
PSSNK
>ID35197-NO
PSSSSSSRIGDP
>ID35198-NO
PSTLTSS
>ID35199-NO
PSTNHAL
>ID35200-ABP_neg
PSVQGAAAQLTADVKK
>ID35204-NO
PTEAQLQ
>ID35206-NO
PTFIAW
>ID35207-NO
PTGERLRTCERLSYP
>ID35208-NO
PTHDAW
>ID35209-NO
PTHGAW
>ID35210-NO

PTHIAW
>ID35212-NO
PTHIK
>ID35213-NO
PTHIKW
>ID35214-NO
PTHIKWD
>ID35215-NO
PTHIKWG
>ID35216-NO
PTHIKWGD
>ID35217-NO
PTHIKWN
>ID35218-NO
PTHIW
>ID35219-NO
PTHKAW
>ID35220-NO
PTHVAW
>ID35221-NO
PTKETIEQEKRSEIS
>ID35222-NO
PTPAP
>ID35224-NO
PTPVP
>ID35228-NO
PVGLVQPASATLYDY
>ID35229-ABP_both
PVGRVHRLLRK
>ID35230-ABP_both
PVIGKLASKVVPSVFSMIKR
>ID35232-NO
PVKVYANGAEEESAEAFPLEF
>ID35240-NO
PVNSGRSSGSRFNFGL
>ID35244-NO
PVPQP
>ID35245-NO
PVQALLLNQELLLNP
>ID35251-ABP_both
PVVGRVASKVFPAVIGLVKK
>ID35252-NO
PVVVPPFLQ
>ID35253-NO
PVYIPQPRPP
>ID35255-NO
PWASPSAARVFALGRDLPRAGCPLPQPS
>ID35263-NO
PWTNF
>ID35272-NO
PYDFGM

>ID35275-NO
PYDRISSSAFSDF
>ID35277-NO
PYILYEKKSIPY
>ID35278-NO
PYILYEKKVNTLLKKLILNWKSPGVEYHLAKC
>ID35281-NO
PYKWLP
>ID35283-ABP_neg
PYLPRPGRRPRFPPPFP
>ID35286-NO
PYRHP
>ID35287-NO
PYRHWSYGLRPG
>ID35288-NO
PYRLYENKPRRPWIL
>ID35289-NO
PYRPLPDCCRQKTCSCRLYELLHGAGNHAAGILTL
>ID35290-ABP_both
PYSLKNGENWLLSEEIIRYP
>ID35294-NO
QAASRVENYMHR
>ID35295-NO
QACPMLLCM
>ID35296-NO
QADFDDPRMFTSSF
>ID35297-NO
QAEATRQAAAQEERLADLASDLLLQYLLQGGARQRDLG
>ID35308-NO
QAGLW
>ID35311-NO
QANQDFMRF
>ID35312-NO
QAPRFF
>ID35313-NO
QAPRFI
>ID35315-NO
QAPWPDTISPP
>ID35320-NO
QARPPHPPIPP
>ID35341-NO
QAVLPPQHLCGAHLVDALYLVCGERGFFYTP
>ID35342-NO
QCADLGEECHTRFCCPGLRCEDLQVPTCLMA
>ID35345-NO
QCCDRNSCEYPKCLCCN
>ID35347-NO
QCCDWPWCDDCICCD
>ID35348-NO
QCCDWQWCDGACDCCA
>ID35349-NO

QCCGWEWCDDICGCCE
>ID35352-NO
QCCPTMPECCRI
>ID35354-NO
QCCRPANMSCCQ
>ID35355-NO
QCCTGSCLNCWPCC
>ID35357-NO
QCCWYFDISCCLWP
>ID35358-NO
QCEDVWMPCTSNWECCSLDCEMYCTQI
>ID35362-NO
QCGQAWC
>ID35365-NO
QCLPPLHWCNMVDDECCHFCVLLACV
>ID35366-NO
QCLPPLSLCNMADDDCCNDCVLFLCSYY
>ID35367-NO
QCLPPLSLCTMDDDECCDDCILFLCLVTS
>ID35369-NO
QCPPWCSGEPCRKGTC
>ID35374-NO
QCSPNGGSCSRHYHCCSLWCNKDSGVCVATSYP
>ID35378-NO
QCTGRF
>ID35380-NO
QCTPHGGSCGLVSTCCGRCSVPRNKCE
>ID35387-NO
QCTPVGGSCSRHYHCCSLYCNKNIGQCLATSYP
>ID35389-NO
QCTPVGGYCSRHHHCCSNHCIKSIGRCVAH
>ID35390-NO
QCTPVGGYCSRHYHCCSNHCIKSIGRCVAH
>ID35393-ABP_both
QCTYSVTPTVKSFELYFKGRMSCP
>ID35400-NO
QDCAAGGQFCGFPKIGGPCCSGWCLGVCA
>ID35403-NO
QDCSSVVCVLGDGACNRVCEMEGHTEGGKCVPRDGCPAGSEICVCGAKKA
>ID35404-NO
QDDDYGHMRF
>ID35407-NO
QDFMKHLDKKTQTPKL
>ID35409-NO
QDGLLNNCR
>ID35411-NO
QDGPIPP
>ID35413-NO
QDIDHVFMRF
>ID35415-NO
QDKCKKVYENYPVSKCQLANQCNYDCKLDKHARSGECFYDEKRNLQCICD

>ID35417-NO
QDLDHVFLRF
>ID35419-NO
QDLDIGMW
>ID35421-NO
QDLQTLCCTDGCSMTDLSALC
>ID35422-ABP_both
QDNSRYTHFLTQHYDAKPQGRDDRYSESIMRRRGLTSPSKDINTFIH
>ID35423-ABP_both
QDNYWVKQGLNKLSK
>ID35424-ABP_both
QDNYWVTQGLNILSG
>ID35425-NO
QDPFLRF
>ID35426-NO
QDPFLRI
>ID35437-NO
QDSGDEWPQQPFVPRL
>ID35441-NO
QDVRQQLRMFDDLVQLRKLIETPSVYPSEEDEARLYD
>ID35442-NO
QDVVHSFLRF
>ID35443-NO
QEADPSSSLEADSTLKDEPRELSNM
>ID35445-NO
QECCSYPACNLDHPELC
>ID35451-NO
QEDGEIVCGEDDPCGTQICECDKAAAICFRNSMDT
>ID35454-NO
QEFSPNLWGLEFQKN
>ID35455-NO
QEFSPYMGLEFKKH
>ID35457-ABP_both
QEKDRTFAGFLLKGFGTSAS
>ID35459-ABP_pos
QEKKKKKTGRAKRR
>ID35464-NO
QELHVPEREA
>ID35466-ABP_both
QELLLNPTHQYPVTQPLAPVHNPISV
>ID35469-NO
QEMCRDLLMRAKNCDDSTCATLCKQKWKGNGSCFPNVYRKSCLCTFPCKT
>ID35470-NO
QEPAYQRFL
>ID35473-NO
QERRAMGFVGMR
>ID35474-NO
QESKKGILLKPKTCNTNADCAKFCKGPIQNCLYHTCACVPGNPHCC
>ID35475-NO
QETCHDLIMKRDCDEATCVNMCQQKWKGSGGSCFQNFNVMSCICNFPCQV
>ID35476-NO

QETTFTPRL
>ID35478-NO
QEVLP
>ID35480-NO
QEYSPNLWGHEFRSH
>ID35484-NO
QFDEPR
>ID35485-NO
QFDEYGHMRF
>ID35486-NO
QFNEYGHMRF
>ID35487-NO
QFPPKLTNNSML
>ID35489-NO
QFQSQPM
>ID35493-NO
QFRPSYQIPP
>ID35495-NO
QFSESLPEECCKYGCPRYYLLMYC
>ID35496-NO
QFSPENCQGESQPC
>ID35497-NO
QFTDVKCTGSKQCWPVCKQMFGKPNGKCMNGKCRCYS
>ID35498-NO
QFTNVSCSASSQCWPVCKKLFGTYRGKCMNSKCRCYS
>ID35502-NO
QFYRF
>ID35505-NO
QGCCGEPNLCFTRWCRNNARCCRQQ
>ID35506-NO
QGCCNVPNGCSGRWCRDHAQCC
>ID35508-NO
QGCCSYPACAVSNPDICGG
>ID35509-NO
QGCKGPYTRPILRPYVRPVVSYNACTLSCRGITTTQARSCCTRLGRCCHV
>ID35511-ABP_both
QGDGNFVIYTWKP
>ID35512-NO
QGGAGWPPIPP
>ID35513-NO
QGGAPWNPIPP
>ID35515-NO
QGGLPRPGPEIPP
>ID35517-NO
QGGWPRNPIPP
>ID35522-NO
QGLIAFPRV
>ID35523-NO
QGLIPFPRV
>ID35524-NO
QGLISFPRV

>ID35525-NO
QGLPPRPKIPP
>ID35526-NO
QGLVPFPRV
>ID35527-NO
QGNFLRF
>ID35529-NO
QGPSPRHPIPP
>ID35530-NO
QGPWLEEEEAAYGWMDF
>ID35531-NO
QGPWLEKEEAAYGWMDF
>ID35537-NO
QGRFG
>ID35540-NO
QGRPPGPPIPP
>ID35543-NO
QGSLFRF
>ID35544-ABP_both
QGSPARCRFCCRCCPRMRGCGICCRF
>ID35545-NO
QGTTNIVCECCMKPCTLSELRQYCP
>ID35548-NO
QGWAWPRPQIPP
>ID35550-NO
QGWCCKENIACCV
>ID35551-NO
QGWPGPKVPP
>ID35552-NO
QGYKGPYTRPILRPYVRPVVSYNACTLSCRGITTTQARSCCTRLGRCCHV
>ID35556-NO
QHAPSNSKSVLT
>ID35557-NO
QHEAT
>ID35558-NO
QHGHGGQDQHGYGHGQQAVYGKGHEGHGVNNLGQDGHGQHGYAHGHSDQH
>ID35559-NO
QHGKISSEQHTMFDPIEGCCQQSCTTCFPC
>ID35560-NO
QHGLW
>ID35563-NO
QHICHQILLNNNCDGATCTSLCDKQLQGTGQCYKTVDKRFICLCNYLCRT
>ID35567-NO
QHPGLW
>ID35568-ABP_both
QHSSGKSDVRRW
>ID35571-NO
QHWSFGLSPG
>ID35572-NO
QHWSHGWFPG
>ID35577-NO

QHWSKGYSPG
>ID35578-NO
QHWSNWWIPGAPGYNG
>ID35580-NO
QHWSYEYMPG
>ID35592-NO
QHWSYGLWPG
>ID35593-NO
QHWSYGWLPG
>ID35594-NO
QHWSYKCIRP
>ID35598-NO
QIDPLGFSGGI
>ID35599-NO
QIDRDPCCSYPDCGANHPEICGGKR
>ID35600-NO
QIDTNVKCSGSSKCVKICIDRYNTRGAKCINGRCTCYP
>ID35610-NO
QIKIWFQNRRMKWKK
>ID35614-NO
QILRG
>ID35615-NO
QILTGIKCPDPNGHDKEDKCNIYCLNQNYMGGSCQGYKNHYMCECYVG
>ID35618-NO
QINCCPWPCPDSCHYQCCH
>ID35619-NO
QIPGLGPLR
>ID35620-NO
QIPMLRL
>ID35631-NO
QITQFTPRL
>ID35632-NO
QIVDCWETWSRCTKWSQGGTGTLWKSCNDRCKELGRKRGQCEEKPSRCPL
>ID35638-NO
QKAVPYPQRDMPI
>ID35639-NO
QKCCGEGSSCPKYFKNNFICGCC
>ID35640-NO
QKCCGKGMTCPRYFRDNFICGCC
>ID35641-NO
QKCCTGKKGSCSGRACKNLRCCA
>ID35646-ABP_both
QKDDEEESRFFFNFIFSAE
>ID35649-NO
QKDLVVTATTTCCGYNPMTMCPPCMCTNTC
>ID35650-NO
QKECIGPCDMFTDCQAACVGIRKGYNYGQCVAWKPKDDDPFTCCCYKLTP
>ID35651-NO
QKECTGPQHCTNFCRKNKCTHGKCMNRKCKCFNCK
>ID35653-NO
QKELVPSKTTTCCGYSPGTMCPSCMCTNTCPPQK

>ID35666-NO
QKGMY
>ID35670-NO
QKIGLW
>ID35673-NO
QKLCARPSGTWSSGNCRNNNACRNFCIKLEKSRHGSCNIPFPSNKCICYF
>ID35676-NO
QKLCEKPSGTWSGVCGNSNACKNQCINLEGAKHGSCNYVFPAHKCICYFP
>ID35677-NO
QKLCEKPSGTWSGVCGNSNACKNQCINLEGAKHGSCNYVFPAHKCICYVP
>ID35686-NO
QKLCERPSGTWSGVCGNNNACKNQCINLEKARHGSCNYVFPAHKCICYFP
>ID35727-NO
QKPDDVIKACGRELARLRIEICGSLSWK
>ID35728-NO
QKPRN
>ID35737-NO
QKTAP
>ID35738-ABP_both
QKVFTNTWAVRIPGGPAVANSVARKHGFLNLQGIF
>ID35742-NO
QLAFRPML
>ID35745-NO
QLASDDYGHMRF
>ID35746-NO
QLDMTVSEKCCQVGCTRRFIANSC
>ID35747-NO
QLDPMLFSGRL
>ID35749-ABP_pos
QLEARFEPKQRNFRKRELDFEKLFANMPDY
>ID35751-NO
QLGFLGPR
>ID35752-NO
QLGLQGPPHLVADLAKKQGPWMEEEEEAYGWMDF
>ID35754-NO
QLGLQGPPQQVADLSKKQGPWLEEEEAAYGWMDF
>ID35755-NO
QLGLQGPQHFIADLSKKERPRMEEEEEAYGWMDF
>ID35756-NO
QLGLQGSPHLVADLSKKQGPWLEKEEAAYGWMDF
>ID35757-NO
QLGLW
>ID35762-NO
QLHVPSIL
>ID35765-ABP_both
QLKVDLWGTR
>ID35766-ABP_both
QLKVDLWGTRSGI
>ID35767-NO
QLLAERH
>ID35768-NO

QLLGGRF
>ID35769-NO
QLLKLK
>ID35771-NO
QLNFSAGW
>ID35772-NO
QLNFSAGWGRRYADPNADPMAFLYRLIQIEARKLAGCSD
>ID35773-NO
QLNFSPGW
>ID35774-NO
QLNFSPNW
>ID35777-NO
QLNFSTGWGRRYADPNADPMAFLYKLIQIEARKLAGCSN
>ID35779-NO
QLNFTPNWGTGKRDAADFADPYSFLYRLIQAEARKMSGCSN
>ID35780-NO
QLNFTPNWGTGKRDAADFGDPYSFLYRLIQAEARKMSGCSN
>ID35784-NO
QLNSCIHSGDRAIRGCMDWV
>ID35785-NO
QLNYSPDW
>ID35790-NO
QLPVM
>ID35793-NO
QLQSNGEPAYRVRTPRL
>ID35794-NO
QLSHKCCYWGCTRKELARQC
>ID35797-NO
QLTFSPDW
>ID35798-NO
QLTFSPDWGK
>ID35801-NO
QLTFSSGWGNCTS
>ID35803-NO
QLTFTPGWGY
>ID35806-NO
QLTFTPSW
>ID35807-NO
QLTFTSSWG
>ID35808-NO
QLTFTSSWGGKRAMTNSISCRNDEAIAAIYKAIQNEAERFIMCQKN
>ID35810-NO
QLVSFRPRL
>ID35811-NO
QLWAVGSFM
>ID35814-NO
QLYENKPRRPYILKRGSYYY
>ID35815-NO
QLYMTLSNKCCHIGCTKKSLAKFC
>ID35816-NO
QMFHLWYLRHMKNKKPMA

>ID35820-NO
QMIVIELGTNPLK
>ID35823-NO
QMNDCDCDHDHRCGEWEDESHGNCKQHHLRVVCTCTLDCLDISSTSNA
>ID35829-NO
QNALIVRYTR
>ID35830-NO
QNCCNGGCSSKWCKGHARCC
>ID35834-NO
QNCPNIFGQWM
>ID35835-NO
QNDPRPNIFGQWM
>ID35836-NO
QNEGPGRDPAPCCQHPIETCC
>ID35838-NO
QNHSEPNIFGQWM
>ID35841-NO
QNILP
>ID35844-NO
QNLLRF
>ID35847-NO
QNPGLW
>ID35848-NO
QNPRGRKCEDPNGVDQKAKCYIYCNEQGFLGGSCQGYTNHYMCECYVG
>ID35850-NO
QNRRMKWKK
>ID35852-NO
QNSAAAFAQWA
>ID35853-NO
QNSAAAFGAWA
>ID35854-NO
QNSAAAFGAWM
>ID35856-NO
QNSAAIFAAWA
>ID35857-NO
QNSAAIFGAWA
>ID35858-NO
QNSANAFAAWA
>ID35859-NO
QNSANAFGAWA
>ID35860-NO
QNSPAAFAAWA
>ID35861-NO
QNSPAAFGAWA
>ID35862-NO
QNSPAAFGAWM
>ID35864-NO
QNSPAIFGAWA
>ID35865-NO
QNSPAIFGAWM
>ID35867-NO

QNSPNAFGAWA
>ID35868-NO
QNSPNIFGAWA
>ID35869-NO
QNSPNIFGQFM
>ID35871-NO
QNSPWIFGAWA
>ID35872-NO
QNWPRPQIPP
>ID35873-NO
QNYHFSNGWYAG
>ID35874-NO
QNYLAFPRM
>ID35878-NO
QPDDYGHMRY
>ID35880-ABP_pos
QPEATKCFQWQRNMRKVR
>ID35881-NO
QPENLPT
>ID35882-ABP_both
QPEWFKARRWQWRMKKLGA
>ID35884-NO
QPGYSHSFV
>ID35892-NO
QPLIYP
>ID35898-NO
QPPLPRY
>ID35899-NO
QPPMEYS
>ID35902-NO
QPQCRWLDGFCHSSPCPSGTTSIGQQDCLWYESCCIPRYEK
>ID35903-NO
QPQPLIYP
>ID35904-NO
QPQPVFYHSTTPRL
>ID35905-NO
QPQSHSIELDEVSKEAASTRAALTSNL
>ID35911-NO
QPSACNINDRPHRRGVCGSALADLVDPACSSSNGPA
>ID35912-NO
QPSFGHSFV
>ID35913-NO
QPSFTHAFV
>ID35914-NO
QPSQDFMRF
>ID35915-NO
QPSYGHSFV
>ID35916-NO
QPSYTHAFV
>ID35924-NO
QPVVPVEAVDPMEQ

>ID35925-NO
QPWGTCSESCGKGTQTRAR
>ID35926-NO
QPWLEQAYYSTF
>ID35927-NO
QPWLVPSKITNCCGYNNMEMCPTCMCTYSCRP
>ID35930-NO
QPWSQCSATCGDGVRERRR
>ID35931-NO
QQCCPPVACNMGCEPCC
>ID35933-NO
QQDSEVEREMM
>ID35940-NO
QQFDDYGHLRF
>ID35942-NO
QQHLLIAINGYPRYN
>ID35948-NO
QQLLGTGRVQGYDGYFVLSVEQYLELSDSANNIHFMRQSEI
>ID35950-NO
QQMNQKDFLSLIVS
>ID35952-NO
QQPGLW
>ID35953-NO
QQPQAVHTYCGRHLARTLADLCWEAGVD
>ID35964-ABP_both
QQRFEWEFEQQ
>ID35967-NO
QQWPPGHHIPP
>ID35968-NO
QQWPRDPAPIPP
>ID35979-ABP_both
QREPQVQWLEQQVAKRRTKR
>ID35980-ABP_both
QRFSQPTFKLPQGRLTLSRKFR
>ID35982-NO
QRGMI
>ID35986-ABP_both
QRKKWFW
>ID35989-NO
QRPPSLKTRF
>ID35997-NO
QRSPMMSRIRLP
>ID36022-NO
QSADRSV
>ID36025-NO
QSAPGNEAIPP
>ID36027-NO
QSCCATPSCAKLY
>ID36028-NO
QSCCATPSCARLYEKVY
>ID36029-NO

QSCCSAPLCALLYRVMC
>ID36030-NO
QSCCSTPPCALLYMEMC
>ID36032-NO
QSDDYGHMRF
>ID36036-NO
QSEGTFSNYYSKYQEERMARDFLHWLMNS
>ID36040-NO
QSHLSLCRFCCKCCRNKGCGYCCKF
>ID36041-ABP_both
QSHLSLCRWCCNCCHNKGCGFCCKF
>ID36053-NO
QSLVYP
>ID36054-NO
QSPGCCWNPACVKNRC
>ID36057-NO
QSPLSQSSHEFTVVSPYLSCFGIEECLFYLYFKLYDLCVILLCTWFDLSE
>ID36058-NO
QSPTDFTFPNPL
>ID36060-NO
QSRDFSISEREIVASLAKQLLRVARMGYVPEGDLPR
>ID36061-NO
QSRIGLW
>ID36062-NO
QSRLSLG
>ID36063-NO
QSRPSIVCECCFNQCTVQELLAYC
>ID36066-NO
QSSFHSWG
>ID36071-NO
QSWLVPSTITTCCGYSPGTMCPPCMCTNTC
>ID36072-NO
QSWMHQPHQPLPPTVM
>ID36078-NO
QTCCGYRMCVPC
>ID36081-NO
QTEDDDKFVRLS
>ID36084-NO
QTFFTNGRY
>ID36085-NO
QTFHYSQGWTN
>ID36086-NO
QTFQYSRGWTN
>ID36089-NO
QTFTYSHGWTN
>ID36097-NO
QTQCQSVRDCQQYCLTPDRCSYGTCYCKTT
>ID36099-NO
QTQSLVYP
>ID36100-NO
QTQYTDAPSFSDIPNPIGSENSEKTTMPLW

>ID36101-NO
QTRRRERRAEKQAQW
>ID36102-NO
QTSFMAPSWALGHLM
>ID36107-NO
QVAQMHIWRAVNHDRHHSTGSGRHSRFLTRNRYRYGGGHLSDA
>ID36110-NO
QVDSLGGFQVHGW
>ID36111-NO
QVETNVKCQGGSCASVCRKAIGVAAGKCINGRCVC
>ID36119-NO
QVKPCRKEHQLCDLIFQNCCRGWYCLLRPCI
>ID36120-NO
QVKPCRKEHQLCDLIFQNCCRGWYCVVLSCT
>ID36126-NO
QVNFSPGW
>ID36127-NO
QVNFSPGWG
>ID36128-NO
QVNFSPGWGT
>ID36130-NO
QVNFSPNW
>ID36131-NO
QVNFSTGW
>ID36132-NO
QVNFSTSW
>ID36133-NO
QVNFTPGW
>ID36134-NO
QVNFTPNWGT
>ID36139-NO
QVPQPIP
>ID36143-NO
QVSLKYPEGKMYSFGL
>ID36144-NO
QVSLNSGYY
>ID36145-NO
QVSWWCGKPEATCGKLYLKCCSGMCNKANWKCL
>ID36146-NO
QVTFSKGWGP
>ID36147-NO
QVTFSRDWNA
>ID36148-NO
QVTFSRDWSP
>ID36152-NO
QVTSTEV
>ID36155-NO
QVYKGGYTRPIPRPPFVRPVPGGPIGPYNGCPVSCRGISFSQARSCCSRL
>ID36156-NO
QVYKGGYTRPIPRPPPFVRPLPGGPIGPYNGCPVSCRGISFSQARSCCSR
>ID36160-NO

QWAAILGAGWN
>ID36161-NO
QWAQWPRPQIPP
>ID36162-NO
QWAQWPRPTPQIPP
>ID36164-NO
QWCSRRWCT
>ID36165-NO
QWGQHPNIPP
>ID36167-NO
QWGYGGY
>ID36168-NO
QWGYGGYGRGYGGYGGYGRGYGGYGGYGRGYGGYGRGMYGGYGRPYGGYG
>ID36172-NO
QWLGGRFG
>ID36173-NO
QWLKGRF
>ID36174-NO
QWLYSMFGL
>ID36175-NO
QWPDPSSDIPP
>ID36176-NO
QWPFQQWAPCTGHWDCPGDRCCFAGYCLETTPSCD
>ID36177-NO
QWPRPQIPP
>ID36178-NO
QWPRPTPQIPP
>ID36180-NO
QWQRNMRKVRGPPVSCIKR
>ID36186-NO
QYDGRGSDMVEGPRVERMHPETSGGCVGAHCLTQNSEGPVGAMWFGPRL
>ID36198-ABP_both
QYRPGSFGPLNQK
>ID36199-NO
QYTSELEEDE
>ID36200-NO
QYWSYGVRPG
>ID36202-NO
QYYYGASPYAYSGGYYDSPYSY
>ID36206-ABP_both
RAAPQRRLRAMARLKKFAEAGGADPDSGGLRARFPER
>ID36209-NO
RADHP
>ID36210-NO
RADHPF
>ID36211-NO
RADHPFL
>ID36212-NO
RADHRYNDLGHR
>ID36217-ABP_both
RAGLQFLVGRVHRLLRK

>ID36218-ABP_both
RAGLQFPVGGIGKFLHSAKKFGK
>ID36219-ABP_both
RAGLQFPVGRVHRL
>ID36221-ABP_both
RAGLQFPVGRVHRLLR
>ID36222-NO
RAGSPSGGPFCALARQPLTGSPPNERAFFCSSRDV
>ID36226-NO
RAIKIWFQNRRMKWKK
>ID36232-ABP_pos
RAKRRMQY
>ID36233-NO
RAKRRQRRR
>ID36234-NO
RAKWFW
>ID36235-NO
RALDQNLLVDEHLMRF
>ID36238-NO
RARAPHKAWYNCMTDAGISGAIAGAVAGCAATIEIGCVEGAIAGIGPSGI
>ID36241-NO
RARPRF
>ID36243-NO
RASDVGSDVVPRYPF
>ID36251-NO
RAWMRWYSPTTRRYG
>ID36253-ABP_both
RAWVAWRNR
>ID36257-NO
RCAHGTYYSNDSQQCLLNCCWWGGGDHCCR
>ID36259-NO
RCCGEGASCPRYFRNSQICSCC
>ID36260-NO
RCCGEGASCPVYSRDRLICSCC
>ID36262-NO
RCCGYKMCHPC
>ID36265-NO
RCCHPACGQQTSC
>ID36266-NO
RCCISPACHDDCICCIT
>ID36268-NO
RCCISPACHEECYCCQ
>ID36270-NO
RCCISPACNDTCYCCQD
>ID36271-NO
RCCIWPECGSCVCCL
>ID36273-NO
RCCNWQECDGNCHCCQ
>ID36274-NO
RCCPASACNGACGCCE
>ID36276-NO

RCCPMEWCDGDCFCCV
>ID36281-NO
RCCRWPCPRKIDGEYCGCCL
>ID36282-NO
RCCSESHCNAGCACCD
>ID36287-NO
RCCTWQECDGNCRCCQ
>ID36289-NO
RCCVHPACHDDCICCIT
>ID36290-NO
RCDEEGTGCSSDSECCSGRCTPEGLFEFCE
>ID36291-ABP_both
RCFRRRGKLTC
>ID36294-NO
RCHFVVCTTDCRRNSPGTYGECVKKEKGKECVCKS
>ID36297-ABP_both
RCICTRGFCRLL
>ID36302-ABP_both
RCLCRRGVCQLL
>ID36304-NO
RCLPAGKPCAGVTQKIPCCGKSRNKCTT
>ID36309-ABP_both
RCPGHTRQIGTSFGPRVKSCRKW
>ID36310-ABP_both
RCPGRTRQIGTIFGPRIKCRSW
>ID36313-NO
RCRLAERRQIAK
>ID36314-NO
RCRSCVPFCGSNERMISTCFSGGVVCCPR
>ID36315-ABP_pos
RCRVYNNGLPTGLYRWC
>ID36316-NO
RCSDDTGATCSDNSDCCGDMCCLSNTCVVTIVACS
>ID36317-NO
RCSDDTGATCSNRFDCCESMCCIGGHCVISTVGCP
>ID36321-ABP_both
RCYNGCCRRGYYGGCRCCAHPDEIPDPEYRAEPAYGHP
>ID36322-NO
RCYTNDDCKDGQPCPVPLACLFGSCICPWKSQSKLPICQIICANLD
>ID36328-NO
RDCCTPPKKCKDRRCKPLKCCA
>ID36333-ABP_both
RDCESDSHKFHGACFSDTNCANVCQTEGFAGKCVGVQRHCHCTKDC
>ID36336-NO
RDDFR
>ID36338-NO
RDFTHTIIDNSDLFSESRNTRLG
>ID36340-NO
RDLPG
>ID36347-NO
RDTMRCMVGRVYRPCWEV

>ID36367-NO
RECKTESHRFKGPCITKPPCRKACISEKFTDGHCSKILRRCLCTKPC
>ID36368-NO
RECKTESNTFPGICITKPPCRKACISEKFSGGDCSKILRRCLCTKPC
>ID36374-NO
REIILPALRLQTSSDCLGFPDVWC
>ID36379-NO
REKVLASS
>ID36382-NO
RELEE
>ID36383-NO
RELEEL
>ID36388-NO
RENSLPAGLSPLR
>ID36390-NO
REQEEL
>ID36391-NO
RESPSSRMECYEQAERYGYGGYGGGRYGGGYGSGRGQPVGQGVERSHDDN
>ID36429-NO
RFLSKFKDIALDVAKNAGKGVLTTLACKIDGSC
>ID36431-ABP_both
RFPGLMMKLLV
>ID36432-ABP_both
RFPKLMMKLLV
>ID36435-NO
RFRGLISLSQVYLSP
>ID36442-ABP_both
RFRPPIRRPPIRPPFRPPFRPPVR
>ID36446-ABP_both
RFRRLRDKTRDRLKKI
>ID36448-ABP_both
RFRRLRKKFRKRLKKI
>ID36449-ABP_both
RFRRLRKKIRKRLKKI
>ID36450-ABP_both
RFRRLRKKKRKRLKKI
>ID36453-ABP_both
RFRRLRKWTRWRLKKI
>ID36454-ABP_both
RFRRLRPKTRPRLKKI
>ID36455-ABP_both
RFRRLRWKTRKRLWKI
>ID36456-ABP_both
RFRRLRWKTRWRLKKI
>ID36457-ABP_both
RFRRLRWRTRWRLRRI
>ID36458-ABP_both
RFRRLRWWTRKRLKKI
>ID36478-ABP_neg
RFWWFRRR
>ID36482-NO

RGADEDDGVEITEEEVKRGLMDTVKNAAKNLAGQLLDRLKCKITGC
>ID36483-NO
RGARRR
>ID36485-NO
RGCCSHPVCNLNNPQMCR
>ID36486-NO
RGCCSYFDCRMMFPEMCGWR
>ID36488-ABP_both
RGCVYAYVRVRGVLVRYRRCW
>ID36489-NO
RGCYTRCWKVGRNGRVCMRVCT
>ID36491-NO
RGDFK
>ID36492-NO
RGDGWK
>ID36494-NO
RGDPAYNGRFL
>ID36495-NO
RGDPAYQGRFL
>ID36496-NO
RGDPAYQRFL
>ID36497-ABP_both
RGDWLWLW
>ID36500-NO
RGEPAYQRFL
>ID36501-NO
RGFARR
>ID36505-NO
RGFRRR
>ID36506-NO
RGFTKMPHVQIHTEASESL
>ID36508-ABP_both
RGGCLCYCRRRFCVCVCR
>ID36511-NO
RGGLSGIVQQQNNLLRAIEAQQHLLQ
>ID36512-ABP_both
RGGRAAVLNA
>ID36516-NO
RGGRLSYSRRRFSTSTGR
>ID36525-NO
RGLERA
>ID36530-ABP_both
RGLRRLGRKIAHGVKKYGPTVLRIIRIAGGGGGSC
>ID36532-ABP_both
RGLRRLGRKIAHGVKKYGPTVLRIIRIAGKKTWWKTWWTKWSQPKKKRKV
>ID36534-NO
RGLWSKIKEAGKAALTAAGKAALGAVSDAV
>ID36538-NO
RGPFPIIV
>ID36541-NO
RGPYAFGL

>ID36544-ABP_both
RGRGRGRGRG
>ID36553-ABP_neg
RGRKGGRRKK
>ID36583-NO
RGSRRAVTRAQRRDGRRRRRSRRESYSVYVYRVLRQ
>ID36587-ABP_both
RGVAKFASKGLGKDLAKLGVDLVACKISKQC
>ID36592-ABP_both
RGVRPVVQGVKEKVR
>ID36593-ABP_both
RGVRPVYIPQPRPPHPRL
>ID36604-NO
RHCFSQWCS
>ID36623-NO
RHIKIWFQNRRMKWKK
>ID36626-ABP_both
RHRHRH
>ID36627-ABP_both
RHRHRHRH
>ID36633-ABP_both
RHSRLQREPQVQWLEQQVAKRRTKR
>ID36634-NO
RHTVCRVSLSSVQGSCSHEY
>ID36639-ABP_both
RHWRRFWH
>ID36640-ABP_both
RHWRRFWHR
>ID36649-NO
RICCYPNVWCCD
>ID36655-ABP_neg
RICRTRLTRRAGNSL
>ID36656-NO
RICSRDKNCVSRPGVGSIIGRPGGGSLIGRPGGGSVIGRPGGGSPPGGGS
>ID36657-ABP_both
RICSRGKNCVSRPGVGSIIGRPGGGSLIGRPGGGSV
>ID36668-NO
RIFGESVSLRVQDWEW
>ID36669-NO
RIFIGC
>ID36670-NO
RIFIHFRIGC
>ID36671-NO
RIFIRIGC
>ID36674-NO
RIGLF
>ID36676-NO
RIGSFLGALASKLPTLISWIKNR
>ID36681-ABP_both
RIGSILGRLAKGLPTLISWIKNR
>ID36687-ABP_both

RIIDLLARVRRPQKPKFVTVWVR
>ID36688-ABP_both
RIIDLLWRVRRPQKPKFVTVAVR
>ID36689-NO
RIIDLLWRVRRPQWPKFVTVWVR
>ID36694-NO
RIIDLLWRVWRPWWPKFVTVWVR
>ID36696-ABP_both
RIIRPIIQIIKQKIR
>ID36698-NO
RIITCSCRTFCFLGERISGRCYQSVFIYRLCCRG
>ID36700-NO
RIKAERKRMRNRIAASKSRKRKLERIARGC
>ID36702-NO
RIKIGLFDQLSKL
>ID36713-NO
RILQQLLFIHF
>ID36715-NO
RILQQLLFIHFRIGCRH
>ID36716-NO
RILQQLLFIHFRIGCRHSRI
>ID36718-NO
RILRG
>ID36719-NO
RILSILRHQNLLKELQDLA
>ID36726-ABP_neg
RINKK
>ID36728-NO
RINNDCQNFIGNR
>ID36732-NO
RIPLEM
>ID36733-NO
RIPTSTGFF
>ID36739-ABP_both
RIRDAIAHGYIVDKV
>ID36740-NO
RIRFH
>ID36741-NO
RIRFN
>ID36749-NO
RIRMIQNLIKKT
>ID36752-NO
RIRTWKSLVKHHM
>ID36757-NO
RISFKKGKGSWIKNGIIKGIKGIGKEIGMDVLRTGIDIAGCKIKGEC
>ID36764-ABP_both
RIVFAVLSIVNRVRQ
>ID36766-ABP_both
RIVQRIAKWAKKWYKAGK
>ID36767-ABP_both
RIVQRIKKWLLKWKKLGY

>ID36768-ABP_both
RIVQRILKWLKKWYKLGK
>ID36787-ABP_both
RIWKIWWKR
>ID36788-ABP_both
RIWKRWWFR
>ID36886-NO
RKARRQRRR
>ID36888-NO
RKAWFW
>ID36889-NO
RKCEVPGCQ
>ID36899-ABP_both
RKHGFLNLQGIFGDYYHFWHRGV
>ID36905-NO
RKKAFW
>ID36906-NO
RKKARQRRR
>ID36924-NO
RKKRAQRRR
>ID36925-NO
RKKRRARRR
>ID36926-NO
RKKRRQARR
>ID36927-NO
RKKRRQR
>ID36928-NO
RKKRRQRAR
>ID36929-NO
RKKRRQRR
>ID36930-NO
RKKRRQRRA
>ID36932-ABP_both
RKKRRQRRRLNLKALLAVAKKIL
>ID36933-NO
RKKRRQRRRPPQCAAVALLPAVLLALLAP
>ID36934-NO
RKKRRQRRRRKKRRQRRR
>ID36936-ABP_both
RKKTRKR
>ID36937-ABP_both
RKKTRKRLKKIGKVLKWI
>ID36938-NO
RKKWAW
>ID36939-NO
RKKWFA
>ID36941-ABP_both
RKLILKRKRILIKR
>ID36942-ABP_both
RKLKHMRF
>ID36951-NO

RKPHPKEFVGLM
>ID36952-NO
RKPPFNGSIF
>ID36953-NO
RKPWLL
>ID36955-NO
RKRHPDCDKAADTRI
>ID36957-ABP_both
RKRIHIGPGRAFYTT
>ID36961-ABP_both
RKRKLILILIKRKR
>ID36963-ABP_both
RKRKSDVDFEAEFELFEDDD
>ID36975-ABP_both
RKRWWWWFR
>ID36979-ABP_both
RKSKEKIGKEFKRIVQRIKDF
>ID36988-ABP_both
RKVAPALIKSFVFLFKFKKG
>ID37002-ABP_both
RKWVAWRNR
>ID37004-ABP_both
RKWVKWRNR
>ID37005-ABP_both
RKWVWWRNR
>ID37008-ABP_both
RKWVYWRNR
>ID37009-ABP_both
RKWWRWIKW
>ID37010-ABP_both
RKWWRWWKK
>ID37017-ABP_both
RKYVRFLHRWVKYFRAYL
>ID37032-NO
RLAMRWYSPTTRRYG
>ID37201-NO
RLCRVRGTRGHCFNDHGCDKVCTREGFVRGKCNGILRRCICDRQC
>ID37202-NO
RLCSLYGCV
>ID37214-NO
RLFKCYGPNSRGFQICE
>ID37215-NO
RLFKCYKPDS
>ID37216-NO
RLFMRFYSPTTRRYG
>ID37222-ABP_both
RLGGILRKAGEKIGGGLKKIGQKIKDFFGKLAPRTES
>ID37228-NO
RLHQNGMPFSPRL
>ID37243-ABP_both
RLKKHLKKIK

>ID37246-ABP_both
RLKKWMQKVIDRFGG
>ID37249-ABP_pos
RLKLLLLRL
>ID37250-ABP_pos
RLKLLLRL
>ID37251-ABP_pos
RLKLLRL
>ID37253-NO
RLKRMTPFWRGVSLRPVGASCRDNSECITMLCRKNRCFLRTASE
>ID37255-NO
RLLDTNRPLLPY
>ID37256-ABP_both
RLLFRKIRRLKR
>ID37275-ABP_neg
RLLLVMIGLRSKIKWHSGI
>ID37276-NO
RLLMRLYSPTTRRYG
>ID37280-NO
RLLRLLRLRRLLRL
>ID37281-NO
RLLRLLRRLLRLLR
>ID37283-ABP_both
RLLRPLLQLLKQKLR
>ID37286-ABP_both
RLLRRLLRRLLRLQKKGI
>ID37292-NO
RLLSLIRKLIT
>ID37302-NO
RLPSEFDLSAFLRA
>ID37303-NO
RLPSYGHSFL
>ID37306-NO
RLQLKL
>ID37310-NO
RLRFD
>ID37311-NO
RLRFDRRDQDEGNFRRFPTNAVSMSADENSPFDLSNEDGAVYQRDL
>ID37313-NO
RLRFH
>ID37314-NO
RLRFN
>ID37315-ABP_both
RLRKRLRKFRNKIKEKLKKIGQKIQGFVPKLQPSKQS
>ID37316-ABP_both
RLRLLLLLRLR
>ID37321-NO
RLSEDMPATPADQEMYQPDPEEMESRTRYFSPRL
>ID37322-NO
RLSGMNEVLSFRWL
>ID37323-NO

RLSGQTIEVTSEYLFRH
>ID37327-ABP_both
RLTYKPRTVTYTRGR
>ID37335-NO
RLVMRVYSPTTRRYG
>ID37337-ABP_neg
RLVRILVSKRPVAIKPYFRL
>ID37344-NO
RLWARWYSPTTRRYG
>ID37345-ABP_pos
RLWDIVRRWVGWL
>ID37352-ABP_both
RLWLAIGRR
>ID37353-ABP_both
RLWLAIKRR
>ID37354-ABP_both
RLWLAIWRR
>ID37355-ABP_both
RLWLAWKRR
>ID37357-NO
RLWMAWYSPTTRRYG
>ID37369-NO
RLWMRWYSPWTRRYG
>ID37371-NO
RLWRALPRVLRRLLRP
>ID37375-ABP_both
RLWRRWRRWRR
>ID37377-ABP_neg
RLWVLWRR
>ID37379-NO
RLYMRYYSPTTRRYG
>ID37384-NO
RMKKLGNHKVSCERNTKRCRKAI
>ID37385-NO
RMKQIEDKIEEIESKIKKIENEIARIKKLLQLTVWGIKQLQARIL
>ID37398-NO
RMLGNTPTK
>ID37399-NO
RMLGQ
>ID37400-NO
RMLGQTP
>ID37402-NO
RMLGQTPWK
>ID37403-NO
RMLGQYPYK
>ID37407-NO
RMRRSKSGKGSGGSKGSGSKGSKGSKGSGSKGSGSKGGSRPGGGSSIAGG
>ID37423-ABP_both
RNCTWLFSTKLKLPA
>ID37425-ABP_both
RNFFKRIRRAGKRIRKAIISAAPAVETLAQAQKIIKGGD

>ID37426-NO
RNFLRF
>ID37431-NO
RNKFEFIRF
>ID37437-NO
RNMYSFGL
>ID37438-NO
RNNWQTNVGGAVGSAMIGATVGGTICGPACAVAGAHYLPILWTAVTAATG
>ID37440-ABP_both
RNPRPVYIPQPRPPHPRL
>ID37443-NO
RNRSRHRR
>ID37445-NO
RNSPSSYGLPSRDMSTAY
>ID37455-NO
RPAGFTPFR
>ID37457-NO
RPARPAR
>ID37458-ABP_neg
RPAWLKAAFRVMRACV
>ID37459-ABP_neg
RPAWRKAAFRVMRACV
>ID37463-NO
RPCGDQACE
>ID37466-NO
RPECCTHPACHVSHPELC
>ID37468-NO
RPEIKKKNVFSKPGYCPEYRVPCPFVLIPKCRRDKGCKDALKCCFFYCQM
>ID37469-NO
RPESALLGGSEAGERLLQQGLAQVEA
>ID37471-NO
RPFVEMYSEIPE
>ID37473-NO
RPGLLDLK
>ID37482-NO
RPKCCCVCGVVGRKCCSTWDKCHPVHLPCPSS
>ID37484-NO
RPKHP
>ID37485-NO
RPKHPI
>ID37486-NO
RPKHPIK
>ID37487-NO
RPKHPIKH
>ID37488-NO
RPKHPIKHQGLPQ
>ID37489-NO
RPKHPIKHQGLPQEV
>ID37490-NO
RPKHPIKHQGLPQEVLNEN
>ID37492-ABP_both

RPKHPIKHQGLPQEVLNENLLRFFVAPFPEVFGK
>ID37493-ABP_both
RPKHPIKHQGLPQEVLNENLLRFFVAPFPEVFGKEK
>ID37496-NO
RPKPQFFGLM
>ID37497-NO
RPKPQQFGLM
>ID37498-ABP_both
RPKPQQWFWLM
>ID37499-ABP_both
RPKPRPRPRPE
>ID37502-NO
RPLKPW
>ID37506-NO
RPPGFSPFG
>ID37507-NO
RPPGFSPFRGKFHSQS
>ID37508-NO
RPPGFSPFRI
>ID37513-NO
RPPGLTPFR
>ID37514-NO
RPPGLW
>ID37515-NO
RPPGSPFR
>ID37517-ABP_both
RPPHPRL
>ID37518-NO
RPPIRPPIFPPIRPPFRPPLGPFP
>ID37520-ABP_both
RPPQFTRAQWFAIQHISLN
>ID37527-NO
RPQIPP
>ID37528-NO
RPQQFGLM
>ID37535-NO
RPRPQQFFGLM
>ID37537-ABP_both
RPRRRATTRRRITTGTRRRR
>ID37541-NO
RPSFNSWG
>ID37544-NO
RPTDIKCSESYQCFPVCKSRFGKTNGRCVNGFCDCF
>ID37548-NO
RPVKVYTPNGVEEESSEVFPGEM
>ID37551-ABP_both
RPVYIPQPRPPHPRL
>ID37552-ABP_neg
RPWAWPRLMRKVR
>ID37559-ABP_neg
RPYLPRPRPPRPV

>ID37562-NO
RQAKIWFQNRRMKWKK
>ID37563-NO
RQAQGWNKFRGAW
>ID37565-NO
RQARRNRRRALWKTLLKKVLKA
>ID37566-NO
RQARRNRRRC
>ID37570-NO
RQDMVDESVCYITDNNCNGGKCLRSKACHADPWEL
>ID37572-NO
RQFLRF
>ID37573-NO
RQGAARVTSWLGRQLRIAGKRLEGRSK
>ID37577-NO
RQIAIWFQNRRMKWKK
>ID37578-NO
RQIKAWFQNRRMKWKK
>ID37581-NO
RQIKIFFQNRRMKFKK
>ID37585-NO
RQIKIW
>ID37589-NO
RQIKIWFQ
>ID37591-NO
RQIKIWFQN
>ID37593-NO
RQIKIWFQNMRRKWKK
>ID37596-NO
RQIKIWFQNRR
>ID37598-NO
RQIKIWFQNRRM
>ID37609-ABP_both
RQIKKAFRKMA
>ID37610-ABP_both
RQKDKRPYSERKNQYTGPQFLYPPERIPPQKVIK
>ID37613-NO
RQLLSGIVQQQNNLLRAIEAQQH
>ID37619-NO
RQNSCTYSDARRWALCWSGE
>ID37620-NO
RQPKIWFPNRRKPWKK
>ID37621-NO
RQQPFVPRL
>ID37627-NO
RQRHPDCDKPPDTGN
>ID37628-NO
RQRHRDCDKPPDKTN
>ID37629-NO
RQRLLEK
>ID37630-NO

RQRSRRRPLNIR
>ID37637-NO
RQVFQVAYIIIKA
>ID37642-ABP_both
RQWQSKIRR
>ID37646-NO
RQYAFGL
>ID37647-NO
RQYSFGL
>ID37828-NO
RRCFPPGTFCSRYLPCCSGRCCSGWCTRRCFPRF
>ID37833-NO
RRCSNRNFVRL
>ID37834-ABP_both
RRCVYAYVRVRGVLVRYRRCW
>ID37839-ABP_both
RREAEPEAEPGNNRPVYIPQPRPPHPRL
>ID37841-NO
RREKQRPCDKPRR
>ID37845-ABP_both
RRFFFRFRRF
>ID37852-ABP_both
RRFRPKVTITIQGSARF
>ID37854-ABP_both
RRFWHR
>ID37855-ABP_both
RRFWHRAH
>ID37857-ABP_both
RRGAGLGLALAKDGWALMLKLGFGRR
>ID37859-ABP_both
RRGCFRVCYRGFCFQRCR
>ID37860-ABP_both
RRGFSLKLALAKDGWALMLRLGYGRR
>ID37862-ABP_both
RRGGGLELALAKDGWALMLDLGAGRR
>ID37864-ABP_neg
RRGHYY
>ID37866-ABP_neg
RRGKPSGGSRGSKMGSKDSKGGWRGRPGSGSRPGFG
>ID37870-ABP_both
RRGWLALRLVLAY
>ID37872-ABP_both
RRGYGLKLALAKDGWALMLRLGFGRR
>ID37875-ABP_both
RRHCIKKCMKSRKHNERMIRIRRK
>ID37876-NO
RRHHCRSKAKRSR
>ID37878-NO
RRHPPCCLYGKCRRYPGCSSASCCQRG
>ID37880-NO
RRHSVSG

>ID37881-ABP_both
RRHWRWWRR
>ID37885-ABP_both
RRIIIRWRRI
>ID37887-NO
RRINNDCQNFIGNRAMYE
>ID37888-NO
RRIPNRRPRR
>ID37890-ABP_neg
RRIRFRPPYLPRPGRR
>ID37893-ABP_both
RRIRPRPPRLPRP
>ID37896-ABP_neg
RRIRPRPPRLPRPRPRC
>ID37899-ABP_both
RRIRPRPPRLPRPRPRPLPFPRPGPRPIPRPLPFP
>ID37900-NO
RRIRPRPPRLPRPRPRPLPFPRPGPRPIPRPLPFPRPGPRPIPRPLPFPR
>ID37902-ABP_neg
RRIRPRPR
>ID37925-ABP_both
RRKKWFW
>ID37927-NO
RRKLSQQKEKK
>ID37931-ABP_both
RRKWLWLW
>ID37935-NO
RRLHPQHQRFPRERPWPKPLSLPLPRPGPRPWPKP
>ID37940-ABP_both
RRLLRRLRR
>ID37943-ABP_both
RRLRKKTRKRLKKIGKVLKWI
>ID37945-NO
RRLRPRHQHFPSERPWPKPLPLPLPRPGPRPWPKPLPLPLPRPGLRPWPK
>ID37946-ABP_both
RRLRPRRPRLPRPRPR
>ID37948-NO
RRLRPRRPRLPRPRPRPRPRPRSLPLPRPKPRPIPRPLPLPRPRPKPIPR
>ID37949-NO
RRLRPRRPRLPRPRPRPRPRPRSLPLPRPQPRRIPRPILLPWRPPRPIPR
>ID37951-ABP_neg
RRLRTTTKLPPV
>ID37953-ABP_both
RRLTLRQLLGLGSRRRRRSR
>ID37955-ABP_both
RRLWLW
>ID37969-NO
RRPAAAGKRRREKQRPSDKPRR
>ID37971-ABP_both
RRPFWIIRR
>ID37972-NO

RRPKGRAMRREKQRPSDKPRR
>ID37973-NO
RRPKGRGKRAAAKQRPSDKPRR
>ID37974-NO
RRPKGRGKRRREKQRP
>ID37975-NO
RRPKGRGKRRREKQRPCDKPRR
>ID37976-NO
RRPKGRGKRRREKQRPDAVPRR
>ID37978-NO
RRPKGRGKRRREKQRPTDCHLCGDAVPRR
>ID37979-ABP_both
RRPPCEDVNGQCQPRGNPCLRLRGACPRGSRCCMPTVAAH
>ID37981-NO
RRPPGWSPLR
>ID37982-NO
RRPPIRPPFYPPFRPP
>ID37984-ABP_both
RRPRPRPRPFFF
>ID37987-ABP_both
RRPRPRPRPWWW
>ID37995-ABP_pos
RRQWRGWVRIWL
>ID37999-NO
RRREKQRPCDKPRR
>ID38000-NO
RRRERRAEK
>ID38001-ABP_both
RRRFFF
>ID38002-ABP_both
RRRFFFFFRRR
>ID38004-ABP_both
RRRFVAEQDAIHSRVSREVPTLSDSV
>ID38005-ABP_both
RRRFVAQQNAIHSRVSRQVPTLSNSV
>ID38006-ABP_both
RRRFVVQQDTISPRLEVDERFLPNSVQEQI
>ID38007-ABP_both
RRRFVVQQNTISPRLQVNQRFLPNSVQQQI
>ID38008-NO
RRRFWWFR
>ID38009-NO
RRRFWWFRRR
>ID38011-ABP_both
RRRIIIIIRRR
>ID38021-ABP_both
RRRLLLLLRRR
>ID38024-ABP_both
RRRPRPPYLNRPRPP
>ID38025-ABP_both
RRRPRPPYLPPRRPP

>ID38026-ABP_both
RRRPRPPYLPRPRPP
>ID38027-ABP_both
RRRPRPPYLPRPRPPPFF
>ID38028-ABP_both
RRRPRPPYLPRPRPPPFFPPRL
>ID38029-ABP_both
RRRPRPPYLPRPRPPPFFPPRLPPRIPPGFPPRFP
>ID38032-ABP_both
RRRPRPPYWPRPRPP
>ID38034-NO
RRRQRRKRGGDIMGEWGNEIFGAIAGFLG
>ID38037-ABP_neg
RRRREELKQSWQRMRKLALKR
>ID38052-NO
RRRRNRTRRNRRRVRGC
>ID38056-ABP_both
RRRRRRRRRRRRRR
>ID38058-NO
RRRRRRRRRRRRRRRR
>ID38059-ABP_both
RRRRRRRRRRRRRRRWWW
>ID38066-ABP_both
RRRRRRRWWWRRRRRRRR
>ID38067-ABP_both
RRRRRRRWWWWW
>ID38069-ABP_both
RRRRRRRWWWWWWW
>ID38070-ABP_both
RRRRRRWFWFWF
>ID38073-ABP_both
RRRRSQSPRRRRS
>ID38078-ABP_both
RRRRWFWF
>ID38080-ABP_both
RRRRWWWW
>ID38081-ABP_both
RRRRWWWWRRRR
>ID38082-ABP_both
RRRRWWWWW
>ID38088-ABP_both
RRRSVGEEDAIPSHIEVNKFFLRKPAKEHI
>ID38089-ABP_both
RRRSVGQQNAIPSHIQVNKFFLRKPAKQHI
>ID38090-ABP_both
RRRSVQWCA
>ID38095-ABP_both
RRRVVVVVRRR
>ID38098-ABP_both
RRRWFW
>ID38099-ABP_both

RRRWLWLW
>ID38102-ABP_both
RRRWWW
>ID38107-ABP_both
RRRYIGRYVRFWK
>ID38111-ABP_neg
RRSQARKCSRGNGGKIGSIRCRGGGTRLGGGSLIGR
>ID38119-NO
RRVAITAGKNCIGYCCSL
>ID38143-NO
RRVTSWLGRQLRIAGKRLEGRSK
>ID38159-NO
RRWCYRKCYKGYCYRKCR
>ID38164-ABP_both
RRWFW
>ID38169-ABP_both
RRWHRWWRR
>ID38174-ABP_pos
RRWIRWL
>ID38186-ABP_both
RRWQRWMKKLG
>ID38189-ABP_both
RRWQWRLCYCRRRFCVCVG
>ID38191-ABP_both
RRWQWRMRRLG
>ID38345-NO
RRWRRWWRRWWRRWRR
>ID38353-ABP_both
RRWSFRVSYRGFSYRKSR
>ID38356-ABP_both
RRWVAWRNR
>ID38361-ABP_both
RRWWCA
>ID38362-ABP_both
RRWWCC
>ID38364-ABP_both
RRWWCD
>ID38365-ABP_both
RRWWCE
>ID38367-ABP_both
RRWWCG
>ID38368-ABP_both
RRWWCH
>ID38369-ABP_both
RRWWCI
>ID38370-ABP_both
RRWWCK
>ID38371-ABP_both
RRWWCL
>ID38372-ABP_both
RRWWCM

>ID38373-ABP_both
RRWWCN
>ID38374-ABP_both
RRWWCP
>ID38375-ABP_both
RRWWCQ
>ID38379-ABP_both
RRWWCT
>ID38380-ABP_both
RRWWCV
>ID38381-ABP_both
RRWWCW
>ID38382-ABP_both
RRWWCY
>ID38384-ABP_both
RRWWHWWRR
>ID38386-ABP_both
RRWWKWWRK
>ID38389-ABP_both
RRWWRHWRR
>ID38391-ABP_both
RRWWRRWRR
>ID38394-ABP_both
RRWWRWWR
>ID38395-ABP_both
RRWWRWWRR
>ID38396-ABP_both
RRWWWR
>ID38397-ABP_both
RRWWWRWRRW
>ID38401-ABP_both
RRWYRWWR
>ID38404-NO
RRYAFEVVG
>ID38413-NO
RSADGFGRME
>ID38414-NO
RSADGFGRMESLLTSL
>ID38415-NO
RSADGFGRMESLLTSLRGSAESPAALGEASAAHPLE
>ID38416-NO
RSAEGLGRM
>ID38419-NO
RSAEGLGRMGRLL
>ID38421-NO
RSALSCQMCELVVKKYEGSADKDANVIKKDFDAECKKLFHTIPFGTRECD
>ID38423-NO
RSAQGMGKMERLLASYRGALEPSTPLGDLSGSLGHPVE
>ID38425-ABP_both
RSARAGLQFPVGRIHRHLK
>ID38427-NO

RSAWLNSGMYGSNVTESPVLDNSVTTHNHILR
>ID38428-NO
RSCAEPWCY
>ID38431-NO
RSCSFPSNTWC
>ID38432-ABP_both
RSCVYAYVRVRGVLVRYRRCW
>ID38435-NO
RSFDASPSATSGNHSLN
>ID38436-NO
RSFLRF
>ID38442-NO
RSGRVSN
>ID38443-ABP_both
RSGTDFKLTISKLQK
>ID38445-ABP_both
RSICCSFPDPWGGLCCEDHCSYIGKPGGQCSDKGVCTCN
>ID38447-NO
RSIRAQRHTDMPKTQKEVHLKNTSRGSAGNKTYRM
>ID38449-NO
RSKRCLVYGTPCDWLTIAGMECCSKKCFMMCW
>ID38450-NO
RSLDASPSSAFSGNHSLS
>ID38451-NO
RSLEGSSSPVASLIRGRSLS
>ID38452-NO
RSLEGSSSPVTSLTRGRSLN
>ID38453-NO
RSLPEVLRARTVESSQEQTHSAPASPAHQDISRVSRL
>ID38454-NO
RSLQDTEEKPRSVSASQTDMLDDPDQMNED
>ID38456-NO
RSLQDTEEKSRSFSAPQTEPLNDLDQMNED
>ID38459-NO
RSLQNTEEKSRSFPAPQTDPLDDPDQMTED
>ID38466-NO
RSNNFTPRI
>ID38468-NO
RSQKEGLHYTCS
>ID38469-NO
RSRGRLRRGAIRLQRG
>ID38470-NO
RSRIGLW
>ID38476-NO
RSTPGYGRMDRIL
>ID38477-NO
RSTPGYGRMDRILAA
>ID38478-NO
RSTPGYGRMDRILAALKTSPMEPSAALAVEHGTTHPLE
>ID38479-NO
RSTQGYGRMDKLLATLMGSSEGGALESASQHSLE

>ID38482-NO
RSTQGYGRMDRILAALKTSPMEPSAALAVEHGTTHPLE
>ID38484-NO
RSTRCLPDGTSCLFSRIRCCGTCSSILKSCVS
>ID38487-NO
RSVEGASRMEKLL
>ID38488-NO
RSVEGASRMEKLLS
>ID38489-NO
RSVEGASRMEKLLSS
>ID38490-NO
RSVEGASRMEKLLSSS
>ID38492-NO
RSVEGASRMEKLLSSSNSPSSTPLGFLSQDHSVN
>ID38494-NO
RSVEGSSRMERLLSSGSSSSEPLSFLSQDQSVN
>ID38496-NO
RSVEGVSRME
>ID38499-NO
RSVEGVSRMEKLLSSISPSSTPLGFLSQDHSVN
>ID38501-NO
RSVEGVSRMEKLLT
>ID38503-NO
RSVESSGSSSSEPLSFLSQDQSVS
>ID38505-NO
RSVRAQRHTDMPKAQKEVHLKNTSRGSAGNKNYRM
>ID38507-NO
RSVTTEINTLFQTLTSIAEKVDP
>ID38517-NO
RTCENLADKYRGPCFSGCDTHCTTKENAVSGRCRDDFQCWCTKRC
>ID38526-NO
RTCESQSHRFKGACLSDTNCASVCQTEGFPAGDCKGARRRCFCVKPC
>ID38527-NO
RTCESQSHRFKGPCARDSNCATVCLTEGFSGGDCRGFRRRCFCTRPC
>ID38534-NO
RTCQSKSHHFKYMCTSNHNCAIVCRNEGFSGGRCHGFHRRCYCTRLC
>ID38538-NO
RTGEDFLEECMGGCAFDFCCK
>ID38548-NO
RTLVNEYKNTLKFSK
>ID38552-ABP_both
RTRCRFLRRC
>ID38558-NO
RTRRNRRRVR
>ID38584-NO
RVAPEEHPT
>ID38585-NO
RVAQGPSAFVAGPH
>ID38588-NO
RVCESQSHGFHGLCNRDHNCALVCRNEGFSGGRCKRSRRCFCTRIC
>ID38590-ABP_both

RVCKAIPLPICH
>ID38594-ABP_both
RVCRAIPLPICH
>ID38595-ABP_both
RVCSAIPLPICK
>ID38599-ABP_both
RVCSAWPLPICH
>ID38600-ABP_both
RVCSWIPLPICH
>ID38602-NO
RVDSADESNDDGFD
>ID38611-ABP_both
RVHRLLRRVHRLLRRVHRLLR
>ID38615-NO
RVINDDCPNLIGNR
>ID38620-NO
RVIRWFQNKRCKDKK
>ID38621-NO
RVIRWFQNKRSKDKK
>ID38622-NO
RVISLPAGFSPFR
>ID38623-NO
RVISLPAGLSPFR
>ID38626-ABP_both
RVKASRRSASHPTYSEMIAAAIRAEKSRGG
>ID38629-ABP_both
RVKRFKKFFRKIKKGFRKIFKKTKIFIG
>ID38630-ABP_both
RVKRVWKLVIRLVKALYKLYRAIKKK
>ID38631-ABP_both
RVKRVWPLVIRTVIA
>ID38632-ABP_both
RVKRVWPLVIRTVIAGYNLYRAI
>ID38633-ABP_both
RVKRVWPLVIRTVIALYNLYRAIKKK
>ID38640-ABP_both
RVLKDVESALRESVANWKIVIG
>ID38654-ABP_both
RVMFKWA
>ID38656-NO
RVPPYLGRDCKHWCRDNNQALYCCGPPGITYPPFIRKHPGKCPSVRSTCT
>ID38657-NO
RVPSL
>ID38658-NO
RVPSLM
>ID38660-NO
RVREWWYTITLKQES
>ID38662-NO
RVRILARFLRTRV
>ID38670-NO
RVRSWLGRQLRIAGKRLEGRSK

>ID38671-NO
RVRVFVVHIPRLT
>ID38675-NO
RVTKEWLDEVIHVCGREYVRAILDICAATVGLEAPPL
>ID38678-NO
RVTYEWMMENVKICRNDFVRTAIEVCGHVHLE
>ID38686-ABP_both
RVVRPVVQGVKEKVR
>ID38688-ABP_both
RVVRPVVQVVKEKVR
>ID38690-ABP_both
RVVRQWPIGRVVRRVVRRVVRF
>ID38696-ABP_neg
RVVRVVRRWVRRVRRVWRRVVRVVRRWVRRVRRWVRRVVRVVRRWVRR
>ID38697-NO
RVVSGSKGSAALALCRQFEQLSAS
>ID38704-NO
RVYDFGL
>ID38705-NO
RVYVHPF
>ID38709-ABP_neg
RWAWRLMRKVR
>ID38711-ABP_both
RWCGYAYVRVRGVLVRYRRCW
>ID38713-NO
RWCREKSCW
>ID38714-ABP_both
RWCRYAYVRVRGVLVRYRRCW
>ID38715-ABP_both
RWCSYAYVRVRGVLVRYRRCW
>ID38716-ABP_both
RWCVRARVRGVRYRRCW
>ID38717-ABP_both
RWCVYACVRGVCYRRCW
>ID38718-ABP_both
RWCVYARRRGVRYRRCW
>ID38719-ABP_both
RWCVYARVRGRRYRRCW
>ID38720-ABP_both
RWCVYARVRGVRYRRCR
>ID38722-ABP_both
RWCVYAYGRVRGVLVRYRRCW
>ID38723-ABP_both
RWCVYAYRRVRGVLVRYRRCW
>ID38744-NO
RWDQLHGAW
>ID38745-NO
RWDVDQCIYYCLNGVVGYSYTECQTMCT
>ID38747-ABP_both
RWFHWK
>ID38758-NO

RWHRLKE
>ID38764-ABP_pos
RWIRWL
>ID38768-ABP_both
RWKIFKKIEKMGGSYCNRRTGKCQRM
>ID38770-ABP_neg
RWKIFKKIERVGQNVRDGIIKAGPAIQVLGTAKALGK
>ID38771-ABP_both
RWKIFKKIPKFLHSAKKF
>ID38774-ABP_both
RWKKWWRWL
>ID38780-ABP_pos
RWKLLLLLRL
>ID38786-ABP_both
RWKRWWRLL
>ID38787-ABP_both
RWKRWWRWI
>ID38795-ABP_both
RWLKKRRWKYYVPP
>ID38796-ABP_pos
RWLLKLGHHGR
>ID38797-ABP_pos
RWLLKLGHHGRAPP
>ID38800-ABP_pos
RWLWWLWWL
>ID38813-NO
RWQWRM
>ID38814-ABP_both
RWQWRWQWR
>ID38827-ABP_pos
RWRLLGLLKRH
>ID38828-ABP_pos
RWRLLLLKKF
>ID38831-ABP_pos
RWRLLLLKRH
>ID38832-ABP_pos
RWRLLLLKWH
>ID38834-ABP_pos
RWRLLLLLRWR
>ID38840-ABP_both
RWRRLLKKLHHLLHSGGFIKHFIHRF
>ID38842-ABP_both
RWRRWRRWRRWR
>ID38845-NO
RWRTNF
>ID38848-ABP_neg
RWRWNRRTGRWRWY
>ID38850-ABP_both
RWRWRWFGGGFIKHFIHRF
>ID38851-ABP_both
RWRWRWFSATFIKHFIHRF

>ID38858-ABP_both
RWRYRW
>ID38859-NO
RWSKFQGSW
>ID38863-NO
RWTKQIPPNQCSGKKKNKNGKSFN
>ID38865-ABP_both
RWTWRGSGRWTWR
>ID39051-ABP_both
RWWRRWWRWWRRWWRR
>ID39052-ABP_both
RWWRWWR
>ID39053-ABP_both
RWWRWWRRWW
>ID39054-ABP_both
RWWRWWRRWWRWWR
>ID39056-ABP_both
RWWRYWR
>ID39059-ABP_both
RWWVAWRNR
>ID39092-NO
RYAFEVVG
>ID39098-NO
RYIRF
>ID39100-NO
RYLGY
>ID39103-NO
RYLMT
>ID39104-NO
RYLPT
>ID39107-NO
RYPSYG
>ID39117-ABP_both
RYWVAWRNR
>ID39122-NO
SAAAPLIRFG
>ID39123-ABP_both
SAADKGNVKAAWGKVGGHAAEYGAEALERMFLSF
>ID39124-NO
SAALAGTIIDGASLGFDILNKV
>ID39126-NO
SADDSAPFGTMRF
>ID39127-NO
SADIVKKLWDNPAL
>ID39128-NO
SADLFGAPMYII
>ID39130-NO
SADPNFLRFG
>ID39131-NO
SADPYRFMTVPT
>ID39132-NO

SADWNSLRGTW
>ID39136-NO
SAEFPDFYDSGEHLSPR
>ID39138-NO
SAEPFGTMRF
>ID39140-NO
SAEPQIDYEDMSADLEEIPVFIQ
>ID39141-NO
SAERGVVAMS
>ID39142-NO
SAETVESCLAKSH
>ID39144-NO
SAFTVWSGPGCNNRAERYSKCGCSAIHQKGGYDFSYTGQTAALYNQAGCS
>ID39148-NO
SAIDRSMIRF
>ID39150-NO
SAIWFWMTPQSPK
>ID39151-NO
SALDKNFMRF
>ID39152-ABP_both
SALGRLASKVVPAVIGKVTT
>ID39153-ABP_both
SALGRVASKVFPAVYCSITK
>ID39154-NO
SALLPWPVLVNY
>ID39155-NO
SALNKNFIRF
>ID39157-NO
SALRGCWTKSYPPKPCLGK
>ID39158-NO
SALVGCWTKSYPPKPCIGR
>ID39159-NO
SALVGCWTKSYPPKPVSVDDKTCLANHLMWNIIWLNARCLMKK
>ID39161-NO
SALYALYDFSPPARKMRAYTVRAYVHGSYSRRGPWYDFEPVPGASMDGL
>ID39162-NO
SANAKNDFMRF
>ID39163-NO
SANPALAPRERKAGCKNFFWKTFTSC
>ID39165-NO
SANSNPALAPR
>ID39167-NO
SANSNPALAPRERKAGCKNFFWKTFTSC
>ID39168-NO
SANSNPAMAPRE
>ID39169-NO
SANSSPLAARERKAGCKNFFWKTFTSC
>ID39170-NO
SANTKNDFMRF
>ID39173-NO
SAPLRVY

>ID39175-NO
SAPMASDYGNQFQMYNRLIDAG
>ID39176-NO
SAPQDFVRS
>ID39177-NO
SAPSDFSRDIMSF
>ID39180-NO
SAPTEFERN
>ID39181-NO
SAQGQDFMRF
>ID39184-NO
SARHHCRSKAKRSRHH
>ID39189-NO
SASFVRF
>ID39190-NO
SASGGAGESSGMWFGPRL
>ID39191-NO
SASGSGESSGMWFGPRL
>ID39193-NO
SASSGESSGMWFGPRL
>ID39195-NO
SATAEEGSENAEIEESHLGNSRS
>ID39196-NO
SATCCNYPPCYETYPESCL
>ID39197-NO
SATGAPWKMWVR
>ID39199-ABP_both
SAVGKLASKVFPAVFSMVTK
>ID39201-ABP_both
SAVGRHLRRFLLRKHRKH
>ID39202-ABP_both
SAVGSVASKVVPSLISKVTK
>ID39204-NO
SAVQDSPCKGSAESLMYIYKLVQNEAQKILECEKFSSN
>ID39206-ABP_both
SAVWRHWRRFWLRKHRKH
>ID39207-ABP_both
SAVWRRWRRFWLRKRRKR
>ID39208-ABP_both
SAVWRWRRFWLRKRK
>ID39209-NO
SAWRACSVTCGKGIQKRSR
>ID39211-NO
SAYPGQITSN
>ID39212-NO
SCADAYKSCDSLKCCNNRTCMCSMIGTNCTCRKK
>ID39222-NO
SCCPNNPACCH
>ID39224-NO
SCCPQEFLCCLYLVK
>ID39225-NO

SCCPREFLCCL
>ID39227-NO
SCCPTILSCCFV
>ID39237-NO
SCDTSTCATQRLADFLSRSGGIGSPDFVPTDVSANSF
>ID39242-NO
SCGGSCFGGCWPGCSCYARTCFRDGLP
>ID39244-NO
SCGHSGAGCYTRPCCPGLHCSGGHAGGLCV
>ID39246-NO
SCGHVGTPCEKNWDCCKGKVCSPRWKLCAYESPF
>ID39247-NO
SCGNLGESCSAHRCCPGLMCMGEASICIPY
>ID39253-NO
SCGRRGKPCPCCRGFRCTGSFCRKWQ
>ID39254-NO
SCHTG
>ID39255-NO
SCIKHGDFCDGDNDDCQCCRDNGF
>ID39259-NO
SCKDGPCVTNRLEGWLARAERMVKNTFMPTDVDPEAFGHQHKELAA
>ID39260-NO
SCKRECSGSKRQK
>ID39261-NO
SCKVPFNECKYGADECCKGYVCSKRDGWCKYHIN
>ID39264-ABP_both
SCLPKEEQIGKSTRGRKCRRKK
>ID39269-NO
SCNNSCQQHSQCASHCVCLLNKCRTVN
>ID39270-NO
SCNRATCVTHKMAGSLSRSGSEIKRNFMSTNVGSKAFGQRSRDLQK
>ID39271-NO
SCNSATCVAHWLGGLLSRAGSVANTNLLPTSMGFKVYNRRRRELKA
>ID39272-NO
SCNTAICVTHKMAGWLSRSGSVVKNNFMPINMGSKVL
>ID39274-NO
SCNTASCVTHKMTGWLSRSGSVAKNNFMPTNVDSKILG
>ID39275-NO
SCNTATCMTHRLVGLLSRSGSMVRSNLLPTKMGFKVFG
>ID39276-NO
SCNTATCVTHRLAGLLSRLGGVVKSNFVPTNVGSQAF
>ID39279-NO
SCNTATCVTHRLAGLLSRSGGVVKSNFVPTDVGSEAF
>ID39282-NO
SCRNEGAMCSFGFQCCKKECCMSHCTDFCRNPD
>ID39284-ABP_both
SCRYSQRPSFYRWELYFNGRMWCP
>ID39287-NO
SCSDDWQYCEYPHDCCSWSCDVVCS
>ID39290-NO
SCSGSGYGCKNTPCCAGLTCRGPRQGPICL

>ID39291-NO
SCSSGCSDCNSDSCQCTLNQFTNSDSCCC
>ID39293-ABP_both
SCSYSVKPDIQGFELYFIGSVTCP
>ID39313-NO
SCVDFQTKCKKDSDCCGKLECSSRWKWCVYPSPF
>ID39317-NO
SCVGEYGRCRSAYEDCCDGYYCNCSQPPYCLCRNNN
>ID39325-NO
SDAFSRNFMNF
>ID39327-NO
SDARLQGLF
>ID39328-NO
SDCSGMSDGTSCGDTGVCQNGLCMGAGS
>ID39329-NO
SDCTLRNHDCTDDRHSCCRSKMFKDVCTCFYPSQ
>ID39331-ABP_neg
SDDAQRCYPHNRTPFTYTYI
>ID39345-NO
SDEENLDFLE
>ID39346-NO
SDEEVQVPGGVISNGYFLFRPRN
>ID39351-NO
SDGKSAAAKAKPSHLTAPFIRDECCSDSRCGKNCL
>ID39360-NO
SDGRNAAADARASPRIALFLRFTCCRRGTCSQHCG
>ID39383-NO
SDGRNIAVDDRWSFYTLFHATCCADPDCRFRPGC
>ID39391-NO
SDGWSPSGDGSW
>ID39394-NO
SDIGISEPNFLRF
>ID39399-NO
SDLEGNGVGIGDDHEQALVHPWLYLWGE
>ID39400-NO
SDLPFEH
>ID39401-NO
SDLTWTYQSPGDPTNSKN
>ID39403-NO
SDMPFEF
>ID39405-NO
SDMYSFGL
>ID39406-NO
SDNFMRF
>ID39407-NO
SDPDLDDVIRASLLAYSLDDSPNN
>ID39408-NO
SDPDMLNNIVE
>ID39409-NO
SDPDMLNSIVE
>ID39410-NO

SDPEAPGIWFGPRL
>ID39412-NO
SDPFFM
>ID39413-NO
SDPFFRF
>ID39414-NO
SDPFLRF
>ID39415-NO
SDPGEDMLKSILLRGAPSNNGLQY
>ID39416-NO
SDPHLSILSKPMSAIPSYKFDD
>ID39418-NO
SDPNFLRF
>ID39419-NO
SDPNFLRFG
>ID39421-NO
SDPRFLRLV
>ID39422-NO
SDPSVPLRPEEDELIDQ
>ID39423-NO
SDPSVPVEPEDDDMVDQ
>ID39424-NO
SDPYLFR
>ID39425-NO
SDPYLRF
>ID39428-NO
SDRNYLRF
>ID39429-NO
SDRPTRAMDSPLIRF
>ID39430-NO
SDRSPSLRLRF
>ID39433-NO
SDSEEEMKALEADLLTNMHT
>ID39435-NO
SDSKIGDGCFGLPLDHIGSVSGLGCNRPVQNRPKK
>ID39438-NO
SDTVTPLRGFE
>ID39441-ABP_both
SDVSS
>ID39442-NO
SDWFAWVIHSCCSNPPCAHVNC
>ID39443-NO
SDYIRYSDRCCNVGCTRKELADLC
>ID39444-NO
SDYLQLAR
>ID39445-NO
SDYLQLARG
>ID39448-NO
SEALVVDGARKTGAEEAQPPEGQGEREHSRQEEEEEEETAGASRGLFRG
>ID39451-NO
SECRWFMGGCDSTLDCCKHLSCKMGLYYCAWDGTF

>ID39452-NO
SECVENGGFCPDPEKMGDWCCGRCIRNECRNG
>ID39453-NO
SEDRNTGNSLRDSSSFFPARY
>ID39456-NO
SEDRSTGNSLKDSSSFSPARY
>ID39462-NO
SEEPPMSDLTFHMLRNMHRAKMEGEREQALNRNLLDEV
>ID39463-NO
SEESQEKEYP
>ID39469-NO
SEGHKNPKELPKFFEIKPRVDQFFIGSRY
>ID39478-NO
SEKWNKLSSSW
>ID39481-NO
SELINSILGLPKVMNDA
>ID39483-NO
SENPSNSRNFIRL
>ID39484-NO
SENRNFLRF
>ID39485-NO
SENTGAIGKVFPRGNHWAVGHLM
>ID39487-NO
SEPYLRF
>ID39489-NO
SEQEAGLDTGDGDGDQQYLVRPWLYLWADN
>ID39491-NO
SEQPDVDDYLRDVVLQSEEPLY
>ID39497-NO
SETDSRKEGRMGQKNAVIRQWCENDCNYAPK
>ID39499-NO
SEVPGVLRF
>ID39500-NO
SEVRSRVASRSADERFFGGPRF
>ID39503-NO
SFAKAPHLDL
>ID39506-NO
SFCIPFKPCKSDENCCKKFKCKTTGIVKLCRW
>ID39509-NO
SFDRIDGSAFGPHRH
>ID39515-ABP_both
SFGAKNAVKNGLQKLRNQCQANNGQGGFCDIFKKNG
>ID39523-ABP_both
SFGVTIGIGPAGSPLLVGVGVSHSQD
>ID39525-NO
SFHQFARATLAS
>ID39528-NO
SFKWHPRCGEAYSMWT
>ID39529-NO
SFLCDLKILATNAAKNAGQCVVTTLSCKLCGTC
>ID39531-NO

SFLLTSFKDMAIKVAKKAGLNILSTASCKLFGTC
>ID39537-NO
SFLSLIRKLIT
>ID39539-NO
SFLTTVKKLVTNVAALAGTVIDTIKCKITGGCRT
>ID39540-NO
SFNAASPLLANGHLHRASELGLTDLYDLQDWSSD
>ID39542-NO
SFNAASPLLTTGHLHRGSELGLSDLYDLQEWTSD
>ID39543-NO
SFNQPYLYKTAF
>ID39545-NO
SFPDSNIAPSSP
>ID39546-NO
SFPWMESDVT
>ID39550-NO
SFRNGV
>ID39552-NO
SFRNGVGSGVKKTSFRRAKQ
>ID39554-NO
SFRNGVGTGMKKTSFRRAKS
>ID39555-NO
SFSCSQNGGFCISPKCLPGSKQIGTCILPGSKCCR
>ID39557-NO
SFSENMINDHRQPAPTNNNY
>ID39558-NO
SFSIIHTPILPL
>ID39561-ABP_both
SFVFKLASKVVPSVFSALTR
>ID39563-NO
SFVRIL
>ID39565-NO
SGADFYPWG
>ID39566-NO
SGAPQPVARYCGEKLSNALKLVCRGNYNTMF
>ID39570-NO
SGDTSSQAKGMWFGPRL
>ID39571-NO
SGDWSSLRGAW
>ID39573-NO
SGETSGEGNGMWFGPRL
>ID39574-NO
SGFFGMR
>ID39575-NO
SGFLGMR
>ID39576-NO
SGFNSPSPRY
>ID39578-NO
SGFYALRY
>ID39579-NO
SGFYANRY

>ID39580-NO
SGFYAPRY
>ID39581-NO
SGFYCNRY
>ID39582-NO
SGFYSDRY
>ID39583-NO
SGFYSNRY
>ID39586-NO
SGGFAFSPRL
>ID39587-NO
SGGGDGSGMWFGPRL
>ID39588-NO
SGGGEGSGMWFGPRL
>ID39589-NO
SGGGMFSTNDVMQQ
>ID39595-NO
SGHYIFGL
>ID39596-NO
SGHYNFGL
>ID39597-ABP_both
SGIQPEQHSS
>ID39598-NO
SGIVQQQNNLLRAIEAQQHLLQ
>ID39602-NO
SGKLSFLE
>ID39603-NO
SGKLSFLEDE
>ID39605-NO
SGKPTFIRF
>ID39606-NO
SGKWSNLRGAW
>ID39607-NO
SGLDSLSGATFGGNR
>ID39608-NO
SGLQFAVLDGQGFIPFPRV
>ID39609-NO
SGLQFAVLDGQGFIPFSRV
>ID39611-NO
SGLQFAVLDGQGFLPFSRV
>ID39612-NO
SGLQFAVMDGQGFLPFPRV
>ID39613-NO
SGMRNALVRF
>ID39614-NO
SGNGGIVLGNSELDARNPERFFIGSRY
>ID39615-NO
SGNSNELRRGKL
>ID39616-NO
SGNTADLYDRRIMAF
>ID39617-NO

SGNYNFGL
>ID39619-NO
SGPAGVL
>ID39620-NO
SGPGFMGVR
>ID39623-NO
SGPQAHEGAGMRFAFA
>ID39624-NO
SGPSYDEKEQENEDGNSVRL
>ID39627-NO
SGQIFAQ
>ID39628-NO
SGQSWRPQGRF
>ID39629-NO
SGQYSFGL
>ID39630-NO
SGRGKQGGKARAKAKTRSSRAGLQFPVGRVHRLLRKG
>ID39636-NO
SGRNMEVSLVRQVLNLPQRF
>ID39637-NO
SGRVDHIHDILSTLQRLQLANE
>ID39644-NO
SGSLSTFFLLFNRSFTQALGK
>ID39645-NO
SGSLSTFFRLFLRSFTQALGK
>ID39646-NO
SGSLSTFFRLFNASFTQALGK
>ID39647-NO
SGSLSTFFRLFNFSFTQALGK
>ID39649-NO
SGSLSTFFRLFNRSFTQ
>ID39651-NO
SGSLSTFFRLFNRSFTQAGK
>ID39652-NO
SGSLSTFFRLFNRSFTQAL
>ID39675-NO
SGTGLSATLPQRF
>ID39676-NO
SGTVGR
>ID39677-NO
SGWCYRC
>ID39679-NO
SGWKQCSFNAVSCF
>ID39680-NO
SGWMDYINGFLKGFGGQRTLPTKDYNIPQV
>ID39681-NO
SGWNKMQGVW
>ID39682-NO
SGYLAFPRM
>ID39683-NO
SGYNANRY

>ID39684-NO
SGYSVLYF
>ID39685-NO
SHAFTWPTYLQL
>ID39688-NO
SHGKPPSFSPYT
>ID39690-ABP_both
SHHWLWLW
>ID39693-ABP_both
SHKWLWLW
>ID39695-NO
SHNWLPLWPLRP
>ID39701-NO
SIATLAKNDDLPISLHDRMAENEDDEE
>ID39710-NO
SIFPAIVSFLSKF
>ID39712-NO
SIFTLVA
>ID39714-NO
SIGDQPSIFNERASFTGLM
>ID39719-ABP_neg
SIGSALKKALPVAKKIG
>ID39724-ABP_both
SIITGTKEAKLGQLGKQIACRLGNTC
>ID39725-ABP_both
SIITGTKEAKLPQLWKQIACRLYNTC
>ID39729-NO
SIKPFANLPLRF
>ID39730-NO
SIKPFSNLPLRF
>ID39731-NO
SIKPSAYLPLRF
>ID39736-NO
SILSGIFGAGKKIVCGLSGLC
>ID39739-NO
SINSQIGKATSNLVECVFSLFKKCN
>ID39740-NO
SINSQIGKATSSISKCVFSFFKKC
>ID39748-NO
SIPLPSLNFEQFGNMIQCTI
>ID39749-NO
SIPNLPQRF
>ID39750-NO
SIRAPQLRLRF
>ID39756-NO
SIRMCRREAQLCDPIFQNCCHGLFCVLVCV
>ID39760-NO
SISCNFPDPFGGLICENHCAYIGKPGGQCSDQKVCTCN
>ID39764-NO
SITRTEACYEYCKEQNKTCCGISNGRPICVGGCI
>ID39765-NO

SIVNCSAALTDLPTRCGGNI
>ID39767-NO
SIWDIIKDTGKGFLVSILDKVRCKVAGGCPP
>ID39769-NO
SKADT
>ID39770-NO
SKCFSPGTFCGIKPGLCCSVRCFSLFCISFE
>ID39772-ABP_both
SKCKCSRKGPKIR
>ID39773-NO
SKCKCSRKGPKIRYSDVKKLEMKPKYPHCEEKMVIITTKSVSRYRGQEHC
>ID39774-NO
SKCRIGKDGFYSVTCTEKENLCFTQF
>ID39776-ABP_neg
SKCYQWQRRMRKLGAPSITCVRRTS
>ID39778-NO
SKDYN
>ID39783-ABP_both
SKFFRKARKKLGKGLQKIKNVLRKY
>ID39784-ABP_both
SKFPLAGIFSVPGVKRVVVI
>ID39787-NO
SKGLRHR
>ID39790-NO
SKKKKTKV
>ID39796-ABP_both
SKKLICYCRIRGCKRRERVFGTCRNLFLTFVFCCS
>ID39800-NO
SKLLYD
>ID39801-NO
SKLMYD
>ID39802-NO
SKMYGFGL
>ID39803-NO
SKNFLRF
>ID39807-NO
SKNYLRF
>ID39808-NO
SKPAD
>ID39810-NO
SKPDT
>ID39811-NO
SKPHT
>ID39814-NO
SKPYMRF
>ID39815-NO
SKQCCHLAACRFGCTPCCW
>ID39818-NO
SKQCCHLPACRFGCTPCCW
>ID39820-NO
SKQLRYHHCYFNPISCF

>ID39822-NO
SKRKSRPVSVKTFEDIPLEEP
>ID39823-NO
SKRLSNGCFGLKLDRIGAMSGLGCWRLINESK
>ID39826-NO
SKSHQFYGLM
>ID39828-NO
SKSSGVS
>ID39829-ABP_both
SKSWLWLW
>ID39830-NO
SKVAFSAVRSTN
>ID39832-ABP_both
SKVGRHLRRFGHRAHRKL
>ID39833-ABP_both
SKVGRHLRRFLHRAHRKL
>ID39834-NO
SKVLPVPE
>ID39835-NO
SKVLPVPQ
>ID39836-NO
SKVLPVPQK
>ID39838-NO
SKVLYYNWE
>ID39839-NO
SKVPP
>ID39840-ABP_both
SKVWRHW
>ID39841-ABP_both
SKVWRHWRRFW
>ID39842-ABP_both
SKVWRHWRRFWHR
>ID39844-ABP_both
SKVWRHWRRFWHRAHRLH
>ID39845-NO
SKVYP
>ID39846-NO
SKVYPFPGPI
>ID39849-NO
SKWSECSRTCGGGVKFQER
>ID39850-NO
SKYHQFYGLM
>ID39857-NO
SLALADDAAFRERARLLAALERRHWLNSYMHKLLVLDAP
>ID39858-NO
SLALADDAAFRERARLLAALERRRWLDSYMQKLLLLDAP
>ID39859-NO
SLALGRVDFRPG
>ID39861-NO
SLATLAKNGQLPTAEPGEDYGDADSGEPSEQ
>ID39863-NO

SLAYDDKVFENVEFTPRL
>ID39864-NO
SLCCPEDAWCC
>ID39867-NO
SLCHPPCTGLSMCHGGMCGYIRF
>ID39870-NO
SLDSLGSFQVHGW
>ID39871-NO
SLDSMSPQWHAD
>ID39872-NO
SLDSPANRGFTHFD
>ID39874-NO
SLEEILDEIK
>ID39886-NO
SLGGKPDLRPCHPPCHYIPRPKPR
>ID39889-ABP_both
SLGTPDHYHGGRHSISRGSQSTGPTHPGYNRRNA
>ID39891-NO
SLGWMLPFSPPF
>ID39894-NO
SLIEFGKMITEETNRPVFPYEATIVVCDCGNGNGS
>ID39896-NO
SLIQFETLIMKVAGQSGMFSYSA
>ID39899-NO
SLKPAANLPLRF
>ID39900-NO
SLKSDTVTPLR
>ID39906-NO
SLLEVLRSRTVESSQEQTHTAPAPWAHISRLFRI
>ID39907-NO
SLLGTVSLIAYWCRQECGPECAERIIL
>ID39908-NO
SLLPLIRKLIT
>ID39909-NO
SLLQFEMMIMEVAKRSGIFWYSA
>ID39911-NO
SLLSLFRKLI
>ID39915-ABP_both
SLLSLIRLLIT
>ID39916-NO
SLLSTLGNMAKAAGRAALNAITGLVNQ
>ID39917-NO
SLNFEELKDWGPKNVIKMSTPAVNKMPHSFANLPLRF
>ID39918-NO
SLNMSRL
>ID39920-NO
SLNTKNDFMRF
>ID39923-ABP_both
SLPPTLEEEFNMKKMG
>ID39926-NO
SLQDNFMHF

>ID39927-NO
SLQGGAPNFPQPSQQNGGWQVSPDLGRDDKGNTRGQIEIQNKGKDHDFNA
>ID39939-NO
SLRGCWTKSFPPQPCLGKRLNMN
>ID39940-NO
SLRGCWTKSYPPQPCLGKR
>ID39942-NO
SLRGPAESSGESAHPLE
>ID39943-NO
SLRGPAESSGESALSE
>ID39948-NO
SLSAASAPLAETSTPLRL
>ID39950-NO
SLSMLRL
>ID39951-NO
SLSTFFRLFNFSFTQALG
>ID39959-NO
SLSYEDKMFDNVEFTPRL
>ID39960-NO
SLTFEEVKDWAPKIKMNKPVVNKMPPSAANLPLRF
>ID39963-NO
SLVELGKMILQETGKNPVTYGAY
>ID39964-NO
SLVLPVPE
>ID39965-NO
SLVPQSYSNNENQI
>ID39966-NO
SLVQFEKMIKEVAGKNGVPWY
>ID39967-NO
SLVQFEMMIMEVAKRSGLLWYSA
>ID39968-NO
SLVSFLG
>ID39970-NO
SLVYP
>ID39971-ABP_both
SLWKNLSRKISGAVKAQPDLHTLLPLPGS
>ID39974-NO
SMADISDMPEDVPM
>ID39976-NO
SMDDLDDPRLMTMSFG
>ID39977-NO
SMEHFRWGKPM
>ID39978-NO
SMEPALPDWWWKMFK
>ID39979-NO
SMFSVLKDLGKVGLGFVACKVNKQC
>ID39980-NO
SMFSVLKNLGKVGLGFVACKVNKQC
>ID39984-ABP_both
SMLAVLKNLGKVGLGFVACKINKQC
>ID39985-ABP_neg

SMLAYVDKNDHINPPHSPRS
>ID39986-ABP_both
SMLGVLKNLGKVGLGFVACKINKQC
>ID39987-NO
SMLKRNHSTSNR
>ID39988-ABP_both
SMLKVLKNLGKVGLGFVACKINKQC
>ID39990-ABP_both
SMLSVLKNLEKVELEFVACKINKQC
>ID39991-ABP_both
SMLSVLKNLGKVGLGFVA
>ID39992-ABP_both
SMLSVLKNLGKVGLGFVAAKIAKQA
>ID39999-ABP_both
SMLSVLKNLKKVKLKFVACKINKQC
>ID40004-NO
SMPINSPYIPWS
>ID40005-NO
SMPSLRLRF
>ID40006-NO
SMRRSSDCFGSRIDRIGAQSGMGCGRRF
>ID40007-NO
SMSIARL
>ID40008-NO
SMSIASPQIPWS
>ID40010-NO
SMSIASPYIPWS
>ID40011-NO
SMSIGSPYITFG
>ID40012-NO
SMSMLRL
>ID40015-NO
SMVARQIPQTVVADH
>ID40017-NO
SNAFDRNFMNF
>ID40018-NO
SNCFYNTEFLWCIDATENTR
>ID40020-NO
SNFDCCLGYTDRILHPKFIVGFTRQLANEGCDINAIIFHTKKKLSVCANP
>ID40021-NO
SNFDGREASLPRF
>ID40022-ABP_neg
SNGDGTLDAGSTCAPFYARA
>ID40026-NO
SNGRNAAADAKASQRIAPFLRDYCCRRPPCTLICG
>ID40027-NO
SNGRNAAADDKPSDWIALAIKQCCANPPCKHVNCR
>ID40033-NO
SNGYLALPRQ
>ID40039-NO
SNKDEEQRELLKA

>ID40040-NO
SNKDEEQRELLKAISNLL
>ID40041-NO
SNKDEEQRELLKAISNLLD
>ID40042-ABP_both
SNKGAIKGLMVKGVK
>ID40043-NO
SNKKDYRKEIVDKHNALSRSVKPTASNM
>ID40046-NO
SNKRKNAAMLDMIAQHAIRGCCSDPRCRYRCR
>ID40049-NO
SNLVECVFSLFKKCN
>ID40050-ABP_both
SNNFGAKLSSTKVGK
>ID40052-NO
SNPETMVSDVWWRESTENIPRSRFEDPSMW
>ID40053-NO
SNPFSKPYGLTV
>ID40054-NO
SNPWDSLLSVST
>ID40055-NO
SNPYSFGL
>ID40060-NO
SNSKCPDGPDCFVGLM
>ID40061-NO
SNSLFEEVRPIVNGMDCKLG
>ID40068-NO
SPAFVRIL
>ID40070-NO
SPAISPAYQFENAFGLSEALERAG
>ID40071-ABP_both
SPAIWGCDSFLGYCRLACFAHEASVGQKECAEGMLCCIPNVF
>ID40072-NO
SPALADEHNDNFLRF
>ID40074-NO
SPALDDEHNDNFLRL
>ID40075-NO
SPALDDERNDNFIRL
>ID40078-NO
SPAPANKVPHSAANLPLRF
>ID40080-NO
SPAQWQRANGLWG
>ID40081-NO
SPASGFFGMR
>ID40082-NO
SPATMGFAGVR
>ID40084-ABP_both
SPEEARVKILTAIPEMREEDLSEE
>ID40086-NO
SPFDCSSPGAFCGLVPCCDSCNVLGRC
>ID40087-NO

SPFPRIL
>ID40088-NO
SPFRYLGAR
>ID40089-ABP_both
SPFVDRPRRPIQHNGPKPRIITNPPFNPNARPAW
>ID40090-NO
SPGALEDEHNDNFLRF
>ID40092-NO
SPGMLGLF
>ID40093-NO
SPGPMKLLKTPL
>ID40094-ABP_both
SPGRPRPFPGRPKPIFRPR
>ID40095-NO
SPGSQDFMRF
>ID40097-NO
SPGSWTW
>ID40099-ABP_both
SPHRPRHSRLQREPQVQWLEQQVAKRRTKR
>ID40100-NO
SPHYDFGL
>ID40101-NO
SPHYNFGL
>ID40102-ABP_both
SPIHARYQRGVIPGPRWPYYRVGSGSGLKSVRNRWA
>ID40104-NO
SPKNFLRF
>ID40105-NO
SPKQDFMRF
>ID40108-NO
SPLARSSIQSLLNLPQ
>ID40109-NO
SPLDEEDFAPESPLQ
>ID40111-NO
SPLGTMRF
>ID40112-NO
SPLLSTRAVQLS
>ID40113-NO
SPLPRIL
>ID40116-NO
SPLQETEEKSRSFKASQAEPLDDSRQLNEV
>ID40117-NO
SPLSSSSSSPSSSAAMEPLTANQLLASALSSGGLNSLKPSEKALL
>ID40120-NO
SPMERSAMVRF
>ID40121-NO
SPMFTMIQGDAQ
>ID40122-NO
SPMQKTMNLPPM
>ID40123-NO
SPMQRSSMVRF

>ID40124-NO
SPMQRSSMVRFG
>ID40125-NO
SPNDWAHFRGSW
>ID40126-NO
SPNIFGQWM
>ID40127-NO
SPNITVTLKKFPL
>ID40129-NO
SPPEIN
>ID40130-NO
SPPFAPRL
>ID40131-NO
SPPPFYL
>ID40132-ABP_both
SPPPGESKVDMSFNYALSNPAQ
>ID40133-ABP_both
SPPQAMGFPPQVNVEHYIPASYSVAALTVTEEE
>ID40134-NO
SPPSQDFMRF
>ID40136-NO
SPQLRLRF
>ID40137-NO
SPREPIRF
>ID40139-NO
SPRPDDKKNQGSASVDVQNERGEGTKVDARVRQELWRSDDGRTRAQAYGH
>ID40144-NO
SPSAAPLIRF
>ID40146-NO
SPSAKWMRFG
>ID40147-NO
SPSAVPFIRF
>ID40149-NO
SPSAVPLIRFG
>ID40150-NO
SPSLDDERNDNFVRL
>ID40154-NO
SPSTHPNEGLEENYCRNPDN
>ID40155-NO
SPTCIPSGQPCPYNENCCSQSCTFKENENGNTVKRCD
>ID40156-NO
SPTLDDEHNDNFVRL
>ID40157-NO
SPTQDFMRF
>ID40158-NO
SPTSQQHNDAASLSKIYPRGSHWAVGHLM
>ID40159-NO
SPVDAFSPPEASLTGGQSLS
>ID40161-NO
SPVDYDRPIMAFG
>ID40162-NO

SPVPEDDRGDNFVRL
>ID40163-NO
SPWDIASVTAGGVQKRS
>ID40164-NO
SPWDICSVTCGGGVQKRSR
>ID40165-NO
SPWSKCSAACGQTGVQTRTR
>ID40166-NO
SPWSPCSGNCSTGKQQRTR
>ID40167-NO
SPWSPCSTSCGLGVSTR
>ID40169-NO
SPWSQCSVRCGRGQRSRQVR
>ID40170-NO
SPWSQCTASCGGGVQTR
>ID40171-NO
SPWSSASVTAGDGVDITRIR
>ID40173-NO
SPWSSCSVTCGDGVITRIR
>ID40174-NO
SPWTKCSATCGGGHYMRTR
>ID40175-NO
SPYAFGL
>ID40177-NO
SPYRAFAFA
>ID40178-NO
SPYRFFGTR
>ID40179-NO
SPYRTFAFA
>ID40189-NO
SQAYDPYSNAAQFQLSSQSRGYPYQHRLVY
>ID40190-NO
SQCCAVKKNCCHV
>ID40191-NO
SQEPPISLDLTFHLLREVLEMTKADQLAQQAHSNRKLLDIA
>ID40193-NO
SQKGVYASQRSFVPSWFRKIFRN
>ID40194-NO
SQLGDLGSGAGQGGGGGGSIRAAGGAFGKLEAAREEEFFYKKQKEQLERL
>ID40197-NO
SQPFSFGL
>ID40200-NO
SQPNFLRFG
>ID40203-NO
SQPSKNYLRF
>ID40204-NO
SQPSMRLRF
>ID40210-ABP_both
SQSSVDKLNWYKQRK
>ID40212-NO
SQWNSPPSSAAF

>ID40213-NO
SQWSPCSRTCGGGVSFRER
>ID40216-NO
SQYTFGL
>ID40219-NO
SRAHHCRSKAKRSRHH
>ID40220-ABP_neg
SRATWARVRRLGLYG
>ID40221-NO
SRAVASCLKGWPASGCK
>ID40222-NO
SRCFPPGIYCTPYLPCCWGICCDTCRNVCHLRF
>ID40223-NO
SRCFPPGIYCTPYLPCCWGICCDTCRNVCHLRI
>ID40227-NO
SRCKTGLCQ
>ID40229-NO
SRDLICLCRNRRCNRGELFYGTCAGPFLRCCRRRR
>ID40230-NO
SRDLICYCRKGGCNRGEQVYGTCSGRLLYCCPRR
>ID40231-NO
SRESPHP
>ID40232-NO
SRFVGGSRY
>ID40235-NO
SRGKQ
>ID40236-NO
SRHDLNS
>ID40237-NO
SRHPKLYFPGIV
>ID40239-NO
SRKAT
>ID40242-NO
SRLLYD
>ID40243-NO
SRLMYD
>ID40251-NO
SRNVT
>ID40252-NO
SRNYLRF
>ID40254-NO
SRPRR
>ID40260-NO
SRPYLFGL
>ID40262-NO
SRRAHCRSKAKRSRHH
>ID40263-NO
SRRHACRSKAKRSRHH
>ID40271-NO
SRRKRQRSNMRI
>ID40273-NO

SRSGRGSGKGGRGGSRGSSGSRGSKGPSGSRGSSGSRGSKGSRGGRSGRG
>ID40276-NO
SRTVRKTSRLWSSLSLNTCNNVHSKS
>ID40279-ABP_both
SRVPLKSPVKIVGSKVMIFA
>ID40280-NO
SRVVKCIGFRPGSLDSRQSC
>ID40282-NO
SRVVKCIGFRPGSPDSRRSC
>ID40283-ABP_both
SRWPSPGRPRPFPG
>ID40284-ABP_both
SRWPSPGRPRPFPGRPKPIF
>ID40285-ABP_both
SRWPSPGRPRPFPGRPKPIFRPR
>ID40289-NO
SRWRWKCSKK
>ID40290-NO
SRWRWKSCKK
>ID40293-NO
SSACVWCV
>ID40294-NO
SSACYWCV
>ID40295-NO
SSADTLISDLLIGETESHPQTRYEDHLVW
>ID40296-NO
SSADTLISDLLIGETESHPQTRYEDQLVW
>ID40298-NO
SSANPQITRKRHKINSFVGLM
>ID40300-NO
SSCQLGDIWGAGDAACSASCIAQGEGYHGGHCNDDSVCVCNY
>ID40301-NO
SSCQLGGIFGAGDAACSASCIRAGTYHGGYCNDKQVCICTH
>ID40303-NO
SSDCFGSRIDRIGAQSGMGCGRRF
>ID40306-NO
SSEDMDRLGFA
>ID40307-NO
SSEDMDRLGFG
>ID40310-NO
SSEDMDRLIGFGFN
>ID40313-NO
SSERWAP
>ID40314-NO
SSERWAPKS
>ID40316-NO
SSEVALAASDKGDEERELLNTLSNLLE
>ID40317-NO
SSFPRY
>ID40321-NO
SSGIENGAFQGMK

>ID40322-NO
SSGQYAFGL
>ID40326-NO
SSGVSLLTSNKDEEQRELLKA
>ID40327-NO
SSGVSLLTSNKDEEQRELLKAISNLLD
>ID40329-NO
SSIQSLLNLPQRF
>ID40333-NO
SSKPYAFGL
>ID40375-NO
SSMISPSYQFEDALGLSDALERA
>ID40376-ABP_both
SSMKLSFRARAYGFRGPGLQL
>ID40381-NO
SSPEALMMTDLMLRENAESFPKYRYDEPFMW
>ID40382-NO
SSPEILDTLVSELLLKESTDQLPQSRYDPSLW
>ID40384-NO
SSPEQTAPSRTLLPHIPLGMDKPDEECRLLIQ
>ID40385-NO
SSPETLISDLLLR
>ID40386-NO
SSPETLISDLLLRESTENIPRSRFEDPSMW
>ID40387-NO
SSPETLISDLLMREGTENVPRTRLEDPS
>ID40389-NO
SSPETLISDLLMRESTGNIPRTRLEDPSMW
>ID40390-NO
SSPETMLSDVWWRENTENIPRSRFEDPPMW
>ID40391-NO
SSPFYGHNFV
>ID40394-NO
SSSEQEEEDVRQVE
>ID40396-NO
SSSIFPPWLSFF
>ID40397-ABP_both
SSSIPIKMVLVRALVFVKSG
>ID40398-NO
SSSMYDRDIMSF
>ID40402-NO
SSTSPHRPRFS
>ID40403-NO
SSVCIPSGQPCPYNEHCCSGSCTYKENENGNTVQRCD
>ID40404-NO
SSVEKNLAACLRDND
>ID40405-NO
SSVIETTKNDVCSTPCTRRYGTYECWHDCLHERYNDGGCVDGRCCCKK
>ID40406-NO
SSWCMRGQYNKICMW
>ID40407-NO

SSYGCYCGAGGQGWPQDASDRCCFEHDCCYAKLTGCDPT
>ID40411-NO
STCAFIM
>ID40420-NO
STDDCSTAGCKNVPCCEGLVCTGPSQGPVCQPLA
>ID40430-NO
STITCYCRSRCRMLEKNSGTCRSSNCTYTLCC
>ID40431-NO
STKLLHE
>ID40432-NO
STLFRF
>ID40433-NO
STLPPPLRFANV
>ID40435-NO
STMPFSGGMYG
>ID40438-NO
STNWSNLRGTW
>ID40439-NO
STNWSSCPTSAW
>ID40440-NO
STNWSSLRSAW
>ID40441-NO
STQMLSPPERPREFRHPNELRQYLKELNEYYAIMGRTRF
>ID40442-NO
STRAMAHLPLRL
>ID40443-NO
STRCQGRGASCRKTMYNCCSGSCNRGRCG
>ID40445-NO
STSCMEAGSYCGSTTRICCGYCAYFGKKCIDYPSN
>ID40446-NO
STSCMKAGSYCRSTTRTCCGYCAYFGKFCIDFPSN
>ID40448-NO
STSLEELANRNAIQSDNVFANCELQKLRLLLQGNINNQLFQTPCELLNFP
>ID40450-ABP_neg
STVATL
>ID40451-NO
STVATSQ
>ID40452-ABP_both
STVKVASKLAVVVSPISKGS
>ID40453-NO
STYQT
>ID40456-NO
SVAALAAQGLLNAP
>ID40458-NO
SVAALAAQGLLYNAPK
>ID40460-NO
SVAKLE
>ID40461-NO
SVAKLEK
>ID40462-NO
SVAKPQTHESLEFIPRL

>ID40464-NO
SVATAPVEAKAVEAGNKDIE
>ID40467-NO
SVAVAGAVIEGATLTFNVLQ
>ID40469-NO
SVCASYF
>ID40470-NO
SVCCSFPDPVGGLCCEAHCQQIGHLEGGQCTAQNVCVCG
>ID40471-NO
SVCCSLPDPWKGFCCEAHCDKIGKPGGQCSDKNVCTCN
>ID40472-NO
SVDEHHGHQ
>ID40473-NO
SVDFDSESPR
>ID40474-NO
SVDMVMKGIKLWPL
>ID40475-NO
SVDNLPPRERKAGCKNFYWKGFTSC
>ID40476-NO
SVDNQQGRERKAGCKNFYWKGPTSC
>ID40477-NO
SVDQDLGPEVPPENVLGAL
>ID40482-NO
SVEGVISTIKDFAVKVCCSVSLKFCCPTA
>ID40485-NO
SVESSNHLPARERKAGCKNFYWKGFTSC
>ID40487-ABP_both
SVETLASQEHLSSLPMDSQETLLRGTR
>ID40490-ABP_pos
SVFGQLVMGLILSSVFYNDD
>ID40495-NO
SVGGSGGNDDNFMRF
>ID40499-NO
SVGNRNFLRF
>ID40500-NO
SVGRNIAVDDRGIFSTLFHAHCCANPICKNTPGC
>ID40501-NO
SVHGLGPVVI
>ID40507-ABP_both
SVIGCWTFSIPPRPCFVK
>ID40508-ABP_both
SVIGKIASKVVPSVYCAISK
>ID40512-NO
SVKPCTGFA
>ID40513-ABP_both
SVKPVGSSVVKGTALVKFFG
>ID40514-ABP_both
SVKVAKSVIPSAVFAGGKVF
>ID40515-NO
SVLEIGLMLQEETEKNPKTSYSI
>ID40517-NO

SVLGKSVAKHLPHVVPVIAEKTG
>ID40519-NO
SVLGSVAKHVLPHVVPVIAEHL
>ID40520-NO
SVLTPSLSSLGESLESGIS
>ID40521-NO
SVLTTLKTDGGLRIFKDAPNEF
>ID40524-NO
SVNPYLQGKRLDNVVA
>ID40527-NO
SVPFKPRL
>ID40529-NO
SVPGVLRFG
>ID40530-NO
SVPHFSDEEE
>ID40532-ABP_both
SVPSVGAVLFFKRAAVMKLI
>ID40534-NO
SVQDNFIRF
>ID40535-NO
SVQDNFMHF
>ID40536-NO
SVQFKPRL
>ID40540-NO
SVRTQDNAVNRQIFGSNGPYRDFQLSDCYLPLETNPYCNEWQFAYHWNNA
>ID40543-NO
SVSFQELKDWGAKNVIKMSPAPANKVPHSAANLPLRF
>ID40544-NO
SVSGGGHHHHHHGGG
>ID40545-NO
SVSQLNQYAGFDTLGGMGL
>ID40547-NO
SVSRAGSPSGGPFC
>ID40551-NO
SVVPSPKVSDTVVEPYNA
>ID40555-NO
SWAQHLSLPPVL
>ID40556-NO
SWCQFEKCL
>ID40560-ABP_both
SWFHWK
>ID40561-ABP_pos
SWFKHKSK
>ID40562-ABP_pos
SWFKTKSK
>ID40564-NO
SWGAPAEKFWMRAMPQRF
>ID40573-NO
SWLAYPGAVSYR
>ID40579-NO
SWLPYPWHVPSS

>ID40633-NO
SWTQLHGVW
>ID40636-NO
SWWTPWHVHSES
>ID40647-NO
SYDILKPNPQRL
>ID40653-NO
SYGWAEGDTTDNEYLRF
>ID40659-NO
SYIQRTPSTTLP
>ID40660-NO
SYMEHFRWGKPV
>ID40664-NO
SYPGWSW
>ID40665-NO
SYPLSFLGPLIS
>ID40670-NO
SYRSLLRDGATF
>ID40672-NO
SYSGYPVTQK
>ID40673-NO
SYSMEHFRWGKPIGH
>ID40675-NO
SYSMEHFRWGKPMGRKRRPIKVYPNSFEDESVENMGPEL
>ID40677-NO
SYSMEHFRWGKPVGKKRRPIKVFPTDAEEESSESFPIEL
>ID40679-NO
SYSMEHFRWGKPVGKKRRPVKVYP
>ID40685-NO
SYSMEHFRWGKPVGRKRRPVKVYPNGVEEESAEAYPTEM
>ID40687-NO
SYSMEHFRWGKPVGRKRRPVKVYTSNGVEEESAEVFPGEM
>ID40690-NO
SYWKQCAFNAVSCF
>ID40694-NO
TAASGVRSMH
>ID40697-NO
TADDNNQVLEHRNLAQQLNIPIL
>ID40701-NO
TAFGLRKCCKRHHGCHPC
>ID40702-NO
TAFGLRLCCKRHHGCHPCGRT
>ID40703-NO
TAGFGVFTNIINGGLECGK
>ID40704-NO
TAIRKCNPRT
>ID40705-NO
TAKTRYKARRAELIAERRGC
>ID40706-ABP_both
TAKVVVFVSFSYVVPKKRAC
>ID40707-ABP_neg

TALKKRRWKKAKPP
>ID40708-NO
TALSPQ
>ID40712-NO
TAPYAFGL
>ID40714-NO
TARGSSR
>ID40715-NO
TARRITPKDVIDVRSVTTEINT
>ID40717-ABP_both
TASQAEWFKARRWQWRMKKLGA
>ID40718-NO
TATCAGQDKPCQKHCDCCGPKGECVCEGPCICRQG
>ID40719-NO
TATEECEEYCEDEEKTCCGEEDGEPVCARFCL
>ID40723-NO
TATTRRRGRPPGFSPFR
>ID40725-NO
TAWGPCSTTCGLGMATRV
>ID40743-NO
TCCKFQFLNFCCNE
>ID40744-NO
TCCPPGICSSDKCSCC
>ID40745-NO
TCCVRPWCDGACDCCVDP
>ID40747-ABP_pos
TCDLLSPFKVGHAACALHCIAMGRRGGWCDGRAVCNCRR
>ID40749-NO
TCDPYYCNDGKVCCPEYPTCGDSTGKLICVRVTD
>ID40750-NO
TCEPSGKPCRPLMRIPCCGSCVRGKCA
>ID40760-NO
TCKQKGEGCSLDVECCSSSCKPGGPLFDFDC
>ID40762-NO
TCLARDELCGASFLSNFLCCDGLCLLICV
>ID40764-NO
TCLGKNALCGAPGVGVLVCCSFKCVVVCV
>ID40768-NO
TCNTPTQYCTLHRHCCSLYCHKTIHACA
>ID40771-NO
TCRGLERA
>ID40772-NO
TCRSSGRYCRSPYDCCRRYCRRITDACV
>ID40774-ABP_neg
TCRTNRPCFYDLDLNVCRCS
>ID40786-NO
TCRYWCKTPENQTYCCEDEREIPSKVGLKPGKCPPVRPVCPPTRGFFEPP
>ID40790-NO
TCSIPYEYSNGKLKRTLYYSNGVYANSFTENN
>ID40794-NO
TCSNKGQQCGDDSDCCWHLCCVNNKCAHLILLCNL

>ID40796-NO
TCSPAGEVCTSKSPCCTGFLCSHIGGMCHH
>ID40800-NO
TCSSPSNCPTGQECCPDKVDEPEGFCADECIIT
>ID40807-NO
TCVGGTCNTPGCTCSWPVCTRNLPVCG
>ID40808-NO
TCVMFGSMCDKEEHSICCYECDYKKGICV
>ID40810-NO
TDCTPSRCT
>ID40812-NO
TDDKKLKACGRDYVRLQIEVCGSIWWGRKAGQLRE
>ID40813-NO
TDEPEEHDELGGNGCCGNPDCTSHSCD
>ID40814-NO
TDGFAFSPRL
>ID40818-NO
TDLGAM
>ID40819-NO
TDMKDESDRGAHSERGALWFGPRL
>ID40825-NO
TDQHQDKIYP
>ID40826-NO
TDRNFIRL
>ID40827-NO
TDRNFLRL
>ID40828-NO
TDSEECCLDSRCAGQHQDLCS
>ID40829-NO
TDSILRSYDWTY
>ID40833-NO
TDVEK
>ID40836-NO
TECDMSRCM
>ID40837-NO
TEDELQDKIHP
>ID40840-NO
TEENRELVSELKRP
>ID40850-NO
TEWSACNVRCGRGWQKRSR
>ID40851-NO
TEWSACSKTCGMGISTRV
>ID40852-NO
TEWSVCNSRCGRGYQKRTR
>ID40853-NO
TEWTACSKSCGMGFSTRV
>ID40875-NO
TFFYGGCRGKRNNFKTEEY
>ID40878-NO
TFFYGGSRGRRNNFRTEEY
>ID40880-ABP_both

TFKRKNGSRKNGHRPGGYSLIALGNKKVLKAPYMESI
>ID40881-ABP_neg
TFLKKRRWKKAKPP
>ID40882-NO
TFPHGP
>ID40883-NO
TFPQTAIGVGAP
>ID40889-NO
TGALVEEEDPF
>ID40890-NO
TGALVQQQDP
>ID40891-NO
TGASSEEEDPF
>ID40894-ABP_both
TGCFQWQRN
>ID40897-NO
TGGGNA
>ID40898-NO
TGGGNV
>ID40902-NO
TGPIPN
>ID40905-NO
TGPSASSGLWFGPRL
>ID40910-NO
TGVSWSVAQPSF
>ID40913-NO
TGWSSTSRAW
>ID40914-NO
TGWSVFQGSW
>ID40916-NO
THDFMRF
>ID40917-ABP_both
THIHMNARLLIRSPFTDPQL
>ID40918-NO
THIKWGD
>ID40919-ABP_pos
THILLLRLRKKVMS
>ID40921-NO
THPFLRF
>ID40922-NO
THPTYSFGL
>ID40923-ABP_neg
THRLRRWCRARGLAR
>ID40933-NO
TICYNHLTIRSEVTEICIICDDDYYF
>ID40934-ABP_neg
TIDFGVRNINQSNLVYDTER
>ID40936-NO
TIINVK
>ID40938-NO
TIINVKCTSPKQCLPPCKAQFGQSAGAKCMNGKCKCYPH

>ID40940-NO
TIINVKCTSPKQCSKPCKEL
>ID40941-NO
TIINVKCTSPKQCSKPCKELYGSSAGAKCMNGKCKCYNN
>ID40946-NO
TIPDRLPQTEESSLPDFGFSHLPALPLEL
>ID40950-NO
TISCTNPKQCYPHCKKETGYPNAKCMNRKCKCFGR
>ID40951-ABP_both
TISQAEWFKARRWQWRMKKLGA
>ID40957-NO
TKAQGDFNEF
>ID40960-ABP_both
TKCFGWGRN
>ID40961-ABP_both
TKCFQWQGN
>ID40962-ABP_both
TKCFQWQRN
>ID40965-ABP_both
TKCFQWQWNMRKYRG
>ID40972-NO
TKIPA
>ID40975-NO
TKIVP
>ID40976-NO
TKKFPNRRKTAFCSNWNQKIKTV
>ID40995-ABP_both
TKLTEEEKNRLNFLKKISQRYQ
>ID40996-NO
TKMMLPALRQQPCCSPSTCDGGCYHCC
>ID41002-NO
TKPRKTKPRKTKPRKTKPR
>ID41003-NO
TKPRPGP
>ID41004-NO
TKRRITPDDVIDVRSVTTEINT
>ID41006-NO
TKRRITPKDVIDVESVTTEINT
>ID41007-NO
TKRRITPKDVIDVRSVTTEINT
>ID41011-ABP_both
TKRSLSPHRPRHSRLQREPQVQWLEQQVAKRRTKR
>ID41014-NO
TKTGRSAHVLSRYRPRA
>ID41016-NO
TKVIP
>ID41017-ABP_both
TKVIPYVRYL
>ID41018-ABP_both
TKVIPYVRYLFFFFF
>ID41019-ABP_both

TKVIPYVRYLWWWWW
>ID41021-NO
TKWTPCSRTCGMGISNRV
>ID41025-ABP_both
TLEEFSAKL
>ID41026-NO
TLFRF
>ID41031-ABP_both
TLKQFAKGVGKWLVK
>ID41033-ABP_neg
TLLKKRRWKKAKPP
>ID41037-NO
TLNINRLILPRT
>ID41038-NO
TLPFAYCNIHQVCHYAQRNDRSYWL
>ID41049-NO
TLQPPASSRRR
>ID41050-NO
TLQPPASSRRRHFHHALPPAR
>ID41051-NO
TLQPPASSRRRHFHHALPPARHHPDLEAQARR
>ID41053-NO
TLQRHWAKSLCCPEDAWCCSHDE
>ID41054-NO
TLQRLSIEYSCCPGIVSCCVIP
>ID41058-ABP_neg
TLSKEKERIVQRVRTS
>ID41059-NO
TLSMLRL
>ID41060-NO
TLSRVPSLPQRF
>ID41064-NO
TLTVLPW
>ID41073-NO
TLTYTWS
>ID41074-ABP_both
TLVGVVAKLVATKIGSSPRA
>ID41079-NO
TMCYSHTTTSRAILTNCGENSCYRKSRVHP
>ID41081-NO
TMNFSPRL
>ID41082-NO
TMPFLFCNVNDCNFASRNDYSYWL
>ID41083-NO
TMSFSPRL
>ID41086-NO
TNCEAHSCSPSCPDECYCDTNEDTCHPE
>ID41088-NO
TNDFMRF
>ID41093-NO
TNEIVEEQYTPQS

>ID41095-NO
TNEIVEEQYTPQSLATLE
>ID41096-NO
TNEIVEGQYTPQSLATLQSVFQELGKLKGQANN
>ID41097-NO
TNFAFSPRL
>ID41100-NO
TNGIIR
>ID41102-NO
TNIMGENRLNRNL
>ID41106-NO
TNRNFLRF
>ID41107-NO
TNRNYGKPNKDIGTCIWSGFRHC
>ID41108-NO
TNTPEDDRYLQDYVYI
>ID41109-NO
TNTPEQQCYLQAKCYIEFYVVV
>ID41119-NO
TNYGGFLRF
>ID41121-NO
TPAEDFMRF
>ID41125-NO
TPDHQRYVELFIVVDHGMYTKYNGD
>ID41127-NO
TPEHQRYIELFLVVDSGMFMKYNGNSDKI
>ID41130-NO
TPEVDDEALE
>ID41131-NO
TPFAIKCATDADCSRKCPGNPPCRNGFCACT
>ID41134-ABP_both
TPFLLVGTQIDLR
>ID41135-NO
TPFTPRL
>ID41137-NO
TPGGFDIISGGPHVAQDVLNAIKDFFK
>ID41138-NO
TPGQDFMRF
>ID41141-NO
TPHWRPQGRF
>ID41142-NO
TPIAEAQGAPEDVDDRRELE
>ID41144-ABP_both
TPIESHQVEKRKSNT
>ID41147-NO
TPKTMTQTYDFS
>ID41150-NO
TPLRGFEGETGHPLE
>ID41152-NO
TPMNHHSQHAER
>ID41155-NO

TPNRDFMRF
>ID41156-NO
TPNSRAFLGMR
>ID41158-NO
TPPAGPDVGPR
>ID41159-NO
TPPEEDLLGRFT
>ID41161-NO
TPPQPADNFIRF
>ID41164-NO
TPQANSFGL
>ID41165-NO
TPQNWNKLNSLW
>ID41168-NO
TPRDIANLY
>ID41174-NO
TPRNLRTSNTHR
>ID41176-NO
TPRTQKA
>ID41177-NO
TPRVF
>ID41178-NO
TPSDGFMGMR
>ID41179-NO
TPSGFLGMR
>ID41180-NO
TPSLSSLGESLESGIS
>ID41181-NO
TPSPR
>ID41183-NO
TPSYAFGL
>ID41185-NO
TPTHDFMRF
>ID41186-NO
TPTQSSDFMRF
>ID41188-ABP_pos
TPVVNPPFLQ
>ID41189-NO
TPVVVPPFLQP
>ID41190-NO
TPWVPPFLQP
>ID41191-NO
TPYPVNCKTDRDCVMCGLGISCKNGYCQGCT
>ID41194-NO
TQPADLQTHNHN
>ID41197-ABP_both
TQQAFQKFLAAVTSALGKQYH
>ID41198-NO
TQQPLEGHQLPY
>ID41200-NO
TQSLVYP

>ID41201-NO
TQSPTFDRQD
>ID41202-NO
TQTPVVVPPFIQPE
>ID41204-NO
TQWSKFQGSW
>ID41205-NO
TQWTSCSKTCNSGTQSRHR
>ID41210-NO
TRCKGKGAPCRKTMYDCCSGSCGRRGKCG
>ID41212-NO
TRCPMIPCYI
>ID41215-NO
TRDQQ
>ID41216-ABP_neg
TRDTNDLISSRTAAPSMV
>ID41220-NO
TRGCKSKGSFCWNGIECCGGNCFFACVY
>ID41221-NO
TRGCKTKGTWCWASRECCLKDCLFVCVY
>ID41222-NO
TRGGLPV
>ID41225-ABP_both
TRGRWGRFKRRAGRFIRRNRWQIISTGLKLIG
>ID41227-NO
TRICCGCYWNGSKDVCSQSCC
>ID41229-NO
TRIMDDSSDCVFKGPCQRRSDCYERCGLKPPSRAALCQPMGLQGRVCCCL
>ID41232-ABP_both
TRKKWFW
>ID41233-NO
TRKLPFNGSIF
>ID41235-ABP_both
TRKRLKKIGKVLKWI
>ID41240-NO
TRNGLPG
>ID41252-NO
TRQARRNRRRRWRERQRGC
>ID41256-NO
TRRQRTRRARRNRGC
>ID41258-NO
TRSAWLNSEVAESGLDGDHLSDFSTTSPELYLR
>ID41259-NO
TRSAWLNSGVAESGLEGDHPYDISATSLELNLR
>ID41260-NO
TRSAWPLSAGAGSGLAGDHLSDISEPEPELDSR
>ID41262-NO
TRSGGACNSHDQCCINFCRKATSTCM
>ID41266-ABP_both
TRSRWRRFIRGAGRFARRYGWRIA
>ID41267-ABP_both

TRSSRAGLQFPVGRVHR
>ID41273-ABP_both
TRVVWCAVG
>ID41279-ABP_both
TSASVSLQVCRVDHTCKDAGYF
>ID41281-NO
TSAVRT
>ID41282-NO
TSCDPSLCE
>ID41283-NO
TSDCCFYHNCCC
>ID41286-NO
TSFAFSPRL
>ID41287-ABP_both
TSFLNELNKYNEKKFI
>ID41288-NO
TSFTPRL
>ID41289-ABP_both
TSGKYPNCRYKEKRQNKSYVVACKPPQKKDSQQFHLVPVHLDRVL
>ID41290-NO
TSGLIAFPRL
>ID41291-NO
TSHTDAPPARSP
>ID41292-NO
TSISRSKDAKKPSLDILEGAGFSPL
>ID41295-NO
TSLDASIIWAMMQN
>ID41296-ABP_both
TSLGGWKLIQQKMDK
>ID41300-NO
TSMGFQGVR
>ID41304-NO
TSPLNIHNGQKL
>ID41305-NO
TSPMEPSAALAVEHGTTHPLE
>ID41306-NO
TSPTNRS
>ID41309-NO
TSRCYIEYRRKVVCS
>ID41310-NO
TSRCYVGYQRKIVCT
>ID41311-NO
TSRCYVGYRRKIVCS
>ID41313-NO
TSRSYSINPYSFGL
>ID41315-NO
TSSLFPHPRL
>ID41323-ABP_neg
TSVRQRWRWRQRVRTS
>ID41324-NO
TSWFKFQGSW

>ID41325-NO
TSWGKFQGSW
>ID41326-NO
TSWGKYQGSW
>ID41327-NO
TSWSPCSASCGGGHYQRTR
>ID41328-NO
TSWSQCSKTCGTGISTRV
>ID41333-ABP_neg
TTAPCKCWIGLRRCFK
>ID41334-NO
TTDEKCNEYCEERDRNCCGKANGEPRCARMCF
>ID41335-NO
TTEECCPFIVGCCS
>ID41337-NO
TTENVLFG
>ID41343-NO
TTITGKKCQSWAAMFPHRHSKT
>ID41344-NO
TTMLIQDEDDLEMA
>ID41349-NO
TTQHTCSILSRPHPRGLCGSTLANMVQWLCSTYTTSS
>ID41351-NO
TTREKNL
>ID41352-NO
TTRFQDSRSKDVYLIDYPEDY
>ID41353-NO
TTRPQPFNFGL
>ID41395-NO
TVDFGLSRGYSGAQEAKHRMAMAVANFAGGP
>ID41397-NO
TVDQHQ
>ID41403-NO
TVIDVKCTSPKQCLPPCAKQ
>ID41404-NO
TVIDVKCTSPKQCLPPCKAQFGIRAGAKCMNGKCKCYPH
>ID41405-NO
TVIMHNCCTRSFCKRIYPDLCS
>ID41406-NO
TVKCGGCNRKCCAGGCRSGKCINGKCQCY
>ID41408-NO
TVKLTPRL
>ID41417-NO
TVRDACCSDPRCSGKHQDLC
>ID41418-NO
TVREACCSDPRCSGQHQEKC
>ID41419-NO
TVRGFCSDPSCRFRNPELCDW
>ID41422-NO
TVSQECCLDTRCAGKNLDEC
>ID41425-NO

TVTEECEEDCEDEEKHCCNTNNGPSCARLCF
>ID41426-ABP_both
TVVAGYNLYRAIKKK
>ID41427-ABP_both
TVVANA
>ID41428-NO
TVVPG
>ID41429-ABP_both
TVVRRRGRSPRRR
>ID41430-ABP_both
TVVRRRGRSPRRRTPSPRAARSQSPRAARSQSRESQC
>ID41440-NO
TVWGFRPTNPKPKPPG
>ID41441-NO
TVWNPVG
>ID41445-NO
TVYTKGRVMP
>ID41447-ABP_neg
TWAKKRRWKKAKPP
>ID41453-NO
TWGHLRA
>ID41457-NO
TWKNLQGGW
>ID41458-NO
TWKSPDIVIRF
>ID41459-NO
TWLAAKRWAAAKPP
>ID41460-ABP_neg
TWLAKRRWKKAKPP
>ID41461-ABP_neg
TWLKARRWKKAKPP
>ID41484-NO
TYLGS
>ID41488-ABP_both
TYVSYT
>ID41496-NO
VAAGRPRF
>ID41497-NO
VACVYRTCDKDCTSRKYRSGKCINNACKCYPY
>ID41500-NO
VADKRPYILHEKKSIPY
>ID41501-NO
VADKRPYILREKKSIPY
>ID41502-NO
VADVYVGK
>ID41504-NO
VAEECEESCEDEEKHCCNTNNGPSCAPQCF
>ID41508-NO
VAGALGVQTAAATTIVNVILNAGTLVTVLGIIASIASGGAGTLMTIGWAT
>ID41511-NO
VAGTW

>ID41515-NO
VAHINVWK
>ID41517-NO
VAIYGRDLRSDVCRQVQHNWLVCDTY
>ID41520-ABP_both
VAKGLIKGVKAKGELPAKGVFKGLKESIGKRAVLKG
>ID41525-ABP_both
VALNENLLR
>ID41528-ABP_both
VALRLAKEVIKVQQGW
>ID41532-ABP_both
VANENLLR
>ID41534-NO
VAPAQHLCGSHLVDALYLVCGDRGFFYNP
>ID41535-NO
VAPEEHPT
>ID41536-NO
VAPFPEV
>ID41543-NO
VARPPGFTPFRVAPEIV
>ID41547-NO
VASVSVA
>ID41550-NO
VATSEPNRYFNPYSYVELIITVDHS
>ID41551-NO
VATSEQFNKTFIELVIVVD
>ID41556-NO
VAWKL
>ID41566-NO
VCCPFGGCHELCLCCD
>ID41568-NO
VCCPFGGCHELCQCCE
>ID41572-NO
VCCPPESCTDRCLCCL
>ID41586-NO
VCIADDMPCGFGLFGGPLCCSGWCLFVCL
>ID41587-ABP_both
VCNEIGLLKSLCKKFVKGHLGE
>ID41588-NO
VCRDWFKETACRHAKSLGNCRTSQKYRANCAKTCELC
>ID41593-NO
VCTPPEGYCTYHRDCCDLYCNKTTNVCLET
>ID41594-NO
VCTRNGLPVCGETCVGGTCNTPGCTCS
>ID41595-NO
VCVDGGTFCGFPKIGGPCCSGWCIFVCL
>ID41596-ABP_both
VCWRIRVAVIRA
>ID41602-ABP_both
VDDDDK
>ID41607-NO

VDFEG
>ID41608-NO
VDHLWQVWLPR
>ID41611-NO
VDKEA
>ID41612-ABP_both
VDKGRYLEAPTPPRPIYNRN
>ID41613-ABP_both
VDKGSYLPRPTPTPRYRPIYNRN
>ID41614-ABP_both
VDKGSYLPRPTPYSPRPPIYNRN
>ID41616-ABP_both
VDKPPYLPRPHPPRRIYNNR
>ID41617-ABP_both
VDKPPYLPRPKPPRRIYNNR
>ID41618-ABP_both
VDKPPYLPRPRPPRAIYNR
>ID41632-NO
VDKRRPIWVM
>ID41635-ABP_both
VDNANDLLSKVKKDKSD
>ID41637-ABP_neg
VDQHQKAMKPWTQPKTNAIPYVRYL
>ID41639-ABP_both
VDRGWGNGCGLFGKGG
>ID41642-NO
VDWICKDCANIFR
>ID41648-NO
VECYGPNRPQF
>ID41649-NO
VECYLIRDNLCIY
>ID41652-NO
VEELKP
>ID41654-NO
VEFEG
>ID41660-NO
VELSEWGVPCATCILDRR
>ID41662-NO
VENLHLPLPL
>ID41665-NO
VEPEEEANGGEILA
>ID41668-ABP_both
VERIPLVRFKSIKKQLHERGDL
>ID41669-NO
VERYAFGL
>ID41671-ABP_neg
VESTVATL
>ID41674-NO
VEVQVRAVGIQGLSPLRQPAP
>ID41677-NO
VEVSDDGSGGNTSLSQ

>ID41682-NO
VFCRFNGQQCTSDGQCCYGKCRTAFLRMICMGG
>ID41683-NO
VFCRFNGQQCTSDGQCCYGKCRTAFMGKICM
>ID41686-NO
VFDSLGGYEVHGF
>ID41688-NO
VFEWAFAR
>ID41689-NO
VFGKEK
>ID41693-NO
VFINAKCRGSPECLPKCKEAIGKAAGKCMNGKCKCYP
>ID41694-NO
VFINVKCRGSPECLPKCKEAFGKAAGKCVN
>ID41695-NO
VFINVKCRGSPECLPKCKEAIGKAAGKCMN
>ID41699-NO
VFKCSYRLGSPDSRCK
>ID41701-NO
VFKCYEPDSRGFQVCE
>ID41732-ABP_both
VFRLKKWMQKVIDRFGG
>ID41733-ABP_both
VFSVKGGKPSVVIKVVVAST
>ID41738-ABP_both
VGALAVVVWKWKWKW
>ID41739-ABP_both
VGALAVVVWKWKWLW
>ID41741-ABP_both
VGALAVVVWKWLWLW
>ID41743-ABP_both
VGALAVVVWRWRWRW
>ID41745-NO
VGARPCGGFF
>ID41746-NO
VGCEECPAHCKGKNAIPTCDDGVCNCNV
>ID41747-NO
VGCEECPAHCKGKNAKPTCDDGVCNCNV
>ID41748-NO
VGCEECPMHCKGKHAVPTCDDGVCNCNV
>ID41749-NO
VGCEECPMHCKGKKALPTCDYGCECND
>ID41751-NO
VGCEECPMHCKGKNAKPTCDNGVCNCNV
>ID41754-NO
VGCEEDPMHCKGKQAKPTCCNGVCNCNV
>ID41757-NO
VGFGKAL
>ID41759-NO
VGGEYDDYGHLRF
>ID41763-NO

VGGPQHLCGSHLVDALYLVCGDRGFFYNPR
>ID41766-NO
VGINYWLAHK
>ID41771-NO
VGLPIF
>ID41776-ABP_both
VGRIHRHLK
>ID41778-NO
VGRPEWW
>ID41781-NO
VGSGGCMFGNGK
>ID41783-NO
VGTALGSLAEELNGYNRKKGGFSFRF
>ID41784-ABP_both
VGTDFSGNDDISDVQK
>ID41787-NO
VGVGEWSV
>ID41788-ABP_neg
VGVKRRLKCLLSLRS
>ID41796-ABP_neg
VHISHREARGPSFRICVGFLGPRWARGCSTGN
>ID41797-NO
VHKMAKNQFGCFANVDVKGDCKRHCKAEDKEGICHGTKCKCGVPISYL
>ID41799-NO
VHLAP
>ID41803-NO
VHLPPPVHLPPP
>ID41804-NO
VHLPPPVHLPPPVHLPPP
>ID41807-NO
VHMNCSWMRVSEGHPCESAD
>ID41810-NO
VHRKC
>ID41815-NO
VHSSIQSQPQAFT
>ID41817-NO
VHTNIPCRGTSDCYEPCEKKYNCARAKCMNRHCNCYNNCPW
>ID41821-NO
VHYEA
>ID41823-ABP_both
VIAFAKTKEAKAKLKGQAKG
>ID41828-NO
VICYRGYNYAQPCPPGENVCFTKTWCDARCYQLGK
>ID41830-ABP_both
VIDKAKMESLGITSRDTT
>ID41832-NO
VIDSCCSNPPCAHVHCH
>ID41833-NO
VIEKYP
>ID41834-NO
VIEPKCYKYEGKKCPPDINPVCGTDKRTYYNECALCVFIRQSTKKADKAI

>ID41838-NO
VIFEWTLLQVLSESDQDQSLEVFLT
>ID41839-NO
VIFTPKL
>ID41841-NO
VIGGAKCNINEHRSIVLLYSSRLFGHTLINKEWVL
>ID41842-NO
VIGGDECDINEHPFL
>ID41843-NO
VIGGDECNIN
>ID41844-NO
VIGGDECNINEHPFLA
>ID41846-NO
VIGGDECNINEHRFLAALYD
>ID41847-NO
VIGGDECNINEHRFLVALYDGLSGTFLCGG
>ID41848-NO
VIGGDECNINEHRFLVALYDPDGFLSGGIL
>ID41849-NO
VIGGDECNINEHRSL
>ID41851-NO
VIGGDECNINEHRSLVAIFDS
>ID41852-NO
VIGGDICNINEHNFLVALYE
>ID41853-NO
VIGGVECDINEHRFL
>ID41855-NO
VIGSPPEIN
>ID41856-NO
VIGSPPQIN
>ID41860-ABP_both
VIHKLAKLAKKLAKLAK
>ID41870-ABP_pos
VIKWLLKILRAI
>ID41876-NO
VINDDCPNLIGNR
>ID41877-NO
VINDDCPNLIGNRDLYK
>ID41878-ABP_both
VINENLLR
>ID41880-NO
VIPAAY
>ID41881-NO
VIPEL
>ID41882-NO
VIPFVAMWLPK
>ID41884-NO
VIPFVASVAAEMMQHVYCAASKKMLKLNWKSSDVENHLAKC
>ID41887-NO
VIPPWARIYYG
>ID41892-NO

VIRVHFRLPVRTV
>ID41904-NO
VKAGE
>ID41905-NO
VKAGF
>ID41915-NO
VKEDQ
>ID41916-ABP_both
VKEEVKFSKKW
>ID41919-NO
VKELQ
>ID41920-NO
VKELY
>ID41922-NO
VKETT
>ID41934-NO
VKHWPW
>ID41940-ABP_neg
VKKFAWWWPFLKK
>ID41941-ABP_neg
VKKFPWWWAFLKK
>ID41943-NO
VKKKQCCNPACGPKYSCGH
>ID41946-NO
VKKTC
>ID41947-NO
VKKVLGNP
>ID41950-ABP_both
VKKYPKVKLYP
>ID41953-ABP_both
VKLKKYPKLKVKLYP
>ID41954-ABP_both
VKLKVKYPKLKVKLYP
>ID41968-ABP_both
VKLKYPVKLYP
>ID41969-NO
VKLPPP
>ID41970-NO
VKLPPPVKLPPP
>ID41971-NO
VKLPPPVKLPPPVKLPPP
>ID41974-ABP_both
VKLVKYPKLKVKLYP
>ID41979-NO
VKNDT
>ID41980-NO
VKNEE
>ID41985-NO
VKPCRKEGQLCDPIFQNCCRGWNCVLFCV
>ID41986-NO
VKPCSEEGQLCDPLSQNCCRGWHCVLVSCV

>ID41990-ABP_both
VKRAGKGVAVVPSPLFKIVV
>ID41991-NO
VKRCCDEEECSSACWPCCWG
>ID41994-ABP_both
VKRFKKFFRKLKKS
>ID42001-NO
VKRKKKPALWKTLLKKVLKA
>ID42009-ABP_both
VKRWKKWRWKWKKWV
>ID42010-ABP_both
VKRWKKWWRKLKKSV
>ID42014-ABP_both
VKSWIRKLVHR
>ID42016-ABP_both
VKTKCSVPAVVYILVKTFKS
>ID42027-ABP_both
VKWRWKWKWRWKWKWKV
>ID42030-ABP_both
VKYLEFKSESIKQVK
>ID42035-NO
VLAQYK
>ID42038-ABP_pos
VLASKNFGDK
>ID42040-NO
VLDTDYK
>ID42041-NO
VLEKDCPPHPVPGMHKCVCLKTC
>ID42042-NO
VLFTPPELLGCGNRCSDDCCKWGRCQPGCTD
>ID42046-NO
VLGPVLGLASNALGGLIKKI
>ID42048-NO
VLGPVRGPFP
>ID42049-NO
VLGPVRGPFPIIV
>ID42050-NO
VLGQSGYLMPMR
>ID42052-NO
VLGWNKAHGLWG
>ID42061-NO
VLIVP
>ID42063-ABP_pos
VLKIKVKIWVVK
>ID42068-ABP_both
VLKQTKGVGASGSFRLAKSD
>ID42069-NO
VLLFLFQAAPGSADAPFADTAACRSQGNFCRAGACPPTFAASGSCHGGLL
>ID42070-ABP_both
VLLRALARKITLGIKKYG
>ID42074-NO

VLMRFG
>ID42077-ABP_both
VLNANLLR
>ID42078-ABP_both
VLNDNLLR
>ID42079-ABP_both
VLNEALLR
>ID42080-ABP_both
VLNENAAR
>ID42081-ABP_both
VLNENALR
>ID42082-ABP_both
VLNENARL
>ID42083-NO
VLNENL
>ID42085-ABP_both
VLNENLAR
>ID42086-ABP_both
VLNENLL
>ID42088-ABP_both
VLNENLLH
>ID42091-ABP_both
VLNNLLR
>ID42095-NO
VLPEI
>ID42099-NO
VLPIP
>ID42100-NO
VLPIPQ
>ID42103-ABP_neg
VLPIVKKVLRGLF
>ID42104-ABP_both
VLPLLETCSMTCWENNQTFGK
>ID42105-NO
VLPLVGNLLNDLLGE
>ID42107-NO
VLPVPQ
>ID42108-NO
VLPVPQK
>ID42110-NO
VLPYP
>ID42111-NO
VLPYPV
>ID42113-ABP_both
VLSAADKGNVKAAWGKVGGHAAEYGAE
>ID42114-ABP_both
VLSAADKGNVKAAWGKVGGHAAEYGAEA
>ID42116-ABP_both
VLSAADKGNVKAAWGKVGGHAAEYGAEALERM
>ID42118-ABP_both
VLSAADKGNVKAAWGKVGGHAAEYGAEALERMFLSFPTTKTYFPHF

>ID42120-NO
VLSMDGYQNILDKKDELLGEWE
>ID42122-NO
VLTVQPW
>ID42124-ABP_pos
VLVLDTDYK
>ID42125-ABP_both
VLVLDTRYKK
>ID42126-ABP_both
VLVPQGFAVVKKANFVAFYT
>ID42129-NO
VLYRDG
>ID42130-NO
VMDKPQG
>ID42136-NO
VMIGVESARDAYIAKPHNCV
>ID42143-NO
VNADIKATTVFGGKYVSLTTP
>ID42144-NO
VNAGPAQWNKFRGSW
>ID42145-NO
VNCCGIDESCCS
>ID42146-NO
VNCCPIDESCCS
>ID42154-ABP_both
VNFKLLSHSLL
>ID42155-ABP_both
VNFLLHKIYDNIRYS
>ID42158-NO
VNFSPNW
>ID42163-NO
VNLDPNSFRMSF
>ID42170-NO
VNSHQALWSPAQ
>ID42173-ABP_both
VNWKKILG
>ID42190-NO
VPALK
>ID42191-NO
VPALR
>ID42196-NO
VPCQKRPGWVCLW
>ID42197-NO
VPCRFKQCW
>ID42199-NO
VPDPR
>ID42200-NO
VPEECEESCEEEEKTCCGLENGQPFCSRICW
>ID42201-NO
VPEQRPM
>ID42202-NO

VPERAPLPPSVPSQFQ
>ID42204-NO
VPFEF
>ID42205-NO
VPFGVG
>ID42207-NO
VPGGDECNINEHRSL
>ID42210-NO
VPHSAANLPLRF
>ID42219-NO
VPKVK
>ID42220-NO
VPKVKE
>ID42221-NO
VPKVKET
>ID42226-NO
VPLQDYHTSTETVEGLLARGQGFTTA
>ID42232-NO
VPNGFLGVR
>ID42233-NO
VPNLPQRF
>ID42234-NO
VPNSVANLPLRF
>ID42238-NO
VPPFLQPEV
>ID42239-NO
VPPGFSPFR
>ID42242-NO
VPPIPP
>ID42245-NO
VPQPIP
>ID42246-NO
VPRRKAKI
>ID42247-NO
VPRYAFG
>ID42248-NO
VPSAADMMIRF
>ID42250-NO
VPSAGDMMVRFG
>ID42252-NO
VPSERYL
>ID42254-NO
VPSGFTGMR
>ID42256-NO
VPTGFTPFR
>ID42258-NO
VPTLE
>ID42259-NO
VPTLK
>ID42260-NO
VPTLQ

>ID42261-NO
VPTQRLCGSHLVDALYFVCGERGFFYSPKQI
>ID42267-NO
VPWTPSPRL
>ID42269-ABP_both
VPYPQRDMPIQAFL
>ID42271-NO
VQAILRRNWNQYKIQ
>ID42272-NO
VQESADGYRMQHFRWGQPLP
>ID42277-NO
VQKVDGESRAHLGALLARYIQQARKAPSGRVSMIKNLQSLDPSHRISDR
>ID42278-ABP_both
VQLRCRVCVIRK
>ID42280-ABP_both
VQLRIRVCVIRK
>ID42283-NO
VQNLATFKTMMRY
>ID42284-ABP_both
VQNQHGQVVKIFHH
>ID42286-NO
VQRKRQKLMP
>ID42288-ABP_both
VQWLEQQVAKRRTKR
>ID42290-ABP_both
VQWRIRIAVIRK
>ID42291-ABP_both
VQWRIRVAVIRK
>ID42292-ABP_both
VQWRIRVCVIRA
>ID42297-NO
VRAVCTTLKSRGHEECWSLQ
>ID42300-NO
VRDAYIAEDYDCVYHCARDA
>ID42309-NO
VRDAYIAQNYNCVYTCFKNDYCND
>ID42311-NO
VRDFV
>ID42318-NO
VREFV
>ID42323-NO
VRFSPWG
>ID42328-NO
VRGPFP
>ID42329-NO
VRGPFPIIV
>ID42343-NO
VRIWFCASRTCSAPADCNPCTCESGVCVDWL
>ID42346-NO
VRKGQ
>ID42366-NO

VRLPPP
>ID42367-NO
VRLPPPVRLPPP
>ID42368-NO
VRLPPPVRLPPPVRLPPP
>ID42369-NO
VRLPT
>ID42517-NO
VRMPQ
>ID42523-NO
VRNSLRN
>ID42524-NO
VRPEK
>ID42529-NO
VRRFLVTLRIRRA
>ID42544-ABP_neg
VRRVWRRVVRVVRRWVRRVRRWVRRVVRVVRRWVRR
>ID42554-ABP_both
VRVKVRVK
>ID42555-ABP_both
VRVKVRVKVRVK
>ID42567-NO
VSALK
>ID42571-NO
VSCEDCPDHCSTQKARAKCDNDKCVCEPI
>ID42573-NO
VSCEDCPEHCATKDQRAKCDNDKCVCEPK
>ID42574-NO
VSCEDCPEHCSTQKARAKCDNDKCVCESV
>ID42578-NO
VSCTGSKDCYAACRKQTGCANAKCINKSCKCYGC
>ID42579-NO
VSCTGSKDCYAPCRKQTGCPNAACINKSCKCYGC
>ID42580-NO
VSCTGSKDCYAPCRKQTGCPNAKCINKSC
>ID42583-NO
VSCTGSKDCYAPCRKQTGCPYGKCMNRKCKCNRC
>ID42587-NO
VSDRLRWCVPSGEVCRRYEFVGCCSGKCFFVCS
>ID42589-NO
VSEACEESCEDEEKHCCHENNGVYTCLRYCW
>ID42591-NO
VSFLEYR
>ID42592-NO
VSGKK
>ID42598-NO
VSIGIKCDPSIDLCEGQCRIRYFTGYCSGDTCHCS
>ID42603-NO
VSIWFCASRTCSAPADCNPCTCESGVCVDWL
>ID42608-NO
VSKQPYYMWNGN

>ID42609-NO
VSKVKET
>ID42613-NO
VSLKK
>ID42614-NO
VSLPEW
>ID42620-NO
VSPKYGHNFV
>ID42631-ABP_both
VSVKKVLPFAPLKSLLSFAF
>ID42637-ABP_both
VTADVLSFEAKGIAVNHSACALHCIALRKKGGSAQNGVCVCRN
>ID42639-NO
VTCDLLSFEILGVALNHSACAAHCLAIGRGGGACQGGICVCRR
>ID42642-ABP_both
VTCDLLSFKGQVNDSACAANCLSLGKAGGHCEKGVCICRK
>ID42643-NO
VTCDLLSFKGQVNDSACAANCLSLGKAGGHCEKGVCICRKTSFKDLWDKR
>ID42645-NO
VTCDLLSFSSKIFSFNHSACAAHCLAKRKKGGRCVNGVCRCRN
>ID42646-NO
VTCDLLSGIGWNHTFCAAHCIFKGYKGGACNSKGVCVCRR
>ID42648-NO
VTCDLLSIKGVAEHSACAANCLSMGKAGGRCENGICLCRKTTFKELWDKR
>ID42652-NO
VTCDLLSLQIMGNSFGDSACAAHCIGLHHSGGHCSGGVCVCR
>ID42655-NO
VTCDVLSWQSKWLSINHSACAIRCLAQRRKGGSCRNGVCICRK
>ID42656-NO
VTCELLMFGGVVGDSACAANCLSMGKAGGSCNGGLCDCRKTTFKELWDKR
>ID42657-NO
VTCGDGVITR
>ID42658-NO
VTCGGGVQKRSRL
>ID42662-NO
VTCRSLMCQ
>ID42672-ABP_both
VTLASHLPSDFTPAVHASLDKF
>ID42673-ABP_both
VTLASHLPSDFTPAVHASLDKFLANVS
>ID42676-NO
VTMGYIKDGDGKKIAKKKNKNGRKHVEIDLNKVG
>ID42677-ABP_both
VTNVGGKVVTGKTAK
>ID42679-NO
VTPALR
>ID42680-NO
VTPEQNMTQMDGSASDLLLRLMHMANRQQQSKHQFY
>ID42685-ABP_both
VTRRRHGV
>ID42688-NO

VTSTAV
>ID42692-NO
VTVNPYKWLP
>ID42695-NO
VTVVQYRSTNASSGYLNLRVYLLDTGLR
>ID42696-NO
VTWGKFQGSW
>ID42699-ABP_both
VVAKKFFVLVKGLAPVLSPS
>ID42706-NO
VVDRFPD
>ID42708-NO
VVEKMFKKFRCGLSGNDC
>ID42711-ABP_both
VVFRVASKVFPAVYCTVSKK
>ID42712-NO
VVGADNCNFN
>ID42713-NO
VVGAK
>ID42714-NO
VVGGAECKIDGHRCLALLY
>ID42716-NO
VVGGDECNINDHRSLVRIF
>ID42717-NO
VVGGDECNINE
>ID42718-NO
VVGGDECNINEHPFLV
>ID42719-NO
VVGGDECNINEHRFLVALYY
>ID42721-NO
VVGGDECNINEHRSLALMYA
>ID42723-NO
VVGQQDFLRF
>ID42724-ABP_both
VVGRVASKVVPSLIGLFTTK
>ID42725-NO
VVGSPSAQDEASPL
>ID42726-NO
VVIGQACYRSPDCYSACKKLVGKATGKCTNGRCDC
>ID42727-NO
VVIGQRCYASPDCYSACKKLVGKATGKCTNGRCDC
>ID42728-NO
VVIGQRCYRSPDCYSACAKLVGKATGKCTNGRCDC
>ID42736-NO
VVKCSFRPGSPAPRCK
>ID42737-NO
VVKCSKRLGSPKRCN
>ID42739-NO
VVKCSYREGSPDSRCK
>ID42740-NO
VVKCSYRLGSPDPRCN

>ID42741-NO
VVKCSYRLGSPDSRCK
>ID42743-NO
VVKCSYRLGSPGSRCN
>ID42745-NO
VVKFPYRCKAAFC
>ID42746-NO
VVKIPFRCKSTFC
>ID42748-ABP_both
VVMKLGKAFVPIGKWKKDGI
>ID42749-NO
VVNTPGHAVSYHVY
>ID42751-NO
VVPPA
>ID42752-NO
VVPPFIQPE
>ID42759-ABP_both
VVRRPVYIPQPRPPHPRL
>ID42760-NO
VVSHFNDCPDSHTQFCFHGTCRFLVQEDKPACVCHSGYVGARCEHADLLA
>ID42762-NO
VVSVVPGAISHA
>ID42763-NO
VVTEACEEYCEDRDKKTCCGLENGEPFCATLCF
>ID42764-NO
VVTETCKEYCEDRDKTCCGLENGQPDCANLCL
>ID42767-NO
VVVPP
>ID42768-NO
VVVPPF
>ID42772-NO
VVYPWT
>ID42777-NO
VWCDWEWCYGDCHCFD
>ID42778-NO
VWDPPKFD
>ID42794-ABP_both
VWRHW
>ID42795-ABP_both
VWRHWRRFW
>ID42796-ABP_both
VWRHWRRFWH
>ID42799-ABP_both
VWRKWRRFW
>ID42800-ABP_both
VWRKWRRFWKR
>ID42801-ABP_both
VWRRWRRFW
>ID42802-ABP_both
VWRRWRRFWRR
>ID42803-NO

VWRTGHL
>ID42806-ABP_both
VWTTAMEKSSAANFSMSRNQRRSSLHSL
>ID42869-NO
VYAQCGVNVRTGRGGCSRLM
>ID42873-NO
VYEDKRLPNRYNFGL
>ID42874-NO
VYGMLFKFL
>ID42875-NO
VYGMLFKFLAKKVAKKLISHVAKKQLQ
>ID42876-NO
VYGPRDIANLY
>ID42877-NO
VYHEL
>ID42882-NO
VYLPQ
>ID42885-NO
VYNEGLPAP
>ID42888-NO
VYPFPG
>ID42890-NO
VYPFPGPIHNSLPQNIPPLTQT
>ID42891-NO
VYPFPGPIPNSL
>ID42893-NO
VYPHK
>ID42897-NO
VYRKRKSILKIYAKLKGWH
>ID42900-NO
VYVEELKPTPE
>ID42901-NO
VYVPRYIANLY
>ID42903-NO
WAEPAYQRFL
>ID42905-NO
WANQVRF
>ID42906-NO
WANQVRFG
>ID42908-NO
WAPEWAPEWILE
>ID42909-NO
WAPEWVGWI
>ID42910-ABP_both
WAQPKTKVIPYVRYL
>ID42912-NO
WATIDECEETCNVTFKTCCGPPGDWQCVEACPV
>ID42914-ABP_both
WAWRDIVRGIRKVAAPVLST
>ID42916-NO
WCASGCRKKRHGGCSC

>ID42919-NO
WCKQSGEMCNLLDQNCCDGYCIVFVCT
>ID42931-NO
WCSGSGEGCDYHSECCGERCCIESMCIGDGVACWP
>ID42933-NO
WDAYDCIQFCMRPEMRHTYAQCLSICT
>ID42934-NO
WDFLKELEGVGQRVRDSIISAGPAIDVLKKSQGPRRWSRP
>ID42935-NO
WDLVVVSAGVAEVGV
>ID42936-NO
WDPVL
>ID42937-NO
WDRGNVTLLCDCPNGPWVWVPAFCQAVG
>ID42938-NO
WDRGNVTLLSDSPNGPWVWV
>ID42941-NO
WDVNDCIHFCLIGVVERSYTECHTMCT
>ID42943-NO
WEAALAEALAEALAEHLAEALAEALEALAA
>ID42944-NO
WEDWAPEWI
>ID42946-NO
WEEPAYQRFL
>ID42947-NO
WEEWDKKIEEYTKKIEELIKKS
>ID42955-NO
WESLYFPRE
>ID42956-NO
WETCKEFLKLSQLEIPQDGTSALRESSPEESHAL
>ID42957-NO
WEWPWNRKPTKFPIPSPNPRDKWCRLNLGPAWGGRC
>ID42961-NO
WFGDVNQKPI
>ID42962-NO
WFGHEECTYWLGPCEVDDTCCSASCESKFCGLW
>ID42967-ABP_both
WFKKLLKKALRLWKKVL
>ID42973-NO
WFVLLTITVLR
>ID42975-ABP_both
WFWKLLWKALRLWWKVL
>ID42981-ABP_both
WGARDIVRGIRKVAAPVLST
>ID42982-NO
WGATRGCAATCPEAKPRETVECCATDKCNL
>ID42983-ABP_both
WGEAFSAGVHRLANGGNG
>ID42990-NO
WGKIEDPLRA
>ID42996-ABP_neg

WGLRRLLKYGKRS
>ID42997-ABP_both
WGNILL
>ID43000-ABP_both
WGRAFRRGVRRLARGGRR
>ID43001-ABP_both
WGRAFRRLVRRLARGLRR
>ID43003-ABP_both
WGRAFSAGVHRLARGGRG
>ID43015-ABP_both
WGWADIVRGIRKVAAPVLST
>ID43016-ABP_both
WGWRAIVRGIRKVAAPVLST
>ID43017-ABP_both
WGWRDAVRGIRKVAAPVLST
>ID43018-ABP_both
WGWRDIARGIRKVAAPVLST
>ID43019-ABP_both
WGWRDIVAGIRKVAAPVLST
>ID43035-NO
WHHTF
>ID43043-ABP_both
WHWTWL
>ID43044-ABP_both
WHWTWLPKKKRKV
>ID43045-ABP_both
WHWTWLPRRRK
>ID43046-ABP_both
WHWTWLRIRKKLR
>ID43049-ABP_both
WIAPKTKVIPYVRYL
>ID43050-NO
WIDNLD
>ID43051-NO
WIDPSHYCCCGGGCTDDCVNC
>ID43052-NO
WIEPAYQRFL
>ID43053-NO
WIFPWIQL
>ID43055-NO
WIIFKIAASHKK
>ID43056-NO
WIIFRAAASHKK
>ID43057-NO
WIIFRALISHKK
>ID43059-NO
WIIFRIAATHKK
>ID43065-ABP_both
WIKRLNSWLRK
>ID43068-ABP_both
WIQAKTKVIAYVRYL

>ID43069-ABP_both
WIQAKTKVIPYVRYL
>ID43072-ABP_both
WIQPATAVIPYVAYL
>ID43073-ABP_both
WIQPATAVIPYVRYL
>ID43075-ABP_both
WIQPATKVIPYVRYL
>ID43076-ABP_both
WIQPKAKVIPYVRYL
>ID43081-ABP_both
WIQPKTKVIP
>ID43082-ABP_both
WIQPKTKVIPAVRAL
>ID43083-ABP_both
WIQPKTKVIPAVRYL
>ID43084-ABP_both
WIQPKTKVIPFFFFF
>ID43085-ABP_both
WIQPKTKVIPWWWWW
>ID43087-ABP_both
WIQPKTKVIPYV
>ID43095-ABP_neg
WIRRIKKWIRRVHK
>ID43097-ABP_pos
WIRWL
>ID43098-ABP_both
WIRYKWPRKKVRYWTGP
>ID43099-ABP_both
WIRYKWPRKVVRYWTGP
>ID43105-ABP_both
WIVYKWPRKVVRYWTGKR
>ID43115-NO
WKEMSVW
>ID43117-NO
WKFALKVDSPDV
>ID43126-ABP_both
WKKAWKPGAKKWAK
>ID43130-ABP_both
WKKIWKPGIKKWIK
>ID43133-ABP_both
WKKKWKCWCKWWKKWW
>ID43134-ABP_both
WKKKWLWL
>ID43135-NO
WKKLFKKLKIL
>ID43137-ABP_pos
WKKVQWLKRLLL
>ID43138-ABP_both
WKKVWKPGVKKWVK
>ID43139-ABP_both

WKKWWKKW
>ID43140-ABP_both
WKKWWKKWWK
>ID43141-ABP_both
WKKWWKKWWKKW
>ID43142-ABP_both
WKKWWKKWWKWWKKWWKK
>ID43143-ABP_both
WKKWWKWWKKWWKK
>ID43148-ABP_both
WKLFLKAVKKLL
>ID43149-ABP_both
WKLLSKAQEKFGKNKSRFKCRRWQWRMKKLG
>ID43154-NO
WKQMSVW
>ID43158-ABP_both
WKRIVRRIWRWLR
>ID43159-ABP_both
WKRIVRWIKRWLR
>ID43160-ABP_both
WKRLVAASAKKK
>ID43162-ABP_neg
WKRRIKIWKKIR
>ID43168-ABP_both
WKRWVQRWKRFLR
>ID43170-ABP_both
WKRWVRRWKRWLR
>ID43177-ABP_both
WKWKPGKWKW
>ID43178-ABP_both
WKWKWKPGKWKWKW
>ID43179-ABP_both
WKWKWKWKPGKWKWKWKW
>ID43180-ABP_both
WKWKWKWKWKPGKWKWKWKWKW
>ID43185-ABP_both
WKWWKKWWKK
>ID43186-ABP_pos
WKWWQWQRNWRKVWG
>ID43190-NO
WLAHK
>ID43201-ABP_both
WLLKRWKKLL
>ID43202-ABP_both
WLLLGKSGGRNDDAVVRK
>ID43207-NO
WLNAKRHHGYKRKFH
>ID43211-ABP_both
WLNALKKVWQGIHEAIKLIWNWVQ
>ID43212-NO
WLNALLHHGYKRKFH

>ID43219-ABP_both
WLRAFRRLVRRLARGLRR
>ID43228-ABP_both
WLRRIKAWLRR
>ID43229-ABP_both
WLRRIKAWLRRIKA
>ID43230-NO
WLRRIKAWLRRIKALNRQLGVAA
>ID43231-ABP_neg
WLRRIKAWLRRKRK
>ID43233-NO
WLRYDA
>ID43238-NO
WLWDSNES
>ID43240-NO
WLWEDNEK
>ID43241-NO
WLWEDNKE
>ID43242-NO
WLWREQER
>ID43243-NO
WLWRESQK
>ID43244-NO
WLWSEKNS
>ID43245-NO
WLWSENSK
>ID43246-NO
WLWSEQSD
>ID43248-NO
WLWSSKEN
>ID43256-NO
WLYKREDY
>ID43259-NO
WMDFG
>ID43263-NO
WMEWDRKIEEYTKKIEELIKKSQEQQEKNEKELK
>ID43268-ABP_both
WMQKVIDRFGG
>ID43277-NO
WNINA
>ID43279-NO
WNLLRQAQEKFGKDKSPK
>ID43280-NO
WNLNA
>ID43281-NO
WNLPWYYSVSPT
>ID43283-NO
WNPFKKIANRNCYPKTTCETAGGKKTCKDFSCCQIVLFGKKTRAKCTVVT
>ID43284-NO
WNPFRKLYRKECNDVTSCDTVSGVKTCTKKNCCHRKFFGKTILKAPECTV
>ID43287-NO

WNSLAIDNLDV
>ID43291-NO
WPCKVAGSPCGLVSECCGTCNVLRNRCV
>ID43295-NO
WPERPPQIP
>ID43296-NO
WPFAHWPWQYPR
>ID43299-ABP_both
WPKRLTKAHWFEIQHIQPSPLQSNRAMSGINNYTQHSKHQNTFLH
>ID43301-ABP_both
WPKWWKWKRRWGRKKAKKRRG
>ID43306-NO
WPLHTSVYPPSP
>ID43307-NO
WPPRPQIPP
>ID43309-ABP_both
WPRFPKPRKPTYPGPTYPGPTWPRPTWRRSATIDTEH
>ID43311-NO
WPRYAESTLQLR
>ID43315-NO
WPTYLNPSSLKA
>ID43344-NO
WQPDTAHHWATL
>ID43347-ABP_neg
WQRMRKL
>ID43348-NO
WQSLTLTHRG
>ID43349-NO
WQVLPNAVPAK
>ID43359-ABP_both
WRCRRRFCRW
>ID43362-NO
WREGSCTSWLATCTDASQCCTGVCYKRAYCALWE
>ID43363-NO
WREMSVW
>ID43364-NO
WREWFL
>ID43366-NO
WRFKKSKRKV
>ID43368-NO
WRFKWRFK
>ID43369-NO
WRFKWRFKWRFK
>ID43371-NO
WRGALKLLFYAT
>ID43380-ABP_both
WRKAWRPGAKRWAK
>ID43381-ABP_both
WRKIWRPGIKRWIK
>ID43382-ABP_both
WRKKWFW

>ID43383-ABP_both
WRKLWRGGLKRWLK
>ID43385-ABP_both
WRKVWRPGVKRWVK
>ID43386-ABP_both
WRKWRKRWWWRKWRKRWW
>ID43391-ABP_pos
WRLWWLWWL
>ID43392-ABP_both
WRNGRWWRNGRW
>ID43395-NO
WRNTIA
>ID43400-ABP_both
WRPGRW
>ID43401-ABP_both
WRPGRWWRPGRW
>ID43402-ABP_both
WRPGRWWRPGRWWRPGRW
>ID43403-ABP_both
WRPGRWWRPGRWWRPGRWWRPGRW
>ID43408-NO
WRRRIL
>ID43411-NO
WRRRRL
>ID43412-NO
WRRRRR
>ID43413-ABP_both
WRRRRRRRR
>ID43414-ABP_both
WRRRYRRWRRRRRWRRRPRR
>ID43418-ABP_both
WRRWWRWWRRWWRR
>ID43420-NO
WRVLAAF
>ID43426-ABP_both
WRWLRRIW
>ID43427-ABP_both
WRWNRRTGRWRWTGP
>ID43430-ABP_both
WRWRCRRRFCRWRW
>ID43435-ABP_both
WRWRW
>ID43436-ABP_both
WRWRWR
>ID43437-NO
WRWRWRWRWRWRWR
>ID43441-ABP_both
WRWWRRWWRR
>ID43448-NO
WSCADRTCM
>ID43449-NO

WSGFTQGVGNPVSCVRNKGICVPIRCPGNMKQIGTC
>ID43450-ABP_both
WSGIRGYKGGYKGLFKQTNY
>ID43456-NO
WSKVVL
>ID43457-NO
WSLDGLARIEKLLSTSSSASAASPTRGQALNL
>ID43461-NO
WSPQEEDRIIEGGI
>ID43467-NO
WSVPQPK
>ID43474-NO
WTEPAYQRFL
>ID43476-NO
WTHHHSYPRPL
>ID43478-NO
WTRCSSSCGRGVSVRSR
>ID43484-NO
WVDVGCTFLLGSCTADAECCSDNCVETYCDLWW
>ID43486-ABP_both
WVIKGYKGGQKITVHRR
>ID43490-NO
WVPNLPQRF
>ID43491-NO
WVPSV
>ID43492-NO
WVPSVY
>ID43501-NO
WWAMKP
>ID43505-NO
WWCLGERVVRCAH
>ID43507-NO
WWDGECRLWSNGCRKHKECCSNHCKGIYCDIW
>ID43511-NO
WWEGDCTDWLGSCSSPSECCYDNCETYCTLW
>ID43512-NO
WWEGDCTGWLDGCTSPAECCTAVCDATCKLW
>ID43513-NO
WWEGECRGWSNGCTTNSDCCSNNCDGTFCKLW
>ID43515-ABP_both
WWFHWK
>ID43518-NO
WWGENDCRVFGSCTADEECCFNNCVQAYCFFV
>ID43522-ABP_both
WWHSWWSTW
>ID43524-ABP_both
WWKDWWERW
>ID43531-ABP_both
WWKRWKKLRRIFLML
>ID43532-ABP_both
WWKRWKRIRRIFMMV

>ID43533-ABP_both
WWKWWKRLRRLFLLV
>ID43538-ABP_pos
WWLRWLWWL
>ID43539-ABP_pos
WWLWRLWWL
>ID43540-ABP_pos
WWLWWLLWL
>ID43541-ABP_pos
WWLWWLR
>ID43542-ABP_pos
WWLWWLRW
>ID43544-ABP_pos
WWLWWLWRL
>ID43546-ABP_pos
WWLWWLWWR
>ID43547-ABP_pos
WWLWWRWWL
>ID43551-ABP_both
WWQDWWNEW
>ID43553-ABP_both
WWRLRRIW
>ID43555-ABP_both
WWRRWWRR
>ID43562-ABP_both
WWRWRW
>ID43563-ABP_pos
WWRWWLWWL
>ID43565-NO
WWSGLEA
>ID43566-ABP_both
WWSYWWTQW
>ID43568-ABP_both
WWVAARAARR
>ID43570-ABP_neg
WWVFRVVYPRIVYVRRRWTGPKK
>ID43591-ABP_both
WWWLKKIW
>ID43592-ABP_both
WWWLKRIW
>ID43593-ABP_both
WWWLRRIR
>ID43594-ABP_both
WWWLRRIW
>ID43595-ABP_both
WWWLRRRW
>ID43596-ABP_both
WWWLSRIW
>ID43597-ABP_both
WWWRRRIW
>ID43603-ABP_both

WWWWWRRRRRRRR
>ID43604-ABP_both
WWYNWWQDW
>ID43613-ABP_both
WYADHSDQYQLLDTP
>ID43614-ABP_both
WYADHSKQYQLKDTK
>ID43632-NO
WYGHGDFDEIDNVGWPGFT
>ID43638-NO
WYKPAAGHSSYSVGRAAGLLSGL
>ID43640-NO
WYPSKRWRLPLGKRQIE
>ID43644-NO
WYSLA
>ID43645-NO
WYSLAM
>ID43646-NO
WYSLAMA
>ID44278-NO
YADAIFTNSYRKILGQLSARKLLQDIMNRQQERNQEQGAKVRL
>ID44279-NO
YADAIFTNSYRKILGQLSARKLLQDIMNRQQGERNQEQGAKVRL
>ID44284-NO
YADPNADPMAFL
>ID44285-NO
YADPNADPMAFLTKLIQIEARKLSGCSN
>ID44287-NO
YADPNADPMAFLYRLIQIEARKLAGCSD
>ID44289-NO
YAEERYPIL
>ID44293-NO
YAFGYP
>ID44294-NO
YAFGYPK
>ID44295-NO
YAFQAVG
>ID44296-NO
YAFSAVG
>ID44297-NO
YAFSVVG
>ID44298-NO
YAFWYPN
>ID44305-NO
YAIAGRPRF
>ID44306-NO
YAIVARPRF
>ID44307-NO
YAKPVA
>ID44308-NO
YALPHA

>ID44310-NO
YANPAVVRP
>ID44312-NO
YAREARRAARR
>ID44313-NO
YARKARRAARR
>ID44314-NO
YATCDFIM
>ID44315-NO
YAVQYASETLFSKSPEININER
>ID44320-NO
YCCYFDYSCCLYLR
>ID44325-NO
YCGFCSG
>ID44327-NO
YCKEFWATFKSC
>ID44329-NO
YCKFEWATFKSC
>ID44331-ABP_both
YCKFKVKPKFKRWKLKFKGRMWCP
>ID44333-ABP_both
YCLLSRSPYLKKLEVHYRAELKCP
>ID44334-NO
YCNIHQVCHYAQRNDRSYWL
>ID44335-NO
YCNINEVCHYARRNDKSYWL
>ID44336-NO
YCQEFLWTCDEERKCCGDMVCRLWCKKRL
>ID44337-NO
YCQFKMWTCDSERKCCEDMVCRLWCKLNL
>ID44338-NO
YCQKFLWTCDTERKCCEDMVCELWCKLEK
>ID44339-NO
YCQKFLWTCDTERKCCEDMVCELWCKYKE
>ID44340-NO
YCQKWLWTCDSERKCCEDMVCRLWCKKRL
>ID44341-NO
YCQKWMWTCDAERKCCEDMACELWCKKRL
>ID44342-NO
YCQKWMWTCDEERKCCEGLVCRLWCKKKIEEG
>ID44344-NO
YCQKWMWTCDSERKCCEGYVCELWCKYNL
>ID44345-NO
YCQKWMWTCDSKRKCCEDMVCQLWCKKRL
>ID44346-NO
YCSDDSQPCSHFYDCCKWSCNNGYCP
>ID44348-NO
YCSDQWKSCSYPHECCRWSCNRYCA
>ID44349-NO
YCSDSGGWCGLDPELCCNSSCFVLC
>ID44350-ABP_both

YCSFNVTPKFKRWQLYFRGRMWCP
>ID44351-NO
YCVPKSGLCTIFQPGKCCSGWCLIYRCT
>ID44352-ABP_both
YCVYSVKPTFQRWQLYFIGSMWCP
>ID44364-NO
YDDGSYKPHIYGF
>ID44373-ABP_neg
YDLSDSNCLPANRDKRYYVI
>ID44379-NO
YDRCIGPCLRFYGNHQCYKNCRKAKYDGGQCDFVKKGEKLPECCCYYNKN
>ID44385-NO
YECCVWPYCDGGCSSCVRSCE
>ID44386-NO
YECYLLVHFCGINGGLCCSNLCLFFVCLTFS
>ID44387-NO
YECYSTGTFCGINGGLCCSNLCLFFVCLTFS
>ID44390-NO
YEDIKPKTSLAFR
>ID44395-NO
YEQDFLRF
>ID44401-NO
YFDALAGQSL
>ID44405-NO
YFMRF
>ID44407-NO
YFPIPF
>ID44408-NO
YFPIPI
>ID44409-NO
YFSPWG
>ID44410-ABP_both
YFTILGGSAEMPWAFDRLYKYDITKTL
>ID44412-NO
YFYPEL
>ID44414-NO
YGAHVFLRF
>ID44418-NO
YGCSNAGAFCGIHPGLCCSELCLGWCT
>ID44422-NO
YGFLP
>ID44423-NO
YGFLPILP
>ID44427-NO
YGGFI
>ID44428-NO
YGGFIGVRK
>ID44429-NO
YGGFIGVRKSA
>ID44430-NO
YGGFIGVRKSARKWNNQ

>ID44432-NO
YGGFLRRIRPKLKWDNE
>ID44433-NO
YGGFLRRQFKVVTRSQEDPNAYSEEFFDV
>ID44434-NO
YGGFLRRQFKVVTRSQEDPNAYYEELFDV
>ID44437-NO
YGGFMKSWDERSQKPLLTLFKNVIIKDGHQKKGQ
>ID44439-NO
YGGFMKSWDERSQRPLLTLFKNVINKDGQQQK
>ID44440-NO
YGGFMKSWEEDRQKPLVTLFKNIINKDEQQ
>ID44441-NO
YGGFMRGY
>ID44442-NO
YGGFMRNY
>ID44443-NO
YGGFMRSL
>ID44445-NO
YGGFMTPERSQTPLMTLFKNAIIKNSHKKGQ
>ID44447-NO
YGGFMTSEKSQTPLVTLFKNAIIKNAH
>ID44453-NO
YGGWG
>ID44458-NO
YGLVAGTW
>ID44459-NO
YGLYP
>ID44460-NO
YGNFPTCSETGEDCSAMHCCRSMTCRNNICAD
>ID44462-NO
YGNKNYLRF
>ID44463-NO
YGNRSFLRF
>ID44465-NO
YGQVPMCDAGEQCAVRKGARIGKLCDCPRGTSCNSFLLK
>ID44466-NO
YGRAARRAARR
>ID44468-NO
YGRKKKRRQRRR
>ID44470-NO
YGRKKRRQRRRGCYGRKKRRQRRRG
>ID44475-NO
YGRRARRAARR
>ID44476-NO
YGRRARRRARR
>ID44479-NO
YGSDRNFLRF
>ID44480-NO
YGSSAG
>ID44481-NO

YGSSAGAKCMNGKCKCYNN
>ID44485-NO
YHECCKHPPCRNTRPDLCG
>ID44486-NO
YHECCKNPPCRNKHPDLC
>ID44492-NO
YHTRIALPDNLP
>ID44499-NO
YIELAVVADHGIFTKYNSNLNTIR
>ID44500-NO
YIFHLMD
>ID44502-NO
YIGSLARAGGLMTY
>ID44503-NO
YIHPF
>ID44504-NO
YIHYIF
>ID44511-NO
YIPIQY
>ID44512-NO
YIPIQYVL
>ID44514-NO
YIPISNRLSDVLHQIEEQRMRENLLEDLFERLAAGDDSVGDV
>ID44517-ABP_both
YIPQPRPPHPRL
>ID44521-NO
YIQQARKAPSGRVSMIKNLQSLDPSHRISDRDYMGWMDF
>ID44528-NO
YIVLRRRRKRVNTKRS
>ID44579-NO
YIYTQ
>ID44587-NO
YKIFEPL
>ID44588-NO
YKIFEPLRESN
>ID44600-ABP_both
YKLLKKLLKKLKKLLKKL
>ID44601-ABP_both
YKLLKLLLPKLKGLLIKL
>ID44605-ABP_both
YKLLKLLLPKLKPLLPKL
>ID44606-ABP_both
YKLLKWLLLKLKALLEKL
>ID44607-NO
YKLPAKVPIRV
>ID44608-NO
YKLPDAVPIRV
>ID44609-NO
YKLPDKAPIRV
>ID44610-NO
YKLPDKVPIRV

>ID44614-NO
YKPITN
>ID44617-NO
YKPRSFAMGFG
>ID44618-NO
YKPWTNF
>ID44626-NO
YKRCHIKGGHCFPKEKICIPPSSDFGKMDCPWRRKSLKKGSG
>ID44631-NO
YKRKFHEKHHSH
>ID44638-ABP_both
YKWKIRFKR
>ID44640-NO
YKWLP
>ID44644-NO
YLAFRCGRYSPCLDDGPNVNLYSCCSFYNCHKCLARLENCPKGLHYNAYL
>ID44646-NO
YLAGNQ
>ID44647-NO
YLAHK
>ID44651-NO
YLDVKQLANYLLCIGNGQVFNGRKTCQIGCRAVCQQPGCSGYKECEQIPN
>ID44653-NO
YLDVNQIASYLLCLGQGAVFNGRKTCQIGCRAACQQPGCGGYKECEQIPN
>ID44654-NO
YLEPA
>ID44655-ABP_both
YLEQLLR
>ID44656-ABP_both
YLEQLLRL
>ID44657-ABP_both
YLEQLLRLK
>ID44658-NO
YLFADVSTIGDFFHSI
>ID44661-NO
YLKNYRIATFKNWPF
>ID44664-NO
YLNQEQAEQGREHLA
>ID44668-NO
YLSGKTKVQSMANLPQRF
>ID44670-NO
YLYEIA
>ID44671-NO
YLYEIAR
>ID44672-NO
YLYEIARR
>ID44673-NO
YMFWTSR
>ID44676-NO
YMNWKSPDAIYHLAK
>ID44685-NO

YNPCANYL
>ID44686-NO
YNPCASYL
>ID44687-NO
YNPCLGFI
>ID44688-NO
YNPCSNYL
>ID44689-NO
YNPCVGYF
>ID44692-NO
YNSMGLF
>ID44698-NO
YPAKPASPRDGAPPEELAKYYSALRHYINLITRQRY
>ID44700-NO
YPASYDDDFDALDDLDDLDLDDLLDLEPADLVLLDMWANMLDSQDFEDFE
>ID44701-NO
YPASYDDDFDALDDLDGLDLDDLLDSEPADLVLLDMWANMLDSQDFEDFE
>ID44706-ABP_both
YPELFRQF
>ID44708-NO
YPELQQDLIARLLGK
>ID44712-NO
YPFGPIPN
>ID44714-NO
YPFPGP
>ID44715-NO
YPFPGPI
>ID44716-NO
YPFPGPIN
>ID44718-NO
YPFPGPIPN
>ID44731-NO
YPIKPENPGEDAPADELAKYYSALRHYINLITRQRY
>ID44734-NO
YPLDL
>ID44735-NO
YPLDLF
>ID44737-ABP_both
YPLPFIP
>ID44738-NO
YPNFQGLF
>ID44741-NO
YPPKPENPGDDAAPEELAKYYSALRHYINLITRQRY
>ID44742-NO
YPPKPENPGDDAAPEELAKYYTALRHYINLITRQRY
>ID44744-NO
YPPKPENPGEDAPPEELARYYTALRHYINLITRQRY
>ID44745-NO
YPPKPENPGEDASPEEMTKYLTALRHYINLVTRQRY
>ID44746-NO
YPPKPENPGEDASPEEQAKYYTALRHYINLITRQRY

>ID44747-NO
YPPKPESPGSNASPEDWAKYHAAVRHYVNLITRQRY
>ID44750-NO
YPQRDMPIQ
>ID44751-NO
YPQSKWQEQ
>ID44756-NO
YPSFQPQPLIYP
>ID44758-NO
YPSKPDNPGDDAPAEDLARYYSALRHYINLITRQRY
>ID44759-NO
YPSKPDNPGEDAPAEDMAKYYSALRHYINLITRQRY
>ID44762-NO
YPSYGLNYY
>ID44764-NO
YPTKPDNPGEDAPAEELAKYYSALRHYINLITRQRY
>ID44765-NO
YPTKPDNPGEGAPAEELAKYYSALRHYINLITRQRY
>ID44766-NO
YPTKPENPGEDAPVEELAKYYSALRHYINLITRQRY
>ID44768-NO
YPTPYDI
>ID44770-NO
YPVEPFTE
>ID44774-NO
YPVQPFTE
>ID44775-NO
YPWFF
>ID44778-NO
YPWTQR
>ID44779-NO
YPYDANHTRSPT
>ID44789-NO
YQEPVL
>ID44792-NO
YQEPVLQPVR
>ID44793-NO
YQEVLGPVRGPFPIIV
>ID44795-NO
YQKFPQY
>ID44799-NO
YQNPRLGPTGELDPATQPIVAVHNPVIV
>ID44801-NO
YQQPVL
>ID44802-NO
YQQPVLGPVR
>ID44810-NO
YRCIARECE
>ID44811-NO
YRCREVLCQ
>ID44815-NO

YRFKYRFKYRLFK
>ID44816-NO
YRFSAR
>ID44819-NO
YRHWPIDYPPP
>ID44821-NO
YRIPIVRRLQRR
>ID44823-ABP_both
YRKKWFW
>ID44828-NO
YRNTQ
>ID44835-NO
YRQSMNNFQGLRSFGCRFGTCTVQKLAHQIYQFTDKDKDNVAPRSKISPQ
>ID44836-NO
YRRAARRAARA
>ID44837-NO
YRRRQCPPWCSGEPCRKGTC
>ID44841-NO
YRSTQ
>ID44844-ABP_both
YRWARWWRR
>ID44846-NO
YRWNSFGLRY
>ID44848-ABP_both
YRWWRWARRW
>ID44849-ABP_both
YRWWRWARRWYRWWRWARRW
>ID44853-NO
YSAYPDSVPMMS
>ID44855-ABP_neg
YSDVKKLEMKPKYPHCEEKMVIITTKSVSRYRGQEHCLHPK
>ID44857-NO
YSFGL
>ID44858-NO
YSFLPRL
>ID44859-NO
YSFNSWM
>ID44860-NO
YSGFLT
>ID44861-NO
YSGKDCLKDMGGYALAGAGSGALWGAPAGGVGALPGAFVGAHVGAIAGGF
>ID44864-NO
YSHIATLPFTPT
>ID44865-NO
YSHNTITNLYFS
>ID44866-NO
YSINDWH
>ID44867-NO
YSKFNFGL
>ID44870-NO
YSLRARPRF

>ID44871-NO
YSPCTNF
>ID44872-NO
YSPCTNFF
>ID44873-NO
YSPITNF
>ID44874-NO
YSQQQQ
>ID44875-NO
YSQVARPRF
>ID44876-NO
YSQVSRPRF
>ID44879-NO
YSSKDCLKDIGKGIGAGTVAGAAGGGLAAGLGAIPGAFVGAHFGVIGGSA
>ID44882-NO
YSTCAFIM
>ID44883-NO
YSTCDAIM
>ID44884-NO
YSTCDFAM
>ID44885-NO
YSTCDFIM
>ID44887-NO
YSTCSYYF
>ID44888-NO
YSTCYFIM
>ID44889-NO
YSTSDFIM
>ID44892-ABP_both
YSYFTVV
>ID44893-NO
YSYKKIDCGGACAARCRLSSRPRLCNRACGTCCARCNCVPPGTSGNTETC
>ID44894-ABP_both
YSYYTIV
>ID44895-NO
YTAGV
>ID44896-NO
YTAIAWVKAFIRKLRK
>ID44898-NO
YTDAPSF
>ID44900-NO
YTGFLS
>ID44901-ABP_both
YTGIFTKQVLSKLKK
>ID44905-NO
YTMNPRKLFDY
>ID44910-NO
YTQDFNKFHTFPQTAIGVGAP
>ID44914-NO
YTSIYMSHQCCFRGCSRRSLTAAC
>ID44955-NO

YTSPHHSTTGHL
>ID45004-NO
YTYGLCTSSR
>ID45007-NO
YVASLARTGDLPIRGQ
>ID45011-NO
YVGALARSGGLMGY
>ID45016-ABP_both
YVLAKRKRAIFI
>ID45017-ABP_both
YVLFKRKRFIFI
>ID45018-ABP_both
YVLLKRKRLIFI
>ID45019-ABP_both
YVLWKRKRFIFI
>ID45020-ABP_both
YVLWKRKRWIFI
>ID45021-ABP_both
YVLWKRKRYIFI
>ID45022-NO
YVMGHFRWDRFG
>ID45023-NO
YVMSHFRWNKF
>ID45024-NO
YVMTHFRWNKF
>ID45029-ABP_both
YVPKIPKPQPNKPNFPSFPGHGPFNPHASRFPR
>ID45035-NO
YVRTCMIKKEGWGKCLIDTTCAHSCKNRGYIGGDCKTCYCLVNC
>ID45036-NO
YVSGKARG
>ID45037-NO
YVTGHFRWGRF
>ID45041-NO
YVVFK
>ID45045-NO
YWAGGDASGE
>ID45046-ABP_both
YWFHWK
>ID45049-ABP_both
YWKKWKKLRRIFMLV
>ID45054-ABP_both
YWRWRW
>ID45058-NO
YWTRSACCYIEEGEKCPASCKLCC
>ID45072-NO
YYAPF
>ID45073-NO
YYAPFDGIL
>ID45074-NO
YYAPFE

>ID45079-ABP_both
YYGALHNTAHIVLGR
>ID45080-ABP_both
YYHFWHRGVTKR
>ID45081-ABP_both
YYHFWHRGVTKRSL
>ID45082-ABP_both
YYHFWHRGVTKRSLSPH
>ID45083-ABP_both
YYHFWHRGVTKRSLSPHRPR
>ID45084-ABP_both
YYHFWHRGVTKRSLSPHRPRHSR
>ID45086-NO
YYICESCWTCESCAGSTESSCVSACNACDLCPNK
>ID45087-ABP_neg
YYNPLPHDCGRDNNTDICSR
>ID45089-NO
YYPQIMQY
>ID45091-ABP_neg
YYQQKPVA
>ID45093-NO
YYVPLGTQ
>ID45095-NO
YYYAAGRKRKKRT
>ID45099-ABP_both
YYYYRRRR

Section S6. List (FASTA format) of all the peptides in the development dataset.

>ID00011-ABP_both
FLPLLAGLAANFLPTIICKISYKC
>ID00017-NO
KTCEHLADTYRGVCFTNASCDDHCKNKAHLISGTCHNWKCFCTQNC
>ID00028-ABP_both
FLPAIVGAAAKFLPKIFCVISKKC
>ID00030-ABP_both
FLPFIAGMAANFLPKIFCAISKKC
>ID00043-ABP_both
RRWCFRVCYRGFCYRKCR
>ID00052-ABP_neg
FFPIVAGVAGQVLKKIYCTISKKC
>ID00053-ABP_neg
FLPAIVGAAGQFLPKIFCAISKKC
>ID00057-ABP_both
FLPMLAGLAASMVPKFVCLITKKC
>ID00078-ABP_neg
GNRPVYIPPPRPPHPRL
>ID00088-ABP_both
SLFSLIKAGAKFLGKNLLKQGACYAACKASKQC
>ID00093-ABP_both
WLGSALKIGAKLLPSVVGLFKKKKQ
>ID00113-ABP_both
FLPLILRKIVTAL
>ID00121-ABP_both
GIMSIVKDVAKNAAKEAAKGALSTLSCKLAKTC
>ID00147-ABP_both
LLGRCKVKSNRFNGPCLTDTHCSTVCRGEGYKGGDCHGLRRRCMCLC
>ID00152-ABP_neg
RSGRGECRRQCLRRHEGQPWETQECMRRCRRRG
>ID00158-ABP_both
VRRFPWWWPFLRR
>ID00159-ABP_both
VTCFCRRRGCASRERHIGYCRFGNTIYRLCCRR
>ID00164-ABP_both
ALWKTLLKNVGKAAGKAALNAVTDMVNQ
>ID00174-ABP_both
FLFPLITSFLSKVL
>ID00176-ABP_both
FLPIIASVAAKVFPKIFCAISKKC
>ID00196-ABP_both
GILKKFMLHRGTKVYKMRTLSKRSH
>ID00211-ABP_pos
GWFDVVKHIASAV
>ID00212-ABP_both
ILGKIWEGIKSLF
>ID00213-ABP_both
ILGTILGLLKGL
>ID00222-ABP_both

QGVRNHVTCRIYGGFCVPIRCPGRTRQIGTCFGRPVKCCRRW
>ID00225-ABP_both
RRRPRPPYLPRPRPPPFFPPRLPPRIPPGFPPRFPPRFP
>ID00228-ABP_both
RVCYAIPLPICY
>ID00230-ABP_neg
SIGSAFKKALPVAKKIGKAALPIAKAALP
>ID00242-ABP_both
VTCFCKRPVCDSGETQIGYCRLGNTFYRLCCRQ
>ID00257-ABP_both
DCLSGRYKGPCAVWDNETCRRVCKEEGRSSGHCSPSLKCWCEGC
>ID00260-ABP_both
DTHFPICIFCCGCCHRSKCGMCCKT
>ID00275-ABP_both
FLPIIAKLLGGLL
>ID00281-ABP_pos
FLSAIASMLGKFL
>ID00310-ABP_both
GKVWDWIKSTAKKLWNSEPVKELKNTALNAAKNLVAEKIGATPS
>ID00314-ABP_both
GLFDIVKKIAGHIAGSI
>ID00317-ABP_both
GLFDIVKKVVGAFGSL
>ID00334-ABP_both
GLMSSIGKALGGLIVDVLKPKTPAS
>ID00335-ABP_both
GLWQKIKDKASELVSGIVEGVK
>ID00362-ABP_both
KYYGNGVHCTKSGCSVNWGEAFSAGVHRLANGGNGFW
>ID00369-ABP_both
MGAIAKLVAKFGWPIVKKYYKQIMQFIGEGWAINKIIDWIKKHI
>ID00375-NO
QTCASRCPRPCNAGLCCSIYGYCGSGAAYCGAGNCRCQCRG
>ID00378-ABP_both
RGGRLCYCRRRFCICV
>ID00391-ABP_pos
VLPLISMALGKLL
>ID00396-ABP_both
YPPKPESPGEDASPEEMNKYLTALRHYINLVTRQRY
>ID00404-ABP_both
ALSILKGLEKLAKMGIALTNCKATKKC
>ID00417-ABP_both
ENFFKEIERAGQRIRDAIISAAPAVETLAQAQKIIKGGD
>ID00426-ABP_pos
FLPFLASLLTKVL
>ID00428-ABP_both
FLPIIAGAAAKVVQKIFCAISKKC
>ID00435-ABP_both
FLSGIVGMLGKLF
>ID00453-ABP_both
GIFPKIIGKGIKTGIVNGIKSLVKGVGMKVFKAGLNNIGNTGCNEDEC

>ID00456-ABP_both
GIFSKFGGKAIKNLFIKGAKNIGKEVGMDVIRTGIDVAGCKIKGEC
>ID00457-ABP_both
GIFSKLAGKKLKNLLISGLKNVGKEVGMDVVRTGIDIAGCKIKGEC
>ID00458-ABP_both
GIFSLIKGAAQLIGKTVAKEAGKTGLELMACKVTKQC
>ID00465-ABP_both
GKLQAFLAKMKEIAAQTL
>ID00472-ABP_both
GLFSVLGAVAKHVLPHVVPVIAEKL
>ID00475-ABP_both
GLLDSLKNLAINAAKGAGQSVLNTLSCKLSKTC
>ID00478-ABP_both
GLLGSLFGAGKKVACALSGLC
>ID00484-ABP_both
GLWSKIKEVGKEAAKAAAKAAGKAALGAVSEAV
>ID00513-ABP_both
KRIVQRIKDFLRNLVPRTES
>ID00516-ABP_both
KWKLFKKIGIGAVLKVLTTGLPALIS
>ID00521-ABP_pos
KYYGNGVTCGKHSCSVDWGKATTCIINNGAMAWATGGHQGNHKC
>ID00531-ABP_both
NLVSGLIEARKYLEQLHRKLKNCKV
>ID00542-ABP_both
RVKRVWPLVIRTVIAGYNLYRAIKKK
>ID00554-ABP_pos
TSYGNGVHCNKSKCWIDVSELETYKAGTVSNPKDILW
>ID00564-ABP_both
YRGGYTGPIPRPPPIGRPPFRPVCNACYRLSVSDARNCCIKFGSCCHLVK
>ID00569-ABP_both
AGWGSIFKHIFKAGKFIHGAIQAHND
>ID00572-NO
AIPCGESCVWIPCISAAIGCSCKNKVCYR
>ID00574-ABP_neg
ALFSILRGLKKLGKMGQAFVNCEIYKKC
>ID00584-ABP_pos
ARSYGNGVYCNNKKCWVNRGEATQSIIGGMISGWASGLAGM
>ID00596-ABP_both
DCTRWIIGINGRICRD
>ID00607-ABP_both
FHPSLWVLIPQYIQLIRKILKSG
>ID00608-ABP_both
FIFPKKNIINSLFGR
>ID00610-ABP_both
FIGGIISFFKRLF
>ID00634-ABP_both
FLPVIAGVAANFLPKLFCAISKKC
>ID00635-ABP_both
FLPVILPVIGKLLSGIL
>ID00656-ABP_both

GFLDSFKNAMIGVAKSAGKTALNTLACKIDKTC
>ID00657-ABP_both
GFLGILFHGVHHGRKKALHMNSERRS
>ID00658-ABP_both
GFMATAKNVAKNMDVTLLDNLKCKITKAC
>ID00659-ABP_both
GFMDTAKNVAKNEAGNLLDNLKCKITKAC
>ID00668-ABP_both
GIFLKVLGVGKKVLCGVSGLC
>ID00669-ABP_both
GIFSKINKKKAKTGLFNIIKTVGKEAGMDVIRAGIDTISCKIKGEC
>ID00682-ABP_both
GIMDTIKDTAKTVAVGLLNKLKCKITGC
>ID00687-ABP_both
GKIPVKAIKKAGAAIGKGLRAINIASTAHDVYSFFKPKHKKK
>ID00699-ABP_both
GLFTLIKGAYKNDAPTVACN
>ID00702-ABP_both
GLGSVFGRLARILGRVIPKV
>ID00710-ABP_both
GLLDTIKNMALNAAKSAGVSVLNTLSCKLSKTC
>ID00713-ABP_both
GLLGLLGSVVSHVLPAITQHL
>ID00724-ABP_neg
GLMSVLGHAVGNVLGGLFKS
>ID00734-ABP_both
GLWSTIKNVGKEAAIAAGKAVLGSL
>ID00745-ABP_both
GVFTLIKGATQLIGKTLGKEVGKTGLELMACKITKQC
>ID00757-ABP_both
GWASKIGQTLGKIAKVGLKELIQPK
>ID00760-ABP_both
GWRTLLKKAEVKTVGKLALKHYL
>ID00794-ABP_both
KDLHTVVSAILQAL
>ID00806-ABP_both
KSKEKIGKEFKRIVQRIKDFLRNLVPRTES
>ID00810-ABP_neg
KVPIGAIKKGGKIIKKGLGVIGAAGTAHEVYSHVKNRQ
>ID00815-ABP_both
LAFVAGVAAEMMQHVYCAASKKC
>ID00829-ABP_both
NKGCATCSIGAACLVDGPIPDFEIAGATGLFGLWG
>ID00844-ABP_both
RPKPQQFFGLM
>ID00852-ABP_both
SFLDTLKNLAISAAKGAGQSVLSTLSCKLSKTC
>ID00853-ABP_both
SFLSTFKELAINAAKNAGQSILHTLSCKLDKTC
>ID00862-ABP_both
SLKDKVKSMGEKLKQYIQTWKAKF

>ID00864-ABP_both
SLWETIKNAGKGFILNILDKIRCKVAGGCKT
>ID00866-ABP_both
SRSELIVHQR
>ID00873-ABP_both
SVMGTVKDLLIGAGKSAAQSVLKSLSCKLSNDC
>ID00874-ABP_both
SWASMAKKLKEYMEKLKQRA
>ID00879-ABP_both
VIPFVASVAAEMMHHVYCAASKRC
>ID00896-ABP_both
AALRGCWTKSIPPKPCPGKR
>ID00905-ABP_both
AKIPIKAIKTVGKAVGKGLRAINIASTANDVFNFLKPKKRKA
>ID00935-ABP_both
EPFKISIHL
>ID00939-ABP_both
FALGAVTKRLPSLFCLITRKC
>ID00940-ABP_both
FFGAIAAALPHVISAIKNAL
>ID00945-ABP_both
FFPIVGKLLFGLFGLL
>ID00951-ABP_both
FFPLLFGALSSHLPKLF
>ID00962-ABP_both
FLGGLMKAFPAIICAVTKKC
>ID00967-ABP_both
FLPIIAGMAAKVICAITKKC
>ID00979-ABP_both
FLPVLAGLTPSIVPKLVCLLTKKC
>ID00991-ABP_both
GFFKKAWRKVKHAGRRVLDTAKGVGRHYVNNWLNRYR
>ID00994-ABP_both
GFGPAFHSVSNFAKKHKTA
>ID00995-ABP_both
GFGSLFKFLAKKVAKTVAKQAAKQGAKYIANKQME
>ID01011-ABP_both
GIGALSAKGALKGLAKGLAEHFAN
>ID01030-ABP_both
GIPCGESCVFIPCITGAIGCSCKSKVCYRN
>ID01032-ABP_both
GKFSGFAKILKSIAKFFKGVGKVRKQFKEASDLDKNQ
>ID01039-ABP_both
GLFDVIKKVASVIGLASP
>ID01041-ABP_both
GLFNVFKKVGKNVLKNVAGSLMDNLKCKVSGEC
>ID01049-ABP_both
GLGSFLKNAIKIAGKVGSTIGKVADAIGNKE
>ID01059-ABP_both
GLLRKGGEKIGEKLKKIGQKIKNFFQKLVPQPEQ
>ID01064-ABP_both

GLLSGTSVRGSI
>ID01098-ABP_both
GWKSVFRKAKKVGKTVGGLALDHYL
>ID01107-ABP_both
IFGSIYHRKCVVKNRCETVSGHKTCKDLTCCRAVIFRHERPEVCRPQT
>ID01109-ABP_both
IKIPAFVKDTLKKVAKGVISAVAGALTQ
>ID01123-ABP_both
KFCEKPSGTWSGVCGNSGACKDQCIRLEGAKHGSCNYKPPAHRCICYYEC
>ID01124-ABP_both
KIAKVALKAL
>ID01138-ABP_both
LFGLIPSLIGGLVSAFK
>ID01141-ABP_both
LIGPVLGLVGSALGGLLKKIG
>ID01197-ABP_both
SWLSKTYKKLENSAKKRISEGVAIAILGGLR
>ID01203-ABP_both
VFHLLGKIIHHVGNFVYGFSHVF
>ID01206-ABP_both
VIPFVASVAAEMMQHVYCAASKKC
>ID01208-ABP_both
VIVFVASVAAEMMQHVYCAASKKC
>ID01213-ABP_both
WNPFKELEKVGQRVRDAVISAGPAVATVAQATALAK
>ID01252-ABP_both
CRFCCRCCPRMRGCGLCCRF
>ID01255-ABP_both
CVHWQTNTARTSCIGP
>ID01262-ABP_both
DLRFLYPRGKLPVPTLPPFNPKPIYIDMGNRY
>ID01280-ABP_both
FFSLLPSLIGGLVSAIK
>ID01285-ABP_both
FLGALFKVASKVLPSVFCAITKKC
>ID01288-ABP_both
FLGMIPGLIGGLISAFK
>ID01297-ABP_both
FLPLIAGLAANFLPKIFCAITKKC
>ID01301-ABP_both
FLPPSPWKETFRTS
>ID01319-ABP_both
FTCDVLGFEIAGTKLNSAACGAHCLALGRTGGYCNSKSVCVCR
>ID01323-ABP_both
GAFGDLLKGVAKEAGMKLLNMAQCKLSGKC
>ID01325-ABP_both
GCLEFWWKCNPNDDKCCRPKLKCSKLFKLCNFSF
>ID01334-ABP_both
GFKLKGMARISCLPNGQWSNFPPKCIRECAMVSS
>ID01354-ABP_both
GIGKFLHSAGKFGKAFLGEVMKS

>ID01355-ABP_both
GIGKHVGKALKGLKGLLKGLGES
>ID01369-NO
GIPCAESCVWIPCTVTALLGCSCSNNVCYN
>ID01371-ABP_both
GKFSVFSKILRSIAKVFKGVGKVRKQFKTASDLDKNQ
>ID01376-ABP_both
GLFGRLRDSLQRGGQKILEKAERIWCKIKDIFR
>ID01377-ABP_both
GLFGVLGSIAKHVLPHVVPVIAEK
>ID01379-ABP_both
GLFKVLGSVAKHLLPHVAPVIAEKL
>ID01386-ABP_both
GLGSLLGKAFKFGLKTVGKMMGGAPREQ
>ID01387-ABP_both
GLGSVLGKALKIGANLL
>ID01398-ABP_both
GLLSFLPKVIGVIGHLIHPPS
>ID01418-ABP_both
GLWNSIKIAGKKLFVNVLDKIRCKVAGGCKTSPDVE
>ID01419-ABP_both
GLWQKIKNAAGDLASGIVEGIKS
>ID01460-ABP_both
IIGPVLGMVGSALGGLLKKIG
>ID01498-ABP_both
KWKLFKKVLKVLTTG
>ID01503-ABP_both
LDVKKIICVACKIKPNPACKKICPK
>ID01512-NO
LPSDATLVLDQTGKELDARL
>ID01534-ABP_both
PFKISIHL
>ID01544-ABP_pos
QQCGRQASGRLCGNRLCCSQWGYCGSTASYCGAGCQSQCR
>ID01545-ABP_both
QRFSQPTFKLPQGRLTLSRKF
>ID01550-ABP_both
RCVCTRGFCRCFCRRGVC
>ID01556-NO
RECQSQSHRYKGACVHDTNCASVCQTEGFSGGKCVGFRGRCFCTKHC
>ID01559-ABP_both
RFRPPIRRPPIRPPFYPPFRPPIRPPIFPPIRPPFRPPLGPFP
>ID01562-ABP_both
RICRCRIGRCLGLEVYFGVCFLHGRLARRCCR
>ID01590-ABP_both
SDEKASPDKHHRFSLSRYAKLANRLANPKLLETFLSKWIGDRGNRSV
>ID01599-ABP_both
SLFSIFKTAAKFVGKNLLKQAGKAGLETLACKAKNEC
>ID01606-ABP_both
SPAGCRFCCGCCPNMRGCGVCCRF
>ID01613-ABP_both

TSRCYIGYRRKVVCS
>ID01626-ABP_both
VTCYCRSTRCGFRERLSGACGYRGRIYRLCCR
>ID01639-ABP_both
ACYCRIPACLAGERRYGTCFYLGRVWAFCC
>ID01647-ABP_both
AHCLAIGRK
>ID01650-ABP_both
ALWKNMLKGIGKLAGQAALGAVKTLVGAE
>ID01670-ABP_both
DFGCARGMIFVCMRRCARMYPGSTGYCQGFRCMCDTMIPIRRPPFIMG
>ID01680-NO
DKLIGSCVWLAVNYTSNCNAECKRRGYKGGHCGSFANVNCWCET
>ID01681-NO
DKLIGSCVWLAVNYTSNCNAECKRRGYKGGHCGSFLNVNCWCET
>ID01682-ABP_both
DPVTYIRNGGICQYRCIGLRHKIGTCGSPFKCCK
>ID01693-ABP_both
EGVRNFVTCRINRGFCVPIRCPGHRRQIGTCLGPRIKCCR
>ID01695-ABP_both
EGVRSYLSCWGNRGICLLNRCPGRMRQIGTCLAPRVKCCR
>ID01696-ABP_both
EPFKLSLHL
>ID01709-ABP_both
FDVMGIIKKIAGAL
>ID01717-ABP_both
FKSWSFCTPGCAKTGSFNSYCC
>ID01727-ABP_both
FLPIVGKLLSGLF
>ID01730-ABP_both
FLPVIAGLAAKVLPKLFCAITKKC
>ID01737-ABP_both
GASCGETCFTGICFTAGCSCNPWPTCTRN
>ID01752-ABP_neg
GFRDVLKGAAKQFVKTVAGHIANI
>ID01757-ABP_both
GGLIKIVPAMICAVTKKC
>ID01769-ABP_both
GIGGALLSAGKSALKGLAKGLAEHFAN
>ID01774-ABP_both
GIKDWIKGAAKKLIKTVASNIANQ
>ID01780-NO
GIPCGESCVYIPCLTSAVGCSCKSKVCYRN
>ID01783-ABP_both
GLASTIGSLLGKFAKGGAQAFLQPK
>ID01786-ABP_both
GLIKIVPAMICAVTKKC
>ID01802-NO
GLPVCGETCFGGTCNTPGCSCDPWPMCSRN
>ID01805-ABP_both
GLVTGLLKTAGKLLGDLFGSLTG

>ID01806-ABP_both
GLWNTIKEAGKKFAINVLDKIRCGIAGGCKT
>ID01808-ABP_both
GLWSKIKETGKEAAKAAGKAALNKIAEAV
>ID01811-ABP_both
GPDSCNHDRGLCRVGNCNPGEYLAKYCFEPVILCCKPLSPTPTKT
>ID01823-ABP_both
GVIIDTLKGAAKTVAAELLRKAHCKLTNSC
>ID01832-ABP_both
GWKKWFNRAKKVGKTVGGLAVDHYLG
>ID01834-ABP_both
GYGCPFNQYQCHSHCSGIRGYKGGYCKGTFKQTCKCY
>ID01853-ABP_both
INWLKLGKMVIDAL
>ID01867-ABP_neg
KKINNPVSCLRKGGRCWNRCIGNTRQIGSCGVPFLKCCKRK
>ID01869-ABP_pos
KKKSGVIPTVSHDCHMNSFQFVFTCCS
>ID01887-ABP_pos
KSYGNGVHCNKKKCWVDWGSAISTIGNNSAANWATGGAAGWKS
>ID01911-ABP_both
LKLLKKLLKLLKKLGGK
>ID01914-ABP_both
LMDTVKNVAKNLAGHMLDKLKCKITGC
>ID01915-ABP_both
LNLKGLFKKVASLLT
>ID01916-ABP_both
LNLKGLIKKVASLLN
>ID01920-ABP_both
LQDAALGWGRRCPQCPRCPSCPSCPRCPRCPRCKCNPK
>ID01929-ABP_neg
LVLKYCPKIGYCSNTCSKTQIWATSHGCKMYCCLPASWKWK
>ID01950-NO
NKLIGSCVWGAVNYTSNCNAECKRRGYKGGHCGSFANVNCWCET
>ID01952-ABP_both
NPVSCVRNKGICVPIRCPGNMKQIGTCVGRAVKCCRKK
>ID01963-ABP_neg
QKKCPGRCTLKCGKHERPTLPYNCGKYICCVPVKVK
>ID01967-ABP_neg
QLKKCWNNYVQGHCRKICRVNEVPEALCENGRYCCLNIKELEAC
>ID01983-NO
RILSILRHQNLLKE
>ID01988-ABP_both
RRCICTTRTCRFPYRRLGTCLFQNRVYTFCC
>ID02000-ABP_pos
RTCRCRFGRCFRRESYSGSCNINGRIFSLCCR
>ID02007-ABP_both
SALVGCWTKSYPPKPCFGR
>ID02012-ABP_both
SGTSEKERESGRLLGVVKRLIVCFRSPFP
>ID02024-ABP_both

TCSYTMEA
>ID02037-ABP_neg
VGIGGGGGGGGGGSCGGQGGGCGGCSNGCSGGNGGSGGSGSHI
>ID02045-ABP_pos
WKSESVCTPGCVTGLLQTCFLQTITCNCKISK
>ID02078-NO
CGESCAMISFCFTEVIGCSCKNKVCYLNSIS
>ID02081-ABP_pos
CLGVGSCNDFAGCGYAVVCFW
>ID02092-ABP_neg
DIPPGIRNTVCFMQRGHCRLFMCRSGERKGDICSDPWNRCCVSSSIKNR
>ID02094-NO
DKLIGSCVWGAVNYTSNCRAECKRRGYKGGHCGSFLNVNCWCET
>ID02157-ABP_both
FVPYNPPRPYQSKPFPSFPGHGPFNPKIQWPYPLPNPGH
>ID02166-ABP_both
GFGCPNNYQCHRHCKSIPGRCGGYCGGWHRLPCTCYRCG
>ID02171-ABP_both
GFLGPLLKLGLKGVAKVIPHLIPSRQQ
>ID02174-ABP_both
GFMDTAKNVAKNVAVTLLDNLKCKITKAC
>ID02183-ABP_both
GIFLDKLKNFAKGVAQSLLNKASCKLSGQC
>ID02191-ABP_both
GIGKFLHSAKKFAKAFVAEIMNS
>ID02194-ABP_both
GILSGILGVGKKLVCGLSGLC
>ID02196-ABP_both
GILSGLLGVGKMLVCGLSGLC
>ID02208-ABP_both
GLFNVFKGLKTAGKHVAGSLLNQLKCKVSGGC
>ID02213-ABP_both
GLFSVVTGVLKAVGKNVAKNVGGSLLEQLKCKKISGGC
>ID02216-ABP_both
GLISGILGVGKMLVCGLSGLC
>ID02221-ABP_both
GLLDTIKNTAKNLAVGLLDKIKCKMTGC
>ID02232-ABP_both
GLVSSIGKVLGGLLADVVKSKGQPA
>ID02238-ABP_both
GLWSTIKNVGKEAAIAAGKAALGALGEQ
>ID02240-ABP_both
GMASKAGSIVGKIAKIALGAL
>ID02258-ABP_both
IGKEFKRIVQRIKDFLRNL
>ID02272-ABP_both
IWLTALKFLGKNLGKHLAKQQLAKL
>ID02279-ABP_both
KISGKAIKNLFIKGAKNVGKEVGMDVVRTGIDVVGCKIKGEC
>ID02288-ABP_both
KRFHSVGSLIQRHQQMIRDKSEATRHGIRIITRPKLLLAS

>ID02290-ABP_both
KRKKHRCRVYNNGMPTGMYRWC
>ID02298-ABP_neg
KWKKFIKNLTKGGSKILTTGLPALIS
>ID02299-ABP_both
KWKLFKKIGPGKFLHSAKKF
>ID02310-ABP_both
LKKLLKKLKKLL
>ID02312-ABP_both
LKLLKKLLKLLKKL
>ID02409-ABP_pos
SILPTIVSFLSKVF
>ID02470-ABP_neg
ATCDLFSFRSKWVTPNHAGCAAHCIFLGNRGGRCVGTVCHCRK
>ID02477-NO
CGESCVFIPCITSVAGCSCKSKVCYRNGIP
>ID02536-ABP_both
FLKWLFKWAKK
>ID02583-ABP_both
GILGKLWEGVKSIF
>ID02595-NO
GIPCGESCVYIPCTVTALLGCSCKDKVCYKN
>ID02612-ABP_both
GLLSLLSLLGKLL
>ID02676-ABP_both
INLKAIAPLAKKLL
>ID02678-ABP_both
INWKKLLDAAKQIL
>ID02686-ABP_both
KIGAKIKIGAKIKIGAKI
>ID02690-ABP_both
KKLALALAKKWLPLAKKLALALAKK
>ID02702-ABP_both
KRWWKWWRRC
>ID02712-NO
KVDFLEENITALLEEAQIQQEKNMYELQKLNSWDV
>ID02715-ABP_both
KWKSFLKTFKSLKKTVLHTLLKLISS
>ID02717-ABP_both
KWLNALLHHGLNCAKGVLA
>ID02718-ABP_both
KWLNALLKKGLNCAKGVLA
>ID02810-ABP_both
NLKAIAALAKKLL
>ID02855-ABP_both
RRRRRRPPCEDVNGQCQPRGNPCLRLRGACPRGSRCCMPTVAAH
>ID02857-ABP_both
RRWQWRMKKLG
>ID02868-ABP_both
RWRWRW
>ID02872-ABP_both

SIRDKIKTMAIDLAKSAGTGVLKTLICKLDKSC
>ID02881-ABP_both
SLWETIKNAGKGFIQNLDKIR
>ID02882-ABP_both
SMWSGMWRRKLKKLRNALKKKLKGEK
>ID02901-ABP_neg
VGIGTPIFSYGGGAGHVPEYF
>ID02903-ABP_both
VIPFVASVAAETMQHVYCAASKKMLKLNWKSSDVENHLAKC
>ID02921-NO
WQEWERKVDFLEENITALLEEAQIQQEKNMYELQK
>ID02933-ABP_both
AAHCLAIGRR
>ID02938-ABP_both
ADTLACRQSHGSCSFVACRAPSVDIGTCRGGKLKCCKWAPSS
>ID02969-ABP_both
ATCDLLSGTGANHSACAAHCLLRGNRGGYCNSKAVCVCRN
>ID02993-NO
DTLIGSCVWGATNYTSDCNAECKRRGYKGGHCGSFLNVNCWCEE
>ID03010-ABP_both
EYHLMNGANGYLTRVNGKYVYRVTKDPVSAVFGVISNGWGSAGAGFGPQH
>ID03022-ABP_both
FFSLIPSLVGGLISAFK
>ID03031-ABP_both
FISGLIGGLMKAL
>ID03034-ABP_both
FKIPPIVKDTLKKFFKGGIAGVMGQ
>ID03040-ABP_both
FLFSLIPSAIAGLVSAIRN
>ID03047-ABP_both
FLPGLIKAAVGIGSTIFCKISKKC
>ID03049-ABP_both
FLPGLIKAAVGVGSTILCKITKKC
>ID03053-ABP_both
FLPIVASLAANFLPKIICKITKKC
>ID03054-ABP_pos
FLPIVGRLISGIL
>ID03063-ABP_both
FLPMLAGLAANFLPKIICKITKKC
>ID03084-ABP_both
FPVTWRWWKWWKG
>ID03095-ABP_both
GAFGNLLKGVAKKAGLKILSIAQCKLSGTC
>ID03103-ABP_both
GFASFLGKALKAALKIGANMLGGTPQQ
>ID03113-ABP_both
GFKEVLKAGLGSLVKGIPAHVAN
>ID03125-ABP_both
GIFPIFAKLLGKVIKVASSLISKGRTE
>ID03131-ABP_both
GIGAILKVLSTGLPALISWIKRKRQQ

>ID03153-NO
GIPCGESCVYIPCTVTALAGCKCKSKVCYN
>ID03163-ABP_both
GLFLDTLKKFAKAGMEAVINPK
>ID03180-ABP_both
GLLSVLKGVLKTAGKHIFKNVGGSLLDQAKCKISGQC
>ID03197-ABP_both
GLVKKIGKKIERVGQHTRDASIQAIGIAQQAANVAATARG
>ID03208-ABP_both
GLWNSIKIAGKKLFVNVLDKIRSKVAGGS
>ID03219-ABP_both
GPIRRPKPRPRQRPE
>ID03224-ABP_both
GRFRRLRKKTRKRLKKIGKVLKWIPPIVGSIPLGC
>ID03238-NO
GTLPCGESCVWIPCISAVGCSCKSKVCYKN
>ID03245-ABP_both
GVGKFLHSAKKFGQALASEIMKS
>ID03258-ABP_both
GWLKKFGKKIERVGQHTRDATIQAIGVAQQAANVAATLKG
>ID03264-ABP_both
GWVACVGACGTVCLASGGVGTEFAAASYFL
>ID03271-ABP_pos
HSSGYTRPLPKPSRPIFIRPIGCDVCYGIPSSTARLCCFRYGDCCHR
>ID03281-ABP_both
IGKKFKRIVQRIKKFLRKL
>ID03282-ABP_both
IGKKFKRIVQRIKKFLRNL
>ID03287-ABP_both
IKIPSFFRNILKKVGKEAVSLIAGALKQS
>ID03298-ABP_both
ILSAIWSGIKGLL
>ID03306-ABP_both
ITIPPIIKDTLKKFFKGGIAGVMGKSQ
>ID03310-ABP_both
ITIPPIVKDTLKKFIKGAISSVM
>ID03318-ABP_both
KIAGKIAKIAGKIAKIAGKIA
>ID03332-ABP_neg
KKLKLAPAKLALLWKALALKLKKA
>ID03341-ABP_both
KLKLLLLLKLK
>ID03372-ABP_both
KWKLFKKIPFLHLAKKF
>ID03373-ABP_both
KWKLFKKIPHLAKKF
>ID03374-ABP_both
KWKLFKKIPKFLH
>ID03377-ABP_both
KWKLFKKIPKFLHLAK
>ID03378-ABP_both

KWKLFKKIPKFLHLAKK
>ID03381-ABP_both
KWKLFKKIPLKKF
>ID03383-ABP_neg
KWKSFIKKLASKFLHSAKKF
>ID03389-ABP_neg
KWKSFIKKLTSKFLHSADKF
>ID03403-ABP_both
KWKWKW
>ID03422-ABP_both
LKLSPETKDTLKKVLKGAIKGAIAIASLA
>ID03434-ABP_both
LQDAALGWGRRCPRCPPCPRCSWCPRCPTCPRCNCNPK
>ID03445-NO
LVEAKQARSDIEKLKEAIRDTNKAVQSVQSSIGNL
>ID03541-NO
NKIALLSTNKAVVSLSNGVSVLTSKVLDLKNYI
>ID03542-ABP_both
NLLGSLLKTGLKVGSNLL
>ID03560-NO
QARSDIEKLKEAIRDTNKAVQSVQSSIGNLIVAIK
>ID03571-NO
QPDPNAFYGLM
>ID03574-ABP_both
QSHLSLCRYCCNCCRNKGCGYCCKF
>ID03582-NO
RDCESDSHKFHGACFSDTNCANVCQTEGFTAGKCVGVQRHCHCTKDC
>ID03593-ABP_both
RGRKSSRRK
>ID03595-NO
RIDLGPPISLERLDVGTNLGNAIAKLEAKELLES
>ID03596-ABP_both
RIKRFWPVVIRTVVAGYNLYRAIK
>ID03611-ABP_both
RQRDPQQQYEQCQERCQRHETEPRHMQTCQQRCERRYEKEKRKQQKR
>ID03627-NO
RTCQSQSHKFKGACFSDTNCASVCRTENFPRGQCNQHHVERKCYCERDC
>ID03629-NO
RVCMGKSQHHSFPCISDRLCSNECVKEEGGWTAGYCHLRYCRCQKAC
>ID03643-NO
SIELNKAKSDLEESKEWIRRSNQKLDSIGNWHQSS
>ID03654-ABP_pos
SNDSLWYGVGQFMGKQANCITNHPVKHMIIPGYCLSKILG
>ID03662-ABP_both
SVKKFWGGVKAIFKGARKGLK
>ID03669-ABP_both
TKCFQWQRNMRKVRGPPVSCIKRDS
>ID03698-ABP_both
VRFRIRVAVIRA
>ID03704-ABP_both
WFKKIPKFLHLAKKF

>ID03707-ABP_both
WKKIPKFLHLAKKF
>ID03709-ABP_both
WKLFKKIPKFLHLAKKF
>ID03711-ABP_both
WLNALLHHGLNCAKGVL
>ID03713-ABP_pos
WNDTGKDADGSEY
>ID03747-ABP_both
AKKPVAKKAAGGVKKPKK
>ID03751-ABP_both
AKKVFKRLEKSFSKIQNDK
>ID03763-ABP_neg
ALYKRLFKKLKKF
>ID03764-NO
ALYNSEDLYEETSDSDD
>ID03808-ABP_both
AVNIPFKVHLRCKAAFC
>ID03822-NO
CNYVFPAHK
>ID03825-NO
CNYVFPAHKCICYF
>ID03830-ABP_both
CTTCECCCSCS
>ID03846-ABP_neg
DKLIGSCVWGAVNYTSDCAAEKRRGYKGGYCGSFANVNCWCET
>ID03852-ABP_both
DPGQPWQVKAGRPPCYSIPCRKHDECRVGSCSRCNNGLWGDRTCR
>ID03891-ABP_both
ESYFVFSVGM
>ID03904-ABP_both
FFLSRIFGK
>ID03907-ABP_both
FGCGQGMIFMCQRRCMRLYPGSTGFCRGFRCMCDTHIPLRPPFMVG
>ID03918-ABP_neg
FKIKASKVLDKFGKIVGKVLKQLKKVSAVAKV
>ID03922-NO
FKKLKKIANIINSIFKK
>ID03932-ABP_both
FLPIIAGMAAKVIPSLFCAITKKC
>ID03938-ABP_both
FLPVIAGVAAKFLPKIACAITKKC
>ID03946-ABP_both
FPLALLCKVFKKC
>ID03964-NO
FVKLKKILNIILSIFKK
>ID03969-ABP_both
FVQHRPRDCESINGVCRHKDTVNCREIFLADCYNDGQKCCRK
>ID03971-ABP_both
FWGALAKGALKLIGSLFSSFSKKD
>ID03976-NO

GAGSQEERMQGQMEGQDFSHEERFLSMVRE
>ID03983-ABP_both
GFCCPGNQLKCNNHCKSISCRAGYCDAATLWLRCTCTDCNGKK
>ID03986-ABP_both
GFGCPLNQGACHNHCRSIKRRGGYCSGIIKQTCTCYRK
>ID03988-NO
GFGSFLGKALKAALKIGANALGGSPQQ
>ID03994-ABP_both
GFLDIVKGVGKVALGAVSKLF
>ID04016-ABP_both
GIIKKIIKKI
>ID04028-NO
GIKQFKRIVQRIKDFLRNLV
>ID04051-NO
GLASFLGKALKAGLKIGAHLLGGAPQQ
>ID04057-NO
GLFDIIKKVASVVGLASQ
>ID04062-ABP_both
GLFGVLAKVAAHVVAAIAEHF
>ID04073-ABP_both
GLLSSLGSVAKHVLPHVVPVIAEHL
>ID04078-NO
GLPTCGETCFGGTCNTPGCTCDPWPICTD
>ID04084-NO
GLWQLIKDKFKDAATGFVTGIQS
>ID04098-ABP_both
GRKSDCFRKSGFCAFLKCPSLTLISGKCSRFYLCCKRIWG
>ID04109-ABP_neg
GSVIKCGESCLLGKCYTPGCTCSRPICKKD
>ID04112-NO
GTPCGSSCVYIPCISGVIGCSCTDKVCYLN
>ID04120-ABP_both
GVIDAAAKVVNVLKNLF
>ID04139-NO
HGSCNYVFPAHKC
>ID04142-NO
HGSCNYVFPAHKCICY
>ID04177-ABP_both
ILPFKFPFFPFRR
>ID04187-NO
INNWVRVPPCDQVCSRSNPEKDECCRAHGHAFHAHCNGGMNCYRR
>ID04196-ABP_both
INWKKIFESVKNLV
>ID04197-ABP_both
INWKKIFQKVKNLV
>ID04198-ABP_both
INWKKLGKKILGAL
>ID04201-ABP_both
INWLKLGKKILGAI
>ID04206-ABP_both
INWSSIFESVKNLV

>ID04211-ABP_both
IRCPKSWKCKAFKQRVLKRLLAMLRQHAF
>ID04220-ABP_both
IRWRIRVWVRRI
>ID04230-NO
KARHGSCNYVFP
>ID04231-NO
KARHGSCNYVFPA
>ID04232-NO
KARHGSCNYVFPAH
>ID04235-NO
KARHGSCNYVFPAHKCI
>ID04236-NO
KARHGSCNYVFPAHKCIC
>ID04237-NO
KARHGSCNYVFPAHKCICY
>ID04242-ABP_both
KFEPPLPPKKAHKKFWEDDGIYYPPNHNFP
>ID04247-ABP_both
KIASIGKEVLKAL
>ID04280-ABP_both
KKLLKWLLKLL
>ID04293-ABP_neg
KLKSLLKTLSKAKKKKLKTLLKALSK
>ID04305-ABP_both
KNWKKIASIGKEVLKAL
>ID04315-ABP_both
KRFKKFFKKLK
>ID04316-ABP_neg
KRFRIRVAVRRA
>ID04317-ABP_both
KRFRIRVRV
>ID04325-ABP_both
KRRWPWWPWRLI
>ID04326-ABP_both
KRWRIRVRVIRK
>ID04328-NO
KSCCKDIMARNCYNVCRIPGTPRPVCATTCRCKIISGNKCPKDYPK
>ID04331-NO
KSCCKNTTGRNCYNACHFAGGSRPVCATACGCKIISGPTCPRDYPK
>ID04352-ABP_both
KWKLFKKIGIGAVLKVLTTGLPALKLTK
>ID04354-ABP_neg
KWKSFIKKLTKAAKKVVTTAKKPLIV
>ID04356-ABP_neg
KWKSFIKKLTSAAKKVVTTAKPLALIS
>ID04359-ABP_both
KWLKKLLKKLL
>ID04377-NO
LEKARHGSCNYVFPAHKC
>ID04383-ABP_both

LFSLIPHAIGGLISAFK
>ID04393-ABP_both
LKKWLKKWLKK
>ID04399-ABP_both
LKWLKKWLK
>ID04401-ABP_both
LKWLLKWLL
>ID04411-ABP_pos
LLKKLLKK
>ID04412-ABP_both
LLKKLLKWLKK
>ID04421-ABP_both
LLKWLLKWLKK
>ID04427-ABP_both
LPKMSTKLRVPYRRGTKDYH
>ID04428-ABP_pos
LPLIASLAANFVPKIFCKITKKC
>ID04433-ABP_both
LRRIIRKIIHIIK
>ID04442-NO
LVQPRGPRSGPGPWQGGRRKFRRQRPRLSHKGPMPF
>ID04443-ABP_both
LVQRGRFGRFLRKIRRFRPKVTITIQGSARF
>ID04446-ABP_both
LYRRRFVVGR
>ID04528-ABP_neg
MPKWKVFKKIEKVGRNIRNGIVKAGPAIAVLGEAKALG
>ID04565-NO
MTPFWRAVSLRPIGASCRDDSECLTRLCRKRRCSLSVAQE
>ID04581-ABP_both
NIRRIIRKIIHIIKKY
>ID04582-ABP_both
NIWKKIASIAKEVLKAL
>ID04599-ABP_neg
PEWFKCRRWQWRMKKLGA
>ID04610-NO
QIGTCFGRPVK
>ID04621-NO
QKLCQRPSGTWSGVCGNNNACRNQCI
>ID04634-ABP_both
QSHISLCRWCCNCCKANKGCGFCCKF
>ID04659-NO
RHGSCNYVFPAHKCICY
>ID04667-ABP_both
RIKRFWPVVIRTVVAG
>ID04670-ABP_both
RIKRFWPVVIRTVVAGYNL
>ID04674-ABP_both
RIKRFWPVVIRTVVAGYNLYRAI
>ID04680-NO
RLEKARHGSCNYV

>ID04681-NO
RLEKARHGSCNYVF
>ID04732-ABP_both
RWFKIQMQIRRWKNKK
>ID04756-ABP_both
SIGSAFKKAAHVGKHVGKAALGAAARRRK
>ID04760-NO
SKEKIGKEFKRIVQRIKDFLR
>ID04768-ABP_both
SNWLKLGKKMMSAL
>ID04784-NO
SWLRDIWDWSCEVLSDFK
>ID04786-NO
SWLRDLWDWICEVLSDFK
>ID04787-NO
SWLRDLWDWLCELLSDFK
>ID04790-NO
TDDRCERMCQHYHDRREKKQCMKGCRYGESD
>ID04819-NO
VFPAHKCICYFP
>ID04833-ABP_both
VQLRIRVRVIRK
>ID04860-ABP_both
WKSYVRRWRSRY
>ID04864-ABP_both
WLSKTAKKLENSAKKRISEGIAIAIKGGSR
>ID04879-NO
YCERSSGTWSGVCGNTDKCSSQCQRLEGAAHGSCNYVFPAHKCICYYPC
>ID04899-NO
AAVALVEAKQARSDIEKLKEAIRDTNKAVQSVQSS
>ID04904-NO
ACGWAGIKQEF
>ID04915-NO
ACWWAGIKQEA
>ID04934-NO
AEAMSQVTNTATIM
>ID04962-ABP_both
AIPWIWIWRLLRKG
>ID04964-ABP_both
AIPWSIWWHLLFKG
>ID04988-ABP_both
AKKVFKRLEKLFS
>ID04990-ABP_both
AKKVFKRLEKLFSKIQN
>ID04999-NO
AKVAKQEKKKKKTGRAKRRA
>ID05004-NO
ALLDKLKSLGKVVGKVALGVAQHYLNPQQ
>ID05013-ABP_both
ALWKTLLKKVLKAAAKAALNAVLVGANA
>ID05014-NO

ALWKTMLKKAAHVGKHVGKAALTHYL
>ID05015-NO
ALWKTMLKKLGTMALHAGKAALGAAADTISQTQ
>ID05018-ABP_neg
ALWMTLLKKVLKAAAKAALDAVLVGANA
>ID05021-NO
ALYKKKIIKKLLES
>ID05088-ABP_both
AWKVFKRLGIGAVLWVLTWG
>ID05089-NO
AWLDKLKNLGKVVGKVALGVVQNYLNPRQ
>ID05098-NO
CATCEQIADSQHRSHRQ
>ID05099-NO
CATCEQIADSQHRSHRQM
>ID05120-ABP_neg
CKLLKTFLSKWIC
>ID05121-ABP_both
CKLLLKWLLKLLKC
>ID05142-ABP_both
DAACAAKCLWR
>ID05155-NO
DFASCHTNGGICLPNRCPGHMIQIGICFRPRVLCCRSW
>ID05158-NO
DGPKKKKKKSPSKSSK
>ID05175-NO
DLWETLRRIIRWILAIPRRIRQGLELCL
>ID05178-NO
DNPSLSIDLTFHLLRTLLELARTQSQRERAEQNRIIFDSV
>ID05188-NO
DSHAKRHHGYKIKFHENHHSHRGY
>ID05238-ABP_both
EWKLPDLIINHITLTRRNCNKYRCG
>ID05240-NO
EWRKKRYSTQV
>ID05248-ABP_both
FAKWAFKWAKK
>ID05253-ABP_neg
FALLGDFFRKSKEKIGKEFKRIVQRIKDFFRKLVPRTES
>ID05254-ABP_neg
FALLGDFFRKSKEKIGKEFKRIVQRIKDFFRNLVPRTES
>ID05258-NO
FDASISQVNEKINQSLAFIRKSDELLHNVNAGKST
>ID05275-ABP_both
FFHHIFRGIVHVAKTIHRLVTG
>ID05282-NO
FFKKFPFFPFKKK
>ID05283-NO
FFKKFPFFPFRRK
>ID05286-ABP_both
FFPIVGKLLFGKLL

>ID05288-ABP_both
FFPLIAGLAARFLPKIFCSITKRC
>ID05316-ABP_both
FIKHFIHRFGGGRWRWRWF
>ID05324-NO
FKAFKAFKAFKAFKAFKAFKAFKAFKA
>ID05337-ABP_both
FKRLEKLFKKIWNWK
>ID05341-NO
FKTWKRPPFQTSCSGIIKE
>ID05348-ABP_both
FLFSLIPSAISGLINAFK
>ID05350-NO
FLGALFHALSKLL
>ID05355-ABP_both
FLGGLIKKWPWWPWRR
>ID05356-ABP_both
FLGGLIKPVPAMICAVRKKC
>ID05361-ABP_both
FLGGLIKWPWWPWRR
>ID05367-NO
FLIRQLIELLTWLFSNCRTLLSEVY
>ID05371-NO
FLIRQLIRLLTWLFSNCRTLLSRVY
>ID05383-NO
FLPFLAGLAANFLPKLFCKITRKC
>ID05386-ABP_both
FLPILGNLLNGLL
>ID05387-ABP_both
FLPLAGRVLSGIL
>ID05393-ABP_both
FLPLIGRVLAGIL
>ID05394-ABP_both
FLPLIGRVLSAIL
>ID05399-NO
FLPLLAGLAANFLPKLFCKITRKC
>ID05421-NO
FRKQNPDIVIYQYMD
>ID05426-NO
FTLSLDVPTNIMNLLFNIAKAKNLRAQAAANAHLMAQI
>ID05436-ABP_both
FVQWFSKFLLRIL
>ID05439-ABP_both
FVRWFSRFLGRIL
>ID05441-ABP_both
FWGALAKGALKLIGVSLFSSFSKKD
>ID05452-ABP_both
GAGALAKFLAKKVAKTVAKQAAKQGAKYVVNKQME
>ID05458-NO
GAYRAIRHIPRRIR
>ID05469-ABP_both

GEKLKKIGQKIKNFFKKL
>ID05476-ABP_both
GFFALIPKIISSPLFKTL
>ID05477-ABP_both
GFFALIPKIISSPLFKTLLSAV
>ID05478-ABP_both
GFFALIPKIISSPLFKTLLSAVGSALS
>ID05479-NO
GFFALIPKIISSPLFKTLLSAVGSALSSSGDQE
>ID05481-ABP_both
GFFGKRKEYFKKFGASFKRRFANLKKRL
>ID05495-NO
GFGSLGKALRLGANVL
>ID05500-ABP_both
GFKDLLKGAKKALVKTVLF
>ID05501-ABP_both
GFKDLLKKAAKALVKTVLF
>ID05506-ABP_both
GFLSILKKVL
>ID05509-ABP_both
GFLSILKKVLKKVMAHMK
>ID05517-NO
GFLSTVKNLATNVAGTVIDTIKCKVTGGC
>ID05519-ABP_both
GFMKYIGPLIPHAVKAISKLI
>ID05521-ABP_both
GFMKYIKPLIPHAVKAIKKLI
>ID05526-ABP_both
GFVALLKKLPLILKHLH
>ID05534-ABP_both
GGLKKLGKKLEGVGKRVFKASEKALPVAVGIKALG
>ID05547-NO
GICRCICGKKICRCICGR
>ID05555-NO
GICRCYCGRGICRCICGR
>ID05557-NO
GICYCICGRGICRCICGR
>ID05581-NO
GIGKFLHSAKKFKAFVGEIMN
>ID05584-ABP_both
GIGKFLKKAKKFGKAFVKMKK
>ID05587-ABP_both
GIGPVTCLKSGAICHPVFCPRRYKQIGTCGLPGTKCCKKP
>ID05598-ABP_both
GIHDILKYGKPA
>ID05608-ABP_neg
GILKTIKSIASKLKRKAK
>ID05610-ABP_neg
GILKTIKSIASKVANTVQKLKRKAKNAVA
>ID05614-ABP_both
GIMDTIKGAAKDLAGQLLDKLKCKITKC

>ID05617-ABP_both
GIPCAESCVWIPCTVTALLGCSCKDKVCYLD
>ID05618-ABP_both
GIRDILKYGKPS
>ID05619-NO
GIRKWFKKAAHVGKEVGKVALNACL
>ID05633-ABP_both
GKKLLKKLKKLLKKW
>ID05637-NO
GKKYRRFRWKFKFGKWFWFG
>ID05647-ABP_both
GKWKKILGKLIR
>ID05649-NO
GKWKLFKKAFKKFLKILAC
>ID05667-ABP_both
GLFAVIKKVASVIKGL
>ID05679-ABP_both
GLFGKLQKKFGRKAISYAVKKARGKH
>ID05690-ABP_neg
GLKDWWNKHKDKIVEVVKDSGKAGLNAA
>ID05692-ABP_both
GLKKIFKAGLGSLKKGIAAHVAS
>ID05693-ABP_both
GLKKIFKAGLGSLVKGIAAHVAS
>ID05697-NO
GLKKWFKKAVHVGKKVGKVALNAYL
>ID05701-NO
GLLDTFKNLALNAAKSAGVSVLNSLSCKLFKTC
>ID05702-NO
GLLDTFKNLALNAPKSAGVSVLNSLSCKLSKTC
>ID05706-NO
GLLDTLKNMATNAAKGAGVSVLKALSCKLFKTC
>ID05714-ABP_both
GLLGPLLKIAKKVGSNLL
>ID05718-NO
GLLQTITEKLKEFAGGLVTGVQS
>ID05722-NO
GLLSGILNSAGGLLGNLIGSLSNGES
>ID05733-ABP_both
GLPALISWIKRKRQQG
>ID05734-ABP_both
GLPALISWSKRKRQQ
>ID05735-ABP_both
GLPALSSWIKRKRQQ
>ID05741-ABP_both
GLPRKILAAIAKKKGKAKGPLKLVAKA
>ID05743-ABP_both
GLPRKILCAIAKKKGKAKGPLKLVCKA
>ID05748-NO
GLPVCGETCVGGTCNTPGCNCSWPVCTRK
>ID05765-NO

GMASKGAIAGKIAKVALKAL
>ID05768-ABP_both
GMWKKILGHLIR
>ID05771-ABP_both
GMWSKILGHLKR
>ID05772-ABP_both
GMWSKILGKLIR
>ID05782-NO
GPLSCRRKGGICILIRCPGPMRQIGTCFGRPVKCCRSW
>ID05786-NO
GRFKRFRKKFKKLFKKIS
>ID05791-NO
GRKKRRRRRR
>ID05793-ABP_both
GRLKKLGKKIEGAGKRVFKAAEKALPVVAGVKAL
>ID05798-ABP_both
GRSPRRRTPSPRRRRSQSPRRRR
>ID05814-NO
GSSKSPSKKKKKKPGDC
>ID05826-NO
GVCRCICGRGVCRCICRR
>ID05828-ABP_both
GVFDIIKGAGKQLIAHAMGKIAEKV
>ID05844-ABP_both
GWGSFFKKAAHVAKHVAKAALTHYL
>ID05845-ABP_both
GWGSFFKKAAHVAKHVGKAALTHYL
>ID05848-NO
GWGSFFKKAAHVGKHVGKAALT
>ID05850-ABP_both
GWLDVAKKIGKAAFNVAKNFLFNKAVNFAAKGIKKAVDLWG
>ID05868-NO
HAEGTFTSDVSSYLEGQAAKEFIAWLVKGRG
>ID05869-ABP_neg
HAEHKVKIKVKQKYGQFPQGTEVTYTCSGNYFLM
>ID05884-NO
HGEGTFTSDLSKQMEEEAVRLFIEWLKNGGPSSGAPPPS
>ID05929-NO
HRILMRIRQMMA
>ID05957-ABP_both
IIGLVSKGTCVLVKTVCKKVLKQ
>ID05960-ABP_both
IISTIGDLVKWIIKTV
>ID05961-ABP_both
IISTIGKLVKWIIDTV
>ID05980-ABP_both
ILGKLLSTAAKLLSNL
>ID05982-NO
ILGKLLSTAWGLLSNL
>ID06000-NO
ILKKWPWWPWK

>ID06001-NO
ILKKWPWWPWKKK
>ID06007-NO
ILKKWPWWPWRRIM
>ID06018-NO
ILKKWPWWWRRK
>ID06022-NO
ILKKWWWPWRRK
>ID06028-NO
ILKWPWWPWRRK
>ID06029-NO
ILKWVWWVWRRK
>ID06030-ABP_both
ILPAKAPAAPARR
>ID06032-ABP_both
ILPKKWPKLPWRR
>ID06036-ABP_both
ILPLKLPWWPWRR
>ID06037-NO
ILPWGWPWWPWRR
>ID06052-ABP_both
ILPWKWPWLPLRR
>ID06059-NO
ILPWKWPWWPWRKWR
>ID06079-NO
ILRWPWWPWARK
>ID06097-ABP_both
INPKSVQSLL
>ID06127-ABP_neg
IVPFLLGMVPKLVCLITKKC
>ID06132-ABP_both
IWEGIKNAGKGFLVSILDKVRCKVAGGCNP
>ID06150-NO
KCNTATCATQRLANFLVRSSNNLGPVLPPTNVGSNTY
>ID06156-ABP_both
KETWWETWWTEWSQPKKKRKV
>ID06163-NO
KFAKKFAKKFAKKAAK
>ID06166-NO
KFAKKFAKKFAKKFAKKFAKKFAK
>ID06174-ABP_both
KFFARLLASVRAAVKKFRKKPRLIGLSTLL
>ID06184-NO
KFKKFKKFKKFKKFKKFKKFKKFKKFK
>ID06210-ABP_both
KISKRILTGKK
>ID06211-ABP_both
KIWVIRWR
>ID06226-ABP_neg
KKKKKKAAFAAWAAFLL
>ID06245-ABP_both

KKLLKLKLKWKK
>ID06260-NO
KKWWRRALQGLKTAGPAIQSVLNK
>ID06266-NO
KLAKKLAKLAKLAKAL
>ID06277-NO
KLKKALRALARHWK
>ID06284-NO
KLKLKLKLKLK
>ID06288-ABP_both
KLLGPLLKIAAKVGSNLL
>ID06297-NO
KLLLKLLKLLLKLLLKLLK
>ID06308-ABP_both
KMWSKILGHLIR
>ID06313-NO
KNLRRGIRKIIHIIKKYG
>ID06316-NO
KNLRRIERKIIHIIKKYG
>ID06321-ABP_both
KNLRRIIRKGIHIIKKYF
>ID06322-NO
KNLRRIIRKGIRIIKKYG
>ID06326-NO
KNLRRIIRKIGHIIKKYG
>ID06336-ABP_both
KNWGKILKHIIK
>ID06342-NO
KNWKGIAGMAKKLLGKNWELMKK
>ID06347-NO
KNWKKILKKIIKVVK
>ID06372-NO
KRLFKELFFSLRKY
>ID06383-ABP_both
KRWWKWIRW
>ID06388-NO
KSCCPSTSVRNVYNSCRFAGGSREACAKLSTCKHFDGSCQPPYDH
>ID06392-NO
KSCCPSTTARNVYNSCRFAGGSRNTCAKLSGCKIVDGNCEPPYVH
>ID06394-NO
KSCCPTTTARNIYNACRFALGTRERCSKLSGCKIVDGKCKPPYIH
>ID06400-NO
KSYKCLHKRCR
>ID06404-NO
KTCENLADTYRGPCFTTGSCDDHCKNKEHLLSGRCRDDVRCWCTRNC
>ID06427-NO
KWKKFIKELQKVLKPGGLLSNIVTSL
>ID06429-ABP_both
KWKKFKKIGAVLKVL
>ID06433-ABP_both
KWKKLLKKLLKLLKKLLK

>ID06436-ABP_both
KWKKLLKKLLKLPKKLLKKLKKLLK
>ID06437-ABP_both
KWKKLLKKLLPLLKKLLK
>ID06451-ABP_both
KWKLFKKIGAVLKKL
>ID06452-NO
KWKLFKKIGIGAVLKVLKVLTTGLPALKLTLK
>ID06465-ABP_both
KWKLKPLLKKLLKKL
>ID06474-NO
KWKSFIKKLTSVLKKVVTTALPALIS
>ID06483-ABP_both
KWKSFLKTFKLAVKTVLHTALKAISS
>ID06484-ABP_both
KWKSFLKTFKSAGKTVLHTALKAISS
>ID06486-ABP_both
KWKSFLKTFKSAKKKVLHKALKAISS
>ID06487-ABP_both
KWKSFLKTFKSAKKKVLHTALKAISS
>ID06488-NO
KWKSFLKTFKSAKKTVAHTAAKAISS
>ID06489-NO
KWKSFLKTFKSAKKTVAHTALKAISS
>ID06490-ABP_both
KWKSFLKTFKSALKTVLHTALKAISS
>ID06493-ABP_both
KWKSFLKTFKSPARTVLYTALKPISS
>ID06494-ABP_both
KWKSFLKTFKSPVKTVFYTALKPISS
>ID06499-NO
KWKVFKKIEKMGRNIRNGIVKAGPAIAVLGEAKALG
>ID06506-ABP_neg
KWNSFIKKLTSKFLHSAKKF
>ID06518-ABP_both
KYYSRVRGGRSAVLSSLDK
>ID06541-ABP_both
LFGMALKLLKKVL
>ID06549-NO
LGRVDIHVWDAVYIRGR
>ID06562-NO
LIRELGQRIRRPIHRIARCAGRVVEIVR
>ID06564-NO
LIRELGQRIRRPIHRIARCAR
>ID06565-NO
LIRELGQRIRRPIHRIARCIG
>ID06566-NO
LIRELGQRIRRPIHRIARCIGQVV
>ID06567-NO
LIRELGQRIRRPIHRIARCIGQVVEIVR
>ID06580-ABP_both

LKKLLKKLLKKL
>ID06587-NO
LKKWPWWPWRRK
>ID06592-NO
LKLKLKLKLKLKLK
>ID06593-NO
LKLKLKLKLKLKLKLK
>ID06595-NO
LKLKLKLKLKLKLKLKLKLK
>ID06596-NO
LKLKLKLKLKLKLKLKLKLKLKLK
>ID06606-ABP_neg
LLAGLAANFLPTIICKISYKC
>ID06621-ABP_both
LLKKLLKLLLKLLKK
>ID06623-ABP_both
LLPWKWPWWKWRR
>ID06624-ABP_both
LLQWLSKLLGRLL
>ID06626-NO
LLRHVVKILSKYL
>ID06635-ABP_both
LLSVLGSVAKHVLPHVVPVIAEHL
>ID06639-ABP_both
LNKGAILKHIIK
>ID06654-ABP_both
LNWKAILKHIIK
>ID06683-NO
LRWWWPWRRK
>ID06692-NO
LVFPSDEFDASISQVNEKINQSLAFIRKSDELLHN
>ID06694-NO
LWELLRRGGRWILAIPRRIR
>ID06697-NO
LWETLRRGCRWILAIPRRIR
>ID06699-NO
LWETLRRGGRWILAIPRAIL
>ID06702-NO
LWETLRRGGRWILAIPRRIRQGLELCL
>ID06709-NO
LWPWWPWRRK
>ID06711-NO
LWRLLRRGGRWILAIPRRIRQGLELTL
>ID06785-NO
MASRAAGLAARLARLALRA
>ID06930-ABP_both
MKTFLRLYRSLINKVLHV
>ID07098-NO
MVSRAAGLAARLARLALR
>ID07106-NO
MWKWFHNVLSSWQLLADKRPARDYNRK

>ID07114-NO
NHRSCYRNKGVCAPARCPRNMRQIGTCHGPPVKCCR
>ID07115-ABP_both
NIGNSVSCLRNKGVCMPGKCAPKMKQIGTCGMPQVKCCKRK
>ID07117-NO
NKSLEQIWNNMTWMEWDREINNYTSLIHSLIEESQN
>ID07118-NO
NKWKKILGKIIKVVK
>ID07130-NO
NQGRHFCGAALIHARFVMTAASSFQ
>ID07135-NO
NQGRHFCGGALIHARFVMTAAKCFQ
>ID07136-NO
NQGRHFCGGALIHARFVMTAAKSFQ
>ID07137-NO
NQGRHFCGGALIHARFVMTAARCFQ
>ID07138-NO
NQGRHFCGGALIHARFVMTAARSFQ
>ID07139-NO
NQGRHFCGGALIHARFVMTAASCYQ
>ID07149-NO
NQIIEQLIKKEKVY
>ID07195-NO
PLVFPSDEFDASISQVNEKINQSLAFIRKSDELLH
>ID07208-NO
PVCTRNGLPVCGETCVGGTCNTPGCTCSW
>ID07220-NO
QCIRLEKAR
>ID07225-NO
QDKPFWPPPIYPM
>ID07226-NO
QDKPFWSPPIYPH
>ID07283-NO
QPHPDEFVGLM
>ID07285-NO
QPLPDCCRQKTCSCRLYELLHGAGNHAAGILTL
>ID07293-NO
QQDYGTGWMDF
>ID07317-ABP_both
RAGLQFPVGRVHRLLRK
>ID07399-ABP_both
RCYCRRRFCVCR
>ID07414-NO
RGDLLRHVVKILEKYL
>ID07432-NO
RHFCGAALIHARFVMTAASS
>ID07436-NO
RHFCGGALIHARFVMTAAHC
>ID07441-NO
RHFCGGALIHARFVMTAARS
>ID07442-NO

RHFCGGALIHARFVMTAASC
>ID07444-NO
RHFCGGALIHARYVMTAASS
>ID07450-NO
RHFSGGALIHARFLMTAASC
>ID07454-NO
RHFSGGALIHARFVMTAASC
>ID07455-NO
RHFSGGALIHARFVMTAASS
>ID07457-NO
RHHYESPHPRISSEV
>ID07459-NO
RHYCGGALIHARFVMTAASS
>ID07462-NO
RIAGYGLRGLAVIIRIICRGLNLIFEIIR
>ID07476-NO
RIRRPIHRIARCIGQVVEIVR
>ID07479-NO
RIRRPIRRIIRCIGQVVEIVR
>ID07485-ABP_both
RLARIVVIRVRR
>ID07486-ABP_both
RLARIVVIRWAR
>ID07499-NO
RLLTWLFSNCRTLLSRVYQILQPIL
>ID07510-NO
RLVRRIRQLTASRQLIPQLIQYV
>ID07515-NO
RLWVWWVWRRK
>ID07526-ABP_both
RPAFRKAAFRVMRACV
>ID07529-NO
RPGTLCTVAGWGRVSMRRGT
>ID07534-NO
RPRLSHKGPMPF
>ID07543-NO
RRENTQQHITARRAIRHPQY
>ID07552-NO
RRGLLEVIRTVILLLRRLRHY
>ID07574-NO
RRKKAAVALLP
>ID07576-NO
RRKKAAVALLPAV
>ID07577-NO
RRKKAAVALLPAVL
>ID07579-NO
RRKKAAVALLPAVLLA
>ID07596-NO
RRKKRRQRRR
>ID07601-ABP_both
RRLCRIVWVIRVCRR

>ID07633-NO
RRWVRRVRRVWRRVVRVVRRWVRR
>ID07642-NO
RSVCRQIKICRRRGGCYYLCTNRPY
>ID07650-NO
RVIEVVQGACRAIRHIPRRIRQILERIL
>ID07651-NO
RVIEVVQGACRAIRHIPRRSRQGLERIL
>ID07653-NO
RVIEVVQGACRAIRRIPRRIRQGLERIL
>ID07655-NO
RVIEVVQGICRAIRHIPRRIR
>ID07657-NO
RVIEVVRGACRAIRHIPRRIR
>ID07665-NO
RVVRVVRRWVRRVRRVWRRVVRVVRRWVRRVRRVWRRVVRVVRRWRVV
>ID07666-NO
RVVRVVRRWVRRVRRVWRRVVRVVRRWVRRVRRVWRRVVRVVRRWVRR
>ID07686-NO
SDEFDASISQVNEKINQSLAFIRKSDELLHNVNAG
>ID07702-ABP_both
SKCYQWQRRMRKLGAPSITCIRRTS
>ID07706-NO
SKMIEGVFAKGFKGASHLFKGIG
>ID07736-NO
SSLLEKGLDGAKKAVGGLGKLGKDA
>ID07761-NO
TASFLLKLAGRW
>ID07772-NO
TAYFLLKLAGRL
>ID07802-NO
TTWEAWDRAIAEYAARIEALIRAAQEQQEKNEAILREL
>ID07803-NO
TTWEAWDRAIAEYAARIEALIRALQELQEKLEAILREL
>ID07805-NO
TTWEAWDRAIAEYAARIEALIRALQELQEKNEAILREL
>ID07815-NO
TWMEWDREINNYTSLIHSLIEESQNQQEKNEQELLE
>ID07820-NO
VAVSKVLHLEGEVNKIALLSTNKAVVSLSNGVS
>ID07848-ABP_both
VKKFPWWWPFLKK
>ID07856-ABP_both
VKLNVYPLKVKLYP
>ID07866-ABP_both
VLPKVMAHMK
>ID07871-NO
VNFKKLLGKLLKVVK
>ID07875-ABP_both
VNSKKISPKSIKVSK
>ID07876-NO

VNWKKIILGKIIKVVK
>ID07908-NO
VRRVWRRVVRVVRRWVRRVRRVWRRVVRVVRRWVRR
>ID07918-ABP_neg
VTLASHLPSDFTPAVHASLDKFLANVSTVL
>ID07919-ABP_both
VTLASHLPSDFTPAVHASLDKFLANVSTVLTSKYR
>ID07929-NO
VVQRACRAIRHIPRRIR
>ID07934-ABP_both
VVYTLKRNGRTLYGF
>ID07951-ABP_both
WGIHRILKYGKPS
>ID07953-ABP_both
WGIRRILKYGKRS
>ID07971-NO
WMEWDREINNYTSLIGSLIEESQNQQEKNEQELLE
>ID07978-NO
WNSLKIANLAV
>ID07983-NO
WNSLKIDNLDV
>ID07988-NO
WRIWKPKWRLPKW
>ID07993-NO
WRWWKVWRWVKW
>ID08000-NO
WWRWPWWPWRRK
>ID08059-NO
YHRLRDLLLIVTRIVCLL
>ID08060-NO
YHRLRDLLLIVTRIVCLLGRR
>ID08069-NO
YHWYGYTPQNVIGGGKLLLKLLKKLLKLLKKK
>ID08084-ABP_both
YPVKLKYPKVKL
>ID08099-NO
YQLLIRMIYKNA
>ID08126-NO
YTSLIHSLIEESQNQQEKLEQELLELDKWASLWNWF
>ID08127-NO
YTSLIHSLIEESQNQQEKNEQELLEFDKWASLWNWF
>ID08128-NO
YTSLIHSLIEESQNQQEKNEQELLELDKPASLWNWF
>ID08129-NO
YTSLIHSLIEESQNQQEKNEQELLELDKWASLANAA
>ID08130-NO
YTSLIHSLIEESQNQQEKNEQELLELDKWASLANWF
>ID08131-NO
YTSLIHSLIEESQNQQEKNEQELLELDKWASLFNFF
>ID08132-NO
YTSLIHSLIEESQNQQEKNEQELLELDKWASLWNAF

>ID08133-NO
YTSLIHSLIEESQNQQEKNEQELLELDKWASLWNSF
>ID08134-NO
YTSLIHSLIEESQNQQEKNEQELLELDKWASPWNWF
>ID08154-NO
AAANPGLLETSEGCRQIL
>ID08165-NO
AAGPMLAPRERKAGCKNFFWKTFTSC
>ID08191-NO
ACGSCRKKCKGSGKCINGRCKCY
>ID08195-NO
ACSGRGSRCPPQCCMGLRCGRGNPQKCIGAHEDV
>ID08200-NO
ACYCRIPACIAGERRAGTCIYQGRLWAACC
>ID08229-NO
ADGTQRSLLGRMKGA
>ID08231-NO
ADKEVVAATYVKKK
>ID08240-NO
ADLEVVAATYVDVD
>ID08244-NO
ADLEVVAATYVLVA
>ID08250-NO
ADRSLLGRMKGA
>ID08257-NO
AEDWVRWI
>ID08293-NO
AGVEAIIRILQQLLF
>ID08304-NO
AIILVPAFF
>ID08305-NO
AIKWEYVLLLFLL
>ID08321-NO
AKDLEVVCSTYVLVEA
>ID08341-NO
AKLEVVAATYKKKK
>ID08362-NO
ALDKAEESNSKADKVNVKAT
>ID08365-NO
ALDKLEESNSKLDKVNVKLT
>ID08366-NO
ALDPIDISIELNKAKSDLEESKEWIRRSNQKLDS
>ID08367-NO
ALGAAATLEEMMTACQGV
>ID08374-NO
ALLGRMKG
>ID08380-NO
ALNSVAYERSAMQNYE
>ID08392-ABP_both
ALWKEVLKNAGKAALNEINNLV
>ID08427-NO

APLEPMYPGDYATPEQMAQYETQLRRYINTLTRPRY
>ID08436-NO
APPGFTPFRS
>ID08456-NO
AREQSNATQLDGPARELLLRLVQLAGTQESVDSAKPRVY
>ID08459-NO
ARLDVAAEFRKKWNKWALSR
>ID08480-NO
ASLRVRIKKQ
>ID08491-NO
ATAEETRRMAHRAFDTLA
>ID08492-NO
ATAEETRRMLARAFDTLA
>ID08493-NO
ATAEETRRMLHAAFDTLA
>ID08501-NO
ATCDLASGFGVGSSLCAAHCIARRYRGGYCNSQQVCVCRN
>ID08505-NO
ATCDLFSFQSQWVTPNHAACAAHCLLRGNRGGECKGTICHCRK
>ID08511-NO
ATCDLLSGFGVGDSACAAHCIARRNRGGYCNAKTVCVC
>ID08512-NO
ATCDLLSGFGVNDSACAAHCIARGNRGGYCNSKKVCVCRN
>ID08519-NO
ATCYCRTGRCATHESLSGVCEISGRLYRLCCR
>ID08525-NO
ATGQETAYFLLKLALKA
>ID08541-ABP_pos
ATYYGNGLYCNKEKCWVDWNQAKGEIGKIIVNGWVNHGPWAPR
>ID08547-NO
AVGIGALFLGFLGAAGSTMGARS
>ID08551-ABP_both
AVIPPLRCKAAFC
>ID08589-NO
CCFLNITNSHVSILQERPPLENRVLTGWGL
>ID08595-NO
CDGGIKKEIEAIKKEQEAIKKKIEAIEKLLQLTVWGIKQLQARIL
>ID08603-NO
CDVIALLACHLNTPSF
>ID08604-NO
CDVIALLACHLNTPSFNTTHYRESWY
>ID08621-ABP_both
CFAWKRNMRKVR
>ID08623-ABP_both
CFAWQRNMRKVR
>ID08627-NO
CFLNITNSHVSILQERPPLENRV
>ID08636-ABP_both
CFQWQRAMRKVR
>ID08640-ABP_both
CFQWQRNMRKAR

>ID08673-NO
CGNLSTCMLGTYTQDLNKFHTFPQTSIGVGAP
>ID08827-NO
CPTPAIEPPTGTFGFFPG
>ID08876-NO
CSCNSWLDKECVYFCHLDIIW
>ID08894-NO
CSNLSTCVLSAYWKDLNNYHRFSGMGFGPETP
>ID09006-NO
DDGPYRVEHFRWSNPPKD
>ID09017-NO
DDSVVCAAMSYSYA
>ID09021-NO
DDVAARLRAAGFGAVGAGATAEETRRMLHRAFDTLA
>ID09029-NO
DEMEECASHLPYK
>ID09063-NO
DHYNCVSSGGQCEYSACPIFTKIQGTCYRGKAKCCK
>ID09064-NO
DHYNCVSSGGQCLYSACPIFTEIQGTCYRGKAKCCK
>ID09075-NO
DISIELNKAKSDLEESKEWIRRSNQKLDSIGNWH
>ID09076-NO
DITLNNSVALDPIDISIELNKAKSDLEESKEWIRR
>ID09086-NO
DLEVVAATYV
>ID09089-NO
DLGPPISLERLDVGTNLGNAIAKLEDAKELLESSD
>ID09110-NO
DPCYEVCLQQHGNVKECEEACKHPVEY
>ID09111-NO
DPIDISIELNKAKSDLEESKEWIRRSNQKLDSIG
>ID09131-NO
DSLSFSYNNFEEDD
>ID09137-NO
DTRACDVIALL
>ID09156-NO
DVSASTTVLPDDVTAYPVGKFFQYDIWKQSTQRL
>ID09160-NO
DVSTSQAVLPDDFPRYPVGKFFQYDTWRQSAGRL
>ID09170-NO
EAIRDTNKAVQSVQSSIGNLIVAIKSVQDYVNKEI
>ID09172-ABP_neg
EAKPEAKPGNNRPVYIPQPRPPHPRL
>ID09181-ABP_both
EATKCFQWQRNMRKVRGPPVSCIKR
>ID09191-NO
ECRYLFGGCKTTSDCCKHLGCKFRDKYCAWDFTFS
>ID09213-NO
EELRVRLASHLRKLRKRLLRDADDL
>ID09214-NO

EELRVRLASHLRKLRKRLLRDADDLQKRLAVY
>ID09233-ABP_both
EKCLRWQWRMRKVGG
>ID09238-NO
EKLKEAIRDTNKAVQSVQSSIGNLIVAIKSVQDYV
>ID09239-NO
ELAGTIIDGASLTFEVLDKVLGELGKVSRK
>ID09253-NO
ELNKAKSDLEESKEWIRRSNQKLDSIGNWHQSST
>ID09316-NO
EWGRRCCGWGPGRRYCRRWC
>ID09355-NO
FAKKLAKKLKKLAKLALAL
>ID09360-NO
FAKKLAKLAKKLAKLALAL
>ID09367-NO
FAKLAKKALAKLL
>ID09371-NO
FAKLFAKLAKKFAL
>ID09380-NO
FAKLLAKLAK
>ID09383-NO
FAKLLAKLAKAKL
>ID09390-NO
FAKLLAKLAKKIL
>ID09391-NO
FAKLLAKLAKKL
>ID09407-NO
FALALKALKK
>ID09411-NO
FALALKALKKLAKKLKKLAKKAL
>ID09415-NO
FALALKLAKKL
>ID09420-NO
FALLKALLKKAL
>ID09431-NO
FCYCRRRFCVCVGR
>ID09444-ABP_both
FEVLPNFKHIQVKVFNHGEHIHHH
>ID09490-ABP_both
FKARRWAWRMK
>ID09501-ABP_both
FKCRRWQWRMAKLGA
>ID09503-ABP_both
FKCRRWQWRMKALGA
>ID09504-ABP_both
FKCRRWQWRMKKAGA
>ID09505-ABP_both
FKCRRWQWRMKKLAA
>ID09506-NO
FKCRRWQWRMKKLGAPSITCVR

>ID09532-NO
FLAGLIGGLAKMLGK
>ID09536-ABP_both
FLFKLIPKAIKGLIKAFK
>ID09545-ABP_both
FLKGIIDTVSKLF
>ID09552-NO
FLPIIGKLLSGIL
>ID09575-NO
FLSLIPHIVSGVASIAKHFG
>ID09578-ABP_both
FLSLIPKIATGIAALAKHL
>ID09580-ABP_both
FLYIVAKLLSGLL
>ID09584-NO
FNLSDHSESIQKKFQLMKEHVNKIG
>ID09589-NO
FNVALDQVFESIENSQALVDQSNRILSSAEKGNTG
>ID09607-ABP_both
FQNRRMKWKK
>ID09638-NO
FVPIFTYGEVRRMQEKERYKGQ
>ID09661-ABP_pos
GADRGWIKTLTKDCPNVISSICAGTIITACKNCA
>ID09669-NO
GALFLGFLGAAGSTMGAWSQPKSKRKV
>ID09678-NO
GCASRCKAKCAGRRCKGWASAFRGRCYCKCFRC
>ID09680-NO
GCCGPYPNAACHPCGCKVGRPPYCDRPSGG
>ID09684-NO
GCCSDPRCAWRC
>ID09689-NO
GCCSHPACNVNNPHICG
>ID09690-NO
GCCSHPACSVNHPELC
>ID09696-NO
GCCSYPPCFATNPDC
>ID09725-NO
GELDELVYLLDGPGYDPIHCDVVTRGGSRLFNF
>ID09733-NO
GERVWDRGNVTLLCDCPNGP
>ID09739-ABP_neg
GFCWYVCVYRNGVRVCYRRCN
>ID09747-ABP_neg
GFGCPNDYPCHRHCKSIPGRCGGYCGGWHRLRCTCYR
>ID09758-NO
GFMDTAKNVAKNVAVTLLDKLKCKISGGC
>ID09795-NO
GIFALIKTAAKFVGKNLLRQAGKAGLEHLACKANNQC
>ID09810-NO

GIGGVLLSAGKAALKGLAKVLAEKYAD
>ID09811-NO
GIGGVLLSAGKAALKGLARVLAEKYAN
>ID09815-NO
GIGKFLHSAKKFGKAFVGEINS
>ID09816-NO
GIGKFLHSAKKWGKAFVGQIMNC
>ID09819-NO
GIGKWLHSAKKFGKAFVGEIMNS
>ID09834-ABP_pos
GIMDTLKNLAKTAGKGALQSLLNHASCKLSKQC
>ID09840-ABP_both
GIPCAESCVWIPCTITALMGCSCKNNVCYNN
>ID09845-NO
GIPCGESCVFIPCLTSAIGCSCKSKVCYRN
>ID09860-NO
GKLEVLHSTKKFAKGFITGLTGQ
>ID09871-ABP_both
GKPTCGETCFKGKCYTPGCTCSYPLCKKD
>ID09879-NO
GLASTLTRWAHYNALIRAF
>ID09886-NO
GLFDVIKAVASVIGGL
>ID09887-ABP_both
GLFDVIKKVASVIGG
>ID09888-NO
GLFDVIKKVASVIKGL
>ID09897-ABP_both
GLFSVLGSVAKHVVPRVVPVIAEHL
>ID09898-NO
GLFSVVKGVLKGVGKNVAGSLLDQLKCKISGGC
>ID09907-NO
GLLCYCRKGHCKRGDRVRGTCGIRFLYCCPRR
>ID09932-ABP_both
GLLSVLGDVAKHVLPHVVPVIAEKL
>ID09939-NO
GLPICGETCVGGTCNTPGCFCTWPVCTRN
>ID09942-NO
GLPTCGETCTLGKCNTPKCTCNWPICYKN
>ID09957-NO
GLWSKIKEAGKAALTAAGKAALGAVSDAV
>ID09990-NO
GQKKIRVRLS
>ID10001-NO
GRILSFIKAGLAEHL
>ID10013-ABP_neg
GRRRSVQWCAVSQPEATKCFQWQRNMRKVRGPPVSCIKRDSPIQCIQA
>ID10032-ABP_both
GSKKPVPIIYCNRRTGKCQ
>ID10033-ABP_both
GSKKPVPIIYCNRRTGKCQR

>ID10050-NO
GSSFLSPEHQRVQQRKESKKPPAKLQP
>ID10051-NO
GSVAFPAENGVQNTESTQE
>ID10054-NO
GSWLRDIWDWICEVLSDFK
>ID10058-NO
GTEVSEALGGAGLTGGFY
>ID10066-NO
GTKALTEVIALTEEAEC
>ID10067-NO
GTKALTEVIPATEEAEC
>ID10068-NO
GTKALTEVIPLAEEAEC
>ID10089-NO
GVCCGVSFCYPC
>ID10116-ABP_both
GWLKKLGKKIERVGQHTRDATIQTIGVAQQAVNVAATLKG
>ID10128-NO
GWWYKGRARPVSAVA
>ID10143-NO
HADGSFSDEMSTILDNLATRDFINWLIQTKITD
>ID10149-NO
HADGVFTSDYSRLLGQISAKKYLESLIGKRISSSISEDPVPV
>ID10191-NO
HFLLTLVNLAKKIL
>ID10221-NO
HPPCCMYGRCRRYPGCSSASCCQG
>ID10234-NO
HSDGTFTSELSRLREGARLQRLLQGLV
>ID10239-NO
HSQGTFTSDYSKYLDSRRAQDFVQWLMNTKRNKNNIA
>ID10258-NO
ICYCRRRFCVCVGR
>ID10276-NO
IEPHDWTKNITDKIDQIIHDFVDKT
>ID10306-NO
IISTIGDLVKWIIDTVNKFTKK
>ID10328-NO
ILDKLKEFGISAARGVAQSLLNTASCKLAKTC
>ID10335-NO
ILGKLLSTAWALLSKL
>ID10346-NO
ILGWKWPWWPWRR
>ID10347-NO
ILHWKWPWWPWRR
>ID10356-NO
ILMCFSIDSPD
>ID10358-NO
ILMCFSIDSPDSL
>ID10365-NO

ILPGKWPWWPWRR
>ID10366-NO
ILPHKWPWWPWRR
>ID10373-NO
ILPWKKPWWPWRR
>ID10402-NO
INASVVNIQKEIDRLNEVAKNLNESLIDL
>ID10404-NO
INASVVNIQKEIDRLNEVAKNLNESLIDLQELGKYEQYI
>ID10405-NO
INASVVNIQKEIDRLNEVAKNLNESLIDLQELGKYEQYIKWPW
>ID10414-NO
INQKKIASIGKEV
>ID10418-NO
INWLKLGKAVIDAL
>ID10430-NO
IQESLTTTSTALGKLQDVVNQNAQALNTLVKQLSS
>ID10432-NO
IQKEIDRLNEVAKNLNESLIDLQELGK
>ID10433-NO
IQKEIDRLNEVAKNLNESLIDLQELGKYEQYIKWPW
>ID10439-NO
IRKAHCNISRAKWND
>ID10454-NO
ISLERLDVGTNLGNAIAKLEDAKELLESSDQILRS
>ID10455-NO
ISLERLDVGTNLGNAIAKLEDAKELLESSDQILRSM
>ID10466-NO
ITIELSNIKENKCNGTDAKVKLIKQELDKYKNAVT
>ID10494-NO
KAAKKAWKAAKKAAKWWKKAA
>ID10498-NO
KAAKKWAKAAKKAAKAWKKAA
>ID10500-NO
KAAKKWAKAWKKAAKAWKKAA
>ID10505-ABP_both
KALAALLKKWAKLLAALK
>ID10511-NO
KAPSGRMSVLKNLQSLDPSHRISDRDYMGWMDF
>ID10526-ABP_both
KCRRWQWRMKKLGA
>ID10585-NO
KGSVVIVGRIILSGRK
>ID10589-NO
KHMHWHPPALNT
>ID10590-NO
KIAEKFSGTRRG
>ID10596-NO
KILRGVAKKILRTFLRRISKDILTGKK
>ID10597-NO
KILRGVAKKIMRTFLRRILTGKK

>ID10624-NO
KKKFPWWWPFKKKKKKFPWWWPFKKKK
>ID10646-NO
KKKKLLLAFLFLF
>ID10653-NO
KKKKLLLPFLVFF
>ID10663-NO
KKKKLLLPTLVFF
>ID10676-NO
KKKKLVLPFLLFF
>ID10689-NO
KKKKVLAATYVFV
>ID10698-NO
KKKKVLLPFLLFF
>ID10725-NO
KKKKVVAATYFFFA
>ID10728-NO
KKKKVVAATYFLVA
>ID10729-NO
KKKKVVAATYGLV
>ID10733-NO
KKKKVVAATYKLKA
>ID10741-NO
KKKKVVAATYVFVA
>ID10742-NO
KKKKVVAATYVGV
>ID10746-NO
KKKKVVAATYVLE
>ID10747-NO
KKKKVVAATYVLF
>ID10748-NO
KKKKVVAATYVLFA
>ID10749-NO
KKKKVVAATYVLG
>ID10791-ABP_neg
KKLAKALKLPALLWLKLAKALKKA
>ID10793-ABP_both
KKLAKLALLKWLLALKKLALLALKK
>ID10797-ABP_neg
KKLALALKKPALLWKKLALALKKA
>ID10800-ABP_both
KKLALKALKLWLLALLKLAKLALKK
>ID10818-ABP_neg
KKWKIVVIRWRR
>ID10876-ABP_both
KPKGMTSSQWFKIQHMQPSPQASNSAMKNINKHTKRSKDLNTFLH
>ID10887-NO
KPPSKPNNDFHFEVFNF
>ID10889-NO
KPPSKPNNDFHFEVFNFV
>ID10917-ABP_pos

KRFKKFFKKLKNSVKKRFKKFFKKLKVIGVTFPF
>ID10946-ABP_both
KRRKLIKILKLIIKLIRKKR
>ID10994-ABP_both
KWCFCVCYRGICRCRCRG
>ID11031-ABP_neg
KWKSFIKKLTSKKLHSAKKF
>ID11032-ABP_neg
KWKSFIKKRTSKFLHSAKKF
>ID11037-NO
KWLTEWIPLTAEAEC
>ID11041-ABP_both
KWPWWPWRR
>ID11081-NO
LCYCRRRFCWCV
>ID11091-NO
LDIVKKVVGAFGSL
>ID11092-NO
LDKYFKNHTSPDVDLGDISGINASVVNIQKEIDRLNEVAK
>ID11093-NO
LDLAKHVIGIASKL
>ID11095-NO
LDPIDISIELNKAKSDLEESKEWIRRSNQKLDSI
>ID11104-NO
LEAIPCSIPPCVAFNKPFVF
>ID11113-NO
LEAIPMSIPPAVKFNKPFVF
>ID11114-NO
LEAIPMSIPPEAKFNKPFVF
>ID11118-NO
LEAIPMSIPPEVFFNKPFVF
>ID11136-NO
LFYLVPGPGH
>ID11157-NO
LGVATSAQITAAVALVEAKQARSDIEKLKEAIRDT
>ID11163-NO
LIEESQNQQEKNEQELLELDKWASLWNWFNITNWLW
>ID11182-NO
LKLGTLVNLAKKIL
>ID11188-NO
LKLKSIVSWAKLVL
>ID11189-NO
LKLKSIVSWALKVL
>ID11191-NO
LKLKSIVSYAKKVL
>ID11205-NO
LLDCWVRLGRYLLRRLKT
>ID11217-ABP_both
LLGDFFRKAKEKIGKEFKRIVQR
>ID11219-ABP_neg
LLGDFFRKSKEKI

>ID11231-ABP_both
LLKKLLKKC
>ID11233-NO
LLLGTEVSEALGGAGLTG
>ID11234-NO
LLLGTEVSEALGGAGLTGG
>ID11253-NO
LNNSVALDPIDISIELNKAKSDLEESKEWIRRSN
>ID11266-NO
LPNYNWNSFGLRF
>ID11271-NO
LPRLLRLDSRMATVDFPKKDPTTSLGRPFFLFRPRN
>ID11275-NO
LPRRLHLEPAFLPYSVKAHECC
>ID11280-NO
LQKWEDWVRWIGNIPQYLKG
>ID11281-ABP_both
LQKYYCRVRGGRCAVLSCLPKEEQIGKCSTRGRKCCRRKK
>ID11285-NO
LQQLLFIHFRIGCQH
>ID11291-NO
LRDIWDWICEVLSDFK
>ID11294-ABP_both
LRFIKKILKKLI
>ID11296-ABP_both
LRFLKKILKHLF
>ID11324-NO
LRRLLRRLLRRL
>ID11340-NO
LSTALKVAANVVPTLFCKITKKC
>ID11341-NO
LSTLLNVASKVVPTLFCKITKKC
>ID11421-NO
MANAGLQLLGFILAFLGW
>ID11424-NO
MANAGLQLLGFILAFLGWIGAIVS
>ID11445-NO
MDELYPMEPEEEANGSEILA
>ID11458-NO
MDVNPTLLF
>ID11462-NO
MDVNPTLLFLKV
>ID11463-NO
MDVNPTLLFLKVP
>ID11465-NO
MDVNPTLLFLKVPAQ
>ID11467-NO
MDVNPTLLFLKVPAQNAI
>ID11727-NO
MHRSLLGRMKGA
>ID11825-NO

MLCVLQGLREC
>ID11857-NO
MNINPTLLFLKVPIQ
>ID11918-NO
MRKWFHNVLSSGQLLADKWPAWDYNRK
>ID11956-NO
MSTNPKPQRKTKRNTNRRPQ
>ID11976-NO
MVDRGWGNHAGLFGKGSIV
>ID11979-NO
MVQGSGCFGRKMDRISSSSGLGCKVL
>ID11982-NO
MVQGSGCFGRKMDRISSSSGLGCKVLRRH
>ID12011-NO
NEVAKNLNESLIDLQELGKYEQYIKWPWYVW
>ID12020-NO
NGVCCGYKLCHPC
>ID12027-ABP_both
NIFEIFRNRNRGGLIKDAATPWLPCEILRPC
>ID12029-NO
NIKENKCNGTDAKVKLIKQELDKYKNAVTELQLLM
>ID12031-ABP_both
NILSSIANGINRALSFFG
>ID12042-ABP_neg
NLKALAALAKKIL
>ID12053-NO
NNCLLLGTEVSEALGGAGLT
>ID12074-NO
NQQEKNEQELLELDKWASLWNWFNITNWLWLIKIFI
>ID12076-NO
NQTKDLQQKFYEIIMDIEQNNVQGKKGIQQLQKWE
>ID12081-NO
NRLRRREDFARAGFVVSKAVGGAVVRNQVKRRLRHLPLVVVLARDL
>ID12091-NO
NRVYVHPF
>ID12095-NO
NSVALDPIDISIELNKAKSDLEESKEWIRRSNQK
>ID12097-NO
NVALDQVFESIENSQALVDQSNRILSSA
>ID12101-ABP_both
NVWKKVLGKIIKVAK
>ID12119-NO
PAICQRATATLGTVGSNTSGTTEIEAAILL
>ID12132-NO
PAWRKAFRAAWRMLKKAA
>ID12133-NO
PAWRKAFRKAWRMLKKAA
>ID12138-NO
PAWRKARRWAWRMKKLAA
>ID12173-NO
PKMVQGSGCFGRKMDRISSSSGLGCKVLRR

>ID12174-NO
PKRKSATKGEPA
>ID12183-NO
PLNNCLLLGTEVSEALGG
>ID12208-NO
PPGFTPFRIY
>ID12251-NO
PRFWEYWLRAME
>ID12274-NO
PTLLFLKVPAQNAISTTFPYT
>ID12293-NO
PYVALFEKCCLIGCTKRSLAKYC
>ID12299-NO
QAVGWGDPITHWSHGQNQWP
>ID12304-NO
QDGSSEAAGFLPADSEKASGPLGTLAEELSSYSRRKGGFSFRF
>ID12307-NO
QEDAELQPRALDIYSAVDDASHEKELIEALQEVLKKLKS
>ID12316-NO
QFWSLAAPQRF
>ID12331-NO
QHGAAPECFWKYCI
>ID12383-NO
QPELDSFKEELDKYFKNHTSPDVDLGDISGINASVVNIQ
>ID12393-NO
QQDYTGAHFDF
>ID12394-NO
QQDYTGSHFDF
>ID12407-NO
QRLRIRVAVIRA
>ID12451-NO
RDCCTPPRKCKDRRCKPMKCCA
>ID12456-NO
RDWHLGQGVSIEWRK
>ID12464-NO
RFGRFLRKIR
>ID12467-NO
RFGRFLRKIRRFR
>ID12480-NO
RGGRLCYCRRRFCVCF
>ID12489-ABP_both
RGKRWWRRKK
>ID12497-NO
RGVSGHGQHGVHG
>ID12531-ABP_neg
RIVWVRIRRWQV
>ID12536-ABP_both
RKCLRWQWAMRKVGG
>ID12539-ABP_both
RKCLRWQWEMRKYGG
>ID12560-ABP_both

RKKRLKLLKRLL
>ID12604-ABP_both
RLRRIVVIRVFR
>ID12642-NO
RPPGFSPFRVD
>ID12643-NO
RPPGFTPFRIA
>ID12654-ABP_both
RQWQSKIRRT
>ID12663-ABP_both
RRGLFKKLRRKIKKGFKKIFKRLPPVGVGVSIPLAGRR
>ID12686-NO
RRKKAAVALKPAVLLALLAP
>ID12688-NO
RRKKAAVALLKAVLLALAAP
>ID12690-NO
RRKKAAVALLPAVALALLAP
>ID12691-NO
RRKKAAVALLPAVELALLAP
>ID12692-NO
RRKKAAVALLPAVKLALLAP
>ID12733-NO
RRRRRRRRGEDIIRNIARHLAQVGDSMDR
>ID12737-ABP_both
RRRRRRRRRRRR
>ID12820-NO
RTCESQSHKFKGPCASDHNCASVCQTERFSGGHCRGFRRRCFCTTHC
>ID12832-NO
RVIRVWFQNKRCKDKK
>ID12900-NO
SANSNPAMAPRERKAGCKNFFWKTFTSC
>ID12901-NO
SAQITAAVALVEAKQARSDIEKLKEAIRDTNKAVQ
>ID12910-NO
SCNTATCVTHRLAGLLRRSGGVVKDNFVPTNVGSKAF
>ID12911-NO
SCNTATCVTHRLAGLLSRSGGVVKDNFVPTNVGSEAF
>ID12929-NO
SDLGAVISLLWGRQLFA
>ID12962-NO
SGPGPWQGGRRKFRRQRPRLSHKGPMPF
>ID12969-NO
SHRFQGTCLSDTNCANVCHSERFSGGKCRGFRRRCFCTTHC
>ID12975-ABP_both
SILSTLKDVGISALKNAGSGVLKTLLCKLNKNCEK
>ID12982-ABP_both
SKCYQWQRRMRKLGA
>ID13022-ABP_both
SPAIWGCDSFLGYCRLACFAHEASVGQKECAEGMLCCIPNV
>ID13028-NO
SPPEAAYGPGNTNSDSGDK

>ID13038-NO
SQEPPISLDLTFHLLREVLEMTKADQLAQQAHNNRKLLDIA
>ID13057-NO
SSCFGGRIDRIGAQSGLGCNSFR
>ID13067-NO
SSPETLISDLLMRESTENAPRTRLEDPSMW
>ID13086-ABP_neg
STLHLVLRLRAG
>ID13096-ABP_neg
SVDGKEDLIWKLLSKAQEKFGKNKSR
>ID13098-ABP_both
SVIGCWTKSIPPRPCFVK
>ID13101-NO
SVPHFSEEEKEAE
>ID13105-NO
SVSNAATRVCRTGRSRWRDVCRNFMR
>ID13113-NO
SWETWEREIENYTRQIYRILEESQEQQDRNERDLLE
>ID13121-NO
SWLRDIWDWICEV
>ID13129-NO
SWLRDIWRWICKVLSRFK
>ID13149-NO
SYSMEHFRWGKPVGRKRRPIKVYTNGVEEESTETLPAEM
>ID13152-NO
TAALELDIKLTQHYFGLLTAFGSNFGTIG
>ID13173-NO
TDVILMCASIDSPDSLENI
>ID13174-NO
TDVILMCFAIDSPDSLENI
>ID13175-NO
TDVILMCFSADSPDSLENI
>ID13177-NO
TDVILMCFSIASPDSLENI
>ID13213-NO
TISCTNEKQCYPHCKKETGYPNAKCMNRKCKCFGR
>ID13219-ABP_both
TKCFQWQRNMRKVR
>ID13220-ABP_both
TKCFQWQRNMRKVRGPPVSCIKR
>ID13233-NO
TLGEWYNQTKDLQQKFYEIIMDIEQNNVQGKKG
>ID13234-ABP_both
TLISWIKNKRKQCRPRVSRRRRRRGGRRRR
>ID13272-NO
TPNITLNNSVALDPIDISIELNKAKSDLEESKEW
>ID13282-NO
TRQARRNRRRRWRERQR
>ID13345-NO
VAKKFAKKFKKFAKKFAKFAFAF
>ID13351-NO

VALALKALKKALKKLKKALKKAL
>ID13362-NO
VALKGCWTKSIPPKPCFGKR
>ID13372-NO
VCCPFGGCHELCYCCD
>ID13373-NO
VCGETCEGGTCNTPGCSCSWPVCTRNGLP
>ID13374-NO
VCGETCVGGTCNTPGCSCSRPVCTRNGLP
>ID13384-NO
VDGIPVGWDADARAPA
>ID13388-NO
VDPDVRAYCKHQCMSTRGDQARKICESVCMRQD
>ID13409-NO
VGCVLGTCQVQNLSHRLWQLMGPAGRQDSAPVDPSSPHSY
>ID13424-NO
VGSASCTIAALGSSDRDTVV
>ID13428-NO
VILMCFSIDSPDSLENI
>ID13429-NO
VILSLDVPIGLLRILLEQARNKAARNQAATNAQILARV
>ID13432-NO
VIPFVASVAAEMMQHVYCAASRKC
>ID13449-ABP_both
VKVGINGFGRIGRLVTRAAFHGKKVEIVAIND
>ID13465-NO
VNPTLLFLKVPAQNAISTTFPYT
>ID13466-ABP_both
VNWKKILAKIIKVAK
>ID13484-NO
VPCSICSNNPTCWAICKRIPNKKPGKK
>ID13489-NO
VPPGFTPFR
>ID13501-NO
VQGSGCFGRKMDRISSSSGLGCKVL
>ID13504-ABP_neg
VQLRAIRVRVIR
>ID13512-NO
VRLASHLRKLRKRLLRDADDLQKRLAVY
>ID13513-NO
VRLGRYLLRRLKTPFTRL
>ID13536-NO
VSFAIKWEYVLLL
>ID13537-NO
VSFAIKWEYVLLLF
>ID13541-NO
VSQRTDGESRAHLGALLARYIQQARKAPSGRMSIVKNLQNLDPSHRISD
>ID13570-NO
VVKCSYQLGSPDSRCN
>ID13596-NO
VYLHRIDLGPPISLERLDVGTNLQNAIAKLEDAKE

>ID13601-NO
VYTDKVDISSQISSMNQSLQQSKDYIKEAQKILDTV
>ID13604-NO
WAGGDASGE
>ID13628-NO
WEYVLLLFLL
>ID13641-NO
WIHAEIKNSLKIDNLDVNRCIEALD
>ID13652-ABP_both
WKLFKKILKVL
>ID13680-ABP_both
WLNALLHHGLNCA
>ID13682-ABP_both
WLNALLHHGLNCAKG
>ID13691-ABP_both
WLWKAIWKLLT
>ID13700-NO
WMEWDREINNYTSLIHSLIEEPQNQQEKNEQELL
>ID13704-NO
WMEWDREINNYTSLIHSLIEESQNQQEKNEQELLELDKWASLWNWFRS
>ID13711-NO
WNPFKELERAGQRVRDAIISAAP
>ID13716-ABP_neg
WQRRMRKLGAPSIT
>ID13726-NO
WRWRWRWRW
>ID13765-NO
WYKPAAGHSSYSVGRAAGLLSGLR
>ID13767-NO
WYKPAAGPHHYSVGRASGLLSSFHRFPST
>ID13850-NO
YAEGTFISDYSIAMDKIRQQDFVNWLLAQRGKKSDWKHNITQ
>ID13873-NO
YCQKWMWTCDSERKCCEGMVCRLWCKKKLW
>ID13889-NO
YGAVVNDL
>ID13890-NO
YGGFLRKYP
>ID13893-NO
YGGFLRRIRPKLKWDNQ
>ID13897-NO
YGGFLRRQFKVVTRSQEDPSAYYEELFDV
>ID13900-NO
YGGFMKSWDERSQKPLLTLFKNVIIKDGQQ
>ID13902-NO
YGGFMRGL
>ID13907-NO
YGQVPMCDAGEQCAVRKGARIGKLCDCPRGTSCNSFLLKCL
>ID13922-NO
YKGANKKGLSKGCFGLKLDRIGSMSGLGC
>ID13933-NO

YKRCHKKGGHCFPKEKICTPPSSDFGKMDCRWKWKCCKKGSVN
>ID13975-ABP_neg
YQWQRRMRKLGAPSIT
>ID13978-ABP_both
YRAWRWAWRWR
>ID13986-NO
YRQSMNQGSRSTGCRFGTCTMQKLAHQIYQFTDKDKDGMAPRNKISPQGY
>ID14001-NO
YVMGHFRWDRF
>ID14025-ABP_both
AAAAGSCVWGAVNYTSDCNGECLLRGYKGGHCGSFANVNCWCRT
>ID14052-NO
AADGKPSDWISLVTPECCSNPPCNLQNC
>ID14100-ABP_both
AAKIILNPKFRCFAAFC
>ID14107-NO
AAKVKYSKTPEECCPNPPCFATNSDICGGRR
>ID14111-NO
AAKVKYSLTPAECCPNPPCFAQHSNLCGARR
>ID14114-NO
AAKVKYSNTPEECCPNPPCFATHSEICGV
>ID14137-NO
AAMRSFNMGFG
>ID14140-NO
AANAKLFAVMQSCCSTPPRALRHMDMC
>ID14143-NO
AANAKLFDVGQSCCSAPLCALLYMVIC
>ID14146-NO
AANAKLFDVRQSCCSTPPCALLYMEMCG
>ID14158-NO
AANDKASDLMALRDGCCSDPACSVNHPDICGGGR
>ID14161-NO
AANDKASDLMALRGGCCSRPPCILKHPEICGRRR
>ID14165-NO
AANDKASVQIALTVQECCADAACSLTNPLICGRR
>ID14169-NO
AANDKTSAWIAWAGSQSCCATPSCARLY
>ID14171-ABP_both
AANIPFKVHFRCKSIFC
>ID14172-NO
AANKKASDLMALTVRGCCVYPPCAVNHPDICG
>ID14185-NO
AANQHLCGSHLVEALYLVCGERGFFYTPNKV
>ID14194-NO
AAPYAFGL
>ID14210-NO
AASDKASELMALAVRGCCSNLACAGNNLHICGRRR
>ID14221-NO
AAVALLPAVLLALLAK
>ID14223-NO
AAVALLPAVLLALLAKKNNLKECGLY

>ID14226-NO
AAVALLPAVLLALLAPRKKRRQRRRPPQC
>ID14239-ABP_both
AAYLLAKINLKALAALAKKIL
>ID14240-ABP_both
ACADLRGKTFCRLFKSYCDKKGIRGRLMRDKCSYSCGCR
>ID14263-NO
ACDFQSCWVLCQRQYNIYFRKAYCEQSRCVCVYNYGG
>ID14266-NO
ACDFQSCWVSCQRQYNIYFRRAYCEHSKCTCVYNYGG
>ID14295-NO
ACNTATCVTHRLADFLSRSGGIGNSNFVPTNVGSKAF
>ID14334-NO
ADCVGDGQKCADWFGPYCCSGYYCSCRSMPYCRCRSDS
>ID14343-NO
ADFAFNPRL
>ID14344-NO
ADFAFSPRL
>ID14352-NO
ADNRRPIWV
>ID14370-NO
ADYLQLAR
>ID14371-NO
ADYLQLARA
>ID14403-NO
AELPQGLWVRPRLG
>ID14426-NO
AFCNLRLCQLSCRSLGLLGKCIGDKCECVKH
>ID14428-NO
AFCNLRMCQLSCRSLGLLGKCMGKKCRCVKH
>ID14429-NO
AFCNLRRCELSCRSLGLLGKCIGEECKCVPH
>ID14435-NO
AFDRFDNSGVFSFGA
>ID14448-ABP_both
AFGCPFDQGTCHSHCRSIRRRGERCSGFAKRTCTCYQK
>ID14451-NO
AFGTILKALAKIAAKVVKKLATKPGATYMLKQNLQ
>ID14463-ABP_both
AFKMALKLLKKVL
>ID14471-NO
AFPPPNVPGPRFPPPNVPGPRFPPPNFPGPRFPPPNFPGPRFPPPNFPGP
>ID14478-ABP_both
AFTHCRRSYSTEYSYGTCTV
>ID14501-NO
AGCKNFFWKTFSSC
>ID14511-NO
AGDTSSEAKGMWFGPRL
>ID14515-NO
AGESSNEAKGMWFGPRL
>ID14530-NO

AGGSGGVGGEYDDYGHLI
>ID14557-NO
AGNDKATDLMALTVRGCCSHPACAGNNPHICG
>ID14574-NO
AGQDNFMRF
>ID14583-NO
AGSDPNFLRF
>ID14592-NO
AGTRMSWEVL
>ID14631-NO
AHRNFLRF
>ID14661-ABP_both
AIHKLAHKLLKKLLRAVKKLAN
>ID14664-ABP_both
AIHKLAHKLTKKTLRAVKKLAN
>ID14713-ABP_both
AKAGGIGGHVIGQIVGVRIRGLGGNV
>ID14796-NO
AKRHHGYKRK
>ID14849-ABP_both
ALASLLKTLSKAKKKKLKTLLKALSA
>ID14862-NO
ALDTLGGFQVHGW
>ID14874-ABP_both
ALGDFFRKSKEKIGKEFKRIVQRIKDFLRNLAKRHHGYKRKFHLEY
>ID14911-ABP_both
ALHCIALRKK
>ID14920-ABP_neg
ALKLLKKLL
>ID14921-ABP_both
ALKSLLATLSKAAKKALKTLLAALSK
>ID14923-ABP_both
ALKSLLKTLSAAAKKALATLLKALSK
>ID14925-ABP_neg
ALKSLLKTLSKAAAAALKTLLKALSK
>ID14998-ABP_both
ALWKNMLKGIGKLAG
>ID15007-ABP_both
ALWKTMLKKLGTM
>ID15009-ABP_both
ALWKTMLKKLGTMAL
>ID15011-ABP_both
ALWKTMLKKLGTMALHAGKAALGAAADTI
>ID15016-ABP_both
ALWMTLLKKVLKA
>ID15030-ABP_neg
ALYKKLLKKLLKSAKKLG
>ID15040-ABP_pos
AMDPTKYYGNGVYCNSKKCWVDWGQASGCIGQTVVGGWLGGAIVGKC
>ID15055-ABP_both
AMRLTGNKPCLYGT

>ID15063-NO
AMRNALVRF
>ID15089-NO
ANKRPIWIMGHMVNAIYQIDEFVNLGANSIETDVS
>ID15139-NO
APEASPFIRFG
>ID15163-NO
APGKIPVKAIKQAGKVIGKGLRAINIAGTTHDVVSFFRPKKKKH
>ID15176-NO
APKPKFIRF
>ID15179-NO
APKQMVFGFG
>ID15183-ABP_both
APKVNVNALRKGGRVIRKGLGVIGAAGTAHEVYNHVRNRNQG
>ID15191-NO
APLEPVYPGDNATPEQMAQYAAEMRRYINMLTRPRY
>ID15194-NO
APLQPGGSPALTKIYPR
>ID15196-NO
APLSWDLPEPRSRAGKIRVHPRGNLWATGHFM
>ID15198-NO
APMEPVYPGDNATPEQMAQYAAELRRYINMLTRPRY
>ID15202-NO
APMGFYGTRG
>ID15207-NO
APNRVLMRF
>ID15209-NO
APNVKDSKASGSCCDNPSCAVNNRHC
>ID15212-NO
APNVKDSKASGSCCDNPSCAVNNSHCGRRR
>ID15223-NO
APQLRLRF
>ID15251-NO
APSAPAGLEEKL
>ID15261-NO
APSGFLGMRG
>ID15278-NO
APSSAQRLYGFGL
>ID15297-NO
APVTAGRAGALAKMYTRGNHWAVGHLM
>ID15299-NO
APWLVPSQITTCCGYNPGTMCPSCMCTNSC
>ID15307-NO
APYAFEVVG
>ID15323-NO
AQEEADAEERRLQEQEELENYIEHVLLHRP
>ID15338-NO
AQKCGEQGRGAKCPNCLCCGRYGFCGSTPDYCGVGCQSQCRGC
>ID15347-NO
AQPSMRLRF
>ID15445-ABP_both

ARNFGKFFTPVLQADFQKVVAGVANALAHRYH
>ID15463-NO
ARPVKEPRSLSAASAPLVETSTPLRL
>ID15537-NO
ASEDALFGTMRFG
>ID15575-NO
ASLKSDTVTPLR
>ID15582-NO
ASNFDCCLGYTDRILHPKFIVGFTRQLANEGCDINAIIFHTKKKLSVCAN
>ID15588-NO
ASPKCFKYNVLACT
>ID15613-NO
ASTCDFIM
>ID15628-NO
ATAWDFGPHGLRPIRPIRIRPLCGKDKS
>ID15636-ABP_pos
ATCDLASIWNVNHTLCAAHCIARRYRGGYCNSKAVCVCR
>ID15637-ABP_pos
ATCDLASIWNWNHTLCAAHCIARRYRGGYCNSKAVCVCR
>ID15638-ABP_pos
ATCDLASKFNVNHTLCAAHCIARRYRGGYCNSKAVCVCR
>ID15717-NO
ATYDGKCYKKDNICKYKAQSGKTAICKCYVKVCPRDGAKCEFDSYKGKCY
>ID15729-NO
AVCNLKRCQLSCASLGLLGKCIGDKCECVKH
>ID15734-NO
AVCVYRTCDKDCKRRGYRSGKCINNACKCYPYGK
>ID15772-NO
AVKPPWRCKAAFC
>ID15817-NO
AVPLIYNRPGVYVTKRPKGK
>ID15822-NO
AVQKVDGEPRAHLGALLARYIQQARKAPSGRMSVIKNLQNLDPSHRISD
>ID15838-NO
AVRSPSLRLRF
>ID15852-NO
AVVSGYDNIYQVLAPRF
>ID15883-NO
AWQDLNSAW
>ID15978-NO
AYSYVSEYKRLPVYNFGL
>ID15986-NO
AYTYVSEYKRLPVYNFGL
>ID16002-NO
CADPNSVRAMC
>ID16025-NO
CAPFLHLCTFFFPNCCNGYCVQFICL
>ID16084-NO
CCDDSECDYSCWPCCMF
>ID16093-NO
CCDRPCSIGCVPCCLP

>ID16096-NO
CCDWPCNAGCVPCCF
>ID16106-NO
CCFWPACWGCVCCY
>ID16108-NO
CCFWPMCGGCDCCYL
>ID16142-NO
CCGPTACLAGCKPCC
>ID16147-NO
CCGVPNAACHPCVCTGKC
>ID16164-NO
CCHPACGQNYSC
>ID16179-NO
CCISPACHEECYCCQ
>ID16183-NO
CCIWPECGSCVCCL
>ID16189-NO
CCKVQCESCTSCC
>ID16194-NO
CCLWPECGGCVCCY
>ID16196-NO
CCMALCSRYHCLPCC
>ID16265-NO
CCRTCFGCTPCC
>ID16282-NO
CCSQDCRVCIPCCPY
>ID16284-NO
CCSQDCSVCIPCCPN
>ID16287-NO
CCSQDCWECIPCCPN
>ID16295-NO
CCSRHCWVCIPCCPNGSA
>ID16301-NO
CCSVSICQPPPVCECCA
>ID16303-NO
CCSWDVCDHPSCTCC
>ID16306-NO
CCSYPPCNVSYPEICG
>ID16312-NO
CCVVCNAGCSGNCCP
>ID16358-NO
CFESWVACESPKRCCSHVCLFVCT
>ID16486-NO
CGRKKRRQRRRPPQ
>ID16508-NO
CIAESEPCNIITQNCCDGKCLFFCIQIPE
>ID16520-NO
CIEQFDPCDMIRHTCCVGVCFLMACI
>ID16581-NO
CKGKGAKCSLLMYDCCTGSCRSGKC
>ID16583-NO

CKGKGAKCSRAMYDCCTGSCRSGKC
>ID16584-NO
CKGKGAKCSRLAYDCCTGSCRSGKC
>ID16598-NO
CKGKGAPCTRLMYDCCHGSCSSSKGRCG
>ID16607-NO
CKGKGASCSRTMYNCCSGSCNRGKCG
>ID16618-NO
CKIALPYNMRCRVLGKC
>ID16632-ABP_both
CKKLLKLLKKLCKLAG
>ID16659-NO
CKSKGAKCSKLMYDCCSGSCSGTVGRCY
>ID16660-NO
CKSKGAKCSKLMYDCCTGSCSGTVGRC
>ID16661-NO
CKSKGAKCSRLLYDCCSGSCSGTVGRC
>ID16668-NO
CKSPGTPCSRGMRDCCTSCLSYSNKCRRY
>ID16681-ABP_both
CKWKWKWKWKC
>ID16688-NO
CKYKGAKCSRLMYDCCSGSCSGTVGRC
>ID16696-NO
CLAGSARCEFHKPSSCCSGHCIFWWCA
>ID16704-NO
CLDAGEICDFFFPTCCGYCILLFCA
>ID16705-NO
CLDAGEMCDLFNSKCCSGWCIILFCA
>ID16728-NO
CLGFGEACLMLYSDCCSYCVGAVCL
>ID16737-NO
CLGSREQCVRDTSCCSMSCTNNICC
>ID16743-NO
CLITNCPRG
>ID16765-ABP_both
CLRKLRKRLLC
>ID16776-NO
CLSPGSSCSPTSYNCCRSCNPYSRKCR
>ID16782-NO
CLTTGETCWLASSCCSFSCTNNVCF
>ID16787-NO
CLWMDWVTEKNINGHQAKFFAC
>ID16806-ABP_both
CNGKRVCVC
>ID16813-NO
CNNRGGGCSQHPHCCSGTCNKTFGVCL
>ID16838-NO
CPLCNGRCAR
>ID16841-NO
CPNTGELCDVVEQNCCYTYCFIVVCPI

>ID16851-NO
CQGRGASCRKTMYNCCSGSCNRGRC
>ID16853-NO
CQGRGASCRKTSYDCCTGSCRSGRC
>ID16929-NO
CRPSGSPCGVTSICCGRCSRGKCT
>ID16932-NO
CRQIKIWFQNRRMKWKK
>ID16963-ABP_pos
CRVYNNGLPTGLYRWC
>ID16969-NO
CRWRWKCSKK
>ID16970-NO
CRWRWKSSKK
>ID16974-NO
CSCKDMTDKECLYFCHQDVIW
>ID16977-NO
CSCSSLLDKECVYFCHLDIIW
>ID16999-NO
CSNLSTCVLGKLSQELHKLQTYPRTDVGAGTP
>ID17005-NO
CSPGGEVCTRHSPCCTGFLCNHIGGMCHH
>ID17008-NO
CSPPGSYCFGPAACCSNFCSTLSDVCQESWSG
>ID17027-NO
CTAPGGACYADNTCCSNACNLNTKKCVLS
>ID17055-NO
CTHPGGACGGHHHCCSQFCNTAANACN
>ID17062-NO
CTPAGDACDATTNCCILFCNLATKKCEVPTFP
>ID17076-NO
CTPPGGYCYHPDPCCSQYCNFPRKHCL
>ID17078-NO
CTPRHGVCFYSYFCCSKACNPSSKRCH
>ID17095-NO
CTSEGYSCSSDSNCCKNVCCWNVCESHCRHPGKR
>ID17131-ABP_both
CVHWGTNTARTACIAP
>ID17161-ABP_both
CVRVRVRPGRVRVRVCW
>ID17178-NO
CWKKKKKKKKKKKKK
>ID17187-ABP_both
CWTKSIPPKPCF
>ID17201-ABP_both
CWWWWKKKKKC
>ID17214-NO
CYDGGTGCDSGNQCCSGWCIFACL
>ID17216-NO
CYDGGTSCDSGIQCCSGWCIFVCF
>ID17220-NO

CYDSGTSCNTGNQCCSGWCIFVSCL
>ID17233-NO
CYIQNCPRG
>ID17249-NO
DAADFGDPYSFLYRLIQAEARKMSGCSN
>ID17261-NO
DADEGDTLANSSDLLKELLGTGDNRAKDSHQQESTNNDEDMS
>ID17291-ABP_neg
DAIWNLLRQAQEKFGKNKSPK
>ID17328-NO
DCCGVKLEMCHPCLCDNSCKNYGK
>ID17340-NO
DCCSLSACVPPPACECC
>ID17350-NO
DCGGQGEGCYTQPCCPGLRCRGGGTGGGVCQL
>ID17367-NO
DCLGWFKSCDPKNDKCCKNYTCSRRDRWCKYYL
>ID17397-NO
DCSGSGYGCKNTPCCDGLTCRGPHQGPICL
>ID17403-NO
DCTPPDGACGFHYHCCSKFCITISSTCN
>ID17405-NO
DCTPPGGACGFYYHCCSNYCITISSTCN
>ID17429-NO
DDCGGLFSGCDSNADCCEGYVCRLWCKYKL
>ID17431-NO
DDCIKPYGFCSLPILKNGLCCSGACVGVCADLP
>ID17435-NO
DDCLGMFSSCDPNNDKCCPNRVCRVRDQWCKYKLW
>ID17488-ABP_both
DDGDKGMLKWKNDFFQEF
>ID17496-NO
DDGVEITEEEVKRGLMDTVKNAAKNLAGQMLDKLKCKITGSC
>ID17539-NO
DECCPDPPCKASNPDLCDWRS
>ID17543-NO
DECCSNPACRLNNPHDCRRR
>ID17596-NO
DENRNFLRF
>ID17623-NO
DFDEIDRAGF
>ID17637-NO
DFDGAMPGVLRF
>ID17640-NO
DFDMLRCMLGRVFRPCWQY
>ID17658-NO
DFPLSKEYETCVRPRKCQPPLKCNKAQICVDPKKGW
>ID17670-NO
DGCCSDPACAVNHPDICGG
>ID17677-NO
DGCSNAGGFCGIHPGLCCSEICLVWCT

>ID17701-NO
DGRCCHPACGKHFSC
>ID17706-NO
DGRMYSFGL
>ID17778-NO
DHLSHDVYSPRL
>ID17799-ABP_both
DIFGAIWPLALGALKNLIK
>ID17812-NO
DIPEVVVSLAWDESLAPKHPGSRKNMACYCRIPACIAGERRYGTCIYQGR
>ID17823-NO
DKEGTLDFLECGSPHSAVPR
>ID17902-NO
DLIWKLLSKAQEGFGKNKSR
>ID17904-NO
DLIWKLLSKAQEKFGANKSR
>ID17905-NO
DLIWKLLSKAQEKFGGNKSR
>ID17911-NO
DLIWKLLSKAQEKFGKNKSRFKCRRWQWRMKKLG
>ID17923-NO
DLLTAIKRVKESMKRRT
>ID17933-NO
DLQRRCVIALPHKMRCRVTGRC
>ID17946-NO
DLSRFYGHFN
>ID17954-NO
DLWIRETLTSPKSLID
>ID18007-NO
DPFFKVPVNKLAAAVSNFGYDLYRVRSSTSPTTN
>ID18013-NO
DPKQDFMRF
>ID18023-NO
DPSFNSWG
>ID18025-NO
DPSHRISDRDYMGWMDFG
>ID18036-NO
DQDEGNFRRFPTNAVSMSADENSPFDLSNEDGAVYQ
>ID18043-NO
DQLIPFPRV
>ID18109-NO
DRTPALRLRF
>ID18150-NO
DSMGALKLAKLLIDKMKCEVTKAC
>ID18180-NO
DTMRCMVGRVYRPCWEV
>ID18226-ABP_both
DVIKKVASVIG
>ID18268-NO
DWEYHAHPKPNSFW
>ID18336-NO

DYTIRTRLHQELSRKVL
>ID18348-NO
EAAGLIPFPRVG
>ID18350-NO
EAAGLLAFPRT
>ID18359-NO
EACYNAGTFCGIKPGLCCSAICLSFVCISFDF
>ID18364-NO
EADPNKFYGLM
>ID18370-NO
EAEEPSAFMTRL
>ID18380-NO
EAGQDLSPSISIV
>ID18395-NO
EAQGWNKFRGAW
>ID18408-NO
ECATKNKRCADWAGPWCCDGLYCSCRSYPGCMCRPSS
>ID18415-NO
ECCGSFACRFGCVPCCV
>ID18416-NO
ECCHPACGKHFSC
>ID18421-NO
ECCNPACGRHYSCGK
>ID18426-NO
ECCSHPACNVDHPEICR
>ID18456-NO
ECKYLWGTCEKDEHCCEHLGCNKKHGWCGWDGTFG
>ID18468-NO
ECLGFGKGCNPSNDQCCKSANLVCSRKHRWCKYEI
>ID18496-NO
ECRKMFGGCSVDSDCCAHLGCKPTLKYCAWDGT
>ID18515-NO
ECTHSGGACNSHDQCCNTFCDTATRTCV
>ID18516-NO
ECTHSGGACNSHNQCCNAFCDTATRTCV
>ID18528-NO
ECTRSGGACNSHDQCCANFCRKATSTCM
>ID18530-NO
ECTRSGGACNSHTQCCDDFCDTATRTCV
>ID18532-NO
ECTRSGGACNSHTQCCDDFCSTATSTCT
>ID18567-NO
EDIKPKTSLAFR
>ID18588-NO
EDVVHSFLRF
>ID18625-NO
EEIIRKLQQLLFIEFRIKRRRRRRRR
>ID18636-NO
EEPAYQRFL
>ID18645-NO
EEVFDDTDVGDELTNALESVLTDFKD

>ID18655-ABP_both
EFELDRICCGYGTARCRKKRSQEYRIGRCPNTYACCLRK
>ID18656-NO
EFELDRICGYGTARCRKKCRSQEYRIGRCPNTFACCLRKWDESLLNRTKP
>ID18658-ABP_both
EFELDRISSGYGTARCRKKRSQEYRIGRSPNTYASCLRK
>ID18672-NO
EFNADDLTLRFG
>ID18678-NO
EGCAFEGESCNVEFYPCCPGLGLTCIPGNPDGTCYYL
>ID18717-NO
EHWSFGLSPG
>ID18723-NO
EHWSHGWYPG
>ID18726-NO
EHWSYGLRPG
>ID18729-NO
EHWSYGWLPG
>ID18742-NO
EIDRSGFGFA
>ID18747-NO
EIGDEENSAKFPIG
>ID18749-NO
EIIIIVGG
>ID18784-ABP_both
EKCLRWQWEMRKVGG
>ID18797-NO
EKGKKIFIMK
>ID18874-ABP_both
ELDRIGYGTARCRKKCR
>ID18903-NO
ELNFSPGW
>ID18904-NO
ELNFSPNW
>ID18913-NO
ELNYSPDW
>ID18926-NO
ELSLEFDYPDTNSEEDLDDGELLDGPVKKDRKYKMHHFRWEGPPKD
>ID18930-NO
ELSLELDYPEIDLDEDIEDNEVERALTKKNGNYRMHHFRWGSPPKD
>ID18935-NO
ELTFSPDW
>ID18940-NO
ELTFTPGWGY
>ID18944-NO
ELTFTSSWG
>ID18976-NO
EMPSLRLRF
>ID18990-NO
ENFAGGCATGFLRTADGRCKPTF
>ID18996-NO

ENFSGGCVAGYMRTPDGRCKPTFYQ
>ID19006-NO
ENPNRFIGLM
>ID19034-NO
EPGWNNLKGLW
>ID19060-NO
EQDYTGWMDF
>ID19145-NO
ESERNERCCHPACARKYNCGR
>ID19149-ABP_both
ESGNEPLWLYQGDNIPKAPSTAEHPFLPSIIDDVKFNPDRRYA
>ID19219-NO
EVNFSPNW
>ID19224-NO
EVNFSTGW
>ID19225-NO
EVNFSTSW
>ID19227-NO
EVNFTPNWGT
>ID19251-ABP_both
EWFKARRWQWRMKKLGA
>ID19261-NO
EWPRPQIPP
>ID19289-NO
FAEPLPSEEEGESYSKEVPEME
>ID19291-NO
FAESLPSDEEAESYSKEVPEIE
>ID19298-ABP_both
FAHHIFRGIVHVGKTIHRLVTG
>ID19300-ABP_both
FAKGIAGMAGKLF
>ID19306-NO
FALGAVTKLLPSLLCMISRKC
>ID19316-NO
FAQTQSLVYP
>ID19349-NO
FCYWKVCW
>ID19371-NO
FDEIDRSGFA
>ID19377-NO
FDEIDRSSFGFA
>ID19418-ABP_both
FFAAIFAPIVHVGKTIHRLVTG
>ID19420-ABP_both
FFARIRGGRAAVLNALGKEEQIGRASNSGRKCARKKK
>ID19442-ABP_both
FFFHIIKGLFHAGRMIHGLVNRRRHRHGMEELDLDQRAFEREKAFA
>ID19463-ABP_both
FFGWLIKGAIHAPKAIHGLIHRRRH
>ID19466-ABP_both
FFHHAFRGIVHVGKTIHRLVTG

>ID19467-ABP_both
FFHHFARGIVHVGKTIHRLVTG
>ID19470-ABP_both
FFHHIFRGAVHVGKTIHRLVTG
>ID19471-ABP_both
FFHHIFRGIKHVGKTIHRLVTG
>ID19472-ABP_both
FFHHIFRGIVHIGKTIHRLVTG
>ID19473-ABP_neg
FFHHIFRGIVHKGKTIHRLVTG
>ID19475-ABP_both
FFHHIFRGIVHVGKKIHRLVTG
>ID19497-NO
FFLPPCAYKGTC
>ID19499-NO
FFPALISCVVLKNC
>ID19502-ABP_both
FFPGIIKVAGAILPTAICAITKRC
>ID19507-ABP_neg
FFPIFKKLLRGLF
>ID19533-ABP_neg
FFPLVKKLLNGLF
>ID19537-ABP_neg
FFPMVKKLLNGLF
>ID19541-ABP_neg
FFPWVKKLLKGLF
>ID19548-ABP_both
FFSLIPKLVKGLISAFK
>ID19551-ABP_both
FFSMIPHIATGIASLVKNL
>ID19555-ABP_both
FFTPVLQADFQKVVAGVANALAHRYH
>ID19572-NO
FGETSGETKGMWFGPRL
>ID19574-NO
FGFTGARKSARKLANQ
>ID19597-NO
FHERDPTHIKWGD
>ID19603-ABP_both
FHFHLHFPSPSPFIKHFIHRF
>ID19617-ABP_both
FIFHIIKGLF
>ID19619-ABP_both
FIFHIIKGLFHA
>ID19626-ABP_both
FIGGLRRLFATVVGTVVGAINKLGGG
>ID19632-NO
FIGPVLKIAAGILPTAICKGFKKC
>ID19645-ABP_both
FIKHFIHRFSGGLQLLKQLLKLLKQF
>ID19646-ABP_both

FIKHFIHRFSGGRWRRLLKKLHHLLH
>ID19660-ABP_neg
FIPIVKKLLSALF
>ID19717-ABP_neg
FKCRRWQWRMKKLGAPSITCVRRAE
>ID19720-NO
FKCRRWQWRMKKLGDLIWKLLSKAQEKFGKNKSR
>ID19738-ABP_both
FKHHIFRGIKHVGKTIHRLVTG
>ID19761-NO
FKNVQLSTARGF
>ID19769-ABP_both
FKRFKGSVKHGKGHLVHHIGVAL
>ID19787-ABP_both
FKWRRWWWRMKKLWA
>ID19794-NO
FLAKKVAKKLVSHVAQKQME
>ID19797-NO
FLAKKVAKTVAKQAAKQGAKYVVNKQME
>ID19811-ABP_both
FLFKLIPKAIKGLVKAIRK
>ID19817-NO
FLFPKANIINSLFGK
>ID19828-ABP_both
FLGALFKALSKVL
>ID19836-ABP_both
FLGALFKVASKVL
>ID19853-ABP_both
FLGALWNVWKSVF
>ID19861-ABP_both
FLGELWNVAKSVF
>ID19869-NO
FLGGLLFGIFKHLGKK
>ID19876-ABP_both
FLGGVFKKASKVFGAVFGKV
>ID19882-ABP_both
FLGKVFKGASKVVPAVFGKV
>ID19883-ABP_both
FLGKVFKGAVKVFPAVFGKV
>ID19889-ABP_both
FLGKVFKKVSKVFPAVFGKV
>ID19890-ABP_both
FLGKVFKLASKVFGAVFGKV
>ID19909-NO
FLGTLLGVGAKILPSLICKISGKC
>ID19922-ABP_both
FLGVVFKLASKVFKAVFGKV
>ID19923-ABP_both
FLGVVFKLASKVFPAVFKKV
>ID19924-ABP_both
FLGVVFKLASKVFPAVVGKV

>ID19941-ABP_both
FLKALFKVASKVL
>ID19947-ABP_both
FLKGIKGMLGKLF
>ID19952-ABP_both
FLKGIVGMLGKLW
>ID19967-ABP_neg
FLLLIPRKIVTAL
>ID19968-NO
FLNPFRWMINKYREWKNKKN
>ID19981-NO
FLPAVIRVAANVLPTVFCAISK
>ID19982-NO
FLPAVIRVAANVLPTVFCAITKKC
>ID19994-NO
FLPFLKSILGKI
>ID19997-ABP_both
FLPGLECVSGKIVPTVFCAITRIC
>ID19998-NO
FLPGLIAGIAKM
>ID20003-NO
FLPIALKALGSIFPKI
>ID20007-NO
FLPIASMLGKYLGK
>ID20012-NO
FLPIIASLLSKLL
>ID20037-ABP_neg
FLPIVKKVLRGLF
>ID20038-NO
FLPIVTGLLTSLL
>ID20041-ABP_both
FLPKLLAGLPSFLCLVFKKC
>ID20077-NO
FLPLVASLAANFLPKLFCKITKKC
>ID20118-NO
FLSGILKLASKIPSVLCAVLKNC
>ID20124-ABP_both
FLSLIPHAIKAVGVHAKHF
>ID20148-ABP_both
FLWALFKVASK
>ID20157-NO
FMPIIGRLMSGS
>ID20185-NO
FPELQQDLIARLLGK
>ID20309-ABP_both
FRRFFKWPRRFFKFF
>ID20324-ABP_both
FRVTWRTKWWKG
>ID20362-NO
FSQAQGKVDMPLPRQRTS
>ID20367-NO

FSVLGAVAKHVLPHVVPVIAEK
>ID20380-NO
FTMKKSLFLLFFLGTISLSLC
>ID20383-NO
FTMKKSLLLIFFLGTISLSLC
>ID20409-NO
FVAPFPEVFG
>ID20410-NO
FVAPFPEVFGK
>ID20412-NO
FVAPFPEVFGKEKVNELSKDIGS
>ID20440-NO
FVNQHLCGSHLVEALYLVCGERGFFYTPKF
>ID20448-NO
FVPIFTHSELQKIREKERNKGQ
>ID20537-ABP_both
FWRRIRVTPVVNPWFLQQT
>ID20617-NO
GAAPAAQFSPRL
>ID20662-ABP_both
GALRRLGRKITHAVKKYGPTVLRIIRIAG
>ID20663-NO
GAMPFSGGMYG
>ID20691-NO
GARPPLRCKAALC
>ID20704-ABP_pos
GATAIKQVKKLFKKKGG
>ID20742-NO
GCCCNPACGPNYGCGTSCSRPSEP
>ID20771-NO
GCCHLLACRMGCTPCCW
>ID20775-NO
GCCIEPLCYQYDCDCCRHL
>ID20782-NO
GCCPIGPCLQSVCSPCCP
>ID20814-NO
GCCSDPPCRNKHPDLCG
>ID20829-NO
GCCSDPRCKHEC
>ID20854-NO
GCCSDPRCRWRCR
>ID20855-NO
GCCSDPRCRYRCR
>ID20867-NO
GCCSFAACRKYRPEMCG
>ID20872-NO
GCCSHPACAGNNPHIC
>ID20915-NO
GCCSLPPCALNNPDYC
>ID20920-NO
GCCSNPACAGSNAHIC

>ID20942-NO
GCCSNPVCHLEHSNLCGGAAGG
>ID20943-NO
GCCSNPVCHLEHSNLCTNG
>ID20955-NO
GCCSRPPCALSNPDYC
>ID20958-NO
GCCSRPPCIANNPDLC
>ID20971-NO
GCCSYPPCFATNPDCAG
>ID20980-NO
GCCSYPPCFATNPDCGGAGGAG
>ID20995-NO
GCGCPLNQGACHRHCKSIGRRGGYCAGFLKQTCTCYRN
>ID21001-NO
GCGYLGEPCCVAPKRAYCHGDLECNNVAMCVN
>ID21013-NO
GCKKYRRFRWKFKGKFWFWCG
>ID21018-NO
GCLEVDYFCGIPFVNNGLCCSGNCVFVCTPQ
>ID21020-NO
GCLGDKCDYNNGCCSGYVCSRTWKWCVLAGPW
>ID21041-ABP_both
GCRRFKKFKKWRYRGRFWFWCFG
>ID21044-ABP_both
GCRRLCYKQRCVTWCRGR
>ID21046-ABP_both
GCRRLCYKQRCVTYCRGR
>ID21069-NO
GCTPPGGACGGHAHCCSQSCDILASTCNA
>ID21073-NO
GCTPRNGFCRYHSHCCSNFCHTWAIMCL
>ID21078-ABP_both
GCTYNVKPDIQGFELYFIGSVTCG
>ID21080-NO
GCVGENQQCADWAGLHCCSGYYCTCRYFPKCICRKDS
>ID21105-NO
GDCVGESQQCADWSGPYCCKGYYCTCRYFPKCICVNDN
>ID21175-NO
GEEEHSKYQECLREIRVNNVQQEC
>ID21194-ABP_both
GEILANLATGLINTLENLLTTKGADKVK
>ID21401-ABP_both
GFCRCLCRRGVCRCICTK
>ID21773-ABP_both
GFFDRIKALTKNVTLELLNTITGKLGVTGG
>ID21788-ABP_both
GFGCNGPWQEDDVKCHNHCKSIKGYKGGYCAKGGFVCKCY
>ID21794-ABP_pos
GFGCPFNLNECHAHCLSIGRKFGFCAGPLRATCTCGKQ
>ID21804-NO

GFGCYRSCWKAGHDEETCKKECS
>ID21816-NO
GFGSFLGKALKAGLKLGANLLGGAPQQ
>ID21829-ABP_both
GFGVLAKVAAHVVPAIAEHF
>ID21836-ABP_both
GFIFHIIKGLFHAGK
>ID21843-ABP_both
GFKAIVQRIKDFLRNLV
>ID21857-NO
GFLDIIKNLGKTFAGHMLDKIKCTIGTCPPSP
>ID21862-NO
GFLDTLKNMAINAAKDAGVSVLNPLSCKLFKTC
>ID21864-NO
GFLDTLKNMAINAAKGAGVSVLNALSCKLFKTC
>ID21881-NO
GFLRGCWTKSFPPKPCLKP
>ID21920-NO
GFRQDAASRVAHGF
>ID21926-ABP_both
GFSSIFRGVAKFASKGLGKDLAKLGVDLVASKISKQS
>ID21930-ABP_both
GFWDSVKEGLKNAAVTILNKIKCKISECGGA
>ID21954-ABP_both
GGARSLGRKALRAWKKYGPIIVPIIRI
>ID21965-NO
GGCCSHPVCNLNNPQMCR
>ID21969-NO
GGCCSRPPCILKHPEIC
>ID21974-NO
GGCLPHNRFCNALSGPRCCSGLTCKELNIWASKCL
>ID21980-NO
GGCYCPFRQDKCHRHCRSFGRKAGYCGNFLKRTCICVKK
>ID21986-NO
GGETGGEGKGMWFGPRL
>ID21988-NO
GGETGSDAKAMWFGPRL
>ID21998-NO
GGGEYDDYGHLRF
>ID22006-NO
GGGGSGETSGMWFGPRL
>ID22035-ABP_both
GGHKLAKLAKKLAKLAK
>ID22068-ABP_both
GGLRSLGRKALRAWKKYGPIIVPIIRI
>ID22069-ABP_both
GGLRSLGRKILRAWKKGGPIIVPIIRI
>ID22078-NO
GGPQGPLRFG
>ID22108-NO
GGSSRSGCFGHKMDRIGTISGMGC

>ID22119-NO
GGVCPKILAKCRRDSDCPGACICRGNGYCGSGSD
>ID22122-NO
GGVGRCIYNCMNSGGGLNFIQCKTMCY
>ID22177-NO
GIADILKGLL
>ID22185-ABP_both
GICKFLHSAKKFGKAFVGEIMNS
>ID22203-NO
GIFGKILGVGKEVLCGLSGMC
>ID22204-NO
GIFGKILGVGKKVLCELSGMC
>ID22205-NO
GIFGKILGVGKKVLCGLSGIC
>ID22206-NO
GIFGKILGVGKKVLCGLSGKW
>ID22232-ABP_neg
GIGAVLDVLTTGLPALISWIKRKRQQ
>ID22233-NO
GIGAVLEVLTTGLPALISWIKRKRQQ
>ID22234-ABP_neg
GIGAVLGVLTTGLPALISWIKRKRQQ
>ID22252-NO
GIGGALLSAGKSALKGLAKGLADHFAN
>ID22258-NO
GIGGALLSVGKSALKGLAKGFAEHF
>ID22264-NO
GIGGKILGGLKTALKGAAKELAATYLQ
>ID22268-NO
GIGGKILGGPKTALKGAAKELASTYLH
>ID22269-NO
GIGGKILGGVKTALKGALKELASTYAN
>ID22270-NO
GIGGKILPGFKTALKGAAKELAATYLH
>ID22271-NO
GIGGKILSGFKTALKGAAKELAATYLH
>ID22272-NO
GIGGKILSGFKTALKGAAKELAFTYLH
>ID22278-ABP_both
GIGHFLHKVKSFGKSWIGEIMNS
>ID22291-ABP_neg
GIGKFLHSAKKFGKAFVGEIMNSGGC
>ID22302-ABP_both
GIGKFLKKAKKFGKAFV
>ID22312-NO
GIGTKFLGGLKTAVKGALKELAFTYVN
>ID22318-NO
GIGTKIIGGLKTAVKGALKELVFTYVN
>ID22326-ABP_both
GIHDILKYGKKS
>ID22335-ABP_both

GIINTLQKYYARVRGGRAAVLSALPKEEQIGKASTRGRKCARRKK
>ID22362-NO
GILDCFKNMALNAAKSAGTSVLNALSCKLSKTC
>ID22366-ABP_both
GILDTFKGVAKGVAKDLAVHMLEKLKCKMTGC
>ID22375-ABP_both
GILDTLKQFAKGVGKDLVKGAAQGVLSTV
>ID22377-ABP_both
GILDTLKQFAKGVGKFLVKGAAQ
>ID22383-NO
GILGKIWEGVKS
>ID22390-NO
GILLPALRKFCCDSNWCNISDCECCY
>ID22398-NO
GILSGLLGAGKKIVCGLSGLC
>ID22405-ABP_both
GILSSLLKKLKKIIAK
>ID22418-ABP_both
GIMSSLMKKLAAIIAK
>ID22429-NO
GINYWLAHK
>ID22496-NO
GIRGNCCMFHTCPIDYSRFYCP
>ID22501-ABP_both
GIRRILRYGRRS
>ID22517-NO
GIVEQCCASTCSLYQLENYCN
>ID22520-NO
GIVEQCCENPCSLYQLENYCN
>ID22529-NO
GIVEQCCHSTCSLFQLESYCN
>ID22531-NO
GIVEQCCTGICSLYQLENYCN
>ID22545-ABP_both
GIWDTIKSMGKVFAGKIKQNL
>ID22548-ABP_both
GIWGTLAKIGIKAVPRVISMLKKKQ
>ID22552-NO
GIWSSIKNLASKAWNSDIGQSLRNKAAGAINKFVADKIGVTPSQAASMTL
>ID22561-ABP_both
GKAMSLLKHILK
>ID22614-NO
GKKKKKKKKK
>ID22630-ABP_both
GKKYRRFRWKFRKGRFWFWG
>ID22632-ABP_both
GKLFKKILKFL
>ID22667-ABP_neg
GKPRPYSPRPSSHPRPIRV
>ID22699-NO
GKRRRRATAKYRSAH

>ID22790-ABP_both
GLFDIVKKVVGAAGSL
>ID22805-ABP_both
GLFDVIKKVAS
>ID22809-ABP_both
GLFDVIKKVASVI
>ID22824-ABP_both
GLFGVLAKVAAKVVPAIAEHF
>ID22829-NO
GLFKTLIKGAGKMLGHVAKEFLGSEGEPES
>ID22831-NO
GLFKTLIKGAGKMLGHVAKEFLGSQGQPES
>ID22863-NO
GLFSKFAGKGIKDLIFKGVKHIGKEVGMDVIRTGIDAAGCKIKGEC
>ID22864-NO
GLFSKFAGKGIKDLIFKGVKHIGKEVGMDVIRTGIDVAGRKIKGEC
>ID22865-NO
GLFSKFAGKGIKDLIFKGVKHIGKEVGTDVIRTGIDVAGCKIKGEC
>ID22866-NO
GLFSKFAGKGIKNFIIKGVKHIGKEVGMDVIRTGIDVAGCKI
>ID22886-NO
GLFTLIKGAAKLIGKAVAKEAGKTGLELMACKITNQC
>ID22887-NO
GLFTLIKGAAKLIGKIVAKEAGKTGLELMACKITNQC
>ID22888-NO
GLFTLIKGAAKLIGKTAAKEAGKTGLELMACKITNQC
>ID22889-NO
GLFTLIKGAAKLIGKTVAKEAGKTGLEFMACKITNQC
>ID22917-NO
GLICESCRKIIQKLEDMVGPQPNEDTVTQAASQVCDKLKILRGLCKKIMR
>ID22920-NO
GLIDTVKNMAINAAKSAGMSVLKTLSCKLSKEC
>ID22923-NO
GLIGSIGKALGGLLVDVLKPKLQ
>ID22930-NO
GLISGILGAGKKIVCGLSGLC
>ID22932-NO
GLISGKSVKGSI
>ID22958-NO
GLLDTIKNMAINAAKGAGVSVLNALSCKLSKTC
>ID22963-NO
GLLDTLKNVAIGVAKGAGVGVLNALSCKLDKSC
>ID23002-ABP_both
GLLKRIKKLL
>ID23009-ABP_both
GLLKRIKTLA
>ID23032-NO
GLLNVIKDTAQNLFTAALEKLKCKVTKCN
>ID23071-NO
GLLSGILGAGEHIVCGLSGLC
>ID23072-NO

GLLSGILGAGKHIVCGLSRLC
>ID23073-NO
GLLSGILGAGKHIVCGLSWLC
>ID23074-NO
GLLSGILGAGKHIVCRLSGLC
>ID23075-NO
GLLSGILGAGKHTVCGLSGLC
>ID23077-ABP_both
GLLSGILGAGKKIV
>ID23090-NO
GLLSGVLGVGEKIVCGLSGLC
>ID23091-NO
GLLSGVLGVGKKIVCGLSGL
>ID23114-NO
GLLSVLGSVAKHVLP
>ID23139-ABP_both
GLNALKKVFQGIHEAFKLINNHVQ
>ID23140-ABP_both
GLNALKKVFQGIHEAIKKINNHVQ
>ID23141-ABP_both
GLNALKKVFQGIHEAIKLFNNHVQ
>ID23148-ABP_both
GLNALKKVFQPIHEAIKKINNHVQ
>ID23152-ABP_both
GLNALKKVSQGIHESIKLINNHVQ
>ID23157-NO
GLNRPSKRCLAGSARCEFHKPSTCCSGHCIFWWCA
>ID23159-NO
GLPACGETCVGGTCNTPGCTCSWPVCTRN
>ID23185-NO
GLPVCAETCVGGTCNTPGCTCSWPVCTRN
>ID23186-NO
GLPVCGATCVGGTCNTPGCTCSWPVCTRN
>ID23188-NO
GLPVCGEACVGGTCNTPGCTCSWPVCTRN
>ID23192-NO
GLPVCGETCAGGTCNTPGCTCSWPVCTRN
>ID23204-ABP_both
GLPVCGETCTLGTCSTQGCTCSWPICKRN
>ID23206-NO
GLPVCGETCVAGTCNTPGCTCSWPVCTRN
>ID23207-NO
GLPVCGETCVGATCNTPGCTCSWPVCTRN
>ID23208-NO
GLPVCGETCVGGACNTPGCTCSWPVCTRN
>ID23245-NO
GLPVCGETCVGGTCNTPGCTCSKNKCTRN
>ID23296-ABP_neg
GLRKRLRKFRNKIKEKLKKIGQKDQGLLPKLAPRTDY
>ID23297-ABP_neg
GLRKRLRKFRNKIKEKLKKIGQKGQGLLPKLAPRTDY

>ID23298-ABP_neg
GLRKRLRKFRNKIKEKLKKIGQKIQGPLPKLAPRTDY
>ID23299-ABP_neg
GLRKRLRKFRNKIKEKLKKIGQKIQTLLPKLAPRTDY
>ID23309-NO
GLRPPIRCKAAFC
>ID23318-NO
GLSRPSKGCIGGGDPCEFHRGYTCCSEHCIIWVCA
>ID23323-NO
GLTFLSPADMQKIAERQSQNKLRHGNM
>ID23358-NO
GLWRALWRALWRSLWKLKWKV
>ID23363-NO
GLWRALWRLLRSLWRLLWRA
>ID23368-NO
GLWSKIKEAAKTAGKAAMGFVDEMV
>ID23390-ABP_neg
GMASKAGAIAGKIAKVAWKALGGC
>ID23395-NO
GMASKAGTIVGKIAKVALNAL
>ID23397-ABP_both
GMASLLAKVLPKVVKLIK
>ID23442-NO
GMWSKIKEAGKAAAKAAAKAAGKAALDVVSGAI
>ID23460-ABP_both
GNGNLLGGLLRPVLGVVKGLTGGLGKK
>ID23480-ABP_neg
GNNRPVYIPQPRPPHPRF
>ID23485-ABP_both
GNNRPVYIPRPRPPHPRI
>ID23500-NO
GNRNFLRF
>ID23592-NO
GPPSECFWKYCV
>ID23604-ABP_both
GPRSSNHKRGLSRVGNSNPGKYLAKYSFRPVILSSKPLSPTPTKT
>ID23608-NO
GPSASSGLWFGPRL
>ID23625-NO
GPSSGFFGTR
>ID23693-NO
GRCCHPACGENTSC
>ID23703-NO
GRCCHPACGKNYSC
>ID23707-NO
GRCCHPACGQNTDC
>ID23708-NO
GRCCHPACGQNTKC
>ID23710-NO
GRCCHPACGQQTSC
>ID23732-ABP_both

GRFKRFRKKAKKLFKKLSPVIPLLHL
>ID23748-ABP_both
GRFRRLRKKTRKRLKKIGKVLKLI
>ID23752-NO
GRGGASNYVRL
>ID23756-ABP_both
GRGKQGGKVRAKAKTRSS
>ID23757-NO
GRGKRRREKQRPCDKPRR
>ID23766-NO
GRKKRRQRARPPQC
>ID23771-NO
GRKKRRQRRRPP
>ID23785-NO
GRKYKMHHFRWEGPPKD
>ID23797-NO
GRLYSFGL
>ID23809-ABP_both
GRPNPVNNKPTPHPRPIRV
>ID23812-ABP_neg
GRPNPVPNPKPPHPRL
>ID23814-NO
GRPPGFSPFRID
>ID23820-NO
GRQLRIAGKRLEGRSK
>ID23824-NO
GRQLRRAGRRLRGRSR
>ID23846-ABP_both
GRRPRPRPRPWWWWW
>ID23862-NO
GRSDNFIRF
>ID23890-NO
GSCLELGEYCNGSKDDCQCCRDNAYCGCDIF
>ID23895-NO
GSDPNFLRF
>ID23902-NO
GSGFFGMR
>ID23904-NO
GSGGSGEANGMWFGPRL
>ID23907-NO
GSGKKGGKKICQKY
>ID23909-NO
GSGQDFMRF
>ID23942-ABP_neg
GSKKPVPIIYCQRRTAKCQRM
>ID23954-NO
GSNFCDSKCKLRCSKAGLADRCLKYCGICCEECKCVPSGTYGNKHECPCY
>ID23956-NO
GSNWSNLRGAW
>ID23978-NO
GSSFLSPSQKPQVRQGKGKPPRV

>ID23988-NO
GSSGLIPMGRT
>ID23991-NO
GSSGLISFPRN
>ID23994-NO
GSSGLISMTRV
>ID23995-NO
GSSGLISVPRV
>ID24003-NO
GSTGLIPFGRP
>ID24049-NO
GTFAILKIATKLIGKTLAKAAGKAGTGLLACKAAKEC
>ID24113-NO
GVDCVGLSSYCGPWNNPPCCSWYTCDYYCKF
>ID24123-ABP_both
GVFDIIKDAGRQLVAHAMGKIAEKV
>ID24125-NO
GVFLDTLKNMAGKMLESLKCKLFGCKP
>ID24163-NO
GVLGTVKDLLIGAGKSAAQSVLKTLSCKLSNDC
>ID24165-NO
GVLGTVKNLLIGAGKSAAQSVLETLSCKLSNDC
>ID24177-NO
GVNACSSLF
>ID24200-NO
GVPKVKETMVPKH
>ID24220-ABP_both
GVVDILKGAAKAIAGHLASKVMNKL
>ID24221-ABP_both
GVVDILKGAAKDAAGHLASKVMNKL
>ID24222-ABP_both
GVVDILKGAAKDIAAHLASKVMNKL
>ID24223-ABP_both
GVVDILKGAAKDIAGALASKVMNKL
>ID24224-ABP_both
GVVDILKGAAKDIAGHASKVMNKL
>ID24251-ABP_both
GVVVRWGRVVVRGVRR
>ID24268-NO
GWASKIGETLGKMAKVGLHELIQPK
>ID24270-ABP_both
GWASKIGQTLGKMAKVGLQEIIQPK
>ID24341-NO
GWTLNSAGYLLGKINLKALAALAKKLL
>ID24342-NO
GWTLNSAGYLLGKINLKAPAALAKKIL
>ID24355-NO
GWTNLSAGYLLGPHAVDNHRSLNDKHGLA
>ID24412-NO
GYGCPNDYACSSYCSSIGRNGGYCGGFLWQTCKCNEKK
>ID24415-ABP_both

GYGGVSLPEWVCTTFALCSEK
>ID24425-NO
GYNRSFLRF
>ID24434-NO
GYRKPPFNGSIFG
>ID24452-NO
HADGIFTSVYSHLLAKLAVKRYLHSLI
>ID24455-NO
HADGMFNKAYRKALGQLSARKYLHSLMAKRVGGGSTMEDDTEPLS
>ID24460-NO
HADGSFSDEMNTVLDHLATKDFINWLIQTKITD
>ID24475-NO
HADGTYTSDVSSYLQEQAAKDFITWLKSGQPKPE
>ID24476-NO
HADGTYTSDVSTYLQDQAAKDFVSWLKSGPA
>ID24478-NO
HADGVFTSDFSRLLGQLSAKKYLESLI
>ID24482-NO
HAEGTFTNDMTNYLEEKAAKEFVGWLIKGRP
>ID24484-NO
HAEGTFTSDMTSYLEEKAAKEFVDWLIKGRPK
>ID24487-NO
HAEGTFTSDVTQQLDEKAAKEFIDWLINGGPSKEIIS
>ID24489-NO
HAEGTYTNDVTEYLEEKATKAFIEWLIKGKP
>ID24534-ABP_both
HFLGTKVNLAKKIL
>ID24535-ABP_both
HFLGTLKNLAKKIL
>ID24603-NO
HIDGIFTDSYSRYRKQMAVKKYLAAVL
>ID24615-ABP_both
HIQKEDVPSERYLGYLEQLLRLKK
>ID24623-NO
HISPSYDVEIDAGNMRNLLDIG
>ID24715-NO
HPFAQTQSLVYP
>ID24774-ABP_both
HRWWRWWRR
>ID24778-NO
HSDAIFTEEYSKLLAKLALQKYLASILGSRTSPPP
>ID24799-NO
HSEGTFSNDYSKYLETRRAQDFVQWLKNS
>ID24812-NO
HSLQNTEEKSSSFPAPQTDPLGDPDQISED
>ID24822-NO
HSQGTFTSDYSKYLDSRRAQDFVQWLMST
>ID24826-NO
HSQGTFTSDYSKYLDTRRAQDFVQWLMST
>ID24836-ABP_both
HTCHIRRRPKFRKFKLYHEGKFWCPG

>ID24888-NO
HYRIKPTARRLKWKYKGKFW
>ID24936-ABP_neg
IARRALKKALRARHTIPQCKKFGRR
>ID24942-NO
IASGEPTSTPTEE
>ID24945-ABP_both
IASKVANTVQKLKRKAKNAVA
>ID24984-NO
IDEIDRTAFDNFF
>ID24985-ABP_both
IDGLKAIVKKVADLLKNT
>ID24994-NO
IDLSRFYGHFN
>ID25082-ABP_neg
IFPIVKKLLKGLF
>ID25101-ABP_both
IGKFLKKAKKFG
>ID25105-ABP_both
IGKLVKWIIKTVNKFTKK
>ID25133-ABP_both
IHKFWRPGRWFKHI
>ID25139-NO
IHPFAQTQSLVYP
>ID25140-ABP_both
IHQGLPQE
>ID25171-ABP_both
IIGPVLGLIGKALGGLL
>ID25185-NO
IINWCCLIFYQCCL
>ID25269-ABP_both
IKKILSKIKKWLK
>ID25273-ABP_both
IKKIVSKIKKVL
>ID25289-ABP_both
IKKWWSKIKKLLK
>ID25295-ABP_both
IKLSKKTKDNLKKVLKGAIKGAIAVAKMV
>ID25296-ABP_both
IKLSPETKKNLKKVLKGAIKGAIAVAKMV
>ID25303-NO
IKNLQSLDPSHRISDRDYMGWMDFG
>ID25308-ABP_both
IKPAGKLFKLFK
>ID25309-NO
IKPEAPGEDASPEELNRYYASLRHYLNLVTRQRY
>ID25321-ABP_both
IKRLNSWLRK
>ID25327-ABP_both
IKSIASKVANTVQKLKRKAKNAV
>ID25348-ABP_neg

ILAWKWAWWPWRR
>ID25350-NO
ILDALEELD
>ID25355-NO
ILECVFSCDIKKEGKPCKPKGEKKCTGGWRCKIKLCLKI
>ID25370-NO
ILGKLLSTAWKLLSKL
>ID25372-ABP_neg
ILGKLWEGVKSI
>ID25377-NO
ILGPILGLVSNALGGLLG
>ID25386-NO
ILGPVLGLIGNALGGLIKKI
>ID25387-NO
ILGPVLGLVDNALGGLIKKI
>ID25398-NO
ILGPVLGLVGSVLGGLLKNL
>ID25403-NO
ILGPVLGLVSNVLGGLL
>ID25428-ABP_neg
ILKKLLKKVKSI
>ID25483-ABP_both
ILPWKKPKKPKRR
>ID25673-ABP_both
INLKAIAALVKKV
>ID25678-NO
INMKASAAVAKKL
>ID25679-NO
INNDCQNFIGNR
>ID25753-ABP_both
IPRPIDTCRLRNGICFPGICRRPYYWIGTCNNGIGSCCARGWRS
>ID25774-ABP_both
IQPKTKVIPYVR
>ID25802-NO
IRDECCSAPACRVNNPHVC
>ID25804-NO
IRDECCSNAACRVNNPHVC
>ID25806-NO
IRDECCSNPACAVNNPHVC
>ID25940-ABP_both
ISRLAGLLRKGGEKIGEKLKKIGQKIKNFFQKLVPQPEQ
>ID26153-NO
IYPSFQPQPLIYP
>ID26227-ABP_both
KARQWQSKIRRTNPIFAIRR
>ID26248-NO
KAYSMPRCKGGFRAVMCW
>ID26250-NO
KAYSTPRCKGLFRALMCW
>ID26256-NO
KCCMRPICTCPCCIGP

>ID26287-NO
KCNTATCATQRLANFLIRSSNNLGAILSPTNVGSNTY
>ID26290-NO
KCNTATCATQRLTNFLVRSSHNLGAALLPTDVGSNTY
>ID26453-ABP_both
KFLKKAKKFGKA
>ID26454-ABP_both
KFLKKAKKFGKAFVKIL
>ID26507-ABP_both
KGLGKDLAKLGVDLVACKISKQC
>ID26520-ABP_both
KGRGKQGGKVRAKA
>ID26556-ABP_both
KHKWLWLW
>ID26565-ABP_pos
KHRCRVYNNGLPTGLYRWC
>ID26587-ABP_both
KIAGKIAKIAGKIAKIAGKIAKIA
>ID26589-ABP_both
KIAGKIAKIAGKIAKIAGKIAKIAGKIA
>ID26590-ABP_both
KIAGKIAKIAKKIAK
>ID26608-NO
KIEEIESKIKKIENEIARIKKLLQLTVWGIKQLQARIL
>ID26614-NO
KIFEPLRDKNL
>ID26619-ABP_both
KIGAIKKWGAIKKIGAIK
>ID26647-NO
KIKWFKTMKSIAKFIAKEQMKKHLKGE
>ID26656-NO
KILKFLFKQVF
>ID26694-NO
KIYPSFQPQPLIYP
>ID26715-ABP_both
KKAAHVGKHVGKAAKK
>ID26722-ABP_both
KKAKKFGKAFVK
>ID26754-NO
KKFFRAWWAPRFLK
>ID26769-NO
KKIATYQER
>ID26778-ABP_both
KKIMRTFLRRISKDILTGKK
>ID26794-ABP_both
KKKIIIIIIKKK
>ID26810-ABP_both
KKKKKKKKKKKKK
>ID26813-NO
KKKKKKKKKKKKKKKKKKK
>ID26855-ABP_both

KKKWLWLW
>ID26870-NO
KKLFKKILKGL
>ID26871-NO
KKLFKKILKHL
>ID26872-NO
KKLFKKILKIL
>ID26915-ABP_both
KKLIKVFAKGFKKAKKLFKGIG
>ID26959-ABP_both
KKPKLPWKPKK
>ID26960-ABP_both
KKPKLPWLPKK
>ID26978-NO
KKRRQRRR
>ID27012-ABP_both
KKVVFKVKFH
>ID27046-NO
KKWWKKWWK
>ID27066-ABP_both
KKYRYHLKPFCKK
>ID27100-ABP_both
KLFAVIKKVAAVIRRL
>ID27117-ABP_both
KLGVPLKRK
>ID27121-NO
KLIKGRTPIKFGKADCDRPPKHSGK
>ID27156-ABP_both
KLKKLLKKLLKK
>ID27168-ABP_both
KLKRKAKNAV
>ID27176-ABP_both
KLLKFIKTLL
>ID27185-NO
KLLKLLKLKKLLKL
>ID27200-ABP_both
KLLLLLKLLLLK
>ID27217-ABP_both
KLPKWIIKTVNKFTKK
>ID27219-ABP_both
KLPLLPWLPKK
>ID27264-ABP_both
KLWKKWKKWKK
>ID27265-ABP_both
KLWKKWKKWLK
>ID27269-ABP_both
KLWKLWLKWLL
>ID27289-NO
KMDCRWRWKCSKK
>ID27291-NO
KMDCRWRWKSCKK

>ID27295-NO
KMDSRWRWKCSKK
>ID27317-NO
KNDFCQLLYVWLVMFCQQCFVQFFKNVET
>ID27335-NO
KNKFEFIRF
>ID27378-NO
KPAEDLARYYSALRHYINLITRQRY
>ID27381-NO
KPCCSIHDSSCCGI
>ID27426-NO
KPRPHQFFGLM
>ID27444-NO
KQCHKKGGHCFPKEKICIPPSSDFGKMDCRWRWKCCKKGSGK
>ID27456-NO
KQKKIENEIARIKKLLQLTVWGIKQLQARIL
>ID27472-ABP_both
KQWRIRVCVIRA
>ID27510-NO
KRFKQDGGWSHWSPWSSC
>ID27523-ABP_both
KRGKQGGKVRAKAKTRSS
>ID27543-NO
KRIPNKKPGKK
>ID27547-NO
KRIPNKKPGKKTTTKPTKKPTIKTTKK
>ID27551-ABP_both
KRIRKRIKKWKR
>ID27585-ABP_both
KRKILILIKRKRK
>ID27598-ABP_pos
KRKKHRCRVWNNGLPTGLYRWC
>ID27599-ABP_pos
KRKKHRCRVYANGLPTGLYRWC
>ID27600-ABP_pos
KRKKHRCRVYNAGLPTGLYRWC
>ID27601-ABP_pos
KRKKHRCRVYNNALPTGLYRWC
>ID27602-ABP_pos
KRKKHRCRVYNNGAPTGLYRWC
>ID27603-ABP_pos
KRKKHRCRVYNNGLATGLYRWC
>ID27640-ABP_both
KRKWLWLW
>ID27674-NO
KRRREKQRPCDKPRR
>ID27696-NO
KRWRWKCCKK
>ID27824-ABP_both
KVANTVQKLKRKAKNAVA
>ID27835-NO

KVHSSIQSQPQAFT
>ID27869-NO
KVPCCLGVRDDWCCAGQIQI
>ID27878-ABP_both
KVQNQHGQVVKIFHH
>ID27906-NO
KVYPFPGPIPNSLPQNIPP
>ID27914-NO
KWAPEWAPEWI
>ID27921-ABP_both
KWCFRACYRGICYRRCR
>ID27925-ABP_both
KWCFRVCARGICYRRCR
>ID27926-ABP_both
KWCFRVCGRGICYRRCR
>ID27927-ABP_both
KWCFRVCYAGICYRRCA
>ID27928-ABP_both
KWCFRVCYAGICYRRCR
>ID27929-ABP_both
KWCFRVCYRAICYRRCR
>ID27980-NO
KWKLFKKIEKVGQNIRDGIIKAGPAVAWVGQATQIAK
>ID27981-ABP_both
KWKLFKKIEKVGQRVRDAVISAGPAVATVAQATALAK
>ID27983-ABP_both
KWKLFKKIFKRIVQRIKDFLR
>ID28025-ABP_neg
KWKSFIKKLTLKFLHSKKKF
>ID28063-ABP_both
KWKVFKKIEKMIRNIRNKIAK
>ID28118-ABP_pos
KWRRWIRWA
>ID28199-NO
KYAFEVVG
>ID28224-NO
KYPVEPFTESQSLTL
>ID28229-NO
KYVMSHFRWNKF
>ID28292-NO
LANQHLCGSHLVEALYLVCGDRGFFYYPKI
>ID28377-NO
LDPCCRDPPCASTHIDRC
>ID28378-NO
LDPCCRDPPCASTHTDICT
>ID28474-NO
LENPSPQAPARRLLPP
>ID28482-ABP_both
LEQLLRLK
>ID28515-ABP_both
LFKVASKVLPSVK

>ID28567-ABP_both
LGKEEQIGRASNSGRKCARKKK
>ID28582-NO
LGPVRGPFPI
>ID28598-ABP_both
LGYLEQLLR
>ID28639-NO
LIAKTALPQTNK
>ID28664-NO
LIIFRILISHRR
>ID28675-ABP_both
LILGKLWKGVKSIF
>ID28683-NO
LINSLLGIPKVMTDA
>ID28703-NO
LIWKLLSKAQEKFGKNKSR
>ID28727-NO
LKCNKLIPLAYKTCPAGKNLCYKMYMVATP
>ID28757-ABP_both
LKGCWTKSIPPKPCFGG
>ID28759-NO
LKGYGGVSLPEW
>ID28789-ABP_both
LKKLKCCLLLLKKLKK
>ID28802-ABP_both
LKKLLKLLKKLLKLAG
>ID28814-ABP_both
LKKVLKAAA
>ID28836-ABP_both
LKLKKKCKCLLLKKLLL
>ID28839-ABP_both
LKLKKLCLCKLKKKLL
>ID28874-ABP_both
LKRVWKAVFKLLKRYWRQLKPVR
>ID28962-NO
LLGMIPLAISAISSLSK
>ID28963-ABP_both
LLGMIPVAIKAISALSKL
>ID28967-NO
LLGPVLGLVSNDLEVYLKIL
>ID28970-ABP_both
LLGSLLKLLPKLL
>ID28979-NO
LLIILRRRARKQAHAHSK
>ID29044-ABP_both
LLPIVANLLKALL
>ID29046-NO
LLPIVDNLLDGLL
>ID29054-ABP_both
LLPIVGNLLKSLLGWKRKRFG
>ID29096-NO

LLYQQPVLGPVRGPFPIIV
>ID29149-NO
LNPFRWVINKYREWKNKKDS
>ID29150-NO
LNPPHQIYP
>ID29153-NO
LNQEQAEQGREHLA
>ID29162-NO
LNSAGYLLGKLKALAALAKIL
>ID29169-NO
LNVRGCCSHPVCRFNYPKYCGGRR
>ID29179-NO
LPCCDVGWCSRRCNCDVYRP
>ID29182-NO
LPDSSFLRL
>ID29198-NO
LPHYNFGL
>ID29218-NO
LPPCCTPPKRLCPAPACKYKPCCKS
>ID29289-ABP_both
LQLLKQLLKLLKQFSATFIKHFIHRF
>ID29306-NO
LQQLLFAHFRIGRRRRRRRR
>ID29307-NO
LQQLLFIAFRIGRRRRRRRR
>ID29341-NO
LRDLVCYCRKRGCKGRERMNGTCRKGHLLYTMCCR
>ID29345-NO
LRDLVCYCRKRGCKRREHMNGTCRKGHLMYTLCCR
>ID29346-NO
LRDLVCYCRKRGCKRREHMNGTCRRGHLMYTLCCR
>ID29366-ABP_both
LRIVKLILKWLR
>ID29385-ABP_both
LRLKKRRWKYRVPP
>ID29395-NO
LRNQLDIGDL
>ID29433-NO
LRWCIPRGDLCFPSDRIQCCSGKCTFVCM
>ID29466-NO
LSEDMPATPADQEMYQPDPEEMESRTRYFSPRL
>ID29507-NO
LSPTQDFMRF
>ID29532-ABP_both
LTAEEKAAVTAFWGKVKVDEVGGEALGRLLVVY
>ID29581-NO
LTVRECCSQPPCRWKHPELCS
>ID29582-NO
LTVREECCSDPRCSVGHQDMCR
>ID29637-ABP_both
LVQRGRFGRFLRKI

>ID29648-NO
LVRVQETKEGTTRLLNSCEYKGRLSKARAGPAPDHQAEASTVTP
>ID29659-NO
LVVYPWTQRY
>ID29669-NO
LVYPFPGPI
>ID29671-NO
LVYPFPGPIHNSLP
>ID29694-ABP_pos
LWRLWWLWW
>ID29730-NO
LYENKPRRPYI
>ID29741-NO
LYLKQADFDDPRMFTSSFG
>ID30123-NO
MALRDECCASPPCRLNNPYVCH
>ID30193-ABP_both
MAQKIISTIGKLPKWIIKTVNKFTKK
>ID30227-NO
MARSICFFAVAILALMLFAAYDAEA
>ID30328-NO
MAVRGCCSHPACAGNNPHICGRRR
>ID30329-NO
MAVRGGCCSHPVCNLNNPQMCRGRR
>ID30365-NO
MCMPCFTTDHNMAKKCRDCCGGNGKCFGYQCLCNR
>ID30369-NO
MCPKHPLGC
>ID30379-NO
MDANAFRMSF
>ID30410-NO
MDIIIIVGG
>ID30411-NO
MDILIIVGG
>ID30470-NO
MDSNKDERAYAQWVIIILHNVGSSPFKIANLGLSWGKLYADGNKDKEVY
>ID31855-NO
MGYILPALSQQTCCVRPWCDGACDCCVDS
>ID31897-NO
MIIFAIAASHKK
>ID31898-NO
MIIFKIAASHKK
>ID31900-NO
MIIFRAAASHKK
>ID31901-NO
MIIFRALISHKK
>ID31903-NO
MIIFRIAASHKK
>ID31904-NO
MIIFRIAATHKK
>ID31925-NO

MIIYRDLISKK
>ID31969-NO
MISMLRCTFFFLSVILITSYFVTPTMSIKCNCKRHVIKPHICRKICGKNG
>ID32235-NO
MKLMLSALRQQECCKPSTCDGGCYHCC
>ID32253-ABP_both
MKLVKTWLKVLK
>ID32264-NO
MKMMLPALRQQPCCSPSTCDGGCYHCC
>ID32639-ABP_neg
MLTAEDKKLIQQAWEKAASHQEEFGAEALTRMFTTYPQTKTY
>ID32860-NO
MNYLAFPRM
>ID32885-NO
MPFPKYPVEP
>ID32917-NO
MPPSAANLPLRF
>ID33427-NO
MTIKGCCSDPSCNVNNPDYCG
>ID33428-NO
MTIKGCCSYPPCFATNPDCGRRR
>ID33503-NO
MTMGCTHPGGACGGHYHCCSQSCNTAANSCN
>ID33512-ABP_both
MTPFWRGVSLRPIGASCRDNSECITMLCRKNRCFLRSASE
>ID33515-ABP_neg
MTPLWRVMGNKPFGAYCQDHVECSTGICKGGHCIYSQPIKS
>ID33797-NO
NAPAEDLARYYSALRHYINLITRQRY
>ID33824-NO
NCFFNEDFLWCVYATER
>ID33828-NO
NCKGAGAKCSRLPYDCCTGSCRSGACG
>ID33870-NO
NENLLRFFVAPFPEVFG
>ID33892-NO
NFDEIDRSG
>ID33897-NO
NFDEIDRSGFG
>ID33898-NO
NFDEIDRSGFGF
>ID33903-NO
NFDEIDRSGFN
>ID33909-NO
NFDEIDRSSF
>ID33910-NO
NFDEIDRSSFA
>ID33912-NO
NFDEIDRSSFGF
>ID33925-NO
NFEHSCNGYMRPHPRGLCGEDLHVIISNLCSSLGGNRRFLA

>ID33941-NO
NGAPQPFVRF
>ID33946-NO
NGCCRNPACESHRCG
>ID33957-NO
NGHCCHPACGGKYVKC
>ID33960-NO
NGKEICLDPEAPFLKKVIQKILD
>ID33976-NO
NGRCCHPACGKYFSC
>ID33993-ABP_both
NGVQPKYRWWRWWRRWWWW
>ID34043-NO
NIPPLTQTPV
>ID34050-NO
NIQSLLRTGMLPSIAP
>ID34092-ABP_both
NKVKEWIKYLKSLFS
>ID34305-NO
NLDEIDRSDFGRFV
>ID34327-ABP_both
NLLNKALGTVNGLLGRS
>ID34332-NO
NLLQFNKMIKIMTKKNAFPFYTS
>ID34334-NO
NLLQFNKMIKVETGKNAIPFYAF
>ID34394-ABP_both
NNEAQCEQAGGICSKDHCFHLHTRAFGHCQRGRPCCRTRYD
>ID34447-NO
NNNWSKFQGSW
>ID34474-ABP_both
NPEKALEKLIAIQKAIKGMLNGWFTGVGFRRKR
>ID34480-NO
NPGSQDFMRF
>ID34481-NO
NPGTPQHLCGSHLVDALYLVCGPTGFFYNP
>ID34498-NO
NPMYNVVSNADLVDFKNLLDHLEEKMPLED
>ID34503-NO
NPRWEMRGKFVGVR
>ID34556-NO
NRLSRCIPSGDLCFPSDHIQCCSAKCAFVCL
>ID34572-NO
NRPYSFGL
>ID34589-ABP_pos
NRWWQGVVPTVSYECRMNSWQHVFF
>ID34590-NO
NRWYCNSAAGGVGGAAGCVLAGYVGEAKENIAGEVRKGWGMAGGFTHNKA
>ID34597-NO
NSEDGSPYPGPGQQPNCCKWPIVTCCN
>ID34600-NO

NSELINAILGSPTLFGEV
>ID34604-NO
NSELINSILGLPKVMNDA
>ID34605-NO
NSELINSLLGIP
>ID34609-NO
NSELINSLLGIPKVMTDA
>ID34610-NO
NSELINSLLGISRLMNEA
>ID34611-NO
NSELINSLLGLPKNMNNA
>ID34642-NO
NSVVLGKKQRFHSWG
>ID34654-NO
NTFYCCELCCNPACAGCY
>ID34680-NO
NVGKEVGMDVVRTGIDIAGCKIKGEC
>ID34704-NO
NVSCTTSKECWSVCQRLHNTSRGKCMNKKCRCYS
>ID34721-NO
NWTPQAMLYLKGT
>ID34722-ABP_both
NWVKEWIKYLKSLFSHAFEVVKT
>ID34747-NO
NYWRQCAFNAVSCF
>ID34819-ABP_pos
PEATKCFQWQRNMRKVR
>ID34839-NO
PFDRISSSAFSDF
>ID34868-NO
PGCCNNPACGKNRC
>ID34894-NO
PGYLAFPRM
>ID34920-NO
PIKVFPTDAEEESSEIFPLEL
>ID34923-NO
PIKVYPNGVEEESAESFPMEL
>ID34926-NO
PIKVYTNGVEEESAESLPAEM
>ID34997-NO
PLGFLSQDHSV
>ID35021-ABP_both
PMLRVRLASHLRKLRKRLLR
>ID35062-ABP_both
PPPPPPPPP
>ID35065-ABP_both
PPPPPPPPPPPP
>ID35066-ABP_both
PPPPPPPPPPPPP
>ID35077-ABP_neg
PPTQNPSMAPPTQNPYGQPMTPPTQNPYGQPMAPP

>ID35084-NO
PQCCSHPACNVDHPEIC
>ID35122-ABP_both
PRIKKIVQKKLAGDE
>ID35139-ABP_both
PRPLPFPRPGPRPIPRPLPFPRPGPRPI
>ID35225-ABP_both
PTTKTYFPHF
>ID35233-NO
PVKVYPNGAEDELAEAFPLEF
>ID35234-NO
PVKVYPNGAEGESAEAFPLEF
>ID35239-NO
PVKVYTSNGVEEESAEVFPGEM
>ID35243-NO
PVPDSSFLRDP
>ID35274-NO
PYDRISNSAFSDF
>ID35298-NO
QAEATRQAAAQEERLADLASDLLLQYLLQGGARQRGLG
>ID35338-NO
QASPNIFGQWM
>ID35343-NO
QCADLGEECYTRFCCPGLRCKDLQVPTCLLA
>ID35346-NO
QCCDSNSCEYPKCLCCN
>ID35356-NO
QCCWYFDISCCITV
>ID35388-NO
QCTPVGGYCFDHHHCCSNHCIKSIGRCVAH
>ID35416-NO
QDLDHVFLR
>ID35439-NO
QDVDHVFLRF
>ID35488-ABP_both
QFPVGRVHRLLRK
>ID35518-NO
QGGWPRPGPEIPP
>ID35533-NO
QGPWMEEEEEAYGWMDF
>ID35549-NO
QGWCCKENIACCI
>ID35553-NO
QGYKGPYTRPILRPYVRPVVSYNACTLSCRGITTTQARSCSTRLGRCCHV
>ID35581-NO
QHWSYGLQPG
>ID35591-NO
QHWSYGLSPG
>ID35627-NO
QIRYHQCYFNPISCF
>ID35628-NO

QIRYRQCYFNPISCF
>ID35710-NO
QKLCQRPSGTWSGVCGNNNACKNQCIRLEKARHGSCNYVFPAHKCICYFP
>ID35735-NO
QKSLVPSVITTCCGYDPGTMCPPCRCTNSC
>ID35743-NO
QLALQLALQALQAALQLA
>ID35753-NO
QLGLQGPPQLVADLSKKQGPWMEEEEAAYGWMDF
>ID35775-NO
QLNFSTGW
>ID35776-NO
QLNFSTGWGRRYADPNADPMAFLTKLIQIEARKLSGCSN
>ID35778-NO
QLNFTPNWGT
>ID35800-NO
QLTFSSGWGN
>ID35802-NO
QLTFTPGW
>ID35804-NO
QLTFTPNW
>ID35805-NO
QLTFTPNWGT
>ID35813-NO
QLYAFPRV
>ID35817-NO
QMIFGAPMWALGHLM
>ID35851-NO
QNSAAAFAAWA
>ID35908-NO
QPRNFLRF
>ID35909-ABP_both
QPRPPHPRL
>ID35983-NO
QRGSRGQRCGPGEVFNQCGSACPRVCGRPPAQACTLQCVSGCFCRRGYIR
>ID36035-NO
QSEEGGSNATKKPYIL
>ID36039-NO
QSHISKARRPYIL
>ID36069-NO
QSVPTFTPRL
>ID36103-NO
QTSFTPRL
>ID36108-NO
QVAQMHVWRAVNHDRNHGTGSGRHGRFLIRNRYRYGGGHLSDA
>ID36109-NO
QVDRLGGFQVHGW
>ID36135-NO
QVNFTPSW
>ID36158-NO
QVYKGGYTRPVPRPPFVRPLPGGPIGPYNGCPVSCRGISFSQARSCCSRL

>ID36267-NO
RCCISPACHDECICCID
>ID36285-NO
RCCTGKKGSCSGRACKNLKCCA
>ID36286-NO
RCCTWQECDGNCHCCQ
>ID36288-NO
RCCVHPACHDDCICCID
>ID36293-NO
RCHFVICTTDCRRNSPGTYGECVKKEKGKECVCKS
>ID36311-ABP_both
RCPGRTRQIGTIFPGRIKCRSW
>ID36346-NO
RDRNFLRF
>ID36359-NO
RDWERREFERRQNELRREQEQRREEL
>ID36369-NO
RECKTESNTFPGICITKPPCRKACISEKFTDGHCRGFRRRCLCTKPC
>ID36404-ABP_both
RFCFKGTPCG
>ID36443-NO
RFRPPIRRPPIRPPFYPPFRPP
>ID36451-ABP_both
RFRRLRKKTRKRLKKI
>ID36498-NO
RGEPAYNGRFL
>ID36499-NO
RGEPAYQGRFL
>ID36526-ABP_both
RGLRRLGKKIAHGVKKYGPTVLRIIRIAG
>ID36537-NO
RGPAESSGESAHPLE
>ID36608-NO
RHGCCKGPKGCSSRECRPQHCC
>ID36628-ABP_both
RHRHRHRHRH
>ID36677-ABP_both
RIGSILGALAKGLPTLISWIKNR
>ID36679-NO
RIGSILGALASKLPTLISWIKNR
>ID36690-NO
RIIDLLWRVRRPWKPKFVTVWVR
>ID36703-NO
RIKIGLFDQLSRL
>ID36707-ABP_both
RIKRFWPVVIRTV
>ID36708-ABP_both
RIKRFWPVVIRTVVA
>ID36742-ABP_both
RIRFPWPWRWPWWPRFRG
>ID36743-ABP_both

RIRGGRAAVLNALGKEEQIGRASNSGRKCARKKK
>ID36758-NO
RISFKKGKGSWIKNGLIKGIKGLGKEIGLDVIRTGIDIAGCKIKGEC
>ID36935-NO
RKKRRRESRKKRRRESC
>ID37247-ABP_pos
RLKLLLLLRL
>ID37301-ABP_both
RLPRPVYIPQPRPPHPRL
>ID37346-ABP_pos
RLWDIVRRWVGWLC
>ID37351-ABP_both
RLWLAIGRG
>ID37358-NO
RLWMRAYSPTTRRYG
>ID37359-NO
RLWMRWASPTTRRYG
>ID37360-NO
RLWMRWYAPTTRRYG
>ID37368-NO
RLWMRWYSPWTRRWG
>ID37374-ABP_both
RLWRRWRRWLR
>ID37386-NO
RMKQIEDKIEEIESKPKKIENEIARIKKLLQLTVWGPKQLQARIL
>ID37461-NO
RPCCYIGWCSRRCNCGGYRP
>ID37491-ABP_both
RPKHPIKHQGLPQEVLNENLLRFF
>ID37522-ABP_both
RPPQFTRAQWFAIQHISLNPPRSTIAMRAINNYRWRSKNQNTFL
>ID37525-ABP_neg
RPPYLPRPRPPPFFPPRLPPRIPPGFPPRFPPRFP
>ID37533-NO
RPRGRRGSRPSGAERRRRRAAAA
>ID37549-ABP_both
RPVRPVYIPQPRPPHPRL
>ID37579-NO
RQIKIAFQNRRMKWKK
>ID37582-NO
RQIKIFFQNRRMKWKK
>ID37586-NO
RQIKIWAQNRRMKWKK
>ID37587-NO
RQIKIWFANRRMKWKK
>ID37594-NO
RQIKIWFQNR
>ID37600-NO
RQIKIWFQNRRMK
>ID37612-NO
RQLLSGIDQEQNNLTRLIEAQIHELQK

>ID37614-NO
RQLLSGIVQQQNNLLRAIEAQQHLL
>ID37632-ABP_both
RQRWLWLW
>ID37827-NO
RRCFPLGTFCSRYLPCCSGMCCSGWCTRRCAPRF
>ID37894-ABP_neg
RRIRPRPPRLPRPC
>ID37898-ABP_both
RRIRPRPPRLPRPRPRPLPFPRP
>ID37947-ABP_both
RRLRPRRPRLPRPRPRPRPRPR
>ID37950-ABP_both
RRLRRLLRRLLRRLRR
>ID37977-NO
RRPKGRGKRRREKQRPSDKPRR
>ID38055-NO
RRRRRRRRRK
>ID38068-ABP_both
RRRRRRRWWWWWW
>ID38074-ABP_both
RRRRSVQWCA
>ID38091-ABP_both
RRRTPSPRRRRSQSPRRRRS
>ID38344-ABP_both
RRWRRWRRWRR
>ID38392-ABP_both
RRWWRRWRRW
>ID38417-NO
RSAEGLGRMG
>ID38418-NO
RSAEGLGRMGRL
>ID38481-NO
RSTQGYGRMDRILAA
>ID38486-NO
RSVEGASRMEKL
>ID38491-NO
RSVEGASRMEKLLSSSN
>ID38493-NO
RSVEGASRMEKLLT
>ID38495-NO
RSVEGSSRMERLLSSGSSSSEPLSFLSQDQSVS
>ID38497-NO
RSVEGVSRMEKLL
>ID38502-NO
RSVESSGSSSSEPLSFLSQDQSVN
>ID38541-ABP_both
RTHGLLKRIKTLL
>ID38566-ABP_both
RTVVAGYNLYRAIKKK
>ID38596-ABP_both

RVCSAIPLPICR
>ID38616-NO
RVINDDCPNLIGNRD
>ID38624-NO
RVISLPAGLSPLR
>ID38659-NO
RVPWTPSPRL
>ID38667-ABP_both
RVRRPVYIPQPRPPHPRL
>ID38689-ABP_both
RVVRPVVQVVKQKVR
>ID38691-ABP_both
RVVRQWPIGRVVRRVVRRVVRL
>ID38724-ABP_both
RWCVYAYSRVRGVLVRYRRCW
>ID38725-ABP_both
RWCVYAYVRGRGVLVRYRRCW
>ID38726-ABP_both
RWCVYAYVRRRGVLVRYRRCW
>ID38727-ABP_both
RWCVYAYVRSRGVLVRYRRCW
>ID38728-ABP_both
RWCVYAYVRVRGGLVRYRRCW
>ID38729-ABP_both
RWCVYAYVRVRGRLVRYRRCW
>ID38730-ABP_both
RWCVYAYVRVRGSLVRYRRCW
>ID38769-NO
RWKIFKKIERVGQNVRDGIIKAGKAIQVLGTAKALGK
>ID38776-NO
RWKLFKKIEKVGRNVRDGLIKAGPAIAVIGQAKSLGK
>ID38829-ABP_pos
RWRLLLLKKH
>ID38830-ABP_pos
RWRLLLLKKR
>ID38833-ABP_pos
RWRLLLLLKRH
>ID38839-ABP_both
RWRRLLKKLHHLLHSATFIKHFIHRF
>ID38852-ABP_both
RWRWRWFSGGFIKHFIHRF
>ID39038-ABP_both
RWWLRRIW
>ID39055-ABP_both
RWWRWWRRWWW
>ID39121-NO
SAAAPLIRF
>ID39129-NO
SADPNFLRF
>ID39139-NO
SAEPNFLRF

>ID39156-NO
SALRGCWTKSYPPKPCFGK
>ID39160-NO
SALVGCWTKSYPPNPCFGR
>ID39164-NO
SANQDFMRF
>ID39172-NO
SAPGQDFMRF
>ID39174-NO
SAPMASDYGNQFQMYNRLIDA
>ID39178-NO
SAPSDFSRDIMSFG
>ID39179-NO
SAPSQDFMRF
>ID39203-ABP_both
SAVLRHLRRFLLRKHRKH
>ID39218-NO
SCCNAGFCRFGCTPCCY
>ID39223-NO
SCCPQEFLCCLYLV
>ID39228-NO
SCCSDSDCNANHPDMCS
>ID39265-ABP_both
SCLPKEEQIGKSTRGRKCRRKR
>ID39277-NO
SCNTATCVTHRLAGLLSRSGGMVKSNFVPTDVGSEAF
>ID39280-NO
SCNTATCVTHRLAGLLSRSGGVVKSNFVPTNVGSQAF
>ID39283-NO
SCRNEGAMCSFGFQCCKKKCCMSHCTDFCRNP
>ID39343-NO
SDDYGHMRF
>ID39411-NO
SDPEVPGMWFGPRL
>ID39417-NO
SDPKIGDGCFGLPLDHIGSVSGLGCNRPVQNRPKK
>ID39446-NO
SDYLQLART
>ID39457-NO
SEDRSTGNSLRDSSSFFPARF
>ID39458-NO
SEEPPISLDLTFHLLRELEMARAEQLAQQAHSNRKLMENF
>ID39468-NO
SEFLKQYLGMSPR
>ID39488-NO
SEQCCHLAACRFGCTPCCW
>ID39516-ABP_both
SFGAKNAVKNGLQKLRNQCQANNGQGPFCDIFKKNP
>ID39535-NO
SFLSKFKDIALDVAKNAGKGVLTTLARKIDGSC
>ID39541-NO

SFNAASPLLANGHLHRGSELGLTDLYDLQDWSSD
>ID39547-NO
SFQPQPLIYP
>ID39551-NO
SFRNGVGSGAKKTSFRRAKQ
>ID39553-NO
SFRNGVGTGMKKTSFQRAKS
>ID39593-ABP_both
SGGYCGGWHRLRCTSYRSG
>ID39604-NO
SGKLSFLEDEM
>ID39625-NO
SGPYAFGL
>ID39631-NO
SGRGKQGGKARAKAKTRSSRAGLQFPVGRVHRLLRKGC
>ID39653-NO
SGSLSTFFRLFNRSFTQALG
>ID39654-NO
SGSLSTFFRLFNRSFTQALGA
>ID39655-NO
SGSLSTFFRLFNRSFTQALGV
>ID39702-NO
SICCSFPDPWGGFCCEDHCSYIGKPGGQCSDKKVCTCN
>ID39715-NO
SIGFDGLNDPDIVAR
>ID39722-NO
SIIDSVKTFCCSTFNLGICCS
>ID39726-ABP_both
SIITMTKEAKLGQLWKQIACRLYNTC
>ID39751-NO
SIRDKIKTIAIDLAKGAGTGVLKTLICKLDKSC
>ID39775-ABP_both
SKCRQWQSKIRRTNP
>ID39805-NO
SKNSQIGKSTSSISKCVFSFFKKC
>ID39831-ABP_both
SKVGRHGRRFGHRAHRKL
>ID39837-ABP_both
SKVLRHLRRFLHRAHRKL
>ID39843-ABP_both
SKVWRHWRRFWHRAHRKK
>ID39862-NO
SLAYDDKSFENVEFTPRL
>ID39873-NO
SLEEILDEI
>ID39890-ABP_both
SLGTPDHYHGGRHSISRGSQSTGPTHPGYNRRNAR
>ID39912-NO
SLLSLFRKLIT
>ID39913-NO
SLLSLIRKLIW

>ID39933-NO
SLQPGAPNVNNKDQPWQVSPHISRDDSGNTRTDINVQRHGENNDFEAGWS
>ID39938-NO
SLRGCWTKSFPPQPCLGKR
>ID39941-NO
SLRGPAESDGESAHPLE
>ID39949-NO
SLSAASAPLVETSTPLRL
>ID39961-NO
SLTFEEVKDWGPKIKMNTPAVNKMPPSAANLPLRF
>ID39975-NO
SMDDLDDPRLMTMSF
>ID39989-ABP_both
SMLSVLKKLGKVGLGFVACKINKQC
>ID39993-ABP_both
SMLSVLKNLGKVGLGFVACKIKKQC
>ID39994-ABP_both
SMLSVLKNLGKVGLGFVACKINKKC
>ID40025-NO
SNGRNAAADAKASQRIAPFLRDYCCRRHACTLICG
>ID40045-NO
SNKRKNAAMLDMIAQHAIRGCCSDPRCKHQCG
>ID40066-NO
SPAFDDEHNDNFLRL
>ID40067-NO
SPAFNSWG
>ID40069-NO
SPAISPAYQFENAFGLSEALERA
>ID40073-NO
SPALDDEHNDNFLRF
>ID40076-NO
SPANAQITRKRHKINSFVGLM
>ID40077-NO
SPANSVWS
>ID40079-NO
SPAQWQRANGLW
>ID40106-NO
SPKTMRDSGCFGRRLDRIGSLSGLGCNVLRRY
>ID40119-NO
SPMEPSAALAVEHGTTHPLE
>ID40140-ABP_both
SPRRRRSQSPRRRR
>ID40145-NO
SPSAKWMRF
>ID40148-NO
SPSAVPLIRF
>ID40151-NO
SPSLRLRF
>ID40153-NO
SPSQDFMRF
>ID40160-NO

SPVDYDRPIMAF
>ID40168-NO
SPWSPCSTSCGLGVSTRI
>ID40172-NO
SPWSSASVTAGDGVITRI
>ID40199-NO
SQPNFLRF
>ID40225-NO
SRCFPPGIYCTPYLPCCWGICCGTCRNVCHLRF
>ID40228-NO
SRDLICLCRKRRCNRGELFYGTCAGPFLRCCRRRR
>ID40264-NO
SRRHHARSKAKRSRHH
>ID40265-NO
SRRHHCRAKAKRSRHH
>ID40266-NO
SRRHHCRSAAKRSRHH
>ID40281-NO
SRVVKCIGFRPGSPDSRQSC
>ID40308-NO
SSEDMDRLGFGFN
>ID40309-NO
SSEDMDRLIGFG
>ID40319-NO
SSGGGDGSGMWFGPRL
>ID40331-NO
SSIQSQPQAFT
>ID40332-NO
SSKMMRDSRCFGRRLDRIGSLSGLGCNVLRRH
>ID40383-NO
SSPEILDTLVSELLLKESTDTLPQSRYDPSLW
>ID40393-NO
SSRFVGGSRY
>ID40399-NO
SSSMYDRDIMSFG
>ID40421-NO
STDWSSLRSAW
>ID40426-ABP_neg
STIGKLPKWIIKTVNKFTKK
>ID40437-NO
STNITVTLKKFPL
>ID40457-NO
SVAALAAQGLLNAPK
>ID40466-NO
SVAVAGAVIEGASLTFNVLQ
>ID40478-NO
SVDQDLGPEVPPENVLGALL
>ID40480-NO
SVDQDLGPEVPPENVLGALLRVKRLENPSPQAPARRLLPP
>ID40486-ABP_both
SVETLASQEHLSSLPMDSQETLLRGT

>ID40523-NO
SVNPYLQGKRLDNVV
>ID40546-NO
SVSQLNQYAGFDTLGGMGLG
>ID40549-NO
SVTFQELKDWGAKKDIKMSPAPANKVPHSAANLPLRF
>ID40646-NO
SYAMEHFRWGKPV
>ID40649-NO
SYFDEKKSVPGVLRF
>ID40676-NO
SYSMEHFRWGKPVGKKRRPIKVFPTDAEEESSEIFPLEL
>ID40678-NO
SYSMEHFRWGKPVGKKRRPVKVYANGAEEESAEAFPLEF
>ID40681-NO
SYSMEHFRWGKPVGKKRRPVKVYPNGAEDELAEAFPLEF
>ID40686-NO
SYSMEHFRWGKPVGRKRRPVKVYPNGVEEESAESYPAEI
>ID40689-ABP_both
SYVGDCGSNGGSCVSSYCPYGNRLNYFCPLGRTCCRHAYV
>ID40700-NO
TAFGLRKCCKKHHGCHPC
>ID40709-NO
TANNKATDLMALTVRGCCGNPSCSIHIPYVCN
>ID40847-NO
TEPAYQRFL
>ID40876-NO
TFFYGGSRGKRNNFKTEEY
>ID40877-NO
TFFYGGSRGKRNNFRTEEY
>ID40895-NO
TGDMSGEGKGMWFGPRL
>ID40912-NO
TGWNKFQGSW
>ID40929-ABP_both
THVFRLKKWMQKVIDRFGG
>ID40956-NO
TKAARITPKDVIDVRSVTTEINT
>ID41008-NO
TKRRITPKDVIDVRSVTTKINT
>ID41091-NO
TNEIVEEQYAPQSLATLESVFQELGKLTGPNNQ
>ID41094-NO
TNEIVEEQYTPQSL
>ID41123-NO
TPAQSSDFMRF
>ID41124-NO
TPASGFFGMR
>ID41140-NO
TPGYSHSFV
>ID41143-ABP_both

TPIESHKVEKRKSNK
>ID41153-NO
TPMQRSSMVRF
>ID41162-NO
TPPQPSDNFIRF
>ID41166-NO
TPQNWNKLNSLWG
>ID41167-NO
TPQQDFMRF
>ID41184-NO
TPTAFYGVR
>ID41219-NO
TRGCKSKGSFCWNGIECCGGNCFFACIY
>ID41250-NO
TRQARRNRRRRWRERQRAAAAC
>ID41263-NO
TRSGGACNSHNQCCDDFCSTATSTCI
>ID41407-NO
TVKCGGCNRKCCPGGCRSGKCINGKCQCY
>ID41439-NO
TVWGFRPTKPKPKPPG
>ID41462-ABP_neg
TWLKKARWKKAKPP
>ID41463-ABP_neg
TWLKKRAWKKAKPP
>ID41464-ABP_neg
TWLKKRRAKKAKPP
>ID41465-ABP_neg
TWLKKRRFKKAKPP
>ID41499-NO
VADKRPHILHEKKSIPY
>ID41513-ABP_both
VAGYNLYRAIKKK
>ID41526-NO
VALPPGFTPFR
>ID41537-NO
VAPFPEVF
>ID41540-ABP_both
VARGWGNGCGLFGKGG
>ID41605-NO
VDELYPVEPEEEANGGEILT
>ID41619-ABP_both
VDKPPYLPRPRPPRRIYN
>ID41620-ABP_both
VDKPPYLPRPRPPRRIYNA
>ID41622-ABP_both
VDKPPYLPRPRPPRRIYNN
>ID41675-NO
VEVQVRDKGKGIYGLSPLRQPTP
>ID41676-NO
VEVQVREVGIQGLSPLRQPAP

>ID41678-NO
VEWICEDCSNIFR
>ID41684-NO
VFCRSNGQQCTSDGQCCYGKCMTAFMGKICMR
>ID41700-NO
VFKCSYRLGSPISRCN
>ID41702-NO
VFKCYKPDSRGFQVCE
>ID41716-ABP_neg
VFQFLGKIIHHVGNFVAGFSAVF
>ID41750-NO
VGCEECPMHCKGKNAKPTCDDGVCNCNV
>ID41753-NO
VGCEECPMHCKGKNAVPTCDNGVCNCNA
>ID41758-NO
VGFYANRY
>ID41975-ABP_both
VKLVYPLKVKLYP
>ID41977-ABP_both
VKLYPKVKLYP
>ID41993-ABP_both
VKRFKKFFRKFKKSV
>ID42026-ABP_both
VKWRWKKWRWKWKWKV
>ID42084-ABP_both
VLNENLAA
>ID42089-ABP_both
VLNENLLK
>ID42096-ABP_neg
VLPFVKKLLRGLF
>ID42101-ABP_neg
VLPIVKKLLKGLF
>ID42156-ABP_both
VNFLLHKIYGNIRDS
>ID42165-NO
VNMEAGTRSHFPSLPQRF
>ID42231-NO
VPNDWAHFRGSW
>ID42240-NO
VPPGFTPFRLT
>ID42249-NO
VPSAGDMMVRF
>ID42253-NO
VPSFGHSFV
>ID42281-ABP_both
VQLRIRVCVIRR
>ID42289-ABP_both
VQWRIRIAVIRA
>ID42570-NO
VSCDLLSGLGWGHSICAGHCLAISWRYRGGYCNDQGVCVCRT
>ID42581-NO

VSCTGSKDCYAPCRKQTGCPNAKCINKSCKCAGC
>ID42590-NO
VSEACEESCEEEEKHCCHENNGVYTCLRYCW
>ID42644-NO
VTCDLLSFKGQVNDSACAANCLSLGKAGGHCEKVGCICRKTSFKDLWDKR
>ID42653-ABP_both
VTCDVLSFEAKGIAVNHSAAALHCIALRKKGGSCQNGVAVCRN
>ID42667-NO
VTEECEENCEEEEKHCCNTNNGPSCAPQCF
>ID42698-ABP_both
VVAGYNLYRAIKKK
>ID42702-NO
VVAPFPEVF
>ID42722-NO
VVGGDECNINEHRSLVAIF
>ID42729-NO
VVIGQRCYRSPDCYSACKALVGKATGKCTNGRCDC
>ID42738-NO
VVKCSYREGSADSRCK
>ID42889-NO
VYPFPGPI
>ID42920-NO
WCKQSGEMCNLLDQNCCEGYCIVLVCT
>ID42939-NO
WDVDQCIYYCLNGVVGYSYTECQTMCT
>ID42942-NO
WDVNDCIHFCLIGVVGRSYTECHTMCT
>ID42948-NO
WEEWDKKIEEYTKKIEELIKKSEEQQKKN
>ID42971-NO
WFQNRRMKWKK
>ID42976-ABP_both
WFWKWWRRRRR
>ID43002-ABP_both
WGRAFSAGVHRLANGGNG
>ID43004-ABP_both
WGRAFSRGVRRLARGGRG
>ID43020-ABP_both
WGWRDIVRAIRKVAAPVLST
>ID43021-ABP_both
WGWRDIVRGARKVAAPVLST
>ID43022-ABP_both
WGWRDIVRGIAKVAAPVLST
>ID43023-ABP_both
WGWRDIVRGIRAVAAPVLST
>ID43060-NO
WIIFRIAAYHKK
>ID43077-ABP_both
WIQPKTAVIPYVRYL
>ID43078-ABP_both
WIQPKTKAIPYVRYL

>ID43079-ABP_both
WIQPKTKVAPYVRYL
>ID43100-ABP_both
WIRYKWRRKKVRYWTGP
>ID43106-ABP_both
WIVYKWRRKVVRYWTGKR
>ID43116-NO
WKEPAYQRFL
>ID43192-NO
WLEPAYQRFL
>ID43210-ABP_both
WLNALKKVFQGIHEAIKLIWNWVQ
>ID43237-NO
WLWDENEK
>ID43247-NO
WLWSEQSK
>ID43249-ABP_pos
WLWWLRWL
>ID43261-NO
WMEWDREINNYTSLIHSLIEESQNQQEKN
>ID43264-NO
WMEWDRKIEEYTKKIKKLIEESQEQQEKNEKELK
>ID43266-ABP_both
WMLKKFRGMF
>ID43288-NO
WNSLKIDNLAV
>ID43294-NO
WPEAAELMMEVDP
>ID43298-ABP_both
WPKRLTKAHWFEIQHIQPSPLQCNRAMSGINNYTQHCKHQNTFLH
>ID43313-ABP_both
WPSPGRPRPFPGRPKPIFRPR
>ID43324-NO
WQEPAYQRFL
>ID43355-NO
WRATRGCAATCPEAKPRETVECCATDKCNL
>ID43375-ABP_both
WRHWRRFWHR
>ID43384-ABP_both
WRKLWRPGLKRWLK
>ID43416-ABP_pos
WRRWIRWL
>ID43527-ABP_both
WWKKWWKK
>ID43536-ABP_pos
WWLLWLRWL
>ID43543-ABP_pos
WWLWWLRWL
>ID43545-ABP_pos
WWLWWLWWL
>ID43554-ABP_both

WWRRRRRRRR
>ID43569-ABP_both
WWVFRVVYPRIVYRRVRWTGPKK
>ID43598-ABP_both
WWWRRRRRRRR
>ID44280-NO
YADAIFTNSYRKVLGQLSARKLLQDIMNRQQGERNQGAKVRL
>ID44286-NO
YADPNADPMAFLYKLIQIEARKLAGCSN
>ID44328-NO
YCKFEWATFAKSC
>ID44343-NO
YCQKWMWTCDEERKCCEGLVCRLWCKKKIEW
>ID44361-NO
YDCEPPGNFCGMIKIGPPCCSGWCFFACA
>ID44388-NO
YECYSTGTFCGVNGGLCCSNLCLFFVCLFS
>ID44396-NO
YEREARRAARR
>ID44404-NO
YFLFRPRN
>ID44419-NO
YGCSNAGAFCGIHPGLCCSELCLVWCT
>ID44431-NO
YGGFLRQFKVVT
>ID44436-NO
YGGFMKPYTKQSHKPLITLLKHITLKNEQ
>ID44438-NO
YGGFMKSWDERSQKPLLTLFKNVMIKDGHEKKGQ
>ID44444-NO
YGGFMTPERSQTPLMTLFKNAIIKNAHKKGQ
>ID44446-NO
YGGFMTPERSQTPLMTLFKNAIIKNTHKKGL
>ID44448-NO
YGGFMTSEKSQTPLVTLFKNAIIKNAYKKGH
>ID44477-NO
YGRRARRRRRR
>ID44508-NO
YINWKSPDAIYHLAK
>ID44519-NO
YIQQARKAPSGRMSIIKNLQNLDPSHRISDRDYMGWMDF
>ID44595-NO
YKKPPFNGSIF
>ID44602-ABP_both
YKLLKLLLPKLKGLLPKL
>ID44604-ABP_both
YKLLKLLLPKLKPLLIKL
>ID44616-NO
YKPRSFAMGF
>ID44623-NO
YKRAARRAARR

>ID44625-NO
YKRCHIKGGHCFPKEKICIPPSSDFGKMDCPWRRKCCKKGSG
>ID44627-NO
YKRCHIKGGHCFPKGKICIPPSSDFGKMDCPWRRKCCKKGSG
>ID44628-NO
YKRCHKKGGHCFPKTVICLPPSSDFGKMDCRWKWKCCKKGSVNNAISI
>ID44630-NO
YKRKARRAARR
>ID44652-NO
YLDVNQIASYLLCLGEGAVFNGRKTCQIGCRAACQQPGCGGYKECEQIPN
>ID44702-NO
YPASYDGDFDALDDLDDLDLDDLLDLEPADLVLLDMWANMLDSQDFEDFE
>ID44703-ABP_both
YPCKLNLKLGKVPFH
>ID44717-NO
YPFPGPIP
>ID44748-NO
YPPQPESPGGNASPEDWAKYHAAVRHYVNLITRQRY
>ID44760-NO
YPSKPDNPGEGAPAEDLAKYYSALRHYINLITRQRY
>ID44771-NO
YPVKPENPGDDAPAEELAKYYSALRHYINLITRQRY
>ID44791-NO
YQEPVLGPVRGPF
>ID44854-NO
YSDRNYLRF

Final pool
ISA_NO_PCR_N2
Z3_NO_PLR_I50
Gs(U)_NO_PLR_I50
IP_NO_PRT_I50
IP_NO_PLR_Q2
Gs(U)_NO_PRT_I50
IP_NO_AHR_I50
Z3_NO_PRT_N1
IP_NO_PRT_Q1
Z2_NO_PLR_I50
Z3_NO_PRT_CV
Z3_NO_PLR_Q2
Z2_NO_AHR_I50
IP_NO_PRT_RA
ISA_NO_PLR_Q3
ECI_NO_AHR_I50
Z3_NO_AHR_I50
Gw(U)_NO_PCR_SI5
IP_NO_AHR_RA
Z1_NO_PCR_V
Z3_NO_ALR_N3
Z1_NO_NPR_N1
Gw(U)_NO_PCR_MX

Z3_NO_AHR_RA
ISA_NO_PRT_Q1
IP_NO_PLR_N3
Mw_NO_AHR_I50
ECI_NO_PLR_MN
ECI_NO_PLR_TI5
IP_NO_AHR_MX
Z3_NO_PLR_SI5
Gw(U)_NO_AHR_I50
ECI_NO_PCR_MN
IP_NO_AHR_DE
Gs(U)_NO_AHR_P2
Gs(U)_NO_BSR_N1
Mw_NO_PLR_Q2
Z2_NO_RTR_P3
Z2_NO_NPR_SI5
Mw_NO_RTR_P3
ECI_NO_PLR_Q3
Z2_NO_PLR_MI5
ISA_NO_PLR_N2
Z2_NO_PRT_RA
Z3_NO_NPR_I50
ECI_NO_PLR_CV
Z2_NO_RTR_I50
Mw_NO_PRT_I50
HP_NO_PLR_Ar
ECI_NO_RTR_I50
Gw(U)_NO_PCR_P3
Z1_NO_PRT_MX
Z2_NO_AHR_P2
Mw_NO_PRT_RA
ISA_NO_AHR_DE
Mw_NO_PRT_Q2
Z3_NO_PLR_P2
Gs(U)_NO_PCR_Q1
ISA_NO_PLR_CV
Z3_NO_PLR_N2
Z3_NO_RTR_I50
Z1_NO_PRT_SI5
Z1_NO_NPR_I50
Z1_NO_PRT_P2
Z1_NO_PLR_N1
ECI_NO_ALR_P3
ISA_NO_NPR_SI5
Mw_NO_RTR_RA
Z2_NO_PCR_Q3
ISA_NO_PLR_V
ISA_NO_NPR_I50
Z1_NO_AHR_Q2
Z1_NO_PRT_Q1
Mw_NO_ALR_SI5

IP_AC1_PRT_Q2
IP_AC1_AHR_Q1
Z1_AC1_PCR_Q3
Z1_AC1_PCR_MN
ECI_AC1_PCR_Q3
HP_AC1_PCR_MN
Z2_AC1_PCR_MI5
Z1_AC1_PCR_CV
IP_AC1_RTR_Q3
Mw_AC1_PCR_Q2
Mw_AC1_NPR_MX
IP_AC1_RTR_MX
IP_AC1_RTR_Q2
Mw_AC1_PCR_SI5
Mw_AC1_PCR_Q1
IP_AC1_ALR_Q2
ISA_AC1_PCR_SI5
Z2_AC1_PCR_MN
Z2_AC1_PCR_Q2
Gs(U)_AC1_BSR_MN
ISA_AC1_ALR_MX
ISA_AC1_PCR_P3
Gs(U)_AC1_PLR_Q2
ISA_AC1_PRT_M
IP_AC1_ARM_Q1
Z3_AC1_RTR_MN
IP_AC1_BSR_N3
Gs(U)_AC1_PCR_Q1
Z1_AC1_ALR_MN
Gs(U)_AC1_PCR_CV
Z3_AC1_PCR_Ar
Z2_AC1_PCR_CV
Z2_AC1_UFR_Q2
Z2_AC1_PCR_MX
IP_AC1_NPR_MN
HP_AC1_AHR_MN
IP_AC1_BSR_MN
Z2_AC1_PRT_MX
Gw(U)_AC1_PCR_S
Z3_AC1_UCR_N3
HP_AC1_UFR_Q3
IP_AC1_UFR_MN
Z3_AC1_PCR_N2
Z3_AC1_UFR_N3
Mw_AC1_AHR_Q2
ISA_AC1_BSR_Q2
ISA_AC1_UFR_Q3
Mw_AC1_ALR_N3
ECI_AC1_PRT_MX
ISA_AC1_UCR_MX
Z3_AC1_RTR_CV

Gs(U)_AC1_NCR_Q3
ISA_AC1_ARM_Q1
Gw(U)_AC1_PCR_G
Z2_AC1_UCR_Q1
Mw_AC1_ARM_Q3
Z2_AC1_NPR_MN
Z2_AC1_PCR_N3
Z3_AC1_ALR_MN
IP_AC1_ARM_Q3
Gw(U)_AC1_PCR_MN
Z1_AC1_BSR_Q3
Mw_AC1_ALR_Q3
HP_AC1_ALR_Q2
Gs(U)_AC1_PCR_I50
Z3_AC1_UCR_Q1
ECI_AC1_AHR_Q3
Mw_AC1_UCR_Q3
Z1_AC1_AHR_Q3
IP_AC1_ALR_RA
Gw(U)_AC1_AHR_MX
ISA_AC1_NPR_Q3
Z2_AC1_PLR_Q3
Z1_AC1_PCR_N2
Mw_AC1_NCR_MN
W(U)_AC1_UCR_MN
ISA_AC1_UCR_Q3
Z1_AC1_UCR_N3
Mw_AC1_PLR_Q2
Z3_AC1_BSR_Q1
Z1_AC1_BSR_MX
Z2_AC1_ARM_Q1
IP_AC2_PRT_Q1
Z1_AC2_PCR_MN
IP_AC2_AHR_MX
Gs(U)_AC2_AHR_MN
Z1_AC2_PCR_N2
Gs(U)_AC2_PLR_Q1
Z3_AC2_PCR_Q3
HP_AC2_PLR_MN
IP_AC2_NPR_P3
HP_AC2_PLR_MX
IP_AC2_NPR_N3
IP_AC2_PCR_Q1
IP_AC2_ALR_P3
Gs(U)_AC2_PCR_Q1
IP_AC2_RTR_MN
Z1_AC2_PCR_Q3
IP_AC2_RTR_Q2
Z3_AC2_PCR_MI5
IP_AC2_ALR_Q2
Z1_AC2_PCR_P3

ECI_AC2_PCR_SI5
Gs(U)_AC2_AHR_N3
Z3_AC2_PCR_S
Gs(U)_AC2_PCR_CV
IP_AC2_UCR_Q2
Z3_AC2_UCR_MX
Gs(U)_AC2_PRT_P2
Gs(U)_AC2_PLR_Q3
IP_AC2_ARM_Q2
Z2_AC2_PCR_Q1
HP_AC2_PCR_Q3
IP_AC2_BSR_P3
IP_AC2_AHR_MN
Z2_AC2_ALR_MN
IP_AC2_PCR_V
Z1_AC2_PRT_N2
Gs(U)_AC2_UFR_MX
Z1_AC2_PRT_MN
HP_AC2_UCR_Q3
IP_AC2_BSR_Q1
HP_AC2_AHR_Q1
ISA_AC2_ARM_Q1
Mw_AC2_PLR_Q2
Gs(U)_AC2_NCR_Q3
ISA_AC2_RTR_Q1
Z1_AC2_AHR_Q2
HP_AC2_UCR_Q2
ISA_AC2_RTR_Q3
Mw_AC2_RTR_MN
IP_AC2_UFR_MN
ISA_AC2_UCR_MN
Z3_AC2_UFR_Q3
IP_AC2_RTR_MX
IP_AC2_ALR_RA
IP_AC2_BSR_MN
Z2_AC2_PCR_N2
Z3_AC2_UFR_Q1
Z3_AC2_PCR_Ar
Z3_AC2_RTR_N3
Z1_AC2_ARM_Q2
ECI_AC2_ARM_MX
HP_AC2_NCR_P2
Z3_AC2_PLR_MN
ECI_AC2_PLR_Q2
HP_AC2_ALR_Q1
Z1_AC2_UFR_MN
Z1_AC2_AHR_Q3
HP_AC2_ALR_N3
HP_AC2_RTR_Q1
Gs(U)_AC2_UCR_Q2
ECI_AC2_PLR_Q3

Gs(U)_AC2_NCR_I50
Z1_AC2_AHR_N2
Mw_AC2_AHR_Q3
Z2_AC2_AHR_N2
Mw_AC2_UCR_MX
Z1_AC2_UCR_Ar
Z1_AC2_NPR_N3
Z1_AC2_RTR_Q2
ECI_AC2_RTR_RA
ECI_AC2_BSR_MX
Gs(U)_AC2_BSR_Q1
Gs(U)_AC2_ARM_Q3
ISA_AC2_PRT_CV
Z3_AC2_ALR_N3
Gw(U)_AC2_PCR_CV
Z2_AC2_RTR_MN
Z3_ES_PCR_TI5
Z3_ES_PCR_S
Gs(U)_ES_PRT_P2
Gs(U)_ES_PCR_CV
ISA_ES_PCR_CV
HP_ES_PCR_S
IP_ES_PCR_SI5
ISA_ES_PCR_S
HP_ES_PCR_SI5
HP_ES_PLR_Q1
Z2_ES_PCR_SI5
IP_ES_PCR_P3
IP_ES_BSR_Ar
IP_ES_BSR_MN
Z3_ES_AHR_MN
Z1_ES_PCR_CV
ISA_ES_RTR_Q2
Z3_ES_UCR_MX
IP_ES_UCR_Q3
Gs(U)_ES_PLR_Q3
Z3_ES_RTR_Q2
Z3_ES_ARM_Ar
ECI_ES_AHR_I50
Z1_ES_NPR_N3
ECI_ES_RTR_MX
IP_ES_UCR_P3
Z3_ES_RTR_Q1
ISA_ES_PCR_MN
IP_ES_ARM_Q2
Gs(U)_ES_PRT_Q3
Z3_ES_ALR_Q3
ISA_ES_RTR_MN
Z1_ES_PCR_Q1
Z3_ES_ARM_N1
ISA_ES_PCR_Q1

Z2_ES_PLR_Ar
Z1_ES_AHR_P2
Gs(U)_ES_ALR_MX

Section S7. List (FASTA format) of all the peptides in the validation dataset.

>ID00054-ABP_pos
FLPFIAGVAAKFLPKIFCAISKKC
>ID00620-ABP_both
FLPIIAGIAAKVFPKIFCAISKKC
>ID00678-ABP_both
GILGNIVGMGKQVVCGLSGLC
>ID00681-ABP_both
GILSSFKGVAKGVAKNLAGKLLDELKCKITGC
>ID00746-ABP_both
GVFTLLKGATQLIGKTLGKELGKTGLELMACKITNQC
>ID00970-ABP_both
FLPIVGKLLSGLSGLL
>ID00976-ABP_both
FLPLLASLFSRLL
>ID01023-ABP_both
GILSTVFKAGKGIVCGLSGLC
>ID01085-ABP_both
GPLSCRRNGGVCIPIRCPGPMRQIGTCFGRPVKCCRSW
>ID01095-ABP_both
GVLGAVKDLLIGAGKSAAQSVLKTLSCKLSNDC
>ID01100-ABP_both
GWLRKLGKKIERIGQHTRDASIQVLGIAQQAANVAATAR
>ID01332-ABP_both
GFGSFLGKALKAALKIGADVLGGAPQQ
>ID01365-ABP_both
GILSNVLGMGKKIVCGLSGLC
>ID01441-ABP_both
GVVDILKGAGKDLLAHALSKLSEKV
>ID01560-ABP_both
RGGRLCYCRPRFCVCVGR
>ID01610-ABP_both
SVLGTVKDLLIGAGKSAAQSVLTTLSCKLSNSC
>ID01640-ABP_pos
ACYCRIPACLAGERRYGTCFYRRRVWAFCC
>ID01725-ABP_both
FLPAVLRVAAKVVPTVFCLISKKC
>ID01784-ABP_both
GLGSLLGKAFKFGLKTVGKMMAGAPREQ
>ID01801-NO
GLPVCGETCFGGTCNTPGCICDPWPVCTRN
>ID01917-ABP_both
LPFVAGVAAEMMQHVYCAASKKC
>ID01960-ABP_both
QGVRNFVTCRINRGFCVPIRCPGHRRQIGTCLAPQIKCCR
>ID01961-ABP_both
QGVRNHVTCRINRGFCVPIRCPGRTRQIGTCFGPRIKCCRSW
>ID02001-ABP_both
RTCRCRFGRCFRRESYSGSCNINGRISSLCCR
>ID02002-ABP_both

RWKIFKKIEKMGRNIRDGIVKAGPAIEVLGSAKAIGK
>ID02144-ABP_both
FLSLIPHAINAVSAIAKHFG
>ID02159-ABP_both
FWGKLWEGVKNAI
>ID02169-ABP_neg
GFKDWIKGAAKKLIKTVASSIANE
>ID02187-ABP_both
GIFSKLAGKKIKNLLISGLKNIGKEVGMDVVRTGIDIAGCKIKGEC
>ID02195-ABP_both
GILSGILGVGKMLVCGLSGLC
>ID02200-NO
GIPCGESCVWIPCISSAIGCSCKNKVCYRN
>ID02316-ABP_both
LLPIVGKLLSGLL
>ID02322-ABP_pos
LTCDLLSFEAKGFAANHSLCAAHCLAIGRKGGACQNGVCVCRR
>ID02377-ABP_both
QVVRNPQSCRWNMGVCIPISCPGNMRQIGTCFGPRVPCCR
>ID02476-ABP_both
CGESCVFIPCISTLLGCSCKNKVCYRNGVIP
>ID02577-ABP_both
GIFSKISGKAIKNLFIKGAENVGKHVGMDVVRTGIDVVGCKIKGEC
>ID02578-ABP_both
GIFSKISGKAIKNLFIKGAKNVGKEVGMDVVRTGMDVVGCKIKGEC
>ID02604-ABP_both
GLFKVLGSVAKHLLPHVVPVIAEKL
>ID02614-ABP_both
GLMDTVKNAAKNLAGQMLDKLKCKITGSC
>ID02623-ABP_both
GLRKRLRKFRNKIKQKLKKI
>ID02625-ABP_both
GLVSSIGKALGGLLADVVKSKGQPA
>ID02655-ABP_both
GVVTDLLNTAGGLLGNLVGSLSG
>ID02670-ABP_both
IIGPVLGLVGSALGGLLKKIG
>ID02677-ABP_both
INLKAITALAKKLL
>ID02688-NO
KIPCGESCVWIPCVTSIFNCKCKENKVCYHD
>ID02805-ABP_neg
NFVTCRINRGFCVPIRCPGHRRQIGTCLGPRIKCCR
>ID02851-NO
RKVDFLEENITALLEEAQIQQEKNMYELQKLNSWD
>ID02898-NO
VDFLEENITALLEEAQIQQEKNMYELQKLNSWDVF
>ID03046-ABP_both
FLPFVGKLLSGLL
>ID03052-ABP_both
FLPILGKLLSGLL

>ID03110-ABP_both
GFKDWIKGAAKKLIKTVASAIANQ
>ID03151-NO
GIPCGESCVWIPCISAAIGCSCKNKVCYRN
>ID03152-NO
GIPCGESCVWIPCLTSAIGCSCKSKVCYRD
>ID03167-ABP_both
GLFSKLNKKKIKSGLIKIIKTAGKEAGLEALRTGIDVIGCKIKGEC
>ID03185-ABP_both
GLMSVLKGVLKTAGKHIFKNVGGSLLDQAKCKISGQC
>ID03188-NO
GLPTCGETCFGGTCNTPGCSCSSWPICTRN
>ID03209-ABP_both
GLWNSIKIAGKNLFVNVLDKIRCKVAGGC
>ID03232-ABP_both
GSKKPVPIIYCNRRATGKCQRM
>ID03239-NO
GTLPCGESCVWIPCISSVVGCSCKSKVCYKN
>ID03252-ABP_both
GVWSTILGGLKKFAKGGLDAIVNPK
>ID03283-ABP_both
IGKKWKRIVKRIKKFLRKL
>ID03391-ABP_neg
KWKSFIKKLTSKFLHSAKKN
>ID03402-ABP_neg
KWKSFLKTFSKAKKKVLKTALKAISK
>ID03413-NO
LEGEVNKIALLSTNKAVVSLSNGVSVLTSKVLD
>ID03587-NO
RECRSQSKQFVGLCVSDTNCASVCLTEHFPGGKCDGYRRCFCTKDC
>ID03589-ABP_both
RFRPPIRRPPIRPPFNPPFRPPVRPPFRPPFRPPFRPPIGPFPGRR
>ID03597-ABP_both
RIKRFWPVVIRTVVAGYNLYRAIKK
>ID03601-ABP_neg
RKRLRKFRNKIKEKLKKIGQKI
>ID03621-NO
RSDIEKLKEAIRDTNKAVQSVQSSIGNLIVAIKSV
>ID03658-NO
SRSELIVHQRRC
>ID03686-NO
VEAKQARSDIEKLKEAIRDTNKAVQSVQSSIGNLI
>ID03696-NO
VNKIALLSTNKAVVSLSNGVSVLTSKVLDLKNY
>ID03705-ABP_both
WFKKIPKFLHLLKKF
>ID03752-ABP_both
AKKVSKRLEKLFSKIQNDK
>ID03754-ABP_both
AKRLKKLAKKIWNWK
>ID03847-ABP_both

DKLIGSCVWGAVNYTSDCNGECKRRGYKGGYCGSFANVNCWRT
>ID03848-ABP_neg
DKLIGSCVWGAVNYTSDCNGECLLRGYKGGHCGSFANVNCWCET
>ID03855-ABP_both
DSAAGAVRAGDDETLLKPVLNSLDNLVSGL
>ID03909-ABP_both
FIGAIAAALPHVINAIKNTF
>ID03930-ABP_both
FLPFIAGMAAKFLPKIFCAISKK
>ID03951-ABP_both
FPVTWPTKWWKG
>ID03961-NO
FVDLKKIANIINSIFKK
>ID03962-ABP_both
FVKKFWGGVKAIFKGARKGLK
>ID04055-NO
GLFDIIKKIAESW
>ID04083-ABP_both
GLWQKIKQAAGDLASGIVEGIKS
>ID04096-ABP_both
GRFRRLRKKTRKRLKKIGKVLKWIPPIVGSIPLG
>ID04099-ABP_both
GRKSDCFRKSGFCAFLKPSLTLISGKCSRFYLCCKRIW
>ID04121-ABP_both
GVIDAAKKVVNVLANLF
>ID04186-ABP_both
INLKAIAAFAKKLL
>ID04199-ABP_both
INWKSIFEKVKNLV
>ID04221-ABP_pos
ITSISLCTPGCKTGALMGCNMKTATCNCSVHVSK
>ID04233-NO
KARHGSCNYVFPAHK
>ID04238-NO
KARHGSCNYVFPAHKCICYF
>ID04355-ABP_neg
KWKSFIKKLTSAAKKVTTAAKPLTK
>ID04369-ABP_both
LCAAHCLAIGRR
>ID04374-NO
LEKARHGSCNYVFPA
>ID04375-NO
LEKARHGSCNYVFPAH
>ID04376-NO
LEKARHGSCNYVFPAHK
>ID04378-NO
LEKARHGSCNYVFPAHKCI
>ID04379-NO
LEKARHGSCNYVFPAHKCIC
>ID04382-ABP_neg
LFRKQLKW

>ID04414-ABP_both
LLKLLKKLLKKLLKL
>ID04418-ABP_both
LLKWLKKLLKK
>ID04431-ABP_both
LQDAALGWGRRCPRCPPCPNCRRCPRCPTCPSCNCNPK
>ID04437-ABP_both
LSLALAALPKLFCLIFKKC
>ID04447-ABP_both
MAFKKLEKVGRNIRDGIIKAGPAVAVIGQATSIARPTGK
>ID04566-NO
MTPFWRGVSLRPIGASCRDDSECITRLCKKRRCSLSVAQE
>ID04583-ABP_both
NIWKKIFEKVKNLV
>ID04594-ABP_both
NWKKIASIGKEVLKAL
>ID04597-NO
PAHKCICYF
>ID04600-ABP_both
PFSLIPHAIGGLISAIK
>ID04615-NO
QKLCERPSGTWSGVCGNSNACKNQCIN
>ID04642-ABP_both
RCICTTRTCRFPYRRLGTCIFQNRVYTFCC
>ID04654-NO
RHGSCNYVFPAH
>ID04655-NO
RHGSCNYVFPAHK
>ID04656-NO
RHGSCNYVFPAHKC
>ID04657-NO
RHGSCNYVFPAHKCI
>ID04658-NO
RHGSCNYVFPAHKCIC
>ID04660-NO
RHGSCNYVFPAHKCICYF
>ID04673-ABP_both
RIKRFWPVVIRTVVAGYNLYRA
>ID04682-NO
RLEKARHGSCNYVFP
>ID04683-NO
RLEKARHGSCNYVFPA
>ID04684-NO
RLEKARHGSCNYVFPAH
>ID04685-NO
RLEKARHGSCNYVFPAHK
>ID04686-NO
RLEKARHGSCNYVFPAHKC
>ID04687-NO
RLEKARHGSCNYVFPAHKCI
>ID04703-ABP_both

RRIIRKIIHIIK
>ID04704-ABP_both
RRIIRKIIHIIKK
>ID04726-ABP_both
RVIRVVQRACRAIRHIVRRIRQGLRRILRVV
>ID04742-NO
SCNYVFPAHKCIC
>ID04743-NO
SCNYVFPAHKCICY
>ID04744-NO
SCNYVFPAHKCICYF
>ID04767-ABP_both
SNWKKIASIGKEVLKAL
>ID04810-ABP_pos
TYYGNGVSCNKKGCSVDWGKAISIIGNNSAANLATGGAAGWKS
>ID04848-ABP_both
VVIRTVVAGYNLYRAIKKK
>ID04852-ABP_both
WKKIASIGKEVLKAL
>ID04865-ABP_both
WLSKTYKKLENSAKKRISEGIAIAIQGGPR
>ID04888-ABP_both
YSRCQLQGFNCVVRSYGLPTIPCCRGSYFPGSTYGRCQRP
>ID04994-ABP_both
AKKVFKRLGIGAVLWVLTTG
>ID05005-NO
ALLDKLKSLGKVVGKVALGVVQNYLNPRQ
>ID05017-ABP_neg
ALWMTLLKKVLKAAAK
>ID05019-ABP_neg
ALWMTLLKKVLKAAAKAALKAVLVGANA
>ID05059-NO
ASWWAGIKQEF
>ID05067-ABP_both
ATCDLLSGTGVGHSACAAHCLLRGNRGGYCNGKGVCVCRN
>ID05074-ABP_pos
ATYYGNGVYCNKQECWVDWNKASKEIGKIIVNGWVQHGPWAPR
>ID05091-NO
AWLDKLKSLGKVVGKVAIGVAQHYLNPQQ
>ID05094-NO
CAESCVWIPCTVTALLGCSCSNKVCYNGIP
>ID05119-NO
CIRLEKARHGSC
>ID05146-ABP_both
DAQEKRQPWLPFG
>ID05165-ABP_both
DKLIGSCVWGAVNYTSDCNGECKRRGYKGGYCGSFANVNCWCET
>ID05189-NO
DSHEKRHHGYKRKFHEKHHSHRGY
>ID05279-ABP_both
FFHHIFRPIVHVAKTIHRLVTG

>ID05280-ABP_both
FFHHIFRPIVHVPKTIHRLVTG
>ID05340-ABP_both
FKRLKKLFSKIWNWK
>ID05347-ABP_both
FLFSLIPSAIGGLISAFK
>ID05354-ABP_both
FLGGLIKIVPAMICAVRKKC
>ID05365-ABP_both
FLGWLFKWASK
>ID05395-ABP_both
FLPLIGRVLSGAL
>ID05396-ABP_both
FLPLIGRVLSGIA
>ID05398-NO
FLPLLAGLAANFLPKLFCKITKKC
>ID05438-ABP_both
FVRWFSKFLGRIL
>ID05442-ABP_both
FWGALAKGALKLIVGSLFSSFSKKD
>ID05443-ABP_both
FWGALAKGALKLIVSLFSSFSKKD
>ID05510-ABP_both
GFLSILKKVLKVMAHMK
>ID05520-ABP_both
GFMKYIGPLIPHKVKAISDLI
>ID05560-ABP_both
GIFSKFVGKGLKNLFMKGAKTIGREVGMDVLRTGIDIAGCKIKGEC
>ID05588-ABP_both
GIGSAILSAGKSIIKGLAKGLAEHF
>ID05628-ABP_both
GKGALKKFLAKKVAKTVAKQAAKQGAKYVVNKQME
>ID05631-ABP_both
GKKLKKIGQKIKNFFQKL
>ID05634-ABP_both
GKKLLKKLKKLWKKW
>ID05675-ABP_both
GLFDVIKKVASVIKKL
>ID05680-ABP_both
GLFGVLAKVAAHVVPAIAKHF
>ID05694-ABP_both
GLKKIFKAGLGSLVKGIKAHVAS
>ID05695-ABP_both
GLKKIFKKGLGSLVKGIAAHVAS
>ID05703-NO
GLLDTFKNMALNAAKSAGVSVLNSLSCKLSKTC
>ID05715-ABP_both
GLLKKLLKIAAKVGKKLL
>ID05725-ABP_both
GLLSTFKNLATNVAGTVIDTLKCKVTGGCRT
>ID05727-ABP_both

GLMSLFRGGVLKTAGKHIFKNVGGSLLDQAKCKITGEC
>ID05736-ABP_both
GLPASISWIKRKRQQ
>ID05739-ABP_both
GLPLLISWIKRKRQQ
>ID05755-ABP_both
GLSALISWIKRKRQQ
>ID05784-NO
GRAGLQFPVGRLLRRLLRRLLR
>ID05809-NO
GSLLGDFFRKSKEKIGKEFKRIVQRIKDFLRNLVPRTES
>ID05830-ABP_both
GVFDIIKGAGKQLIARAMGKIAEKVGLNKDGN
>ID05846-ABP_both
GWGSFFKKAAHVGKHVAKAALTHYL
>ID05862-NO
GYCRCICGRGICRCICGR
>ID05930-NO
HRILMRIRQMMT
>ID05951-NO
IGKFLHAAKKFAKAFVAEIMNS
>ID05984-NO
ILGKLLSTWAGLLSNL
>ID05985-NO
ILGKLLSWAAGLLSNL
>ID05993-ABP_both
ILKKLLSTAAGLLSNL
>ID06006-NO
ILKKWPWWPWRK
>ID06009-NO
ILKKWPWWPWRRKM
>ID06011-NO
ILKKWPWWPWRRMILKKAGS
>ID06042-ABP_both
ILPWKLPLLPLRR
>ID06054-ABP_both
ILPWKWPWLPYRR
>ID06070-NO
ILRRWPWWPWRRR
>ID06080-NO
ILRWPWWPWRAK
>ID06081-NO
ILRWPWWPWRRA
>ID06085-NO
ILRWPWWPWRRKM
>ID06086-NO
ILRWPWWPWRRKMILKKAGS
>ID06145-ABP_both
KAKLFKKIPKFLHLWKKF
>ID06203-ABP_both
KIKLFKKWPKFLHLAKKF

>ID06216-NO
KKFAKKFAKKFAKKFAKKFAKKFA
>ID06227-ABP_neg
KKKKKKAAFALWAAFAA
>ID06232-ABP_neg
KKKKKKALFALWLAFAA
>ID06239-ABP_both
KKKLFWKIPKFLHLAKKF
>ID06250-ABP_both
KKSYVRRWRS
>ID06317-NO
KNLRRIGRKIIHIIKKYG
>ID06327-NO
KNLRRIIRKISHIIKKYG
>ID06328-NO
KNLRRIIRKITHIIKKYG
>ID06329-NO
KNLRRIIRKSIHIIKKYG
>ID06330-NO
KNLRRIIRKTIHIIKKYG
>ID06331-NO
KNLRRISRKIIHIIKKYG
>ID06332-NO
KNLRRSIRKIIHIIKKYG
>ID06333-NO
KNLRRTIRKIIHIIKKYG
>ID06343-ABP_both
KNWKGIAGMAKKLLGKNWKLM
>ID06344-NO
KNWKGIAGMAKKLLGKNWKLMKK
>ID06367-ABP_both
KRFWPLVPVAINTVAAGINLYKAIRRK
>ID06370-NO
KRKFHEKHHSHRGYC
>ID06373-NO
KRLFKELKKSLRKY
>ID06378-NO
KRLRRIIRKGIHIIKKYG
>ID06420-NO
KWKEFIKKLTTAVKKVLTTGLPALIS
>ID06443-ABP_both
KWKKLLKKPLKLLKKLLK
>ID06453-NO
KWKLFKKIGIGAVLKVLTM
>ID06455-NO
KWKLFKKIGIGAVLKVLTTGLPALKKTK
>ID06456-NO
KWKLFKKIGIGAVLKVLTTGLPALTLTK
>ID06458-ABP_both
KWKLFKKIGKVLKVL
>ID06463-ABP_both

KWKLFKKPKFLHLAKKF
>ID06491-ABP_both
KWKSFLKTFKSASKTVLHTALKAISS
>ID06495-ABP_both
KWKSFLKTFKVAVKTVLHTALKAISS
>ID06504-ABP_both
KWLLKLKLKLLK
>ID06527-NO
LCQRPSGTWSGV
>ID06536-NO
LEKGLDGAKKAVGGLGKLGKDAVEDLESVGKGAVHDVKDVLDSVL
>ID06539-ABP_both
LFCKGGSCHFGGCPSHLIKVGSCFGFRSCCAWPWNA
>ID06568-NO
LIRELGQRIRRPIRRIARCAG
>ID06569-NO
LIRELGQRIRRPIRRIARCAGQVV
>ID06570-NO
LIRELGQRIRRPIRRIARCAGQVVEIVR
>ID06573-NO
LIRRLGQRIRRPIHRIARCAG
>ID06574-NO
LIRRLGQRIRRPIHRIARCAGQVV
>ID06575-NO
LIRRLGQRIRRPIHRIARCAGQVVEIVR
>ID06581-NO
LKKLLKKLLKKLLKKLLKKL
>ID06613-ABP_neg
LLGDFFRKSKEKIGKEFKRIVQRIKDFLRNL
>ID06622-ABP_both
LLPALISWIKRKRQQ
>ID06634-NO
LLSRVYQILQPILQRLSATLQRIREVLR
>ID06645-ABP_both
LNWGAILKHIKK
>ID06646-ABP_both
LNWGAILKHKIK
>ID06680-NO
LRRGGRWILAIPRRIR
>ID06701-NO
LWETLRRGGRWILAIPRRIR
>ID06703-NO
LWETLRRGGRWILAIPRRIRQGLELTL
>ID06704-NO
LWETLRRGGRWILAIPRRIRQGLRLTL
>ID06705-NO
LWETLRRGGRWILAIPRRIRRGLELTL
>ID06715-NO
MAARAAGLAARLAALALRAL
>ID06788-NO
MASRAAGLARRLARLARRAL

>ID06840-NO
MGECVRGRCPSGMCCSQFGYCGKGPKYCG
>ID07100-NO
MVSRAAGLAARLARLALRAL
>ID07140-NO
NQGRHFCGGALIHARFVMTAATCFQ
>ID07141-NO
NQGRHFCGGALIHARYVMTAASCFQ
>ID07142-NO
NQGRHFSAAALIHARFVMTAASCFQ
>ID07143-NO
NQGRHFSAGALIHARFVMTAASCFQ
>ID07144-NO
NQGRHFSGAALIHARFVMTAASCFQ
>ID07145-NO
NQGRHFSGGALIHARFVMTAAHCFQ
>ID07146-NO
NQGRHFSGGALIHARFVMTAAKCFQ
>ID07147-NO
NQGRHFSGGALIHARFVMTAARCFQ
>ID07148-NO
NQGRHYCGGALIHARFVMTAASCFQ
>ID07163-NO
NWKGIAAMAKKLL
>ID07240-NO
QIWNNMTWMEWDREINNYTSLIHSLIEESQNQQEKN
>ID07250-NO
QKLCQRPSG
>ID07314-NO
RACRAIRHIPRRIR
>ID07443-NO
RHFCGGALIHARFVMTAASS
>ID07445-NO
RHFSAAALIHARFVMTAASC
>ID07448-NO
RHFSGGALIHARFAMTAASC
>ID07451-NO
RHFSGGALIHARFVMTAAHC
>ID07452-NO
RHFSGGALIHARFVMTAAKC
>ID07478-NO
RIRRPIRRIARCAGQVVEIVR
>ID07488-NO
RLCERPSGTWSGVCGNNNACRNQCRNLERAEHGSCNYVFPAHKCICYFPC
>ID07505-ABP_neg
RLRRIVVIRVAR
>ID07513-ABP_both
RLWRIVVIRVAR
>ID07541-ABP_both
RRARIVVIRVAR
>ID07556-NO

RRGLLRVIRTVILLLDRLRHY
>ID07560-NO
RRICRCICGRGICRCICG
>ID07606-NO
RRLRRIIRKGIRIIKKYG
>ID07645-NO
RVCRRRSAGFKGLCMSDHNCAQVCLQEGWGGGNCDGVMRQCKCIRQC
>ID07654-NO
RVIEVVQGACRASRHIPRRIRQGLERIL
>ID07656-NO
RVIEVVQGICRAIRHIPRRIRQGLERIL
>ID07658-NO
RVIEVVRGACRAIRHIPRRIRQGLERIL
>ID07660-NO
RVIRVVQGACRAIRHIPRRIRQGLERIL
>ID07661-NO
RVIRVVRGACRAIRHIPRRIR
>ID07662-NO
RVISVVQGACRAIRRIPRRIR
>ID07663-NO
RVISVVQGACRAIRRIPRRIRQGLERIL
>ID07680-NO
RYICRCICGRGICRCICG
>ID07692-ABP_both
SFLDKFKDVAIGVAKGAGTGVLKALLCKLDNSC
>ID07699-NO
SIGSAFKKAAHVGKHVGKAALTHYL
>ID07701-ABP_pos
SILPTIVTFLSKFL
>ID07709-NO
SLEQIWNNMTWMEWDREINNYTSLIHSLIEESQNQQ
>ID07749-ABP_both
SWKSFLKTFSSAKSTVLHTALKAISS
>ID07770-NO
TAYFLLKLAGAW
>ID07773-NO
TAYFLLKLAGRW
>ID07804-NO
TTWEAWDRAIAEYAARIEALIRALQELQEKNEAALREL
>ID07807-NO
TTWEAWDRAIAEYAARIEALIRASQEQQEKNEAELREL
>ID07808-NO
TTWEAWDRAIAEYAARIEALLRALQEQQEKNEAALREL
>ID07835-NO
VFPSDEFDASISQVNEKINQSLAFIRKSDELLHNV
>ID07858-ABP_both
VKLYVYPLKVKLYP
>ID07914-NO
VSKVLHLEGEVNKIALLSTNKAVVSLSNGVSVL
>ID07931-NO
VVRGACRAIRHIPRRIR

>ID07932-NO
VVRGACRAIRHIPRRIRGLERIL
>ID07937-NO
VYWKKILGKIIKVVK
>ID07952-ABP_both
WGIRDILKYGKPS
>ID07972-NO
WMEWDREINNYTSLIHSLIEESQNQQEKNEQELLEL
>ID07974-NO
WNNMTWMEWDREINNYTSLIHSLIEESQNQQEKNEQ
>ID07999-NO
WWKKWPWWPWRRK
>ID08037-NO
YDPLVFPSDEFDASISQVNEKINQSLAFIRKSDEL
>ID08061-NO
YHRLRDLLLIVTRIVELL
>ID08062-NO
YHRLRDLLLIVTRIVELLGRR
>ID08064-NO
YHRLRDLLLIVTRIVRLLGRR
>ID08068-NO
YHRLRRLLLIVTRIVELLGRR
>ID08083-ABP_both
YPVKLKVYPLKVKL
>ID08100-NO
YQLLIRMIYKNI
>ID08135-NO
YTSLIHSLIEESQNQQEKNEQELLELNKWASLWNWF
>ID08136-NO
YTSLIHSLIEESQNQQEKNEQELLQLDKWASLWNWF
>ID08137-NO
YTSLIHSLIEESQNQQEKNEQQLLELDKWASLWNWF
>ID08138-NO
YTSLIHSLIEESQNQQEKNQQELLQLDKWASLWNWF
>ID08139-NO
YTSLIHSLIEESQQQQEKNEQELLELDKWASLWNWF
>ID08140-NO
YTSLIHSLIEETQNQQEKNEQELLELDKWASLWNWF
>ID08141-NO
YTSLIHSLIEEVQNQQEKNEQELLELDKWASLWNWF
>ID08142-NO
YTSLIHSLIEEWQNQQEKNEQELLELDKWASLWNWF
>ID08143-NO
YTSLIHSLIEEYQNQQEKNEQELLELDKWASLWNWF
>ID08144-NO
YTSLIHSLIEQSQNQQEKNEQELLELDKWASLWNWF
>ID08145-NO
YTSLIHSLIQESQNQQEKNEQELLELDKWASLWNWF
>ID08147-NO
YTSLIQSLIEESQNQQEKNEQQLLELDKWASLWNWF
>ID08203-NO

ACYCRIPACIAGERRYGTCAYQGRAWAFCC
>ID08204-NO
ACYCRIPACIAGERRYGTCAYQGRLWAFCC
>ID08219-NO
ADGALLGRMKRA
>ID08221-NO
ADGMHRSLLGRMKGA
>ID08227-NO
ADGSRSSLLGRMKGA
>ID08242-NO
ADLEVVAATYVLDA
>ID08243-NO
ADLEVVAATYVLDD
>ID08245-NO
ADLEVVAATYVLVD
>ID08327-NO
AKKAWKKAKKAAKKAKKWAKK
>ID08390-NO
ALVEAKQARSDIEKLKEAIRDTNKAVQSVQSSIGN
>ID08395-ABP_both
ALWKTMLKKLGTVALHAGKAALGAVADTISQ
>ID08467-NO
ARRAWRRARRAARRARRAARR
>ID08494-NO
ATAEETRRMLHRAADTLA
>ID08495-NO
ATAEETRRMLHRAFATLA
>ID08496-NO
ATAEETRRMLHRAFDTAA
>ID08497-NO
ATAEETRRMLHRAFDTLA
>ID08506-NO
ATCDLFSLQSKWVTPNHAACAAHCLLRGNRGGQCKGTICHCRK
>ID08510-NO
ATCDLLSFSSKWVTPNHAGCAAHCLLRGNRGGHCKGTICHCRK
>ID08513-NO
ATCDLLSGLGVNDSACAAHCIARGNRGGYCNSKKVCVCRN
>ID08520-NO
ATCYCRTGRCATRESLSGVCEIRGRLYRLCCR
>ID08521-NO
ATCYCRTGRCATRESLSGVCEISGRLYRACCR
>ID08523-NO
ATCYCRTGRCATRESRSGVCEISGRLYRLCCR
>ID08549-NO
AVGIGALFLGFLGAAGSTMGARSMTLTVQARQL
>ID08641-ABP_both
CFQWQRNMRKVA
>ID08642-ABP_both
CFQWQRNMRKVR
>ID08653-NO
CGESCVFIPCVTALLGCSCKSKVCYKNSIP

>ID08654-NO
CGESCVWIPCISAAVGCSCKSKVCYKNGTLP
>ID08655-NO
CGESCVYIPCISGVIGCSCTDKVCYLNGTP
>ID08718-NO
CIKNGNGCQPNGSQGNCCSGCHKQPGWVAGYCRRK
>ID08754-NO
CLGIGSCNNFAGCGYAVVCFW
>ID08958-ABP_neg
CVNWKKILGKIIKVVK
>ID08980-ABP_both
DAACAAHCLFR
>ID08983-NO
DADSSVEKQVALLKALYGHGQISHKRHKTDSFVGLM
>ID08989-NO
DASISQVNEKINQSLAFIRKSDELLHNVNAGKSIT
>ID09018-NO
DDSVVSAAMSYSFA
>ID09020-NO
DDSVVSAAMSYSYA
>ID09051-NO
DGSLLGRMKGAA
>ID09065-NO
DHYNCVSSGGQCLYSACPIFTKIAGTCYRGKAKCCK
>ID09066-NO
DHYNCVSSGGQCLYSACPIFTKIEGTCYRGKAKCCK
>ID09068-NO
DHYNCVSSGGQCLYSACPIFTKIQGTCYRGAAKCCK
>ID09069-NO
DHYNCVSSGGQCLYSACPIFTKIQGTCYRGEAKCCK
>ID09088-NO
DLFQVIKEKLKELTGGVIEGIQG
>ID09115-NO
DPVTCLKSGAICHPVFCPRRYKQIGTCGLPGTKCCKP
>ID09231-NO
EINNYTSLIHSLIEESQNQQEKNEQELLELDKWASL
>ID09317-NO
EWGRRCCGWGPGRRYCVRWC
>ID09347-NO
FAKKLAKKLAKLAL
>ID09351-NO
FAKKLAKKLKKLAKKLAKLALAL
>ID09375-NO
FAKLLAKALKKFL
>ID09392-NO
FAKLLAKLAKKSL
>ID09393-NO
FAKLLAKLAKKVL
>ID09394-NO
FAKLLAKLAKLKL
>ID09410-NO

FALALKALKKL
>ID09432-NO
FDASISQVNEKINQSLAFIRKSDELLHNVNAGKSI
>ID09485-ABP_both
FIKELLPHLSGIIDSVANAIK
>ID09542-NO
FLGPIIKIATGILPTAICKILKKC
>ID09577-NO
FLSLIPHIVSGVASLAKHFG
>ID09679-ABP_both
GCASRCKAKCAGRRCKGWASASFRGRCCYCKCFRC
>ID09740-NO
GFFALIAKIISSPLFKTLLSAVGSALSSSGEQE
>ID09748-ABP_both
GFKDLLKGAAKQLVKTVLF
>ID09759-NO
GFMDTAKNVAKNVAVTLLDNLRCKITKAC
>ID09787-NO
GGYYCPFFQDKCHRHCRSFGRKAGYCGGFLKKTCICVMK
>ID09812-NO
GIGGVLLSAGKAALKGLVKVLAEKYVN
>ID09813-NO
GIGGVLPSAGKAALKGLAKVLAEKYAN
>ID09823-NO
GIGVLLSAGKAALKGLAKVLAEKYAN
>ID09878-ABP_both
GKYTCGETCFKGKCYTPGCTCSYPICKKD
>ID09889-NO
GLFGKLIKKFARKAISYAVKKARGKH
>ID09908-NO
GLLCYCRKGHCKRGERVRGTCTCGIRFLYCCPRR
>ID09909-NO
GLLCYCRKGHCKRGGRVRGTCGIRFLYCCPRR
>ID09933-NO
GLLSVLKGVLKTAGKHIFKNVGGSLLDQAKCKISGEC
>ID09936-ABP_both
GLNTLKKVIQGLHEVIKLVNNHA
>ID09944-NO
GLPVCGETCVGGTCNTPGCSCSWPVCFRN
>ID09950-ABP_both
GLVGTLLGHIGKAILGG
>ID09952-NO
GLVSSIGRALGGLLADVVKSKQPA
>ID10045-NO
GSRIPTGERVWDRGNVTLLC
>ID10069-NO
GTKALTEVIPLTAEAEC
>ID10071-NO
GTKALTEVIPLTEAAEC
>ID10072-NO
GTKALTEVIPLTEEAEC

>ID10074-NO
GTKGLTEVIPLTEEAEC
>ID10080-NO
GTLPCGESCVWIPCISSVVGCACKSKVCYKD
>ID10114-ABP_both
GWLDVAKKKGKAAFNVAKNFI
>ID10151-NO
HAILMRIRQMMT
>ID10153-NO
HALWKNMLKGIGKLAGKAALGAVKKLVGAES
>ID10154-NO
HALWMTLLKKVLKAAAKAALNAVLVGANA
>ID10224-NO
HRIDLGPPISLERLDVGTNLGNAIAKLEDAKELLE
>ID10259-NO
IDISIELNKAKSDLEESKEWIRRSNQKLDSIGNW
>ID10260-NO
IDLGPPISLERLDVGTNLGNAIAKLEDAKELLESS
>ID10271-NO
IEKLKEAIRDTNKAVQSVQSSIGNLIVAIKSVQDY
>ID10274-NO
IELNKAKSDLEESKEWIRRSNQKLDSIGNWHQSS
>ID10295-NO
IHSLIEESQNQQEKNEQELLELDKWASLWNWFNITN
>ID10303-ABP_both
IIPLPLGYFAKKP
>ID10339-ABP_both
ILGPVISTIGNVLGGLLKNL
>ID10360-NO
ILMCFSIDSPDSLEN
>ID10383-ABP_both
ILPWKWPWWAWRR
>ID10384-NO
ILPWKWPWWGWRR
>ID10385-NO
ILPWKWPWWHWRR
>ID10386-NO
ILPWKWPWWPGRR
>ID10387-NO
ILPWKWPWWPHRR
>ID10389-NO
ILPWKWPWWPWGR
>ID10391-NO
ILPWKWPWWPWRG
>ID10406-ABP_both
INLKAIAALAKKLF
>ID10412-NO
INNYTSLIHSLIEESQNQQEKNEQELLELDKWASL
>ID10413-NO
INNYTSLIHSLIEESQNQQEKNEQELLELDKWASLW
>ID10420-NO

INWLKLGKQILGAL
>ID10421-ABP_both
INWSKIFEKVKNLV
>ID10437-NO
IRDTNKAVQSVQSSIGNLIVAIKSVQDYVNKEIVP
>ID10449-NO
ISGINASVVNIQKEIDRLNEVAKNLNESLIDLQEL
>ID10450-NO
ISGINASVVNIQKEIDRLNEVAKNLNESLIDLQELGK
>ID10453-NO
ISIELNKAKSDLEESKEWIRRSNQKLDSIGNWHQ
>ID10531-NO
KDLKVVAATYVKKK
>ID10534-NO
KEAIRDTNKAVQSVQSSIGNLIVAIKSVQDYVNKE
>ID10537-ABP_both
KEFKRIVQRIKDFLRNLV
>ID10550-NO
KFGSFIKRMWRSKLAKKLRAKGKELLRDYANRVLSPEEEAAAPAPVPA
>ID10598-NO
KILRGVAKKIMRTFLRRISKKILTGKK
>ID10614-ABP_both
KKCFRWQWRMKKLGA
>ID10616-ABP_both
KKCRRWQWRMKKLGA
>ID10620-NO
KKIGKKIERVGQHTRDATQTIGVAQQAANVAATLKG
>ID10643-NO
KKKKLLAPFLVFF
>ID10647-NO
KKKKLLLAFYFFF
>ID10654-NO
KKKKLLLPFLVFV
>ID10656-NO
KKKKLLLPFYFFF
>ID10657-NO
KKKKLLLPFYFFV
>ID10664-NO
KKKKLLLPTYFFF
>ID10668-NO
KKKKLVAATYVLV
>ID10677-NO
KKKKLVLPFYFFF
>ID10699-NO
KKKKVLLPFYFFF
>ID10708-NO
KKKKVVAAFYVLV
>ID10734-NO
KKKKVVAATYLLV
>ID10735-NO
KKKKVVAATYPLV

>ID10736-NO
KKKKVVAATYTLV
>ID10743-NO
KKKKVVAATYVKKA
>ID10744-NO
KKKKVVAATYVKV
>ID10750-NO
KKKKVVAATYVLK
>ID10751-NO
KKKKVVAATYVLKK
>ID10752-NO
KKKKVVAATYVLL
>ID10753-NO
KKKKVVAATYVLP
>ID10754-NO
KKKKVVAATYVLT
>ID10755-NO
KKKKVVAATYVLV
>ID10756-NO
KKKKVVAATYVLVA
>ID10757-NO
KKKKVVAATYVLVF
>ID10758-NO
KKKKVVAATYVLVK
>ID10759-NO
KKKKVVAATYVPV
>ID10760-NO
KKKKVVAATYVTV
>ID10771-NO
KKKKVVAPTYVLV
>ID10780-NO
KKKKVVLATYVLV
>ID10792-ABP_neg
KKLAKAPKLLALLWLKLAKALKKA
>ID10798-ABP_neg
KKLALAPKKLALLWKKLALALKKA
>ID10799-ABP_both
KKLALKALKLWLLALKKLALLALKK
>ID10803-ABP_neg
KKLKLALAKLALLWKALALKLKKA
>ID10849-NO
KLLKLLLKLYKKLLKLL
>ID10916-NO
KRFAEPLPSEEEGESYSKEVPEMEKRYGGFM
>ID10965-ABP_neg
KRWRIRVRVIRKC
>ID10999-ABP_both
KWCRCVCRRGICYCRCRG
>ID11046-NO
KWWKKAAKAAKKAAKAAKKWA
>ID11082-NO

LCYCRRRFCYCV
>ID11107-NO
LEAIPCSIPPCVGFGKPFVF
>ID11115-NO
LEAIPMSIPPEFLFGKPFVF
>ID11119-NO
LEAIPMSIPPEVKANKPFVF
>ID11120-NO
LEAIPMSIPPEVKFAKPFVF
>ID11121-NO
LEAIPMSIPPEVKFNAPFVF
>ID11122-NO
LEAIPMSIPPEVKFNKAFVF
>ID11123-NO
LEAIPMSIPPEVKFNKPAVF
>ID11124-NO
LEAIPMSIPPEVKFNKPFAF
>ID11125-NO
LEAIPMSIPPEVKFNKPFVA
>ID11126-NO
LEAIPMSIPPEVKFNKPFVF
>ID11129-NO
LERLDVGTNLGNAIAKLEDAKELLESSDQILRSMK
>ID11147-NO
LGPPISLERLDVGTNLGNAIAKLEDAKELLESSDQ
>ID11161-NO
LHRIDLGPPISLERLDVGTNLGNAIAKLEDAKELL
>ID11194-NO
LKLLSIVSWAKKVL
>ID11195-NO
LKLNSIVSWAKKVL
>ID11202-NO
LKWKSIVSWAKKVL
>ID11206-NO
LLDCWVRLGRYLLRRLKTP
>ID11208-NO
LLDCWVRLGRYLLRRLKTPFTRL
>ID11216-NO
LLGDEFRKSKEKIGKEFKRIVQRIKDFLRNLVPRTES
>ID11218-ABP_neg
LLGDFFRKAKEKIGKEFKRIVQRIKDFLRNLVPRTES
>ID11222-ABP_both
LLGDFFRKVKEKIGKEFKRIVQR
>ID11223-ABP_neg
LLGDFFRKVKEKIGKEFKRIVQRIKDFLRNLVPRTES
>ID11246-NO
LNITNSHVSILQERPPLENRVL
>ID11249-NO
LNLKSIVSWAKKVL
>ID11250-NO
LNLNSIVSWAKKVL

>ID11259-NO
LPIVAGLAANFLPKIVCKITKKC
>ID11260-NO
LPKWKVFKKIEKVGRNIRNGIVKAGPAIAVLGEAKALG
>ID11261-NO
LPLIASVAANLVPKIFCKITKKC
>ID11318-NO
LRLLTPSQFTRIGLTVAKKNVRRAHERNRIKRLTRELDFVVLSEAL
>ID11342-NO
LSTLLNVASNVVPTLICKITKKC
>ID11466-NO
MDVNPTLLFLKVPAQN
>ID11470-NO
MDVNPWLLFLKVPAQ
>ID11472-NO
MDVNPYLLFLKVPAQ
>ID11496-NO
MEWDREINNYTSLIHSLIEESQNQQEKNEQELLELD
>ID11959-NO
MSTNPKPQRKTKRNTNRRPQDVKFPGGGQIVGGVYL
>ID12000-NO
NCLLLGTEVSEALGGAGL
>ID12001-NO
NCLLLGTEVSEALGGAGLT
>ID12005-NO
NDITLNNSVALDPIDISIELNKAKSDLEESKEWIR
>ID12012-NO
NFYDPLVFPSDEFDASISQVNEKINQSLAFIRKSD
>ID12033-NO
NITLNNSVALDPIDISIELNKAKSDLEESKEWIR
>ID12040-NO
NLCERASLTWTGNCGNTGHCDTQCRNWESAKHGACHKRGINWKCFCYFDC
>ID12054-NO
NNCLLLGTEVSEALGGAGLTGG
>ID12057-NO
NNSVALDPIDISIELNKAKSDLEESKEWIRRSNQ
>ID12060-NO
NNYTSLIHSLIEESQNQQEKNEQELLELDK
>ID12061-NO
NNYTSLIHSLIEESQNQQEKNEQELLELDKWASLWN
>ID12072-NO
NQGRHFCGGALIHARFVMTAAHCFQ
>ID12090-NO
NRRPQDVKFPGGGQIVGGVYL
>ID12120-NO
PAICQRATATLGTVGSNTSGTTEIEACILL
>ID12134-NO
PAWRKAFRWAARMLKKAA
>ID12135-NO
PAWRKAFRWAKRMLKKAA
>ID12166-NO

PIDISIELNKAKSDLEESKEWIRRSNQKLDSIGN
>ID12168-NO
PISLERLDVGTNLGNAIAKLEDAKELLESSDQILR
>ID12184-NO
PLNNCLLLGTEVSEALGGAG
>ID12214-NO
PPISLERLDVGTNLGNAIAKLEDAKELLESSDQIL
>ID12218-NO
PPLNNCLLLGTEVSEALGGAGLTGG
>ID12357-NO
QKKIRVRL
>ID12359-NO
QKKIRVRLSW
>ID12380-NO
QNQQEKNEQELLELDKWASLWNWFNITNWLWLIKIF
>ID12422-NO
QTKDLQQKFYEIIMDIEQNNVQGKKGIQQLQKWED
>ID12460-NO
REINNYTSLIHSLIEESQNQQEKNEQELLELDKWAS
>ID12468-NO
RFGRFLRKIRRFRGKVTITIQGSARF
>ID12481-NO
RGGRLCYCRRRFCVCL
>ID12482-NO
RGGRLCYCRRRFCVCW
>ID12483-NO
RGGRLCYCRRRFCVCY
>ID12490-NO
RGLRRLGRKIAHTVKKYG
>ID12496-NO
RGTKALTEVIPLTEEAEC
>ID12506-NO
RIDLGPPISLERLDVGTNLGNAIAKLEDAKELLES
>ID12538-ABP_both
RKCLRWQWEMRKVGG
>ID12540-ABP_both
RKCLRWQWRMRKVGG
>ID12541-ABP_both
RKCLRWQWRMRKYGG
>ID12575-ABP_neg
RKSKEKIGKEFKRIVQRIKDFLRNL
>ID12589-NO
RLCYTRGRFTVCVR
>ID12597-ABP_both
RLLKFIKKLL
>ID12650-NO
RQLLSQIVQQQNNLLRAIEAQQHLLQLT
>ID12668-NO
RRGWVLALVLYYGRR
>ID12693-NO
RRKKAAVALLPAVLAALLAP

>ID12694-NO
RRKKAAVALLPAVLEALLAP
>ID12695-NO
RRKKAAVALLPAVLKALLAP
>ID12696-NO
RRKKAAVALLPAVLLAELAP
>ID12697-NO
RRKKAAVALLPAVLLAKLAP
>ID12698-NO
RRKKAAVALLPAVLLALEAP
>ID12706-NO
RRLHLEPAFLPYSVKAHECC
>ID12828-NO
RVCMGKSAGFKGLCMRDQNCAQVCLQEGWGGGNCDGVMRQCKCIRQC
>ID12844-NO
RVWDRGNVTLLCDCPNGPWV
>ID12957-NO
SGIVQQQNNLLRAIEAQQHLLQLTVWGIKQLQAR
>ID12965-NO
SGSWLRDIWDWICEVLSDFK
>ID12972-NO
SIELNKAKSDLEESKEWIRRSNQKLDSIGNWHQS
>ID12992-NO
SLERLDVGTNLGNAIAKLEDAKELLESSDQILRSM
>ID12996-NO
SLIEESQNQQEKNEQELLELDKWASLWNWFNITNWL
>ID13003-NO
SLLGRMKGA
>ID13017-NO
SNIKENKCNGTDAKVKLIKQELDKYKNAVTELQLL
>ID13025-NO
SPKMVQGSGCFGRKMDRISSSSGLGCKVL
>ID13026-NO
SPKMVQGSGCFGRKMDRISSSSGLGCKVLR
>ID13036-NO
SQAFLFQPQRF
>ID13042-NO
SQGVVESMNKELPKIIGQVRDQAEHLKTAY
>ID13043-NO
SQNQQEKNEQELLELDKWASLWNWFNITNWLWLIKI
>ID13051-NO
SRTHRHSMEIRTPDINPAWYASRGIRPVGRF
>ID13058-NO
SSCFGGRIDRIGAQSGLGCNSFRY
>ID13087-ABP_both
STLHLVLRLRGA
>ID13088-ABP_both
STLHLVLRLRGG
>ID13095-NO
SVALDPIDISIELNKAKSDLEESKEWIRRSNQKL
>ID13099-NO

SVITIELSNIKENKCNGTDAKVKLIKQELDKYKNA
>ID13123-NO
SWLRDIWDWICEVLSDFKT
>ID13124-NO
SWLRDIWDWICEVLSDFKTW
>ID13125-NO
SWLRDIWDWICKVLSDFK
>ID13126-NO
SWLRDIWDWIEEVLSDFK
>ID13127-NO
SWLRDIWDWIREVLSDFK
>ID13128-NO
SWLRDIWDWISEVLSDFK
>ID13165-NO
TCYCRRRFCVCVGR
>ID13178-NO
TDVILMCFSIDAPDSLENI
>ID13179-NO
TDVILMCFSIDSADSLENI
>ID13181-NO
TDVILMCFSIDSPASLENI
>ID13182-NO
TDVILMCFSIDSPDALENI
>ID13183-NO
TDVILMCFSIDSPDSAENI
>ID13185-NO
TDVILMCFSIDSPDSLANI
>ID13186-NO
TDVILMCFSIDSPDSLEAI
>ID13187-NO
TDVILMCFSIDSPDSLENA
>ID13189-NO
TEVSEALGGAGLTGGFYE
>ID13202-NO
TGPGAWQGGRRKFRRQRPRLSHKGPMPF
>ID13209-NO
TIELSNIKENKCNGTDAKVKLIKQELDKYKNAVTE
>ID13235-ABP_both
TLISWIKNKRKQRPRVSRRRRRRGGRRRRC
>ID13239-NO
TLLCDCPNGPWVWVPAFCQA
>ID13263-NO
TPDINPAWYTGRGIRPVGRF
>ID13273-NO
TQAQLLRVGCVLGTCQVQNLSHRLWQLMGPAGRQDSAPVDPSSPHSY
>ID13277-NO
TQTVYEWCGVATQLLAAYILL
>ID13278-NO
TRACDVIALLECHLNT
>ID13297-NO
TSAQITAAVALVEAKQARSDIEKLKEAIRDTNKAV

>ID13302-NO
TSRTGPGAWQGGRRKFRRQRPRLSHKGPMPF
>ID13304-NO
TSVITIELSNIKENKCNGTDAKVKLIKQELDKYKN
>ID13317-NO
TTWEAWDRAIAEYAARIEALIRAAQELQEKLEAALREL
>ID13318-NO
TTWMEWDREINNYTSLIHSLIEESQNQQEKNEQELLEL
>ID13333-NO
TWQEWERKVDFLEENITALLEEAQIQQEKNMYELQ
>ID13343-NO
VAKALKALLKALKAL
>ID13344-NO
VAKFLAKFLKKAL
>ID13347-NO
VAKKLAKLAKKLLAL
>ID13348-NO
VAKLLAKALKKLL
>ID13349-NO
VAKLLAKLAKKLL
>ID13350-NO
VAKLLAKLAKKVL
>ID13353-NO
VALALKALKKLAKKLKKLAKKAL
>ID13356-NO
VALDPIDISIELNKAKSDLEESKEWIRRSNQKLD
>ID13357-NO
VALDPIDISIELNKAKSDLEESKEWIRRSNQKLDSD
>ID13358-NO
VALDPIDISIELNKAKSDLEESKEWIRRSNQKLDSI
>ID13363-NO
VALVEAKQARSDIEKLKEAIRDTNKAVQSVQSSIG
>ID13364-NO
VANDPIDISIELNKAKSDLEESKEWIRRSNQKLDSD
>ID13365-NO
VANDPIDISIELNKAKSDLEESKEWIRRSNQKLDSI
>ID13367-NO
VATSAQITAAVALVEAKQARSDIEKLKEAIRDTNK
>ID13375-NO
VCGETCVGGTCNTPGCTCSWPVCTRDGLP
>ID13385-NO
VDGIPVSWDADARAPA
>ID13386-NO
VDLGDISGINASVVNIQKEIDRLNEVAKNLNESLIDLQELGKYE
>ID13468-ABP_both
VNWKKILKKIIKVAK
>ID13469-ABP_both
VNWKKILPKIIKVAK
>ID13472-ABP_both
VNWKKVLPKIIKVAK
>ID13488-NO

VPPGFTPF
>ID13494-NO
VPPLNNCLLLGTEVSEALGG
>ID13502-NO
VQGSGCFGRKMDRISSSSGLGCKVLRR
>ID13503-NO
VQGSGCFGRKMDRISSSSGLGCKVLRRH
>ID13505-ABP_neg
VQLRRIRVWVIR
>ID13538-NO
VSFAIKWEYVLLLFL
>ID13610-NO
WCYCRRRFCVCVGR
>ID13623-NO
WEDWVRWIGNIP
>ID13702-NO
WMEWDREINNYTSLIHSLIEESQNQQEKNEQELLE
>ID13705-NO
WMEWDREINNYTSLIHSLIEETQNQQEKNEQELL
>ID13706-NO
WMEWDREINNYTSLIHSLIEEWQNQQEKNEQELL
>ID13742-NO
WVRLGRYLLRRLKTPFTR
>ID13747-NO
WVWVPAFCQAVGWGDPITHW
>ID13771-NO
WYNQTKDLQQKFYEIIMDIEQNNVQGKKGIQQLQK
>ID13859-NO
YAKLLAKLAKKAL
>ID13869-ABP_both
YCNGKRVCVCR
>ID13874-NO
YCYCRRRFCVCVGR
>ID13925-NO
YKLFKKILKVL
>ID13936-NO
YKRGGGWGGGGWKGGGGGGGWKGGGGGKGGG
>ID13942-NO
YLHRIDLGPPISLERLDVGTNLGNAIAKLEDAKEL
>ID13951-NO
YNQTKDLQQKFYEIIMDIEQNNVQGKKGIQQLQKW
>ID13976-ABP_neg
YQWQRRMRLGAPSIT
>ID13998-NO
YTPNDITLNNSVALDPIDISIELNKAKSDLEESKE
>ID13999-NO
YTPNITLNNSVALDPIDISIELNKAKSDLEESKE
>ID14000-NO
YTSLIHSLIEEKQNQQEKNEQELLELDKWASLWNWF
>ID14003-ABP_pos
YVSCLFRGARCEVYSGRSCCFGYYCRRDFPGSYFGTCSRRNF

>ID14018-ABP_both
AAAAGSCVWGAVNYTSDCAAECKRRGYKGGHCGSFANVNCWCET
>ID14019-ABP_both
AAAAGSCVWGAVNYTSDCAAECKRRGYKGGHCGSFANVNCWCRT
>ID14022-ABP_both
AAAAGSCVWGAVNYTSDCNGECKRRGYKGGHCGSFANVNCWCET
>ID14023-ABP_both
AAAAGSCVWGAVNYTSDCNGECKRRGYKGGHCGSFANVNCWCRT
>ID14112-NO
AAKVKYSLTPAECCTNPPCFAQHSDLCGA
>ID14113-NO
AAKVKYSLTPAECCTNPPCFAQHSDLCGARR
>ID14115-NO
AAKVKYSNTPEECCPNPPCFATHSEICGVRR
>ID14116-NO
AAKVKYSNTPEECCSNPPCFATHSEICG
>ID14117-NO
AAKVKYSNTPEECCSNPPCFATHSEICGGRR
>ID14118-NO
AAKVKYSNTREECCPNPPCFATHSEICGGRR
>ID14128-ABP_both
AALKGCWTKSIPPKPCFGF
>ID14141-NO
AANAKLFAVMQSCCSTPPRALRHMDMCG
>ID14150-NO
AANAKLSERLDPCCREPPCASTHTDICTRRR
>ID14166-NO
AANDKASVQIALTVQECCADSACSLTNPLIC
>ID14167-NO
AANDKASVQIALTVQECCADSACSLTNPLICGRR
>ID14170-ABP_both
AANIPFKVHFRCKAAFC
>ID14211-NO
AASDKASELMALAVRGCCSNPACAGSNAHICGRRR
>ID14265-NO
ACDFQSCWVSCQRQYNIYFRKAYCEKSKCMCVYNYGG
>ID14290-NO
ACNFQSCWATCKAHYGIYFRRAYCDGPNCQCVHLTQD
>ID14294-NO
ACNTATCMTHRLAGWLSRSGSMVRSNLLPTKMGFKIFSGPRRNFWF
>ID14392-NO
AEGEFWGDSHWLQYWYEGDPAK
>ID14430-NO
AFCNLRRCQLSCRSLGLLGKCIGDKCECVKH
>ID14504-NO
AGCRNFFWKTFTSC
>ID14513-NO
AGEGLSSPFWSLAPQRF
>ID14544-NO
AGLFRRLRDSIRRGQQKILEKARRIGERIKDIFR
>ID14547-ABP_both

AGLQFPVGRVHRLLRK
>ID14609-NO
AGYLLGKLKALAALAKKIL
>ID14665-ABP_both
AIHKLAHKTLKKTLRAVKKLAN
>ID14716-ABP_both
AKAWGIPPHVIPQIVPVRIRPLGGNV
>ID14771-ABP_both
AKKVFKRLPKLFSKIWNWK
>ID14919-ABP_both
ALKGCWTKSIPPKPCFGK
>ID14991-NO
ALWKDILKNVGKAAGKAVLNTVTDMVNE
>ID14992-NO
ALWKDVLKKIGTVALHAGKAALGAVADTISE
>ID14994-ABP_both
ALWKKILKNAGKAALNKINQIVQ
>ID15014-ABP_neg
ALWMTLKKKVLKAAAKALNAVLVGANA
>ID15032-ABP_both
ALYKTMLKKLGTMAL
>ID15041-ABP_pos
AMDPTKYYGNGVYCNSKKCWVDWGQGSGCIGQTVVGGWLGGAIPGKC
>ID15042-ABP_pos
AMDPTKYYGNGVYCNSKKCWVDWGSASGCIGQTVVGGWLGGAIPGKC
>ID15533-NO
ASDRSNATLLDGPSGALLLRLVQLAGAPEPAEPAQPGVY
>ID15543-NO
ASFPWSCPSLSGVCRKVCLPTELFFGPLGCGKGFLCGVSHF
>ID15573-NO
ASLEPEYPGDNATPEQMAQYAAELRRYINMLTRPRY
>ID15624-NO
ATAEETRRMLHRAFDALA
>ID15639-ABP_pos
ATCDLASKFNWNHALCAAHCIARRYRGGYCNSKAVCVCR
>ID15640-ABP_pos
ATCDLASKFNWNHTLCAAHCIARRYRGGYCNSKAVCVCR
>ID15641-ABP_pos
ATCDLASKWNVNHALCAAHCIARRYRGGYCNSKAVCVCR
>ID15642-ABP_pos
ATCDLASKWNVNHTLCAAHCIARRYRGGYCNSKAVCVCR
>ID15646-NO
ATCDLLSGFGVGDSACAAHCIARRNRGGYCNKKKVCVCRN
>ID15647-NO
ATCDLLSGFGVNDSACAVHCILRGNRGGYCNSKKVCVCRN
>ID15730-NO
AVCNLKRCQLSCRSLGLLGKCIGDKCECVKA
>ID15732-NO
AVCNLRRCQLSCRSLGLLGKCIGVKCECVKH
>ID15753-NO
AVFCLGYLSPKLKDMEPKPRG

>ID15797-NO
AVNQHLCGSHLVEALYLVCGERGFFYSPKA
>ID15864-ABP_both
AWKETIRKYLKNEIKKKWRKAVIAW
>ID15868-ABP_both
AWKKTIRQYLKNKIKKKGRKAVIAW
>ID15869-ABP_both
AWKKTIRQYLKNKIKKKWRKAVIAW
>ID15922-NO
AYALCLTERQIKIWFANRRMKWKKEN
>ID16192-NO
CCKYGWTCWLGCSPCGC
>ID16229-NO
CCPGWELCCEWDEW
>ID16253-NO
CCRAACSPWLCLPCC
>ID16285-NO
CCSQDCSVCIPCCPPP
>ID16286-NO
CCSQDCSVCIPCCPW
>ID16298-NO
CCSRRCWVCIPCCPNGS
>ID16586-NO
CKGKGAKCSRLMFDCCTGSCRSGKC
>ID16588-NO
CKGKGAKCSRLMYACCTGSCRSGKC
>ID16590-NO
CKGKGAKCSRLMYDCCTGSCASGKC
>ID16591-NO
CKGKGAKCSRLMYDCCTGSCRSGAC
>ID16592-NO
CKGKGAKCSRLMYDCCTGSCRSGKCG
>ID16605-NO
CKGKGASCRRTSYGCCTGSCRSGRC
>ID16616-NO
CKGTGKPCSRIAYNCCTGSCRSGKCG
>ID16662-NO
CKSKGAKCSRLMYDCCSGSCSGTVGRC
>ID16663-NO
CKSKGAQCSKLMYDCCSGSCSGTVGRC
>ID16664-NO
CKSKGARCSKLMYDCCSGSCSGTVGRC
>ID16738-NO
CLGSREQCVRDTSCCSMSCTNNICF
>ID16785-NO
CLTTGEYCWLASSCCSYSCTNNVCF
>ID16915-NO
CRKKRRQRRR
>ID16940-ABP_both
CRRWQWRMKKLGC
>ID16946-NO

CRSKGAKCSKLMYDCCSGSCSGTVGRC
>ID17001-NO
CSNLSTCVLGTYTQDLNKFHTFPQTAIGVGAP
>ID17058-NO
CTHPGGACGGHSHCCSLSCNTAANSCN
>ID17063-NO
CTPAGGACDATTECCILFCNLATKKCQVPTFP
>ID17132-ABP_both
CVHWMTNTARTACIAG
>ID17189-ABP_both
CWTKSIPPRPC
>ID17197-ABP_both
CWWKKKKWWWC
>ID17252-NO
DAADVKPVARTNEGPGRDPAPCCQHPIETCC
>ID17272-ABP_both
DAEFRHDSGYEVRHQKLVFFAEDVGSNKGAIIGLMVGGVVIA
>ID17282-ABP_both
DAHKLAKLAKKLAKLAK
>ID17351-NO
DCGGQGKGCYTQPCCPGLRCRGGGTGGGVCQP
>ID17406-NO
DCTPPGGACGFYYHCCSNYCITISSTCR
>ID17407-NO
DCTPPGGACGFYYHCCSNYCVTISSTCN
>ID17415-NO
DCVGESQQCADWAGPHCCDGYYCTCRYFPKCICVNNN
>ID17553-NO
DECFSPGTFCGTKPGLCCSARCFSFFCISLEF
>ID17584-NO
DEGPYKMEHFRWGSPPKD
>ID17712-NO
DGSYKMNHFRWSGPPKD
>ID17834-ABP_both
DKLIGSCVWGAVNYTSDCAAECKRRGYKGGHCGSFANVNCWCET
>ID17835-ABP_both
DKLIGSCVWGAVNYTSDCAAECKRRGYKGGHCGSFANVNCWCRT
>ID17836-ABP_both
DKLIGSCVWGAVNYTSDCAAECLLRGYKGGHCGSFANVNCWCET
>ID17837-ABP_both
DKLIGSCVWGAVNYTSDCAAECLLRGYKGGHCGSFANVNCWCRT
>ID17841-ABP_both
DKLIGSCVWGAVNYTSDCNGECKRRGYKGGHCGSFANVNCWCRT
>ID17845-ABP_both
DKLIGSCVWGAVNYTSDCNGECLLRGYKGGHCGSFANVNCWCRT
>ID17907-NO
DLIWKLLSKAQEKFGKNGSR
>ID17909-NO
DLIWKLLSKAQEKFGKNKSG
>ID17914-NO
DLIWKLLSKAQGKFGKNKSR

>ID18163-NO
DSWMDEVIKLCGRELVRAQIAICGKSTWS
>ID18235-NO
DVLAGLSSNCCKWGCSKSEISSLC
>ID18251-NO
DVRTPALRLRF
>ID18260-NO
DVSTPPTVLPDNFPRYPVGKFFRYDTWKQSAQRL
>ID18353-NO
EAAGLLPFPRV
>ID18372-NO
EAEKCFKWQRNMRKVRGPPVSCIKR
>ID18386-NO
EAIIRILQQLLFIHFRIGRRRRRRRR
>ID18400-NO
EATKCFEWQRKMRKVRGPPVSCIKR
>ID18402-ABP_both
EATKCFQWQRNMRKVR
>ID18427-NO
ECCSNPACRVNNPHVC
>ID18437-NO
ECENPACGNHTSK
>ID18438-NO
ECENPACGQHTSK
>ID18472-NO
ECPPWCPTSHCNAGTC
>ID18529-NO
ECTRSGGACNSHDQCCNAFCDTATRTCV
>ID18533-NO
ECTRSGGACNSHTQCCDHFCSTATSTCI
>ID18572-NO
EDLIWKLLSKAQEKFGKNKSR
>ID18660-NO
EFEWDRICGYGTARCRNKCRSQEYRIGRCPNTFACCLRKWDESLLNSTKP
>ID18681-NO
EGCSSGGTFCGIHPGLCCSEFCFLWCITFID
>ID18782-ABP_both
EKCLRWQWAMRKVGG
>ID18783-ABP_both
EKCLRWQWAMRKYGG
>ID18785-ABP_both
EKCLRWQWEMRKYGG
>ID18820-NO
EKRACSKKWEYCIVPILGFVYCCPGLICGPFVCV
>ID19065-NO
EQFDDYGHMRF
>ID19085-NO
EQRLGNQWAVGHLM
>ID19293-NO
FAESLPSDEEGESYSKEVPEME
>ID19310-NO

FANQHLCGSHLVEALYLVCGERGFFYTPKA
>ID19343-NO
FCQAIGWGDPITHWSHGQ
>ID19356-NO
FDAFTTGFGHS
>ID19372-NO
FDEIDRSGFG
>ID19427-NO
FFDEKCGKLKGTCKNNCGKNEELIALCQKSLKCCRTIQPCGSIID
>ID19464-ABP_both
FFGWLIKGAIHAPKAIHPLIHRRRH
>ID19465-ABP_both
FFGWLIKPAIHAGKAIHGLIHRRRH
>ID19476-ABP_both
FFHHIFRGIVHVGKTAHRLVTG
>ID19477-ABP_both
FFHHIFRGIVHVGKTVHRLVTG
>ID19478-ABP_neg
FFHHIFRGKVHVGKTIHRLVTG
>ID19479-ABP_both
FFHHIFRGVVHVGKTIHRLVTG
>ID19483-ABP_both
FFHHIWRGIVHVGKTIHRLVTG
>ID19484-ABP_both
FFHHVFRGIVHVGKTIHRLVTG
>ID19522-ABP_neg
FFPIVKKILSGLF
>ID19552-ABP_both
FFSMIPKIAGGIASLVKNL
>ID19621-ABP_both
FIFHIIKGLFHAGKMIHGLVT
>ID19633-NO
FIGPVLKIATSILPTAICKIFKKC
>ID19635-ABP_pos
FIHHIIGGLISVGKHIHGLIHGH
>ID19658-NO
FIPGLRRLSATVVPTVVCAINKLPPG
>ID19704-ABP_both
FKARRWQWRMKK
>ID19705-ABP_both
FKAWRWAWRMKKLAAPS
>ID19711-ABP_both
FKCRRAQWRMKKLGA
>ID19713-ABP_both
FKCRRFQWRMKKLGA
>ID19714-ABP_both
FKCRRWQARMKKLGA
>ID19715-ABP_both
FKCRRWQFRMKKLGA
>ID19718-ABP_both
FKCRRWQWRMKKLGAPSITCVRRAFAL

>ID19724-ABP_both
FKCRRWQWRMKKLWA
>ID19733-ABP_both
FKFRRWQWRMKKLGA
>ID19739-ABP_both
FKHHIFRGIVHVGKTIHRLVTG
>ID19744-ABP_both
FKKFFKKLKNSVKKRAKKFFKKPRVIGVSIPF
>ID19776-ABP_both
FKSRRWQWRMKKLGAPSITSVRRAF
>ID19780-NO
FKVLGSVAKHLLPHVAPIIAEKL
>ID19785-ABP_both
FKWRRWQWRMKKLGA
>ID19896-ABP_both
FLGKVVKKASKVFPAVFGKV
>ID19903-NO
FLGSIIGALAKGLPSLIALIKK
>ID19925-ABP_both
FLGVVFKLASKVVPAVFGKV
>ID19926-ABP_both
FLGVVFKLAVKVFPAVFGKV
>ID19927-ABP_both
FLGVVFKLVSKVFPAVFGKV
>ID19928-ABP_both
FLGVVFKSASKVFPAVFGKV
>ID19929-ABP_both
FLGVVVKLASKVFPAVFGKV
>ID19939-ABP_both
FLKALFKALSKLL
>ID19943-ABP_both
FLKALWNVAKSVF
>ID19966-ABP_both
FLKVVFKLASKVFPAVFGKV
>ID19983-NO
FLPAVIRVAANVLPTVLCAISKKC
>ID19984-NO
FLPAVIRVAGNVLPTVFCAISKKC
>ID19985-NO
FLPAVIRVTANVLPTVFCAISKEC
>ID19995-NO
FLPFVGNLLKGL
>ID20017-ABP_both
FLPILASLAAKFGPKLFSLVTKKS
>ID20031-ABP_neg
FLPIVKKLLKGLF
>ID20051-NO
FLPLFLPKIICAITKKC
>ID20063-NO
FLPLLAGLAANFLPKLFCKITRKG
>ID20064-NO

FLPLLAGLAVNFLPKLFCKITRKC
>ID20081-NO
FLPMLAGLAANFLPKIFCKITRKC
>ID20096-ABP_both
FLRFAGSVIHGAGHLVHHIGVAL
>ID20127-ABP_both
FLSLIPHIVSGVASLAKHF
>ID20133-NO
FLSMIPHIVSGVAALAKHLG
>ID20134-ABP_both
FLSMIPKIAGGIASLVKNL
>ID20136-NO
FLSSLIPSAISGLISAFK
>ID20139-ABP_both
FLSTIWNGIKSLF
>ID20219-NO
FPRPRICNLACRTGIGYKYPFCHCR
>ID20261-ABP_both
FQWQRNMRK
>ID20314-ABP_both
FRRPFKWFRRFFKFF
>ID20318-ABP_both
FRSGILKLASKIGSVLCAVLKNC
>ID20368-NO
FSVLGSVAKHLLPHVAPIIAEKL
>ID20385-NO
FTMKKSLLLLFFLGTINLSLCEKERNAEEEKRDGDDETDVEVQK
>ID20393-ABP_both
FTQRVRNPQSCRWNMGVCIPFLCRVGMRQIGTCFGPRVPCCRR
>ID20422-ABP_both
FVGALFKALSKLL
>ID20441-NO
FVNQHLCGSHLVEALYLVCGERGFFYTPKS
>ID20458-NO
FVPIFTYSELQRMQEKERNRGQ
>ID20465-ABP_both
FVPWFSKFLWRIL
>ID20487-ABP_both
FWGALFKVASK
>ID20489-ABP_both
FWHHIFRGIVHVGKTIHRLVTG
>ID20538-ABP_both
FWRRIRVTPWVNPPFLQQT
>ID20563-NO
FYAGAVVNDL
>ID20628-NO
GAFGDFLKGAAKKAGLKILSIAQCKLFGTC
>ID20652-ABP_both
GALFKVASKVL
>ID20677-ABP_both
GAPKGCWTKSYPPKPCK

>ID20740-NO
GCCCNPACGPKYSC
>ID20748-NO
GCCDPQWCDAGCYDGCC
>ID20774-NO
GCCHPSTCHVRKGCSRCCS
>ID20873-NO
GCCSHPACAGNNPHICS
>ID20949-NO
GCCSPPCAANNPDYC
>ID20974-NO
GCCSYPPCFATNPDCAGGG
>ID20979-NO
GCCSYPPCFATNPDCGGAAGG
>ID21002-NO
GCGYLGEPCCVAPKRAYCHGDLECNSVAMCVN
>ID21008-ABP_both
GCKFTVKPYLKRFQVYYKGRMWCG
>ID21014-ABP_both
GCKKYRRFRWKFKGKFWFWGG
>ID21030-ABP_pos
GCPLNQGACHNHCRSIGRRGGYCAGIIKQTCTCYRK
>ID21047-ABP_both
GCRRLCYRQRCVTYCRGR
>ID21048-ABP_both
GCRRWCYKQRCVTYCRGR
>ID21062-NO
GCTCSWPVCTRNGLPVCGETCVGGTCN
>ID21063-NO
GCTHPGGACGGHHHCCSLFCNTAANACN
>ID21086-NO
GCWLCLGPNACCRGSVCHDYCPS
>ID21113-NO
GDEEYSKFIEREREAGRLDLSKFP
>ID21185-NO
GEEEYSKMAAELARENIAKGCKVNCYP
>ID21771-NO
GFFCPYNGYCDRHCRKKLRRRGGYCGGRWKLTCICVQ
>ID21774-ABP_both
GFFDRIKALTKNVTLELLNTITGKLPVTPP
>ID21789-ABP_pos
GFGCNGPWSEDDLRCHRHCKSIKGYRGGYCAKGGFVCKCY
>ID21797-ABP_pos
GFGCPFNQNECHAHCLSIGRKFGFCAGPLRATCTCGKQ
>ID21798-ABP_pos
GFGCPFNRNECHAHCLSIGRKFGFCAGPLRATCTCGKQ
>ID21799-ABP_both
GFGCPLNQGACHRHCRSIRRRGGYCSGIIKQTCTCY
>ID21805-NO
GFGCYRSCWKAGHDEETCKRECS
>ID21809-ABP_both

GFGKALKLLKKVL
>ID21812-NO
GFGMALKLLKKV
>ID21823-NO
GFGSLFKFLGKKVLKTVAKQAAKKQME
>ID21828-NO
GFGTILKALAKIAGKVVKKLATKPGATYMLKQNLQ
>ID21845-ABP_both
GFKDLLKGAAKALVKTVLK
>ID21850-ABP_both
GFKRIVQAIKDFLRNLV
>ID21852-ABP_both
GFKRIVQRIADFLRNLV
>ID21853-ABP_both
GFKRIVQRIKDFLANLV
>ID21870-ABP_both
GFLEKLKTGAKDFASAFVNSIK
>ID21871-NO
GFLETFKNLALNAAKSAGVSVLNSLSCKLSKTC
>ID21918-ABP_neg
GFRKRFNKLVKKVKHTIKETANVSKDVAIVAGSGVAVGAAM
>ID21927-ABP_both
GFSSIFRGVAKFASKGLGKKLAKLGVKLVACKISKQC
>ID21947-NO
GGAGEPLAFSPDMLSLRFG
>ID22064-ABP_both
GGLKKLGKKLEGAGKRVFNAAEKALPVVAGAKALRK
>ID22070-ABP_both
GGLRSLGRKILRAWKKYGPAIVPIIRI
>ID22094-NO
GGSGSGETSGMWFGPRL
>ID22120-NO
GGVCPKILKACRRDSDCPGACICRGNGYCGSGSD
>ID22121-ABP_both
GGVCPKILQRCRRDSDCPGACICRGNGYCGSGSD
>ID22136-NO
GHACYRNCWREGNDEETCKKEC
>ID22150-NO
GHGQLSHKRHKTDSFVGLM
>ID22198-NO
GIFDSIKEGFKNAAVTLLNKIKCKISECPPA
>ID22202-NO
GIFGKILGTGKKVLCGLSGLC
>ID22207-NO
GIFGKILGVGKKVLCGLSGMW
>ID22208-NO
GIFGKILGVGKKVLCGLSGTC
>ID22209-NO
GIFGKILGVGKKVPCGLSGMC
>ID22210-NO
GIFGKSSVWGRKYYADLAGCAKA

>ID22218-NO
GIFSKISGKAIKNLFIKGAKNTGKHVGMDVVRTGIDVVGCKIKGEC
>ID22222-NO
GIFSLIKTAAKFVGKNLLKQAGKAGMEHLACKANNQC
>ID22235-ABP_both
GIGAVLKVLTTGLCALISWIKRKRQQ
>ID22236-ABP_both
GIGAVLKVLTTGLPACISWCKRKRQQ
>ID22239-ABP_pos
GIGAVLKVLTTGLPALKSWIKRKRQQ
>ID22240-ABP_neg
GIGAVLLVLTTGLPALISWIKRKRQQ
>ID22242-ABP_neg
GIGAVLVVLTTGLPALISWIKRKRQQ
>ID22243-ABP_neg
GIGAVLWVLTTGLPALISWIKRKRQQ
>ID22249-NO
GIGGAILSAGKSALKGLAKGLAEHF
>ID22256-ABP_both
GIGGALLSFGKSALKGLAKGLAEHF
>ID22259-NO
GIGGALLSVGKSALKGLAKGLAEHF
>ID22273-NO
GIGGKILSGLKPALKGAAKELAFTYLH
>ID22274-NO
GIGGKILSGLKTALKGAAKELAATYLH
>ID22275-NO
GIGGKILSGLKTALKGAAKELAFTYLH
>ID22276-NO
GIGGKILSGLKTALKGAAKQLAATYLH
>ID22277-NO
GIGGRILGGLKTALKGAAKELAATYLH
>ID22280-ABP_both
GIGKALHSAKKFGKAFVGEIMNS
>ID22281-ABP_both
GIGKFAHSAKKFGKAFVGEIMNS
>ID22285-ABP_both
GIGKFLHSAKKAGKAFVGEIMNS
>ID22286-ABP_both
GIGKFLHSAKKFAKAFVGEIMNS
>ID22288-ABP_both
GIGKFLHSAKKFGKAAVGEIMNS
>ID22289-ABP_both
GIGKFLHSAKKFGKAFAGEIMNS
>ID22290-ABP_both
GIGKFLHSAKKFGKAFVGEAMNS
>ID22292-ABP_neg
GIGKFLHSAKKFGKAFVGEIMNSGGS
>ID22298-ABP_both
GIGKFLHSAKKPGKAFVGEIMNS
>ID22299-ABP_both

GIGKFLHSAKKWGKAFVGEIMNS
>ID22303-NO
GIGKFLKKAKKGIGAVLKVLTTG
>ID22309-ABP_both
GIGRKFLGGVKTTFRGGVKDFASKHLY
>ID22310-NO
GIGRKILGGLKTALKGAAKELAATYLH
>ID22315-NO
GIGTKFLGGLKTAVKGALKELASTYVY
>ID22319-NO
GIGTKIIGGLKTAVKGALKESAFTYVN
>ID22320-NO
GIGTKILGGVKTALKGALKELAFTYAN
>ID22321-NO
GIGTKILGGVKTALKGALKELAFTYVN
>ID22322-NO
GIGTKILGGVKTALKGALKELAPTYVN
>ID22323-NO
GIGTKILGGVKTALKGALKGLASTYAN
>ID22352-ABP_both
GIKDILKYGKPS
>ID22353-ABP_neg
GIKDLLKGAAKALVKTVLF
>ID22367-NO
GILDTFKNLALNAAKSAGVSVLNALSCKLSKTC
>ID22368-NO
GILDTFKNLALNAPKSAGVSVLNALSCKLSKTC
>ID22372-ABP_both
GILDTLKQFAKGVGKDLVKGAAQGVL
>ID22376-ABP_both
GILDTLKQFAKGVGKDLVKGAAQGVLSTVS
>ID22378-ABP_both
GILDTLKQFAKGVGKWLVKGAAQGVLSTVSCKLAKTC
>ID22396-NO
GILSAFKEFGKTAAKGIAQSLLSTASCKLAKTC
>ID22403-ABP_both
GILSSFKDVAKGVAKNVAAQLLDKLKCKITGC
>ID22412-NO
GIMDTLKNLAKTAGKGALQSLLKMASCKLSGQC
>ID22413-NO
GIMDTVKNAAKDLAGQLLDKLKCKITAC
>ID22415-ABP_both
GIMDTVKNAAKNLAGQLLDKLKCKITAC
>ID22419-ABP_both
GIMSSLMKKLAKHIAK
>ID22422-ABP_both
GIMSSLMKKLKAHIAK
>ID22424-ABP_both
GIMSSLMKKLKKIIAK
>ID22493-ABP_neg
GIRDVLKGAAKAFVKTVAGHIAN

>ID22502-NO
GISDPVTCLKSGAICHPVFCPRRYKQIGTCGLPGTKCCKKP
>ID22518-NO
GIVEQCCASVCSLYQLEHYCN
>ID22526-NO
GIVEQCCHRPCNIFDLQNYCN
>ID22532-NO
GIVEQCCTGVCSLYQLENYCN
>ID22547-ABP_both
GIWDTIKSMGKVFAGLILQNL
>ID22551-ABP_both
GIWKTIKSMGKVFAGKIKQNL
>ID22636-ABP_both
GKLGPLLKIAAKVGSKLL
>ID22637-ABP_neg
GKLGPLLKIAAKVGSNLL
>ID22641-ABP_both
GKLSSLWKKLKKIIAK
>ID22669-ABP_neg
GKPRPYSPRPTSHPKPIRV
>ID22671-ABP_both
GKPRPYSPRPTSHPRPIR
>ID22678-ABP_both
GKPRPYTPRPTSHPRPIRV
>ID22745-ABP_both
GLADVIKKVASVIGGL
>ID22764-NO
GLDLGLSRGFSGSQAAKHLMGLAAANYAGGP
>ID22778-NO
GLFAVIKHVASVIGGL
>ID22782-ABP_both
GLFAVIKKVASVIGG
>ID22794-ABP_both
GLFDIVKKVVGAVGSL
>ID22795-NO
GLFDIVKKVVGTLAG
>ID22806-ABP_both
GLFDVIKKVASKIGGL
>ID22810-ABP_both
GLFDVIKKVASVIG
>ID22811-ABP_both
GLFDVIKKVASVIGGA
>ID22812-ABP_both
GLFDVIKKVLKKIGGL
>ID22815-ABP_both
GLFEVIKKVASVIGGL
>ID22822-ABP_both
GLFGVLAKVAAHVVPAIAE
>ID22823-ABP_both
GLFGVLAKVAAHVVPAIAEH
>ID22833-NO

GLFLDTLKGAAKDVAGKLLEGLKCKITGCKP
>ID22835-NO
GLFNVFKGALKTAGKHVAGSLLNQLKCKVSGEC
>ID22860-NO
GLFSILKIGAKVIGKSLLKQAGKAGMEYAACKATNQC
>ID22867-NO
GLFSKFAGKGIKNFIIKGVKHIGKEVGMDVIRTGIDVAGCKIKGEC
>ID22868-NO
GLFSKFAGKGIKNFLFKGVKHIGKEVGMDVIRTGIDVAGCKIKGEC
>ID22869-NO
GLFSKFAGKGIKNFLIEGVKHIGKEVGMDVIRTGIDVAGCKIKGEC
>ID22870-NO
GLFSKFAGKGIKNFLIKGVKHIGKEVGMDVIGTGIDVAGCKIKGEC
>ID22871-NO
GLFSKFAGKGIKNFLIKGVKHIGKEVGMDVIRTGIDVAGCKIKGVC
>ID22872-NO
GLFSKFAGKGIKNLIFKGVKNIGKEVGMDVIRTGIDVAGCKIKGEC
>ID22873-NO
GLFSKFAGKGTKNFIIKGVKHIGKEVGMDVIRTGIDVAGCKIKGEC
>ID22874-NO
GLFSKFPGKGIKDLIFKGVKHIGKEVGMDVIRTGIDVPGCKIKGEC
>ID22882-NO
GLFSVVKGVLKGVGKNVAGSLLEQLKCKISGGC
>ID22890-NO
GLFTLIKGAAKLIGKTVAKEAGKTGLELMACKITHQC
>ID22891-NO
GLFTLIKGAAKLIGKTVAKEAGKTGPELMACKITNQC
>ID22893-NO
GLFTLIKGAAKLIGKTVAKEAGRTGLELMACKITNQC
>ID22894-NO
GLFTLIKGAAKLIGKTVAKKAGKTGLELMACKITNQC
>ID22895-NO
GLFTLIKGAAKSIGKTVAKEAGKTGLELMACKITNQC
>ID22915-NO
GLGSVLGKILKMGVNLLGGAPKQ
>ID22924-NO
GLIGSIGKALGGLLVDVLKPKLQAA
>ID22934-NO
GLIWKLLSKAQEKFGKNKSR
>ID22937-ABP_both
GLKALKKVFKGIHKAIKLINNHVQ
>ID22949-NO
GLLDFAKHVIGIASK
>ID22954-NO
GLLDPIKNMALNAAKSAGVSVVNTLSCKLSKTC
>ID22955-ABP_both
GLLDSVKEGLKKVAGQLLDTLKCKISGCTGA
>ID22957-NO
GLLDTFKNLALNAAKSAGVSVLNSLSCKLPKTC
>ID22962-NO
GLLDTLKNMAINAAKGAGVSVLSALSCKLSKTC

>ID22976-ABP_both
GLLGPLLIAAKVGSNLL
>ID22978-NO
GLLGTVKDLLIGAGKSAAQSVLKGLSCKLSKDC
>ID23033-NO
GLLNVIRDTAQNLFAAALEKLKCKVTKCN
>ID23076-NO
GLLSGILGAGKHVVCGLSGLC
>ID23078-NO
GLLSGILGAGKNIVCGLSGPC
>ID23079-NO
GLLSGILGAGTNIVCGLSGLS
>ID23080-NO
GLLSGILGTGKHIVCGLSGLC
>ID23081-NO
GLLSGILGVGKHIVCGLSGLC
>ID23082-NO
GLLSGILSAGKHIVCGLSGLC
>ID23083-NO
GLLSGIPGAGKHIVCGLSGLC
>ID23086-NO
GLLSGTLGAGKNIVCGLSGLC
>ID23089-NO
GLLSGVLGAGKHIVCGLSGLC
>ID23092-NO
GLLSGVLGVGKKIVCGLSGPC
>ID23093-NO
GLLSGVLGVGKKIVCGLSGRC
>ID23094-NO
GLLSGVLGVGKKVPCGLSGLC
>ID23096-NO
GLLSKILGVGKKVLCGLSGMC
>ID23097-NO
GLLSKILGVGKKVLCGVSGLC
>ID23111-ABP_both
GLLSVLGSVAKHVLAHVVAVIAEHL
>ID23112-ABP_both
GLLSVLGSVAKHVLGHVVGVIAEHL
>ID23113-ABP_both
GLLSVLGSVAKHVLGHVVPVIAEHL
>ID23116-ABP_both
GLLSVLGSVAKHVLPHVVGVIAEHL
>ID23126-NO
GLMDAAKNVAKNVAATLLDKLKCKITGGC
>ID23138-ABP_both
GLNALKKVFQGFHEAIKLINNHVQ
>ID23142-ABP_both
GLNALKKVFQGIHEAIKLINKHVQ
>ID23143-ABP_both
GLNALKKVFQGIHEAIKLINNHVK
>ID23147-ABP_both

GLNALKKVFQKIHEAIKLINNHVQ
>ID23149-ABP_both
GLNALKKVFQPIHEAIKLINNHVQ
>ID23150-ABP_both
GLNALKKVFQPIHKAIKKINNHVQ
>ID23163-ABP_both
GLPALISWIKRKRQ
>ID23182-ABP_both
GLPTCGETCFKGKCYTPGCSCSYPICKKD
>ID23209-NO
GLPVCGETCVGGTCATPGCTCSWPVCTRN
>ID23210-NO
GLPVCGETCVGGTCDTPGCTCSWPVCTRN
>ID23212-NO
GLPVCGETCVGGTCNAPGCTCSWPVCTRN
>ID23213-NO
GLPVCGETCVGGTCNSPGCTCSWPVCTRN
>ID23214-NO
GLPVCGETCVGGTCNTAGCTCSWPVCTRN
>ID23217-NO
GLPVCGETCVGGTCNTPACTCSWPVCTRN
>ID23227-NO
GLPVCGETCVGGTCNTPGCTCAWPVCTRN
>ID23252-NO
GLPVCGETCVGGTCNTPGCTCSWPACTRN
>ID23254-NO
GLPVCGETCVGGTCNTPGCTCSWPVCARN
>ID23256-NO
GLPVCGETCVGGTCNTPGCTCSWPVCTRA
>ID23258-NO
GLPVCGETCVGGTCNTPGCTCSWWPVCTRN
>ID23260-NO
GLPVCGETCVGGTCNTPGCTCSYPVCTRN
>ID23268-NO
GLPVCGETCVGGTCNYPGCTCSWPVCTRN
>ID23271-NO
GLPVCGETCVGGTCYTPGCTCSWPVCTRN
>ID23273-NO
GLPVCKGKGAKCSRLMYDCCTGSCRSGKCTRG
>ID23300-ABP_neg
GLRKRLRKFRNKIKEKLKKIGQKNQGLLPKLAPRTDY
>ID23301-ABP_neg
GLRKRLRKFRNKIKEKLKKNGQKIQGLLPKLAPRTDY
>ID23302-ABP_neg
GLRKRLRKFRNKIKEKLPKIGQKIQGLLPKLAPRTDY
>ID23303-ABP_neg
GLRKRLRKFRNKIKEKPKKIGQKIQGLLPKLAPRTDY
>ID23304-ABP_neg
GLRKRLRKFRNKMKEKLKKIGQKIQGLLPKLAPRTDY
>ID23305-ABP_neg
GLRKRLRKFRNKPKEKLKKIGQKIQGLLPKLAPRTDY

>ID23306-ABP_neg
GLRKRLRKFRNKRKEKLKKIGQKIQGLLPKLAPRTDY
>ID23308-ABP_neg
GLRKRPRKFRNKIKEKLKKIGQKIQGLLPKLAPRTDY
>ID23342-NO
GLWDSVKEGLKNAAVTILNKIKCKIFECPPA
>ID23343-NO
GLWDSVKEGLKNAAVTILNKIKCKISECPPA
>ID23344-NO
GLWDTIKQAGKKLFLNVLDKIRCKVAGGCRT
>ID23359-NO
GLWRALWRALWRSLWKSKRKV
>ID23360-NO
GLWRALWRGLRSLWKKKRKV
>ID23388-ABP_neg
GMASKAGAIAGKIAKVAWKAL
>ID23391-ABP_neg
GMASKAGAIAGKIAKVAWKALGGS
>ID23398-ABP_both
GMASLWAKVLPHVVKLIK
>ID23400-NO
GMASTAGSVLGKLAKVAIGAL
>ID23431-NO
GMRLTYNRPCIYATKRTKEM
>ID23446-ABP_both
GMWSKILGHLI
>ID23478-ABP_both
GNNRPVYIPQPRPPHPAL
>ID23486-ABP_both
GNNRPVYIPRPRPPHPRLV
>ID23493-ABP_both
GNNRPVYLPQPRPPHPRI
>ID23494-ABP_neg
GNNRVYIPQPRPHPRL
>ID23548-NO
GPFTLIKGAAKLIGKTVAKEAGKTGLELMACKITNQC
>ID23574-NO
GPLSGILGAGKHIVCGLSGLC
>ID23709-NO
GRCCHPACGQNTSC
>ID23712-NO
GRCCNPACGQNTSC
>ID23715-NO
GRCEHPACGNNTSK
>ID23733-ABP_both
GRFKRFRKKFKKAFKKASPVIPLLHL
>ID23734-ABP_both
GRFKRFRKKFKKLFKKASPVIPLLHL
>ID23749-ABP_both
GRFRRLRKKTRKRLKKIGKVLKWI
>ID23772-NO

GRKKRRQRRRPPQC
>ID23854-ABP_neg
GRRRRSVQWCAVSQPEATKCFQWQRNMRRVRGPPVSCIKRDSPIQCI
>ID23939-ABP_neg
GSKKPVPIIYCNRRSAKCQRM
>ID23940-ABP_neg
GSKKPVPIIYCNRRTAKCQRM
>ID23941-ABP_neg
GSKKPVPIIYCQRRSGKCQRM
>ID23949-ABP_both
GSLHGFMYRYLKNMVLNLF
>ID23977-NO
GSSFLSPEHQRAQQRKESKKPPAKLQPR
>ID23984-NO
GSSGLIAFPRL
>ID23999-NO
GSSSGLISMPRV
>ID24006-ABP_both
GSTLACRQSHGSCSFVACRAPSVDIGTCRGGKLKCCKWAPSS
>ID24076-NO
GTSKGCFGLKLDRIGAMSGLGC
>ID24130-NO
GVFTLIKGATQLIGKTLGKELGKTGLELMACKITKQC
>ID24166-NO
GVLGTVKNLLIGAGKSAAQSVLKGLACKLSNDC
>ID24168-NO
GVLGTVKNLLIGTGKGAAQSVLKTLSCKLSNDC
>ID24169-NO
GVLGTVKNLLIGTGKSAAQSVLKTLSCKLSNDC
>ID24194-NO
GVPGTVKDLLIGAGKSAAQSVLKALSCKLSNDC
>ID24225-ABP_both
GVVDILKGAAKDIAGHLAAKVMNKL
>ID24226-ABP_both
GVVDILKGAAKDIAGHLASAVMNKL
>ID24227-ABP_both
GVVDILKGAAKDIAGHLASKAMNKL
>ID24228-ABP_both
GVVDILKGAAKDIAGHLASKVANKL
>ID24229-ABP_both
GVVDILKGAAKDIAGHLASKVMAKL
>ID24230-ABP_both
GVVDILKGAAKDIAGHLASKVMNAL
>ID24232-ABP_both
GVVDILKGAAKDIAGHLASKVMNKA
>ID24233-ABP_both
GVVDILKGAAKDIAGHLLSKVMNKL
>ID24234-ABP_both
GVVDILKGAAKDILGHLASKVMNKL
>ID24236-ABP_both
GVVDILKGALKDIAGHLASKVMNKL

>ID24237-ABP_both
GVVDILKGLAKDIAGHLASKVMNKL
>ID24238-NO
GVVDQCCTSICSLYQLQNYCN
>ID24249-ABP_both
GVVVRVPRVVVRWVRR
>ID24250-ABP_both
GVVVRWGRVIVRGVRR
>ID24323-NO
GWMSKIASGIGTFLSGVQQ
>ID24343-NO
GWTLNSAGYLLGKLKALAALAKKIL
>ID24346-NO
GWTLNSAGYLLGPHAIDNHRSFNDKHGLA
>ID24352-NO
GWTLNSAGYLLGPINLKALAALAKKIL
>ID24459-NO
HADGSFSDEMNTILDSLATRDFINWLIQTKITD
>ID24463-NO
HADGSFSDEMNTVLDTLATRDFINWLLQTKITD
>ID24477-NO
HADGTYTSNVSTYLQDQAAKDFVSWLKSGRA
>ID24480-NO
HADGVFTSDYSRLLGQLSARKYLESLI
>ID24492-NO
HAEGTYTSDITSYLEGQAAKEFIAWLVNGRG
>ID24536-ABP_both
HFLGTLVKLAKKIL
>ID24537-ABP_both
HFLGTLVNKAKKIL
>ID24539-ABP_both
HFLGTLVNLAKKIK
>ID24541-ABP_both
HFLGTLVNLAKKKL
>ID24543-ABP_both
HFLGTLVNLKKKIL
>ID24580-ABP_both
HGVSGHGQHGTHG
>ID24660-ABP_both
HKLGTLVNLAKKIL
>ID24671-ABP_both
HKWMSLLKHILK
>ID24787-NO
HSDGIFTDSYSRYRKQMAVKKYLAAVLGKRYKQRIKNK
>ID24789-NO
HSDGIFTDSYSRYRKQMAVQKYLAAVLGRRYRQRVRNK
>ID24793-NO
HSDGTFTSELSRLRESARLQRLLQGLV
>ID24794-NO
HSDGTFTSEYSRLRDSARLQRLLQGLV
>ID24800-NO

HSEGTFSNDYSKYLETRRAQDFVQWLMNS
>ID24825-NO
HSQGTFTSDYSKYLDTRRAQDFVQWLMNT
>ID24866-NO
HWSYGLRPG
>ID24889-NO
HYRIKPTFRRLAWKYKGKFW
>ID24932-ABP_neg
IARRALKKAKRAAHKIPACKKFGRR
>ID24935-ABP_neg
IARRALKKAKRARHTIPQCKKFGRR
>ID24939-ABP_neg
IARRNLCASLRARHTIPQCKKFGRR
>ID25069-ABP_both
IFGAIWPLALGALKNLIK
>ID25074-NO
IFKAIWSGIKRLC
>ID25083-ABP_neg
IFPIVKKLLNGLF
>ID25166-ABP_pos
IIGAVLKVLTTGLPALISWIKRKRQQ
>ID25169-NO
IIGHLIKTALGFLG
>ID25194-NO
IIRILQQLLFIHFRIGRRRRRRRR
>ID25227-ABP_both
IKHQGLPQEV
>ID25274-ABP_both
IKKIVSKIKKVLK
>ID25288-ABP_both
IKKWLSKIKKLLK
>ID25297-ABP_both
IKLSPKTKDNLKKVLKGAIKGAIAVAKMV
>ID25298-ABP_both
IKLSPKTKKNLKKVLKGAIKGAIAVAKMV
>ID25389-NO
ILGPVLGLVGNALGGLIKEI
>ID25393-NO
ILGPVLGLVGNPLGGLIKKI
>ID25394-NO
ILGPVLGLVGNTLGGLIKKI
>ID25400-NO
ILGPVLGLVSGTLDDVLGIL
>ID25406-NO
ILGSVLGLVGNALGGLIKKI
>ID25459-NO
ILPLLGNLLNGLL
>ID25493-ABP_neg
ILPWKWAWWAWRR
>ID25499-ABP_both
ILPWKWKWWKWRR

>ID25563-NO
ILPWKWPWWPWP
>ID25566-ABP_both
ILPWKWPWWPWR
>ID25675-NO
INLKALAALAKKI
>ID25700-NO
INWLKLGKKILGA
>ID25701-NO
INWLKLGKKMMSA
>ID25733-NO
IPIYEKKYGQVPMCDAGEQCAVRKGARIGKLCDCPRGTSCNSFLLK
>ID25808-NO
IRDECCSNPACRANNPHVC
>ID25810-NO
IRDECCSNPACRVANPHVC
>ID25812-NO
IRDECCSNPACRVNAPHVC
>ID25814-NO
IRDECCSNPACRVNNAHVC
>ID25815-NO
IRDECCSNPACRVNNPAVC
>ID25816-NO
IRDECCSNPACRVNNPHAC
>ID25817-NO
IRDECCSNPACRVNNPHVCRRR
>ID25913-NO
ISGPVLGLVGSALGGLIKKI
>ID25922-NO
ISINQDLKAITDMLLTEQIQARRRCLDALRQRLLDL
>ID25942-NO
ISRPPGFSPFR
>ID26104-NO
IWNHGNLTLGEWYNQTKDLQQKFYEIITMDIEQNNV
>ID26202-ABP_both
KALWKTLLKKVLKA
>ID26203-ABP_both
KALWKTMLKKLGTMAL
>ID26214-NO
KAPSGRMSIIKNLQNLDPSHRISDRDYMGWMDF
>ID26215-NO
KAPSGRMSVIKNLQNLDPSHRISDRDYMGWMDF
>ID26244-NO
KAVRSPSLRLRF
>ID26249-NO
KAYSMPRCKYLFRAVLCW
>ID26265-NO
KCFQWQRNMRKVRGPPVSSIKR
>ID26297-ABP_both
KCRRLCYRQRCVTYCRGR
>ID26319-NO

KDEPQRRSARLSAKPAPPKPEPKPKKAPAKKC
>ID26363-NO
KEDLIWKLLSKAQEKFGKNKSR
>ID26421-ABP_both
KFFRKLKKSVKKRAKKFFKKPRVIGVSIPF
>ID26427-ABP_both
KFHHIFRGIKHVGKTIHRLVTG
>ID26428-ABP_both
KFHHIFRGIVHVGKTIHRLVTG
>ID26517-ABP_both
KGLRKLGRKILRAWKKGGPIIVPIIRI
>ID26518-ABP_both
KGLRKLGRKILRAWKKYGPIIVPIIRI
>ID26528-NO
KGRGKRRREKQRPSDKPRR
>ID26592-ABP_both
KIAGKIAKKAGKIAK
>ID26593-ABP_both
KIAGKIAKKAGKIAKIA
>ID26594-ABP_both
KIAGKIASIAGKIAKIAGKIAK
>ID26595-ABP_both
KIAGKIASIAGKIAKIAGKIAKIA
>ID26596-ABP_both
KIAGKIASIAGKIAKIAGSIAKIAGK
>ID26597-ABP_both
KIAGKIASIAGKIAKIAGSIAKIAGKIA
>ID26602-ABP_both
KIAKVALKALKIAKGALKAL
>ID26662-ABP_both
KILSSLLKKLKKIIAK
>ID26727-ABP_both
KKALLALALHHLALLALHLAHALKKA
>ID26758-ABP_both
KKFGKAFVKILK
>ID26774-ABP_both
KKIGKKIERVGQHTRDATIQTIAVAQQAANVAATLKG
>ID26808-ABP_both
KKKKKKKKKKK
>ID26873-NO
KKLFKKILKLL
>ID26874-NO
KKLFKKILKQL
>ID26875-NO
KKLFKKILKRL
>ID26876-NO
KKLFKKILKTL
>ID26942-NO
KKLLKWLKKL
>ID27013-ABP_pos
KKVVFKVKFKKK

>ID27034-ABP_both
KKWMSLLKHILK
>ID27070-ABP_both
KLAGLAKKWAGLAKKLAGLAK
>ID27078-NO
KLALKLALKALKAALKLAGC
>ID27101-ABP_neg
KLFKKIGIGAVLKVLTTGLPALKLTLK
>ID27104-ABP_both
KLFKRIVKRILKFLRKLV
>ID27124-NO
KLIKGRTPIKFGKADCDRPPKHSQNGMGK
>ID27127-ABP_both
KLILILSKTIPAGKNLFYKI
>ID27132-ABP_both
KLIWILSKTIPAIKNLFYKI
>ID27139-ABP_both
KLKKCLCLLLLKKLKK
>ID27157-ABP_both
KLKKLLKKLLKL
>ID27190-ABP_both
KLLKRIKTLL
>ID27218-ABP_pos
KLPLIGRVLSGIL
>ID27266-ABP_neg
KLWKLFKKIGIGAVLKVLTTGLPALKLTLK
>ID27294-NO
KMDSRWRWKCCKK
>ID27296-NO
KMDSRWRWKSCKK
>ID27471-ABP_both
KQWRIRVAVIRA
>ID27508-ABP_both
KRFKKFFRKLKKSVKKRKKEFKKKPRVIKVSIPF
>ID27531-NO
KRHHGYKRKFH
>ID27563-ABP_both
KRIVQRIKDWLR
>ID27604-ABP_pos
KRKKHRCRVYNNGLPAGLYRWC
>ID27605-ABP_pos
KRKKHRCRVYNNGLPTALYRWC
>ID27606-ABP_pos
KRKKHRCRVYNNGLPTGAYRWC
>ID27607-ABP_pos
KRKKHRCRVYNNGLPTGLARWC
>ID27608-ABP_pos
KRKKHRCRVYNNGLPTGLFRWC
>ID27609-ABP_pos
KRKKHRCRVYNNGLPTGLSRWC
>ID27610-ABP_pos

KRKKHRCRVYNNGLPTGLWRWC
>ID27611-ABP_pos
KRKKHRCRVYNNGLPTGLYAWC
>ID27612-ABP_pos
KRKKHRCRVYNNGLPTGLYRAC
>ID27613-ABP_pos
KRKKHRCRVYNNGLPTGLYRWA
>ID27614-ABP_pos
KRKKHRCRVYNNGLPTGLYRWC
>ID27644-NO
KRLFKKLLKFSLRKY
>ID27728-ABP_both
KSDVRRWRSRY
>ID27768-NO
KTCENLADTYKGPCFTTGSCDDHCKNKEHLRSGRCRDDFRCWCTKNC
>ID27890-ABP_pos
KVVFKVKFKKK
>ID27930-ABP_both
KWCFRVCYRGACYRRCR
>ID27931-ABP_both
KWCFRVCYRGGCYRRCR
>ID27932-ABP_both
KWCFRVCYRGICARRCR
>ID27933-ABP_both
KWCFRVCYRGICYARCR
>ID27934-ABP_both
KWCFRVCYRGICYRACR
>ID27935-ABP_both
KWCFRVCYRGICYRRCA
>ID27936-ABP_both
KWCFRVSYRGIAYRRCR
>ID27937-ABP_both
KWCFVCYRGICYRRCG
>ID27938-ABP_both
KWCGRVCYRGICYRRCR
>ID27940-ABP_both
KWCSRVCYRGICYRRCR
>ID28004-ABP_both
KWKLFKKIHKVGQNIRKGIIKAGPAVAVVGQAAQIAK
>ID28005-NO
KWKLFKKILKVL
>ID28016-NO
KWKLLKKIGAVLKVL
>ID28023-NO
KWKSFIKKGTSKFLHSAKKF
>ID28024-NO
KWKSFIKKLTLKFLHSAKKF
>ID28028-NO
KWKSFIKKLTSKFLHSAKKS
>ID28031-ABP_both
KWKSFLKTFKSAEKTVLHTALKAISS

>ID28032-ABP_neg
KWKSFLKTFKSAKKTALHTALKAISS
>ID28033-ABP_neg
KWKSFLKTFKSAKKTELHTALKAISS
>ID28034-ABP_neg
KWKSFLKTFKSAKKTGLHTALKAISS
>ID28035-ABP_neg
KWKSFLKTFKSAKKTKLHTALKAISS
>ID28037-ABP_neg
KWKSFLKTFKSAKKTLLHTALKAISS
>ID28038-ABP_neg
KWKSFLKTFKSAKKTSLHTALKAISS
>ID28039-NO
KWKSFLKTFKSAKKTVLHTAAKAISS
>ID28064-ABP_both
KWKVFKKIEKMIRNIRNKIVK
>ID28065-NO
KWKVFKKIEKNGRNIRNGIVKAGPAIAVLGEAKAL
>ID28131-ABP_both
KWSFRVCYRGICYRRAR
>ID28281-NO
LALERRDGWLRLFGLKTRRKH
>ID28333-NO
LCHNSISCMMGGDSTCNNVCVRQGNPSGGRCLPRDGCPGYDICACYPNS
>ID28517-ABP_neg
LFPIVKKLLNGLF
>ID28518-ABP_neg
LFPMVKKLLSGLF
>ID28545-ABP_both
LFWKLLLKALRLWAKVL
>ID28550-ABP_both
LGALFKVASKVLP
>ID28662-NO
LIIFRILISHKK
>ID28758-ABP_both
LKGCWTKSIPPKPCFGK
>ID28768-ABP_both
LKKAKKFGKAFV
>ID28769-ABP_both
LKKAKKFGKAFVKILKK
>ID28798-ABP_both
LKKLLKKLLK
>ID28830-ABP_both
LKLKKCCLLLLKKLKK
>ID28951-NO
LLGDFFRKSKEKIGKEFKRIVQRIKDFLRNLVPRTESC
>ID28965-NO
LLGMIPVAISAISALSKLG
>ID28980-NO
LLIILRRRIARKQAHAHSK
>ID28986-NO

LLIILRRRIRKQAHAHSA
>ID29000-ABP_both
LLKKLLKKLLKL
>ID29002-ABP_both
LLKKLLKLLLKL
>ID29009-ABP_both
LLKRIKTLL
>ID29050-ABP_both
LLPIVGNLLKALL
>ID29074-ABP_both
LLRKRLRKFRNKIKEKLKKIGQKIQGFVPKLVPRTES
>ID29114-ABP_both
LNALKKVFQKIHEAIKKI
>ID29222-NO
LPPGFTPFR
>ID29246-NO
LPVCGETCVGGTCNTPGCTCSWPVCTRN
>ID29308-NO
LQQLLFIHARIGRRRRRRRR
>ID29309-NO
LQQLLFIHFAIGRRRRRRRR
>ID29310-NO
LQQLLFIHFRAGRRRRRRRR
>ID29311-NO
LQQLLFIHFRIARRRRRRRR
>ID29312-NO
LQQLLFIHFRIGRRRRRRRR
>ID29333-ABP_both
LRCMCIKTTSGIHPK
>ID29344-NO
LRDLVCYCRKRGCKRREHMNGTCRKGHLLYTLCCR
>ID29347-NO
LRDLVCYCRSRGCKGRERMNGTCRKGHLLYMLCCR
>ID29349-NO
LRDLVCYCRTRGCKRRERMNGTCRKGHLIYTLCC
>ID29350-NO
LRDLVCYCRTRGCKRRERMNGTCRKGHLMHTLCCR
>ID29436-NO
LRWCIPSGELCFRSDHIGCCSGKCAFVCL
>ID29464-NO
LSDDMPATPADQEMYRQDPEQIDSRTKYFSPRL
>ID29468-ABP_both
LSFPTTKTYFPHF
>ID29470-ABP_both
LSFPTTKTYFPHFDLSHGSAQVKGHGAKVAAAL
>ID29525-NO
LSVLGSVAKHVLPHVVPVIAEHL
>ID29527-NO
LSWDLPEPRSRAGKIRVHPRGNLWATGHFM
>ID29548-NO
LTFSDWWKLLAE

>ID29627-NO
LVNQHLCGSHLVEALYLVCGERGFFYTPKA
>ID29696-ABP_pos
LWRRWIRWL
>ID29737-NO
LYKKIIKKLLES
>ID30162-NO
MAQDIISTISDLVKWIIDTVNKFTKK
>ID30194-ABP_both
MAQKIISTIGKLVKWIIKTVNKFTKK
>ID30367-NO
MCMPCFTTDPNMANKCRDCCGGGKKCFGPQCLCNR
>ID30384-NO
MDCRWRWKCCKK
>ID31923-NO
MIIYRDLIS
>ID32602-ABP_both
MLLKKLLKKLLKKM
>ID32958-NO
MQFITDLIKKAVDVFKGLFGNK
>ID33695-NO
MVVRGGCCSHPVCYFNNPQMCRGRR
>ID33710-ABP_both
MWSKILGHLIR
>ID33850-NO
NDPCYEVCLQHTGNVKACEEACQ
>ID33887-NO
NFDEIDRAGMGF
>ID33888-NO
NFDEIDRAGMGFA
>ID33893-NO
NFDEIDRSGF
>ID33899-NO
NFDEIDRSGFGFA
>ID33900-NO
NFDEIDRSGFGFH
>ID33901-NO
NFDEIDRSGFGFN
>ID33902-NO
NFDEIDRSGFGFV
>ID33906-NO
NFDEIDRSGFV
>ID33911-NO
NFDEIDRSSFG
>ID33913-NO
NFDEIDRSSFGFA
>ID33914-NO
NFDEIDRSSFGFH
>ID33915-NO
NFDEIDRSSFGFN
>ID33916-NO

NFDEIDRSSFGFV
>ID33956-NO
NGGTSGLFAFPRV
>ID33994-ABP_both
NGVQPWYKWWKWWKKWW
>ID34386-NO
NNAEVVNHLLKNFGTLDRLGDV
>ID34395-ABP_both
NNEAQCEQAGGRCSKDHCFHLHTRAFGHCQRGVPCCRRVYD
>ID34453-ABP_both
NNRPVYIPQPRPPHPRI
>ID34466-NO
NPAEDLARYYSALRHYINLITRQRY
>ID34475-ABP_neg
NPEKALEPLIAIQIAIKGMLNGWFTGVGFRRKR
>ID34557-NO
NRLSWCIPSGDLCFPSDHIQCCNAKCAFVCL
>ID34558-NO
NRLSWCIPSGDLCFPSDHIQCCSAKCAFVCL
>ID34583-NO
NRVYIHPFHL
>ID34594-NO
NSCKGAGAKCSRLPYDCCTGSCRSGAC
>ID34665-ABP_both
NTLQKYYCRVRGGRCAVLSCLPKEEQIGKCSTRGRKCCRRKK
>ID34757-NO
PAEDLARYYSALRHYINLITRQRY
>ID34802-NO
PCKTPGRKCFPHQKDCCGRACIITICP
>ID34970-ABP_both
PKTLRKFFARIRGGRAAVLNALGKEEQIGRASNSGRKCARKKK
>ID35046-NO
PPGCCNNPACVKHRCG
>ID35146-ABP_both
PRPPRLPRPRPRPLPFPRP
>ID35185-NO
PSKDAFIGLM
>ID35247-NO
PVRGPFPIIV
>ID35438-NO
QDSGDGWPQQPFVPRL
>ID35532-NO
QGPWMEEEEAAYGWMDF
>ID35547-ABP_both
QGVRNSQSCRRNKGICVPIRCPGSMRQIGTCLGAQVKCCRRK
>ID35554-NO
QGYKGPYTRPILRPYVRPVVSYNVCTLSCRGITTTQARSCCTRLGRCCHV
>ID35608-NO
QIISRDLISH
>ID35689-NO
QKLCERPSGTWSGVCGNSNACKNQCINLEKARHGSCNYVFPAHKCICYFP

>ID35712-NO
QKLCQRPSGTWSGVCGNNNACRNQCINLEKARHGSCNYVFPAHKCICYFP
>ID35741-ABP_both
QKYYCRVRGGRCAVLSCLPKEEQIGKCSTRGRKCCRRKK
>ID35758-NO
QLGPQGPQHFIADLSKKQRPRMEEEEEAYGWMDF
>ID35761-NO
QLHVNKARRPYIL
>ID35781-NO
QLNFTPNWGTGKRDAGDYGDPYSFLYRLIQAEARKMSGCSN
>ID35799-NO
QLTFSPDWGKR
>ID35855-NO
QNSAAAFGQWA
>ID35863-NO
QNSPAAFGQWA
>ID35866-NO
QNSPAIFGQWA
>ID35870-NO
QNSPNIFGQWM
>ID35990-NO
QRPRMEEEEEAYGWMDF
>ID36033-NO
QSDDYGHMRFG
>ID36034-ABP_neg
QSEAGWLKKIGKKIERVGQHTRDATIQGLGVAQQAPNVAATAR
>ID36140-NO
QVRFRQCYFNPISCF
>ID36142-NO
QVRYRQCYFNPISCF
>ID36159-NO
QVYKGGYTRPVPRPPPFVRPLPGGPIGPYNGCPVSCRGISFSQARSCCSR
>ID36220-ABP_both
RAGLQFPVGRVHRLL
>ID36237-NO
RAPAEDLARYYSALRHYINLITRQRY
>ID36246-NO
RASPYGVKLCGREFIRAVIFTCGGSRW
>ID36307-ABP_both
RCMCIKTTSGIHPKN
>ID36342-NO
RDPCCSNPACNVNNPQ
>ID36370-NO
RECKTESNTFPGICITKPPCRKACISEKFTDGHCSKILRRCFCTRPC
>ID36371-NO
RECKTESNTFPGICITKPPCRKACLTEGFTDGHCSKILRRCLCTKPC
>ID36452-ABP_both
RFRRLRKKWRKRLKKI
>ID36527-ABP_both
RGLRRLGRKIAHGVKKGGPTVLRIIRIAG
>ID36533-ABP_both

RGLRRLGRKIAHGVKKYGPTVLRIIRTAT
>ID36543-ABP_both
RGRGKQGGKVRAKAKTRSS
>ID36678-ABP_both
RIGSILGALAKGLPTLKSWIKNR
>ID36680-NO
RIGSILGALASKLPTLISWIKNRG
>ID36682-ABP_both
RIGSILGRLAKGLPTLKSWIKNR
>ID36683-ABP_both
RIGSILGRLAKGLPTLRSWIKNR
>ID36691-NO
RIIDLLWRVRRPWWPKFVTVWVR
>ID36692-NO
RIIDLLWRVWRPQKPKFVTVWVR
>ID36693-NO
RIIDLLWRVWRPWKPKFVTVWVR
>ID36705-ABP_both
RIKKIVQKKLAGDES
>ID36714-NO
RILQQLLFIHFRIGC
>ID36717-NO
RILQQLLFIHFRIGRRRRRRRR
>ID36898-ABP_both
RKGLQKLVGRVHRLLRK
>ID36900-ABP_both
RKILILIKRK
>ID36958-ABP_both
RKRKILILIKRK
>ID37248-ABP_pos
RLKLLLLLRW
>ID37299-ABP_both
RLNRPVYIPQPRPPHPRL
>ID37361-NO
RLWMRWYSPATRRYG
>ID37362-NO
RLWMRWYSPTARRYG
>ID37363-NO
RLWMRWYSPTTARYG
>ID37364-NO
RLWMRWYSPTTRAYG
>ID37365-NO
RLWMRWYSPTTRRAG
>ID37366-NO
RLWMRWYSPTTRRYA
>ID37367-NO
RLWMRWYSPTTRRYG
>ID37388-NO
RMKQIEDKIEEIESKQKKIENEIARIKKLLQLTVWDIKQLQARIL
>ID37422-NO
RNCRIVVIRVCR

>ID37428-NO
RNGLPVCGETCVGGTCNTPGCTCSWPVCT
>ID37465-ABP_neg
RPDKPRPYLPRPRPPRPV
>ID37467-NO
RPECCTHPACHVSNPELC
>ID37475-NO
RPGPPGLQGRLQRLLQASGNHAAGILTM
>ID37494-ABP_both
RPKHPIKHQGLPQEVLNENLLRFFVAPFPEVFGKEKV
>ID37509-NO
RPPGFSPFRIA
>ID37510-NO
RPPGFTPFRIAPAS
>ID37511-NO
RPPGFTPFRKA
>ID37512-NO
RPPGFTPFRVY
>ID37547-NO
RPVKVYPNVAENESAEAFPLEF
>ID37590-NO
RQIKIWFQARRMKWKK
>ID37592-NO
RQIKIWFQNARMKWKK
>ID37595-NO
RQIKIWFQNRAMKWKK
>ID37597-NO
RQIKIWFQNRRAKWKK
>ID37599-NO
RQIKIWFQNRRMAWKK
>ID37601-NO
RQIKIWFQNRRMKAKK
>ID37602-NO
RQIKIWFQNRRMKW
>ID37603-NO
RQIKIWFQNRRMKWAK
>ID37604-NO
RQIKIWFQNRRMKWK
>ID37605-NO
RQIKIWFQNRRMKWKA
>ID37615-NO
RQLRIAGRRLRGRSR
>ID37895-ABP_neg
RRIRPRPPRLPRPRPC
>ID37897-ABP_both
RRIRPRPPRLPRPRPRPL
>ID37901-ABP_neg
RRIRPRPPRLPRPRPRPLPYPRP
>ID37934-NO
RRLFRRILRRL
>ID37980-NO

RRPPGFTPFR
>ID37985-ABP_both
RRPRPRPRPFFFF
>ID37986-ABP_both
RRPRPRPRPFFFFF
>ID37988-ABP_both
RRPRPRPRPWWWW
>ID38031-ABP_both
RRRPRPPYLRPRPPP
>ID38065-NO
RRRRRRRW
>ID38157-ABP_both
RRWCFRVCYKGRCRYKCR
>ID38483-NO
RSTQGYGRMDRILAALKTSPMEPSAALAVENGTTHPLE
>ID38498-NO
RSVEGVSRMEKLLS
>ID38500-NO
RSVEGVSRMEKLLSSSISPSSTPLGFLSQDHSVN
>ID38506-NO
RSVRAQRHTDMPKTQKEVHLKNASRGSAGNKNYRM
>ID38518-NO
RTCENLADKYRGPCFSGCDTHCTTKENAVSGRCRGFRRRCWCTKRC
>ID38528-NO
RTCESQSNTFPGICITKPPCRKACISEKFTDGHCSKILRRCLCTKPC
>ID38597-ABP_both
RVCSAIPLPWCH
>ID38677-NO
RVTSWLGRQLRIAGKRLEGRSK
>ID38687-ABP_both
RVVRPVVQGVKQKVR
>ID38692-ABP_both
RVVRQWPIGRVVRRVVRRVVRY
>ID38710-ABP_both
RWCFRVCYRGICYRRCR
>ID38721-ABP_both
RWCVYARVRGVRYRRCW
>ID38731-ABP_both
RWCVYAYVRVRGVGVRYRRCW
>ID38732-ABP_both
RWCVYAYVRVRGVLGRYRRCW
>ID38733-ABP_both
RWCVYAYVRVRGVLRRYRRCW
>ID38734-ABP_both
RWCVYAYVRVRGVLSRYRRCW
>ID38735-ABP_both
RWCVYAYVRVRGVLVRYRRCG
>ID38736-ABP_both
RWCVYAYVRVRGVLVRYRRCR
>ID38737-ABP_both
RWCVYAYVRVRGVLVRYRRCS

>ID38738-ABP_both
RWCVYAYVRVRGVRVRYRRCW
>ID38739-ABP_both
RWCVYAYVRVRGVSVRYRRCW
>ID38740-ABP_both
RWCVYGYVRVRGVLVRYRRCW
>ID38741-ABP_both
RWCVYRYVRVRGVLVRYRRCW
>ID38742-ABP_both
RWCVYSYVRVRGVLVRYRRCW
>ID39166-NO
SANSNPALAPRE
>ID39200-ABP_both
SAVGRHLRRFGLRKHRKH
>ID39278-NO
SCNTATCVTHRLAGLLSRSGGVVKNNFVPTNVGSEAF
>ID39427-NO
SDRNFLRF
>ID39459-NO
SEEPPISLDLTFHLLREVLEMARAEQLAQQAHSNRKMMEIF
>ID39460-NO
SEEPPISLDLTFHLLREVLEMARAEQLVQQAHSNRKMMEIF
>ID39494-NO
SESEVPGMWFGPRL
>ID39517-ABP_both
SFGAKNAVKNGLQKLRNQCQANNYQGGFCDIFKKNG
>ID39536-NO
SFLSKIKDIALDVAKNAGKGVLTTLACKIDGSC
>ID39538-NO
SFLTSFKDMALKVAKDAGVNILNTIFCKIFKTC
>ID39610-NO
SGLQFAVLDGQGFLPFPRV
>ID39626-NO
SGPYSFGL
>ID39632-ABP_both
SGRGKQGGKVRAKAKTRSS
>ID39633-ABP_both
SGRGKQGGKVRAKAKTRSSRAGLQFPVGRVHRLLRKGNY
>ID39635-NO
SGRNFLRF
>ID39640-NO
SGSAKVAFSATRSTNH
>ID39650-NO
SGSLSTFFRLFNRSFTQA
>ID39656-NO
SGSLSTFFRLFNRSFTQALK
>ID39657-NO
SGSLSTFFRLFNRSQTQALGK
>ID39658-NO
SGSLSTFFRLQNRSFTQALGK
>ID39659-NO

SGSLSTQFRLFNRSFTQALGK
>ID39703-NO
SICCSFPDPWGGLCCEDHCSYIGKPGGQCSDKSVCTCN
>ID39711-NO
SIFSLFKAGAKFFGKNLLKEAGKAGAAHLACKATNQC
>ID39713-NO
SIGAKVLGGVKTFFKGALKELASTYQQ
>ID39717-ABP_both
SIGSAFKKAAHVGKHVGKAALTHYLN
>ID39727-ABP_both
SIITMTKEAKLPQLGKQIACRLYNTC
>ID39728-ABP_both
SIITMTKEAKLPQLWKQIACRLGNTC
>ID39733-ABP_both
SILGKLWKGVKSIF
>ID39737-NO
SILSGIFGVGKKIVCGLSGLC
>ID39738-NO
SILSTLKDVGISAIKSAGSGVISTLLCKLNKNC
>ID39752-NO
SIRDKIKTIAIDLAKSAGTGVLKTLICKLDKSR
>ID39753-NO
SIRDKIKTIAIDLAKSAGTGVLKTSICKLDKSC
>ID39754-NO
SIRDKIKTTAIDLAKSAGTGVLKTLICKLDKSC
>ID39789-ABP_both
SKHWLWLW
>ID39848-ABP_both
SKWMSLLKHILK
>ID39887-NO
SLGGKPDLRPCYPPCHYIPRPKPR
>ID39910-NO
SLLQFNKMIKFETRKNAIPFYAF
>ID39914-ABP_both
SLLSLIRKLLT
>ID39945-NO
SLRRSSCFGGRMDRIGAQSSLGCNSFRY
>ID39952-NO
SLSTFFRLFNRSFTQALGK
>ID39981-NO
SMFSVLKNLGKVGLGFVACKVSKQC
>ID39995-ABP_both
SMLSVLKNLGKVGLGFVASKINKQS
>ID39996-ABP_both
SMLSVLKNLGKVGLGFVKCKINKQC
>ID39997-ABP_both
SMLSVLKNLGRVGLGFVACKINKQC
>ID39998-ABP_both
SMLSVLKNLKKVGLGFVACKINKQC
>ID40000-ABP_both
SMLSVLRNLGKVGLGFVACKINKQC

>ID40001-NO
SMLVGCWTKSYPPKPCFGRG
>ID40003-NO
SMPGVLRF
>ID40056-NO
SNRSPSLRLRF
>ID40110-NO
SPLEPVYPGDNATPEEMAQYAAELRRYINMLTRPRY
>ID40138-NO
SPRNFLRF
>ID40141-ABP_both
SPRRRTPSPRRRRSQSPRRR
>ID40142-NO
SPRWKIFKKIEKVGRNVRDGIIKAGPAVAVVGQAATVVKG
>ID40226-NO
SRCFPPGIYCTPYLPCCWGICCGTCRNVCHLRI
>ID40261-NO
SRPYSFGL
>ID40267-NO
SRRHHCRSKAARSRHH
>ID40268-NO
SRRHHCRSKAKASRHH
>ID40269-NO
SRRHHCRSKAKRARHH
>ID40270-NO
SRRHHCRSKAKRSAHH
>ID40288-NO
SRWRWKCCKK
>ID40297-NO
SSADTLISDLLIGETESRPQTRYEDHLAW
>ID40320-NO
SSGGGEGSGMWFGPRL
>ID40330-NO
SSIQSLLNLSQRF
>ID40378-ABP_both
SSMKLSFRARAYSFRGPGPQL
>ID40388-NO
SSPETLISDLLMRESTENVPRTRLEDPSMW
>ID40395-NO
SSSGLISMPRV
>ID40412-NO
STCCGYRMCVPC
>ID40423-NO
STFFRLFNRSFTQALGK
>ID40427-ABP_both
STIGKLVKWIIKTVNKFTKK
>ID40479-NO
SVDQDLGPEVPPENVLGALLRV
>ID40518-NO
SVLGSVAKHLLPHVAPIIAEKL
>ID40525-NO

SVNTKNDFMRF
>ID40528-NO
SVPGVLRF
>ID40612-NO
SWLSKTAKKLENSAKKRISEGIAIAQGPR
>ID40671-NO
SYSEHFRWGKPV
>ID40674-NO
SYSMEHFRWGKPM
>ID40682-NO
SYSMEHFRWGKPVGKKRRPVKVYPNGAEGESAEAFPLEF
>ID40683-NO
SYSMEHFRWGKPVGKKRRPVKVYPNGAENESAEAFPVEV
>ID40684-NO
SYSMEHFRWGKPVGRKRRPIKVYPNGVDEESAESYPMEF
>ID40698-NO
TAEDLARYYSALRHYINLITRQRY
>ID40720-NO
TATEECEEYCEDEEKTCCGLEDGEPVCATTCL
>ID40757-NO
TCGECVGGTCNTPGCTCSWPVCTRNGLPV
>ID40797-NO
TCSPAGEVCTSKSPCCTGFLCTHIGGMCHH
>ID40832-NO
TDVDHVFLRF
>ID40856-NO
TFCNLRRCQLSCRSLGLLGKCIGVKCECVKH
>ID40885-NO
TFQYSRGWTN
>ID40906-NO
TGPSATTGVWFGPRL
>ID40925-NO
THSGGACNSHDQCCNAFCSTATRTCV
>ID40948-NO
TIRAPQLRLRF
>ID40952-ABP_both
TISQPEWFKARRWQWRMKKLGA
>ID41009-NO
TKRRITPKKVIDVRSVTTEINT
>ID41034-ABP_both
TLLKKVLKAAA
>ID41089-NO
TNEGPGRDPAPCCQHPIETCC
>ID41092-NO
TNEIVEEQYTPQNLATLESVFQELGKLTGPNSQ
>ID41110-NO
TNVSCTTSKECWSVCQRLHNTSRGKCMNKKCRCYS
>ID41111-NO
TNWNKFQGSW
>ID41122-NO
TPAEDLARYYSALRHYINLITRQRY

>ID41149-NO
TPLGTMRF
>ID41154-NO
TPMQRSSMVRFG
>ID41182-NO
TPSQDFMRF
>ID41249-NO
TRPYSFGL
>ID41264-NO
TRSGGACNSHNQCCDDFCSTATSTCV
>ID41268-ABP_neg
TRSSRAGLQWAVGRVHRLLRK
>ID41298-NO
TSLRGPAESSGESAHPLE
>ID41302-NO
TSNKDEEQRELLKAISNLLD
>ID41375-NO
TTWEAWDRAIAEYAARIEALIRAAQELQEKLEAALRE
>ID41392-NO
TVCNLRMCQLSCRSLGLLGKCIGVKCECVKH
>ID41393-NO
TVCNLRRCQLSCRSLGLLGKCIGDKCECVKH
>ID41444-ABP_both
TVYQHQKAMKPWIQPKTKVIPYVRYL
>ID41466-ABP_neg
TWLKKRRLKKAKPP
>ID41467-ABP_neg
TWLKKRRWAKAKPP
>ID41468-ABP_neg
TWLKKRRWKAAKPP
>ID41469-ABP_neg
TWLKKRRWKKAAPP
>ID41470-ABP_both
TWLKKRRWKKVKPP
>ID41471-ABP_neg
TWLKKRRYKKAKPP
>ID41483-NO
TYKKPPFNGSIF
>ID41485-ABP_neg
TYLKKRRWKKAKPP
>ID41518-NO
VAIYGRDLRSLVCRQVQHNWLVCDTY
>ID41538-NO
VAPFPEVFGKE
>ID41604-NO
VDEECNEYCDDRNKECCGRTNGHPRCANVCF
>ID41621-ABP_both
VDKPPYLPRPRPPRRIYNH
>ID41623-ABP_both
VDKPPYLPRPRPPRRIYNNR
>ID41624-ABP_both

VDKPPYLPRPRPPRRIYNRN
>ID41631-ABP_both
VDKPPYLPRPTPPRRIYNNR
>ID41663-NO
VENLHLPLPLL
>ID41696-NO
VFINVKCRGSPECLPKCKEAIGKAAGKCVN
>ID41717-ABP_neg
VFQFLGKIIHHVGNFVAGFSHVF
>ID41718-ABP_neg
VFQFLGKIIHHVGNFVHGFSAVF
>ID41740-ABP_both
VGALAVVVWKWLWKW
>ID41742-ABP_both
VGALAVVVWLWLWKW
>ID41752-NO
VGCEECPMHCKGKNANPTCDDGVCNCNV
>ID41775-NO
VGPYAFGL
>ID41845-NO
VIGGDECNINEHRFL
>ID41854-NO
VIGSILGVIAKGLPTLISWIKNR
>ID41859-ABP_both
VIHGLLKRIKTLL
>ID41861-ABP_both
VIHRAGLQFPVGRVHRLLRK
>ID41867-NO
VIKNLQNLDPSHRISDRDYMGWMDF
>ID41883-NO
VIPFVASVAAEKMQHVYCAASKKC
>ID41885-NO
VIPFVASVAAEMMQHVYCAASKKR
>ID41886-NO
VIPFVASVAAEMMQPVYCAASKKC
>ID41888-NO
VIPSVASVAAEMMQHVYCAASKKC
>ID41964-ABP_both
VKLKVYPKLKVKLYP
>ID41967-ABP_both
VKLKYPLKVKLYP
>ID41995-ABP_both
VKRFKKFFRKLKKSV
>ID41996-ABP_both
VKRFKKFFRKLKKSVK
>ID41998-ABP_both
VKRFKKFFRKLKKSVKKL
>ID42047-NO
VLGPVLGLVSNALGGLL
>ID42087-ABP_both
VLNENLLA

>ID42102-ABP_neg
VLPIVKKLLRGLF
>ID42106-ABP_both
VLPLVGNLLNDLLGK
>ID42115-ABP_both
VLSAADKGNVKAAWGKVGGHAAEYGAEAL
>ID42117-ABP_both
VLSAADKGNVKAAWGKVGGHAAEYGAEALERMF
>ID42157-NO
VNFSPGWGT
>ID42159-ABP_both
VNGLLHKIYGNIRYS
>ID42175-NO
VNWKKVLAKIIKVVK
>ID42193-NO
VPASGFFGMR
>ID42209-NO
VPGYSHSFV
>ID42224-NO
VPLEPVYPGDNATPEQMAHYAAELRRYINMLTRPRY
>ID42241-NO
VPPGFTPFRQT
>ID42572-NO
VSCEDCPDHCSTQKARAKCDNDKCVCEPK
>ID42582-NO
VSCTGSKDCYAPCRKQTGCPNAKCINKSCKCYAC
>ID42584-NO
VSCTGSKDCYAPCRQQTGCPNAKCINKSCKCYGC
>ID42604-NO
VSIWFCASRTCSTPADCNPCTCESGVCVDWL
>ID42654-ABP_both
VTCDVLSFEAKGIAVNHSACALHAIALRKKGGSCQNGVCVARN
>ID42720-NO
VVGGDECNINEHRSL
>ID42730-NO
VVIGQRCYRSPDCYSACKKLVGKATGKCTNGRCDC
>ID42742-NO
VVKCSYRLGSPDSRSK
>ID42744-NO
VVKCSYRPGSPDSRCK
>ID42791-ABP_both
VWPLVIRTVIAGYNLYRAIKKK
>ID42797-ABP_both
VWRHWRRFWHR
>ID42896-NO
VYRKPPFNGSIF
>ID42921-NO
WCKQSGEMCNVLDQNCCDGYCIVFVCT
>ID42929-ABP_both
WCRRLCYKQRCVTYCRGR
>ID42963-ABP_both

WFHHIFRGIVHVGKTIHRLVTG
>ID43024-ABP_both
WGWRDIVRGIRKAAAPVLST
>ID43025-ABP_both
WGWRDIVRGIRKVAAAVLST
>ID43026-ABP_both
WGWRDIVRGIRKVAAPALST
>ID43027-ABP_both
WGWRDIVRGIRKVAAPVAST
>ID43028-ABP_both
WGWRDIVRGIRKVAAPVLAT
>ID43029-ABP_both
WGWRDIVRGIRKVAAPVLSA
>ID43030-ABP_both
WGWRDIVRGIRKVAAPVLST
>ID43058-NO
WIIFRIAASHKK
>ID43074-ABP_both
WIQPATKVIPYVAYL
>ID43080-ABP_both
WIQPKTKVIAYVRYL
>ID43086-ABP_both
WIQPKTKVIPYARYL
>ID43088-ABP_both
WIQPKTKVIPYVAYL
>ID43089-ABP_both
WIQPKTKVIPYVRAL
>ID43090-ABP_both
WIQPKTKVIPYVRYA
>ID43091-ABP_both
WIQPKTKVIPYVRYL
>ID43104-ABP_both
WIVYKWPRKKVRYWTGP
>ID43128-ABP_both
WKKILSKIKKLLK
>ID43131-ABP_both
WKKIWSKIKKLLK
>ID43169-ABP_both
WKRWVQRWKRWLR
>ID43173-ABP_both
WKTLLKKVLKAAA
>ID43193-ABP_both
WLGALFKVASK
>ID43194-ABP_both
WLGALFKVASKVL
>ID43208-ABP_both
WLNALKKVFQGIHEAIKLINNHVQ
>ID43209-ABP_both
WLNALKKVFQGIHEAIKLIWNHVQ
>ID43216-NO
WLPLIGRVLSGIL

>ID43220-ABP_both
WLRAFRRLVRRLARLLRR
>ID43365-NO
WRFKAAVALLPAVLLALLAP
>ID43454-NO
WSKMDRLAKELTAE
>ID43634-NO
WYKHTASPRYHTVGRAAGLLMGL
>ID43635-NO
WYKHTASPRYHTVGRAAGLLMGLRRSPYMW
>ID44282-NO
YADAIFTNSYRKVLGQLSARKLLQDIMSRQQGERNQEQGARVRL
>ID44283-NO
YADAIFTSSYRKVLGQLSARKLLQDIMSRQQGERNQEQGPRVRL
>ID44290-NO
YAEGTFISDYSIAMDKIRQQDFVNWLLAQKGKKNDWKHNLTQ
>ID44317-NO
YCCHPACGKNFDC
>ID44332-NO
YCKSKGAKCSKLMYDCCSGSCSGTVGRC
>ID44362-NO
YDCEPPGNFCGMIKVGPPCCSGWCFFACA
>ID44416-NO
YGCCSNPVCHLEHSNLC
>ID44417-NO
YGCCSYPPCFATNSDYC
>ID44424-ABP_both
YGFYTHVFRLKKWMQKVIDRFGG
>ID44449-NO
YGGFMTSEKSQTPLVTLFKNAIIKNAYKKGQ
>ID44450-NO
YGGFMTSEKSQTPLVTLFKNAIVKNAHKKGQ
>ID44469-NO
YGRKKRRQRRRD
>ID44478-NO
YGRRRRRRRRR
>ID44520-NO
YIQQARKAPSGRMSVIKNLQNLDPSHRISDRDYMGWMDF
>ID44589-NO
YKIFEPLRESNL
>ID44603-ABP_both
YKLLKLLLPKLKPLLFKL
>ID44619-NO
YKQCHKKGGHCFPKEKICIPPSSDFGKMDCRWRWKCCKKGSGK
>ID44620-NO
YKQCHKKGGHCFPKEKICIPPSSDFGKMDCRWRWKCCKKRSGK
>ID44629-NO
YKRCHKKGGHCFPKTVICLPPSSDFGKMDCRWRWKCCKKGSVNNAISI
>ID44719-NO
YPFPGPIPNSL
>ID44730-NO

YPIKPEAPGEDASPEELNRYYASLRHYLNLVTRQRY
>ID44743-NO
YPPKPENPGEDAPPEELAKYYSALRHYINLITRQRY
>ID44753-NO
YPRAARRAARR
>ID44757-NO
YPSGFLGMR
>ID44761-NO
YPSKPDSPGEDAPAEDMARYYSALRHYINLITRQRY
>ID44769-NO
YPTSYDDDFDALDDLDDLDLDDLLDLEPADLVLLDMWANMLDSQDFEDFE
>ID44772-NO
YPVKPENPGEDAPAEELAKYYSALRHYINLITRQRY
>ID44773-NO
YPVKPENPGEDAPTEELAKYYTALRHYINLITRQRY
>ID44824-NO
YRKPPFNGSIF
>ID44838-NO
YRRRRRRRRRR
>ID45032-NO
YVRGMASKAGAIAGKIAKVALKAL

Section S8. List (FASTA format) of all the peptides in the test dataset.

>ID00016-ABP_both
IWLTALKFLGKHAAKHLAKQQLSKL
>ID00044-ABP_both
SFGLCRLRRGFCARGRCRFPSIPIGRCSRFVQCCRRVW
>ID00071-ABP_both
GIWGTALKWGVKLLPKLVGMAQTKKQ
>ID00091-ABP_both
VDKPDYRPRPRPPNM
>ID00092-ABP_both
VTCDLLSFEAKGFAANHSLCAAHCLAIGRRGGSCERGVCICRR
>ID00220-ABP_both
KTCENLADTY
>ID00258-ABP_both
DDTPSSRCGSGGWGPCLPIVDLLCIVHVTVGCSGGFGCCRIG
>ID00265-ABP_both
FGLPMLSILPKALCILLKRKC
>ID00266-ABP_both
FISAIASFLGKFL
>ID00276-ABP_both
FLPIIAKVLSGLL
>ID00277-ABP_both
FLPILASLAATLGPKLLCLITKKC
>ID00283-ABP_both
FMGGLIKAATKIVPAAYCAITKKC
>ID00356-ABP_both
IPRPLDPCIAQNGRCFTGICRYPYFWIGTCRNGKSCCRRR
>ID00370-ABP_neg
MRTGNAN
>ID00390-ABP_both
VIDDLKKVAKKVRRELLCKKHHKKLN
>ID00399-ABP_both
AGCIKNGGRCNASAGPPYCCSSYCFQIAGQSYGVCKNR
>ID00405-ABP_both
ALYLAIRKR
>ID00449-ABP_both
GFLGPLLKLGLKGAAKLLPQLLPSRQQ
>ID00450-ABP_neg
GFLSALKKYLPIVLKHV
>ID00499-ABP_both
HPHVCTSYYCSKFCGTAGCTRYGCRNLHRGKLCFCLHCSR
>ID00585-NO
ATCKAECPTWDSVCINKKPCVACCKKAKFSDGHCSKILRRCLCTKEC
>ID00602-ABP_both
FFGHLFKLATKIIPSLFQ
>ID00609-ABP_both
FIGAIARLLSKIF
>ID00611-ABP_both
FIGGIISFIKKLF
>ID00633-ABP_both

FLPVIAGLLSKLF
>ID00753-ABP_both
GVSKILHSAGKFGKAFLGEIMKS
>ID00758-ABP_both
GWKKWFTKGERLSQRHFA
>ID00800-NO
KLCERSSGTWSGVCGNNNACKNQCIRLEGAQHGSCNYVFPAHKCICYFPC
>ID00843-ABP_both
RLKELITTGGQKIGEKIRRIGQRIKDFFKNLQPREEKS
>ID00860-ABP_pos
SKGKKANKDVELARG
>ID00861-ABP_both
SLGGVISGAKKVAKVAIPIGKAVLPVVAKLVG
>ID00884-ABP_both
VWPLGLVICKALKIC
>ID00890-ABP_both
YENPYGCPTDEGKCFDRCNDSEFEGGYCGGSYRATCVCYRT
>ID00901-ABP_both
AERVGAGAPVYL
>ID00903-NO
AGETHTVMINHAGRGAPKLVVGGKKLS
>ID00913-ABP_neg
ATCDALSFSSKWLTVNHSACAIHCLTKGYKGGRCVNTICNCRN
>ID00936-ABP_both
ESVFSKIGNAVGPAAYWILKGLGNMSDVNQADRINRKKH
>ID00937-NO
EWEPVQNGGSSYYMVPRIWA
>ID00952-ABP_both
FFPLVLGALGSILPKIF
>ID00977-ABP_both
FLPLVLGALSGILPKIL
>ID01046-ABP_both
GLFTLIKGAAKLIGKTVPKKQARLGMNLWLVKLPTNVKT
>ID01072-NO
GLPVCGETCFTGTCYTNGCTCDPWPVCTRN
>ID01168-ABP_both
RFRLPFRRPPIRIHPPPFYPPFRRFL
>ID01211-ABP_both
VTCDILSVEAKGVKLNDAACAAHCLFRGRSGGYCNGKRVCVCR
>ID01230-ABP_both
AIPCGESCVWIPCISTVIGCSCSNKVCYR
>ID01232-ABP_both
ALWKTIIKGAGKMIGSLAKNLLGSQAQPES
>ID01313-ABP_pos
FMGSALRIAAKVLPAALCQIFKKC
>ID01337-ABP_both
GFMDTAKNVAKNVAVTLIDKLRCKVTGGC
>ID01408-NO
GLPVCGETCFGGTCNTPGCSCETWPVCSRN
>ID01457-ABP_both
IFGAIWKGISSLL

>ID01481-NO
KIMAKPSKFYEQLRGR
>ID01501-ABP_pos
KYYGNGVSCNSHGCSVNWGQAWTCGVNHLANGGHGVC
>ID01509-ABP_pos
LLLFLLKKRKKRKY
>ID01535-ABP_both
PFKLSLHL
>ID01620-NO
VISIIPV
>ID01689-ABP_both
DWTAWSALVAAACSVELL
>ID01761-ABP_both
GGTIFDCGESCFLGTCYTKGCSCGEWKLCYGTN
>ID01763-ABP_both
GICRCICTRGFCRCICVL
>ID01844-ABP_both
ILGIITSLLKSL
>ID01858-ABP_both
KCWNLRGSCREKCIKNEKLYIFCTSGKLCCLKPK
>ID01882-ABP_both
KRGLWESLKRKATKLGDDIRNTLRNFKIKFPVPRQG
>ID02018-ABP_both
SPPSEQLGKSFNF
>ID02084-ABP_both
DAECEICKFVIQQVEAFIESNHSQAEIQKELNKL
>ID02156-ABP_both
FVGLAKVAAHVVPAIAEHF
>ID02168-ABP_both
GFGSLFKFLAKKVAKTVAKQAAKQGAKYVANKHMQ
>ID02192-ABP_both
GIINTLQKYYSRVRGGR
>ID02259-ABP_both
IGPDTKKCVQRKNACHYFECPWLYYSVGTCYKGKGKCCQKRY
>ID02261-ABP_both
IIGGR
>ID02289-ABP_pos
KRKCPKTPFDNTPGAWFAHLILGC
>ID02331-ABP_pos
MGAIAKLVTKFGWPLIKKFYKQIMQFIGQGWTIDQIEKWLKRH
>ID02373-ABP_both
QLGELIQQGGQKIVEKIQKIGQRIRDFFSNLRPRQEA
>ID02395-ABP_both
RRWQWR
>ID02438-ABP_both
YPELQQDLIARLL
>ID02445-ABP_both
AGLQFPVGRIGRLLRK
>ID02520-ABP_both
FIGPVLKMATSILPTAICKGFKKC
>ID02608-ABP_both

GLGSLLGKALKFGLKAAGKFMGGEPQQ
>ID02631-NO
GNEGGGHGGHGGYGGYHHHGGGGGGYGGYHGGGGS
>ID02708-NO
KSCCRSTLGRNCYNLCRVRGAQKLCANACRCKLTSGLKCPSSFPK
>ID02861-NO
RVCESQSHGFKGACTGDHNCALVCRNEGFSGGNCRGFRRRCFCTLKC
>ID02928-ABP_both
YRGGYTGPIPRPPPIGRPPLRLVVCACYRLS
>ID03081-ABP_both
FMGTALKIAANVLPAAFCKIFKKC
>ID03143-ABP_both
GILDTLKQLGKAAAQSLLSKAACKLAKTC
>ID03148-ABP_both
GILSTIKDFAIKAGKGAAKGLLEMASCKLSGQC
>ID03230-ABP_both
GSKKPVPIIYANRRTGKAQRM
>ID03266-ABP_both
GYGDGCYSEDDLSVCCKKKFKVIGKCFKSVRECQNSGCKYH
>ID03305-NO
ISVCITVC
>ID03313-ABP_both
IWSFLIKAATKLLPSLFGGGKKDS
>ID03315-ABP_both
KFFKRLLKSVRRAVKKFRKKPRLIGLSTLL
>ID03323-ABP_both
KIIFLIAI
>ID03333-ABP_neg
KKWRKSFFKQVGSFDNSV
>ID03352-NO
KSCCPNTTGRNIYNACRLTGAPCPTCAKLSGCKIISGSTCPSDYPK
>ID03382-ABP_neg
KWKSFIKKAKTSFLHSAKKF
>ID03388-ABP_neg
KWKSFIKKLTSKFLHLAKKF
>ID03405-ABP_both
KYALMKKIAELIPNLKSRQVK
>ID03409-ABP_both
LCAALCLAIGRR
>ID03410-ABP_both
LCAARCLAIGRR
>ID03495-NO
MLWSASMRIFASAFSTRGLGTRMLMYCSLPSRCWRK
>ID03581-ABP_neg
RAIGGGLSSVGGGSSTIKY
>ID03585-NO
RDCTSQSHKFVGLCLSDRNCASVCLTEYFTGGKCDHRRCVCTKGC
>ID03591-NO
RGCYKICGETCLFIPCLTSVFGCSCKN
>ID03614-ABP_neg
RRRFPWVCWPFLRRR

>ID03645-ABP_both
SIMSTLKQFGISAIKGAAQNVLGVLSCKIAKTC
>ID03675-ABP_pos
TTPLCVGVIIGITASIKICK
>ID03676-ABP_pos
TTPLCVGVIIGLTTSIKICK
>ID03689-ABP_both
VIPIVSGLLSSLL
>ID03727-ABP_both
AAKKLSKLLKTLLKLL
>ID03738-ABP_both
AGDDETLLKPVLNSLDNLVSGL
>ID03739-NO
AHKCIC
>ID03836-NO
CYFQNCPRG
>ID03862-ABP_both
EAALKAALDLAAKLA
>ID03898-NO
FEMQYCWSHSGVCRDKSERNNKPMAWTYCENRQKKCEF
>ID03911-ABP_both
FIKRIARLLRKIF
>ID03943-NO
FPAHKCICY
>ID03960-ABP_both
FSTKTRNWFSEHFKKVKEKLKDTFA
>ID04023-ABP_both
GIINTLQKYYSRVRGGRSAVLSSLPKEEQIGKSSTRGRKSSRRKK
>ID04072-ABP_both
GLLRRLRKKIGEIFKKYG
>ID04082-ABP_both
GLRRALLRLLRSLRRLLLRA
>ID04087-ABP_both
GMASKAGTIAGKIAKTAIKLAL
>ID04103-NO
GSCGASIAEFNSSQILAKRAPPCRRPRLQNSEDVTHTTLP
>ID04266-ABP_both
KKFFLKVLTKIRCKVAGGCRT
>ID04357-ABP_both
KWKWKWKW
>ID04439-ABP_both
LSSALSALSSALSSK
>ID04440-NO
LTCDLLSFEAKGFAA
>ID04661-NO
RICKSRSQKFKGPCVSEDNCANVCHTEGFPDGDCDGLLRRCYCNTHC
>ID04678-ABP_both
RKKRWWRRKK
>ID04679-ABP_both
RKLRRLKRKIAHKVKKY
>ID04691-NO

RMCKTPSAKFKGYCVSSTNCKNVCRTEGFPTGSCDFHITSRKCYCYKPCP
>ID04710-ABP_both
RRRRRRAAFAAWAAFAA
>ID04719-ABP_both
RVCMAIPLPLCH
>ID04753-ABP_both
SGRGKTGGKARAKAKTRSSRAGLQFPVGRVHRLLR
>ID04838-ABP_neg
VRRVVRRVVRVVRRVVRRVRRVVRRVVRVVRRVVRR
>ID04883-ABP_both
YKFRWRIYI
>ID04887-ABP_both
YSLKKTSMKIIPFTRL
>ID04905-NO
ACKFWW
>ID04924-NO
ADSGEGDFLAEGGGVRKLIK
>ID04930-NO
AEAMSQV
>ID04938-NO
AEPERRNIKYL
>ID04991-ABP_both
AKKVFKRLGIGAVLKVLKKG
>ID04998-NO
AKSRWY
>ID05028-NO
APKEWMAWAREIAAYAKLIAALIKQGI
>ID05033-NO
APQYNQR
>ID05052-NO
ASCDKCQLKGEAMHG
>ID05063-ABP_pos
ATCDLASGFGVGSSLCAAHCLVKGYRGGYCKNKICHCRDKF
>ID05069-NO
ATHQETIEKVTEALKINNLRLVTLEHQVLVIGLKVE
>ID05124-ABP_neg
CMSYGGSCQRSCNGGFRLGGHCGHPKIRCCRRK
>ID05129-NO
CSSHFPYSQYQFWK
>ID05149-NO
DDASDRAKKFYGLM
>ID05159-ABP_pos
DGVCSNRRQCNKEVCGSSYDVAIVGA
>ID05167-ABP_both
DLLGTLGNLPLPFI
>ID05196-NO
DTLREVQLRVQRDRQCLRIF
>ID05216-ABP_both
ELSKAVRLIK
>ID05221-ABP_pos
ENDHRMPYELNRPNNLSKGGAKCGAAIA

>ID05229-NO
ESELVSQIIEQLIKK
>ID05293-ABP_both
FFRNLWKGAKAAFRAGHAAWRA
>ID05296-ABP_pos
FFRRFFRRFFRRFFRR
>ID05299-ABP_both
FFVLKFLLKWAGKVGLEHLACKFKNWC
>ID05313-NO
FIHNFKRK
>ID05375-ABP_neg
FLKLLAGLLKNFA
>ID05377-ABP_both
FLKWLFKWLKK
>ID05389-ABP_both
FLPLIARVLSGIL
>ID05390-ABP_both
FLPLIGAVLSGIL
>ID05406-ABP_both
FLTGLIGGLMKALGK
>ID05411-NO
FPPWL
>ID05434-NO
FVPIWM
>ID05446-ABP_neg
FWRIRIRR
>ID05450-NO
GACRAIRRIPRRIRGLERIL
>ID05464-ABP_both
GECIWDAIFHGAKHFLHRLVNP
>ID05552-NO
GICRCICGRRICKCICGR
>ID05574-ABP_both
GIGKFIHAVKKWGKTFIGEIAKS
>ID05579-ABP_both
GIGKFLHSAKKFGKAFVGEIMNSKKKKKKKKK
>ID05600-NO
GIIDIAKKLFESW
>ID05605-NO
GILDALTGIL
>ID05648-NO
GKWKLFKKAAKKFLKKCS
>ID05696-ABP_both
GLKKLISWIKRAAQQG
>ID05773-ABP_both
GMWSKILKHLIR
>ID05794-ABP_both
GRLKRLRKKLKKLLKKLS
>ID05801-NO
GRWPVKT
>ID05883-NO

HFPRIWLHSLGQHIY
>ID05892-NO
HLKTAVQMAVFIHNFKR
>ID05907-NO
HPQFNQR
>ID05925-NO
HRILMRIR
>ID05967-NO
IKKEKVYLAWVPAHKGIGN
>ID05972-NO
IKWPWYVWL
>ID05986-ABP_both
ILGPVISTIGNALGGLLKNL
>ID05990-NO
ILKKFPFWPWRRK
>ID06014-NO
ILKKWPWWPWWPWRRK
>ID06016-NO
ILKKWPWWVWRRK
>ID06038-NO
ILPWICPWRPSKAN
>ID06087-NO
ILRWPWWPWWPWRRK
>ID06106-NO
IRALQRAVRHPRAIRRIYRGWKKAIR
>ID06131-NO
IVWQVDRM
>ID06209-ABP_both
KISKKIMRTFLRRILTGKK
>ID06235-ABP_both
KKKKKKKKKKGIGKLFLHAAKKFAKAFVAEKMNS
>ID06240-ABP_both
KKKLKKLKKKLK
>ID06351-NO
KPGQTCSVAGWGQTAPLGKS
>ID06361-NO
KQLTEAVQKITTESIVIWGK
>ID06405-NO
KTKKTKKTKKTKKTKKTKKTK
>ID06412-ABP_neg
KVLKAAAKAALNAVLVGANA
>ID06414-ABP_both
KVMAHMK
>ID06422-ABP_neg
KWKEFLKTFKEAKKEVLHTALKAISS
>ID06430-ABP_both
KWKKFKKIGKVLKVL
>ID06525-NO
LCQRPS
>ID06534-ABP_both
LDPKVVQSLL

>ID06543-NO
LFHLSVDNEHRGQGIAKALV
>ID06584-NO
LKKLLKKLLKKLLKKLLKKLLKKLLKKLLKKL
>ID06590-ABP_both
LKLKLFPLKLKLFP
>ID06598-NO
LKLKLKLKLKLKLKLKLKLKLKLKLKLKLKLKLKLK
>ID06610-NO
LLEYSL
>ID06637-NO
LMRIR
>ID06660-NO
LQQLLF
>ID06845-NO
MGKNGSLCCFSLLLLLLLAGLASGHQVL
>ID06846-NO
MGRIARGSKMSSLIVSLLVVLVSLNLASETTA
>ID06858-NO
MIVLPRKYGKASRKCSRCGDHSALVRRYGLMLCRQCFRELAPKIGFKKYN
>ID06934-ABP_both
MKTLVLLSPSSCWPSRSRLILSKTQMKRLKLRSSQRKRTR
>ID07150-NO
NRILPTLIGPL
>ID07162-NO
NVTLLCDCPNGPWVWVPAFC
>ID07230-NO
QETAYFLLKLAGRWP
>ID07273-NO
QLLIRMI
>ID07300-NO
QQWAVGHFM
>ID07322-NO
RCICGLGFC
>ID07324-NO
RCICGLGVC
>ID07330-NO
RCICGRGVC
>ID07368-NO
RCLCGLRIC
>ID07384-NO
RCLCTLRVC
>ID07391-NO
RCLCVLGVC
>ID07393-NO
RCLCVLRVC
>ID07397-NO
RCLCVRRVC
>ID07404-NO
RFARRFARRFARRFARRFARRFAR
>ID07411-ABP_both

RFRRLFRIRVRVLKKI
>ID07512-NO
RLVVWWVVRR
>ID07516-NO
RLWWVVWWRR
>ID07523-NO
RMKQIEDKIEEIESKQKKIENEIARIKKLIGERY
>ID07525-NO
RNSLPKVAYATA
>ID07536-NO
RQLKSSRRGALVCVRLKLCSAILSRGLSCGMFSCNARR
>ID07599-NO
RRKKVLLALLAP
>ID07609-ABP_both
RRRCPIVVIRVCRR
>ID07622-NO
RRWCFIVCRRGRCYVACRR
>ID07640-NO
RSQKEGLHYTCSSHFPYSQYQFWK
>ID07641-NO
RSQMQDGQLQSCCQELQNVEEQCQC
>ID07677-ABP_both
RWWRWRKWW
>ID07700-ABP_both
SIGSALKKALPVAKKIGKIALPIAKAALPVAAGLVG
>ID07725-ABP_both
SMSGFSKPHD
>ID07727-NO
SNTALRRYNQWATGHFM
>ID07732-NO
SRAAGLAARLARLAL
>ID07753-NO
SWSSFFKKAAHSGKHVGKSASTHYL
>ID07823-NO
VDIHVWAGV
>ID07826-NO
VDIHVWSGV
>ID07859-NO
VKRGLKLKLAKLAKKLAKLAK
>ID07912-ABP_pos
VSGPAGPPGTH
>ID07916-ABP_both
VSWKKSLGKIIKVVK
>ID07936-NO
VYIHPF
>ID07956-NO
WGRVSMRRGT
>ID08004-ABP_both
WYSEMKRNVQRLERAIEE
>ID08042-NO
YGGFL

>ID08044-NO
YGGFM
>ID08070-NO
YIRDFITRRPPFGNI
>ID08102-NO
YQVIQSWEHWRE
>ID08111-NO
YSTGMVHLLLQVTIDGRNYI
>ID08177-NO
AAPGWPEDGAGKMGAEEAKPPEGKGEWAHSRQEEEEMARAPQVLFRG
>ID08179-NO
AAQRRGRIGRNPSQVGD
>ID08284-NO
AGEGLNSQFWSLAAPQRF
>ID08308-NO
AIPALGTNVKKRRHG
>ID08320-NO
AKDEMEECASHLPYEA
>ID08351-NO
AKRHHGYKRKFHGGG
>ID08396-NO
ALYVGDLCGSVFLVGQLF
>ID08402-NO
ANFYVCPPPTGATVV
>ID08443-NO
APVPGLSPFRVV
>ID08453-NO
AQLVGELGSLYGPLSVSA
>ID08458-NO
ARLDTSSQFRKKWNKWALSR
>ID08477-NO
ASGCKADACKSYCKSLGSGGGYCDQGTWCVCN
>ID08500-ABP_both
ATAWNLGPHGLRPIRPIRIRPLCGKDKS
>ID08507-NO
ATCDLLSFEIKGFKLNDSACAAHCIQLGKRGGHCNNSKVCVCRR
>ID08558-NO
AVQRWRWVV
>ID08563-ABP_both
AVWKFVKRV
>ID08570-NO
AWLVHRQWFLDLPLPWLPG
>ID08597-NO
CDLLLPGRC
>ID08599-NO
CDQVAGYSC
>ID08609-NO
CEEIRARLSTHLRKMRKRLMRDADDLQKRLAVY
>ID08616-NO
CERPYSRLC
>ID08620-ABP_both

CFALKKAMKKVR
>ID08650-NO
CGEKSAQLC
>ID08659-NO
CGETCTLGTCYTQGCTCSWPICKRNGLPV
>ID08674-NO
CGPSLRGVC
>ID08688-NO
CHGKAALAC
>ID08694-NO
CHHNLTAAC
>ID08736-NO
CKPSTSGQC
>ID08743-NO
CKSLGSSQC
>ID08750-NO
CLALNMSYC
>ID08756-NO
CLKNWFQPC
>ID08757-NO
CLLGRMKC
>ID08765-NO
CLPTDPIQC
>ID08778-NO
CMSTRGDQARKICE
>ID08779-NO
CMTSHPTLC
>ID08800-NO
CNTKNFHSC
>ID08805-NO
CPGHHLSHC
>ID08832-NO
CQDRKTSQC
>ID08837-NO
CQPHLPWHC
>ID08840-NO
CQRCGWETGVGVSGFLVRILRFVVL
>ID08851-NO
CQYNPLPYC
>ID08854-NO
CRDTTPWWC
>ID08870-NO
CSAGAPEFC
>ID08900-NO
CSQFPPRLC
>ID08915-NO
CTDVYRPTC
>ID08949-NO
CTTMTRMTC
>ID08963-NO
CVSLEHQNC

>ID08965-NO
CWPPSSRTC
>ID09055-NO
DGSYKMSHFRWSSPPAS
>ID09082-NO
DKYCSENPLDCNEHCLKTKNQIGICHGANGNEKCSCMES
>ID09093-NO
DLKYNPSRVEAFHRY
>ID09101-ABP_both
DLWQFGKMILKVAGKLPFPYYGAYGCYCGWGGRGKPKDPTDRCCFVHDCC
>ID09107-NO
DNSDNMF
>ID09112-NO
DPKGDPKGVTVTVTVTVTGKGDPKPD
>ID09124-NO
DRVYIHP
>ID09136-ABP_both
DTIPCGESCVWIPCISSILGCSCKDKVCYHN
>ID09150-NO
DVKFPGGGQIVGGVYL
>ID09161-NO
DVTFSLLGANTKSYAAFITNFRKDVASEKK
>ID09173-NO
EALAKAAEALAKAAEALAKAAEALAKAAWASLWNWF
>ID09224-NO
EGPTLGNWAREIWATLFKKA
>ID09249-NO
ELLEELKNEAVRHFP
>ID09257-NO
ELQREESPTGPPGSIRTWFQRIPLGWFHCTYQKGKQHCR
>ID09258-NO
ELQREESPTGPPGSIRTWFQRIPLGWFHCTYQKGKQHCRLRIRQKVEE
>ID09271-NO
ENREILKEPVHGVYY
>ID09324-NO
EYPLSRVDLGDCIGK
>ID09339-NO
FAKGVGKVGKKAL
>ID09352-NO
FAKKLAKKLKKLAKKLAKLALALKALALKAL
>ID09363-NO
FAKKLKKLAKKL
>ID09368-NO
FAKLAKKLL
>ID09396-NO
FAKLLFKALKKAL
>ID09441-NO
FEPIPIHYCAFPGF
>ID09458-NO
FFVIFYRRKK
>ID09466-NO

FHGHGLY
>ID09470-NO
FHNHGIL
>ID09482-NO
FIGSALKVLAGVLPSVISWVKQ
>ID09508-ABP_both
FKCRRWQWRMKKLGKRSKNKGFKEQAKSLLKWILD
>ID09522-NO
FKNHTSPDVDLGDISGINASVVNIQKEIDRLNEVAKNLNES
>ID09534-ABP_both
FLEGLLNTVTGLL
>ID09554-ABP_both
FLPIVAKLLSGLLGRKKRRQRRR
>ID09586-NO
FNRWWWKKI
>ID09671-ABP_both
GAPKGCWTKSYPPQPCS
>ID09685-NO
GCCSDPRCNMNNPDYC
>ID09697-NO
GCGGLMAGCDGKSTFCCSGYNCSPTWKWCVYARP
>ID09699-ABP_both
GCKKFRRFKLKCKQKLWLWCG
>ID09723-NO
GEEEYQKMLENLREAEVKKNA
>ID09729-NO
GELGRLVYLLDGPGYDPIHCSLAYGDASTLVVF
>ID09774-NO
GGAGLTGGFYEPLVRRC
>ID09802-NO
GIGAVLKVLTTGLPALISWI
>ID09807-NO
GIGDPVTCLKSGAIA
>ID09931-NO
GLLSVFKGVLKGVGKNVAGSLLDQLKCKISGGC
>ID09978-NO
GPMGWVPVFYRF
>ID09989-NO
GQIINLK
>ID10005-NO
GRKRRQTSMTDFYHS
>ID10019-NO
GRWPVKTIHTDNGSNFTGAT
>ID10025-NO
GSDTSLIDTCGNTPNVSGAN
>ID10031-ABP_both
GSKKPVPIIYCNRRTGKC
>ID10073-NO
GTKAWTEVWPLC
>ID10077-NO
GTKWLTEWIPLC

>ID10081-NO
GTPGVAAATQAANGG
>ID10090-ABP_both
GVCRCVCRRGVCRCVCRR
>ID10093-ABP_both
GVIPCGESCVFIPCINKKKCSCKNKVCYRD
>ID10104-NO
GWAGWLLSPRGSRPS
>ID10108-ABP_both
GWGCNIFGGNDYRCHRHCKSISGYKGGYCKLGGICKCY
>ID10126-NO
GWWNQMAYYNSCKWEEAKVK
>ID10134-NO
GYHLMSFPQAAPHGVVFLHVTW
>ID10139-NO
GYVYFEEYAYSHQLS
>ID10157-NO
HATCSLAFALATSVALATRNDLLLRWAAARDAQTILSKRDR
>ID10164-NO
HCKFWH
>ID10165-NO
HCKFWK
>ID10228-NO
HRKRRR
>ID10250-NO
HWGMWSY
>ID10289-NO
IGLKVEAMEKFLYTAFAMQE
>ID10296-NO
IHVQGHLQEVDAGNFIPP
>ID10353-NO
ILMCFSID
>ID10372-NO
ILPWKHPWWPWRR
>ID10407-NO
INLKALAALAKALL
>ID10443-NO
IRQRVRWRW
>ID10479-NO
IWKVRIFKR
>ID10482-ABP_both
IWRVWRRWK
>ID10496-NO
KAAKKAWKAWKKAAKAAWKKAA
>ID10501-NO
KAFEEVLAKKFYDKALWD
>ID10516-NO
KCCRCK
>ID10519-NO
KCLPPGKPCYGATQKIPCCGVCSHNKCT
>ID10560-NO

KFTIVFPHNQKGNWKNVPSNYHYCP
>ID10592-NO
KIFGSLAFL
>ID10606-ABP_both
KKAAALAAAAALAAWAALAAAKKKK
>ID10615-ABP_both
KKCPIVVIRVCK
>ID10631-NO
KKKKGVAATYVLV
>ID10640-NO
KKKKLLAATYVLV
>ID10687-NO
KKKKVLAATLVLV
>ID10765-NO
KKKKVVALTYVLV
>ID10768-NO
KKKKVVAPTYFLV
>ID10777-NO
KKKKVVLAFYVLV
>ID10805-NO
KKLMSNNVQIVRQQSYS
>ID10830-ABP_both
KLALKAALKAWKAAAKLA
>ID10867-NO
KNEWGCRCNDSSDGNEWGCRCNDSSD
>ID10871-NO
KNKKQTDILEKVKEILDKKKKTKSVGQKLY
>ID10872-ABP_both
KNNFCQVLYVWLLRLGKQCFVKFSKDVET
>ID10922-ABP_neg
KRIRWVILWRQV
>ID10932-NO
KRKRRR
>ID10975-NO
KTELQAIYLALQDSGLEVNI
>ID11042-ABP_pos
KWRRWI
>ID11048-NO
KWWWYWYRR
>ID11058-NO
KYKKGWRVV
>ID11086-NO
LCYTHHHFTVCV
>ID11139-NO
LGCETAALRLGIKLTQHYFGLLTAFGSNFGTIG
>ID11145-NO
LGLTAGVAYAAQPTNQPTNQPTNQPTNQPTNQPTNQPRW
>ID11168-NO
LIILVPPFF
>ID11173-NO
LKAKTNISIREGPTLGNWAR

>ID11184-NO
LKLKLK
>ID11211-NO
LLEYVM
>ID11215-ABP_both
LLGAALSALSSVIPSVISWFQK
>ID11224-NO
LLGDLLDDVTSIRHAVLQNRAAIDF
>ID11248-NO
LNLADATNFLQDSKAELEKARKILSEVGRWY
>ID11258-NO
LPFEF
>ID11276-ABP_both
LPRRNRWSKIWKKVVTVFS
>ID11295-ABP_both
LRFLKKALKKLF
>ID11299-NO
LRGEREFRRIGLVVSKKTLKHAVKRNRARRRVRELLRAILLAQALQRGA
>ID11313-NO
LRLLTPEHYQRLGLAVPKKQIKTAVGRNRFKRICRELDFVVLFNLL
>ID11329-ABP_both
LRWQNEMRKV
>ID11352-NO
LTVRAAQSFGRCNQKQCDADCVKKGYFGGLCTLTSCFCTGSRS
>ID11354-NO
LVCHPAVPALLCAR
>ID11371-NO
LVVLLLFAGVDAETHVTG
>ID11426-NO
MANSGLQLLGFSMALLGW
>ID11456-NO
MDVNPTL
>ID11830-NO
MLQDLLSSLGDLLKSWLDTLNKFTKK
>ID11988-NO
MWKTPTLKYFGGFNFSQI
>ID11996-NO
NAHCALL
>ID12015-NO
NGESSADWAKN
>ID12028-ABP_both
NIGLFTSTCFSSQCFSSKCFTDTCFSSNCFTGRHQCGYTHGSC
>ID12035-ABP_pos
NKGCSACAIGAACLADGPIPDFEVAGITGTFGIAS
>ID12047-NO
NLYIQWLKDGGPSSGRPPPS
>ID12075-NO
NQTKDLQQKFYEIIMDIEQN
>ID12103-NO
NWCKRGRKQCKTHPHFVIPY
>ID12122-NO

PAIYIGATVGPSVWAYLVALVGAAAVTAAN
>ID12147-NO
PDYLLVPEEVMEYKPRRKRAAI
>ID12158-ABP_neg
PFWRRRIRIRR
>ID12171-NO
PKDLKETLQEKKP
>ID12216-NO
PPLAVMRPGIQILPPPGTDVHPK
>ID12223-NO
PPNPTPPPPGASANA
>ID12231-NO
PPRLLDSPEVMVILHLGFRIGLVRLWIH
>ID12241-NO
PPWCCCSPMKRASPPPAQSDLPATPKCPP
>ID12256-NO
PRPGP
>ID12276-NO
PTSNHSPTSCPPTCPGYRWMCLRRF
>ID12284-NO
PVPFEEVIDKINAKG
>ID12287-NO
PWLKPGDLDL
>ID12291-NO
PYKFKATMYYKDVTV
>ID12301-NO
QDCNCSIYPGHITGHRMA
>ID12321-NO
QGHLQEVDAGNFIPPPRW
>ID12333-NO
QHLLQLTVWGIKQLQARILAVERYLKDQ
>ID12344-ABP_both
QKAIRVRLSA
>ID12349-ABP_both
QKKIAVRLSA
>ID12351-NO
QKKIRFRLSA
>ID12356-NO
QKKIRVRISA
>ID12360-NO
QKKIRVRWSA
>ID12379-NO
QNPDIVIYQYMDDLY
>ID12399-NO
QRCCNGRRGCSSRWCRDHSRCC
>ID12403-NO
QRLCCGFPKSCRSRQCKPHRCC
>ID12419-NO
QSPPAIPALGTNVKK
>ID12420-NO
QSRVIQGLVAGETAQQICED

>ID12423-NO
QTLLQELPIPP
>ID12439-NO
QYGSFCTQLNRALSGIAAEQ
>ID12446-NO
RARRSLLIASALCTSDVAAATNADLRTALARADHQKTLFWL
>ID12495-NO
RGRRGIYR
>ID12514-NO
RIPTGERVWDRGNVTLLCDC
>ID12522-ABP_both
RIRRWKFRW
>ID12527-ABP_neg
RIVRVRAIRWQV
>ID12542-ABP_both
RKCNFLCKVKNKLKSVGSKSLIGSATHHGIYRV
>ID12572-NO
RKRVIKRWR
>ID12576-NO
RKTSERSQPRGRRQPIPK
>ID12593-NO
RLILIKKKW
>ID12618-NO
RMRGAHTNDVKQLTEAVQKI
>ID12629-NO
RPEGQNYTEGIAVVF
>ID12639-NO
RPPGFDPFR
>ID12656-ABP_both
RRCPIVVIRVCR
>ID12657-NO
RRCPTRPEGQNYTEG
>ID12675-ABP_neg
RRIRFRPPYLPRPGRRPRFPPPFPIPRIPRIP
>ID12676-NO
RRIRGRVGR
>ID12701-NO
RRKKLALLAP
>ID12702-NO
RRKKWLVFFVIFYFFR
>ID12718-NO
RRRLGWRRG
>ID12726-NO
RRRRHWCW
>ID12778-NO
RRWWCRA
>ID12784-NO
RRWWCRH
>ID12787-NO
RRWWCRM
>ID12825-NO

RTQRRGRTGRGKPGIYR
>ID12849-NO
RWLRIRKVY
>ID12860-NO
RWRRYGRVY
>ID12882-ABP_neg
RWWRWRR
>ID12887-NO
RYGRKWMIW
>ID12898-NO
SALVGCWTKS
>ID12923-NO
SDEDSDGDRPQASPGLGPGP
>ID12924-NO
SDFRRQNRRGGTNKRTT
>ID12953-NO
SGFDPLITHA
>ID12954-NO
SGGSNRSP
>ID12971-NO
SIELNKAKSDLEESKEWIRRSNQKLDSI
>ID12985-NO
SKHSSLDCVLRP
>ID13012-NO
SMEKLAGFGAVGAGATAEETRRMLHRAFDTLA
>ID13034-NO
SPYPSWSTPAGR
>ID13039-NO
SQGSTLRVQQRPQNSKVTHISSCFGHKIDRIGSVSRLGCNALKLL
>ID13056-NO
SSCFGGRIDRIGAQSGLGCNS
>ID13065-NO
SSMTKILEPFRKQNP
>ID13085-ABP_both
STLHLVLRLAGG
>ID13137-NO
SWWWIWLKK
>ID13188-NO
TEEAELELAENREILKEPVH
>ID13193-NO
TFLVHREWFMDLNLPWSSA
>ID13194-NO
TFLVHREWFMDLNLPWSSAGSTVWR
>ID13252-NO
TMLEDHEFVPLEVYT
>ID13308-NO
TTESIVIWGKTPKFKLPIQK
>ID13326-NO
TVSYEL
>ID13329-ABP_both
TVVRRRGRSPRRRTPSPRRAASQSPRRAASQSRESQC

>ID13371-NO
VCCGYPLCHPC
>ID13376-NO
VCRSTAKYVRNNLET
>ID13378-NO
VCSTGRRQRSICKQLLKKLRQQ
>ID13390-NO
VEDTLYGDHECGSLLQDAALYLVDGMTNTI
>ID13395-NO
VESMNEELKKIIAQVRAQAEHLKTAY
>ID13404-ABP_neg
VFLRRIRVIVIR
>ID13408-NO
VGCAECPMHCKGKMAKPTCENEVCKCNIGKKD
>ID13422-NO
VGQKWRKRT
>ID13461-NO
VLYNSTFFSTFKCYGVSATK
>ID13467-ABP_both
VNWKKILGKI
>ID13523-NO
VRRFKWWWKFLRR
>ID13640-NO
WICEVLSDFK
>ID13657-NO
WKMWLRKHW
>ID13667-NO
WLEEEEEAYGWMDF
>ID13692-NO
WMEWDREI
>ID13721-ABP_both
WRKFWKYLK
>ID13730-NO
WRYWWTRRI
>ID13736-NO
WSTIVKLTICPTLKSMAKKCEGSIATMIKKKCDK
>ID13740-NO
WVPAFCQAVGWGDPITHWSH
>ID13846-NO
YADHTGLVRDNMAKL
>ID13880-NO
YEDQGPLVEGQLGEN
>ID13884-NO
YENQKQIANQFNKAISQIQESLTTTSTA
>ID13892-NO
YGGFLRRI
>ID13901-NO
YGGFMKSWDERSQKPLLTLFKNVINKEHQKKDQ
>ID13918-ABP_both
YKARRWAWRWK
>ID13929-NO

YKQCHKKGGHCFPKEVLICIPPSSDFGKMDCRWKRKCCKKRS
>ID13962-NO
YPGHITGHRMANMMMNW
>ID13982-ABP_both
YRLRVKWKW
>ID14006-NO
YWRYLWIRF
>ID14054-NO
AADHDVGSELPPEGVLGALLRV
>ID14062-NO
AAEWLDAFFVRHVDR
>ID14069-ABP_neg
AAGKWKLFKKLPKFHLHLAKKF
>ID14076-ABP_both
AAGRYQLLSRYWDAYR
>ID14086-ABP_both
AAHCLLRGNR
>ID14156-NO
AANDKASAWTTRTIKQSCCVTPSCTRLYKKVY
>ID14159-NO
AANDKASDLMALRDGCCSNPSCSVNNPDICGGGR
>ID14177-NO
AANNKATDLMALTVRGCCAHLPCALMYTACSWGR
>ID14178-NO
AANNKATDLMALTVRGCCDDPPCRARYPFLCI
>ID14193-NO
AAPSAPLIRF
>ID14212-NO
AASDKASELMALAVRGCCSNPTCAGNNGNICG
>ID14270-ABP_both
ACFLTRLGTYVC
>ID14346-NO
ADIKRCVVDVKLSKPCTFQECIPLCFQRYNGNGVCTGKKNEICTCAYNC
>ID14381-NO
AEDYERQIMAF
>ID14396-NO
AEIKVRDGYIVYPNNCVYHCGLDPYCNDLCTGA
>ID14398-NO
AEKVDPVKLNLTLSAAAEALTGLGDK
>ID14437-NO
AFDRIEGAGFGLS
>ID14466-NO
AFLTLTPGSHVDSYVEA
>ID14469-NO
AFPGS
>ID14479-ABP_both
AFTHRRSYSTEYRYGTTVRGIRHRFL
>ID14487-NO
AFVRIL
>ID14506-ABP_pos
AGCVCSGTVAVANSHNAGPAYCVGYCGNNGEVTRNANYNIARRS

>ID14532-NO
AGGTGANSAMWFGPRL
>ID14548-NO
AGLSALYSFG
>ID14628-NO
AHLPIVRASLPS
>ID14645-ABP_pos
AIFIFIRWLLK
>ID14651-NO
AIGNILKTLGNLAQKILGK
>ID14682-NO
AILTTLANWARKFLGK
>ID14734-NO
AKGNLMRF
>ID14843-NO
ALAGTIIAGASLTFKILDEV
>ID14856-NO
ALDGLDGAGFGFD
>ID14915-NO
ALILTLVS
>ID14933-NO
ALLHHGYKRKFH
>ID14941-ABP_both
ALLSPYKYSTTKVVK
>ID14943-NO
ALMGFQGVR
>ID14950-NO
ALNSLDGNGFGFD
>ID14965-NO
ALQHFSSLDTLGGMGFG
>ID14978-ABP_both
ALSSSGGQE
>ID15033-NO
AMAHLPLRLGKNREDSLSRWVPNLPQRF
>ID15060-ABP_both
AMRLTYNKPGLYGT
>ID15078-ABP_both
ANAICAKIHAC
>ID15097-NO
ANPAVVRP
>ID15119-NO
APACVGFQGMR
>ID15141-NO
APEKWAAFHGSW
>ID15149-NO
APERNFLRF
>ID15165-NO
APGLFELPSRSVRLI
>ID15166-NO
APGPR
>ID15175-NO

APKLSDGAAAGYVTKA
>ID15205-NO
APNGFLGMR
>ID15235-ABP_both
APRKNVRWCTISQPEWFKCR
>ID15269-NO
APSLGFHGVR
>ID15285-NO
APTGFTGMR
>ID15289-NO
APTSSFIGMR
>ID15294-NO
APVNSFVGMR
>ID15295-NO
APVPGGQGTVLDKMYPRGNHWAVGHLM
>ID15352-NO
AQREPIRF
>ID15357-NO
AQSNFVTWGYNVAV
>ID15359-NO
AQTFVRF
>ID15369-NO
AQYTNHFVHSLDTLPLRF
>ID15527-NO
ASAYRYHFGL
>ID15540-NO
ASFDDEYY
>ID15550-NO
ASGPGPSHKIKDRDYLGWMDF
>ID15583-NO
ASNQDFMRF
>ID15585-NO
ASNWNTRIAYIGCCDIPDCYNKNREQCLDESS
>ID15599-NO
ASQYTFGL
>ID15610-NO
ASSQHLCGSHLVDALYMVCGEKGFFYQPKT
>ID15658-NO
ATGAASLYSFGL
>ID15763-NO
AVHYSGGQPLGS
>ID15783-NO
AVLLPKKTEKK
>ID15826-NO
AVQLLEVNPEIAQNS
>ID15851-NO
AVVRP
>ID15874-NO
AWNKINGGW
>ID15876-ABP_pos
AWNTYDYMKREHSLVKPYQG

>ID15885-NO
AWQDLQAGW
>ID15889-NO
AWRDLSGGW
>ID15896-NO
AWSDLSQQGW
>ID15907-NO
AWYRGAAPPKQEFLDIEDP
>ID16000-NO
CADGGDLCDPSSDNCCSECIDEGGSGVCAIVADVV
>ID16095-NO
CCDWPCGIGCIPCCLP
>ID16098-NO
CCDWPCTIGCVPCC
>ID16239-NO
CCPRRLACCII
>ID16244-NO
CCQAGCSRYMCLPCCQ
>ID16289-NO
CCSQNCLTCIPCCPY
>ID16300-NO
CCSRYCYICIPCCPN
>ID16326-NO
CDSDGTSCTSNMECCGYGCCSGTCQTPCRFGP
>ID16343-NO
CEMCNGRCMG
>ID16463-NO
CGKRGDSIC
>ID16490-NO
CGSLVRC
>ID16495-NO
CGVCNGRCGL
>ID16552-NO
CKAAGKSCSRIAYNCCTGSCRSGKC
>ID16575-NO
CKGKGAKAARSGKC
>ID16577-NO
CKGKGAKCCRSGKC
>ID16622-ABP_both
CKINKQC
>ID16633-ABP_pos
CKKRKC
>ID16685-NO
CKYKAR
>ID16690-NO
CKYLLKWR
>ID16753-ABP_both
CLLLLKKKKKC
>ID16777-NO
CLSPGSSCSRLMYNCCRSCNPYSRKCR
>ID16808-NO

CNGRCGGKLAKLAKKLAKLAK
>ID16814-NO
CNNVGSYC
>ID16827-NO
CPEHRSLVC
>ID16892-NO
CRGDRCPDC
>ID16922-NO
CRLGIAC
>ID16952-NO
CRTCNGRCLE
>ID17050-NO
CTGSWLGC
>ID17085-NO
CTPSPFSHC
>ID17097-NO
CTSPGGACYSASTCCSKACNLTTKRCVLS
>ID17098-NO
CTSPKQCLPPCKAQFGQSAGAKCMNGKCKC
>ID17100-NO
CTTGPCCRQCKLKPAGTTCWRTSVSSHYCTGRSCECPSYPG
>ID17102-NO
CTTMNCLKGHCGCSPDCGSC
>ID17103-NO
CTTSKECWSVCQRLHNTSRGKCMNKKCRCYS
>ID17143-NO
CVLVTL
>ID17150-NO
CVPPSRYCTRHRPCCRGTCCSGLCRPMCNLWY
>ID17153-NO
CVPYEGPCNWLTQNCCDATCVVFWCL
>ID17155-NO
CVQCNGRCAL
>ID17184-NO
CWSGVDC
>ID17199-ABP_both
CWWWKKKKC
>ID17218-NO
CYDGGTSCNTGNQCCSGWCIFLCL
>ID17256-NO
DAARPNYYFL
>ID17319-NO
DCAGYMRECKEKLCCSGYVCSSRWKWCVLPAP
>ID17329-NO
DCCHNTQLPFIYKTCPEGCNL
>ID17330-NO
DCCLRHFLCCV
>ID17344-NO
DCCYGRVNGCNPKMADKNYE
>ID17364-NO
DCLGFLWKCNPSNDKCCRPNLVCSRKDKWCKYQI

>ID17373-NO
DCLPGWSVYEGRCYKVFNQKTWKAAEKFC
>ID17377-NO
DCPSDWSNHEGHCYRVFNEWMNWAD
>ID17384-NO
DCQALWDYCPVPLLSSGDCCYGLICGPFVCIGW
>ID17392-NO
DCQPCGHNVCC
>ID17419-ABP_both
DDALHHLLHHLLHHL
>ID17571-NO
DEEHDTSEGNWLGSGPDPLDYADEEADSSYAEN
>ID17590-NO
DEKPKLILPTPAPPNLPQLVGGGGGNRKDGFGVSVDAHQKVWTSDNGGHS
>ID17599-NO
DEPPAFFLKIAKNIPRI
>ID17641-NO
DFDRDFMHF
>ID17643-NO
DFDTLSCMLGRVYQSCWQV
>ID17644-NO
DFFKVNIFGLSVRKREMLMKMKERK
>ID17654-NO
DFNKFHTFPQTAIGVGAP
>ID17710-NO
DGSYKMHHFRWSRPPKD
>ID17776-NO
DHLPHDVYSPRL
>ID17831-NO
DKIHPF
>ID17921-NO
DLLAVSWLRA
>ID17925-NO
DLMQFETLIMKIAGRSGVWIYGSYGCYCG
>ID17937-NO
DLRGVPNPWGWIFGR
>ID17939-NO
DLRNIFLKIKFKKK
>ID17957-ABP_both
DLYPWFN
>ID17968-NO
DMSVF
>ID17996-NO
DPAFSSWG
>ID18022-NO
DPSFLRF
>ID18038-NO
DQESCKGRCTEGFNVDKKCQCDELCSYYQSCCTDYTAECKPQVT
>ID18052-NO
DQVKIVL
>ID18101-NO

DRNYLRF
>ID18104-NO
DRRRRGSRPSGAERRRR
>ID18113-NO
DRVYHPF
>ID18123-NO
DRWRPALPVVLFPLH
>ID18130-NO
DSACYVSA
>ID18131-NO
DSADVKELIKKWEKQVSQKKKQKNGKK
>ID18147-NO
DSKLMDPLIRF
>ID18175-ABP_both
DTGPIRRPKPRPRPRPE
>ID18225-NO
DVGTTE
>ID18240-NO
DVPSERY
>ID18256-NO
DVSHQHY
>ID18319-NO
DYCCRRPPCTLIC
>ID18357-NO
EACYNAGSFCGIHPGLCCSEFCILWCITFVDS
>ID18394-NO
EAPPGFSPFR
>ID18450-ABP_both
ECKFTVKPYLCP
>ID18454-NO
ECKWYLGTCSKDGDCCKHLQCHSNYE
>ID18502-NO
ECRWYLGGCSQDGDCCKHLQCHSNYEWCVWDGT
>ID18512-NO
ECTDSGGACNSHDQCCNEFCSTATRTCI
>ID18593-NO
EEAGG
>ID18596-NO
EECRLTIDKATPCHLSDCRLSCYTGYNGVGECFDDPNVPGPDNCGCRYNC
>ID18612-ABP_neg
EEEEAAAGRLWVLWRR
>ID18650-NO
EEYTKKIEEYTKKIEEYTKKIEEYTKKIEEYTKKI
>ID18685-NO
EGFYSQRY
>ID18687-NO
EGGSSGEASGMWFGPRL
>ID18743-NO
EIDRSSFGFH
>ID18755-NO
EILNIIDSISDVAKQICCQITVDCCVLDEE

>ID18984-NO
ENCCPR
>ID19005-NO
ENNNGYIRF
>ID19042-NO
EPQCIGSCEMLADCNTACIRMGYLFGQCVGWKTPDMCCCNH
>ID19075-NO
EQLIPFPRI
>ID19091-NO
ERAEECRTTQLRYHYHRNGAQSRSLCAAVLCC
>ID19106-NO
ERGDT
>ID19116-NO
ERNMT
>ID19155-NO
ESKDQLSEDASKVITYL
>ID19164-NO
ESRLPKILLDFLFLRKK
>ID19189-NO
ETHFGANTRFGW
>ID19226-NO
EVNFTPNW
>ID19283-NO
FAAGRR
>ID19295-ABP_both
FAGAAFKALSKLL
>ID19301-ABP_pos
FAKLAARLYRKA
>ID19324-ABP_both
FAVTWATKWWKG
>ID19326-NO
FCCDSNWCNISDCECCY
>ID19338-NO
FCHDSISCMVGGDNVCNNVCVRQGNPNGGRCLPRDGCPGNDICACYPQS
>ID19341-ABP_both
FCPRRYKQIGTGLPGTKCK
>ID19344-NO
FCSDPPCRISNPESCGWEP
>ID19353-NO
FDAFTTGF
>ID19385-NO
FDGRNAPADDKASDLIAQIVRRACCSDRRCRWRCG
>ID19435-ABP_pos
FFFFF
>ID19438-ABP_pos
FFFFFFFF
>ID19494-ABP_both
FFLLPIPNDVKCKVLGICKS
>ID19496-NO
FFLPPCAHKGTC
>ID19501-NO

FFPFVINELAKLPSLISLLKK
>ID19519-ABP_neg
FFPIVKKFLKGLF
>ID19601-ABP_both
FHFHLHFGGGFIKHFIHRF
>ID19636-ABP_both
FIHHIVHAIGRLF
>ID19655-NO
FINTIRLLINKYREWKNKKDS
>ID19686-NO
FITKALGISYGRKKRRQRRRPPQ
>ID19703-ABP_both
FKARLWQWRM
>ID19750-ABP_both
FKKLKKLFKKILKLK
>ID19753-ABP_both
FKKLYIEPVETKVDEINDNRRKANRKRFSAHQG
>ID19798-NO
FLAKKVGKQLASHLAKKQLE
>ID19831-ABP_both
FLGALFKKASK
>ID19848-ABP_both
FLGALVKALSKLL
>ID19866-NO
FLGGILNTLTGLL
>ID19872-NO
FLGGLLSSVLGPLGKK
>ID19875-ABP_both
FLGGVFKGASKVFPAVFGKV
>ID19895-ABP_both
FLGKVVKGASKVFPAVFGKV
>ID19959-ABP_neg
FLKLLKALL
>ID19977-NO
FLPALAGIAGLLGKIFGK
>ID19986-NO
FLPAVLRVAAKIVPSVVCAITKKC
>ID20042-ABP_both
FLPKTLRKFF
>ID20052-NO
FLPLIAGLAVNFLPKLFCKITKKC
>ID20159-NO
FMPILVCSRFKRC
>ID20170-NO
FNGRSAAADQNAPGLIAQVVRGGCCSDPRCAWRCG
>ID20175-NO
FNVPLYE
>ID20187-NO
FPEVFGK
>ID20192-NO
FPFFNQYVAL

>ID20214-NO
FPPQSVL
>ID20271-NO
FRCLERVCT
>ID20322-NO
FRVPTPN
>ID20360-NO
FSQAQG
>ID20386-NO
FTMKKSLLLLFFLGTINLSLCEQERNAEEERRDDLGERQAEVEKR
>ID20400-ABP_pos
FTVATFI
>ID20406-NO
FVAPFP
>ID20629-NO
GAFGDFLKGAAKKAGLKILSIAQCKLSGTC
>ID20640-NO
GAKFIRF
>ID20646-ABP_both
GAKLAKKQVRALGKFFSF
>ID20659-ABP_both
GALFSLASKVVPAVIGLIKK
>ID20671-NO
GANMGLYAFPRV
>ID20730-NO
GCCAIRECRLQNAAYCGGIY
>ID20752-NO
GCCEPDWCDSGCDDGCC
>ID20919-NO
GCCSNLACAGNNLHIC
>ID20926-NO
GCCSNPPCIAKNPHMCGGRR
>ID20959-NO
GCCSRPPCILNNPDLC
>ID20993-NO
GCETR
>ID21103-NO
GDCPPWCVGARCRAGKC
>ID21128-NO
GDLGKTTTVSNWSPPKYKDTP
>ID21166-NO
GECDGKKDCITNDDCTGCLCSDFGSYRKCA
>ID21170-NO
GEDEYAEGIREYQLIHGKI
>ID21266-NO
GFCCPSGWSSYDR
>ID21762-NO
GFDHYGFTGGI
>ID21844-NO
GFKDGAADRISHGF
>ID21848-NO

GFKNVEMMTARGF
>ID21856-NO
GFKTVGLATARGF
>ID21875-NO
GFLGMR
>ID21901-NO
GFMNTVSNVLTNVAGTVKDKIKCKFTGGC
>ID21903-NO
GFNSALMF
>ID21928-NO
GFVRI
>ID21983-NO
GGEEQFDDYGHMRF
>ID22020-NO
GGGRAFAGSWSPYLERFYDY
>ID22022-NO
GGGRAFNHNANLFRYE
>ID22042-ABP_both
GGKCKNGDCVCR
>ID22081-NO
GGPYSYGL
>ID22183-NO
GICKDDWCQ
>ID22199-NO
GIFDSIKEGFKNAAVTLLNKIKCKNF
>ID22217-NO
GIFSILKIATKLIGKTLAKAAGKAGAELAACKAANQC
>ID22246-ABP_both
GIGDILKNLAKAAGKAALHAVGESL
>ID22254-NO
GIGGALLSAGKSALKGLAKVLADKFAN
>ID22348-ABP_both
GIIPIISIIGKII
>ID22510-NO
GIVDQCCNNICTFNQLQNYCNVP
>ID22512-NO
GIVDQCCTGVCSLYQLQNYCN
>ID22523-NO
GIVEQCCHKPCSIFELQNYCN
>ID22539-ABP_both
GIVLKDLFSEKLRRYKIVIG
>ID22563-NO
GKASQFFGLM
>ID22577-NO
GKEGYPTDKRGCKLTCFFT
>ID22646-ABP_both
GKLWLKGGKLWLKGGKLWLKGGKLWLKG
>ID22668-ABP_both
GKPRPYSPRPTPHPRL
>ID22692-NO
GKQDFIRF

>ID22708-NO
GKTKGVTSGLIAFPRL
>ID22742-NO
GKYVSLTTPKNPTKRRITPKDV
>ID22747-ABP_both
GLAKRIKTLL
>ID22769-NO
GLDWWSL
>ID22772-ABP_neg
GLEETVYIYGANMAS
>ID22779-ABP_both
GLFAVIKKVASV
>ID22789-ABP_both
GLFDIVKKVV
>ID22799-NO
GLFDSIKEGFQNAAVKLLDKLKCKLSACPPA
>ID22825-ABP_both
GLFKGKKKNKKTK
>ID22912-NO
GLGSVFGRLARILGRVIPKVAKKLGPKVAKVLPKVMKEAIPMAVEMAKSQ
>ID22951-NO
GLLDILRGAGKNLIATGLNTLRCKLTKC
>ID22961-NO
GLLDTLKNMAINAAKGAGLKLLNALSCKLSGTC
>ID22989-ABP_both
GLLKKIKWLL
>ID23004-ABP_both
GLLKRIKLLL
>ID23085-NO
GLLSGPRCGEESTMWT
>ID23129-NO
GLMPSFAF
>ID23153-NO
GLNFSPSW
>ID23175-NO
GLPKKGLGSGCFGLKLDRIGSMSGLGC
>ID23275-NO
GLPVKWS
>ID23332-NO
GLVDFAKHVIGIASKL
>ID23346-NO
GLWEDILYSLNIIKHNNTKGLHHPIQL
>ID23347-NO
GLWEDLLYNINRYAHYIT
>ID23364-NO
GLWRALWRLLRSLWRLLWSQPKKKRKV
>ID23383-ABP_both
GLYNFIKVLGRTVFGLYKQF
>ID23408-NO
GMDSLAFAGGL
>ID23418-ABP_neg

GMLKRIKTLM
>ID23423-ABP_neg
GMMKRIKTMM
>ID23432-NO
GMRLTYNRPCLYATKKTKEM
>ID23503-NO
GNSNFVRF
>ID23517-NO
GPAGAP
>ID23518-NO
GPAGAPGAA
>ID23578-NO
GPMEDPLEIIRI
>ID23587-NO
GPPGPP
>ID23593-NO
GPPSLRLRF
>ID23616-NO
GPSGGFNGALAR
>ID23642-NO
GPWEPCSVTCSKGTRTRRR
>ID23688-NO
GRCCDVPNACSGRWCRDHAQCC
>ID23720-NO
GRCRGFRRRC
>ID23754-NO
GRGGSSNYVRL
>ID23773-NO
GRKKRRQRRRPPQGRKKRRQRRRPPQGRKKRRQRRRPPQ
>ID23837-NO
GRRNH
>ID23849-NO
GRRRRKRLSHRT
>ID23857-NO
GRRTRSSRLRNS
>ID23900-NO
GSFVPRL
>ID23911-ABP_both
GSGSGSGSLKKIFKKPMVIGVTIPF
>ID23950-NO
GSLSNMMRI
>ID23959-NO
GSPGLIPFGRS
>ID23982-NO
GSSGGMIPFPRV
>ID23985-NO
GSSGLIAMPRV
>ID24005-NO
GSTGLISFGRT
>ID24009-NO
GSVAFPAENGVQNAESTQE

>ID24031-NO
GSWYAWSPLVPSAQI
>ID24039-NO
GTACSCGNSKGIYWFYRPSCPTDRGYTGSCRYFLGTCCTPAD
>ID24052-NO
GTGSCGYGKLHTGYWCSYFP
>ID24100-NO
GVAACSSLF
>ID24136-NO
GVHHA
>ID24171-NO
GVLSGIMSNLGTVGNMVGGFCCTVYSGCCSE
>ID24272-NO
GWCGDPGATCGKLRLYCCSGFCDCYTKTCKDKSSA
>ID24322-NO
GWMSEKKVQGILDKKLPEGIIRNAAKAIVHKMAKNQFGCFANVDVKGDCK
>ID24329-NO
GWQDLQSGW
>ID24404-NO
GYCYHRYYCCSRACKLTTKRCL
>ID24466-NO
HADGTFTNDMTSYLDAKAARDFVSWLARSDKS
>ID24490-NO
HAEGTYTSDISSFLRDQAAQNFVAWLKSGQPKQE
>ID24503-NO
HCCPIDLPCCPP
>ID24527-NO
HEVPSGPNPISN
>ID24561-ABP_both
HGKKVLDSFSNGMKHLDDLKGTFAALSELH
>ID24568-NO
HGQNRWPLSCPQYVYGSV
>ID24602-NO
HIDGIFTDSYSRY
>ID24678-ABP_both
HLHLLLLLHLH
>ID24716-NO
HPGSPFPPEHRP
>ID24727-NO
HPLRLPA
>ID24770-ABP_both
HRVKRNGFRKFMRRLKKFFAGG
>ID24810-NO
HSLQDTEEKSRSFPASQTDPLEDPDQINED
>ID24818-NO
HSQGTFTSDYSKFLDTRRAQDFLDWLKNTKRNRNEIA
>ID24842-NO
HTHQDFQPVLHLVALNTPLSGGMRGIR
>ID24847-ABP_pos
HVEQLLR
>ID24860-ABP_both

HWFHWK
>ID24956-NO
IAYKPE
>ID24969-ABP_both
ICHHCI
>ID24979-NO
IDALNENK
>ID24980-NO
IDCCEICCNPACFGCLNDANGLINGDRPIRAQHVC
>ID24987-NO
IDHFGFVGGL
>ID25004-NO
IDTCRLPSDRGRCKASFERWYFNGRTCAKFIYG
>ID25095-NO
IGDEPLANYL
>ID25134-ABP_both
IHKFWRRWFKHI
>ID25183-NO
IILLQGR
>ID25285-ABP_both
IKKRWWWFR
>ID25305-NO
IKNTAASNKASSLVALVVRGCCYNPVCKKYYCWKG
>ID25412-ABP_both
ILGTIPGLLKGL
>ID25418-ABP_both
ILILIKRK
>ID25419-ABP_both
ILILILILKRK
>ID25432-ABP_both
ILKWKWKWWKWRR
>ID25631-ABP_both
ILSLRWRWWKWKK
>ID25650-NO
IMRIKQGQIGQMTI
>ID25660-NO
INEFLERSGIPRQRNQ
>ID25661-NO
INEQTVVTK
>ID25703-NO
INYWL
>ID25739-NO
IPMYDFGI
>ID25788-NO
IRAQQ
>ID25841-ABP_both
IRKLKSWKWLRWL
>ID25903-NO
ISEMTWEECCTNPVCRQHYMHYC
>ID25918-NO
ISINQDLKAIADMLIVEQKQEREKYLADLRQRLLNK

>ID26017-NO
ITTNP
>ID26047-NO
IVGRRRHQG
>ID26050-ABP_both
IVLNPWDQVK
>ID26054-ABP_both
IVNHLVKLFDKGLNSIVNLR
>ID26064-NO
IVRRGCCSDPRCAWRCG
>ID26098-NO
IWLTALKFLGKNAAKHFAKRQLSKL
>ID26115-NO
IWSGYGVYW
>ID26152-NO
IYPRY
>ID26185-NO
KAKFKAD
>ID26195-ABP_both
KAKRRARNLNQKKRK
>ID26229-ABP_both
KARRAVRAI
>ID26232-ABP_pos
KARRWIRWL
>ID26303-NO
KCVLVTL
>ID26308-NO
KCYGKWAMHACWGGN
>ID26330-NO
KDGYLVGTDGCKYGCFTRPGHFCANEECL
>ID26419-ABP_neg
KFFKKLKNSVKKRAKKFFKKPKVIGVTFPF
>ID26444-ABP_both
KFKKLFKKLSPVIGKEFKRIVERIKRFLR
>ID26455-NO
KFLSGGFKEIVCHRYCAKGIAKEFCNCPD
>ID26495-ABP_both
KGIRGYKGGYKGAFKQTKY
>ID26526-NO
KGRGKRRREKQRPSDKP
>ID26535-NO
KGVSLSYRKKGVSLSYR
>ID26575-NO
KIAAKSIAKIWKSILKIA
>ID26657-NO
KILKKLFKFVF
>ID26687-NO
KITRCPMIPC
>ID26708-ABP_both
KKAAARAAAAARAAWAARAAAKKKK
>ID26721-ABP_both

KKAKKAKKA
>ID26726-ABP_both
KKALLAHFFHLLALLALHFFHALKKA
>ID26734-NO
KKCADIDQDCKTSCDCCE
>ID26745-NO
KKDGKKRKRSRKESYSVYVYKVLKQ
>ID26791-ABP_both
KKKFFFFFKKK
>ID26829-ABP_both
KKKKWKLFKKIGIGKFL
>ID26854-ABP_both
KKKVVVVVKKK
>ID26865-ABP_both
KKLFKKGLKFL
>ID26983-ABP_both
KKRYKKKYKAYKPYKKKKKF
>ID26990-ABP_both
KKTSMKIIPFTRL
>ID26992-ABP_both
KKTWWKTWWTKW
>ID27036-ABP_both
KKWQWKMKKLG
>ID27111-NO
KLGLKLGLKGLKGGLKLG
>ID27129-ABP_both
KLILKRKRKRILIK
>ID27285-NO
KMDCRPRPKCCKK
>ID27286-NO
KMDCRWRPKCCKK
>ID27402-NO
KPNAYKGKLPIGLW
>ID27425-NO
KPRPGQFFGLM
>ID27429-NO
KPRPQQFIGLM
>ID27449-NO
KQCTSNMCSADCSPGCCIIDKLEWCTCDC
>ID27464-NO
KQQLATEAESAGPI
>ID27500-ABP_both
KRFIKWYKAWNKKRRVY
>ID27541-ABP_both
KRIKTLL
>ID27556-ABP_both
KRIVKLLLKWLR
>ID27574-ABP_both
KRKIKRK
>ID27577-ABP_both
KRKILIKRK

>ID27666-NO
KRQKYDI
>ID27684-ABP_both
KRSQEYRIGRCPNTYACLKR
>ID27752-ABP_both
KSPFVLVVSSRVAAVIKSLP
>ID27753-NO
KSPPQALNKPLPAPSAPSL
>ID27772-NO
KTCQRRWDFCPGALVGVITCCGGLICLGVMCI
>ID27836-NO
KVIDVKCTSPKQCLPPCKAQFGD
>ID27882-NO
KVREGTTY
>ID27954-ABP_both
KWFKWS
>ID27986-ABP_both
KWKLFKKIGAVLKVLTTG
>ID28020-ABP_both
KWKRWWWFR
>ID28058-ABP_neg
KWKVFKKIEKKWKVFKKIEKAGPKWKVFKKIEK
>ID28067-ABP_both
KWKWWKWK
>ID28074-ABP_both
KWLFFK
>ID28205-NO
KYGLCRDETVFPSHSCTFTG
>ID28273-NO
LAHKAL
>ID28279-ABP_both
LAKRRVLTLLRQLRRVSPSS
>ID28297-NO
LAQSQFVGSR
>ID28361-NO
LDGYPLSKIN
>ID28371-NO
LDKRKCFPKNHFCGFVVMLNYLCCSGRCIFVCV
>ID28434-NO
LECVVDSCR
>ID28471-NO
LENLHLP
>ID28483-ABP_both
LEQLLRLKKY
>ID28501-NO
LFECSFSCEIEKEGDKPCKKKKCKGGWKCKFNMCVKV
>ID28574-NO
LGLLLRHLRHHSNLLANI
>ID28580-NO
LGPLGHQ
>ID28619-NO

LHLPGP
>ID28620-NO
LHLPLL
>ID28622-NO
LHLPLPL
>ID28625-NO
LHLPS
>ID28652-ABP_both
LIGSLVRGILPLF
>ID28670-ABP_both
LIKRK
>ID28688-NO
LIPPGVPY
>ID28744-NO
LKDGYPTNSKGCKISGCLPGENKFCLNECQ
>ID28762-ABP_both
LKIAKGIIKSL
>ID28772-ABP_neg
LKKISQRYQKFALPQY
>ID28849-ABP_both
LKLLLLLKCCKKKK
>ID28860-NO
LKPTPQGN
>ID28919-ABP_both
LLCKLKCKLKL
>ID28977-NO
LLIILRRAIRKQAHAHSK
>ID28978-ABP_neg
LLIILRRGHYY
>ID29011-NO
LLKTTELLKTTELLKTTE
>ID29039-ABP_both
LLNQELLLNPTHQIYPV
>ID29073-NO
LLRISLLLIQSWLE
>ID29090-ABP_both
LLWKALRLWWKVL
>ID29117-NO
LNECQ
>ID29119-NO
LNEERRANRYGFGL
>ID29141-NO
LNKRIICFPDYMFCGVNVFLCCSGNCLLICVP
>ID29167-NO
LNVPGEIVE
>ID29170-NO
LNVVGETVE
>ID29205-ABP_neg
LPLPSCSSHGGDADNTSQRN
>ID29208-NO
LPLYNFGL

>ID29272-NO
LQDKIHP
>ID29298-NO
LQPGS
>ID29303-NO
LQQGG
>ID29358-NO
LRGEPIRFG
>ID29371-NO
LRKFLHKLF
>ID29374-ABP_pos
LRKLLLLLRL
>ID29397-NO
LRNVEQQCRADALVERAQELIHGQQ
>ID29406-NO
LRRGQ
>ID29408-ABP_both
LRRLRKWLRRLLKLL
>ID29424-NO
LRSPKMMHKSGCFGRRLDRIGSLSGLGCNVLRKY
>ID29493-ABP_neg
LSLIAAAGKETIRQYLKNEIKKKGRKAVIAW
>ID29524-ABP_neg
LSVDKRPVLHPEHIYGHNHY
>ID29562-NO
LTNYLATTGHGTNTGGPVLT
>ID29585-NO
LTVSPWT
>ID29587-NO
LTVTPWL
>ID29590-NO
LTWNS
>ID29653-NO
LVSVSPAFNGNYFVE
>ID29675-NO
LVYPFTGPIPN
>ID29701-ABP_pos
LWWLRWL
>ID29726-NO
LYAAIIKKLLES
>ID29747-NO
LYPVK
>ID29753-ABP_both
LYSPTCVKAAVSRFIGKVSA
>ID29833-NO
MAFHETLARCALYGEC
>ID29967-NO
MAKFASIIVLLFVALVVFAAFEEPTMVEA
>ID30224-NO
MARRASVGTD
>ID30366-NO

MCMPCFTTDHQMARKCDDCCGGKGRGKCYGPQCLCR
>ID30387-NO
MDELYPLEVEEEANGGEVLG
>ID32605-NO
MLLLTRRRST
>ID32616-NO
MLPAY
>ID32617-NO
MLPKPSSFPVPG
>ID32644-NO
MLTKFETKSARVKGLSFHPKRPWIL
>ID32681-NO
MMLGPGIL
>ID32690-ABP_both
MMRVMRRKTKVIWEKKDFIGLYSID
>ID33148-NO
MRNYSFGL
>ID33223-ABP_both
MSDNKPDISDVTSFDKTKLKKTETQEKNTLPTKEIEQEKSESS
>ID33345-NO
MSSGGLLLLLGLLTLCAELTPVSSKDRH
>ID33651-NO
MVPVPVHHMADELLRNGPDTVI
>ID33667-NO
MVRRFLVTLRIRRACGPPRVRV
>ID33773-NO
NAAANDKASGVNGCCSNPACHVDHAELC
>ID33861-NO
NDVNTMADAYKFLQDLDTYYGDRARVRF
>ID33878-NO
NEYHGFVDKANNENKRKKQQGRDDFVVKPNNFANRRRKDDYNENYYDDVD
>ID33907-NO
NFDEIDRSGYGFV
>ID33908-NO
NFDEIDRSS
>ID33929-NO
NFGAPGGGKY
>ID33953-NO
NGFWN
>ID33954-NO
NGFYN
>ID33955-NO
NGGTADALYNLPDLEKI
>ID33959-NO
NGKCVLVTL
>ID33970-NO
NGRCCHPACAKYFSCGR
>ID33981-NO
NGRKISLDLRAPLYKKIIKKLLES
>ID34013-NO
NIENSTLATPLS

>ID34033-ABP_both
NILLWG
>ID34093-NO
NLAKGKEESLDSDLYAELRCMCIKTTSGIHPKNIQSLEVIGKGTHCNQVE
>ID34318-NO
NLHLPLP
>ID34319-NO
NLHLPLPLL
>ID34322-NO
NLINFMEMIRYTIP
>ID34328-NO
NLLQFAFMIRQANKRRRPVIPYEEYGLYYM
>ID34370-NO
NLYYASW
>ID34389-NO
NNANVFYPWG
>ID34396-NO
NNFSDCFWKYCV
>ID34579-NO
NRSFLRF
>ID34584-NO
NRVYIHPFTL
>ID34595-NO
NSDEQFDDYGYMRF
>ID34661-NO
NTGRVHRQPKVVIRNPFHAWG
>ID34662-NO
NTHMTAF
>ID34689-NO
NVLLSPLSVATALSALSLGAEQRTES
>ID34696-ABP_both
NVMTYWWLDPPL
>ID34734-NO
NYCQEKWDYCPVPFLGSRYCCDGLFCTLFFCA
>ID34767-NO
PALIPFPRV
>ID34770-NO
PANIKWGD
>ID34776-NO
PAPESNFVRDP
>ID34788-NO
PAWHHAFHWAWRMLKKAA
>ID34811-NO
PDMYAFGL
>ID34876-NO
PGLKGKRGDSGSPATWTTRG
>ID34913-NO
PIDERLRTCERLSYP
>ID34960-NO
PKMTLQRSNIRPSMP
>ID34977-NO

PKWLLFS
>ID35006-NO
PLPLL
>ID35007-NO
PLPSKETIEQEKQAGES
>ID35014-NO
PLTQTP
>ID35030-NO
PNFLRF
>ID35060-ABP_both
PPPPPPP
>ID35071-NO
PPQSVLSLSQSKVLPVPQ
>ID35091-NO
PQNSKIPGPTFLDPH
>ID35102-NO
PQRDMP
>ID35107-NO
PQVGLIPFPRV
>ID35119-NO
PRHQG
>ID35135-NO
PRNYAFGL
>ID35148-ABP_both
PRPRCRRRFCRPRP
>ID35162-NO
PRVYGFGL
>ID35170-NO
PSAALAVEHGTTHPLE
>ID35191-NO
PSMRLRF
>ID35211-NO
PTHIDW
>ID35235-NO
PVKVYPNGAENESAEAFPVEV
>ID35238-NO
PVKVYTNGVEEESSEAFPSEM
>ID35246-NO
PVRAVP
>ID35250-ABP_both
PVVFSVASKVVPSLISALKR
>ID35273-NO
PYDRISGSAFSDF
>ID35276-NO
PYGGYGW
>ID35302-NO
QAEQLPPEGSYAGSDELEGMA
>ID35361-NO
QCESGPCCRNCKFLKEGTICKRARGDDMDDYCNGKTCDCPRNPHKGPAT
>ID35371-NO
QCRVEGEICGMLFEAQCCDGWCFFVCM

>ID35410-ABP_both
QDGMYQRFLRQHVHPEETGGSDRYSNLMMQRRKMTLYHSKRFNTFIH
>ID35418-NO
QDLDHVFMRF
>ID35452-NO
QEEVLKACGREFVRLQIRICGSLSWG
>ID35471-NO
QEPHRHSIFTPQTNPRADLEKN
>ID35500-NO
QFTQESCTASNQCWSICKRLHNTNRGKCMNKKCRCYS
>ID35503-NO
QFYRI
>ID35516-NO
QGGPPRPQIPP
>ID35539-NO
QGRPLGPPIPP
>ID35541-NO
QGRPPRPHIPP
>ID35579-NO
QHWSYEFMPG
>ID35595-ABP_both
QIAALEQEIAALEQEIAALQ
>ID35597-NO
QICCGYGDCGFVPNVCV
>ID35603-NO
QIGLF
>ID35637-NO
QKAGLW
>ID35660-NO
QKELVVTATTTCCGYNPMTSCPRCMCDSSCNKKKP
>ID35748-NO
QLDRNFLRF
>ID35764-NO
QLKSTCRIAEAWKGAKECNAKCAALGTTRGGVCQKFLGDLYCCCWD
>ID35782-NO
QLNFTPWW
>ID35812-NO
QLWAVGSLM
>ID35821-NO
QMIVIELGTNPLKSSGIENGAFQGMK
>ID35833-NO
QNCCSIPSCWEKYKCS
>ID35842-NO
QNIPP
>ID35843-NO
QNLIPFPRV
>ID35883-NO
QPGLW
>ID35886-NO
QPIIITSPYLPS
>ID35984-NO

QRIRKSKISRTL
>ID36015-NO
QRTESIIHRALYYDLIS
>ID36016-NO
QRTTNLVCECCFNYCTPDVVRKYCY
>ID36026-NO
QSCCAAPSCFMLC
>ID36042-ABP_both
QSHLSMCSVCCNCCKNYKGCGFCCRF
>ID36055-NO
QSPGLW
>ID36059-ABP_both
QSQYGCPIISNMCEDHCRRKKMEGQCDLLDCVCS
>ID36065-NO
QSSCSLSSRPHPRGICGSNLAGFRAFICSNQNSPS
>ID36068-NO
QSTNDFIKACGRELVRLWVEICGSVSWGRTAL
>ID36093-NO
QTLIPMPRL
>ID36182-NO
QWSSMRGAW
>ID36197-NO
QYREGLV
>ID36228-NO
RAILHAPREQECCEPQWCDGGCDACC
>ID36269-NO
RCCISPACHKDCYCCL
>ID36277-NO
RCCPMPGCFAGPFCPCCPP
>ID36284-NO
RCCTFSECDGNCHCCQ
>ID36299-NO
RCICVLGICRLL
>ID36301-NO
RCKPCFTTDPQMSKKCADCCGGKGKGKCYGPQCLC
>ID36305-NO
RCLPSGKACAGVTQKIPCCGSCVRGKCS
>ID36308-NO
RCPET
>ID36312-ABP_both
RCRCRCRCRC
>ID36335-NO
RDCRPVGQYCGIPYEHNWRCCSQLCAIICVS
>ID36337-NO
RDEEVTQREE
>ID36341-NO
RDMPIQAF
>ID36445-ABP_both
RFRRLRCKTRCRLKKI
>ID36447-ABP_both
RFRRLRIKTRIRLKKI

>ID36477-NO
RFWWF
>ID36513-NO
RGGRLAYLRRRWAVLGR
>ID36540-NO
RGPSGPLRF
>ID36542-NO
RGQQNE
>ID36629-ABP_both
RHRHRHRHRHRH
>ID36686-NO
RIHYIF
>ID37234-NO
RLIMRIYSPTTRRYG
>ID37244-ABP_both
RLKKIGKVLKWI
>ID37278-ABP_both
RLLRKFFRKLKKSV
>ID37309-NO
RLRAS
>ID37336-NO
RLVPSGPNPLHN
>ID37350-ABP_both
RLWKRWWIR
>ID37396-NO
RMKWKK
>ID37401-NO
RMLGQTPTK
>ID37409-NO
RMTLSEKCCQVGCIRKDIARLC
>ID37446-ABP_both
RNSVRNRVMLWRTKR
>ID37460-NO
RPCCSLRWCSNYCRCGI
>ID37480-ABP_both
RPHGAGEGIDRVPAGPSPSEVGLAIPSGK
>ID37523-NO
RPPSWIPK
>ID37532-ABP_neg
RPRFPPPFPIPRIPRIP
>ID37606-NO
RQIKIWFQNRRMKWKKDIMGEWGNEIFGAIAGFLG
>ID37608-NO
RQIKIWFQNRRMKWKKTYADFIASGRTGRRNAI
>ID37861-ABP_both
RRGFWKRLRRRLRRFGDRIRNRFRNFREKLPDPFPG
>ID37903-ABP_neg
RRIRPRPRC
>ID37952-NO
RRLSYSRRRF
>ID37990-NO

RRPYIL
>ID38060-NO
RRRRRRRRRRRTYADFIASGRTGRRNAI
>ID38071-ABP_both
RRRRRWFWFW
>ID38168-ABP_both
RRWHRHWRR
>ID38366-ABP_both
RRWWCF
>ID38376-ABP_both
RRWWCR
>ID38378-ABP_both
RRWWCS
>ID38402-ABP_both
RRWYRWWRRRWYRWWR
>ID38407-NO
RRYKKFKWRYRGRFWFW
>ID38429-NO
RSCIKHQCP
>ID38444-NO
RSIAVVVL
>ID38471-NO
RSSEDLSAPEDRSLS
>ID38480-NO
RSTQGYGRMDPIL
>ID38560-NO
RTRYED
>ID38589-NO
RVCFLWQDGRCVF
>ID38598-ABP_both
RVCSAIPWPICH
>ID38601-NO
RVDGNSDQKAVIGAMLAKDLQTRKAGSSTGRYAVLPNR
>ID38846-NO
RWRWKCCKK
>ID39111-ABP_both
RYRRKKKMKKALQYIKLLKE
>ID39145-NO
SAGATANLPLRS
>ID39146-NO
SAGPYAFGL
>ID39171-NO
SAPFIECHGRGTCNYYANS
>ID39198-NO
SAVDWWRL
>ID39221-NO
SCCPEEPCCFW
>ID39263-NO
SCLEYGHSCWGAH
>ID39273-NO
SCNTASCLTHRLAGLLSSAGSMANSNLLPTEMGFKVS

>ID39378-NO
SDGRNAARKAFGCCDLIPCLERYGNRCNEVH
>ID39397-NO
SDKPDMAEIEKFDKSKLKKTETQEKNPLPSKETIEQEKQAGES
>ID39404-NO
SDMPGVLRF
>ID39454-NO
SEDRSAGNSLKDSSLFSSARF
>ID39455-NO
SEDRSSGNSLKESSFFSPGRY
>ID39467-NO
SEFIHHWTPPPS
>ID39498-NO
SETGNTVTVKGFSPLR
>ID39501-NO
SEWSDCSVTCGKGMRTRQR
>ID39510-NO
SFDRMGGTEFGLM
>ID39519-NO
SFGGNPTGDPNLNIYSFGL
>ID39577-NO
SGFYADRY
>ID39678-NO
SGWFAGS
>ID39705-NO
SIDSTTF
>ID39798-NO
SKLAYD
>ID39809-NO
SKPANFIRF
>ID39817-NO
SKQCCHLAACRFRCTPCCW
>ID39854-NO
SLAAPQRF
>ID39865-NO
SLCDKPHHNCIDGQTCYHTCCQNGLKCVRYP
>ID39901-NO
SLLADTQSGHRW
>ID39983-NO
SMIGGVMSKG
>ID40009-NO
SMSIASPYIALE
>ID40024-ABP_both
SNGPVKKWGSIKGLK
>ID40047-NO
SNLFGNLVNQFQQDDVMQQ
>ID40062-NO
SNTDVLTPDLLFGEAEIRLQSRYDDPLMG
>ID40107-NO
SPKYNFGL
>ID40114-NO

SPLQEAEDNSSLETADSLLEDLRGVPNM
>ID40115-NO
SPLQEAEDNSSLETTDPLLEDLMGVSNV
>ID40118-NO
SPMDRSKMVRF
>ID40152-NO
SPSNSKCPDGPDCFVGLM
>ID40206-NO
SQRQTLD
>ID40208-NO
SQSKVLPVPQ
>ID40246-NO
SRNAT
>ID40272-ABP_both
SRSEKAGLQFPVGRIGRMLKK
>ID40318-NO
SSFSPPRGPPGWGPPCVQQPCPKCPYDDYKCPTCDKFPECEECPHISIGC
>ID40444-NO
STRVMAHLPLRL
>ID40447-NO
STSCVEAGSYCRPNVKLCCGFCSPYSKICMNFPKN
>ID40459-NO
SVAKL
>ID40465-NO
SVAVAGAIIKGAALTFNVLQ
>ID40498-NO
SVGNPNNLPPRERKAGCKNFYWKGFTSC
>ID40505-NO
SVIELGKMILQETGKNPVTHYGA
>ID40548-NO
SVSSLARTGDLPVREQ
>ID40550-ABP_both
SVVFGVASKVVPSVIGKVKT
>ID40565-ABP_both
SWIKKDKFPSSTGPYNPNPPPPRF
>ID40650-NO
SYGISSGCFGLKLDRIGTMSGLGCWRLLQDSP
>ID40713-NO
TAQVTSTEV
>ID40820-NO
TDPLWQLPGAHLEQYLS
>ID40821-NO
TDPLWSSFNENALLEE
>ID40822-NO
TDPSWAMYNEHQLTTGQQAQPANEASE
>ID40835-ABP_both
TEAVESTVATL
>ID40842-NO
TEGPGMWFGPRL
>ID40849-NO
TEWEAQWHWSHCRQKKKKIKIKIK

>ID40903-NO
TGPIPNSLPQ
>ID40904-NO
TGPQTTCQASTCEAGCKQIGKSMKSCQGDTCECA
>ID40926-ABP_both
THSWLWLW
>ID40930-ABP_both
TIAMRAINNYRWRSKNQNTFLR
>ID40937-NO
TIINVKCTS
>ID40939-NO
TIINVKCTSPKQCS
>ID40942-NO
TIIPLPV
>ID40958-ABP_both
TKAVEHLDDLPGALSELSDL
>ID40959-ABP_both
TKAVEHLDDLPGALSELSDLHAHKLRVDPVNFKLLSHSLL
>ID40966-ABP_both
TKCGQWQRN
>ID41005-NO
TKRRITPKDVIDV
>ID41023-NO
TKYVSKQFFSWG
>ID41035-NO
TLLNILRDGDNCCIDKQGCCPW
>ID41039-NO
TLPSPLALLTVH
>ID41063-NO
TLTSFGEWR
>ID41148-NO
TPLFCGNHGRQPSPLCMKWD
>ID41157-NO
TPPAGPDGGPP
>ID41196-NO
TQPKTNAIPY
>ID41214-NO
TRDCKTKGSVCFASSECCIQDCWFVCLY
>ID41224-NO
TRGKQ
>ID41251-NO
TRQARRNRRRRWRERQRAAAACYGRKKRRQRRR
>ID41257-NO
TRRSKRRSHRKF
>ID41307-NO
TSQDITSGMWFGPRL
>ID41312-NO
TSRCYVYRLKVVCS
>ID41356-ABP_neg
TTSIRRRYQVSLIRRHRGKR
>ID41399-NO

TVGDVNTDRPGMLDF
>ID41481-NO
TYGIYDAKPPFSCAGLRGGCVLPPNLRPKFKE
>ID41514-NO
VAHINVGK
>ID41539-NO
VAQGPSAFVAG
>ID41598-NO
VCYNQ
>ID41658-NO
VELPPPVELPPPVELPPP
>ID41765-NO
VGINVKCKHSGQCLKPCKDAGMRFGKCINGKCDCTPK
>ID41767-NO
VGINYWLAHKHK
>ID41793-NO
VHIPP
>ID41801-NO
VHLPP
>ID41802-NO
VHLPPP
>ID41805-ABP_both
VHLRCKAAFC
>ID41840-NO
VIGGAEENINEHRSL
>ID41850-NO
VIGGDECNINEHRSLALVY
>ID41897-ABP_both
VIVKAIATLASKLL
>ID41912-ABP_both
VKDLAKFIAKTVAKQGGCYL
>ID41932-NO
VKHTT
>ID41939-ABP_neg
VKKFAWWWAFLKK
>ID41949-ABP_both
VKKVYPLKVKLYP
>ID42008-ABP_both
VKRWKKFWRKWKKWW
>ID42056-ABP_both
VLIKTRLFIKRK
>ID42090-ABP_both
VLNENLRR
>ID42097-NO
VLPHVVPVIAEHL
>ID42229-NO
VPMIK
>ID42230-NO
VPMLK
>ID42235-NO
VPNTNSLPAAVN

>ID42251-NO
VPSERY
>ID42266-NO
VPWMEPAYQRFL
>ID42268-NO
VPYPQRDMPIQA
>ID42273-NO
VQETQKLAKTVGANLEETNKKLAPQIKSAYDDFVKQAQEVQKKLHEAASK
>ID42543-ABP_both
VRRVRKWVRRVLKLV
>ID42545-NO
VRSCMFGNGK
>ID42551-NO
VRTPE
>ID42559-NO
VRWCT
>ID42596-NO
VSHNNFLRF
>ID42601-NO
VSITKCSSDMNGYCLHGQCIYLVDMSQNYCRCEVGYTGVRCEHFFL
>ID42628-NO
VSRSSRWGSI
>ID42701-NO
VVAPFPE
>ID42710-ABP_both
VVFKLASKVVPSVYCTITKK
>ID42715-NO
VVGGDCIPQVPFLAFLYSEYFC
>ID42917-NO
WCCRQFN
>ID42932-NO
WCSTCLDLACGASRECYDPCFKAFGRAHGKCMNNKCRCYT
>ID43012-NO
WGTGLC
>ID43042-ABP_both
WHWRIRKKLR
>ID43101-NO
WIVGVCRLPGDLCAGDASCCEHSCNIVHTCD
>ID43114-NO
WKCRRQCFRVLHHWN
>ID43136-ABP_both
WKKLWKPGLKKWLK
>ID43151-ABP_both
WKPGKW
>ID43157-ABP_both
WKRIVRRIKRWLR
>ID43199-ABP_both
WLKGLIKFIR
>ID43393-ABP_both
WRNGRWWRNGRWWRNGRW
>ID43398-NO

WRPCES
>ID43409-NO
WRRRIR
>ID43425-ABP_both
WRWKWRWK
>ID43453-ABP_both
WSKILGHLIR
>ID43472-NO
WTCRASWCS
>ID43517-NO
WWGDNGCSLWGSCTVDAECCLGNCGGMYCSLL
>ID43550-ABP_both
WWPWRR
>ID44288-NO
YAEAAGEQVPEYQALVRDYPQLLDSGMKRQDVVHSFLRF
>ID44292-NO
YAFGL
>ID44366-NO
YDFEASSYSFGL
>ID44381-NO
YDSFAYSAGL
>ID44426-NO
YGGFF
>ID44435-NO
YGGFMKKKFMRF
>ID44452-NO
YGGVSLPEW
>ID44513-NO
YIPIQYVLSR
>ID44615-NO
YKPITNF
>ID44634-NO
YKSDSFYGLM
>ID44636-NO
YKVPQL
>ID44666-NO
YLQQARKGPTGRISMMGNRVQNIDPTHRINDRDYMGWMDF
>ID44709-NO
YPENGFPEN
>ID44724-NO
YPGFQGLF
>ID44725-NO
YPGIA
>ID44728-NO
YPHYSLPGSSTL
>ID44749-NO
YPQEHRFSFGL
>ID44767-NO
YPTKPESPGPDATPEELAEYMTKIRQYINLVTRQRY
>ID44781-NO
YPYDVPDYASL

>ID44782-NO
YPYLIFPASPSSGDSRRLV
>ID44790-NO
YQEPVLGPVRG
>ID44818-NO
YRGGLEPINF
>ID44880-NO
YSSQHLCGSNLVEALYMTCGRSGFYRPHD
>ID44886-NO
YSTCSSLF

Table S2. Minimum (Min) and maximum (Max) boundaries of the applicability domains defined for the models derived from the training and the production datasets. The first model distinguishes antibacterial peptides from non-antibacterial peptides (ABP) and the second model categorizes antibacterial peptides as anti-Gram+, anti-Gram- or anti- both types of bacteria (Gram).

	Training (ABP)		*Training (Gram)*		*Production (ABP)*		*Production (Gram)*	
Descriptor	Min	Max	Min	Max	Min	Max	Min	Max
ISA_NO_PCR_N2	52,98	581,41	52,98	511,043	52,98	581,41	52,98	511,043
Z3_NO_PLR_I50	-3,44	7,57	-3,44	7,57	-3,44	7,57	-3,44	7,57
Gs(U)_NO_PLR_I50	-1015,71	1338,19	-1015,709	1338,189	-1015,71	1338,19	-1015,709	1338,189
IP_NO_PRT_I50	0	7,99	0	6,52	0	7,99	0	6,52
IP_NO_PLR_Q2	2,77	10,76	2,77	10,76	2,77	10,76	2,77	10,76
Gs(U)_NO_PRT_I50	0	1793,24	0	1793,239	0	1793,24	0	1793,239
IP_NO_AHR_I50	0	9,74	0	9,74	0	9,74	0	9,74
Z3_NO_PRT_N1	-116,18	50,54	-113,52	46,65	-116,18	50,54	-113,52	46,65
IP_NO_PRT_Q1	2,77	10,76	2,77	10,76	2,77	10,76	2,77	10,76
Z2_NO_PLR_I50	-2,09	4,61	-2,09	4,61	-2,09	4,61	-2,09	4,61
Z3_NO_PRT_CV	-6,38346E+16	9,45044E+16	-3,61425E+16	1,96182E+16	-6,38346E+16	9,45044E+16	-3,61425E+16	1,96182E+16
Z3_NO_PLR_Q2	-3,44	4,13	-3,44	4,13	-3,44	4,13	-3,44	4,13
Z2_NO_AHR_I50	-1,73	3,47	-1,73	3,47	-1,73	3,47	-1,73	3,47
IP_NO_PRT_RA	0	7,99	0	7,99	0	7,99	0	7,99
ISA_NO_PLR_Q3	17,87	132,16	17,87	132,16	17,87	132,16	17,87	132,16
ECI_NO_AHR_I50	0	1,36	0	1,36	0	1,36	0	1,36
Z3_NO_AHR_I50	-3,14	7,27	-3,14	7,27	-3,14	7,27	-3,14	7,27
Gw(U)_NO_PCR_SI5	0	1	0	1	0	1	0	1
IP_NO_AHR_RA	0	6,52	0	6,52	0	6,52	0	6,52
Z1_NO_PCR_V	0	0,11	0	0,11	0	0,11	0	0,11
Z3_NO_ALR_N3	-3,72	6,5	-3,721	6,004	-3,72	6,5	-3,721	6,004
Z1_NO_NPR_N1	-100,56	55,75	-90,82	55,75	-100,56	55,75	-90,82	55,75

Gw(U)_NO_PCR_MX	-35,07	-1,72	-34,315	-1,718	-35,072	-1,718	-34,315	-1,718
Z3_NO_AHR_RA	0	7,27	0	7,27	0	7,27	0	7,27
ISA_NO_PRT_Q1	17,87	189,42	17,87	189,42	17,87	189,42	17,87	189,42
IP_NO_PLR_N3	2,77	33,38	2,77	31,037	2,77	33,38	2,77	31,037
Mw_NO_AHR_I50	0	137	0	137	0	137	0	137
ECI_NO_PLR_MN	0,15	1,69	0,15	1,69	0,15	1,69	0,15	1,69
ECI_NO_PLR_TI5	0	75,86	0	74,98	0	79,338	0	79,338
IP_NO_AHR_MX	3,22	9,74	3,22	9,74	3,22	9,74	3,22	9,74
Z3_NO_PLR_SI5	0	1	0	1	0	1	0	1
Gw(U)_NO_AHR_I50	-28,91	30,86	-28,359	28,912	-28,91	30,86	-28,359	28,912
ECI_NO_PCR_MN	0,53	1,69	0,53	1,69	0,53	1,69	0,53	1,69
IP_NO_AHR_DE	0	4,61	0	4,61	0	4,61	0	4,61
Gs(U)_NO_AHR_P2	185,74	818,65	185,74	818,649	185,74	818,65	185,74	818,649
Gs(U)_NO_BSR_N1	-4052,65	14716,69	-2472,433	14716,693	-4052,65	14716,69	-2472,433	14716,693
Mw_NO_PLR_Q2	87	163	87	163	87	163	87	163
Z2_NO_RTR_P3	-5,36	1,45	-5,36	1,45	-5,36	1,45	-5,36	1,45
Z2_NO_NPR_SI5	0	1	0	1	0	1	0	1
Mw_NO_RTR_P3	57	115	57	115	57	115	57	115
ECI_NO_PLR_Q3	0,15	1,69	0,53	1,69	0,15	1,69	0,53	1,69
Z2_NO_PLR_MI5	0	2,58	0	2,55	0	2,58	0	2,561
ISA_NO_PLR_N2	17,87	581,41	17,87	513,006	17,87	581,41	17,87	513,006
Z2_NO_PRT_RA	0	9,01	0	9,01	0	9,01	0	9,01
Z3_NO_NPR_I50	-1,29	3,52	-1,29	3,52	-1,29	3,52	-1,29	3,52
ECI_NO_PLR_CV	0	1,44	0	1,263	0	1,44	0	1,263
Z2_NO_RTR_I50	-5,36	6,81	-5,36	6,81	-5,36	6,81	-5,36	6,81
Mw_NO_PRT_I50	0	115	0	99	0	115	0	99
HP_NO_PLR_Ar	-4,5	2,5	-4,5	1,3	-4,5	2,5	-4,5	1,3
ECI_NO_RTR_I50	0	1,31	0	1,31	0	1,31	0	1,31
Gw(U)_NO_PCR_P3	-37,77	-1,72	-37,77	-1,718	-37,77	-1,718	-37,77	-1,718
Z1_NO_PRT_MX	-4,92	3,64	-4,92	3,64	-4,92	3,64	-4,92	3,64

Z2_NO_AHR_P2	0,27	1,74	0,27	1,74	0,27	1,74	0,27	1,74
Mw_NO_PRT_RA	0	129	0	129	0	129	0	129
ISA_NO_AHR_DE	0	95,33	0	95,332	0	95,332	0	95,332
Mw_NO_PRT_Q2	57	186	57	186	57	186	57	186
Z3_NO_PLR_P2	0,01	4,13	0,01	3,869	0,01	4,13	0,01	3,879
Gs(U)_NO_PCR_Q1	-1015,71	-583,48	-1015,709	-583,484	-1015,71	-583,48	-1015,709	-583,484
ISA_NO_PLR_CV	0	1,24	0	1,208	0	1,24	0	1,208
Z3_NO_PLR_N2	0,01	19,46	0,01	16,852	0,01	19,46	0,01	16,852
Z3_NO_RTR_I50	0	2,36	0	2,36	0	2,36	0	2,36
Z1_NO_PRT_SI5	0	1	0	1	0	1	0	1
Z1_NO_NPR_I50	-4,92	7,15	-4,92	7,15	-4,92	7,15	-4,92	7,15
Z1_NO_PRT_P2	0,9	4,92	0,901	4,92	0,9	4,92	0,901	4,92
Z1_NO_PLR_N1	-4,17	108,88	-1,39	97,02	-4,17	108,88	-1,39	102,74
ECI_NO_ALR_P3	0,01	0,34	0,01	0,34	0,01	0,34	0,01	0,34
ISA_NO_NPR_SI5	0	1	0	1	0	1	0	1
Mw_NO_RTR_RA	0	58	0	58	0	58	0	58
Z2_NO_PCR_Q3	1,41	2,52	1,41	2,52	1,41	2,52	1,41	2,52
ISA_NO_PLR_V	0	6531,1	0	5198,94	0	6531,1	0	5198,94
ISA_NO_NPR_I50	0	189,42	0	189,42	0	189,42	0	189,42
Z1_NO_AHR_Q2	-4,19	3,08	-4,19	3,08	-4,19	3,08	-4,19	3,08
Z1_NO_PRT_Q1	-4,92	3,64	-4,92	3,64	-4,92	3,64	-4,92	3,64
Mw_NO_ALR_SI5	0	1	0	1	0	1	0	1
IP_AC1_PRT_Q2	15,35	231,56	15,346	231,555	15,35	231,56	15,346	231,555
IP_AC1_AHR_Q1	8,92	209,6	10,368	209,605	8,919	209,6	10,368	209,605
Z1_AC1_PCR_Q3	-28,34	20,97	-27,946	19,757	-28,34	20,97	-27,946	19,757
Z1_AC1_PCR_MN	-28,34	20,97	-28,339	19,354	-28,34	20,97	-28,339	19,354
ECI_AC1_PCR_Q3	0,01	5,71	0,005	5,712	0,005	5,712	0,005	5,712
HP_AC1_PCR_MN	-40,5	36	-40,5	31,5	-40,5	36	-40,5	31,5
Z2_AC1_PCR_MI5	0	2,58	0	2,585	0	2,585	0	2,585
Z1_AC1_PCR_CV	-6194,93	4647,27	-2936,387	2488,821	-32640,381	4647,27	-3155,779	2674,221
IP_AC1_RTR_Q3	14,04	139,45	14,044	139,45	14,04	139,45	14,044	139,45

Mw_AC1_PCR_Q2	7296	58032	7296	58032	7296	58032	7296	58032
Mw_AC1_NPR_MX	4959	69192	7171	69192	4959	69192	7171	69192
IP_AC1_RTR_MX	14,04	139,45	14,044	139,45	14,04	139,45	14,044	139,45
IP_AC1_RTR_Q2	14,04	139,45	14,044	139,45	14,04	139,45	14,044	139,45
Mw_AC1_PCR_SI5	0	1	0	1	0	1	0	1
Mw_AC1_PCR_Q1	7296	58032	7296	58032	7296	58032	7296	58032
IP_AC1_ALR_Q2	16,54	139,45	16,537	139,45	16,54	139,45	16,537	139,45
ISA_AC1_PCR_SI5	0	1	0	1	0	1	0	1
Z2_AC1_PCR_MN	-27,01	18,4	-27,014	18,396	-27,014	18,4	-27,014	18,396
Z2_AC1_PCR_Q2	-27,01	18,4	-27,014	18,396	-27,01	18,4	-27,014	18,396
Gs(U)_AC1_BSR_MN	-1579487,69	1057312,79	-1579487,692	893700,369	-1579487,692	1133208,399	-1579487,692	893700,369
ISA_AC1_ALR_MX	356,15	58473,95	367,908	58473,954	356,15	58473,954	367,908	58473,954
ISA_AC1_PCR_P3	946,75	38937,18	1034,699	38937,175	946,75	38937,18	1034,699	38937,175
Gs(U)_AC1_PLR_Q2	-1579487,69	2063330,66	-1579487,692	2063330,664	-1579487,69	2063330,664	-1579487,692	2063330,664
ISA_AC1_PRT_M	470,47	55813,23	511,157	55813,234	470,47	55813,23	511,157	55813,234
IP_AC1_ARM_Q1	15,18	163,34	15,18	163,337	15,18	163,34	15,18	163,337
Z3_AC1_RTR_MN	-16,24	11,69	-16,237	9,747	-16,24	11,69	-16,237	9,747
IP_AC1_BSR_N3	15,18	296,86	15,678	296,859	15,18	300,115	15,678	296,859
Gs(U)_AC1_PCR_Q1	-1579487,69	2063330,66	-1579487,692	2063330,664	-1579487,692	2063330,664	-1579487,692	2063330,664
Z1_AC1_ALR_MN	-32,32	43,69	-32,323	43,69	-32,32	43,69	-32,323	43,69
Gs(U)_AC1_PCR_CV	-4468,31	33259,35	-4468,312	8490,981	-4468,31	33259,35	-4468,312	8490,981
Z3_AC1_PCR_Ar	-28,41	22,19	-28,414	21,977	-28,41	22,19	-28,414	21,977
Z2_AC1_PCR_CV	-4,1852E+16	4,74163E+15	-4,1852E+16	997,75	-4,1852E+16	4,74163E+15	-4,1852E+16	997,75
Z2_AC1_UFR_Q2	-39,13	57,46	-39,128	57,459	-39,13	57,46	-39,128	57,459
Z2_AC1_PCR_MX	-27,01	18,4	-19,883	18,396	-27,01	18,4	-19,883	18,396

IP_AC1_NPR_MN	15,18	139,45	15,18	129,55	15,18	139,45	15,18	129,55
HP_AC1_AHR_MN	-35,1	35,1	-35,1	35,1	-35,1	35,1	-35,1	35,1
IP_AC1_BSR_MN	15,18	129,55	15,18	129,55	15,18	129,55	15,18	129,55
Z2_AC1_PRT_MX	-13,51	57,46	-13,507	57,459	-13,51	57,46	-13,507	57,459
Gw(U)_AC1_PCR_S	-3,68	3,2	-2,584	3,201	-3,68	3,2	-2,584	3,201
Z3_AC1_UCR_N3	-41,54	54,4	-41,541	54,398	-41,54	54,4	-41,541	54,398
HP_AC1_UFR_Q3	-14,4	14,4	-13,92	14,4	-14,4	14,4	-14,4	14,4
IP_AC1_UFR_MN	16,54	139,45	16,537	139,45	16,537	139,45	16,537	139,45
Z3_AC1_PCR_N2	0,01	109,83	0,022	86,609	0,01	109,83	0,022	86,609
Z3_AC1_UFR_N3	-35,87	22,93	-25,566	22,927	-35,87	22,93	-29,715	22,927
Mw_AC1_AHR_Q2	4047	50964	4047	50964	4047	50964	4047	50964
ISA_AC1_BSR_Q2	1062,19	71759,87	2231,999	71759,873	1062,19	71759,87	2231,999	71759,873
ISA_AC1_UFR_Q3	356,15	46351,07	356,149	46351,074	356,149	46351,074	356,149	46351,074
Mw_AC1_ALR_N3	4959	82987,4	5871	76407,394	4959	82987,4	5871	76407,394
ECI_AC1_PRT_MX	0	5,71	0,002	5,712	0	5,712	0,002	5,712
ISA_AC1_UCR_MX	329,88	50067,49	393,618	50067,494	329,88	50067,49	393,618	50067,494
Z3_AC1_RTR_CV	-1,43686E+16	77823,08	-479,559	1577,997	-1,43686E+16	81603,356	-479,559	1577,997
Gs(U)_AC1_NCR_Q3	-882104,01	1229493,56	-882104,009	1229493,558	-882104,01	1229493,56	-882104,009	1229493,558
ISA_AC1_ARM_Q1	1561,48	65116,91	1561,481	65116,913	1561,48	65116,91	1561,481	65116,913
Gw(U)_AC1_PCR_G	0	2621,92	0	2621,918	0	2621,92	0	2621,918
Z2_AC1_UCR_Q1	-24,87	22,4	-24,87	17,474	-24,87	22,4	-24,87	17,474
Mw_AC1_ARM_Q3	7809	69192	7809	69192	7809	69192	7809	69192
Z2_AC1_NPR_MN	-39,13	28,73	-39,128	22,52	-39,13	28,73	-39,128	22,52
Z2_AC1_PCR_N3	-46,08	33,66	-43,325	32,454	-46,08	33,66	-43,325	32,454
Z3_AC1_ALR_MN	-15,34	11,69	-15,342	9,232	-15,342	11,69	-15,342	9,232
IP_AC1_ARM_Q3	15,18	163,34	15,18	163,337	15,18	163,34	15,18	163,337
Gw(U)_AC1_PCR_MN	0	2226,07	0	1884,606	0	2226,07	0	1884,606

Z1_AC1_BSR_Q3	-33,06	48,41	-28,31	48,413	-33,06	48,41	-28,31	48,413
Mw_AC1_ALR_Q3	4959	48732	5871	44802	4959	48732	5871	44802
HP_AC1_ALR_Q2	-40,5	40,5	-40,5	40,5	-40,5	40,5	-40,5	40,5
Gs(U)_AC1_PCR_I50	-1579487,69	2999111,86	-1480343,057	2999111,859	-1579487,692	2999111,86	-1480343,057	2999111,859
Z3_AC1_UCR_Q1	-28,41	26,27	-28,414	17,057	-28,414	26,27	-28,414	17,057
ECI_AC1_AHR_Q3	0	4,6	0	4,597	0	4,6	0	4,597
Mw_AC1_UCR_Q3	4959	60636	4959	60636	4959	60636	4959	60636
Z1_AC1_AHR_Q3	-30,5	41,23	-29,26	40,517	-30,5	41,23	-29,26	40,517
IP_AC1_ALR_RA	0	122,91	0	122,913	0	122,913	0	122,913
Gw(U)_AC1_AHR_MX	0	2326,26	0	2326,259	0	2326,26	0	2326,259
ISA_AC1_NPR_Q3	393,62	71759,87	787,235	71759,873	393,62	71759,87	787,235	71759,873
Z2_AC1_PLR_Q3	-27,01	22,4	-27,014	18,396	-27,01	22,4	-27,014	18,396
Z1_AC1_PCR_N2	0,17	114,76	0,199	78,499	0,17	114,76	0,199	78,499
Mw_AC1_NCR_MN	6555	47988	6555	47988	6555	47988	6555	47988
W(U)_AC1_UCR_MN	0	1104	0	880	0	1104	0	880
ISA_AC1_UCR_Q3	329,88	50067,49	393,618	47355,571	329,88	50067,49	393,618	47355,571
Z1_AC1_UCR_N3	-40,97	39,48	-40,971	32,283	-40,97	39,48	-40,971	32,676
Mw_AC1_PLR_Q2	4959	58032	5757	58032	4959	58032	5757	58032
Z3_AC1_BSR_Q1	-11,56	9,63	-11,564	9,212	-11,564	9,63	-11,564	9,212
Z1_AC1_BSR_MX	-33,06	48,41	-28,31	48,413	-33,06	48,413	-28,31	48,413
Z2_AC1_ARM_Q1	-39,13	22,52	-39,128	22,52	-39,13	22,52	-39,128	22,52
IP_AC2_PRT_Q1	7,67	231,56	7,673	128,69	7,67	231,56	7,673	128,69
Z1_AC2_PCR_MN	-28,34	20,97	-28,339	16,813	-28,34	20,97	-28,339	19,482
IP_AC2_AHR_MX	15,65	209,6	15,651	209,605	15,65	209,605	15,651	209,605
Gs(U)_AC2_AHR_MN	-1381198,42	1663019,09	-1381198,421	1663019,093	-1381198,421	1663019,09	-1381198,421	1663019,093

Z1_AC2_PCR_N2	0,17	98,8	0,169	78,554	0,17	98,8	0,169	78,554
Gs(U)_AC2_PLR_Q1	-1579487,69	2063330,66	-1579487,692	1340372,899	-1579487,692	2063330,66	-1579487,692	1340372,899
Z3_AC2_PCR_Q3	-28,41	23,67	-28,414	23,667	-28,414	23,67	-28,414	23,667
HP_AC2_PLR_MN	-40,5	21,35	-40,5	20,25	-40,5	21,35	-40,5	20,25
IP_AC2_NPR_P3	15,18	136,22	16,537	128,69	15,18	136,22	16,537	128,69
HP_AC2_PLR_MX	-40,5	40,5	-40,5	40,5	-40,5	40,5	-40,5	40,5
IP_AC2_NPR_N3	15,18	348,41	16,537	348,408	15,18	348,41	16,537	348,408
IP_AC2_PCR_Q1	21,02	231,56	21,024	189,735	21,02	231,56	21,024	189,735
IP_AC2_ALR_P3	15,9	139,45	16,537	139,45	15,9	139,45	16,537	139,45
Gs(U)_AC2_PCR_Q1	-1579487,69	2063330,66	-1579487,692	1646412,111	-1579487,692	2063330,66	-1579487,692	1646412,111
IP_AC2_RTR_MN	7,67	139,45	7,673	139,45	7,67	139,45	7,673	139,45
Z1_AC2_PCR_Q3	-28,34	20,97	-28,339	20,966	-28,34	20,97	-28,339	20,966
IP_AC2_RTR_Q2	7,67	139,45	7,673	139,45	7,67	139,45	7,673	139,45
Z3_AC2_PCR_MI5	0	2,58	0	2,585	0	2,585	0	2,585
IP_AC2_ALR_Q2	15,9	139,45	16,537	139,45	15,9	139,45	16,537	139,45
Z1_AC2_PCR_P3	-28,34	20,97	-28,339	17,014	-28,34	20,97	-28,339	19,482
ECI_AC2_PCR_SI5	0	1	0	1	0	1	0	1
Gs(U)_AC2_AHR_N3	-3233254,1	4144046,87	-2790877,168	3312445,968	-3233254,1	4144046,87	-2790877,168	3312445,968
Z3_AC2_PCR_S	-3,09	3,68	-2,475	2,041	-3,09	3,68	-2,475	2,041
Gs(U)_AC2_PCR_CV	-124046,25	5815,45	-2605,349	3167,065	-124046,25	5815,45	-2605,349	3167,065
IP_AC2_UCR_Q2	8,92	126,32	15,734	126,322	8,92	126,322	15,734	126,322
Z3_AC2_UCR_MX	-25,94	34,11	-25,936	34,114	-25,94	34,114	-25,936	34,114
Gs(U)_AC2_PRT_P2	2826,9	1859861,13	4434,144	1828947,155	2826,9	1859861,13	4434,144	1828947,155

Gs(U)_AC2_PLR_Q3	-1579487,69	2063330,66	-1579487,692	2063330,664	-1579487,69	2063330,664	-1579487,692	2063330,664
IP_AC2_ARM_Q2	15,18	163,34	15,18	163,337	15,18	163,34	15,18	163,337
Z2_AC2_PCR_Q1	-27,01	18,4	-27,014	18,396	-27,014	18,4	-27,014	18,396
HP_AC2_PCR_Q3	-40,5	40,5	-40,5	40,5	-40,5	40,5	-40,5	40,5
IP_AC2_BSR_P3	15,18	129,55	16,537	126,753	15,18	129,55	16,537	126,753
IP_AC2_AHR_MN	8,92	209,6	8,919	209,605	8,919	209,6	8,919	209,605
Z2_AC2_ALR_MN	-39,13	37,47	-39,128	28,73	-39,13	37,47	-39,128	28,73
IP_AC2_PCR_V	0	7459,83	0	6571,732	0	7459,83	0	6571,732
Z1_AC2_PRT_N2	0,29	183,29	0,286	126,991	0,29	183,29	0,286	126,991
Gs(U)_AC2_UFR_MX	-304785,56	398150,24	-304785,562	398150,235	-304785,562	398150,24	-304785,562	398150,235
Z1_AC2_PRT_MN	-35,82	24,21	-35,818	24,206	-35,82	24,21	-35,818	24,206
HP_AC2_UCR_Q3	-31,5	31,5	-31,5	31,5	-31,5	31,5	-31,5	31,5
IP_AC2_BSR_Q1	15,18	129,55	15,18	128,474	15,18	129,55	15,18	128,474
HP_AC2_AHR_Q1	-35,1	35,1	-35,1	35,1	-35,1	35,1	-35,1	35,1
ISA_AC2_ARM_Q1	1561,48	71759,87	1561,481	58931,099	1561,48	71759,87	1561,481	59055,473
Mw_AC2_PLR_Q2	4959	58032	4959	58032	4959	58032	4959	58032
Gs(U)_AC2_NCR_Q3	-941182,13	1229493,56	-941182,126	1229493,558	-941182,13	1229493,56	-941182,126	1229493,558
ISA_AC2_RTR_Q1	319,34	46351,07	319,337	41499,896	319,337	46351,07	319,337	41499,896
Z1_AC2_AHR_Q2	-29,78	41,23	-29,26	39,805	-29,78	41,23	-29,26	39,805
HP_AC2_UCR_Q2	-31,5	31,5	-31,5	31,5	-31,5	31,5	-31,5	31,5
ISA_AC2_RTR_Q3	319,34	46351,07	349,001	46351,074	319,34	46351,074	349,001	46351,074
Mw_AC2_RTR_MN	3249	42780	3249	42780	3249	42780	3249	42780
IP_AC2_UFR_MN	16,54	139,45	16,537	139,45	16,537	139,45	16,537	139,45
ISA_AC2_UCR_MN	319,34	50067,49	319,337	50067,494	319,337	50067,49	319,337	50067,494
Z3_AC2_UFR_Q3	-15,34	18,42	-15,342	18,42	-15,34	18,42	-15,342	18,42
IP_AC2_RTR_MX	7,67	139,45	15,346	139,45	7,67	139,45	15,346	139,45
IP_AC2_ALR_RA	0	123,55	0	116,303	0	123,55	0	116,303

IP_AC2_BSR_MN	15,18	129,55	15,18	126,753	15,18	129,55	15,18	126,753
Z2_AC2_PCR_N2	0,13	68,95	0,212	68,951	0,127	68,95	0,212	68,951
Z3_AC2_UFR_Q1	-15,34	18,42	-15,342	18,42	-15,342	18,42	-15,342	18,42
Z3_AC2_PCR_Ar	-28,41	21,52	-28,414	20,286	-28,414	21,52	-28,414	20,286
Z3_AC2_RTR_N3	-31,15	25,63	-31,148	24,654	-31,15	25,63	-31,148	24,654
Z1_AC2_ARM_Q2	-35,82	48,41	-35,818	48,413	-35,82	48,41	-35,818	48,413
ECI_AC2_ARM_MX	0	3,65	0,001	3,65	0	3,65	0,001	3,65
HP_AC2_NCR_P2	0	31,5	0	31,5	0	31,5	0	31,5
Z3_AC2_PLR_MN	-28,41	15,2	-28,414	15,205	-28,414	15,2	-28,414	15,205
ECI_AC2_PLR_Q2	0	5,71	0,005	5,712	0	5,712	0,005	5,712
HP_AC2_ALR_Q1	-40,5	28,88	-40,5	25,2	-40,5	28,88	-40,5	25,2
Z1_AC2_UFR_MN	-21,94	16,23	-21,943	14,54	-21,943	16,23	-21,943	14,54
Z1_AC2_AHR_Q3	-29,78	41,23	-29,26	41,23	-29,78	41,23	-29,26	41,23
HP_AC2_ALR_N3	-97,43	81,23	-97,433	65,602	-97,43	81,23	-97,433	65,602
HP_AC2_RTR_Q1	-31,5	31,5	-31,5	31,5	-31,5	31,5	-31,5	31,5
Gs(U)_AC2_UCR_Q2	-1001308,09	1308037,85	-1001308,086	1308037,846	-1001308,09	1308037,85	-1001308,086	1308037,846
ECI_AC2_PLR_Q3	0	5,71	0,005	5,712	0	5,712	0,005	5,712
Gs(U)_AC2_NCR_I50	-941182,13	1787105,08	-941182,126	1767297,892	-941182,13	1787105,08	-941182,126	1767297,892
Z1_AC2_AHR_N2	0,01	183,29	0,013	99,909	0,01	183,29	0,013	99,909
Mw_AC2_AHR_Q3	4047	50964	4047	50964	4047	50964	4047	50964
Z2_AC2_AHR_N2	0,01	53,23	0,01	50,224	0,01	53,23	0,01	50,224
Mw_AC2_UCR_MX	4959	60636	4959	60636	4959	60636	4959	60636
Z1_AC2_UCR_Ar	-30,72	20,99	-30,722	18,547	-30,722	20,99	-30,722	18,547
Z1_AC2_NPR_N3	-67,75	111,6	-62,551	84,284	-67,75	111,6	-62,551	84,284
Z1_AC2_RTR_Q2	-35,2	26,5	-34,58	23,587	-35,2	26,5	-34,58	23,587
ECI_AC2_RTR_RA	0	4,43	0	4,427	0	4,43	0	4,427
ECI_AC2_BSR_MX	0	3,65	0,001	3,65	0	3,65	0,001	3,65

Gs(U)_AC2_BSR_Q1	-1579487,69	1057312,79	-1579487,692	1057312,793	-1579487,692	1057312,79	-1579487,692	1057312,793
Gs(U)_AC2_ARM_Q3	-1138695,5	1185300,96	-1138695,501	1185300,962	-1138695,501	1185300,962	-1138695,501	1185300,962
ISA_AC2_PRT_CV	0,19	1,87	0,196	1,829	0,19	1,87	0,196	1,829
Z3_AC2_ALR_N3	-31,31	26,03	-31,312	25,089	-31,31	26,03	-31,312	25,089
Gw(U)_AC2_PCR_CV	0	3,46	0	3,464	0	3,46	0	3,464
Z2_AC2_RTR_MN	-39,13	33,93	-39,128	28,73	-39,13	34,25	-39,128	34,25
Z3_ES_PCR_TI5	0	69,5	0	44,29	0	69,5	0	44,29
Z3_ES_PCR_S	-3,06	3,86	-2,406	3,37	-3,06	3,86	-2,406	3,37
Gs(U)_ES_PRT_P2	65,44	2009,9	195,996	1890,971	65,44	2009,9	195,996	1890,971
Gs(U)_ES_PCR_CV	-0,7	0	-0,617	0	-0,7	0	-0,617	0
ISA_ES_PCR_CV	-10838,16	354,9	-10838,162	354,895	-10838,16	354,9	-10838,162	354,895
HP_ES_PCR_S	-2,68	3,32	-2,406	2,238	-2,68	3,32	-2,406	2,238
IP_ES_PCR_SI5	0	1	0	1	0	1	0	1
ISA_ES_PCR_S	-3,61	3,32	-3,607	2,556	-3,61	3,32	-3,607	2,556
HP_ES_PCR_SI5	0	1	0	1	0	1	0	1
HP_ES_PLR_Q1	-12,14	4,06	-12,01	2,271	-12,14	4,06	-12,01	2,271
Z2_ES_PCR_SI5	0	1	0	1	0	1	0	1
IP_ES_PCR_P3	8,01	17,79	8,401	17,204	8,01	17,79	8,401	17,204
IP_ES_BSR_Ar	1,46	7,67	1,55	7,349	1,46	7,67	1,55	7,349
IP_ES_BSR_MN	0,55	7,51	0,547	7,349	0,55	7,51	0,547	7,349
Z3_ES_AHR_MN	-10,04	10,19	-8,839	10,193	-10,04	10,19	-9,157	10,193
Z1_ES_PCR_CV	0	0,62	0	0,577	0	0,635	0	0,577
ISA_ES_RTR_Q2	-114,28	204,1	-114,276	204,102	-114,28	204,247	-114,276	204,247
Z3_ES_UCR_MX	-3,89	11,54	-3,235	11,54	-3,89	11,577	-3,235	11,577
IP_ES_UCR_Q3	-4,32	7,71	-2,376	7,176	-4,32	7,71	-2,561	7,176
Gs(U)_ES_PLR_Q3	-2314,66	1098,63	-2253,505	1098,627	-2474,666	1098,63	-2474,666	1098,627

Z3_ES_RTR_Q2	-1,3	7,69	-0,819	7,519	-1,322	7,69	-0,819	7,519
Z3_ES_ARM_Ar	-2,96	5,61	-2,228	4,908	-2,96	5,61	-2,228	4,908
ECI_ES_AHR_I50	-1,42	3,29	-1,25	3,148	-1,42	3,29	-1,25	3,148
Z1_ES_NPR_N3	-30,9	7,49	-24,947	7,49	-30,9	7,49	-24,959	7,49
ECI_ES_RTR_MX	-1,34	2,78	-1,34	2,785	-1,34	2,78	-1,34	2,785
IP_ES_UCR_P3	-4,51	7,15	-3,085	6,776	-4,51	7,15	-3,085	6,776
Z3_ES_RTR_Q1	-2,42	7,69	-2,422	7,509	-2,42	7,69	-2,422	7,509
ISA_ES_PCR_MN	-60,58	181,29	-60,582	171,078	-60,58	181,29	-60,582	172,277
IP_ES_ARM_Q2	1,07	11,1	1,071	10,402	1,07	11,1	1,071	10,402
Gs(U)_ES_PRT_Q3	-1052,65	2023,94	-1046,549	1982,117	-1052,65	2023,94	-1046,549	1982,117
Z3_ES_ALR_Q3	-4,91	7,69	-4,908	7,39	-4,91	7,69	-4,908	7,39
ISA_ES_RTR_MN	-132,12	195,41	-132,116	195,413	-132,12	195,41	-132,116	195,413
Z1_ES_PCR_Q1	2,04	9,89	2,125	9,89	2,04	9,89	2,125	9,89
Z3_ES_ARM_N1	-6,91	61,37	-4,621	32,974	-6,91	61,37	-4,621	32,974
ISA_ES_PCR_Q1	-59,37	181,29	-59,373	171,078	-59,37	181,29	-59,373	172,277
Z2_ES_PLR_Ar	-4,01	8,28	-3,257	8,21	-4,01	8,28	-3,257	8,21
Z1_ES_AHR_P2	0,01	10,82	0,111	9,966	0,01	10,82	0,111	10,016
Gs(U)_ES_ALR_MX	-661,06	2504,44	-516,742	2446,231	-661,06	2504,44	-516,742	2446,231

Table S3. Values resulting from the radial diffusion assay applied to the peptide Urine-3462 against the *Pseudomonas aeruginosa* strain ATCC 27853.

EXPERIMENTS	1000	500	250	125	62.5	31.25	15.6	7.8	LL 37
**1**	1.2	1.1	0.9	0.8	0.6	0.4	0	0	0.7
**2**	1.3	1.1	0.9	0.7	0.6	0.4	0	0	0.7
**3**	1.1	1	0.9	0.8	0.5	0.3	0	0	0.6
**4**	1.3	1	0.9	0.8	0.6	0.3	0	0	0.6
**5**	1.2	1.1	1	0.8	0.6	0.4	0.3	0	0.7
**6**	1.2	1.1	0.9	0.8	0.6	0.4	0	0	0.7
